# Generation of multimillion chemical space based on the parallel Groebke–Blackburn–Bienaymé reaction

**DOI:** 10.3762/bjoc.20.143

**Published:** 2024-07-16

**Authors:** Evgen V Govor, Vasyl Naumchyk, Ihor Nestorak, Dmytro S Radchenko, Dmytro Dudenko, Yurii S Moroz, Olexiy D Kachkovsky, Oleksandr O Grygorenko

**Affiliations:** 1 Enamine Ltd., Winston Churchill Street 78, Kyїv 02094, Ukraine; 2 Taras Shevchenko National University of Kyiv, Volodymyrska Street 60, Kyїv 01601, Ukraine; 3 V. P. Kukhar Institute of Bioorganic Chemistry and Petrochemistry, Akademik Kukhar Street 1, Kyїv 02094, Ukraine

**Keywords:** fused rings, heterocycles, imidazoles, isonitrile, multicomponent reactions

## Abstract

Parallel Groebke–Blackburn–Bienaymé reaction was evaluated as a source of multimillion chemically accessible chemical space. Two most popular classical protocols involving the use of Sc(OTf)_3_ and TsOH as the catalysts were tested on a broad substrate scope, and prevalence of the first method was clearly demonstrated. Furthermore, the scope and limitations of the procedure were established. A model 790-member library was obtained with 85% synthesis success rate. These results were used to generate a 271-Mln. readily accessible (REAL) heterocyclic chemical space mostly containing unique chemotypes, which was confirmed by comparative analysis with commercially available compound collections. Meanwhile, this chemical space contained 432 compounds that already showed biological activity according to the ChEMBL database.

## Introduction

**Scheme 1:**
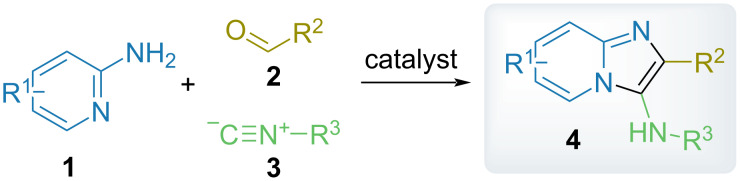
Groebke–Blackburn–Bienaymé (GBB) reaction.

**Figure 1:**
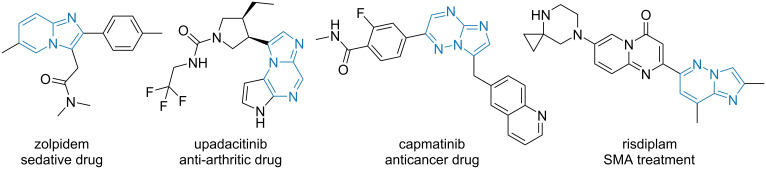
Marketed drugs comprising imidazo[1,2-*a*]azine scaffolds.

Multicomponent reactions are widely recognized as a powerful source of biologically active compounds, in particular, for drug discovery purposes [1–6]. Isonitrile-based multicomponent reactions, such as the Groebke–Blackburn–Bienaymé (GBB) reaction, is an important tool in chemical synthesis providing easy access to a huge compound diversity and complexity [7–17]. Essentially, the GBB reaction is a three-component condensation of an α-amino heterocycle (e.g., 2-aminopyridine) **1**, an aldehyde **2**, and an isonitrile **3** providing the corresponding fused imidazoles (e.g., imidazo[1,2-*a*]pyridines) of general formula **4** (Scheme 1) [18–21]. Imidazo[1,2-*a*]pyridines and related heterocycles can be considered as privileged chemotypes in drug discovery: their representatives include the sedative drug zolpidem, disease-modifying antirheumatic agent upadacitinib, anticancer drug capmatinib, or risdiplam, a medication for spinal muscular atrophy (SMA) treatment (Figure 1) [22–23].

Over the last two decades, the GBB reaction has been studied in more than 200 research papers [21] and a number of works (including the original publication by Blackburn and coauthors [20]) described its parallel synthesis version [24–29]. Recently, we have shown that other (pseudo-)three-component reactions are very effective for the generation of synthetically tractable ultra-large chemical space [30–32]. Such virtual but readily accessible (REAL) compound libraries demonstrated excellent performance in early drug discovery programs when combined with modern computational tools such as high-throughput docking or machine learning [33–37]. In this work, we aimed at the implementation of the GBB reaction for the generation of such ultra-large chemical space, including experimental evaluation of the synthesis success rate (SSR, i.e., percentage of experiments that allowed obtaining the target library member in pure form) on a large set of starting materials.

Through the article, the compound numbering system common for the works on combinatorial chemistry was used: the starting materials used for the library generation were marked as **1**{*i*}, **2**{*j*}, and **3**{*k*}, whereas the corresponding library members were denoted as **4**{*i*,*j*,*k*}.

## Results and Discussion

### Library synthesis

**Figure 2:**
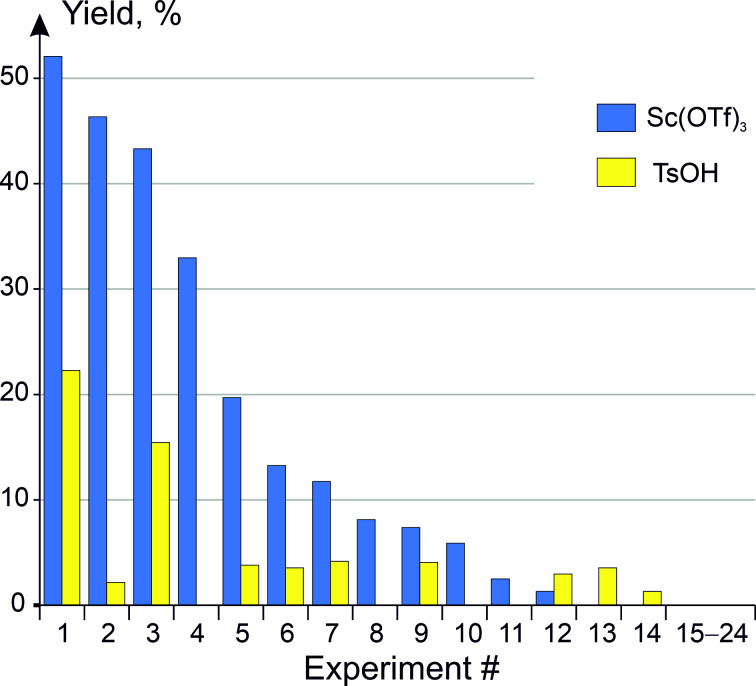
Yields of library members **4** synthesized using both Sc(OTf)_3_ and TsOH as the catalysts.

Preliminary experiments on the parallel GBB reaction were performed with heterocyclic amines **1**{1–430}, aldehydes **2**{1–583}, and isonitriles **3**{1–73} available from our stock (based on our previous in-house experience on isonitrile-based parallel reactions, electron-poor (hetero)aromatic isonitriles were not included in the study). According to Boltjes and Dömling, the following three catalysts were applied most often to promote the title reaction [21]: Sc(OTf)_3_ (described first in the original work by Blackburn and coauthors [20]), HClO_4_, and TsOH. We wanted to avoid the use of HClO_4_ in our parallel reaction set-up, so that only two remaining catalytic systems were evaluated. 580 library members were deliberately selected for both reaction conditions, and the corresponding experiments were performed (reactants at 1:1:1 ratio, 10 mol % of the catalyst, MeOH, rt, 16 h). It was found that TsOH, albeit being cheaper, demonstrated a poorer performance as the reaction promotor (62% SSR vs 67% for Sc(OTf)_3_; 34% average yield in both cases). This is especially apparent if the product yields are compared for 24 library members that we attempted to obtain by both methods (Figure 2).

**Figure 3:**
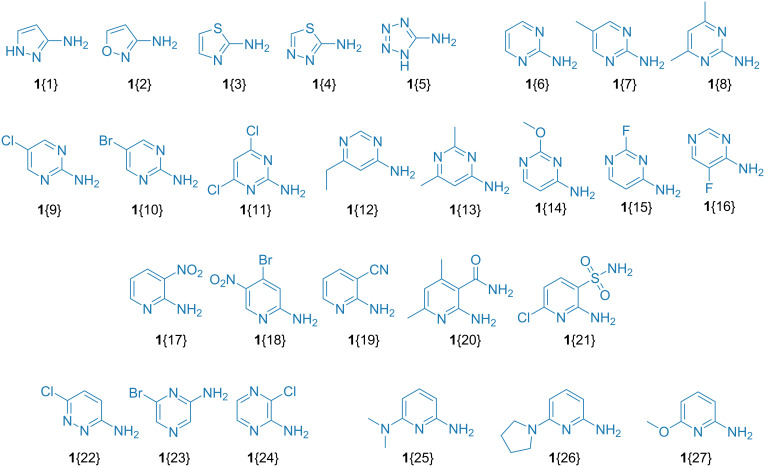
Amino heterocycles **1**{1–27} demonstrating poor performance in the parallel GBB reaction.

These preliminary experiments also allowed establishing the following limitations of the method and excluding the corresponding reactants from further studies (Figure 3):

- α-aminoazoles of varied electronic nature (e.g., 3-aminopyrazole (**1**{1}), 3-aminoisoxazole (**1**{2}), 2-aminothiazole (**1**{3}), 2-amino-1,3,4-thiadiazole (**1**{4}), or 2-aminotetrazole (**1**{5}) demonstrated poor conversion to the target products;
- 2-aminopyrimidines either gave isomeric mixtures (e.g., parent compound **1**{6}, alkyl-substituted derivatives **1**{7} and **1**{8}) or showed low conversion (halogenated derivatives **1**{9–11});
- 4-aminopyrimidines **1**{12–16} also demonstrated low conversion;
- pyridine derivatives with electron-withdrawing substituents (such as NO_2_ at C-3 or C-5 positions, as well as CN, SO_2_NH_2_, or C(O)NH_2_ at the C-3 atom) did not work (compounds **1**{17–21});
- for pyridazine or pyrazine derivatives, even the presence of halogen atoms was sufficient to deactivate the substrate (e.g., compounds **1**{22–24});
- a dialkylamino or alkoxy group at the C-6 position of pyridine derivatives also hampered the substrate’s reactivity (e.g., compounds **1**{25–27}).

Some of these results (e.g., on the reactivity of aminopyrimidine derivatives) were in accordance with the previous literature data [21,29]. Meanwhile, electronic effects of the substituents in the amino heterocycle reported in the previous works were somewhat contradictory. Whereas for the NO_2_ group, lowering the reactivity has been documented, other electron-withdrawing groups were reported to be generally compatible with the GBB reaction [21]. Interference of dialkylamino or alkoxy groups at the C-6 position was also mentioned previously [29].

**Figure 4:**
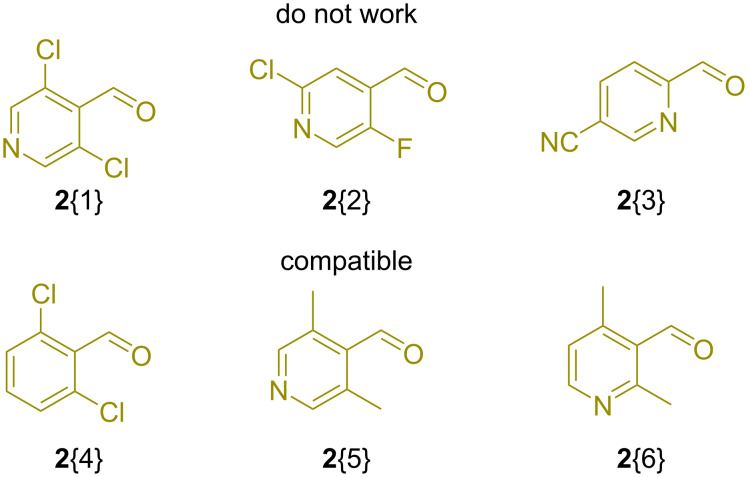
(Hetero)aromatic aldehydes **2**{1–6} illustrating electronic and steric effects on the parallel GBB reaction.

In addition to that, it was found that electron-poor aromatic aldehydes (e.g., **2**{1–3}) did not work in the GBB reaction (Figure 4). This result is in accordance with the previous findings [21]. Notably, steric effects were not significant since *o*,*o*′-disubstituted aldehydes (e.g., **2**{4–6}) displayed usual efficiency. As might be expected from our previous experience, 4-fluoro- and 4-chloro-2-fluoro-1-isocyanobenzenes (**3**{1} and **3**{2}) showed poor performance and unsatisfactory results were also observed for isocyanocyclopropane (**3**{3}).

**Scheme 2:**
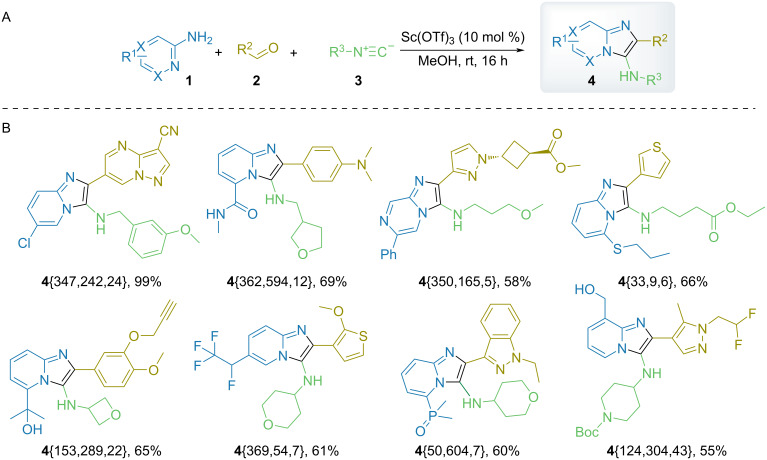
A) Parallel GBB reaction and B) examples of library members **4** obtained (relative configurations are shown).

Using the guidelines described above, we have updated the reactant lists with additional representatives and excluded those demonstrating poor performance. Using the resulting sets of amino heterocycles **1**{22–485}, aldehydes **2**{4–867}, and isonitriles **3**{4–78}, 892 library members **4**{22–485,4–867,4–78} were deliberately selected and subjected to the parallel synthesis using the Sc(OTf)_3_-based protocol. As a result, 790 library members were obtained successfully (SSR = 85%, average yield of 37%) (Scheme 2).

### Chemical space generation

Since validation of the GBB reaction showed that it is compatible with the main readily accessible chemical space criteria (at least 80% synthesizability) [30], we have aimed at the generation of the corresponding REAL space. For this purpose, 686 amino heterocycles **1**, 3,927 aldehydes **2**, and 107 isonitriles **3** complying with our general in-house reactivity/availability filters were subjected to virtual coupling using the limitations mentioned above. Additionally, combinations providing compounds with more than two chiral centers were not included. This resulted in 271,026,660 library members with nearly 85% expected synthetic accessibility according to the model experiments described in the previous section.

**Figure 5:**
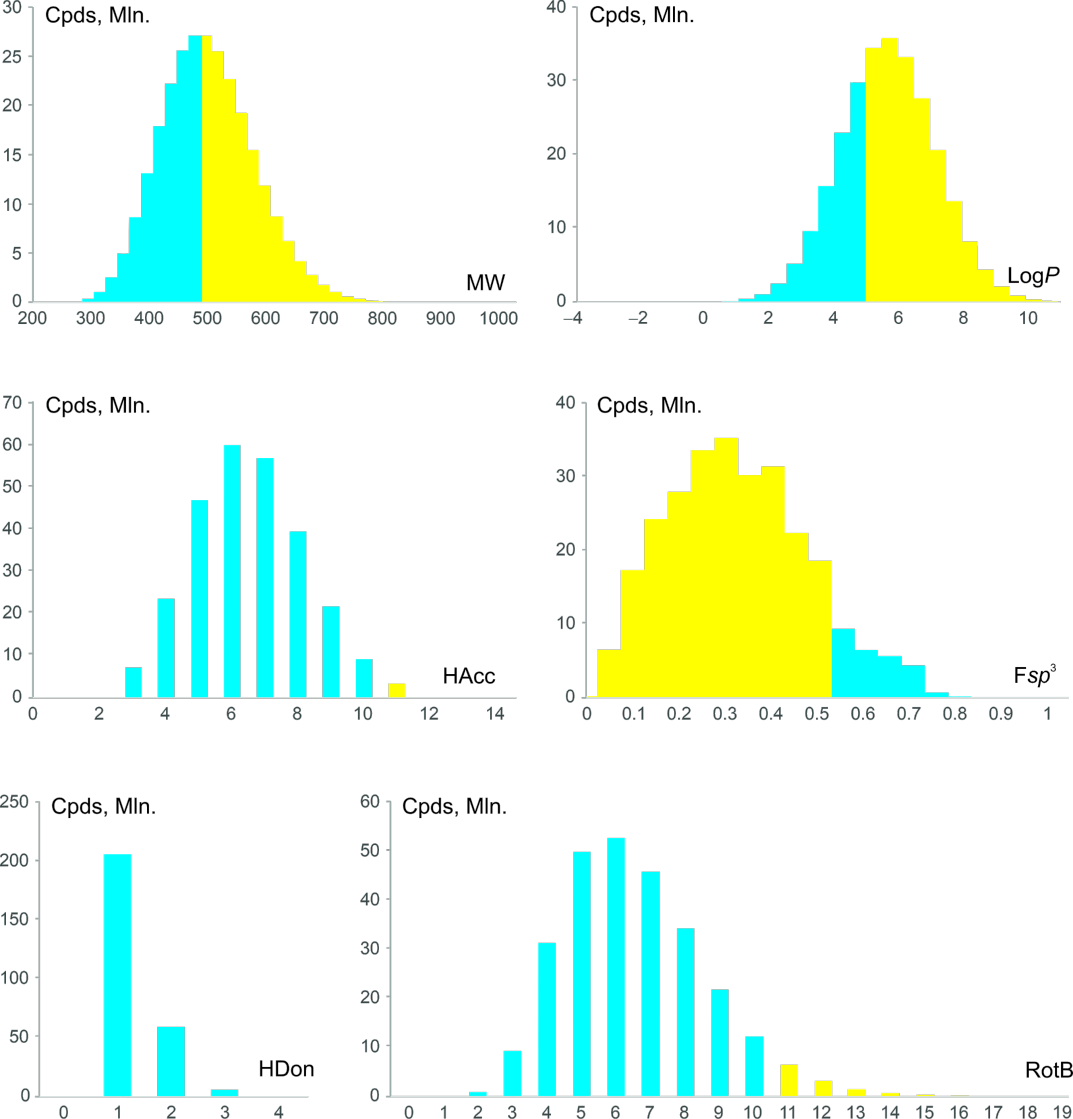
Physicochemical properties of the chemical space of 271 Mln. members obtained by virtual GBB reaction (MW – molecular weight; HAcc/HDon – H-bond acceptor/donor count; F(sp^3^) – fraction of sp^3^-hybrid carbon atoms; RotB – rotatable bond count); compounds complying with specific Lipinski/Veber rules (MW ≤ 500, log *P* ≤ 5, HDon ≤ 5, HAcc ≤ 10, RotB ≤ 10 [38,41]) as well as compounds with F(sp^3^) > 0.5 are highlighted in blue, the rest of the compounds are shown in yellow.

Distributions of the resulting chemical space over molecular weight (MW), 1-octanol–water partition coefficient logarithm (log *P*), H-bond acceptor/donor count (HAcc/HDon), fraction of sp^3^-hybrid carbon atoms (F(sp^3^)), and rotatable bond count (RotB) are shown in Figure 5. It is apparent that the GBB chemical space contains many drug-like (69,043,101 molecules, 25%) and “beyond-Ro5” compounds (75%) [38]. Furthermore, although the proposed approach cannot be considered lead-oriented, it may provide 12,122,351 lead-like compounds compliant with the “rule-of-four” (MW < 400, log *P* < 4) [39], and 1,383,298 with the even stricter Churcher’s rules (MW = 200–350, log *P* = −1 to 3) [40].

**Figure 6:**
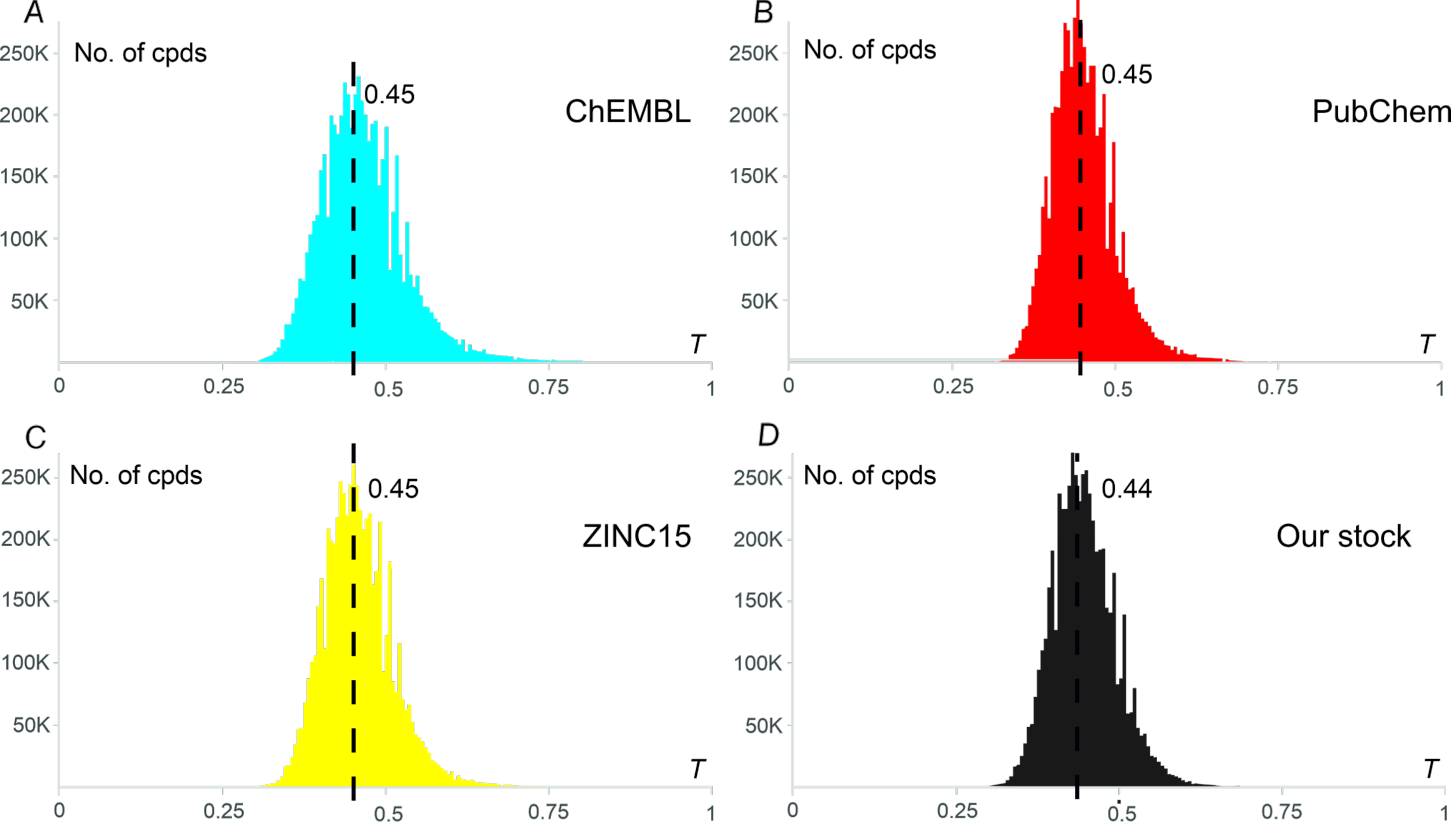
Distribution of maximal values among pairwise-calculated Tanimoto similarities *T* (MFP2 fingerprints [46]) of extended Bemis–Murcko scaffolds for the generated chemical space members (5.60 Mln. scaffolds) to the extended Bemis–Murcko scaffolds of A) ChEMBL compounds (v. 33); B) PubChem compounds (due to the large size of the dataset, a preliminary clusterization was performed to achieve ca. 5-fold size reduction); C) ZINC15 drug-like compounds, and D) enamine’s stock screening collection. Average *T* values are shown by dotted lines.

Next, we compared the GBB chemical space with common chemical databases (ChEMBL [42], PubChem [43], and ZINC15 [44]), as well as our stock screening compound collection [45]. Due to the enormous size of the databases, pairwise Tanimoto analysis was performed at the extended Bemis–Murcko scaffold level. First, extended Bemis–Murcko scaffolds were generated by cutting off the side chains of the molecules and retaining ring systems and linkers between them. After removal of duplicates, Tanimoto similarity coefficients were calculated for each pair of the molecules in the compared databases (*T* = 1 and 0 for similar and very dissimilar molecules, respectively). Average pairwise values for each molecule from the database of comparison are depicted in Figure 6. The mean values for all the databases were in the range of 0.44–0.45, which shows that despite there are some representatives that are similar to already known compounds, the generated GBB chemical space is unique as compared to the available compound collections.

**Figure 7:**
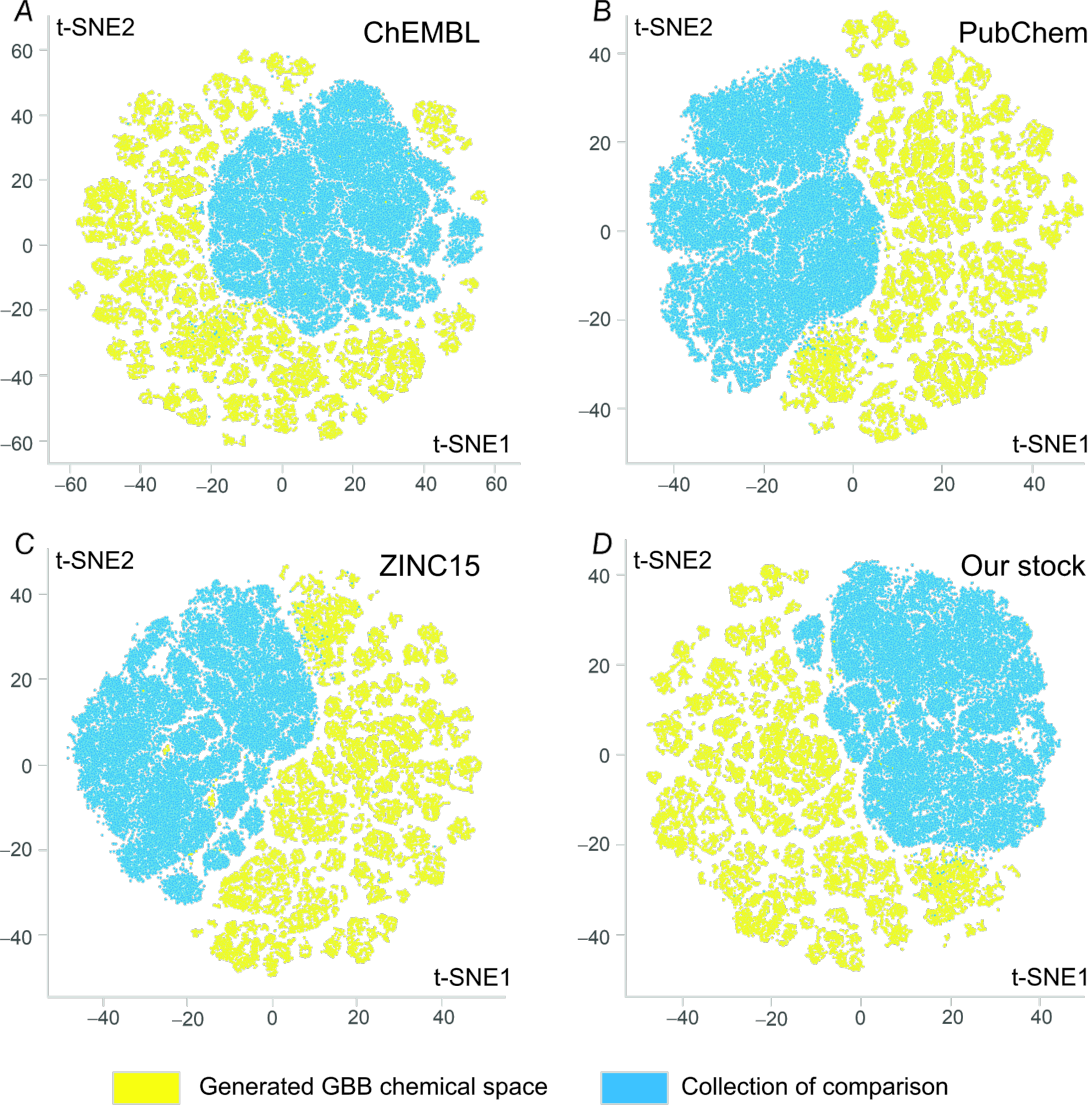
t-Distributed stochastic neighbor embedding (t-SNE) comparative analysis of 50,000 randomly selected molecules picked from the generated chemical space and A) ChEMBL compounds; B) PubChem compounds; C) ZINC15 compounds; and D) enamine’s stock screening collection.

This fact is even more apparent from the t-distributed stochastic neighbour embedding (t-SNE) analysis, a technique widely used for the dimension reduction in data visualization [47]. Due to the relatively high computational costs of this method, we randomly selected 50,000 compounds to represent each database. The dimension reduction algorithm uses molecular features as the starting inputs to generate a few coordinates (in this case, t-SNE1 and t-SNE2) reflecting the probability of the molecules to be similar. In this way, data visualization becomes possible since similar molecules will likely have close values of t-SNE1 and t-SNE2. As apparent from Figure 7, there is a small overlap between the GBB chemical space (yellow datapoints) with all four databases of comparison (blue data points), which is another confirmation of the GBB space uniqueness.

**Figure 8:**
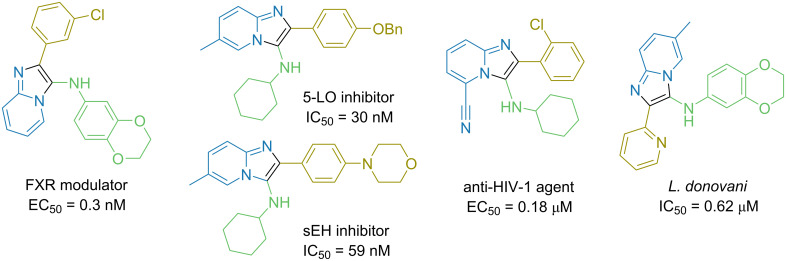
Some biologically active representatives of the generated GBB chemical space found in the ChEMBL database.

Notably, 432 members of the generated GBB chemical space were already present in the ChEMBL database [42]. Among them, potent nonacidic farnesoid X receptor (FXR) modulators [48], 5-lipooxygenase (5-LO) inhibitors [49], soluble epoxide hydrolase (sEH) inhibitors [50], HIV-1 non-nucleoside reverse transcriptase inhibitors [51], or potential agents against visceral leishmaniasis [52] were found (Figure 8).

## Conclusion

The Groebke–Blackburn–Bienaymé (GBB) reaction, a three-component condensation of amino heterocycles, aldehydes, and isonitriles, is a powerful tool for the combinatorial synthesis of compound libraries. We have shown that the Sc(OTf)_3_-catalyzed version of the reaction has a wide substrate applicability and established some limitations under the parallel synthesis conditions. In particular, while the method was applicable to a wide range of aminopyridines, pyrazines, and pyridazines, it worked poorly for aminoazoles, aminopyrimidines, substrates with strong electron-withdrawing groups, and substrates bearing additional dialkylamino or alkoxy substituents. The electronic nature was a major limiting factor for other two components of the reaction, the aldehyde and isonitrile, while the steric factor was found to be not significant. The protocol was used to prepare a 790-member compound library with 85% synthesis success rate. Furthermore, a readily available (REAL) chemical space comprising 271 Mln. members was generated. It was rich in both drug-like and “beyond rule-of-five” compounds and had considerable uniqueness as compared to the available collections (as was shown by Tanimoto similarity and t-distributed stochastic neighbor embedding (t-SNE) comparative analyses). Still, 432 members of the generated chemical space were found in the ChEMBL database, and some of them had high potency against various biological targets.

## Supporting Information

The Supporting Information for this article contains structures of reactants **1**–**3**, experimental details on the parallel synthesis of compound library **4**, experimental section including compound characterization data, and copies of NMR spectra.

File 1 Structures of reactants **1**, **2**, and **3**.

File 2 Parallel synthesis of compound library **4**.

File 3 Experimental part.

File 4 Copies of NMR spectra.

## Data Availability

Most data that supports the findings of this study is available in the published article and/or the supporting information to this article. Additional data cannot be shared due to restrictions that apply to availability.
