# Supplementary material for: Generation of multimillion chemical space based on the parallel Groebke–Blackburn–Bienaymé reaction
Source: Beilstein J Org Chem. 2024 Jul 16;20:1604–13. doi: 10.3762/bjoc.20.143 (PMC11285076; doi:10.3762/bjoc.20.143)
Supplement: File 1 — Structures of reactants 1, 2, and 3. [file Beilstein_J_Org_Chem-20-1604-s001.zip › Structures of substrates 1.pdf]

Structures of reactants 1

ID	Structure
**1**{*1*}	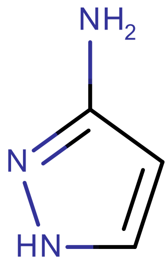
**1**{*2*}	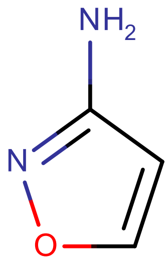
**1**{*3*}	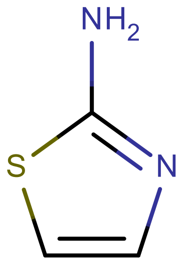
**1**{*4*}	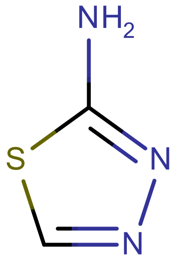

**1**{*5*}	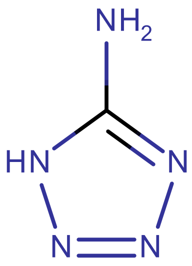
**1**{*6*}	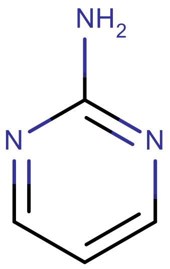
**1**{*7*}	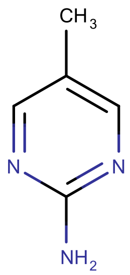
**1**{*8*}	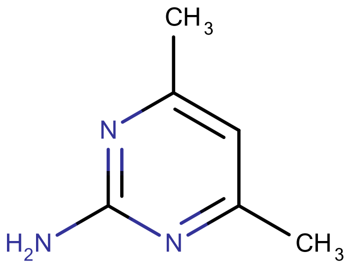

**1**{*9*}	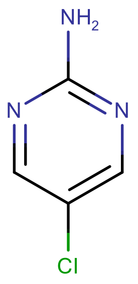
**1**{*10*}	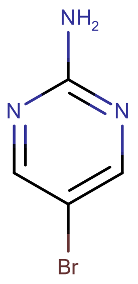
**1**{*11*}	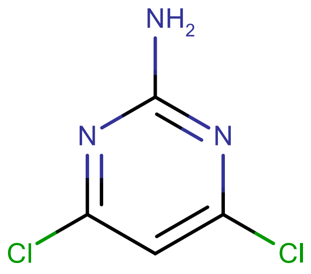
**1**{*12*}	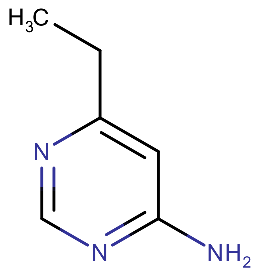

**1**{*13*}	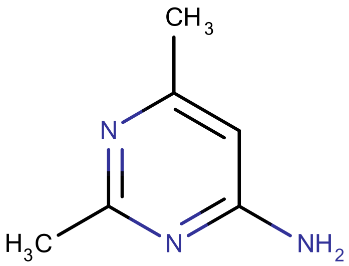
**1**{*14*}	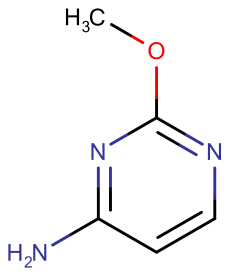
**1**{*15*}	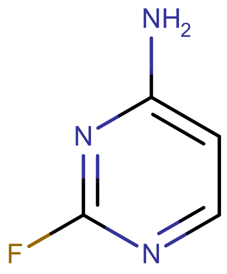
**1**{*16*}	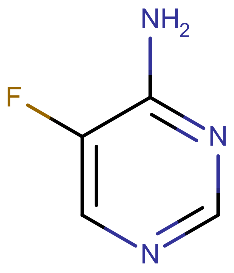

**1**{*17*}	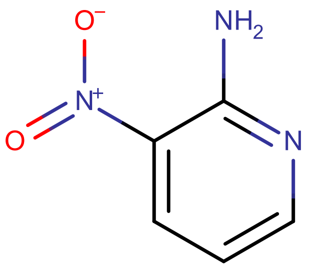
**1**{*18*}	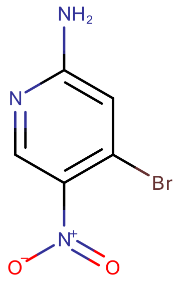
**1**{*19*}	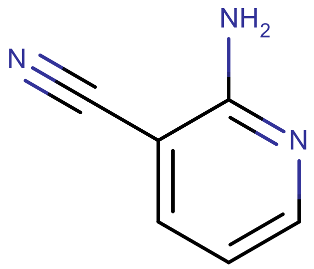
**1**{*20*}	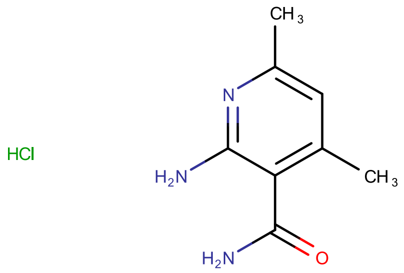

**1**{*21*}	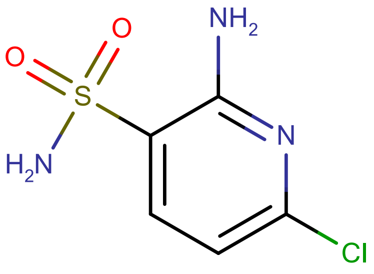
**1**{*22*}	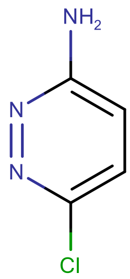
**1**{*23*}	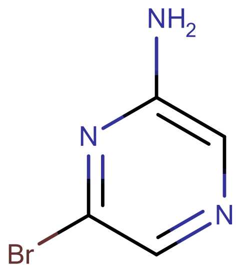
**1**{*24*}	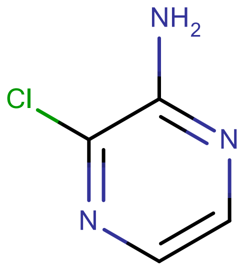

**1**{*25*}	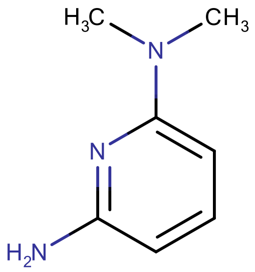
**1**{*26*}	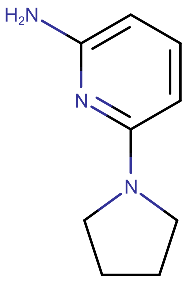
**1**{*27*}	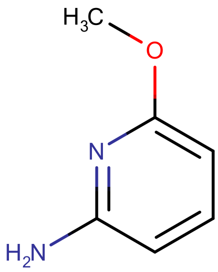
**1**{*28*}	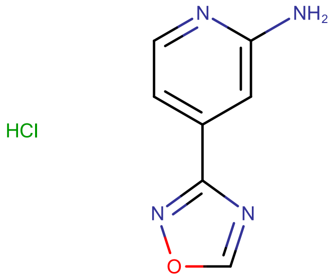

**1**{*29*}	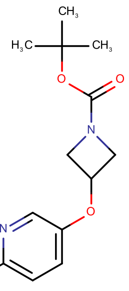
**1**{*30*}	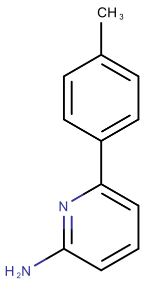
**1**{*31*}	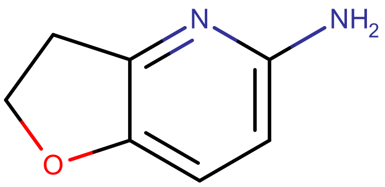
**1**{*32*}	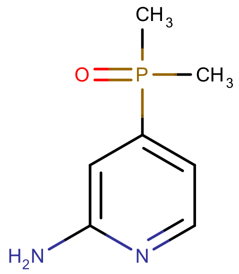

**1**{*33*}	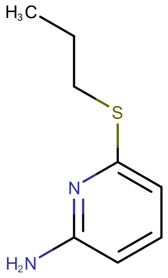
**1**{*34*}	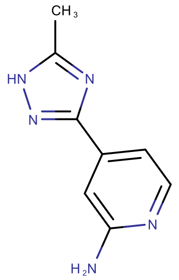
**1**{*35*}	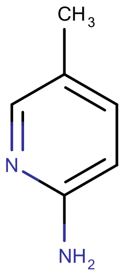
**1**{*36*}	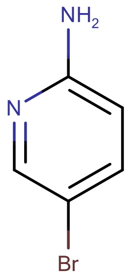

**1**{*37*}	HCl 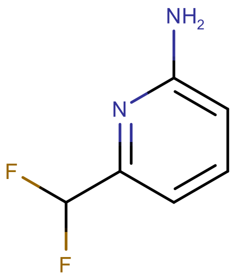
**1**{*38*}	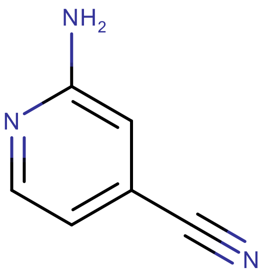
**1**{*39*}	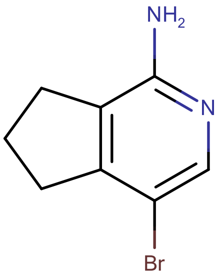
**1**{*40*}	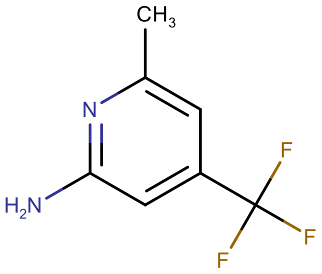

**1**{*41*}	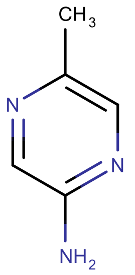
**1**{*42*}	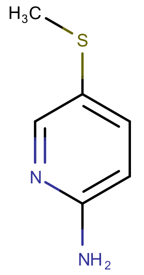
**1**{*43*}	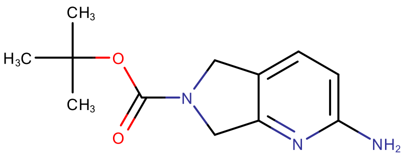
**1**{*44*}	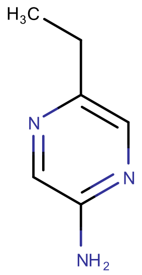

**1**{*45*}	HCl 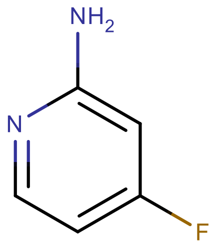
**1**{*46*}	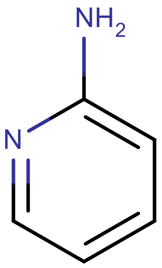
**1**{*47*}	HCl 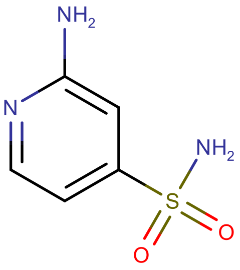
**1**{*48*}	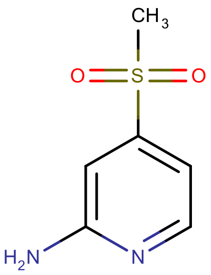

**1**{*49*}	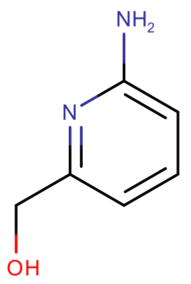
**1**{*50*}	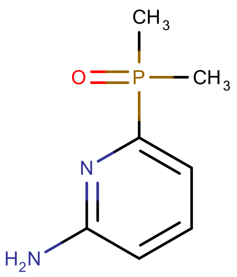
**1**{*51*}	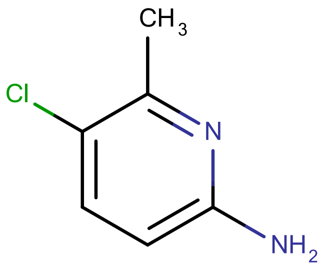
**1**{*52*}	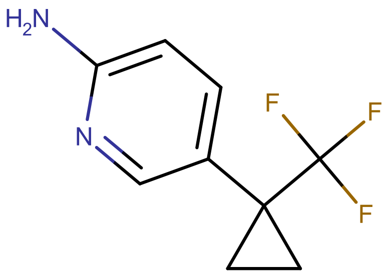

**1**{*53*}	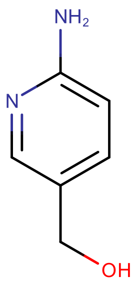
**1**{*54*}	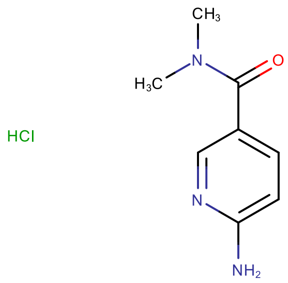
**1**{*55*}	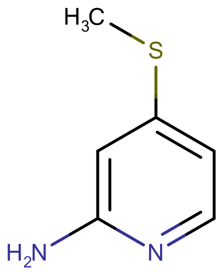
**1**{*56*}	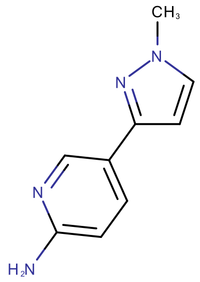

**1**{*57*}	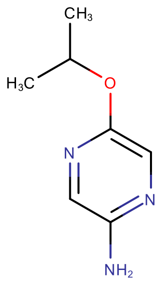
**1**{*58*}	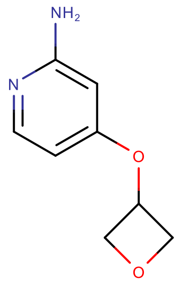
**1**{*59*}	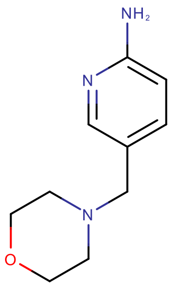
**1**{*60*}	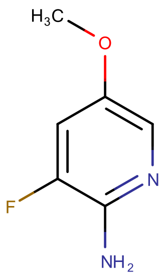

**1**{*61*}	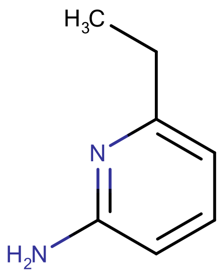
**1**{*62*}	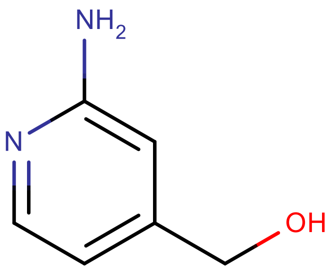
**1**{*63*}	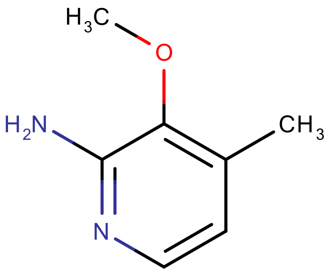
**1**{*64*}	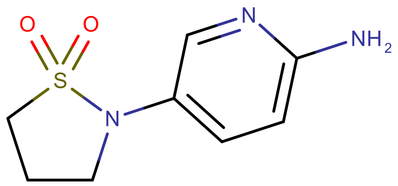

**1**{*65*}	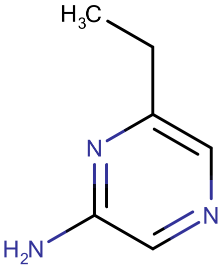
**1**{*66*}	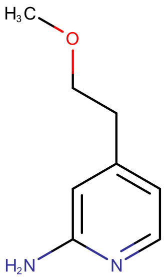
**1**{*67*}	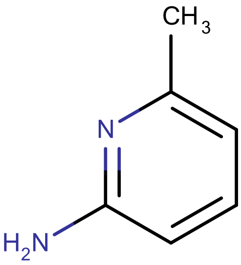
**1**{*68*}	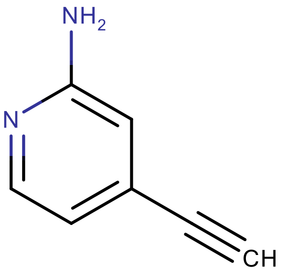

**1**{*69*}	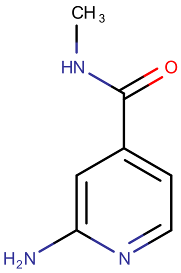
**1**{*70*}	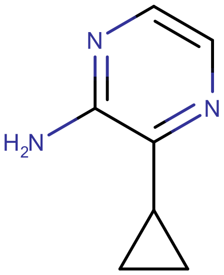
**1**{*71*}	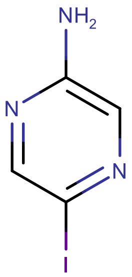
**1**{*72*}	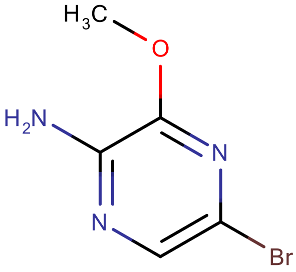

**1**{*73*}	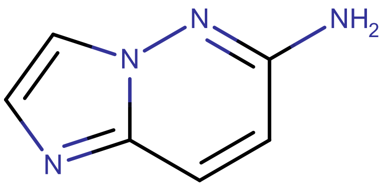	**1**{*77*}	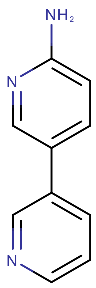
**1**{*74*}	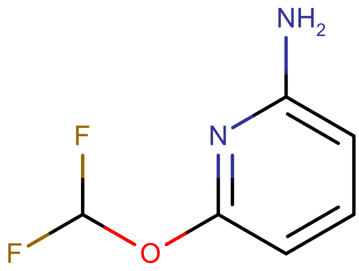	**1**{*78*}	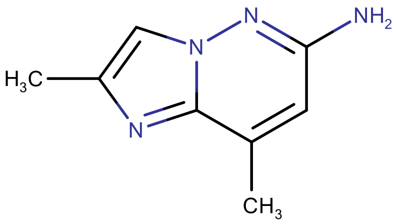
**1**{*75*}	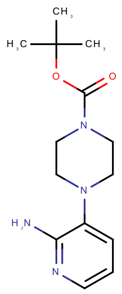	**1**{*79*}	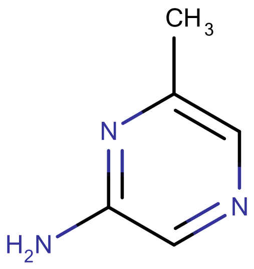
**1**{*76*}	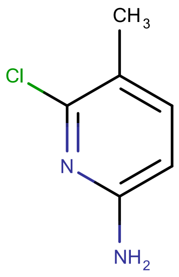	**1**{*80*}	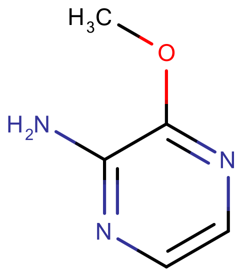

**1**{*81*}	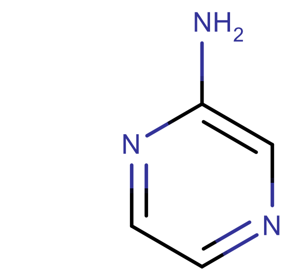
**1**{*82*}	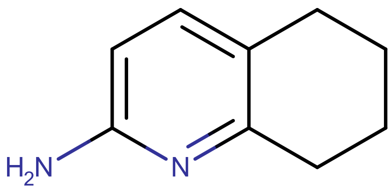
**1**{*83*}	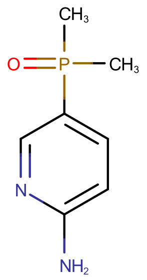
**1**{*84*}	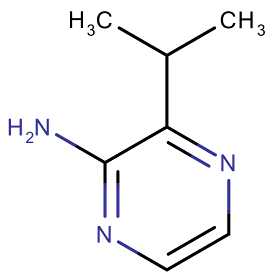

**1**{*85*}	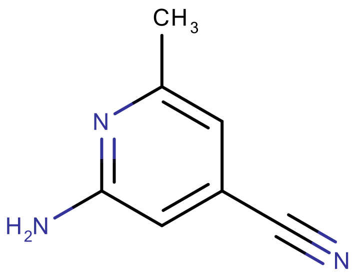
**1**{*86*}	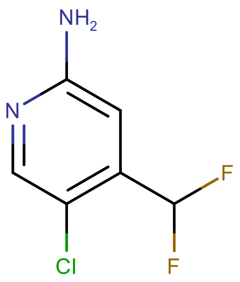
**1**{*87*}	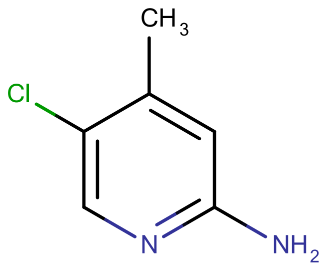
**1**{*88*}	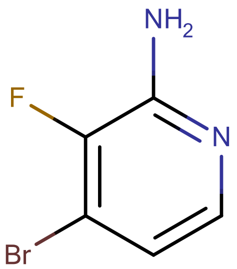

**1**{*89*}	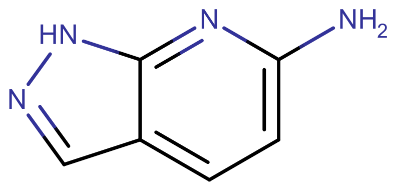
**1**{*90*}	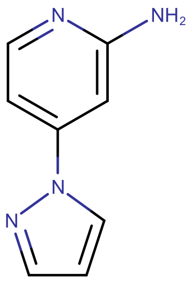
**1**{*91*}	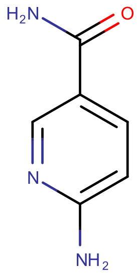
**1**{*92*}	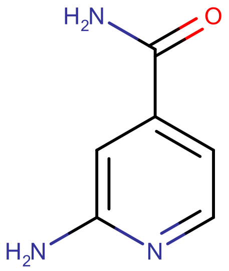

**1**{*93*}	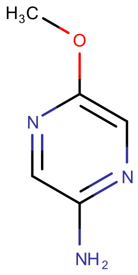
**1**{*94*}	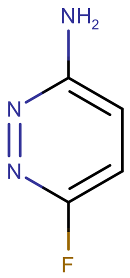
**1**{*95*}	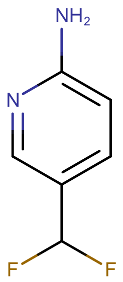
**1**{*96*}	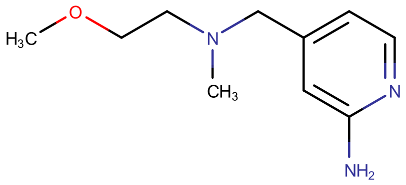

**1**{*97*}	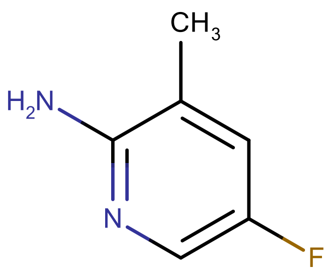
**1**{*98*}	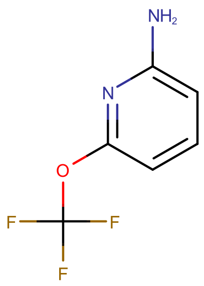
**1**{*99*}	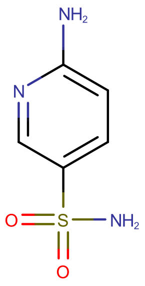
**1**{*100*}	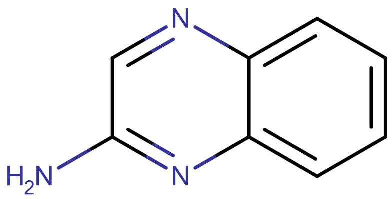

**1**{*101*}	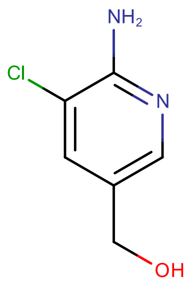
**1**{*102*}	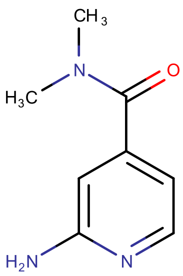
**1**{*103*}	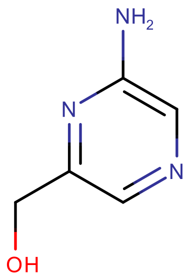
**1**{*104*}	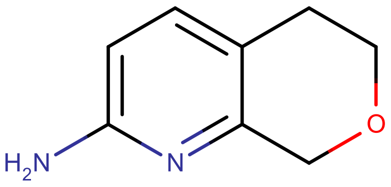

**1**{*105*}	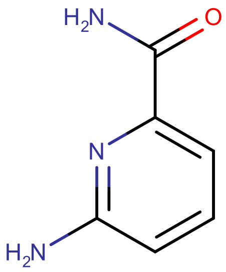
**1**{*106*}	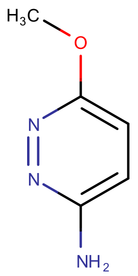
**1**{*107*}	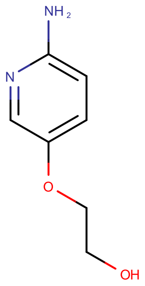
**1**{*108*}	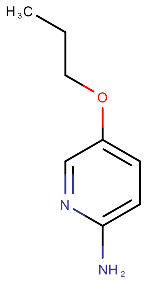

**1**{*109*}	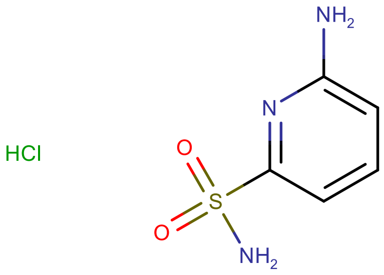
**1**{*110*}	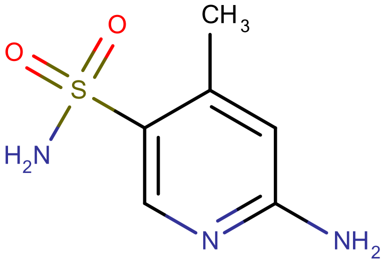
**1**{*111*}	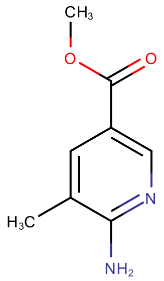
**1**{*112*}	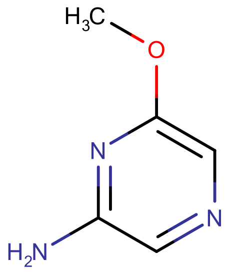

**1**{*113*}	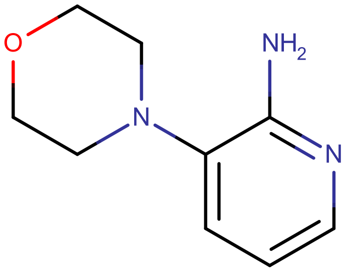
**1**{*114*}	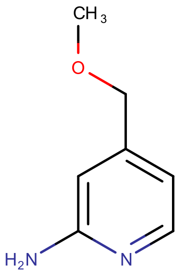
**1**{*115*}	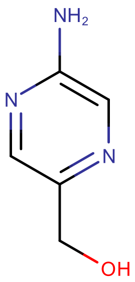
**1**{*116*}	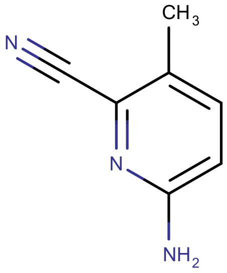

**1**{*117*}	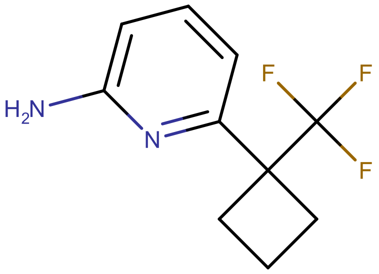
**1**{*118*}	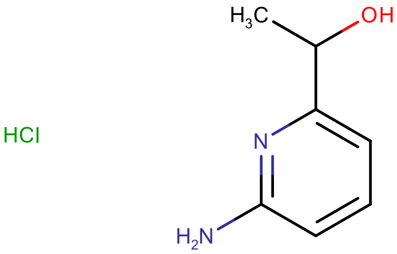
**1**{*119*}	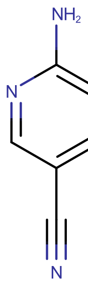
**1**{*120*}	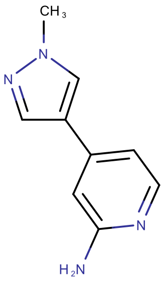

**1**{*121*}	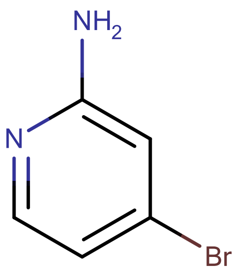
**1**{*122*}	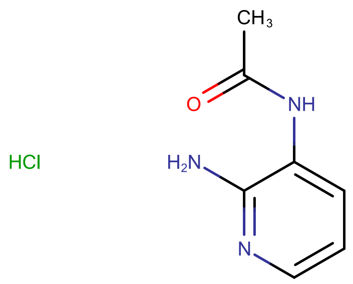
**1**{*123*}	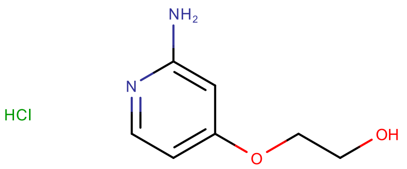
**1**{*124*}	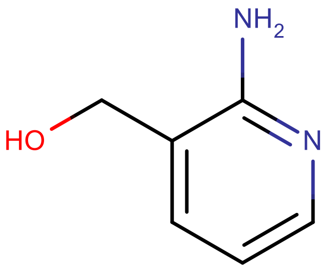

**1**{*125*}	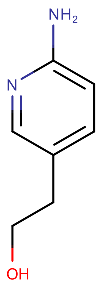
**1**{*126*}	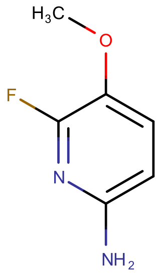
**1**{*127*}	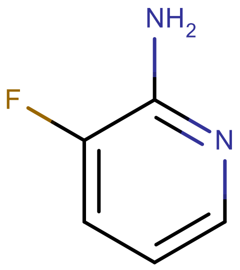
**1**{*128*}	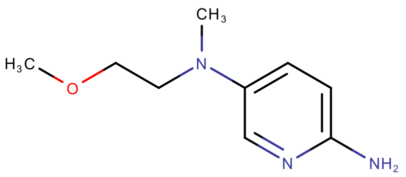

**1**{*129*}	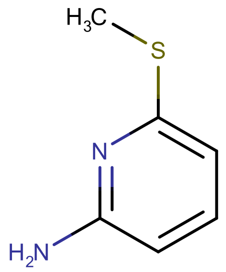
**1**{*130*}	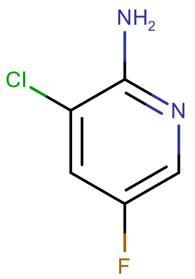
**1**{*131*}	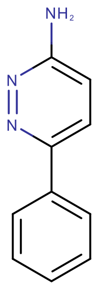
**1**{*132*}	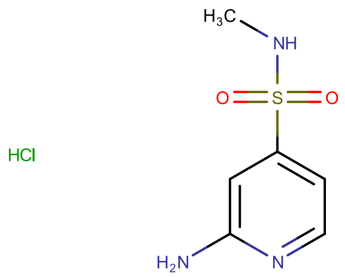

**1**{*133*}	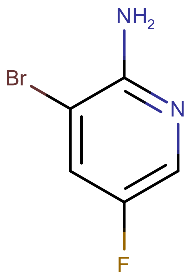
**1**{*134*}	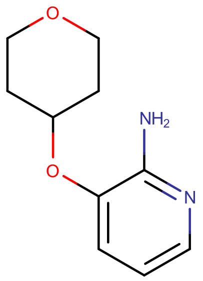
**1**{*135*}	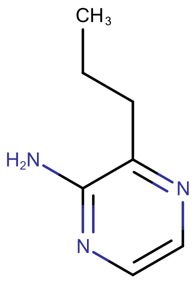
**1**{*136*}	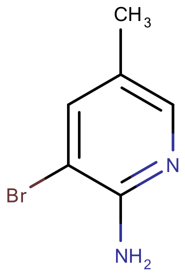

**1**{*137*}	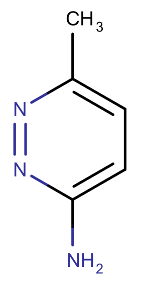
**1**{*138*}	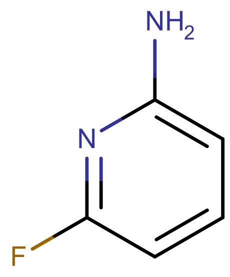
**1**{*139*}	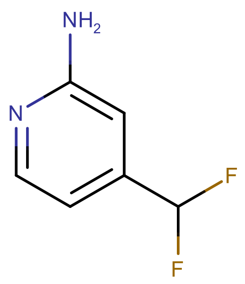
**1**{*140*}	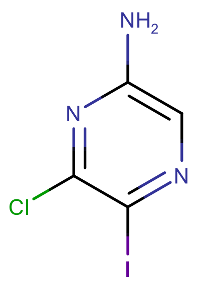
**1**{*141*}	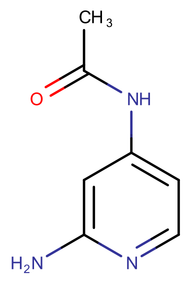
**1**{*142*}	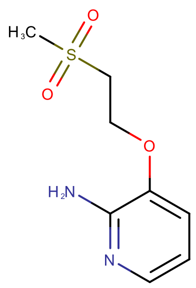
**1**{*143*}	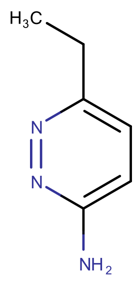
**1**{*144*}	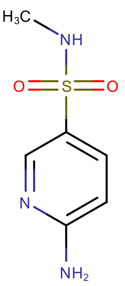

**1**{*145*}	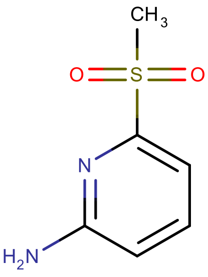
**1**{*146*}	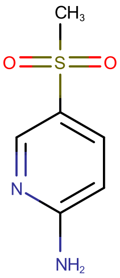
**1**{*147*}	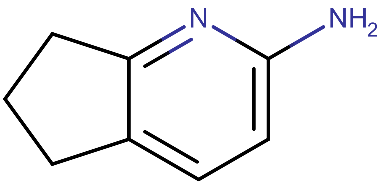
**1**{*148*}	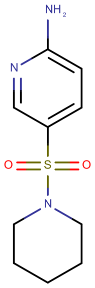

**1**{*149*}	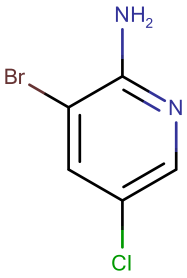
**1**{*150*}	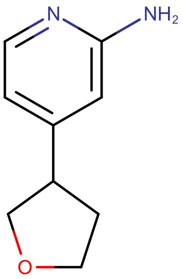
**1**{*151*}	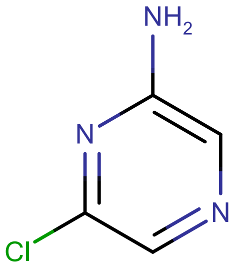
**1**{*152*}	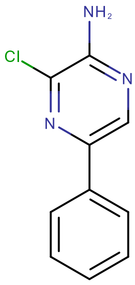

**1**{*153*}	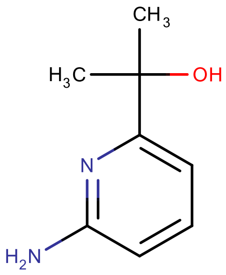
**1**{*154*}	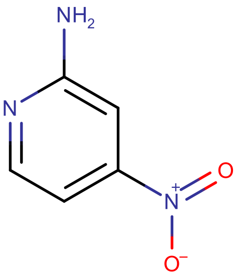
**1**{*155*}	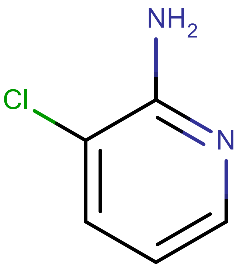
**1**{*156*}	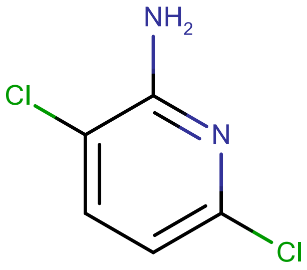

**1**{*157*}	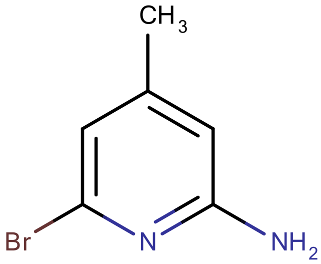
**1**{*158*}	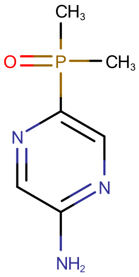
**1**{*159*}	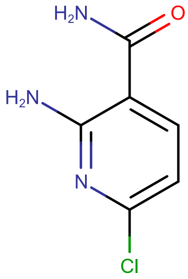
**1**{*160*}	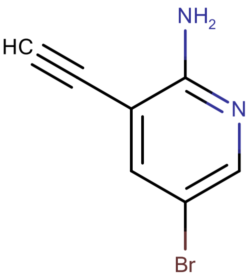

**1**{*161*}	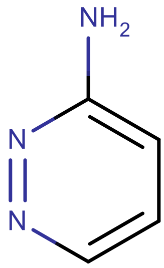
**1**{*162*}	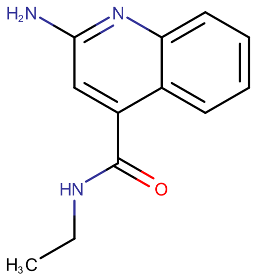
**1**{*163*}	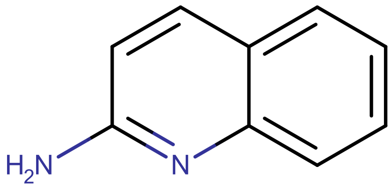
**1**{*164*}	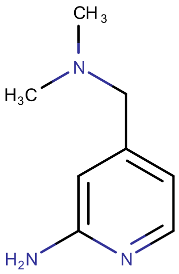

**1**{*165*}	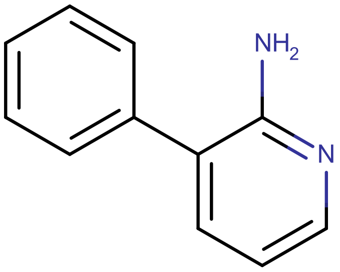
**1**{*166*}	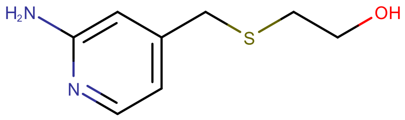
**1**{*167*}	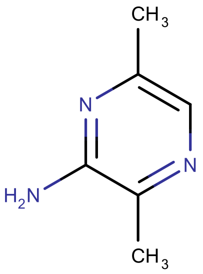
**1**{*168*}	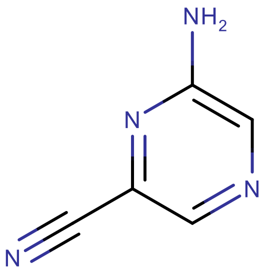

**1**{*169*}	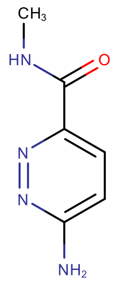
**1**{*170*}	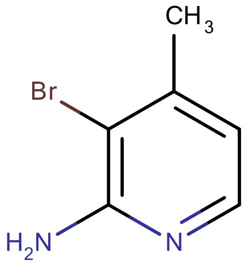
**1**{*171*}	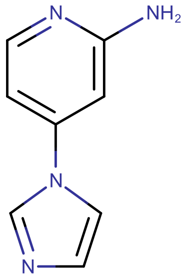
**1**{*172*}	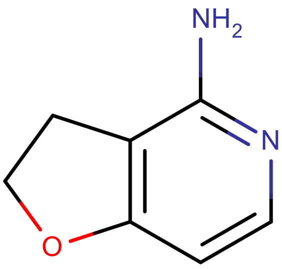

**1**{*173*}	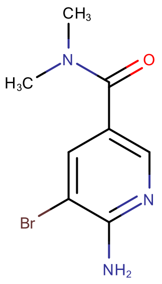
**1**{*174*}	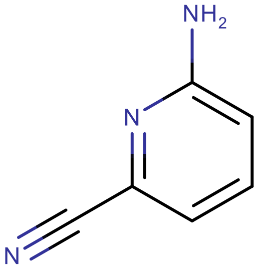
**1**{*175*}	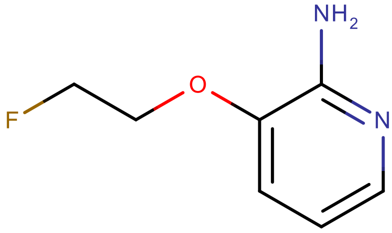
**1**{*176*}	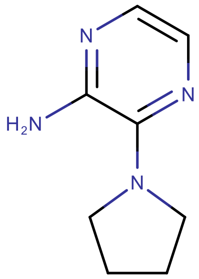

**1**{*177*}	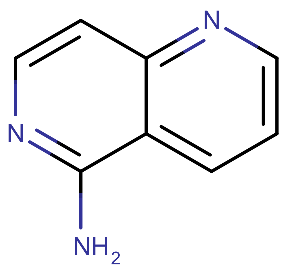
**1**{*178*}	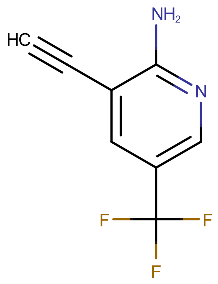
**1**{*179*}	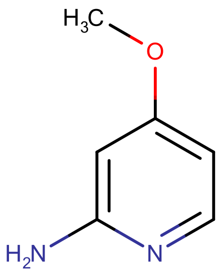
**1**{*180*}	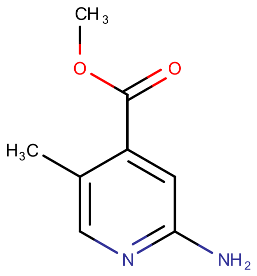

**1**{*181*}	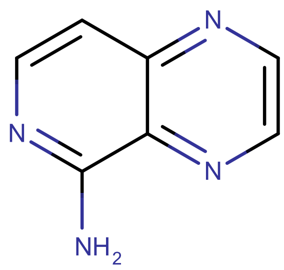
**1**{*182*}	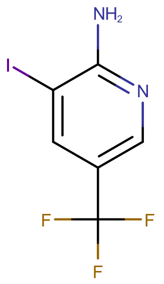
**1**{*183*}	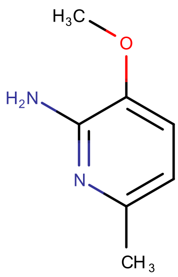
**1**{*184*}	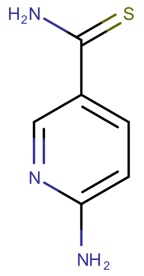

**1**{*185*}	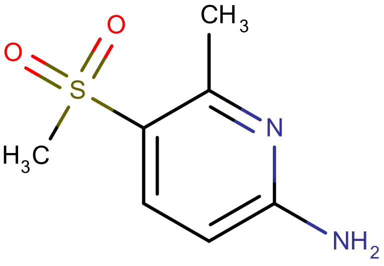
**1**{*186*}	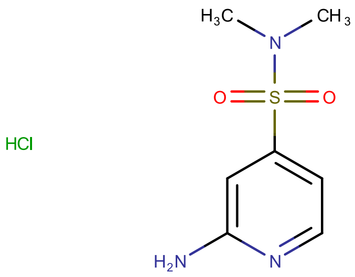
**1**{*187*}	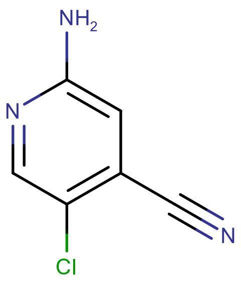
**1**{*188*}	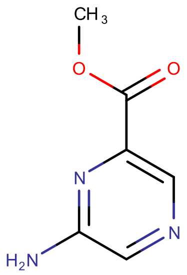

**1**{*189*}	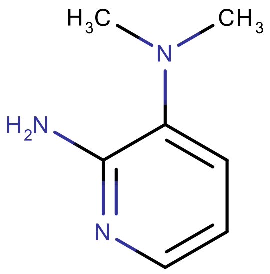
**1**{*190*}	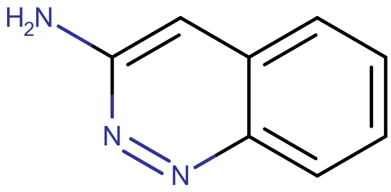
**1**{*191*}	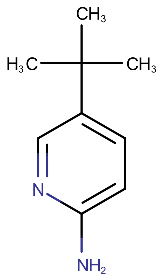
**1**{*192*}	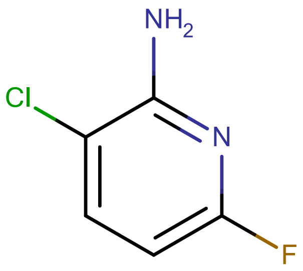

**1**{*193*}	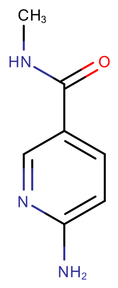
**1**{*194*}	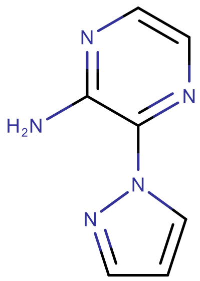
**1**{*195*}	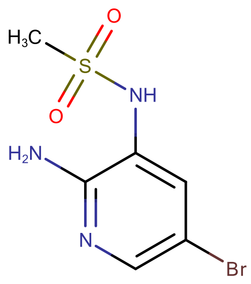
**1**{*196*}	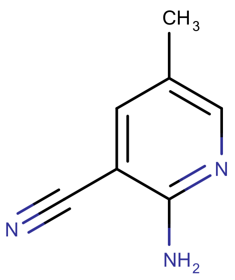

**1**{*197*}	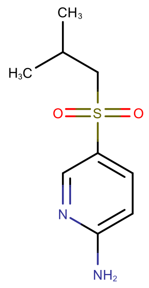
**1**{*198*}	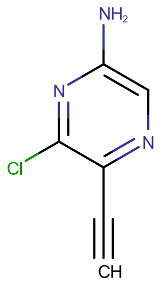
**1**{*199*}	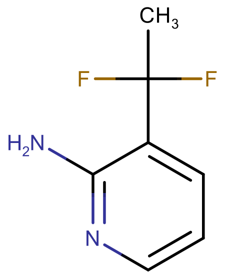
**1**{*200*}	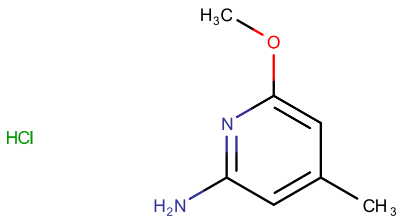

**1**{*201*}	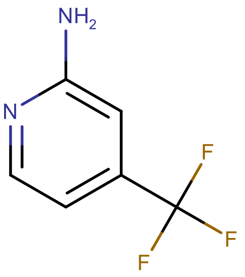
**1**{*202*}	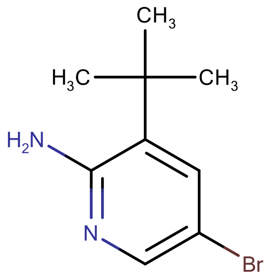
**1**{*203*}	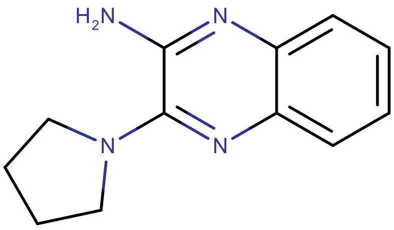
**1**{*204*}	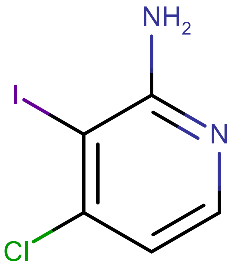

**1**{*205*}	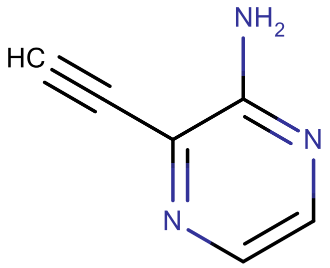
**1**{*206*}	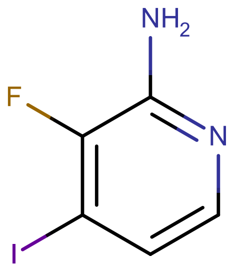
**1**{*207*}	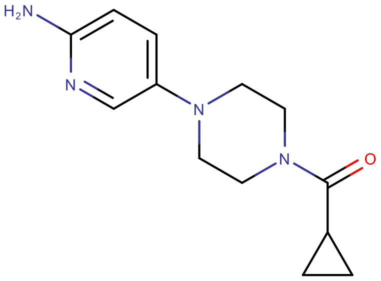
**1**{*208*}	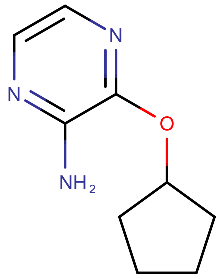

**1**{*209*}	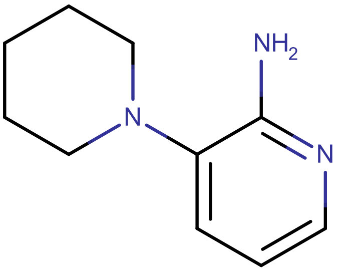
**1**{*210*}	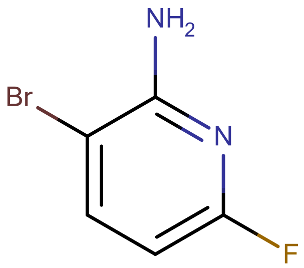
**1**{*211*}	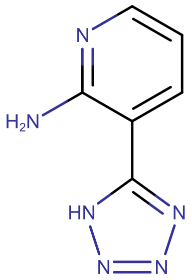
**1**{*212*}	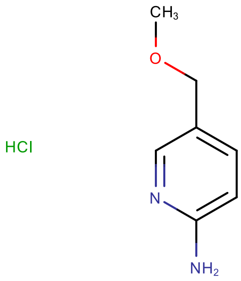

**1**{*213*}	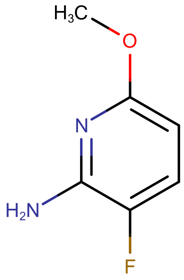
**1**{*214*}	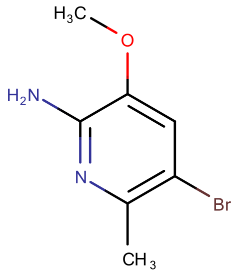
**1**{*215*}	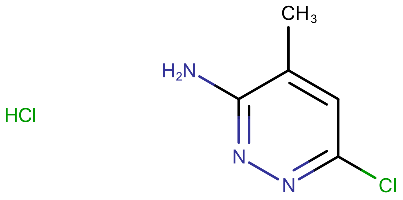
**1**{*216*}	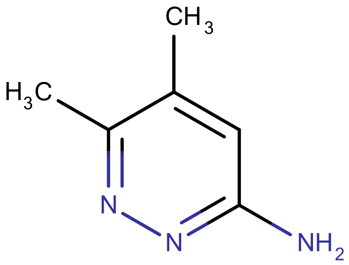

**1**{*217*}	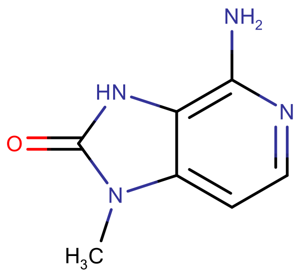	**1**{*221*}	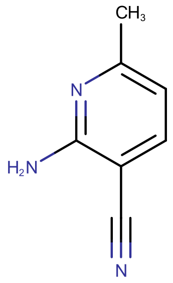
**1**{*218*}	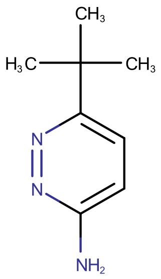	**1**{*222*}	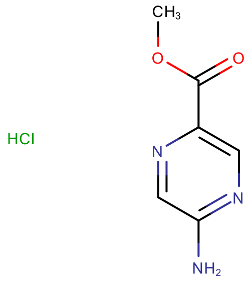
**1**{*219*}	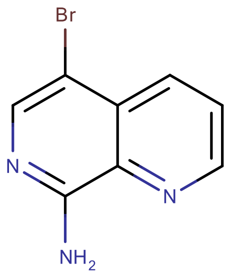	**1**{*223*}	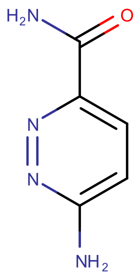
**1**{*220*}	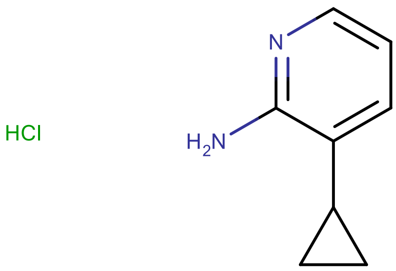	**1**{*224*}	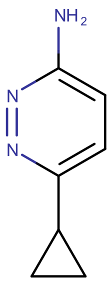

**1**{*225*}	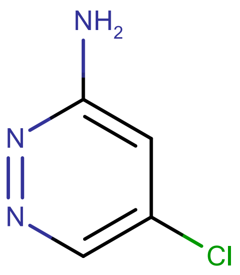
**1**{*226*}	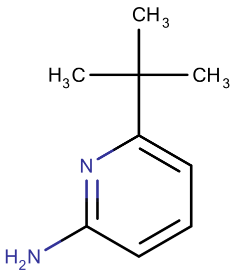
**1**{*227*}	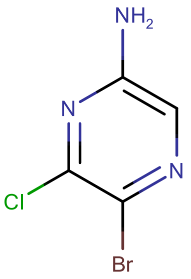
**1**{*228*}	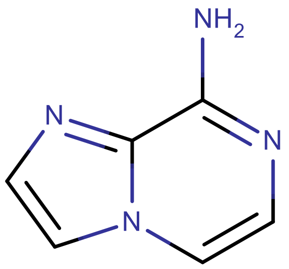

**1**{*229*}	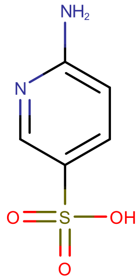
**1**{*230*}	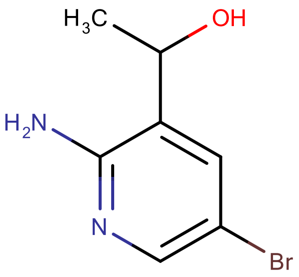
**1**{*231*}	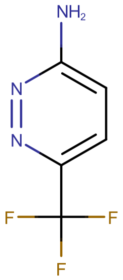
**1**{*232*}	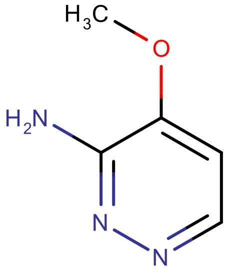

**1**{*233*}	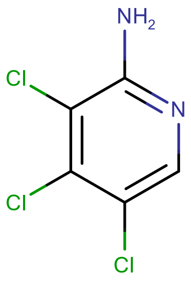
**1**{*234*}	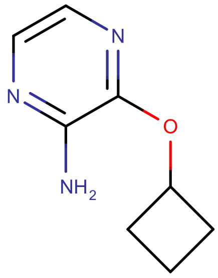
**1**{*235*}	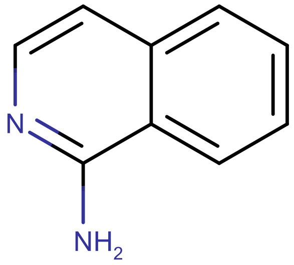
**1**{*236*}	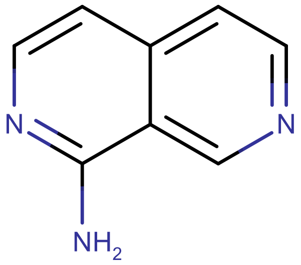

**1**{*237*}	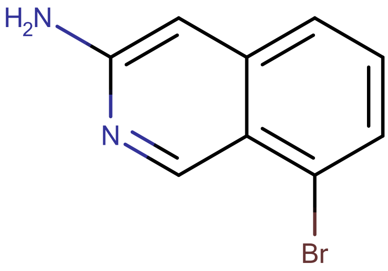
**1**{*238*}	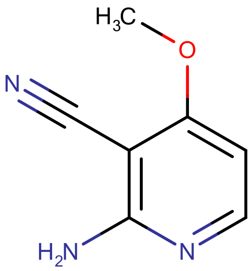
**1**{*239*}	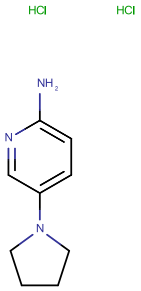
**1**{*240*}	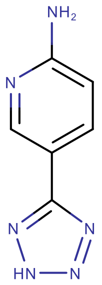

**1**{*241*}	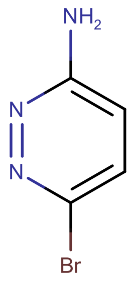
**1**{*242*}	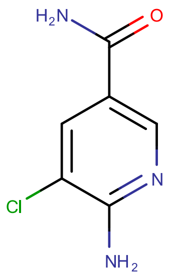
**1**{*243*}	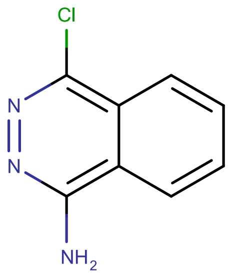
**1**{*244*}	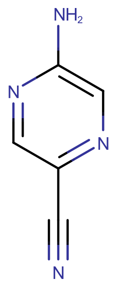

**1**{*245*}	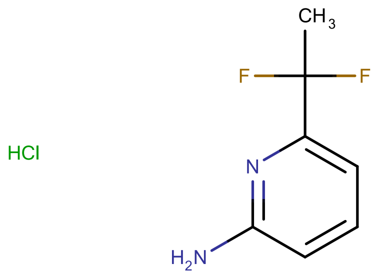
**1**{*246*}	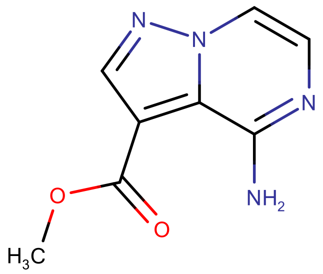
**1**{*247*}	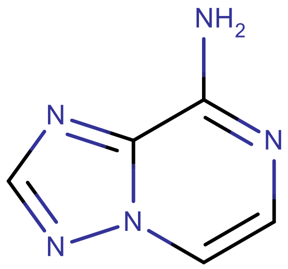
**1**{*248*}	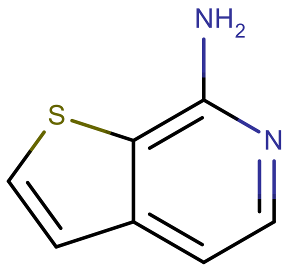

**1**{*249*}	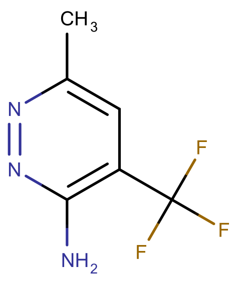	**1**{*253*}	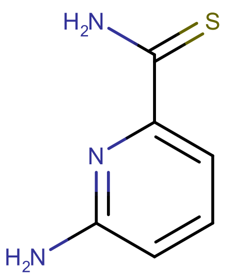
**1**{*250*}	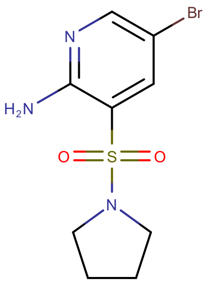	**1**{*254*}	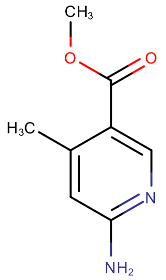
**1**{*251*}	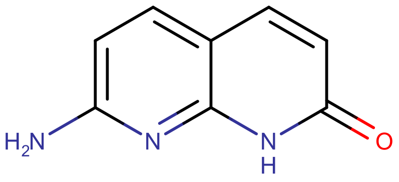	**1**{*255*}	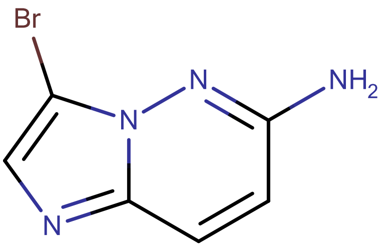
**1**{*252*}	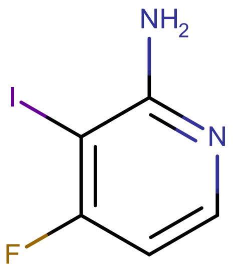	**1**{*256*}	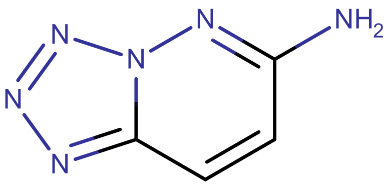

**1**{*257*}	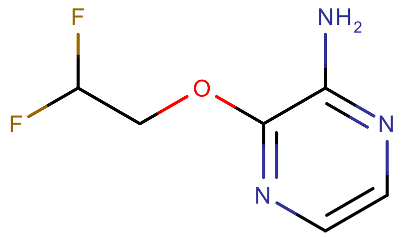
**1**{*258*}	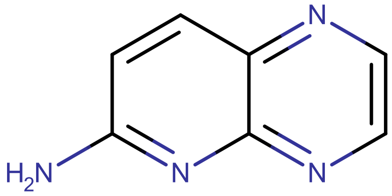
**1**{*259*}	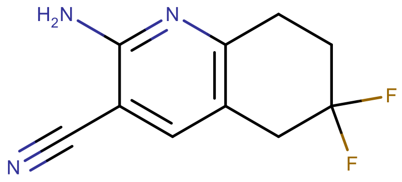
**1**{*260*}	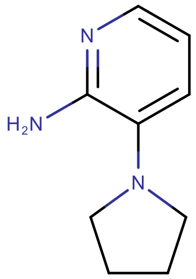

**1**{*261*}	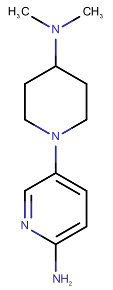
**1**{*262*}	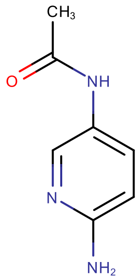
**1**{*263*}	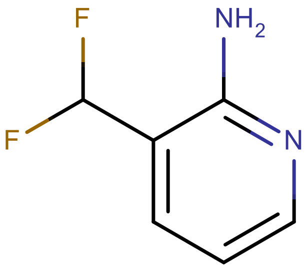
**1**{*264*}	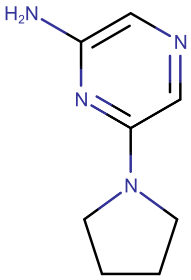

**1**{*265*}	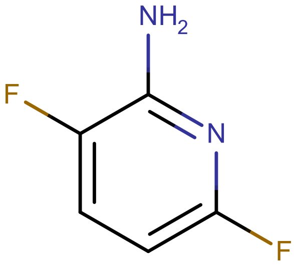
**1**{*266*}	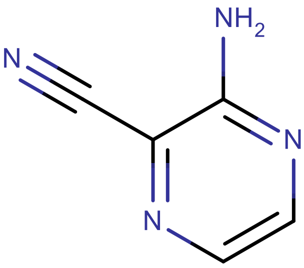
**1**{*267*}	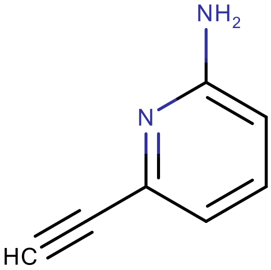
**1**{*268*}	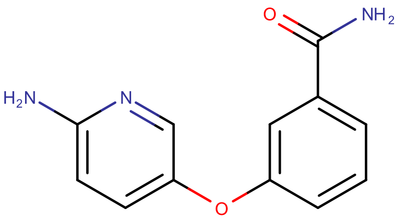

**1**{*269*}	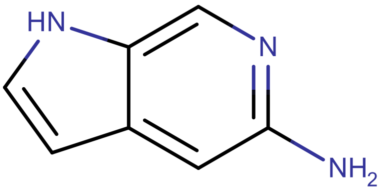
**1**{*270*}	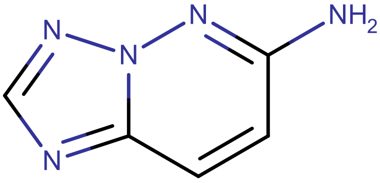
**1**{*271*}	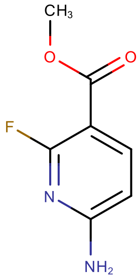
**1**{*272*}	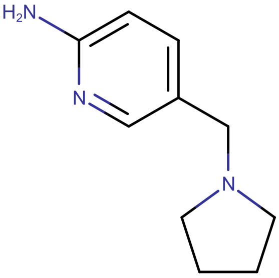

**1**{*273*}	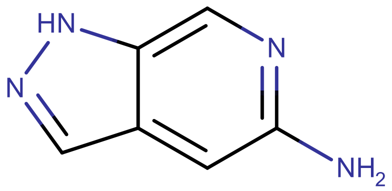
**1**{*274*}	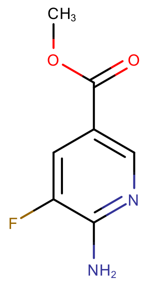
**1**{*275*}	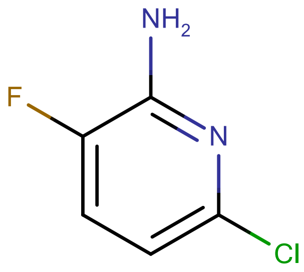
**1**{*276*}	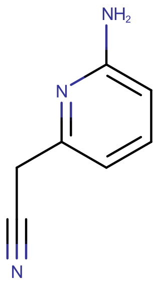

**1**{*277*}	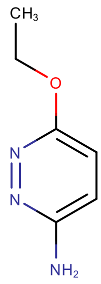
**1**{*278*}	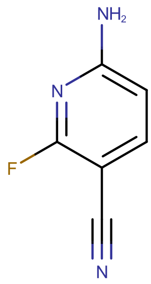
**1**{*279*}	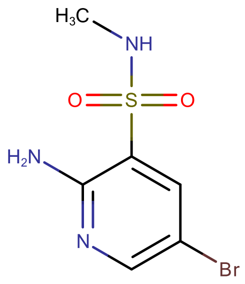
**1**{*280*}	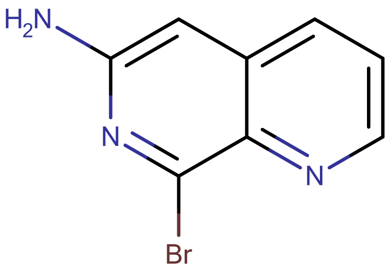

**1**{*281*}	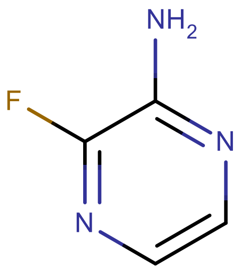
**1**{*282*}	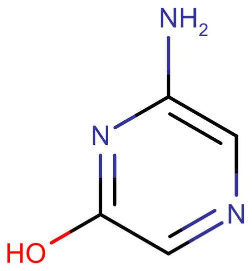
**1**{*283*}	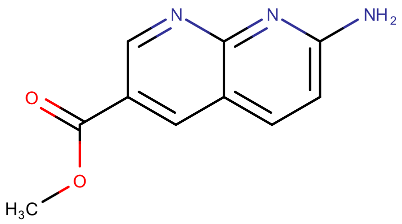
**1**{*284*}	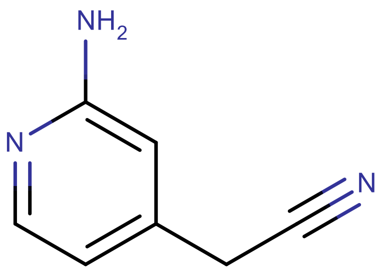
**1**{*285*}	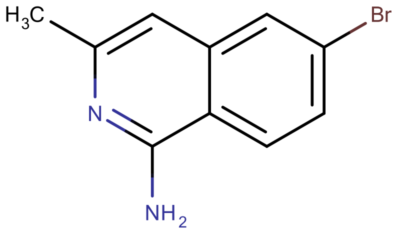
**1**{*286*}	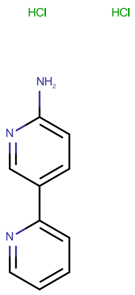
**1**{*287*}	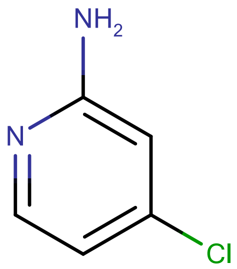
**1**{*288*}	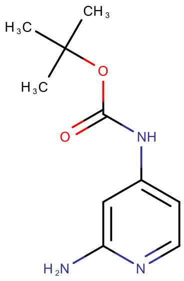

**1**{*289*}	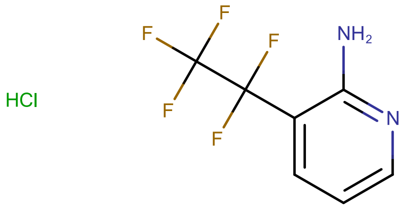
**1**{*290*}	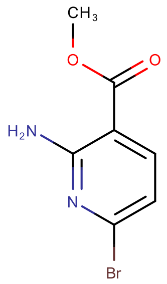
**1**{*291*}	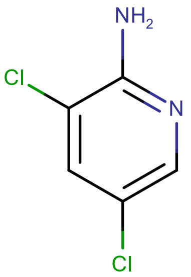
**1**{*292*}	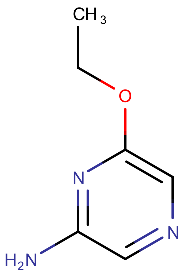

**1**{*293*}	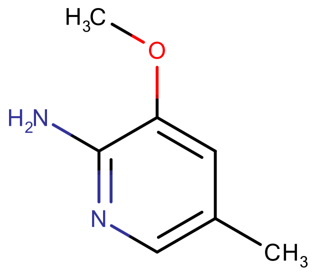
**1**{*294*}	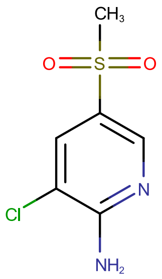
**1**{*295*}	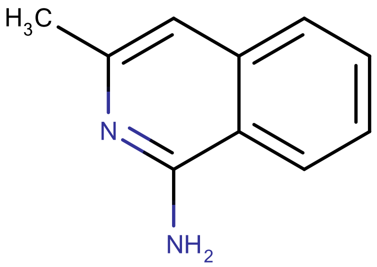
**1**{*296*}	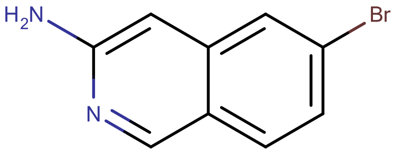

**1**{*297*}	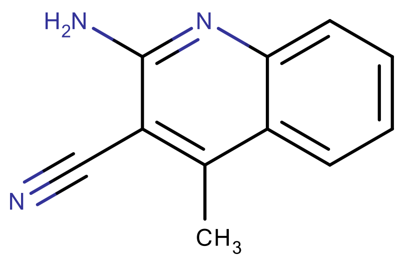	**1**{*301*}	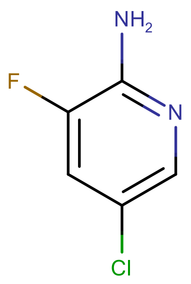
**1**{*298*}	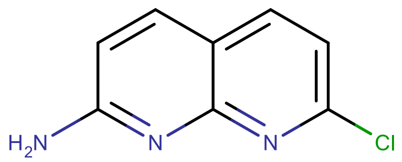	**1**{*302*}	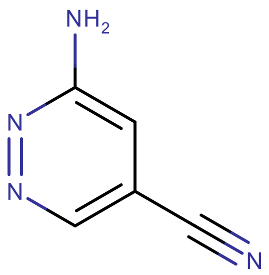
**1**{*299*}	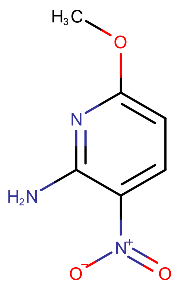	**1**{*303*}	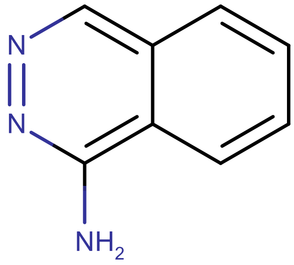
**1**{*300*}	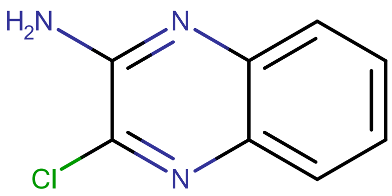	**1**{*304*}	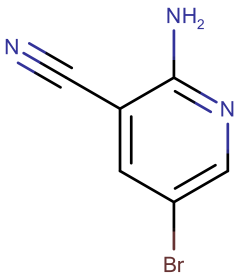

**1**{*305*}	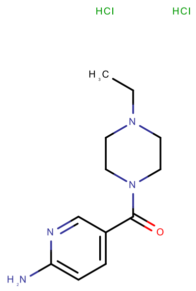
**1**{*306*}	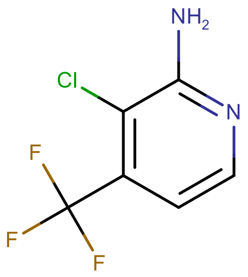
**1**{*307*}	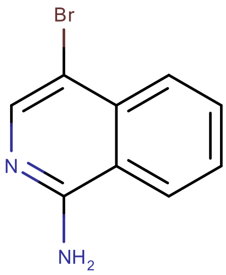
**1**{*308*}	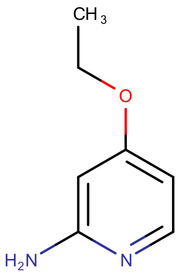

**1**{*309*}	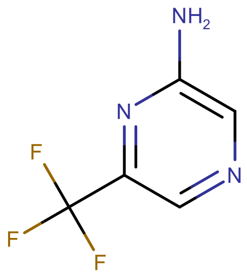
**1**{*310*}	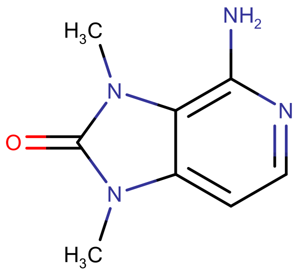
**1**{*311*}	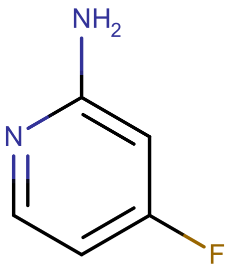
**1**{*312*}	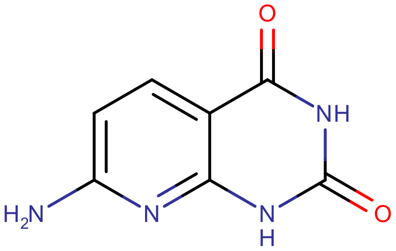

**1**{*313*}	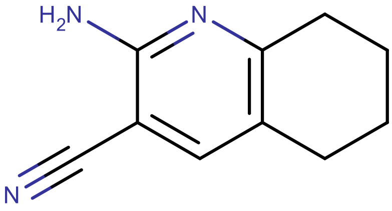
**1**{*314*}	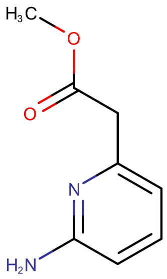
**1**{*315*}	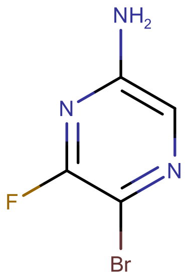
**1**{*316*}	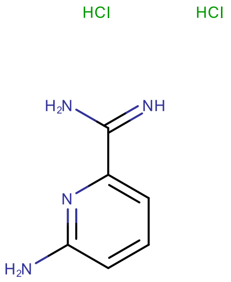

**1**{*317*}	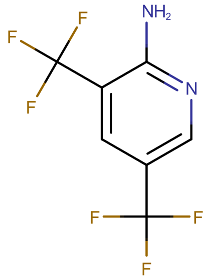
**1**{*318*}	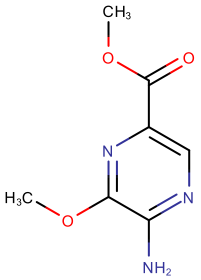
**1**{*319*}	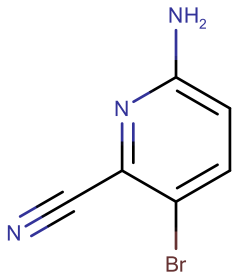
**1**{*320*}	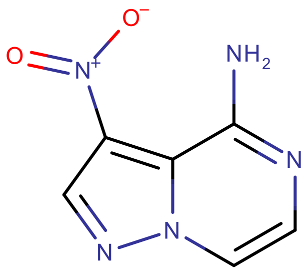

**1**{*321*}	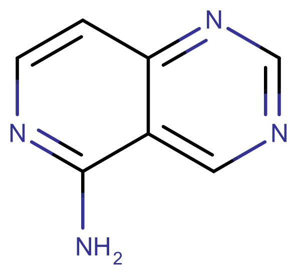
**1**{*322*}	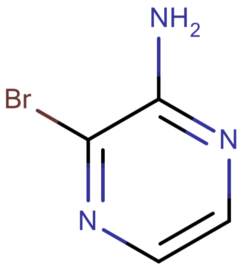
**1**{*323*}	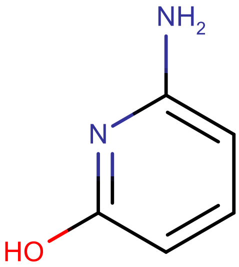
**1**{*324*}	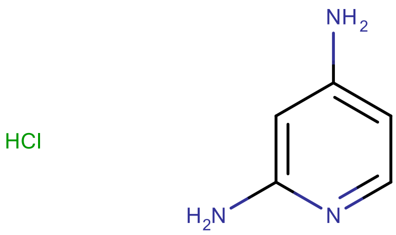

**1**{*325*}	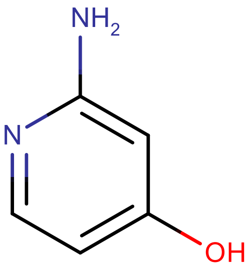
**1**{*326*}	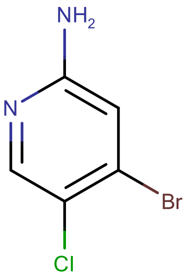
**1**{*327*}	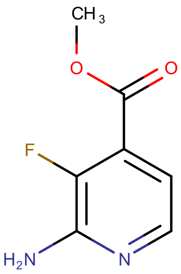
**1**{*328*}	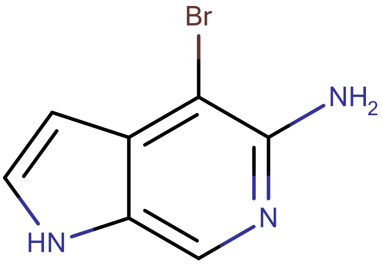

1{*329*}

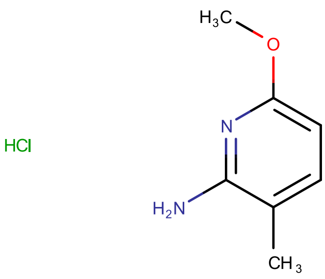

1{*330*}

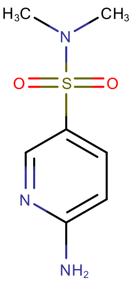

1{*331*}

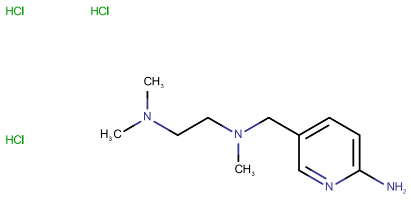

1{*332*}

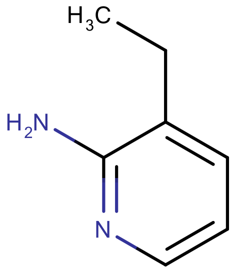

1{*333*}

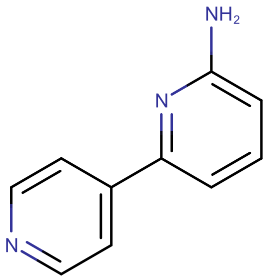

1{*334*}

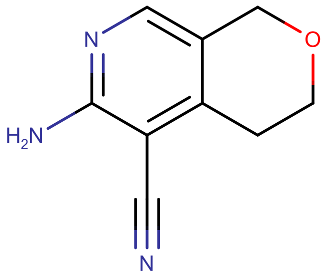

1{*335*}

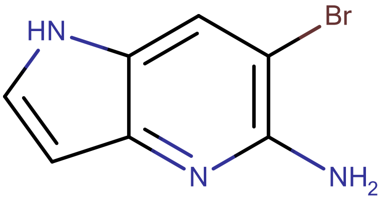

1{*336*}

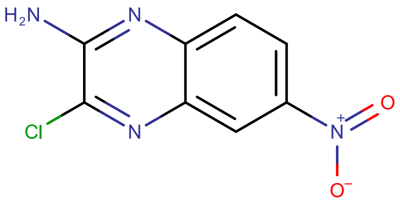

**1**{*337*}	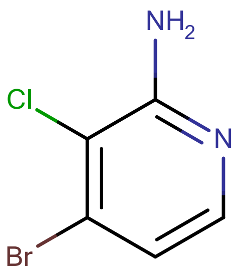
**1**{*338*}	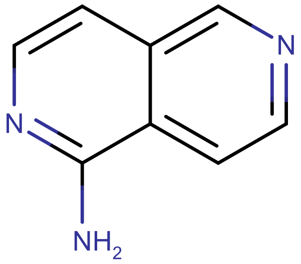
**1**{*339*}	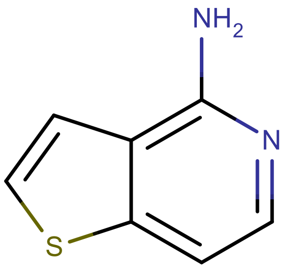
**1**{*340*}	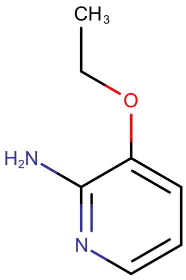

**1**{*341*}	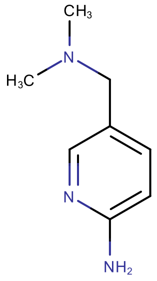
**1**{*342*}	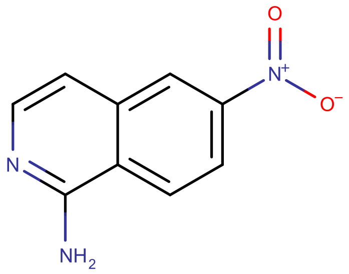
**1**{*343*}	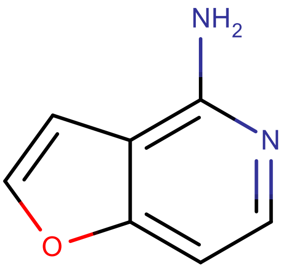
**1**{*344*}	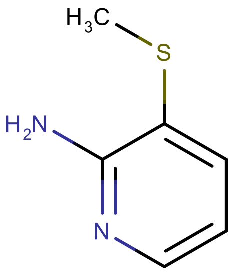

**1**{*345*}	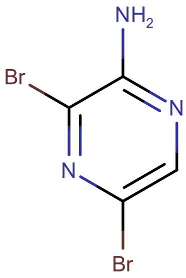
**1**{*346*}	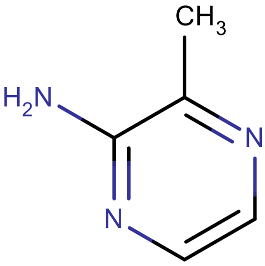
**1**{*347*}	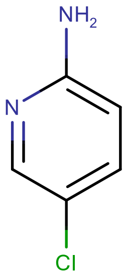
**1**{*348*}	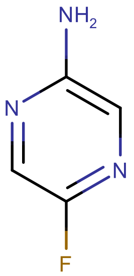

**1**{*349*}	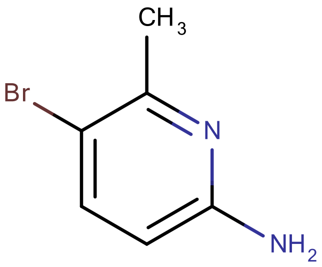
**1**{*350*}	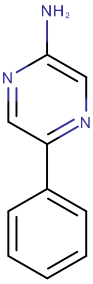
**1**{*351*}	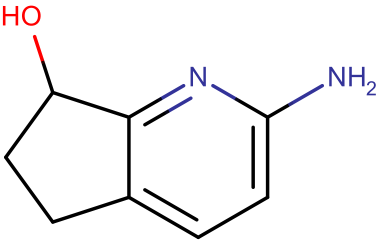
**1**{*352*}	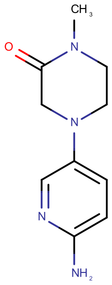

**1**{*353*}	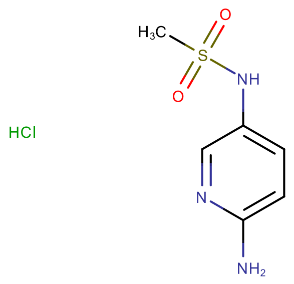	**1**{*357*}	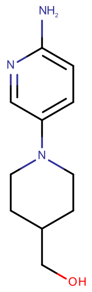
**1**{*354*}	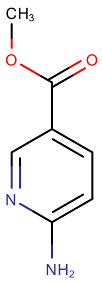	**1**{*358*}	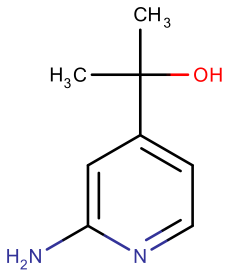
**1**{*355*}	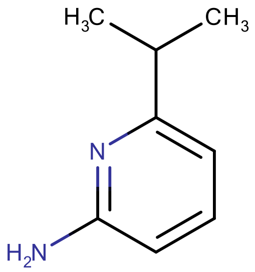	**1**{*359*}	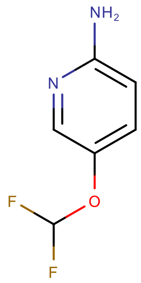
**1**{*356*}	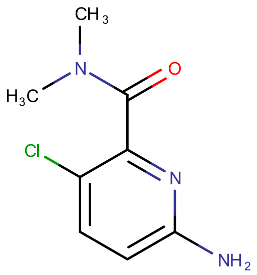	**1**{*360*}	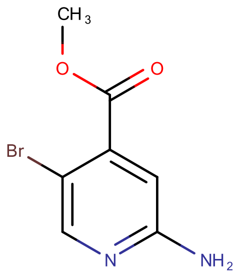

**1**{*361*}	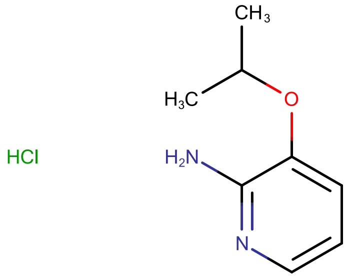
**1**{*362*}	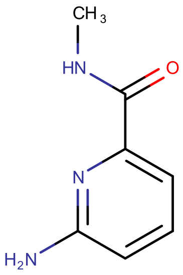
**1**{*363*}	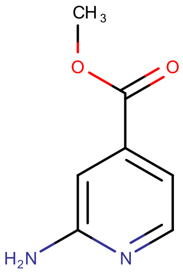
**1**{*364*}	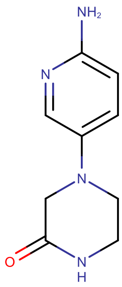

**1**{*365*}	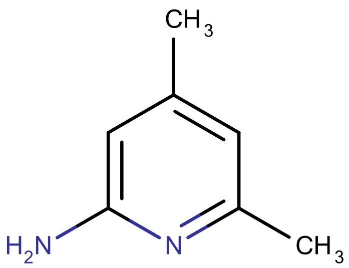
**1**{*366*}	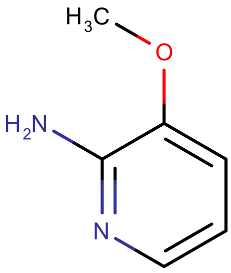
**1**{*367*}	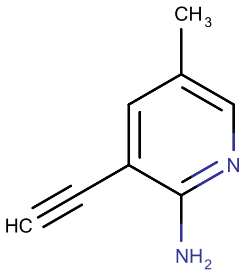
**1**{*368*}	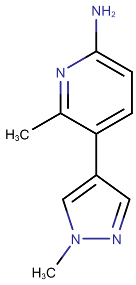

**1**{*369*}	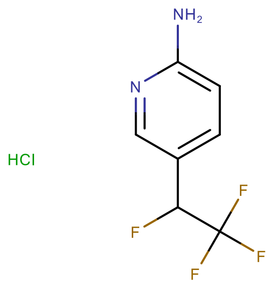
**1**{*370*}	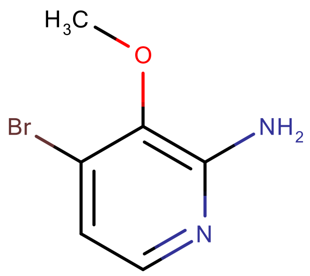
**1**{*371*}	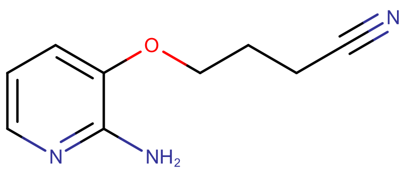
**1**{*372*}	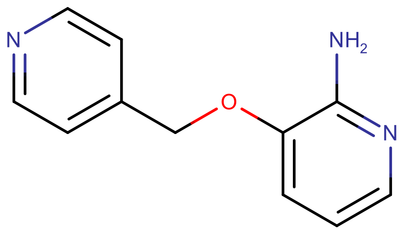

**1**{*373*}	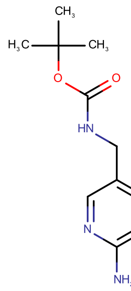
**1**{*374*}	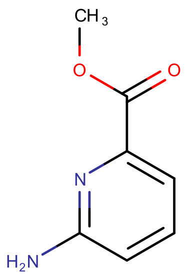
**1**{*375*}	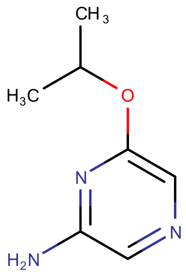
**1**{*376*}	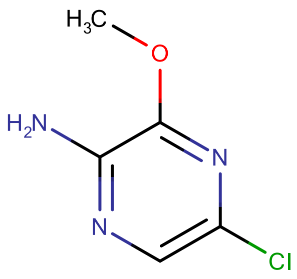

**1**{*377*}	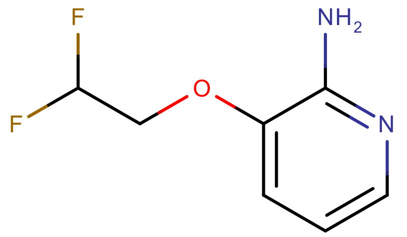
**1**{*378*}	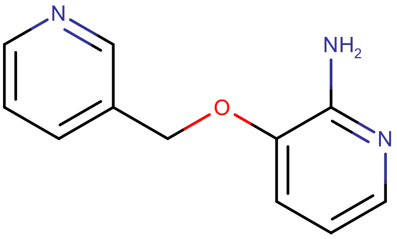
**1**{*379*}	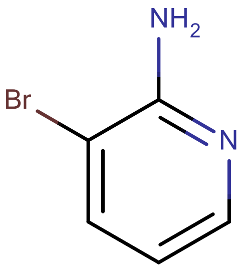
**1**{*380*}	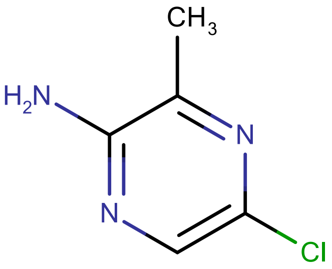

**1**{*381*}	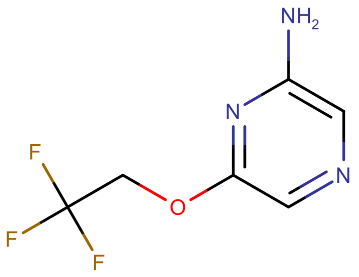
**1**{*382*}	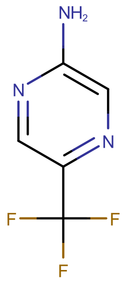
**1**{*383*}	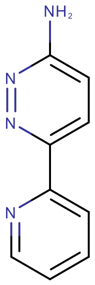
**1**{*384*}	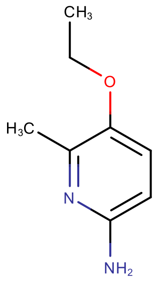

**1**{*385*}	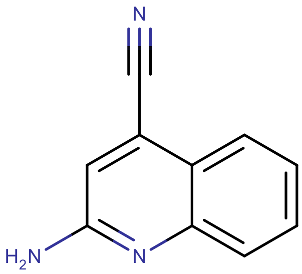
**1**{*386*}	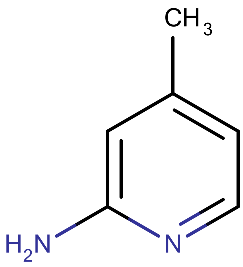
**1**{*387*}	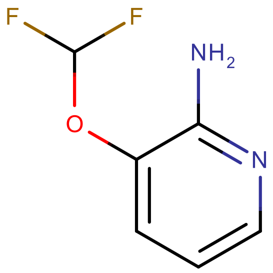
**1**{*388*}	HCl 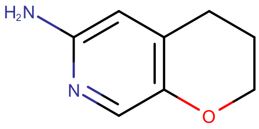

**1**{*389*}	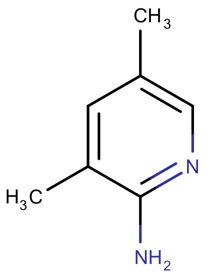
**1**{*390*}	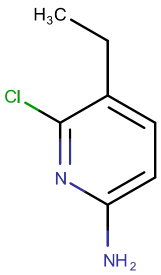
**1**{*391*}	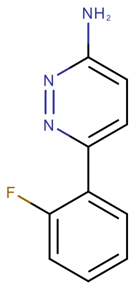
**1**{*392*}	HCl 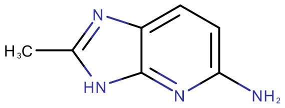

**1**{*393*}	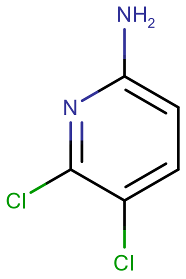	**1**{*397*}	HCl 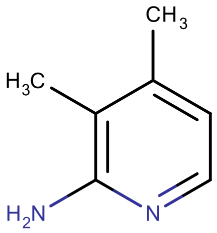
**1**{*394*}	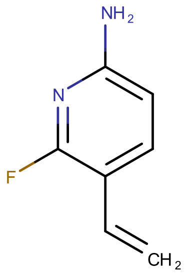	**1**{*398*}	HCl 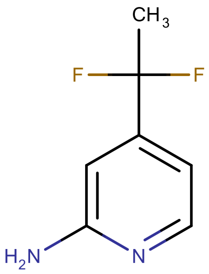
**1**{*395*}	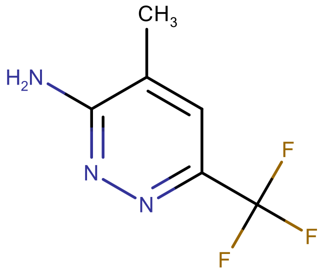	**1**{*399*}	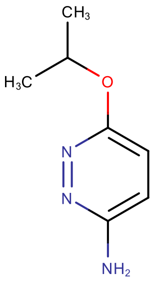
**1**{*396*}	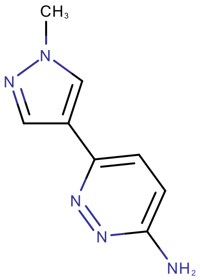	**1**{*400*}	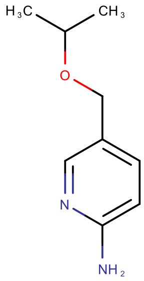

**1**{*401*}	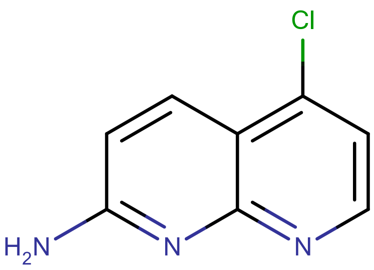
**1**{*402*}	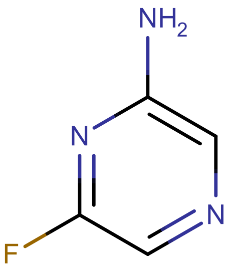
**1**{*403*}	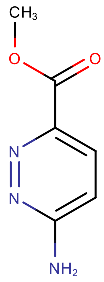
**1**{*404*}	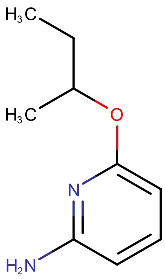

**1**{*405*}	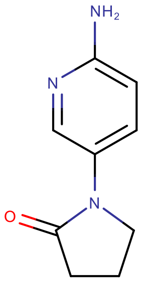
**1**{*406*}	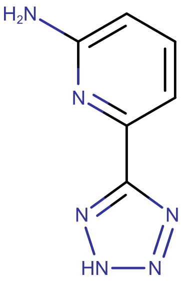
**1**{*407*}	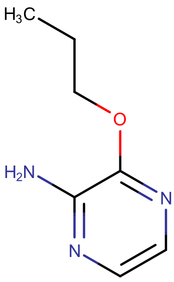
**1**{*408*}	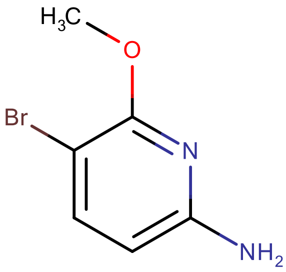

**1**{*409*}	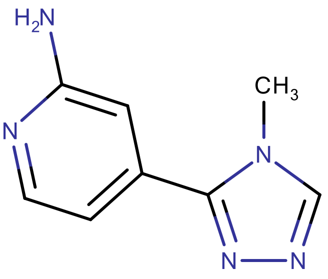
**1**{*410*}	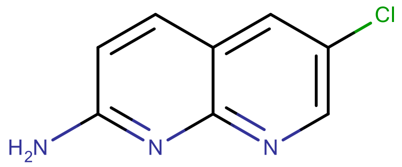
**1**{*411*}	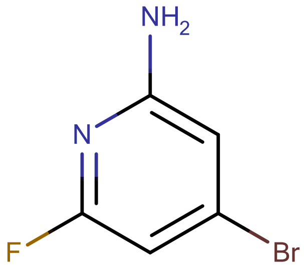
**1**{*412*}	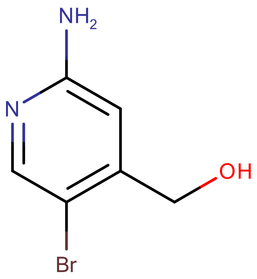

**1**{*413*}	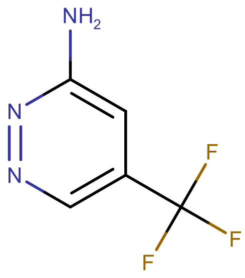
**1**{*414*}	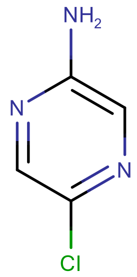
**1**{*415*}	HCl 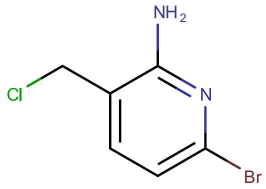
**1**{*416*}	HCl 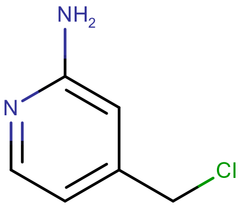

**1**{*417*}	HCl 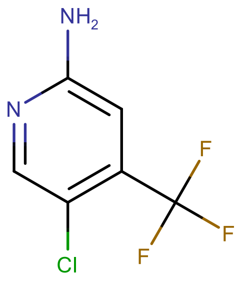
**1**{*418*}	HBr 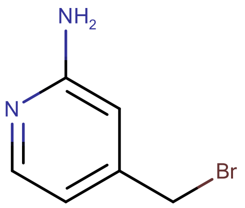
**1**{*419*}	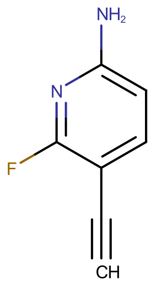
**1**{*420*}	HCl HCl 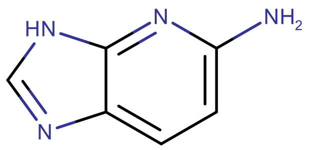
**1**{*421*}	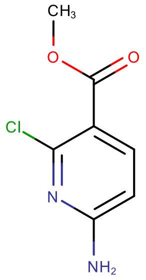
**1**{*422*}	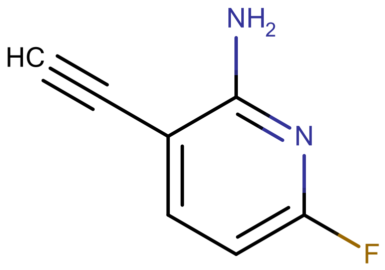
**1**{*423*}	HCl 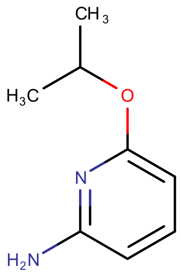
**1**{*424*}	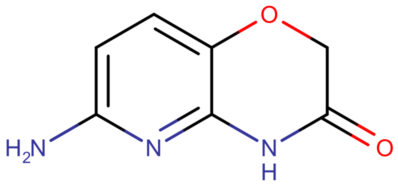

**1**{*425*}	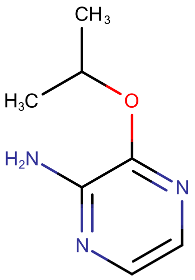	**1**{*429*}	HBr 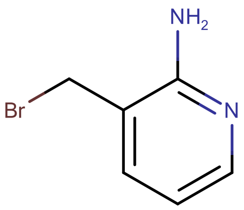
**1**{*426*}	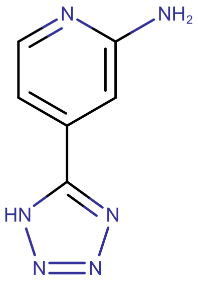	**1**{*430*}	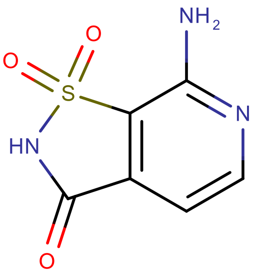
**1**{*427*}	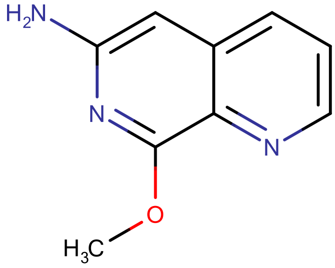	**1**{*431*}	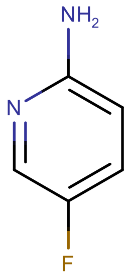
**1**{*428*}	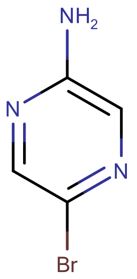	**1**{*432*}	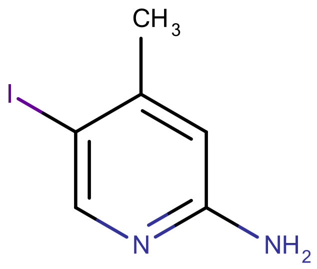

**1**{*433*}	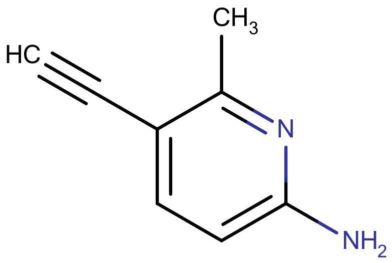
**1**{*434*}	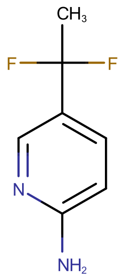
**1**{*435*}	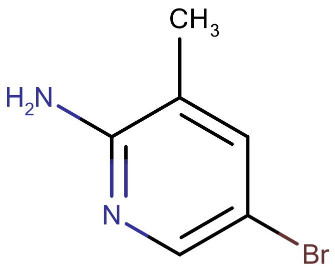
**1**{*436*}	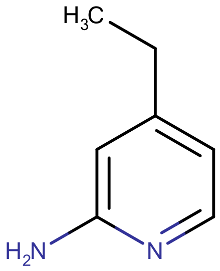

**1**{*437*}	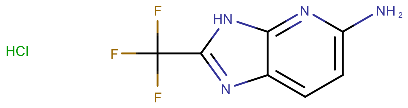
**1**{*438*}	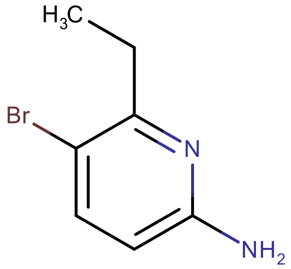
**1**{*439*}	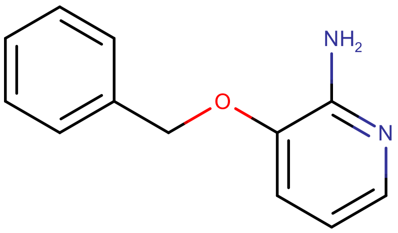
**1**{*440*}	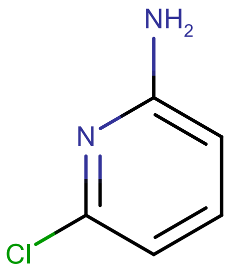

**1**{*441*}	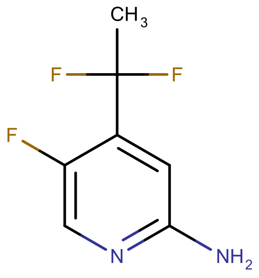
**1**{*442*}	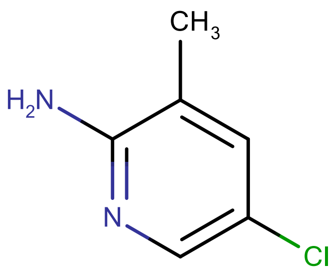
**1**{*443*}	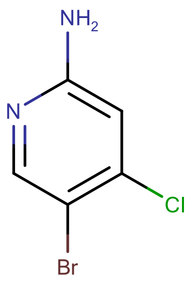
**1**{*444*}	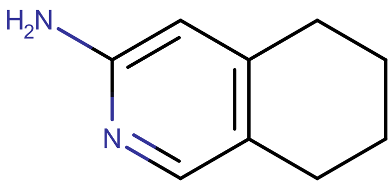

**1**{*445*}	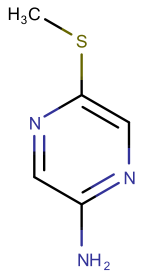
**1**{*446*}	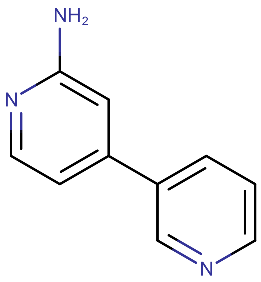
**1**{*447*}	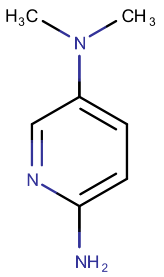
**1**{*448*}	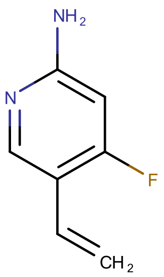

**1**{*449*}	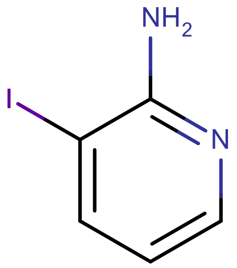
**1**{*450*}	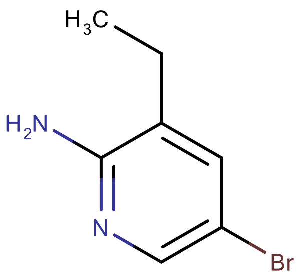
**1**{*451*}	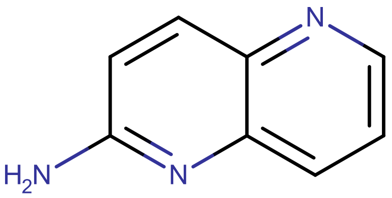
**1**{*452*}	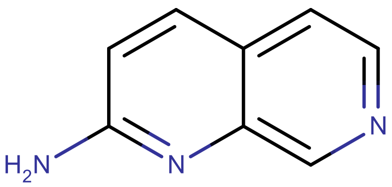

**1**{*453*}	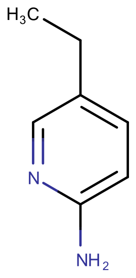
**1**{*454*}	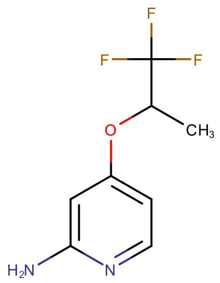
**1**{*455*}	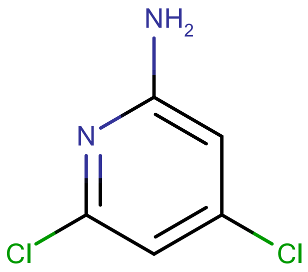
**1**{*456*}	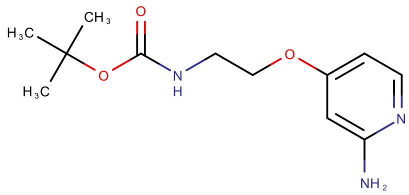

**1**{*457*}	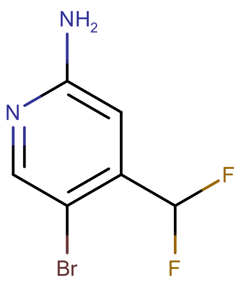
**1**{*458*}	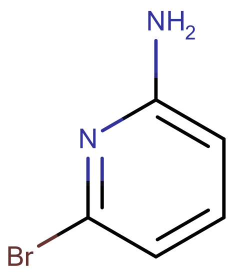
**1**{*459*}	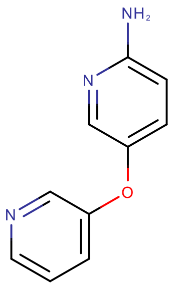
**1**{*460*}	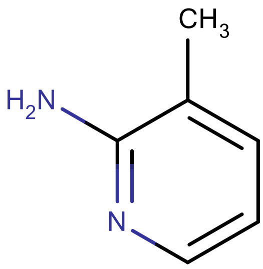

**1**{*461*}	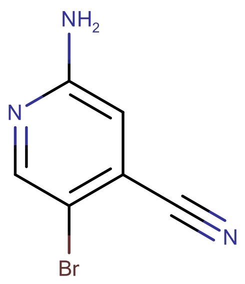
**1**{*462*}	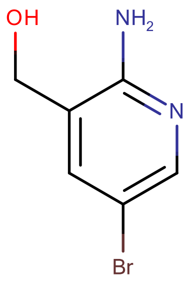
**1**{*463*}	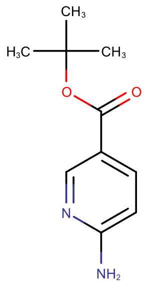
**1**{*464*}	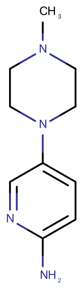

**1**{*465*}	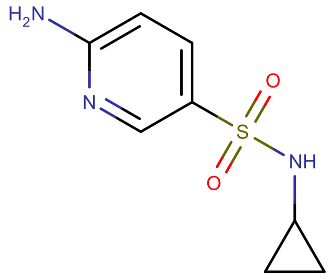
**1**{*466*}	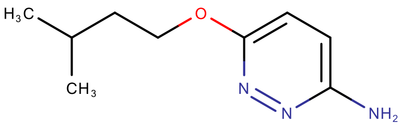
**1**{*467*}	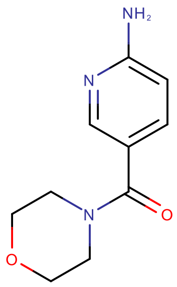
**1**{*468*}	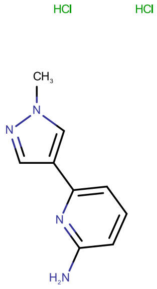

**1**{*469*}	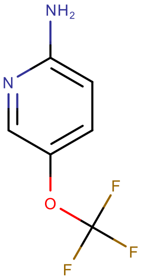
**1**{*470*}	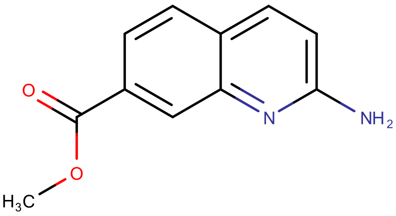
**1**{*471*}	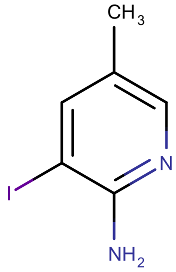
**1**{*472*}	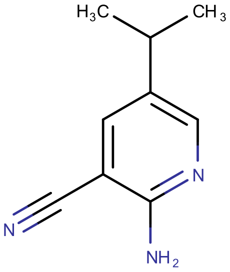

**1**{*473*}	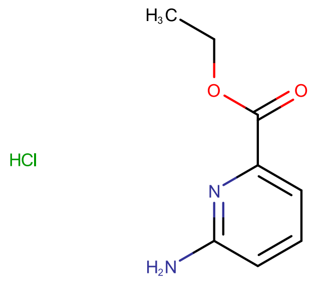
**1**{*474*}	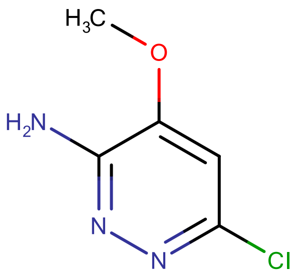
**1**{*475*}	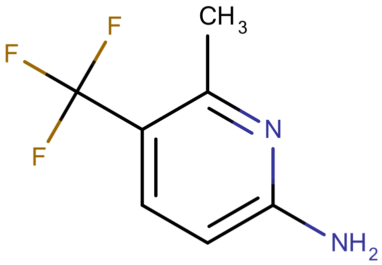
**1**{*476*}	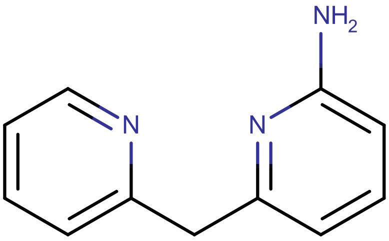

**1**{*477*}	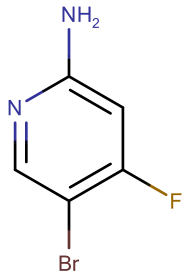
**1**{*478*}	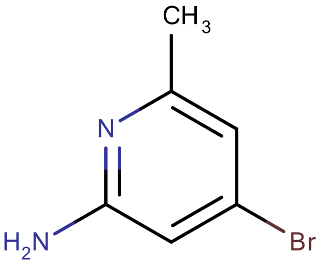
**1**{*479*}	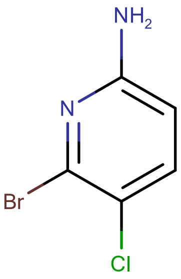
**1**{*480*}	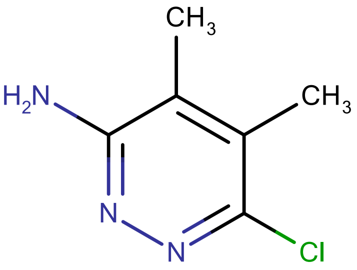

**1**{*481*}	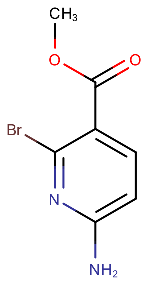
**1**{*482*}	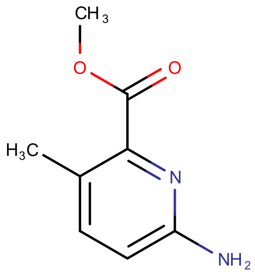
**1**{*483*}	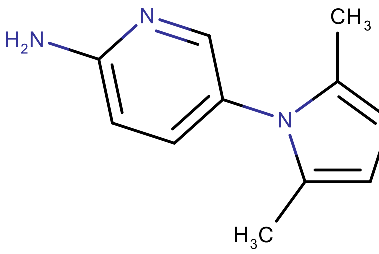
**1**{*484*}	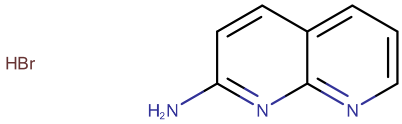

**1**{*485*}	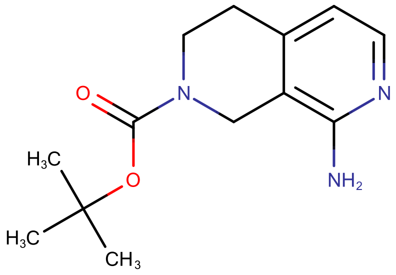
