# Supplementary material for: Generation of multimillion chemical space based on the parallel Groebke–Blackburn–Bienaymé reaction
Source: Beilstein J Org Chem. 2024 Jul 16;20:1604–13. doi: 10.3762/bjoc.20.143 (PMC11285076; doi:10.3762/bjoc.20.143)
Supplement: File 1 — Structures of reactants 1, 2, and 3. [file Beilstein_J_Org_Chem-20-1604-s001.zip › Structures of substrates 2.pdf]

Structures of reactants 2

ID	Structure
**2**{*1*}	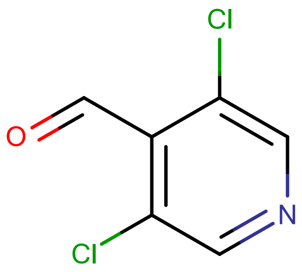
**2**{*2*}	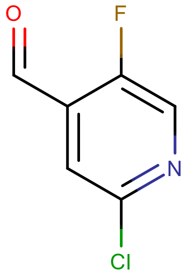
**2**{*3*}	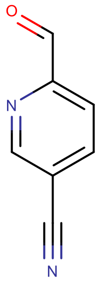
**2**{*4*}	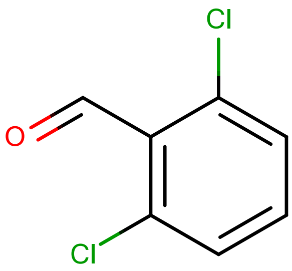
**2**{*5*}	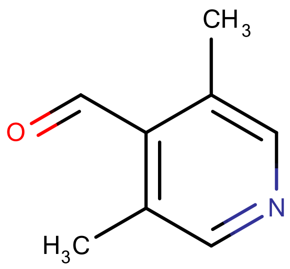
**2**{*6*}	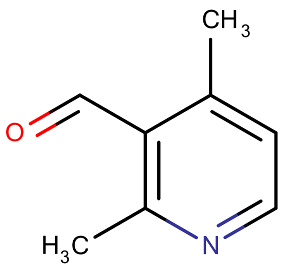
**2**{*7*}	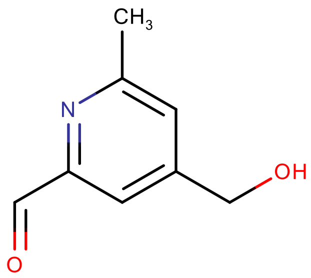
**2**{*8*}	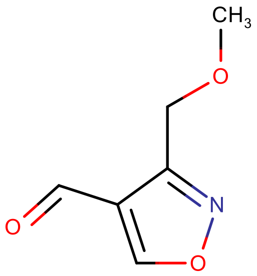

**2**{*9*}	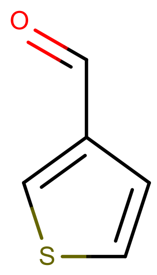
**2**{*10*}	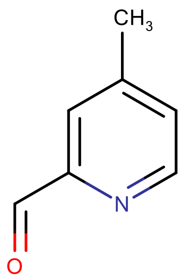
**2**{*11*}	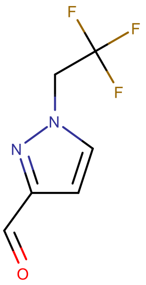
**2**{*12*}	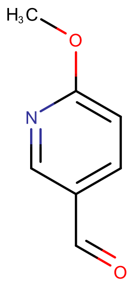

**2**{*13*}	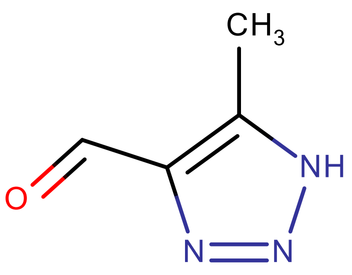
**2**{*14*}	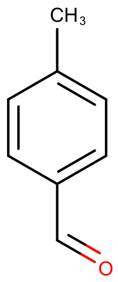
**2**{*15*}	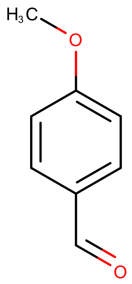
**2**{*16*}	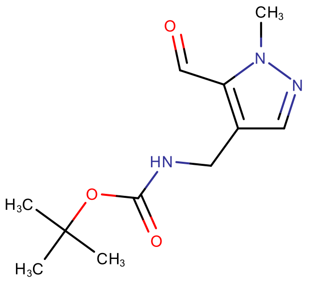

**2**{*17*}	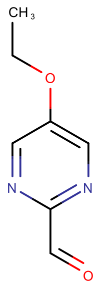
**2**{*18*}	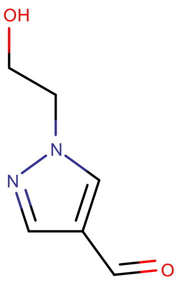
**2**{*19*}	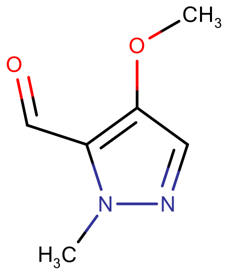
**2**{*20*}	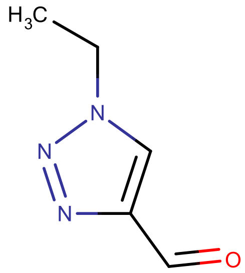

**2**{*21*}	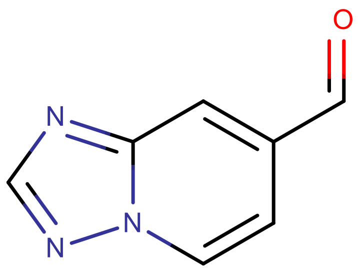
**2**{*22*}	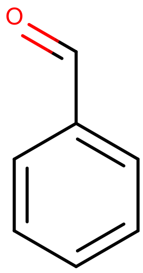
**2**{*23*}	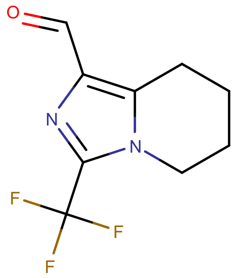
**2**{*24*}	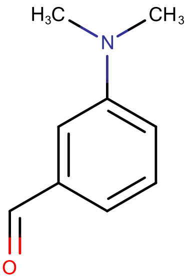

**2**{*25*}	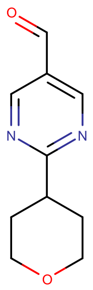
**2**{*26*}	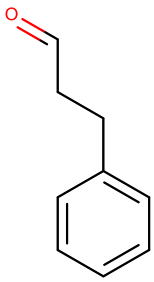
**2**{*27*}	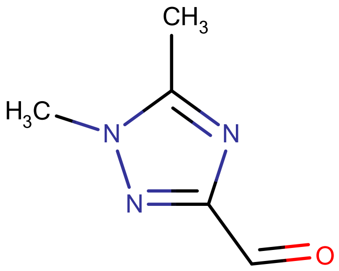
**2**{*28*}	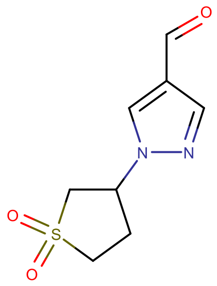

**2**{*29*}	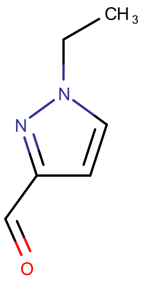
**2**{*30*}	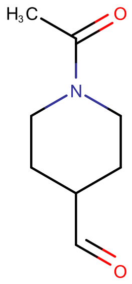
**2**{*31*}	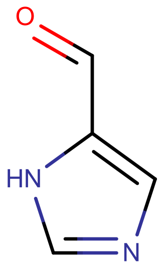
**2**{*32*}	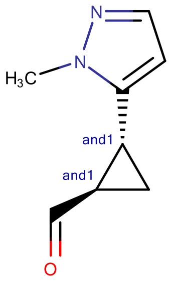

2{*33*}

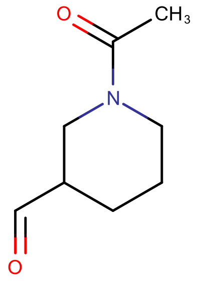

2{*34*}

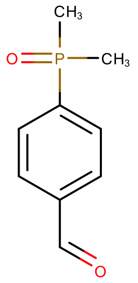

2{*35*}

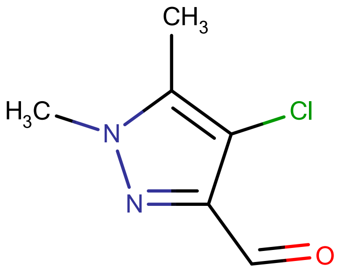

2{*36*}

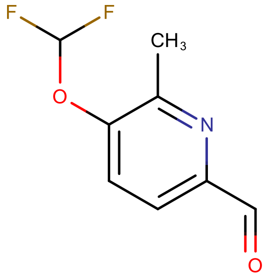

2{*37*}

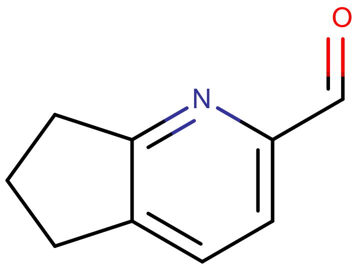

2{*38*}

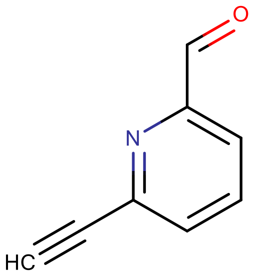

2{*39*}

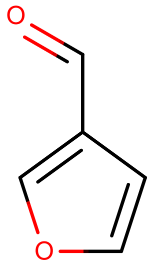

2{*40*}

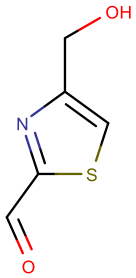

**2**{*41*}	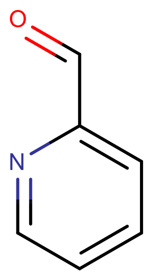
**2**{*42*}	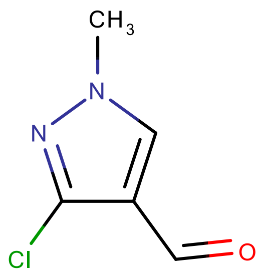
**2**{*43*}	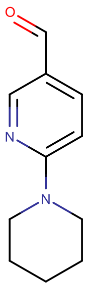
**2**{*44*}	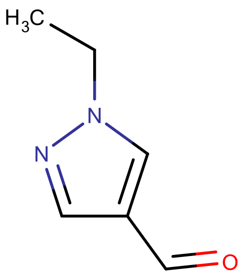

**2**{*45*}	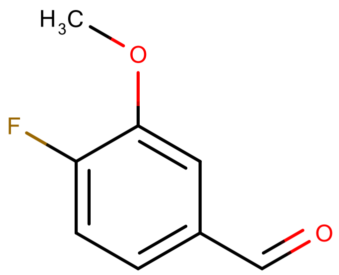
**2**{*46*}	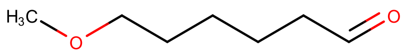
**2**{*47*}	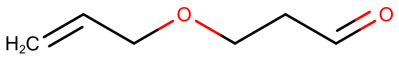
**2**{*48*}	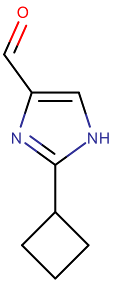

**2**{*49*}	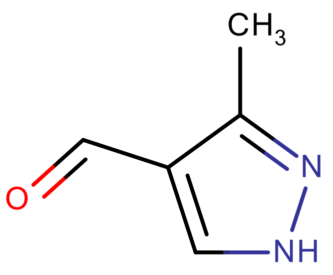
**2**{*50*}	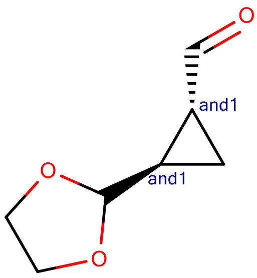
**2**{*51*}	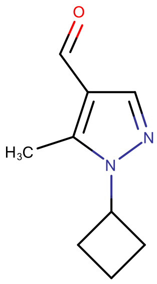
**2**{*52*}	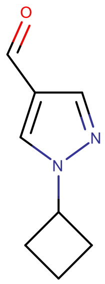

**2**{*53*}	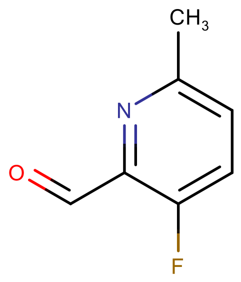
**2**{*54*}	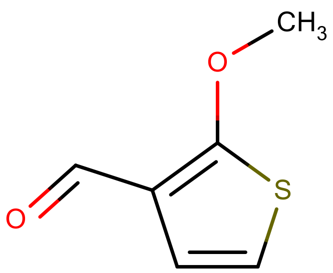
**2**{*55*}	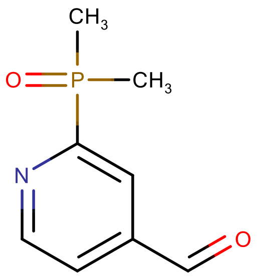
**2**{*56*}	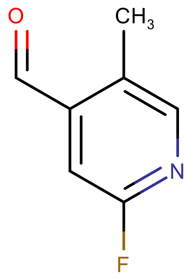

**2**{*57*}	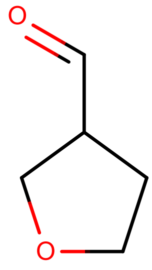
**2**{*58*}	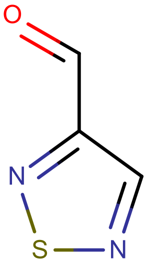
**2**{*59*}	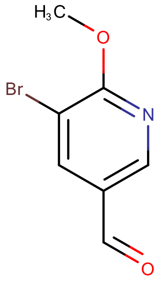
**2**{*60*}	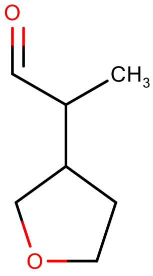

**2**{*61*}	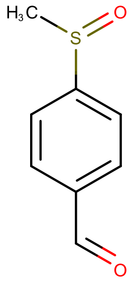
**2**{*62*}	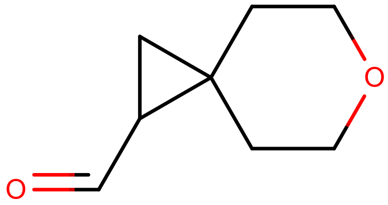
**2**{*63*}	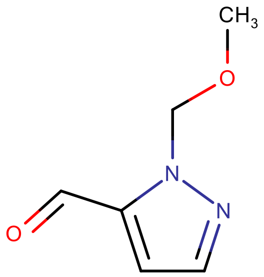
**2**{*64*}	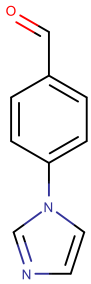

**2**{*65*}	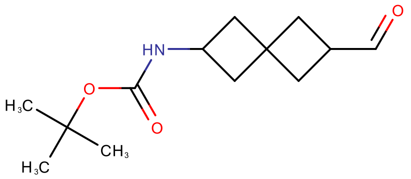
**2**{*66*}	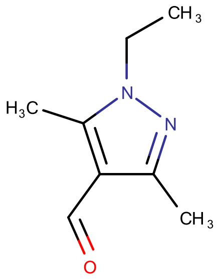
**2**{*67*}	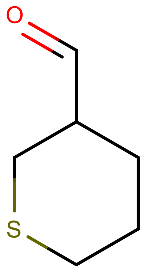
**2**{*68*}	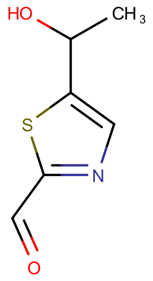

**2**{*69*}	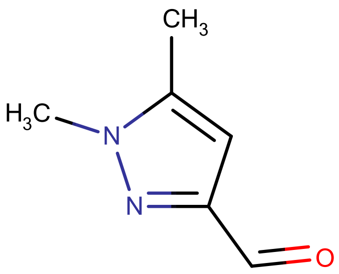
**2**{*70*}	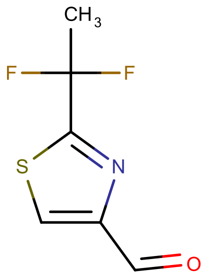
**2**{*71*}	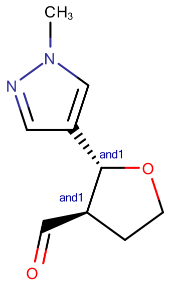
**2**{*72*}	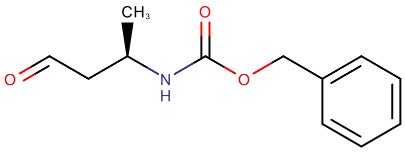

**2**{*73*}	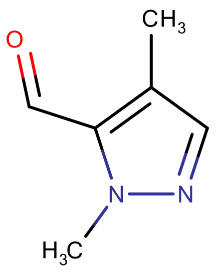
**2**{*74*}	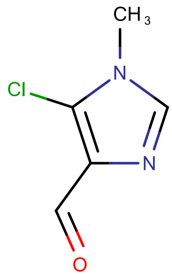
**2**{*75*}	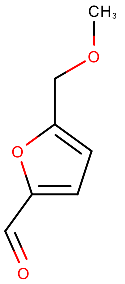
**2**{*76*}	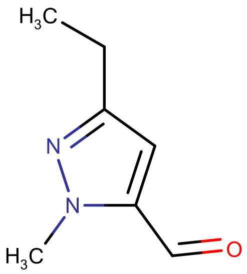

**2**{*77*}	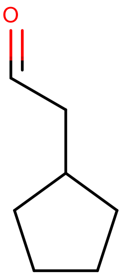
**2**{*78*}	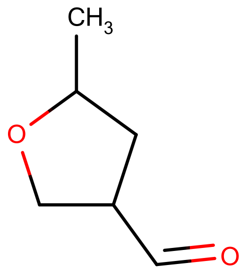
**2**{*79*}	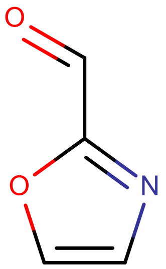
**2**{*80*}	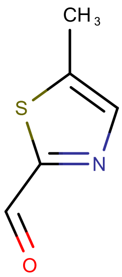

**2**{*81*}	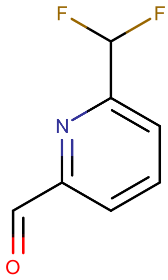
**2**{*82*}	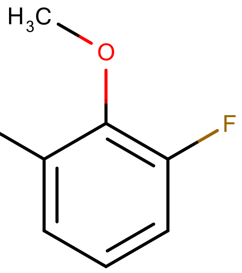
**2**{*83*}	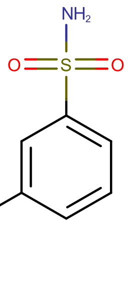
**2**{*84*}	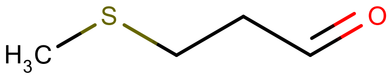

**2**{*85*}	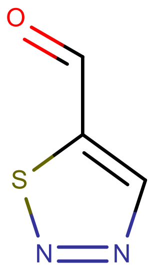
**2**{*86*}	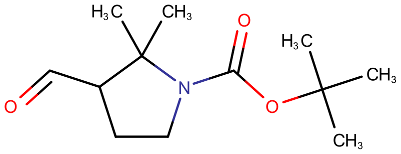
**2**{*87*}	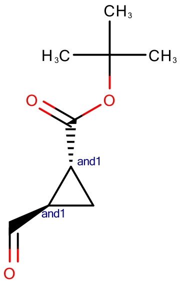
**2**{*88*}	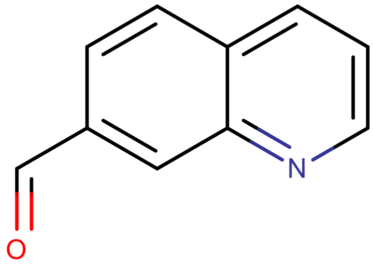

**2**{*89*}	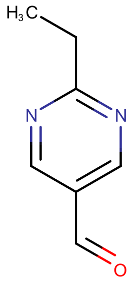
**2**{*90*}	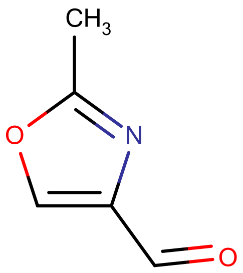
**2**{*91*}	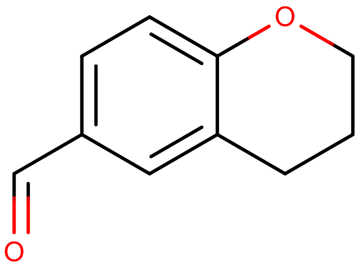
**2**{*92*}	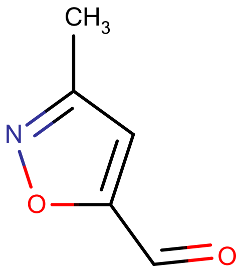
**2**{*93*}	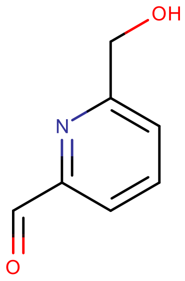
**2**{*94*}	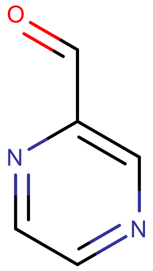
**2**{*95*}	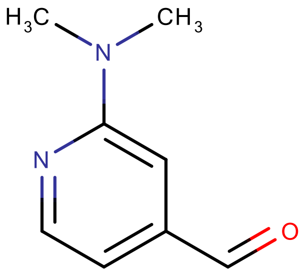
**2**{*96*}	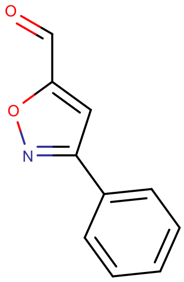

**2**{*97*}	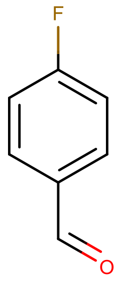
**2**{*98*}	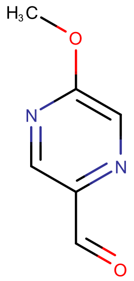
**2**{*99*}	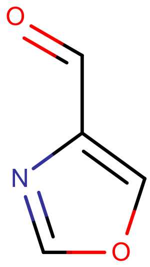
**2**{*100*}	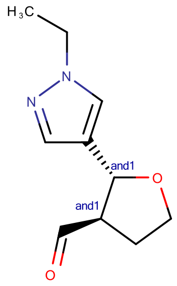

**2**{*101*}	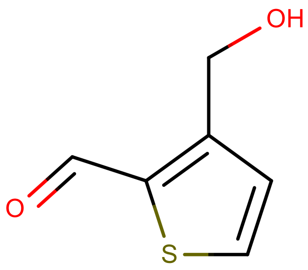
**2**{*102*}	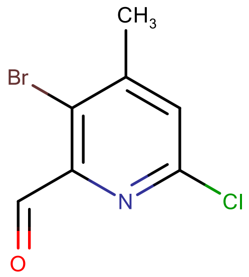
**2**{*103*}	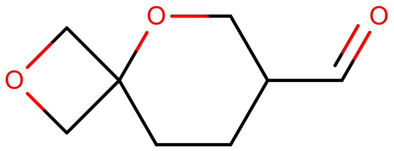
**2**{*104*}	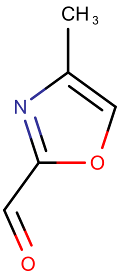

**2**{*105*}	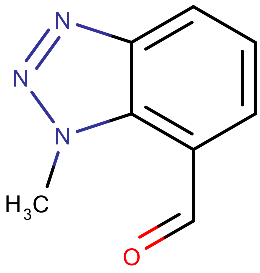
**2**{*106*}	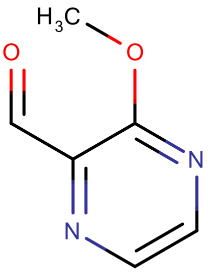
**2**{*107*}	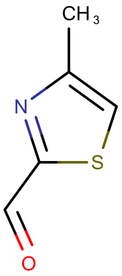
**2**{*108*}	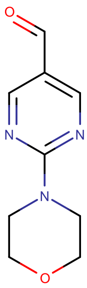

**2**{*109*}	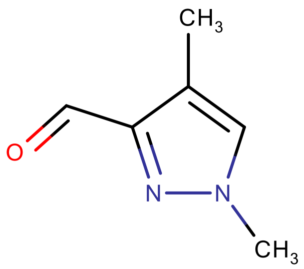
**2**{*110*}	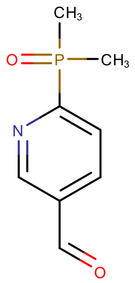
**2**{*111*}	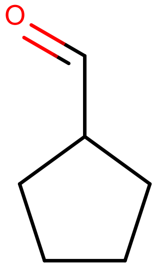
**2**{*112*}	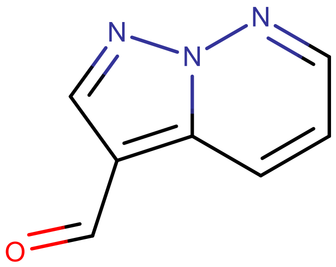

**2**{*113*}	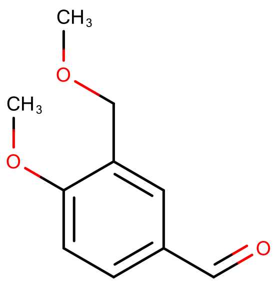
**2**{*114*}	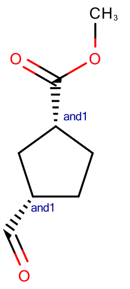
**2**{*115*}	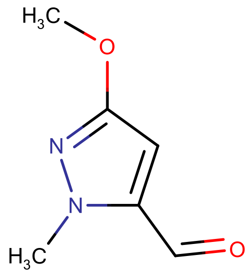
**2**{*116*}	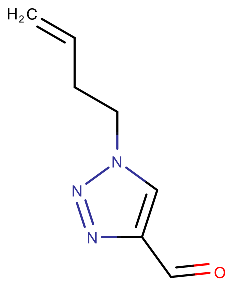
**2**{*117*}	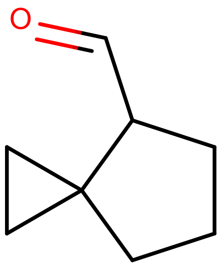
**2**{*118*}	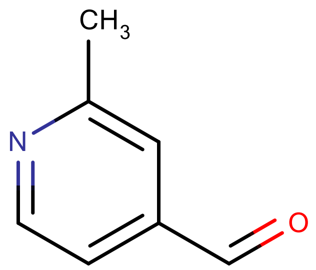
**2**{*119*}	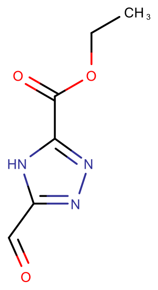
**2**{*120*}	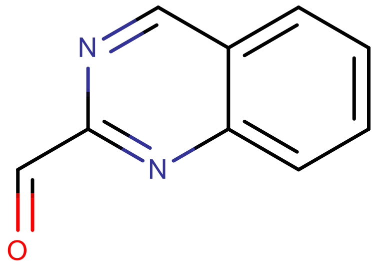

**2**{*121*}	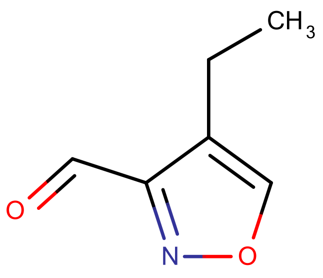
**2**{*122*}	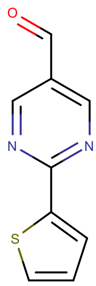
**2**{*123*}	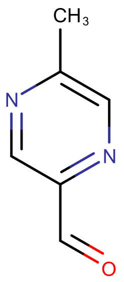
**2**{*124*}	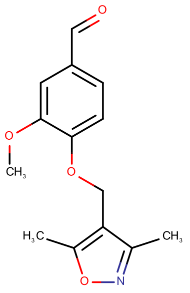

**2**{*125*}	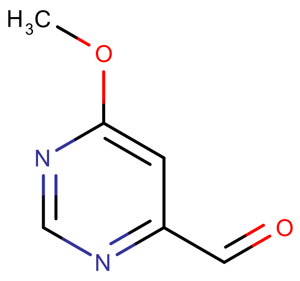
**2**{*126*}	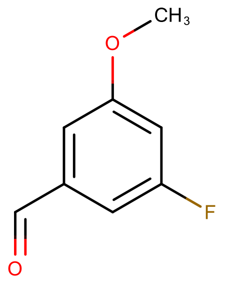
**2**{*127*}	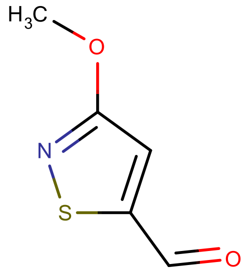
**2**{*128*}	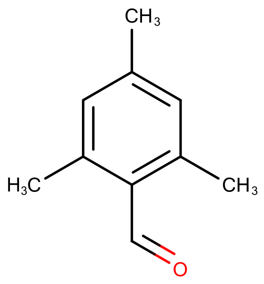

**2**{*129*}	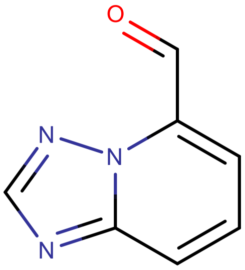
**2**{*130*}	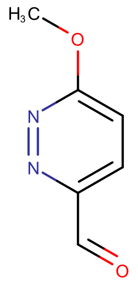
**2**{*131*}	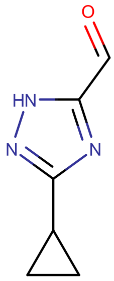
**2**{*132*}	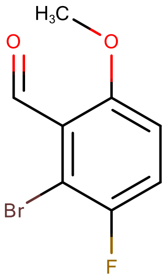

**2**{*133*}	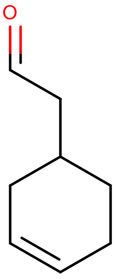
**2**{*134*}	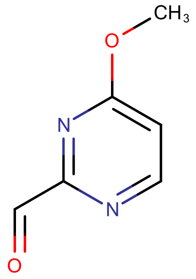
**2**{*135*}	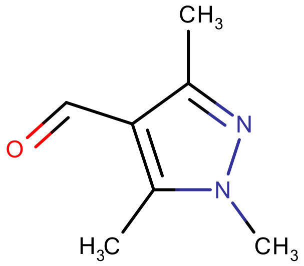
**2**{*136*}	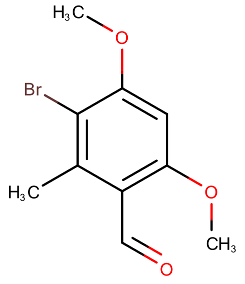

**2**{*137*}	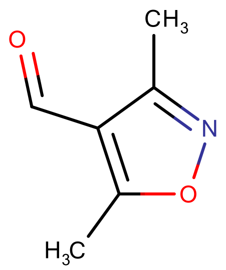
**2**{*138*}	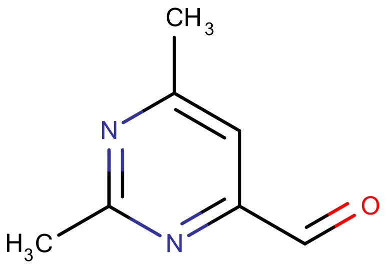
**2**{*139*}	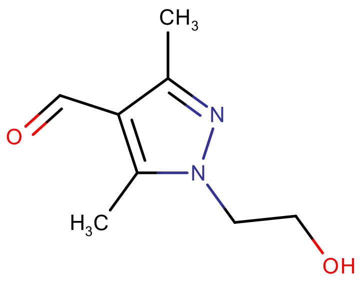
**2**{*140*}	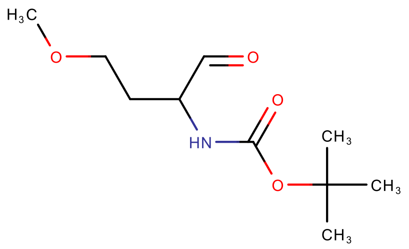

**2**{*141*}	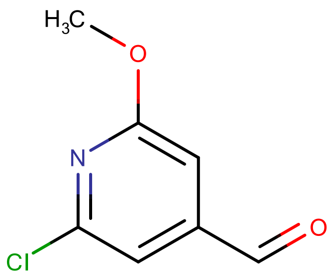
**2**{*142*}	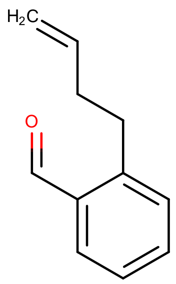
**2**{*143*}	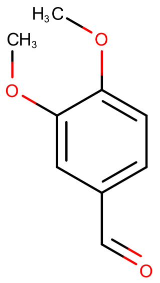
**2**{*144*}	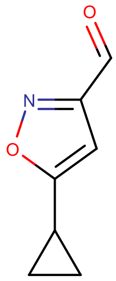

**2**{*145*}	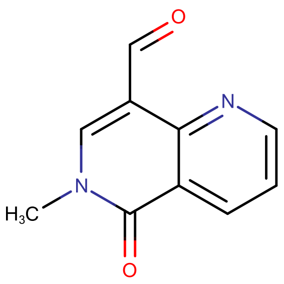
**2**{*146*}	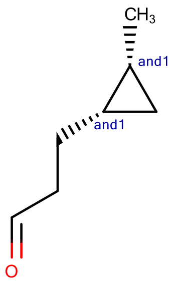
**2**{*147*}	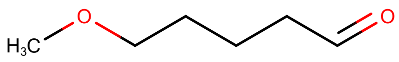
**2**{*148*}	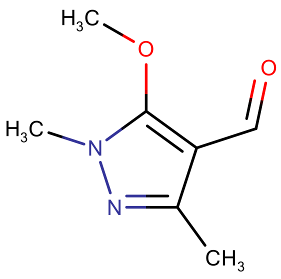

**2**{*149*}	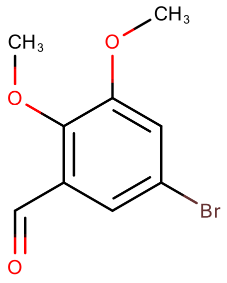
**2**{*150*}	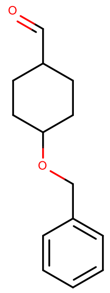
**2**{*151*}	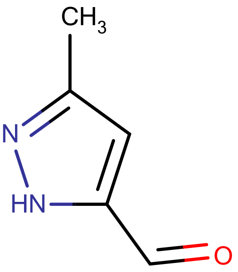
**2**{*152*}	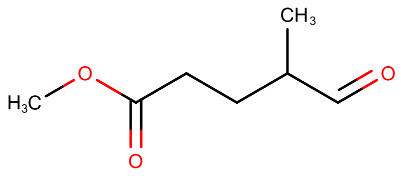

**2**{*153*}	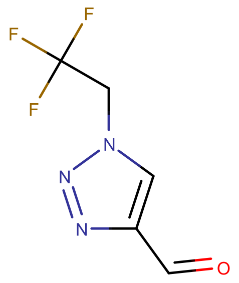
**2**{*154*}	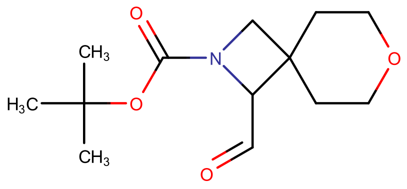
**2**{*155*}	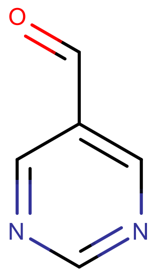
**2**{*156*}	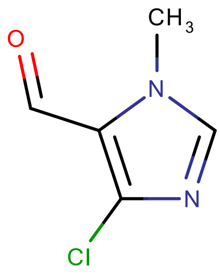

**2**{*157*}	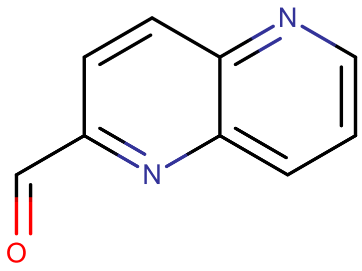
**2**{*158*}	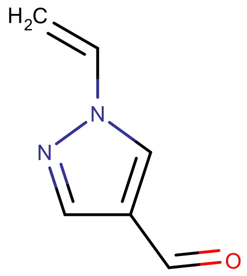
**2**{*159*}	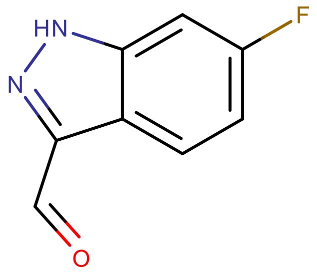
**2**{*160*}	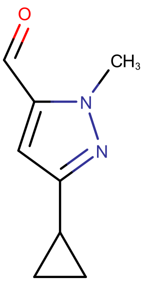

**2**{*161*}	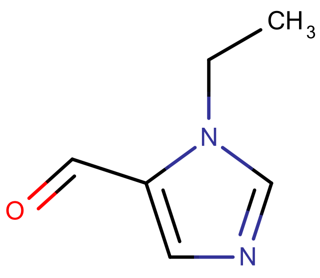	**2**{*165*}	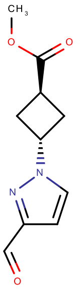
**2**{*162*}	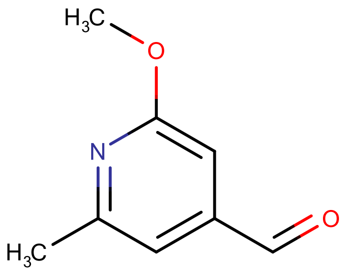	**2**{*166*}	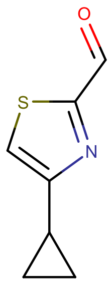
**2**{*163*}	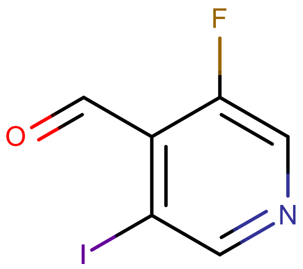	**2**{*167*}	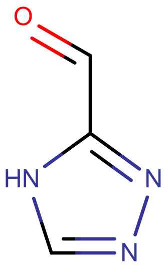
**2**{*164*}	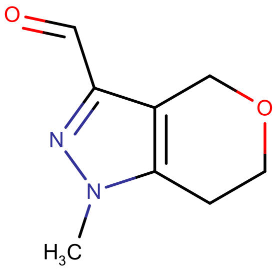	**2**{*168*}	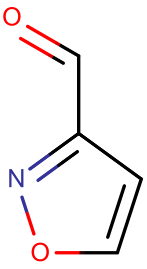

**2**{*169*}	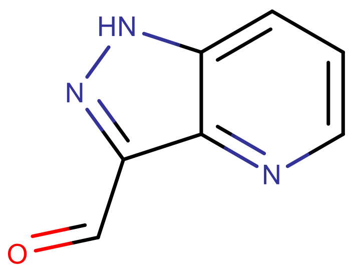
**2**{*170*}	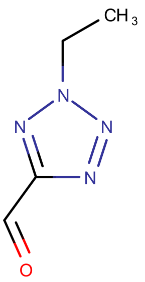
**2**{*171*}	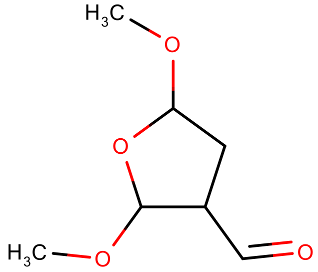
**2**{*172*}	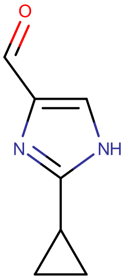

**2**{*173*}	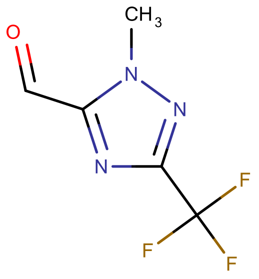
**2**{*174*}	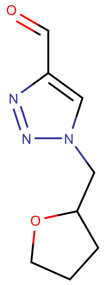
**2**{*175*}	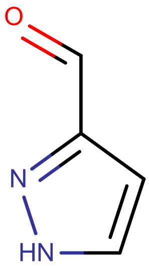
**2**{*176*}	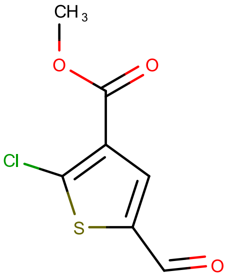

**2**{*177*}	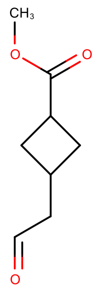
**2**{*178*}	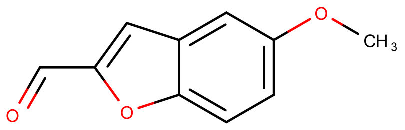
**2**{*179*}	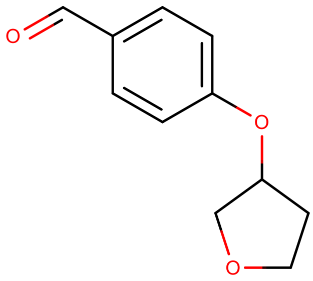
**2**{*180*}	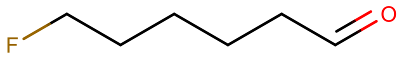

**2**{*181*}	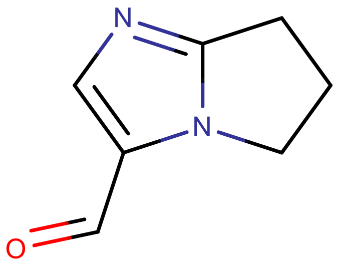
**2**{*182*}	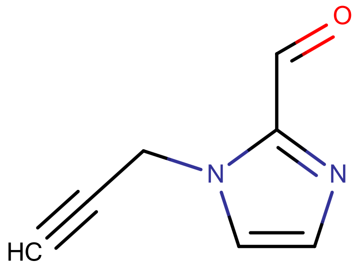
**2**{*183*}	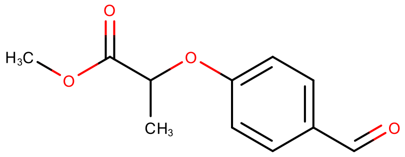
**2**{*184*}	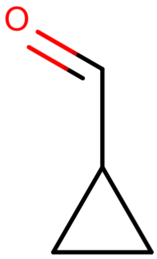

**2**{*185*}	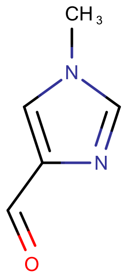
**2**{*186*}	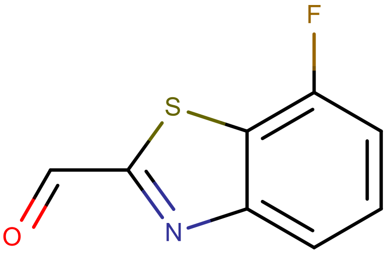
**2**{*187*}	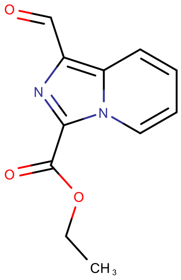
**2**{*188*}	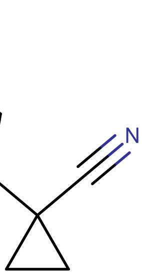

**2**{*189*}	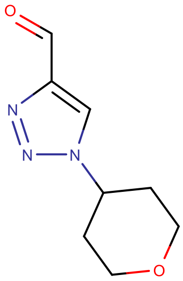
**2**{*190*}	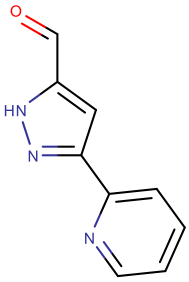
**2**{*191*}	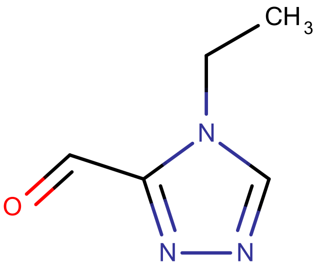
**2**{*192*}	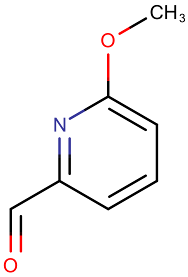

**2**{*193*}	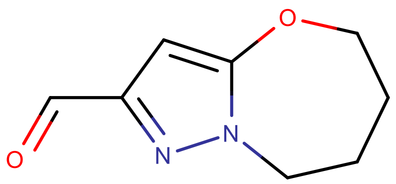
**2**{*194*}	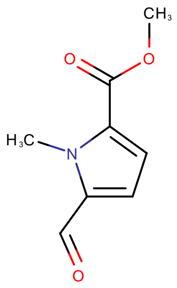
**2**{*195*}	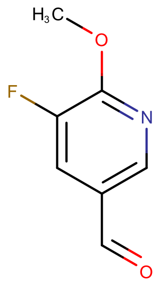
**2**{*196*}	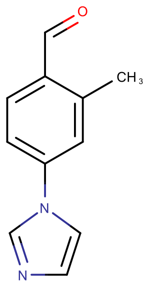

**2**{*197*}	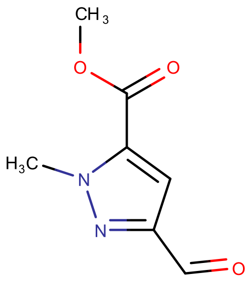
**2**{*198*}	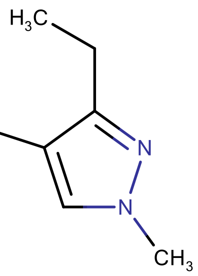
**2**{*199*}	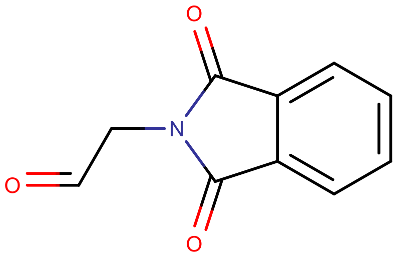
**2**{*200*}	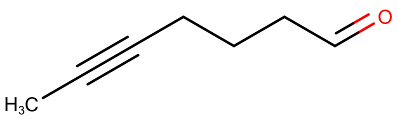

**2**{*201*}	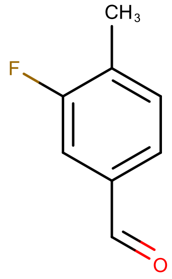
**2**{*202*}	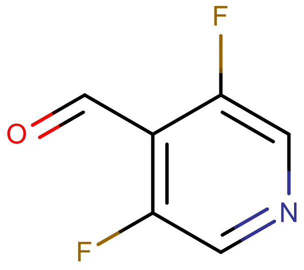
**2**{*203*}	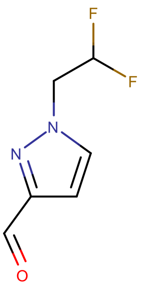
**2**{*204*}	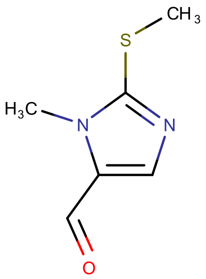

**2**{*205*}	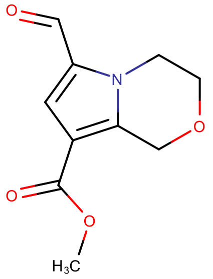
**2**{*206*}	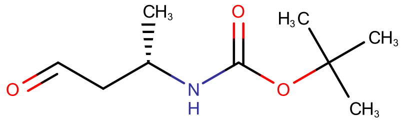
**2**{*207*}	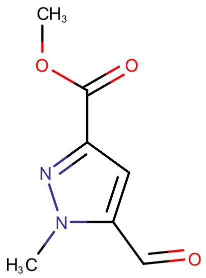
**2**{*208*}	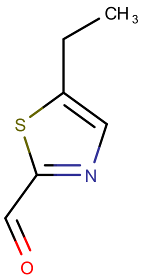

**2**{*209*}	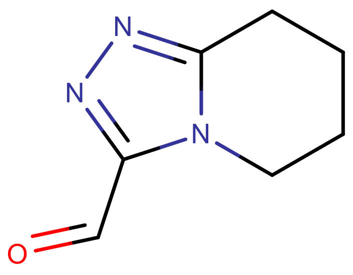
**2**{*210*}	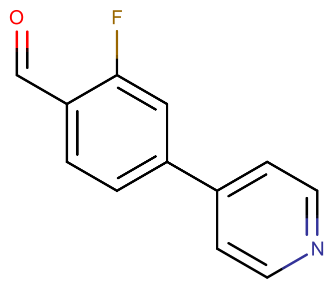
**2**{*211*}	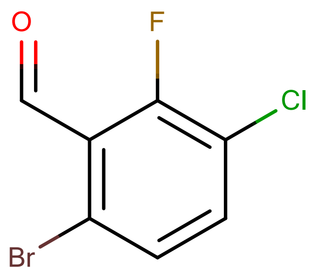
**2**{*212*}	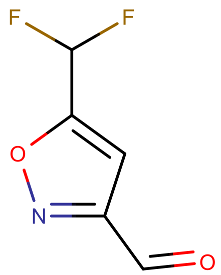

**2**{*213*}	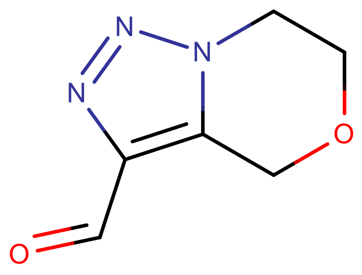
**2**{*214*}	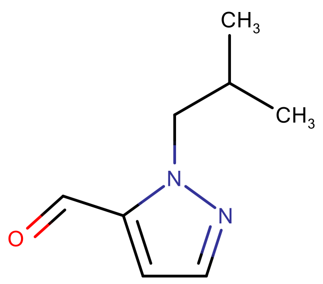
**2**{*215*}	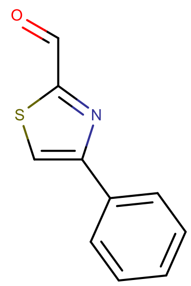
**2**{*216*}	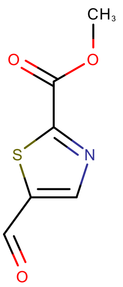

**2**{*217*}	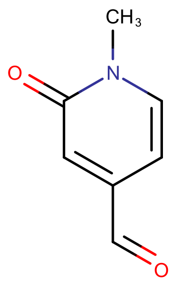
**2**{*218*}	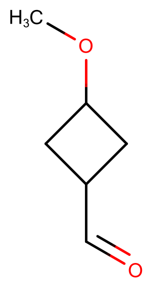
**2**{*219*}	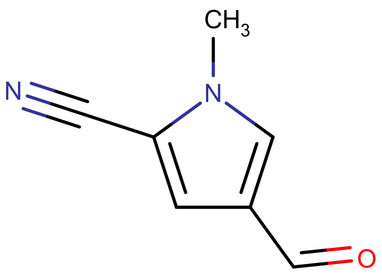
**2**{*220*}	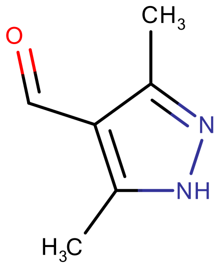

**2**{*221*}	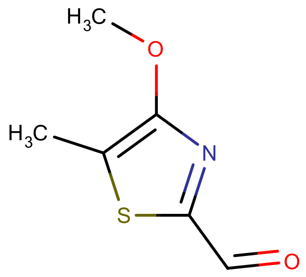
**2**{*222*}	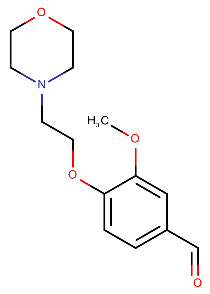
**2**{*223*}	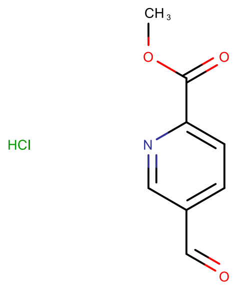
**2**{*224*}	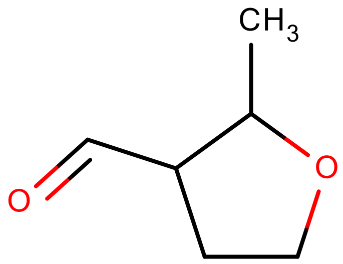

**2**{*225*}	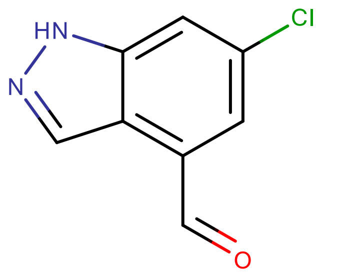
**2**{*226*}	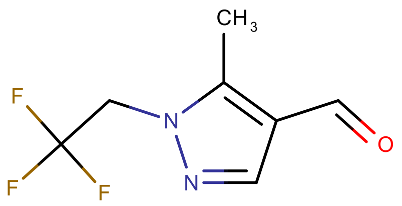
**2**{*227*}	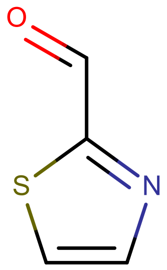
**2**{*228*}	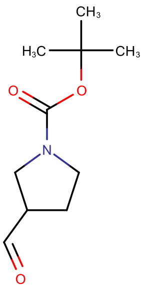

**2**{*229*}	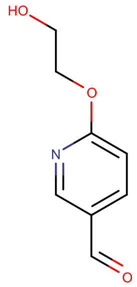
**2**{*230*}	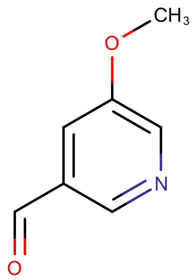
**2**{*231*}	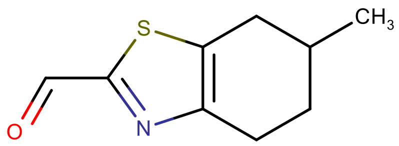
**2**{*232*}	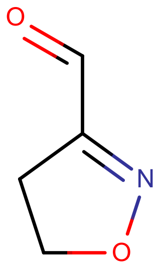

**2**{*233*}	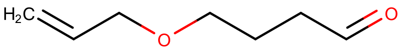
**2**{*234*}	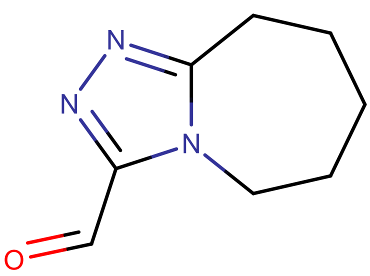
**2**{*235*}	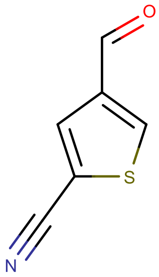
**2**{*236*}	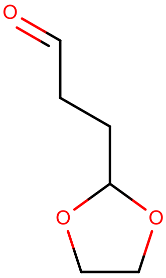

**2**{*237*}	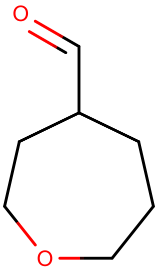
**2**{*238*}	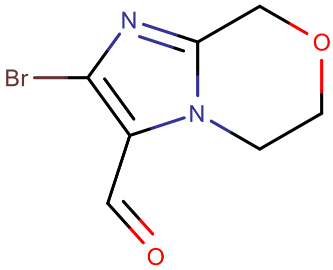
**2**{*239*}	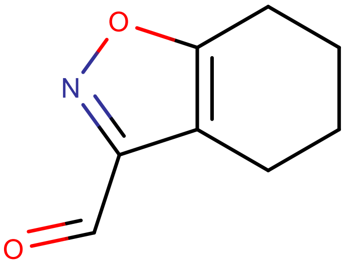
**2**{*240*}	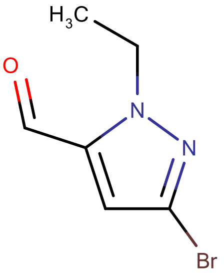

**2**{*241*}	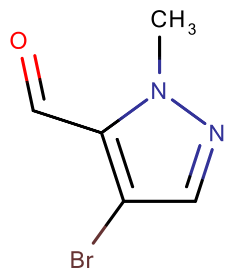
**2**{*242*}	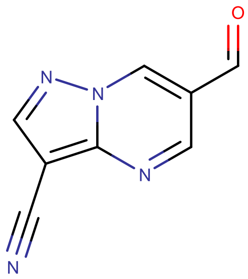
**2**{*243*}	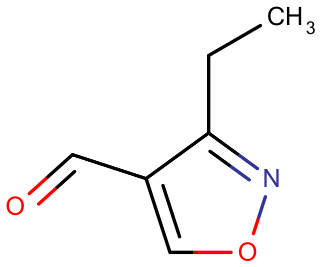
**2**{*244*}	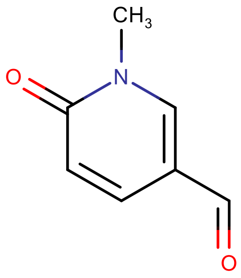

**2**{*245*}	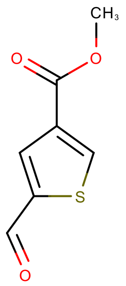
**2**{*246*}	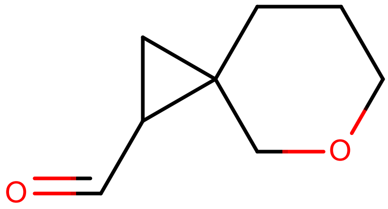
**2**{*247*}	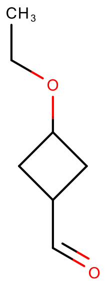
**2**{*248*}	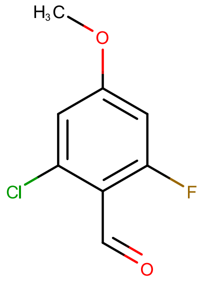

**2**{*249*}	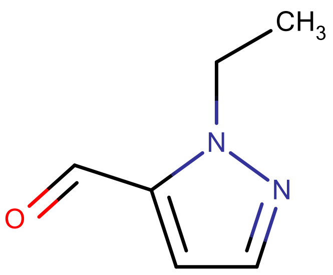
**2**{*250*}	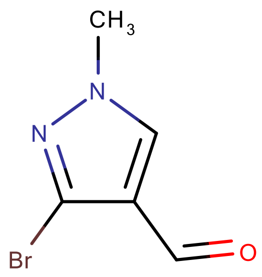
**2**{*251*}	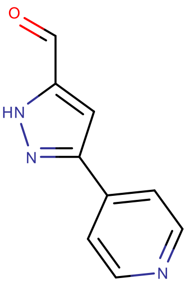
**2**{*252*}	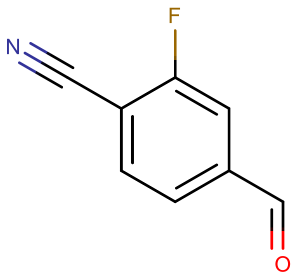

**2**{*253*}	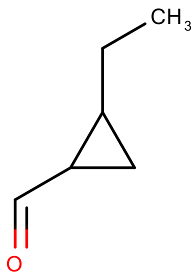
**2**{*254*}	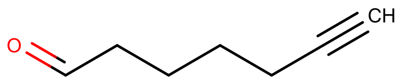
**2**{*255*}	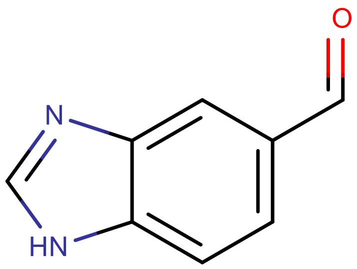
**2**{*256*}	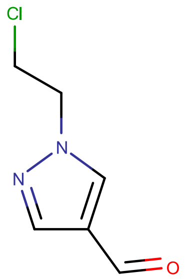

**2**{*257*}	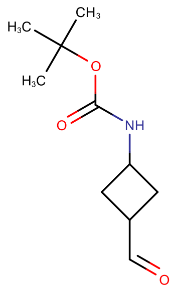
**2**{*258*}	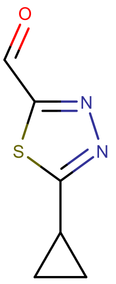
**2**{*259*}	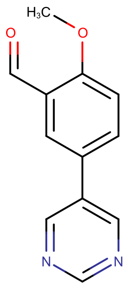
**2**{*260*}	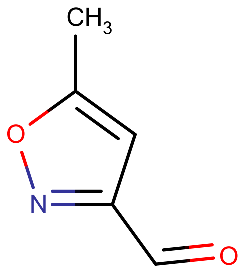

**2**{*261*}	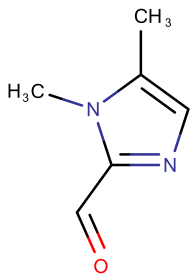
**2**{*262*}	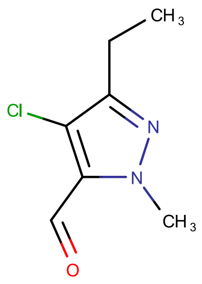
**2**{*263*}	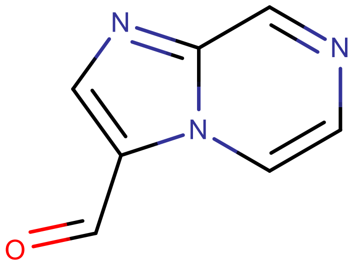
**2**{*264*}	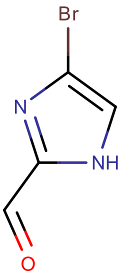

**2**{*265*}	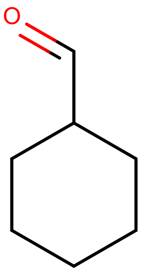
**2**{*266*}	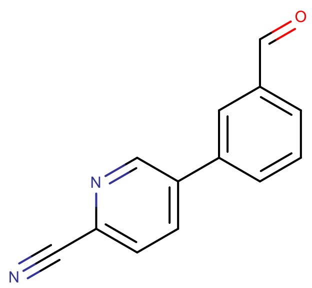
**2**{*267*}	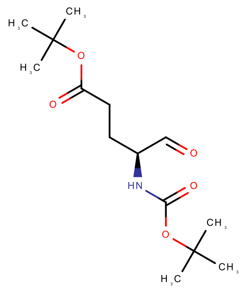
**2**{*268*}	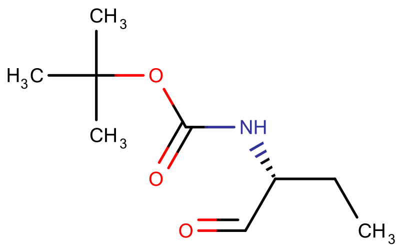

**2**{*269*}	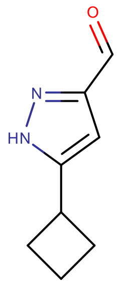
**2**{*270*}	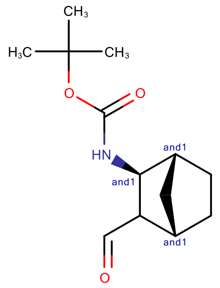
**2**{*271*}	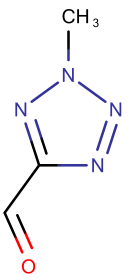
**2**{*272*}	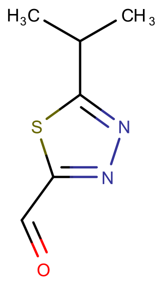

**2**{*273*}	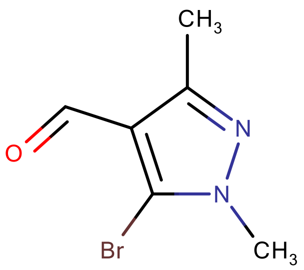
**2**{*274*}	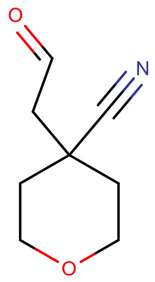
**2**{*275*}	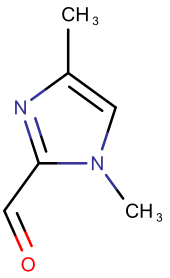
**2**{*276*}	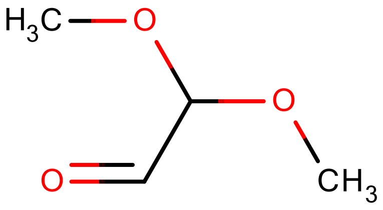

**2**{*277*}	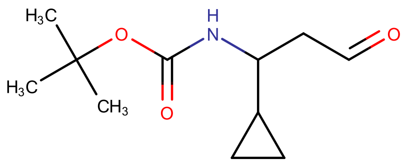
**2**{*278*}	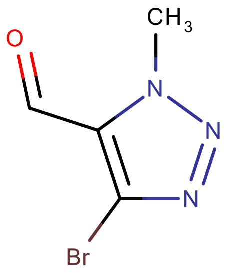
**2**{*279*}	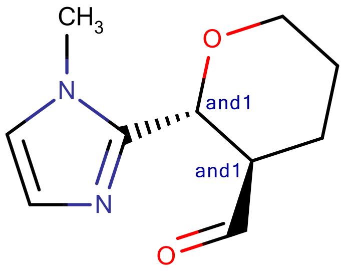
**2**{*280*}	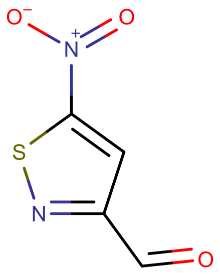

**2**{*281*}	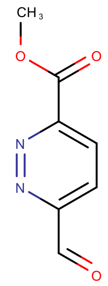
**2**{*282*}	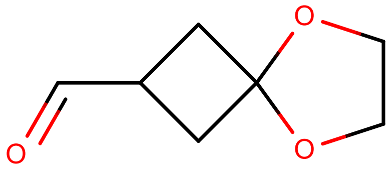
**2**{*283*}	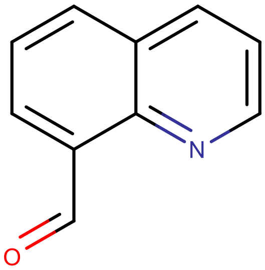
**2**{*284*}	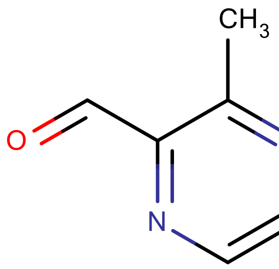

**2**{*285*}	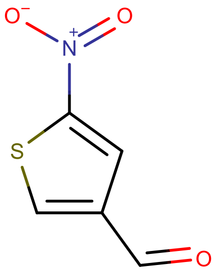
**2**{*286*}	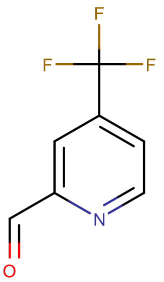
**2**{*287*}	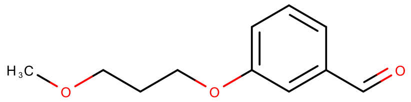
**2**{*288*}	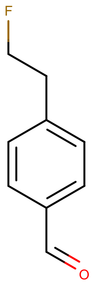

**2**{*289*}	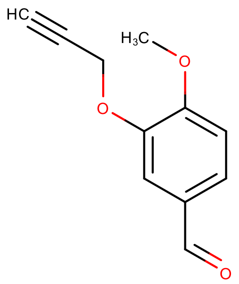
**2**{*290*}	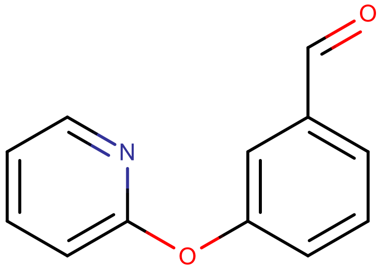
**2**{*291*}	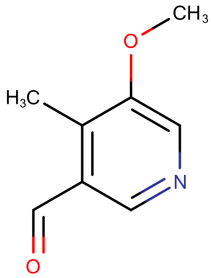
**2**{*292*}	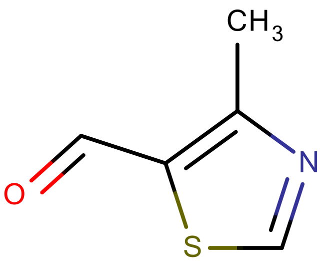
**2**{*293*}	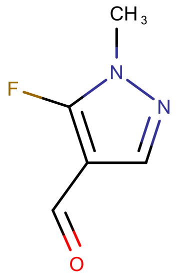
**2**{*294*}	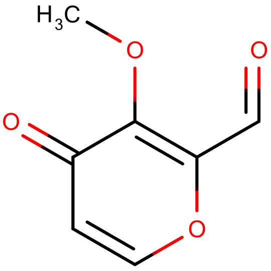
**2**{*295*}	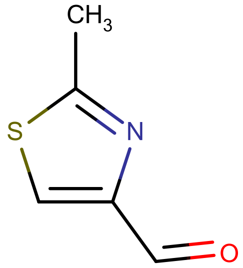
**2**{*296*}	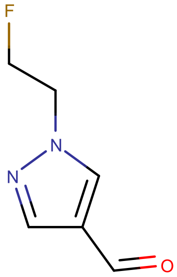

**2**{*297*}	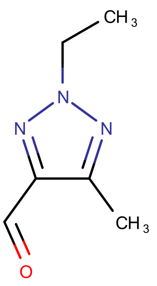
**2**{*298*}	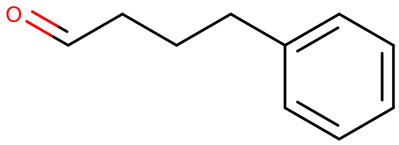
**2**{*299*}	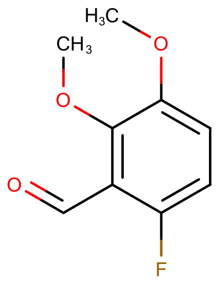
**2**{*300*}	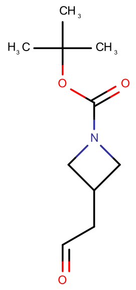

**2**{*301*}	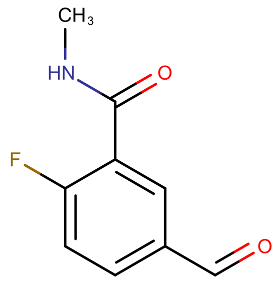
**2**{*302*}	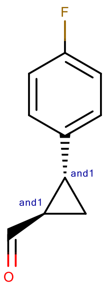
**2**{*303*}	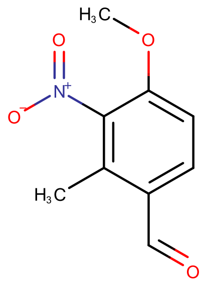
**2**{*304*}	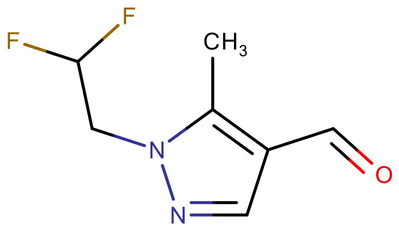

**2**{*305*}	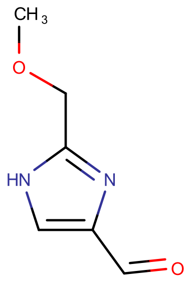	**2**{*309*}	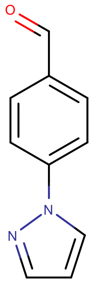
**2**{*306*}	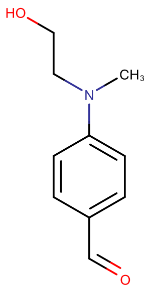	**2**{*310*}	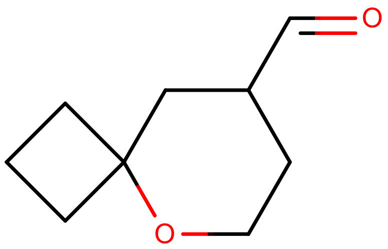
**2**{*307*}	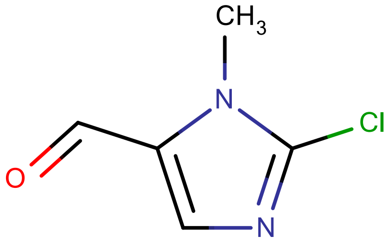	**2**{*311*}	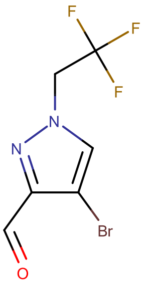
**2**{*308*}	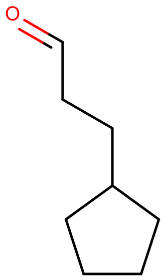	**2**{*312*}	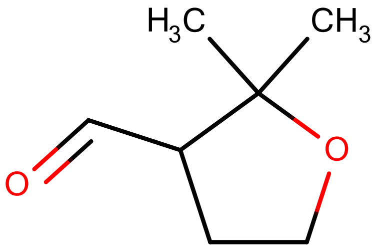

**2**{*313*}	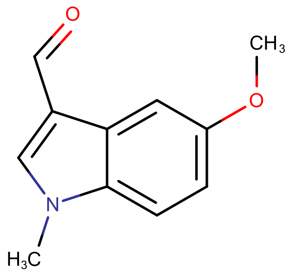
**2**{*314*}	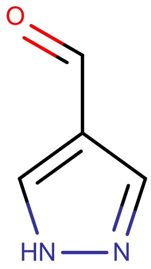
**2**{*315*}	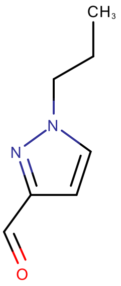
**2**{*316*}	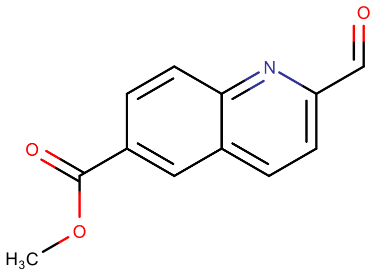

**2**{*317*}	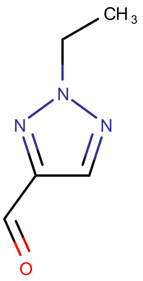
**2**{*318*}	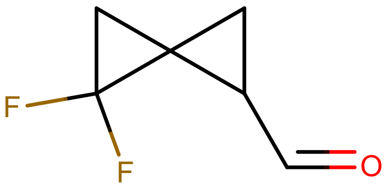
**2**{*319*}	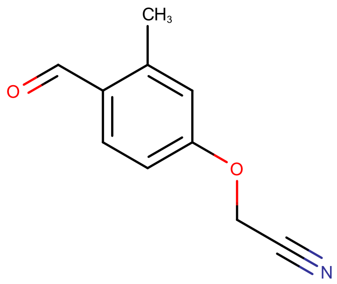
**2**{*320*}	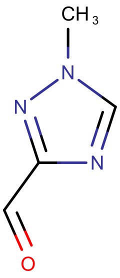

**2**{*321*}	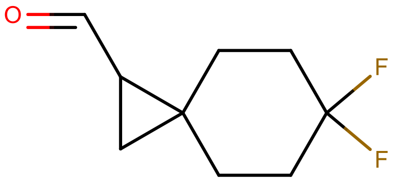
**2**{*322*}	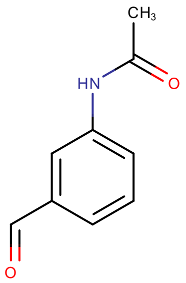
**2**{*323*}	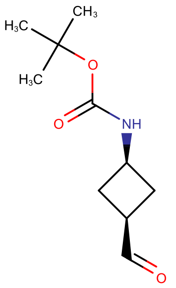
**2**{*324*}	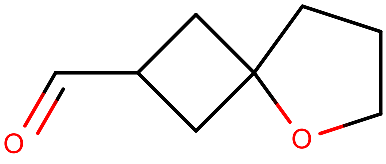

**2**{*325*}	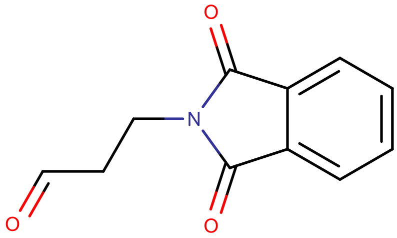
**2**{*326*}	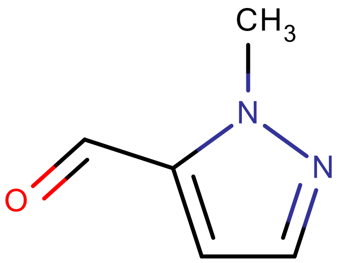
**2**{*327*}	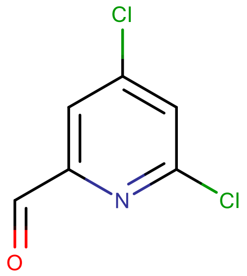
**2**{*328*}	HCl 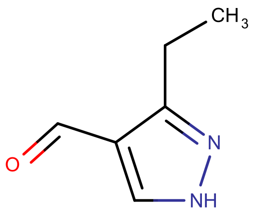

**2**{*329*}	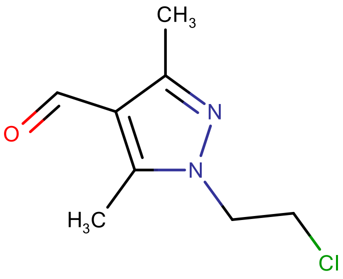
**2**{*330*}	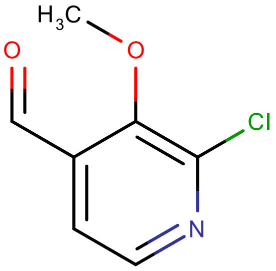
**2**{*331*}	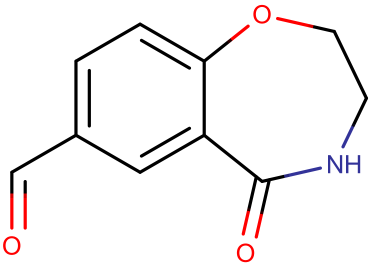
**2**{*332*}	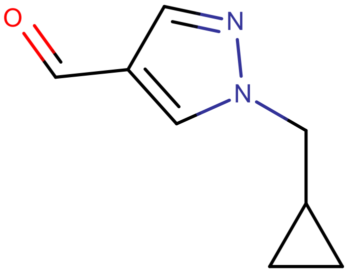

**2**{*333*}	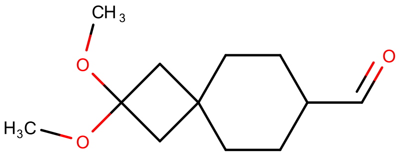
**2**{*334*}	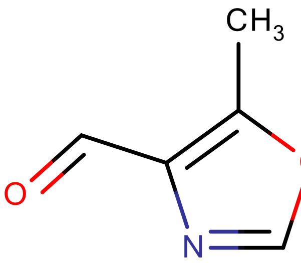
**2**{*335*}	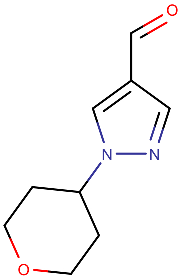
**2**{*336*}	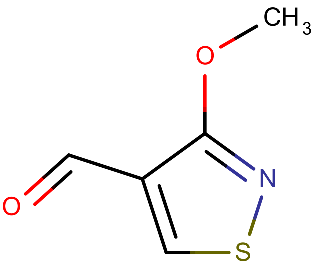

**2**{*337*}	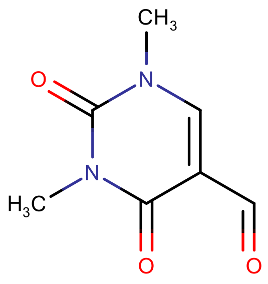
**2**{*338*}	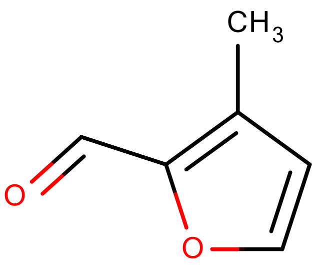
**2**{*339*}	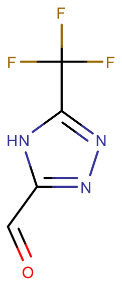
**2**{*340*}	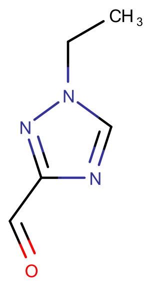

**2**{*341*}	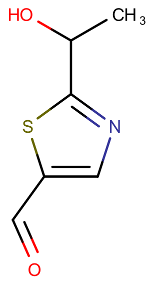
**2**{*342*}	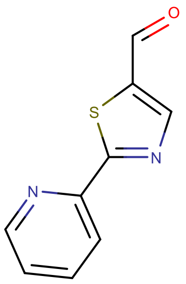
**2**{*343*}	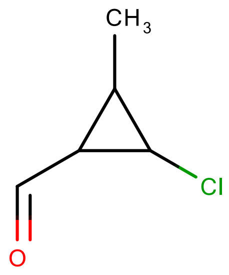
**2**{*344*}	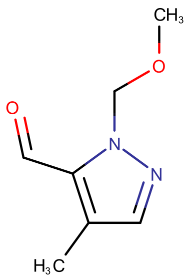

**2**{*345*}	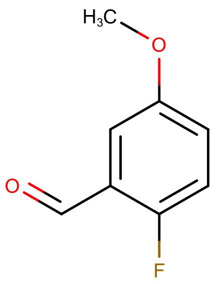
**2**{*346*}	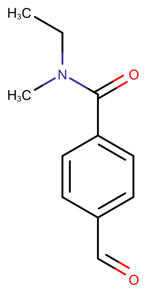
**2**{*347*}	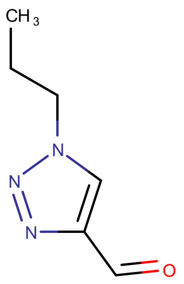
**2**{*348*}	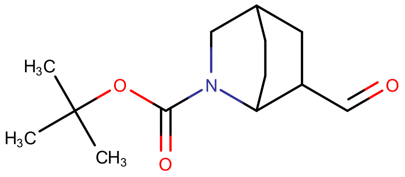

**2**{*349*}	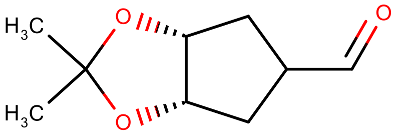
**2**{*350*}	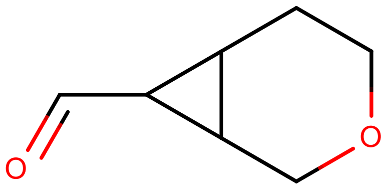
**2**{*351*}	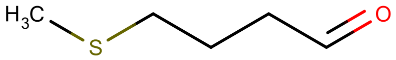
**2**{*352*}	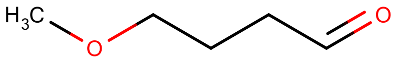

**2**{*353*}	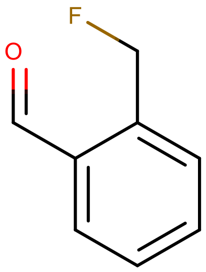
**2**{*354*}	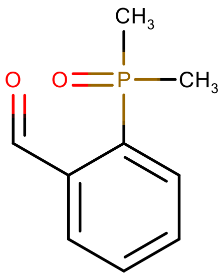
**2**{*355*}	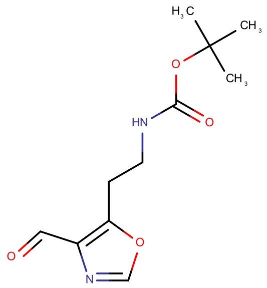
**2**{*356*}	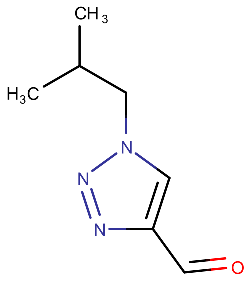

**2**{*357*}	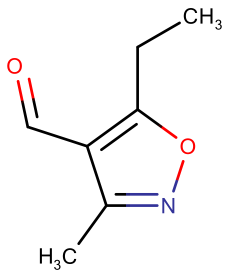
**2**{*358*}	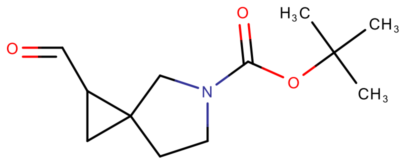
**2**{*359*}	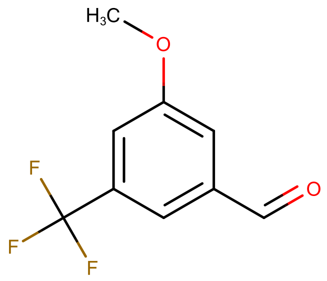
**2**{*360*}	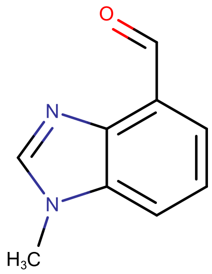

**2**{*361*}	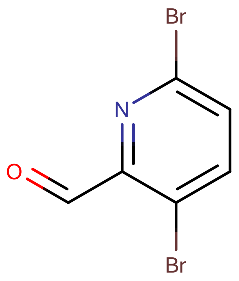
**2**{*362*}	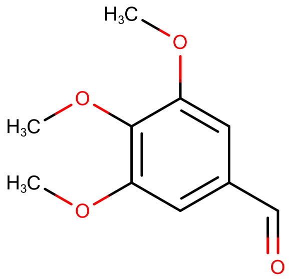
**2**{*363*}	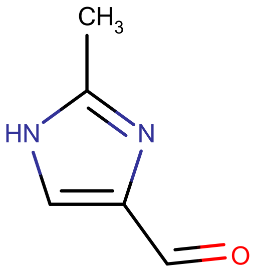
**2**{*364*}	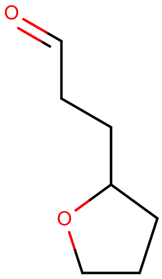

**2**{*365*}	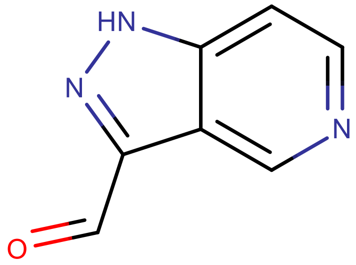
**2**{*366*}	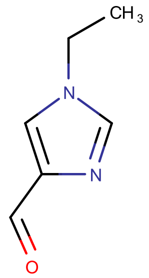
**2**{*367*}	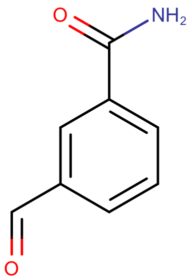
**2**{*368*}	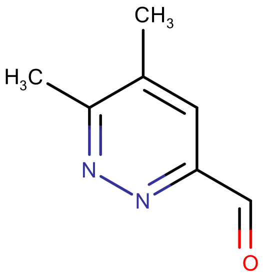

**2**{*369*}	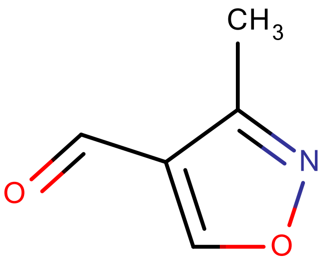
**2**{*370*}	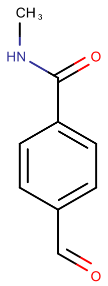
**2**{*371*}	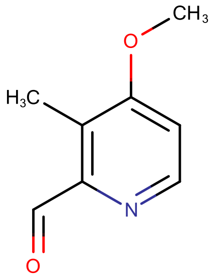
**2**{*372*}	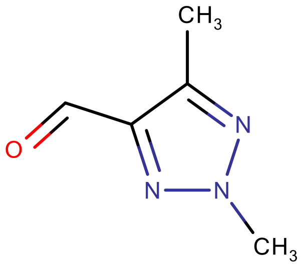

**2**{*373*}	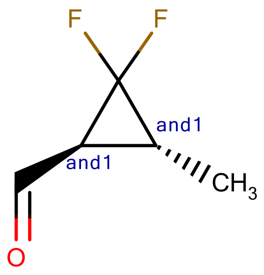
**2**{*374*}	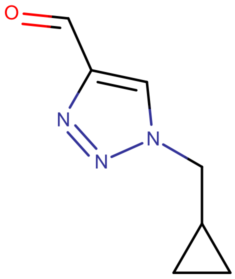
**2**{*375*}	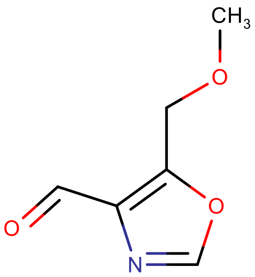
**2**{*376*}	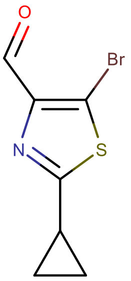

**2**{*377*}	HCl 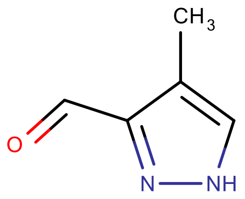
**2**{*378*}	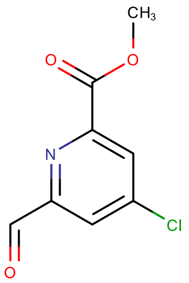
**2**{*379*}	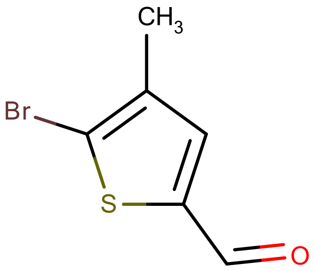
**2**{*380*}	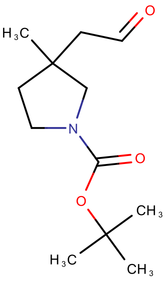

**2**{*381*}	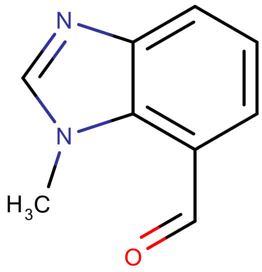
**2**{*382*}	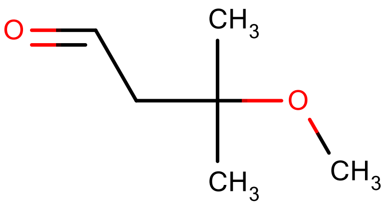
**2**{*383*}	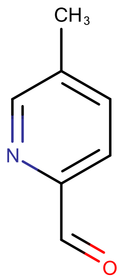
**2**{*384*}	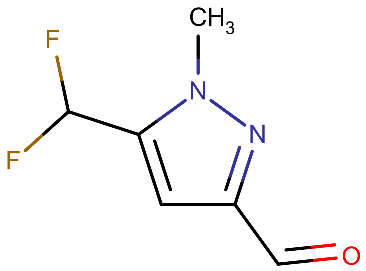

**2**{*385*}	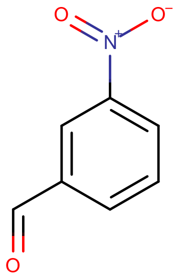
**2**{*386*}	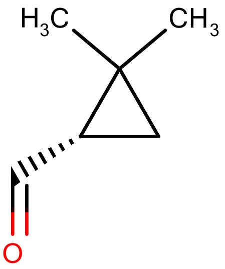
**2**{*387*}	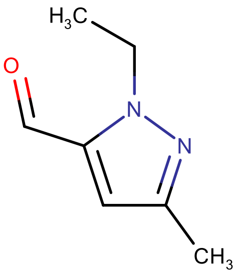
**2**{*388*}	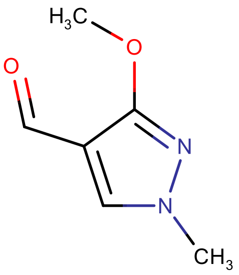

**2**{*389*}	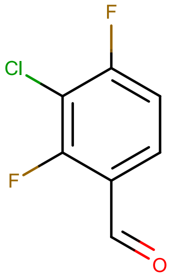
**2**{*390*}	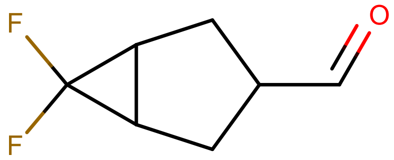
**2**{*391*}	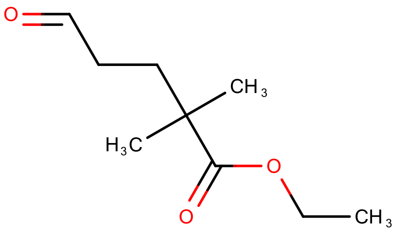
**2**{*392*}	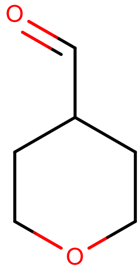

**2**{*393*}	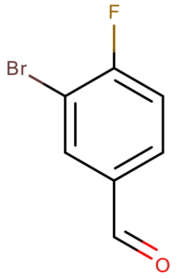
**2**{*394*}	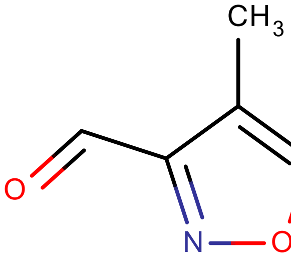
**2**{*395*}	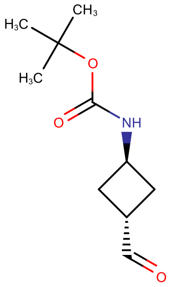
**2**{*396*}	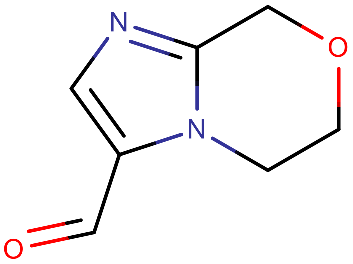

**2**{*397*}	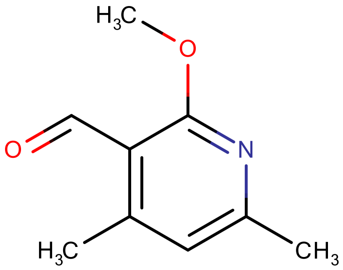
**2**{*398*}	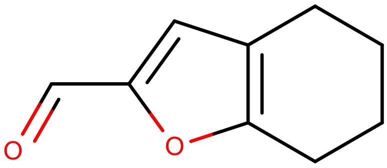
**2**{*399*}	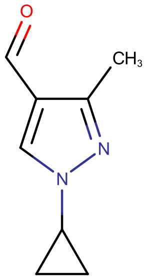
**2**{*400*}	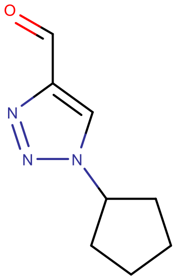

**2**{*401*}	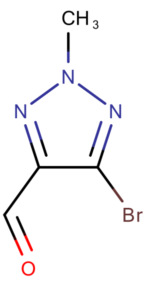
**2**{*402*}	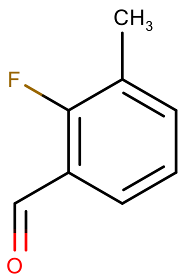
**2**{*403*}	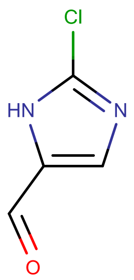
**2**{*404*}	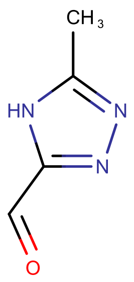

**2**{*405*}	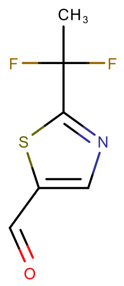
**2**{*406*}	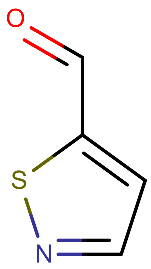
**2**{*407*}	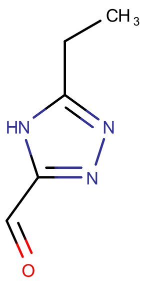
**2**{*408*}	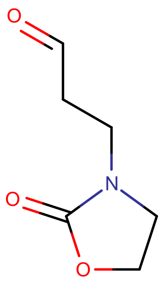

**2**{*409*}	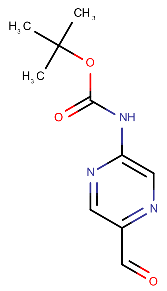
**2**{*410*}	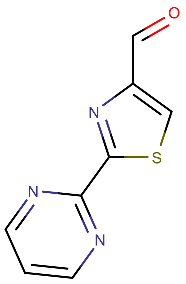
**2**{*411*}	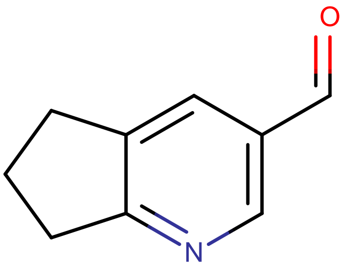
**2**{*412*}	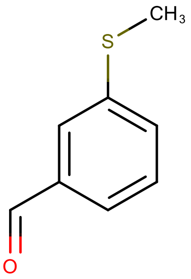

**2**{*413*}	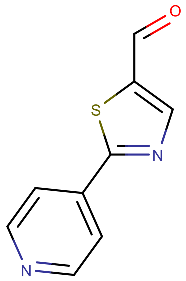
**2**{*414*}	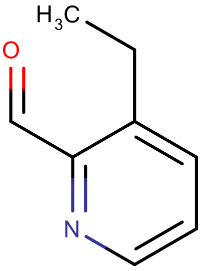
**2**{*415*}	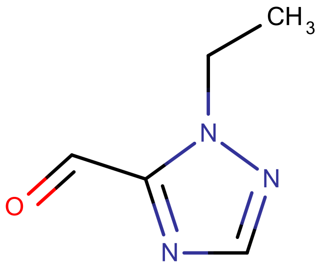
**2**{*416*}	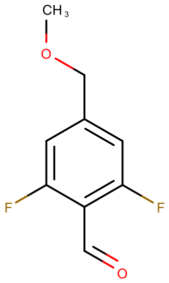

**2**{*417*}	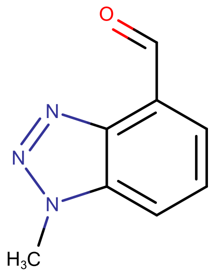
**2**{*418*}	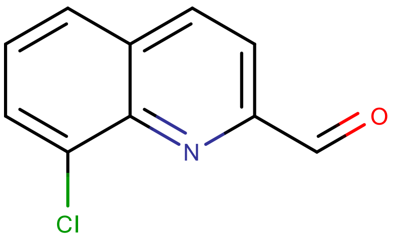
**2**{*419*}	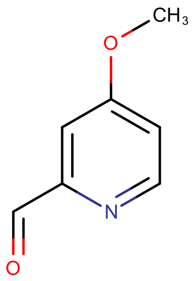
**2**{*420*}	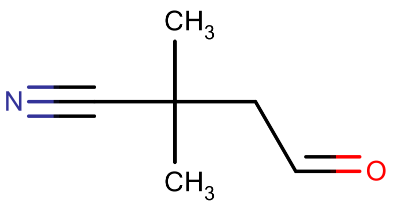

**2**{*421*}	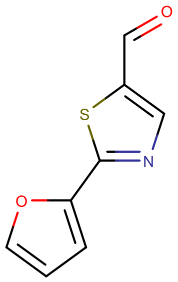
**2**{*422*}	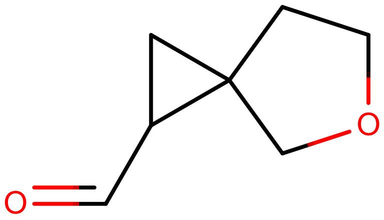
**2**{*423*}	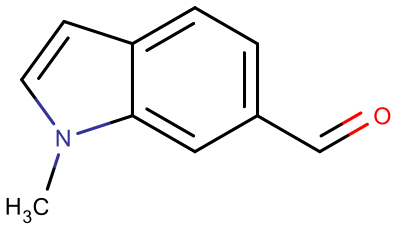
**2**{*424*}	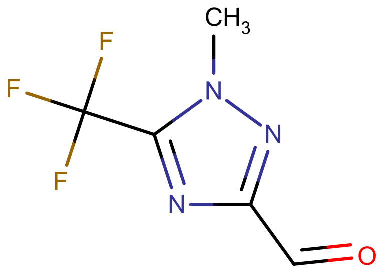

**2**{*425*}	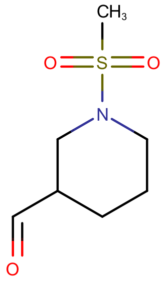
**2**{*426*}	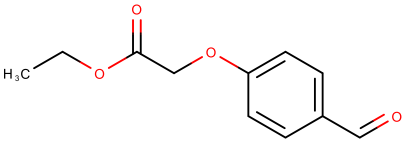
**2**{*427*}	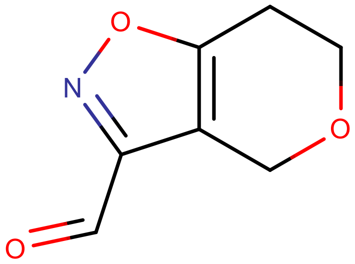
**2**{*428*}	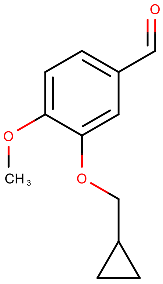

**2**{*429*}	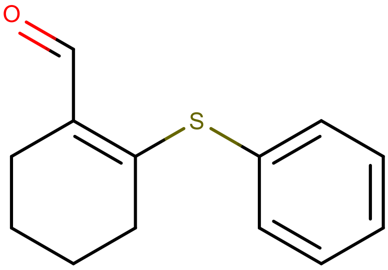
**2**{*430*}	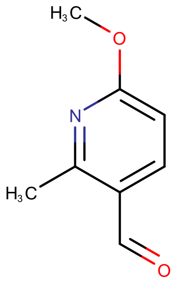
**2**{*431*}	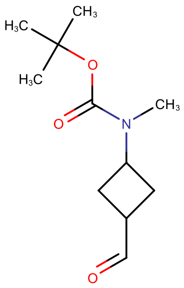
**2**{*432*}	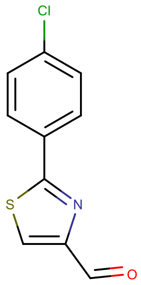

**2**{*433*}	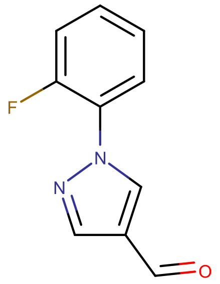
**2**{*434*}	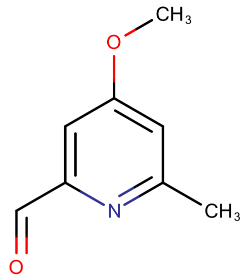
**2**{*435*}	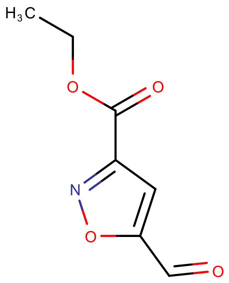
**2**{*436*}	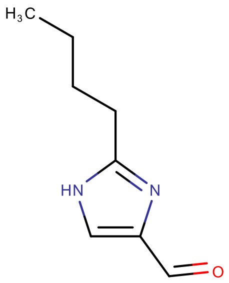

**2**{*437*}	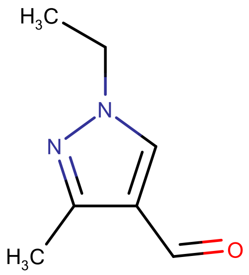
**2**{*438*}	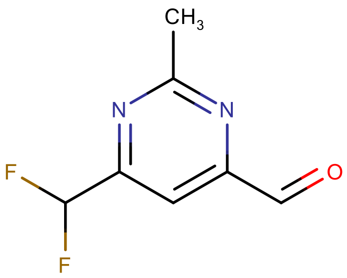
**2**{*439*}	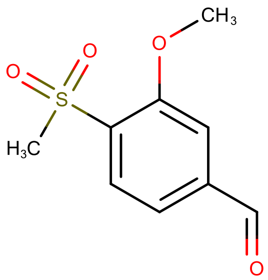
**2**{*440*}	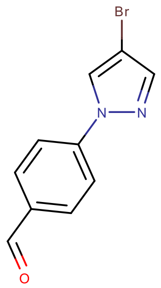

**2**{*441*}	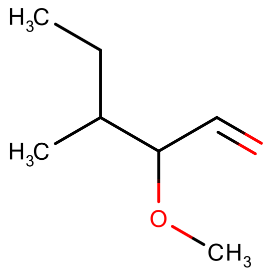	**2**{*445*}	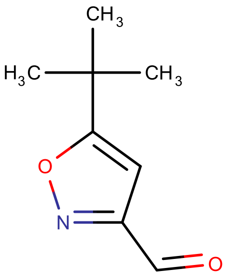
**2**{*442*}	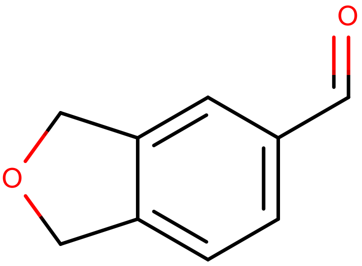	**2**{*446*}	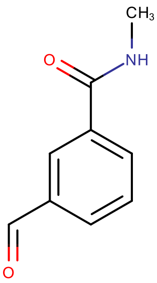
**2**{*443*}	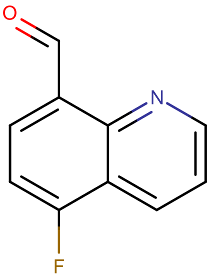	**2**{*447*}	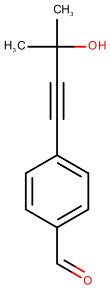
**2**{*444*}	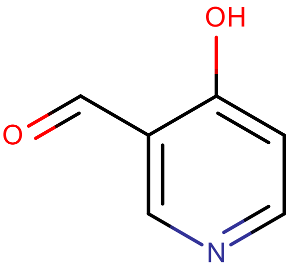	**2**{*448*}	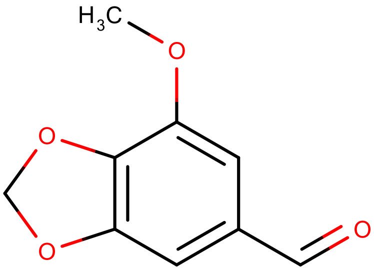

**2**{*449*}	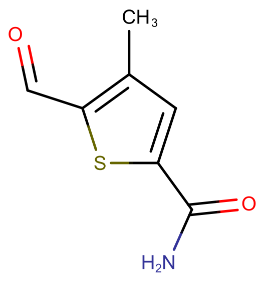
**2**{*450*}	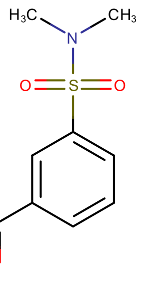
**2**{*451*}	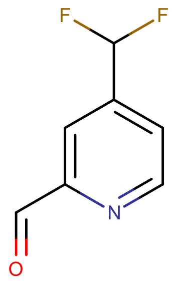
**2**{*452*}	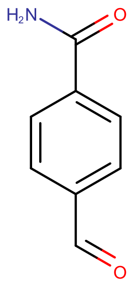

**2**{*453*}	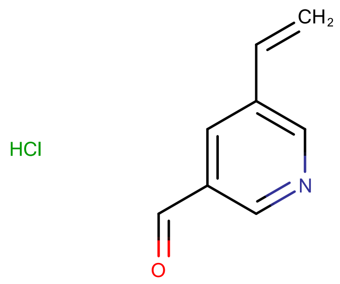
**2**{*454*}	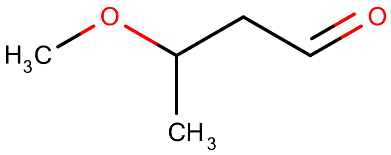
**2**{*455*}	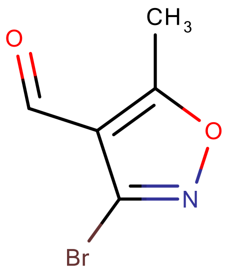
**2**{*456*}	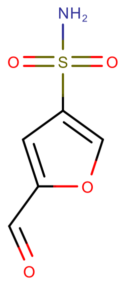

**2**{*457*}	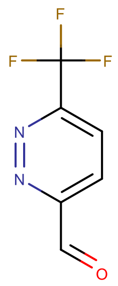
**2**{*458*}	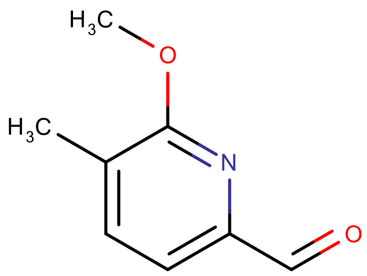
**2**{*459*}	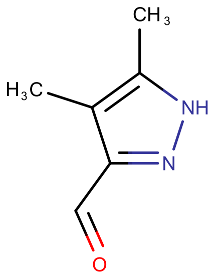
**2**{*460*}	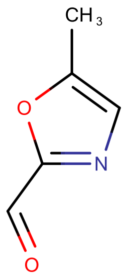

**2**{*461*}	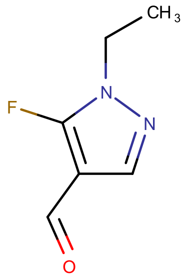
**2**{*462*}	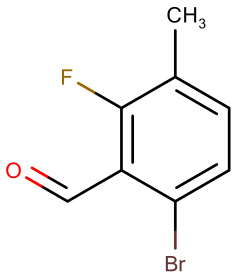
**2**{*463*}	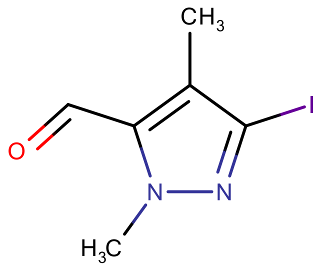
**2**{*464*}	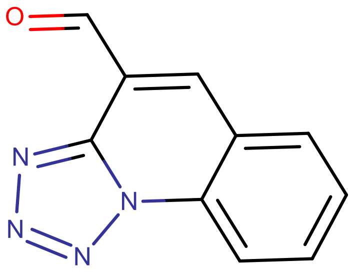

**2**{*465*}	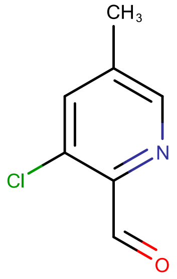
**2**{*466*}	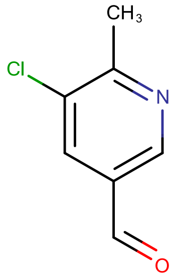
**2**{*467*}	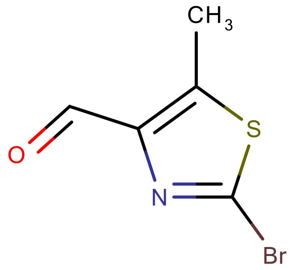
**2**{*468*}	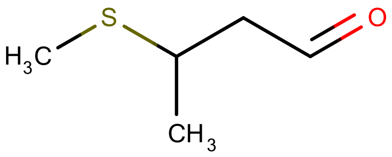

**2**{*469*}	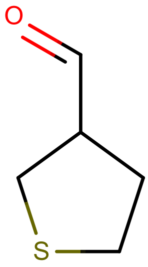
**2**{*470*}	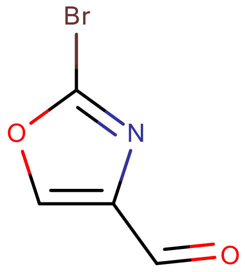
**2**{*471*}	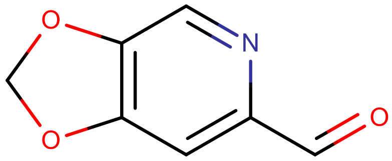
**2**{*472*}	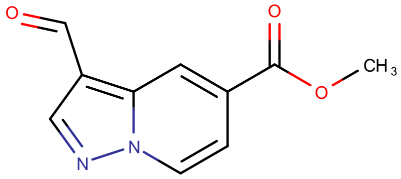

**2**{*473*}	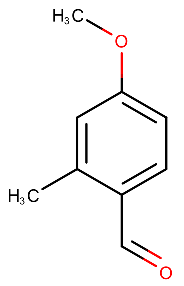
**2**{*474*}	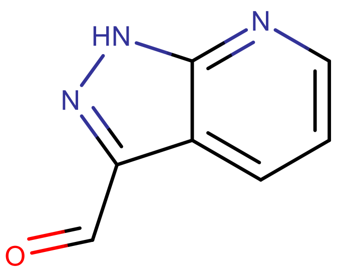
**2**{*475*}	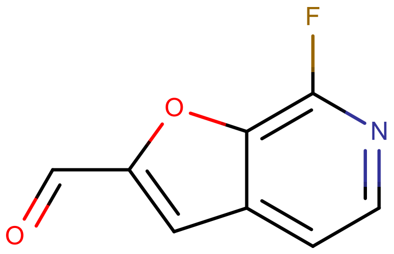
**2**{*476*}	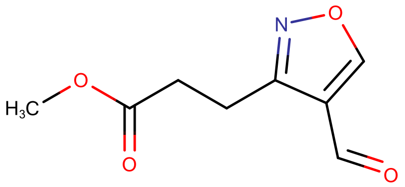

**2**{*477*}	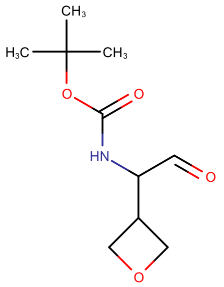
**2**{*478*}	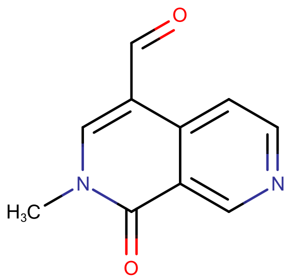
**2**{*479*}	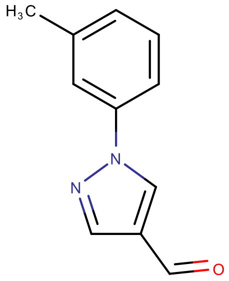
**2**{*480*}	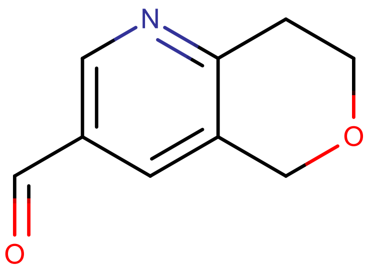

**2**{*481*}	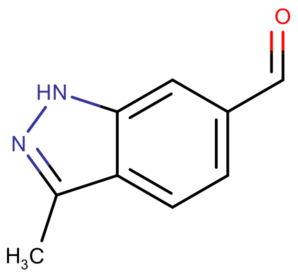
**2**{*482*}	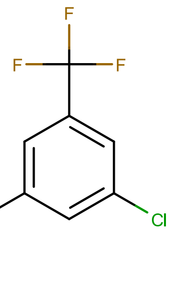
**2**{*483*}	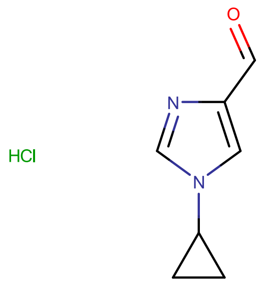
**2**{*484*}	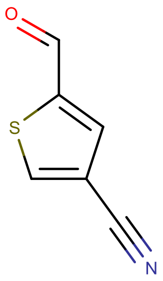

**2**{*485*}	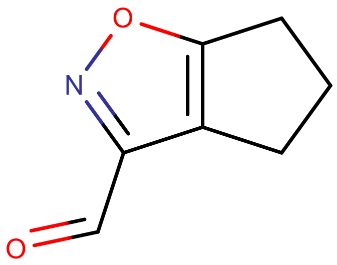
**2**{*486*}	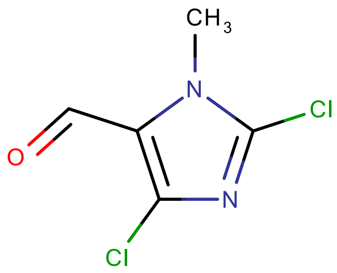
**2**{*487*}	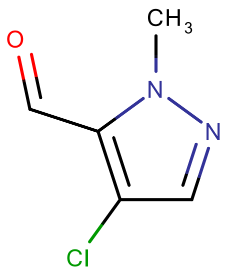
**2**{*488*}	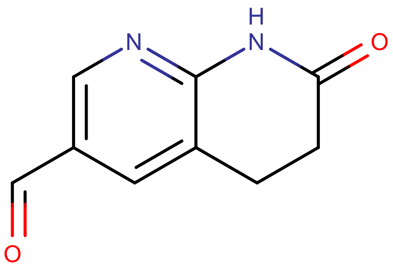

**2**{*489*}	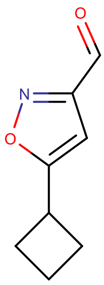
**2**{*490*}	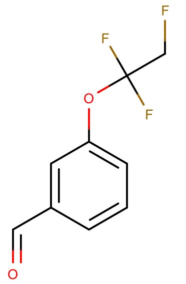
**2**{*491*}	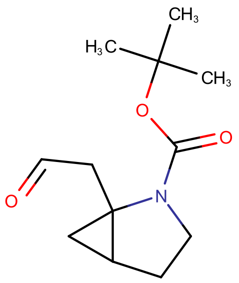
**2**{*492*}	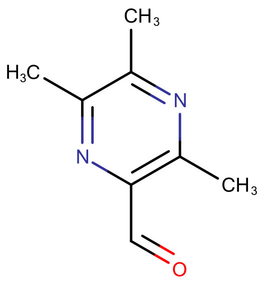

**2**{*493*}	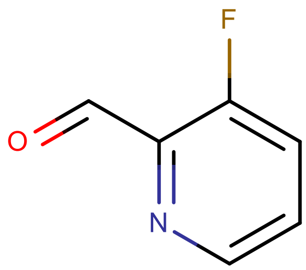
**2**{*494*}	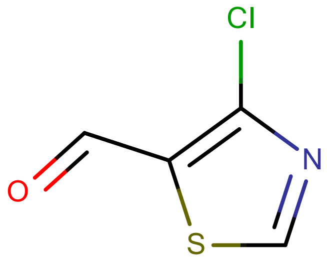
**2**{*495*}	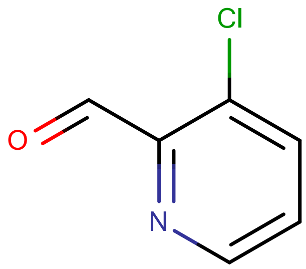
**2**{*496*}	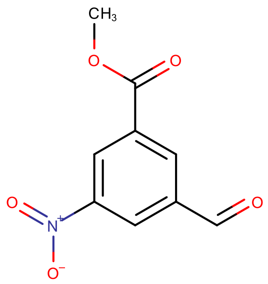

**2**{*497*}	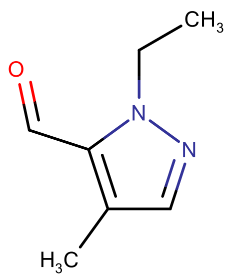
**2**{*498*}	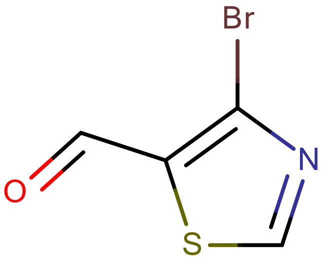
**2**{*499*}	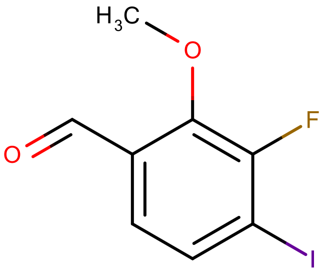
**2**{*500*}	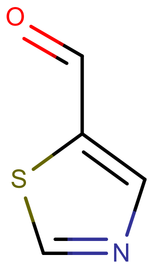

**2**{*501*}	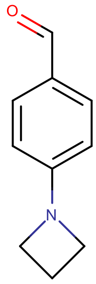
**2**{*502*}	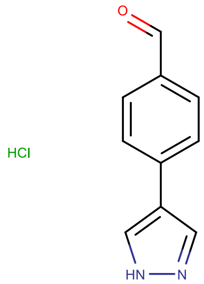
**2**{*503*}	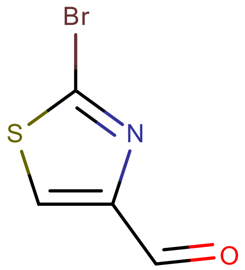
**2**{*504*}	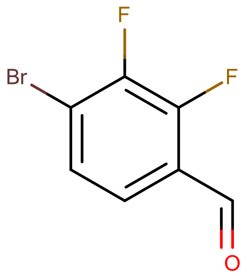

**2**{*505*}	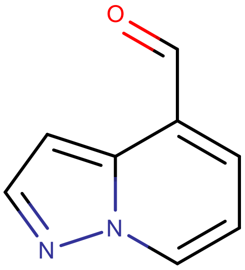
**2**{*506*}	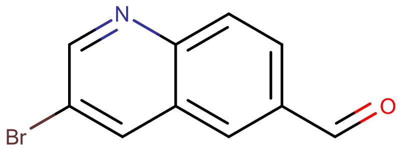
**2**{*507*}	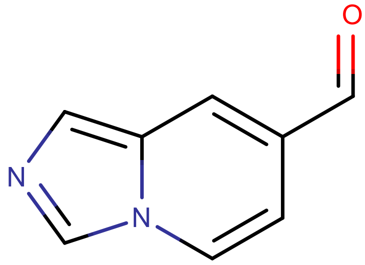
**2**{*508*}	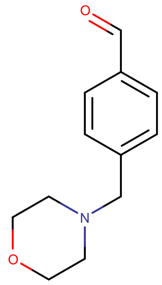

**2**{*509*}	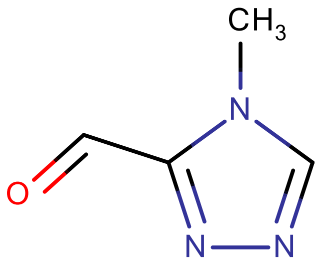
**2**{*510*}	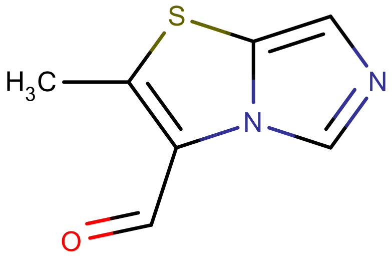
**2**{*511*}	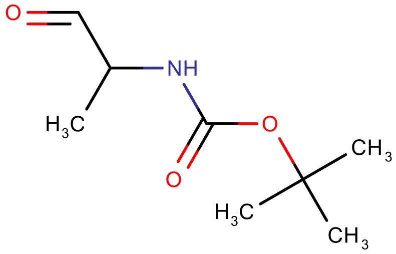
**2**{*512*}	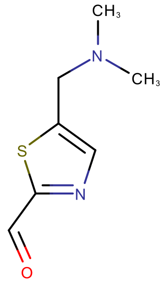

**2**{*513*}	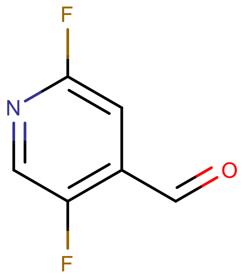
**2**{*514*}	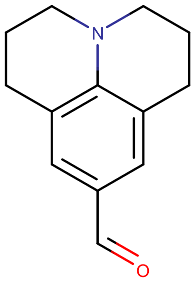
**2**{*515*}	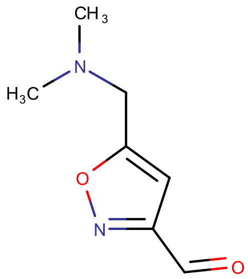
**2**{*516*}	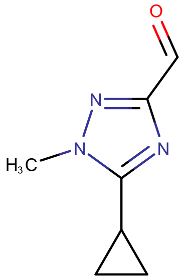

**2**{*517*}	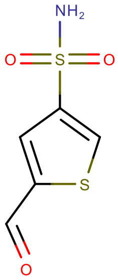
**2**{*518*}	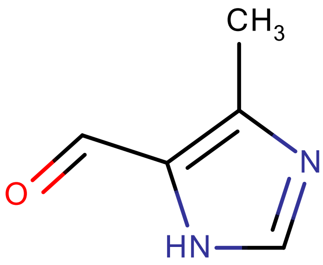
**2**{*519*}	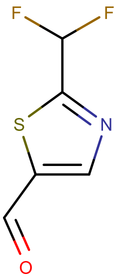
**2**{*520*}	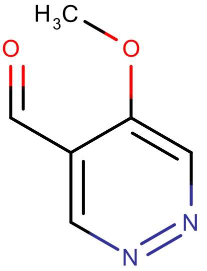

**2**{*521*}	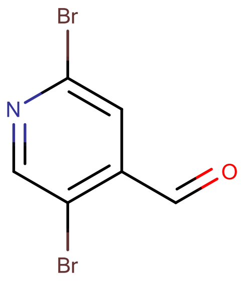
**2**{*522*}	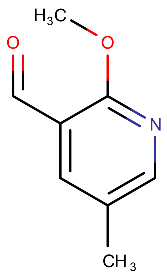
**2**{*523*}	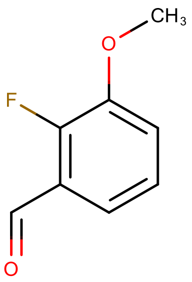
**2**{*524*}	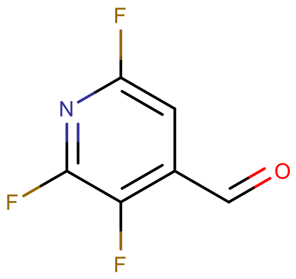

**2**{*525*}	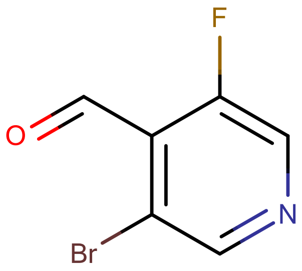
**2**{*526*}	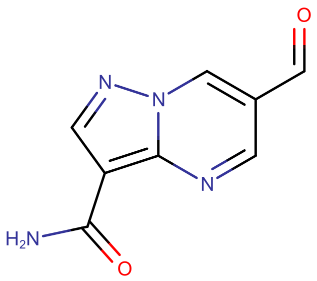
**2**{*527*}	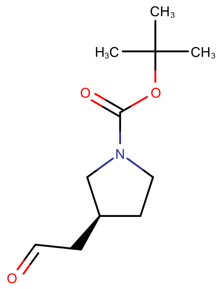
**2**{*528*}	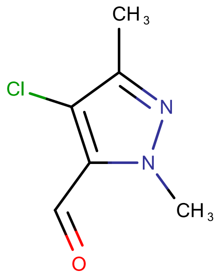

**2**{*529*}	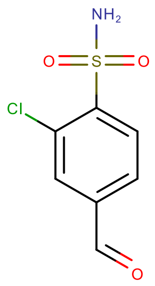
**2**{*530*}	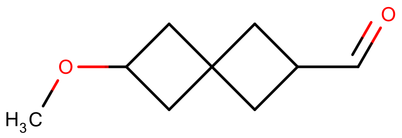
**2**{*531*}	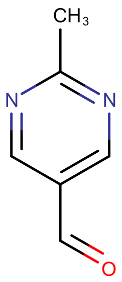
**2**{*532*}	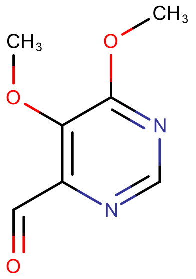

**2**{*533*}	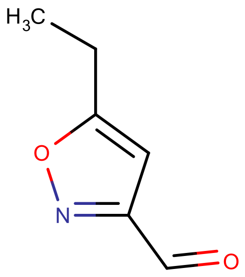
**2**{*534*}	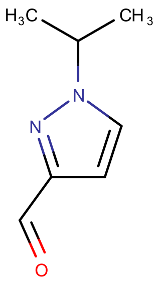
**2**{*535*}	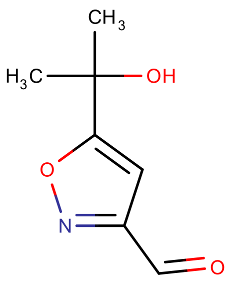
**2**{*536*}	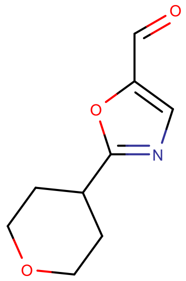

**2**{*537*}	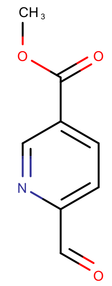
**2**{*538*}	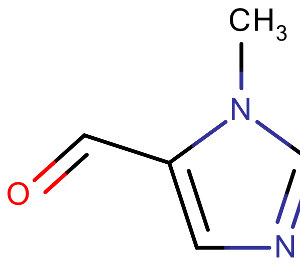
**2**{*539*}	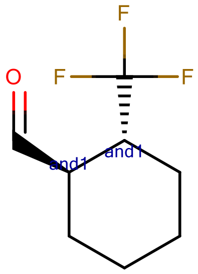
**2**{*540*}	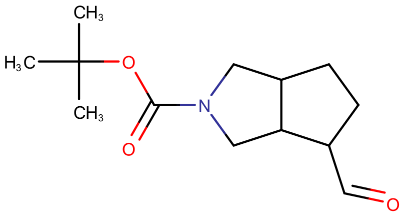

**2**{*541*}	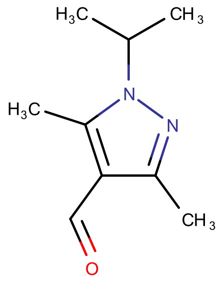
**2**{*542*}	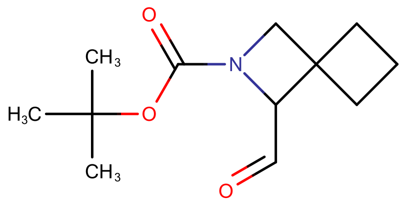
**2**{*543*}	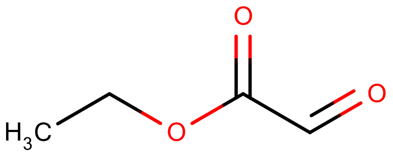
**2**{*544*}	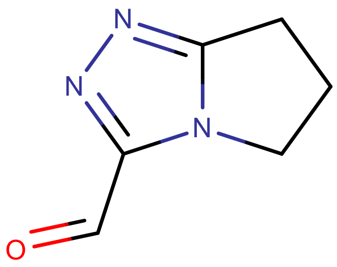

**2**{*545*}	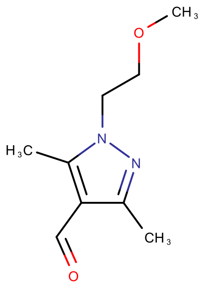
**2**{*546*}	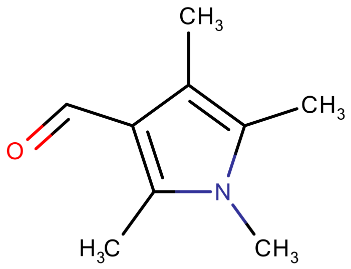
**2**{*547*}	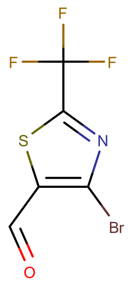
**2**{*548*}	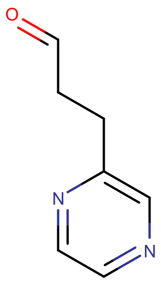

**2**{*549*}	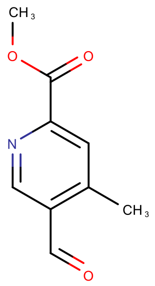
**2**{*550*}	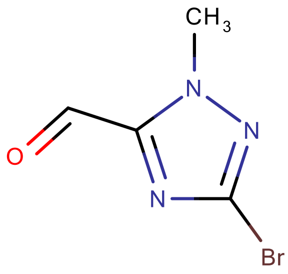
**2**{*551*}	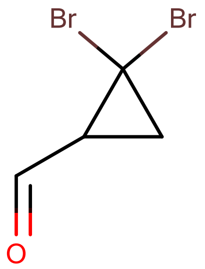
**2**{*552*}	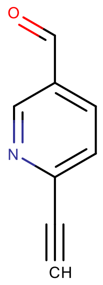

**2**{*553*}	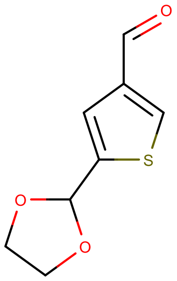
**2**{*554*}	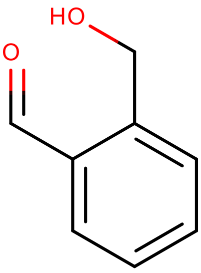
**2**{*555*}	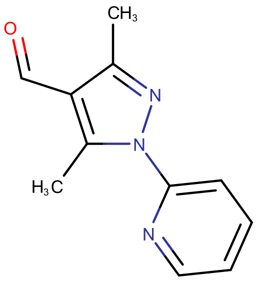
**2**{*556*}	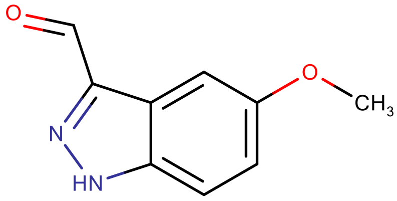

**2**{*557*}	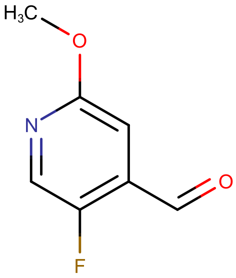
**2**{*558*}	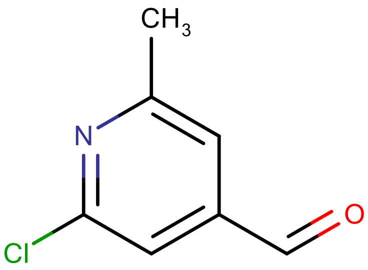
**2**{*559*}	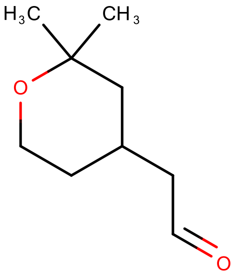
**2**{*560*}	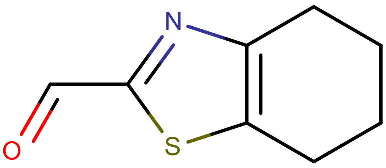

**2**{*561*}	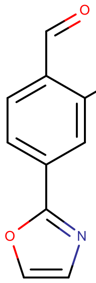
**2**{*562*}	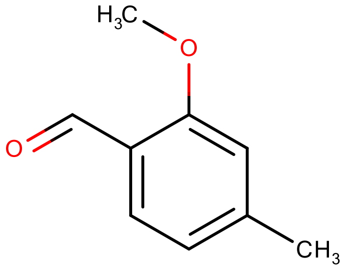
**2**{*563*}	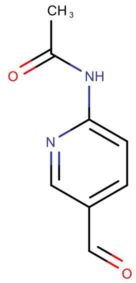
**2**{*564*}	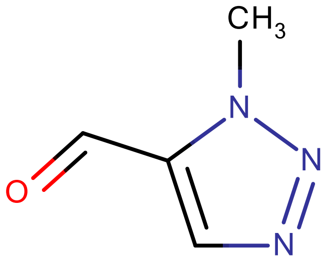

**2**{*565*}	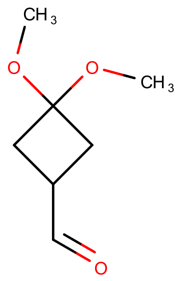
**2**{*566*}	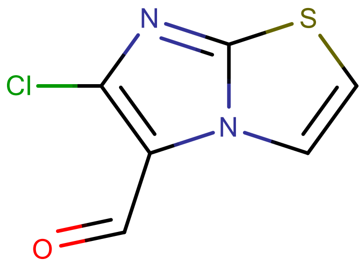
**2**{*567*}	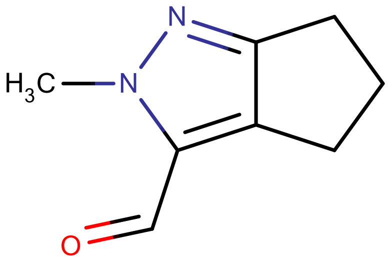
**2**{*568*}	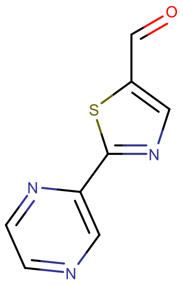

**2**{*569*}	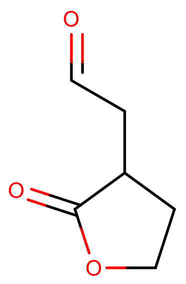
**2**{*570*}	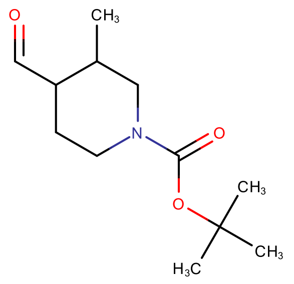
**2**{*571*}	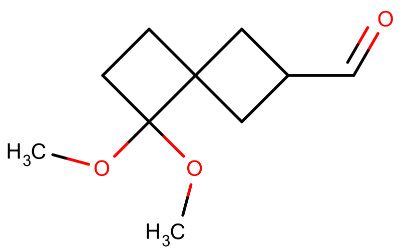
**2**{*572*}	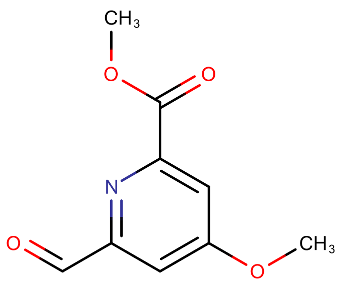

**2**{*573*}	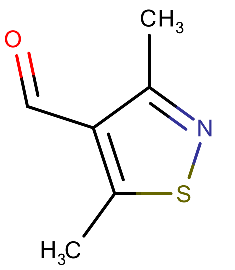
**2**{*574*}	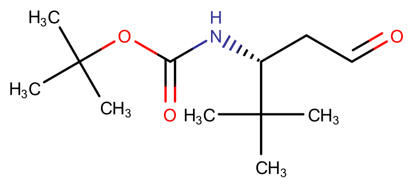
**2**{*575*}	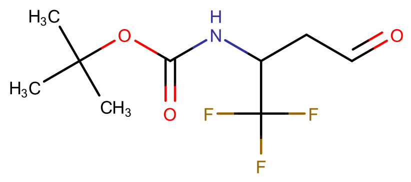
**2**{*576*}	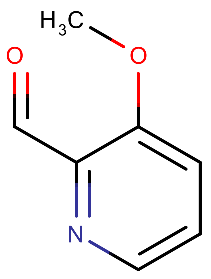

**2**{*577*}	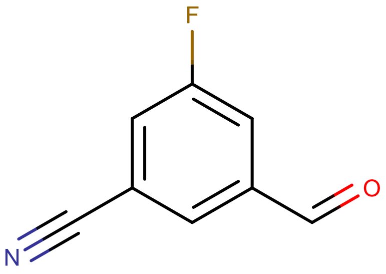
**2**{*578*}	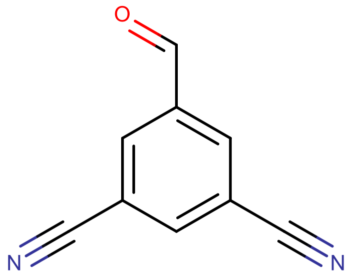
**2**{*579*}	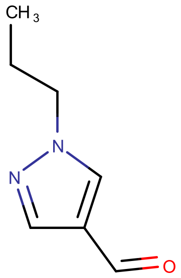
**2**{*580*}	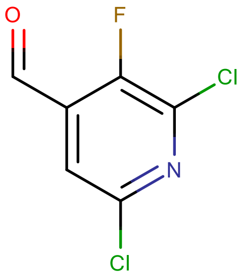

**2**{*581*}	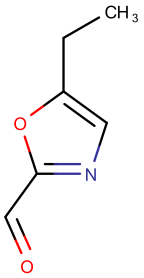
**2**{*582*}	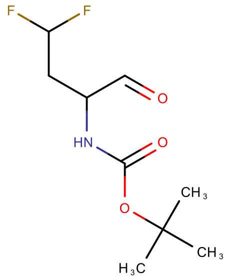
**2**{*583*}	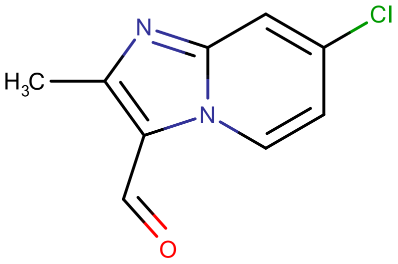
**2**{*584*}	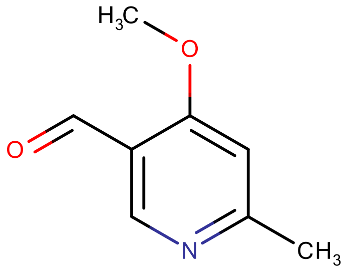

**2**{*585*}	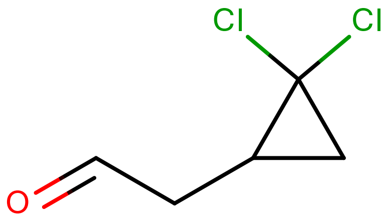
**2**{*586*}	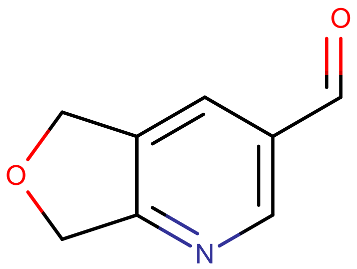
**2**{*587*}	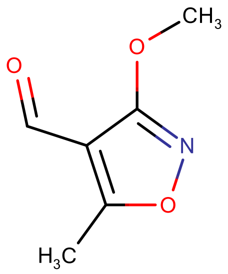
**2**{*588*}	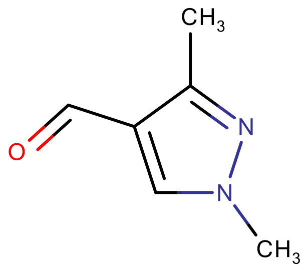

**2**{*589*}	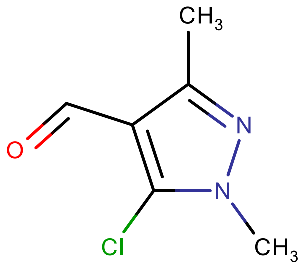
**2**{*590*}	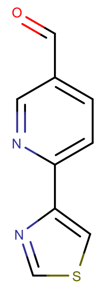
**2**{*591*}	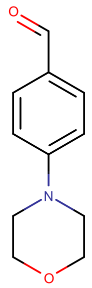
**2**{*592*}	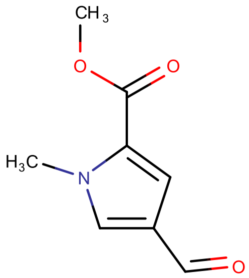

**2**{*593*}	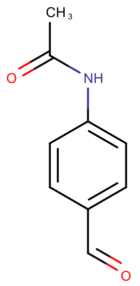
**2**{*594*}	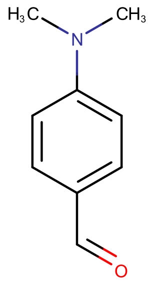
**2**{*595*}	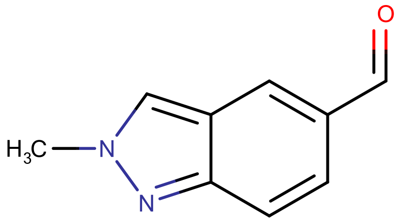
**2**{*596*}	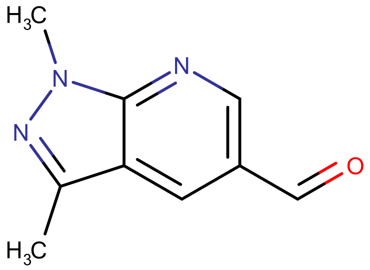

**2**{*597*}	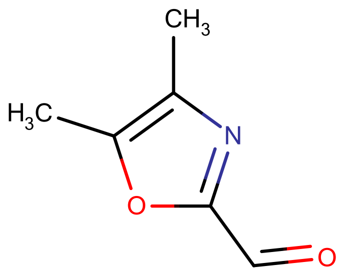
**2**{*598*}	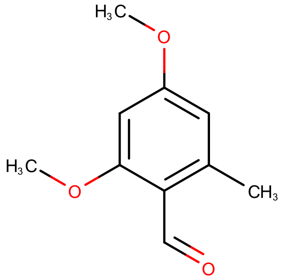
**2**{*599*}	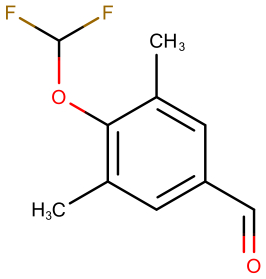
**2**{*600*}	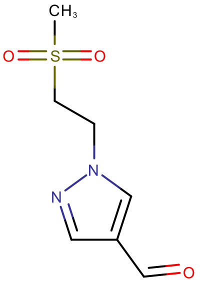

**2**{*601*}	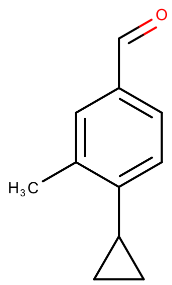
**2**{*602*}	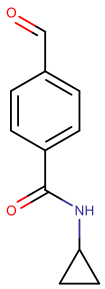
**2**{*603*}	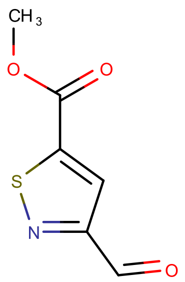
**2**{*604*}	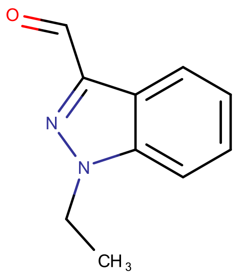

**2**{*605*}	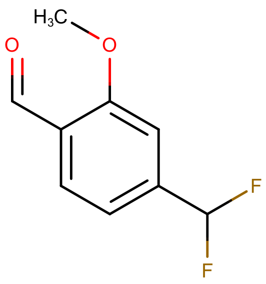
**2**{*606*}	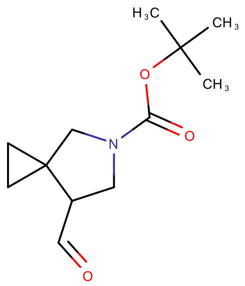
**2**{*607*}	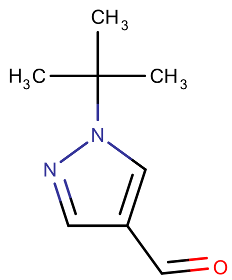
**2**{*608*}	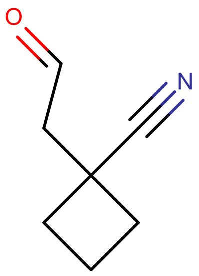

**2**{*609*}	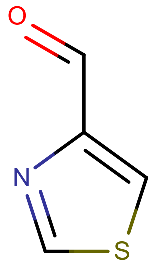
**2**{*610*}	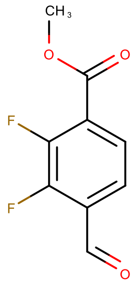
**2**{*611*}	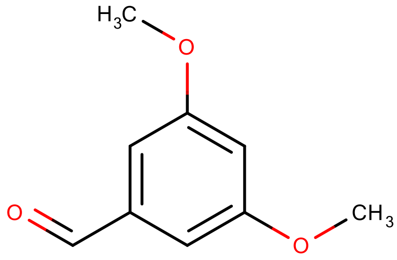
**2**{*612*}	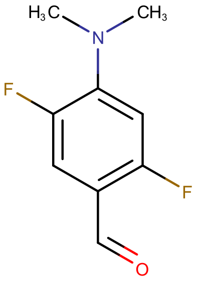

**2**{*613*}	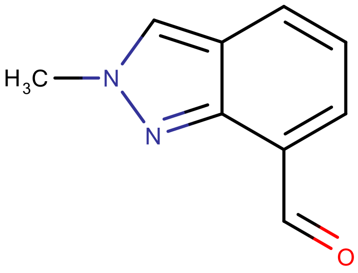
**2**{*614*}	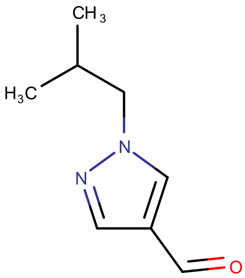
**2**{*615*}	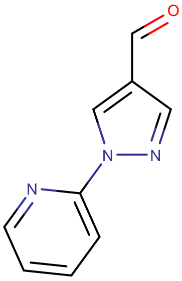
**2**{*616*}	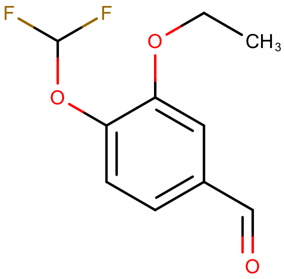

**2**{*617*}	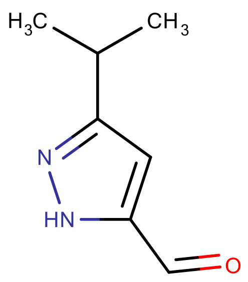
**2**{*618*}	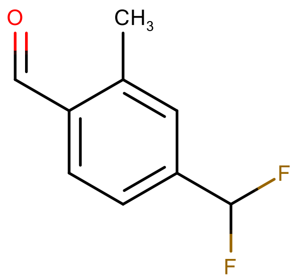
**2**{*619*}	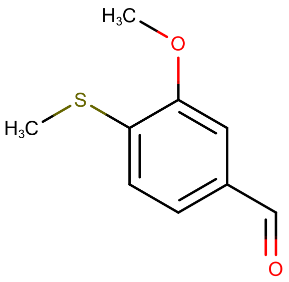
**2**{*620*}	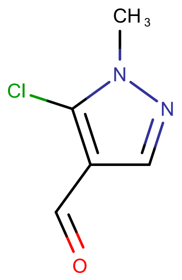

**2**{*621*}	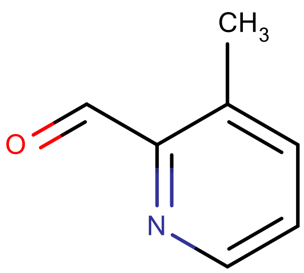
**2**{*622*}	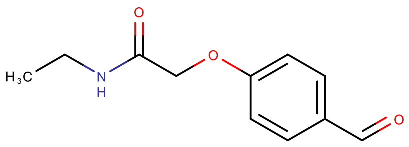
**2**{*623*}	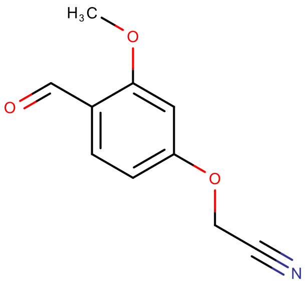
**2**{*624*}	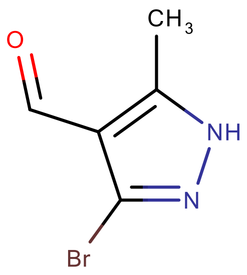

**2**{*625*}	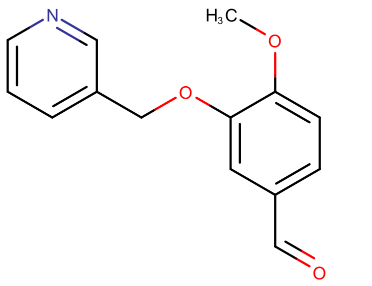
**2**{*626*}	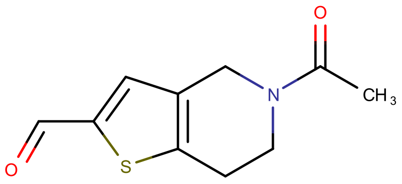
**2**{*627*}	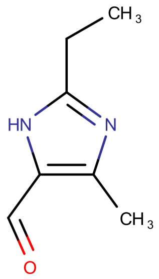
**2**{*628*}	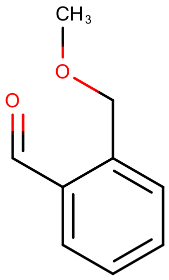

**2**{*629*}	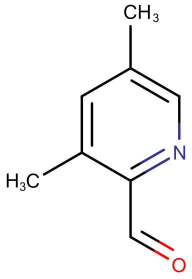
**2**{*630*}	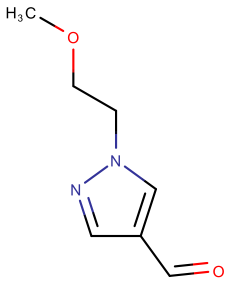
**2**{*631*}	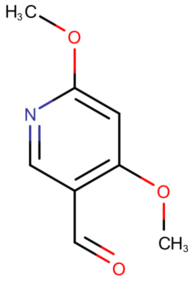
**2**{*632*}	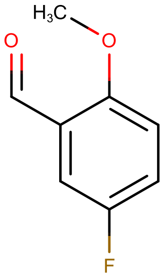

**2**{*633*}	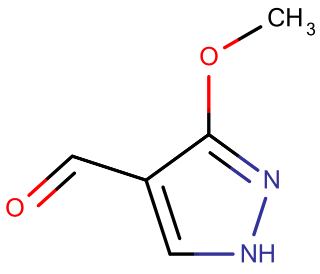
**2**{*634*}	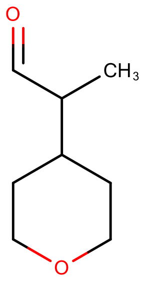
**2**{*635*}	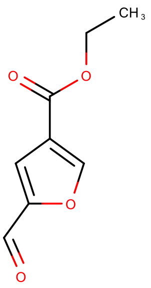
**2**{*636*}	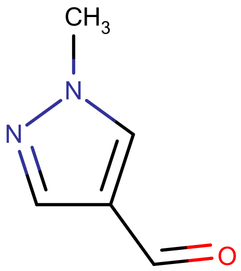

**2**{*637*}	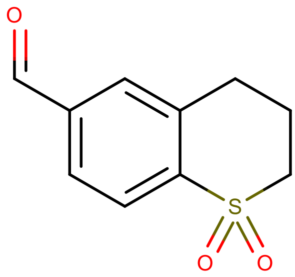
**2**{*638*}	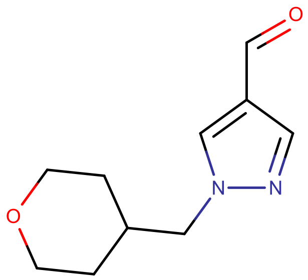
**2**{*639*}	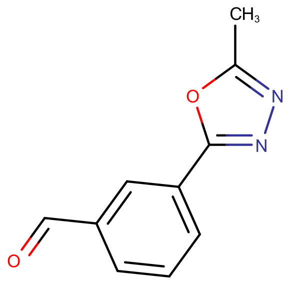
**2**{*640*}	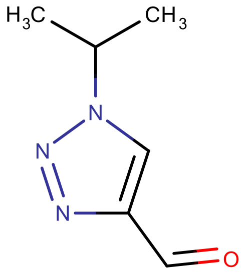

**2**{*641*}	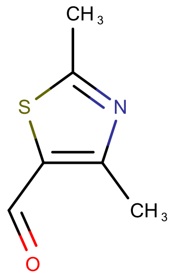
**2**{*642*}	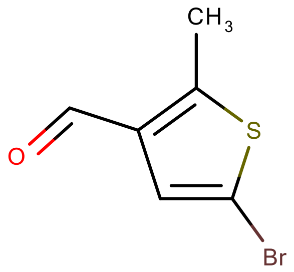
**2**{*643*}	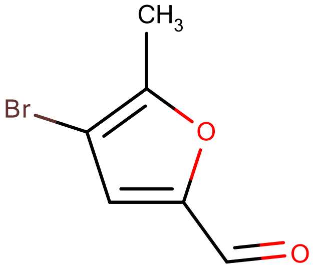
**2**{*644*}	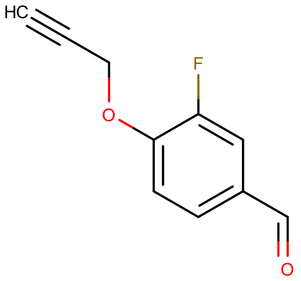

**2**{*645*}	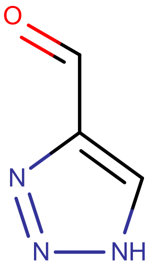
**2**{*646*}	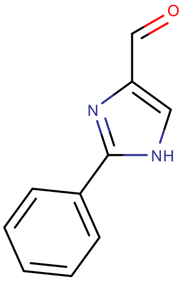
**2**{*647*}	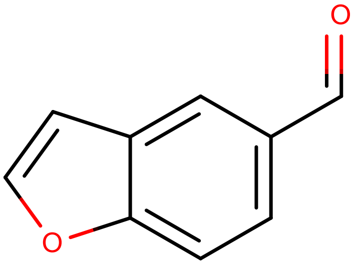
**2**{*648*}	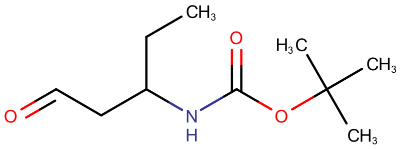

**2**{*649*}	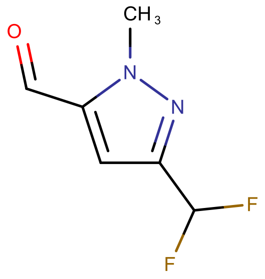
**2**{*650*}	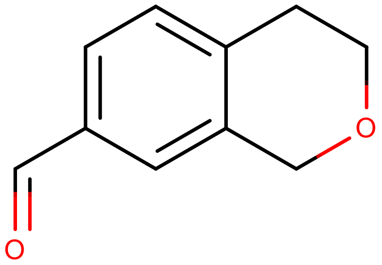
**2**{*651*}	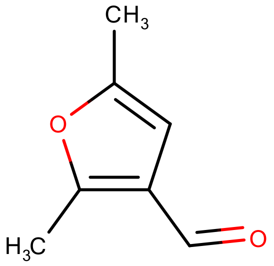
**2**{*652*}	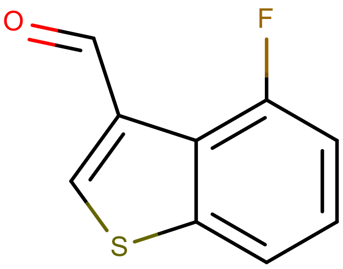

**2**{*653*}	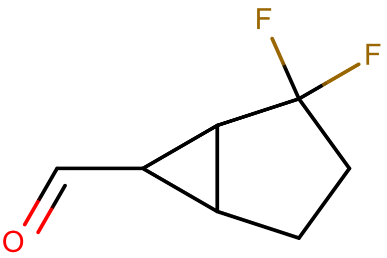
**2**{*654*}	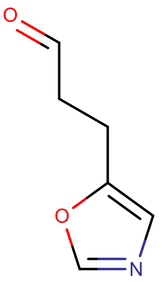
**2**{*655*}	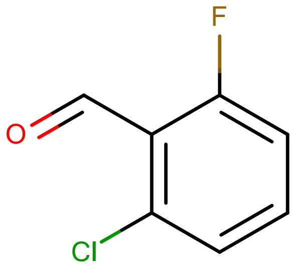
**2**{*656*}	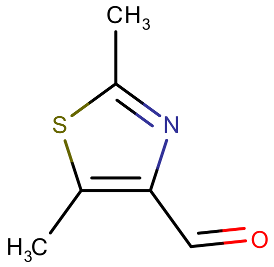

**2**{*657*}	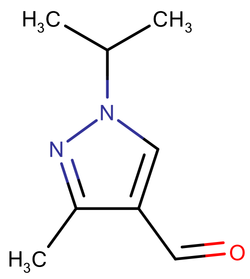
**2**{*658*}	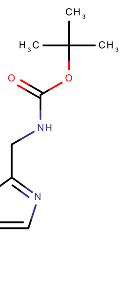
**2**{*659*}	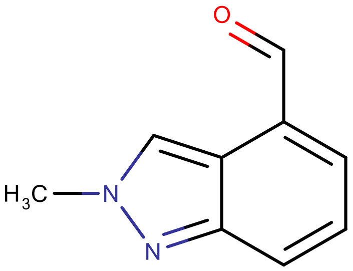
**2**{*660*}	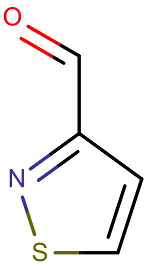

**2**{*661*}	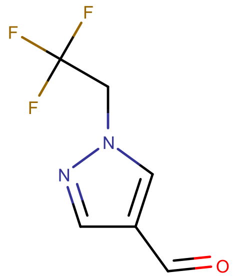
**2**{*662*}	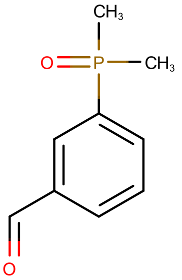
**2**{*663*}	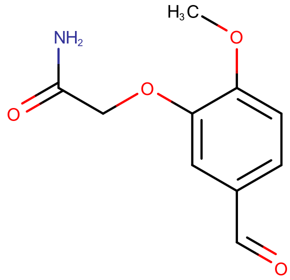
**2**{*664*}	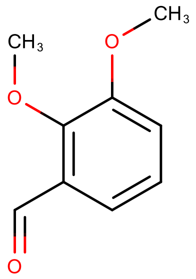

**2**{*665*}	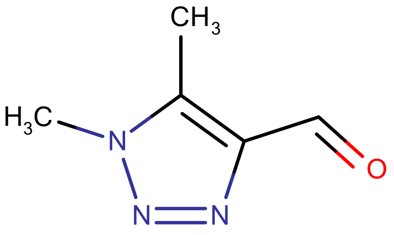
**2**{*666*}	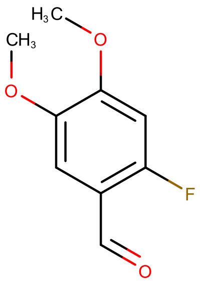
**2**{*667*}	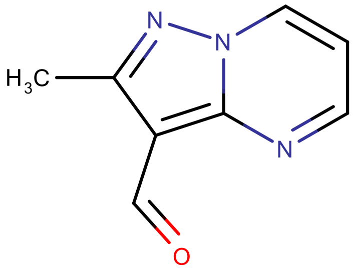
**2**{*668*}	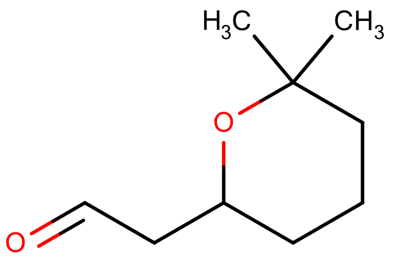

**2**{*669*}	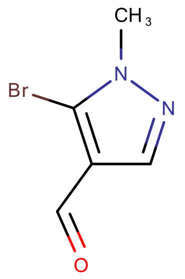
**2**{*670*}	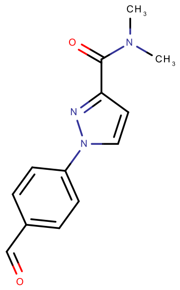
**2**{*671*}	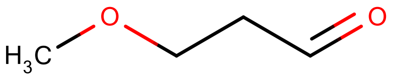
**2**{*672*}	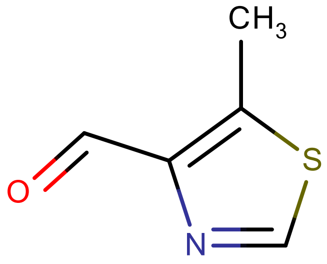

**2**{*673*}	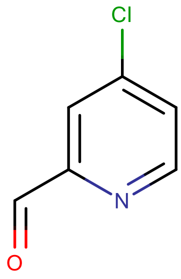
**2**{*674*}	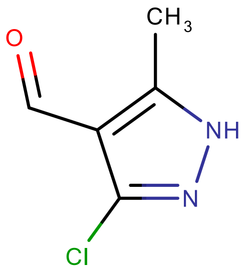
**2**{*675*}	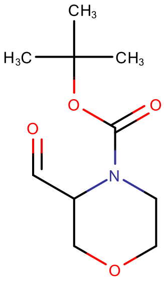
**2**{*676*}	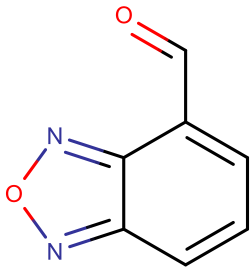

**2**{*677*}	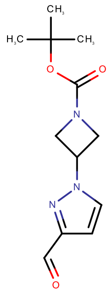
**2**{*678*}	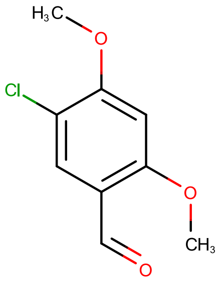
**2**{*679*}	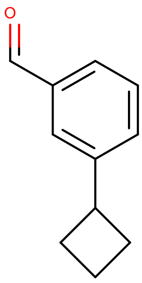
**2**{*680*}	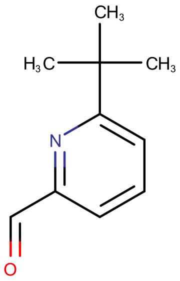

**2**{*681*}	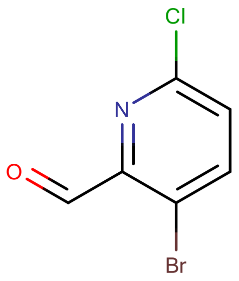
**2**{*682*}	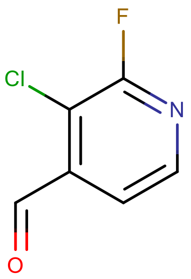
**2**{*683*}	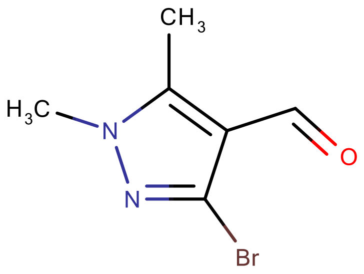
**2**{*684*}	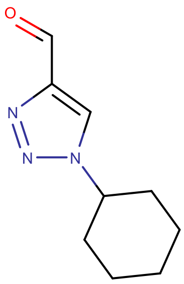

**2**{*685*}	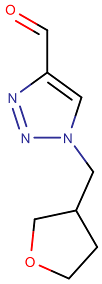
**2**{*686*}	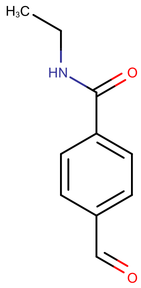
**2**{*687*}	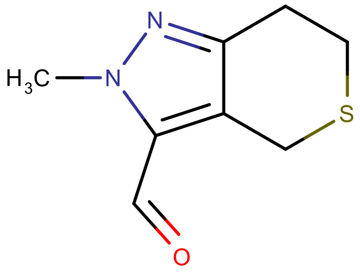
**2**{*688*}	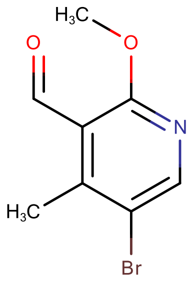

**2**{*689*}	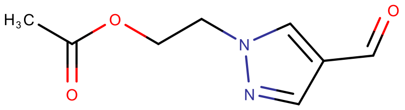
**2**{*690*}	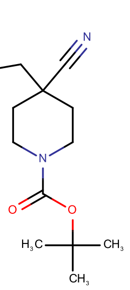
**2**{*691*}	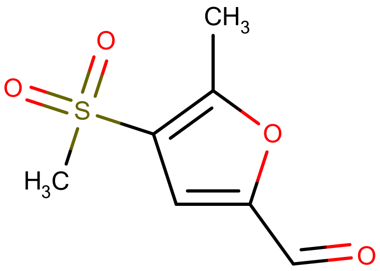
**2**{*692*}	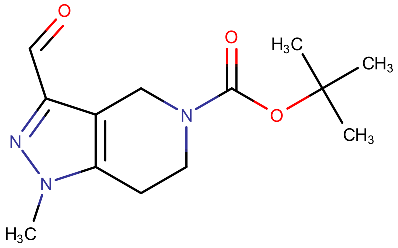
**2**{*693*}	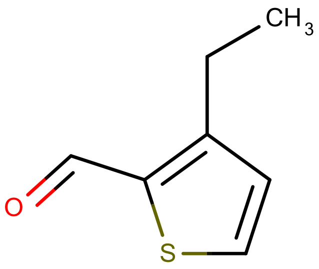
**2**{*694*}	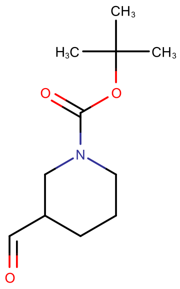
**2**{*695*}	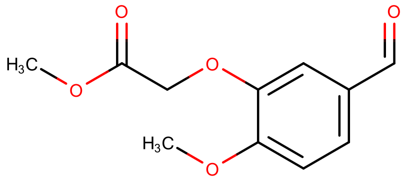
**2**{*696*}	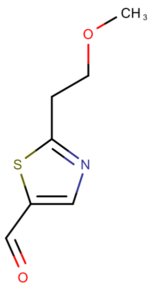

**2**{*697*}	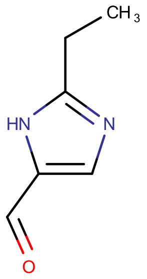
**2**{*698*}	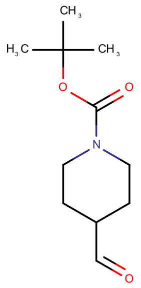
**2**{*699*}	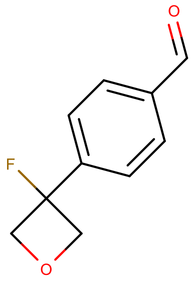
**2**{*700*}	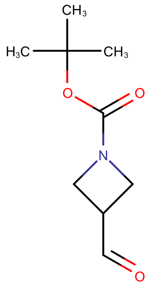

**2**{*701*}	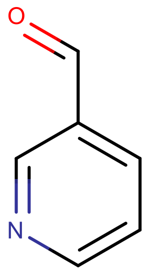
**2**{*702*}	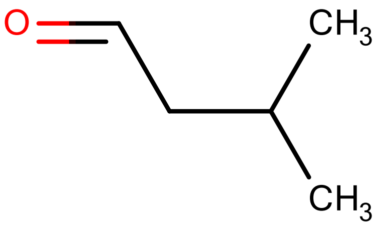
**2**{*703*}	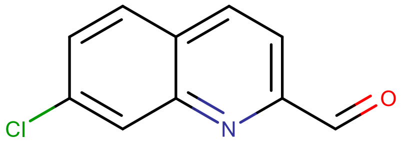
**2**{*704*}	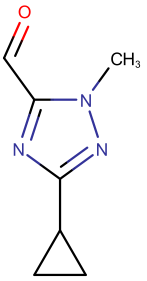

**2**{*705*}	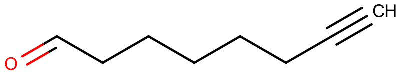
**2**{*706*}	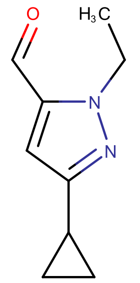
**2**{*707*}	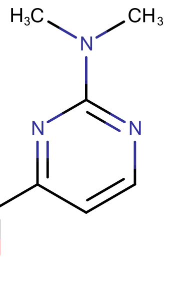
**2**{*708*}	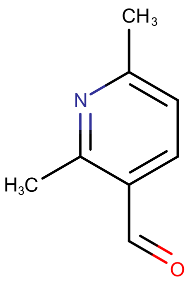

**2**{*709*}	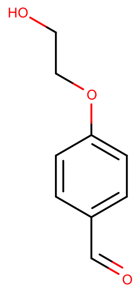
**2**{*710*}	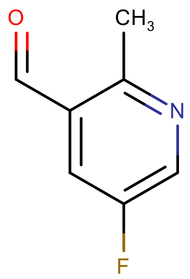
**2**{*711*}	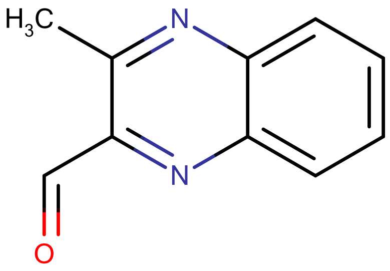
**2**{*712*}	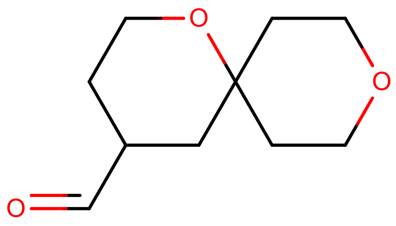

**2**{*713*}	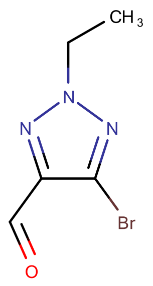
**2**{*714*}	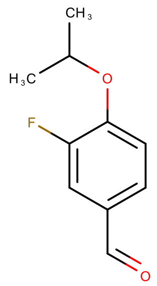
**2**{*715*}	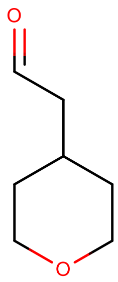
**2**{*716*}	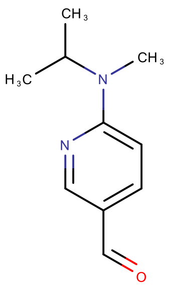

**2**{*717*}	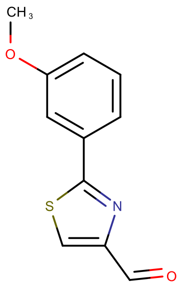
**2**{*718*}	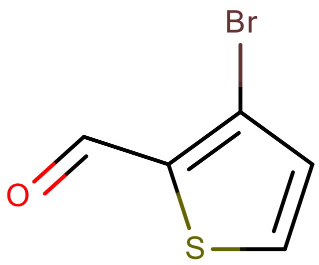
**2**{*719*}	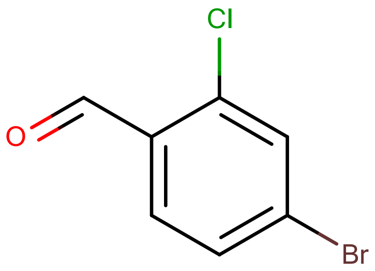
**2**{*720*}	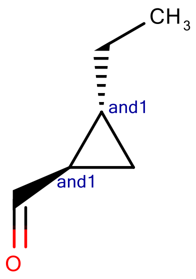

**2**{*721*}	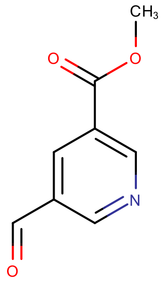
**2**{*722*}	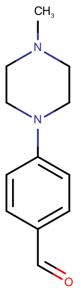
**2**{*723*}	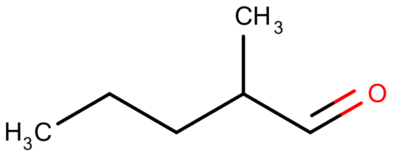
**2**{*724*}	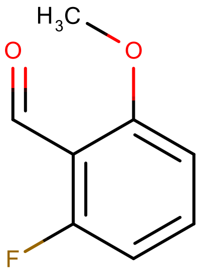

**2**{*725*}	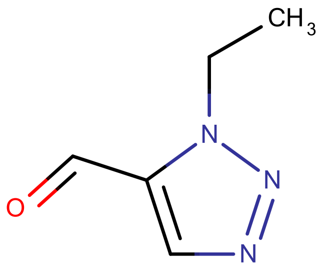
**2**{*726*}	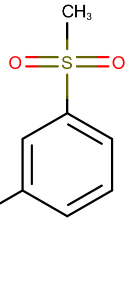
**2**{*727*}	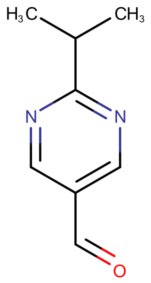
**2**{*728*}	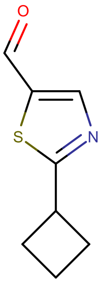

**2**{*729*}	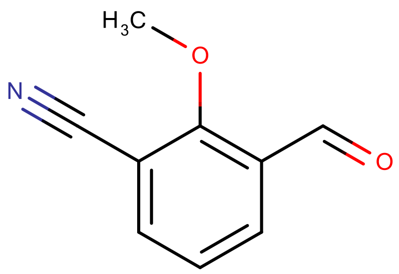
**2**{*730*}	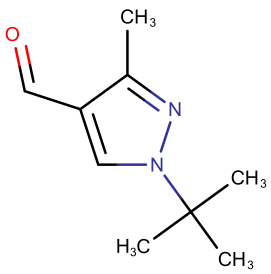
**2**{*731*}	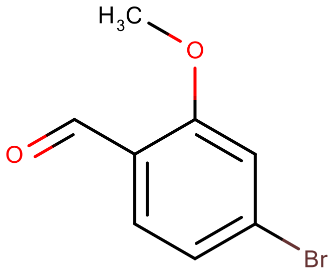
**2**{*732*}	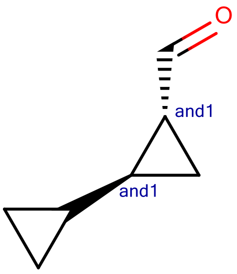

**2**{*733*}	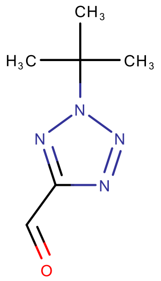
**2**{*734*}	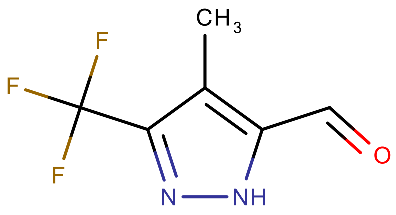
**2**{*735*}	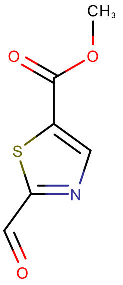
**2**{*736*}	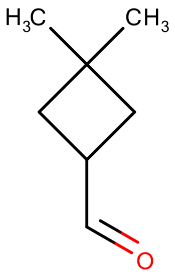

**2**{*737*}	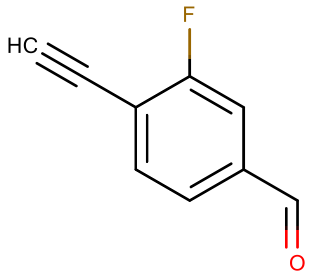
**2**{*738*}	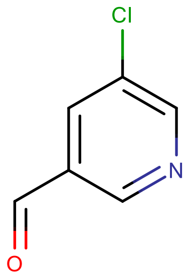
**2**{*739*}	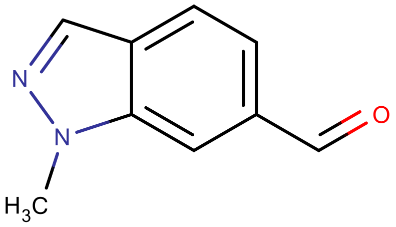
**2**{*740*}	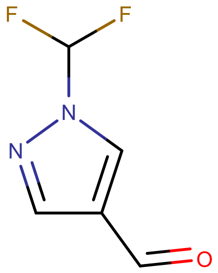

**2**{*741*}	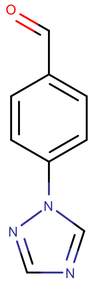
**2**{*742*}	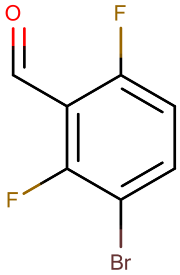
**2**{*743*}	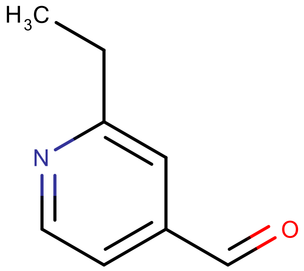
**2**{*744*}	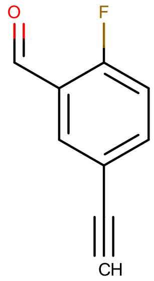

**2**{*745*}	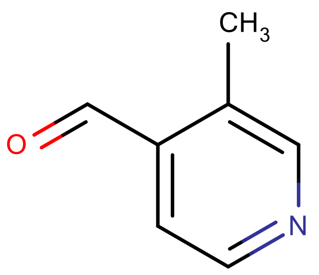
**2**{*746*}	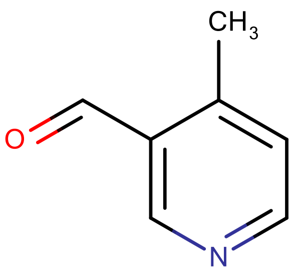
**2**{*747*}	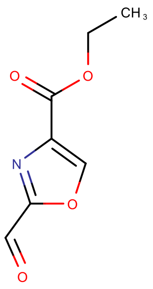
**2**{*748*}	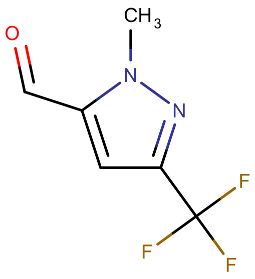

**2**{*749*}	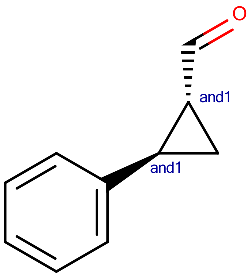
**2**{*750*}	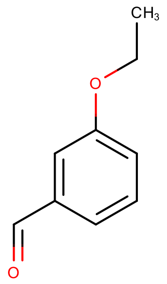
**2**{*751*}	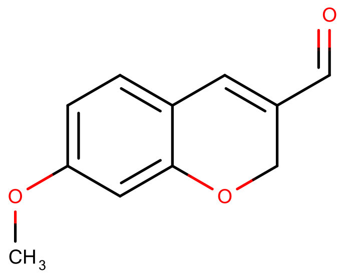
**2**{*752*}	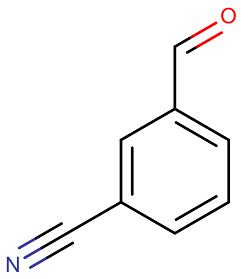

**2**{*753*}	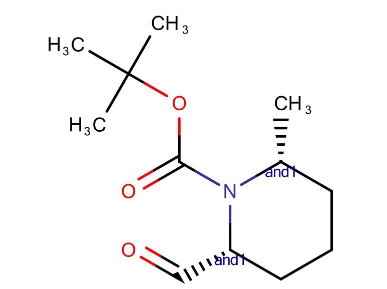
**2**{*754*}	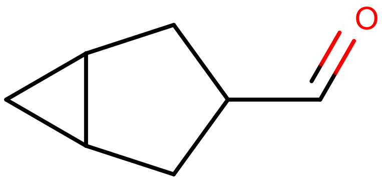
**2**{*755*}	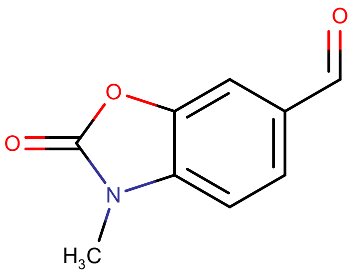
**2**{*756*}	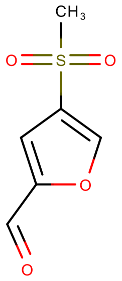

**2**{*757*}	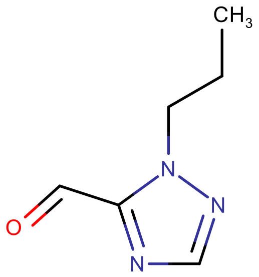
**2**{*758*}	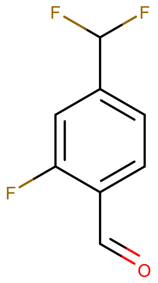
**2**{*759*}	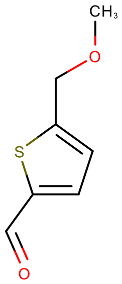
**2**{*760*}	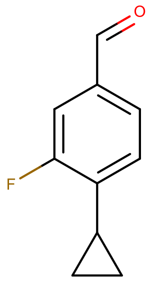

**2**{*761*}	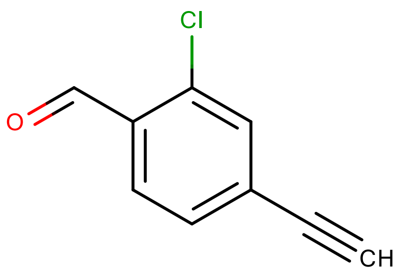
**2**{*762*}	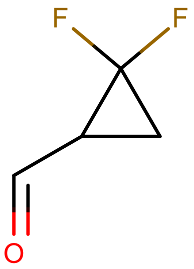
**2**{*763*}	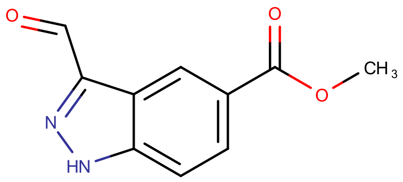
**2**{*764*}	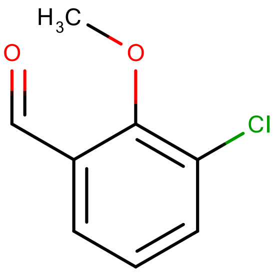

**2**{*765*}	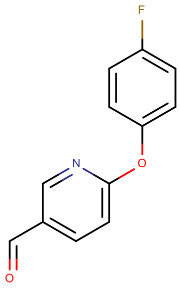
**2**{*766*}	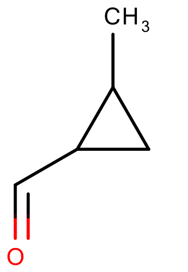
**2**{*767*}	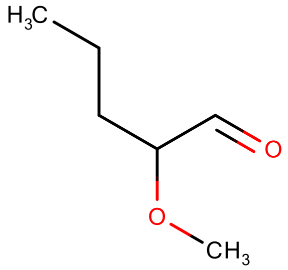
**2**{*768*}	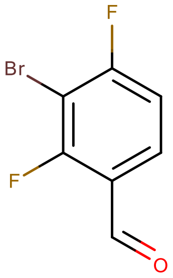

**2**{*769*}	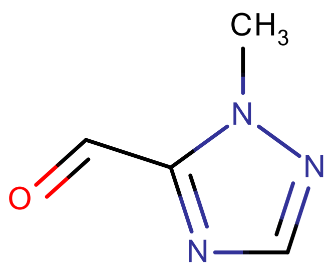
**2**{*770*}	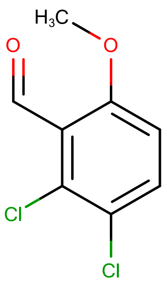
**2**{*771*}	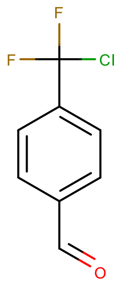
**2**{*772*}	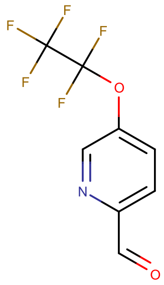

**2**{*773*}	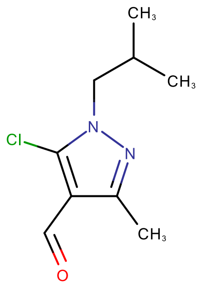
**2**{*774*}	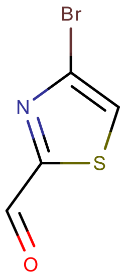
**2**{*775*}	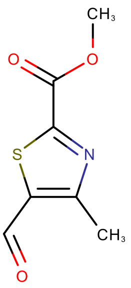
**2**{*776*}	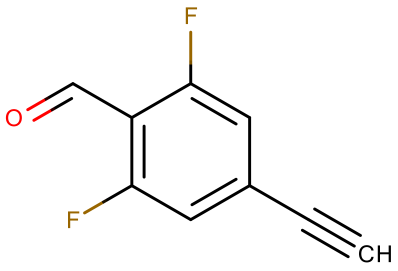

**2**{*777*}	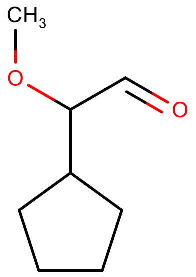
**2**{*778*}	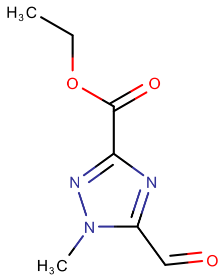
**2**{*779*}	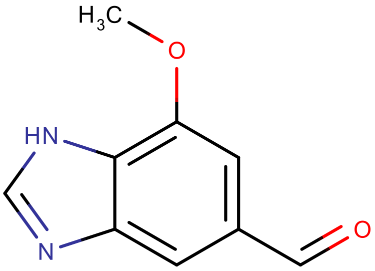
**2**{*780*}	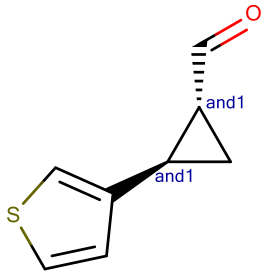
**2**{*781*}	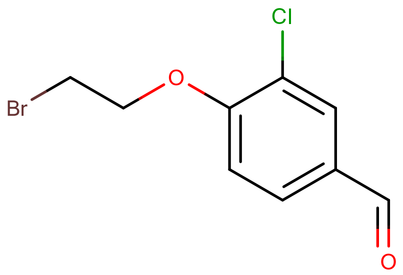
**2**{*782*}	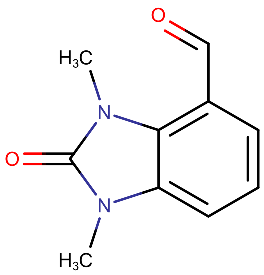
**2**{*783*}	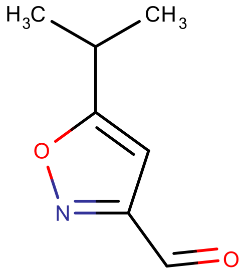
**2**{*784*}	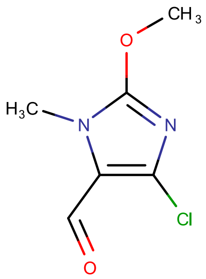

**2**{*785*}	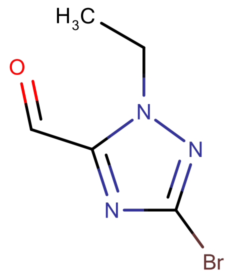
**2**{*786*}	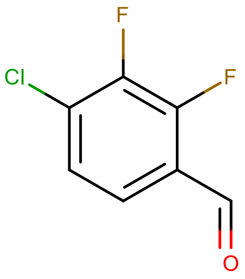
**2**{*787*}	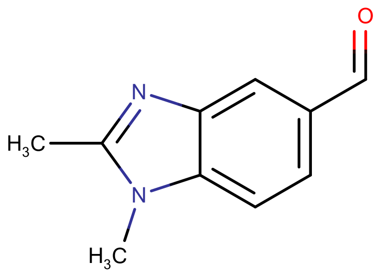
**2**{*788*}	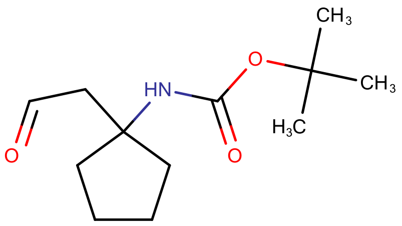

**2**{*789*}	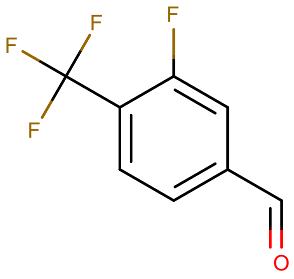
**2**{*790*}	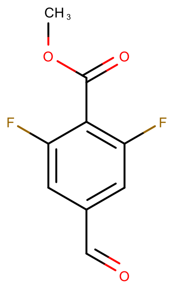
**2**{*791*}	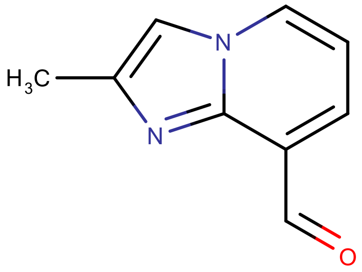
**2**{*792*}	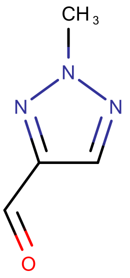

**2**{*793*}	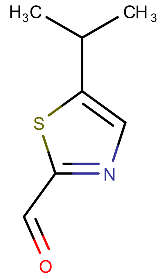
**2**{*794*}	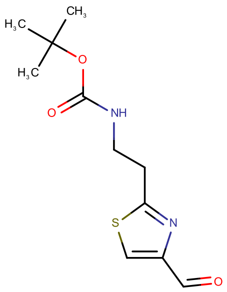
**2**{*795*}	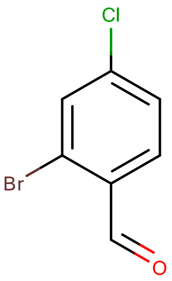
**2**{*796*}	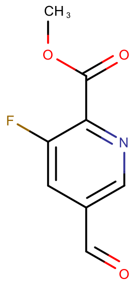

**2**{*797*}	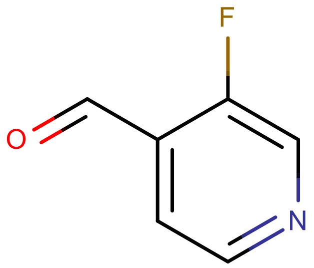
**2**{*798*}	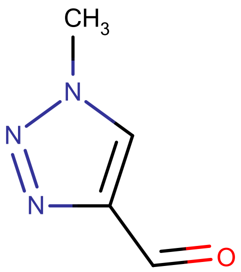
**2**{*799*}	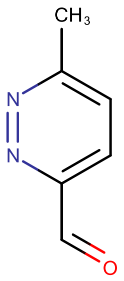
**2**{*800*}	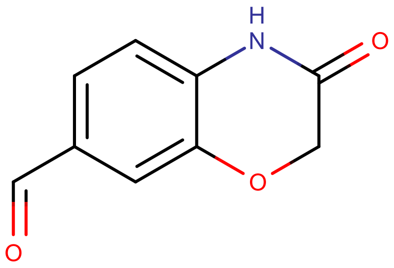

**2**{*801*}	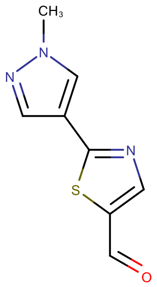
**2**{*802*}	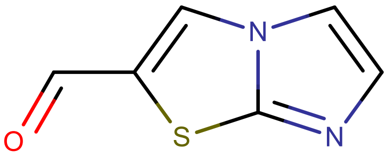
**2**{*803*}	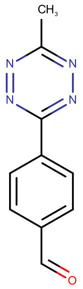
**2**{*804*}	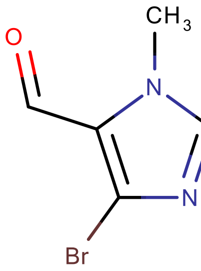

**2**{*805*}	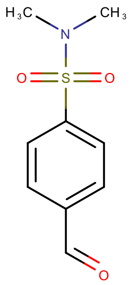
**2**{*806*}	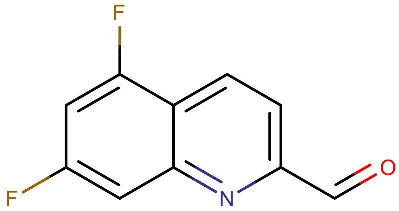
**2**{*807*}	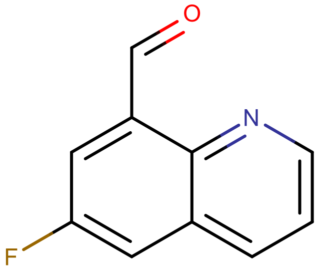
**2**{*808*}	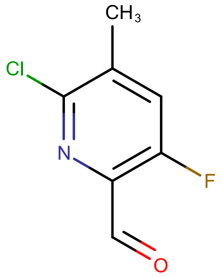

**2**{*809*}	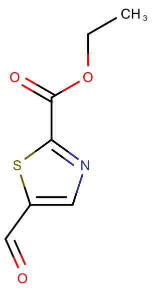
**2**{*810*}	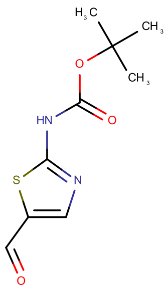
**2**{*811*}	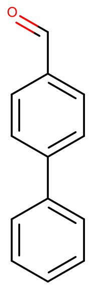
**2**{*812*}	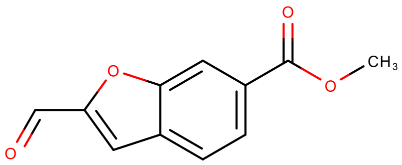

**2**{*813*}	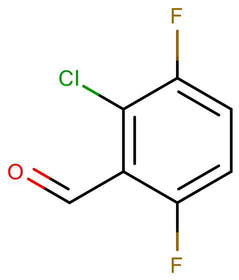
**2**{*814*}	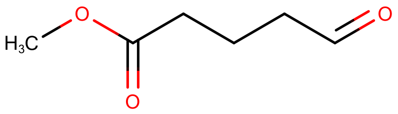
**2**{*815*}	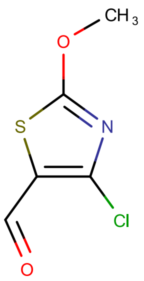
**2**{*816*}	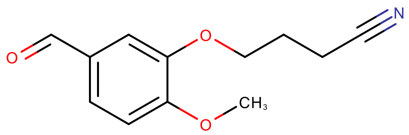

**2**{*817*}	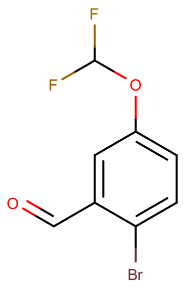
**2**{*818*}	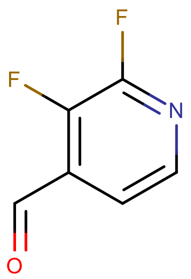
**2**{*819*}	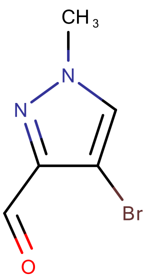
**2**{*820*}	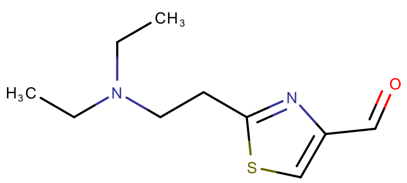

**2**{*821*}	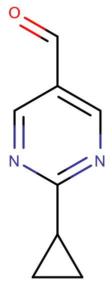
**2**{*822*}	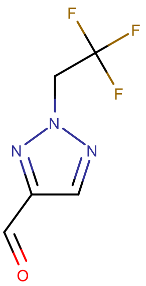
**2**{*823*}	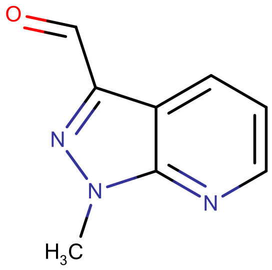
**2**{*824*}	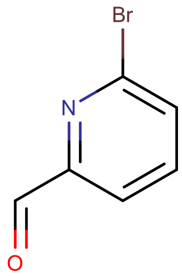

**2**{*825*}	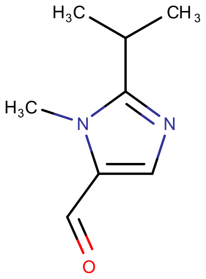
**2**{*826*}	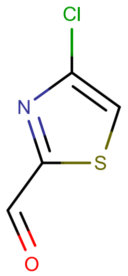
**2**{*827*}	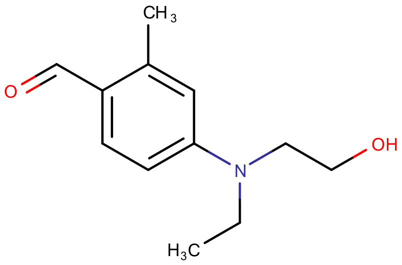
**2**{*828*}	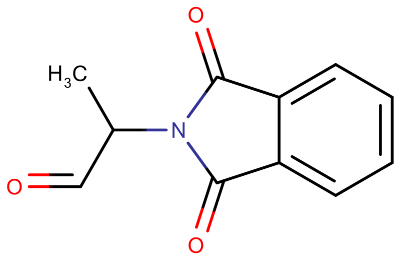

**2**{*829*}	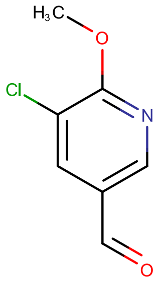
**2**{*830*}	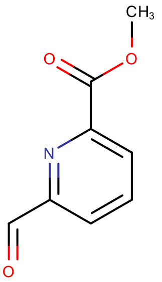
**2**{*831*}	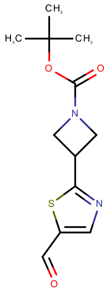
**2**{*832*}	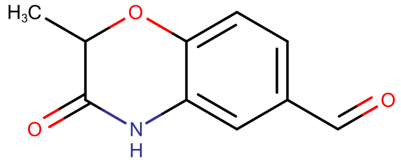

**2**{*833*}	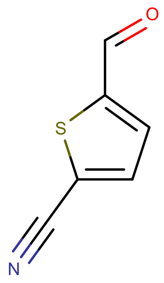
**2**{*834*}	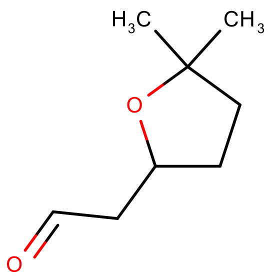
**2**{*835*}	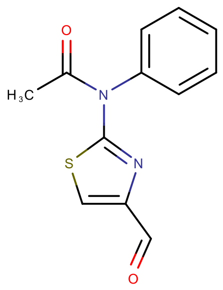
**2**{*836*}	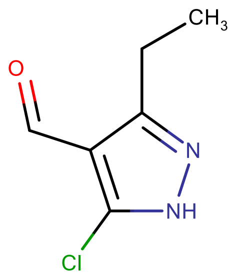

**2**{*837*}	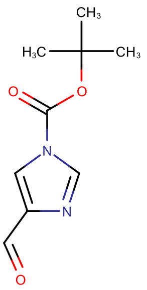
**2**{*838*}	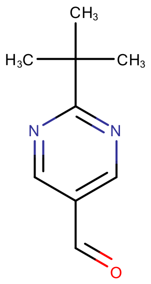
**2**{*839*}	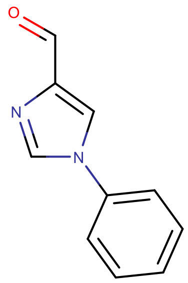
**2**{*840*}	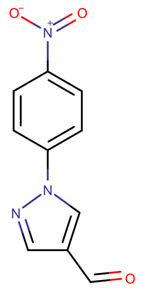

**2**{*841*}	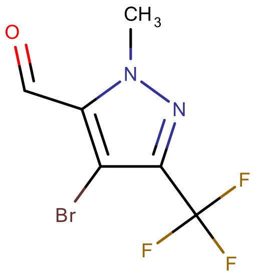	**2**{*845*}	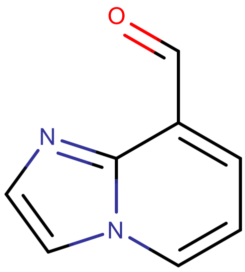
**2**{*842*}	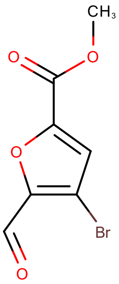	**2**{*846*}	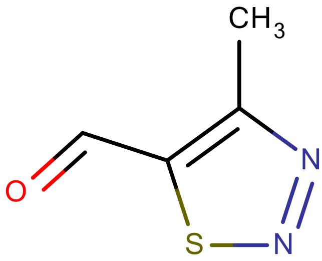
**2**{*843*}	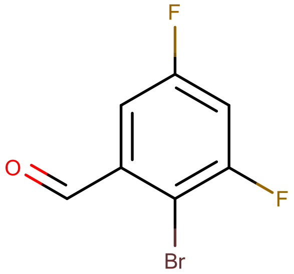	**2**{*847*}	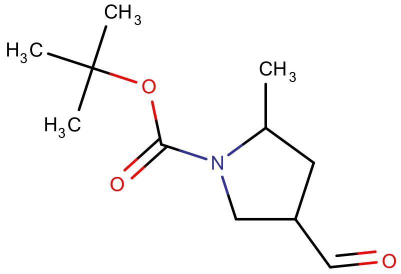
**2**{*844*}	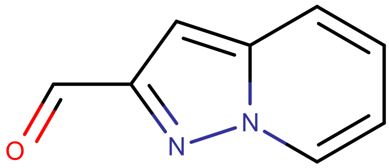	**2**{*848*}	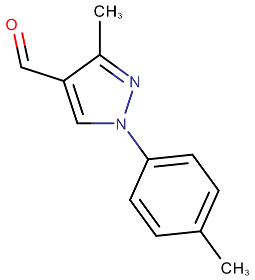

**2**{*849*}	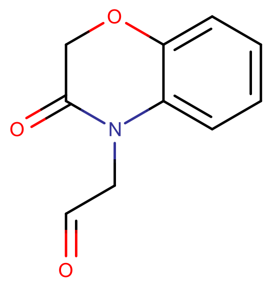
**2**{*850*}	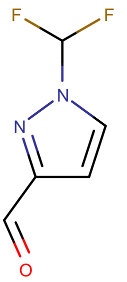
**2**{*851*}	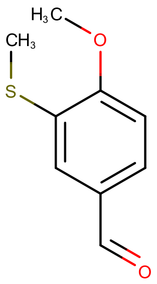
**2**{*852*}	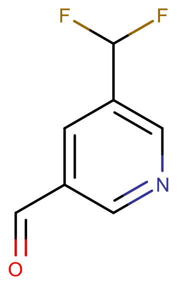

**2**{*853*}	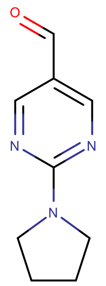
**2**{*854*}	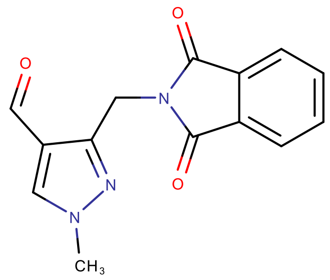
**2**{*855*}	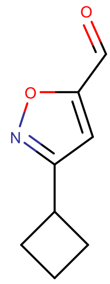
**2**{*856*}	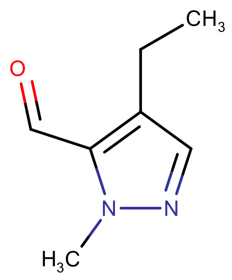

**2**{*857*}	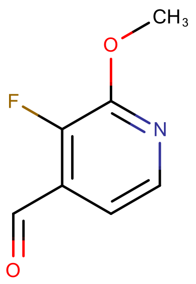
**2**{*858*}	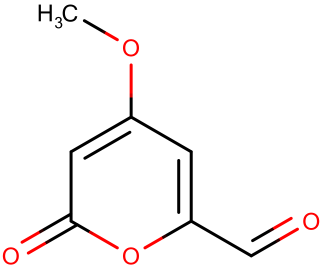
**2**{*859*}	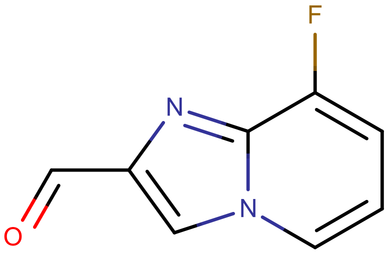
**2**{*860*}	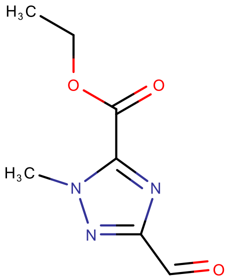

**2**{*861*}	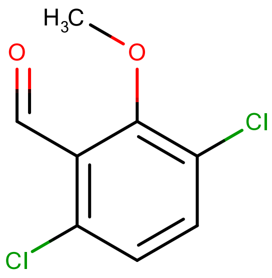
**2**{*862*}	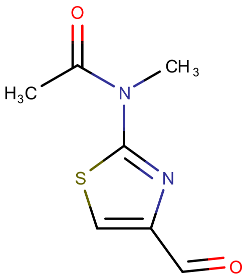
**2**{*863*}	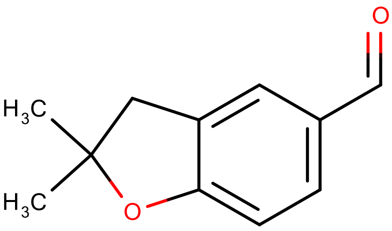
**2**{*864*}	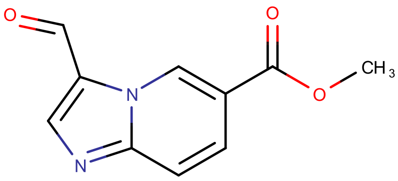

**2**{*865*}	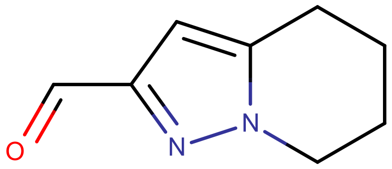
**2**{*866*}	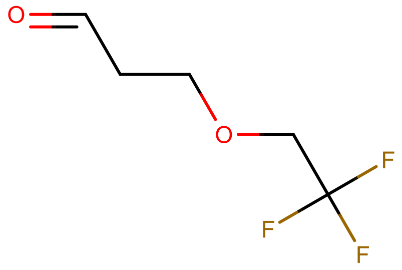
**2**{*867*}	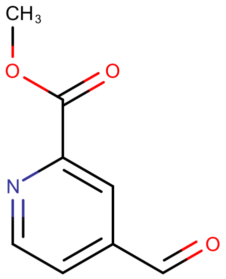
