# Supplementary material for: Generation of multimillion chemical space based on the parallel Groebke–Blackburn–Bienaymé reaction
Source: Beilstein J Org Chem. 2024 Jul 16;20:1604–13. doi: 10.3762/bjoc.20.143 (PMC11285076; doi:10.3762/bjoc.20.143)
Supplement: File 1 — Structures of reactants 1, 2, and 3. [file Beilstein_J_Org_Chem-20-1604-s001.zip › Structures of substrates 3.pdf]

Structures of reactants 3

ID	Structure
**3**{*1*}	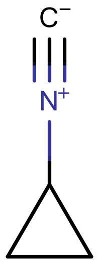
**3**{*2*}	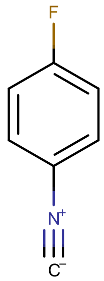
**3**{*3*}	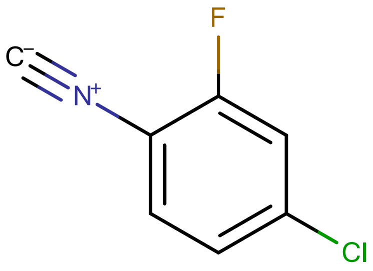
**3**{*4*}	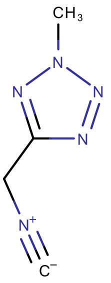
**3**{*5*}	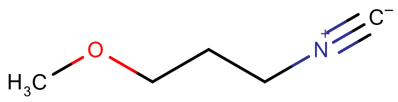
**3**{*6*}	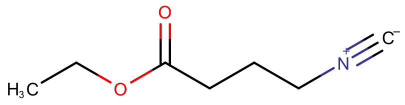
**3**{*7*}	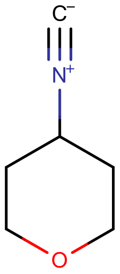
**3**{*8*}	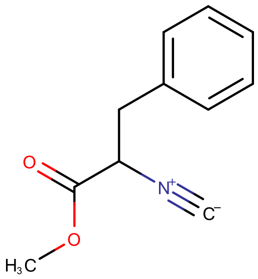

**3**{*9*}	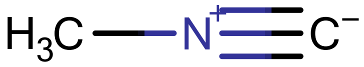
**3**{*10*}	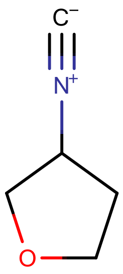
**3**{*11*}	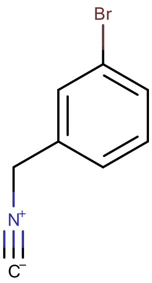
**3**{*12*}	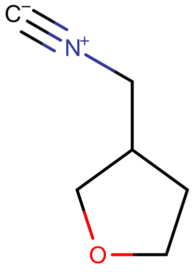

**3**{*13*}	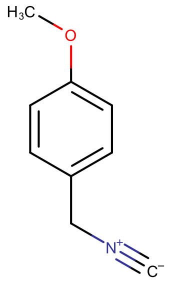
**3**{*14*}	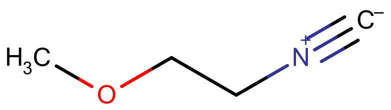
**3**{*15*}	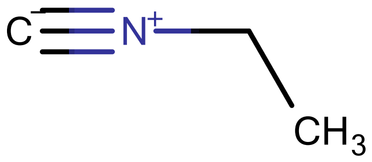
**3**{*16*}	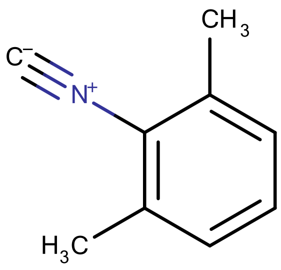

**3**{*17*}	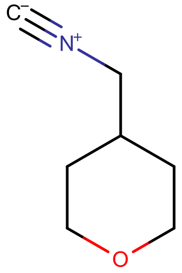
**3**{*18*}	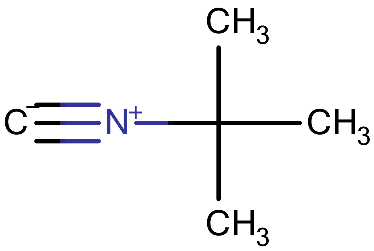
**3**{*19*}	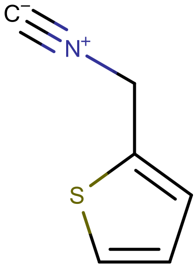
**3**{*20*}	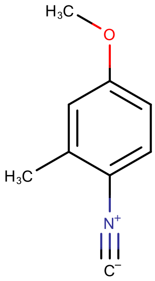

**3**{*21*}	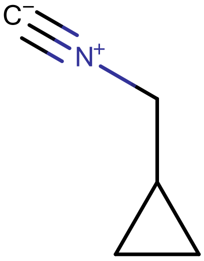
**3**{*22*}	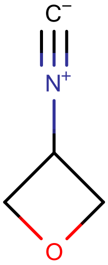
**3**{*23*}	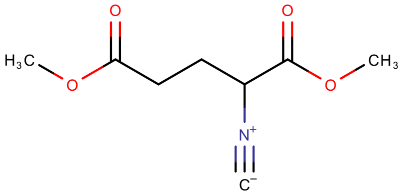
**3**{*24*}	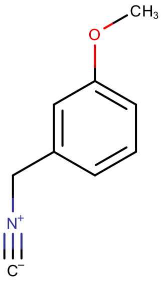

**3**{*25*}	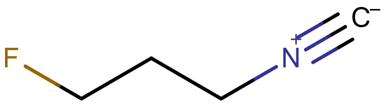
**3**{*26*}	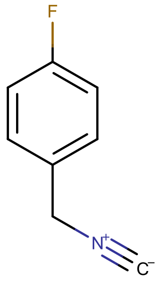
**3**{*27*}	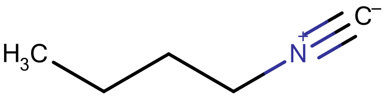
**3**{*28*}	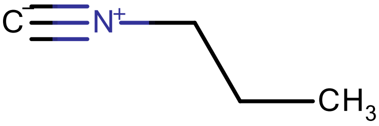

**3**{*29*}	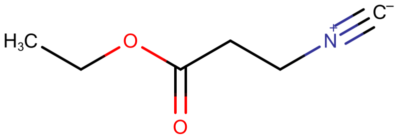
**3**{*30*}	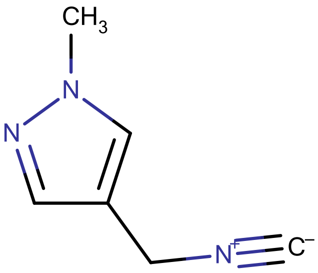
**3**{*31*}	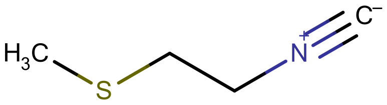
**3**{*32*}	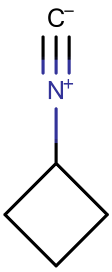

**3**{*33*}	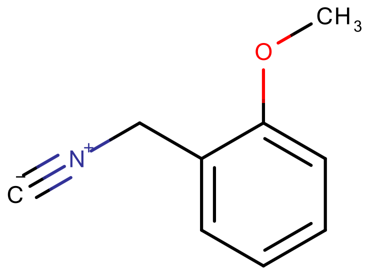
**3**{*34*}	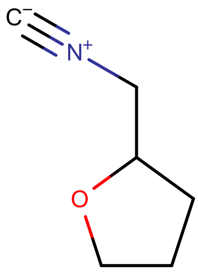
**3**{*35*}	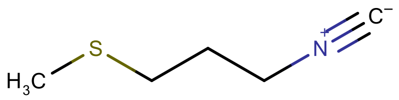
**3**{*36*}	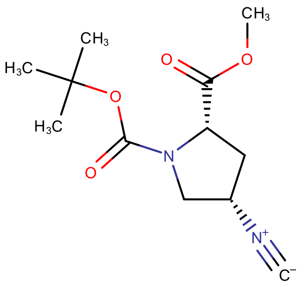

**3**{*37*}	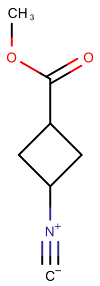
**3**{*38*}	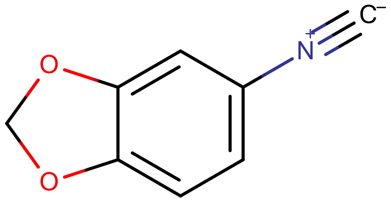
**3**{*39*}	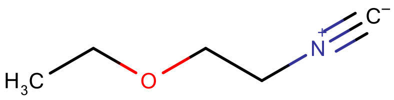
**3**{*40*}	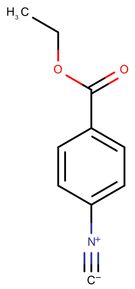

**3**{*41*}	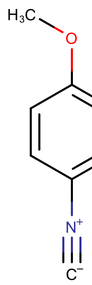
**3**{*42*}	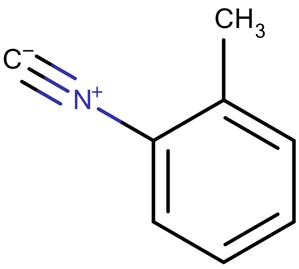
**3**{*43*}	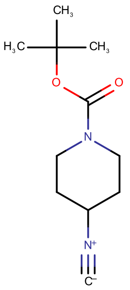
**3**{*44*}	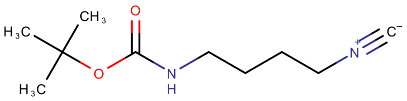
**3**{*45*}	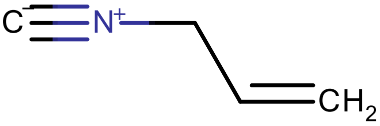
**3**{*46*}	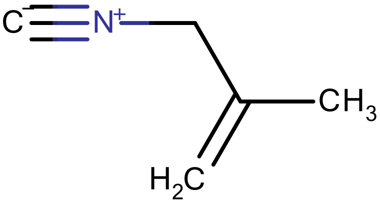
**3**{*47*}	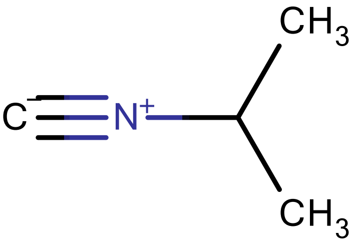
**3**{*48*}	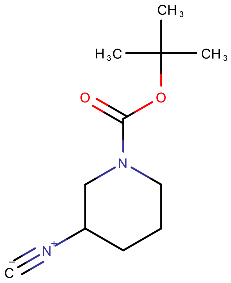

**3**{*49*}	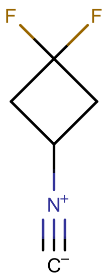	**3**{*53*}	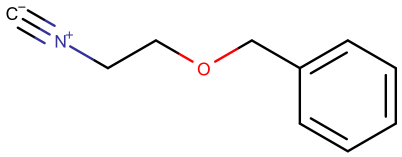
**3**{*50*}	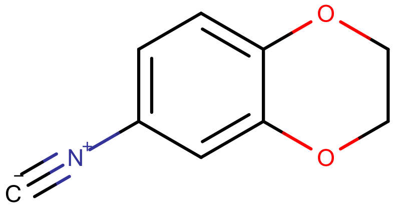	**3**{*54*}	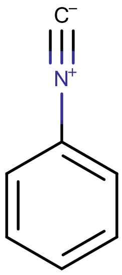
**3**{*51*}	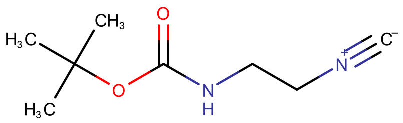	**3**{*55*}	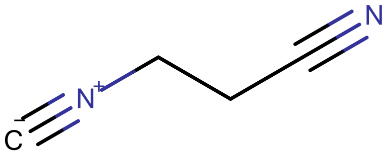
**3**{*52*}	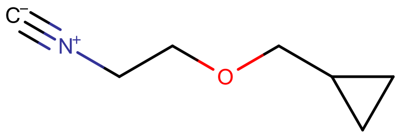	**3**{*56*}	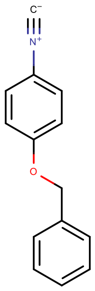

**3**{*57*}	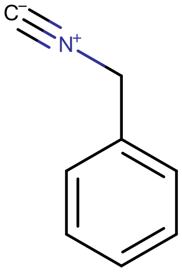
**3**{*58*}	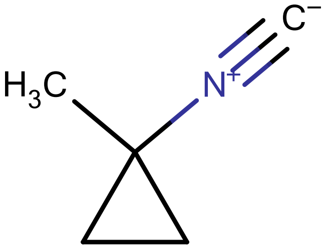
**3**{*59*}	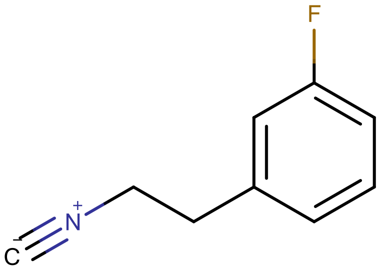
**3**{*60*}	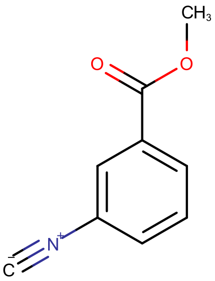

**3**{*61*}	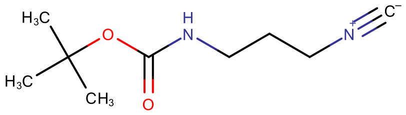
**3**{*62*}	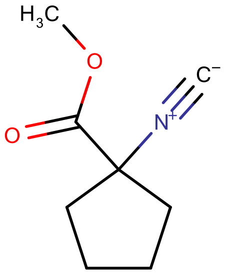
**3**{*63*}	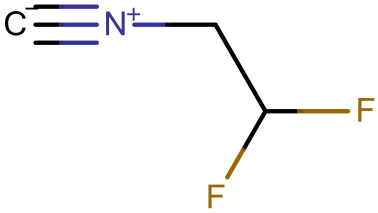
**3**{*64*}	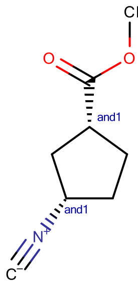

**3**{*65*}	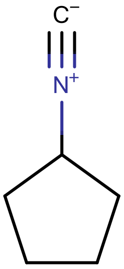
**3**{*66*}	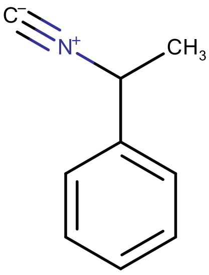
**3**{*67*}	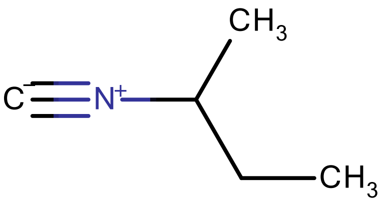
**3**{*68*}	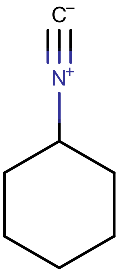

**3**{*69*}	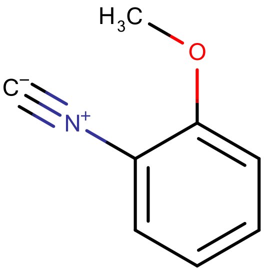
**3**{*70*}	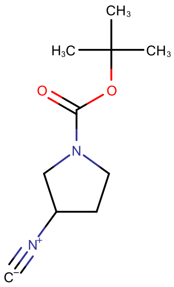
**3**{*71*}	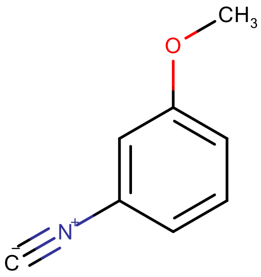
**3**{*72*}	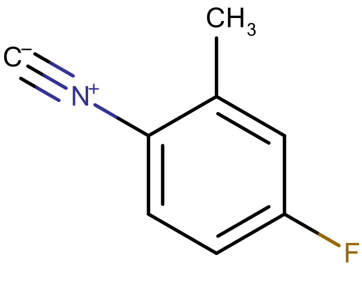

**3**{*73*}	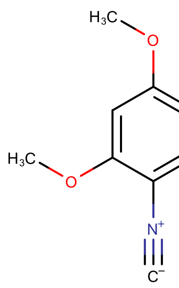
**3**{*74*}	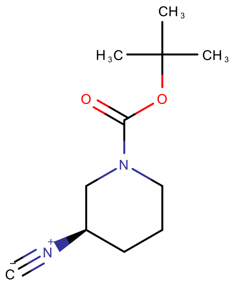
**3**{*75*}	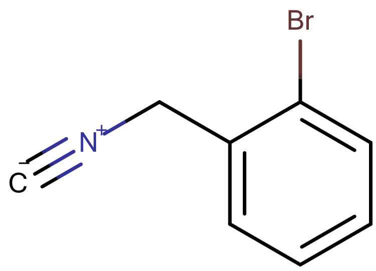
**3**{*76*}	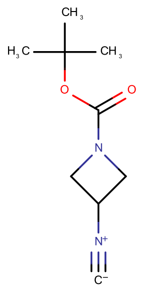

**3**{*77*}	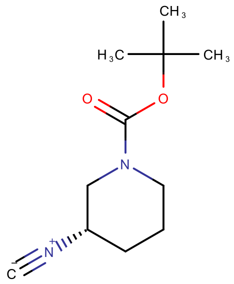
**3**{*78*}	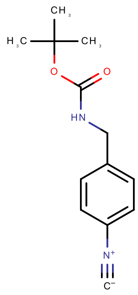
