# Supplementary material for: Generation of multimillion chemical space based on the parallel Groebke–Blackburn–Bienaymé reaction
Source: Beilstein J Org Chem. 2024 Jul 16;20:1604–13. doi: 10.3762/bjoc.20.143 (PMC11285076; doi:10.3762/bjoc.20.143)
Supplement: File 2 — Parallel synthesis of compound library 4. [file Beilstein_J_Org_Chem-20-1604-s002.pdf]

BEILSTEIN JOURNAL OF ORGANIC CHEMISTRY

Supporting Information

for

Generation of multimillion chemical space based on the parallel Groebke–Blackburn–Bienaymé reaction

Evgen V. Govor, Vasyl Naumchyk, Ihor Nestorak, Dmytro S. Radchenko, Dmytro Dudenko, Yurii S. Moroz, Olexiy D. Kachkovsky and Oleksandr O. Grygorenko

Beilstein J. Org. Chem. **2024,** *20,* 1604–1613. doi:10.3762/bjoc.20.143

Parallel synthesis of compound library 4

License and Terms: This is a supporting information file under the terms of the Creative Commons Attribution License (https://creativecommons.org/licenses/by/4.0). Please note that the reuse, redistribution and reproduction in particular requires that the author(s) and source are credited and that individual graphics may be subject to special legal provisions.
The license is subject to the *Beilstein Journal of Organic Chemistry* terms and conditions: (https://www.beilstein-journals.org/bjoc/terms)

<table>
<tr><th rowspan="2">#</th><th rowspan="2">ID</th><th rowspan="2">Structure</th><th rowspan="2">Internal ID</th><th colspan="2">Amine 1</th><th colspan="2">Aldehyde 2</th><th colspan="2">Isonitrile 3</th><th colspan="2">Yield</th></tr>
<tr><th>ID</th><th>SMILES</th><th>ID</th><th>SMILES</th><th>ID</th><th>SMILES</th><th>mg</th><th>%</th></tr>
<tr><td colspan="12">Preliminary test experiments, TsOH as the catalyst</td></tr>
<tr><td>1</td><td>4{28,7,4}</td><td>CN1N=NC(CNC2=C(N=C3C=C(C=CN23)C2=NOC=N2)C2=CC(CO)=CC(C)=N2)=N1 |c:2,11,13,21,28,31,33,t:7,9,18,24|</td><td>Z8786958949</td><td>1{28}</td><td>Cl.Nc1cc(ccn1)c2ncon2</td><td>2{7}</td><td>Cc1cc(CO)cc(C=O)n1</td><td>3{4}</td><td>Cn1nnc(C[N+]#[C-])n1</td><td>94.4</td><td>84</td></tr>
<tr><td>2</td><td>4{29,8,5}</td><td>COCCCNC1=C(N=C2C=CC(OC3CN(C3)C(=O)OC(C)(C)C)=CN12)C1=CON=C1COC |c:10,25,33,t:6,8,30|</td><td>Z8801681836</td><td>1{29}</td><td>CC(C)(C)OC(=O)N1CC(C1)Oc2ccc(N)nc2</td><td>2{8}</td><td>COCc1noccc1C=O</td><td>3{5}</td><td>COCCC[N+]#[C-]</td><td>94.8</td><td>72</td></tr>
<tr><td>3</td><td>4{30,9,6}</td><td>CCOC(=O)CCCNC1=C(N=C2C=CC=C(N12)C1=CC=C(C)C=C1)C1=CSC=C1 |c:13,15,25,31,t:9,11,20,22,28|</td><td>Z8784060473</td><td>1{30}</td><td>Cc1ccc(cc1)c2cccc(N)n2</td><td>2{9}</td><td>O=Cc1ccsc1</td><td>3{6}</td><td>CCOC(=O)CCC[N+]#[C-]</td><td>79.9</td><td>71</td></tr>
<tr><td>4</td><td>4{31,10,6}</td><td>CCOC(=O)CCCNC1=C(N=C2C=CC3=C(CCO3)N12)C1=NC=CC(C)=C1 |c:13,26,29,t:9,11,15,24|</td><td>Z8778277510</td><td>1{31}</td><td>Nc1ccc2OCCc2n1</td><td>2{10}</td><td>Cc1ccnc(C=O)c1</td><td>3{6}</td><td>CCOC(=O)CCC[N+]#[C-]</td><td>72.0</td><td>70</td></tr>
<tr><td>5</td><td>4{32,11,7}</td><td>CP(C)(=O)C1=CC2=NC(=C(NC3CCOCC3)N2C=C1)C1=NN(CC(F)(F)F)C=C1 |c:20,31,t:4,6,8,23|</td><td>Z8803896935</td><td>1{32}</td><td>CP(=O)(C)c1ccnc(N)c1</td><td>2{11}</td><td>FC(F)(F)Cn1ccc(C=O)n1</td><td>3{7}</td><td>[C-]#[N+]C1CCOCC1</td><td>82.4</td><td>69</td></tr>
<tr><td>6</td><td>4{33,9,6}</td><td>CCCSC1=CC=CC2=NC(=C(NCCCC(=O)OCC)N12)C1=CSC=C1 |c:6,27,t:4,8,10,24|</td><td>Z8797783068</td><td>1{33}</td><td>CCCSc1cccc(N)n1</td><td>2{9}</td><td>O=Cc1ccsc1</td><td>3{6}</td><td>CCOC(=O)CCC[N+]#[C-]</td><td>72.0</td><td>66</td></tr>
<tr><td>7</td><td>4{32,12,7}</td><td>COC1=NC=C(C=C1)C1=C(NC2CCOCC2)N2C=CC(=CC2=N1)P(C)(C)=O |c:4,6,9,20,22,25,t:2|</td><td>Z8798985026</td><td>1{32}</td><td>CP(=O)(C)c1ccnc(N)c1</td><td>2{12}</td><td>COc1ccc(C=O)cn1</td><td>3{7}</td><td>[C-]#[N+]C1CCOCC1</td><td>70.9</td><td>66</td></tr>
<tr><td>8</td><td>4{34,13,8}</td><td>COC(=O)C(CC1=CC=CC=C1)NC1=C(N=C2C=C(C=CN12)C1=NNC(C)=N1)C1=C(C)NN=N1 |c:8,10,18,20,29,32,36,t:6,14,16,25|</td><td>Z8801681814</td><td>1{34}</td><td>Cc1nc(n[nH]1)c2ccnc(N)c2</td><td>2{13}</td><td>Cc1[nH]nnc1C=O</td><td>3{8}</td><td>COC(=O)C(Cc1ccccc1)[N+]#[C-]</td><td>80.1</td><td>65</td></tr>
<tr><td>9</td><td>4{35,14,6}</td><td>CCOC(=O)CCCNC1=C(N=C2C=CC(C)=CN12)C1=CC=C(C)C=C1 |c:13,16,26,t:9,11,21,23|</td><td>Z8712546411</td><td>1{35}</td><td>Cc1ccc(N)nc1</td><td>2{14}</td><td>Cc1ccc(C=O)cc1</td><td>3{6}</td><td>CCOC(=O)CCC[N+]#[C-]</td><td>61.2</td><td>64</td></tr>
<tr><td>10</td><td>4{36,15,9}</td><td>CNC1=C(N=C2C=CC(Br)=CN12)C1=CC=C(OC)C=C1 |c:6,9,20,t:2,4,14,16|</td><td>Z8776690344</td><td>1{36}</td><td>Nc1ccc(Br)cn1</td><td>2{15}</td><td>COc1ccc(C=O)cc1</td><td>3{9}</td><td>C[N+]#[C-]</td><td>57.7</td><td>64</td></tr>
</table>

11	**4**{*37,16,10*}	CN1N=CC(CNC(=O)OC(C)(C)C)=C1C1=C(NC2CCOC2)N2C(C=CC=C2C(F)F)=N1 |c:2,13,16,27,29,34|	Z8810903040	**1**{*37*}	Cl.Nc1cccc(n1)C(F)F	**2**{*16*}	Cn1ncc(CNC(=O)OC(C)(C)C)c1C=O	**3**{*10*}	[C-]#[N+]C1CCOC1	78.9	63
12	**4**{*38,17,9*}	CCOC1=CN=C(N=C1)C1=C(NC)N2C=CC(=CC2=N1)C#N |c:5,7,10,15,17,20,t:3|	Z8810902991	**1**{*38*}	Nc1cc(C#N)ccn1	**2**{*17*}	CCOc1cnc(C=O)nc1	**3**{*9*}	C[N+]#[C-]	50.0	63
13	**4**{*39,10,6*}	CCOC(=O)CCCNC1=C(N=C2N1C=C(Br)C1=C2CCC1)C1=NC=CC(C)=C1 |c:11,18,27,30,t:9,15,25|	Z8784060925	**1**{*39*}	Nc1ncc(Br)c2CCCc21	**2**{*10*}	Cc1ccnc(C=O)c1	**3**{*6*}	CCOC(=O)CCC[N+]#[C-]	75.9	61
14	**4**{*40,18,4*}	CN1N=NC(CNC2=C(N=C3C=C(C=C(C)N23)C(F)(F)F)C2=CN(CCO)N=C2)=N1 |c:2,11,29,31,t:7,9,13,23|	Z8781341796	**1**{*40*}	Cc1cc(cc(N)n1)C(F)(F)F	**2**{*18*}	OCCn1cc(C=O)cn1	**3**{*4*}	Cn1nnc(C[N+]#[C-])n1	69.7	61
15	**4**{*41,19,11*}	COC1=C(N(C)N=C1)C1=C(NCC2=CC(Br)=CC=C2)N2C=C(C)N=CC2=N1 |c:6,9,16,18,25,28,t:2,13,22|	Z8781341780	**1**{*41*}	Cc1cnc(N)cn1	**2**{*19*}	COc1cnn(C)c1C=O	**3**{*11*}	Brc1cccc(C[N+]#[C-])c1	70.3	61
16	**4**{*42,20,7*}	CCN1C=C(N=N1)C1=C(NC2CCOCC2)N2C=C(SC)C=CC2=N1 |c:3,5,8,23,26,t:19|	Z8810902980	**1**{*42*}	CSc1ccc(N)nc1	**2**{*20*}	CCn1cc(C=O)nn1	**3**{*7*}	[C-]#[N+]C1CCOCC1	57.5	59
17	**4**{*43,21,12*}	CC(C)(C)OC(=O)N1CC2=C(C1)N1C(NCC3CCOC3)=C(N=C1C=C2)C1=CC2=NC=NN2C=C1 |c:9,22,24,27,34,38,t:30,32|	Z8798985027	**1**{*43*}	CC(C)(C)OC(=O)N1Cc2ccc(N)nc2C1	**2**{*21*}	O=Cc1ccn2ncnc2c1	**3**{*12*}	[C-]#[N+]CC1CCOC1	74.6	58
18	**4**{*44,22,6*}	CCOC(=O)CCCNC1=C(N=C2C=NC(CC)=CN12)C1=CC=CC=C1 |c:13,17,24,26,t:9,11,22|	Z8741907003	**1**{*44*}	CCc1cnc(N)cn1	**2**{*22*}	O=Cc1ccccc1	**3**{*6*}	CCOC(=O)CCC[N+]#[C-]	54.9	58
19	**4**{*45,23,10*}	FC1=CC2=NC(=C(NC3CCOC3)N2C=C1)C1=C2CCCCN2C(=N1)C(F)(F)F |c:16,19,27,t:1,3,5|	Z8803896946	**1**{*45*}	Cl.Nc1cc(F)ccn1	**2**{*23*}	FC(F)(F)c1nc(C=O)c2CCCCn12	**3**{*10*}	[C-]#[N+]C1CCOC1	62.9	57
20	**4**{*46,18,13*}	COC1=CC=C(CNC2=C(N=C3C=CC=CN23)C2=CN(CCO)N=C2)C=C1 |c:12,14,25,28,t:2,4,8,10,19|	Z8810903006	**1**{*46*}	Nc1ccccn1	**2**{*18*}	OCCn1cc(C=O)cn1	**3**{*13*}	COc1ccc(C[N+]#[C-])cc1	55.8	57
21	**4**{*47,24,14*}	COCCNC1=C(N=C2C=C(C=CN12)S(N)(=O)=O)C1=CC(=CC=C1)N(C)C |c:9,11,22,24,t:5,7,20|	Z8798985048	**1**{*47*}	Cl.Nc1cc(ccn1)S(=O)(=O)N	**2**{*24*}	CN(C)c1cccc(C=O)c1	**3**{*14*}	COCC[N+]#[C-]	59.6	57
22	**4**{*48,25,15*}	CCNC1=C(N=C2C=C(C=CN12)S(C)(=O)=O)C1=CN=C(N=C1)C1CCOCC1 |c:7,9,20,22,t:3,5,18|	Z8781341798	**1**{*48*}	CS(=O)(=O)c1ccnc(N)c1	**2**{*25*}	O=Cc1cnc(nc1)C2CCOCC2	**3**{*15*}	CC[N+]#[C-]	61.4	57

23	**4**{*49*,*26*,*7*}	OCC1=CC=CC2=NC(CCC3=CC=CC=C3)=C(NC3CCOCC3)N12 |c:4,13,15,t:2,6,11,17|	Z8741906954	**1**{*49*}	Nc1cccc(CO)n1	**2**{*26*}	O=CCCc1ccccc1	**3**{*7*}	[C-]#[N+]C1CCOCC1	53.8	57
24	**4**{*50*,*27*,*16*}	CN1N=C(N=C1C)C1=C(NC2=C(C)C=CC=C2C)N2C(C=CC=C2P(C)(C)=O)=N1 |c:2,4,8,11,14,16,22,24,30|	Z8781341789	**1**{*50*}	CP(=O)(C)c1cccc(N)n1	**2**{*27*}	Cc1nc(C=O)nn1C	**3**{*16*}	Cc1cccc(C)c1[N+]#[C-]	62.5	57
25	**4**{*51*,*22*,*17*}	CC1=C(Cl)C=CC2=NC(=C(NCC3CCOCC3)N12)C1=CC=CC=C1 |c:1,4,24,26,t:6,8,22|	Z8741907089	**1**{*51*}	Cc1nc(N)ccc1Cl	**2**{*22*}	O=Cc1ccccc1	**3**{*17*}	[C-]#[N+]CC1CCOCC1	54.4	57
26	**4**{*52*,*28*,*18*}	CC(C)(C)NC1=C(N=C2C=CC(=CN12)C1(CC1)C(F)(F)F)C1=CN(N=C1)C1CCS(=O)(=O)C1 |c:9,11,27,t:5,7,24|	Z8801681827	**1**{*52*}	Nc1ccc(cn1)C2(CC2)C(F)(F)F	**2**{*28*}	O=Cc1cnn(c1)C2CCS(=O)(=O)C2	**3**{*18*}	CC(C)(C)[N+]#[C-]	73.6	57
27	**4**{*53*,*14*,*17*}	CC1=CC=C(C=C1)C1=C(NCC2CCOCC2)N2C=C(CO)C=CC2=N1 |c:3,5,8,24,27,t:1,20|	Z8776690320	**1**{*53*}	Nc1ccc(CO)cn1	**2**{*14*}	Cc1ccc(C=O)cc1	**3**{*17*}	[C-]#[N+]CC1CCOCC1	53.7	57
28	**4**{*36*,*22*,*17*}	BrC1=CN2C(C=C1)=NC(=C2NCC1CCOCC1)C1=CC=CC=C1 |c:5,7,9,23,25,t:1,21|	Z8700285600	**1**{*36*}	Nc1ccc(Br)cn1	**2**{*22*}	O=Cc1ccccc1	**3**{*17*}	[C-]#[N+]CC1CCOCC1	59.0	57
29	**4**{*54*,*14*,*6*}	CCOC(=O)CCCNC1=C(N=C2C=CC(=CN12)C(=O)N(C)C)C1=CC=C(C)C=C1 |c:13,15,30,t:9,11,25,27|	Z8776690341	**1**{*54*}	Cl.CN(C)C(=O)c1ccc(N)nc1	**2**{*14*}	Cc1ccc(C=O)cc1	**3**{*6*}	CCOC(=O)CCC[N+]#[C-]	62.3	56
30	**4**{*55*,*29*,*6*}	CCOC(=O)CCCNC1=C(N=C2C=C(SC)C=CN12)C1=NN(CC)C=C1 |c:17,27,t:9,11,13,22|	Z8797781157	**1**{*55*}	CSc1ccnc(N)c1	**2**{*29*}	CCn1ccc(C=O)n1	**3**{*6*}	CCOC(=O)CCC[N+]#[C-]	58.7	56
31	**4**{*56*,*9*,*14*}	COCCNC1=C(N=C2C=CC(=CN12)C1=NN(C)C=C1)C1=CSC=C1 |c:9,11,20,26,t:5,7,16,23|	Z8780122427	**1**{*56*}	Cn1ccc(n1)c2ccc(N)nc2	**2**{*9*}	O=Cc1ccsc1	**3**{*14*}	COCC[N+]#[C-]	53.2	56
32	**4**{*57*,*30*,*19*}	CC(C)OC1=CN2C(C=N1)=NC(C1CCN(CC1)C(C)=O)=C2NCC1=CC=CS1 |c:8,10,22,29,t:4,27|	Z8786958905	**1**{*57*}	CC(C)Oc1cnc(N)cn1	**2**{*30*}	CC(=O)N1CCC(CC1)C=O	**3**{*19*}	[C-]#[N+]Cc1cccs1	62.2	56
33	**4**{*58*,*26*,*7*}	C(CC1=CC=CC=C1)C1=C(NC2CCOCC2)N2C=CC(OC3COC3)=CC2=N1 |c:4,6,9,20,28,31,t:2|	Z8776690336	**1**{*58*}	Nc1cc(OC2COC2)ccn1	**2**{*26*}	O=CCCc1ccccc1	**3**{*7*}	[C-]#[N+]C1CCOCC1	59.1	56
34	**4**{*59*,*14*,*17*}	CC1=CC=C(C=C1)C1=C(NCC2CCOCC2)N2C=C(CN3CCOCC3)C=CC2=N1 |c:3,5,8,30,33,t:1,20|	Z8776690335	**1**{*59*}	Nc1ccc(CN2CCOCC2)cn1	**2**{*14*}	Cc1ccc(C=O)cc1	**3**{*17*}	[C-]#[N+]CC1CCOCC1	62.8	55
35	**4**{*60*,*29*,*14*}	CCN1C=CC(=N1)C1=C(NCCOC)N2C=C(OC)C=C(F)C2=N1 |c:3,5,8,24,t:16,20|	Z8780122035	**1**{*60*}	COc1cnc(N)c(F)c1	**2**{*29*}	CCn1ccc(C=O)n1	**3**{*14*}	COCC[N+]#[C-]	49.7	55

36	**4**{*38*,*15*,*14*}	COCCNC1=C(N=C2C=C(C=CN12)C#N)C1=CC=C(OC)C=C1 |c:9,11,24,t:5,7,18,20|	Z8711876682	**1**{*38*}	Nc1cc(C#N)ccn1	**2**{*15*}	COc1ccc(C=O)cc1	**3**{*14*}	COCC[N+]#[C-]	48.1	55
37	**4**{*61*,*26*,*7*}	CCC1=CC=CC2=NC(CCC3=CC=CC=C3)=C(NC3CCOCC3)N12 |c:4,13,15,t:2,6,11,17|	Z8776690325	**1**{*61*}	CCc1cccc(N)n1	**2**{*26*}	O=CCCc1ccccc1	**3**{*7*}	[C-]#[N+]C1CCOCC1	52.1	55
38	**4**{*62*,*31*,*20*}	COC1=CC(C)=C(NC2=C(N=C3C=C(CO)C=CN23)C2=CN=CN2)C=C1 |c:16,23,27,t:2,5,8,10,12,21|	Z8810902966	**1**{*62*}	Nc1cc(CO)ccn1	**2**{*31*}	O=Cc1cnc[nH]1	**3**{*20*}	COc1ccc([N+]#[C-])c(C)c1	52.1	55
39	**4**{*63*,*32*,*10*}	COC1=C(C)C=CN2C(NC3CCOC3)=C(N=C12)[C@@H]1C[C@H]1C1=CC=NN1C |&1:18,20,r,c:2,5,15,27,t:17,25|	Z8810903003	**1**{*63*}	COc1c(C)ccnc1N	**2**{*32*}	Cn1nccc1[C@@H]2C[C@H]2C=O |&1:6,8,r|	**3**{*10*}	[C-]#[N+]C1CCOC1	54.6	55
40	**4**{*62*,*15*,*13*}	COC1=CC=C(CNC2=C(N=C3C=C(CO)C=CN23)C2=CC=C(OC)C=C2)C=C1 |c:16,27,30,t:2,4,8,10,12,21,23|	Z8776690332	**1**{*62*}	Nc1cc(CO)ccn1	**2**{*15*}	COc1ccc(C=O)cc1	**3**{*13*}	COc1ccc(C[N+]#[C-])cc1	57.9	55
41	**4**{*37*,*33*,*10*}	CC(=O)N1CCCC(C1)C1=C(NC2CCOC2)N2C(C=CC=C2C(F)F)=N1 |c:10,21,23,28|	Z8810903046	**1**{*37*}	Cl.Nc1cccc(n1)C(F)F	**2**{*33*}	CC(=O)N1CCCC(C1)C=O	**3**{*10*}	[C-]#[N+]C1CCOC1	56.2	55
42	**4**{*64*,*14*,*17*}	CC1=CC=C(C=C1)C1=C(NCC2CCOCC2)N2C=C(C=CC2=N1)N1CCCS1(=O)=O |c:3,5,8,20,22,25,t:1|	Z8776690346	**1**{*64*}	Nc1ccc(cn1)N2CCCS2(=O)=O	**2**{*14*}	Cc1ccc(C=O)cc1	**3**{*17*}	[C-]#[N+]CC1CCOCC1	65.3	55
43	**4**{*53*,*34*,*21*}	CP(C)(=O)C1=CC=C(C=C1)C1=C(NCC2CC2)N2C=C(CO)C=CC2=N1 |c:6,8,11,24,27,t:4,20|	Z8810902970	**1**{*53*}	Nc1ccc(CO)cn1	**2**{*34*}	CP(=O)(C)c1ccc(C=O)cc1	**3**{*21*}	[C-]#[N+]CC1CC1	54.7	55
44	**4**{*65*,*35*,*5*}	CCC1=CN=CC2=NC(=C(NCCCOC)N12)C1=NN(C)C(C)=C1Cl |c:4,24,t:2,6,8,19|	Z8801681825	**1**{*65*}	CCc1cncc(N)n1	**2**{*35*}	Cc1c(Cl)c(C=O)nn1C	**3**{*5*}	COCCC[N+]#[C-]	53.7	55
45	**4**{*66*,*36*,*22*}	COCCC1=CC2=NC(=C(NC3COC3)N2C=C1)C1=NC(C)=C(OC(F)F)C=C1 |c:18,30,t:4,6,8,21,24|	Z8810902992	**1**{*66*}	COCCc1ccnc(N)c1	**2**{*36*}	Cc1nc(C=O)ccc1OC(F)F	**3**{*22*}	[C-]#[N+]C1COC1	59.8	55
46	**4**{*67*,*15*,*9*}	CNC1=C(N=C2C=CC=C(C)N12)C1=CC=C(OC)C=C1 |c:6,20,t:2,4,8,14,16|	Z8776690314	**1**{*67*}	Cc1cccc(N)n1	**2**{*15*}	COc1ccc(C=O)cc1	**3**{*9*}	C[N+]#[C-]	39.5	55
47	**4**{*68*,*37*,*23*}	COC(=O)CCC(NC1=C(N=C2C=C(C=CN12)C#C)C1=NC2=C(CCC2)C=C1)C(=O)OC |c:12,14,29,t:8,10,21,23|	Z8803896955	**1**{*68*}	Nc1cc(C#C)ccn1	**2**{*37*}	O=Cc1ccc2CCCc2n1	**3**{*23*}	COC(=O)CCC([N+]#[C-])C(=O)OC	63.6	54
48	**4**{*69*,*33*,*24*}	CNC(=O)C1=CC2=NC(C3CCCN(C3)C(C)=O)=C(NCC3=CC(OC)=CC=C3)N2C=C1 |c:26,28,33,t:4,6,18,22|	Z8810903000	**1**{*69*}	CNC(=O)c1ccnc(N)c1	**2**{*33*}	CC(=O)N1CCCC(C1)C=O	**3**{*24*}	COc1cccc(C[N+]#[C-])c1	64.0	54

49	**4**{*70*,*38*,*25*}	FCCCNC1=C(N=C2N1C=CN=C2C1CC1)C1=NC(=CC=C1)C#C |c:7,11,13,22,24,t:5,20|	Z8781341774	**1**{*70*}	Nc1nccnc1C2CC2	**2**{*38*}	O=Cc1cccc(C#C)n1	**3**{*25*}	FCCC[N+]#[C-]	49.2	54
50	**4**{*71*,*14*,*17*}	CC1=CC=C(C=C1)C1=C(NCC2CCOCC2)N2C=C(I)N=CC2=N1 |c:3,5,8,23,26,t:1,20|	Z8776690316	**1**{*71*}	Nc1cnc(I)cn1	**2**{*14*}	Cc1ccc(C=O)cc1	**3**{*17*}	[C-]#[N+]CC1CCOCC1	65.4	54
51	**4**{*36*,*7*,*9*}	CNC1=C(N=C2C=CC(Br)=CN12)C1=NC(C)=CC(CO)=C1 |c:6,9,17,21,t:2,4,14|	Z8798985029	**1**{*36*}	Nc1ccc(Br)cn1	**2**{*7*}	Cc1cc(CO)cc(C=O)n1	**3**{*9*}	C[N+]#[C-]	50.7	54
52	**4**{*72*,*39*,*12*}	COC1=NC(Br)=CN2C(NCC3CCOC3)=C(N=C12)C1=COC=C1 |c:5,16,25,t:2,18,22|	Z8798985022	**1**{*72*}	COc1nc(Br)cnc1N	**2**{*39*}	O=Cc1ccoc1	**3**{*12*}	[C-]#[N+]CC1CCOC1	57.3	54
53	**4**{*73*,*15*,*13*}	COC1=CC=C(CNC2=C(N=C3C=CC4=NC=CN4N23)C2=CC=C(OC)C=C2)C=C1 |c:12,16,29,32,t:2,4,8,10,14,23,25|	Z8741907043	**1**{*73*}	Nc1ccc2nccn2n1	**2**{*15*}	COc1ccc(C=O)cc1	**3**{*13*}	COc1ccc(C[N+]#[C-])cc1	58.2	54
54	**4**{*74*,*40*,*17*}	OCC1=CSC(=N1)C1=C(NCC2CCOCC2)N2C(C=CC=C2OC(F)F)=N1 |c:5,8,21,23,29,t:2|	Z8797782835	**1**{*74*}	Nc1cccc(OC(F)F)n1	**2**{*40*}	OCc1csc(C=O)n1	**3**{*17*}	[C-]#[N+]CC1CCOCC1	59.8	54
55	**4**{*75*,*41*,*7*}	CC(C)(C)OC(=O)N1CCN(CC1)C1=CC=CN2C(NC3CCOCC3)=C(N=C12)C1=NC=CC=C1 |c:16,27,35,37,t:14,29,33|	Z8797783886	**1**{*75*}	CC(C)(C)OC(=O)N1CCN(CC1)c2cccnc2N	**2**{*41*}	O=Cc1ccccn1	**3**{*7*}	[C-]#[N+]C1CCOCC1	69.4	54
56	**4**{*76*,*42*,*23*}	COC(=O)CCC(NC1=C(N=C2C=CC(C)=C(Cl)N12)C1=CN(C)N=C1Cl)C(=O)OC |c:12,25,t:8,10,15,21|	Z8810903037	**1**{*76*}	Cc1ccc(N)nc1Cl	**2**{*42*}	Cn1cc(C=O)c(Cl)n1	**3**{*23*}	COC(=O)CCC([N+]#[C-])C(=O)OC	65.5	53
57	**4**{*35*,*22*,*20*}	COC1=CC(C)=C(NC2=C(N=C3C=CC(C)=CN23)C2=CC=CC=C2)C=C1 |c:12,15,22,24,27,t:2,5,8,10,20|	Z8712568084	**1**{*35*}	Cc1ccc(N)nc1	**2**{*22*}	O=Cc1ccccc1	**3**{*20*}	COc1ccc([N+]#[C-])c(C)c1	49.4	53
58	**4**{*77*,*41*,*26*}	FC1=CC=C(CNC2=C(N=C3C=CC(=CN23)C2=CN=CC=C2)C2=NC=CC=C2)C=C1 |c:11,13,20,22,27,29,32,t:1,3,7,9,18,25|	Z8778278038	**1**{*77*}	Nc1ccc(cn1)c2cccnc2	**2**{*41*}	O=Cc1ccccn1	**3**{*26*}	Fc1ccc(C[N+]#[C-])cc1	56.9	53
59	**4**{*42*,*9*,*27*}	CCCCNC1=C(N=C2C=CC(SC)=CN12)C1=CSC=C1 |c:9,13,21,t:5,7,18|	Z8784058856	**1**{*42*}	CSc1ccc(N)nc1	**2**{*9*}	O=Cc1ccsc1	**3**{*27*}	CCCC[N+]#[C-]	45.6	53
60	**4**{*78*,*43*,*28*}	CCCNC1=C(N=C2C=C(C)C3=NC(C)=CN3N12)C1=CN=C(C=C1)N1CCCCC1 |c:14,23,25,t:4,6,8,11,21|	Z8801681820	**1**{*78*}	Cc1cn2nc(N)cc(C)c2n1	**2**{*43*}	O=Cc1ccc(nc1)N2CCCCC2	**3**{*28*}	CCC[N+]#[C-]	57.9	53
61	**4**{*79*,*22*,*27*}	CCCCNC1=C(N=C2C=NC=C(C)N12)C1=CC=CC=C1 |c:9,19,21,t:5,7,11,17|	Z8711872692	**1**{*79*}	Cc1cncc(N)n1	**2**{*22*}	O=Cc1ccccc1	**3**{*27*}	CCCC[N+]#[C-]	40.0	53

62	**4**{*80*,*22*,*7*}	COC1=NC=CN2C(NC3CCOCC3)=C(N=C12)C1=CC=CC=C1 |c:4,15,23,25,t:2,17,21|	Z8778277285	**1**{*80*}	COc1nccnc1N	**2**{*22*}	O=Cc1ccccc1	**3**{*7*}	[C-]#[N+]C1CCOCC1	46.3	53
63	**4**{*81*,*14*,*27*}	CCCCNC1=C(N=C2C=NC=CN12)C1=CC=C(C)C=C1 |c:9,11,21,t:5,7,16,18|	Z8700285862	**1**{*81*}	Nc1cnccn1	**2**{*14*}	Cc1ccc(C=O)cc1	**3**{*27*}	CCCC[N+]#[C-]	40.0	53
64	**4**{*82*,*44*,*7*}	CCN1C=C(C=N1)C1=C(NC2CCOCC2)N2C(C=CC3=C2CCCC3)=N1 |c:3,5,8,20,22,29|	Z8778277692	**1**{*82*}	Nc1ccc2CCCCc2n1	**2**{*44*}	CCn1cc(C=O)cn1	**3**{*7*}	[C-]#[N+]C1CCOCC1	52.1	53
65	**4**{*83*,*45*,*18*}	COC1=C(F)C=CC(=C1)C1=C(NC(C)(C)C)N2C=C(C=CC2=N1)P(C)(C)=O |c:2,5,7,10,18,20,23|	Z8810903025	**1**{*83*}	CP(=O)(C)c1ccc(N)nc1	**2**{*45*}	COc1cc(C=O)ccc1F	**3**{*18*}	CC(C)(C)[N+]#[C-]	55.5	53
66	**4**{*84*,*46*,*7*}	COCCCCCC1=C(NC2CCOCC2)N2C=CN=C(C(C)C)C2=N1 |c:7,18,26,t:20|	Z8786958868	**1**{*84*}	CC(C)c1nccnc1N	**2**{*46*}	COCCCCCC=O	**3**{*7*}	[C-]#[N+]C1CCOCC1	51.3	53
67	**4**{*61*,*47*,*29*}	CCOC(=O)CCNC1=C(CCOCC=C)N=C2C=CC=C(CC)N12 |c:8,18,t:16,20|	Z8808559858	**1**{*61*}	CCc1cccc(N)n1	**2**{*47*}	C=CCOCCC=O	**3**{*29*}	CCOC(=O)CC[N+]#[C-]	49.1	53
68	**4**{*85*,*48*,*30*}	CN1C=C(CNC2=C(N=C3C=C(C=C(C)N23)C#N)C2=CNC(=N2)C2CCC2)C=N1 |c:10,23,31,t:2,6,8,12,20|	Z8781341795	**1**{*85*}	Cc1cc(C#N)cc(N)n1	**2**{*48*}	O=Cc1c[nH]c(n1)C2CCC2	**3**{*30*}	Cn1cc(C[N+]#[C-])cn1	54.7	52
69	**4**{*47*,*49*,*6*}	CCOC(=O)CCCNC1=C(N=C2C=C(C=CN12)S(N)(=O)=O)C1=CNN=C1C |c:13,15,27,t:9,11,24|	Z8801681821	**1**{*47*}	Cl.Nc1cc(ccn1)S(=O)(=O)N	**2**{*49*}	Cc1n[nH]cc1C=O	**3**{*6*}	CCOC(=O)CCC[N+]#[C-]	57.4	52
70	**4**{*86*,*50*,*31*}	CSCCNC1=C(N=C2C=C(C(F)F)C(Cl)=CN12)[C@@H]1C[C@H]1C1OCCO1 |&1:18,20,r,c:15,t:5,7,9|	Z8801681826	**1**{*86*}	Nc1cc(C(F)F)c(Cl)cn1	**2**{*50*}	O=C[C@@H]1C[C@H]1C2OCCO2 |&1:2,4,r|	**3**{*31*}	CSCC[N+]#[C-]	57.0	52
71	**4**{*87*,*26*,*7*}	CC1=CC2=NC(CCC3=CC=CC=C3)=C(NC3CCOCC3)N2C=C1Cl |c:10,12,26,t:1,3,8,14|	Z8776690323	**1**{*87*}	Cc1cc(N)ncc1Cl	**2**{*26*}	O=CCCc1ccccc1	**3**{*7*}	[C-]#[N+]C1CCOCC1	52.1	52
72	**4**{*88*,*29*,*7*}	CCN1C=CC(=N1)C1=C(NC2CCOCC2)N2C=CC(Br)=C(F)C2=N1 |c:3,5,8,19,26,t:22|	Z8797786849	**1**{*88*}	Nc1nccc(Br)c1F	**2**{*29*}	CCn1ccc(C=O)n1	**3**{*7*}	[C-]#[N+]C1CCOCC1	57.5	52
73	**4**{*89*,*51*,*1*}	CC1=C(C=NN1C1CCC1)C1=C(NC2CC2)N2C(C=CC3=C2NN=C3)=N1 |c:3,12,21,23,27,29,t:1|	Z8801681839	**1**{*89*}	Nc1ccc2cn[nH]c2n1	**2**{*51*}	Cc1c(C=O)cnn1C2CCC2	**3**{*1*}	[C-]#[N+]C1CC1	48.9	52

74	**4**{*90*,*52*,*15*}	CCNC1=C(N=C2C=C(C=CN12)N1C=CC=N1)C1=CN(N=C1)C1CCC1 |c:7,9,15,17,23,t:3,5,20|	Z8781341788	**1**{*90*}	Nc1cc(ccn1)n2cccn2	**2**{*52*}	O=Cc1cnn(c1)C2CCC2	**3**{*15*}	CC[N+]#[C-]	48.8	52
75	**4**{*91*,*53*,*18*}	CC1=NC(C2=C(NC(C)(C)C)N3C=C(C=CC3=N2)C(N)=O)=C(F)C=C1 |c:4,12,14,17,25,t:1,22|	Z8798985039	**1**{*91*}	NC(=O)c1ccc(N)nc1	**2**{*53*}	Cc1ccc(F)c(C=O)n1	**3**{*18*}	CC(C)(C)[N+]#[C-]	48.0	52
76	**4**{*92*,*54*,*32*}	COC1=C(C=CS1)C1=C(NC2CCC2)N2C=CC(=CC2=N1)C(N)=O |c:4,8,17,19,22,t:2|	Z8808559893	**1**{*92*}	NC(=O)c1ccnc(N)c1	**2**{*54*}	COc1sccc1C=O	**3**{*32*}	[C-]#[N+]C1CCC1	48.0	52
77	**4**{*92*,*41*,*17*}	NC(=O)C1=CC2=NC(=C(NCC3CCOCC3)N2C=C1)C1=NC=CC=C1 |c:20,25,27,t:3,5,7,23|	Z8784059425	**1**{*92*}	NC(=O)c1ccnc(N)c1	**2**{*41*}	O=Cc1ccccn1	**3**{*17*}	[C-]#[N+]CC1CCOCC1	49.1	52
78	**4**{*93*,*55*,*33*}	COC1=CN2C(C=N1)=NC(=C2NCC1=C(OC)C=CC=C1)C1=CC(=NC=C1)P(C)(C)=O |c:6,8,10,15,19,21,26,28,t:2,24|	Z8798985018	**1**{*93*}	COc1cnc(N)cn1	**2**{*55*}	CP(=O)(C)c1cc(C=O)ccn1	**3**{*33*}	COc1ccccc1C[N+]#[C-]	61.2	52
79	**4**{*94*,*14*,*17*}	CC1=CC=C(C=C1)C1=C(NCC2CCOCC2)N2N=C(F)C=CC2=N1 |c:3,5,8,23,26,t:1,20|	Z8776690309	**1**{*94*}	Nc1ccc(F)nn1	**2**{*14*}	Cc1ccc(C=O)cc1	**3**{*17*}	[C-]#[N+]CC1CCOCC1	47.4	52
80	**4**{*76*,*56*,*23*}	COC(=O)CCC(NC1=C(N=C2C=CC(C)=C(Cl)N12)C1=C(C)C=NC(F)=C1)C(=O)OC |c:12,21,24,27,t:8,10,15|	Z8810903005	**1**{*76*}	Cc1ccc(N)nc1Cl	**2**{*56*}	Cc1cnc(F)cc1C=O	**3**{*23*}	COC(=O)CCC([N+]#[C-])C(=O)OC	62.5	52
81	**4**{*95*,*46*,*25*}	COCCCCCC1=C(NCCCF)N2C=C(C=CC2=N1)C(F)F |c:7,15,17,20|	Z8781341784	**1**{*95*}	Nc1ccc(cn1)C(F)F	**2**{*46*}	COCCCCCC=O	**3**{*25*}	FCCC[N+]#[C-]	47.8	52
82	**4**{*96*,*14*,*17*}	COCCN(C)CC1=CC2=NC(=C(NCC3CCOCC3)N2C=C1)C1=CC=C(C)C=C1 |c:24,32,t:7,9,11,27,29|	Z8776690343	**1**{*96*}	COCCN(C)Cc1ccnc(N)c1	**2**{*14*}	Cc1ccc(C=O)cc1	**3**{*17*}	[C-]#[N+]CC1CCOCC1	58.6	51
83	**4**{*97*,*57*,*10*}	CC1=CC(F)=CN2C(NC3CCOC3)=C(N=C12)C1CCOC1 |c:4,14,t:1,16|	Z8798985034	**1**{*97*}	Cc1cc(F)cnc1N	**2**{*57*}	O=CC1CCOC1	**3**{*10*}	[C-]#[N+]C1CCOC1	42.2	51
84	**4**{*98*,*58*,*27*}	CCCCNC1=C(N=C2C=CC=C(OC(F)(F)F)N12)C1=NSN=C1 |c:9,24,t:5,7,11,21|	Z8797783281	**1**{*98*}	Nc1cccc(OC(F)(F)F)n1	**2**{*58*}	O=Cc1cnsn1	**3**{*27*}	CCCC[N+]#[C-]	49.3	51
85	**4**{*99*,*59*,*34*}	COC1=C(Br)C=C(C=N1)C1=C(NCC2CCCO2)N2C=C(C=CC2=N1)S(N)(=O)=O |c:2,5,7,10,21,23,26|	Z8808559882	**1**{*99*}	Nc1ccc(cn1)S(=O)(=O)N	**2**{*59*}	COc1ncc(C=O)cc1Br	**3**{*34*}	[C-]#[N+]CC1CCCO1	66.6	51
86	**4**{*100*,*29*,*14*}	CCN1C=CC(=N1)C1=C(NCCOC)N2C(C=NC3=C2C=CC=C3)=N1 |c:3,5,8,17,19,22,24,26|	Z8778277663	**1**{*100*}	Nc1cnc2ccccc2n1	**2**{*29*}	CCn1ccc(C=O)n1	**3**{*14*}	COCC[N+]#[C-]	46.3	51

87	**4**{*95,60,35*}	CSCCCNC1=C(N=C2C=CC(=CN12)C(F)F)C(C)C1CCOC1 |c:10,12,t:6,8|	Z8810903012	**1**{*95*}	Nc1ccc(cn1)C(F)F	**2**{*60*}	CC(C=O)C1CCOC1	**3**{*35*}	CSCCC[N+]#[C-]	50.5	51
88	**4**{*70,61,32*}	CS(=O)C1=CC=C(C=C1)C1=C(NC2CCC2)N2C=CN=C(C3CC3)C2=N1 |c:5,7,10,19,28,t:3,21|	Z8810903026	**1**{*70*}	Nc1nccnc1C2CC2	**2**{*61*}	CS(=O)c1ccc(C=O)cc1	**3**{*32*}	[C-]#[N+]C1CCC1	49.9	50
89	**4**{*81,14,17*}	CC1=CC=C(C=C1)C1=C(NCC2CCOCC2)N2C=CN=CC2=N1 |c:3,5,8,20,22,25,t:1|	Z8781341793	**1**{*81*}	Nc1cnccn1	**2**{*14*}	Cc1ccc(C=O)cc1	**3**{*17*}	[C-]#[N+]CC1CCOCC1	43.8	50
90	**4**{*92,31,36*}	COC(=O)[C@@H]1CC(CN1C(=O)OC(C)(C)C)NC1=C(N=C2C=C(C=CN12)C(N)=O)C1=CN=CN1 |c:22,24,34,t:18,20,32|	Z8798985053	**1**{*92*}	NC(=O)c1ccnc(N)c1	**2**{*31*}	O=Cc1cnc[nH]1	**3**{*36*}	COC(=O)[C@@H]1C[C@@H](CN1C(=O)OC(C)(C)C)[N+]#[C-]	63.7	50
91	**4**{*46,15,9*}	CNC1=C(N=C2C=CC=CN12)C1=CC=C(OC)C=C1 |c:6,8,19,t:2,4,13,15|	Z8776690321	**1**{*46*}	Nc1ccccn1	**2**{*15*}	COc1ccc(C=O)cc1	**3**{*9*}	C[N+]#[C-]	34.4	50
92	**4**{*101,62,14*}	COCCNC1=C(N=C2N1C=C(CO)C=C2Cl)C1CC11CCOCC1 |c:7,15,t:5,11|	Z8810902961	**1**{*101*}	Nc1ncc(CO)cc1Cl	**2**{*62*}	O=CC1CC21CCOCC2	**3**{*14*}	COCC[N+]#[C-]	49.6	50
93	**4**{*102,63,37*}	COCN1N=CC=C1C1=C(NC2CC(C2)C(=O)OC)N2C=CC(=CC2=N1)C(=O)N(C)C |c:4,6,9,22,24,27|	Z8781341782	**1**{*102*}	CN(C)C(=O)c1ccnc(N)c1	**2**{*63*}	COCn1nccc1C=O	**3**{*37*}	COC(=O)C1CC(C1)[N+]#[C-]	57.8	50
94	**4**{*46,22,14*}	COCCNC1=C(N=C2C=CC=CN12)C1=CC=CC=C1 |c:9,11,18,20,t:5,7,16|	Z8711876379	**1**{*46*}	Nc1ccccn1	**2**{*22*}	O=Cc1ccccc1	**3**{*14*}	COCC[N+]#[C-]	36.2	50
95	**4**{*50,64,26*}	CP(C)(=O)C1=CC=CC2=NC(=C(NCC3=CC=C(F)C=C3)N12)C1=CC=C(C=C1)N1C=CN=C1 |c:6,19,27,29,33,35,t:4,8,10,14,16,25|	Z8810902997	**1**{*50*}	CP(=O)(C)c1cccc(N)n1	**2**{*64*}	O=Cc1ccc(cc1)n2ccnc2	**3**{*26*}	Fc1ccc(C[N+]#[C-])cc1	62.0	50
96	**4**{*103,65,38*}	CC(C)(C)OC(=O)NC1CC2(C1)CC(C2)C1=C(NC2=CC3=C(OCO3)C=C2)N2C(C=NC=C2CO)=N1 |c:17,28,33,35,39,t:20,22|	Z8803896970	**1**{*103*}	Nc1cncc(CO)n1	**2**{*65*}	CC(C)(C)OC(=O)NC1CC2(C1)CC(C2)C=O	**3**{*38*}	[C-]#[N+]c1ccc2OCOc2c1	66.4	50
97	**4**{*104,66,39*}	CCOCCNC1=C(N=C2C=CC3=C(COCC3)N12)C1=C(C)N(CC)N=C1C |c:10,22,28,t:6,8,12|	Z8810903028	**1**{*104*}	Nc1ccc2CCOCc2n1	**2**{*66*}	CCn1nc(C)c(C=O)c1C	**3**{*39*}	CCOCC[N+]#[C-]	51.6	50
98	**4**{*103,67,40*}	CCOC(=O)C1=CC=C(NC2=C(N=C3C=NC=C(CO)N23)C2CCCSC2)C=C1 |c:14,30,t:5,7,10,12,16|	Z8798985062	**1**{*103*}	Nc1cncc(CO)n1	**2**{*67*}	O=CC1CCCSC1	**3**{*40*}	CCOC(=O)c1ccc([N+]#[C-])cc1	55.5	50

99	**4**{*105*,*68*,*41*}	COC1=CC=C(NC2=C(N=C3C=CC=C(N23)C(N)=O)C2=NC=C(S2)C(C)O)C=C1 |c:11,13,23,30,t:2,4,7,9,21|	Z8810902972	**1**{*105*}	NC(=O)c1cccc(N)n1	**2**{*68*}	CC(O)c1cnc(C=O)s1	**3**{*41*}	COc1ccc([N+]#[C-])cc1	54.5	49
100	**4**{*36*,*40*,*13*}	COC1=CC=C(CNC2=C(N=C3C=CC(Br)=CN23)C2=NC(CO)=CS2)C=C1 |c:12,15,24,28,t:2,4,8,10,20|	Z8808559864	**1**{*36*}	Nc1ccc(Br)cn1	**2**{*40*}	OCc1csc(C=O)n1	**3**{*13*}	COc1ccc(C[N+]#[C-])cc1	59.3	49
101	**4**{*47*,*69*,*6*}	CCOC(=O)CCCNC1=C(N=C2C=C(C=CN12)S(N)(=O)=O)C1=NN(C)C(C)=C1 |c:13,15,29,t:9,11,24|	Z8808559899	**1**{*47*}	Cl.Nc1cc(ccn1)S(=O)(=O)N	**2**{*69*}	Cc1cc(C=O)nn1C	**3**{*6*}	CCOC(=O)CCC[N+]#[C-]	55.9	49
102	**4**{*45*,*70*,*12*}	CC(F)(F)C1=NC(=CS1)C1=C(NCC2CCOC2)N2C=CC(F)=CC2=N1 |c:6,10,21,24,27,t:4|	Z8801681810	**1**{*45*}	Cl.Nc1cc(F)ccn1	**2**{*70*}	CC(F)(F)c1nc(C=O)cs1	**3**{*12*}	[C-]#[N+]CC1CCOC1	50.8	49
103	**4**{*106*,*15*,*13*}	COC1=CC=C(CNC2=C(N=C3C=CC(OC)=NN23)C2=CC=C(OC)C=C2)C=C1 |c:12,16,27,30,t:2,4,8,10,21,23|	Z8776690338	**1**{*106*}	COc1ccc(N)nn1	**2**{*15*}	COc1ccc(C=O)cc1	**3**{*13*}	COc1ccc(C[N+]#[C-])cc1	51.9	49
104	**4**{*59*,*71*,*42*}	CN1C=C(C=N1)[C@@H]1OCC[C@H]1C1=C(NC2=C(C)C=CC=C2)N2C=C(CN3CCOCC3)C=CC2=N1 |&1:6,10,r,c:2,4,13,16,19,21,35,38,t:25|	Z8810903017	**1**{*59*}	Nc1ccc(CN2CCOCC2)cn1	**2**{*71*}	Cn1cc(cn1)[C@@H]2OCC[C@H]2C=O |&1:6,10,r|	**3**{*42*}	Cc1ccccc1[N+]#[C-]	62.6	49
105	**4**{*104*,*72*,*39*}	CCOCCNC1=C(C[C@@H](C)NC(=O)OCC2=CC=CC=C2)N=C2C=CC3=C(COCC3)N12 |c:6,18,20,25,t:16,23,27|	Z8808559855	**1**{*104*}	Nc1ccc2CCOCc2n1	**2**{*72*}	C[C@H](CC=O)NC(=O)OCc1ccccc1	**3**{*39*}	CCOCC[N+]#[C-]	59.9	49
106	**4**{*107*,*16*,*10*}	CN1N=CC(CNC(=O)OC(C)(C)C)=C1C1=C(NC2CCOC2)N2C=C(OCCO)C=CC2=N1 |c:2,13,16,32,35,t:26|	Z8808559898	**1**{*107*}	Nc1ccc(OCCO)cn1	**2**{*16*}	Cn1ncc(CNC(=O)OC(C)(C)C)c1C=O	**3**{*10*}	[C-]#[N+]C1CCOC1	62.6	49
107	**4**{*108*,*73*,*12*}	CCCOC1=CN2C(NCC3CCOC3)=C(N=C2C=C1)C1=C(C)C=NN1C |c:15,17,20,23,26,t:4|	Z8810902969	**1**{*108*}	CCCOc1ccc(N)nc1	**2**{*73*}	Cc1cnn(C)c1C=O	**3**{*12*}	[C-]#[N+]CC1CCOC1	48.8	49
108	**4**{*109*,*74*,*41*}	COC1=CC=C(NC2=C(N=C3C=CC=C(N23)S(N)(=O)=O)C2=C(Cl)N(C)C=N2)C=C1 |c:11,13,22,27,30,t:2,4,7,9|	Z8810902958	**1**{*109*}	Cl.Nc1cccc(n1)S(=O)(=O)N	**2**{*74*}	Cn1cnc(C=O)c1Cl	**3**{*41*}	COc1ccc([N+]#[C-])cc1	57.1	49
109	**4**{*67*,*26*,*27*}	CCCCNC1=C(CCC2=CC=CC=C2)N=C2C=CC=C(C)N12 |c:5,11,13,18,t:9,16,20|	Z8711876474	**1**{*67*}	Cc1cccc(N)n1	**2**{*26*}	O=CCCc1ccccc1	**3**{*27*}	CCCC[N+]#[C-]	40.6	49

110	**4**{*110,44,7*}	CCN1C=C(C=N1)C1=C(NC2CCOCC2)N2C=C(C(C)=CC2=N1)S(N)(=O)=O |c:3,5,8,19,22,25|	Z8780122393	**1**{*110*}	Cc1cc(N)ncc1S(=O)(=O)N	**2**{*44*}	CCn1cc(C=O)cn1	**3**{*7*}	[C-]#[N+]C1CCOCC1	53.3	49
111	**4**{*22,26,16*}	CC1=CC=CC(C)=C1NC1=C(CCC2=CC=CC=C2)N=C2C=CC(Cl)=NN12 |c:3,6,10,16,18,23,26,t:1,14,21|	Z8741907012	**1**{*22*}	Nc1ccc(Cl)nn1	**2**{*26*}	O=CCCc1ccccc1	**3**{*16*}	Cc1cccc(C)c1[N+]#[C-]	49.6	49
112	**4**{*36,15,11*}	COC1=CC=C(C=C1)C1=C(NCC2=CC(Br)=CC=C2)N2C=C(Br)C=CC2=N1 |c:4,6,9,16,18,25,28,t:2,13,22|	Z8712552581	**1**{*36*}	Nc1ccc(Br)cn1	**2**{*15*}	COc1ccc(C=O)cc1	**3**{*11*}	Brc1cccc(C[N+]#[C-])c1	63.4	48
113	**4**{*53,75,33*}	COCC1=CC=C(O1)C1=C(NCC2=C(OC)C=CC=C2)N2C=C(CO)C=CC2=N1 |c:5,9,13,17,19,27,30,t:3,23|	Z8803896929	**1**{*53*}	Nc1ccc(CO)cn1	**2**{*75*}	COCc1ccc(C=O)o1	**3**{*33*}	COc1ccccc1C[N+]#[C-]	51.2	48
114	**4**{*50,76,26*}	CCC1=NN(C)C(=C1)C1=C(NCC2=CC=C(F)C=C2)N2C(C=CC=C2P(C)(C)=O)=N1 |c:6,9,18,23,25,31,t:2,13,15|	Z8803896928	**1**{*50*}	CP(=O)(C)c1cccc(N)n1	**2**{*76*}	CCc1cc(C=O)n(C)n1	**3**{*26*}	Fc1ccc(C[N+]#[C-])cc1	55.3	48
115	**4**{*111,18,43*}	COC(=O)C1=CN2C(NC3CCN(CC3)C(=O)OC(C)(C)C)=C(N=C2C(C)=C1)C1=CN(CCO)N=C1 |c:22,24,28,37,t:4,31|	Z8786958933	**1**{*111*}	COC(=O)c1cnc(N)c(C)c1	**2**{*18*}	OCCn1cc(C=O)cn1	**3**{*43*}	CC(C)(C)OC(=O)N1CCC(CC1)[N+]#[C-]	64.7	48
116	**4**{*112,77,10*}	COC1=CN=CC2=NC(CC3CCCC3)=C(NC3CCOC3)N12 |c:4,t:2,6,15|	Z8781341791	**1**{*112*}	COc1cncc(N)n1	**2**{*77*}	O=CCC1CCCC1	**3**{*10*}	[C-]#[N+]C1CCOC1	40.6	48
117	**4**{*113,78,14*}	COCCNC1=C(N=C2N1C=CC=C2N1CCOCC1)C1COC(C)C1 |c:7,11,13,t:5|	Z8810903019	**1**{*113*}	Nc1ncccc1N2CCOCC2	**2**{*78*}	CC1CC(CO1)C=O	**3**{*14*}	COCC[N+]#[C-]	46.3	48
118	**4**{*114,40,7*}	COCC1=CC2=NC(=C(NC3CCOCC3)N2C=C1)C1=NC(CO)=CS1 |c:19,26,t:3,5,7,22|	Z8786958883	**1**{*114*}	COCc1ccnc(N)c1	**2**{*40*}	OCc1csc(C=O)n1	**3**{*7*}	[C-]#[N+]C1CCOCC1	48.0	48
119	**4**{*46,7,9*}	CNC1=C(N=C2C=CC=CN12)C1=NC(C)=CC(CO)=C1 |c:6,8,16,20,t:2,4,13|	Z8803896924	**1**{*46*}	Nc1ccccn1	**2**{*7*}	Cc1cc(CO)cc(C=O)n1	**3**{*9*}	C[N+]#[C-]	34.4	47
120	**4**{*62,15,27*}	CCCCNC1=C(N=C2C=C(CO)C=CN12)C1=CC=C(OC)C=C1 |c:13,24,t:5,7,9,18,20|	Z8741907001	**1**{*62*}	Nc1cc(CO)ccn1	**2**{*15*}	COc1ccc(C=O)cc1	**3**{*27*}	CCCC[N+]#[C-]	41.5	47
121	**4**{*44,79,13*}	CCC1=CN2C(NCC3=CC=C(OC)C=C3)=C(N=C2C=N1)C1=NC=CO1 |c:14,16,18,21,26,t:2,8,10,24|	Z8803896965	**1**{*44*}	CCc1cnc(N)cn1	**2**{*79*}	O=Cc1ncco1	**3**{*13*}	COc1ccc(C[N+]#[C-])cc1	44.4	47
122	**4**{*115,26,26*}	OCC1=CN2C(C=N1)=NC(CCC1=CC=CC=C1)=C2NCC1=CC=C(F)C=C1 |c:6,8,15,17,19,29,t:2,13,24,26|	Z8776690347	**1**{*115*}	Nc1cnc(CO)cn1	**2**{*26*}	O=CCCc1ccccc1	**3**{*26*}	Fc1ccc(C[N+]#[C-])cc1	47.8	47

123	**4**{*116,15,9*}	CNC1=C(N=C2C=CC(C)=C(C#N)N12)C1=CC=C(OC)C=C1 |c:6,22,t:2,4,9,16,18|	Z8776690312	**1**{*116*}	Cc1ccc(N)nc1C#N	**2**{*15*}	COc1ccc(C=O)cc1	**3**{9}	C[N+]#[C-]	36.9	47
124	**4**{*117,80,20*}	COC1=CC(C)=C(NC2=C(N=C3C=CC=C(N23)C2(CCC2)C(F)(F)F)C2=NC=C(C)S2)C=C1 |c:12,14,35,t:2,5,8,10,28,30|	Z8797785072	**1**{*117*}	Nc1cccc(n1)C2(CCC2)C(F)(F)F	**2**{*80*}	Cc1cnc(C=O)s1	**3**{*20*}	COc1ccc([N+]#[C-])c(C)c1	58.8	46
125	**4**{*34,81,23*}	COC(=O)CCC(NC1=C(N=C2C=C(C=CN12)C1=NNC(C)=N1)C1=NC(=CC=C1)C(F)F)C(=O)OC |c:12,14,23,28,30,t:8,10,19,26|	Z8810902953	**1**{*34*}	Cc1nc(n[nH]1)c2ccnc(N)c2	**2**{*81*}	FC(F)c1cccc(C=O)n1	**3**{*23*}	COC(=O)CCC([N+]#[C-])C(=O)OC	62.1	46
126	**4**{*118,82,32*}	COC1=C(C=CC=C1F)C1=C(NC2CCC2)N2C(C=CC=C2C(C)O)=N1 |c:4,6,10,20,22,27,t:2|	Z8801681833	**1**{*118*}	Cl.CC(O)c1cccc(N)n1	**2**{*82*}	COc1c(F)cccc1C=O	**3**{*32*}	[C-]#[N+]C1CCC1	44.2	46
127	**4**{*119,14,17*}	CC1=CC=C(C=C1)C1=C(NCC2CCOCC2)N2C=C(C=CC2=N1)C#N |c:3,5,8,20,22,25,t:1|	Z8776690318	**1**{*119*}	Nc1ccc(C#N)cn1	**2**{*14*}	Cc1ccc(C=O)cc1	**3**{*17*}	[C-]#[N+]CC1CCOCC1	43.0	46
128	**4**{*120,83,5*}	COCCCNC1=C(N=C2C=C(C=CN12)C1=CN(C)N=C1)C1=CC(=CC=C1)S(N)(=O)=O |c:10,12,21,26,28,t:6,8,17,24|	Z8798985044	**1**{*120*}	Cn1cc(cn1)c2ccnc(N)c2	**2**{*83*}	NS(=O)(=O)c1cccc(C=O)c1	**3**{*5*}	COCCC[N+]#[C-]	54.4	46
129	**4**{*65,84,7*}	CCC1=CN=CC2=NC(CCSC)=C(NC3CCOCC3)N12 |c:4,t:2,6,12|	Z8778278039	**1**{*65*}	CCc1cncc(N)n1	**2**{*84*}	CSCCC=O	**3**{*7*}	[C-]#[N+]C1CCOCC1	39.0	45
130	**4**{*104,85,44*}	CC(C)(C)OC(=O)NCCCCNC1=C(N=C2C=CC3=C(COCC3)N12)C1=CN=NS1 |c:17,31,t:13,15,19,29|	Z8810903041	**1**{*104*}	Nc1ccc2CCOCc2n1	**2**{*85*}	O=Cc1cnns1	**3**{*44*}	CC(C)(C)OC(=O)NCCCC[N+]#[C-]	53.8	45
131	**4**{*121,39,10*}	BrC1=CC2=NC(C3=COC=C3)=C(NC3CCOC3)N2C=C1 |c:9,22,t:1,3,6,11|	Z8808559862	**1**{*121*}	Nc1cc(Br)ccn1	**2**{*39*}	O=Cc1ccoc1	**3**{*10*}	[C-]#[N+]C1CCOC1	42.0	45
132	**4**{*81,46,13*}	COCCCCCC1=C(NCC2=CC=C(OC)C=C2)N2C=CN=CC2=N1 |c:7,17,21,23,26,t:11,13|	Z8808559867	**1**{*81*}	Nc1cnccn1	**2**{*46*}	COCCCCCC=O	**3**{*13*}	COc1ccc(C[N+]#[C-])cc1	42.4	44
133	**4**{*122,10,7*}	CC(=O)NC1=CC=CN2C(NC3CCOCC3)=C(N=C12)C1=NC=CC(C)=C1 |c:6,17,25,28,t:4,19,23|	Z8784059625	**1**{*122*}	Cl.CC(=O)Nc1cccnc1N	**2**{*10*}	Cc1ccnc(C=O)c1	**3**{*7*}	[C-]#[N+]C1CCOCC1	43.7	44
134	**4**{*32,86,35*}	CSCCCNC1=C(N=C2C=C(C=CN12)P(C)(C)=O)C1CCN(C(=O)OC(C)(C)C)C1(C)C |c:10,12,t:6,8|	Z8803896948	**1**{*32*}	CP(=O)(C)c1ccnc(N)c1	**2**{*86*}	CC(C)(C)OC(=O)N1CCC(C=O)C1(C)C	**3**{*35*}	CSCCC[N+]#[C-]	59.1	44

135	**4**{*123*,*87*,*22*}	CC(C)(C)OC(=O)[C@@H]1C[C@H]1C1=C(NC2COC2)N2C=CC(OCCO)=CC2=N1 |&1:7,9,r,c:11,20,26,29|	Z8810903001	**1**{*123*}	Cl.Nc1cc(OCCO)ccn1	**2**{*87*}	CC(C)(C)OC(=O)[C@@H]1C[C@H]1C=O |&1:7,9,r|	**3**{*22*}	[C-]#[N+]C1COC1	46.5	44
136	**4**{*38*,*19*,*9*}	CNC1=C(N=C2C=C(C=CN12)C#N)C1=C(OC)C=NN1C |c:6,8,15,19,t:2,4|	Z8801681823	**1**{*38*}	Nc1cc(C#N)ccn1	**2**{*19*}	COc1cnn(C)c1C=O	**3**{*9*}	C[N+]#[C-]	33.7	44
137	**4**{*73*,*88*,*20*}	COC1=CC(C)=C(NC2=C(N=C3C=CC4=NC=CN4N23)C2=CC3=C(C=CC=N3)C=C2)C=C1 |c:12,16,27,29,32,35,t:2,5,8,10,14,23,25|	Z8810903009	**1**{*73*}	Nc1ccc2nccn2n1	**2**{*88*}	O=Cc1ccc2ccnc2c1	**3**{*20*}	COc1ccc([N+]#[C-])c(C)c1	49.7	44
138	**4**{*70*,*89*,*35*}	CCC1=NC=C(C=N1)C1=C(NCCCSC)N2C=CN=C(C3CC3)C2=N1 |c:4,6,9,18,27,t:2,20|	Z8798985038	**1**{*70*}	Nc1nccnc1C2CC2	**2**{*89*}	CCc1ncc(C=O)cn1	**3**{*35*}	CSCCC[N+]#[C-]	43.3	43
139	**4**{*109*,*90*,*41*}	COC1=CC=C(NC2=C(N=C3C=CC=C(N23)S(N)(=O)=O)C2=COC(C)=N2)C=C1 |c:11,13,26,29,t:2,4,7,9,22|	Z8808559860	**1**{*109*}	Cl.Nc1cccc(n1)S(=O)(=O)N	**2**{*90*}	Cc1nc(C=O)co1	**3**{*41*}	COc1ccc([N+]#[C-])cc1	46.6	43
140	**4**{*124*,*91*,*14*}	COCCNC1=C(N=C2N1C=CC=C2CO)C1=CC2=C(OCCC2)C=C1 |c:7,11,13,27,t:5,18,20|	Z8778278032	**1**{*124*}	Nc1ncccc1CO	**2**{*91*}	O=Cc1ccc2OCCCc2c1	**3**{*14*}	COCC[N+]#[C-]	41.0	43
141	**4**{*50*,*92*,*7*}	CC1=NOC(=C1)C1=C(NC2CCOCC2)N2C(C=CC=C2P(C)(C)=O)=N1 |c:4,7,19,21,27,t:1|	Z8810903002	**1**{*50*}	CP(=O)(C)c1cccc(N)n1	**2**{*92*}	Cc1cc(C=O)on1	**3**{*7*}	[C-]#[N+]C1CCOCC1	43.4	43
142	**4**{*54*,*93*,*3*}	CN(C)C(=O)C1=CN2C(C=C1)=NC(=C2NC1=C(F)C=C(Cl)C=C1)C1=NC(CO)=CC=C1 |c:9,11,13,17,23,30,32,t:5,20,26|	Z8798985024	**1**{*54*}	Cl.CN(C)C(=O)c1ccc(N)nc1	**2**{*93*}	OCc1cccc(C=O)n1	**3**{*3*}	Fc1cc(Cl)ccc1[N+]#[C-]	50.9	43
143	**4**{*125*,*50*,*26*}	OCCC1=CN2C(C=C1)=NC([C@@H]1C[C@H]1C1OCCO1)=C2NCC1=CC=C(F)C=C1 |&1:11,13,r,c:7,9,21,31,t:3,26,28|	Z8803896967	**1**{*125*}	Nc1ccc(CCO)cn1	**2**{*50*}	O=C[C@@H]1C[C@H]1C2OCCO2 |&1:2,4,r|	**3**{*26*}	Fc1ccc(C[N+]#[C-])cc1	46.0	43
144	**4**{*126*,*94*,*45*}	COC1=C(F)N2C(NCC=C)=C(N=C2C=C1)C1=NC=CN=C1 |c:2,10,12,15,20,22,t:18|	Z8781341797	**1**{*126*}	COc1ccc(N)nc1F	**2**{*94*}	O=Cc1cnccn1	**3**{*45*}	C=CC[N+]#[C-]	34.6	43
145	**4**{*127*,*9*,*27*}	CCCCNC1=C(N=C2N1C=CC=C2F)C1=CSC=C1 |c:7,11,13,20,t:5,17|	Z8780121542	**1**{*127*}	Nc1ncccc1F	**2**{*9*}	O=Cc1ccsc1	**3**{*27*}	CCCC[N+]#[C-]	33.0	42
146	**4**{*67*,*95*,*9*}	CNC1=C(N=C2C=CC=C(C)N12)C1=CC(=NC=C1)N(C)C |c:6,16,18,t:2,4,8,14|	Z8810903035	**1**{*67*}	Cc1cccc(N)n1	**2**{*95*}	CN(C)c1cc(C=O)ccn1	**3**{*9*}	C[N+]#[C-]	32.1	42

<table>
<tr><th></th><th></th><th></th><th></th><th></th><th></th><th></th><th></th><th></th><th></th><th></th><th></th></tr>
<tr><td>147</td><td>4{128,26,7}</td><td>COCCN(C)C1=CN2C(C=C1)=NC(CCC1=CC=CC=C1)=C2NC1CCOCC1 |c:10,12,19,21,23,t:6,17|</td><td>Z8776690315</td><td>1{128}</td><td>COCCN(C)c1ccc(N)nc1</td><td>2{26}</td><td>O=CCCc1ccccc1</td><td>3{7}</td><td>[C-]#[N+]C1CCOCC1</td><td>46.5</td><td>42</td></tr>
<tr><td>148</td><td>4{50,96,26}</td><td>CP(C)(=O)C1=CC=CC2=NC(C3=CC(=NO3)C3=CC=CC=C3)=C(NCC3=CC=C(F)C=C3)N12 |c:6,13,19,21,32,t:4,8,11,17,23,27,29|</td><td>Z8810902990</td><td>1{50}</td><td>CP(=O)(C)c1cccc(N)n1</td><td>2{96}</td><td>O=Cc1cc(no1)c2ccccc2</td><td>3{26}</td><td>Fc1ccc(C[N+]#[C-])cc1</td><td>52.0</td><td>42</td></tr>
<tr><td>149</td><td>4{40,25,22}</td><td>CC1=CC(=CC2=NC(=C(NC3COC3)N12)C1=CN=C(N=C1)C1CCOCC1)C(F)(F)F |c:3,20,22,t:1,5,7,18|</td><td>Z8786958875</td><td>1{40}</td><td>Cc1cc(cc(N)n1)C(F)(F)F</td><td>2{25}</td><td>O=Cc1cnc(nc1)C2CCOCC2</td><td>3{22}</td><td>[C-]#[N+]C1COC1</td><td>48.9</td><td>42</td></tr>
<tr><td>150</td><td>4{129,97,17}</td><td>CSC1=CC=CC2=NC(=C(NCC3CCOCC3)N12)C1=CC=C(F)C=C1 |c:4,27,t:2,6,8,22,24|</td><td>Z8797781065</td><td>1{129}</td><td>CSc1cccc(N)n1</td><td>2{97}</td><td>Fc1ccc(C=O)cc1</td><td>3{17}</td><td>[C-]#[N+]CC1CCOCC1</td><td>41.7</td><td>42</td></tr>
<tr><td>151</td><td>4{130,77,27}</td><td>CCCCNC1=C(CC2CCCC2)N=C2N1C=C(F)C=C2Cl |c:5,14,21,t:18|</td><td>Z8780121734</td><td>1{130}</td><td>Nc1ncc(F)cc1Cl</td><td>2{77}</td><td>O=CCC1CCCC1</td><td>3{27}</td><td>CCCC[N+]#[C-]</td><td>35.9</td><td>41</td></tr>
<tr><td>152</td><td>4{131,26,7}</td><td>C(CC1=CC=CC=C1)C1=C(NC2CCOCC2)N2N=C(C=CC2=N1)C1=CC=CC=C1 |c:4,6,9,20,22,25,30,32,t:2,28|</td><td>Z8776690317</td><td>1{131}</td><td>Nc1ccc(nn1)c2ccccc2</td><td>2{26}</td><td>O=CCCc1ccccc1</td><td>3{7}</td><td>[C-]#[N+]C1CCOCC1</td><td>44.1</td><td>41</td></tr>
<tr><td>153</td><td>4{76,26,7}</td><td>CC1=C(Cl)N2C(C=C1)=NC(CCC1=CC=CC=C1)=C2NC1CCOCC1 |c:1,6,8,15,17,19,t:13|</td><td>Z8776690313</td><td>1{76}</td><td>Cc1ccc(N)nc1Cl</td><td>2{26}</td><td>O=CCCc1ccccc1</td><td>3{7}</td><td>[C-]#[N+]C1CCOCC1</td><td>40.4</td><td>40</td></tr>
<tr><td>154</td><td>4{132,15,13}</td><td>CNS(=O)(=O)C1=CC2=NC(=C(NCC3=CC=C(OC)C=C3)N2C=C1)C1=CC=C(OC)C=C1 |c:19,24,33,t:5,7,9,13,15,27,29|</td><td>Z8776690328</td><td>1{132}</td><td>Cl.CNS(=O)(=O)c1ccnc(N)c1</td><td>2{15}</td><td>COc1ccc(C=O)cc1</td><td>3{13}</td><td>COc1ccc(C[N+]#[C-])cc1</td><td>49.4</td><td>40</td></tr>
<tr><td>155</td><td>4{91,94,6}</td><td>CCOC(=O)CCCNC1=C(N=C2C=CC(=CN12)C(N)=O)C1=NC=CN=C1 |c:13,15,25,27,t:9,11,23|</td><td>Z8784059427</td><td>1{91}</td><td>NC(=O)c1ccc(N)nc1</td><td>2{94}</td><td>O=Cc1cnccn1</td><td>3{6}</td><td>CCOC(=O)CCC[N+]#[C-]</td><td>40.2</td><td>40</td></tr>
<tr><td>156</td><td>4{133,41,14}</td><td>COCCNC1=C(N=C2N1C=C(F)C=C2Br)C1=NC=CC=C1 |c:7,14,20,22,t:5,11,18|</td><td>Z8786838536</td><td>1{133}</td><td>Nc1ncc(F)cc1Br</td><td>2{41}</td><td>O=Cc1ccccn1</td><td>3{14}</td><td>COCC[N+]#[C-]</td><td>39.6</td><td>40</td></tr>
<tr><td>157</td><td>4{134,77,27}</td><td>CCCCNC1=C(CC2CCCC2)N=C2N1C=CC=C2OC1CCOCC1 |c:5,14,18,20|</td><td>Z8778277289</td><td>1{134}</td><td>Nc1ncccc1OC2CCOCC2</td><td>2{77}</td><td>O=CCC1CCCC1</td><td>3{27}</td><td>CCCC[N+]#[C-]</td><td>40.0</td><td>40</td></tr>
<tr><td>158</td><td>4{124,98,46}</td><td>COC1=NC=C(N=C1)C1=C(NCC(C)=C)N2C=CC=C(CO)C2=N1 |c:4,6,9,17,24,t:2,19|</td><td>Z8781341794</td><td>1{124}</td><td>Nc1ncccc1CO</td><td>2{98}</td><td>COc1cnc(C=O)cn1</td><td>3{46}</td><td>CC(=C)C[N+]#[C-]</td><td>35.0</td><td>40</td></tr>
<tr><td>159</td><td>4{135,99,35}</td><td>CCCC1=NC=CN2C(NCCCSC)=C(N=C12)C1=COC=N1 |c:5,14,23,t:3,16,20|</td><td>Z8803896937</td><td>1{135}</td><td>CCCc1nccnc1N</td><td>2{99}</td><td>O=Cc1cocn1</td><td>3{35}</td><td>CSCCC[N+]#[C-]</td><td>35.6</td><td>40</td></tr>
<tr><td>160</td><td>4{35,15,9}</td><td>CNC1=C(N=C2C=CC(C)=CN12)C1=CC=C(OC)C=C1 |c:6,9,20,t:2,4,14,16|</td><td>Z8712537091</td><td>1{35}</td><td>Cc1ccc(N)nc1</td><td>2{15}</td><td>COc1ccc(C=O)cc1</td><td>3{9}</td><td>C[N+]#[C-]</td><td>28.7</td><td>40</td></tr>
</table>

161	**4**{*96,100,47*}	CCN1C=C(C=N1)[C@@H]1OCC[C@H]1C1=C(NC(C)C)N2C=CC(CN(C)CCOC)=CC2=N1 |&1:7,11,r,c:3,5,14,21,30,33|	Z8801681818	**1**{*96*}	COCCN(C)Cc1ccnc(N)c1	**2**{*100*}	CCn1cc(cn1)[C@@H]2OCC[C@H]2C=O |&1:7,11,r|	**3**{*47*}	CC(C)[N+]#[C-]	47.3	40
162	**4**{*36,26,7*}	BrC1=CN2C(C=C1)=NC(CCC1=CC=CC=C1)=C2NC1CCOCC1 |c:5,7,14,16,18,t:1,12|	Z8712587521	**1**{*36*}	Nc1ccc(Br)cn1	**2**{*26*}	O=CCCc1ccccc1	**3**{*7*}	[C-]#[N+]C1CCOCC1	42.6	39
163	**4**{*136,101,14*}	COCCNC1=C(N=C2N1C=C(C)C=C2Br)C1=C(CO)C=CS1 |c:7,14,18,22,t:5,11|	Z8810903027	**1**{*136*}	Cc1cnc(N)c(Br)c1	**2**{*101*}	OCc1ccsc1C=O	**3**{*14*}	COCC[N+]#[C-]	41.9	39
164	**4**{*137,15,17*}	COC1=CC=C(C=C1)C1=C(NCC2CCOCC2)N2N=C(C)C=CC2=N1 |c:4,6,9,24,27,t:2,21|	Z8741907028	**1**{*137*}	Cc1ccc(N)nn1	**2**{*15*}	COc1ccc(C=O)cc1	**3**{*17*}	[C-]#[N+]CC1CCOCC1	37.2	39
165	**4**{*32,102,31*}	CSCCNC1=C(N=C2C=C(C=CN12)P(C)(C)=O)C1=C(Br)C(C)=CC(Cl)=N1 |c:9,11,20,24,27,t:5,7|	Z8801681845	**1**{*32*}	CP(=O)(C)c1ccnc(N)c1	**2**{*102*}	Cc1cc(Cl)nc(C=O)c1Br	**3**{*31*}	CSCC[N+]#[C-]	51.2	39
166	**4**{*138,103,48*}	CC(C)(C)OC(=O)N1CCCC(C1)NC1=C(N=C2C=CC=C(F)N12)C1CCC2(COC2)OC1 |c:19,t:15,17,21|	Z8798985030	**1**{*138*}	Nc1cccc(F)n1	**2**{*103*}	O=CC1CCC2(COC2)OC1	**3**{*48*}	CC(C)(C)OC(=O)N1CCCC(C1)[N+]#[C-]	48.4	39
167	**4**{*139,104,24*}	COC1=CC(CNC2=C(N=C3C=C(C=CN23)C(F)F)C2=NC(C)=CO2)=CC=C1 |c:11,13,24,27,29,t:2,7,9,21|	Z8810902993	**1**{*139*}	Nc1cc(ccn1)C(F)F	**2**{*104*}	Cc1coc(C=O)n1	**3**{*24*}	COc1cccc(C[N+]#[C-])c1	40.0	39
168	**4**{*109,105,34*}	CN1N=NC2=C1C(=CC=C2)C1=C(NCC2CCCO2)N2C(C=CC=C2S(N)(=O)=O)=N1 |c:2,4,7,9,12,24,26,32|	Z8803896953	**1**{*109*}	Cl.Nc1cccc(n1)S(=O)(=O)N	**2**{*105*}	Cn1nnc2cccc(C=O)c12	**3**{*34*}	[C-]#[N+]CC1CCCO1	44.4	38
169	**4**{*140,106,49*}	COC1=C(N=CC=N1)C1=C(NC2CC(F)(F)C2)N2C(C=NC(I)=C2Cl)=N1 |c:4,6,9,21,24,27,t:2|	Z8781341776	**1**{*140*}	Nc1cnc(I)c(Cl)n1	**2**{*106*}	COc1nccnc1C=O	**3**{*49*}	FC1(F)CC(C1)[N+]#[C-]	50.9	38
170	**4**{*36,26,16*}	CC1=CC=CC(C)=C1NC1=C(CCC2=CC=CC=C2)N=C2C=CC(Br)=CN12 |c:3,6,10,16,18,23,26,t:1,14,21|	Z8712537093	**1**{*36*}	Nc1ccc(Br)cn1	**2**{*26*}	O=CCCc1ccccc1	**3**{*16*}	Cc1cccc(C)c1[N+]#[C-]	43.5	38
171	**4**{*125,107,31*}	CSCCNC1=C(N=C2C=CC(CCO)=CN12)C1=NC(C)=CS1 |c:9,14,22,t:5,7,19|	Z8810902950	**1**{*125*}	Nc1ccc(CCO)cn1	**2**{*107*}	Cc1csc(C=O)n1	**3**{*31*}	CSCC[N+]#[C-]	36.0	38
172	**4**{*80,108,23*}	COC(=O)CCC(NC1=C(N=C2N1C=CN=C2OC)C1=CN=C(N=C1)N1CCOCC1)C(=O)OC |c:10,14,16,23,25,t:8,21|	Z8801681842	**1**{*80*}	COc1nccnc1N	**2**{*108*}	O=Cc1cnc(nc1)N2CCOCC2	**3**{*23*}	COC(=O)CCC([N+]#[C-])C(=O)OC	49.5	38

173	**4**{*81,26,13*}	COC1=CC=C(CNC2=C(CCC3=CC=CC=C3)N=C3C=NC=CN23)C=C1 |c:8,14,16,21,23,28,t:2,4,12,19|	Z8712562312	**1**{*81*}	Nc1cnccn1	**2**{*26*}	O=CCCc1ccccc1	**3**{*13*}	COc1ccc(C[N+]#[C-])cc1	36.5	38
174	**4**{*141,109,32*}	CN1C=C(C)C(=N1)C1=C(NC2CCC2)N2C=CC(NC(C)=O)=CC2=N1 |c:5,8,17,23,26,t:2|	Z8786958863	**1**{*141*}	CC(=O)Nc1ccnc(N)c1	**2**{*109*}	Cc1cn(C)nc1C=O	**3**{*32*}	[C-]#[N+]C1CCC1	34.4	38
175	**4**{*109,110,50*}	CP(C)(=O)C1=NC=C(C=C1)C1=C(NC2=CC3=C(OCCO3)C=C2)N2C(C=CC=C2S(N)(=O)=O)=N1 |c:6,8,11,23,28,30,36,t:4,14,16|	Z8803896963	**1**{*109*}	Cl.Nc1cccc(n1)S(=O)(=O)N	**2**{*110*}	CP(=O)(C)c1ccc(C=O)cn1	**3**{*50*}	[C-]#[N+]c1ccc2OCCOc2c1	50.5	37
176	**4**{*48,93,35*}	CSCCCNC1=C(N=C2C=C(C=CN12)S(C)(=O)=O)C1=NC(CO)=CC=C1 |c:10,12,25,27,t:6,8,21|	Z8801681824	**1**{*48*}	CS(=O)(=O)c1ccnc(N)c1	**2**{*93*}	OCc1cccc(C=O)n1	**3**{*35*}	CSCCC[N+]#[C-]	41.1	37
177	**4**{*142,111,51*}	CC(C)(C)OC(=O)NCCNC1=C(N=C2N1C=CC=C2OCCS(C)(=O)=O)C1CCCC1 |c:13,17,19,t:11|	Z8808559876	**1**{*142*}	CS(=O)(=O)CCOc1cccnc1N	**2**{*111*}	O=CC1CCCC1	**3**{*51*}	CC(C)(C)OC(=O)NCC[N+]#[C-]	47.2	37
178	**4**{*83,112,52*}	CP(C)(=O)C1=CN2C(C=C1)=NC(=C2NCCOCC1CC1)C1=C2C=CC=NN2N=C1 |c:8,10,12,24,26,28,32,t:4|	Z8786958945	**1**{*83*}	CP(=O)(C)c1ccc(N)nc1	**2**{*112*}	O=Cc1cnn2ncccc12	**3**{*52*}	[C-]#[N+]CCOCC1CC1	42.8	37
179	**4**{*143,113,10*}	CCC1=NN2C(C=C1)=NC(=C2NC1CCOC1)C1=CC(COC)=C(OC)C=C1 |c:6,8,10,29,t:2,20,25|	Z8801681837	**1**{*143*}	CCc1ccc(N)nn1	**2**{*113*}	COCc1cc(C=O)ccc1OC	**3**{*10*}	[C-]#[N+]C1CCOC1	38.6	37
180	**4**{*37,114,39*}	CCOCCNC1=C(N=C2C=CC=C(C(F)F)N12)[C@@H]1CC[C@@H](C1)C(=O)OC |&1:18,21,r,c:10,t:6,8,12|	Z8801681838	**1**{*37*}	Cl.Nc1cccc(n1)C(F)F	**2**{*114*}	COC(=O)[C@@H]1CC[C@@H](C1)C=O |&1:4,7,r|	**3**{*39*}	CCOCC[N+]#[C-]	38.4	37
181	**4**{*144,115,15*}	CCNC1=C(N=C2C=CC(=CN12)S(=O)(=O)NC)C1=CC(OC)=NN1C |c:7,9,23,t:3,5,19|	Z8808559877	**1**{*144*}	CNS(=O)(=O)c1ccc(N)nc1	**2**{*115*}	COc1cc(C=O)n(C)n1	**3**{*15*}	CC[N+]#[C-]	36.4	37
182	**4**{*145,116,17*}	CS(=O)(=O)C1=CC=CC2=NC(=C(NCC3CCOCC3)N12)C1=CN(CCC=C)N=N1 |c:6,31,t:4,8,10,24|	Z8778278046	**1**{*145*}	CS(=O)(=O)c1cccc(N)n1	**2**{*116*}	C=CCCn1cc(C=O)nn1	**3**{*17*}	[C-]#[N+]CC1COCC1	42.0	36
183	**4**{*146,117,12*}	CS(=O)(=O)C1=CN2C(C=C1)=NC(C1CCCC11CC1)=C2NCC1CCOC1 |c:8,10,21,t:4|	Z8810903032	**1**{*146*}	CS(=O)(=O)c1ccc(N)nc1	**2**{*117*}	O=CC1CCCC21CC2	**3**{*12*}	[C-]#[N+]CC1COC1	37.9	36

184	**4**{*147,118,17*}	CC1=NC=CC(=C1)C1=C(NCC2CCOCC2)N2C3=C(CCC3)C=CC2=N1 |c:3,5,8,26,29,t:1,20|	Z8778277442	**1**{*147*}	Nc1ccc2CCCc2n1	**2**{*118*}	Cc1cc(C=O)ccn1	**3**{*17*}	[C-]#[N+]CC1CCOCC1	35.2	36
185	**4**{*148,14,14*}	COCCNC1=C(N=C2C=CC(=CN12)S(=O)(=O)N1CCCCC1)C1=CC=C(C)C=C1 |c:9,11,31,t:5,7,26,28|	Z8776690322	**1**{*148*}	Nc1ccc(cn1)S(=O)(=O)N2CCCCC2	**2**{*14*}	Cc1ccc(C=O)cc1	**3**{*14*}	COCC[N+]#[C-]	41.5	36
186	**4**{*149,77,17*}	ClC1=CN2C(NCC3CCOCC3)=C(CC3CCCC3)N=C2C(Br)=C1 |c:22,26,t:1,13|	Z8786845282	**1**{*149*}	Nc1ncc(Cl)cc1Br	**2**{*77*}	O=CCC1CCCC1	**3**{*17*}	[C-]#[N+]CC1CCOCC1	41.3	36
187	**4**{*150,119,53*}	CCOC(=O)C1=NN=C(N1)C1=C(NCCOCC2=CC=CC=C2)N2C=CC(=CC2=N1)C1CCOC1 |c:7,11,20,22,26,28,31,t:5,18|	Z8801681847	**1**{*150*}	Nc1cc(ccn1)C2CCOC2	**2**{*119*}	CCOC(=O)c1nnc(C=O)[nH]1	**3**{*53*}	[C-]#[N+]CCOCc1ccccc1	46.1	36
188	**4**{*81,48,13*}	COC1=CC=C(CNC2=C(N=C3C=NC=CN23)C2=CNC(=N2)C2CCC2)C=C1 |c:12,14,22,30,t:2,4,8,10,19|	Z8810903030	**1**{*81*}	Nc1cnccn1	**2**{*48*}	O=Cc1c[nH]c(n1)C2CCC2	**3**{*13*}	COc1ccc(C[N+]#[C-])cc1	36.0	36
189	**4**{*36,14,18*}	CC1=CC=C(C=C1)C1=C(NC(C)(C)C)N2C=C(Br)C=CC2=N1 |c:3,5,8,19,22,t:1,16|	Z3220348675	**1**{*36*}	Nc1ccc(Br)cn1	**2**{*14*}	Cc1ccc(C=O)cc1	**3**{*18*}	CC(C)(C)[N+]#[C-]	34.4	36
190	**4**{*151,14,14*}	COCCNC1=C(N=C2C=NC=C(Cl)N12)C1=CC=C(C)C=C1 |c:9,22,t:5,7,11,17,19|	Z8711872648	**1**{*151*}	Nc1cncc(Cl)n1	**2**{*14*}	Cc1ccc(C=O)cc1	**3**{*14*}	COCC[N+]#[C-]	29.6	35
191	**4**{*35,26,54*}	CC1=CN2C(C=C1)=NC(CCC1=CC=CC=C1)=C2NC1=CC=CC=C1 |c:5,7,14,16,18,24,26,t:1,12,22|	Z8700285742	**1**{*35*}	Cc1ccc(N)nc1	**2**{*26*}	O=CCCc1ccccc1	**3**{*54*}	[C-]#[N+]c1ccccc1	30.6	35
192	**4**{*145,120,22*}	CS(=O)(=O)C1=CC=CC2=NC(=C(NC3COC3)N12)C1=NC2=C(C=CC=C2)C=N1 |c:6,25,27,30,t:4,8,10,21,23|	Z8808559874	**1**{*145*}	CS(=O)(=O)c1cccc(N)n1	**2**{*120*}	O=Cc1ncc2ccccc2n1	**3**{*22*}	[C-]#[N+]C1COC1	36.8	34
193	**4**{*152,29,7*}	CCN1C=CC(=N1)C1=C(NC2CCOCC2)N2C=C(N=C(Cl)C2=N1)C1=CC=CC=C1 |c:3,5,8,19,25,30,32,t:21,28|	Z8786927263	**1**{*152*}	Nc1ncc(nc1Cl)c2ccccc2	**2**{*29*}	CCn1ccc(C=O)n1	**3**{*7*}	[C-]#[N+]C1CCOCC1	38.9	34
194	**4**{*141,121,55*}	CCC1=CON=C1C1=C(NCCC#N)N2C=CC(NC(C)=O)=CC2=N1 |c:5,8,16,22,25,t:2|	Z8786958870	**1**{*141*}	CC(=O)Nc1ccnc(N)c1	**2**{*121*}	CCc1conc1C=O	**3**{*55*}	[C-]#[N+]CCC#N	31.1	34
195	**4**{*153,122,15*}	CCNC1=C(N=C2C=CC=C(N12)C(C)(C)O)C1=CN=C(N=C1)C1=CC=CS1 |c:7,9,20,22,27,t:3,5,18,25|	Z8798985031	**1**{*153*}	CC(C)(O)c1cccc(N)n1	**2**{*122*}	O=Cc1cnc(nc1)c2cccs2	**3**{*15*}	CC[N+]#[C-]	34.3	33
196	**4**{*143,103,33*}	CCC1=NN2C(NCC3=C(OC)C=CC=C3)=C(N=C2C=C1)C1CCC2(COC2)OC1 |c:8,12,14,16,18,21,t:2|	Z8798985020	**1**{*143*}	CCc1ccc(N)nn1	**2**{*103*}	O=CC1CCC2(COC2)OC1	**3**{*33*}	COc1ccccc1C[N+]#[C-]	36.8	33

<table>
<tr><td>197</td><td>4{154,123,49}</td><td>CC1=NC=C(N=C1)C1=C(NC2CC(F)(F)C2)N2C=CC(=CC2=N1)[N+]([O-])=O |c:3,5,8,19,21,24,t:1|</td><td>Z8803896961</td><td>1{154}</td><td>Nc1cc(ccn1)[N+](=O)[O-]</td><td>2{123}</td><td>Cc1cnc(C=O)cn1</td><td>3{49}</td><td>FC1(F)CC(C1)[N+]#[C-]</td><td>32.3</td><td>33</td></tr>
<tr><td>198</td><td>4{92,124,7}</td><td>COC1=C(OCC2=C(C)ON=C2C)C=CC(=C1)C1=C(NC2CCOCC2)N2C=CC(=CC2=N1)C(N)=O |c:2,6,10,14,16,19,30,32,35|</td><td>Z8810903018</td><td>1{92}</td><td>NC(=O)c1ccnc(N)c1</td><td>2{124}</td><td>COc1cc(C=O)ccc1OCc2c(C)noc2C</td><td>3{7}</td><td>[C-]#[N+]C1CCOCC1</td><td>43.0</td><td>32</td></tr>
<tr><td>199</td><td>4{155,125,7}</td><td>COC1=NC=NC(=C1)C1=C(NC2CCOCC2)N2C=CC=C(Cl)C2=N1 |c:4,6,9,20,26,t:2,22|</td><td>Z8810902988</td><td>1{155}</td><td>Nc1ncccc1Cl</td><td>2{125}</td><td>COc1cc(C=O)ncn1</td><td>3{7}</td><td>[C-]#[N+]C1CCOCC1</td><td>31.3</td><td>32</td></tr>
<tr><td>200</td><td>4{156,9,14}</td><td>COCCNC1=C(N=C2N1C(Cl)=CC=C2Cl)C1=CSC=C1 |c:7,12,14,21,t:5,18|</td><td>Z8786838532</td><td>1{156}</td><td>Nc1nc(Cl)ccc1Cl</td><td>2{9}</td><td>O=Cc1ccsc1</td><td>3{14}</td><td>COCC[N+]#[C-]</td><td>29.8</td><td>32</td></tr>
<tr><td>201</td><td>4{157,19,9}</td><td>CNC1=C(N=C2C=C(C)C=C(Br)N12)C1=C(OC)C=NN1C |c:15,19,t:2,4,6,9|</td><td>Z8808559897</td><td>1{157}</td><td>Cc1cc(N)nc(Br)c1</td><td>2{19}</td><td>COc1cnn(C)c1C=O</td><td>3{9}</td><td>C[N+]#[C-]</td><td>30.3</td><td>32</td></tr>
<tr><td>202</td><td>4{94,126,10}</td><td>COC1=CC(=CC(F)=C1)C1=C(NC2CCOC2)N2N=C(F)C=CC2=N1 |c:4,7,10,23,26,t:2,20|</td><td>Z8801681829</td><td>1{94}</td><td>Nc1ccc(F)nn1</td><td>2{126}</td><td>COc1cc(F)cc(C=O)c1</td><td>3{10}</td><td>[C-]#[N+]C1CCOC1</td><td>29.7</td><td>32</td></tr>
<tr><td>203</td><td>4{41,127,9}</td><td>CNC1=C(N=C2C=NC(C)=CN12)C1=CC(OC)=NS1 |c:6,9,18,t:2,4,14|</td><td>Z8798985021</td><td>1{41}</td><td>Cc1cnc(N)cn1</td><td>2{127}</td><td>COc1cc(C=O)sn1</td><td>3{9}</td><td>C[N+]#[C-]</td><td>23.5</td><td>32</td></tr>
<tr><td>204</td><td>4{158,128,18}</td><td>CC1=CC(C)=C(C2=C(NC(C)(C)C)N3C=C(N=CC3=N2)P(C)(C)=O)C(C)=C1 |c:6,14,16,19,27,t:1,4|</td><td>Z8798985019</td><td>1{158}</td><td>CP(=O)(C)c1cnc(N)cn1</td><td>2{128}</td><td>Cc1cc(C)c(C=O)c(C)c1</td><td>3{18}</td><td>CC(C)(C)[N+]#[C-]</td><td>32.7</td><td>32</td></tr>
<tr><td>205</td><td>4{38,129,13}</td><td>COC1=CC=C(CNC2=C(N=C3C=C(C=CN23)C#N)C2=CC=CC3=NC=NN23)C=C1 |c:12,14,23,27,32,t:2,4,8,10,21,25|</td><td>Z8801681835</td><td>1{38}</td><td>Nc1cc(C#N)ccn1</td><td>2{129}</td><td>O=Cc1cccc2ncnn12</td><td>3{13}</td><td>COc1ccc(C[N+]#[C-])cc1</td><td>33.6</td><td>31</td></tr>
<tr><td>206</td><td>4{106,15,9}</td><td>CNC1=C(N=C2C=CC(OC)=NN12)C1=CC=C(OC)C=C1 |c:6,10,21,t:2,4,15,17|</td><td>Z8776690310</td><td>1{106}</td><td>COc1ccc(N)nn1</td><td>2{15}</td><td>COc1ccc(C=O)cc1</td><td>3{9}</td><td>C[N+]#[C-]</td><td>23.9</td><td>31</td></tr>
<tr><td>207</td><td>4{41,15,9}</td><td>CNC1=C(N=C2C=NC(C)=CN12)C1=CC=C(OC)C=C1 |c:6,9,20,t:2,4,14,16|</td><td>Z8776690348</td><td>1{41}</td><td>Cc1cnc(N)cn1</td><td>2{15}</td><td>COc1ccc(C=O)cc1</td><td>3{9}</td><td>C[N+]#[C-]</td><td>22.4</td><td>31</td></tr>
<tr><td>208</td><td>4{159,9,7}</td><td>NC(=O)C1=CC=C(Cl)N2C(NC3CCOCC3)=C(N=C12)C1=CSC=C1 |c:17,26,t:3,5,19,23|</td><td>Z8780122422</td><td>1{159}</td><td>NC(=O)c1ccc(Cl)nc1N</td><td>2{9}</td><td>O=Cc1ccsc1</td><td>3{7}</td><td>[C-]#[N+]C1CCOCC1</td><td>31.3</td><td>31</td></tr>
<tr><td>209</td><td>4{160,77,27}</td><td>CCCCNC1=C(CC2CCCC2)N=C2N1C=C(Br)C=C2C#C |c:5,14,21,t:18|</td><td>Z8784059428</td><td>1{160}</td><td>Nc1ncc(Br)cc1C#C</td><td>2{77}</td><td>O=CCC1CCCC1</td><td>3{27}</td><td>CCCC[N+]#[C-]</td><td>31.1</td><td>31</td></tr>
<tr><td>210</td><td>4{85,130,35}</td><td>COC1=NN=C(C=C1)C1=C(NCCCSC)N2C(C=C(C=C2C)C#N)=N1 |c:4,6,9,19,21,26,t:2|</td><td>Z8810902989</td><td>1{85}</td><td>Cc1cc(C#N)cc(N)n1</td><td>2{130}</td><td>COc1ccc(C=O)nn1</td><td>3{35}</td><td>CSCCC[N+]#[C-]</td><td>30.6</td><td>31</td></tr>
</table>

211	**4**{*150*,*131*,*22*}	C1CC1C1=NNC(=N1)C1=C(NC2COC2)N2C=CC(=CC2=N1)C1CCOC1 |c:7,10,19,21,24,t:4|	Z8801681805	**1**{*150*}	Nc1cc(ccn1)C2CCOC2	**2**{*131*}	O=Cc1nc(n[nH]1)C2CC2	**3**{*22*}	[C-]#[N+]C1COC1	30.4	31
212	**4**{*83*,*132*,*18*}	COC1=C(C2=C(NC(C)(C)C)N3C=C(C=CC3=N2)P(C)(C)=O)C(Br)=C(F)C=C1 |c:2,4,12,14,17,28,t:25|	Z8801681816	**1**{*83*}	CP(=O)(C)c1ccc(N)nc1	**2**{*132*}	COc1ccc(F)c(Br)c1C=O	**3**{*18*}	CC(C)(C)[N+]#[C-]	38.4	30
213	**4**{*161*,*133*,*7*}	C(C1CCC=CC1)C1=C(NC2CCOCC2)N2N=CC=CC2=N1 |c:4,8,19,21,24|	Z8808559853	**1**{*161*}	Nc1cccnn1	**2**{*133*}	O=CCC1CCC=CC1	**3**{*7*}	[C-]#[N+]C1CCOCC1	25.6	30
214	**4**{*81*,*22*,*26*}	FC1=CC=C(CNC2=C(N=C3C=NC=CN23)C2=CC=CC=C2)C=C1 |c:11,13,20,22,25,t:1,3,7,9,18|	Z8712538668	**1**{*81*}	Nc1cnccn1	**2**{*22*}	O=Cc1ccccc1	**3**{*26*}	Fc1ccc(C[N+]#[C-])cc1	25.9	30
215	**4**{*162*,*134*,*12*}	CCNC(=O)C1=CC2=NC(=C(NCC3CCOC3)N2C2=C1C=CC=C2)C1=NC(OC)=CC=N1 |c:21,24,26,33,35,t:5,7,9,29|	Z8810902973	**1**{*162*}	CCNC(=O)c1cc(N)nc2ccccc12	**2**{*134*}	COc1ccnc(C=O)n1	**3**{*12*}	[C-]#[N+]CC1CCOC1	35.9	30
216	**4**{*163*,*97*,*27*}	CCCCNC1=C(N=C2C=CC3=C(C=CC=C3)N12)C1=CC=C(F)C=C1 |c:9,13,15,26,t:5,7,11,21,23|	Z8778277625	**1**{*163*}	Nc1ccc2ccccc2n1	**2**{*97*}	Fc1ccc(C=O)cc1	**3**{*27*}	CCCC[N+]#[C-]	26.7	30
217	**4**{*164*,*135*,*21*}	CN(C)CC1=CC2=NC(=C(NCC3CC3)N2C=C1)C1=C(C)N(C)N=C1C |c:18,21,26,t:4,6,8|	Z8798985065	**1**{*164*}	CN(C)Cc1ccnc(N)c1	**2**{*135*}	Cc1nn(C)c(C)c1C=O	**3**{*21*}	[C-]#[N+]CC1CC1	27.5	29
218	**4**{*153*,*136*,*22*}	COC1=CC(OC)=C(Br)C(C)=C1C1=C(NC2COC2)N2C(C=CC=C2C(C)(C)O)=N1 |c:10,13,23,25,31,t:2,6|	Z8810903033	**1**{*153*}	CC(C)(O)c1cccc(N)n1	**2**{*136*}	COc1cc(OC)c(C=O)c(C)c1Br	**3**{*22*}	[C-]#[N+]C1COC1	37.0	29
219	**4**{*93*,*79*,*14*}	COCCNC1=C(N=C2C=NC(OC)=CN12)C1=NC=CO1 |c:9,13,20,t:5,7,18|	Z8778278042	**1**{*93*}	COc1cnc(N)cn1	**2**{*79*}	O=Cc1ncco1	**3**{*14*}	COCC[N+]#[C-]	22.3	29
220	**4**{*165*,*40*,*17*}	OCC1=CSC(=N1)C1=C(NCC2CCOCC2)N2C=CC=C(C2=N1)C1=CC=CC=C1 |c:5,8,20,22,25,30,32,t:2,28|	Z8780122087	**1**{*165*}	Nc1ncccc1c2ccccc2	**2**{*40*}	OCc1csc(C=O)n1	**3**{*17*}	[C-]#[N+]CC1CCOCC1	32.2	28
221	**4**{*62*,*137*,*11*}	CC1=C(C(C)=NO1)C1=C(NCC2=CC(Br)=CC=C2)N2C=CC(CO)=CC2=N1 |c:4,8,15,17,21,25,28,t:1,12|	Z8810902983	**1**{*62*}	Nc1cc(CO)ccn1	**2**{*137*}	Cc1noc(C)c1C=O	**3**{*11*}	Brc1cccc(C[N+]#[C-])c1	32.4	28
222	**4**{*144*,*138*,*37*}	CNS(=O)(=O)C1=CN2C(C=C1)=NC(=C2NC1CC(C1)C(=O)OC)C1=NC(C)=NC(C)=C1 |c:9,11,13,29,32,t:5,26|	Z8810903016	**1**{*144*}	CNS(=O)(=O)c1ccc(N)nc1	**2**{*138*}	Cc1cc(C=O)nc(C)n1	**3**{*37*}	COC(=O)C1CC(C1)[N+]#[C-]	33.3	28
223	**4**{*164*,*139*,*24*}	COC1=CC(CNC2=C(N=C3C=C(CN(C)C)C=CN23)C2=C(C)N(CCO)N=C2C)=CC=C1 |c:17,22,29,32,34,t:2,7,9,11|	Z8810903038	**1**{*164*}	CN(C)Cc1ccnc(N)c1	**2**{*139*}	Cc1nn(CCO)c(C)c1C=O	**3**{*24*}	COc1cccc(C[N+]#[C-])c1	33.3	27

224	**4**{*31*,*140*,*22*}	COCCC(NC(=O)OC(C)(C)C)C1=C(NC2COC2)N2C3=C(OCC3)C=CC2=N1 |c:13,28,31,t:22|	Z8810903043	**1**{*31*}	Nc1ccc2OCCc2n1	**2**{*140*}	COCCC(NC(=O)OC(C)(C)C)C=O	**3**{*22*}	[C-]#[N+]C1COC1	30.8	27
225	**4**{*166*,*141*,*22*}	COC1=NC(Cl)=CC(=C1)C1=C(NC2COC2)N2C=CC(CSCCO)=CC2=N1 |c:5,7,10,19,26,29,t:2|	Z8801681806	**1**{*166*}	Nc1cc(CSCCO)ccn1	**2**{*141*}	COc1cc(C=O)cc(Cl)n1	**3**{*22*}	[C-]#[N+]C1COC1	31.0	27
226	**4**{*106*,*142*,*9*}	CNC1=C(N=C2C=CC(OC)=NN12)C1=C(CCC=C)C=CC=C1 |c:6,10,15,21,23,t:2,4|	Z8778278045	**1**{*106*}	COc1ccc(N)nn1	**2**{*142*}	C=CCCc1ccccc1C=O	**3**{*9*}	C[N+]#[C-]	22.4	27
227	**4**{*167*,*110*,*56*}	CC1=CN=C(C)C2=NC(=C(NC3=CC=C(OCC4=CC=CC=C4)C=C3)N12)C1=CN=C(C=C1)P(C)(C)=O |c:19,21,24,32,34,t:1,3,6,8,11,13,17,30|	Z8803896947	**1**{*167*}	Cc1cnc(C)c(N)n1	**2**{*110*}	CP(=O)(C)c1ccc(C=O)cn1	**3**{*56*}	[C-]#[N+]c1ccc(OCc2ccccc2)cc1	35.9	27
228	**4**{*168*,*14*,*17*}	CC1=CC=C(C=C1)C1=C(NCC2CCOCC2)N2C(C=NC=C2C#N)=N1 |c:3,5,8,21,23,27,t:1|	Z8776690324	**1**{*168*}	Nc1cncc(C#N)n1	**2**{*14*}	Cc1ccc(C=O)cc1	**3**{*17*}	[C-]#[N+]CC1CCOCC1	25.0	27
229	**4**{*169*,*143*,*13*}	CNC(=O)C1=NN2C(C=C1)=NC(=C2NCC1=CC=C(OC)C=C1)C1=CC(OC)=C(OC)C=C1 |c:8,10,12,23,34,t:4,17,19,26,30|	Z8810902954	**1**{*169*}	CNC(=O)c1ccc(N)nn1	**2**{*143*}	COc1ccc(C=O)cc1OC	**3**{*13*}	COc1ccc(C[N+]#[C-])cc1	32.1	27
230	**4**{*170*,*94*,*6*}	CCOC(=O)CCCNC1=C(N=C2N1C=CC(C)=C2Br)C1=NC=CN=C1 |c:11,15,18,24,26,t:9,22|	Z8780121700	**1**{*170*}	Cc1ccnc(N)c1Br	**2**{*94*}	O=Cc1cnccn1	**3**{*6*}	CCOC(=O)CCC[N+]#[C-]	29.9	26
231	**4**{*171*,*115*,*15*}	CCNC1=C(N=C2C=C(C(=CN12)N1C=CN=C1)C1=CC(OC)=NN1C |c:7,9,15,17,24,t:3,5,20|	Z8808559875	**1**{*171*}	Nc1cc(ccn1)n2ccnc2	**2**{*115*}	COc1cc(C=O)n(C)n1	**3**{*15*}	CC[N+]#[C-]	23.7	26
232	**4**{*121*,*144*,*10*}	BrC1=CC2=NC(C3=NOC(=C3)C3CC3)=C(NC3CCOC3)N2C=C1 |c:9,26,t:1,3,6,15|	Z8810902956	**1**{*121*}	Nc1cc(Br)ccn1	**2**{*144*}	O=Cc1cc(on1)C2CC2	**3**{*10*}	[C-]#[N+]C1CCOC1	26.8	26
233	**4**{*172*,*145*,*29*}	CCOC(=O)CCNC1=C(N=C2N1C=CC1=C2CCO1)C1=CN(C)C(=O)C2=C1N=CC=C2 |c:10,14,16,29,32,34,t:8,23|	Z8810902987	**1**{*172*}	Nc1nccc2OCCc21	**2**{*145*}	Cn1cc(C=O)c2ncccc2c1=O	**3**{*29*}	CCOC(=O)CC[N+]#[C-]	29.7	25
234	**4**{*32*,*146*,*7*}	C[C@@H]1C[C@@H]1CCC1=C(NC2CCOCC2)N2C=CC(=CC2=N1)P(C)(C)=O |&1:1,3,r,c:7,18,20,23|	Z8810903008	**1**{*32*}	CP(=O)(C)c1ccnc(N)c1	**2**{*146*}	C[C@@H]1C[C@@H]1CCC=O |&1:1,3,r|	**3**{*7*}	[C-]#[N+]C1CCOCC1	25.7	25
235	**4**{*173*,*147*,*57*}	COCCCCC1=C(NCC2=CC=CC=C2)N2C=C(C=C(Br)C2=N1)C(=O)N(C)C |c:6,12,14,18,24,t:10,20|	Z8808559883	**1**{*173*}	CN(C)C(=O)c1cnc(N)c(Br)c1	**2**{*147*}	COCCCCC=O	**3**{*57*}	[C-]#[N+]Cc1ccccc1	31.4	25

236	**4**{*174*,*14*,*17*}	CC1=CC=C(C=C1)C1=C(NCC2CCOCC2)N2C(C=CC=C2C#N)=N1 |c:3,5,8,21,23,27,t:1|	Z8776690319	**1**{*174*}	Nc1cccc(C#N)n1	**2**{*14*}	Cc1ccc(C=O)cc1	**3**{*17*}	[C-]#[N+]CC1CCOCC1	23.3	25
237	**4**{*175*,*148*,*24*}	COC1=C(C(C)=NN1C)C1=C(NCC2=CC(OC)=CC=C2)N2C=CC=C(OCCF)C2=N1 |c:5,10,18,20,24,33,t:2,14,26|	Z8803896932	**1**{*175*}	Nc1ncccc1OCCF	**2**{*148*}	COc1c(C=O)c(C)nn1C	**3**{*24*}	COc1cccc(C[N+]#[C-])c1	29.5	25
238	**4**{*176*,*29*,*6*}	CCOC(=O)CCCNC1=C(N=C2N1C=CN=C2N1CCCC1)C1=NN(CC)C=C1 |c:11,15,17,31,t:9,26|	Z8784060255	**1**{*176*}	Nc1nccnc1N2CCCC2	**2**{*29*}	CCn1ccc(C=O)n1	**3**{*6*}	CCOC(=O)CCC[N+]#[C-]	27.6	25
239	**4**{*141*,*149*,*22*}	COC1=C(OC)C(=CC(Br)=C1)C1=C(NC2COC2)N2C=CC(NC(C)=O)=CC2=N1 |c:2,6,9,12,21,27,30|	Z8808559892	**1**{*141*}	CC(=O)Nc1ccnc(N)c1	**2**{*149*}	COc1cc(Br)cc(C=O)c1OC	**3**{*22*}	[C-]#[N+]C1COC1	30.9	25
240	**4**{*177*,*69*,*17*}	CN1N=C(C=C1C)C1=C(NCC2CCOCC2)N2C=CC3=C(C=CC=N3)C2=N1 |c:2,4,8,20,24,26,30,t:22|	Z8778277872	**1**{*177*}	Nc1nccc2ncccc12	**2**{*69*}	Cc1cc(C=O)nn1C	**3**{*17*}	[C-]#[N+]CC1CCOCC1	25.2	25
241	**4**{*54*,*150*,*49*}	CN(C)C(=O)C1=CN2C(C=C1)=NC(C1CCC(CC1)OCC1=CC=CC=C1)=C2NC1CC(F)(F)C1 |c:9,11,25,27,29,t:5,23|	Z8801681834	**1**{*54*}	Cl.CN(C)C(=O)c1ccc(N)nc1	**2**{*150*}	O=CC1CCC(CC1)OCc2ccccc2	**3**{*49*}	FC1(F)CC(C1)[N+]#[C-]	32.3	25
242	**4**{*178*,*9*,*6*}	CCOC(=O)CCCNC1=C(N=C2N1C=C(C=C2C#C)C(F)(F)F)C1=CSC=C1 |c:11,15,17,29,t:9,26|	Z8780122088	**1**{*178*}	Nc1ncc(cc1C#C)C(F)(F)F	**2**{*9*}	O=Cc1ccsc1	**3**{*6*}	CCOC(=O)CCC[N+]#[C-]	28.1	25
243	**4**{*139*,*151*,*39*}	CCOCCNC1=C(N=C2C=C(C=CN12)C(F)F)C1=CC(C)=NN1 |c:10,12,23,t:6,8,20|	Z8781341768	**1**{*139*}	Nc1cc(ccn1)C(F)F	**2**{*151*}	Cc1cc(C=O)[nH]n1	**3**{*39*}	CCOCC[N+]#[C-]	22.1	24
244	**4**{*54*,*14*,*58*}	CN(C)C(=O)C1=CN2C(C=C1)=NC(=C2NC1(C)CC1)C1=CC=C(C)C=C1 |c:9,11,13,27,t:5,22,24|	Z8776690331	**1**{*54*}	Cl.CN(C)C(=O)c1ccc(N)nc1	**2**{*14*}	Cc1ccc(C=O)cc1	**3**{*58*}	CC1(CC1)[N+]#[C-]	22.7	24
245	**4**{*41*,*15*,*54*}	COC1=CC=C(C=C1)C1=C(NC2=CC=CC=C2)N2C=C(C)N=CC2=N1 |c:4,6,9,14,16,23,26,t:2,12,20|	Z8711872689	**1**{*41*}	Cc1cnc(N)cn1	**2**{*15*}	COc1ccc(C=O)cc1	**3**{*54*}	[C-]#[N+]c1ccccc1	21.4	24
246	**4**{*61*,*81*,*23*}	CCC1=CC=CC2=NC(=C(NC(CCC(=O)OC)C(=O)OC)N12)C1=NC(=CC=C1)C(F)F |c:4,27,29,t:2,6,8,25|	Z8810903044	**1**{*61*}	CCc1cccc(N)n1	**2**{*81*}	FC(F)c1cccc(C=O)n1	**3**{*23*}	COC(=O)CCC([N+]#[C-])C(=O)OC	28.7	24
247	**4**{*179*,*152*,*23*}	COC(=O)CCC(C)C1=C(NC(CCC(=O)OC)C(=O)OC)N2C=CC(OC)=CC2=N1 |c:8,23,27,30|	Z8801681822	**1**{*179*}	COc1ccnc(N)c1	**2**{*152*}	COC(=O)CCC(C)C=O	**3**{*23*}	COC(=O)CCC([N+]#[C-])C(=O)OC	27.6	23
248	**4**{*158*,*153*,*18*}	CC(C)(C)NC1=C(N=C2C=NC(=CN12)P(C)(C)=O)C1=CN(CC(F)(F)F)N=N1 |c:9,11,28,t:5,7,20|	Z8803896925	**1**{*158*}	CP(=O)(C)c1cnc(N)cn1	**2**{*153*}	FC(F)(F)Cn1cc(C=O)nn1	**3**{*18*}	CC(C)(C)[N+]#[C-]	26.1	23

<table>
<tr><td>249</td><td>4{73,15,9}</td><td>CNC1=C(N=C2C=CC3=NC=CN3N12)C1=CC=C(OC)C=C1 |c:6,10,23,t:2,4,8,17,19|</td><td>Z8776690333</td><td>1{73}</td><td>Nc1ccc2nccn2n1</td><td>2{15}</td><td>COc1ccc(C=O)cc1</td><td>3{9}</td><td>C[N+]#[C-]</td><td>18.1</td><td>23</td></tr>
<tr><td>250</td><td>4{167,154,29}</td><td>CCOC(=O)CCNC1=C(N=C2N1C(C)=CN=C2C)C1N(CC11CCOCC1)C(=O)OC(C)(C)C |c:10,15,17,t:8|</td><td>Z8810902967</td><td>1{167}</td><td>Cc1cnc(C)c(N)n1</td><td>2{154}</td><td>CC(C)(C)OC(=O)N1CC2(CCOCC2)C1C=O</td><td>3{29}</td><td>CCOC(=O)CC[N+]#[C-]</td><td>30.0</td><td>23</td></tr>
<tr><td>251</td><td>4{180,155,53}</td><td>COC(=O)C1=CC2=NC(=C(NCCOCC3=CC=CC=C3)N2C=C1C)C1=CN=CN=C1 |c:17,19,24,30,32,t:4,6,8,15,28|</td><td>Z8803896942</td><td>1{180}</td><td>COC(=O)c1cc(N)ncc1C</td><td>2{155}</td><td>O=Cc1cncnc1</td><td>3{53}</td><td>[C-]#[N+]CCOCc1ccccc1</td><td>25.1</td><td>22</td></tr>
<tr><td>252</td><td>4{47,137,6}</td><td>CCOC(=O)CCCNC1=C(N=C2C=C(C=CN12)S(N)(=O)=O)C1=C(C)ON=C1C |c:13,15,24,28,t:9,11|</td><td>Z8803896968</td><td>1{47}</td><td>Cl.Nc1cc(ccn1)S(=O)(=O)N</td><td>2{137}</td><td>Cc1noc(C)c1C=O</td><td>3{6}</td><td>CCOC(=O)CCC[N+]#[C-]</td><td>25.0</td><td>22</td></tr>
<tr><td>253</td><td>4{181,77,6}</td><td>CCOC(=O)CCCNC1=C(CC2CCCC2)N=C2N1C=CC1=C2N=CC=N1 |c:9,18,22,24,27,29|</td><td>Z8784060825</td><td>1{181}</td><td>Nc1nccc2nccnc12</td><td>2{77}</td><td>O=CCC1CCCC1</td><td>3{6}</td><td>CCOC(=O)CCC[N+]#[C-]</td><td>22.6</td><td>22</td></tr>
<tr><td>254</td><td>4{182,14,7}</td><td>CC1=CC=C(C=C1)C1=C(NC2CCOCC2)N2C=C(C=C(I)C2=N1)C(F)(F)F |c:3,5,8,19,25,t:1,21|</td><td>Z8797786219</td><td>1{182}</td><td>Nc1ncc(cc1I)C(F)(F)F</td><td>2{14}</td><td>Cc1ccc(C=O)cc1</td><td>3{7}</td><td>[C-]#[N+]C1CCOCC1</td><td>29.7</td><td>22</td></tr>
<tr><td>255</td><td>4{183,156,10}</td><td>COC1=CC=C(C)N2C(NC3CCOC3)=C(N=C12)C1=C(Cl)N=CN1C |c:15,21,24,t:2,4,17|</td><td>Z8801681815</td><td>1{183}</td><td>COc1ccc(C)nc1N</td><td>2{156}</td><td>Cn1cnc(Cl)c1C=O</td><td>3{10}</td><td>[C-]#[N+]C1CCOC1</td><td>21.0</td><td>21</td></tr>
<tr><td>256</td><td>4{184,22,7}</td><td>NC(=S)C1=CN2C(C=C1)=NC(=C2NC1CCOCC1)C1=CC=CC=C1 |c:7,9,11,24,26,t:3,22|</td><td>Z8784059422</td><td>1{184}</td><td>NC(=S)c1ccc(N)nc1</td><td>2{22}</td><td>O=Cc1ccccc1</td><td>3{7}</td><td>[C-]#[N+]C1CCOCC1</td><td>20.1</td><td>21</td></tr>
<tr><td>257</td><td>4{185,26,7}</td><td>CC1=C(C=CC2=NC(CCC3=CC=CC=C3)=C(NC3CCOCC3)N12)S(C)(=O)=O |c:3,12,14,t:1,5,10,16|</td><td>Z8776690329</td><td>1{185}</td><td>Cc1nc(N)ccc1S(=O)(=O)C</td><td>2{26}</td><td>O=CCCc1ccccc1</td><td>3{7}</td><td>[C-]#[N+]C1CCOCC1</td><td>23.5</td><td>21</td></tr>
<tr><td>258</td><td>4{46,157,9}</td><td>CNC1=C(N=C2C=CC=CN12)C1=NC2=C(C=C1)N=CC=C2 |c:6,8,15,17,20,22,t:2,4,13|</td><td>Z8798985061</td><td>1{46}</td><td>Nc1ccccn1</td><td>2{157}</td><td>O=Cc1ccc2ncccc2n1</td><td>3{9}</td><td>C[N+]#[C-]</td><td>15.5</td><td>21</td></tr>
<tr><td>259</td><td>4{161,14,26}</td><td>CC1=CC=C(C=C1)C1=C(NCC2=CC=C(F)C=C2)N2N=CC=CC2=N1 |c:3,5,8,17,21,23,26,t:1,12,14|</td><td>Z8741907011</td><td>1{161}</td><td>Nc1cccnn1</td><td>2{14}</td><td>Cc1ccc(C=O)cc1</td><td>3{26}</td><td>Fc1ccc(C[N+]#[C-])cc1</td><td>18.6</td><td>21</td></tr>
<tr><td>260</td><td>4{28,158,31}</td><td>CSCCNC1=C(N=C2C=C(C=CN12)C1=NOC=N1)C1=CN(C=C)N=C1 |c:9,11,19,27,t:5,7,16,22|</td><td>Z8798985032</td><td>1{28}</td><td>Cl.Nc1cc(ccn1)c2ncon2</td><td>2{158}</td><td>C=Cn1cc(C=O)cn1</td><td>3{31}</td><td>CSCC[N+]#[C-]</td><td>20.0</td><td>20</td></tr>
</table>

261	**4**{*19,22,7*}	N#CC1=CC=CN2C(NC3CCOCC3)=C(N=C12)C1=CC=CC=C1 |c:4,15,23,25,t:2,17,21|	Z8780121582	**1**{*19*}	Nc1ncccc1C#N	**2**{*22*}	O=Cc1ccccc1	**3**{*7*}	[C-]#[N+]C1CCOCC1	17.2	20
262	**4**{*32,58,16*}	CC1=CC=CC(C)=C1NC1=C(N=C2C=C(C=CN12)P(C)(C)=O)C1=NSN=C1 |c:3,6,14,16,28,t:1,10,12,25|	Z8781341783	**1**{*32*}	CP(=O)(C)c1ccnc(N)c1	**2**{*58*}	O=Cc1cnsn1	**3**{*16*}	Cc1cccc(C)c1[N+]#[C-]	21.4	20
263	**4**{*38,129,9*}	CNC1=C(N=C2C=C(C=CN12)C#N)C1=CC=CC2=NC=NN12 |c:6,8,17,21,t:2,4,15,19|	Z8798985023	**1**{*38*}	Nc1cc(C#N)ccn1	**2**{*129*}	O=Cc1cccc2ncnn12	**3**{*9*}	C[N+]#[C-]	15.5	20
264	**4**{*186,159,1*}	CN(C)S(=O)(=O)C1=CC2=NC(=C(NC3CC3)N2C=C1)C1=NNC2=C1C=CC(F)=C2 |c:19,25,28,31,t:6,8,10,22|	Z8810903045	**1**{*186*}	Cl.CN(C)S(=O)(=O)c1ccnc(N)c1	**2**{*159*}	Fc1ccc2c(C=O)n[nH]c2c1	**3**{*1*}	[C-]#[N+]C1CC1	22.0	20
265	**4**{*123,160,49*}	CN1N=C(C=C1C1=C(NC2CC(F)(F)C2)N2C=CC(OCCO)=CC2=N1)C1CC1 |c:2,4,7,18,24,27|	Z8803896943	**1**{*123*}	Cl.Nc1cc(OCCO)ccn1	**2**{*160*}	Cn1nc(cc1C=O)C2CC2	**3**{*49*}	FC1(F)CC(C1)[N+]#[C-]	21.0	19
266	**4**{*97,161,14*}	CCN1C=NC=C1C1=C(NCCOC)N2C=C(F)C=C(C)C2=N1 |c:3,5,8,23,t:16,19|	Z8778278036	**1**{*97*}	Cc1cc(F)cnc1N	**2**{*161*}	CCn1cncc1C=O	**3**{*14*}	COCC[N+]#[C-]	16.4	19
267	**4**{*46,162,9*}	CNC1=C(N=C2C=CC=CN12)C1=CC(OC)=NC(C)=C1 |c:6,8,17,20,t:2,4,13|	Z8810902981	**1**{*46*}	Nc1ccccn1	**2**{*162*}	COc1cc(C=O)cc(C)n1	**3**{*9*}	C[N+]#[C-]	13.8	19
268	**4**{*70,163,22*}	FC1=C(C2=C(NC3COC3)N3C=CN=C(C4CC4)C3=N2)C(I)=CN=C1 |c:1,3,12,21,25,27,t:14|	Z8801681849	**1**{*70*}	Nc1nccnc1C2CC2	**2**{*163*}	Fc1cncc(I)c1C=O	**3**{*22*}	[C-]#[N+]C1COC1	23.2	19
269	**4**{*187,164,46*}	CN1N=C(C2=C1CCOC2)C1=C(NCC(C)=C)N2C=C(Cl)C(=CC2=N1)C#N |c:2,4,12,23,26,t:20|	Z8810903034	**1**{*187*}	Nc1cc(C#N)c(Cl)cn1	**2**{*164*}	Cn1nc(C=O)c2COCCc21	**3**{*46*}	CC(=C)C[N+]#[C-]	19.6	19
270	**4**{*188,165,59*}	COC(=O)[C@H]1C[C@@H](C1)N1C=CC(=N1)C1=C(NCCC2=CC(F)=CC=C2)N2C(C=NC=C2C(=O)OC)=N1 |r,c:10,12,15,23,25,30,32,38,t:20|	Z8810902960	**1**{*188*}	COC(=O)c1cncc(N)n1	**2**{*165*}	COC(=O)[C@H]1C[C@@H](C1)n2ccc(C=O)n2	**3**{*59*}	Fc1cccc(CC[N+]#[C-])c1	25.2	19
271	**4**{*189,44,27*}	CCCCNC1=C(N=C2N1C=CC=C2N(C)C)C1=CN(CC)N=C1 |c:7,11,13,24,t:5,19|	Z8797781359	**1**{*189*}	CN(C)c1cccnc1N	**2**{*44*}	CCn1cc(C=O)cn1	**3**{*27*}	CCCC[N+]#[C-]	16.7	19
272	**4**{*190,166,12*}	C(NC1=C(N=C2C=C3C=CC=CC3=NN12)C1=NC(=CS1)C1CC1)C1CCOC1 |c:8,10,13,20,t:2,4,6,18|	Z8786958851	**1**{*190*}	Nc1cc2ccccc2nn1	**2**{*166*}	O=Cc1nc(cs1)C2CC2	**3**{*12*}	[C-]#[N+]CC1CCOC1	19.7	19
273	**4**{*186,167,29*}	CCOC(=O)CCNC1=C(N=C2C=C(C=CN12)S(=O)(=O)N(C)C)C1=NN=CN1 |c:12,14,27,t:8,10,25|	Z8808559859	**1**{*186*}	Cl.CN(C)S(=O)(=O)c1ccnc(N)c1	**2**{*167*}	O=Cc1nnc[nH]1	**3**{*29*}	CCOC(=O)CC[N+]#[C-]	20.4	19
274	**4**{*118,46,25*}	COCCCCCCC1=C(NCCCF)N2C(C=CC=C2C(C)O)=N1 |c:7,16,18,23|	Z8781341799	**1**{*118*}	Cl.CC(O)c1cccc(N)n1	**2**{*46*}	COCCCCCCC=O	**3**{*25*}	FCCC[N+]#[C-]	16.7	18

275	**4**{*191*,*168*,*22*}	CC(C)(C)C1=CN2C(NC3COC3)=C(N=C2C=C1)C1=NOC=C1 |c:13,15,18,24,t:4,21|	Z8810902975	**1**{*191*}	CC(C)(C)c1ccc(N)nc1	**2**{*168*}	O=Cc1ccon1	**3**{*22*}	[C-]#[N+]C1COC1	15.3	18
276	**4**{*114*,*40*,*25*}	COCC1=CC2=NC(=C(NCCCF)N2C=C1)C1=NC(CO)=CS1 |c:16,23,t:3,5,7,19|	Z8781341790	**1**{*114*}	COCc1ccnc(N)c1	**2**{*40*}	OCc1csc(C=O)n1	**3**{*25*}	FCCC[N+]#[C-]	16.5	17
277	**4**{*157*,*14*,*54*}	CC1=CC=C(C=C1)C1=C(NC2=CC=CC=C2)N2C(C=C(C)C=C2Br)=N1 |c:3,5,8,13,15,23,26,t:1,11,20|	Z8741907090	**1**{*157*}	Cc1cc(N)nc(Br)c1	**2**{*14*}	Cc1ccc(C=O)cc1	**3**{*54*}	[C-]#[N+]c1ccccc1	18.3	17
278	**4**{*192*,*22*,*27*}	CCCCNC1=C(N=C2N1C(F)=CC=C2Cl)C1=CC=CC=C1 |c:7,12,14,20,22,t:5,18|	Z8786838535	**1**{*192*}	Nc1nc(F)ccc1Cl	**2**{*22*}	O=Cc1ccccc1	**3**{*27*}	CCCC[N+]#[C-]	14.4	17
279	**4**{*22*,*19*,*13*}	COC1=C(N(C)N=C1)C1=C(NCC2=CC=C(OC)C=C2)N2N=C(Cl)C=CC2=N1 |c:6,9,19,26,29,t:2,13,15,23|	Z8798985042	**1**{*22*}	Nc1ccc(Cl)nn1	**2**{*19*}	COc1cnn(C)c1C=O	**3**{*13*}	COc1ccc(C[N+]#[C-])cc1	18.0	17
280	**4**{*193*,*169*,*12*}	CNC(=O)C1=CN2C(C=C1)=NC(=C2NCC1COC1)C1=NNC2=C1N=CC=C2 |c:8,10,12,26,29,31,t:4,23|	Z8803896944	**1**{*193*}	CNC(=O)c1ccc(N)nc1	**2**{*169*}	O=Cc1n[nH]c2cccnc12	**3**{*12*}	[C-]#[N+]CC1COC1	16.3	15
281	**4**{*73*,*151*,*13*}	COC1=CC=C(CNC2=C(N=C3C=CC4=NC=CN4N23)C2=CC(C)=NN2)C=C1 |c:12,16,26,30,t:2,4,8,10,14,23|	Z8810902952	**1**{*73*}	Nc1ccc2nccn2n1	**2**{*151*}	Cc1cc(C=O)[nH]n1	**3**{*13*}	COc1ccc(C[N+]#[C-])cc1	15.4	15
282	**4**{*169*,*15*,*13*}	CNC(=O)C1=NN2C(C=C1)=NC(=C2NCC1=CC=C(OC)C=C1)C1=CC=C(OC)C=C1 |c:8,10,12,23,32,t:4,17,19,26,28|	Z8776690339	**1**{*169*}	CNC(=O)c1ccc(N)nn1	**2**{*15*}	COc1ccc(C=O)cc1	**3**{*13*}	COc1ccc(C[N+]#[C-])cc1	16.9	15
283	**4**{*194*,*170*,*7*}	CCN1N=NC(=N1)C1=C(NC2CCOCC2)N2C=CN=C(N3C=CC=N3)C2=N1 |c:3,5,8,19,24,26,30,t:21|	Z8780122086	**1**{*194*}	Nc1nccnc1n2cccn2	**2**{*170*}	CCn1nnc(C=O)n1	**3**{*7*}	[C-]#[N+]C1CCOCC1	15.1	15
284	**4**{*73*,*19*,*13*}	COC1=C(N(C)N=C1)C1=C(NCC2=CC=C(OC)C=C2)N2N3C=CN=C3C=CC2=N1 |c:6,9,19,24,26,29,32,t:2,13,15|	Z8798985040	**1**{*73*}	Nc1ccc2nccn2n1	**2**{*19*}	COc1cnn(C)c1C=O	**3**{*13*}	COc1ccc(C[N+]#[C-])cc1	15.8	15
285	**4**{*195*,*118*,*7*}	CC1=NC=CC(=C1)C1=C(NC2CCOCC2)N2C=C(Br)C=C(NS(C)(=O)=O)C2=N1 |c:3,5,8,30,t:1,19,22|	Z8780122346	**1**{*195*}	CS(=O)(=O)Nc1cc(Br)cnc1N	**2**{*118*}	Cc1cc(C=O)ccn1	**3**{*7*}	[C-]#[N+]C1CCOCC1	18.5	14
286	**4**{*196*,*171*,*43*}	COC1CC(C(OC)O1)C1=C(NC2CCN(CC2)C(=O)OC(C)(C)C)N2C=C(C)C=C(C#N)C2=N1 |c:10,36,t:28,31|	Z8808559881	**1**{*196*}	Cc1cnc(N)c(C#N)c1	**2**{*171*}	COC1CC(C=O)C(OC)O1	**3**{*43*}	CC(C)(C)OC(=O)N1CCC(CC1)[N+]#[C-]	18.5	14
287	**4**{*73*,*172*,*11*}	BrC1=CC(CNC2=C(N=C3C=CC4=NC=CN4N23)C2=CNC(=N2)C2CC2)=CC=C1 |c:10,14,24,30,32,t:1,6,8,12,21|	Z8808559861	**1**{*73*}	Nc1ccc2nccn2n1	**2**{*172*}	O=Cc1c[nH]c(n1)C2CC2	**3**{*11*}	Brc1cccc(C[N+]#[C-])c1	16.9	14

288	**4**{*197*,*29*,*45*}	CCN1C=CC(=N1)C1=C(NCC=C)N2C=C(C=CC2=N1)S(=O)(=O)CC(C)C |c:3,5,8,15,17,20|	Z8786958927	**1**{*197*}	CC(C)CS(=O)(=O)c1ccc(N)nc1	**2**{*29*}	CCn1ccc(C=O)n1	**3**{*45*}	C=CC[N+]#[C-]	14.2	14
289	**4**{*180*,*173*,*15*}	CCNC1=C(N=C2C=C(C(=O)OC)C(C)=CN12)C1=NC(=NN1C)C(F)(F)F |c:14,21,t:3,5,7,19|	Z8810902977	**1**{*180*}	COC(=O)c1cc(N)ncc1C	**2**{*173*}	Cn1nc(nc1C=O)C(F)(F)F	**3**{*15*}	CC[N+]#[C-]	13.5	13
290	**4**{*198*,*174*,*31*}	CSCCNC1=C(N=C2C=NC(C#C)=C(Cl)N12)C1=CN(CC2CCCO2)N=N1 |c:9,29,t:5,7,13,19|	Z8801681832	**1**{*198*}	Nc1cnc(C#C)c(Cl)n1	**2**{*174*}	O=Cc1cn(CC2CCCO2)nn1	**3**{*31*}	CSCC[N+]#[C-]	14.3	13
291	**4**{*113*,*170*,*7*}	CCN1N=NC(=N1)C1=C(NC2CCOCC2)N2C=CC=C(N3CCOCC3)C2=N1 |c:3,5,8,19,31,t:21|	Z8784060348	**1**{*113*}	Nc1ncccc1N2CCOCC2	**2**{*170*}	CCn1nnc(C=O)n1	**3**{*7*}	[C-]#[N+]C1CCOCC1	13.0	12
292	**4**{*79*,*109*,*9*}	CNC1=C(N=C2C=NC=C(C)N12)C1=NN(C)C=C1C |c:6,18,t:2,4,8,14|	Z8803896951	**1**{*79*}	Cc1cncc(N)n1	**2**{*109*}	Cc1cn(C)nc1C=O	**3**{*9*}	C[N+]#[C-]	8.4	12
293	**4**{*194*,*175*,*60*}	COC(=O)C1=CC(NC2=C(N=C3N2C=CN=C3N2C=CC=N2)C2=NNC=C2)=CC=C1 |c:10,14,16,20,22,28,30,32,t:4,8,25|	Z8803896952	**1**{*194*}	Nc1nccnc1n2cccn2	**2**{*175*}	O=Cc1cc[nH]n1	**3**{*60*}	COC(=O)c1cccc([N+]#[C-])c1	12.9	12
294	**4**{*104*,*176*,*12*}	COC(=O)C1=C(Cl)SC(=C1)C1=C(NCC2CCOC2)N2C(C=CC3=C2COCC3)=N1 |c:4,8,11,23,25,32|	Z8810902978	**1**{*104*}	Nc1ccc2CCOCc2n1	**2**{*176*}	COC(=O)c1cc(C=O)sc1Cl	**3**{*12*}	[C-]#[N+]CC1COC1	13.6	11
295	**4**{*199*,*77*,*6*}	CCOC(=O)CCCNC1=C(CC2CCCC2)N=C2N1C=CC=C2C(C)(F)F |c:9,18,22,24|	Z8797784767	**1**{*199*}	CC(F)(F)c1cccnc1N	**2**{*77*}	O=CCC1CCCC1	**3**{*6*}	CCOC(=O)CCC[N+]#[C-]	12.0	11
296	**4**{*200*,*15*,*9*}	CNC1=C(N=C2C=C(C)C=C(OC)N12)C1=CC=C(OC)C=C1 |c:22,t:2,4,6,9,16,18|	Z8776690326	**1**{*200*}	Cl.COc1cc(C)cc(N)n1	**2**{*15*}	COc1ccc(C=O)cc1	**3**{*9*}	C[N+]#[C-]	9.0	11
297	**4**{*201*,*108*,*5*}	COCCCNC1=C(N=C2C=C(C=CN12)C(F)(F)F)C1=CN=C(N=C1)N1CCOCC1 |c:10,12,23,25,t:6,8,21|	Z8803896939	**1**{*201*}	Nc1cc(ccn1)C(F)(F)F	**2**{*108*}	O=Cc1cnc(nc1)N2CCOCC2	**3**{*5*}	COCCC[N+]#[C-]	12.9	11
298	**4**{*161*,*98*,*6*}	CCOC(=O)CCCNC1=C(N=C2C=CC=NN12)C1=NC=C(OC)N=C1 |c:13,15,26,t:9,11,20,22|	Z8801681809	**1**{*161*}	Nc1cccnn1	**2**{*98*}	COc1cnc(C=O)cn1	**3**{*6*}	CCOC(=O)CCC[N+]#[C-]	10.3	11
299	**4**{*135*,*130*,*32*}	CCCC1=NC=CN2C(NC3CCC3)=C(N=C12)C1=NN=C(OC)C=C1 |c:5,14,26,t:3,16,20,22|	Z8801681840	**1**{*135*}	CCCc1nccnc1N	**2**{*130*}	COc1ccc(C=O)nn1	**3**{*32*}	[C-]#[N+]C1CCC1	9.8	11
300	**4**{*179*,*177*,*5*}	COCCCNC1=C(CC2CC(C2)C(=O)OC)N=C2C=C(OC)C=CN12 |c:6,24,t:18,20|	Z8808559865	**1**{*179*}	COc1ccnc(N)c1	**2**{*177*}	COC(=O)C1CC(CC=O)C1	**3**{*5*}	COCCC[N+]#[C-]	10.3	11

301	**4**{*202*,*69*,*14*}	COCCNC1=C(N=C2N1C=C(Br)C=C2C(C)(C)C)C1=NN(C)C(C)=C1 |c:7,14,26,t:5,11,21|	Z8780122084	**1**{*202*}	CC(C)(C)c1cc(Br)cnc1N	**2**{*69*}	Cc1cc(C=O)nn1C	**3**{*14*}	COCC[N+]#[C-]	11.8	10
302	**4**{*123*,*178*,*22*}	COC1=CC2=C(OC(=C2)C2=C(NC3COC3)N3C=CC(OCCO)=CC3=N2)C=C1 |c:7,10,19,25,28,31,t:2,4|	Z8810903015	**1**{*123*}	Cl.Nc1cc(OCCO)ccn1	**2**{*178*}	COc1ccc2oc(C=O)cc2c1	**3**{*22*}	[C-]#[N+]C1COC1	10.9	10
303	**4**{*101*,*179*,*14*}	COCCNC1=C(N=C2N1C=C(CO)C=C2Cl)C1=CC=C(OC2CCOC2)C=C1 |c:7,15,30,t:5,11,19,21|	Z8801681830	**1**{*101*}	Nc1ncc(CO)cc1Cl	**2**{*179*}	O=Cc1ccc(OC2CCOC2)cc1	**3**{*14*}	COCC[N+]#[C-]	11.5	10
304	**4**{*78*,*180*,*2*}	CC1=CN2N3C(C=C(C)C2=N1)=NC(CCCCCF)=C3NC1=CC=C(F)C=C1 |c:10,12,20,29,t:1,6,24,26|	Z8808559869	**1**{*78*}	Cc1cn2nc(N)cc(C)c2n1	**2**{*180*}	FCCCCCC=O	**3**{*2*}	Fc1ccc([N+]#[C-])cc1	10.0	10
305	**4**{*203*,*22*,*14*}	COCCNC1=C(N=C2N1C1=C(C=CC=C1)N=C2N1CCCC1)C1=CC=CC=C1 |c:7,13,15,18,29,31,t:5,11,27|	Z8797783649	**1**{*203*}	Nc1nc2ccccc2nc1N3CCCC3	**2**{*22*}	O=Cc1ccccc1	**3**{*14*}	COCC[N+]#[C-]	9.8	9
306	**4**{*73*,*181*,*13*}	COC1=CC=C(CNC2=C(N=C3C=CC4=NC=CN4N23)C2=CN=C3CCCN23)C=C1 |c:12,16,33,t:2,4,8,10,14,23,25|	Z8801681846	**1**{*73*}	Nc1ccc2nccn2n1	**2**{*181*}	O=Cc1cnc2CCCn12	**3**{*13*}	COc1ccc(C[N+]#[C-])cc1	9.7	9
307	**4**{*73*,*19*,*58*}	COC1=C(N(C)N=C1)C1=C(NC2(C)CC2)N2N3C=CN=C3C=CC2=N1 |c:6,9,19,21,24,27,t:2|	Z8781341775	**1**{*73*}	Nc1ccc2nccn2n1	**2**{*19*}	COc1cnn(C)c1C=O	**3**{*58*}	CC1(CC1)[N+]#[C-]	8.0	9
308	**4**{*204*,*14*,*27*}	CCCCNC1=C(N=C2N1C=CC(Cl)=C2I)C1=CC=C(C)C=C1 |c:7,11,14,23,t:5,18,20|	Z8786897242	**1**{*204*}	Nc1nccc(Cl)c1I	**2**{*14*}	Cc1ccc(C=O)cc1	**3**{*27*}	CCCC[N+]#[C-]	10.1	8
309	**4**{*145*,*182*,*37*}	COC(=O)C1CC(C1)NC1=C(N=C2C=CC=C(N12)S(C)(=O)=O)C1=NC=CN1CC#C |c:14,16,27,t:10,12,25|	Z8781341787	**1**{*145*}	CS(=O)(=O)c1cccc(N)n1	**2**{*182*}	O=Cc1nccn1CC#C	**3**{*37*}	COC(=O)C1CC(C1)[N+]#[C-]	9.3	8
310	**4**{*169*,*49*,*13*}	CNC(=O)C1=NN2C(NCC3=CC=C(OC)C=C3)=C(N=C2C=C1)C1=CNN=C1C |c:16,18,20,23,29,t:4,10,12,26|	Z8808559891	**1**{*169*}	CNC(=O)c1ccc(N)nn1	**2**{*49*}	Cc1n[nH]cc1C=O	**3**{*13*}	COc1ccc(C[N+]#[C-])cc1	8.0	8
311	**4**{*205*,*9*,*27*}	CCCCNC1=C(N=C2N1C=CN=C2C#C)C1=CSC=C1 |c:7,11,13,21,t:5,18|	Z8778277313	**1**{*205*}	Nc1nccnc1C#C	**2**{*9*}	O=Cc1ccsc1	**3**{*27*}	CCCC[N+]#[C-]	5.8	7
312	**4**{*124*,*183*,*12*}	COC(=O)C(C)OC1=CC=C(C=C1)C1=C(NCC2CCOC2)N2C=CC=C(CO)C2=N1 |c:9,11,14,25,32,t:7,27|	Z8798985043	**1**{*124*}	Nc1ncccc1CO	**2**{*183*}	COC(=O)C(C)Oc1ccc(C=O)cc1	**3**{*12*}	[C-]#[N+]CC1COC1	8.2	7
313	**4**{*206*,*80*,*6*}	CCOC(=O)CCCNC1=C(N=C2N1C=CC(I)=C2F)C1=NC=C(C)S1 |c:11,15,18,t:9,22,24|	Z8786849399	**1**{*206*}	Nc1nccc(I)c1F	**2**{*80*}	Cc1cnc(C=O)s1	**3**{*6*}	CCOC(=O)CCC[N+]#[C-]	9.4	7

314	**4**{*106*,*22*,*54*}	COC1=NN2C(C=C1)=NC(=C2NC1=CC=CC=C1)C1=CC=CC=C1 |c:6,8,10,16,18,23,25,t:2,14,21|	Z8741907033	**1**{*106*}	COc1ccc(N)nn1	**2**{*22*}	O=Cc1ccccc1	**3**{*54*}	[C-]#[N+]c1ccccc1	6.0	7
315	**4**{*207*,*184*,*14*}	COCCNC1=C(N=C2C=CC(=CN12)N1CCN(CC1)C(=O)C1CC1)C1CC1 |c:9,11,t:5,7|	Z8780122426	**1**{*207*}	Nc1ccc(cn1)N2CCN(CC2)C(=O)C3CC3	**2**{*184*}	O=CC1CC1	**3**{*14*}	COCC[N+]#[C-]	7.1	7
316	**4**{*137*,*58*,*13*}	COC1=CC=C(CNC2=C(N=C3C=CC(C)=NN23)C2=NSN=C2)C=C1 |c:12,15,23,26,t:2,4,8,10,20|	Z8798985035	**1**{*137*}	Cc1ccc(N)nn1	**2**{*58*}	O=Cc1cnsn1	**3**{*13*}	COc1ccc(C[N+]#[C-])cc1	6.4	7
317	**4**{*208*,*94*,*6*}	CCOC(=O)CCCNC1=C(N=C2N1C=CN=C2OC1CCCC1)C1=NC=CN=C1 |c:11,15,17,29,31,t:9,27|	Z8778277288	**1**{*208*}	Nc1nccnc1OC2CCCC2	**2**{*94*}	O=Cc1cnccn1	**3**{*6*}	CCOC(=O)CCC[N+]#[C-]	7.1	6
318	**4**{*209*,*40*,*17*}	OCC1=CSC(=N1)C1=C(NCC2CCOCC2)N2C=CC=C(N3CCCCC3)C2=N1 |c:5,8,20,32,t:2,22|	Z8784060440	**1**{*209*}	Nc1ncccc1N2CCCCC2	**2**{*40*}	OCc1csc(C=O)n1	**3**{*17*}	[C-]#[N+]CC1CCOCC1	7.3	6
319	**4**{*161*,*15*,*13*}	COC1=CC=C(CNC2=C(N=C3C=CC=NN23)C2=CC=C(OC)C=C2)C=C1 |c:12,14,25,28,t:2,4,8,10,19,21|	Z8776690334	**1**{*161*}	Nc1cccnn1	**2**{*15*}	COc1ccc(C=O)cc1	**3**{*13*}	COc1ccc(C[N+]#[C-])cc1	6.2	6
320	**4**{*210*,*185*,*34*}	CN1C=NC(=C1)C1=C(NCC2CCCO2)N2C(=N1)C(Br)=CC=C2F |c:2,4,7,18,22,24|	Z8810903013	**1**{*210*}	Nc1nc(F)ccc1Br	**2**{*185*}	Cn1cnc(C=O)c1	**3**{*34*}	[C-]#[N+]CC1CCO1	6.6	6
321	**4**{*169*,*53*,*13*}	CNC(=O)C1=NN2C(C=C1)=NC(=C2NCC1=CC=C(OC)C=C1)C1=C(F)C=CC(C)=N1 |c:8,10,12,23,26,29,32,t:4,17,19|	Z8803896966	**1**{*169*}	CNC(=O)c1ccc(N)nn1	**2**{*53*}	Cc1ccc(F)c(C=O)n1	**3**{*13*}	COc1ccc(C[N+]#[C-])cc1	7.0	6
322	**4**{*211*,*59*,*34*}	COC1=C(Br)C=C(C=N1)C1=C(NCC2CCCO2)N2C=CC=C(C3=NN=NN3)C2=N1 |c:2,5,7,10,21,27,32,t:23,25|	Z8808559885	**1**{*211*}	Nc1ncccc1c2nnn[nH]2	**2**{*59*}	COc1ncc(C=O)cc1Br	**3**{*34*}	[C-]#[N+]CC1CCO1	7.7	6
323	**4**{*104*,*186*,*12*}	FC1=C2SC(=NC2=CC=C1)C1=C(NCC2CCOC2)N2C(C=CC3=C2COCC3)=N1 |c:1,4,7,9,12,24,26,33|	Z8810902982	**1**{*104*}	Nc1ccc2CCOCc2n1	**2**{*186*}	Fc1cccc2nc(C=O)sc12	**3**{*12*}	[C-]#[N+]CC1COC1	7.0	6
324	**4**{*212*,*187*,*49*}	CCOC(=O)C1=NC(=C2C=CC=CN12)C1=C(NC2CC(F)(F)C2)N2C=C(COC)C=CC2=N1 |c:9,11,16,32,35,t:5,7,27|	Z8803896960	**1**{*212*}	Cl.COCc1ccc(N)nc1	**2**{*187*}	CCOC(=O)c1nc(C=O)c2ccccn12	**3**{*49*}	FC1(F)CC(C1)[N+]#[C-]	7.2	6
325	**4**{*213*,*15*,*9*}	CNC1=C(N=C2N1C(OC)=CC=C2F)C1=CC=C(OC)C=C1 |c:4,10,12,22,t:2,16,18|	Z8776690311	**1**{*213*}	COc1ccc(F)c(N)n1	**2**{*15*}	COc1ccc(C=O)cc1	**3**{*9*}	C[N+]#[C-]	4.5	5

326	**4**{*214*,*188*,*12*}	COC1=CC(Br)=C(C)N2C(NCC3CCOC3)=C(CCC3(CC3)C#N)N=C12 |t:2,5,17,27|	Z8798985054	**1**{*214*}	COc1cc(Br)c(C)nc1N	**2**{*188*}	O=CCCC1(CC1)C#N	**3**{*12*}	[C-]#[N+]CC1COC1	6.4	5
327	**4**{*215*,*84*,*7*}	CSCCC1=C(NC2CCOCC2)N2N=C(Cl)C=C(C)C2=N1 |c:4,22,t:15,18|	Z8778278037	**1**{*215*}	Cl.Cc1cc(Cl)nnc1N	**2**{*84*}	CSCCC=O	**3**{*7*}	[C-]#[N+]C1CCOCC1	4.7	5
328	**4**{*156*,*103*,*47*}	CC(C)NC1=C(N=C2N1C(Cl)=CC=C2Cl)C1CCC2(COC2)OC1 |c:6,11,13,t:4|	Z8810903010	**1**{*156*}	Nc1nc(Cl)ccc1Cl	**2**{*103*}	O=CC1CCC2(COC2)OC1	**3**{*47*}	CC(C)[N+]#[C-]	4.5	4
329	**4**{*47*,*118*,*14*}	COCCNC1=C(N=C2C=C(C=CN12)S(N)(=O)=O)C1=CC(C)=NC=C1 |c:9,11,23,25,t:5,7,20|	Z8784059519	**1**{*47*}	Cl.Nc1cc(ccn1)S(=O)(=O)N	**2**{*118*}	Cc1cc(C=O)ccn1	**3**{*14*}	COCC[N+]#[C-]	4.3	4
330	**4**{*198*,*189*,*31*}	CSCCNC1=C(N=C2C=NC(C#C)=C(Cl)N12)C1=CN(N=N1)C1CCOCC1 |c:9,22,t:5,7,13,19|	Z8801681831	**1**{*198*}	Nc1cnc(C#C)c(Cl)n1	**2**{*189*}	O=Cc1cn(nn1)C2CCOCC2	**3**{*31*}	CSCC[N+]#[C-]	4.7	4
331	**4**{*73*,*161*,*20*}	CCN1C=NC=C1C1=C(NC2=C(C)C=C(OC)C=C2)N2N3C=CN=C3C=CC2=N1 |c:3,5,8,11,18,23,25,28,31,t:14|	Z8801681850	**1**{*73*}	Nc1ccc2nccn2n1	**2**{*161*}	CCn1cncc1C=O	**3**{*20*}	COc1ccc([N+]#[C-])c(C)c1	4.3	4
332	**4**{*216*,*190*,*10*}	CC1=CC2=NC(C3=CC(=NN3)C3=NC=CC=C3)=C(NC3CCOC3)N2N=C1C |c:8,14,16,29,t:1,3,6,12,18|	Z8801681808	**1**{*216*}	Cc1cc(N)nnc1C	**2**{*190*}	O=Cc1cc(n[nH]1)c2ccccn2	**3**{*10*}	[C-]#[N+]C1CCOC1	4.1	4
333	**4**{*217*,*14*,*7*}	CN1C(=O)NC2=C1C=CN1C(NC3CCOCC3)=C(N=C21)C1=CC=C(C)C=C1 |c:5,8,19,30,t:21,25,27|	Z8784060908	**1**{*217*}	Cn1c(=O)[nH]c2c(N)nccc12	**2**{*14*}	Cc1ccc(C=O)cc1	**3**{*7*}	[C-]#[N+]C1CCOCC1	3.9	4
334	**4**{*218*,*34*,*61*}	CC(C)(C)OC(=O)NCCCNC1=C(N=C2C=CC(=NN12)C(C)(C)C)C1=CC=C(C=C1)P(C)(C)=O |c:16,18,29,31,t:12,14,27|	Z8803896931	**1**{*218*}	CC(C)(C)c1ccc(N)nn1	**2**{*34*}	CP(=O)(C)c1ccc(C=O)cc1	**3**{*61*}	CC(C)(C)OC(=O)NCCC[N+]#[C-]	5.1	4
335	**4**{*219*,*14*,*27*}	CCCCNC1=C(N=C2N1C=C(Br)C1=C2N=CC=C1)C1=CC=C(C)C=C1 |c:7,14,17,19,27,t:5,11,22,24|	Z8797778731	**1**{*219*}	Nc1ncc(Br)c2cccnc12	**2**{*14*}	Cc1ccc(C=O)cc1	**3**{*27*}	CCCC[N+]#[C-]	4.1	4
336	**4**{*220*,*191*,*7*}	CCN1C=NN=C1C1=C(NC2CCOCC2)N2C=CC=C(C3CC3)C2=N1 |c:3,5,8,19,28,t:21|	Z8803896959	**1**{*220*}	Cl.Nc1ncccc1C2CC2	**2**{*191*}	CCn1cnnc1C=O	**3**{*7*}	[C-]#[N+]C1CCOCC1	3.5	4
337	**4**{*38*,*58*,*9*}	CNC1=C(N=C2C=C(C=CN12)C#N)C1=NSN=C1 |c:6,8,18,t:2,4,15|	Z8786958823	**1**{*38*}	Nc1cc(C#N)ccn1	**2**{*58*}	O=Cc1cnsn1	**3**{*9*}	C[N+]#[C-]	2.6	4
338	**4**{*221*,*192*,*14*}	COCCNC1=C(N=C2N1C(C)=CC=C2C#N)C1=NC(OC)=CC=C1 |c:7,12,14,23,25,t:5,19|	Z8798985058	**1**{*221*}	Cc1ccc(C#N)c(N)n1	**2**{*192*}	COc1cccc(C=O)n1	**3**{*14*}	COCC[N+]#[C-]	3.3	4

<table>
<tr><td>339</td><td>4{161,27,13}</td><td>COC1=CC=C(CNC2=C(N=C3C=CC=NN23)C2=NN(C)C(C)=N2)C=C1 |c:12,14,24,27,t:2,4,8,10,19|</td><td>Z8786958899</td><td>1{161}</td><td>Nc1cccnn1</td><td>2{27}</td><td>Cc1nc(C=O)nn1C</td><td>3{13}</td><td>COc1ccc(C[N+]#[C-])cc1</td><td>3.4</td><td>4</td></tr>
<tr><td>340</td><td>4{222,193,62}</td><td>COC(=O)C1=CN2C(NC3(CCCC3)C(=O)OC)=C(N=C2C=N1)C1=NN2CCCCOC2=C1 |c:18,20,23,35,t:4,26|</td><td>Z8808559886</td><td>1{222}</td><td>Cl.COC(=O)c1cnc(N)cn1</td><td>2{193}</td><td>O=Cc1cc2OCCCCn2n1</td><td>3{62}</td><td>COC(=O)C1(CCCC1)[N+]#[C-]</td><td>4.3</td><td>4</td></tr>
<tr><td>341</td><td>4{24,26,14}</td><td>COCCNC1=C(CCC2=CC=CC=C2)N=C2N1C=CN=C2Cl |c:5,11,13,16,20,22,t:9|</td><td>Z8741907130</td><td>1{24}</td><td>Nc1nccnc1Cl</td><td>2{26}</td><td>O=CCCc1ccccc1</td><td>3{14}</td><td>COCC[N+]#[C-]</td><td>3.2</td><td>4</td></tr>
<tr><td>342</td><td>4{223,194,29}</td><td>CCOC(=O)CCNC1=C(N=C2C=CC(=NN12)C(N)=O)C1=CC=C(N1C)C(=O)OC |c:12,14,24,t:8,10,22|</td><td>Z8798985052</td><td>1{223}</td><td>NC(=O)c1ccc(N)nn1</td><td>2{194}</td><td>COC(=O)c1ccc(C=O)n1C</td><td>3{29}</td><td>CCOC(=O)CC[N+]#[C-]</td><td>3.9</td><td>4</td></tr>
<tr><td>343</td><td>4{161,94,11}</td><td>BrC1=CC(CNC2=C(N=C3C=CC=NN23)C2=NC=CN=C2)=CC=C1 |c:10,12,19,21,23,25,t:1,6,8,17|</td><td>Z8803896956</td><td>1{161}</td><td>Nc1cccnn1</td><td>2{94}</td><td>O=Cc1cnccn1</td><td>3{11}</td><td>Brc1cccc(C[N+]#[C-])c1</td><td>3.6</td><td>3</td></tr>
<tr><td>344</td><td>4{224,95,63}</td><td>CN(C)C1=NC=CC(=C1)C1=C(NCC(F)F)N2N=C(C=CC2=N1)C1CC1 |c:5,7,10,18,20,23,t:3|</td><td>Z8781341778</td><td>1{224}</td><td>Nc1ccc(nn1)C2CC2</td><td>2{95}</td><td>CN(C)c1cc(C=O)ccn1</td><td>3{63}</td><td>FC(F)C[N+]#[C-]</td><td>3.2</td><td>3</td></tr>
<tr><td>345</td><td>4{225,195,27}</td><td>CCCCNC1=C(N=C2C=C(Cl)C=NN12)C1=CC(F)=C(OC)N=C1 |c:12,24,t:5,7,9,17,20|</td><td>Z8778278044</td><td>1{225}</td><td>Nc1cc(Cl)cnn1</td><td>2{195}</td><td>COc1ncc(C=O)cc1F</td><td>3{27}</td><td>CCCC[N+]#[C-]</td><td>3.0</td><td>3</td></tr>
<tr><td>346</td><td>4{226,97,17}</td><td>CC(C)(C)C1=CC=CC2=NC(=C(NCC3CCOCC3)N12)C1=CC=C(F)C=C1 |c:6,29,t:4,8,10,24,26|</td><td>Z8784060117</td><td>1{226}</td><td>CC(C)(C)c1cccc(N)n1</td><td>2{97}</td><td>Fc1ccc(C=O)cc1</td><td>3{17}</td><td>[C-]#[N+]CC1CCOCC1</td><td>3.2</td><td>3</td></tr>
<tr><td>347</td><td>4{227,135,47}</td><td>CC(C)NC1=C(N=C2C=NC(Br)=C(Cl)N12)C1=C(C)N(C)N=C1C |c:8,17,22,t:4,6,11|</td><td>Z8810903047</td><td>1{227}</td><td>Nc1cnc(Br)c(Cl)n1</td><td>2{135}</td><td>Cc1nn(C)c(C)c1C=O</td><td>3{47}</td><td>CC(C)[N+]#[C-]</td><td>3.2</td><td>3</td></tr>
<tr><td>348</td><td>4{228,14,7}</td><td>CC1=CC=C(C=C1)C1=C(NC2CCOCC2)N2C=CN3C=CN=C3C2=N1 |c:3,5,8,19,22,24,28,t:1|</td><td>Z8778277397</td><td>1{228}</td><td>Nc1nccn2ccnc12</td><td>2{14}</td><td>Cc1ccc(C=O)cc1</td><td>3{7}</td><td>[C-]#[N+]C1CCOCC1</td><td>2.6</td><td>3</td></tr>
<tr><td>349</td><td>4{229,196,64}</td><td>COC(=O)[C@@H]1CC[C@@H](C1)NC1=C(N=C2C=CC(=CN12)S(O)(=O)=O)C1=C(C)C=C(C=C1)N1C=CN=C1 |&1:4,7,r,c:15,17,26,29,31,35,37,t:11,13|</td><td>Z8810903023</td><td>1{229}</td><td>Nc1ccc(cn1)S(=O)(=O)O</td><td>2{196}</td><td>Cc1cc(ccc1C=O)n2ccnc2</td><td>3{64}</td><td>COC(=O)[C@@H]1CC[C@@H](C1)[N+]#[C-] |&1:4,7,r|</td><td>3.4</td><td>3</td></tr>
<tr><td>350</td><td>4{176,197,14}</td><td>COCCNC1=C(N=C2N1C=CN=C2N1CCCC1)C1=NN(C)C(=C1)C(=O)OC |c:7,11,13,26,t:5,22|</td><td>Z8798985046</td><td>1{176}</td><td>Nc1nccnc1N2CCCC2</td><td>2{197}</td><td>COC(=O)c1cc(C=O)nn1C</td><td>3{14}</td><td>COCC[N+]#[C-]</td><td>2.4</td><td>2</td></tr>
<tr><td>351</td><td>4{230,198,34}</td><td>CCC1=NN(C)C=C1C1=C(NCC2CCCO2)N2C=C(Br)C=C(C(C)O)C2=N1 |c:6,9,29,t:2,20,23|</td><td>Z8810902957</td><td>1{230}</td><td>CC(O)c1cc(Br)cnc1N</td><td>2{198}</td><td>CCc1nn(C)cc1C=O</td><td>3{34}</td><td>[C-]#[N+]CC1CCCO1</td><td>2.6</td><td>2</td></tr>
</table>

<table>
<tr><th></th><th></th><th></th><th></th><th></th><th></th><th></th><th></th><th></th><th></th><th></th><th></th></tr>
<tr><td>352</td><td>4{231,63,37}</td><td>COCN1N=CC=C1C1=C(NC2CC(C2)C(=O)OC)N2N=C(C=CC2=N1)C(F)(F)F |c:4,6,9,22,24,27|</td><td>Z8781341773</td><td>1{231}</td><td>Nc1ccc(nn1)C(F)(F)F</td><td>2{63}</td><td>COCn1nccc1C=O</td><td>3{37}</td><td>COC(=O)C1CC(C1)[N+]#[C-]</td><td>2.4</td><td>2</td></tr>
<tr><td>353</td><td>4{232,41,17}</td><td>COC1=CC=NN2C(NCC3CCOCC3)=C(N=C12)C1=NC=CC=C1 |c:4,16,24,26,t:2,18,22|</td><td>Z8778277287</td><td>1{232}</td><td>COc1ccnnc1N</td><td>2{41}</td><td>O=Cc1ccccn1</td><td>3{17}</td><td>[C-]#[N+]CC1CCOCC1</td><td>1.8</td><td>2</td></tr>
<tr><td>354</td><td>4{233,97,27}</td><td>CCCCNC1=C(N=C2N1C=C(Cl)C(Cl)=C2Cl)C1=CC=C(F)C=C1 |c:7,15,24,t:5,11,19,21|</td><td>Z8780122034</td><td>1{233}</td><td>Nc1ncc(Cl)c(Cl)c1Cl</td><td>2{97}</td><td>Fc1ccc(C=O)cc1</td><td>3{27}</td><td>CCCC[N+]#[C-]</td><td>2.0</td><td>2</td></tr>
<tr><td>355</td><td>4{234,199,10}</td><td>O=C1N(CC2=C(NC3CCOC3)N3C=CN=C(OC4CCC4)C3=N2)C(=O)C2=C1C=CC=C2 |c:4,14,25,30,33,35,t:16|</td><td>Z8810903007</td><td>1{234}</td><td>Nc1nccnc1OC2CCC2</td><td>2{199}</td><td>O=CCN1C(=O)c2ccccc2C1=O</td><td>3{10}</td><td>[C-]#[N+]C1CCOC1</td><td>2.2</td><td>2</td></tr>
<tr><td>356</td><td>4{128,200,23}</td><td>COCCN(C)C1=CN2C(C=C1)=NC(CCCC#CC)=C2NC(CCC(=O)OC)C(=O)OC |c:10,12,20,t:6|</td><td>Z8808559870</td><td>1{128}</td><td>COCCN(C)c1ccc(N)nc1</td><td>2{200}</td><td>CC#CCCCC=O</td><td>3{23}</td><td>COC(=O)CCC([N+]#[C-])C(=O)OC</td><td>1.6</td><td>1</td></tr>
<tr><td>357</td><td>4{229,201,64}</td><td>COC(=O)[C@@H]1CC[C@@H](C1)NC1=C(N=C2C=CC(=CN12)S(O)(=O)=O)C1=CC(F)=C(C)C=C1 |&1:4,7,r,c:15,17,32,t:11,13,26,29|</td><td>Z8803896930</td><td>1{229}</td><td>Nc1ccc(cn1)S(=O)(=O)O</td><td>2{201}</td><td>Cc1ccc(C=O)cc1F</td><td>3{64}</td><td>COC(=O)[C@@H]1CC[C@@H](C1)[N+]#[C-] |&1:4,7,r|</td><td>1.4</td><td>1</td></tr>
<tr><td>358</td><td>4{235,77,27}</td><td>CCCCNC1=C(CC2CCCC2)N=C2N1C=CC1=C2C=CC=C1 |c:5,14,18,20,23,25|</td><td>Z8797776760</td><td>1{235}</td><td>Nc1nccc2ccccc12</td><td>2{77}</td><td>O=CCC1CCCC1</td><td>3{27}</td><td>CCCC[N+]#[C-]</td><td>0.0</td><td>0</td></tr>
<tr><td>359</td><td>4{236,94,6}</td><td>CCOC(=O)CCCNC1=C(N=C2N1C=CC1=C2C=NC=C1)C1=NC=CN=C1 |c:11,15,17,20,22,27,29,t:9,25|</td><td>Z8797775788</td><td>1{236}</td><td>Nc1nccc2ccncc12</td><td>2{94}</td><td>O=Cc1cnccn1</td><td>3{6}</td><td>CCOC(=O)CCC[N+]#[C-]</td><td>0.0</td><td>0</td></tr>
<tr><td>360</td><td>4{237,58,14}</td><td>COCCNC1=C(N=C2C=C3C=CC=C(Br)C3=CN12)C1=NSN=C1 |c:11,17,25,t:5,7,9,13,22|</td><td>Z8797774930</td><td>1{237}</td><td>Nc1cc2cccc(Br)c2cn1</td><td>2{58}</td><td>O=Cc1cnsn1</td><td>3{14}</td><td>COCC[N+]#[C-]</td><td>0.0</td><td>0</td></tr>
<tr><td>361</td><td>4{23,202,21}</td><td>FC1=CN=CC(F)=C1C1=C(NCC2CC2)N2C(C=NC=C2Br)=N1 |c:3,6,9,19,21,24,t:1|</td><td>Z8808559895</td><td>1{23}</td><td>Nc1cncc(Br)n1</td><td>2{202}</td><td>Fc1cncc(F)c1C=O</td><td>3{21}</td><td>[C-]#[N+]CC1CC1</td><td>0.0</td><td>0</td></tr>
<tr><td>362</td><td>4{238,203,6}</td><td>CCOC(=O)CCCNC1=C(N=C2N1C=CC(OC)=C2C#N)C1=NN(CC(F)F)C=C1 |c:11,15,19,31,t:9,24|</td><td>Z8808559887</td><td>1{238}</td><td>COc1ccnc(N)c1C#N</td><td>2{203}</td><td>FC(F)Cn1ccc(C=O)n1</td><td>3{6}</td><td>CCOC(=O)CCC[N+]#[C-]</td><td>0.0</td><td>0</td></tr>
<tr><td>363</td><td>4{184,204,7}</td><td>CSC1=NC=C(N1C)C1=C(NC2CCOCC2)N2C=C(C=CC2=N1)C(N)=S |c:4,9,20,22,25,t:2|</td><td>Z8808559884</td><td>1{184}</td><td>NC(=S)c1ccc(N)nc1</td><td>2{204}</td><td>CSc1ncc(C=O)n1C</td><td>3{7}</td><td>[C-]#[N+]C1CCOCC1</td><td>0.0</td><td>0</td></tr>
</table>

364	**4**{*111,55,3*}	COC(=O)C1=CN2C(NC3=C(F)C=C(Cl)C=C3)=C(N=C2C(C)=C1)C1=CC(=NC=C1)P(C)(C)=O |c:9,15,17,19,23,28,30,t:4,12,26|	Z8808559857	**1**{*111*}	COC(=O)c1cnc(N)c(C)c1	**2**{*55*}	CP(=O)(C)c1cc(C=O)ccn1	**3**{*3*}	Fc1cc(Cl)ccc1[N+]#[C-]	0.0	0
365	**4**{*211,90,65*}	CC1=NC(=CO1)C1=C(NC2CCCC2)N2C=CC=C(C3=NN=NN3)C2=N1 |c:3,7,17,23,28,t:1,19,21|	Z8808559854	**1**{*211*}	Nc1ncccc1c2nnn[nH]2	**2**{*90*}	Cc1nc(C=O)co1	**3**{*65*}	[C-]#[N+]C1CCCC1	0.0	0
366	**4**{*239,123,22*}	CC1=NC=C(N=C1)C1=C(NC2COC2)N2C=C(C=CC2=N1)N1CCCC1 |c:3,5,8,17,19,22,t:1|	Z8810903042	**1**{*239*}	Cl.Cl.Nc1ccc(cn1)N2CCCC2	**2**{*123*}	Cc1cnc(C=O)cn1	**3**{*22*}	[C-]#[N+]C1COC1	0.0	0
367	**4**{*63,205,10*}	COC(=O)C1=C2COCCN2C(=C1)C1=C(NC2COC2)N2C=CC(C)=C(OC)C2=N1 |c:4,12,15,25,33,t:28|	Z8810902998	**1**{*63*}	COc1c(C)ccnc1N	**2**{*205*}	COC(=O)c1cc(C=O)n2CCOCc12	**3**{*10*}	[C-]#[N+]C1COC1	0.0	0
368	**4**{*240,206,15*}	CCNC1=C(C[C@H](C)NC(=O)OC(C)(C)C)N=C2C=CC(=CN12)C1=NNN=N1 |c:3,18,20,28,t:16,25|	Z8810902974	**1**{*240*}	Nc1ccc(cn1)c2nn[nH]n2	**2**{*206*}	C[C@@H](CC=O)NC(=O)OC(C)(C)C	**3**{*15*}	CC[N+]#[C-]	0.0	0
369	**4**{*241,199,21*}	BrC1=NN2C(C=C1)=NC(CN1C(=O)C3=C(C=CC=C3)C1=O)=C2NCC1CC1 |c:5,7,16,18,23,t:1,14|	Z8810902971	**1**{*241*}	Nc1ccc(Br)nn1	**2**{*199*}	O=CCN1C(=O)c2ccccc2C1=O	**3**{*21*}	[C-]#[N+]CC1CC1	0.0	0
370	**4**{*242,10,27*}	CCCCNC1=C(N=C2N1C=C(C=C2Cl)C(N)=O)C1=NC=CC(C)=C1 |c:7,11,13,22,25,t:5,20|	Z8786904184	**1**{*242*}	NC(=O)c1cnc(N)c(Cl)c1	**2**{*10*}	Cc1ccnc(C=O)c1	**3**{*27*}	CCCC[N+]#[C-]	0.0	0
371	**4**{*243,40,20*}	COC1=CC(C)=C(NC2=C(N=C3N2N=C(Cl)C2=C3C=CC=C2)C2=NC(CO)=CS2)C=C1 |c:10,17,20,22,29,33,t:2,5,8,14,25|	Z8778277875	**1**{*243*}	Nc1nnc(Cl)c2ccccc12	**2**{*40*}	OCc1csc(C=O)n1	**3**{*20*}	COc1ccc([N+]#[C-])c(C)c1	0.0	0
372	**4**{*244,84,66*}	CSCCC1=C(NC(C)C2=CC=CC=C2)N2C=C(N=CC2=N1)C#N |c:4,11,13,17,19,22,t:9|	Z8781341800	**1**{*244*}	Nc1cnc(C#N)cn1	**2**{*84*}	CSCCC=O	**3**{*66*}	CC([N+]#[C-])c1ccccc1	0.0	0
373	**4**{*245,207,37*}	COC(=O)C1CC(C1)NC1=C(N=C2C=CC=C(N12)C(C)(F)F)C1=CC(=NN1C)C(=O)OC |c:14,16,27,t:10,12,25|	Z8781341781	**1**{*245*}	Cl.CC(F)(F)c1cccc(N)n1	**2**{*207*}	COC(=O)c1cc(C=O)n(C)n1	**3**{*37*}	COC(=O)C1CC(C1)[N+]#[C-]	0.0	0
374	**4**{*246,97,20*}	COC(=O)C1=C2N(C=CN3C(NC4=C(C)C=C(OC)C=C4)=C(N=C23)C2=CC=C(F)C=C2)N=C1 |c:7,12,19,21,32,35,t:4,15,23,27,29|	Z8784060942	**1**{*246*}	COC(=O)c1cnn2ccnc(N)c12	**2**{*97*}	Fc1ccc(C=O)cc1	**3**{*20*}	COc1ccc([N+]#[C-])c(C)c1	0.0	0
375	**4**{*247,41,7*}	C1CC(CCO1)NC1=C(N=C2N1C=CN1N=CN=C21)C1=NC=CC=C1 |c:10,14,17,25,27,t:8,19,23|	Z8784060639	**1**{*247*}	Nc1nccn2ncnc12	**2**{*41*}	O=Cc1ccccn1	**3**{*7*}	[C-]#[N+]C1CCOCC1	0.0	0

376	**4**{*248*,*22*,*14*}	COCCNC1=C(N=C2N1C=CC1=C2SC=C1)C1=CC=CC=C1 |c:7,11,13,17,22,24,t:5,20|	Z8784060579	**1**{*248*}	Nc1nccc2ccsc12	**2**{*22*}	O=Cc1ccccc1	**3**{*14*}	COCC[N+]#[C-]	0.0	0
377	**4**{*20*,*170*,*6*}	CCOC(=O)CCCNC1=C(N=C2N1C(C)=CC(C)=C2C(N)=O)C1=NN(CC)N=N1 |c:11,16,19,30,t:9,25|	Z8780122411	**1**{*20*}	Cl.Cc1cc(C)c(C(=O)N)c(N)n1	**2**{*170*}	CCn1nnc(C=O)n1	**3**{*6*}	CCOC(=O)CCC[N+]#[C-]	0.0	0
378	**4**{*249*,*10*,*17*}	CC1=CC(=NC=C1)C1=C(NCC2CCOCC2)N2N=C(C)C=C(C2=N1)C(F)(F)F |c:3,5,8,23,26,t:1,20|	Z8780122347	**1**{*249*}	Cc1cc(c(N)nn1)C(F)(F)F	**2**{*10*}	Cc1ccnc(C=O)c1	**3**{*17*}	[C-]#[N+]CC1CCOCC1	0.0	0
379	**4**{*250*,*14*,*14*}	COCCNC1=C(N=C2N1C=C(Br)C=C2S(=O)(=O)N1CCCC1)C1=CC=C(C)C=C1 |c:7,14,31,t:5,11,26,28|	Z8780122344	**1**{*250*}	Nc1ncc(Br)cc1S(=O)(=O)N2CCCC2	**2**{*14*}	Cc1ccc(C=O)cc1	**3**{*14*}	COCC[N+]#[C-]	0.0	0
380	**4**{*14*,*26*,*54*}	COC1=NC=CC2=NC(CCC3=CC=CC=C3)=C(NC3=CC=CC=C3)N12 |c:4,13,15,22,24,t:2,6,11,17,20|	Z8711876243	**1**{*14*}	COc1nccc(N)n1	**2**{*26*}	O=CCCc1ccccc1	**3**{*54*}	[C-]#[N+]c1ccccc1	0.0	0
381	**4**{*9*,*14*,*17*}	CC1=CC=C(C=C1)C1=C(NCC2CCOCC2)N2C=C(Cl)C=NC2=N1 |c:3,5,8,23,26,t:1,20|	Z8711871953	**1**{*9*}	Nc1ncc(Cl)cn1	**2**{*14*}	Cc1ccc(C=O)cc1	**3**{*17*}	[C-]#[N+]CC1CCOCC1	0.0	0
382	**4**{*251*,*208*,*23*}	CCC1=CN=C(S1)C1=C(NC(CCC(=O)OC)C(=O)OC)N2C(C=CC3=C2NC(=O)C=C3)=N1 |c:4,8,24,26,32,34,t:2|	Z8801681813	**1**{*251*}	Nc1ccc2ccc(=O)[nH]c2n1	**2**{*208*}	CCc1cnc(C=O)s1	**3**{*23*}	COC(=O)CCC([N+]#[C-])C(=O)OC	0.0	0
383	**4**{*252*,*97*,*20*}	COC1=CC(C)=C(NC2=C(N=C3N2C=CC(F)=C3I)C2=CC=C(F)C=C2)C=C1 |c:10,14,17,26,29,t:2,5,8,21,23|	Z8797786651	**1**{*252*}	Nc1nccc(F)c1I	**2**{*97*}	Fc1ccc(C=O)cc1	**3**{*20*}	COc1ccc([N+]#[C-])c(C)c1	0.0	0
384	**4**{*253*,*69*,*14*}	COCCNC1=C(N=C2C=CC=C(N12)C(N)=S)C1=NN(C)C(C)=C1 |c:9,11,24,t:5,7,19|	Z8797781693	**1**{*253*}	NC(=S)c1ccc(N)n1	**2**{*69*}	Cc1cc(C=O)nn1C	**3**{*14*}	COCC[N+]#[C-]	0.0	0
385	**4**{*254*,*182*,*54*}	COC(=O)C1=CN2C(C=C1C)=NC(=C2NC1=CC=CC=C1)C1=NC=CN1CC#C |c:8,11,13,19,21,26,t:4,17,24|	Z8778278035	**1**{*254*}	COC(=O)c1cnc(N)cc1C	**2**{*182*}	O=Cc1nccn1CC#C	**3**{*54*}	[C-]#[N+]c1ccccc1	0.0	0
386	**4**{*22*,*207*,*13*}	COC(=O)C1=NN(C)C(=C1)C1=C(NCC2=CC=C(OC)C=C2)N2N=C(Cl)C=CC2=N1 |c:8,11,21,28,31,t:4,15,17,25|	Z8803896962	**1**{*22*}	Nc1ccc(Cl)nn1	**2**{*207*}	COC(=O)c1cc(C=O)n(C)n1	**3**{*13*}	COc1ccc(C[N+]#[C-])cc1	0.0	0
387	**4**{*220*,*209*,*22*}	C1CC1C1=CC=CN2C(NC3COC3)=C(N=C12)C1=NN=C2CCCCN12 |c:6,15,t:4,17,21,23|	Z8803896927	**1**{*220*}	Cl.Nc1ncccc1C2CC2	**2**{*209*}	O=Cc1nnc2CCCCn12	**3**{*22*}	[C-]#[N+]C1COC1	0.0	0

388	**4**{*255*,*210*,*39*}	CCOCCNC1=C(N=C2C=CC3=NC=C(Br)N3N12)C1=C(F)C=C(C=C1)C1=CC=NC=C1 |c:10,22,25,27,32,34,t:6,8,12,14,30|	Z8803896923	**1**{*255*}	Nc1ccc2ncc(Br)n2n1	**2**{*210*}	Fc1cc(ccc1C=O)c2ccncc2	**3**{*39*}	CCOCC[N+]#[C-]	0.0	0
389	**4**{*256*,*211*,*67*}	CCC(C)NC1=C(N=C2C=CC3=NN=NN3N12)C1=C(Br)C=CC(Cl)=C1F |c:9,13,20,23,26,t:5,7,11|	Z8803896957	**1**{*256*}	Nc1ccc2nnnn2n1	**2**{*211*}	Fc1c(Cl)ccc(Br)c1C=O	**3**{*67*}	CCC(C)[N+]#[C-]	0.0	0
390	**4**{*125*,*212*,*15*}	CCNC1=C(N=C2C=CC(CCO)=CN12)C1=NOC(=C1)C(F)F |c:7,12,20,t:3,5,17|	Z8808559873	**1**{*125*}	Nc1ccc(CCO)cn1	**2**{*212*}	FC(F)c1cc(C=O)no1	**3**{*15*}	CC[N+]#[C-]	0.0	0
391	**4**{*257*,*213*,*28*}	CCCNC1=C(N=C2N1C=CN=C2OCC(F)F)C1=C2COCCN2N=N1 |c:6,10,12,20,28,t:4|	Z8808559872	**1**{*257*}	Nc1nccnc1OCC(F)F	**2**{*213*}	O=Cc1nnn2CCOCc12	**3**{*28*}	CCC[N+]#[C-]	0.0	0
392	**4**{*92*,*214*,*14*}	COCCNC1=C(N=C2C=C(C=CN12)C(N)=O)C1=CC=NN1CC(C)C |c:9,11,21,t:5,7,19|	Z8810903048	**1**{*92*}	NC(=O)c1ccnc(N)c1	**2**{*214*}	CC(C)Cn1ncc1C=O	**3**{*14*}	COCC[N+]#[C-]	0.0	0
393	**4**{*78*,*215*,*39*}	CCOCCNC1=C(N=C2C=C(C)C3=NC(C)=CN3N12)C1=NC(=CS1)C1=CC=CC=C1 |c:16,25,31,33,t:6,8,10,13,23,29|	Z8810903022	**1**{*78*}	Cc1cn2nc(N)cc(C)c2n1	**2**{*215*}	O=Cc1nc(cs1)c2ccccc2	**3**{*39*}	CCOCC[N+]#[C-]	0.0	0
394	**4**{*111*,*216*,*22*}	COC(=O)C1=NC=C(S1)C1=C(NC2COC2)N2C=C(C=C(C)C2=N1)C(=O)OC |c:6,10,19,25,t:4,21|	Z8810902995	**1**{*111*}	COC(=O)c1cnc(N)c(C)c1	**2**{*216*}	COC(=O)c1ncc(C=O)s1	**3**{*22*}	[C-]#[N+]C1COC1	0.0	0
395	**4**{*169*,*207*,*13*}	CNC(=O)C1=NN2C(C=C1)=NC(=C2NCC1=CC=C(OC)C=C1)C1=CC(=NN1C)C(=O)OC |c:8,10,12,23,28,t:4,17,19,26|	Z8810902985	**1**{*169*}	CNC(=O)c1ccc(N)nn1	**2**{*207*}	COC(=O)c1cc(C=O)n(C)n1	**3**{*13*}	COc1ccc(C[N+]#[C-])cc1	0.0	0
396	**4**{*258*,*217*,*67*}	CCC(C)NC1=C(N=C2C=CC3=C(N=CC=N3)N12)C1=CC(=O)N(C)C=C1 |c:9,13,15,27,t:5,7,11,21|	Z8810902984	**1**{*258*}	Nc1ccc2nccnc2n1	**2**{*217*}	Cn1ccc(C=O)cc1=O	**3**{*67*}	CCC(C)[N+]#[C-]	0.0	0
397	**4**{*259*,*11*,*7*}	FC(F)(F)CN1C=CC(=N1)C1=C(NC2CCOCC2)N2C(=N1)C(=CC1=C2CCC(F)(F)C1)C#N |c:6,8,11,22,25,27|	Z8810902976	**1**{*259*}	Nc1nc2CCC(F)(F)Cc2cc1C#N	**2**{*11*}	FC(F)(F)Cn1ccc(C=O)n1	**3**{*7*}	[C-]#[N+]C1CCOCC1	0.0	0
398	**4**{*260*,*218*,*14*}	COCCNC1=C(N=C2N1C=CC=C2N1CCCC1)C1CC(C1)OC |c:7,11,13,t:5|	Z8810902962	**1**{*260*}	Nc1ncccc1N2CCCC2	**2**{*218*}	COC1CC(C1)C=O	**3**{*14*}	COCC[N+]#[C-]	0.0	0
399	**4**{*261*,*21*,*39*}	CCOCCNC1=C(N=C2C=CC(=CN12)N1CCC(CC1)N(C)C)C1=CC2=NC=NN2C=C1 |c:10,12,31,35,t:6,8,27,29|	Z8798985033	**1**{*261*}	CN(C)C1CCN(CC1)c2ccc(N)nc2	**2**{*21*}	O=Cc1ccn2ncnc2c1	**3**{*39*}	CCOCC[N+]#[C-]	0.0	0
400	**4**{*262*,*181*,*28*}	CCCNC1=C(N=C2C=CC(NC(C)=O)=CN12)C1=CN=C2CCCN12 |c:8,14,t:4,6,19,21|	Z8786958954	**1**{*262*}	CC(=O)Nc1ccc(N)nc1	**2**{*181*}	O=Cc1cnc2CCCn12	**3**{*28*}	CCC[N+]#[C-]	0.0	0
401	**4**{*263*,*121*,*25*}	CCC1=CON=C1C1=C(NCCCF)N2C=CC=C(C(F)F)C2=N1 |c:5,8,16,24,t:2,18|	Z8781341772	**1**{*263*}	Nc1ncccc1C(F)F	**2**{*121*}	CCc1conc1C=O	**3**{*25*}	FCCC[N+]#[C-]	0.0	0

402	**4**{*264*,*44*,*27*}	CCCCNC1=C(N=C2C=NC=C(N3CCCC3)N12)C1=CN(CC)N=C1 |c:9,27,t:5,7,11,22|	Z8784060344	**1**{*264*}	Nc1cncc(n1)N2CCCC2	**2**{*44*}	CCn1cc(C=O)cn1	**3**{*27*}	CCCC[N+]#[C-]	0.0	0
403	**4**{*18*,*40*,*20*}	COC1=CC(C)=C(NC2=C(N=C3C=C(Br)C(=CN23)[N+]([O-])=O)C2=NC(CO)=CS2)C=C1 |c:15,27,31,t:2,5,8,10,12,23|	Z8780122420	**1**{*18*}	Nc1cc(Br)c(cn1)[N+](=O)[O-]	**2**{*40*}	OCc1csc(C=O)n1	**3**{*20*}	COc1ccc([N+]#[C-])c(C)c1	0.0	0
404	**4**{*17*,*14*,*7*}	CC1=CC=C(C=C1)C1=C(NC2CCOCC2)N2C=CC=C(C2=N1)[N+]([O-])=O |c:3,5,8,19,21,24,t:1|	Z8780122033	**1**{*17*}	Nc1ncccc1[N+](=O)[O-]	**2**{*14*}	Cc1ccc(C=O)cc1	**3**{*7*}	[C-]#[N+]C1CCOCC1	0.0	0
405	**4**{*265*,*80*,*14*}	COCCNC1=C(N=C2N1C(F)=CC=C2F)C1=NC=C(C)S1 |c:7,12,14,t:5,18,20|	Z8780121774	**1**{*265*}	Nc1nc(F)ccc1F	**2**{*80*}	Cc1cnc(C=O)s1	**3**{*14*}	COCC[N+]#[C-]	0.0	0
406	**4**{*3*,*26*,*17*}	C(CC1=CC=CC=C1)C1=C(NCC2CCOCC2)N2C=CSC2=N1 |c:4,6,9,21,25,t:2|	Z8711876688	**1**{*3*}	Nc1nccs1	**2**{*26*}	O=CCCc1ccccc1	**3**{*17*}	[C-]#[N+]CC1CCOCC1	0.0	0
407	**4**{*266*,*22*,*13*}	COC1=CC=C(CNC2=C(N=C3N2C=CN=C3C#N)C2=CC=CC=C2)C=C1 |c:10,14,16,23,25,28,t:2,4,8,21|	Z8741907203	**1**{*266*}	Nc1nccnc1C#N	**2**{*22*}	O=Cc1ccccc1	**3**{*13*}	COc1ccc(C[N+]#[C-])cc1	0.0	0
408	**4**{*267*,*219*,*1*}	CN1C=C(C(C=C1C#N)C1=C(NC2CC2)N2C(C=CC=C2C#C)=N1 |c:2,4,9,18,20,24|	Z8801681851	**1**{*267*}	Nc1cccc(C#C)n1	**2**{*219*}	Cn1cc(C=O)cc1C#N	**3**{*1*}	[C-]#[N+]C1CC1	0.0	0
409	**4**{*174*,*220*,*51*}	CC1=C(C(C)=NN1)C1=C(NCCNC(=O)OC(C)(C)C)N2C(C=CC=C2C#N)=N1 |c:4,8,23,25,29,t:1|	Z8801681843	**1**{*174*}	Nc1cccc(C#N)n1	**2**{*220*}	Cc1n[nH]c(C)c1C=O	**3**{*51*}	CC(C)(C)OC(=O)NCC[N+]#[C-]	0.0	0
410	**4**{*99*,*68*,*41*}	COC1=CC=C(NC2=C(N=C3C=CC(=CN23)S(N)(=O)=O)C2=NC=C(S2)C(C)O)C=C1 |c:11,13,24,31,t:2,4,7,9,22|	Z8801681811	**1**{*99*}	Nc1ccc(cn1)S(=O)(=O)N	**2**{*68*}	CC(O)c1cnc(C=O)s1	**3**{*41*}	COc1ccc([N+]#[C-])cc1	0.0	0
411	**4**{*268*,*94*,*17*}	NC(=O)C1=CC(OC2=CN3C(C=C2)=NC(=C3NCC2CCOCC2)C2=NC=CN=C2)=CC=C1 |c:11,13,15,29,31,33,35,t:3,7,27|	Z8797784545	**1**{*268*}	NC(=O)c1cccc(Oc2ccc(N)nc2)c1	**2**{*94*}	O=Cc1cnccn1	**3**{*17*}	[C-]#[N+]CC1CCOCC1	0.0	0
412	**4**{*269*,*9*,*27*}	CCCCNC1=C(N=C2C=C3C=CNC3=CN12)C1=CSC=C1 |c:11,15,23,t:5,7,9,20|	Z8797773920	**1**{*269*}	Nc1cc2cc[nH]c2cn1	**2**{*9*}	O=Cc1ccsc1	**3**{*27*}	CCCC[N+]#[C-]	0.0	0
413	**4**{*270*,*112*,*13*}	COC1=CC=C(CNC2=C(N=C3C=CC4=NC=NN4N23)C2=C3C=CC=NN3N=C2)C=C1 |c:12,16,23,25,27,31,34,t:2,4,8,10,14|	Z8778278047	**1**{*270*}	Nc1ccc2ncnn2n1	**2**{*112*}	O=Cc1cnn2ncccc12	**3**{*13*}	COc1ccc(C[N+]#[C-])cc1	0.0	0
414	**4**{*76*,*84*,*18*}	CSCCC1=C(NC(C)(C)C)N2C(C=CC(C)=C2Cl)=N1 |c:4,13,16,19|	Z8778278030	**1**{*76*}	Cc1ccc(N)nc1Cl	**2**{*84*}	CSCCC=O	**3**{*18*}	CC(C)(C)[N+]#[C-]	0.0	0

415	**4**{*151*,*157*,*9*}	CNC1=C(N=C2C=NC=C(Cl)N12)C1=NC2=C(C=C1)N=CC=C2 |c:6,16,18,21,23,t:2,4,8,14|	Z8803896945	**1**{*151*}	Nc1cncc(Cl)n1	**2**{*157*}	O=Cc1ccc2ncccc2n1	**3**{*9*}	C[N+]#[C-]	0.0	0
416	**4**{*222*,*221*,*5*}	COCCCNC1=C(N=C2C=NC(=CN12)C(=O)OC)C1=NC(OC)=C(C)S1 |c:10,12,t:6,8,21,25|	Z8803896936	**1**{*222*}	Cl.COC(=O)c1cnc(N)cn1	**2**{*221*}	COc1nc(C=O)sc1C	**3**{*5*}	COCCC[N+]#[C-]	0.0	0
417	**4**{*262*,*222*,*10*}	COC1=C(OCCN2CCOCC2)C=CC(=C1)C1=C(NC2CCOC2)N2C=C(NC(C)=O)C=CC2=N1 |c:2,14,16,19,35,38,t:29|	Z8808559889	**1**{*262*}	CC(=O)Nc1ccc(N)nc1	**2**{*222*}	COc1cc(C=O)ccc1OCCN2CCOCC2	**3**{*10*}	[C-]#[N+]C1CCOC1	0.0	0
418	**4**{*145*,*223*,*3*}	COC(=O)C1=NC=C(C=C1)C1=C(NC2=C(F)C=C(Cl)C=C2)N2C(C=CC=C2S(C)(=O)=O)=N1 |c:6,8,11,14,20,25,27,33,t:4,17|	Z8808559871	**1**{*145*}	CS(=O)(=O)c1cccc(N)n1	**2**{*223*}	Cl.COC(=O)c1ccc(C=O)cn1	**3**{*3*}	Fc1cc(Cl)ccc1[N+]#[C-]	0.0	0
419	**4**{*105*,*224*,*66*}	CC(NC1=C(N=C2C=CC=C(N12)C(N)=O)C1CCOC1C)C1=CC=CC=C1 |c:7,9,26,28,t:3,5,24|	Z8810903039	**1**{*105*}	NC(=O)c1cccc(N)n1	**2**{*224*}	CC1OCCC1C=O	**3**{*66*}	CC([N+]#[C-])c1ccccc1	0.0	0
420	**4**{*158*,*225*,*18*}	CC(C)(C)NC1=C(N=C2C=NC(=CN12)P(C)(C)=O)C1=C2C=NNC2=CC(Cl)=C1 |c:9,11,20,22,26,29,t:5,7|	Z8810903021	**1**{*158*}	CP(=O)(C)c1cnc(N)cn1	**2**{*225*}	Clc1cc(C=O)c2cn[nH]c2c1	**3**{*18*}	CC(C)(C)[N+]#[C-]	0.0	0
421	**4**{*271*,*226*,*67*}	CCC(C)NC1=C(N=C2C=CC(C(=O)OC)=C(F)N12)C1=C(C)N(CC(F)(F)F)N=C1 |c:9,21,30,t:5,7,15|	Z8810903011	**1**{*271*}	COC(=O)c1ccc(N)nc1F	**2**{*226*}	Cc1c(C=O)cnn1CC(F)(F)F	**3**{*67*}	CCC(C)[N+]#[C-]	0.0	0
422	**4**{*144*,*227*,*37*}	CNS(=O)(=O)C1=CN2C(NC3CC(C3)C(=O)OC)=C(N=C2C=C1)C1=NC=CS1 |c:18,20,23,28,t:5,26|	Z8810902996	**1**{*144*}	CNS(=O)(=O)c1ccc(N)nc1	**2**{*227*}	O=Cc1nccs1	**3**{*37*}	COC(=O)C1CC(C1)[N+]#[C-]	0.0	0
423	**4**{*158*,*228*,*32*}	CC(C)(C)OC(=O)N1CCC(C1)C1=C(NC2CCC2)N2C=C(N=CC2=N1)P(C)(C)=O |c:13,22,24,27|	Z8798985055	**1**{*158*}	CP(=O)(C)c1cnc(N)cn1	**2**{*228*}	CC(C)(C)OC(=O)N1CCC(C1)C=O	**3**{*32*}	[C-]#[N+]C1CCC1	0.0	0
424	**4**{*272*,*229*,*29*}	CCOC(=O)CCNC1=C(N=C2C=CC(CN3CCCC3)=CN12)C1=CN=C(OCCO)C=C1 |c:12,21,34,t:8,10,26,28|	Z8798985037	**1**{*272*}	Nc1ccc(CN2CCCC2)cn1	**2**{*229*}	OCCOc1ccc(C=O)cn1	**3**{*29*}	CCOC(=O)CC[N+]#[C-]	0.0	0
425	**4**{*273*,*230*,*64*}	COC(=O)[C@@H]1CC[C@@H](C1)NC1=C(N=C2C=C3C=NNC3=CN12)C1=CC(OC)=CN=C1 |&1:4,7,r,c:17,21,30,32,t:11,13,15,26|	Z8786958894	**1**{*273*}	Nc1cc2cn[nH]c2cn1	**2**{*230*}	COc1cncc(C=O)c1	**3**{*64*}	COC(=O)[C@@H]1CC[C@@H](C1)[N+]#[C-] |&1:4,7,r|	0.0	0

426	**4**{*274,69,6*}	CCOC(=O)CCCNC1=C(N=C2N1C=C(C=C2F)C(=O)OC)C1=NN(C)C(C)=C1 |c:11,15,17,30,t:9,25|	Z8786938003	**1**{*274*}	COC(=O)c1cnc(N)c(F)c1	**2**{*69*}	Cc1cc(C=O)nn1C	**3**{*6*}	CCOC(=O)CCC[N+]#[C-]	0.0	0
427	**4**{*275,58,7*}	FC1=CC=C(Cl)N2C(NC3CCOCC3)=C(N=C12)C1=NSN=C1 |c:15,24,t:1,3,17,21|	Z8786838534	**1**{*275*}	Nc1nc(Cl)ccc1F	**2**{*58*}	O=Cc1cnsn1	**3**{*7*}	[C-]#[N+]C1CCOCC1	0.0	0
428	**4**{*276,48,7*}	N#CCC1=CC=CC2=NC(=C(NC3CCOCC3)N12)C1=CNC(=N1)C1CCC1 |c:5,25,t:3,7,9,22|	Z8781341802	**1**{*276*}	Nc1cccc(CC#N)n1	**2**{*48*}	O=Cc1c[nH]c(n1)C2CCC2	**3**{*7*}	[C-]#[N+]C1CCOCC1	0.0	0
429	**4**{*137,112,14*}	COCCNC1=C(N=C2C=CC(C)=NN12)C1=C2C=CC=NN2N=C1 |c:9,12,17,19,21,25,t:5,7|	Z8781341792	**1**{*137*}	Cc1ccc(N)nn1	**2**{*112*}	O=Cc1cnn2ncccc12	**3**{*14*}	COCC[N+]#[C-]	0.0	0
430	**4**{*277,231,4*}	CCOC1=NN2C(C=C1)=NC(C1=NC3=C(CC(C)CC3)S1)=C2NCC1=NN(C)N=N1 |c:7,9,23,32,t:3,12,14,28|	Z8781341786	**1**{*277*}	CCOc1ccc(N)nn1	**2**{*231*}	CC1CCc2nc(C=O)sc2C1	**3**{*4*}	Cn1nnc(C[N+]#[C-])n1	0.0	0
431	**4**{*213,232,27*}	CCCCNC1=C(N=C2N1C(OC)=CC=C2F)C1=NOCC1 |c:7,13,15,t:5,19|	Z8780122425	**1**{*213*}	COc1ccc(F)c(N)n1	**2**{*232*}	O=CC1=NOCC1	**3**{*27*}	CCCC[N+]#[C-]	0.0	0
432	**4**{*278,80,27*}	CCCCNC1=C(N=C2C=CC(C#N)=C(F)N12)C1=NC=C(C)S1 |c:9,t:5,7,13,19,21|	Z8780122343	**1**{*278*}	Nc1ccc(C#N)c(F)n1	**2**{*80*}	Cc1cnc(C=O)s1	**3**{*27*}	CCCC[N+]#[C-]	0.0	0
433	**4**{*279,77,6*}	CCOC(=O)CCCNC1=C(CC2CCCC2)N=C2N1C=C(Br)C=C2S(=O)(=O)NC |c:9,18,25,t:22|	Z8780122340	**1**{*279*}	CNS(=O)(=O)c1cc(Br)cnc1N	**2**{*77*}	O=CCC1CCCC1	**3**{*6*}	CCOC(=O)CCC[N+]#[C-]	0.0	0
434	**4**{*279,58,27*}	CCCCNC1=C(N=C2N1C=C(Br)C=C2S(=O)(=O)NC)C1=NSN=C1 |c:7,14,25,t:5,11,22|	Z8780122268	**1**{*279*}	CNS(=O)(=O)c1cc(Br)cnc1N	**2**{*58*}	O=Cc1cnsn1	**3**{*27*}	CCCC[N+]#[C-]	0.0	0
435	**4**{*280,26,7*}	BrC1=C2N=CC=CC2=CC2=NC(CCC3=CC=CC=C3)=C(NC3CCOCC3)N12 |c:1,3,5,8,17,19,t:10,15,21|	Z8776690337	**1**{*280*}	Nc1cc2cccnc2c(Br)n1	**2**{*26*}	O=CCCc1ccccc1	**3**{*7*}	[C-]#[N+]C1CCOCC1	0.0	0
436	**4**{*244,26,26*}	FC1=CC=C(CNC2=C(CCC3=CC=CC=C3)N=C3C=NC(=CN23)C#N)C=C1 |c:7,13,15,20,22,29,t:1,3,11,18|	Z8776690340	**1**{*244*}	Nc1cnc(C#N)cn1	**2**{*26*}	O=CCCc1ccccc1	**3**{*26*}	Fc1ccc(C[N+]#[C-])cc1	0.0	0
437	**4**{*15,15,27*}	CCCCNC1=C(N=C2C=CN=C(F)N12)C1=CC=C(OC)C=C1 |c:9,23,t:5,7,11,17,19|	Z8711876135	**1**{*15*}	Nc1ccnc(F)n1	**2**{*15*}	COc1ccc(C=O)cc1	**3**{*27*}	CCCC[N+]#[C-]	0.0	0
438	**4**{*281,14,54*}	CC1=CC=C(C=C1)C1=C(NC2=CC=CC=C2)N2C=CN=C(F)C2=N1 |c:3,5,8,13,15,19,25,t:1,11,21|	Z8711876133	**1**{*281*}	Nc1nccnc1F	**2**{*14*}	Cc1ccc(C=O)cc1	**3**{*54*}	[C-]#[N+]c1ccccc1	0.0	0
439	**4**{*282,26,14*}	COCCNC1=C(CCC2=CC=CC=C2)N=C2C=NC=C(O)N12 |c:5,11,13,18,t:9,16,20|	Z8711875965	**1**{*282*}	Nc1cncc(O)n1	**2**{*26*}	O=CCCc1ccccc1	**3**{*14*}	COCC[N+]#[C-]	0.0	0

<table>
<tr><td>440</td><td>4{8,26,17}</td><td>CC1=NC2=NC(CCC3=CC=CC=C3)=C(NCC3CCOCC3)N2C(C)=C1 |c:10,12,28,t:1,3,8,14|</td><td>Z8711872601</td><td>1{8}</td><td>Cc1cc(C)nc(N)n1</td><td>2{26}</td><td>O=CCCc1ccccc1</td><td>3{17}</td><td>[C-]#[N+]CC1CCOCC1</td><td>0.0</td><td>0</td></tr>
<tr><td>441</td><td>4{283,233,39}</td><td>CCOCCNC1=C(CCCOCC=C)N=C2C=CC3=C(N=CC(=C3)C(=O)OC)N12 |c:6,17,21,23,t:15,19|</td><td>Z8801681852</td><td>1{283}</td><td>COC(=O)c1cnc2nc(N)ccc2c1</td><td>2{233}</td><td>C=CCOCCCC=O</td><td>3{39}</td><td>CCOCC[N+]#[C-]</td><td>0.0</td><td>0</td></tr>
<tr><td>442</td><td>4{284,234,32}</td><td>N#CCC1=CC2=NC(=C(NC3CCC3)N2C=C1)C1=NN=C2CCCCCN12 |c:17,t:3,5,7,20,22|</td><td>Z8801681812</td><td>1{284}</td><td>Nc1cc(CC#N)ccn1</td><td>2{234}</td><td>O=Cc1nnc2CCCCCn12</td><td>3{32}</td><td>[C-]#[N+]C1CCC1</td><td>0.0</td><td>0</td></tr>
<tr><td>443</td><td>4{285,10,7}</td><td>CC1=CC(=NC=C1)C1=C(NC2CCOCC2)N2C(=N1)C1=C(C=C(Br)C=C1)C=C2C |c:3,5,8,19,27,30,t:1,22,24|</td><td>Z8797779857</td><td>1{285}</td><td>Cc1cc2cc(Br)ccc2c(N)n1</td><td>2{10}</td><td>Cc1ccnc(C=O)c1</td><td>3{7}</td><td>[C-]#[N+]C1CCOCC1</td><td>0.0</td><td>0</td></tr>
<tr><td>444</td><td>4{284,157,11}</td><td>BrC1=CC(CNC2=C(N=C3C=C(CC#N)C=CN23)C2=NC3=C(C=C2)N=CC=C3)=CC=C1 |c:15,22,24,27,29,31,33,t:1,6,8,10,20|</td><td>Z8778278043</td><td>1{284}</td><td>Nc1cc(CC#N)ccn1</td><td>2{157}</td><td>O=Cc1ccc2ncccc2n1</td><td>3{11}</td><td>Brc1cccc(C[N+]#[C-])c1</td><td>0.0</td><td>0</td></tr>
<tr><td>445</td><td>4{94,79,27}</td><td>CCCCNC1=C(N=C2C=CC(F)=NN12)C1=NC=CO1 |c:9,12,19,t:5,7,17|</td><td>Z8778278041</td><td>1{94}</td><td>Nc1ccc(F)nn1</td><td>2{79}</td><td>O=Cc1ncco1</td><td>3{27}</td><td>CCCC[N+]#[C-]</td><td>0.0</td><td>0</td></tr>
<tr><td>446</td><td>4{81,129,9}</td><td>CNC1=C(N=C2C=NC=CN12)C1=CC=CC2=NC=NN12 |c:6,8,15,19,t:2,4,13,17|</td><td>Z8808559888</td><td>1{81}</td><td>Nc1cnccn1</td><td>2{129}</td><td>O=Cc1cccc2ncnn12</td><td>3{9}</td><td>C[N+]#[C-]</td><td>0.0</td><td>0</td></tr>
<tr><td>447</td><td>4{286,235,23}</td><td>COC(=O)CCC(NC1=C(N=C2C=CC(=CN12)C1=NC=CC=C1)C1=CSC(=C1)C#N)C(=O)OC |c:12,14,21,23,29,t:8,10,19,26|</td><td>Z8808559878</td><td>1{286}</td><td>Cl.Cl.Nc1ccc(cn1)c2ccccn2</td><td>2{235}</td><td>O=Cc1csc(C#N)c1</td><td>3{23}</td><td>COC(=O)CCC([N+]#[C-])C(=O)OC</td><td>0.0</td><td>0</td></tr>
<tr><td>448</td><td>4{287,236,21}</td><td>ClC1=CC2=NC(CCC3OCCO3)=C(NCC3CC3)N2C=C1 |c:23,t:1,3,13|</td><td>Z8810903029</td><td>1{287}</td><td>Nc1cc(Cl)ccn1</td><td>2{236}</td><td>O=CCCC1OCCO1</td><td>3{21}</td><td>[C-]#[N+]CC1CC1</td><td>0.0</td><td>0</td></tr>
<tr><td>449</td><td>4{288,148,39}</td><td>CCOCCNC1=C(N=C2C=C(NC(=O)OC(C)(C)C)C=CN12)C1=C(OC)N(C)N=C1C |c:20,25,31,t:6,8,10|</td><td>Z8810903014</td><td>1{288}</td><td>CC(C)(C)OC(=O)Nc1ccnc(N)c1</td><td>2{148}</td><td>COc1c(C=O)c(C)nn1C</td><td>3{39}</td><td>CCOCC[N+]#[C-]</td><td>0.0</td><td>0</td></tr>
<tr><td>450</td><td>4{238,237,26}</td><td>COC1=C(C#N)C2=NC(C3CCCOCC3)=C(NCC3=CC=C(F)C=C3)N2C=C1 |c:2,25,30,t:6,16,20,22|</td><td>Z8810902986</td><td>1{238}</td><td>COc1ccnc(N)c1C#N</td><td>2{237}</td><td>O=CC1CCCOCC1</td><td>3{26}</td><td>Fc1ccc(C[N+]#[C-])cc1</td><td>0.0</td><td>0</td></tr>
<tr><td>451</td><td>4{158,2,31}</td><td>CSCCNC1=C(N=C2C=NC(=CN12)P(C)(C)=O)C1=C(F)C=NC(Cl)=C1 |c:9,11,20,23,26,t:5,7|</td><td>Z8798985045</td><td>1{158}</td><td>CP(=O)(C)c1cnc(N)cn1</td><td>2{2}</td><td>Fc1cnc(Cl)cc1C=O</td><td>3{31}</td><td>CSCC[N+]#[C-]</td><td>0.0</td><td>0</td></tr>
<tr><td>452</td><td>4{284,95,30}</td><td>CN(C)C1=NC=CC(=C1)C1=C(NCC2=CN(C)N=C2)N2C=CC(CC#N)=CC2=N1 |c:5,7,10,18,22,27,30,t:3,14|</td><td>Z8781341770</td><td>1{284}</td><td>Nc1cc(CC#N)ccn1</td><td>2{95}</td><td>CN(C)c1cc(C=O)ccn1</td><td>3{30}</td><td>Cn1cc(C[N+]#[C-])cn1</td><td>0.0</td><td>0</td></tr>
</table>

453	**4**{21,69,17}	CN1N=C(C=C1C)C1=C(NCC2CCOCC2)N2C(=N1)C(=CC=C2Cl)S(N)(=O)=O |c:2,4,8,20,23,25|	Z8780122404	**1**{21}	Nc1nc(Cl)ccc1S(=O)(=O)N	**2**{69}	Cc1cc(C=O)nn1C	**3**{17}	[C-]#[N+]CC1CCOCC1	0.0	0
454	**4**{289,97,6}	CCOC(=O)CCCNC1=C(N=C2N1C=CC=C2C(F)(F)C(F)(F)F)C1=CC=C(F)C=C1 |c:11,15,17,32,t:9,27,29|	Z8780122348	**1**{289}	Cl.Nc1ncccc1C(F)(F)C(F)(F)F	**2**{97}	Fc1ccc(C=O)cc1	**3**{6}	CCOC(=O)CCC[N+]#[C-]	0.0	0
455	**4**{290,41,7}	COC(=O)C1=CC=C(Br)N2C(NC3CCOCC3)=C(N=C12)C1=NC=CC=C1 |c:18,26,28,t:4,6,20,24|	Z8780122335	**1**{290}	COC(=O)c1ccc(Br)nc1N	**2**{41}	O=Cc1ccccn1	**3**{7}	[C-]#[N+]C1CCOCC1	0.0	0
456	**4**{5,26,17}	C(NC1=C(CCC2=CC=CC=C2)N=C2NN=NN12)C1CCOCC1 |c:2,8,10,16,t:6,13|	Z8711892497	**1**{5}	Nc1nnn[nH]1	**2**{26}	O=CCCc1ccccc1	**3**{17}	[C-]#[N+]CC1CCOCC1	0.0	0
457	**4**{291,22,54}	ClC1=CN2C(NC3=CC=CC=C3)=C(N=C2C(Cl)=C1)C1=CC=CC=C1 |c:8,10,12,14,18,23,25,t:1,6,21|	Z8711876685	**1**{291}	Nc1ncc(Cl)cc1Cl	**2**{22}	O=Cc1ccccc1	**3**{54}	[C-]#[N+]c1ccccc1	0.0	0
458	**4**{10,26,27}	CCCCNC1=C(CCC2=CC=CC=C2)N=C2N=CC(Br)=CN12 |c:5,11,13,18,21,t:9,16|	Z8711871783	**1**{10}	Nc1ncc(Br)cn1	**2**{26}	O=CCCc1ccccc1	**3**{27}	CCCC[N+]#[C-]	0.0	0
459	**4**{292,130,32}	CCOC1=CN=CC2=NC(=C(NC3CCC3)N12)C1=NN=C(OC)C=C1 |c:5,26,t:3,7,9,20,22|	Z8801681848	**1**{292}	CCOc1cncc(N)n1	**2**{130}	COc1ccc(C=O)nn1	**3**{32}	[C-]#[N+]C1CCC1	0.0	0
460	**4**{293,156,10}	COC1=CC(C)=CN2C(NC3CCOC3)=C(N=C12)C1=C(Cl)N=CN1C |c:5,15,21,24,t:2,17|	Z8801681819	**1**{293}	COc1cc(C)cnc1N	**2**{156}	Cn1cnc(Cl)c1C=O	**3**{10}	[C-]#[N+]C1CCOC1	0.0	0
461	**4**{234,238,39}	CCOCCNC1=C(N=C2N1C=CN=C2OC1CCC1)C1=C(Br)N=C2COCCN12 |c:8,12,14,23,t:6,26|	Z8801681807	**1**{234}	Nc1nccnc1OC2CCC2	**2**{238}	Brc1nc2COCCn2c1C=O	**3**{39}	CCOCC[N+]#[C-]	0.0	0
462	**4**{294,10,6}	CCOC(=O)CCCNC1=C(N=C2N1C=C(C=C2Cl)S(C)(=O)=O)C1=NC=CC(C)=C1 |c:11,15,17,27,30,t:9,25|	Z8797786418	**1**{294}	CS(=O)(=O)c1cnc(N)c(Cl)c1	**2**{10}	Cc1ccnc(C=O)c1	**3**{6}	CCOC(=O)CCC[N+]#[C-]	0.0	0
463	**4**{229,170,7}	CCN1N=NC(=N1)C1=C(NC2CCOCC2)N2C=C(C=CC2=N1)S(O)(=O)=O |c:3,5,8,19,21,24|	Z8797781947	**1**{229}	Nc1ccc(cn1)S(=O)(=O)O	**2**{170}	CCn1nnc(C=O)n1	**3**{7}	[C-]#[N+]C1CCOCC1	0.0	0
464	**4**{295,80,14}	COCCNC1=C(N=C2N1C(C)=CC1=C2C=CC=C1)C1=NC=C(C)S1 |c:7,12,14,17,19,t:5,22,24|	Z8797778300	**1**{295}	Cc1cc2ccccc2c(N)n1	**2**{80}	Cc1cnc(C=O)s1	**3**{14}	COCC[N+]#[C-]	0.0	0
465	**4**{296,22,7}	BrC1=CC2=CC3=NC(=C(NC4CCOCC4)N3C=C2C=C1)C1=CC=CC=C1 |c:19,22,27,29,t:1,3,5,7,25|	Z8797775351	**1**{296}	Nc1cc2cc(Br)ccc2cn1	**2**{22}	O=Cc1ccccc1	**3**{7}	[C-]#[N+]C1CCOCC1	0.0	0

466	**4**{*123*,*239*,*6*}	CCOC(=O)CCCNC1=C(N=C2C=C(OCCO)C=CN12)C1=NOC2=C1CCCC2 |c:19,27,t:9,11,13,24|	Z8778278033	**1**{*123*}	Cl.Nc1cc(OCCO)ccn1	**2**{*239*}	O=Cc1noc2CCCCc12	**3**{*6*}	CCOC(=O)CCC[N+]#[C-]	0.0	0
467	**4**{*46*,*240*,*9*}	CCN1N=C(Br)C=C1C1=C(NC)N2C=CC=CC2=N1 |c:6,9,14,16,19,t:3|	Z8803896938	**1**{*46*}	Nc1ccccn1	**2**{*240*}	CCn1nc(Br)cc1C=O	**3**{*9*}	C[N+]#[C-]	0.0	0
468	**4**{*99*,*241*,*41*}	COC1=CC=C(NC2=C(N=C3C=CC(=CN23)S(N)(=O)=O)C2=C(Br)C=NN2C)C=C1 |c:11,13,22,25,30,t:2,4,7,9|	Z8808559900	**1**{*99*}	Nc1ccc(cn1)S(=O)(=O)N	**2**{*241*}	Cn1ncc(Br)c1C=O	**3**{*41*}	COc1ccc([N+]#[C-])cc1	0.0	0
469	**4**{*121*,*242*,*10*}	BrC1=CC2=NC(=C(NC3CCOC3)N2C=C1)C1=CN2N=CC(C#N)=C2N=C1 |c:16,22,26,29,t:1,3,5,19|	Z8808559894	**1**{*121*}	Nc1cc(Br)ccn1	**2**{*242*}	O=Cc1cnc2c(C#N)cnn2c1	**3**{*10*}	[C-]#[N+]C1CCOC1	0.0	0
470	**4**{*297*,*243*,*7*}	CCC1=NOC=C1C1=C(NC2CCOCC2)N2C(=N1)C(C#N)=C(C)C1=C2C=CC=C1 |c:5,8,19,27,30,32,t:2,24|	Z8808559868	**1**{*297*}	Cc1c(C#N)c(N)nc2ccccc12	**2**{*243*}	CCc1nocc1C=O	**3**{*7*}	[C-]#[N+]C1CCOCC1	0.0	0
471	**4**{*298*,*244*,*62*}	COC(=O)C1(CCCC1)NC1=C(N=C2C=CC3=C(N=C(Cl)C=C3)N12)C1=CN(C)C(=O)C=C1 |c:15,22,34,t:11,13,17,19,28|	Z8810903004	**1**{*298*}	Nc1ccc2ccc(Cl)nc2n1	**2**{*244*}	Cn1cc(C=O)ccc1=O	**3**{*62*}	COC(=O)C1(CCCC1)[N+]#[C-]	0.0	0
472	**4**{*125*,*245*,*31*}	COC(=O)C1=CSC(=C1)C1=C(NCCSC)N2C=C(CCO)C=CC2=N1 |c:7,10,23,26,t:4,18|	Z8810902968	**1**{*125*}	Nc1ccc(CCO)cn1	**2**{*245*}	COC(=O)c1csc(C=O)c1	**3**{*31*}	CSCC[N+]#[C-]	0.0	0
473	**4**{*260*,*246*,*14*}	COCCNC1=C(N=C2N1C=CC=C2N1CCCC1)C1CC11CCCOC1 |c:7,11,13,t:5|	Z8810902964	**1**{*260*}	Nc1ncccc1N2CCCC2	**2**{*246*}	O=CC1CC21CCCOC2	**3**{*14*}	COCC[N+]#[C-]	0.0	0
474	**4**{*101*,*247*,*6*}	CCOC1CC(C1)C1=C(NCCCC(=O)OCC)N2C=C(CO)C=C(Cl)C2=N1 |c:8,28,t:20,24|	Z8810902959	**1**{*101*}	Nc1ncc(CO)cc1Cl	**2**{*247*}	CCOC1CC(C1)C=O	**3**{*6*}	CCOC(=O)CCC[N+]#[C-]	0.0	0
475	**4**{*24*,*27*,*13*}	COC1=CC=C(CNC2=C(N=C3N2C=CN=C3Cl)C2=NN(C)C(C)=N2)C=C1 |c:10,14,16,25,28,t:2,4,8,20|	Z8798985028	**1**{*24*}	Nc1nccnc1Cl	**2**{*27*}	Cc1nc(C=O)nn1C	**3**{*13*}	COc1ccc(C[N+]#[C-])cc1	0.0	0
476	**4**{*22*,*232*,*9*}	CNC1=C(N=C2C=CC(Cl)=NN12)C1=NOCC1 |c:6,9,t:2,4,14|	Z8786958907	**1**{*22*}	Nc1ccc(Cl)nn1	**2**{*232*}	O=CC1=NOCC1	**3**{*9*}	C[N+]#[C-]	0.0	0
477	**4**{*299*,*170*,*27*}	CCCCNC1=C(N=C2N1C(OC)=CC=C2[N+]([O-])=O)C1=NN(CC)N=N1 |c:7,13,15,26,t:5,21|	Z8786939735	**1**{*299*}	COc1ccc([N+](=O)[O-])c(N)n1	**2**{*170*}	CCn1nnc(C=O)n1	**3**{*27*}	CCCC[N+]#[C-]	0.0	0
478	**4**{*300*,*97*,*14*}	COCCNC1=C(N=C2N1C1=C(C=CC=C1)N=C2Cl)C1=CC=C(F)C=C1 |c:7,13,15,18,27,t:5,11,22,24|	Z8786917530	**1**{*300*}	Nc1nc2ccccc2nc1Cl	**2**{*97*}	Fc1ccc(C=O)cc1	**3**{*14*}	COCC[N+]#[C-]	0.0	0

479	**4**{*301*,*94*,*7*}	FC1=CC(Cl)=CN2C(NC3CCOCC3)=C(N=C12)C1=NC=CN=C1 |c:4,15,23,25,t:1,17,21|	Z8786838538	**1**{*301*}	Nc1ncc(Cl)cc1F	**2**{*94*}	O=Cc1cnccn1	**3**{*7*}	[C-]#[N+]C1CCOCC1	0.0	0
480	**4**{*241*,*79*,*12*}	BrC1=NN2C(C=C1)=NC(C1=NC=CO1)=C2NCC1CCOC1 |c:5,7,12,15,t:1,10|	Z8781341779	**1**{*241*}	Nc1ccc(Br)nn1	**2**{*79*}	O=Cc1ncco1	**3**{*12*}	[C-]#[N+]CC1COC1	0.0	0
481	**4**{*302*,*49*,*38*}	CC1=NNC=C1C1=C(NC2=CC3=C(OCO3)C=C2)N2N=CC(=CC2=N1)C#N |c:4,7,18,22,24,27,t:1,10,12|	Z8781341777	**1**{*302*}	Nc1cc(C#N)cnn1	**2**{*49*}	Cc1n[nH]cc1C=O	**3**{*38*}	[C-]#[N+]c1ccc2OCOc2c1	0.0	0
482	**4**{*303*,*94*,*17*}	C(NC1=C(N=C2N1N=CC1=C2C=CC=C1)C1=NC=CN=C1)C1CCOCC1 |c:4,8,10,13,15,20,22,t:2,18|	Z8784060785	**1**{*303*}	Nc1nncc2ccccc12	**2**{*94*}	O=Cc1cnccn1	**3**{*17*}	[C-]#[N+]CC1COCC1	0.0	0
483	**4**{*304*,*10*,*6*}	CCOC(=O)CCCNC1=C(N=C2N1C=C(Br)C=C2C#N)C1=NC=CC(C)=C1 |c:11,18,25,28,t:9,15,23|	Z8780121875	**1**{*304*}	Nc1ncc(Br)cc1C#N	**2**{*10*}	Cc1ccnc(C=O)c1	**3**{*6*}	CCOC(=O)CCC[N+]#[C-]	0.0	0
484	**4**{*196*,*118*,*17*}	CC1=CN2C(NCC3CCOCC3)=C(N=C2C(=C1)C#N)C1=CC(C)=NC=C1 |c:13,15,18,26,28,t:1,23|	Z8780121777	**1**{*196*}	Cc1cnc(N)c(C#N)c1	**2**{*118*}	Cc1cc(C=O)ccn1	**3**{*17*}	[C-]#[N+]CC1COCC1	0.0	0
485	**4**{*305*,*14*,*14*}	CCN1CCN(CC1)C(=O)C1=CN2C(C=C1)=NC(=C2NCCOC)C1=CC=C(C)C=C1 |c:15,17,19,32,t:11,27,29|	Z8776690327	**1**{*305*}	Cl.Cl.CCN1CCN(CC1)C(=O)c2ccc(N)nc2	**2**{*14*}	Cc1ccc(C=O)cc1	**3**{*14*}	COCC[N+]#[C-]	0.0	0
486	**4**{*102*,*14*,*58*}	CN(C)C(=O)C1=CC2=NC(=C(NC3(C)CC3)N2C=C1)C1=CC=C(C)C=C1 |c:19,27,t:5,7,9,22,24|	Z8776690342	**1**{*102*}	CN(C)C(=O)c1ccnc(N)c1	**2**{*14*}	Cc1ccc(C=O)cc1	**3**{*58*}	CC1(CC1)[N+]#[C-]	0.0	0
487	**4**{*4*,*14*,*14*}	COCCNC1=C(N=C2SC=NN12)C1=CC=C(C)C=C1 |c:10,20,t:5,7,15,17|	Z8711891697	**1**{*4*}	Nc1nncs1	**2**{*14*}	Cc1ccc(C=O)cc1	**3**{*14*}	COCC[N+]#[C-]	0.0	0
488	**4**{*6*,*15*,*14*}	COCCNC1=C(N=C2N=CC=CN12)C1=CC=C(OC)C=C1 |c:9,11,22,t:5,7,16,18|	Z8700285979	**1**{*6*}	Nc1ncccn1	**2**{*15*}	COc1ccc(C=O)cc1	**3**{*14*}	COCC[N+]#[C-]	0.0	0
489	**4**{*81*,*15*,*58*}	COC1=CC=C(C=C1)C1=C(NC2(C)CC2)N2C=CN=CC2=N1 |c:4,6,9,18,20,23,t:2|	Z8712581562	**1**{*81*}	Nc1cnccn1	**2**{*15*}	COc1ccc(C=O)cc1	**3**{*58*}	CC1(CC1)[N+]#[C-]	0.0	0
490	**4**{*223*,*248*,*34*}	COC1=CC(Cl)=C(C2=C(NCC3CCCO3)N3N=C(C=CC3=N2)C(N)=O)C(F)=C1 |c:7,18,20,23,30,t:2,5|	Z8801681817	**1**{*223*}	NC(=O)c1ccc(N)nn1	**2**{*248*}	COc1cc(F)c(C=O)c(Cl)c1	**3**{*34*}	[C-]#[N+]CC1CCO1	0.0	0
491	**4**{*306*,*118*,*17*}	CC1=NC=CC(=C1)C1=C(NCC2CCOCC2)N2C=CC(=C(Cl)C2=N1)C(F)(F)F |c:3,5,8,20,26,t:1,22|	Z8797785420	**1**{*306*}	Nc1nccc(c1Cl)C(F)(F)F	**2**{*118*}	Cc1cc(C=O)ccn1	**3**{*17*}	[C-]#[N+]CC1COCC1	0.0	0

492	4{307,118,14}	COCCNC1=C(N=C2N1C=C(Br)C1=C2C=CC=C1)C1=CC(C)=NC=C1 |c:7,14,17,19,25,27,t:5,11,22|	Z8797779551	1{307}	Nc1ncc(Br)c2ccccc12	2{118}	Cc1cc(C=O)ccn1	3{14}	COCC[N+]#[C-]	0.0	0
493	4{308,18,58}	CCOC1=CC2=NC(C3=CN(CCO)N=C3)=C(NC3(C)CC3)N2C=C1 |c:14,26,t:3,5,8,16|	Z8778278040	1{308}	CCOc1ccnc(N)c1	2{18}	OCCn1cc(C=O)cn1	3{58}	CC1(CC1)[N+]#[C-]	0.0	0
494	4{309,249,14}	CCN1N=CC=C1C1=C(NCCOC)N2C(C=NC=C2C(F)(F)F)=N1 |c:3,5,8,17,19,25|	Z8778278031	1{309}	Nc1cncc(n1)C(F)(F)F	2{249}	CCn1nccc1C=O	3{14}	COCC[N+]#[C-]	0.0	0
495	4{210,167,65}	FC1=CC=C(Br)C2=NC(C3=NN=CN3)=C(NC3CCCC3)N12 |c:11,t:1,3,6,9,14|	Z8803896971	1{210}	Nc1nc(F)ccc1Br	2{167}	O=Cc1nnc[nH]1	3{65}	[C-]#[N+]C1CCCC1	0.0	0
496	4{310,250,66}	CC(NC1=C(N=C2N1C=CC1=C2N(C)C(=O)N1C)C1=CN(C)N=C1Br)C1=CC=CC=C1 |c:5,9,11,25,31,33,t:3,21,29|	Z8803896958	1{310}	Cn1c(=O)n(C)c2c(N)nccc12	2{250}	Cn1cc(C=O)c(Br)n1	3{66}	CC([N+]#[C-])c1ccccc1	0.0	0
497	4{119,251,10}	N#CC1=CN2C(NC3CCOC3)=C(N=C2C=C1)C1=CC(=NN1)C1=CC=NC=C1 |c:12,14,17,22,28,30,t:2,20,26|	Z8803896950	1{119}	Nc1ccc(C#N)cn1	2{251}	O=Cc1cc(n[nH]1)c2ccncc2	3{10}	[C-]#[N+]C1CCOC1	0.0	0
498	4{77,13,1}	CC1=C(N=NN1)C1=C(NC2CC2)N2C=C(C=CC2=N1)C1=CN=CC=C1 |c:3,7,15,17,20,25,27,t:1,23|	Z8803896941	1{77}	Nc1ccc(cn1)c2cccnc2	2{13}	Cc1[nH]nnc1C=O	3{1}	[C-]#[N+]C1CC1	0.0	0
499	4{176,252,14}	COCCNC1=C(N=C2N1C=CN=C2N1CCCC1)C1=CC(F)=C(C=C1)C#N |c:7,11,13,25,27,t:5,22|	Z8803896922	1{176}	Nc1nccnc1N2CCCC2	2{252}	Fc1cc(C=O)ccc1C#N	3{14}	COCC[N+]#[C-]	0.0	0
500	4{229,253,68}	CCC1CC1C1=C(NC2CCCCC2)N2C=C(C=CC2=N1)S(O)(=O)=O |c:6,17,19,22|	Z8803896954	1{229}	Nc1ccc(cn1)S(=O)(=O)O	2{253}	CCC1CC1C=O	3{68}	[C-]#[N+]C1CCCCC1	0.0	0
501	4{164,104,24}	COC1=CC(CNC2=C(N=C3C=C(CN(C)C)C=CN23)C2=NC(C)=CO2)=CC=C1 |c:17,25,28,30,t:2,7,9,11,22|	Z8808559890	1{164}	CN(C)Cc1ccnc(N)c1	2{104}	Cc1coc(C=O)n1	3{24}	COc1cccc(C[N+]#[C-])c1	0.0	0
502	4{261,254,39}	CCOCCNC1=C(CCCCC#C)N=C2C=CC(=CN12)N1CCC(CC1)N(C)C |c:6,16,18,t:14|	Z8808559879	1{261}	CN(C)C1CCN(CC1)c2ccc(N)nc2	2{254}	O=CCCCCC#C	3{39}	CCOCC[N+]#[C-]	0.0	0
503	4{297,89,7}	CCC1=NC=C(C=N1)C1=C(NC2CCOCC2)N2C(=N1)C(C#N)=C(C)C1=C2C=CC=C1 |c:4,6,9,20,28,31,33,t:2,25|	Z8808559866	1{297}	Cc1c(C#N)c(N)nc2ccccc12	2{89}	CCc1ncc(C=O)cn1	3{7}	[C-]#[N+]C1CCOCC1	0.0	0
504	4{311,255,10}	FC1=CC2=NC(=C(NC3CCOC3)N2C=C1)C1=CC2=C(NC=N2)C=C1 |c:16,24,27,t:1,3,5,19,21|	Z8808559856	1{311}	Nc1cc(F)ccn1	2{255}	O=Cc1ccc2[nH]cnc2c1	3{10}	[C-]#[N+]C1CCOC1	0.0	0

<table>
<tr><th></th><th></th><th></th><th></th><th></th><th></th><th></th><th></th><th></th><th></th><th></th><th></th></tr>
<tr><td>505</td><td>4{312,256,39}</td><td>CCOCCNC1=C(N=C2C=CC3=C(NC(=O)NC3=O)N12)C1=CN(CCCl)N=C1 |c:10,30,t:6,8,12,24|</td><td>Z8810903024</td><td>1{312}</td><td>Nc1ccc2c(=O)[nH]c(=O)[nH]c2n1</td><td>2{256}</td><td>ClCCn1cc(C=O)cn1</td><td>3{39}</td><td>CCOCC[N+]#[C-]</td><td>0.0</td><td>0</td></tr>
<tr><td>506</td><td>4{151,257,9}</td><td>CNC1=C(N=C2C=NC=C(Cl)N12)C1CC(C1)NC(=O)OC(C)(C)C |c:6,t:2,4,8|</td><td>Z8810902999</td><td>1{151}</td><td>Nc1cncc(Cl)n1</td><td>2{257}</td><td>CC(C)(C)OC(=O)NC1CC(C1)C=O</td><td>3{9}</td><td>C[N+]#[C-]</td><td>0.0</td><td>0</td></tr>
<tr><td>507</td><td>4{271,258,67}</td><td>CCC(C)NC1=C(N=C2C=CC(C(=O)OC)=C(F)N12)C1=NN=C(S1)C1CC1 |c:9,23,t:5,7,15,21|</td><td>Z8810902994</td><td>1{271}</td><td>COC(=O)c1ccc(N)nc1F</td><td>2{258}</td><td>O=Cc1nnc(s1)C2CC2</td><td>3{67}</td><td>CCC(C)[N+]#[C-]</td><td>0.0</td><td>0</td></tr>
<tr><td>508</td><td>4{168,205,21}</td><td>COC(=O)C1=C2COCCN2C(=C1)C1=C(NCC2CC2)N2C(C=NC=C2C#N)=N1 |c:4,12,15,25,27,31|</td><td>Z8810902955</td><td>1{168}</td><td>Nc1cncc(C#N)n1</td><td>2{205}</td><td>COC(=O)c1cc(C=O)n2CCOCc12</td><td>3{21}</td><td>[C-]#[N+]CC1CC1</td><td>0.0</td><td>0</td></tr>
<tr><td>509</td><td>4{109,259,34}</td><td>COC1=C(C=C(C=C1)C1=CN=CN=C1)C1=C(NCC2CCCO2)N2C(C=CC=C2S(N)(=O)=O)=N1 |c:4,6,11,13,16,28,30,36,t:2,9|</td><td>Z8798985063</td><td>1{109}</td><td>Cl.Nc1cccc(n1)S(=O)(=O)N</td><td>2{259}</td><td>COc1ccc(cc1C=O)c2cncnc2</td><td>3{34}</td><td>[C-]#[N+]CC1CCCO1</td><td>0.0</td><td>0</td></tr>
<tr><td>510</td><td>4{313,260,14}</td><td>COCCNC1=C(N=C2N1C1=C(CCCC1)C=C2C#N)C1=NOC(C)=C1 |c:7,18,27,t:5,11,23|</td><td>Z8798985060</td><td>1{313}</td><td>Nc1nc2CCCCc2cc1C#N</td><td>2{260}</td><td>Cc1cc(C=O)no1</td><td>3{14}</td><td>COCC[N+]#[C-]</td><td>0.0</td><td>0</td></tr>
<tr><td>511</td><td>4{271,261,44}</td><td>COC(=O)C1=C(F)N2C(NCCCCNC(=O)OC(C)(C)C)=C(N=C2C=C1)C1=NC=C(C)N1C |c:4,21,23,26,t:29,31|</td><td>Z8798985025</td><td>1{271}</td><td>COC(=O)c1ccc(N)nc1F</td><td>2{261}</td><td>Cc1cnc(C=O)n1C</td><td>3{44}</td><td>CC(C)(C)OC(=O)NCCCC[N+]#[C-]</td><td>0.0</td><td>0</td></tr>
<tr><td>512</td><td>4{314,262,22}</td><td>CCC1=NN(C)C(=C1Cl)C1=C(NC2COC2)N2C(C=CC=C2CC(=O)OC)=N1 |c:6,10,20,22,29,t:2|</td><td>Z8786958839</td><td>1{314}</td><td>COC(=O)Cc1cccc(N)n1</td><td>2{262}</td><td>CCc1nn(C)c(C=O)c1Cl</td><td>3{22}</td><td>[C-]#[N+]C1COC1</td><td>0.0</td><td>0</td></tr>
<tr><td>513</td><td>4{23,263,46}</td><td>CC(=C)CNC1=C(N=C2C=NC=C(Br)N12)C1=CN=C2C=NC=CN12 |c:9,21,23,t:5,7,11,17,19|</td><td>Z8786958792</td><td>1{23}</td><td>Nc1cncc(Br)n1</td><td>2{263}</td><td>O=Cc1cnc2cnccn12</td><td>3{46}</td><td>CC(=C)C[N+]#[C-]</td><td>0.0</td><td>0</td></tr>
<tr><td>514</td><td>4{315,264,14}</td><td>COCCNC1=C(N=C2C=NC(Br)=C(F)N12)C1=NC(Br)=CN1 |c:9,21,t:5,7,12,18|</td><td>Z8781341801</td><td>1{315}</td><td>Nc1cnc(Br)c(F)n1</td><td>2{264}</td><td>Brc1c[nH]c(C=O)n1</td><td>3{14}</td><td>COCC[N+]#[C-]</td><td>0.0</td><td>0</td></tr>
<tr><td>515</td><td>4{316,14,27}</td><td>CCCCNC1=C(N=C2C=CC=C(N12)C(N)=N)C1=CC=C(C)C=C1 |c:9,11,24,t:5,7,19,21|</td><td>Z8784060088</td><td>1{316}</td><td>Cl.Cl.NC(=N)c1cccc(N)n1</td><td>2{14}</td><td>Cc1ccc(C=O)cc1</td><td>3{27}</td><td>CCCC[N+]#[C-]</td><td>0.0</td><td>0</td></tr>
<tr><td>516</td><td>4{317,29,20}</td><td>CCN1C=CC(=N1)C1=C(NC2=C(C)C=C(OC)C=C2)N2C=C(C=C(C2=N1)C(F)(F)F)C(F)(F)F |c:3,5,8,11,18,22,24,27,t:14|</td><td>Z8780122386</td><td>1{317}</td><td>Nc1ncc(cc1C(F)(F)F)C(F)(F)F</td><td>2{29}</td><td>CCn1ccc(C=O)n1</td><td>3{20}</td><td>COc1ccc([N+]#[C-])c(C)c1</td><td>0.0</td><td>0</td></tr>
</table>

<table>
<tr><th></th><th></th><th></th><th></th><th></th><th></th><th></th><th></th><th></th><th></th><th></th><th></th></tr>
<tr><td>517</td><td>4{13,14,27}</td><td>CCCCNC1=C(N=C2C=C(C)N=C(C)N12)C1=CC=C(C)C=C1 |c:23,t:5,7,9,12,18,20|</td><td>Z8711876248</td><td>1{13}</td><td>Cc1cc(N)nc(C)n1</td><td>2{14}</td><td>Cc1ccc(C=O)cc1</td><td>3{27}</td><td>CCCC[N+]#[C-]</td><td>0.0</td><td>0</td></tr>
<tr><td>518</td><td>4{169,14,14}</td><td>CNC(=O)C1=NN2C(C=C1)=NC(=C2NCCOC)C1=CC=C(C)C=C1 |c:8,10,12,25,t:4,20,22|</td><td>Z8741907038</td><td>1{169}</td><td>CNC(=O)c1ccc(N)nn1</td><td>2{14}</td><td>Cc1ccc(C=O)cc1</td><td>3{14}</td><td>COCC[N+]#[C-]</td><td>0.0</td><td>0</td></tr>
<tr><td>519</td><td>4{267,177,34}</td><td>COC(=O)C1CC(CC2=C(NCC3CCCO3)N3C(C=CC=C3C#C)=N2)C1 |c:8,20,22,26|</td><td>Z8801681841</td><td>1{267}</td><td>Nc1cccc(C#C)n1</td><td>2{177}</td><td>COC(=O)C1CC(CC=O)C1</td><td>3{34}</td><td>[C-]#[N+]CC1CCCO1</td><td>0.0</td><td>0</td></tr>
<tr><td>520</td><td>4{229,265,64}</td><td>COC(=O)[C@@H]1CC[C@@H](C1)NC1=C(N=C2C=CC(=CN12)S(O)(=O)=O)C1CCCCC1 |&1:4,7,r,c:15,17,t:11,13|</td><td>Z8803896964</td><td>1{229}</td><td>Nc1ccc(cn1)S(=O)(=O)O</td><td>2{265}</td><td>O=CC1CCCCC1</td><td>3{64}</td><td>COC(=O)[C@@H]1CC[C@@H](C1)[N+]#[C-] |&1:4,7,r|</td><td>0.0</td><td>0</td></tr>
<tr><td>521</td><td>4{38,266,9}</td><td>CNC1=C(N=C2C=C(C=CN12)C#N)C1=CC(=CC=C1)C1=CN=C(C=C1)C#N |c:6,8,17,19,24,26,t:2,4,15,22|</td><td>Z8803896949</td><td>1{38}</td><td>Nc1cc(C#N)ccn1</td><td>2{266}</td><td>O=Cc1cccc(c1)c2ccc(C#N)nc2</td><td>3{9}</td><td>C[N+]#[C-]</td><td>0.0</td><td>0</td></tr>
<tr><td>522</td><td>4{48,267,15}</td><td>CCNC1=C(N=C2C=C(C=CN12)S(C)(=O)=O)[C@H](CCC(=O)OC(C)(C)C)NC(=O)OC(C)(C)C |c:7,9,t:3,5|</td><td>Z8803896934</td><td>1{48}</td><td>CS(=O)(=O)c1ccnc(N)c1</td><td>2{267}</td><td>CC(C)(C)OC(=O)CC[C@H](NC(=O)OC(C)(C)C)C=O</td><td>3{15}</td><td>CC[N+]#[C-]</td><td>0.0</td><td>0</td></tr>
<tr><td>523</td><td>4{238,268,7}</td><td>CC[C@@H](NC(=O)OC(C)(C)C)C1=C(NC2CCOCC2)N2C=CC(OC)=C(C#N)C2=N1 |c:11,22,31,t:26|</td><td>Z8808559880</td><td>1{238}</td><td>COc1ccnc(N)c1C#N</td><td>2{268}</td><td>CC[C@@H](NC(=O)OC(C)(C)C)C=O</td><td>3{7}</td><td>[C-]#[N+]C1CCOCC1</td><td>0.0</td><td>0</td></tr>
<tr><td>524</td><td>4{31,269,22}</td><td>C1CC(C1)C1=CC(=NN1)C1=C(NC2COC2)N2C3=C(OCC3)C=CC2=N1 |c:7,11,26,29,t:5,20|</td><td>Z8810903036</td><td>1{31}</td><td>Nc1ccc2OCCc2n1</td><td>2{269}</td><td>O=Cc1cc([nH]n1)C2CCC2</td><td>3{22}</td><td>[C-]#[N+]C1COC1</td><td>0.0</td><td>0</td></tr>
<tr><td>525</td><td>4{145,270,15}</td><td>CCNC1=C(N=C2C=CC=C(N12)S(C)(=O)=O)C1[C@H]2CC[C@H](C2)[C@@H]1NC(=O)OC(C)(C)C |&1:17,20,22,r,c:7,9,t:3,5|</td><td>Z8810903031</td><td>1{145}</td><td>CS(=O)(=O)c1cccc(N)n1</td><td>2{270}</td><td>CC(C)(C)OC(=O)N[C@@H]1C(C=O)[C@H]2CC[C@@H]1C2 |&1:8,12,15,r|</td><td>3{15}</td><td>CC[N+]#[C-]</td><td>0.0</td><td>0</td></tr>
</table>

526	**4**{*258*,*271*,*67*}	CCC(C)NC1=C(N=C2C=CC3=C(N=CC=N3)N12)C1=NN(C)N=N1 |c:9,13,15,25,t:5,7,11,21|	Z8810902979	**1**{*258*}	Nc1ccc2nccnc2n1	**2**{*271*}	Cn1nnc(C=O)n1	**3**{*67*}	CCC(C)[N+]#[C-]	0.0	0
527	**4**{*211*,*3*,*66*}	CC(NC1=C(N=C2N1C=CC=C2C1=NN=NN1)C1=NC=C(C=C1)C#N)C1=CC=CC=C1 |c:5,9,11,16,22,24,31,33,t:3,14,20,29|	Z8798985051	**1**{*211*}	Nc1ncccc1c2nnn[nH]2	**2**{*3*}	O=Cc1ccc(C#N)cn1	**3**{*66*}	CC([N+]#[C-])c1ccccc1	0.0	0
528	**4**{*275*,*182*,*14*}	COCCNC1=C(N=C2N1C(Cl)=CC=C2F)C1=NC=CN1CC#C |c:7,12,14,20,t:5,18|	Z8798985050	**1**{*275*}	Nc1nc(Cl)ccc1F	**2**{*182*}	O=Cc1nccn1CC#C	**3**{*14*}	COCC[N+]#[C-]	0.0	0
529	**4**{*318*,*273*,*12*}	COC(=O)C1=CN2C(NCC3CCOC3)=C(N=C2C(OC)=N1)C1=C(Br)N(C)N=C1C |c:15,17,22,25,30,t:4|	Z8798985049	**1**{*318*}	COC(=O)c1cnc(N)c(OC)n1	**2**{*273*}	Cc1nn(C)c(Br)c1C=O	**3**{*12*}	[C-]#[N+]CC1CCOC1	0.0	0
530	**4**{*198*,*118*,*31*}	CSCCNC1=C(N=C2C=NC(C#C)=C(Cl)N12)C1=CC(C)=NC=C1 |c:9,22,24,t:5,7,13,19|	Z8798985041	**1**{*198*}	Nc1cnc(C#C)c(Cl)n1	**2**{*118*}	Cc1cc(C=O)ccn1	**3**{*31*}	CSCC[N+]#[C-]	0.0	0
531	**4**{*35*,*129*,*9*}	CNC1=C(N=C2C=CC(C)=CN12)C1=CC=CC2=NC=NN12 |c:6,9,16,20,t:2,4,14,18|	Z8798985036	**1**{*35*}	Cc1ccc(N)nc1	**2**{*129*}	O=Cc1cccc2ncnn12	**3**{*9*}	C[N+]#[C-]	0.0	0
532	**4**{*314*,*231*,*4*}	COC(=O)CC1=CC=CC2=NC(C3=NC4=C(CC(C)CC4)S3)=C(NCC3=NN(C)N=N3)N12 |c:7,31,t:5,9,12,14,23,27|	Z8786958826	**1**{*314*}	COC(=O)Cc1cccc(N)n1	**2**{*231*}	CC1CCc2nc(C=O)sc2C1	**3**{*4*}	Cn1nnc(C[N+]#[C-])n1	0.0	0
533	**4**{*25*,*274*,*25*}	CN(C)C1=CC=CC2=NC(CC3(CCOCC3)C#N)=C(NCCCF)N12 |c:5,t:3,7,19|	Z8781341785	**1**{*25*}	CN(C)c1cccc(N)n1	**2**{*274*}	O=CCC1(CCOCC1)C#N	**3**{*25*}	FCCC[N+]#[C-]	0.0	0
534	**4**{*319*,*275*,*25*}	CN1C=C(C)N=C1C1=C(NCCCF)N2C(C=CC(Br)=C2C#N)=N1 |c:5,8,17,20,24,t:2|	Z8781341771	**1**{*319*}	Nc1ccc(Br)c(C#N)n1	**2**{*275*}	Cc1cn(C)c(C=O)n1	**3**{*25*}	FCCC[N+]#[C-]	0.0	0
535	**4**{*320*,*29*,*7*}	CCN1C=CC(=N1)C1=C(NC2CCOCC2)N2C=CN3N=CC(=C3C2=N1)[N+]([O-])=O |c:3,5,8,19,22,24,28|	Z8784060976	**1**{*320*}	Nc1nccn2ncc([N+](=O)[O-])c12	**2**{*29*}	CCn1ccc(C=O)n1	**3**{*7*}	[C-]#[N+]C1CCOCC1	0.0	0
536	**4**{*321*,*80*,*20*}	COC1=CC(C)=C(NC2=C(N=C3N2C=CC2=C3C=NC=N2)C2=NC=C(C)S2)C=C1 |c:10,14,16,19,21,31,t:2,5,8,24,26|	Z8784060875	**1**{*321*}	Nc1nccc2ncncc12	**2**{*80*}	Cc1cnc(C=O)s1	**3**{*20*}	COc1ccc([N+]#[C-])c(C)c1	0.0	0
537	**4**{*260*,*69*,*14*}	COCCNC1=C(N=C2N1C=CC=C2N1CCCC1)C1=NN(C)C(C)=C1 |c:7,11,13,27,t:5,22|	Z8784060346	**1**{*260*}	Nc1ncccc1N2CCCC2	**2**{*69*}	Cc1cc(C=O)nn1C	**3**{*14*}	COCC[N+]#[C-]	0.0	0
538	**4**{*322*,*58*,*14*}	COCCNC1=C(N=C2N1C=CN=C2Br)C1=NSN=C1 |c:7,11,13,20,t:5,17|	Z8780121564	**1**{*322*}	Nc1nccnc1Br	**2**{*58*}	O=Cc1cnsn1	**3**{*14*}	COCC[N+]#[C-]	0.0	0
539	**4**{*22*,*15*,*9*}	CNC1=C(N=C2C=CC(Cl)=NN12)C1=CC=C(OC)C=C1 |c:6,9,20,t:2,4,14,16|	Z8776690345	**1**{*22*}	Nc1ccc(Cl)nn1	**2**{*15*}	COc1ccc(C=O)cc1	**3**{*9*}	C[N+]#[C-]	0.0	0

540	**4**{*323*,*14*,*17*}	CC1=CC=C(C=C1)C1=C(NCC2CCOCC2)N2C(C=CC=C2O)=N1 |c:3,5,8,21,23,26,t:1|	Z8711876680	**1**{*323*}	Nc1cccc(O)n1	**2**{*14*}	Cc1ccc(C=O)cc1	**3**{*17*}	[C-]#[N+]CC1CCOCC1	0.0	0
541	**4**{*12*,*15*,*17*}	CCC1=CC2=NC(=C(NCC3CCOCC3)N2C=N1)C1=CC=C(OC)C=C1 |c:19,28,t:2,4,6,22,24|	Z8711876287	**1**{*12*}	CCc1cc(N)ncn1	**2**{*15*}	COc1ccc(C=O)cc1	**3**{*17*}	[C-]#[N+]CC1CCOCC1	0.0	0
542	**4**{*16*,*22*,*17*}	FC1=CN=CN2C(NCC3CCOCC3)=C(N=C12)C1=CC=CC=C1 |c:3,15,23,25,t:1,17,21|	Z8711876147	**1**{*16*}	Nc1ncncc1F	**2**{*22*}	O=Cc1ccccc1	**3**{*17*}	[C-]#[N+]CC1CCOCC1	0.0	0
543	**4**{*7*,*22*,*54*}	CC1=CN2C(NC3=CC=CC=C3)=C(N=C2N=C1)C1=CC=CC=C1 |c:8,10,12,14,17,22,24,t:1,6,20|	Z8711872483	**1**{*7*}	Cc1cnc(N)nc1	**2**{*22*}	O=Cc1ccccc1	**3**{*54*}	[C-]#[N+]c1ccccc1	0.0	0
544	**4**{*11*,*15*,*14*}	COCCNC1=C(N=C2N=C(Cl)C=C(Cl)N12)C1=CC=C(OC)C=C1 |c:24,t:5,7,9,12,18,20|	Z8711872231	**1**{*11*}	Nc1nc(Cl)cc(Cl)n1	**2**{*15*}	COc1ccc(C=O)cc1	**3**{*14*}	COCC[N+]#[C-]	0.0	0
545	**4**{*270*,*26*,*9*}	CNC1=C(CCC2=CC=CC=C2)N=C2C=CC3=NC=NN3N12 |c:2,8,10,15,19,t:6,13,17|	Z8741907041	**1**{*270*}	Nc1ccc2ncnn2n1	**2**{*26*}	O=CCCc1ccccc1	**3**{*9*}	C[N+]#[C-]	0.0	0
546	**4**{*324*,*14*,*11*}	CC1=CC=C(C=C1)C1=C(NCC2=CC(Br)=CC=C2)N2C=CC(N)=CC2=N1 |c:3,5,8,15,17,21,24,27,t:1,12|	Z8741906951	**1**{*324*}	Cl.Nc1ccnc(N)c1	**2**{*14*}	Cc1ccc(C=O)cc1	**3**{*11*}	Brc1cccc(C[N+]#[C-])c1	0.0	0
547	**4**{*325*,*22*,*20*}	COC1=CC(C)=C(NC2=C(N=C3C=C(O)C=CN23)C2=CC=CC=C2)C=C1 |c:15,22,24,27,t:2,5,8,10,12,20|	Z8741906950	**1**{*325*}	Nc1cc(O)ccn1	**2**{*22*}	O=Cc1ccccc1	**3**{*20*}	COc1ccc([N+]#[C-])c(C)c1	0.0	0
548	**4**{*326*,*276*,*47*}	COC(OC)C1=C(NC(C)C)N2C=C(Cl)C(Br)=CC2=N1 |c:5,16,19,t:12|	Z8801681844	**1**{*326*}	Nc1cc(Br)c(Cl)cn1	**2**{*276*}	COC(OC)C=O	**3**{*47*}	CC(C)[N+]#[C-]	0.0	0
549	**4**{*240*,*277*,*37*}	COC(=O)C1CC(C1)NC1=C(CC(NC(=O)OC(C)(C)C)C2CC2)N=C2C=CC(=CN12)C1=NNN=N1 |c:10,28,30,38,t:26,35|	Z8801681828	**1**{*240*}	Nc1ccc(cn1)c2nn[nH]n2	**2**{*277*}	CC(C)(C)OC(=O)NC(CC=O)C1CC1	**3**{*37*}	COC(=O)C1CC(C1)[N+]#[C-]	0.0	0
550	**4**{*327*,*170*,*27*}	CCCCNC1=C(N=C2N1C=CC(C(=O)OC)=C2F)C1=NN(CC)N=N1 |c:7,11,17,26,t:5,21|	Z8797787109	**1**{*327*}	COC(=O)c1ccnc(N)c1F	**2**{*170*}	CCn1nnc(C=O)n1	**3**{*27*}	CCCC[N+]#[C-]	0.0	0
551	**4**{*328*,*41*,*17*}	BrC1=C2C=CNC2=CN2C(NCC3CCOCC3)=C(N=C12)C1=NC=CC=C1 |c:1,3,7,19,27,29,t:21,25|	Z8797775508	**1**{*328*}	Nc1ncc2[nH]ccc2c1Br	**2**{*41*}	O=Cc1ccccn1	**3**{*17*}	[C-]#[N+]CC1CCOCC1	0.0	0
552	**4**{*113*,*278*,*6*}	CCOC(=O)CCCNC1=C(N=C2N1C=CC=C2N1CCOCC1)C1=C(Br)N=NN1C |c:11,15,17,27,30,t:9|	Z8803896969	**1**{*113*}	Nc1ncccc1N2CCOCC2	**2**{*278*}	Cn1nnc(Br)c1C=O	**3**{*6*}	CCOC(=O)CCC[N+]#[C-]	0.0	0

553	**4**{*329*,*107*,*7*}	COC1=CC=C(C)C2=NC(=C(NC3CCOCC3)N12)C1=NC(C)=CS1 |c:25,t:2,4,7,9,22|	Z8803896940	**1**{*329*}	Cl.COc1ccc(C)c(N)n1	**2**{*107*}	Cc1csc(C=O)n1	**3**{*7*}	[C-]#[N+]C1CCOCC1	0.0	0
554	**4**{*154*,*279*,*33*}	COC1=C(CNC2=C(N=C3C=C(C=CN23)[N+]([O-])=O)[C@@H]2CCCO[C@H]2C2=NC=CN2C)C=CC=C1 |&1:18,23,r,c:2,10,12,29,34,36,t:6,8,27|	Z8808559896	**1**{*154*}	Nc1cc(ccn1)[N+](=O)[O-]	**2**{*279*}	Cn1ccnc1[C@@H]2OCCC[C@H]2C=O |&1:6,11,r|	**3**{*33*}	COc1ccccc1C[N+]#[C-]	0.0	0
555	**4**{*330*,*250*,*1*}	CN(C)S(=O)(=O)C1=CN2C(C=C1)=NC(=C2NC1CC1)C1=CN(C)N=C1Br |c:10,12,14,26,t:6,22|	Z8808559863	**1**{*330*}	CN(C)S(=O)(=O)c1ccc(N)nc1	**2**{*250*}	Cn1cc(C=O)c(Br)n1	**3**{*1*}	[C-]#[N+]C1CC1	0.0	0
556	**4**{*331*,*280*,*49*}	CN(C)CCN(C)CC1=CN2C(NC3CC(F)(F)C3)=C(N=C2C=C1)C1=NSC(=C1)[N+]([O-])=O |c:19,21,24,30,t:8,27|	Z8810903020	**1**{*331*}	Cl.Cl.Cl.CN(C)CCN(C)Cc1ccc(N)nc1	**2**{*280*}	[O-][N+](=O)c1cc(C=O)ns1	**3**{*49*}	FC1(F)CC(C1)[N+]#[C-]	0.0	0
557	**4**{*332*,*281*,*21*}	CCC1=CC=CN2C(NCC3CC3)=C(N=C12)C1=NN=C(C=C1)C(=O)OC |c:4,13,21,23,t:2,15,19|	Z8810902965	**1**{*332*}	CCc1cccnc1N	**2**{*281*}	COC(=O)c1ccc(C=O)nn1	**3**{*21*}	[C-]#[N+]CC1CC1	0.0	0
558	**4**{*41*,*266*,*9*}	CNC1=C(N=C2C=NC(C)=CN12)C1=CC(=CC=C1)C1=CN=C(C=C1)C#N |c:6,9,16,18,23,25,t:2,4,14,21|	Z8810902963	**1**{*41*}	Cc1cnc(N)cn1	**2**{*266*}	O=Cc1cccc(c1)c2ccc(C#N)nc2	**3**{*9*}	C[N+]#[C-]	0.0	0
559	**4**{*35*,*79*,*9*}	CNC1=C(N=C2C=CC(C)=CN12)C1=NC=CO1 |c:6,9,16,t:2,4,14|	Z8810902951	**1**{*35*}	Cc1ccc(N)nc1	**2**{*79*}	O=Cc1ncco1	**3**{*9*}	C[N+]#[C-]	0.0	0
560	**4**{*256*,*126*,*44*}	COC1=CC(=CC(F)=C1)C1=C(NCCCCNC(=O)OC(C)(C)C)N2N3N=NN=C3C=CC2=N1 |c:4,7,10,27,29,32,35,t:2|	Z8798985064	**1**{*256*}	Nc1ccc2nnnn2n1	**2**{*126*}	COc1cc(F)cc(C=O)c1	**3**{*44*}	CC(C)(C)OC(=O)NCCCC[N+]#[C-]	0.0	0
561	**4**{*333*,*282*,*8*}	COC(=O)C(CC1=CC=CC=C1)NC1=C(N=C2C=CC=C(N12)C1=CC=NC=C1)C1CC2(C1)OCCO2 |c:8,10,18,20,27,29,t:6,14,16,25|	Z8798985059	**1**{*333*}	Nc1cccc(n1)c2ccncc2	**2**{*282*}	O=CC1CC2(C1)OCCO2	**3**{*8*}	COC(=O)C(Cc1ccccc1)[N+]#[C-]	0.0	0
562	**4**{*146*,*283*,*12*}	CS(=O)(=O)C1=CN2C(C=C1)=NC(=C2NCC1CCOC1)C1=C2N=CC=CC2=CC=C1 |c:8,10,12,23,25,27,30,32,t:4|	Z8798985057	**1**{*146*}	CS(=O)(=O)c1ccc(N)nc1	**2**{*283*}	O=Cc1cccc2cccnc12	**3**{*12*}	[C-]#[N+]CC1CCOC1	0.0	0
563	**4**{*192*,*182*,*14*}	COCCNC1=C(N=C2N1C(F)=CC=C2Cl)C1=NC=CN1CC#C |c:7,12,14,20,t:5,18|	Z8798985056	**1**{*192*}	Nc1nc(F)ccc1Cl	**2**{*182*}	O=Cc1nccn1CC#C	**3**{*14*}	COCC[N+]#[C-]	0.0	0

564	**4**{*334*,*284*,*7*}	CC1=C(N=CC=N1)C1=C(NC2CCOCC2)N2C=C3COCCC3=C(C#N)C2=N1 |c:3,5,8,31,t:1,19,26|	Z8798985047	**1**{*334*}	Nc1ncc2COCCc2c1C#N	**2**{*284*}	Cc1nccnc1C=O	**3**{*7*}	[C-]#[N+]C1CCOCC1	0.0	0
565	**4**{*335*,*27*,*58*}	CN1N=C(N=C1C)C1=C(NC2(C)CC2)N2C3=C(NC=C3)C=C(Br)C2=N1 |c:2,4,8,20,27,t:17,23|	Z8786958812	**1**{*335*}	Nc1nc2cc[nH]c2cc1Br	**2**{*27*}	Cc1nc(C=O)nn1C	**3**{*58*}	CC1(CC1)[N+]#[C-]	0.0	0
566	**4**{*336*,*44*,*17*}	CCN1C=C(C=N1)C1=C(NCC2CCOCC2)N2C(=N1)C(Cl)=NC1=C2C=CC(=C1)[N+]([O-])=O |c:3,5,8,20,24,26,29,31|	Z8786935772	**1**{*336*}	Nc1nc2ccc(cc2nc1Cl)[N+](=O)[O-]	**2**{*44*}	CCn1cc(C=O)cn1	**3**{*17*}	[C-]#[N+]CC1CCOCC1	0.0	0
567	**4**{*337*,*118*,*14*}	COCCNC1=C(N=C2N1C=CC(Br)=C2Cl)C1=CC(C)=NC=C1 |c:7,11,14,21,23,t:5,18|	Z8786901713	**1**{*337*}	Nc1nccc(Br)c1Cl	**2**{*118*}	Cc1cc(C=O)ccn1	**3**{*14*}	COCC[N+]#[C-]	0.0	0
568	**4**{*338*,*170*,*6*}	CCOC(=O)CCCNC1=C(N=C2N1C=CC1=C2C=CN=C1)C1=NN(CC)N=N1 |c:11,15,17,20,22,30,t:9,25|	Z8778277874	**1**{*338*}	Nc1nccc2cnccc12	**2**{*170*}	CCn1nnc(C=O)n1	**3**{*6*}	CCOC(=O)CCC[N+]#[C-]	0.0	0
569	**4**{*339*,*80*,*14*}	COCCNC1=C(N=C2N1C=CC1=C2C=CS1)C1=NC=C(C)S1 |c:7,11,13,16,t:5,20,22|	Z8778277362	**1**{*339*}	Nc1nccc2sccc12	**2**{*80*}	Cc1cnc(C=O)s1	**3**{*14*}	COCC[N+]#[C-]	0.0	0
570	**4**{*340*,*58*,*14*}	CCOC1=CC=CN2C(NCCOC)=C(N=C12)C1=NSN=C1 |c:5,13,22,t:3,15,19|	Z8778277284	**1**{*340*}	CCOc1cccnc1N	**2**{*58*}	O=Cc1cnsn1	**3**{*14*}	COCC[N+]#[C-]	0.0	0
571	**4**{*341*,*80*,*39*}	CCOCCNC1=C(N=C2C=CC(CN(C)C)=CN12)C1=NC=C(C)S1 |c:10,16,t:6,8,21,23|	Z8781341769	**1**{*341*}	CN(C)Cc1ccc(N)nc1	**2**{*80*}	Cc1cnc(C=O)s1	**3**{*39*}	CCOCC[N+]#[C-]	0.0	0
572	**4**{*342*,*118*,*17*}	CC1=NC=CC(=C1)C1=C(NCC2CCOCC2)N2C=CC3=C(C=CC(=C3)[N+]([O-])=O)C2=N1 |c:3,5,8,20,24,26,33,t:1,22|	Z8784060910	**1**{*342*}	Nc1nccc2cc(ccc12)[N+](=O)[O-]	**2**{*118*}	Cc1cc(C=O)ccn1	**3**{*17*}	[C-]#[N+]CC1CCOCC1	0.0	0
573	**4**{*343*,*58*,*27*}	CCCCNC1=C(N=C2N1C=CC1=C2C=CO1)C1=NSN=C1 |c:7,11,13,16,23,t:5,20|	Z8784060493	**1**{*343*}	Nc1nccc2occc12	**2**{*58*}	O=Cc1cnsn1	**3**{*27*}	CCCC[N+]#[C-]	0.0	0
574	**4**{*344*,*58*,*14*}	COCCNC1=C(N=C2N1C=CC=C2SC)C1=NSN=C1 |c:7,11,13,21,t:5,18|	Z8784059177	**1**{*344*}	CSc1cccnc1N	**2**{*58*}	O=Cc1cnsn1	**3**{*14*}	COCC[N+]#[C-]	0.0	0
575	**4**{*26*,*22*,*14*}	COCCNC1=C(N=C2C=CC=C(N3CCCC3)N12)C1=CC=CC=C1 |c:9,24,26,t:5,7,11,22|	Z8780122312	**1**{*26*}	Nc1cccc(n1)N2CCCC2	**2**{*22*}	O=Cc1ccccc1	**3**{*14*}	COCC[N+]#[C-]	0.0	0
576	**4**{*345*,*41*,*17*}	BrC1=CN2C(NCC3CCOCC3)=C(N=C2C(Br)=N1)C1=NC=CC=C1 |c:13,15,19,24,26,t:1,22|	Z8780121691	**1**{*345*}	Nc1ncc(Br)nc1Br	**2**{*41*}	O=Cc1ccccn1	**3**{*17*}	[C-]#[N+]CC1CCOCC1	0.0	0
577	**4**{*188*,*14*,*14*}	COCCNC1=C(N=C2C=NC=C(N12)C(=O)OC)C1=CC=C(C)C=C1 |c:9,11,25,t:5,7,20,22|	Z8776690330	**1**{*188*}	COC(=O)c1cncc(N)n1	**2**{*14*}	Cc1ccc(C=O)cc1	**3**{*14*}	COCC[N+]#[C-]	0.0	0

<table>
<tr><td>578</td><td>4{1 ,22 ,27 }</td><td>CCCCNC1=C(N=C2C=CNN12)C1=CC=CC=C1 |c:9,17,19,t:5,7,15|</td><td>Z8711892168</td><td>1{1 }</td><td>Nc1cc[nH]n1</td><td>2{22 }</td><td>O=Cc1ccccc1</td><td>3{27 }</td><td>CCCC[N+]#[C-]</td><td>0.0</td><td>0</td></tr>
<tr><td>579</td><td>4{2 ,15 ,54 }</td><td>COC1=CC=C(C=C1)C1=C(NC2=CC=CC=C2)N2OC=CC2=N1 |c:4,6,9,14,16,21,24,t:2,12|</td><td>Z8711891895</td><td>1{2 }</td><td>Nc1ccon1</td><td>2{15 }</td><td>COc1ccc(C=O)cc1</td><td>3{54 }</td><td>[C-]#[N+]c1ccccc1</td><td>0.0</td><td>0</td></tr>
<tr><td>580</td><td>4{346 ,15 ,9 }</td><td>CNC1=C(N=C2N1C=CN=C2C)C1=CC=C(OC)C=C1 |c:4,8,10,20,t:2,14,16|</td><td>Z8741907202</td><td>1{346 }</td><td>Cc1nccnc1N</td><td>2{15 }</td><td>COc1ccc(C=O)cc1</td><td>3{9 }</td><td>C[N+]#[C-]</td><td>0.0</td><td>0</td></tr>
<tr><td colspan="12">Preliminary test experiments, Sc(OTf) $_3$ as the catalyst</td></tr>
<tr><td>581</td><td>4{347 ,242 ,24 }</td><td>COC1=CC(CNC2=C(N=C3C=CC(Cl)=CN23)C2=CN3N=CC(C#N)=C3N=C2)=CC=C1 |c:11,14,22,26,29,31,33,t:2,7,9,19|</td><td>Z8878918808</td><td>1{347 }</td><td>Nc1ccc(Cl)cn1</td><td>2{242 }</td><td>O=Cc1cnc2c(C#N)cnn2c1</td><td>3{24 }</td><td>COc1cccc(C[N+]#[C-])c1</td><td>114.9</td><td>99</td></tr>
<tr><td>582</td><td>4{54 ,285 ,53 }</td><td>CN(C)C(=O)C1=CN2C(NCCOCC3=CC=CC=C3)=C(N=C2C=C1)C1=CSC(=C1)[N+]([O-])=O |c:16,18,20,22,25,31,t:5,14,28|</td><td>Z8878918446</td><td>1{54 }</td><td>Cl.CN(C)C(=O)c1ccc(N)nc1</td><td>2{285 }</td><td>[O-][N+](=O)c1cc(C=O)cs1</td><td>3{53 }</td><td>[C-]#[N+]CCOCc1ccccc1</td><td>99.6</td><td>79</td></tr>
<tr><td>583</td><td>4{105 ,286 ,34 }</td><td>NC(=O)C1=CC=CC2=NC(=C(NCC3CCCO3)N12)C1=NC=CC(=C1)C(F)(F)F |c:5,24,26,t:3,7,9,22|</td><td>Z8878918833</td><td>1{105 }</td><td>NC(=O)c1cccc(N)n1</td><td>2{286 }</td><td>FC(F)(F)c1ccnc(C=O)c1</td><td>3{34 }</td><td>[C-]#[N+]CC1CCCO1</td><td>81.8</td><td>75</td></tr>
<tr><td>584</td><td>4{69 ,287 ,12 }</td><td>CNC(=O)C1=CC2=NC(=C(NCC3CCOC3)N2C=C1)C1=CC(OCCCOC)=CC=C1 |c:20,31,33,t:4,6,8,23|</td><td>Z8878918860</td><td>1{69 }</td><td>CNC(=O)c1ccnc(N)c1</td><td>2{287 }</td><td>COCCCOc1cccc(C=O)c1</td><td>3{12 }</td><td>[C-]#[N+]CC1CCOC1</td><td>82.5</td><td>70</td></tr>
<tr><td>585</td><td>4{348 ,288 ,39 }</td><td>CCOCCNC1=C(N=C2C=NC(F)=CN12)C1=CC=C(CCF)C=C1 |c:10,13,25,t:6,8,18,20|</td><td>Z8878918639</td><td>1{348 }</td><td>Nc1cnc(F)cn1</td><td>2{288 }</td><td>FCCc1ccc(C=O)cc1</td><td>3{39 }</td><td>CCOCC[N+]#[C-]</td><td>60.2</td><td>68</td></tr>
<tr><td>586</td><td>4{349 ,110 ,69 }</td><td>COC1=C(NC2=C(N=C3C=CC(Br)=C(C)N23)C2=CN=C(C=C2)P(C)(C)=O)C=CC=C1 |c:2,9,20,22,29,31,t:5,7,12,18|</td><td>Z8846492345</td><td>1{349 }</td><td>Cc1nc(N)ccc1Br</td><td>2{110 }</td><td>CP(=O)(C)c1ccc(C=O)cn1</td><td>3{69 }</td><td>COc1ccccc1[N+]#[C-]</td><td>88.3</td><td>67</td></tr>
<tr><td>587</td><td>4{153 ,289 ,22 }</td><td>COC1=C(OCC#C)C=C(C=C1)C1=C(NC2COC2)N2C(C=CC=C2C(C)(C)O)=N1 |c:2,8,10,13,23,25,31|</td><td>Z8873684876</td><td>1{153 }</td><td>CC(C)(O)c1cccc(N)n1</td><td>2{289 }</td><td>COc1ccc(C=O)cc1OCC#C</td><td>3{22 }</td><td>[C-]#[N+]C1COC1</td><td>71.8</td><td>65</td></tr>
<tr><td>588</td><td>4{28 ,174 ,7 }</td><td>C(C1CCCO1)N1C=C(N=N1)C1=C(NC2CCOCC2)N2C=CC(=CC2=N1)C1=NOC=N1 |c:8,10,13,24,26,29,35,t:32|</td><td>Z8878918438</td><td>1{28 }</td><td>Cl.Nc1cc(ccn1)c2ncon2</td><td>2{174 }</td><td>O=Cc1cn(CC2CCCO2)nn1</td><td>3{7 }</td><td>[C-]#[N+]C1CCOCC1</td><td>76.3</td><td>65</td></tr>
<tr><td>589</td><td>4{193 ,290 ,10 }</td><td>CNC(=O)C1=CN2C(C=C1)=NC(=C2NC1CCOC1)C1=CC(OC2=NC=CC=C2)=CC=C1 |c:8,10,12,28,30,32,34,t:4,22,26|</td><td>Z8878918325</td><td>1{193 }</td><td>CNC(=O)c1ccc(N)nc1</td><td>2{290 }</td><td>O=Cc1cccc(Oc2ccccn2)c1</td><td>3{10 }</td><td>[C-]#[N+]C1CCOC1</td><td>74.8</td><td>65</td></tr>
</table>

590	**4**{*41,30,13*}	COC1=CC=C(CNC2=C(N=C3C=NC(C)=CN23)C2CCN(CC2)C(C)=O)C=C1 |c:12,15,30,t:2,4,8,10|	Z8873684727	**1**{*41*}	Cc1cnc(N)cn1	**2**{*30*}	CC(=O)N1CCC(CC1)C=O	**3**{*13*}	COc1ccc(C[N+]#[C-])cc1	67.7	64
591	**4**{*107,291,24*}	COC1=CC(CNC2=C(N=C3C=CC(OCCO)=CN23)C2=C(C)C(OC)=CN=C2)=CC=C1 |c:11,17,22,27,29,31,33,t:2,7,9|	Z8878918508	**1**{*107*}	Nc1ccc(OCCO)cn1	**2**{*291*}	COc1cncc(C=O)c1C	**3**{*24*}	COc1cccc(C[N+]#[C-])c1	74.7	64
592	**4**{*107,292,21*}	CC1=C(SC=N1)C1=C(NCC2CC2)N2C=C(OCCO)C=CC2=N1 |c:4,7,22,25,t:1,16|	Z8873684602	**1**{*107*}	Nc1ccc(OCCO)cn1	**2**{*292*}	Cc1ncsc1C=O	**3**{*21*}	[C-]#[N+]CC1CC1	57.6	62
593	**4**{*108,139,21*}	CCCOC1=CN2C(C=C1)=NC(=C2NCC1CC1)C1=C(C)N(CCO)N=C1C |c:8,10,12,21,28,t:4|	Z8873684946	**1**{*108*}	CCCOc1ccc(N)nc1	**2**{*139*}	Cc1nn(CCO)c(C)c1C=O	**3**{*21*}	[C-]#[N+]CC1CC1	63.0	61
594	**4**{*347,171,49*}	COC1CC(C(OC)O1)C1=C(NC2CC(F)(F)C2)N2C=C(Cl)C=CC2=N1 |c:10,24,27,t:21|	Z8873685323	**1**{*347*}	Nc1ccc(Cl)cn1	**2**{*171*}	COC1CC(C=O)C(OC)O1	**3**{*49*}	FC1(F)CC(C1)[N+]#[C-]	60.9	58
595	**4**{*350,293,5*}	COCCCNC1=C(N=C2C=NC(=CN12)C1=CC=CC=C1)C1=C(F)N(C)N=C1 |c:10,12,19,21,24,29,t:6,8,17|	Z8873684623	**1**{*350*}	Nc1cnc(cn1)c2ccccc2	**2**{*293*}	Cn1ncc(C=O)c1F	**3**{*5*}	COCCC[N+]#[C-]	59.7	58
596	**4**{*267,294,23*}	COC(=O)CCC(NC1=C(N=C2C=CC=C(C#C)N12)C1=C(OC)C(=O)C=CO1)C(=O)OC |c:12,21,27,t:8,10,14|	Z8878918868	**1**{*267*}	Nc1cccc(C#C)n1	**2**{*294*}	COc1c(C=O)occc1=O	**3**{*23*}	COC(=O)CCC([N+]#[C-])C(=O)OC	68.7	58
597	**4**{*81,112,9*}	CNC1=C(N=C2C=NC=CN12)C1=C2C=CC=NN2N=C1 |c:6,8,13,15,17,21,t:2,4|	Z8873685319	**1**{*81*}	Nc1cnccn1	**2**{*112*}	O=Cc1cnn2ncccc12	**3**{*9*}	C[N+]#[C-]	41.3	58
598	**4**{*95,295,31*}	CSCCNC1=C(N=C2C=CC(=CN12)C(F)F)C1=CSC(C)=N1 |c:9,11,23,t:5,7,19|	Z8873684632	**1**{*95*}	Nc1ccc(cn1)C(F)F	**2**{*295*}	Cc1nc(C=O)cs1	**3**{*31*}	CSCC[N+]#[C-]	55.0	57
599	**4**{*351,296,44*}	CC(C)(C)OC(=O)NCCCCNC1=C(N=C2C=CC3=C(C(O)CC3)N12)C1=CN(CCF)N=C1 |c:17,35,t:13,15,19,29|	Z8878918517	**1**{*351*}	Nc1ccc2CCC(O)c2n1	**2**{*296*}	FCCn1cc(C=O)cn1	**3**{*44*}	CC(C)(C)OC(=O)NCCCC[N+]#[C-]	72.7	57
600	**4**{*118,124,31*}	COC1=C(OCC2=C(C)ON=C2C)C=CC(=C1)C1=C(NCCSC)N2C(C=CC=C2C(C)O)=N1 |c:2,6,10,14,16,19,28,30,35|	Z8854581141	**1**{*118*}	Cl.CC(O)c1cccc(N)n1	**2**{*124*}	COc1cc(C=O)ccc1OCc2c(C)noc2C	**3**{*31*}	CSCC[N+]#[C-]	73.8	57
601	**4**{*28,297,26*}	CCN1N=C(C)C(=N1)C1=C(NCC2=CC=C(F)C=C2)N2C=CC(=CC2=N1)C1=NOC=N1 |c:6,9,18,22,24,27,33,t:3,13,15,30|	Z8846491885	**1**{*28*}	Cl.Nc1cc(ccn1)c2ncon2	**2**{*297*}	CCn1nc(C)c(C=O)n1	**3**{*26*}	Fc1ccc(C[N+]#[C-])cc1	64.0	57
602	**4**{*142,298,12*}	CS(=O)(=O)CCOC1=CC=CN2C(NCC3CCOC3)=C(CCCC3=CC=CC=C3)N=C12 |c:9,27,29,t:7,20,25,32|	Z8873684783	**1**{*142*}	CS(=O)(=O)CCOc1cccnc1N	**2**{*298*}	O=CCCCc1ccccc1	**3**{*12*}	[C-]#[N+]CC1COC1	69.9	57

603	**4**{*153*,*299*,*15*}	CCNC1=C(N=C2C=CC=C(N12)C(C)(C)O)C1=C(OC)C(OC)=CC=C1F |c:7,9,18,24,26,t:3,5|	Z8873684819	**1**{*153*}	CC(C)(O)c1cccc(N)n1	**2**{*299*}	COc1ccc(F)c(C=O)c1OC	**3**{*15*}	CC[N+]#[C-]	56.6	56
604	**4**{*105*,*300*,*29*}	CCOC(=O)CCNC1=C(CC2CN(C2)C(=O)OC(C)(C)C)N=C2C=CC=C(N12)C(N)=O |c:8,25,27,t:23|	Z8855739218	**1**{*105*}	NC(=O)c1cccc(N)n1	**2**{*300*}	CC(C)(C)OC(=O)N1CC(CC=O)C1	**3**{*29*}	CCOC(=O)CC[N+]#[C-]	67.3	56
605	**4**{*352*,*301*,*49*}	CNC(=O)C1=C(F)C=CC(=C1)C1=C(NC2CC(F)(F)C2)N2C=C(C=CC2=N1)N1CCN(C)C(=O)C1 |c:4,7,9,12,23,25,28|	Z8873684938	**1**{*352*}	CN1CCN(CC1=O)c2ccc(N)nc2	**2**{*301*}	CNC(=O)c1cc(C=O)ccc1F	**3**{*49*}	FC1(F)CC(C1)[N+]#[C-]	73.5	56
606	**4**{*91*,*302*,*7*}	NC(=O)C1=CN2C(C=C1)=NC([C@@H]1C[C@H]1C1=CC=C(F)C=C1)=C2NC1CCOCC1 |&1:11,13,r,c:7,9,21,23,t:3,16,18|	Z8829498723	**1**{*91*}	NC(=O)c1ccc(N)nc1	**2**{*302*}	Fc1ccc(cc1)[C@@H]2C[C@H]2C=O |&1:7,9,r|	**3**{*7*}	[C-]#[N+]C1CCOCC1	59.4	56
607	**4**{*67*,*284*,*13*}	COC1=CC=C(CNC2=C(N=C3C=CC=C(C)N23)C2=C(C)N=CC=N2)C=C1 |c:12,20,23,25,28,t:2,4,8,10,14|	Z8878918440	**1**{*67*}	Cc1cccc(N)n1	**2**{*284*}	Cc1nccnc1C=O	**3**{*13*}	COc1ccc(C[N+]#[C-])cc1	53.6	55
608	**4**{*267*,*303*,*23*}	COC(=O)CCC(NC1=C(N=C2C=CC=C(C#C)N12)C1=C(C)C(=C(OC)C=C1)[N+]([O-])=O)C(=O)OC |c:12,21,28,t:8,10,14,24|	Z8854581183	**1**{*267*}	Nc1cccc(C#C)n1	**2**{*303*}	COc1ccc(C=O)c(C)c1[N+](=O)[O-]	**3**{*23*}	COC(=O)CCC([N+]#[C-])C(=O)OC	71.7	55
609	**4**{*124*,*304*,*43*}	CC1=C(C=NN1CC(F)F)C1=C(NC2CCN(CC2)C(=O)OC(C)(C)C)N2C=CC=C(CO)C2=N1 |c:3,11,29,36,t:1,31|	Z8878918512	**1**{*124*}	Nc1ncccc1CO	**2**{*304*}	Cc1c(C=O)cnn1CC(F)F	**3**{*43*}	CC(C)(C)OC(=O)N1CCC(CC1)[N+]#[C-]	73.1	55
610	**4**{*52*,*189*,*7*}	FC(F)(F)C1(CC1)C1=CN2C(C=C1)=NC(C1=CN(N=N1)C1CCOCC1)=C2NC1CCOCC1 |c:12,14,20,29,t:8,17|	Z8878918380	**1**{*52*}	Nc1ccc(cn1)C2(CC2)C(F)(F)F	**2**{*189*}	O=Cc1cn(nn1)C2CCOCC2	**3**{*7*}	[C-]#[N+]C1CCOCC1	71.0	55
611	**4**{*353*,*223*,*15*}	CCNC1=C(N=C2C=CC(NS(C)(=O)=O)=CN12)C1=CN=C(C=C1)C(=O)OC |c:7,14,21,23,t:3,5,19|	Z8878918898	**1**{*353*}	Cl.CS(=O)(=O)Nc1ccc(N)nc1	**2**{*223*}	Cl.COC(=O)c1ccc(C=O)cn1	**3**{*15*}	CC[N+]#[C-]	57.9	55
612	**4**{*354*,*305*,*70*}	COCC1=NC(=CN1)C1=C(NC2CCN(C2)C(=O)OC(C)(C)C)N2C=C(C=CC2=N1)C(=O)OC |c:5,9,26,28,31,t:3|	Z8855619579	**1**{*354*}	COC(=O)c1ccc(N)nc1	**2**{*305*}	COCc1nc(C=O)c[nH]1	**3**{*70*}	CC(C)(C)OC(=O)N1CCC(C1)[N+]#[C-]	69.9	55
613	**4**{*144*,*306*,*47*}	CNS(=O)(=O)C1=CN2C(C=C1)=NC(=C2NC(C)C)C1=CC=C(C=C1)N(C)CCO |c:9,11,13,22,24,t:5,20|	Z8855739270	**1**{*144*}	CNS(=O)(=O)c1ccc(N)nc1	**2**{*306*}	CN(CCO)c1ccc(C=O)cc1	**3**{*47*}	CC(C)[N+]#[C-]	61.8	55

614	**4**{*41*,*307*,*9*}	CNC1=C(N=C2C=NC(C)=CN12)C1=CN=C(Cl)N1C |c:6,9,t:2,4,14,16|	Z8849597811	**1**{*41*}	Cc1cnc(N)cn1	**2**{*307*}	Cn1c(Cl)ncc1C=O	**3**{*9*}	C[N+]#[C-]	40.9	55
615	**4**{*50*,*308*,*31*}	CSCCNC1=C(CCC2CCCC2)N=C2C=CC=C(N12)P(C)(C)=O |c:5,17,19,t:15|	Z8873685271	**1**{*50*}	CP(=O)(C)c1cccc(N)n1	**2**{*308*}	O=CCCC1CCCC1	**3**{*31*}	CSCC[N+]#[C-]	56.1	55
616	**4**{*54*,*309*,*47*}	CC(C)NC1=C(N=C2C=CC(=CN12)C(=O)N(C)C)C1=CC=C(C=C1)N1C=CC=N1 |c:8,10,22,24,28,30,t:4,6,20|	Z8829498465	**1**{*54*}	Cl.CN(C)C(=O)c1ccc(N)nc1	**2**{*309*}	O=Cc1ccc(cc1)n2cccn2	**3**{*47*}	CC(C)[N+]#[C-]	57.4	55
617	**4**{*52*,*310*,*7*}	FC(F)(F)C1(CC1)C1=CN2C(C=C1)=NC(C1COC3(CCC3)C1)=C2NC1CCOCC1 |c:12,14,27,t:8|	Z8878918339	**1**{*52*}	Nc1ccc(cn1)C2(CC2)C(F)(F)F	**2**{*310*}	O=CC1CCOC2(CCC2)C1	**3**{*7*}	[C-]#[N+]C1CCOCC1	66.2	55
618	**4**{*49*,*84*,*13*}	COC1=CC=C(CNC2=C(CCSC)N=C3C=CC=C(CO)N23)C=C1 |c:8,16,25,t:2,4,14,18|	Z8855739168	**1**{*49*}	Nc1cccc(CO)n1	**2**{*84*}	CSCCC=O	**3**{*13*}	COc1ccc(C[N+]#[C-])cc1	52.5	54
619	**4**{*355*,*93*,*22*}	CC(C)C1=CC=CC2=NC(=C(NC3COC3)N12)C1=NC(CO)=CC=C1 |c:5,24,26,t:3,7,9,20|	Z8873684910	**1**{*355*}	CC(C)c1cccc(N)n1	**2**{*93*}	OCc1cccc(C=O)n1	**3**{*22*}	[C-]#[N+]C1COC1	49.6	54
620	**4**{*351*,*311*,*39*}	CCOCCNC1=C(N=C2C=CC3=C(C(O)CC3)N12)C1=NN(CC(F)(F)F)C=C1Br |c:10,30,t:6,8,12,22|	Z8855739266	**1**{*351*}	Nc1ccc2CCC(O)c2n1	**2**{*311*}	FC(F)(F)Cn1cc(Br)c(C=O)n1	**3**{*39*}	CCOCC[N+]#[C-]	71.5	54
621	**4**{*356*,*312*,*8*}	COC(=O)C(CC1=CC=CC=C1)NC1=C(N=C2C=CC(Cl)=C(N12)C(=O)N(C)C)C1CCOC1(C)C |c:8,10,18,21,t:6,14,16|	Z8846491967	**1**{*356*}	CN(C)C(=O)c1nc(N)ccc1Cl	**2**{*312*}	CC1(C)OCCC1C=O	**3**{*8*}	COC(=O)C(Cc1ccccc1)[N+]#[C-]	72.5	54
622	**4**{*357*,*175*,*49*}	OCC1CCN(CC1)C1=CN2C(NC3CC(F)(F)C3)=C(N=C2C=C1)C1=NNC=C1 |c:20,22,25,31,t:9,28|	Z8873685388	**1**{*357*}	Nc1ccc(cn1)N2CCC(CO)CC2	**2**{*175*}	O=Cc1cc[nH]n1	**3**{*49*}	FC1(F)CC(C1)[N+]#[C-]	58.4	54
623	**4**{*53*,*236*,*42*}	CC1=C(NC2=C(CCC3OCCO3)N=C3C=CC(CO)=CN23)C=CC=C1 |c:1,4,16,20,25,27,t:14|	Z8837933178	**1**{*53*}	Nc1ccc(CO)cn1	**2**{*236*}	O=CCCC1OCCO1	**3**{*42*}	Cc1ccccc1[N+]#[C-]	51.0	53
624	**4**{*48*,*313*,*22*}	COC1=CC2=C(C=C1)N(C)C=C2C1=C(NC2COC2)N2C=CC(=CC2=N1)S(C)(=O)=O |c:4,6,11,14,23,25,28,t:2|	Z8878918334	**1**{*48*}	CS(=O)(=O)c1ccnc(N)c1	**2**{*313*}	COc1ccc2n(C)cc(C=O)c2c1	**3**{*22*}	[C-]#[N+]C1COC1	62.4	53
625	**4**{*69*,*314*,*46*}	CNC(=O)C1=CC2=NC(C3=CNN=C3)=C(NCC(C)=C)N2C=C1 |c:12,23,t:4,6,9,14|	Z8878918522	**1**{*69*}	CNC(=O)c1ccnc(N)c1	**2**{*314*}	O=Cc1cn[nH]c1	**3**{*46*}	CC(=C)C[N+]#[C-]	44.3	53
626	**4**{*118*,*82*,*18*}	COC1=C(C=CC=C1F)C1=C(NC(C)(C)C)N2C(C=CC=C2C(C)O)=N1 |c:4,6,10,19,21,26,t:2|	Z8873685369	**1**{*118*}	Cl.CC(O)c1cccc(N)n1	**2**{*82*}	COc1c(F)cccc1C=O	**3**{*18*}	CC(C)(C)[N+]#[C-]	51.0	53

627	**4**{*49*,*52*,*13*}	COC1=CC=C(CNC2=C(N=C3C=CC=C(CO)N23)C2=CN(N=C2)C2CCC2)C=C1 |c:12,24,32,t:2,4,8,10,14,21|	Z8873684618	**1**{*49*}	Nc1cccc(CO)n1	**2**{*52*}	O=Cc1cnn(c1)C2CCC2	**3**{*13*}	COc1ccc(C[N+]#[C-])cc1	57.4	53
628	**4**{*193*,*4*,*12*}	CNC(=O)C1=CN2C(C=C1)=NC(=C2NCC1CCOC1)C1=C(Cl)C=CC=C1Cl |c:8,10,12,23,26,28,t:4|	Z8855739153	**1**{*193*}	CNC(=O)c1ccc(N)nc1	**2**{*4*}	Clc1cccc(Cl)c1C=O	**3**{*12*}	[C-]#[N+]CC1CCOC1	59.6	53
629	**4**{*193*,*315*,*12*}	CCCN1C=CC(=N1)C1=C(NCC2CCOC2)N2C=C(C=CC2=N1)C(=O)NC |c:4,6,9,20,22,25|	Z8854581159	**1**{*193*}	CNC(=O)c1ccc(N)nc1	**2**{*315*}	CCCn1ccc(C=O)n1	**3**{*12*}	[C-]#[N+]CC1CCOC1	54.3	53
630	**4**{*358*,*316*,*22*}	COC(=O)C1=CC2=C(C=C1)N=C(C=C2)C1=C(NC2COC2)N2C=CC(=CC2=N1)C(C)(C)O |c:6,8,11,13,16,25,27,30,t:4|	Z8846491841	**1**{*358*}	CC(C)(O)c1ccnc(N)c1	**2**{*316*}	COC(=O)c1ccc2nc(C=O)ccc2c1	**3**{*22*}	[C-]#[N+]C1COC1	61.4	53
631	**4**{*92*,*317*,*14*}	CCN1N=CC(=N1)C1=C(NCCOC)N2C=CC(=CC2=N1)C(N)=O |c:3,5,8,16,18,21|	Z8873685381	**1**{*92*}	NC(=O)c1ccnc(N)c1	**2**{*317*}	CCn1ncc(C=O)n1	**3**{*14*}	COCC[N+]#[C-]	46.7	53
632	**4**{*359*,*318*,*22*}	FC(F)OC1=CN2C(C=C1)=NC(C1CC11CC1(F)F)=C2NC1COC1 |c:8,10,21,t:4|	Z8878918410	**1**{*359*}	Nc1ccc(OC(F)F)cn1	**2**{*318*}	FC1(F)CC21CC2C=O	**3**{*22*}	[C-]#[N+]C1COC1	49.9	53
633	**4**{*71*,*319*,*10*}	CC1=C(C=CC(OCC#N)=C1)C1=C(NC2CCOC2)N2C=C(I)N=CC2=N1 |c:3,9,12,25,28,t:1,22|	Z8855739134	**1**{*71*}	Nc1cnc(I)cn1	**2**{*319*}	Cc1cc(OCC#N)ccc1C=O	**3**{*10*}	[C-]#[N+]C1CCOC1	67.2	52
634	**4**{*113*,*62*,*14*}	COCCNC1=C(N=C2N1C=CC=C2N1CCOCC1)C1CC11CCOCC1 |c:7,11,13,t:5|	Z8878918357	**1**{*113*}	Nc1ncccc1N2CCOCC2	**2**{*62*}	O=CC1CC21CCOCC2	**3**{*14*}	COCC[N+]#[C-]	54.6	52
635	**4**{*360*,*320*,*29*}	CCOC(=O)CCNC1=C(N=C2C=C(C(=O)OC)C(Br)=CN12)C1=NN(C)C=N1 |c:19,28,t:8,10,12,24|	Z8878918308	**1**{*360*}	COC(=O)c1cc(N)ncc1Br	**2**{*320*}	Cn1cnc(C=O)n1	**3**{*29*}	CCOC(=O)CC[N+]#[C-]	63.7	52
636	**4**{*352*,*103*,*47*}	CC(C)NC1=C(N=C2C=CC(=CN12)N1CCN(C)C(=O)C1)C1CCC2(COC2)OC1 |c:8,10,t:4,6|	Z8849597817	**1**{*352*}	CN1CCN(CC1=O)c2ccc(N)nc2	**2**{*103*}	O=CC1CCC2(COC2)OC1	**3**{*47*}	CC(C)[N+]#[C-]	58.4	52
637	**4**{*261*,*321*,*12*}	CN(C)C1CCN(CC1)C1=CN2C(C=C1)=NC(C1CC11CCC(F)(F)CC1)=C2NCC1CCOC1 |c:14,16,30,t:10|	Z8873685382	**1**{*261*}	CN(C)C1CCN(CC1)c2ccc(N)nc2	**2**{*321*}	FC1(F)CCC2(CC2C=O)CC1	**3**{*12*}	[C-]#[N+]CC1CCOC1	68.6	52
638	**4**{*37*,*139*,*24*}	COC1=CC(CNC2=C(N=C3C=CC=C(C(F)F)N23)C2=C(C)N(CCO)N=C2C)=CC=C1 |c:11,21,28,31,33,t:2,7,9,13|	Z8878918891	**1**{*37*}	Cl.Nc1cccc(n1)C(F)F	**2**{*139*}	Cc1nn(CCO)c(C)c1C=O	**3**{*24*}	COc1cccc(C[N+]#[C-])c1	62.1	52
639	**4**{*180*,*155*,*53*}	COC(=O)C1=CC2=NC(=C(NCCOCC3=CC=CC=C3)N2C=C1C)C1=CN=CN=C1 |c:17,19,24,30,32,t:4,6,8,15,28|	Z8803896942	**1**{*180*}	COC(=O)c1cc(N)ncc1C	**2**{*155*}	O=Cc1cncnc1	**3**{*53*}	[C-]#[N+]CCOCc1ccccc1	58.7	52

640	**4**{*87*,*322*,*23*}	COC(=O)CCC(NC1=C(N=C2C=C(C)C(Cl)=CN12)C1=CC(NC(C)=O)=CC=C1)C(=O)OC |c:16,27,29,t:8,10,12,21|	Z8873684717	**1**{*87*}	Cc1cc(N)ncc1Cl	**2**{*322*}	CC(=O)Nc1cccc(C=O)c1	**3**{*23*}	COC(=O)CCC([N+]#[C-])C(=O)OC	66.5	52
641	**4**{*311*,*220*,*61*}	CC1=C(C(C)=NN1)C1=C(NCCCNC(=O)OC(C)(C)C)N2C=CC(F)=CC2=N1 |c:4,8,23,26,29,t:1|	Z8878918419	**1**{*311*}	Nc1cc(F)ccn1	**2**{*220*}	Cc1n[nH]c(C)c1C=O	**3**{*61*}	CC(C)(C)OC(=O)NCCC[N+]#[C-]	56.6	52
642	**4**{*361*,*323*,*12*}	CC(C)OC1=CC=CN2C(NCC3CCOC3)=C(N=C12)[C@@H]1C[C@@H](C1)NC(=O)OC(C)(C)C |r,c:6,17,t:4,19|	Z8846492360	**1**{*361*}	Cl.CC(C)Oc1cccnc1N	**2**{*323*}	CC(C)(C)OC(=O)N[C@H]1C[C@H](C1)C=O	**3**{*12*}	[C-]#[N+]CC1CCOC1	62.2	52
643	**4**{*120*,*324*,*70*}	CN1C=C(C=N1)C1=CC2=NC(C3CC4(C3)CCCO4)=C(NC3CCN(C3)C(=O)OC(C)(C)C)N2C=C1 |c:2,4,39,t:7,9,21|	Z8829498458	**1**{*120*}	Cn1cc(cn1)c2ccnc(N)c2	**2**{*324*}	O=CC1CC2(C1)CCCO2	**3**{*70*}	CC(C)(C)OC(=O)N1CCC(C1)[N+]#[C-]	68.6	52
644	**4**{*193*,*325*,*21*}	CNC(=O)C1=CN2C(C=C1)=NC(CCN1C(=O)C3=C(C=CC=C3)C1=O)=C2NCC1CC1 |c:8,10,20,22,27,t:4,18|	Z8878918490	**1**{*193*}	CNC(=O)c1ccc(N)nc1	**2**{*325*}	O=CCCN1C(=O)c2ccccc2C1=O	**3**{*21*}	[C-]#[N+]CC1CC1	58.1	52
645	**4**{*362*,*326*,*10*}	CNC(=O)C1=CC=CC2=NC(=C(NC3CCOC3)N12)C1=CC=NN1C |c:6,24,t:4,8,10,22|	Z8873684581	**1**{*362*}	CNC(=O)c1cccc(N)n1	**2**{*326*}	Cn1nccc1C=O	**3**{*10*}	[C-]#[N+]C1CCOC1	47.3	51
646	**4**{*109*,*327*,*29*}	CCOC(=O)CCNC1=C(N=C2C=CC=C(N12)S(N)(=O)=O)C1=NC(Cl)=CC(Cl)=C1 |c:12,14,26,29,t:8,10,23|	Z8855739173	**1**{*109*}	Cl.Nc1cccc(n1)S(=O)(=O)N	**2**{*327*}	Clc1cc(Cl)nc(C=O)c1	**3**{*29*}	CCOC(=O)CC[N+]#[C-]	63.4	51
647	**4**{*107*,*328*,*43*}	CCC1=NNC=C1C1=C(NC2CCN(CC2)C(=O)OC(C)(C)C)N2C=C(OCCO)C=CC2=N1 |c:5,8,32,35,t:2,26|	Z8873685371	**1**{*107*}	Nc1ccc(OCCO)cn1	**2**{*328*}	Cl.CCc1n[nH]cc1C=O	**3**{*43*}	CC(C)(C)OC(=O)N1CCC(CC1)[N+]#[C-]	65.0	51
648	**4**{*363*,*329*,*62*}	COC(=O)C1=CC2=NC(=C(NC3(CCCC3)C(=O)OC)N2C=C1)C1=C(C)N(CCCl)N=C1C |c:23,26,33,t:4,6,8|	Z8878918904	**1**{*363*}	COC(=O)c1ccnc(N)c1	**2**{*329*}	Cc1nn(CCCl)c(C)c1C=O	**3**{*62*}	COC(=O)C1(CCCC1)[N+]#[C-]	65.4	51
649	**4**{*364*,*330*,*62*}	COC(=O)C1(CCCC1)NC1=C(N=C2C=CC(=CN12)N1CCNC(=O)C1)C1=C(OC)C(Cl)=NC=C1 |c:15,17,30,35,37,t:11,13|	Z8849597816	**1**{*364*}	Nc1ccc(cn1)N2CCNC(=O)C2	**2**{*330*}	COc1c(Cl)ncc1C=O	**3**{*62*}	COC(=O)C1(CCCC1)[N+]#[C-]	68.8	51
650	**4**{*48*,*331*,*35*}	CSCCCNC1=C(N=C2C=C(C=CN12)S(C)(=O)=O)C1=CC2=C(OCCNC2=O)C=C1 |c:10,12,32,t:6,8,21,23|	Z8873685336	**1**{*48*}	CS(=O)(=O)c1ccnc(N)c1	**2**{*331*}	O=Cc1ccc2OCCNC(=O)c2c1	**3**{*35*}	CSCCC[N+]#[C-]	63.4	51

651	**4**{*113*,*246*,*14*}	COCCNC1=C(N=C2N1C=CC=C2N1CCOCC1)C1CC11CCCOC1 |c:7,11,13,t:5|	Z8878918399	**1**{*113*}	Nc1ncccc1N2CCOCC2	**2**{*246*}	O=CC1CC21CCCOC2	**3**{*14*}	COCC[N+]#[C-]	53.2	51
652	**4**{*36*,*46*,*9*}	CNC1=C(CCCCCOC)N=C2C=CC(Br)=CN12 |c:2,13,16,t:11|	Z8873684758	**1**{*36*}	Nc1ccc(Br)cn1	**2**{*46*}	COCCCCCC=O	**3**{*9*}	C[N+]#[C-]	44.7	51
653	**4**{*327*,*332*,*34*}	COC(=O)C1=C(F)C2=NC(=C(NCC3CCCO3)N2C=C1)C1=CN(CC2CC2)N=C1 |c:4,21,32,t:7,9,24|	Z8878918569	**1**{*327*}	COC(=O)c1cnc(N)c1F	**2**{*332*}	O=Cc1cnn(CC2CC2)c1	**3**{*34*}	[C-]#[N+]CC1CCCO1	56.6	51
654	**4**{*358*,*333*,*15*}	CCNC1=C(N=C2C=C(C=CN12)C(C)(C)O)C1CCC2(CC(C2)(OC)OC)CC1 |c:7,9,t:3,5|	Z8878918336	**1**{*358*}	CC(C)(O)c1ccnc(N)c1	**2**{*333*}	COC1(CC2(C1)CCC(CC2)C=O)OC	**3**{*15*}	CC[N+]#[C-]	54.5	50
655	**4**{*66*,*99*,*22*}	COCCC1=CC2=NC(=C(NC3COC3)N2C=C1)C1=COC=N1 |c:18,24,t:4,6,8,21|	Z8873684845	**1**{*66*}	COCCc1ccnc(N)c1	**2**{*99*}	O=Cc1cocn1	**3**{*22*}	[C-]#[N+]C1COC1	42.4	50
656	**4**{*365*,*334*,*10*}	CC1=C(N=CO1)C1=C(NC2CCOC2)N2C(C=C(C)C=C2C)=N1 |c:3,7,21,24,t:1,18|	Z8878918832	**1**{*365*}	Cc1cc(C)nc(N)c1	**2**{*334*}	Cc1ocnc1C=O	**3**{*10*}	[C-]#[N+]C1CCOC1	42.0	50
657	**4**{*366*,*134*,*12*}	COC1=NC(=NC=C1)C1=C(NCC2CCOC2)N2C=CC=C(OC)C2=N1 |c:4,6,9,20,27,t:2,22|	Z8873684742	**1**{*366*}	COc1cccnc1N	**2**{*134*}	COc1ccnc(C=O)n1	**3**{*12*}	[C-]#[N+]CC1COC1	47.8	50
658	**4**{*367*,*335*,*34*}	CC1=CN2C(NCC3CCCO3)=C(N=C2C(=C1)C#C)C1=CN(N=C1)C1CCOCC1 |c:12,14,17,25,t:1,22|	Z8873685329	**1**{*367*}	Cc1cnc(N)c(C#C)c1	**2**{*335*}	O=Cc1cnn(c1)C2CCOCC2	**3**{*34*}	[C-]#[N+]CC1CCCO1	54.5	50
659	**4**{*166*,*336*,*32*}	COC1=NSC=C1C1=C(NC2CCC2)N2C=CC(CSCCO)=CC2=N1 |c:5,8,17,24,27,t:2|	Z8873684839	**1**{*166*}	Nc1cc(CSCCO)ccn1	**2**{*336*}	COc1nscc1C=O	**3**{*32*}	[C-]#[N+]C1CCC1	52.5	50
660	**4**{*368*,*337*,*49*}	CN1C=C(C=N1)C1=C(C)N2C(C=C1)=NC(=C2NC1CC(F)(F)C1)C1=CN(C)C(=O)N(C)C1=O |c:2,4,7,12,14,16,t:27|	Z8873684788	**1**{*368*}	Cc1nc(N)ccc1c2cnn(C)c2	**2**{*337*}	Cn1cc(C=O)c(=O)n(C)c1=O	**3**{*49*}	FC1(F)CC(C1)[N+]#[C-]	61.1	50
661	**4**{*92*,*338*,*7*}	CC1=C(OC=C1)C1=C(NC2CCOCC2)N2C=CC(=CC2=N1)C(N)=O |c:4,7,18,20,23,t:1|	Z8878918819	**1**{*92*}	NC(=O)c1ccnc(N)c1	**2**{*338*}	Cc1ccoc1C=O	**3**{*7*}	[C-]#[N+]C1CCOCC1	45.6	50
662	**4**{*164*,*328*,*24*}	CCC1=NNC=C1C1=C(NCC2=CC(OC)=CC=C2)N2C=CC(CN(C)C)=CC2=N1 |c:5,8,16,18,22,28,31,t:2,12|	Z8873684947	**1**{*164*}	CN(C)Cc1ccnc(N)c1	**2**{*328*}	Cl.CCc1n[nH]cc1C=O	**3**{*24*}	COc1cccc(C[N+]#[C-])c1	54.1	50

663	**4**{*351*,*339*,*67*}	CCC(C)NC1=C(N=C2C=CC3=C(C(O)CC3)N12)C1=NN=C(N1)C(F)(F)F |c:9,23,t:5,7,11,21|	Z8878918810	**1**{*351*}	Nc1ccc2CCC(O)c2n1	**2**{*339*}	FC(F)(F)c1nnc(C=O)[nH]1	**3**{*67*}	CCC(C)[N+]#[C-]	50.9	50
664	**4**{*103*,*165*,*40*}	CCOC(=O)C1=CC=C(NC2=C(N=C3C=NC=C(CO)N23)C2=NN(C=C2)[C@H]2C[C@@H](C2)C(=O)OC)C=C1 |r,c:14,26,38,t:5,7,10,12,16,23|	Z8878918652	**1**{*103*}	Nc1cncc(CO)n1	**2**{*165*}	COC(=O)[C@H]1C[C@@H](C1)n2ccc(C=O)n2	**3**{*40*}	CCOC(=O)c1ccc([N+]#[C-])cc1	65.6	50
665	**4**{*369*,*340*,*18*}	CCN1C=NC(=N1)C1=C(NC(C)(C)C)N2C=C(C=CC2=N1)C(F)C(F)(F)F |c:3,5,8,16,18,21|	Z8878918892	**1**{*369*}	Cl.Nc1ccc(cn1)C(F)C(F)(F)F	**2**{*340*}	CCn1cnc(C=O)n1	**3**{*18*}	CC(C)(C)[N+]#[C-]	51.1	49
666	**4**{*348*,*341*,*39*}	CCOCCNC1=C(N=C2C=NC(F)=CN12)C1=CN=C(S1)C(C)O |c:10,13,20,t:6,8,18|	Z8878918626	**1**{*348*}	Nc1cnc(F)cn1	**2**{*341*}	CC(O)c1ncc(C=O)s1	**3**{*39*}	CCOCC[N+]#[C-]	51.1	49
667	**4**{*54*,*36*,*22*}	CN(C)C(=O)C1=CN2C(C=C1)=NC(=C2NC1COC1)C1=NC(C)=C(OC(F)F)C=C1 |c:9,11,13,31,t:5,22,25|	Z8846491870	**1**{*54*}	Cl.CN(C)C(=O)c1ccc(N)nc1	**2**{*36*}	Cc1nc(C=O)ccc1OC(F)F	**3**{*22*}	[C-]#[N+]C1COC1	55.1	49
668	**4**{*366*,*342*,*12*}	COC1=CC=CN2C(NCC3CCOC3)=C(N=C12)C1=CN=C(S1)C1=NC=CC=C1 |c:4,15,23,29,31,t:2,17,21,27|	Z8873684850	**1**{*366*}	COc1cccnc1N	**2**{*342*}	O=Cc1cnc(s1)c2ccccn2	**3**{*12*}	[C-]#[N+]CC1CCOC1	53.7	49
669	**4**{*370*,*343*,*10*}	COC1=C(Br)C=CN2C(NC3CCOC3)=C(N=C12)C1C(C)C1Cl |c:2,5,15,t:17|	Z8854581178	**1**{*370*}	COc1c(N)nccc1Br	**2**{*343*}	CC1C(Cl)C1C=O	**3**{*10*}	[C-]#[N+]C1CCOC1	52.7	49
670	**4**{*113*,*344*,*6*}	CCOC(=O)CCCNC1=C(N=C2N1C=CC=C2N1CCOCC1)C1=C(C)C=NN1COC |c:11,15,17,27,30,t:9|	Z8873685396	**1**{*113*}	Nc1ncccc1N2CCOCC2	**2**{*344*}	COCn1ncc(C)c1C=O	**3**{*6*}	CCOC(=O)CCC[N+]#[C-]	59.8	49
671	**4**{*50*,*174*,*7*}	CP(C)(=O)C1=CC=CC2=NC(=C(NC3CCOCC3)N12)C1=CN(CC2CCCO2)N=N1 |c:6,33,t:4,8,10,23|	Z8849597810	**1**{*50*}	CP(=O)(C)c1cccc(N)n1	**2**{*174*}	O=Cc1cn(CC2CCCO2)nn1	**3**{*7*}	[C-]#[N+]C1CCOCC1	58.2	49
672	**4**{*105*,*300*,*34*}	CC(C)(C)OC(=O)N1CC(CC2=C(NCC3CCCO3)N3C(C=CC=C3C(N)=O)=N2)C1 |c:11,23,25,30|	Z8878918635	**1**{*105*}	NC(=O)c1cccc(N)n1	**2**{*300*}	CC(C)(C)OC(=O)N1CC(CC=O)C1	**3**{*34*}	[C-]#[N+]CC1CCCO1	55.9	48
673	**4**{*54*,*138*,*53*}	CN(C)C(=O)C1=CN2C(C=C1)=NC(=C2NCCOCC1=CC=CC=C1)C1=NC(C)=NC(C)=C1 |c:9,11,13,23,25,31,34,t:5,21,28|	Z8873684825	**1**{*54*}	Cl.CN(C)C(=O)c1ccc(N)nc1	**2**{*138*}	Cc1cc(C=O)nc(C)n1	**3**{*53*}	[C-]#[N+]CCOCc1ccccc1	57.5	48
674	**4**{*369*,*203*,*7*}	FC(F)CN1C=CC(=N1)C1=C(NC2CCOCC2)N2C=C(C=CC2=N1)C(F)C(F)(F)F |c:5,7,10,21,23,26|	Z8878918909	**1**{*369*}	Cl.Nc1ccc(cn1)C(F)C(F)(F)F	**2**{*203*}	FC(F)Cn1ccc(C=O)n1	**3**{*7*}	[C-]#[N+]C1CCOCC1	57.7	48

675	**4**{*48*,*345*,*22*}	COC1=CC(C2=C(NC3COC3)N3C=CC(=CC3=N2)S(C)(=O)=O)=C(F)C=C1 |c:5,14,16,19,28,t:2,25|	Z8878918335	**1**{*48*}	CS(=O)(=O)c1ccnc(N)c1	**2**{*345*}	COc1ccc(F)c(C=O)c1	**3**{*22*}	[C-]#[N+]C1COC1	49.4	48
676	**4**{*371*,*164*,*10*}	CN1N=C(C2=C1CCOC2)C1=C(NC2CCOC2)N2C=CC=C(OCCCC#N)C2=N1 |c:2,4,12,22,33,t:24|	Z8873685339	**1**{*371*}	Nc1ncccc1OCCCC#N	**2**{*164*}	Cn1nc(C=O)c2COCCc21	**3**{*10*}	[C-]#[N+]C1CCOC1	54.4	48
677	**4**{*327*,*346*,*34*}	CCN(C)C(=O)C1=CC=C(C=C1)C1=C(NCC2CCCO2)N2C=CC(C(=O)OC)=C(F)C2=N1 |c:8,10,13,24,34,t:6,30|	Z8878918338	**1**{*327*}	COC(=O)c1ccnc(N)c1F	**2**{*346*}	CCN(C)C(=O)c1ccc(C=O)cc1	**3**{*34*}	[C-]#[N+]CC1CCCO1	58.5	48
678	**4**{*187*,*70*,*12*}	CC(F)(F)C1=NC(=CS1)C1=C(NCC2CCOC2)N2C=C(Cl)C(=CC2=N1)C#N |c:6,10,24,27,t:4,21|	Z8878918511	**1**{*187*}	Nc1cc(C#N)c(Cl)cn1	**2**{*70*}	CC(F)(F)c1nc(C=O)cs1	**3**{*12*}	[C-]#[N+]CC1CCOC1	54.6	48
679	**4**{*125*,*122*,*31*}	CSCCNC1=C(N=C2C=CC(CCO)=CN12)C1=CN=C(N=C1)C1=CC=CS1 |c:9,14,21,23,28,t:5,7,19,26|	Z8873684811	**1**{*125*}	Nc1ccc(CCO)cn1	**2**{*122*}	O=Cc1cnc(nc1)c2cccs2	**3**{*31*}	CSCC[N+]#[C-]	53.0	48
680	**4**{*92*,*347*,*18*}	CCCN1C=C(N=N1)C1=C(NC(C)(C)C)N2C=CC(=CC2=N1)C(N)=O |c:4,6,9,17,19,22|	Z8855739156	**1**{*92*}	NC(=O)c1ccnc(N)c1	**2**{*347*}	CCCn1cc(C=O)nn1	**3**{*18*}	CC(C)(C)[N+]#[C-]	43.9	48
681	**4**{*347*,*139*,*21*}	CC1=NN(CCO)C(C)=C1C1=C(NCC2CC2)N2C=C(Cl)C=CC2=N1 |c:8,11,23,26,t:1,20|	Z8873685465	**1**{*347*}	Nc1ccc(Cl)cn1	**2**{*139*}	Cc1nn(CCO)c(C)c1C=O	**3**{*21*}	[C-]#[N+]CC1CC1	46.2	48
682	**4**{*372*,*314*,*46*}	CC(=C)CNC1=C(N=C2N1C=CC=C2OCC1=CC=NC=C1)C1=CNN=C1 |c:7,11,13,20,22,28,t:5,18,25|	Z8873684836	**1**{*372*}	Nc1ncccc1OCc2ccncc2	**2**{*314*}	O=Cc1cn[nH]c1	**3**{*46*}	CC(=C)C[N+]#[C-]	46.0	47
683	**4**{*79*,*30*,*13*}	COC1=CC=C(CNC2=C(N=C3C=NC=C(C)N23)C2CCN(CC2)C(C)=O)C=C1 |c:12,30,t:2,4,8,10,14|	Z8878918306	**1**{*79*}	Cc1cncc(N)n1	**2**{*30*}	CC(=O)N1CCC(CC1)C=O	**3**{*13*}	COc1ccc(C[N+]#[C-])cc1	50.0	47
684	**4**{*48*,*348*,*22*}	CC(C)(C)OC(=O)N1CC2CCC1C(C2)C1=C(NC2COC2)N2C=CC(=CC2=N1)S(C)(=O)=O |c:17,26,28,31|	Z8878918482	**1**{*48*}	CS(=O)(=O)c1ccnc(N)c1	**2**{*348*}	CC(C)(C)OC(=O)N1CC2CCC1C(C2)C=O	**3**{*22*}	[C-]#[N+]C1COC1	64.6	47
685	**4**{*48*,*345*,*15*}	CCNC1=C(N=C2C=C(C=CN12)S(C)(=O)=O)C1=C(F)C=CC(OC)=C1 |c:7,9,18,21,25,t:3,5|	Z8878918575	**1**{*48*}	CS(=O)(=O)c1ccnc(N)c1	**2**{*345*}	COc1ccc(F)c(C=O)c1	**3**{*15*}	CC[N+]#[C-]	46.0	47
686	**4**{*367*,*349*,*34*}	CC1=CN2C(NCC3CCCO3)=C(N=C2C(=C1)C#C)C1C[C@H]2OC(C)(C)O[C@H]2C1 |c:12,14,17,t:1|	Z8873684754	**1**{*367*}	Cc1cnc(N)c(C#C)c1	**2**{*349*}	CC1(C)O[C@@H]2CC(C[C@@H]2O1)C=O	**3**{*34*}	[C-]#[N+]CC1CCCO1	50.0	47

687	**4**{*293*,*350*,*12*}	COC1=CC(C)=CN2C(NCC3CCOC3)=C(N=C12)C1C2CCOCC12 |c:5,16,t:2,18|	Z8873684691	**1**{*293*}	COc1cc(C)cnc1N	**2**{*350*}	O=CC1C2CCOCC12	**3**{*12*}	[C-]#[N+]CC1CCOC1	44.9	47
688	**4**{*373*,*351*,*39*}	CCOCCNC1=C(CCCSC)N=C2C=CC(CNC(=O)OC(C)(C)C)=CN12 |c:6,15,26,t:13|	Z8878918621	**1**{*373*}	CC(C)(C)OC(=O)NCc1ccc(N)nc1	**2**{*351*}	CSCCCC=O	**3**{*39*}	CCOCC[N+]#[C-]	54.2	46
689	**4**{*230*,*198*,*34*}	CCC1=NN(C)C=C1C1=C(NCC2CCCO2)N2C=C(Br)C=C(C(C)O)C2=N1 |c:6,9,29,t:2,20,23|	Z8810902957	**1**{*230*}	CC(O)c1cc(Br)cnc1N	**2**{*198*}	CCc1nn(C)cc1C=O	**3**{*34*}	[C-]#[N+]CC1CCCO1	56.1	46
690	**4**{*374*,*352*,*70*}	COCCCC1=C(NC2CCN(C2)C(=O)OC(C)(C)C)N2C(C=CC=C2C(=O)OC)=N1 |c:5,23,25,31|	Z8878918489	**1**{*374*}	COC(=O)c1cccc(N)n1	**2**{*352*}	COCCCC=O	**3**{*70*}	CC(C)(C)OC(=O)N1CCC(C1)[N+]#[C-]	54.0	46
691	**4**{*375*,*139*,*10*}	CC(C)OC1=CN=CC2=NC(=C(NC3CCOC3)N12)C1=C(C)N(CCO)N=C1C |c:6,22,29,t:4,8,10|	Z8873684781	**1**{*375*}	CC(C)Oc1cncc(N)n1	**2**{*139*}	Cc1nn(CCO)c(C)c1C=O	**3**{*10*}	[C-]#[N+]C1CCOC1	50.0	46
692	**4**{*150*,*223*,*35*}	COC(=O)C1=NC=C(C=C1)C1=C(NCCCSC)N2C=CC(=CC2=N1)C1CCOC1 |c:6,8,11,20,22,25,t:4|	Z8855619591	**1**{*150*}	Nc1cc(ccn1)C2CCOC2	**2**{*223*}	Cl.COC(=O)c1ccc(C=O)cn1	**3**{*35*}	CSCCC[N+]#[C-]	53.0	46
693	**4**{*167*,*353*,*23*}	COC(=O)CCC(NC1=C(N=C2N1C(C)=CN=C2C)C1=C(CF)C=CC=C1)C(=O)OC |c:10,15,17,21,25,27,t:8|	Z8878918618	**1**{*167*}	Cc1cnc(C)c(N)n1	**2**{*353*}	FCc1ccccc1C=O	**3**{*23*}	COC(=O)CCC([N+]#[C-])C(=O)OC	53.2	46
694	**4**{*77*,*354*,*5*}	COCCCNC1=C(N=C2C=CC(=CN12)C1=CN=CC=C1)C1=C(C=CC=C1)P(C)(C)=O |c:10,12,19,21,26,28,t:6,8,17,24|	Z8873684627	**1**{*77*}	Nc1ccc(cn1)c2cccnc2	**2**{*354*}	CP(=O)(C)c1ccccc1C=O	**3**{*5*}	COCCC[N+]#[C-]	53.8	46
695	**4**{*206*,*62*,*14*}	COCCNC1=C(N=C2N1C=CC(I)=C2F)C1CC11CCOCC1 |c:7,11,14,t:5|	Z8873684638	**1**{*206*}	Nc1nccc(I)c1F	**2**{*62*}	O=CC1CC21CCOCC2	**3**{*14*}	COCC[N+]#[C-]	55.1	46
696	**4**{*353*,*355*,*15*}	CCNC1=C(N=C2C=CC(NS(C)(=O)=O)=CN12)C1=C(CCNC(=O)OC(C)(C)C)OC=N1 |c:7,14,19,32,t:3,5|	Z8873684671	**1**{*353*}	Cl.CS(=O)(=O)Nc1ccc(N)nc1	**2**{*355*}	CC(C)(C)OC(=O)NCCc1ocnc1C=O	**3**{*15*}	CC[N+]#[C-]	57.1	46
697	**4**{*69*,*242*,*61*}	CNC(=O)C1=CC2=NC(=C(NCCCNC(=O)OC(C)(C)C)N2C=C1)C1=CN2N=CC(C#N)=C2N=C1 |c:24,30,34,37,t:4,6,8,27|	Z8873685295	**1**{*69*}	CNC(=O)c1ccnc(N)c1	**2**{*242*}	O=Cc1cnc2c(C#N)cnn2c1	**3**{*61*}	CC(C)(C)OC(=O)NCCC[N+]#[C-]	60.0	45
698	**4**{*50*,*356*,*18*}	CC(C)CN1C=C(N=N1)C1=C(NC(C)(C)C)N2C(C=CC=C2P(C)(C)=O)=N1 |c:5,7,10,19,21,27|	Z8873684868	**1**{*50*}	CP(=O)(C)c1cccc(N)n1	**2**{*356*}	CC(C)Cn1cc(C=O)nn1	**3**{*18*}	CC(C)(C)[N+]#[C-]	47.5	45

699	**4**{*109*,*357*,*50*}	CCC1=C(C(C)=NO1)C1=C(NC2=CC3=C(OCCO3)C=C2)N2C(C=CC=C2S(N)(=O)=O)=N1 |c:5,9,21,26,28,34,t:2,12,14|	Z8878918305	**1**{*109*}	Cl.Nc1cccc(n1)S(=O)(=O)N	**2**{*357*}	CCc1onc(C)c1C=O	**3**{*50*}	[C-]#[N+]c1ccc2OCCOc2c1	55.6	45
700	**4**{*113*,*358*,*14*}	COCCNC1=C(N=C2N1C=CC=C2N1CCOCC1)C1CC11CCN(C1)C(=O)OC(C)(C)C |c:7,11,13,t:5|	Z8878918398	**1**{*113*}	Nc1ncccc1N2CCOCC2	**2**{*358*}	CC(C)(C)OC(=O)N1CCC2(CC2C=O)C1	**3**{*14*}	COCC[N+]#[C-]	57.4	45
701	**4**{*48*,*359*,*22*}	COC1=CC(=CC(=C1)C1=C(NC2COC2)N2C=CC(=CC2=N1)S(C)(=O)=O)C(F)(F)F |c:4,6,9,18,20,23,t:2|	Z8873684872	**1**{*48*}	CS(=O)(=O)c1ccnc(N)c1	**2**{*359*}	COc1cc(C=O)cc(c1)C(F)(F)F	**3**{*22*}	[C-]#[N+]C1COC1	53.3	45
702	**4**{*47*,*195*,*26*}	COC1=C(F)C=C(C=N1)C1=C(NCC2=CC=C(F)C=C2)N2C=CC(=CC2=N1)S(N)(=O)=O |c:2,5,7,10,19,23,25,28,t:14,16|	Z8849597834	**1**{*47*}	Cl.Nc1cc(ccn1)S(=O)(=O)N	**2**{*195*}	COc1ncc(C=O)cc1F	**3**{*26*}	Fc1ccc(C[N+]#[C-])cc1	53.5	44
703	**4**{*376*,*360*,*12*}	COC1=NC(Cl)=CN2C(NCC3CCOC3)=C(N=C12)C1=C2N=CN(C)C2=CC=C1 |c:5,16,22,24,29,31,t:2,18|	Z8873684704	**1**{*376*}	COc1nc(Cl)cnc1N	**2**{*360*}	Cn1cnc2c(C=O)cccc12	**3**{*12*}	[C-]#[N+]CC1CCOC1	49.4	44
704	**4**{*38*,*7*,*9*}	CNC1=C(N=C2C=C(C=CN12)C#N)C1=NC(C)=CC(CO)=C1 |c:6,8,18,22,t:2,4,15|	Z8873685471	**1**{*38*}	Nc1cc(C#N)ccn1	**2**{*7*}	Cc1cc(CO)cc(C=O)n1	**3**{*9*}	C[N+]#[C-]	35.0	44
705	**4**{*91*,*361*,*7*}	NC(=O)C1=CN2C(C=C1)=NC(=C2NC1CCOCC1)C1=C(Br)C=CC(Br)=N1 |c:7,9,11,22,25,28,t:3|	Z8873685278	**1**{*91*}	NC(=O)c1ccc(N)nc1	**2**{*361*}	Brc1ccc(Br)c(C=O)n1	**3**{*7*}	[C-]#[N+]C1CCOCC1	59.0	44
706	**4**{*104*,*362*,*39*}	CCOCCNC1=C(N=C2C=CC3=C(COCC3)N12)C1=CC(OC)=C(OC)C(OC)=C1 |c:10,32,t:6,8,12,22,26|	Z8846492263	**1**{*104*}	Nc1ccc2CCOCc2n1	**2**{*362*}	COc1cc(C=O)cc(OC)c1OC	**3**{*39*}	CCOCC[N+]#[C-]	50.3	44
707	**4**{*54*,*93*,*22*}	CN(C)C(=O)C1=CN2C(C=C1)=NC(=C2NC1COC1)C1=NC(CO)=CC=C1 |c:9,11,13,26,28,t:5,22|	Z8829498567	**1**{*54*}	Cl.CN(C)C(=O)c1ccc(N)nc1	**2**{*93*}	OCc1cccc(C=O)n1	**3**{*22*}	[C-]#[N+]C1COC1	43.0	43
708	**4**{*193*,*169*,*12*}	CNC(=O)C1=CN2C(C=C1)=NC(=C2NCC1CCOC1)C1=NNC2=C1N=CC=C2 |c:8,10,12,26,29,31,t:4,23|	Z8803896944	**1**{*193*}	CNC(=O)c1ccc(N)nc1	**2**{*169*}	O=Cc1n[nH]c2cccnc12	**3**{*12*}	[C-]#[N+]CC1CCOC1	45.8	43
709	**4**{*79*,*307*,*13*}	COC1=CC=C(CNC2=C(N=C3C=NC=C(C)N23)C2=CN=C(Cl)N2C)C=C1 |c:12,28,t:2,4,8,10,14,20,22|	Z8855739194	**1**{*79*}	Cc1cncc(N)n1	**2**{*307*}	Cn1c(Cl)ncc1C=O	**3**{*13*}	COc1ccc(C[N+]#[C-])cc1	44.3	43
710	**4**{*62*,*363*,*6*}	CCOC(=O)CCCNC1=C(N=C2C=C(CO)C=CN12)C1=CNC(C)=N1 |c:17,26,t:9,11,13,22|	Z8878918835	**1**{*62*}	Nc1cc(CO)ccn1	**2**{*363*}	Cc1nc(C=O)c[nH]1	**3**{*6*}	CCOC(=O)CCC[N+]#[C-]	41.0	42

711	**4**{*99*,*364*,*66*}	CC(NC1=C(CCC2CCCO2)N=C2C=CC(=CN1 2)S(N)(=O)=O)C1=CC=CC=C1 |c:3,15,17,28,30,t:13,26|	Z8873685354	**1**{*99*}	Nc1ccc(cn1)S(=O)(=O) N	**2**{*364*}	O=CCCC1C CCO1	**3**{*66*}	CC([N+]#[C-])c1ccccc1	47.5	42
712	**4**{*139*,*365*,*10*}	FC(F)C1=CC2=NC(=C(NC3CCOC3)N2C=C1) C1=NNC2=C1C=NC=C2 |c:18,24,27,29,t:3,5,7,21|	Z8873684877	**1**{*139*}	Nc1cc(ccn1)C(F)F	**2**{*365*}	O=Cc1n[nH] c2ccncc12	**3**{*10*}	[C-]#[N+]C1CC OC1	41.9	42
713	**4**{*92*,*366*,*26*}	CCN1C=NC(=C1)C1=C(NCC2=CC=C(F)C=C 2)N2C=CC(=CC2=N1)C(N)=O |c:3,5,8,17,21,23,26,t:12,14|	Z8878918679	**1**{*92*}	NC(=O)c1c cnc(N)c1	**2**{*366*}	CCn1cnc(C= O)c1	**3**{*26*}	Fc1ccc(C[N+]#[C-])cc1	42.6	42
714	**4**{*101*,*89*,*6*}	CCOC(=O)CCCNC1=C(N=C2N1C=C(CO)C= C2Cl)C1=CN=C(CC)N=C1 |c:11,19,29,t:9,15,23,25|	Z8873685362	**1**{*101*}	Nc1ncc(CO)cc1Cl	**2**{*89*}	CCc1ncc(C= O)cn1	**3**{*6*}	CCOC(=O)C CC[N+]#[C-]	46.8	41
715	**4**{*179*,*42*,*5*}	COCCCNC1=C(N=C2C=C(OC)C=CN12)C1= CN(C)N=C1Cl |c:14,23,t:6,8,10,19|	Z8878918672	**1**{*179*}	COc1ccnc(N)c1	**2**{*42*}	Cn1cc(C=O) c(Cl)n1	**3**{*5*}	COCCC[N+] #[C-]	39.2	41
716	**4**{*377*,*367*,*12*}	NC(=O)C1=CC(=CC=C1)C1=C(NCC2CCOC2)N2C=CC=C(OCC(F)F)C2=N1 |c:5,7,10,21,31,t:3,23|	Z8854581151	**1**{*377*}	Nc1ncccc1 OCC(F)F	**2**{*367*}	NC(=O)c1cc cc(C=O)c1	**3**{*12*}	[C-]#[N+]CC1C COC1	46.6	41
717	**4**{*127*,*320*,*29*}	CCOC(=O)CCNC1=C(N=C2N1C=CC=C2F)C 1=NN(C)C=N1 |c:10,14,16,24,t:8,20|	Z8878918360	**1**{*127*}	Nc1ncccc1F	**2**{*320*}	Cn1cnc(C=O)n1	**3**{*29*}	CCOC(=O)C C[N+]#[C-]	37.2	41
718	**4**{*32*,*11*,*18*}	CC(C)(C)NC1=C(N=C2C=C(C=CN12)P(C)(C) =O)C1=NN(CC(F)(F)F)C=C1 |c:9,11,28,t:5,7,20|	Z8878918527	**1**{*32*}	CP(=O)(C)c 1ccnc(N)c1	**2**{*11*}	FC(F)(F)Cn1 ccc(C=O)n1	**3**{*18*}	CC(C)(C)[N +]#[C-]	46.2	41
719	**4**{*377*,*368*,*12*}	CC1=C(C)N=NC(=C1)C1=C(NCC2CCOC2)N 2C=CC=C(OCC(F)F)C2=N1 |c:1,4,6,9,20,30,t:22|	Z8846492294	**1**{*377*}	Nc1ncccc1 OCC(F)F	**2**{*368*}	Cc1cc(C=O) nnc1C	**3**{*12*}	[C-]#[N+]CC1C COC1	45.1	41
720	**4**{*378*,*369*,*24*}	COC1=CC(CNC2=C(N=C3N2C=CC=C3OCC 2=CN=CC=C2)C2=CON=C2C)=CC=C1 |c:9,13,15,22,24,30,33,35,t:2,7,20,27|	Z8878918644	**1**{*378*}	Nc1ncccc1 OCc2cccnc 2	**2**{*369*}	Cc1nocc1C= O	**3**{*24*}	COc1cccc(C[N+]#[C-])c1	49.2	41
721	**4**{*73*,*370*,*20*}	CNC(=O)C1=CC=C(C=C1)C1=C(NC2=C(C)C =C(OC)C=C2)N2N3C=CN=C3C=CC2=N1 |c:6,8,11,14,21,26,28,31,34,t:4,17|	Z8873684621	**1**{*73*}	Nc1ccc2ncc n2n1	**2**{*370*}	CNC(=O)c1c cc(C=O)cc1	**3**{*20*}	COc1ccc([N +]#[C-])c(C)c1	47.2	41
722	**4**{*267*,*371*,*23*}	COC(=O)CCC(NC1=C(N=C2C=CC=C(C#C)N 12)C1=C(C)C(OC)=CC=N1)C(=O)OC |c:12,21,26,28,t:8,10,14|	Z8878918809	**1**{*267*}	Nc1cccc(C# C)n1	**2**{*371*}	COc1ccnc(C =O)c1C	**3**{*23*}	COC(=O)CC C([N+]#[C-])C(=O)OC	48.3	41
723	**4**{*50*,*11*,*18*}	CC(C)(C)NC1=C(N=C2C=CC=C(N12)P(C)(C) =O)C1=NN(CC(F)(F)F)C=C1 |c:9,11,28,t:5,7,20|	Z8878918561	**1**{*50*}	CP(=O)(C)c 1cccc(N)n1	**2**{*11*}	FC(F)(F)Cn1 ccc(C=O)n1	**3**{*18*}	CC(C)(C)[N +]#[C-]	45.7	41

724	**4**{*105* ,*185* ,*41* }	COC1=CC=C(NC2=C(N=C3C=CC=C(N23)C(N)=O)C2=CN(C)C=N2)C=C1 |c:11,13,25,28,t:2,4,7,9,21|	Z8855619757	**1**{*105* }	NC(=O)c1cccc(N)n1	**2**{*185* }	Cn1cnc(C=O)c1	**3**{*41* }	COc1ccc([N+]#[C-])cc1	40.1	41
725	**4**{*188* ,*372* ,*62* }	COC(=O)C1=CN=CC2=NC(=C(NC3(CCCC3)C(=O)OC)N12)C1=NN(C)N=C1C |c:6,30,t:4,8,10,26|	Z8873684844	**1**{*188* }	COC(=O)c1cncc(N)n1	**2**{*372* }	Cc1nn(C)nc1C=O	**3**{*62* }	COC(=O)C1(CCCC1)[N+]#[C-]	45.3	41
726	**4**{*92* ,*204* ,*26* }	CSC1=NC=C(N1C)C1=C(NCC2=CC=C(F)C=C2)N2C=CC(=CC2=N1)C(N)=O |c:4,9,18,22,24,27,t:2,13,15|	Z8854581172	**1**{*92* }	NC(=O)c1ccnc(N)c1	**2**{*204* }	CSc1ncc(C=O)n1C	**3**{*26* }	Fc1ccc(C[N+]#[C-])cc1	44.8	40
727	**4**{*113* ,*373* ,*14* }	COCCNC1=C(N=C2N1C=CC=C2N1CCOCC1)[C@@H]1[C@@H](C)C1(F)F |&1:20,21,r,c:7,11,13,t:5|	Z8855619505	**1**{*113* }	Nc1ncccc1N2CCOCC2	**2**{*373* }	C[C@@H]1[C@@H](C=O)C1(F)F |&1:1,2,r|	**3**{*14* }	COCC[N+]#[C-]	39.9	40
728	**4**{*28* ,*374* ,*7* }	C(C1CC1)N1C=C(N=N1)C1=C(NC2CCOCC2)N2C=CC(=CC2=N1)C1=NOC=N1 |c:6,8,11,22,24,27,33,t:30|	Z8878918456	**1**{*28* }	Cl.Nc1cc(ccn1)c2ncon2	**2**{*374* }	O=Cc1cn(CC2CC2)nn1	**3**{*7* }	[C-]#[N+]C1CCOCC1	44.3	40
729	**4**{*142* ,*375* ,*24* }	COCC1=C(N=CO1)C1=C(NCC2=CC(OC)=CC=C2)N2C=CC=C(OCCS(C)(=O)=O)C2=N1 |c:5,9,17,19,23,35,t:3,13,25|	Z8873684680	**1**{*142* }	CS(=O)(=O)CCOc1cccnc1N	**2**{*375* }	COCc1ocnc1C=O	**3**{*24* }	COc1cccc(C[N+]#[C-])c1	53.0	40
730	**4**{*103* ,*376* ,*5* }	COCCCNC1=C(N=C2C=NC=C(CO)N12)C1=C(Br)SC(=N1)C1CC1 |c:10,19,23,t:6,8,12|	Z8873684652	**1**{*103* }	Nc1cncc(CO)n1	**2**{*376* }	Brc1sc(nc1C=O)C2CC2	**3**{*5* }	COCCC[N+]#[C-]	47.7	40
731	**4**{*369* ,*340* ,*31* }	CCN1C=NC(=N1)C1=C(NCCSC)N2C=C(C=CC2=N1)C(F)C(F)(F)F |c:3,5,8,16,18,21|	Z8873685474	**1**{*369* }	Cl.Nc1ccc(cn1)C(F)C(F)(F)F	**2**{*340* }	CCn1cnc(C=O)n1	**3**{*31* }	CSCC[N+]#[C-]	43.6	40
732	**4**{*50* ,*295* ,*18* }	CC1=NC(=CS1)C1=C(NC(C)(C)C)N2C(C=CC=C2P(C)(C)=O)=N1 |c:3,7,16,18,24,t:1|	Z8846492023	**1**{*50* }	CP(=O)(C)c1cccc(N)n1	**2**{*295* }	Cc1nc(C=O)cs1	**3**{*18* }	CC(C)(C)[N+]#[C-]	39.1	40
733	**4**{*379* ,*377* ,*7* }	CC1=CNN=C1C1=C(NC2CCOCC2)N2C=CC=C(Br)C2=N1 |c:4,7,18,24,t:1,20|	Z8835022881	**1**{*379* }	Nc1ncccc1Br	**2**{*377* }	Cl.Cc1c[nH]nc1C=O	**3**{*7* }	[C-]#[N+]C1CCOCC1	40.4	40
734	**4**{*179* ,*378* ,*5* }	COCCCNC1=C(N=C2C=C(OC)C=CN12)C1=NC(=CC(Cl)=C1)C(=O)OC |c:14,21,24,t:6,8,10,19|	Z8878918656	**1**{*179* }	COc1ccnc(N)c1	**2**{*378* }	COC(=O)c1cc(Cl)cc(C=O)n1	**3**{*5* }	COCCC[N+]#[C-]	43.4	40
735	**4**{*81* ,*116* ,*13* }	COC1=CC=C(CNC2=C(N=C3C=NC=CN23)C2=CN(CCC=C)N=N2)C=C1 |c:12,14,26,29,t:2,4,8,10,19|	Z8878918775	**1**{*81* }	Nc1cnccn1	**2**{*116* }	C=CCCn1cc(C=O)nn1	**3**{*13* }	COc1ccc(C[N+]#[C-])cc1	40.2	40

736	**4**{*48* ,*379* ,*22* }	CC1=C(Br)SC(=C1)C1=C(NC2COC2)N2C=CC(=CC2=N1)S(C)(=O)=O |c:1,5,8,17,19,22|	Z8878918403	**1**{*48* }	CS(=O)(=O)c1ccnc(N)c1	**2**{*379* }	Cc1cc(C=O)sc1Br	**3**{*22* }	[C-]#[N+]C1COC1	43.1	40
737	**4**{*36* ,*40* ,*9* }	CNC1=C(N=C2C=CC(Br)=CN12)C1=NC(CO)=CS1 |c:6,9,18,t:2,4,14|	Z8849597803	**1**{*36* }	Nc1ccc(Br)cn1	**2**{*40* }	OCc1csc(C=O)n1	**3**{*9* }	C[N+]#[C-]	36.1	39
738	**4**{*375* ,*334* ,*43* }	CC(C)OC1=CN=CC2=NC(=C(NC3CCN(CC3)C(=O)OC(C)(C)C)N12)C1=C(C)OC=N1 |c:6,30,34,t:4,8,10|	Z8873684703	**1**{*375* }	CC(C)Oc1cncc(N)n1	**2**{*334* }	Cc1ocnc1C=O	**3**{*43* }	CC(C)(C)OC(=O)N1CCC(CC1)[N+]#[C-]	48.4	39
739	**4**{*81* ,*257* ,*13* }	COC1=CC=C(CNC2=C(N=C3C=NC=CN23)C2CC(C2)NC(=O)OC(C)(C)C)C=C1 |c:12,14,32,t:2,4,8,10|	Z8873684711	**1**{*81* }	Nc1cnccn1	**2**{*257* }	CC(C)(C)OC(=O)NC1CC(C1)C=O	**3**{*13* }	COc1ccc(C[N+]#[C-])cc1	44.8	39
740	**4**{*41* ,*116* ,*13* }	COC1=CC=C(CNC2=C(N=C3C=NC(C)=CN23)C2=CN(CCC=C)N=N2)C=C1 |c:12,15,27,30,t:2,4,8,10,20|	Z8878918374	**1**{*41* }	Cc1cnc(N)cn1	**2**{*116* }	C=CCCn1cc(C=O)nn1	**3**{*13* }	COc1ccc(C[N+]#[C-])cc1	41.2	39
741	**4**{*91* ,*380* ,*7* }	CC(C)(C)OC(=O)N1CCC(C)(CC2=C(NC3CCOCC3)N3C=C(C=CC3=N2)C(N)=O)C1 |c:13,24,26,29|	Z8878918828	**1**{*91* }	NC(=O)c1ccc(N)nc1	**2**{*380* }	CC(C)(C)OC(=O)N1CCC(C)(CC=O)C1	**3**{*7* }	[C-]#[N+]C1CCOCC1	47.7	39
742	**4**{*142* ,*326* ,*21* }	CN1N=CC=C1C1=C(NCC2CC2)N2C=CC=C(OCCS(C)(=O)=O)C2=N1 |c:2,4,7,16,28,t:18|	Z8878918680	**1**{*142* }	CS(=O)(=O)CCOc1cccnc1N	**2**{*326* }	Cn1nccc1C=O	**3**{*21* }	[C-]#[N+]CC1CC1	40.5	39
743	**4**{*146* ,*139* ,*10* }	CC1=NN(CCO)C(C)=C1C1=C(NC2CCOC2)N2C=C(C=CC2=N1)S(C)(=O)=O |c:8,11,21,23,26,t:1|	Z8878918388	**1**{*146* }	CS(=O)(=O)c1ccc(N)nc1	**2**{*139* }	Cc1nn(CCO)c(C)c1C=O	**3**{*10* }	[C-]#[N+]C1CCOC1	43.6	38
744	**4**{*65* ,*381* ,*5* }	CCC1=CN=CC2=NC(=C(NCCCOC)N12)C1=C2N(C)C=NC2=CC=C1 |c:4,19,23,26,28,t:2,6,8|	Z8855619749	**1**{*65* }	CCc1cncc(N)n1	**2**{*381* }	Cn1cnc2cccc(C=O)c12	**3**{*5* }	COCCC[N+]#[C-]	37.8	38
745	**4**{*199* ,*382* ,*34* }	COC(C)(C)CC1=C(NCC2CCCO2)N2C=CC=C(C2=N1)C(C)(F)F |c:6,17,19,22|	Z8873685428	**1**{*199* }	CC(F)(F)c1cccnc1N	**2**{*382* }	COC(C)(C)CC=O	**3**{*34* }	[C-]#[N+]CC1CCCO1	38.0	38
746	**4**{*181* ,*62* ,*14* }	COCCNC1=C(N=C2N1C=CC1=C2N=CC=N1)C1CC11CCOCC1 |c:7,11,13,16,18,t:5|	Z8878918816	**1**{*181* }	Nc1nccc2nccnc12	**2**{*62* }	O=CC1CC21CCOCC2	**3**{*14* }	COCC[N+]#[C-]	36.3	38
747	**4**{*380* ,*383* ,*5* }	COCCCNC1=C(N=C2N1C=C(Cl)N=C2C)C1=NC=C(C)C=C1 |c:8,15,24,t:6,12,19,21|	Z8878918328	**1**{*380* }	Cc1nc(Cl)cnc1N	**2**{*383* }	Cc1ccc(C=O)nc1	**3**{*5* }	COCCC[N+]#[C-]	35.3	38

748	**4**{*218*,*384*,*10*}	CN1N=C(C=C1C(F)F)C1=C(NC2CCOC2)N2N=C(C=CC2=N1)C(C)(C)C |c:2,4,10,20,22,25|	Z8878918504	**1**{*218*}	CC(C)(C)c1ccc(N)nn1	**2**{*384*}	Cn1nc(C=O)cc1C(F)F	**3**{*10*}	[C-]#[N+]C1CCOC1	39.9	38
749	**4**{*78*,*385*,*44*}	CC1=CN2N3C(NCCCCNC(=O)OC(C)(C)C)=C(N=C3C=C(C)C2=N1)C1=CC(=CC=C1)[N+]([O-])=O |c:18,20,27,32,34,t:1,23,30|	Z8855739078	**1**{*78*}	Cc1cn2nc(N)cc(C)c2n1	**2**{*385*}	[O-][N+](=O)c1cccc(C=O)c1	**3**{*44*}	CC(C)(C)OC(=O)NCCCC[N+]#[C-]	50.3	38
750	**4**{*381*,*378*,*5*}	COCCCNC1=C(N=C2C=NC=C(OCC(F)(F)F)N12)C1=NC(=CC(Cl)=C1)C(=O)OC |c:10,25,28,t:6,8,12,23|	Z8878918804	**1**{*381*}	Nc1cncc(OCC(F)(F)F)n1	**2**{*378*}	COC(=O)c1cc(Cl)cc(C=O)n1	**3**{*5*}	COCCC[N+]#[C-]	48.2	38
751	**4**{*185*,*386*,*23*}	COC(=O)CCC(NC1=C(N=C2C=CC(=C(C)N12)S(C)(=O)=O)[C@H]1CC1(C)C)C(=O)OC |c:12,t:8,10,14|	Z8878918777	**1**{*185*}	Cc1nc(N)ccc1S(=O)(=O)C	**2**{*386*}	CC1(C)C[C@@H]1C=O	**3**{*23*}	COC(=O)CCC([N+]#[C-])C(=O)OC	45.9	38
752	**4**{*371*,*114*,*12*}	COC(=O)[C@@H]1CC[C@@H](C1)C1=C(NCC2CCOC2)N2C=CC=C(OCCCC#N)C2=N1 |&1:4,7,r,c:10,21,32,t:23|	Z8873684896	**1**{*371*}	Nc1ncccc1OCCCC#N	**2**{*114*}	COC(=O)[C@@H]1CC[C@@H](C1)C=O |&1:4,7,r|	**3**{*12*}	[C-]#[N+]CC1CCOC1	43.1	37
753	**4**{*50*,*387*,*14*}	CCN1N=C(C)C=C1C1=C(NCCOC)N2C(C=CC=C2P(C)(C)=O)=N1 |c:6,9,18,20,26,t:3|	Z8829498583	**1**{*50*}	CP(=O)(C)c1cccc(N)n1	**2**{*387*}	CCn1nc(C)cc1C=O	**3**{*14*}	COCC[N+]#[C-]	37.6	37
754	**4**{*382*,*233*,*62*}	COC(=O)C1(CCCC1)NC1=C(CCCOCC=C)N=C2C=NC(=CN12)C(F)(F)F |c:11,22,24,t:20|	Z8849597830	**1**{*382*}	Nc1cnc(cn1)C(F)(F)F	**2**{*233*}	C=CCOCCCC=O	**3**{*62*}	COC(=O)C1(CCCC1)[N+]#[C-]	42.6	37
755	**4**{*187*,*16*,*10*}	CN1N=CC(CNC(=O)OC(C)(C)C)=C1C1=C(NC2CCOC2)N2C=C(Cl)C(=CC2=N1)C#N |c:2,13,16,29,32,t:26|	Z8873684856	**1**{*187*}	Nc1cc(C#N)c(Cl)cn1	**2**{*16*}	Cn1ncc(CNC(=O)OC(C)(C)C)c1C=O	**3**{*10*}	[C-]#[N+]C1CCOC1	47.1	37
756	**4**{*144*,*99*,*53*}	CNS(=O)(=O)C1=CN2C(NCCOCC3=CC=CC=C3)=C(N=C2C=C1)C1=COC=N1 |c:16,18,20,22,25,31,t:5,14,28|	Z8873684634	**1**{*144*}	CNS(=O)(=O)c1ccc(N)nc1	**2**{*99*}	O=Cc1cocn1	**3**{*53*}	[C-]#[N+]CCOCc1ccccc1	42.0	36
757	**4**{*383*,*388*,*69*}	COC1=NN(C)C=C1C1=C(NC2=C(OC)C=CC=C2)N2N=C(C=CC2=N1)C1=NC=CC=C1 |c:6,9,12,16,18,22,24,27,32,34,t:2,30|	Z8854581142	**1**{*383*}	Nc1ccc(nn1)c2ccccn2	**2**{*388*}	COc1nn(C)cc1C=O	**3**{*69*}	COc1ccccc1[N+]#[C-]	42.0	36
758	**4**{*119*,*236*,*61*}	CC(C)(C)OC(=O)NCCCNC1=C(CCC2OCCO2)N=C2C=CC(=CN12)C#N |c:12,24,26,t:22|	Z8878918645	**1**{*119*}	Nc1ccc(C#N)cn1	**2**{*236*}	O=CCCC1OCCO1	**3**{*61*}	CC(C)(C)OC(=O)NCCC[N+]#[C-]	40.7	36

759	**4**{*384* ,*104* ,*10* }	CCOC1=C(C)N2C(NC3CCOC3)=C(N=C2C=C1)C1=NC(C)=CO1 |c:3,14,16,19,25,t:22|	Z8873684881	**1**{*384* }	CCOc1ccc(N)nc1C	**2**{*104* }	Cc1coc(C=O)n1	**3**{*10* }	[C-]#[N+]C1CCOC1	33.6	36
760	**4**{*48* ,*389* ,*22* }	CS(=O)(=O)C1=CC2=NC(=C(NC3COC3)N2C=C1)C1=C(F)C(Cl)=C(F)C=C1 |c:18,21,28,t:4,6,8,25|	Z8873684750	**1**{*48* }	CS(=O)(=O)c1ccnc(N)c1	**2**{*389* }	Fc1ccc(C=O)c(F)c1Cl	**3**{*22* }	[C-]#[N+]C1COC1	40.6	36
761	**4**{*385* ,*282* ,*67* }	CCC(C)NC1=C(N=C2C=C(C#N)C3=C(C=CC=C3)N12)C1CC2(C1)OCCO2 |c:15,17,t:5,7,9,13|	Z8878918363	**1**{*385* }	Nc1cc(C#N)c2ccccc2n1	**2**{*282* }	O=CC1CC2(C1)OCCO2	**3**{*67* }	CCC(C)[N+]#[C-]	36.9	36
762	**4**{*92* ,*125* ,*26* }	COC1=NC=NC(=C1)C1=C(NCC2=CC=C(F)C=C2)N2C=CC(=CC2=N1)C(N)=O |c:4,6,9,18,22,24,27,t:2,13,15|	Z8878918302	**1**{*92* }	NC(=O)c1ccnc(N)c1	**2**{*125* }	COc1cc(C=O)ncn1	**3**{*26* }	Fc1ccc(C[N+]#[C-])cc1	38.1	36
763	**4**{*46* ,*40* ,*13* }	COC1=CC=C(CNC2=C(N=C3C=CC=CN23)C2=NC(CO)=CS2)C=C1 |c:12,14,23,27,t:2,4,8,10,19|	Z8873685476	**1**{*46* }	Nc1ccccn1	**2**{*40* }	OCc1csc(C=O)n1	**3**{*13* }	COc1ccc(C[N+]#[C-])cc1	35.4	36
764	**4**{*99* ,*286* ,*34* }	NS(=O)(=O)C1=CN2C(C=C1)=NC(=C2NCC1CCCO1)C1=NC=CC(=C1)C(F)(F)F |c:8,10,12,25,27,t:4,23|	Z8878918636	**1**{*99* }	Nc1ccc(cn1)S(=O)(=O)N	**2**{*286* }	FC(F)(F)c1ccnc(C=O)c1	**3**{*34* }	[C-]#[N+]CC1CCCO1	42.7	36
765	**4**{*386* ,*390* ,*5* }	COCCCNC1=C(N=C2C=C(C)C=CN12)C1CC2C(C1)C2(F)F |c:13,t:6,8,10|	Z8878918859	**1**{*386* }	Cc1ccnc(N)c1	**2**{*390* }	FC1(F)C2CC(CC21)C=O	**3**{*5* }	COCCC[N+]#[C-]	32.4	36
766	**4**{*109* ,*391* ,*29* }	CCOC(=O)CCNC1=C(CCC(C)(C)C(=O)OCC)N=C2C=CC=C(N12)S(N)(=O)=O |c:8,22,24,t:20|	Z8878918665	**1**{*109* }	Cl.Nc1cccc(n1)S(=O)(=O)N	**2**{*391* }	CCOC(=O)C(C)(C)CCC=O	**3**{*29* }	CCOC(=O)CC[N+]#[C-]	43.9	36
767	**4**{*193* ,*328* ,*10* }	CCC1=NNC=C1C1=C(NC2CCOC2)N2C=C(C=CC2=N1)C(=O)NC |c:5,8,18,20,23,t:2|	Z8873684878	**1**{*193* }	CNC(=O)c1ccc(N)nc1	**2**{*328* }	Cl.CCc1n[nH]cc1C=O	**3**{*10* }	[C-]#[N+]C1CCOC1	34.2	36
768	**4**{*371* ,*365* ,*12* }	N#CCCCOC1=CC=CN2C(NCC3CCOC3)=C(N=C12)C1=NNC2=C1C=NC=C2 |c:8,19,28,31,33,t:6,21,25|	Z8837933140	**1**{*371* }	Nc1ncccc1OCCCC#N	**2**{*365* }	O=Cc1n[nH]c2ccncc12	**3**{*12* }	[C-]#[N+]CC1CCOC1	40.0	35
769	**4**{*387* ,*375* ,*46* }	COCC1=C(N=CO1)C1=C(NCC(C)=C)N2C=CC=C(OC(F)F)C2=N1 |c:5,9,17,26,t:3,19|	Z8873684838	**1**{*387* }	Nc1ncccc1OC(F)F	**2**{*375* }	COCc1ocnc1C=O	**3**{*46* }	CC(=C)C[N+]#[C-]	34.9	35
770	**4**{*146* ,*392* ,*46* }	CC(=C)CNC1=C(N=C2C=CC(=CN12)S(C)(=O)=O)C1CCOCC1 |c:9,11,t:5,7|	Z8878918503	**1**{*146* }	CS(=O)(=O)c1ccc(N)nc1	**2**{*392* }	O=CC1CCOCC1	**3**{*46* }	CC(=C)C[N+]#[C-]	32.9	35

771	**4**{*32* ,*393* ,*18* }	CC(C)(C)NC1=C(N=C2C=C(C=CN12)P(C)(C)=O)C1=CC(Br)=C(F)C=C1 |c:9,11,26,t:5,7,20,23|	Z8878918310	**1**{*32* }	CP(=O)(C)c1ccnc(N)c1	**2**{*393* }	Fc1ccc(C=O)cc1Br	**3**{*18* }	CC(C)(C)[N+]#[C-]	40.8	34
772	**4**{*327* ,*90* ,*65* }	COC(=O)C1=C(F)C2=NC(=C(NC3CCCC3)N2C=C1)C1=COC(C)=N1 |c:4,20,27,t:7,9,23|	Z8855739109	**1**{*327* }	COC(=O)c1ccnc(N)c1F	**2**{*90* }	Cc1nc(C=O)co1	**3**{*65* }	[C-]#[N+]C1CCCC1	33.0	34
773	**4**{*380* ,*312* ,*5* }	COCCCNC1=C(N=C2N1C=C(Cl)N=C2C)C1CCOC1(C)C |c:8,15,t:6,12|	Z8873685390	**1**{*380* }	Cc1nc(Cl)cnc1N	**2**{*312* }	CC1(C)OCCC1C=O	**3**{*5* }	COCCC[N+]#[C-]	32.4	34
774	**4**{*142* ,*394* ,*24* }	COC1=CC(CNC2=C(N=C3N2C=CC=C3OCCS(C)(=O)=O)C2=NOC=C2C)=CC=C1 |c:9,13,15,28,31,33,t:2,7,25|	Z8878918913	**1**{*142* }	CS(=O)(=O)CCOc1cccnc1N	**2**{*394* }	Cc1conc1C=O	**3**{*24* }	COc1cccc(C[N+]#[C-])c1	41.8	34
775	**4**{*348* ,*258* ,*44* }	CC(C)(C)OC(=O)NCCCCNC1=C(N=C2C=NC(F)=CN12)C1=NN=C(S1)C1CC1 |c:17,20,27,t:13,15,25|	Z8873684733	**1**{*348* }	Nc1cnc(F)cn1	**2**{*258* }	O=Cc1nnc(s1)C2CC2	**3**{*44* }	CC(C)(C)OC(=O)NCCCC[N+]#[C-]	40.9	34
776	**4**{*146* ,*395* ,*10* }	CC(C)(C)OC(=O)N[C@H]1C[C@@H](C1)C1=C(NC2CCOC2)N2C=C(C=CC2=N1)S(C)(=O)=O |r,c:13,23,25,28|	Z8873684865	**1**{*146* }	CS(=O)(=O)c1ccc(N)nc1	**2**{*395* }	CC(C)(C)OC(=O)N[C@H]1C[C@@H](C1)C=O	**3**{*10* }	[C-]#[N+]C1CCOC1	41.1	34
777	**4**{*41* ,*396* ,*13* }	COC1=CC=C(CNC2=C(N=C3C=NC(C)=CN23)C2=CN=C3COCCN23)C=C1 |c:12,15,31,t:2,4,8,10,20,22|	Z8873684595	**1**{*41* }	Cc1cnc(N)cn1	**2**{*396* }	O=Cc1cnc2COCCn12	**3**{*13* }	COc1ccc(C[N+]#[C-])cc1	35.6	34
778	**4**{*356* ,*397* ,*1* }	COC1=C(C2=C(NC3CC3)N3C(C=CC(Cl)=C3C(=O)N(C)C)=N2)C(C)=CC(C)=N1 |c:2,4,13,16,23,27,30|	Z8846492036	**1**{*356* }	CN(C)C(=O)c1nc(N)ccc1Cl	**2**{*397* }	COc1nc(C)cc(C)c1C=O	**3**{*1* }	[C-]#[N+]C1CC1	37.4	33
779	**4**{*181* ,*358* ,*14* }	COCCNC1=C(N=C2N1C=CC1=C2N=CC=N1)C1CC11CCN(C1)C(=O)OC(C)(C)C |c:7,11,13,16,18,t:5|	Z8878918434	**1**{*181* }	Nc1nccc2nccnc12	**2**{*358* }	CC(C)(C)OC(=O)N1CCC2(CC2C=O)C1	**3**{*14* }	COCC[N+]#[C-]	39.6	33
780	**4**{*286* ,*383* ,*23* }	COC(=O)CCC(NC1=C(N=C2C=CC(=CN12)C1=NC=CC=C1)C1=NC=C(C)C=C1)C(=O)OC |c:12,14,21,23,31,t:8,10,19,26,28|	Z8855739252	**1**{*286* }	Cl.Cl.Nc1ccc(cn1)c2ccccn2	**2**{*383* }	Cc1ccc(C=O)nc1	**3**{*23* }	COC(=O)CCC([N+]#[C-])C(=O)OC	41.5	33
781	**4**{*214* ,*32* ,*10* }	COC1=CC(Br)=C(C)N2C(NC3CCOC3)=C(N=C12)[C@@H]1C[C@H]1C1=CC=NN1C |&1:19,21,r,c:16,28,t:2,5,18,26|	Z8873684886	**1**{*214* }	COc1cc(Br)c(C)nc1N	**2**{*32* }	Cn1nccc1[C@@H]2C[C@H]2C=O |&1:6,8,r|	**3**{*10* }	[C-]#[N+]C1CCOC1	40.3	33

782	**4**{*81*,*19*,*9*}	CNC1=C(N=C2C=NC=CN12)C1=C(OC)C=NN1C |c:6,8,13,17,t:2,4|	Z8855619682	**1**{*81*}	Nc1cnccn1	**2**{*19*}	COc1cnn(C)c1C=O	**3**{9}	C[N+]#[C-]	23.3	33
783	**4**{*78*,*398*,*28*}	CCCNC1=C(N=C2C=C(C)C3=NC(C)=CN3N12)C1=CC2=C(CCCC2)O1 |c:14,t:4,6,8,11,21,23|	Z8878918628	**1**{*78*}	Cc1cn2nc(N)cc(C)c2n1	**2**{*398*}	O=Cc1cc2CCCCc2o1	**3**{*28*}	CCC[N+]#[C-]	32.7	33
784	**4**{*286*,*235*,*23*}	COC(=O)CCC(NC1=C(N=C2C=CC(=CN12)C1=NC=CC=C1)C1=CSC(=C1)C#N)C(=O)OC |c:12,14,21,23,29,t:8,10,19,26|	Z8808559878	**1**{*286*}	Cl.Cl.Nc1ccc(cn1)c2ccccn2	**2**{*235*}	O=Cc1csc(C#N)c1	**3**{*23*}	COC(=O)CCC([N+]#[C-])C(=O)OC	42.3	33
785	**4**{*361*,*399*,*12*}	CC(C)OC1=CC=CN2C(NCC3CCOC3)=C(N=C12)C1=CN(N=C1C)C1CC1 |c:6,17,26,t:4,19,23|	Z8878918330	**1**{*361*}	Cl.CC(C)Oc1cccnc1N	**2**{*399*}	Cc1nn(cc1C=O)C2CC2	**3**{*12*}	[C-]#[N+]CC1COC1	35.0	33
786	**4**{*118*,*400*,*35*}	CSCCCNC1=C(N=C2C=CC=C(C(C)O)N12)C1=CN(N=N1)C1CCCC1 |c:10,23,t:6,8,12,20|	Z8873684919	**1**{*118*}	Cl.CC(O)c1cccc(N)n1	**2**{*400*}	O=Cc1cn(nn1)C2CCCC2	**3**{*35*}	CSCCC[N+]#[C-]	35.3	33
787	**4**{*111*,*206*,*22*}	COC(=O)C1=CN2C(NC3COC3)=C(C[C@H](C)NC(=O)OC(C)(C)C)N=C2C(C)=C1 |c:26,30,t:4,13|	Z8873684805	**1**{*111*}	COC(=O)c1cnc(N)c(C)c1	**2**{*206*}	C[C@@H](CC=O)NC(=O)OC(C)(C)C	**3**{*22*}	[C-]#[N+]C1COC1	36.7	33
788	**4**{*79*,*106*,*13*}	COC1=CC=C(CNC2=C(N=C3C=NC=C(C)N23)C2=C(OC)N=CC=N2)C=C1 |c:12,20,24,26,29,t:2,4,8,10,14|	Z8835022911	**1**{*79*}	Cc1cncc(N)n1	**2**{*106*}	COc1nccnc1C=O	**3**{*13*}	COc1ccc(C[N+]#[C-])cc1	32.8	32
789	**4**{*187*,*401*,*12*}	CN1N=C(Br)C(=N1)C1=C(NCC2CCOC2)N2C=C(Cl)C(=CC2=N1)C#N |c:5,8,22,25,t:2,19|	Z8873684795	**1**{*187*}	Nc1cc(C#N)c(Cl)cn1	**2**{*401*}	Cn1nc(Br)c(C=O)n1	**3**{*12*}	[C-]#[N+]CC1COC1	37.8	32
790	**4**{*350*,*56*,*23*}	COC(=O)CCC(NC1=C(N=C2C=NC(=CN12)C1=CC=CC=C1)C1=C(C)C=NC(F)=C1)C(=O)OC |c:12,14,21,23,26,29,32,t:8,10,19|	Z8849597821	**1**{*350*}	Nc1cnc(cn1)c2ccccc2	**2**{*56*}	Cc1cnc(F)cc1C=O	**3**{*23*}	COC(=O)CCC([N+]#[C-])C(=O)OC	40.7	32
791	**4**{*73*,*402*,*13*}	COC1=CC=C(CNC2=C(N=C3C=CC4=NC=CN4N23)C2=C(F)C(C)=CC=C2)C=C1 |c:12,16,23,27,29,32,t:2,4,8,10,14|	Z8878918874	**1**{*73*}	Nc1ccc2nccn2n1	**2**{*402*}	Cc1cccc(C=O)c1F	**3**{*13*}	COc1ccc(C[N+]#[C-])cc1	33.9	31
792	**4**{*127*,*403*,*29*}	CCOC(=O)CCNC1=C(N=C2N1C=CC=C2F)C1=CN=C(Cl)N1 |c:10,14,16,t:8,20,22|	Z8873684670	**1**{*127*}	Nc1ncccc1F	**2**{*403*}	Clc1ncc(C=O)[nH]1	**3**{*29*}	CCOC(=O)CC[N+]#[C-]	29.6	31
793	**4**{*388*,*404*,*67*}	CCC(C)NC1=C(N=C2C=C3CCCOC3=CN12)C1=NN=C(C)N1 |c:16,t:5,7,9,21,23|	Z8873685425	**1**{*388*}	Cl.Nc1cc2CCCOc2cn1	**2**{*404*}	Cc1nnc(C=O)[nH]1	**3**{*67*}	CCC(C)[N+]#[C-]	27.2	31

794	**4**{*81*,*157*,*9*}	CNC1=C(N=C2C=NC=CN12)C1=NC2=C(C=C1)N=CC=C2 |c:6,8,15,17,20,22,t:2,4,13|	Z8835022913	**1**{*81*}	Nc1cnccn1	**2**{*157*}	O=Cc1ccc2ncccc2n1	**3**{*9*}	C[N+]#[C-]	22.9	31
795	**4**{*81*,*396*,*13*}	COC1=CC=C(CNC2=C(N=C3C=NC=CN23)C2=CN=C3COCCN23)C=C1 |c:12,14,30,t:2,4,8,10,19,21|	Z8878918856	**1**{*81*}	Nc1cnccn1	**2**{*396*}	O=Cc1cnc2COCCn12	**3**{*13*}	COc1ccc(C[N+]#[C-])cc1	30.9	30
796	**4**{*187*,*405*,*12*}	CC(F)(F)C1=NC=C(S1)C1=C(NCC2CCOC2)N2C=C(Cl)C(=CC2=N1)C#N |c:6,10,24,27,t:4,21|	Z8878918560	**1**{*187*}	Nc1cc(C#N)c(Cl)cn1	**2**{*405*}	CC(F)(F)c1ncc(C=O)s1	**3**{*12*}	[C-]#[N+]CC1CCOC1	34.6	30
797	**4**{*186*,*406*,*8*}	COC(=O)C(CC1=CC=CC=C1)NC1=C(N=C2C=C(C=CN12)S(=O)(=O)N(C)C)C1=CC=NS1 |c:8,10,18,20,33,t:6,14,16,31|	Z8873684677	**1**{*186*}	Cl.CN(C)S(=O)(=O)c1ccnc(N)c1	**2**{*406*}	O=Cc1ccns1	**3**{*8*}	COC(=O)C(Cc1ccccc1)[N+]#[C-]	39.2	30
798	**4**{*68*,*371*,*23*}	COC(=O)CCC(NC1=C(N=C2C=C(C=CN12)C#C)C1=C(C)C(OC)=CC=N1)C(=O)OC |c:12,14,21,26,28,t:8,10|	Z8878918811	**1**{*68*}	Nc1cc(C#C)ccn1	**2**{*371*}	COc1ccnc(C=O)c1C	**3**{*23*}	COC(=O)CCC([N+]#[C-])C(=O)OC	35.2	30
799	**4**{*348*,*407*,*2*}	CCC1=NN=C(N1)C1=C(NC2=CC=C(F)C=C2)N2C=C(F)N=CC2=N1 |c:4,8,16,23,26,t:2,11,13,20|	Z8873684930	**1**{*348*}	Nc1cnc(F)cn1	**2**{*407*}	CCc1nnc(C=O)[nH]1	**3**{*2*}	Fc1ccc([N+]#[C-])cc1	27.3	30
800	**4**{*96*,*408*,*60*}	COCCN(C)CC1=CC2=NC(CCN3CCOC3=O)=C(NC3=CC(=CC=C3)C(=O)OC)N2C=C1 |c:25,27,36,t:7,9,20,23|	Z8849597825	**1**{*96*}	COCCN(C)Cc1ccnc(N)c1	**2**{*408*}	O=CCCN1CCOC1=O	**3**{*60*}	COC(=O)c1cccc([N+]#[C-])c1	38.4	30
801	**4**{*359*,*409*,*22*}	CC(C)(C)OC(=O)NC1=NC=C(N=C1)C1=C(NC2COC2)N2C=C(OC(F)F)C=CC2=N1 |c:10,12,15,30,33,t:8,24|	Z8854581143	**1**{*359*}	Nc1ccc(OC(F)F)cn1	**2**{*409*}	CC(C)(C)OC(=O)Nc1cnc(C=O)cn1	**3**{*22*}	[C-]#[N+]C1COC1	35.7	29
802	**4**{*61*,*320*,*23*}	CCC1=CC=CC2=NC(=C(NC(CCC(=O)OC)C(=O)OC)N12)C1=NN(C)C=N1 |c:4,29,t:2,6,8,25|	Z8873685304	**1**{*61*}	CCc1cccc(N)n1	**2**{*320*}	Cn1cnc(C=O)n1	**3**{*23*}	COC(=O)CCC([N+]#[C-])C(=O)OC	31.7	29
803	**4**{*188*,*410*,*67*}	CCC(C)NC1=C(N=C2C=NC=C(N12)C(=O)OC)C1=CSC(=N1)C1=NC=CC=N1 |c:9,11,23,28,30,t:5,7,20,26|	Z8873685325	**1**{*188*}	COC(=O)c1cncc(N)n1	**2**{*410*}	O=Cc1csc(n1)c2ncccn2	**3**{*67*}	CCC(C)[N+]#[C-]	32.4	29
804	**4**{*70*,*92*,*31*}	CSCCNC1=C(N=C2N1C=CN=C2C1CC1)C1=CC(C)=NO1 |c:7,11,13,23,t:5,20|	Z8873684624	**1**{*70*}	Nc1nccnc1C2CC2	**2**{*92*}	Cc1cc(C=O)on1	**3**{*31*}	CSCC[N+]#[C-]	26.0	29
805	**4**{*128*,*320*,*29*}	CCOC(=O)CCNC1=C(N=C2C=CC(=CN12)N(C)CCOC)C1=NN(C)C=N1 |c:12,14,29,t:8,10,25|	Z8878918844	**1**{*128*}	COCCN(C)c1ccc(N)nc1	**2**{*320*}	Cn1cnc(C=O)n1	**3**{*29*}	CCOC(=O)CC[N+]#[C-]	31.5	29
806	**4**{*83*,*411*,*7*}	CP(C)(=O)C1=CN2C(C=C1)=NC(=C2NC1CCOCC1)C1=CC2=C(CCC2)N=C1 |c:8,10,12,31,t:4,23,25|	Z8835022873	**1**{*83*}	CP(=O)(C)c1ccc(N)nc1	**2**{*411*}	O=Cc1cnc2CCCc2c1	**3**{*7*}	[C-]#[N+]C1CCOCC1	31.6	29

807	**4**{*239*,*223*,*37*}	COC(=O)C1CC(C1)NC1=C(N=C2C=CC(=CN12)N1CCCC1)C1=CN=C(C=C1)C(=O)OC |c:14,16,29,31,t:10,12,27|	Z8855619681	**1**{*239*}	Cl.Cl.Nc1ccc(cn1)N2CCCC2	**2**{*223*}	Cl.COC(=O)c1ccc(C=O)cn1	**3**{*37*}	COC(=O)C1CC(C1)[N+]#[C-]	34.6	28
808	**4**{*83*,*412*,*31*}	CSCCNC1=C(N=C2C=CC(=CN12)P(C)(C)=O)C1=CC(SC)=CC=C1 |c:9,11,24,26,t:5,7,20|	Z8878918765	**1**{*83*}	CP(=O)(C)c1ccc(N)nc1	**2**{*412*}	CSc1cccc(C=O)c1	**3**{*31*}	CSCC[N+]#[C-]	30.7	28
809	**4**{*311*,*413*,*12*}	FC1=CC2=NC(C3=CN=C(S3)C3=CC=NC=C3)=C(NCC3CCOC3)N2C=C1 |c:8,14,16,30,t:1,3,6,12,18|	Z8849597805	**1**{*311*}	Nc1cc(F)ccn1	**2**{*413*}	O=Cc1cnc(s1)c2ccncc2	**3**{*12*}	[C-]#[N+]CC1CCOC1	29.9	28
810	**4**{*32*,*5*,*31*}	CSCCNC1=C(N=C2C=C(C=CN12)P(C)(C)=O)C1=C(C)C=NC=C1C |c:9,11,20,23,25,t:5,7|	Z8878918836	**1**{*32*}	CP(=O)(C)c1ccnc(N)c1	**2**{*5*}	Cc1cncc(C)c1C=O	**3**{*31*}	CSCC[N+]#[C-]	29.2	28
811	**4**{*154*,*414*,*49*}	CCC1=C(N=CC=C1)C1=C(NC2CC(F)(F)C2)N2C=CC(=CC2=N1)[N+]([O-])=O |c:4,6,9,20,22,25,t:2|	Z8873685281	**1**{*154*}	Nc1cc(ccn1)[N+](=O)[O-]	**2**{*414*}	CCc1cccnc1C=O	**3**{*49*}	FC1(F)CC(C1)[N+]#[C-]	27.8	28
812	**4**{*348*,*415*,*67*}	CCC(C)NC1=C(N=C2C=NC(F)=CN12)C1=NC=NN1CC |c:9,12,19,t:5,7,17|	Z8873685461	**1**{*348*}	Nc1cnc(F)cn1	**2**{*415*}	CCn1ncnc1C=O	**3**{*67*}	CCC(C)[N+]#[C-]	22.5	27
813	**4**{*28*,*416*,*7*}	COCC1=CC(F)=C(C2=C(NC3CCOCC3)N3C=CC(=CC3=N2)C2=NOC=N2)C(F)=C1 |c:8,19,21,24,30,34,t:3,6,27|	Z8873685350	**1**{*28*}	Cl.Nc1cc(ccn1)c2ncon2	**2**{*416*}	COCc1cc(F)c(C=O)c(F)c1	**3**{*7*}	[C-]#[N+]C1CCOCC1	32.7	27
814	**4**{*103*,*293*,*40*}	CCOC(=O)C1=CC=C(NC2=C(N=C3C=NC=C(CO)N23)C2=C(F)N(C)N=C2)C=C1 |c:14,23,28,31,t:5,7,10,12,16|	Z8878918658	**1**{*103*}	Nc1cncc(CO)n1	**2**{*293*}	Cn1ncc(C=O)c1F	**3**{*40*}	CCOC(=O)c1ccc([N+]#[C-])cc1	30.4	27
815	**4**{*85*,*417*,*31*}	CSCCNC1=C(N=C2C=C(C=C(C)N12)C#N)C1=C2N=NN(C)C2=CC=C1 |c:9,19,21,26,28,t:5,7,11|	Z8873684768	**1**{*85*}	Cc1cc(C#N)cc(N)n1	**2**{*417*}	Cn1nnc2c(C=O)cccc12	**3**{*31*}	CSCC[N+]#[C-]	27.4	27
816	**4**{*50*,*418*,*15*}	CCNC1=C(N=C2C=CC=C(N12)P(C)(C)=O)C1=NC2=C(C=CC=C2Cl)C=C1 |c:7,9,22,24,28,t:3,5,18,20|	Z8846492148	**1**{*50*}	CP(=O)(C)c1cccc(N)n1	**2**{*418*}	Clc1cccc2ccc(C=O)nc12	**3**{*15*}	CC[N+]#[C-]	28.9	27
817	**4**{*72*,*375*,*24*}	COCC1=C(N=CO1)C1=C(NCC2=CC(OC)=CC=C2)N2C=C(Br)N=C(OC)C2=N1 |c:5,9,17,19,31,t:3,13,23,26|	Z8855619691	**1**{*72*}	COc1nc(Br)cnc1N	**2**{*375*}	COCc1ocnc1C=O	**3**{*24*}	COc1cccc(C[N+]#[C-])c1	34.0	27
818	**4**{*185*,*419*,*8*}	COC(=O)C(CC1=CC=CC=C1)NC1=C(N=C2C=CC(=C(C)N12)S(C)(=O)=O)C1=NC=CC(OC)=C1 |c:8,10,18,32,36,t:6,14,16,20,30|	Z8878918452	**1**{*185*}	Cc1nc(N)ccc1S(=O)(=O)C	**2**{*419*}	COc1ccnc(C=O)c1	**3**{*8*}	COC(=O)C(Cc1ccccc1)[N+]#[C-]	35.4	27
819	**4**{*81*,*420*,*9*}	CNC1=C(CC(C)(C)C#N)N=C2C=NC=CN12 |c:2,12,14,t:10|	Z8878918348	**1**{*81*}	Nc1cnccn1	**2**{*420*}	CC(C)(CC=O)C#N	**3**{*9*}	C[N+]#[C-]	16.1	26

820	**4**{*41*,*421*,*9*}	CNC1=C(N=C2C=NC(C)=CN12)C1=CN=C(S1)C1=CC=CO1 |c:6,9,16,22,t:2,4,14,20|	Z8873685445	**1**{*41*}	Cc1cnc(N)cn1	**2**{*421*}	O=Cc1cnc(s1)c2ccco2	**3**{*9*}	C[N+]#[C-]	21.8	26
821	**4**{*176*,*377*,*14*}	COCCNC1=C(N=C2N1C=CN=C2N1CCCC1)C1=NNC=C1C |c:7,11,13,25,t:5,22|	Z8878918326	**1**{*176*}	Nc1nccnc1N2CCCC2	**2**{*377*}	Cl.Cc1c[nH]nc1C=O	**3**{*14*}	COCC[N+]#[C-]	23.8	26
822	**4**{*85*,*422*,*35*}	CSCCCNC1=C(N=C2C=C(C=C(C)N12)C#N)C1CC11CCOC1 |c:10,t:6,8,12|	Z8855739119	**1**{*85*}	Cc1cc(C#N)cc(N)n1	**2**{*422*}	O=CC1CC21CCOC2	**3**{*35*}	CSCCC[N+]#[C-]	24.8	26
823	**4**{*28*,*92*,*18*}	CC1=NOC(=C1)C1=C(NC(C)(C)C)N2C=CC(=CC2=N1)C1=NOC=N1 |c:4,7,15,17,20,26,t:1,23|	Z8878918344	**1**{*28*}	Cl.Nc1cc(ccn1)c2ncon2	**2**{*92*}	Cc1cc(C=O)on1	**3**{*18*}	CC(C)(C)[N+]#[C-]	23.2	25
824	**4**{*362*,*423*,*10*}	CNC(=O)C1=CC=CC2=NC(=C(NC3CCOC3)N12)C1=CC2=C(C=CN2C)C=C1 |c:6,26,31,t:4,8,10,22,24|	Z8855619560	**1**{*362*}	CNC(=O)c1cccc(N)n1	**2**{*423*}	Cn1ccc2ccc(C=O)cc12	**3**{*10*}	[C-]#[N+]C1CCOC1	26.6	25
825	**4**{*356*,*353*,*1*}	CN(C)C(=O)C1=C(Cl)C=CC2=NC(=C(NC3CC3)N12)C1=C(CF)C=CC=C1 |c:5,8,22,26,28,t:10,12|	Z8846492014	**1**{*356*}	CN(C)C(=O)c1nc(N)ccc1Cl	**2**{*353*}	FCc1ccccc1C=O	**3**{*1*}	[C-]#[N+]C1CC1	26.1	25
826	**4**{*389*,*424*,*5*}	COCCCNC1=C(N=C2N1C=C(C)C=C2C)C1=NN(C)C(=N1)C(F)(F)F |c:8,15,23,t:6,12,19|	Z8873684934	**1**{*389*}	Cc1cnc(N)c(C)c1	**2**{*424*}	Cn1nc(C=O)nc1C(F)(F)F	**3**{*5*}	COCCC[N+]#[C-]	25.8	25
827	**4**{*141*,*209*,*35*}	CSCCCNC1=C(N=C2C=C(NC(C)=O)C=CN12)C1=NN=C2CCCCN12 |c:16,t:6,8,10,21,23|	Z8878918331	**1**{*141*}	CC(=O)Nc1ccnc(N)c1	**2**{*209*}	O=Cc1nnc2CCCCn12	**3**{*35*}	CSCCC[N+]#[C-]	26.8	25
828	**4**{*390*,*191*,*31*}	CCN1C=NN=C1C1=C(NCCSC)N2C(C=CC(C)=C2Cl)=N1 |c:3,5,8,17,21,24|	Z8878918633	**1**{*390*}	CCc1ccc(N)nc1Cl	**2**{*191*}	CCn1cnnc1C=O	**3**{*31*}	CSCC[N+]#[C-]	24.2	25
829	**4**{*391*,*425*,*49*}	CS(=O)(=O)N1CCCC(C1)C1=C(NC2CC(F)(F)C2)N2N=C(C=CC2=N1)C1=C(F)C=CC=C1 |c:11,22,24,27,30,33,35|	Z8873684675	**1**{*391*}	Nc1ccc(nn1)c2ccccc2F	**2**{*425*}	CS(=O)(=O)N1CCCC(C1)C=O	**3**{*49*}	FC1(F)CC(C1)[N+]#[C-]	31.5	24
830	**4**{*392*,*258*,*67*}	CCC(C)NC1=C(N=C2C=CC3=C(NC(C)=N3)N12)C1=NN=C(S1)C1CC1 |c:9,15,23,t:5,7,11,21|	Z8878918394	**1**{*392*}	Cl.Cc1nc2ccc(N)nc2[nH]1	**2**{*258*}	O=Cc1nnc(s1)C2CC2	**3**{*67*}	CCC(C)[N+]#[C-]	24.1	24
831	**4**{*123*,*426*,*22*}	CCOC(=O)COC1=CC=C(C=C1)C1=C(NC2COC2)N2C=CC(OCCO)=CC2=N1 |c:9,11,14,23,29,32,t:7|	Z8878918409	**1**{*123*}	Cl.Nc1cc(OCCO)ccn1	**2**{*426*}	CCOC(=O)COc1ccc(C=O)cc1	**3**{*22*}	[C-]#[N+]C1COC1	28.4	24
832	**4**{*46*,*112*,*9*}	CNC1=C(N=C2C=CC=CN12)C1=C2C=CC=NN2N=C1 |c:6,8,13,15,17,21,t:2,4|	Z8873684790	**1**{*46*}	Nc1ccccn1	**2**{*112*}	O=Cc1cnn2ncccc12	**3**{*9*}	C[N+]#[C-]	17.3	24
833	**4**{*142*,*427*,*24*}	COC1=CC(CNC2=C(N=C3N2C=CC=C3OCCS(C)(=O)=O)C2=NOC3=C2COCC3)=CC=C1 |c:9,13,15,28,35,37,t:2,7,25|	Z8873685374	**1**{*142*}	CS(=O)(=O)CCOc1cccnc1N	**2**{*427*}	O=Cc1noc2CCOCc12	**3**{*24*}	COc1cccc(C[N+]#[C-])c1	32.5	24

834	**4**{*54*,*130*,*53*}	COC1=NN=C(C=C1)C1=C(NCCOCC2=CC=CC=C2)N2C=C(C=CC2=N1)C(=O)N(C)C |c:4,6,9,18,20,24,26,29,t:2,16|	Z8849597837	**1**{*54*}	Cl.CN(C)C(=O)c1ccc(N)nc1	**2**{*130*}	COc1ccc(C=O)nn1	**3**{*53*}	[C-]#[N+]CCOCc1ccccc1	29.1	24
835	**4**{*78*,*428*,*28*}	CCCNC1=C(N=C2C=C(C)C3=NC(C)=CN3N12)C1=CC(OCC2CC2)=C(OC)C=C1 |c:14,33,t:4,6,8,11,21,29|	Z8873684682	**1**{*78*}	Cc1cn2nc(N)cc(C)c2n1	**2**{*428*}	COc1ccc(C=O)cc1OCC2CC2	**3**{*28*}	CCC[N+]#[C-]	27.3	24
836	**4**{*59*,*279*,*42*}	CN1C=CN=C1[C@@H]1OCCC[C@H]1C1=C(NC2=C(C)C=CC=C2)N2C=C(CN3CCOCC3)C=CC2=N1 |&1:6,11,r,c:2,4,14,17,20,22,36,39,t:26|	Z8878918314	**1**{*59*}	Nc1ccc(CN2CCOCC2)cn1	**2**{*279*}	Cn1ccnc1[C@@H]2OCCC[C@H]2C=O |&1:6,11,r|	**3**{*42*}	Cc1ccccc1[N+]#[C-]	31.5	24
837	**4**{*291*,*30*,*9*}	CNC1=C(N=C2N1C=C(Cl)C=C2Cl)C1CCN(CC1)C(C)=O |c:4,11,t:2,8|	Z8829498779	**1**{*291*}	Nc1ncc(Cl)cc1Cl	**2**{*30*}	CC(=O)N1CCC(CC1)C=O	**3**{*9*}	C[N+]#[C-]	21.8	24
838	**4**{*158*,*429*,*31*}	CSCCNC1=C(N=C2C=NC(=CN12)P(C)(C)=O)C1=C(CCCC1)SC1=CC=CC=C1 |c:9,11,30,32,t:5,7,20,28|	Z8835022878	**1**{*158*}	CP(=O)(C)c1cnc(N)cn1	**2**{*429*}	O=CC1=C(CCCC1)Sc2ccccc2	**3**{*31*}	CSCC[N+]#[C-]	30.1	24
839	**4**{*73*,*430*,*13*}	COC1=CC=C(CNC2=C(N=C3C=CC4=NC=CN4N23)C2=C(C)N=C(OC)C=C2)C=C1 |c:12,16,23,30,33,t:2,4,8,10,14,26|	Z8873685436	**1**{*73*}	Nc1ccc2nccn2n1	**2**{*430*}	COc1ccc(C=O)c(C)n1	**3**{*13*}	COc1ccc(C[N+]#[C-])cc1	26.4	24
840	**4**{*172*,*110*,*29*}	CCOC(=O)CCNC1=C(N=C2N1C=CC1=C2CCO1)C1=CN=C(C=C1)P(C)(C)=O |c:10,14,16,25,27,t:8,23|	Z8873684941	**1**{*172*}	Nc1nccc2OCCc21	**2**{*110*}	CP(=O)(C)c1ccc(C=O)cn1	**3**{*29*}	CCOC(=O)CC[N+]#[C-]	27.3	24
841	**4**{*353*,*431*,*15*}	CCNC1=C(N=C2C=CC(NS(C)(=O)=O)=CN12)C1CC(C1)N(C)C(=O)OC(C)(C)C |c:7,14,t:3,5|	Z8878918906	**1**{*353*}	Cl.CS(=O)(=O)Nc1ccc(N)nc1	**2**{*431*}	CN(C1CC(C1)C=O)C(=O)OC(C)(C)C	**3**{*15*}	CC[N+]#[C-]	27.6	23
842	**4**{*92*,*432*,*7*}	NC(=O)C1=CC2=NC(=C(NC3CCOCC3)N2C=C1)C1=CSC(=N1)C1=CC=C(Cl)C=C1 |c:19,25,33,t:3,5,7,22,28,30|	Z8873685470	**1**{*92*}	NC(=O)c1ccnc(N)c1	**2**{*432*}	Clc1ccc(cc1)c2nc(C=O)cs2	**3**{*7*}	[C-]#[N+]C1CCOCC1	28.4	23
843	**4**{*181*,*433*,*14*}	COCCNC1=C(N=C2N1C=CC1=C2N=CC=N1)C1=CN(N=C1)C1=C(F)C=CC=C1 |c:7,11,13,16,18,24,27,30,32,t:5,21|	Z8878918573	**1**{*181*}	Nc1nccc2nccnc12	**2**{*433*}	Fc1ccccc1n2cc(C=O)cn2	**3**{*14*}	COCC[N+]#[C-]	25.1	23
844	**4**{*109*,*434*,*34*}	COC1=CC(=NC(C)=C1)C1=C(NCC2CCCO2)N2C(C=CC=C2S(N)(=O)=O)=N1 |c:4,7,10,22,24,30,t:2|	Z8873685267	**1**{*109*}	Cl.Nc1cccc(n1)S(=O)(=O)N	**2**{*434*}	COc1cc(C)nc(C=O)c1	**3**{*34*}	[C-]#[N+]CC1CCCO1	25.8	23
845	**4**{*393*,*13*,*5*}	COCCCNC1=C(N=C2C=CC(Cl)=C(Cl)N12)C1=C(C)NN=N1 |c:10,19,23,t:6,8,13|	Z8878918379	**1**{*393*}	Nc1ccc(Cl)c(Cl)n1	**2**{*13*}	Cc1[nH]nnc1C=O	**3**{*5*}	COCCC[N+]#[C-]	21.8	23

846	**4**{*387*,*435*,*12*}	CCOC(=O)C1=NOC(=C1)C1=C(NCC2CCOC2)N2C=CC=C(OC(F)F)C2=N1 |c:8,11,22,31,t:5,24|	Z8878918785	**1**{*387*}	Nc1ncccc1OC(F)F	**2**{*435*}	CCOC(=O)c1cc(C=O)on1	**3**{*12*}	[C-]#[N+]CC1CCOC1	25.6	22
847	**4**{*47*,*436*,*6*}	CCCCC1=NC(=CN1)C1=C(NCCCC(=O)OCC)N2C=CC(=CC2=N1)S(N)(=O)=O |c:6,10,22,24,27,t:4|	Z8854581177	**1**{*47*}	Cl.Nc1cc(ccn1)S(=O)(=O)N	**2**{*436*}	CCCCc1nc(C=O)c[nH]1	**3**{*6*}	CCOC(=O)CCC[N+]#[C-]	27.0	22
848	**4**{*123*,*437*,*15*}	CCNC1=C(N=C2C=C(OCCO)C=CN12)C1=CN(CC)N=C1C |c:13,23,t:3,5,7,18|	Z8878918598	**1**{*123*}	Cl.Nc1cc(OCCO)ccn1	**2**{*437*}	CCn1cc(C=O)c(C)n1	**3**{*15*}	CC[N+]#[C-]	19.6	22
849	**4**{*386*,*438*,*5*}	COCCCNC1=C(N=C2C=C(C)C=CN12)C1=NC(C)=NC(=C1)C(F)F |c:13,21,23,t:6,8,10,18|	Z8873684622	**1**{*386*}	Cc1ccnc(N)c1	**2**{*438*}	Cc1nc(C=O)cc(n1)C(F)F	**3**{*5*}	COCCC[N+]#[C-]	21.4	22
850	**4**{*50*,*439*,*31*}	COC1=C(C=CC(=C1)C1=C(NCCSC)N2C(C=CC=C2P(C)(C)=O)=N1)S(C)(=O)=O |c:4,6,9,18,20,26,t:2|	Z8873684911	**1**{*50*}	CP(=O)(C)c1cccc(N)n1	**2**{*439*}	COc1cc(C=O)ccc1S(=O)(=O)C	**3**{*31*}	CSCC[N+]#[C-]	27.6	22
851	**4**{*48*,*440*,*22*}	CS(=O)(=O)C1=CC2=NC(=C(NC3COC3)N2C=C1)C1=CC=C(C=C1)N1C=C(Br)C=N1 |c:18,23,25,32,t:4,6,8,21,29|	Z8873684765	**1**{*48*}	CS(=O)(=O)c1ccnc(N)c1	**2**{*440*}	Brc1cnn(c1)c2ccc(C=O)cc2	**3**{*22*}	[C-]#[N+]C1COC1	28.9	22
852	**4**{*83*,*441*,*31*}	CCC(C)C(OC)C1=C(NCCSC)N2C=C(C=CC2=N1)P(C)(C)=O |c:7,15,17,20|	Z8837933137	**1**{*83*}	CP(=O)(C)c1ccc(N)nc1	**2**{*441*}	CCC(C)C(OC)C=O	**3**{*31*}	CSCC[N+]#[C-]	22.5	22
853	**4**{*146*,*164*,*51*}	CN1N=C(C2=C1CCOC2)C1=C(NCCNC(=O)OC(C)(C)C)N2C=C(C=CC2=N1)S(C)(=O)=O |c:2,4,12,26,28,31|	Z8855619628	**1**{*146*}	CS(=O)(=O)c1ccc(N)nc1	**2**{*164*}	Cn1nc(C=O)c2COCCc21	**3**{*51*}	CC(C)(C)OC(=O)NCC[N+]#[C-]	28.0	21
854	**4**{*187*,*375*,*46*}	COCC1=C(N=CO1)C1=C(NCC(C)=C)N2C=C(Cl)C(=CC2=N1)C#N |c:5,9,20,23,t:3,17|	Z8873684920	**1**{*187*}	Nc1cc(C#N)c(Cl)cn1	**2**{*375*}	COCc1ocnc1C=O	**3**{*46*}	CC(=C)C[N+]#[C-]	20.0	21
855	**4**{*83*,*442*,*37*}	COC(=O)C1CC(C1)NC1=C(N=C2C=CC(=CN12)P(C)(C)=O)C1=CC2=C(COC2)C=C1 |c:14,16,33,t:10,12,25,27|	Z8846492124	**1**{*83*}	CP(=O)(C)c1ccc(N)nc1	**2**{*442*}	O=Cc1ccc2COCc2c1	**3**{*37*}	COC(=O)C1CC(C1)[N+]#[C-]	24.6	21
856	**4**{*166*,*443*,*22*}	OCCSCC1=CC2=NC(=C(NC3COC3)N2C=C1)C1=C2N=CC=CC2=C(F)C=C1 |c:19,22,24,26,32,t:5,7,9,29|	Z8873685480	**1**{*166*}	Nc1cc(CSCCO)ccn1	**2**{*443*}	Fc1ccc(C=O)c2ncccc12	**3**{*22*}	[C-]#[N+]C1COC1	23.7	21
857	**4**{*181*,*344*,*14*}	COCCNC1=C(N=C2N1C=CC1=C2N=CC=N1)C1=C(C)C=NN1COC |c:7,11,13,16,18,21,24,t:5|	Z8873684762	**1**{*181*}	Nc1nccc2nccnc12	**2**{*344*}	COCn1ncc(C)c1C=O	**3**{*14*}	COCC[N+]#[C-]	20.3	20

858	**4**{*95 ,444 ,35* }	CSCCCNC1=C(N=C2C=CC(=CN12)C(F)F)C1=C(O)C=CN=C1 |c:10,12,20,23,25,t:6,8|	Z8873685420	**1**{*95* }	Nc1ccc(cn1)C(F)F	**2**{*444* }	Oc1ccncc1C=O	**3**{*35* }	CSCCC[N+]#[C-]	20.1	20
859	**4**{*330 ,445 ,29* }	CCOC(=O)CCNC1=C(N=C2C=CC(=CN12)S(=O)(=O)N(C)C)C1=NOC(=C1)C(C)(C)C |c:12,14,28,t:8,10,25|	Z8873685490	**1**{*330* }	CN(C)S(=O)(=O)c1ccc(N)nc1	**2**{*445* }	CC(C)(C)c1cc(C=O)no1	**3**{*29* }	CCOC(=O)CC[N+]#[C-]	25.5	20
860	**4**{*22 ,307 ,13* }	COC1=CC=C(CNC2=C(N=C3C=CC(Cl)=NN23)C2=CN=C(Cl)N2C)C=C1 |c:12,15,28,t:2,4,8,10,20,22|	Z8835022916	**1**{*22* }	Nc1ccc(Cl)nn1	**2**{*307* }	Cn1c(Cl)ncc1C=O	**3**{*13* }	COc1ccc(C[N+]#[C-])cc1	22.0	20
861	**4**{*351 ,446 ,28* }	CCCNC1=C(N=C2C=CC3=C(C(O)CC3)N12)C1=CC(=CC=C1)C(=O)NC |c:8,22,24,t:4,6,10,20|	Z8873684684	**1**{*351* }	Nc1ccc2CCC(O)c2n1	**2**{*446* }	CNC(=O)c1cccc(C=O)c1	**3**{*28* }	CCC[N+]#[C-]	19.8	20
862	**4**{*101 ,260 ,6* }	CCOC(=O)CCCNC1=C(N=C2N1C=C(CO)C=C2Cl)C1=NOC(C)=C1 |c:11,19,27,t:9,15,23|	Z8873685370	**1**{*101* }	Nc1ncc(CO)cc1Cl	**2**{*260* }	Cc1cc(C=O)no1	**3**{*6* }	CCOC(=O)CCC[N+]#[C-]	21.1	20
863	**4**{*181 ,447 ,6* }	CCOC(=O)CCCNC1=C(N=C2N1C=CC1=C2N=CC=N1)C1=CC=C(C=C1)C#CC(C)(C)O |c:11,15,17,20,22,27,29,t:9,25|	Z8873684692	**1**{*181* }	Nc1nccc2nccnc12	**2**{*447* }	CC(C)(O)C#Cc1ccc(C=O)cc1	**3**{*6* }	CCOC(=O)CCC[N+]#[C-]	24.4	20
864	**4**{*28 ,448 ,7* }	COC1=C2OCOC2=CC(=C1)C1=C(NC2CCOCC2)N2C=CC(=CC2=N1)C1=NOC=N1 |c:2,8,10,13,24,26,29,35,t:32|	Z8878918875	**1**{*28* }	Cl.Nc1cc(ccn1)c2ncon2	**2**{*448* }	COc1cc(C=O)cc2OCOc21	**3**{*7* }	[C-]#[N+]C1CCOCC1	23.2	20
865	**4**{*218 ,34 ,61* }	CC(C)(C)OC(=O)NCCCNC1=C(N=C2C=CC(=NN12)C(C)(C)C)C1=CC=C(C=C1)P(C)(C)=O |c:16,18,29,31,t:12,14,27|	Z8803896931	**1**{*218* }	CC(C)(C)c1ccc(N)nn1	**2**{*34* }	CP(=O)(C)c1ccc(C=O)cc1	**3**{*61* }	CC(C)(C)OC(=O)NCCC[N+]#[C-]	26.6	20
866	**4**{*46 ,58 ,9* }	CNC1=C(N=C2C=CC=CN12)C1=NSN=C1 |c:6,8,16,t:2,4,13|	Z8855739152	**1**{*46* }	Nc1ccccn1	**2**{*58* }	O=Cc1cnsn1	**3**{*9* }	C[N+]#[C-]	12.2	19
867	**4**{*155 ,449 ,14* }	COCCNC1=C(N=C2N1C=CC=C2Cl)C1=C(C)C=C(S1)C(N)=O |c:7,11,13,17,20,t:5|	Z8878918329	**1**{*155* }	Nc1ncccc1Cl	**2**{*449* }	Cc1cc(sc1C=O)C(=O)N	**3**{*14* }	COCC[N+]#[C-]	19.1	19
868	**4**{*176 ,450 ,14* }	COCCNC1=C(N=C2N1C=CN=C2N1CCCC1)C1=CC(=CC=C1)S(=O)(=O)N(C)C |c:7,11,13,24,26,t:5,22|	Z8846491730	**1**{*176* }	Nc1nccnc1N2CCCC2	**2**{*450* }	CN(C)S(=O)(=O)c1cccc(C=O)c1	**3**{*14* }	COCC[N+]#[C-]	23.1	19
869	**4**{*213 ,92 ,31* }	COC1=CC=C(F)C2=NC(C3=CC(C)=NO3)=C(NCCSC)N12 |c:13,t:2,4,7,10,16|	Z8873684810	**1**{*213* }	COc1ccc(F)c(N)n1	**2**{*92* }	Cc1cc(C=O)on1	**3**{*31* }	CSCC[N+]#[C-]	17.3	19
870	**4**{*46 ,27 ,9* }	CNC1=C(N=C2C=CC=CN12)C1=NN(C)C(C)=N1 |c:6,8,18,t:2,4,13|	Z8855739086	**1**{*46* }	Nc1ccccn1	**2**{*27* }	Cc1nc(C=O)nn1C	**3**{*9* }	C[N+]#[C-]	12.4	19
871	**4**{*330 ,229 ,69* }	COC1=C(NC2=C(N=C3C=CC(=CN23)S(=O)(=O)N(C)C)C2=CN=C(OCCO)C=C2)C=CC=C1 |c:2,9,11,30,33,35,t:5,7,22,24|	Z8878918769	**1**{*330* }	CN(C)S(=O)(=O)c1ccc(N)nc1	**2**{*229* }	OCCOc1ccc(C=O)cn1	**3**{*69* }	COc1ccccc1[N+]#[C-]	24.3	19

872	**4**{*327*,*451*,*34*}	COC(=O)C1=C(F)C2=NC(=C(NCC3CCCO3)N2C=C1)C1=NC=CC(=C1)C(F)F |c:4,21,26,28,t:7,9,24|	Z8878918780	**1**{*327*}	COC(=O)c1ccnc(N)c1F	**2**{*451*}	FC(F)c1ccnc(C=O)c1	**3**{*34*}	[C-]#[N+]CC1CCCO1	20.8	18
873	**4**{*218*,*334*,*21*}	CC1=C(N=CO1)C1=C(NCC2CC2)N2N=C(C=CC2=N1)C(C)(C)C |c:3,7,16,18,21,t:1|	Z8837933146	**1**{*218*}	CC(C)(C)c1ccc(N)nn1	**2**{*334*}	Cc1ocnc1C=O	**3**{*21*}	[C-]#[N+]CC1CC1	16.0	18
874	**4**{*331*,*452*,*49*}	CN(C)CCN(C)CC1=CN2C(C=C1)=NC(=C2NC1CC(F)(F)C1)C1=CC=C(C=C1)C(N)=O |c:12,14,16,29,31,t:8,27|	Z8878918445	**1**{*331*}	Cl.Cl.Cl.CN(C)CCN(C)Cc1ccc(N)nc1	**2**{*452*}	NC(=O)c1ccc(C=O)cc1	**3**{*49*}	FC1(F)CC(C1)[N+]#[C-]	22.4	18
875	**4**{*81*,*453*,*9*}	CNC1=C(N=C2C=NC=CN12)C1=CC(C=C)=CN=C1 |c:6,8,17,19,t:2,4,13|	Z8873684792	**1**{*81*}	Nc1cnccn1	**2**{*453*}	Cl.C=Cc1cncc(C=O)c1	**3**{*9*}	C[N+]#[C-]	12.3	18
876	**4**{*394*,*169*,*10*}	FC1=C(C=C)C=CC2=NC(=C(NC3CCOC3)N12)C1=NNC2=C1N=CC=C2 |c:1,5,24,27,29,t:7,9,21|	Z8878918526	**1**{*394*}	Nc1ccc(C=C)c(F)n1	**2**{*169*}	O=Cc1n[nH]c2cccnc12	**3**{*10*}	[C-]#[N+]C1CCOC1	17.7	18
877	**4**{*93*,*454*,*61*}	COC(C)CC1=C(NCCCNC(=O)OC(C)(C)C)N2C=C(OC)N=CC2=N1 |c:5,24,27,t:20|	Z8878918849	**1**{*93*}	COc1cnc(N)cn1	**2**{*454*}	COC(C)CC=O	**3**{*61*}	CC(C)(C)OC(=O)NCCC[N+]#[C-]	18.6	18
878	**4**{*103*,*455*,*59*}	CC1=C(C(Br)=NO1)C1=C(NCCC2=CC(F)=CC=C2)N2C(C=NC=C2CO)=N1 |c:4,8,16,18,23,25,29,t:1,13|	Z8878918462	**1**{*103*}	Nc1cncc(CO)n1	**2**{*455*}	Cc1onc(Br)c1C=O	**3**{*59*}	Fc1cccc(CC[N+]#[C-])c1	21.0	17
879	**4**{*171*,*216*,*53*}	COC(=O)C1=NC=C(S1)C1=C(NCCOCC2=CC=CC=C2)N2C=CC(=CC2=N1)N1C=CN=C1 |c:6,10,19,21,25,27,30,34,36,t:4,17|	Z8878918572	**1**{*171*}	Nc1cc(ccn1)n2ccnc2	**2**{*216*}	COC(=O)c1ncc(C=O)s1	**3**{*53*}	[C-]#[N+]CCOCc1ccccc1	22.2	17
880	**4**{*120*,*456*,*71*}	COC1=CC(NC2=C(N=C3C=C(C=CN23)C2=CN(C)N=C2)C2=CC(=CO2)S(N)(=O)=O)=CC=C1 |c:10,12,21,26,33,35,t:2,6,8,17,24|	Z8873685316	**1**{*120*}	Cn1cc(cn1)c2ccnc(N)c2	**2**{*456*}	NS(=O)(=O)c1coc(C=O)c1	**3**{*71*}	COc1cccc([N+]#[C-])c1	21.7	17
881	**4**{*186*,*457*,*29*}	CCOC(=O)CCNC1=C(N=C2C=C(C=CN12)S(=O)(=O)N(C)C)C1=NN=C(C=C1)C(F)(F)F |c:12,14,27,29,t:8,10,25|	Z8878918863	**1**{*186*}	Cl.CN(C)S(=O)(=O)c1ccnc(N)c1	**2**{*457*}	FC(F)(F)c1ccc(C=O)nn1	**3**{*29*}	CCOC(=O)CC[N+]#[C-]	22.6	17
882	**4**{*78*,*458*,*51*}	COC1=C(C)C=CC(=N1)C1=C(NCCNC(=O)OC(C)(C)C)N2N3C=C(C)N=C3C(C)=CC2=N1 |c:2,5,7,10,28,32,35,t:25|	Z8878918675	**1**{*78*}	Cc1cn2nc(N)cc(C)c2n1	**2**{*458*}	COc1nc(C=O)ccc1C	**3**{*51*}	CC(C)(C)OC(=O)NCC[N+]#[C-]	21.1	17
883	**4**{*363*,*459*,*62*}	COC(=O)C1=CC2=NC(=C(NC3(CCCC3)C(=O)OC)N2C=C1)C1=NNC(C)=C1C |c:23,30,t:4,6,8,26|	Z8873684726	**1**{*363*}	COC(=O)c1ccnc(N)c1	**2**{*459*}	Cc1[nH]nc(C=O)c1C	**3**{*62*}	COC(=O)C1(CCCC1)[N+]#[C-]	18.5	17

884	**4**{*35* ,*396* ,*13* }	COC1=CC=C(CNC2=C(N=C3C=CC(C)=CN23)C2=CN=C3COCCN23)C=C1 |c:12,15,31,t:2,4,8,10,20,22|	Z8873684635	**1**{*35* }	Cc1ccc(N)nc1	**2**{*396* }	O=Cc1cnc2COCCn12	**3**{*13* }	COc1ccc(C[N+]#[C-])cc1	17.3	16
885	**4**{*193* ,*460* ,*60* }	CNC(=O)C1=CN2C(NC3=CC(=CC=C3)C(=O)OC)=C(N=C2C=C1)C1=NC=C(C)O1 |c:11,13,19,21,24,t:4,9,27,29|	Z8835022877	**1**{*193* }	CNC(=O)c1ccc(N)nc1	**2**{*460* }	Cc1cnc(C=O)o1	**3**{*60* }	COC(=O)c1cccc([N+]#[C-])c1	17.9	16
886	**4**{*395* ,*461* ,*7* }	CCN1N=CC(=C1F)C1=C(NC2CCOCC2)N2N=C(C=C(C)C2=N1)C(F)(F)F |c:3,5,9,20,26,t:22|	Z8878918396	**1**{*395* }	Cc1cc(nnc1N)C(F)(F)F	**2**{*461* }	CCn1ncc(C=O)c1F	**3**{*7* }	[C-]#[N+]C1CCOCC1	18.1	16
887	**4**{*137* ,*284* ,*20* }	COC1=CC(C)=C(NC2=C(N=C3C=CC(C)=NN23)C2=C(C)N=CC=N2)C=C1 |c:12,15,20,23,25,28,t:2,5,8,10|	Z8873684616	**1**{*137* }	Cc1ccc(N)nn1	**2**{*284* }	Cc1nccnc1C=O	**3**{*20* }	COc1ccc([N+]#[C-])c(C)c1	15.7	16
888	**4**{*99* ,*229* ,*50* }	NS(=O)(=O)C1=CN2C(NC3=CC4=C(OCCO4)C=C3)=C(N=C2C=C1)C1=CN=C(OCCO)C=C1 |c:18,20,22,25,36,t:4,9,11,28,30|	Z8837933134	**1**{*99* }	Nc1ccc(cn1)S(=O)(=O)N	**2**{*229* }	OCCOc1ccc(C=O)cn1	**3**{*50* }	[C-]#[N+]c1ccc2OCCOc2c1	21.0	16
889	**4**{*103* ,*354* ,*40* }	CCOC(=O)C1=CC=C(NC2=C(N=C3C=NC=C(CO)N23)C2=C(C=CC=C2)P(C)(C)=O)C=C1 |c:14,25,27,34,t:5,7,10,12,16,23|	Z8878918669	**1**{*103* }	Nc1cncc(CO)n1	**2**{*354* }	CP(=O)(C)c1ccccc1C=O	**3**{*40* }	CCOC(=O)c1ccc([N+]#[C-])cc1	20.1	16
890	**4**{*78* ,*462* ,*39* }	CCOCCNC1=C(N=C2C=C(C)C3=NC(C)=CN3N12)C1=C(Br)C=CC(C)=C1F |c:16,23,26,29,t:6,8,10,13|	Z8873685495	**1**{*78* }	Cc1cn2nc(N)cc(C)c2n1	**2**{*462* }	Cc1ccc(Br)c(C=O)c1F	**3**{*39* }	CCOCC[N+]#[C-]	19.7	16
891	**4**{*291* ,*274* ,*13* }	COC1=CC=C(CNC2=C(CC3(CCOCC3)C#N)N=C3N2C=C(Cl)C=C3Cl)C=C1 |c:8,20,27,31,t:2,4,24|	Z8873685489	**1**{*291* }	Nc1ncc(Cl)cc1Cl	**2**{*274* }	O=CCC1(CCOCC1)C#N	**3**{*13* }	COc1ccc(C[N+]#[C-])cc1	18.8	16
892	**4**{*167* ,*463* ,*5* }	COCCCNC1=C(N=C2N1C(C)=CN=C2C)C1=C(C)C(I)=NN1C |c:8,13,15,19,23,t:6|	Z8873684713	**1**{*167* }	Cc1cnc(C)c(N)n1	**2**{*463* }	Cc1c(I)nn(C)c1C=O	**3**{*5* }	COCCC[N+]#[C-]	18.9	15
893	**4**{*50* ,*464* ,*31* }	CSCCNC1=C(N=C2C=CC=C(N12)P(C)(C)=O)C1=CC2=C(C=CC=C2)N2N=NN=C12 |c:9,11,24,26,30,t:5,7,20,22,32|	Z8873685251	**1**{*50* }	CP(=O)(C)c1cccc(N)n1	**2**{*464* }	O=Cc1cc2ccccc2n3nnnc13	**3**{*31* }	CSCC[N+]#[C-]	17.7	14
894	**4**{*230* ,*349* ,*34* }	CC(O)C1=CC(Br)=CN2C(NCC3CCCO3)=C(N=C12)C1C[C@H]2OC(C)(C)O[C@H]2C1 |c:6,17,t:3,19|	Z8873685493	**1**{*230* }	CC(O)c1cc(Br)cnc1N	**2**{*349* }	CC1(C)O[C@@H]2CC(C[C@@H]2O1)C=O	**3**{*34* }	[C-]#[N+]CC1CCCO1	18.8	14
895	**4**{*79* ,*129* ,*13* }	COC1=CC=C(CNC2=C(N=C3C=NC=C(C)N23)C2=CC=CC3=NC=NN23)C=C1 |c:12,22,26,31,t:2,4,8,10,14,20,24|	Z8878918798	**1**{*79* }	Cc1cncc(N)n1	**2**{*129* }	O=Cc1cccc2ncnn12	**3**{*13* }	COc1ccc(C[N+]#[C-])cc1	15.0	14

896	**4**{*382*,*305*,*71*}	COCC1=NC(=CN1)C1=C(NC2=CC(OC)=CC=C2)N2C=C(N=CC2=N1)C(F)(F)F |c:5,9,16,18,22,24,27,t:3,12|	Z8878918670	**1**{*382*}	Nc1cnc(cn1)C(F)(F)F	**2**{*305*}	COCc1nc(C=O)c[nH]1	**3**{*71*}	COc1cccc([N+]#[C-])c1	16.1	14
897	**4**{*179*,*465*,*23*}	COC(=O)CCC(NC1=C(N=C2C=C(OC)C=CN12)C1=C(Cl)C=C(C)C=N1)C(=O)OC |c:16,21,27,t:8,10,12,24|	Z8878918401	**1**{*179*}	COc1ccnc(N)c1	**2**{*465*}	Cc1cnc(C=O)c(Cl)c1	**3**{*23*}	COC(=O)CCC([N+]#[C-])C(=O)OC	17.2	14
898	**4**{*396*,*466*,*23*}	COC(=O)CCC(NC1=C(N=C2C=CC(=NN12)C1=CN(C)N=C1)C1=CC(Cl)=C(C)N=C1)C(=O)OC |c:12,14,23,32,t:8,10,19,26,29|	Z8829498618	**1**{*396*}	Cn1cc(cn1)c2ccc(N)nn2	**2**{*466*}	Cc1ncc(C=O)cc1Cl	**3**{*23*}	COC(=O)CCC([N+]#[C-])C(=O)OC	19.0	14
899	**4**{*181*,*467*,*14*}	COCCNC1=C(N=C2N1C=CC1=C2N=CC=N1)C1=C(C)SC(Br)=N1 |c:7,11,13,16,18,21,26,t:5|	Z8855739274	**1**{*181*}	Nc1nccc2nccnc12	**2**{*467*}	Cc1sc(Br)nc1C=O	**3**{*14*}	COCC[N+]#[C-]	15.9	14
900	**4**{*54*,*468*,*37*}	COC(=O)C1CC(C1)NC1=C(CC(C)SC)N=C2C=CC(=CN12)C(=O)N(C)C |c:10,19,21,t:17|	Z8873685443	**1**{*54*}	Cl.CN(C)C(=O)c1ccc(N)nc1	**2**{*468*}	CSC(C)CC=O	**3**{*37*}	COC(=O)C1CC(C1)[N+]#[C-]	15.2	14
901	**4**{*169*,*469*,*11*}	CNC(=O)C1=NN2C(C=C1)=NC(C1CCSC1)=C2NCC1=CC(Br)=CC=C1 |c:8,10,18,26,28,t:4,23|	Z8829498482	**1**{*169*}	CNC(=O)c1ccc(N)nn1	**2**{*469*}	O=CC1CCSC1	**3**{*11*}	Brc1cccc(C[N+]#[C-])c1	16.5	14
902	**4**{*72*,*470*,*12*}	COC1=NC(Br)=CN2C(NCC3CCOC3)=C(N=C12)C1=COC(Br)=N1 |c:5,16,26,t:2,18,22|	Z8878918789	**1**{*72*}	COc1nc(Br)cnc1N	**2**{*470*}	Brc1nc(C=O)co1	**3**{*12*}	[C-]#[N+]CC1CCOC1	17.5	14
903	**4**{*251*,*471*,*62*}	COC(=O)C1(CCCC1)NC1=C(N=C2C=CC3=C(NC(=O)C=C3)N12)C1=NC=C2OCOC2=C1 |c:15,22,36,t:11,13,17,28,30|	Z8873684899	**1**{*251*}	Nc1ccc2ccc(=O)[nH]c2n1	**2**{*471*}	O=Cc1cc2OCOc2cn1	**3**{*62*}	COC(=O)C1(CCCC1)[N+]#[C-]	16.4	14
904	**4**{*73*,*94*,*11*}	BrC1=CC(CNC2=C(N=C3C=CC4=NC=CN4N23)C2=NC=CN=C2)=CC=C1 |c:10,14,23,25,27,29,t:1,6,8,12,21|	Z8873685473	**1**{*73*}	Nc1ccc2nccn2n1	**2**{*94*}	O=Cc1cnccn1	**3**{*11*}	Brc1cccc(C[N+]#[C-])c1	15.3	13
905	**4**{*83*,*472*,*26*}	COC(=O)C1=CC2=C(C=NN2C=C1)C1=C(NC2=CC=C(F)C=C2)N2C=C(C=CC2=N1)P(C)(C)=O |c:8,12,15,24,28,30,33,t:4,6,19,21|	Z8846491855	**1**{*83*}	CP(=O)(C)c1ccc(N)nc1	**2**{*472*}	COC(=O)c1ccn2ncc(C=O)c2c1	**3**{*26*}	Fc1ccc(C[N+]#[C-])cc1	17.9	13
906	**4**{*397*,*177*,*5*}	COCCCNC1=C(CC2CC(C2)C(=O)OC)N=C2N1C=CC(C)=C2C |c:6,18,22,25|	Z8873684719	**1**{*397*}	Cl.Cc1ccnc(N)c1C	**2**{*177*}	COC(=O)C1CC(CC=O)C1	**3**{*5*}	COCCC[N+]#[C-]	13.1	13
907	**4**{*229*,*473*,*68*}	COC1=CC(C)=C(C=C1)C1=C(NC2CCCCC2)N2C=C(C=CC2=N1)S(O)(=O)=O |c:5,7,10,21,23,26,t:2|	Z8829498563	**1**{*229*}	Nc1ccc(cn1)S(=O)(=O)O	**2**{*473*}	COc1ccc(C=O)c(C)c1	**3**{*68*}	[C-]#[N+]C1CCCCC1	15.0	13

<table>
<tr><td></td><td></td><td></td><td></td><td></td><td></td><td></td><td></td><td></td><td></td><td></td><td></td></tr>
<tr><td>908</td><td>**4**{*222*,*193*,*62*}</td><td>COC(=O)C1=CN2C(NC3(CCCC3)C(=O)OC)=C(N=C2C=N1)C1=NN2CCCCOC2=C1 |c:18,20,23,35,t:4,26|</td><td>Z8808559886</td><td>**1**{*222*}</td><td>Cl.COC(=O)c1cnc(N)cn1</td><td>**2**{*193*}</td><td>O=Cc1cc2OCCCCn2n1</td><td>**3**{*62*}</td><td>COC(=O)C1(CCCC1)[N+]#[C-]</td><td>16.3</td><td>13</td></tr>
<tr><td>909</td><td>**4**{*78*,*474*,*51*}</td><td>CC1=CN2N3C(NCCNC(=O)OC(C)(C)C)=C(N=C3C=C(C)C2=N1)C1=NNC2=C1C=CC=N2 |c:16,18,25,31,34,36,t:1,21,28|</td><td>Z8878918667</td><td>**1**{*78*}</td><td>Cc1cn2nc(N)cc(C)c2n1</td><td>**2**{*474*}</td><td>O=Cc1n[nH]c2ncccc12</td><td>**3**{*51*}</td><td>CC(C)(C)OC(=O)NCC[N+]#[C-]</td><td>16.5</td><td>13</td></tr>
<tr><td>910</td><td>**4**{*167*,*475*,*5*}</td><td>COCCCNC1=C(N=C2N1C(C)=CN=C2C)C1=CC2=C(O1)C(F)=NC=C2 |c:8,13,15,21,26,28,t:6,19|</td><td>Z8878918907</td><td>**1**{*167*}</td><td>Cc1cnc(C)c(N)n1</td><td>**2**{*475*}</td><td>Fc1nccc2cc(C=O)oc12</td><td>**3**{*5*}</td><td>COCCC[N+]#[C-]</td><td>12.8</td><td>13</td></tr>
<tr><td>911</td><td>**4**{*398*,*130*,*32*}</td><td>COC1=NN=C(C=C1)C1=C(NC2CCC2)N2C=CC(=CC2=N1)C(C)(F)F |c:4,6,9,18,20,23,t:2|</td><td>Z8854581139</td><td>**1**{*398*}</td><td>Cl.CC(F)(F)c1ccnc(N)c1</td><td>**2**{*130*}</td><td>COc1ccc(C=O)nn1</td><td>**3**{*32*}</td><td>[C-]#[N+]C1CCC1</td><td>12.4</td><td>13</td></tr>
<tr><td>912</td><td>**4**{*27*,*368*,*21*}</td><td>COC1=CC=CC2=NC(=C(NCC3CC3)N12)C1=NN=C(C)C(C)=C1 |c:4,25,t:2,6,8,19,21|</td><td>Z8855739205</td><td>**1**{*27*}</td><td>COc1cccc(N)n1</td><td>**2**{*368*}</td><td>Cc1cc(C=O)nnc1C</td><td>**3**{*21*}</td><td>[C-]#[N+]CC1CC1</td><td>10.9</td><td>13</td></tr>
<tr><td>913</td><td>**4**{*99*,*476*,*57*}</td><td>COC(=O)CCC1=NOC=C1C1=C(NCC2=CC=CC=C2)N2C=C(C=CC2=N1)S(N)(=O)=O |c:9,12,18,20,24,26,29,t:6,16|</td><td>Z8854581136</td><td>**1**{*99*}</td><td>Nc1ccc(cn1)S(=O)(=O)N</td><td>**2**{*476*}</td><td>COC(=O)CCc1noccc1C=O</td><td>**3**{*57*}</td><td>[C-]#[N+]Cc1ccccc1</td><td>15.4</td><td>13</td></tr>
<tr><td>914</td><td>**4**{*399*,*477*,*37*}</td><td>COC(=O)C1CC(C1)NC1=C(N=C2C=CC(OC(C)C)=NN12)C(NC(=O)OC(C)(C)C)C1COC1 |c:14,20,t:10,12|</td><td>Z8846492223</td><td>**1**{*399*}</td><td>CC(C)Oc1ccc(N)nn1</td><td>**2**{*477*}</td><td>CC(C)(C)OC(=O)NC(C=O)C1COC1</td><td>**3**{*37*}</td><td>COC(=O)C1CC(C1)[N+]#[C-]</td><td>16.5</td><td>12</td></tr>
<tr><td>915</td><td>**4**{*77*,*478*,*5*}</td><td>COCCCNC1=C(N=C2C=CC(=CN12)C1=CN=CC=C1)C1=CN(C)C(=O)C2=C1C=CN=C2 |c:10,12,19,21,30,33,35,t:6,8,17,24|</td><td>Z8873684782</td><td>**1**{*77*}</td><td>Nc1ccc(cn1)c2cccnc2</td><td>**2**{*478*}</td><td>Cn1cc(C=O)c2ccncc2c1=O</td><td>**3**{*5*}</td><td>COCCC[N+]#[C-]</td><td>14.9</td><td>12</td></tr>
<tr><td>916</td><td>**4**{*181*,*479*,*14*}</td><td>COCCNC1=C(N=C2N1C=CC1=C2N=CC=N1)C1=CN(N=C1)C1=CC(C)=CC=C1 |c:7,11,13,16,18,24,30,32,t:5,21,27|</td><td>Z8878918565</td><td>**1**{*181*}</td><td>Nc1nccc2nccnc12</td><td>**2**{*479*}</td><td>Cc1cccc(c1)n2cc(C=O)cn2</td><td>**3**{*14*}</td><td>COCC[N+]#[C-]</td><td>13.4</td><td>12</td></tr>
<tr><td>917</td><td>**4**{*67*,*396*,*9*}</td><td>CNC1=C(N=C2C=CC=C(C)N12)C1=CN=C2COCCN12 |c:6,t:2,4,8,14,16|</td><td>Z8873685379</td><td>**1**{*67*}</td><td>Cc1cccc(N)n1</td><td>**2**{*396*}</td><td>O=Cc1cnc2COCCn12</td><td>**3**{*9*}</td><td>C[N+]#[C-]</td><td>9.5</td><td>12</td></tr>
<tr><td>918</td><td>**4**{*169*,*25*,*13*}</td><td>CNC(=O)C1=NN2C(C=C1)=NC(=C2NCC1=CC=C(OC)C=C1)C1=CN=C(N=C1)C1CCOCC1 |c:8,10,12,23,28,30,t:4,17,19,26|</td><td>Z8855619638</td><td>**1**{*169*}</td><td>CNC(=O)c1ccc(N)nn1</td><td>**2**{*25*}</td><td>O=Cc1cnc(nc1)C2CCOCC2</td><td>**3**{*13*}</td><td>COc1ccc(C[N+]#[C-])cc1</td><td>15.6</td><td>12</td></tr>
<tr><td>919</td><td>**4**{*198*,*189*,*31*}</td><td>CSCCNC1=C(N=C2C=NC(C#C)=C(Cl)N12)C1=CN(N=N1)C1CCOCC1 |c:9,22,t:5,7,13,19|</td><td>Z8801681831</td><td>**1**{*198*}</td><td>Nc1cnc(C#C)c(Cl)n1</td><td>**2**{*189*}</td><td>O=Cc1cn(nn1)C2CCOCC2</td><td>**3**{*31*}</td><td>CSCC[N+]#[C-]</td><td>13.2</td><td>12</td></tr>
</table>

920	**4**{*88*,*480*,*34*}	FC1=C(Br)C=CN2C(NCC3CCCO3)=C(N=C12)C1=CC2=C(CCOC2)N=C1 |c:1,4,15,30,t:17,21,23|	Z8878918389	**1**{*88*}	Nc1nccc(Br)c1F	**2**{*480*}	O=Cc1cnc2CCOCc2c1	**3**{*34*}	[C-]#[N+]CC1CCCO1	14.0	12
921	**4**{*150*,*481*,*22*}	CC1=NNC2=C1C=CC(=C2)C1=C(NC2COC2)N2C=CC(=CC2=N1)C1CCOC1 |c:4,7,9,12,21,23,26,t:1|	Z8873685244	**1**{*150*}	Nc1cc(ccn1)C2CCOC2	**2**{*481*}	Cc1n[nH]c2cc(C=O)ccc12	**3**{*22*}	[C-]#[N+]C1COC1	11.8	11
922	**4**{*370*,*281*,*10*}	COC(=O)C1=NN=C(C=C1)C1=C(NC2CCOC2)N2C=CC(Br)=C(OC)C2=N1 |c:6,8,11,21,29,t:4,24|	Z8878918604	**1**{*370*}	COc1c(N)nccc1Br	**2**{*281*}	COC(=O)c1ccc(C=O)nn1	**3**{*10*}	[C-]#[N+]C1CCOC1	13.5	11
923	**4**{*158*,*482*,*7*}	CP(C)(=O)C1=CN2C(C=N1)=NC(=C2NC1CCOCC1)C1=CC(=CC(Cl)=C1)C(F)(F)F |c:8,10,12,25,28,t:4,23|	Z8855739184	**1**{*158*}	CP(=O)(C)c1cnc(N)cn1	**2**{*482*}	FC(F)(F)c1cc(Cl)cc(C=O)c1	**3**{*7*}	[C-]#[N+]C1CCOCC1	13.7	11
924	**4**{*351*,*483*,*71*}	COC1=CC(NC2=C(N=C3C=CC4=C(C(O)CC4)N23)C2=CN(C=N2)C2CC2)=CC=C1 |c:10,25,31,33,t:2,6,8,12,22|	Z8849597808	**1**{*351*}	Nc1ccc2CCC(O)c2n1	**2**{*483*}	Cl.O=Cc1cn(cn1)C2CC2	**3**{*71*}	COc1cccc([N+]#[C-])c1	11.5	11
925	**4**{*99*,*484*,*50*}	NS(=O)(=O)C1=CN2C(NC3=CC4=C(OCCO4)C=C3)=C(N=C2C=C1)C1=CC(=CS1)C#N |c:18,20,22,25,30,t:4,9,11,28|	Z8873685404	**1**{*99*}	Nc1ccc(cn1)S(=O)(=O)N	**2**{*484*}	O=Cc1cc(C#N)cs1	**3**{*50*}	[C-]#[N+]c1ccc2OCCOc2c1	12.8	10
926	**4**{*166*,*173*,*32*}	CN1N=C(N=C1C1=C(NC2CCC2)N2C=CC(CSCCO)=CC2=N1)C(F)(F)F |c:2,4,7,16,23,26|	Z8878918387	**1**{*166*}	Nc1cc(CSCCO)ccn1	**2**{*173*}	Cn1nc(nc1C=O)C(F)(F)F	**3**{*32*}	[C-]#[N+]C1CCC1	11.9	10
927	**4**{*79*,*485*,*13*}	COC1=CC=C(CNC2=C(N=C3C=NC=C(C)N23)C2=NOC3=C2CCC3)C=C1 |c:12,23,30,t:2,4,8,10,14,20|	Z8873684741	**1**{*79*}	Cc1cncc(N)n1	**2**{*485*}	O=Cc1noc2CCc12	**3**{*13*}	COc1ccc(C[N+]#[C-])cc1	10.2	10
928	**4**{*146*,*486*,*46*}	CN1C(Cl)=NC(Cl)=C1C1=C(NCC(C)=C)N2C=C(C=CC2=N1)S(C)(=O)=O |c:3,6,9,17,19,22|	Z8878918499	**1**{*146*}	CS(=O)(=O)c1ccc(N)nc1	**2**{*486*}	Cn1c(Cl)nc(Cl)c1C=O	**3**{*46*}	CC(=C)C[N+]#[C-]	11.1	10
929	**4**{*112*,*487*,*61*}	COC1=CN=CC2=NC(=C(NCCCNC(=O)OC(C)(C)C)N12)C1=C(Cl)C=NN1C |c:4,25,28,t:2,6,8|	Z8873684760	**1**{*112*}	COc1cncc(N)n1	**2**{*487*}	Cn1ncc(Cl)c1C=O	**3**{*61*}	CC(C)(C)OC(=O)NCCC[N+]#[C-]	11.0	9
930	**4**{*400*,*488*,*15*}	CCNC1=C(N=C2C=CC(COC(C)C)=CN12)C1=CC2=C(NC(=O)CC2)N=C1 |c:7,14,29,t:3,5,19,21|	Z8878918822	**1**{*400*}	CC(C)OCc1ccc(N)nc1	**2**{*488*}	O=Cc1cnc2NC(=O)CCc2c1	**3**{*15*}	CC[N+]#[C-]	9.5	9
931	**4**{*399*,*489*,*22*}	CC(C)OC1=NN2C(NC3COC3)=C(N=C2C=C1)C1=NOC(=C1)C1CCC1 |c:13,15,18,24,t:4,21|	Z8854581166	**1**{*399*}	CC(C)Oc1ccc(N)nn1	**2**{*489*}	O=Cc1cc(on1)C2CCC2	**3**{*22*}	[C-]#[N+]C1COC1	9.1	9

932	**4**{*401,296,39*}	CCOCCNC1=C(N=C2C=CC3=C(N=CC=C3Cl)N12)C1=CN(CCF)N=C1 |c:10,14,16,29,t:6,8,12,23|	Z8873684628	**1**{*401*}	Nc1ccc2c(Cl)ccnc2n1	**2**{*296*}	FCCn1cc(C=O)cn1	**3**{*39*}	CCOCC[N+]#[C-]	9.9	9
933	**4**{*83,490,18*}	CC(C)(C)NC1=C(N=C2C=CC(=CN12)P(C)(C)=O)C1=CC(OC(F)(F)CF)=CC=C1 |c:9,11,28,30,t:5,7,20|	Z8878918343	**1**{*83*}	CP(=O)(C)c1ccc(N)nc1	**2**{*490*}	FCC(F)(F)Oc1cccc(C=O)c1	**3**{*18*}	CC(C)(C)[N+]#[C-]	10.5	9
934	**4**{*79,129,9*}	CNC1=C(N=C2C=NC=C(C)N12)C1=CC=CC2=NC=NN12 |c:6,16,20,t:2,4,8,14,18|	Z8849597841	**1**{*79*}	Cc1cncc(N)n1	**2**{*129*}	O=Cc1cccc2ncnn12	**3**{*9*}	C[N+]#[C-]	6.3	8
935	**4**{*125,212,15*}	CCNC1=C(N=C2C=CC(CCO)=CN12)C1=NOC(=C1)C(F)F |c:7,12,20,t:3,5,17|	Z8808559873	**1**{*125*}	Nc1ccc(CCO)cn1	**2**{*212*}	FC(F)c1cc(C=O)no1	**3**{*15*}	CC[N+]#[C-]	7.1	8
936	**4**{*291,27,9*}	CNC1=C(N=C2N1C=C(Cl)C=C2Cl)C1=NN(C)C(C)=N1 |c:4,11,20,t:2,8,15|	Z8878918327	**1**{*291*}	Nc1ncc(Cl)cc1Cl	**2**{*27*}	Cc1nc(C=O)nn1C	**3**{*9*}	C[N+]#[C-]	6.5	8
937	**4**{*402,491,49*}	CC(C)(C)OC(=O)N1CCC2CC12CC1=C(NC2CC(F)(F)C2)N2C(C=NC=C2F)=N1 |c:16,28,30,33|	Z8835022917	**1**{*402*}	Nc1cncc(F)n1	**2**{*491*}	CC(C)(C)OC(=O)N1CCC2CC21CC=O	**3**{*49*}	FC1(F)CC(C1)[N+]#[C-]	8.8	7
938	**4**{*216,190,10*}	CC1=CC2=NC(C3=CC(=NN3)C3=NC=CC=C3)=C(NC3CCOC3)N2N=C1C |c:8,14,16,29,t:1,3,6,12,18|	Z8801681808	**1**{*216*}	Cc1cc(N)nnc1C	**2**{*190*}	O=Cc1cc(n[nH]1)c2ccccn2	**3**{*10*}	[C-]#[N+]C1CCOC1	7.5	7
939	**4**{*251,492,70*}	CC1=NC(C)=C(C)N=C1C1=C(NC2CCN(C2)C(=O)OC(C)(C)C)N2C(C=CC3=C2NC(=O)C=C3)=N1 |c:7,10,28,30,36,38,t:1,4|	Z8873684809	**1**{*251*}	Nc1ccc2ccc(=O)[nH]c2n1	**2**{*492*}	Cc1nc(C)c(C=O)nc1C	**3**{*70*}	CC(C)(C)OC(=O)N1CCC(C1)[N+]#[C-]	8.9	7
940	**4**{*240,277,22*}	CC(C)(C)OC(=O)NC(CC1=C(NC2COC2)N2C=C(C=CC2=N1)C1=NNN=N1)C1CC1 |c:10,19,21,24,30,t:27|	Z8878918548	**1**{*240*}	Nc1ccc(cn1)c2nn[nH]n2	**2**{*277*}	CC(C)(C)OC(=O)NC(CC=O)C1CC1	**3**{*22*}	[C-]#[N+]C1COC1	8.7	7
941	**4**{*73,493,13*}	COC1=CC=C(CNC2=C(N=C3C=CC4=NC=CN4N23)C2=C(F)C=CC=N2)C=C1 |c:12,16,23,26,28,31,t:2,4,8,10,14|	Z8878918871	**1**{*73*}	Nc1ccc2nccn2n1	**2**{*493*}	Fc1cccnc1C=O	**3**{*13*}	COc1ccc(C[N+]#[C-])cc1	6.6	6
942	**4**{*92,310,18*}	CC(C)(C)NC1=C(N=C2C=C(C=CN12)C(N)=O)C1CCOC2(CCC2)C1 |c:9,11,t:5,7|	Z8855619753	**1**{*92*}	NC(=O)c1ccnc(N)c1	**2**{*310*}	O=CC1CCOC2(CCC2)C1	**3**{*18*}	CC(C)(C)[N+]#[C-]	6.0	6
943	**4**{*158,448,31*}	COC1=C2OCOC2=CC(=C1)C1=C(NCCSC)N2C=C(N=CC2=N1)P(C)(C)=O |c:2,8,10,13,21,23,26|	Z8835022883	**1**{*158*}	CP(=O)(C)c1cnc(N)cn1	**2**{*448*}	COc1cc(C=O)cc2OCOc21	**3**{*31*}	CSCC[N+]#[C-]	7.0	6

944	**4**{*330*,*250*,*1*}	CN(C)S(=O)(=O)C1=CN2C(C=C1)=NC(=C2NC1CC1)C1=CN(C)N=C1Br |c:10,12,14,26,t:6,22|	Z8808559863	**1**{*330*}	CN(C)S(=O)(=O)c1ccc(N)nc1	**2**{*250*}	Cn1cc(C=O)c(Br)n1	**3**{*1*}	[C-]#[N+]C1CC1	7.0	6
945	**4**{*197*,*404*,*2*}	CC(C)CS(=O)(=O)C1=CN2C(C=C1)=NC(C1=NN=C(C)N1)=C2NC1=CC=C(F)C=C1 |c:11,13,22,31,t:7,16,18,26,28|	Z8878918439	**1**{*197*}	CC(C)CS(=O)(=O)c1ccc(N)nc1	**2**{*404*}	Cc1nnc(C=O)[nH]1	**3**{*2*}	Fc1ccc([N+]#[C-])cc1	6.4	6
946	**4**{*73*,*494*,*20*}	COC1=CC(C)=C(NC2=C(N=C3C=CC4=NC=CN4N23)C2=C(Cl)N=CS2)C=C1 |c:12,16,23,26,30,t:2,5,8,10,14|	Z8878918470	**1**{*73*}	Nc1ccc2nccn2n1	**2**{*494*}	Clc1ncsc1C=O	**3**{*20*}	COc1ccc([N+]#[C-])c(C)c1	5.6	5
947	**4**{*28*,*11*,*26*}	FC1=CC=C(CNC2=C(N=C3C=C(C=CN23)C2=NOC=N2)C2=NN(CC(F)(F)F)C=C2)C=C1 |c:11,13,21,32,35,t:1,3,7,9,18,24|	Z8873685392	**1**{*28*}	Cl.Nc1cc(ccn1)c2ncon2	**2**{*11*}	FC(F)(F)Cn1ccc(C=O)n1	**3**{*26*}	Fc1ccc(C[N+]#[C-])cc1	6.2	5
948	**4**{*403*,*495*,*26*}	COC(=O)C1=NN2C(C=C1)=NC(=C2NCC1=CC=C(F)C=C1)C1=C(Cl)C=CC=N1 |c:8,10,12,22,25,28,30,t:4,17,19|	Z8778278034	**1**{*403*}	COC(=O)c1ccc(N)nn1	**2**{*495*}	Clc1cccnc1C=O	**3**{*26*}	Fc1ccc(C[N+]#[C-])cc1	5.5	5
949	**4**{*188*,*496*,*62*}	COC(=O)C1=CC(=CC(=C1)[N+]([O-])=O)C1=C(NC2(CCCC2)C(=O)OC)N2C(C=NC=C2C(=O)OC)=N1 |c:6,8,14,29,31,37,t:4|	Z8854581134	**1**{*188*}	COC(=O)c1cncc(N)n1	**2**{*496*}	COC(=O)c1cc(C=O)cc(c1)[N+](=O)[O-]	**3**{*62*}	COC(=O)C1(CCCC1)[N+]#[C-]	6.1	5
950	**4**{*94*,*497*,*43*}	CCN1N=CC(C)=C1C1=C(NC2CCN(CC2)C(=O)OC(C)(C)C)N2N=C(F)C=CC2=N1 |c:3,6,9,30,33,t:27|	Z8878918795	**1**{*94*}	Nc1ccc(F)nn1	**2**{*497*}	CCn1ncc(C)c1C=O	**3**{*43*}	CC(C)(C)OC(=O)N1CCC(CC1)[N+]#[C-]	5.3	4
951	**4**{*354*,*108*,*71*}	COC(=O)C1=CN2C(C=C1)=NC(=C2NC1=CC(OC)=CC=C1)C1=CN=C(N=C1)N1CCOCC1 |c:8,10,12,20,22,27,29,t:4,16,25|	Z8878918649	**1**{*354*}	COC(=O)c1ccc(N)nc1	**2**{*108*}	O=Cc1cnc(nc1)N2CCOCC2	**3**{*71*}	COc1cccc([N+]#[C-])c1	5.5	4
952	**4**{*267*,*498*,*23*}	COC(=O)CCC(NC1=C(N=C2C=CC=C(C#C)N12)C1=C(Br)N=CS1)C(=O)OC |c:12,21,24,t:8,10,14|	Z8854581160	**1**{*267*}	Nc1cccc(C#C)n1	**2**{*498*}	Brc1ncsc1C=O	**3**{*23*}	COC(=O)CCC([N+]#[C-])C(=O)OC	5.5	4
953	**4**{*181*,*499*,*14*}	COCCNC1=C(N=C2N1C=CC1=C2N=CC=N1)C1=C(OC)C(F)=C(I)C=C1 |c:7,11,13,16,18,21,29,t:5,26|	Z8878918786	**1**{*181*}	Nc1nccc2nccnc12	**2**{*499*}	COc1c(F)c(I)ccc1C=O	**3**{*14*}	COCC[N+]#[C-]	5.5	4
954	**4**{*251*,*500*,*62*}	COC(=O)C1(CCCC1)NC1=C(N=C2C=CC3=C(NC(=O)C=C3)N12)C1=CN=CS1 |c:15,22,30,t:11,13,17,28|	Z8878918867	**1**{*251*}	Nc1ccc2ccc(=O)[nH]c2n1	**2**{*500*}	O=Cc1cncs1	**3**{*62*}	COC(=O)C1(CCCC1)[N+]#[C-]	4.3	4
955	**4**{*223*,*501*,*34*}	NC(=O)C1=NN2C(C=C1)=NC(=C2NCC1CCCO1)C1=CC=C(C=C1)N1CCC1 |c:7,9,11,24,26,t:3,22|	Z8829498718	**1**{*223*}	NC(=O)c1ccc(N)nn1	**2**{*501*}	O=Cc1ccc(cc1)N2CCC2	**3**{*34*}	[C-]#[N+]CC1CCCO1	3.9	4

956	**4**{*194*,*502*,*49*}	FC1(F)CC(C1)NC1=C(N=C2N1C=CN=C2N1C=CC=N1)C1=CC=C(C=C1)C1=CNN=C1 |c:10,14,16,20,22,27,29,35,t:8,25,32|	Z8855739082	**1**{*194*}	Nc1nccnc1n2cccn2	**2**{*502*}	Cl.O=Cc1ccc(cc1)c2cn[nH]c2	**3**{*49*}	FC1(F)CC(C1)[N+]#[C-]	4.3	4
957	**4**{*181*,*503*,*14*}	COCCNC1=C(N=C2N1C=CC1=C2N=CC=N1)C1=CSC(Br)=N1 |c:7,11,13,16,18,25,t:5,21|	Z8878918553	**1**{*181*}	Nc1nccc2nccnc12	**2**{*503*}	Brc1nc(C=O)cs1	**3**{*14*}	COCC[N+]#[C-]	3.9	4
958	**4**{*83*,*504*,*31*}	CSCCNC1=C(N=C2C=CC(=CN12)P(C)(C)=O)C1=C(F)C(F)=C(Br)C=C1 |c:9,11,20,27,t:5,7,24|	Z8878918772	**1**{*83*}	CP(=O)(C)c1ccc(N)nc1	**2**{*504*}	Fc1c(F)c(C=O)ccc1Br	**3**{*31*}	CSCC[N+]#[C-]	4.5	4
959	**4**{*73*,*137*,*11*}	CC1=C(C(C)=NO1)C1=C(NCC2=CC(Br)=CC=C2)N2N3C=CN=C3C=CC2=N1 |c:4,8,15,17,22,24,27,30,t:1,12|	Z8878918842	**1**{*73*}	Nc1ccc2nccn2n1	**2**{*137*}	Cc1noc(C)c1C=O	**3**{*11*}	Brc1cccc(C[N+]#[C-])c1	4.1	4
960	**4**{*22*,*207*,*9*}	CNC1=C(N=C2C=CC(Cl)=NN12)C1=CC(=NN1C)C(=O)OC |c:6,9,16,t:2,4,14|	Z8846491866	**1**{*22*}	Nc1ccc(Cl)nn1	**2**{*207*}	COC(=O)c1cc(C=O)n(C)n1	**3**{*9*}	C[N+]#[C-]	3.0	3
961	**4**{*389*,*505*,*23*}	COC(=O)CCC(NC1=C(N=C2N1C=C(C)C=C2C)C1=CC=CN2N=CC=C12)C(=O)OC |c:10,17,23,26,t:8,14,21,28|	Z8878918448	**1**{*389*}	Cc1cnc(N)c(C)c1	**2**{*505*}	O=Cc1cccn2nccc12	**3**{*23*}	COC(=O)CCC([N+]#[C-])C(=O)OC	3.6	3
962	**4**{*329*,*50*,*18*}	COC1=CC=C(C)C2=NC([C@@H]3C[C@H]3C3OCCO3)=C(NC(C)(C)C)N12 |&1:10,12,r,t:2,4,7,19|	Z8873684730	**1**{*329*}	Cl.COc1ccc(C)c(N)n1	**2**{*50*}	O=C[C@@H]1C[C@H]1C2OCCO2 |&1:2,4,r|	**3**{*18*}	CC(C)(C)[N+]#[C-]	2.6	3
963	**4**{*158*,*506*,*18*}	CC(C)(C)NC1=C(N=C2C=NC(=CN12)P(C)(C)=O)C1=CC2=C(C=C1)N=CC(Br)=C2 |c:9,11,22,24,27,30,t:5,7,20|	Z8855739253	**1**{*158*}	CP(=O)(C)c1cnc(N)cn1	**2**{*506*}	Brc1cnc2ccc(C=O)cc2c1	**3**{*18*}	CC(C)(C)[N+]#[C-]	3.4	3
964	**4**{*193*,*319*,*10*}	CNC(=O)C1=CN2C(NC3CCOC3)=C(N=C2C=C1)C1=C(C)C=C(OCC#N)C=C1 |c:14,16,19,22,31,t:4,25|	Z8878918349	**1**{*193*}	CNC(=O)c1ccc(N)nc1	**2**{*319*}	Cc1cc(OCC#N)ccc1C=O	**3**{*10*}	[C-]#[N+]C1CCOC1	2.8	3
965	**4**{*288*,*148*,*39*}	CCOCCNC1=C(N=C2C=C(NC(=O)OC(C)(C)C)C=CN12)C1=C(OC)N(C)N=C1C |c:20,25,31,t:6,8,10|	Z8810903014	**1**{*288*}	CC(C)(C)OC(=O)Nc1ccnc(N)c1	**2**{*148*}	COc1c(C=O)c(C)nn1C	**3**{*39*}	CCOCC[N+]#[C-]	3.0	2
966	**4**{*404*,*110*,*29*}	CCOC(=O)CCNC1=C(N=C2C=CC=C(OC(C)CC)N12)C1=CN=C(C=C1)P(C)(C)=O |c:12,26,28,t:8,10,14,24|	Z8878918631	**1**{*404*}	CCC(C)Oc1cccc(N)n1	**2**{*110*}	CP(=O)(C)c1ccc(C=O)cn1	**3**{*29*}	CCOC(=O)CC[N+]#[C-]	3.0	2
967	**4**{*334*,*363*,*11*}	CC1=NC(=CN1)C1=C(NCC2=CC(Br)=CC=C2)N2C=C3COCCC3=C(C#N)C2=N1 |c:3,7,14,16,32,t:1,11,20,27|	Z8837933173	**1**{*334*}	Nc1ncc2COCCc2c1C#N	**2**{*363*}	Cc1nc(C=O)c[nH]1	**3**{*11*}	Brc1cccc(C[N+]#[C-])c1	2.2	2

968	**4**{*227*,*135*,*47*}	CC(C)NC1=C(N=C2C=NC(Br)=C(Cl)N12)C1=C(C)N(C)N=C1C |c:8,17,22,t:4,6,11|	Z8810903047	**1**{*227*}	Nc1cnc(Br)c(Cl)n1	**2**{*135*}	Cc1nn(C)c(C)c1C=O	**3**{*47*}	CC(C)[N+]#[C-]	1.4	1
969	**4**{*19*,*227*,*37*}	COC(=O)C1CC(C1)NC1=C(N=C2N1C=CC=C2C#N)C1=NC=CS1 |c:12,16,18,25,t:10,23|	Z8878918900	**1**{*19*}	Nc1ncccc1C#N	**2**{*227*}	O=Cc1nccs1	**3**{*37*}	COC(=O)C1CC(C1)[N+]#[C-]	0.0	0
970	**4**{*376*,*507*,*12*}	COC1=NC(Cl)=CN2C(NCC3CCOC3)=C(N=C12)C1=CC2=CN=CN2C=C1 |c:5,16,26,30,t:2,18,22,24|	Z8878918603	**1**{*376*}	COc1nc(Cl)cnc1N	**2**{*507*}	O=Cc1ccn2cncc2c1	**3**{*12*}	[C-]#[N+]CC1CCOC1	0.0	0
971	**4**{*366*,*507*,*12*}	COC1=CC=CN2C(NCC3CCOC3)=C(N=C12)C1=CC2=CN=CN2C=C1 |c:4,15,25,29,t:2,17,21,23|	Z8878918592	**1**{*366*}	COc1cccnc1N	**2**{*507*}	O=Cc1ccn2cncc2c1	**3**{*12*}	[C-]#[N+]CC1CCOC1	0.0	0
972	**4**{*181*,*508*,*14*}	COCCNC1=C(N=C2N1C=CC1=C2N=CC=N1)C1=CC=C(CN2CCOCC2)C=C1 |c:7,11,13,16,18,33,t:5,21,23|	Z8878918550	**1**{*181*}	Nc1nccc2nccnc12	**2**{*508*}	O=Cc1ccc(CN2CCOCC2)cc1	**3**{*14*}	COCC[N+]#[C-]	0.0	0
973	**4**{*197*,*509*,*2*}	CC(C)CS(=O)(=O)C1=CN2C(C=C1)=NC(=C2NC1=CC=C(F)C=C1)C1=NN=CN1C |c:11,13,15,24,29,t:7,19,21,27|	Z8878918495	**1**{*197*}	CC(C)CS(=O)(=O)c1ccc(N)nc1	**2**{*509*}	Cn1cnnc1C=O	**3**{*2*}	Fc1ccc([N+]#[C-])cc1	0.0	0
974	**4**{*179*,*424*,*23*}	COC(=O)CCC(NC1=C(N=C2C=C(OC)C=CN12)C1=NN(C)C(=N1)C(F)(F)F)C(=O)OC |c:16,25,t:8,10,12,21|	Z8878918337	**1**{*179*}	COc1ccnc(N)c1	**2**{*424*}	Cn1nc(C=O)nc1C(F)(F)F	**3**{*23*}	COC(=O)CCC([N+]#[C-])C(=O)OC	0.0	0
975	**4**{*389*,*510*,*23*}	COC(=O)CCC(NC1=C(N=C2N1C=C(C)C=C2C)C1=C(C)SC2=CN=CN12)C(=O)OC |c:10,17,21,27,t:8,14,25|	Z8878918322	**1**{*389*}	Cc1cnc(N)c(C)c1	**2**{*510*}	Cc1sc2cncn2c1C=O	**3**{*23*}	COC(=O)CCC([N+]#[C-])C(=O)OC	0.0	0
976	**4**{*179*,*81*,*23*}	COC(=O)CCC(NC1=C(N=C2C=C(OC)C=CN12)C1=NC(=CC=C1)C(F)F)C(=O)OC |c:16,23,25,t:8,10,12,21|	Z8873684674	**1**{*179*}	COc1ccnc(N)c1	**2**{*81*}	FC(F)c1cccc(C=O)n1	**3**{*23*}	COC(=O)CCC([N+]#[C-])C(=O)OC	0.0	0
977	**4**{*213*,*191*,*31*}	CCN1C=NN=C1C1=C(NCCSC)N2C(=N1)C(F)=CC=C2OC |c:3,5,8,16,20,22|	Z8873685469	**1**{*213*}	COc1ccc(F)c(N)n1	**2**{*191*}	CCn1cnnc1C=O	**3**{*31*}	CSCC[N+]#[C-]	0.0	0
978	**4**{*405*,*511*,*1*}	CC(NC(=O)OC(C)(C)C)C1=C(NC2CC2)N2C=C(C=CC2=N1)N1CCCC1=O |c:10,18,20,23|	Z8829498661	**1**{*405*}	Nc1ccc(cn1)N2CCCC2=O	**2**{*511*}	CC(NC(=O)OC(C)(C)C)C=O	**3**{*1*}	[C-]#[N+]C1CC1	0.0	0
979	**4**{*221*,*512*,*14*}	COCCNC1=C(N=C2N1C(C)=CC=C2C#N)C1=NC=C(CN(C)C)S1 |c:7,12,14,t:5,19,21|	Z8829498460	**1**{*221*}	Cc1ccc(C#N)c(N)n1	**2**{*512*}	CN(C)Cc1cnc(C=O)s1	**3**{*14*}	COCC[N+]#[C-]	0.0	0
980	**4**{*251*,*324*,*70*}	CC(C)(C)OC(=O)N1CCC(C1)NC1=C(N=C2C=CC3=C(NC(=O)C=C3)N12)C1CC2(C1)CCCO2 |c:18,25,t:14,16,20|	Z8854581157	**1**{*251*}	Nc1ccc2ccc(=O)[nH]c2n1	**2**{*324*}	O=CC1CC2(C1)CCCO2	**3**{*70*}	CC(C)(C)OC(=O)N1CCC(C1)[N+]#[C-]	0.0	0

981	**4**{*198*,*464*,*14*}	COCCNC1=C(N=C2C=NC(C#C)=C(Cl)N12)C1=CC2=C(C=CC=C2)N2N=NN=C12 |c:9,23,25,29,t:5,7,13,19,21,31|	Z8835022894	**1**{*198*}	Nc1cnc(C#C)c(Cl)n1	**2**{*464*}	O=Cc1cc2ccccc2n3nnnc13	**3**{*14*}	COCC[N+]#[C-]	0.0	0
982	**4**{*406*,*356*,*31*}	CSCCNC1=C(N=C2C=CC=C(N12)C1=NNN=N1)C1=CN(CC(C)C)N=N1 |c:9,11,19,29,t:5,7,16,22|	Z8855739251	**1**{*406*}	Nc1cccc(n1)c2nn[nH]n2	**2**{*356*}	CC(C)Cn1cc(C=O)nn1	**3**{*31*}	CSCC[N+]#[C-]	0.0	0
983	**4**{*113*,*278*,*6*}	CCOC(=O)CCCNC1=C(N=C2N1C=CC=C2N1CCOCC1)C1=C(Br)N=NN1C |c:11,15,17,27,30,t:9|	Z8803896969	**1**{*113*}	Nc1ncccc1N2CCOCC2	**2**{*278*}	Cn1nnc(Br)c1C=O	**3**{*6*}	CCOC(=O)CCC[N+]#[C-]	0.0	0
984	**4**{*318*,*273*,*12*}	COC(=O)C1=CN2C(NCC3CCOC3)=C(N=C2C(OC)=N1)C1=C(Br)N(C)N=C1C |c:15,17,22,25,30,t:4|	Z8798985049	**1**{*318*}	COC(=O)c1cnc(N)c(OC)n1	**2**{*273*}	Cc1nn(C)c(Br)c1C=O	**3**{*12*}	[C-]#[N+]CC1CCOC1	0.0	0
985	**4**{*340*,*513*,*23*}	CCOC1=CC=CN2C(NC(CCC(=O)OC)C(=O)OC)=C(N=C12)C1=C(F)C=NC(F)=C1 |c:5,20,26,29,32,t:3,22|	Z8846492252	**1**{*340*}	CCOc1cccnc1N	**2**{*513*}	Fc1cc(C=O)c(F)cn1	**3**{*23*}	COC(=O)CCC([N+]#[C-])C(=O)OC	0.0	0
986	**4**{*179*,*445*,*23*}	COC(=O)CCC(NC1=C(N=C2C=C(OC)C=CN12)C1=NOC(=C1)C(C)(C)C)C(=O)OC |c:16,24,t:8,10,12,21|	Z8846491733	**1**{*179*}	COc1ccnc(N)c1	**2**{*445*}	CC(C)(C)c1cc(C=O)no1	**3**{*23*}	COC(=O)CCC([N+]#[C-])C(=O)OC	0.0	0
987	**4**{*211*,*47*,*41*}	COC1=CC=C(NC2=C(CCOCC=C)N=C3N2C=CC=C3C2=NN=NN2)C=C1 |c:7,15,19,21,26,30,t:2,4,24|	Z8837933183	**1**{*211*}	Nc1ncccc1c2nnn[nH]2	**2**{*47*}	C=CCOCCC=O	**3**{*41*}	COc1ccc([N+]#[C-])cc1	0.0	0
988	**4**{*262*,*139*,*12*}	CC(=O)NC1=CN2C(C=C1)=NC(=C2NCC1CCOC1)C1=C(C)N(CCO)N=C1C |c:8,10,12,23,30,t:4|	Z8837933180	**1**{*262*}	CC(=O)Nc1ccc(N)nc1	**2**{*139*}	Cc1nn(CCO)c(C)c1C=O	**3**{*12*}	[C-]#[N+]CC1CCOC1	0.0	0
989	**4**{*312*,*514*,*12*}	O=C1NC(=O)C2=C(N1)N1C(C=C2)=NC(=C1NCC1CCOC1)C1=CC2=C3N(CCCC3=C1)CCC2 |c:5,11,13,15,35,t:26,28|	Z8837933160	**1**{*312*}	Nc1ccc2c(=O)[nH]c(=O)[nH]c2n1	**2**{*514*}	O=Cc1cc2CCCN3CCCc(c1)c32	**3**{*12*}	[C-]#[N+]CC1CCOC1	0.0	0
990	**4**{*407*,*435*,*12*}	CCCOC1=NC=CN2C(NCC3CCOC3)=C(N=C12)C1=CC(=NO1)C(=O)OCC |c:6,17,25,t:4,19,23|	Z8837933152	**1**{*407*}	CCCOc1nccnc1N	**2**{*435*}	CCOC(=O)c1cc(C=O)on1	**3**{*12*}	[C-]#[N+]CC1CCOC1	0.0	0
991	**4**{*144*,*515*,*53*}	CNS(=O)(=O)C1=CN2C(NCCOCC3=CC=CC=C3)=C(N=C2C=C1)C1=NOC(CN(C)C)=C1 |c:16,18,20,22,25,35,t:5,14,28|	Z8837933145	**1**{*144*}	CNS(=O)(=O)c1ccc(N)nc1	**2**{*515*}	CN(C)Cc1cc(C=O)no1	**3**{*53*}	[C-]#[N+]CCOCc1ccccc1	0.0	0
992	**4**{*5*,*26*,*17*}	C(NC1=C(CCC2=CC=CC=C2)N=C2NN=NN12)C1CCOCC1 |c:2,8,10,16,t:6,13|	Z8711892497	**1**{*5*}	Nc1nnn[nH]1	**2**{*26*}	O=CCCc1ccccc1	**3**{*17*}	[C-]#[N+]CC1CCOCC1	0.0	0

993	**4**{*408*,*516*,*10*}	COC1=C(Br)C=CC2=NC(=C(NC3CCOC3)N12)C1=NN(C)C(=N1)C1CC1 |c:2,5,25,t:7,9,21|	Z8878918788	**1**{*408*}	COc1nc(N)ccc1Br	**2**{*516*}	Cn1nc(C=O)nc1C2CC2	**3**{*10*}	[C-]#[N+]C1CCOC1	0.0	0
994	**4**{*409*,*517*,*38*}	CN1C=NN=C1C1=CC2=NC(C3=CC(=CS3)S(N)(=O)=O)=C(NC3=CC4=C(OCO4)C=C3)N2C=C1 |c:2,4,14,32,37,t:7,9,12,21,24,26|	Z8878918632	**1**{*409*}	Cn1cnnc1c2ccnc(N)c2	**2**{*517*}	NS(=O)(=O)c1csc(C=O)c1	**3**{*38*}	[C-]#[N+]c1ccc2OCOc2c1	0.0	0
995	**4**{*410*,*518*,*62*}	COC(=O)C1(CCCC1)NC1=C(N=C2C=CC3=C(N=CC(Cl)=C3)N12)C1=C(C)N=CN1 |c:15,19,22,28,31,t:11,13,17|	Z8878918579	**1**{*410*}	Nc1ccc2cc(Cl)cnc2n1	**2**{*518*}	Cc1nc[nH]c1C=O	**3**{*62*}	COC(=O)C1(CCCC1)[N+]#[C-]	0.0	0
996	**4**{*408*,*519*,*12*}	COC1=C(Br)C=CC2=NC(=C(NCC3CCOC3)N12)C1=CN=C(S1)C(F)F |c:2,5,24,t:7,9,22|	Z8878918551	**1**{*408*}	COc1nc(N)ccc1Br	**2**{*519*}	FC(F)c1ncc(C=O)s1	**3**{*12*}	[C-]#[N+]CC1CCOC1	0.0	0
997	**4**{*251*,*271*,*71*}	COC1=CC(NC2=C(N=C3C=CC4=C(NC(=O)C=C4)N23)C2=NN(C)N=N2)=CC=C1 |c:10,17,27,29,31,t:2,6,8,12,23|	Z8878918383	**1**{*251*}	Nc1ccc2ccc(=O)[nH]c2n1	**2**{*271*}	Cn1nnc(C=O)n1	**3**{*71*}	COc1cccc([N+]#[C-])c1	0.0	0
998	**4**{*411*,*103*,*37*}	COC(=O)C1CC(C1)NC1=C(N=C2C=C(Br)C=C(F)N12)C1CCC2(COC2)OC1 |t:10,12,14,17|	Z8873684672	**1**{*411*}	Nc1cc(Br)cc(F)n1	**2**{*103*}	O=CC1CCC2(COC2)OC1	**3**{*37*}	COC(=O)C1CC(C1)[N+]#[C-]	0.0	0
999	**4**{*412*,*520*,*10*}	COC1=C(C=NN=C1)C1=C(NC2CCOC2)N2C=C(Br)C(CO)=CC2=N1 |c:4,6,9,24,27,t:2,19|	Z8873684643	**1**{*412*}	Nc1cc(CO)c(Br)cn1	**2**{*520*}	COc1cnncc1C=O	**3**{*10*}	[C-]#[N+]C1CCOC1	0.0	0
1000	**4**{*340*,*478*,*5*}	CCOC1=CC=CN2C(NCCCOC)=C(N=C12)C1=CN(C)C(=O)C2=C1C=CN=C2 |c:5,14,26,29,31,t:3,16,20|	Z8873685464	**1**{*340*}	CCOc1cccnc1N	**2**{*478*}	Cn1cc(C=O)c2ccncc2c1=O	**3**{*5*}	COCCC[N+]#[C-]	0.0	0
1001	**4**{*413*,*521*,*22*}	FC(F)(F)C1=CC2=NC(=C(NC3COC3)N2N=C1)C1=C(Br)C=NC(Br)=C1 |c:18,21,24,27,t:4,6,8|	Z8873685394	**1**{*413*}	Nc1cc(cnn1)C(F)(F)F	**2**{*521*}	Brc1cc(C=O)c(Br)cn1	**3**{*22*}	[C-]#[N+]C1COC1	0.0	0
1002	**4**{*329*,*191*,*7*}	CCN1C=NN=C1C1=C(NC2CCOCC2)N2C(=N1)C(C)=CC=C2OC |c:3,5,8,19,23,25|	Z8873685376	**1**{*329*}	Cl.COc1ccc(C)c(N)n1	**2**{*191*}	CCn1cnnc1C=O	**3**{*7*}	[C-]#[N+]C1CCOCC1	0.0	0
1003	**4**{*37*,*520*,*24*}	COC1=CC(CNC2=C(N=C3C=CC=C(C(F)F)N23)C2=C(OC)C=NN=C2)=CC=C1 |c:11,21,25,27,29,31,t:2,7,9,13|	Z8873684890	**1**{*37*}	Cl.Nc1cccc(n1)C(F)F	**2**{*520*}	COc1cnncc1C=O	**3**{*24*}	COc1cccc(C[N+]#[C-])c1	0.0	0
1004	**4**{*48*,*173*,*15*}	CCNC1=C(N=C2C=C(C=CN12)S(C)(=O)=O)C1=NC(=NN1C)C(F)(F)F |c:7,9,20,t:3,5,18|	Z8873684636	**1**{*48*}	CS(=O)(=O)c1ccnc(N)c1	**2**{*173*}	Cn1nc(nc1C=O)C(F)(F)F	**3**{*15*}	CC[N+]#[C-]	0.0	0

1005	**4**{*348*,*404*,*2*}	CC1=NN=C(N1)C1=C(NC2=CC=C(F)C=C2)N2C=C(F)N=CC2=N1 |c:3,7,15,22,25,t:1,10,12,19|	Z8873684580	**1**{*348*}	Nc1cnc(F)cn1	**2**{*404*}	Cc1nnc(C=O)[nH]1	**3**{*2*}	Fc1ccc([N+]#[C-])cc1	0.0	0
1006	**4**{*251*,*522*,*70*}	COC1=C(C=C(C)C=N1)C1=C(NC2CCN(C2)C(=O)OC(C)(C)C)N2C(C=CC3=C2NC(=O)C=C3)=N1 |c:7,10,28,30,36,38,t:2,4|	Z8873684826	**1**{*251*}	Nc1ccc2ccc(=O)[nH]c2n1	**2**{*522*}	COc1ncc(C)cc1C=O	**3**{*70*}	CC(C)(C)OC(=O)N1CCC(C1)[N+]#[C-]	0.0	0
1007	**4**{*169*,*523*,*13*}	CNC(=O)C1=NN2C(C=C1)=NC(=C2NCC1=CC=C(OC)C=C1)C1=C(F)C(OC)=CC=C1 |c:8,10,12,23,26,31,33,t:4,17,19|	Z8829498665	**1**{*169*}	CNC(=O)c1ccc(N)nn1	**2**{*523*}	COc1cccc(C=O)c1F	**3**{*13*}	COc1ccc(C[N+]#[C-])cc1	0.0	0
1008	**4**{*378*,*524*,*12*}	FC1=NC(F)=C(F)C(=C1)C1=C(NCC2CCOC2)N2C=CC=C(OCC3=CN=CC=C3)C2=N1 |c:7,10,21,29,31,35,t:1,4,23,27|	Z8829498545	**1**{*378*}	Nc1ncccc1OCc2cccnc2	**2**{*524*}	Fc1cc(C=O)c(F)c(F)n1	**3**{*12*}	[C-]#[N+]CC1CCOC1	0.0	0
1009	**4**{*223*,*382*,*50*}	COC(C)(C)CC1=C(NC2=CC3=C(OCCO3)C=C2)N2N=C(C=CC2=N1)C(N)=O |c:6,18,22,24,27,t:9,11|	Z8829498488	**1**{*223*}	NC(=O)c1ccc(N)nn1	**2**{*382*}	COC(C)(C)CC=O	**3**{*50*}	[C-]#[N+]c1ccc2OCCOc2c1	0.0	0
1010	**4**{*223*,*525*,*66*}	CC(NC1=C(N=C2C=CC(=NN12)C(N)=O)C1=C(Br)C=NC=C1F)C1=CC=CC=C1 |c:7,9,17,20,22,28,30,t:3,5,26|	Z8835022910	**1**{*223*}	NC(=O)c1ccc(N)nn1	**2**{*525*}	Fc1cncc(Br)c1C=O	**3**{*66*}	CC([N+]#[C-])c1ccccc1	0.0	0
1011	**4**{*69*,*526*,*21*}	CNC(=O)C1=CC2=NC(=C(NCC3CC3)N2C=C1)C1=CN2N=CC(C(N)=O)=C2N=C1 |c:18,24,29,32,t:4,6,8,21|	Z8835022893	**1**{*69*}	CNC(=O)c1ccnc(N)c1	**2**{*526*}	NC(=O)c1cnn2cc(C=O)cnc12	**3**{*21*}	[C-]#[N+]CC1CC1	0.0	0
1012	**4**{*231*,*408*,*33*}	COC1=C(CNC2=C(CCN3CCOC3=O)N=C3C=CC(=NN23)C(F)(F)F)C=CC=C1 |c:2,6,19,21,30,32,t:17|	Z8855739158	**1**{*231*}	Nc1ccc(nn1)C(F)(F)F	**2**{*408*}	O=CCCN1CCOC1=O	**3**{*33*}	COc1ccccc1C[N+]#[C-]	0.0	0
1013	**4**{*158*,*527*,*7*}	CC(C)(C)OC(=O)N1CC[C@@H](CC2=C(NC3CCOCC3)N3C=C(N=CC3=N2)P(C)(C)=O)C1 |c:12,23,25,28|	Z8846492320	**1**{*158*}	CP(=O)(C)c1cnc(N)cn1	**2**{*527*}	CC(C)(C)OC(=O)N1CC[C@@H](CC=O)C1	**3**{*7*}	[C-]#[N+]C1CCOCC1	0.0	0
1014	**4**{*383*,*528*,*1*}	CN1N=C(C)C(Cl)=C1C1=C(NC2CC2)N2N=C(C=CC2=N1)C1=NC=CC=C1 |c:6,9,17,19,22,27,29,t:2,25|	Z8837933181	**1**{*383*}	Nc1ccc(nn1)c2ccccn2	**2**{*528*}	Cc1nn(C)c(C=O)c1Cl	**3**{*1*}	[C-]#[N+]C1CC1	0.0	0
1015	**4**{*399*,*477*,*22*}	CC(C)OC1=NN2C(C=C1)=NC(C(NC(=O)OC(C)(C)C)C1COC1)=C2NC1COC1 |c:8,10,26,t:4|	Z8837933163	**1**{*399*}	CC(C)Oc1ccc(N)nn1	**2**{*477*}	CC(C)(C)OC(=O)NC(C=O)C1COC1	**3**{*22*}	[C-]#[N+]C1COC1	0.0	0

1016	**4**{*2* ,*15* ,*54* }	COC1=CC=C(C=C1)C1=C(NC2=CC=CC=C2)N2OC=CC2=N1 |c:4,6,9,14,16,21,24,t:2,12|	Z8711891895	**1**{*2* }	Nc1ccon1	**2**{*15* }	COc1ccc(C=O)cc1	**3**{*54* }	[C-]#[N+]c1ccccc1	0.0	0
1017	**4**{*181* ,*529* ,*6* }	CCOC(=O)CCCNC1=C(N=C2N1C=CC1=C2N=CC=N1)C1=CC(Cl)=C(C=C1)S(N)(=O)=O |c:11,15,17,20,22,28,30,t:9,25|	Z8878918889	**1**{*181* }	Nc1nccc2nccnc12	**2**{*529* }	NS(=O)(=O)c1ccc(C=O)cc1Cl	**3**{*6* }	CCOC(=O)CCC[N+]#[C-]	0.0	0
1018	**4**{*414* ,*281* ,*48* }	COC(=O)C1=NN=C(C=C1)C1=C(NC2CCCN(C2)C(=O)OC(C)(C)C)N2C=C(Cl)N=CC2=N1 |c:6,8,11,32,35,t:4,29|	Z8878918817	**1**{*414* }	Nc1cnc(Cl)cn1	**2**{*281* }	COC(=O)c1ccc(C=O)nn1	**3**{*48* }	CC(C)(C)OC(=O)N1CCCC(C1)[N+]#[C-]	0.0	0
1019	**4**{*388* ,*530* ,*39* }	CCOCCNC1=C(N=C2C=C3CCCOC3=CN12)C1CC2(CC(C2)OC)C1 |c:17,t:6,8,10|	Z8878918764	**1**{*388* }	Cl.Nc1cc2CCCOc2cn1	**2**{*530* }	COC1CC2(C1)CC(C2)C=O	**3**{*39* }	CCOCC[N+]#[C-]	0.0	0
1020	**4**{*415* ,*531* ,*10* }	CC1=NC=C(C=N1)C1=C(NC2CCOC2)N2C(=N1)C(CCl)=CC=C2Br |c:3,5,8,18,23,25,t:1|	Z8878918581	**1**{*415* }	Cl.Nc1nc(Br)ccc1CCl	**2**{*531* }	Cc1ncc(C=O)cn1	**3**{*10* }	[C-]#[N+]C1CCOC1	0.0	0
1021	**4**{*416* ,*532* ,*23* }	COC(=O)CCC(NC1=C(N=C2C=C(CCl)C=CN12)C1=C(OC)C(OC)=NC=N1)C(=O)OC |c:16,21,27,29,t:8,10,12|	Z8873684772	**1**{*416* }	Cl.Nc1cc(CCl)ccn1	**2**{*532* }	COc1ncnc(C=O)c1OC	**3**{*23* }	COC(=O)CCC([N+]#[C-])C(=O)OC	0.0	0
1022	**4**{*83* ,*464* ,*7* }	CP(C)(=O)C1=CN2C(C=C1)=NC(=C2NC1CCOCC1)C1=CC2=C(C=CC=C2)N2N=NN=C12 |c:8,10,12,27,29,33,t:4,23,25,35|	Z8873684678	**1**{*83* }	CP(=O)(C)c1ccc(N)nc1	**2**{*464* }	O=Cc1cc2ccccc2n3nnnc13	**3**{*7* }	[C-]#[N+]C1CCOCC1	0.0	0
1023	**4**{*409* ,*533* ,*38* }	CCC1=CC(=NO1)C1=C(NC2=CC3=C(OCO3)C=C2)N2C=CC(=CC2=N1)C1=NN=CN1C |c:4,8,19,23,25,28,33,t:2,11,13,31|	Z8873685397	**1**{*409* }	Cn1cnnc1c2ccnc(N)c2	**2**{*533* }	CCc1cc(C=O)no1	**3**{*38* }	[C-]#[N+]c1ccc2OCOc2c1	0.0	0
1024	**4**{*329* ,*534* ,*7* }	COC1=CC=C(C)C2=NC(C3=NN(C=C3)C(C)C)=C(NC3CCOCC3)N12 |c:13,t:2,4,7,10,18|	Z8873685344	**1**{*329* }	Cl.COc1ccc(C)c(N)n1	**2**{*534* }	CC(C)n1ccc(C=O)n1	**3**{*7* }	[C-]#[N+]C1CCOCC1	0.0	0
1025	**4**{*179* ,*535* ,*5* }	COCCCNC1=C(N=C2C=C(OC)C=CN12)C1=NOC(=C1)C(C)(C)O |c:14,22,t:6,8,10,19|	Z8873685310	**1**{*179* }	COc1ccnc(N)c1	**2**{*535* }	CC(C)(O)c1cc(C=O)no1	**3**{*5* }	COCCC[N+]#[C-]	0.0	0
1026	**4**{*89* ,*271* ,*71* }	COC1=CC(NC2=C(N=C3C=CC4=C(NN=C4)N23)C2=NN(C)N=N2)=CC=C1 |c:10,15,25,27,29,t:2,6,8,12,21|	Z8873684617	**1**{*89* }	Nc1ccc2cn[nH]c2n1	**2**{*271* }	Cn1nnc(C=O)n1	**3**{*71* }	COc1cccc([N+]#[C-])c1	0.0	0
1027	**4**{*408* ,*536* ,*12* }	COC1=C(Br)C=CC2=NC(C3=CN=C(O3)C3CCOCC3)=C(NCC3CCOC3)N12 |c:2,5,12,t:7,10,22|	Z8873684800	**1**{*408* }	COc1nc(N)ccc1Br	**2**{*536* }	O=Cc1cnc(o1)C2CCOCC2	**3**{*12* }	[C-]#[N+]CC1CCOC1	0.0	0

1028	**4**{*68*,*537*,*1*}	COC(=O)C1=CN=C(C=C1)C1=C(NC2CC2)N2C=CC(=CC2=N1)C#C |c:6,8,11,19,21,24,t:4|	Z8829498778	**1**{*68*}	Nc1cc(C#C)ccn1	**2**{*537*}	COC(=O)c1ccc(C=O)nc1	**3**{*1*}	[C-]#[N+]C1CC1	0.0	0
1029	**4**{*151*,*17*,*9*}	CCOC1=CN=C(N=C1)C1=C(NC)N2C(C=NC=C2Cl)=N1 |c:5,7,10,16,18,21,t:3|	Z8829498592	**1**{*151*}	Nc1cncc(Cl)n1	**2**{*17*}	CCOc1cnc(C=O)nc1	**3**{*9*}	C[N+]#[C-]	0.0	0
1030	**4**{*181*,*538*,*11*}	CN1C=NC=C1C1=C(NCC2=CC(Br)=CC=C2)N2C=CC3=C(N=CC=N3)C2=N1 |c:2,4,7,14,16,20,24,26,30,t:11,22|	Z8854581173	**1**{*181*}	Nc1nccc2nccnc12	**2**{*538*}	Cn1cncc1C=O	**3**{*11*}	Brc1cccc(C[N+]#[C-])c1	0.0	0
1031	**4**{*334*,*98*,*13*}	COC1=CC=C(CNC2=C(N=C3N2C=C2COCCC2=C3C#N)C2=NC=C(OC)N=C2)C=C1 |c:10,21,32,35,t:2,4,8,14,26,28|	Z8835022915	**1**{*334*}	Nc1ncc2COCCc2c1C#N	**2**{*98*}	COc1cnc(C=O)cn1	**3**{*13*}	COc1ccc(C[N+]#[C-])cc1	0.0	0
1032	**4**{*223*,*539*,*50*}	NC(=O)C1=NN2C(C=C1)=NC([C@@H]1CCCC[C@H]1C(F)(F)F)=C2NC1=CC2=C(OCCO2)C=C1 |&1:11,16,r,c:7,9,22,35,t:3,26,28|	Z8835022898	**1**{*223*}	NC(=O)c1ccc(N)nn1	**2**{*539*}	FC(F)(F)[C@@H]1CCCC[C@H]1C=O |&1:4,9,r|	**3**{*50*}	[C-]#[N+]c1ccc2OCCOc2c1	0.0	0
1033	**4**{*223*,*540*,*66*}	CC(NC1=C(N=C2C=CC(=NN12)C(N)=O)C1CCC2CN(CC12)C(=O)OC(C)(C)C)C1=CC=CC=C1 |c:7,9,36,38,t:3,5,34|	Z8835022885	**1**{*223*}	NC(=O)c1ccc(N)nn1	**2**{*540*}	CC(C)(C)OC(=O)N1CC2CCC(C=O)C2C1	**3**{*66*}	CC([N+]#[C-])c1ccccc1	0.0	0
1034	**4**{*417*,*515*,*49*}	CN(C)CC1=CC(=NO1)C1=C(NC2CC(F)(F)C2)N2C=C(Cl)C(=CC2=N1)C(F)(F)F |c:6,10,24,27,t:4,21|	Z8849597801	**1**{*417*}	Cl.Nc1cc(c(Cl)cn1)C(F)(F)F	**2**{*515*}	CN(C)Cc1cc(C=O)no1	**3**{*49*}	FC1(F)CC(C1)[N+]#[C-]	0.0	0
1035	**4**{*406*,*541*,*35*}	CSCCCNC1=C(N=C2C=CC=C(N12)C1=NNN=N1)C1=C(C)N(N=C1C)C(C)C |c:10,12,20,23,27,t:6,8,17|	Z8855739120	**1**{*406*}	Nc1cccc(n1)c2nn[nH]n2	**2**{*541*}	CC(C)n1nc(C)c(C=O)c1C	**3**{*35*}	CSCCC[N+]#[C-]	0.0	0
1036	**4**{*128*,*200*,*23*}	COCCN(C)C1=CN2C(C=C1)=NC(CCCC#CC)=C2NC(CCC(=O)OC)C(=O)OC |c:10,12,20,t:6|	Z8808559870	**1**{*128*}	COCCN(C)c1ccc(N)nc1	**2**{*200*}	CC#CCCCC=O	**3**{*23*}	COC(=O)CCC([N+]#[C-])C(=O)OC	0.0	0
1037	**4**{*223*,*194*,*29*}	CCOC(=O)CCNC1=C(N=C2C=CC(=NN12)C(N)=O)C1=CC=C(N1C)C(=O)OC |c:12,14,24,t:8,10,22|	Z8798985052	**1**{*223*}	NC(=O)c1ccc(N)nn1	**2**{*194*}	COC(=O)c1ccc(C=O)n1C	**3**{*29*}	CCOC(=O)CC[N+]#[C-]	0.0	0
1038	**4**{*73*,*79*,*9*}	CNC1=C(N=C2C=CC3=NC=CN3N12)C1=NC=CO1 |c:6,10,19,t:2,4,8,17|	Z8846491979	**1**{*73*}	Nc1ccc2nccn2n1	**2**{*79*}	O=Cc1ncco1	**3**{*9*}	C[N+]#[C-]	0.0	0
1039	**4**{*312*,*522*,*38*}	COC1=C(C=C(C)C=N1)C1=C(NC2=CC3=C(OCO3)C=C2)N2C(C=CC3=C2NC(=O)NC3=O)=N1 |c:7,10,21,26,28,37,t:2,4,13,15|	Z8837933184	**1**{*312*}	Nc1ccc2c(=O)[nH]c(=O)[nH]c2n1	**2**{*522*}	COc1ncc(C)cc1C=O	**3**{*38*}	[C-]#[N+]c1ccc2OCOc2c1	0.0	0

1040	**4**{*83*,*542*,*18*}	CC(C)(C)NC1=C(N=C2C=CC(=CN12)P(C)(C)=O)C1N(CC11CCC1)C(=O)OC(C)(C)C |c:9,11,t:5,7|	Z8837933158	**1**{*83*}	CP(=O)(C)c1ccc(N)nc1	**2**{*542*}	CC(C)(C)OC(=O)N1CC2(CCC2)C1C=O	**3**{*18*}	CC(C)(C)[N+]#[C-]	0.0	0
1041	**4**{*271*,*8*,*62*}	COCC1=NOC=C1C1=C(NC2(CCCC2)C(=O)OC)N2C(C=CC(C(=O)OC)=C2F)=N1 |c:6,9,24,30,33,t:3|	Z8837933155	**1**{*271*}	COC(=O)c1ccc(N)nc1F	**2**{*8*}	COCc1nocc1C=O	**3**{*62*}	COC(=O)C1(CCCC1)[N+]#[C-]	0.0	0
1042	**4**{*181*,*340*,*14*}	CCN1C=NC(=N1)C1=C(NCCOC)N2C=CC3=C(N=CC=N3)C2=N1 |c:3,5,8,16,20,22,26,t:18|	Z8855619535	**1**{*181*}	Nc1nccc2nccnc12	**2**{*340*}	CCn1cnc(C=O)n1	**3**{*14*}	COCC[N+]#[C-]	0.0	0
1043	**4**{*111*,*543*,*15*}	CCNC1=C(N=C2N1C=C(C=C2C)C(=O)OC)C(=O)OCC |c:5,9,11,t:3|	Z8878918801	**1**{*111*}	COC(=O)c1cnc(N)c(C)c1	**2**{*543*}	CCOC(=O)C=O	**3**{*15*}	CC[N+]#[C-]	0.0	0
1044	**4**{*176*,*512*,*14*}	COCCNC1=C(N=C2N1C=CN=C2N1CCCC1)C1=NC=C(CN(C)C)S1 |c:7,11,13,t:5,22,24|	Z8878918774	**1**{*176*}	Nc1nccnc1N2CCCC2	**2**{*512*}	CN(C)Cc1cnc(C=O)s1	**3**{*14*}	COCC[N+]#[C-]	0.0	0
1045	**4**{*188*,*329*,*71*}	COC(=O)C1=CN=CC2=NC(=C(NC3=CC(OC)=CC=C3)N12)C1=C(C)N(CCCl)N=C1C |c:6,17,19,25,32,t:4,8,10,13|	Z8878918673	**1**{*188*}	COC(=O)c1cncc(N)n1	**2**{*329*}	Cc1nn(CCCl)c(C)c1C=O	**3**{*71*}	COc1cccc([N+]#[C-])c1	0.0	0
1046	**4**{*193*,*544*,*72*}	CNC(=O)C1=CN2C(C=C1)=NC(=C2NC1=C(C)C=C(F)C=C1)C1=NN=C2CCCN12 |c:8,10,12,16,22,t:4,19,25,27|	Z8878918671	**1**{*193*}	CNC(=O)c1ccc(N)nc1	**2**{*544*}	O=Cc1nnc2CCCn12	**3**{*72*}	Cc1cc(F)ccc1[N+]#[C-]	0.0	0
1047	**4**{*240*,*545*,*32*}	COCCN1N=C(C)C(=C1C)C1=C(NC2CCC2)N2C=C(C=CC2=N1)C1=NNN=N1 |c:8,12,21,23,26,32,t:5,29|	Z8878918647	**1**{*240*}	Nc1ccc(cn1)c2nn[nH]n2	**2**{*545*}	COCCn1nc(C)c(C=O)c1C	**3**{*32*}	[C-]#[N+]C1CCC1	0.0	0
1048	**4**{*251*,*546*,*62*}	COC(=O)C1(CCCC1)NC1=C(N=C2C=CC3=C(NC(=O)C=C3)N12)C1=C(C)N(C)C(C)=C1C |c:15,22,28,34,t:11,13,17|	Z8878918476	**1**{*251*}	Nc1ccc2ccc(=O)[nH]c2n1	**2**{*546*}	Cc1c(C)n(C)c(C)c1C=O	**3**{*62*}	COC(=O)C1(CCCC1)[N+]#[C-]	0.0	0
1049	**4**{*413*,*547*,*22*}	FC(F)(F)C1=NC(Br)=C(S1)C1=C(NC2COC2)N2N=CC(=CC2=N1)C(F)(F)F |c:7,11,20,22,25,t:4|	Z8873684720	**1**{*413*}	Nc1cc(cnn1)C(F)(F)F	**2**{*547*}	FC(F)(F)c1nc(Br)c(C=O)s1	**3**{*22*}	[C-]#[N+]C1COC1	0.0	0
1050	**4**{*404*,*320*,*29*}	CCOC(=O)CCNC1=C(N=C2C=CC=C(OC(C)CC)N12)C1=NN(C)C=N1 |c:12,28,t:8,10,14,24|	Z8873684653	**1**{*404*}	CCC(C)Oc1cccc(N)n1	**2**{*320*}	Cn1cnc(C=O)n1	**3**{*29*}	CCOC(=O)CC[N+]#[C-]	0.0	0
1051	**4**{*418*,*293*,*23*}	COC(=O)CCC(NC1=C(N=C2C=C(CBr)C=CN12)C1=C(F)N(C)N=C1)C(=O)OC |c:16,21,26,t:8,10,12|	Z8829498702	**1**{*418*}	Br.Nc1cc(CBr)ccn1	**2**{*293*}	Cn1ncc(C=O)c1F	**3**{*23*}	COC(=O)CCC([N+]#[C-])C(=O)OC	0.0	0

1052	**4**{*283*,*548*,*2*}	COC(=O)C1=CC2=C(N=C1)N1C(C=C2)=NC(CCC2=NC=CN=C2)=C1NC1=CC=C(F)C=C1 |c:6,8,13,15,22,24,26,35,t:4,20,30,32|	Z8829498469	**1**{*283*}	COC(=O)c1cnc2nc(N)ccc2c1	**2**{*548*}	O=CCCc1cnccn1	**3**{*2*}	Fc1ccc([N+]#[C-])cc1	0.0	0
1053	**4**{*107*,*520*,*21*}	COC1=C(C=NN=C1)C1=C(NCC2CC2)N2C=C(OCCO)C=CC2=N1 |c:4,6,9,24,27,t:2,18|	Z8854581165	**1**{*107*}	Nc1ccc(OCCO)cn1	**2**{*520*}	COc1cnncc1C=O	**3**{*21*}	[C-]#[N+]CC1CC1	0.0	0
1054	**4**{*419*,*425*,*21*}	CS(=O)(=O)N1CCCC(C1)C1=C(NCC2CC2)N2C(C=CC(C#C)=C2F)=N1 |c:11,21,25,28|	Z8854581152	**1**{*419*}	Nc1ccc(C#C)c(F)n1	**2**{*425*}	CS(=O)(=O)N1CCCC(C1)C=O	**3**{*21*}	[C-]#[N+]CC1CC1	0.0	0
1055	**4**{*62*,*278*,*6*}	CCOC(=O)CCCNC1=C(N=C2C=C(CO)C=CN12)C1=C(Br)N=NN1C |c:17,22,25,t:9,11,13|	Z8835022869	**1**{*62*}	Nc1cc(CO)ccn1	**2**{*278*}	Cn1nnc(Br)c1C=O	**3**{*6*}	CCOC(=O)CCC[N+]#[C-]	0.0	0
1056	**4**{*262*,*323*,*12*}	CC(=O)NC1=CN2C(NCC3CCOC3)=C(N=C2C=C1)[C@@H]1C[C@@H](C1)NC(=O)OC(C)(C)C |r,c:15,17,20,t:4|	Z8837933177	**1**{*262*}	CC(=O)Nc1ccc(N)nc1	**2**{*323*}	CC(C)(C)OC(=O)N[C@H]1C[C@H](C1)C=O	**3**{*12*}	[C-]#[N+]CC1CCOC1	0.0	0
1057	**4**{*364*,*478*,*5*}	COCCCNC1=C(N=C2C=CC(=CN12)N1CCNC(=O)C1)C1=CN(C)C(=O)C2=C1C=CN=C2 |c:10,12,31,34,36,t:6,8,25|	Z8837933150	**1**{*364*}	Nc1ccc(cn1)N2CCNC(=O)C2	**2**{*478*}	Cn1cc(C=O)c2ccncc2c1=O	**3**{*5*}	COCCC[N+]#[C-]	0.0	0
1058	**4**{*409*,*549*,*73*}	COC(=O)C1=NC=C(C2=C(NC3=C(OC)C=C(OC)C=C3)N3C=CC(=CC3=N2)C2=NN=CN2C)C(C)=C1 |c:8,11,19,23,25,28,33,39,t:4,6,15,31|	Z8837933147	**1**{*409*}	Cn1cnnc1c2ccnc(N)c2	**2**{*549*}	COC(=O)c1cc(C)c(C=O)cn1	**3**{*73*}	COc1ccc([N+]#[C-])c(OC)c1	0.0	0
1059	**4**{*179*,*550*,*1*}	COC1=CC2=NC(=C(NC3CC3)N2C=C1)C1=NC(Br)=NN1C |c:15,21,t:2,4,6,18|	Z8855619768	**1**{*179*}	COc1ccnc(N)c1	**2**{*550*}	Cn1nc(Br)nc1C=O	**3**{*1*}	[C-]#[N+]C1CC1	0.0	0
1060	**4**{*420*,*324*,*70*}	CC(C)(C)OC(=O)N1CCC(C1)NC1=C(N=C2C=CC3=C(NC=N3)N12)C1CC2(C1)CCCO2 |c:18,23,t:14,16,20|	Z8878918829	**1**{*420*}	Cl.Cl.Nc1ccc2nc[nH]c2n1	**2**{*324*}	O=CC1CC2(C1)CCCO2	**3**{*70*}	CC(C)(C)OC(=O)N1CCC(C1)[N+]#[C-]	0.0	0
1061	**4**{*376*,*435*,*12*}	CCOC(=O)C1=NOC(=C1)C1=C(NCC2CCOC2)N2C=C(Cl)N=C(OC)C2=N1 |c:8,11,30,t:5,22,25|	Z8878918770	**1**{*376*}	COc1nc(Cl)cnc1N	**2**{*435*}	CCOC(=O)c1cc(C=O)on1	**3**{*12*}	[C-]#[N+]CC1COC1	0.0	0
1062	**4**{*141*,*551*,*35*}	CSCCCNC1=C(N=C2C=C(NC(C)=O)C=CN12)C1CC1(Br)Br |c:16,t:6,8,10|	Z8878918596	**1**{*141*}	CC(=O)Nc1ccnc(N)c1	**2**{*551*}	BrC1(Br)CC1C=O	**3**{*35*}	CSCCC[N+]#[C-]	0.0	0

1063	**4**{*187 ,507 ,12* }	ClC1=CN2C(C=C1C#N)=NC(=C2NCC1CCOC1)C1=CC2=CN=CN2C=C1 |c:5,9,11,26,30,t:1,22,24|	Z8878918538	**1**{*187* }	Nc1cc(C#N)c(Cl)cn1	**2**{*507* }	O=Cc1ccn2cncc2c1	**3**{*12* }	[C-]#[N+]CC1CCOC1	0.0	0
1064	**4**{*401 ,341 ,39* }	CCOCCNC1=C(N=C2C=CC3=C(N=CC=C3Cl)N12)C1=CN=C(S1)C(C)O |c:10,14,16,25,t:6,8,12,23|	Z8878918509	**1**{*401* }	Nc1ccc2c(Cl)ccnc2n1	**2**{*341* }	CC(O)c1ncc(C=O)s1	**3**{*39* }	CCOCC[N+]#[C-]	0.0	0
1065	**4**{*19 ,552 ,37* }	COC(=O)C1CC(C1)NC1=C(N=C2N1C=CC=C2C#N)C1=CN=C(C=C1)C#C |c:12,16,18,25,27,t:10,23|	Z8878918485	**1**{*19* }	Nc1ncccc1C#N	**2**{*552* }	O=Cc1ccc(C#C)nc1	**3**{*37* }	COC(=O)C1CC(C1)[N+]#[C-]	0.0	0
1066	**4**{*251 ,217 ,62* }	COC(=O)C1(CCCC1)NC1=C(N=C2C=CC3=C(NC(=O)C=C3)N12)C1=CC(=O)N(C)C=C1 |c:15,22,34,t:11,13,17,28|	Z8878918355	**1**{*251* }	Nc1ccc2ccc(=O)[nH]c2n1	**2**{*217* }	Cn1ccc(C=O)cc1=O	**3**{*62* }	COC(=O)C1(CCCC1)[N+]#[C-]	0.0	0
1067	**4**{*411 ,55 ,37* }	COC(=O)C1CC(C1)NC1=C(N=C2C=C(Br)C=C(F)N12)C1=CC(=NC=C1)P(C)(C)=O |c:25,27,t:10,12,14,17,23|	Z8873684748	**1**{*411* }	Nc1cc(Br)cc(F)n1	**2**{*55* }	CP(=O)(C)c1cc(C=O)ccn1	**3**{*37* }	COC(=O)C1CC(C1)[N+]#[C-]	0.0	0
1068	**4**{*72 ,553 ,12* }	COC1=NC(Br)=CN2C(NCC3CCOC3)=C(N=C12)C1=CSC(=C1)C1OCCO1 |c:5,16,25,t:2,18,22|	Z8873684745	**1**{*72* }	COc1nc(Br)cnc1N	**2**{*553* }	O=Cc1csc(c1)C2OCCO2	**3**{*12* }	[C-]#[N+]CC1CCOC1	0.0	0
1069	**4**{*421 ,543 ,15* }	CCNC1=C(N=C2C=CC(C(=O)OC)=C(Cl)N12)C(=O)OCC |c:7,t:3,5,13|	Z8873684725	**1**{*421* }	COC(=O)c1ccc(N)nc1Cl	**2**{*543* }	CCOC(=O)C=O	**3**{*15* }	CC[N+]#[C-]	0.0	0
1070	**4**{*188 ,492 ,71* }	COC(=O)C1=CN=CC2=NC(=C(NC3=CC(OC)=CC=C3)N12)C1=C(C)N=C(C)C(C)=N1 |c:6,17,19,25,32,t:4,8,10,13,28|	Z8873684709	**1**{*188* }	COC(=O)c1cncc(N)n1	**2**{*492* }	Cc1nc(C)c(C=O)nc1C	**3**{*71* }	COc1cccc([N+]#[C-])c1	0.0	0
1071	**4**{*251 ,372 ,71* }	COC1=CC(NC2=C(N=C3C=CC4=C(NC(=O)C=C4)N23)C2=NN(C)N=C2C)=CC=C1 |c:10,17,27,30,32,t:2,6,8,12,23|	Z8873685367	**1**{*251* }	Nc1ccc2ccc(=O)[nH]c2n1	**2**{*372* }	Cc1nn(C)nc1C=O	**3**{*71* }	COc1cccc([N+]#[C-])c1	0.0	0
1072	**4**{*372 ,526 ,46* }	CC(=C)CNC1=C(N=C2N1C=CC=C2OCC1=CC=NC=C1)C1=CN2N=CC(C(N)=O)=C2N=C1 |c:7,11,13,20,22,28,33,36,t:5,18,25|	Z8873684842	**1**{*372* }	Nc1ncccc1OCc2ccncc2	**2**{*526* }	NC(=O)c1cnn2cc(C=O)cnc12	**3**{*46* }	CC(=C)C[N+]#[C-]	0.0	0
1073	**4**{*119 ,544 ,72* }	CC1=C(NC2=C(N=C3C=CC(=CN23)C#N)C2=NN=C3CCCN23)C=CC(F)=C1 |c:1,8,10,27,30,t:4,6,17,19|	Z8873684824	**1**{*119* }	Nc1ccc(C#N)cn1	**2**{*544* }	O=Cc1nnc2CCCn12	**3**{*72* }	Cc1cc(F)ccc1[N+]#[C-]	0.0	0
1074	**4**{*329 ,92 ,31* }	COC1=CC=C(C)C2=NC(C3=CC(C)=NO3)=C(NCCSC)N12 |c:13,t:2,4,7,10,16|	Z8873684801	**1**{*329* }	Cl.COc1ccc(C)c(N)n1	**2**{*92* }	Cc1cc(C=O)on1	**3**{*31* }	CSCC[N+]#[C-]	0.0	0
1075	**4**{*213 ,340 ,31* }	CCN1C=NC(=N1)C1=C(NCCSC)N2C(=N1)C(F)=CC=C2OC |c:3,5,8,16,20,22|	Z8829498705	**1**{*213* }	COc1ccc(F)c(N)n1	**2**{*340* }	CCn1cnc(C=O)n1	**3**{*31* }	CSCC[N+]#[C-]	0.0	0

1076	**4**{*128*,*554*,*1*}	COCCN(C)C1=CN2C(C=C1)=NC(=C2NC1CC1)C1=C(CO)C=CC=C1 |c:10,12,14,22,26,28,t:6|	Z8829498587	**1**{*128*}	COCCN(C)c1ccc(N)nc1	**2**{*554*}	OCc1ccccc1C=O	**3**{*1*}	[C-]#[N+]C1CC1	0.0	0
1077	**4**{*158*,*555*,*7*}	CC1=NN(C(C)=C1C1=C(NC2CCOCC2)N2C=C(N=CC2=N1)P(C)(C)=O)C1=NC=CC=C1 |c:5,8,19,21,24,33,35,t:1,31|	Z8854581148	**1**{*158*}	CP(=O)(C)c1cnc(N)cn1	**2**{*555*}	Cc1nn(c(C)c1C=O)c2ccccn2	**3**{*7*}	[C-]#[N+]C1CCOCC1	0.0	0
1078	**4**{*234*,*520*,*12*}	COC1=C(C=NN=C1)C1=C(NCC2CCOC2)N2C=CN=C(OC3CCC3)C2=N1 |c:4,6,9,20,31,t:2,22|	Z8849597838	**1**{*234*}	Nc1nccnc1OC2CCC2	**2**{*520*}	COc1cnncc1C=O	**3**{*12*}	[C-]#[N+]CC1CCOC1	0.0	0
1079	**4**{*158*,*556*,*35*}	COC1=CC2=C(NN=C2C2=C(NCCCSC)N3C=C(N=CC3=N2)P(C)(C)=O)C=C1 |c:7,10,19,21,24,31,t:2,4|	Z8849597799	**1**{*158*}	CP(=O)(C)c1cnc(N)cn1	**2**{*556*}	COc1ccc2[nH]nc(C=O)c2c1	**3**{*35*}	CSCCC[N+]#[C-]	0.0	0
1080	**4**{*419*,*164*,*46*}	CN1N=C(C2=C1CCOC2)C1=C(NCC(C)=C)N2C(C=CC(C#C)=C2F)=N1 |c:2,4,12,21,25,28|	Z8855739188	**1**{*419*}	Nc1ccc(C#C)c(F)n1	**2**{*164*}	Cn1nc(C=O)c2COCCc21	**3**{*46*}	CC(=C)C[N+]#[C-]	0.0	0
1081	**4**{*35*,*129*,*9*}	CNC1=C(N=C2C=CC(C)=CN12)C1=CC=CC2=NC=NN12 |c:6,9,16,20,t:2,4,14,18|	Z8798985036	**1**{*35*}	Cc1ccc(N)nc1	**2**{*129*}	O=Cc1cccc2ncnn12	**3**{*9*}	C[N+]#[C-]	0.0	0
1082	**4**{*397*,*532*,*1*}	COC1=C(OC)C(=NC=N1)C1=C(NC2CC2)N2C=CC(C)=C(C)C2=N1 |c:2,6,8,11,19,26,t:22|	Z8846492153	**1**{*397*}	Cl.Cc1ccnc(N)c1C	**2**{*532*}	COc1ncnc(C=O)c1OC	**3**{*1*}	[C-]#[N+]C1CC1	0.0	0
1083	**4**{*84*,*173*,*32*}	CC(C)C1=NC=CN2C(NC3CCC3)=C(N=C12)C1=NC(=NN1C)C(F)(F)F |c:5,14,22,t:3,16,20|	Z8846491988	**1**{*84*}	CC(C)c1nccnc1N	**2**{*173*}	Cn1nc(nc1C=O)C(F)(F)F	**3**{*32*}	[C-]#[N+]C1CCC1	0.0	0
1084	**4**{*422*,*480*,*29*}	CCOC(=O)CCNC1=C(N=C2N1C(F)=CC=C2C#C)C1=CC2=C(CCOC2)N=C1 |c:10,15,17,31,t:8,22,24|	Z8846491751	**1**{*422*}	Nc1nc(F)ccc1C#C	**2**{*480*}	O=Cc1cnc2CCOCc2c1	**3**{*29*}	CCOC(=O)CC[N+]#[C-]	0.0	0
1085	**4**{*223*,*557*,*50*}	COC1=NC=C(F)C(=C1)C1=C(NC2=CC3=C(OCCO3)C=C2)N2N=C(C=CC2=N1)C(N)=O |c:7,10,22,26,28,31,t:2,4,13,15|	Z8837933142	**1**{*223*}	NC(=O)c1ccc(N)nn1	**2**{*557*}	COc1cc(C=O)c(F)cn1	**3**{*50*}	[C-]#[N+]c1ccc2OCCOc2c1	0.0	0
1086	**4**{*169*,*558*,*9*}	CNC(=O)C1=NN2C(C=C1)=NC(=C2NC)C1=CC(Cl)=NC(C)=C1 |c:8,10,12,20,23,t:4,17|	Z8837933138	**1**{*169*}	CNC(=O)c1ccc(N)nn1	**2**{*558*}	Cc1cc(C=O)cc(Cl)n1	**3**{*9*}	C[N+]#[C-]	0.0	0
1087	**4**{*251*,*108*,*62*}	COC(=O)C1(CCCC1)NC1=C(N=C2C=CC3=C(NC(=O)C=C3)N12)C1=CN=C(N=C1)N1CCOCC1 |c:15,22,30,32,t:11,13,17,28|	Z8878918895	**1**{*251*}	Nc1ccc2ccc(=O)[nH]c2n1	**2**{*108*}	O=Cc1cnc(nc1)N2CCOCC2	**3**{*62*}	COC(=O)C1(CCCC1)[N+]#[C-]	0.0	0

1088	**4**{*416,559,23*}	COC(=O)CCC(NC1=C(CC2CCOC(C)(C)C2)N=C2C=C(CCl)C=CN12)C(=O)OC |c:8,26,t:20,22|	Z8878918846	**1**{*416*}	Cl.Nc1cc(CCl)ccn1	**2**{*559*}	CC1(C)CC(CC=O)CCO1	**3**{*23*}	COC(=O)CCC([N+]#[C-])C(=O)OC	0.0	0
1089	**4**{*181,560,14*}	COCCNC1=C(N=C2N1C=CC1=C2N=CC=N1)C1=NC2=C(CCCC2)S1 |c:7,11,13,16,18,t:5,21,23|	Z8878918501	**1**{*181*}	Nc1nccc2nccnc12	**2**{*560*}	O=Cc1nc2CCCCc2s1	**3**{*14*}	COCC[N+]#[C-]	0.0	0
1090	**4**{*181,450,6*}	CCOC(=O)CCCNC1=C(N=C2N1C=CC1=C2N=CC=N1)C1=CC(=CC=C1)S(=O)(=O)N(C)C |c:11,15,17,20,22,27,29,t:9,25|	Z8878918356	**1**{*181*}	Nc1nccc2nccnc12	**2**{*450*}	CN(C)S(=O)(=O)c1cccc(C=O)c1	**3**{*6*}	CCOC(=O)CCC[N+]#[C-]	0.0	0
1091	**4**{*423,123,22*}	CC(C)OC1=CC=CC2=NC(=C(NC3COC3)N12)C1=NC=C(C)N=C1 |c:6,26,t:4,8,10,21,23|	Z8873684718	**1**{*423*}	Cl.CC(C)Oc1cccc(N)n1	**2**{*123*}	Cc1cnc(C=O)cn1	**3**{*22*}	[C-]#[N+]C1COC1	0.0	0
1092	**4**{*251,108,67*}	CCC(C)NC1=C(N=C2C=CC3=C(NC(=O)C=C3)N12)C1=CN=C(N=C1)N1CCOCC1 |c:9,16,24,26,t:5,7,11,22|	Z8873685341	**1**{*251*}	Nc1ccc2ccc(=O)[nH]c2n1	**2**{*108*}	O=Cc1cnc(nc1)N2CCOCC2	**3**{*67*}	CCC(C)[N+]#[C-]	0.0	0
1093	**4**{*240,561,15*}	CCNC1=C(N=C2C=CC(=CN12)C1=NNN=N1)C1=C(F)C=C(C=C1)C1=NC=CO1 |c:7,9,17,20,23,25,30,t:3,5,14,28|	Z8873685268	**1**{*240*}	Nc1ccc(cn1)c2nn[nH]n2	**2**{*561*}	Fc1cc(ccc1C=O)c2ncco2	**3**{*15*}	CC[N+]#[C-]	0.0	0
1094	**4**{*415,139,21*}	CC1=NN(CCO)C(C)=C1C1=C(NCC2CC2)N2C(=N1)C(CCl)=CC=C2Br |c:8,11,20,25,27,t:1|	Z8873684925	**1**{*415*}	Cl.Nc1nc(Br)ccc1CCl	**2**{*139*}	Cc1nn(CCO)c(C)c1C=O	**3**{*21*}	[C-]#[N+]CC1CC1	0.0	0
1095	**4**{*424,562,39*}	CCOCCNC1=C(N=C2C=CC3=C(NC(=O)CO3)N12)C1=C(OC)C=C(C)C=C1 |c:10,23,30,t:6,8,12,27|	Z8873684903	**1**{*424*}	Nc1ccc2OCC(=O)Nc2n1	**2**{*562*}	COc1cc(C)ccc1C=O	**3**{*39*}	CCOCC[N+]#[C-]	0.0	0
1096	**4**{*211,563,41*}	COC1=CC=C(NC2=C(N=C3N2C=CC=C3C2=NN=NN2)C2=CN=C(NC(C)=O)C=C2)C=C1 |c:9,13,15,20,32,35,t:2,4,7,18,24,26|	Z8829498711	**1**{*211*}	Nc1ncccc1c2nnn[nH]2	**2**{*563*}	CC(=O)Nc1ccc(C=O)cn1	**3**{*41*}	COc1ccc([N+]#[C-])cc1	0.0	0
1097	**4**{*425,524,12*}	CC(C)OC1=NC=CN2C(NCC3CCOC3)=C(N=C12)C1=C(F)C(F)=NC(F)=C1 |c:6,17,23,27,30,t:4,19|	Z8829498569	**1**{*425*}	CC(C)Oc1nccnc1N	**2**{*524*}	Fc1cc(C=O)c(F)c(F)n1	**3**{*12*}	[C-]#[N+]CC1CCOC1	0.0	0
1098	**4**{*167,564,1*}	CN1N=NC=C1C1=C(NC2CC2)N2C(=N1)C(C)=NC=C2C |c:2,4,7,15,19,21|	Z8835022914	**1**{*167*}	Cc1cnc(C)c(N)n1	**2**{*564*}	Cn1nncc1C=O	**3**{*1*}	[C-]#[N+]C1CC1	0.0	0
1099	**4**{*422,565,50*}	COC1(CC(C1)C1=C(NC2=CC3=C(OCCO3)C=C2)N2C(=N1)C(=CC=C2F)C#C)OC |c:7,19,23,26,28,t:10,12|	Z8835022909	**1**{*422*}	Nc1nc(F)ccc1C#C	**2**{*565*}	COC1(CC(C1)C=O)OC	**3**{*50*}	[C-]#[N+]c1ccc2OCCOc2c1	0.0	0

1100	**4**{*198* ,*60* ,*32* }	CC(C1CCOC1)C1=C(NC2CCC2)N2C(C=NC(C#C)=C2Cl)=N1 |c:8,18,22,25|	Z8835022907	**1**{*198* }	Nc1cnc(C#C)c(Cl)n1	**2**{*60* }	CC(C=O)C1CCOC1	**3**{*32* }	[C-]#[N+]C1CCC1	0.0	0
1101	**4**{*61* ,*388* ,*1* }	CCC1=CC=CC2=NC(=C(NC3CC3)N12)C1=CN(C)N=C1OC |c:4,22,t:2,6,8,18|	Z8855739210	**1**{*61* }	CCc1cccc(N)n1	**2**{*388* }	COc1nn(C)cc1C=O	**3**{*1* }	[C-]#[N+]C1CC1	0.0	0
1102	**4**{*231* ,*515* ,*33* }	COC1=C(CNC2=C(N=C3C=CC(=NN23)C(F)(F)F)C2=NOC(CN(C)C)=C2)C=CC=C1 |c:2,10,12,28,31,33,t:6,8,21|	Z8855739129	**1**{*231* }	Nc1ccc(nn1)C(F)(F)F	**2**{*515* }	CN(C)Cc1cc(C=O)no1	**3**{*33* }	COc1ccccc1C[N+]#[C-]	0.0	0
1103	**4**{*260* ,*534* ,*14* }	COCCNC1=C(N=C2N1C=CC=C2N1CCCC1)C1=NN(C=C1)C(C)C |c:7,11,13,25,t:5,22|	Z8855739106	**1**{*260* }	Nc1ncccc1N2CCCC2	**2**{*534* }	CC(C)n1ccc(C=O)n1	**3**{*14* }	COCC[N+]#[C-]	0.0	0
1104	**4**{*271* ,*226* ,*67* }	CCC(C)NC1=C(N=C2C=CC(C(=O)OC)=C(F)N12)C1=C(C)N(CC(F)(F)F)N=C1 |c:9,21,30,t:5,7,15|	Z8810903011	**1**{*271* }	COC(=O)c1ccc(N)nc1F	**2**{*226* }	Cc1c(C=O)cnn1CC(F)(F)F	**3**{*67* }	CCC(C)[N+]#[C-]	0.0	0
1105	**4**{*169* ,*207* ,*13* }	CNC(=O)C1=NN2C(C=C1)=NC(=C2NCC1=CC=C(OC)C=C1)C1=CC(=NN1C)C(=O)OC |c:8,10,12,23,28,t:4,17,19,26|	Z8810902985	**1**{*169* }	CNC(=O)c1ccc(N)nn1	**2**{*207* }	COC(=O)c1cc(C=O)n(C)n1	**3**{*13* }	COc1ccc(C[N+]#[C-])cc1	0.0	0
1106	**4**{*260* ,*246* ,*14* }	COCCNC1=C(N=C2N1C=CC=C2N1CCCC1)C1CC11CCCOC1 |c:7,11,13,t:5|	Z8810902964	**1**{*260* }	Nc1ncccc1N2CCCC2	**2**{*246* }	O=CC1CC21CCCOC2	**3**{*14* }	COCC[N+]#[C-]	0.0	0
1107	**4**{*211* ,*3* ,*66* }	CC(NC1=C(N=C2N1C=CC=C2C1=NN=NN1)C1=NC=C(C=C1)C#N)C1=CC=CC=C1 |c:5,9,11,16,22,24,31,33,t:3,14,20,29|	Z8798985051	**1**{*211* }	Nc1ncccc1c2nnn[nH]2	**2**{*3* }	O=Cc1ccc(C#N)cn1	**3**{*66* }	CC([N+]#[C-])c1ccccc1	0.0	0
1108	**4**{*92* ,*566* ,*32* }	NC(=O)C1=CC2=NC(=C(NC3CCC3)N2C=C1)C1=C(Cl)N=C2SC=CN12 |c:17,20,26,t:3,5,7,23|	Z8846492139	**1**{*92* }	NC(=O)c1ccnc(N)c1	**2**{*566* }	Clc1nc2sccn2c1C=O	**3**{*32* }	[C-]#[N+]C1CCC1	0.0	0
1109	**4**{*151* ,*170* ,*13* }	CCN1N=NC(=N1)C1=C(NCC2=CC=C(OC)C=C2)N2C(C=NC=C2Cl)=N1 |c:3,5,8,18,23,25,28,t:12,14|	Z8837933149	**1**{*151* }	Nc1cncc(Cl)n1	**2**{*170* }	CCn1nnc(C=O)n1	**3**{*13* }	COc1ccc(C[N+]#[C-])cc1	0.0	0
1110	**4**{*419* ,*236* ,*21* }	FC1=C(C=CC2=NC(CCC3OCCO3)=C(NCC3CC3)N12)C#C |c:3,t:1,5,15|	Z8855619583	**1**{*419* }	Nc1ccc(C#C)c(F)n1	**2**{*236* }	O=CCCC1OCCO1	**3**{*21* }	[C-]#[N+]CC1CC1	0.0	0
1111	**4**{*419* ,*567* ,*10* }	CN1N=C2CCCC2=C1C1=C(NC2CCOC2)N2C(C=CC(C#C)=C2F)=N1 |c:8,11,22,26,29,t:2|	Z8855619516	**1**{*419* }	Nc1ccc(C#C)c(F)n1	**2**{*567* }	Cn1nc2CCCc2c1C=O	**3**{*10* }	[C-]#[N+]C1CCOC1	0.0	0
1112	**4**{*179* ,*42* ,*1* }	COC1=CC2=NC(=C(NC3CC3)N2C=C1)C1=CN(C)N=C1Cl |c:15,22,t:2,4,6,18|	Z8855619513	**1**{*179* }	COc1ccnc(N)c1	**2**{*42* }	Cn1cc(C=O)c(Cl)n1	**3**{*1* }	[C-]#[N+]C1CC1	0.0	0

1113	**4**{*181*,*512*,*26*}	CN(C)CC1=CN=C(S1)C1=C(NCC2=CC=C(F)C=C2)N2C=CC3=C(N=CC=N3)C2=N1 |c:6,10,19,23,27,29,33,t:4,14,16,25|	Z8878918854	**1**{*181*}	Nc1nccc2nccnc12	**2**{*512*}	CN(C)Cc1cnc(C=O)s1	**3**{*26*}	Fc1ccc(C[N+]#[C-])cc1	0.0	0
1114	**4**{*392*,*530*,*67*}	CCC(C)NC1=C(N=C2C=CC3=C(NC(C)=N3)N12)C1CC2(CC(C2)OC)C1 |c:9,15,t:5,7,11|	Z8878918595	**1**{*392*}	Cl.Cc1nc2ccc(N)nc2[nH]1	**2**{*530*}	COC1CC2(C1)CC(C2)C=O	**3**{*67*}	CCC(C)[N+]#[C-]	0.0	0
1115	**4**{*251*,*568*,*71*}	COC1=CC(NC2=C(N=C3C=CC4=C(NC(=O)C=C4)N23)C2=CN=C(S2)C2=NC=CN=C2)=CC=C1 |c:10,17,25,31,33,35,37,t:2,6,8,12,23,29|	Z8878918353	**1**{*251*}	Nc1ccc2ccc(=O)[nH]c2n1	**2**{*568*}	O=Cc1cnc(s1)c2cnccn2	**3**{*71*}	COc1cccc([N+]#[C-])c1	0.0	0
1116	**4**{*410*,*217*,*62*}	COC(=O)C1(CCCC1)NC1=C(N=C2C=CC3=C(N=CC(Cl)=C3)N12)C1=CC(=O)N(C)C=C1 |c:15,19,22,34,t:11,13,17,28|	Z8873684787	**1**{*410*}	Nc1ccc2cc(Cl)cnc2n1	**2**{*217*}	Cn1ccc(C=O)cc1=O	**3**{*62*}	COC(=O)C1(CCCC1)[N+]#[C-]	0.0	0
1117	**4**{*420*,*569*,*67*}	CCC(C)NC1=C(CC2CCOC2=O)N=C2C=CC3=C(NC=N3)N12 |c:5,17,22,t:15,19|	Z8873685393	**1**{*420*}	Cl.Cl.Nc1ccc2nc[nH]c2n1	**2**{*569*}	O=CCC1CCOC1=O	**3**{*67*}	CCC(C)[N+]#[C-]	0.0	0
1118	**4**{*19*,*316*,*22*}	COC(=O)C1=CC2=C(C=C1)N=C(C=C2)C1=C(NC2COC2)N2C=CC=C(C#N)C2=N1 |c:6,8,11,13,16,25,32,t:4,27|	Z8873685313	**1**{*19*}	Nc1ncccc1C#N	**2**{*316*}	COC(=O)c1ccc2nc(C=O)ccc2c1	**3**{*22*}	[C-]#[N+]C1COC1	0.0	0
1119	**4**{*392*,*530*,*39*}	CCOCCNC1=C(N=C2C=CC3=C(NC(C)=N3)N12)C1CC2(CC(C2)OC)C1 |c:10,16,t:6,8,12|	Z8873685309	**1**{*392*}	Cl.Cc1nc2ccc(N)nc2[nH]1	**2**{*530*}	COC1CC2(C1)CC(C2)C=O	**3**{*39*}	CCOCC[N+]#[C-]	0.0	0
1120	**4**{*292*,*444*,*32*}	CCOC1=CN=CC2=NC(=C(NC3CCC3)N12)C1=C(O)C=CN=C1 |c:5,20,23,25,t:3,7,9|	Z8873684915	**1**{*292*}	CCOc1cncc(N)n1	**2**{*444*}	Oc1ccncc1C=O	**3**{*32*}	[C-]#[N+]C1CCC1	0.0	0
1121	**4**{*151*,*453*,*9*}	CNC1=C(N=C2C=NC=C(Cl)N12)C1=CC(C=C)=CN=C1 |c:6,18,20,t:2,4,8,14|	Z8829498655	**1**{*151*}	Nc1cncc(Cl)n1	**2**{*453*}	Cl.C=Cc1cncc(C=O)c1	**3**{9}	C[N+]#[C-]	0.0	0
1122	**4**{*99*,*224*,*41*}	COC1=CC=C(NC2=C(N=C3C=CC(=CN23)S(N)(=O)=O)C2CCOC2C)C=C1 |c:11,13,29,t:2,4,7,9|	Z8854581182	**1**{*99*}	Nc1ccc(cn1)S(=O)(=O)N	**2**{*224*}	CC1OCCC1C=O	**3**{*41*}	COc1ccc([N+]#[C-])cc1	0.0	0
1123	**4**{*262*,*399*,*43*}	CC(=O)NC1=CN2C(C=C1)=NC(=C2NC1CCN(CC1)C(=O)OC(C)(C)C)C1=CN(N=C1C)C1CC1 |c:8,10,12,33,t:4,30|	Z8835022897	**1**{*262*}	CC(=O)Nc1ccc(N)nc1	**2**{*399*}	Cc1nn(cc1C=O)C2CC2	**3**{*43*}	CC(C)(C)OC(=O)N1CCC(CC1)[N+]#[C-]	0.0	0
1124	**4**{*144*,*477*,*47*}	CNS(=O)(=O)C1=CN2C(C=C1)=NC(C(NC(=O)OC(C)(C)C)C1COC1)=C2NC(C)C |c:9,11,27,t:5|	Z8855739262	**1**{*144*}	CNS(=O)(=O)c1ccc(N)nc1	**2**{*477*}	CC(C)(C)OC(=O)NC(C=O)C1COC1	**3**{*47*}	CC(C)[N+]#[C-]	0.0	0

1125	**4**{*174*,*202*,*61*}	CC(C)(C)OC(=O)NCCCNC1=C(N=C2C=CC=C(C#N)N12)C1=C(F)C=NC=C1F |c:16,25,28,30,t:12,14,18|	Z8846492017	**1**{*174*}	Nc1cccc(C#N)n1	**2**{*202*}	Fc1cncc(F)c1C=O	**3**{*61*}	CC(C)(C)OC(=O)NCCC[N+]#[C-]	0.0	0
1126	**4**{*169*,*570*,*14*}	CNC(=O)C1=NN2C(C=C1)=NC(C1CCN(CC1C)C(=O)OC(C)(C)C)=C2NCCOC |c:8,10,27,t:4|	Z8855619678	**1**{*169*}	CNC(=O)c1ccc(N)nn1	**2**{*570*}	CC1CN(CCC1C=O)C(=O)OC(C)(C)C	**3**{*14*}	COCC[N+]#[C-]	0.0	0
1127	**4**{*426*,*571*,*67*}	CCC(C)NC1=C(N=C2C=C(C=CN12)C1=NN=NN1)C1CC2(CCC2(OC)OC)C1 |c:9,11,18,t:5,7,16|	Z8855619619	**1**{*426*}	Nc1cc(ccn1)c2nnn[nH]2	**2**{*571*}	COC1(CCC21CC(C2)C=O)OC	**3**{*67*}	CCC(C)[N+]#[C-]	0.0	0
1128	**4**{*1*,*22*,*27*}	CCCCNC1=C(N=C2C=CNN12)C1=CC=CC=C1 |c:9,17,19,t:5,7,15|	Z8711892168	**1**{*1*}	Nc1cc[nH]n1	**2**{*22*}	O=Cc1ccccc1	**3**{*27*}	CCCC[N+]#[C-]	0.0	0
1129	**4**{*427*,*572*,*5*}	COCCCNC1=C(N=C2C=C3C=CC=NC3=C(OC)N12)C1=NC(=CC(OC)=C1)C(=O)OC |c:12,14,26,30,t:6,8,10,17,24|	Z8878918797	**1**{*427*}	COc1nc(N)cc2cccnc12	**2**{*572*}	COC(=O)c1cc(OC)cc(C=O)n1	**3**{*5*}	COCCC[N+]#[C-]	0.0	0
1130	**4**{*251*,*573*,*62*}	COC(=O)C1(CCCC1)NC1=C(N=C2C=CC3=C(NC(=O)C=C3)N12)C1=C(C)SN=C1C |c:15,22,28,32,t:11,13,17|	Z8878918589	**1**{*251*}	Nc1ccc2ccc(=O)[nH]c2n1	**2**{*573*}	Cc1nsc(C)c1C=O	**3**{*62*}	COC(=O)C1(CCCC1)[N+]#[C-]	0.0	0
1131	**4**{*240*,*574*,*22*}	CC(C)(C)OC(=O)N[C@H](CC1=C(NC2COC2)N2C=C(C=CC2=N1)C1=NNN=N1)C(C)(C)C |c:10,19,21,24,30,t:27|	Z8878918571	**1**{*240*}	Nc1ccc(cn1)c2nn[nH]n2	**2**{*574*}	CC(C)(C)OC(=O)N[C@H](CC=O)C(C)(C)C	**3**{*22*}	[C-]#[N+]C1COC1	0.0	0
1132	**4**{*240*,*575*,*22*}	CC(C)(C)OC(=O)NC(CC1=C(NC2COC2)N2C=C(C=CC2=N1)C1=NNN=N1)C(F)(F)F |c:10,19,21,24,30,t:27|	Z8878918544	**1**{*240*}	Nc1ccc(cn1)c2nn[nH]n2	**2**{*575*}	CC(C)(C)OC(=O)NC(CC=O)C(F)(F)F	**3**{*22*}	[C-]#[N+]C1COC1	0.0	0
1133	**4**{*424*,*576*,*39*}	CCOCCNC1=C(N=C2C=CC3=C(NC(=O)CO3)N12)C1=C(OC)C=CC=N1 |c:10,23,27,29,t:6,8,12|	Z8878918477	**1**{*424*}	Nc1ccc2OCC(=O)Nc2n1	**2**{*576*}	COc1cccnc1C=O	**3**{*39*}	CCOCC[N+]#[C-]	0.0	0
1134	**4**{*428*,*544*,*60*}	COC(=O)C1=CC(NC2=C(N=C3C=NC(Br)=CN23)C2=NN=C3CCCN23)=CC=C1 |c:12,15,29,31,t:4,8,10,20,22|	Z8878918461	**1**{*428*}	Nc1cnc(Br)cn1	**2**{*544*}	O=Cc1nnc2CCCn12	**3**{*60*}	COC(=O)c1cccc([N+]#[C-])c1	0.0	0
1135	**4**{*424*,*577*,*39*}	CCOCCNC1=C(N=C2C=CC3=C(NC(=O)CO3)N12)C1=CC(=CC(F)=C1)C#N |c:10,25,28,t:6,8,12,23|	Z8878918450	**1**{*424*}	Nc1ccc2OCC(=O)Nc2n1	**2**{*577*}	Fc1cc(C=O)cc(C#N)c1	**3**{*39*}	CCOCC[N+]#[C-]	0.0	0
1136	**4**{*19*,*55*,*3*}	CP(C)(=O)C1=NC=CC(=C1)C1=C(NC2=C(F)C=C(Cl)C=C2)N2C=CC=C(C#N)C2=N1 |c:6,8,11,14,20,24,31,t:4,17,26|	Z8878918414	**1**{*19*}	Nc1ncccc1C#N	**2**{*55*}	CP(=O)(C)c1cc(C=O)ccn1	**3**{*3*}	Fc1cc(Cl)ccc1[N+]#[C-]	0.0	0

1137	**4**{*425*,*281*,*46*}	COC(=O)C1=NN=C(C=C1)C1=C(NCC(C)=C)N2C=CN=C(OC(C)C)C2=N1 |c:6,8,11,19,28,t:4,21|	Z8878918359	**1**{*425*}	CC(C)Oc1nccnc1N	**2**{*281*}	COC(=O)c1ccc(C=O)nn1	**3**{*46*}	CC(=C)C[N+]#[C-]	0.0	0
1138	**4**{*353*,*547*,*15*}	CCNC1=C(N=C2C=CC(NS(C)(=O)=O)=CN12)C1=C(Br)N=C(S1)C(F)(F)F |c:7,14,19,22,t:3,5|	Z8873684737	**1**{*353*}	Cl.CS(=O)(=O)Nc1ccc(N)nc1	**2**{*547*}	FC(F)(F)c1nc(Br)c(C=O)s1	**3**{*15*}	CC[N+]#[C-]	0.0	0
1139	**4**{*420*,*578*,*67*}	CCC(C)NC1=C(N=C2C=CC3=C(NC=N3)N12)C1=CC(=CC(=C1)C#N)C#N |c:9,14,22,24,t:5,7,11,20|	Z8873684712	**1**{*420*}	Cl.Cl.Nc1ccc2nc[nH]c2n1	**2**{*578*}	O=Cc1cc(C#N)cc(C#N)c1	**3**{*67*}	CCC(C)[N+]#[C-]	0.0	0
1140	**4**{*411*,*188*,*37*}	COC(=O)C1CC(C1)NC1=C(CCC2(CC2)C#N)N=C2C=C(Br)C=C(F)N12 |c:10,t:20,22,25|	Z8873684705	**1**{*411*}	Nc1cc(Br)cc(F)n1	**2**{*188*}	O=CCCC1(CC1)C#N	**3**{*37*}	COC(=O)C1CC(C1)[N+]#[C-]	0.0	0
1141	**4**{*54*,*543*,*15*}	CCNC1=C(N=C2C=CC(=CN12)C(=O)N(C)C)C(=O)OCC |c:7,9,t:3,5|	Z8873684702	**1**{*54*}	Cl.CN(C)C(=O)c1ccc(N)nc1	**2**{*543*}	CCOC(=O)C=O	**3**{*15*}	CC[N+]#[C-]	0.0	0
1142	**4**{*251*,*579*,*67*}	CCCN1C=C(C=N1)C1=C(NC(C)CC)N2C(C=CC3=C2NC(=O)C=C3)=N1 |c:4,6,9,18,20,26,28|	Z8873685293	**1**{*251*}	Nc1ccc2ccc(=O)[nH]c2n1	**2**{*579*}	CCCn1cc(C=O)cn1	**3**{*67*}	CCC(C)[N+]#[C-]	0.0	0
1143	**4**{*348*,*509*,*2*}	CN1C=NN=C1C1=C(NC2=CC=C(F)C=C2)N2C=C(F)N=CC2=N1 |c:2,4,7,15,22,25,t:10,12,19|	Z8873684897	**1**{*348*}	Nc1cnc(F)cn1	**2**{*509*}	Cn1cnnc1C=O	**3**{*2*}	Fc1ccc([N+]#[C-])cc1	0.0	0
1144	**4**{*193*,*520*,*10*}	CNC(=O)C1=CN2C(C=C1)=NC(=C2NC1CCOC1)C1=C(OC)C=NN=C1 |c:8,10,12,22,26,28,t:4|	Z8873684606	**1**{*193*}	CNC(=O)c1ccc(N)nc1	**2**{*520*}	COc1cnncc1C=O	**3**{*10*}	[C-]#[N+]C1CCOC1	0.0	0
1145	**4**{*89*,*217*,*71*}	COC1=CC(NC2=C(N=C3C=CC4=C(NN=C4)N23)C2=CC(=O)N(C)C=C2)=CC=C1 |c:10,15,27,29,31,t:2,6,8,12,21|	Z8873684593	**1**{*89*}	Nc1ccc2cn[nH]c2n1	**2**{*217*}	Cn1ccc(C=O)cc1=O	**3**{*71*}	COc1cccc([N+]#[C-])c1	0.0	0
1146	**4**{*187*,*281*,*12*}	COC(=O)C1=NN=C(C=C1)C1=C(NCC2CCOC2)N2C=C(Cl)C(=CC2=N1)C#N |c:6,8,11,25,28,t:4,22|	Z8873684820	**1**{*187*}	Nc1cc(C#N)c(Cl)cn1	**2**{*281*}	COC(=O)c1ccc(C=O)nn1	**3**{*12*}	[C-]#[N+]CC1CCOC1	0.0	0
1147	**4**{*73*,*1*,*13*}	COC1=CC=C(CNC2=C(N=C3C=CC4=NC=CN4N23)C2=C(Cl)C=NC=C2Cl)C=C1 |c:12,16,23,26,28,32,t:2,4,8,10,14|	Z8854581154	**1**{*73*}	Nc1ccc2nccn2n1	**2**{*1*}	Clc1cncc(Cl)c1C=O	**3**{*13*}	COc1ccc(C[N+]#[C-])cc1	0.0	0
1148	**4**{*169*,*97*,*14*}	CNC(=O)C1=NN2C(C=C1)=NC(=C2NCCOC)C1=CC=C(F)C=C1 |c:8,10,12,25,t:4,20,22|	Z8835022906	**1**{*169*}	CNC(=O)c1ccc(N)nn1	**2**{*97*}	Fc1ccc(C=O)cc1	**3**{*14*}	COCC[N+]#[C-]	0.0	0

1149	**4**{*166*,*580*,*22*}	OCCSCC1=CC2=NC(=C(NC3COC3)N2C=C1)C1=C(F)C(Cl)=NC(Cl)=C1 |c:19,22,26,29,t:5,7,9|	Z8835022900	**1**{*166*}	Nc1cc(CSCCO)ccn1	**2**{*580*}	Fc1c(Cl)nc(Cl)cc1C=O	**3**{*22*}	[C-]#[N+]C1COC1	0.0	0
1150	**4**{*330*,*581*,*1*}	CCC1=CN=C(O1)C1=C(NC2CC2)N2C=C(C=CC2=N1)S(=O)(=O)N(C)C |c:4,8,16,18,21,t:2|	Z8835022884	**1**{*330*}	CN(C)S(=O)(=O)c1ccc(N)nc1	**2**{*581*}	CCc1cnc(C=O)o1	**3**{*1*}	[C-]#[N+]C1CC1	0.0	0
1151	**4**{*399*,*582*,*35*}	CSCCCNC1=C(N=C2C=CC(OC(C)C)=NN12)C(CC(F)F)NC(=O)OC(C)(C)C |c:10,16,t:6,8|	Z8849597814	**1**{*399*}	CC(C)Oc1ccc(N)nn1	**2**{*582*}	CC(C)(C)OC(=O)NC(CC(F)F)C=O	**3**{*35*}	CSCCC[N+]#[C-]	0.0	0
1152	**4**{*406*,*146*,*7*}	C[C@@H]1C[C@@H]1CCC1=C(NC2CCOCC2)N2C(C=CC=C2C2=NNN=N2)=N1 |&1:1,3,r,c:7,19,21,27,29,t:24|	Z8849597813	**1**{*406*}	Nc1cccc(n1)c2nn[nH]n2	**2**{*146*}	C[C@@H]1C[C@@H]1CCC=O |&1:1,3,r|	**3**{*7*}	[C-]#[N+]C1CCOCC1	0.0	0
1153	**4**{*50*,*268*,*7*}	CC[C@@H](NC(=O)OC(C)(C)C)C1=C(NC2CCOCC2)N2C(C=CC=C2P(C)(C)=O)=N1 |c:11,23,25,31|	Z8855739264	**1**{*50*}	CP(=O)(C)c1cccc(N)n1	**2**{*268*}	CC[C@@H](NC(=O)OC(C)(C)C)C=O	**3**{*7*}	[C-]#[N+]C1CCOCC1	0.0	0
1154	**4**{*429*,*200*,*23*}	COC(=O)CCC(NC1=C(CCCC#CC)N=C2N1C=CC=C2CBr)C(=O)OC |c:8,16,20,22|	Z8846492230	**1**{*429*}	Br.Nc1ncccc1CBr	**2**{*200*}	CC#CCCCC=O	**3**{*23*}	COC(=O)CCC([N+]#[C-])C(=O)OC	0.0	0
1155	**4**{*334*,*137*,*11*}	CC1=C(C(C)=NO1)C1=C(NCC2=CC(Br)=CC=C2)N2C=C3COCCC3=C(C#N)C2=N1 |c:4,8,15,17,33,t:1,12,21,28|	Z8837933169	**1**{*334*}	Nc1ncc2COCCc2c1C#N	**2**{*137*}	Cc1noc(C)c1C=O	**3**{*11*}	Brc1cccc(C[N+]#[C-])c1	0.0	0
1156	**4**{*283*,*8*,*71*}	COCC1=NOC=C1C1=C(NC2=CC(OC)=CC=C2)N2C(C=CC3=C2N=CC(=C3)C(=O)OC)=N1 |c:6,9,16,18,23,25,28,30,36,t:3,12|	Z8837933153	**1**{*283*}	COC(=O)c1cnc2nc(N)ccc2c1	**2**{*8*}	COCc1nocc1C=O	**3**{*71*}	COc1cccc([N+]#[C-])c1	0.0	0
1157	**4**{*169*,*30*,*9*}	CNC(=O)C1=NN2C(C=C1)=NC(C1CCN(CC1)C(C)=O)=C2NC |c:8,10,22,t:4|	Z8855619766	**1**{*169*}	CNC(=O)c1ccc(N)nn1	**2**{*30*}	CC(=O)N1CCC(CC1)C=O	**3**{*9*}	C[N+]#[C-]	0.0	0
1158	**4**{*167*,*550*,*1*}	CN1N=C(Br)N=C1C1=C(NC2CC2)N2C(=N1)C(C)=NC=C2C |c:5,8,16,20,22,t:2|	Z8855619760	**1**{*167*}	Cc1cnc(C)c(N)n1	**2**{*550*}	Cn1nc(Br)nc1C=O	**3**{*1*}	[C-]#[N+]C1CC1	0.0	0
1159	**4**{*430*,*583*,*34*}	CC1=C(N2C=CC(Cl)=CC2=N1)C1=C(NCC2CCCO2)N2C=CC3=C(C2=N1)S(=O)(=O)NC3=O |c:4,7,10,13,24,26,29,t:1|	Z8855619649	**1**{*430*}	Nc1cccc2C(=O)NS(=O)(=O)c21	**2**{*583*}	Cc1nc2cc(Cl)ccn2c1C=O	**3**{*34*}	[C-]#[N+]CC1CCCO1	0.0	0
1160	**4**{*192*,*79*,*14*}	COCCNC1=C(N=C2N1C(F)=CC=C2Cl)C1=NC=CO1 |c:7,12,14,20,t:5,18|	Z8855619641	**1**{*192*}	Nc1nc(F)ccc1Cl	**2**{*79*}	O=Cc1ncco1	**3**{*14*}	COCC[N+]#[C-]	0.0	0

Parallel synthesis of compound library ***4*** *, main experiment*

<table>
<tr><td>1161</td><td>4{28,189,7}</td><td>C1CC(CCO1)NC1=C(N=C2C=C(C=CN12)C1=NOC=N1)C1=CN(N=N1)C1CCOCC1 |c:12,14,22,28,t:8,10,19,25|</td><td>Z8878918418</td><td>1{28}</td><td>Cl.Nc1cc(ccn1)c2ncon2</td><td>2{189}</td><td>O=Cc1cn(nn1)C2CCOCC2</td><td>3{7}</td><td>[C-]#[N+]C1CCOCC1</td><td>105.1</td><td>89</td></tr>
<tr><td>1162</td><td>4{180,336,32}</td><td>COC(=O)C1=CC2=NC(=C(NC3CCC3)N2C=C1C)C1=CSN=C1OC |c:18,25,t:4,6,8,22|</td><td>Z8878918612</td><td>1{180}</td><td>COC(=O)c1cc(N)ncc1C</td><td>2{336}</td><td>COc1nscc1C=O</td><td>3{32}</td><td>[C-]#[N+]C1CCC1</td><td>83.5</td><td>83</td></tr>
<tr><td>1163</td><td>4{76,584,23}</td><td>COC(=O)CCC(NC1=C(N=C2C=CC(C)=C(Cl)N12)C1=C(OC)C=C(C)N=C1)C(=O)OC |c:12,21,28,t:8,10,15,25|</td><td>Z8873684644</td><td>1{76}</td><td>Cc1ccc(N)nc1Cl</td><td>2{584}</td><td>COc1cc(C)ncc1C=O</td><td>3{23}</td><td>COC(=O)CCC([N+]#[C-])C(=O)OC</td><td>100.6</td><td>81</td></tr>
<tr><td>1164</td><td>4{180,545,35}</td><td>COCCN1N=C(C)C(=C1C)C1=C(NCCCSC)N2C=C(C)C(=CC2=N1)C(=O)OC |c:8,12,24,27,t:5,21|</td><td>Z8878918407</td><td>1{180}</td><td>COC(=O)c1cc(N)ncc1C</td><td>2{545}</td><td>COCCn1nc(C)c(C=O)c1C</td><td>3{35}</td><td>CSCCC[N+]#[C-]</td><td>96.9</td><td>81</td></tr>
<tr><td>1165</td><td>4{405,585,5}</td><td>COCCCNC1=C(CC2CC2(Cl)Cl)N=C2C=CC(=CN12)N1CCCC1=O |c:6,17,19,t:15|</td><td>Z8855739175</td><td>1{405}</td><td>Nc1ccc(cn1)N2CCCC2=O</td><td>2{585}</td><td>ClC1(Cl)CC1CC=O</td><td>3{5}</td><td>COCCC[N+]#[C-]</td><td>86.6</td><td>78</td></tr>
<tr><td>1166</td><td>4{392,586,67}</td><td>CCC(C)NC1=C(N=C2C=CC3=C(NC(C)=N3)N12)C1=CC2=C(COC2)N=C1 |c:9,15,29,t:5,7,11,21,23|</td><td>Z8878918627</td><td>1{392}</td><td>Cl.Cc1nc2ccc(N)nc2[nH]1</td><td>2{586}</td><td>O=Cc1cnc2COCc2c1</td><td>3{67}</td><td>CCC(C)[N+]#[C-]</td><td>75.2</td><td>77</td></tr>
<tr><td>1167</td><td>4{374,587,5}</td><td>COCCCNC1=C(N=C2C=CC=C(N12)C(=O)OC)C1=C(C)ON=C1OC |c:10,12,21,25,t:6,8|</td><td>Z8835022866</td><td>1{374}</td><td>COC(=O)c1cccc(N)n1</td><td>2{587}</td><td>COc1noc(C)c1C=O</td><td>3{5}</td><td>COCCC[N+]#[C-]</td><td>76.2</td><td>75</td></tr>
<tr><td>1168</td><td>4{431,588,61}</td><td>CN1C=C(C(C)=N1)C1=C(NCCCNC(=O)OC(C)(C)C)N2C=C(F)C=CC2=N1 |c:2,5,8,26,29,t:23|</td><td>Z8878918395</td><td>1{431}</td><td>Nc1ccc(F)cn1</td><td>2{588}</td><td>Cc1nn(C)cc1C=O</td><td>3{61}</td><td>CC(C)(C)OC(=O)NCCC[N+]#[C-]</td><td>80.5</td><td>74</td></tr>
<tr><td>1169</td><td>4{64,589,49}</td><td>CN1N=C(C)C(=C1Cl)C1=C(NC2CC(F)(F)C2)N2C=C(C=CC2=N1)N1CCCS1(=O)=O |c:5,9,20,22,25,t:2|</td><td>Z8878918677</td><td>1{64}</td><td>Nc1ccc(cn1)N2CCCS2(=O)=O</td><td>2{589}</td><td>Cc1nn(C)c(Cl)c1C=O</td><td>3{49}</td><td>FC1(F)CC(C1)[N+]#[C-]</td><td>91.5</td><td>72</td></tr>
<tr><td>1170</td><td>4{153,336,53}</td><td>COC1=NSC=C1C1=C(NCCOCC2=CC=CC=C2)N2C(C=CC=C2C(C)(C)O)=N1 |c:5,8,17,19,24,26,32,t:2,15|</td><td>Z8855619615</td><td>1{153}</td><td>CC(C)(O)c1cccc(N)n1</td><td>2{336}</td><td>COc1nscc1C=O</td><td>3{53}</td><td>[C-]#[N+]CCOCc1ccccc1</td><td>84.8</td><td>72</td></tr>
<tr><td>1171</td><td>4{103,590,59}</td><td>OCC1=CN=CC2=NC(=C(NCCC3=CC(F)=CC=C3)N12)C1=CN=C(C=C1)C1=CSC=N1 |c:4,16,18,26,28,34,t:2,6,8,13,24,31|</td><td>Z8878918413</td><td>1{103}</td><td>Nc1cncc(CO)n1</td><td>2{590}</td><td>O=Cc1ccc(nc1)c2cscn2</td><td>3{59}</td><td>Fc1cccc(CC[N+]#[C-])c1</td><td>86.3</td><td>72</td></tr>
</table>

1172	**4**{*362* ,*326* ,*21* }	CNC(=O)C1=CC=CC2=NC(=C(NCC3CC3)N12)C1=CC=NN1C |c:6,23,t:4,8,10,21|	Z8878918629	**1**{*362* }	CNC(=O)c1cccc(N)n1	**2**{*326* }	Cn1nccc1C=O	**3**{*21* }	[C-]#[N+]CC1CC1	61.3	70
1173	**4**{*105* ,*591* ,*29* }	CCOC(=O)CCNC1=C(N=C2C=CC=C(N12)C(N)=O)C1=CC=C(C=C1)N1CCOCC1 |c:12,14,24,26,t:8,10,22|	Z8878918651	**1**{*105* }	NC(=O)c1cccc(N)n1	**2**{*591* }	O=Cc1ccc(cc1)N2CCOCC2	**3**{*29* }	CCOC(=O)CC[N+]#[C-]	82.0	69
1174	**4**{*365* ,*592* ,*10* }	COC(=O)C1=CC(=CN1C)C1=C(NC2CCOC2)N2C(C=C(C)C=C2C)=N1 |c:6,11,25,28,t:4,22|	Z8878918853	**1**{*365* }	Cc1cc(C)nc(N)c1	**2**{*592* }	COC(=O)c1cc(C=O)cn1C	**3**{*10* }	[C-]#[N+]C1CCOC1	68.9	69
1175	**4**{*62* ,*593* ,*6* }	CCOC(=O)CCCNC1=C(N=C2C=C(CO)C=CN12)C1=CC=C(NC(C)=O)C=C1 |c:17,30,t:9,11,13,22,24|	Z8873685260	**1**{*62* }	Nc1cc(CO)ccn1	**2**{*593* }	CC(=O)Nc1ccc(C=O)cc1	**3**{*6* }	CCOC(=O)CCC[N+]#[C-]	76.2	69
1176	**4**{*28* ,*461* ,*7* }	CCN1N=CC(=C1F)C1=C(NC2CCOCC2)N2C=CC(=CC2=N1)C1=NOC=N1 |c:3,5,9,20,22,25,31,t:28|	Z8873684724	**1**{*28* }	Cl.Nc1cc(ccn1)c2ncon2	**2**{*461* }	CCn1ncc(C=O)c1F	**3**{*7* }	[C-]#[N+]C1CCOCC1	73.7	69
1177	**4**{*362* ,*594* ,*12* }	CNC(=O)C1=CC=CC2=NC(=C(NCC3CCOC3)N12)C1=CC=C(C=C1)N(C)C |c:6,25,27,t:4,8,10,23|	Z8855739195	**1**{*362* }	CNC(=O)c1cccc(N)n1	**2**{*594* }	CN(C)c1ccc(C=O)cc1	**3**{*12* }	[C-]#[N+]CC1CCOC1	72.9	69
1178	**4**{*50* ,*595* ,*7* }	CN1C=C2C=C(C=CC2=N1)C1=C(NC2CCOCC2)N2C(C=CC=C2P(C)(C)=O)=N1 |c:4,6,9,12,24,26,32,t:2|	Z8878918460	**1**{*50* }	CP(=O)(C)c1cccc(N)n1	**2**{*595* }	Cn1cc2cc(C=O)ccc2n1	**3**{*7* }	[C-]#[N+]C1CCOCC1	77.4	68
1179	**4**{*109* ,*335* ,*41* }	COC1=CC=C(NC2=C(N=C3C=CC=C(N23)S(N)(=O)=O)C2=CN(N=C2)C2CCOCC2)C=C1 |c:11,13,25,35,t:2,4,7,9,22|	Z8855739105	**1**{*109* }	Cl.Nc1cccc(n1)S(=O)(=O)N	**2**{*335* }	O=Cc1cnn(c1)C2CCOCC2	**3**{*41* }	COc1ccc([N+]#[C-])cc1	84.6	67
1180	**4**{*37* ,*596* ,*12* }	CN1N=C(C)C2=C1N=CC(=C2)C1=C(NCC2CCOC2)N2C(C=CC=C2C(F)F)=N1 |c:5,8,10,13,25,27,32,t:2|	Z8873685247	**1**{*37* }	Cl.Nc1cccc(n1)C(F)F	**2**{*596* }	Cc1nn(C)c2ncc(C=O)cc12	**3**{*12* }	[C-]#[N+]CC1CCOC1	73.7	66
1181	**4**{*42* ,*192* ,*7* }	COC1=NC(=CC=C1)C1=C(NC2CCOCC2)N2C=C(SC)C=CC2=N1 |c:4,6,9,24,27,t:2,20|	Z8855619653	**1**{*42* }	CSc1ccc(N)nc1	**2**{*192* }	COc1cccc(C=O)n1	**3**{*7* }	[C-]#[N+]C1CCOCC1	65.2	65
1182	**4**{*125* ,*597* ,*7* }	CC1=C(C)N=C(O1)C1=C(NC2CCOCC2)N2C=C(CCO)C=CC2=N1 |c:1,4,8,24,27,t:19|	Z8873685318	**1**{*125* }	Nc1ccc(CCO)cn1	**2**{*597* }	Cc1nc(C=O)oc1C	**3**{*7* }	[C-]#[N+]C1CCOCC1	62.6	65
1183	**4**{*333* ,*152* ,*29* }	CCOC(=O)CCNC1=C(N=C2C=CC=C(N12)C1=CC=NC=C1)C(C)CCC(=O)OC |c:12,14,21,23,t:8,10,19|	Z8878918843	**1**{*333* }	Nc1cccc(n1)c2ccncc2	**2**{*152* }	COC(=O)CCC(C)C=O	**3**{*29* }	CCOC(=O)CC[N+]#[C-]	73.1	64

1184	**4**{*432*,*452*,*22*}	CC1=CC2=NC(=C(NC3COC3)N2C=C1I)C1=CC=C(C=C1)C(N)=O |c:15,21,23,t:1,3,5,19|	Z8835022888	**1**{*432*}	Cc1cc(N)ncc1I	**2**{*452*}	NC(=O)c1ccc(C=O)cc1	**3**{*22*}	[C-]#[N+]C1COC1	76.9	64
1185	**4**{*414*,*598*,*10*}	COC1=CC(OC)=C(C2=C(NC3CCOC3)N3C=C(Cl)N=CC3=N2)C(C)=C1 |c:8,21,24,28,t:2,6,18|	Z8878918855	**1**{*414*}	Nc1cnc(Cl)cn1	**2**{*598*}	COc1cc(C)c(C=O)c(OC)c1	**3**{*10*}	[C-]#[N+]C1CCOC1	66.7	64
1186	**4**{*50*,*599*,*7*}	CC1=CC(=CC(C)=C1OC(F)F)C1=C(NC2CCOCC2)N2C(C=CC=C2P(C)(C)=O)=N1 |c:3,6,13,25,27,33,t:1|	Z8873684592	**1**{*50*}	CP(=O)(C)c1cccc(N)n1	**2**{*599*}	Cc1cc(C=O)cc(C)c1OC(F)F	**3**{*7*}	[C-]#[N+]C1CCOCC1	79.2	63
1187	**4**{*36*,*30*,*13*}	COC1=CC=C(CNC2=C(N=C3C=CC(Br)=CN23)C2CCN(CC2)C(C)=O)C=C1 |c:12,15,30,t:2,4,8,10|	Z8835022908	**1**{*36*}	Nc1ccc(Br)cn1	**2**{*30*}	CC(=O)N1CCC(CC1)C=O	**3**{*13*}	COc1ccc(C[N+]#[C-])cc1	77.7	63
1188	**4**{*105*,*600*,*50*}	CS(=O)(=O)CCN1C=C(C=N1)C1=C(NC2=CC3=C(OCCO3)C=C2)N2C(C=CC=C2C(N)=O)=N1 |c:7,9,12,24,29,31,36,t:15,17|	Z8829498490	**1**{*105*}	NC(=O)c1cccc(N)n1	**2**{*600*}	CS(=O)(=O)CCn1cc(C=O)cn1	**3**{*50*}	[C-]#[N+]c1ccc2OCCOc2c1	81.3	62
1189	**4**{*73*,*24*,*20*}	COC1=CC(C)=C(NC2=C(N=C3C=CC4=NC=CN4N23)C2=CC(=CC=C2)N(C)C)C=C1 |c:12,16,25,27,33,t:2,5,8,10,14,23|	Z8873685335	**1**{*73*}	Nc1ccc2nccn2n1	**2**{*24*}	CN(C)c1cccc(C=O)c1	**3**{*20*}	COc1ccc([N+]#[C-])c(C)c1	69.5	62
1190	**4**{*50*,*601*,*7*}	CC1=C(C=CC(=C1)C1=C(NC2CCOCC2)N2C(C=CC=C2P(C)(C)=O)=N1)C1CC1 |c:3,5,8,20,22,28,t:1|	Z8873684736	**1**{*50*}	CP(=O)(C)c1cccc(N)n1	**2**{*601*}	Cc1cc(C=O)ccc1C2CC2	**3**{*7*}	[C-]#[N+]C1CCOCC1	67.6	62
1191	**4**{*118*,*422*,*32*}	CC(O)C1=CC=CC2=NC(C3CC33CCOC3)=C(NC3CCC3)N12 |c:5,t:3,7,18|	Z8855739136	**1**{*118*}	Cl.CC(O)c1cccc(N)n1	**2**{*422*}	O=CC1CC21CCOC2	**3**{*32*}	[C-]#[N+]C1CCC1	54.4	62
1192	**4**{*369*,*54*,*7*}	COC1=C(C=CS1)C1=C(NC2CCOCC2)N2C=C(C=CC2=N1)C(F)C(F)(F)F |c:4,8,19,21,24,t:2|	Z8873684631	**1**{*369*}	Cl.Nc1ccc(cn1)C(F)C(F)(F)F	**2**{*54*}	COc1sccc1C=O	**3**{*7*}	[C-]#[N+]C1CCOCC1	71.3	61
1193	**4**{*118*,*602*,*31*}	CSCCNC1=C(N=C2C=CC=C(C(C)O)N12)C1=CC=C(C=C1)C(=O)NC1CC1 |c:9,21,23,t:5,7,11,19|	Z8878918307	**1**{*118*}	Cl.CC(O)c1cccc(N)n1	**2**{*602*}	O=Cc1ccc(cc1)C(=O)NC2CC2	**3**{*31*}	CSCC[N+]#[C-]	67.9	61
1194	**4**{*394*,*603*,*12*}	COC(=O)C1=CC(=NS1)C1=C(NCC2CCOC2)N2C(C=CC(C=C)=C2F)=N1 |c:6,10,22,26,29,t:4|	Z8873685487	**1**{*394*}	Nc1ccc(C=C)c(F)n1	**2**{*603*}	COC(=O)c1cc(C=O)ns1	**3**{*12*}	[C-]#[N+]CC1CCOC1	66.3	61
1195	**4**{*50*,*604*,*7*}	CCN1N=C(C2=C1C=CC=C2)C1=C(NC2CCOCC2)N2C(C=CC=C2P(C)(C)=O)=N1 |c:3,5,8,10,13,25,27,33|	Z8873684666	**1**{*50*}	CP(=O)(C)c1cccc(N)n1	**2**{*604*}	CCn1nc(C=O)c2ccccc12	**3**{*7*}	[C-]#[N+]C1CCOCC1	71.3	60

1196	**4**{*28*,*340*,*18*}	CCN1C=NC(=N1)C1=C(NC(C)(C)C)N2C=CC(=CC2=N1)C1=NOC=N1 |c:3,5,8,16,18,21,27,t:24|	Z8878918333	**1**{*28*}	Cl.Nc1cc(ccn1)c2ncon2	**2**{*340*}	CCn1cnc(C=O)n1	**3**{*18*}	CC(C)(C)[N+]#[C-]	57.4	60
1197	**4**{*142*,*605*,*10*}	COC1=C(C=CC(=C1)C(F)F)C1=C(NC2CCOC2)N2C=CC=C(OCCS(C)(=O)=O)C2=N1 |c:4,6,12,22,34,t:2,24|	Z8878918427	**1**{*142*}	CS(=O)(=O)CCOc1cccnc1N	**2**{*605*}	COc1cc(ccc1C=O)C(F)F	**3**{*10*}	[C-]#[N+]C1CCOC1	78.3	60
1198	**4**{*431*,*16*,*10*}	CN1N=CC(CNC(=O)OC(C)(C)C)=C1C1=C(NC2CCOC2)N2C=C(F)C=CC2=N1 |c:2,13,16,29,32,t:26|	Z8878918826	**1**{*431*}	Nc1ccc(F)cn1	**2**{*16*}	Cn1ncc(CNC(=O)OC(C)(C)C)c1C=O	**3**{*10*}	[C-]#[N+]C1CCOC1	69.8	60
1199	**4**{*431*,*606*,*10*}	CC(C)(C)OC(=O)N1CC(C2=C(NC3CCOC3)N3C=C(F)C=CC3=N2)C2(CC2)C1 |c:10,23,26,t:20|	Z8878918807	**1**{*431*}	Nc1ccc(F)cn1	**2**{*606*}	CC(C)(C)OC(=O)N1CC(C=O)C2(CC2)C1	**3**{*10*}	[C-]#[N+]C1CCOC1	67.5	60
1200	**4**{*125*,*64*,*31*}	CSCCNC1=C(N=C2C=CC(CCO)=CN12)C1=CC=C(C=C1)N1C=CN=C1 |c:9,14,21,23,27,29,t:5,7,19|	Z8873684773	**1**{*125*}	Nc1ccc(CCO)cn1	**2**{*64*}	O=Cc1ccc(cc1)n2ccnc2	**3**{*31*}	CSCC[N+]#[C-]	63.7	60
1201	**4**{*69*,*328*,*21*}	CCC1=NNC=C1C1=C(NCC2CC2)N2C=CC(=CC2=N1)C(=O)NC |c:5,8,17,19,22,t:2|	Z8878918912	**1**{*69*}	CNC(=O)c1ccnc(N)c1	**2**{*328*}	Cl.CCc1n[nH]cc1C=O	**3**{*21*}	[C-]#[N+]CC1CC1	54.7	60
1202	**4**{*73*,*607*,*20*}	COC1=CC(C)=C(NC2=C(N=C3C=CC4=NC=CN4N23)C2=CN(N=C2)C(C)(C)C)C=C1 |c:12,16,26,33,t:2,5,8,10,14,23|	Z8878918447	**1**{*73*}	Nc1ccc2nccn2n1	**2**{*607*}	CC(C)(C)n1cc(C=O)cn1	**3**{*20*}	COc1ccc([N+]#[C-])c(C)c1	66.8	60
1203	**4**{*142*,*608*,*10*}	CS(=O)(=O)CCOC1=CC=CN2C(NC3CCOC3)=C(CC3(CCC3)C#N)N=C12 |c:9,t:7,19,29|	Z8878918597	**1**{*142*}	CS(=O)(=O)CCOc1cccnc1N	**2**{*608*}	O=CCC1(CCC1)C#N	**3**{*10*}	[C-]#[N+]C1CCOC1	66.7	59
1204	**4**{*164*,*609*,*43*}	CN(C)CC1=CC2=NC(=C(NC3CCN(CC3)C(=O)OC(C)(C)C)N2C=C1)C1=CSC=N1 |c:27,33,t:4,6,8,30|	Z8878918897	**1**{*164*}	CN(C)Cc1ccnc(N)c1	**2**{*609*}	O=Cc1cscn1	**3**{*43*}	CC(C)(C)OC(=O)N1CCC(CC1)[N+]#[C-]	72.6	59
1205	**4**{*353*,*610*,*15*}	CCNC1=C(N=C2C=CC(NS(C)(=O)=O)=CN12)C1=C(F)C(F)=C(C=C1)C(=O)OC |c:7,14,19,23,25,t:3,5|	Z8873684834	**1**{*353*}	Cl.CS(=O)(=O)Nc1ccc(N)nc1	**2**{*610*}	COC(=O)c1ccc(C=O)c(F)c1F	**3**{*15*}	CC[N+]#[C-]	67.4	59
1206	**4**{*52*,*297*,*7*}	CCN1N=C(C)C(=N1)C1=C(NC2CCOCC2)N2C=C(C=CC2=N1)C1(CC1)C(F)(F)F |c:6,9,20,22,25,t:3|	Z8878918531	**1**{*52*}	Nc1ccc(cn1)C2(CC2)C(F)(F)F	**2**{*297*}	CCn1nc(C)c(C=O)n1	**3**{*7*}	[C-]#[N+]C1CCOCC1	68.9	59

1207	**4**{*125*,*124*,*31*}	COC1=C(OCC2=C(C)ON=C2C)C=CC(=C1)C1=C(NCCSC)N2C=C(CCO)C=CC2=N1 |c:2,6,10,14,16,19,32,35,t:27|	Z8873684793	**1**{*125*}	Nc1ccc(CCO)cn1	**2**{*124*}	COc1cc(C=O)ccc1OCc2c(C)noc2C	**3**{*31*}	CSCC[N+]#[C-]	75.9	58
1208	**4**{*64*,*611*,*49*}	COC1=CC(=CC(OC)=C1)C1=C(NC2CC(F)(F)C2)N2C=C(C=CC2=N1)N1CCCS1(=O)=O |c:4,8,11,22,24,27,t:2|	Z8878918362	**1**{*64*}	Nc1ccc(cn1)N2CCCS2(=O)=O	**2**{*611*}	COc1cc(OC)cc(C=O)c1	**3**{*49*}	FC1(F)CC(C1)[N+]#[C-]	75.1	58
1209	**4**{*350*,*165*,*5*}	COCCCNC1=C(N=C2C=NC(=CN12)C1=CC=CC=C1)C1=NN(C=C1)[C@H]1C[C@@H](C1)C(=O)OC |r,c:10,12,19,21,27,t:6,8,17,24|	Z8873684588	**1**{*350*}	Nc1cnc(cn1)c2ccccc2	**2**{*165*}	COC(=O)[C@H]1C[C@@H](C1)n2ccc(C=O)n2	**3**{*5*}	COCCC[N+]#[C-]	72.2	58
1210	**4**{*125*,*612*,*7*}	CN(C)C1=C(F)C=C(C2=C(NC3CCOCC3)N3C=C(CCO)C=CC3=N2)C(F)=C1 |c:3,8,24,27,31,t:6,19|	Z8873685302	**1**{*125*}	Nc1ccc(CCO)cn1	**2**{*612*}	CN(C)c1cc(F)c(C=O)cc1F	**3**{*7*}	[C-]#[N+]C1CCOCC1	65.2	58
1211	**4**{*433*,*472*,*7*}	COC(=O)C1=CC2=C(C=NN2C=C1)C1=C(NC2CCOCC2)N2C(C=CC(C#C)=C2C)=N1 |c:8,12,15,27,31,34,t:4,6|	Z8873685352	**1**{*433*}	Cc1nc(N)ccc1C#C	**2**{*472*}	COC(=O)c1ccn2ncc(C=O)c2c1	**3**{*7*}	[C-]#[N+]C1CCOCC1	67.2	58
1212	**4**{*50*,*613*,*7*}	CN1C=C2C=CC=C(C2=N1)C1=C(NC2CCOCC2)N2C(C=CC=C2P(C)(C)=O)=N1 |c:4,6,9,12,24,26,32,t:2|	Z8878918464	**1**{*50*}	CP(=O)(C)c1cccc(N)n1	**2**{*613*}	Cn1cc2cccc(C=O)c2n1	**3**{*7*}	[C-]#[N+]C1CCOCC1	65.9	58
1213	**4**{*371*,*614*,*12*}	CC(C)CN1C=C(C=N1)C1=C(NCC2CCOC2)N2C=CC=C(OCCCC#N)C2=N1 |c:5,7,10,21,32,t:23|	Z8878918368	**1**{*371*}	Nc1ncccc1OCCCC#N	**2**{*614*}	CC(C)Cn1cc(C=O)cn1	**3**{*12*}	[C-]#[N+]CC1CCOC1	65.7	58
1214	**4**{*362*,*486*,*12*}	CNC(=O)C1=CC=CC2=NC(=C(NCC3CCOC3)N12)C1=C(Cl)N=C(Cl)N1C |c:6,23,t:4,8,10,26|	Z8878918802	**1**{*362*}	CNC(=O)c1cccc(N)n1	**2**{*486*}	Cn1c(Cl)nc(Cl)c1C=O	**3**{*12*}	[C-]#[N+]CC1CCOC1	65.8	58
1215	**4**{*71*,*615*,*21*}	IC1=CN2C(C=N1)=NC(C1=CN(N=C1)C1=NC=CC=C1)=C2NCC1CC1 |c:5,7,13,18,20,22,t:1,10,16|	Z8878918800	**1**{*71*}	Nc1cnc(I)cn1	**2**{*615*}	O=Cc1cnn(c1)c2ccccn2	**3**{*21*}	[C-]#[N+]CC1CC1	71.0	58
1216	**4**{*92*,*616*,*7*}	CCOC1=C(OC(F)F)C=CC(=C1)C1=C(NC2CCOCC2)N2C=CC(=CC2=N1)C(N)=O |c:3,9,11,14,25,27,30|	Z8873685472	**1**{*92*}	NC(=O)c1ccnc(N)c1	**2**{*616*}	CCOc1cc(C=O)ccc1OC(F)F	**3**{*7*}	[C-]#[N+]C1CCOCC1	69.3	57
1217	**4**{*64*,*617*,*49*}	CC(C)C1=NNC(=C1)C1=C(NC2CC(F)(F)C2)N2C=C(C=CC2=N1)N1CCCS1(=O)=O |c:6,9,20,22,25,t:3|	Z8846491763	**1**{*64*}	Nc1ccc(cn1)N2CCCS2(=O)=O	**2**{*617*}	CC(C)c1cc(C=O)[nH]n1	**3**{*49*}	FC1(F)CC(C1)[N+]#[C-]	69.7	57
1218	**4**{*362*,*618*,*10*}	CNC(=O)C1=CC=CC2=NC(=C(NC3CCOC3)N12)C1=C(C)C=C(C=C1)C(F)F |c:6,22,25,27,t:4,8,10|	Z8835022880	**1**{*362*}	CNC(=O)c1cccc(N)n1	**2**{*618*}	Cc1cc(ccc1C=O)C(F)F	**3**{*10*}	[C-]#[N+]C1CCOC1	61.9	57

1219	**4**{*164*,*619*,*12*}	COC1=C(SC)C=CC(=C1)C1=C(NCC2CCOC2)N2C=CC(CN(C)C)=CC2=N1 |c:2,6,8,11,22,28,31|	Z8878918823	**1**{*164*}	CN(C)Cc1ccnc(N)c1	**2**{*619*}	COc1cc(C=O)ccc1SC	**3**{*12*}	[C-]#[N+]CC1COC1	65.9	57
1220	**4**{*412*,*531*,*12*}	CC1=NC=C(C=N1)C1=C(NCC2CCOC2)N2C=C(Br)C(CO)=CC2=N1 |c:3,5,8,24,27,t:1,19|	Z8873685276	**1**{*412*}	Nc1cc(CO)c(Br)cn1	**2**{*531*}	Cc1ncc(C=O)cn1	**3**{*12*}	[C-]#[N+]CC1COC1	64.5	57
1221	**4**{*69*,*33*,*51*}	CNC(=O)C1=CC2=NC(C3CCCN(C3)C(C)=O)=C(NCCNC(=O)OC(C)(C)C)N2C=C1 |c:33,t:4,6,18|	Z8829498629	**1**{*69*}	CNC(=O)c1ccnc(N)c1	**2**{*33*}	CC(=O)N1CCCC(C1)C=O	**3**{*51*}	CC(C)(C)OC(=O)NCC[N+]#[C-]	70.7	57
1222	**4**{*386*,*42*,*5*}	COCCCNC1=C(N=C2C=C(C)C=CN12)C1=CN(C)N=C1Cl |c:13,22,t:6,8,10,18|	Z8855739213	**1**{*386*}	Cc1ccnc(N)c1	**2**{*42*}	Cn1cc(C=O)c(Cl)n1	**3**{*5*}	COCCC[N+]#[C-]	51.3	57
1223	**4**{*286*,*620*,*5*}	COCCCNC1=C(N=C2C=CC(=CN12)C1=NC=CC=C1)C1=C(Cl)N(C)N=C1 |c:10,12,19,21,24,29,t:6,8,17|	Z8873684813	**1**{*286*}	Cl.Cl.Nc1ccc(cn1)c2ccccn2	**2**{*620*}	Cn1ncc(C=O)c1Cl	**3**{*5*}	COCCC[N+]#[C-]	61.0	57
1224	**4**{*107*,*621*,*43*}	CC1=C(N=CC=C1)C1=C(NC2CCN(CC2)C(=O)OC(C)(C)C)N2C=C(OCCO)C=CC2=N1 |c:3,5,8,32,35,t:1,26|	Z8878918760	**1**{*107*}	Nc1ccc(OCCO)cn1	**2**{*621*}	Cc1cccnc1C=O	**3**{*43*}	CC(C)(C)OC(=O)N1CCC(CC1)[N+]#[C-]	71.8	57
1225	**4**{*333*,*219*,*23*}	COC(=O)CCC(NC1=C(N=C2C=CC=C(N12)C1=CC=NC=C1)C1=CN(C)C(=C1)C#N)C(=O)OC |c:12,14,21,23,30,t:8,10,19,26|	Z8878918787	**1**{*333*}	Nc1cccc(n1)c2ccncc2	**2**{*219*}	Cn1cc(C=O)cc1C#N	**3**{*23*}	COC(=O)CCC([N+]#[C-])C(=O)OC	72.6	57
1226	**4**{*434*,*54*,*7*}	COC1=C(C=CS1)C1=C(NC2CCOCC2)N2C=C(C=CC2=N1)C(C)(F)F |c:4,8,19,21,24,t:2|	Z8878918451	**1**{*434*}	CC(F)(F)c1ccc(N)nc1	**2**{*54*}	COc1sccc1C=O	**3**{*7*}	[C-]#[N+]C1CCOCC1	60.2	57
1227	**4**{*371*,*622*,*12*}	CCNC(=O)COC1=CC=C(C=C1)C1=C(NCC2COC2)N2C=CC=C(OCCCC#N)C2=N1 |c:9,11,14,25,36,t:7,27|	Z8873684585	**1**{*371*}	Nc1ncccc1OCCCC#N	**2**{*622*}	CCNC(=O)COc1ccc(C=O)cc1	**3**{*12*}	[C-]#[N+]CC1COC1	73.0	57
1228	**4**{*167*,*623*,*5*}	COCCCNC1=C(N=C2N1C(C)=CN=C2C)C1=C(OC)C=C(OCC#N)C=C1 |c:8,13,15,19,29,t:6,23|	Z8878918766	**1**{*167*}	Cc1cnc(C)c(N)n1	**2**{*623*}	COc1cc(OCC#N)ccc1C=O	**3**{*5*}	COCCC[N+]#[C-]	60.5	57
1229	**4**{*48*,*624*,*15*}	CCNC1=C(N=C2C=C(C=CN12)S(C)(=O)=O)C1=C(C)NN=C1Br |c:7,9,18,22,t:3,5|	Z8846492000	**1**{*48*}	CS(=O)(=O)c1ccnc(N)c1	**2**{*624*}	Cc1[nH]nc(Br)c1C=O	**3**{*15*}	CC[N+]#[C-]	60.9	57
1230	**4**{*187*,*164*,*12*}	CN1N=C(C2=C1CCOC2)C1=C(NCC2CCOC2)N2C=C(Cl)C(=CC2=N1)C#N |c:2,4,12,26,29,t:23|	Z8878918577	**1**{*187*}	Nc1cc(C#N)c(Cl)cn1	**2**{*164*}	Cn1nc(C=O)c2COCCc21	**3**{*12*}	[C-]#[N+]CC1COC1	63.1	57

<table>
<tr><td>1231</td><td>4{357,452,49}</td><td>NC(=O)C1=CC=C(C=C1)C1=C(NC2CC(F)(F)C2)N2C=C(C=CC2=N1)N1CCC(CO)CC1 |c:5,7,10,21,23,26,t:3|</td><td>Z8873685383</td><td>1{357}</td><td>Nc1ccc(cn1)N2CCC(CO)CC2</td><td>2{452}</td><td>NC(=O)c1ccc(C=O)cc1</td><td>3{49}</td><td>FC1(F)CC(C1)[N+]#[C-]</td><td>69.6</td><td>57</td></tr>
<tr><td>1232</td><td>4{92,361,7}</td><td>NC(=O)C1=CC2=NC(=C(NC3CCOCC3)N2C=C1)C1=C(Br)C=CC(Br)=N1 |c:19,22,25,28,t:3,5,7|</td><td>Z8837933172</td><td>1{92}</td><td>NC(=O)c1ccnc(N)c1</td><td>2{361}</td><td>Brc1ccc(Br)c(C=O)n1</td><td>3{7}</td><td>[C-]#[N+]C1CCOCC1</td><td>75.7</td><td>57</td></tr>
<tr><td>1233</td><td>4{212,425,49}</td><td>COCC1=CN2C(C=C1)=NC(C1CCCN(C1)S(C)(=O)=O)=C2NC1CC(F)(F)C1 |c:7,9,22,t:3|</td><td>Z8878918794</td><td>1{212}</td><td>Cl.COCc1ccc(N)nc1</td><td>2{425}</td><td>CS(=O)(=O)N1CCCC(C1)C=O</td><td>3{49}</td><td>FC1(F)CC(C1)[N+]#[C-]</td><td>65.5</td><td>57</td></tr>
<tr><td>1234</td><td>4{435,219,23}</td><td>COC(=O)CCC(NC1=C(N=C2N1C=C(Br)C=C2C)C1=CN(C)C(=C1)C#N)C(=O)OC |c:10,17,25,t:8,14,21|</td><td>Z8873685363</td><td>1{435}</td><td>Cc1cc(Br)cnc1N</td><td>2{219}</td><td>Cn1cc(C=O)cc1C#N</td><td>3{23}</td><td>COC(=O)CCC([N+]#[C-])C(=O)OC</td><td>74.6</td><td>57</td></tr>
<tr><td>1235</td><td>4{118,625,31}</td><td>COC1=C(OCC2=CN=CC=C2)C=C(C=C1)C1=C(NCCSC)N2C(C=CC=C2C(C)O)=N1 |c:2,8,10,13,15,18,27,29,34,t:6|</td><td>Z8878918332</td><td>1{118}</td><td>Cl.CC(O)c1cccc(N)n1</td><td>2{625}</td><td>COc1ccc(C=O)cc1OCc2cccnc2</td><td>3{31}</td><td>CSCC[N+]#[C-]</td><td>70.8</td><td>56</td></tr>
<tr><td>1236</td><td>4{69,392,46}</td><td>CNC(=O)C1=CC2=NC(C3CCOCC3)=C(NCC(C)=C)N2C=C1 |c:24,t:4,6,15|</td><td>Z8878918588</td><td>1{69}</td><td>CNC(=O)c1ccnc(N)c1</td><td>2{392}</td><td>O=CC1CCOCC1</td><td>3{46}</td><td>CC(=C)C[N+]#[C-]</td><td>49.8</td><td>56</td></tr>
<tr><td>1237</td><td>4{62,243,6}</td><td>CCOC(=O)CCCNC1=C(N=C2C=C(CO)C=CN12)C1=CON=C1CC |c:17,25,t:9,11,13,22|</td><td>Z8835022875</td><td>1{62}</td><td>Nc1cc(CO)ccn1</td><td>2{243}</td><td>CCc1nocc1C=O</td><td>3{6}</td><td>CCOC(=O)CCC[N+]#[C-]</td><td>56.5</td><td>56</td></tr>
<tr><td>1238</td><td>4{436,585,23}</td><td>CCC1=CC2=NC(CC3CC3(Cl)Cl)=C(NC(CCC(=O)OC)C(=O)OC)N2C=C1 |c:29,t:2,4,13|</td><td>Z8878918311</td><td>1{436}</td><td>CCc1ccnc(N)c1</td><td>2{585}</td><td>ClC1(Cl)CC1CC=O</td><td>3{23}</td><td>COC(=O)CCC([N+]#[C-])C(=O)OC</td><td>67.1</td><td>56</td></tr>
<tr><td>1239</td><td>4{40,626,22}</td><td>CC(=O)N1CCC2=C(C1)C=C(S2)C1=C(NC2COC2)N2C(C=C(C=C2C)C(F)(F)F)=N1 |c:6,10,14,24,26,33|</td><td>Z8878918861</td><td>1{40}</td><td>Cc1cc(cc(N)n1)C(F)(F)F</td><td>2{626}</td><td>CC(=O)N1CCc2sc(C=O)cc2C1</td><td>3{22}</td><td>[C-]#[N+]C1COC1</td><td>63.3</td><td>56</td></tr>
<tr><td>1240</td><td>4{107,627,61}</td><td>CCC1=NC(C)=C(N1)C1=C(NCCCNC(=O)OC(C)(C)C)N2C=C(OCCO)C=CC2=N1 |c:5,9,30,33,t:2,24|</td><td>Z8878918682</td><td>1{107}</td><td>Nc1ccc(OCCO)cn1</td><td>2{627}</td><td>CCc1nc(C)c(C=O)[nH]1</td><td>3{61}</td><td>CC(C)(C)OC(=O)NCCC[N+]#[C-]</td><td>69.3</td><td>56</td></tr>
<tr><td>1241</td><td>4{187,628,10}</td><td>COCC1=C(C=CC=C1)C1=C(NC2CCOC2)N2C=C(Cl)C(=CC2=N1)C#N |c:5,7,10,23,26,t:3,20|</td><td>Z8878918779</td><td>1{187}</td><td>Nc1cc(C#N)c(Cl)cn1</td><td>2{628}</td><td>COCc1ccccc1C=O</td><td>3{10}</td><td>[C-]#[N+]C1CCOC1</td><td>57.7</td><td>56</td></tr>
<tr><td>1242</td><td>4{351,629,39}</td><td>CCOCCNC1=C(N=C2C=CC3=C(C(O)CC3)N12)C1=C(C)C=C(C)C=N1 |c:10,22,28,t:6,8,12,25|</td><td>Z8873684794</td><td>1{351}</td><td>Nc1ccc2CCC(O)c2n1</td><td>2{629}</td><td>Cc1cnc(C=O)c(C)c1</td><td>3{39}</td><td>CCOCC[N+]#[C-]</td><td>55.2</td><td>56</td></tr>
<tr><td>1243</td><td>4{76,630,23}</td><td>COCCN1C=C(C=N1)C1=C(NC(CCC(=O)OC)C(=O)OC)N2C(C=CC(C)=C2Cl)=N1 |c:5,7,10,26,29,32|</td><td>Z8855739199</td><td>1{76}</td><td>Cc1ccc(N)nc1Cl</td><td>2{630}</td><td>COCCn1cc(C=O)cn1</td><td>3{23}</td><td>COC(=O)CCC([N+]#[C-])C(=O)OC</td><td>69.8</td><td>56</td></tr>
</table>

1244	**4**{*437,586,67*}	CCC(C)NC1=C(N=C2C=CC3=C(NC(=N3)C(F)(F)F)N12)C1=CC2=C(COC2)N=C1 |c:9,14,32,t:5,7,11,24,26|	Z8835022891	**1**{*437*}	Cl.Nc1ccc2nc([nH]c2n1)C(F)(F)F	**2**{*586*}	O=Cc1cnc2COCc2c1	**3**{*67*}	CCC(C)[N+]#[C-]	62.7	56
1245	**4**{*112,304,61*}	COC1=CN=CC2=NC(=C(NCCCNC(=O)OC(C)(C)C)N12)C1=C(C)N(CC(F)F)N=C1 |c:4,25,33,t:2,6,8|	Z8829498762	**1**{*112*}	COc1cncc(N)n1	**2**{*304*}	Cc1c(C=O)cnn1CC(F)F	**3**{*61*}	CC(C)(C)OC(=O)NCCC[N+]#[C-]	70.0	56
1246	**4**{*180,206,22*}	COC(=O)C1=CC2=NC(C[C@H](C)NC(=O)OC(C)(C)C)=C(NC3COC3)N2C=C1C |c:29,t:4,6,19|	Z8878918791	**1**{*180*}	COC(=O)c1cc(N)ncc1C	**2**{*206*}	C[C@@H](CC=O)NC(=O)OC(C)(C)C	**3**{*22*}	[C-]#[N+]C1COC1	59.5	56
1247	**4**{*109,631,34*}	COC1=NC=C(C2=C(NCC3CCCO3)N3C(C=CC=C3S(N)(=O)=O)=N2)C(OC)=C1 |c:6,18,20,26,31,t:2,4|	Z8837933179	**1**{*109*}	Cl.Nc1cccc(n1)S(=O)(=O)N	**2**{*631*}	COc1cc(OC)c(C=O)cn1	**3**{*34*}	[C-]#[N+]CC1CCCO1	65.0	56
1248	**4**{*363,305,38*}	COCC1=NC(=CN1)C1=C(NC2=CC3=C(OCO3)C=C2)N2C=CC(=CC2=N1)C(=O)OC |c:5,9,20,24,26,29,t:3,12,14|	Z8849597828	**1**{*363*}	COC(=O)c1ccnc(N)c1	**2**{*305*}	COCc1nc(C=O)c[nH]1	**3**{*38*}	[C-]#[N+]c1ccc2OCOc2c1	63.0	55
1249	**4**{*384,399,10*}	CCOC1=C(C)N2C(NC3CCOC3)=C(N=C2C=C1)C1=CN(N=C1C)C1CC1 |c:3,14,16,19,25,t:22|	Z8878918784	**1**{*384*}	CCOc1ccc(N)nc1C	**2**{*399*}	Cc1nn(cc1C=O)C2CC2	**3**{*10*}	[C-]#[N+]C1CCOC1	57.1	55
1250	**4**{*212,632,49*}	COCC1=CN2C(C=C1)=NC(=C2NC1CC(F)(F)C1)C1=C(OC)C=CC(F)=C1 |c:7,9,11,22,26,29,t:3|	Z8855739201	**1**{*212*}	Cl.COCc1ccc(N)nc1	**2**{*632*}	COc1ccc(F)cc1C=O	**3**{*49*}	FC1(F)CC(C1)[N+]#[C-]	58.5	55
1251	**4**{*105,357,34*}	CCC1=C(C(C)=NO1)C1=C(NCC2CCCO2)N2C(C=CC=C2C(N)=O)=N1 |c:5,9,21,23,28,t:2|	Z8837933175	**1**{*105*}	NC(=O)c1cccc(N)n1	**2**{*357*}	CCc1onc(C)c1C=O	**3**{*34*}	[C-]#[N+]CC1CCCO1	55.2	55
1252	**4**{*327,633,41*}	COC(=O)C1=C(F)C2=NC(=C(NC3=CC=C(OC)C=C3)N2C=C1)C1=CNN=C1OC |c:4,18,23,29,t:7,9,12,14,26|	Z8878918608	**1**{*327*}	COC(=O)c1ccnc(N)c1F	**2**{*633*}	COc1n[nH]cc1C=O	**3**{*41*}	COc1ccc([N+]#[C-])cc1	61.5	55
1253	**4**{*42,153,7*}	CSC1=CN2C(NC3CCOCC3)=C(N=C2C=C1)C1=CN(CC(F)(F)F)N=N1 |c:13,15,18,29,t:2,21|	Z8855619755	**1**{*42*}	CSc1ccc(N)nc1	**2**{*153*}	FC(F)(F)Cn1cc(C=O)nn1	**3**{*7*}	[C-]#[N+]C1CCOCC1	61.6	55
1254	**4**{*187,314,51*}	CC(C)(C)OC(=O)NCCNC1=C(N=C2C=C(C#N)C(Cl)=CN12)C1=CNN=C1 |c:20,28,t:11,13,15,25|	Z8873685419	**1**{*187*}	Nc1cc(C#N)c(Cl)cn1	**2**{*314*}	O=Cc1cn[nH]c1	**3**{*51*}	CC(C)(C)OC(=O)NCC[N+]#[C-]	59.9	55
1255	**4**{*368,425,49*}	CN1C=C(C=N1)C1=C(C)N2C(C=C1)=NC(C1CCCN(C1)S(C)(=O)=O)=C2NC1CC(F)(F)C1 |c:2,4,7,12,14,27|	Z8878918422	**1**{*368*}	Cc1nc(N)ccc1c2cnn(C)c2	**2**{*425*}	CS(=O)(=O)N1CCCC(C1)C=O	**3**{*49*}	FC1(F)CC(C1)[N+]#[C-]	71.4	55

1256	**4**{*118*,*634*,*31*}	CSCCNC1=C(N=C2C=CC=C(C(C)O)N12)C(C)C1CCOCC1 |c:9,t:5,7,11|	Z8873684888	**1**{*118*}	Cl.CC(O)c1cccc(N)n1	**2**{*634*}	CC(C=O)C1CCOCC1	**3**{*31*}	CSCC[N+]#[C-]	54.2	55
1257	**4**{*109*,*635*,*34*}	CCOC(=O)C1=COC(=C1)C1=C(NCC2CCCO2)N2C(C=CC=C2S(N)(=O)=O)=N1 |c:8,11,23,25,31,t:5|	Z8878918497	**1**{*109*}	Cl.Nc1cccc(n1)S(=O)(=O)N	**2**{*635*}	CCOC(=O)c1coc(C=O)c1	**3**{*34*}	[C-]#[N+]CC1CCCO1	64.7	55
1258	**4**{*28*,*310*,*18*}	CC(C)(C)NC1=C(N=C2C=C(C=CN12)C1=NOC=N1)C1CCOC2(CCC2)C1 |c:9,11,19,t:5,7,16|	Z8878918576	**1**{*28*}	Cl.Nc1cc(ccn1)c2ncon2	**2**{*310*}	O=CC1CCOC2(CCC2)C1	**3**{*18*}	CC(C)(C)[N+]#[C-]	56.8	55
1259	**4**{*80*,*233*,*62*}	COC(=O)C1(CCCC1)NC1=C(CCCOCC=C)N=C2N1C=CN=C2OC |c:11,20,24,26|	Z8855739111	**1**{*80*}	COc1nccnc1N	**2**{*233*}	C=CCOCCC=O	**3**{*62*}	COC(=O)C1(CCCC1)[N+]#[C-]	57.9	55
1260	**4**{*353*,*138*,*53*}	CC1=NC(C)=NC(=C1)C1=C(NCCOCC2=CC=CC=C2)N2C=C(NS(C)(=O)=O)C=CC2=N1 |c:4,6,9,18,20,31,34,t:1,16,24|	Z8878918620	**1**{*353*}	Cl.CS(=O)(=O)Nc1ccc(N)nc1	**2**{*138*}	Cc1cc(C=O)nc(C)n1	**3**{*53*}	[C-]#[N+]CCOCc1ccccc1	69.5	55
1261	**4**{*64*,*636*,*33*}	COC1=C(CNC2=C(N=C3C=CC(=CN23)N2CCCS2(=O)=O)C2=CN(C)N=C2)C=CC=C1 |c:2,10,12,29,32,34,t:6,8,25|	Z8878918493	**1**{*64*}	Nc1ccc(cn1)N2CCCS2(=O)=O	**2**{*636*}	Cn1cc(C=O)cn1	**3**{*33*}	COc1ccccc1C[N+]#[C-]	67.3	55
1262	**4**{*104*,*637*,*46*}	CC(=C)CNC1=C(N=C2C=CC3=C(COCC3)N12)C1=CC2=C(C=C1)S(=O)(=O)CCC2 |c:9,23,25,t:5,7,11,21|	Z8835022899	**1**{*104*}	Nc1ccc2CCOCc2n1	**2**{*637*}	O=Cc1ccc2c(CCCS2(=O)=O)c1	**3**{*46*}	CC(=C)C[N+]#[C-]	63.0	55
1263	**4**{*438*,*314*,*12*}	CCC1=C(Br)C=CC2=NC(C3=CNN=C3)=C(NCC3CCOC3)N12 |c:2,5,13,t:7,10,15|	Z8878918792	**1**{*438*}	CCc1nc(N)ccc1Br	**2**{*314*}	O=Cc1cn[nH]c1	**3**{*12*}	[C-]#[N+]CC1COC1	58.0	55
1264	**4**{*99*,*638*,*34*}	NS(=O)(=O)C1=CN2C(NCC3CCCO3)=C(N=C2C=C1)C1=CN(CC2CCOCC2)N=C1 |c:15,17,20,34,t:4,23|	Z8855739112	**1**{*99*}	Nc1ccc(cn1)S(=O)(=O)N	**2**{*638*}	O=Cc1cnn(CC2CCOCC2)c1	**3**{*34*}	[C-]#[N+]CC1CCCO1	68.4	55
1265	**4**{*123*,*277*,*22*}	CC(C)(C)OC(=O)NC(CC1=C(NC2COC2)N2C=CC(OCCO)=CC2=N1)C1CC1 |c:10,19,25,28|	Z8878918908	**1**{*123*}	Cl.Nc1cc(OCCO)ccn1	**2**{*277*}	CC(C)(C)OC(=O)NC(CC=O)C1CC1	**3**{*22*}	[C-]#[N+]C1COC1	59.2	55
1266	**4**{*91*,*639*,*7*}	CC1=NN=C(O1)C1=CC(=CC=C1)C1=C(NC2CCOCC2)N2C=C(C=CC2=N1)C(N)=O |c:3,9,11,14,25,27,30,t:1,7|	Z8873684799	**1**{*91*}	NC(=O)c1ccc(N)nc1	**2**{*639*}	Cc1nnc(o1)c2cccc(C=O)c2	**3**{*7*}	[C-]#[N+]C1CCOCC1	62.1	55
1267	**4**{*49*,*63*,*13*}	COCN1N=CC=C1C1=C(NCC2=CC=C(OC)C=C2)N2C(C=CC=C2CO)=N1 |c:4,6,9,19,24,26,30,t:13,15|	Z8873684609	**1**{*49*}	Nc1cccc(CO)n1	**2**{*63*}	COCn1nccc1C=O	**3**{*13*}	COc1ccc(C[N+]#[C-])cc1	58.4	55

1268	**4**{*97*,*280*,*49*}	CC1=CC(F)=CN2C(NC3CC(F)(F)C3)=C(N=C12)C1=NSC(=C1)[N+]([O-])=O |c:4,15,24,t:1,17,21|	Z8837933162	**1**{*97*}	Cc1cc(F)cnc1N	**2**{*280*}	[O-][N+](=O)c1cc(C=O)ns1	**3**{*49*}	FC1(F)CC(C1)[N+]#[C-]	56.9	55
1269	**4**{*69*,*596*,*61*}	CNC(=O)C1=CC2=NC(=C(NCCCNC(=O)OC(C)(C)C)N2C=C1)C1=CC2=C(N=C1)N(C)N=C2C |c:24,29,31,36,t:4,6,8,27|	Z8873685321	**1**{*69*}	CNC(=O)c1ccnc(N)c1	**2**{*596*}	Cc1nn(C)c2ncc(C=O)cc12	**3**{*61*}	CC(C)(C)OC(=O)NCCC[N+]#[C-]	73.1	55
1270	**4**{*32*,*640*,*7*}	CC(C)N1C=C(N=N1)C1=C(NC2CCOCC2)N2C=CC(=CC2=N1)P(C)(C)=O |c:4,6,9,20,22,25|	Z8878918430	**1**{*32*}	CP(=O)(C)c1ccnc(N)c1	**2**{*640*}	CC(C)n1cc(C=O)nn1	**3**{*7*}	[C-]#[N+]C1CCOCC1	59.6	55
1271	**4**{*37*,*641*,*10*}	CC1=NC(C)=C(S1)C1=C(NC2CCOC2)N2C(C=CC=C2C(F)F)=N1 |c:4,8,19,21,26,t:1|	Z8878918319	**1**{*37*}	Cl.Nc1cccc(n1)C(F)F	**2**{*641*}	Cc1nc(C)c(C=O)s1	**3**{*10*}	[C-]#[N+]C1CCOC1	54.0	55
1272	**4**{*77*,*35*,*23*}	COC(=O)CCC(NC1=C(N=C2C=CC(=CN12)C1=CN=CC=C1)C1=NN(C)C(C)=C1Cl)C(=O)OC |c:12,14,21,23,31,t:8,10,19,26|	Z8854581168	**1**{*77*}	Nc1ccc(cn1)c2cccnc2	**2**{*35*}	Cc1c(Cl)c(C=O)nn1C	**3**{*23*}	COC(=O)CCC([N+]#[C-])C(=O)OC	73.5	55
1273	**4**{*49*,*112*,*6*}	CCOC(=O)CCCNC1=C(N=C2C=CC=C(CO)N12)C1=C2C=CC=NN2N=C1 |c:13,22,24,26,30,t:9,11,15|	Z8849597835	**1**{*49*}	Nc1cccc(CO)n1	**2**{*112*}	O=Cc1cnn2ncccc12	**3**{*6*}	CCOC(=O)CCC[N+]#[C-]	58.3	55
1274	**4**{*92*,*642*,*7*}	CC1=C(C=C(Br)S1)C1=C(NC2CCOCC2)N2C=CC(=CC2=N1)C(N)=O |c:8,19,21,24,t:1,3|	Z8837933167	**1**{*92*}	NC(=O)c1ccnc(N)c1	**2**{*642*}	Cc1sc(Br)cc1C=O	**3**{*7*}	[C-]#[N+]C1CCOCC1	64.4	55
1275	**4**{*439*,*164*,*12*}	CN1N=C(C2=C1CCOC2)C1=C(NCC2CCOC2)N2C=CC=C(OCC3=CC=CC=C3)C2=N1 |c:2,4,12,23,31,33,37,t:25,29|	Z8878918613	**1**{*439*}	Nc1ncccc1OCc2ccccc2	**2**{*164*}	Cn1nc(C=O)c2COCCc21	**3**{*12*}	[C-]#[N+]CC1CCOC1	67.9	55
1276	**4**{*125*,*643*,*7*}	CC1=C(Br)C=C(O1)C1=C(NC2CCOCC2)N2C=C(CCO)C=CC2=N1 |c:1,4,8,24,27,t:19|	Z8873685294	**1**{*125*}	Nc1ccc(CCO)cn1	**2**{*643*}	Cc1oc(C=O)cc1Br	**3**{*7*}	[C-]#[N+]C1CCOCC1	62.1	55
1277	**4**{*50*,*644*,*7*}	CP(C)(=O)C1=CC=CC2=NC(=C(NC3CCOCC3)N12)C1=CC(F)=C(OCC#C)C=C1 |c:6,32,t:4,8,10,23,26|	Z8878918412	**1**{*50*}	CP(=O)(C)c1cccc(N)n1	**2**{*644*}	Fc1cc(C=O)ccc1OCC#C	**3**{*7*}	[C-]#[N+]C1CCOCC1	65.2	55
1278	**4**{*105*,*382*,*50*}	COC(C)(C)CC1=C(NC2=CC3=C(OCCO3)C=C2)N2C(C=CC=C2C(N)=O)=N1 |c:6,18,23,25,30,t:9,11|	Z8878918605	**1**{*105*}	NC(=O)c1cccc(N)n1	**2**{*382*}	COC(C)(C)CC=O	**3**{*50*}	[C-]#[N+]c1ccc2OCCOc2c1	58.5	55
1279	**4**{*267*,*353*,*23*}	COC(=O)CCC(NC1=C(N=C2C=CC=C(C#C)N12)C1=C(CF)C=CC=C1)C(=O)OC |c:12,21,25,27,t:8,10,14|	Z8878918888	**1**{*267*}	Nc1cccc(C#C)n1	**2**{*353*}	FCc1ccccc1C=O	**3**{*23*}	COC(=O)CCC([N+]#[C-])C(=O)OC	62.4	55

1280	**4**{*66*,*299*,*22*}	COCCC1=CC2=NC(=C(NC3COC3)N2C=C1)C1=C(OC)C(OC)=CC=C1F |c:18,21,27,29,t:4,6,8|	Z8873684894	**1**{*66*}	COCCc1ccnc(N)c1	**2**{*299*}	COc1ccc(F)c(C=O)c1OC	**3**{*22*}	[C-]#[N+]C1COC1	59.0	54
1281	**4**{*405*,*535*,*23*}	COC(=O)CCC(NC1=C(N=C2C=CC(=CN12)N1CCCC1=O)C1=NOC(=C1)C(C)(C)O)C(=O)OC |c:12,14,29,t:8,10,26|	Z8835022904	**1**{*405*}	Nc1ccc(cn1)N2CCCC2=O	**2**{*535*}	CC(C)(O)c1cc(C=O)no1	**3**{*23*}	COC(=O)CCC([N+]#[C-])C(=O)OC	73.4	54
1282	**4**{*125*,*645*,*36*}	COC(=O)[C@@H]1CC(CN1C(=O)OC(C)(C)C)NC1=C(N=C2C=CC(CCO)=CN12)C1=CNN=N1 |c:22,27,35,t:18,20,32|	Z8873685427	**1**{*125*}	Nc1ccc(CCO)cn1	**2**{*645*}	O=Cc1c[nH]nn1	**3**{*36*}	COC(=O)[C@@H]1C[C@@H](CN1C(=O)OC(C)(C)C)[N+]#[C-]	69.3	54
1283	**4**{*62*,*49*,*13*}	COC1=CC=C(CNC2=C(N=C3C=C(CO)C=CN23)C2=CNN=C2C)C=C1 |c:16,24,28,t:2,4,8,10,12,21|	Z8873685386	**1**{*62*}	Nc1cc(CO)ccn1	**2**{*49*}	Cc1n[nH]cc1C=O	**3**{*13*}	COc1ccc(C[N+]#[C-])cc1	53.4	54
1284	**4**{*377*,*622*,*12*}	CCNC(=O)COC1=CC=C(C=C1)C1=C(NCC2CCOC2)N2C=CC=C(OCC(F)F)C2=N1 |c:9,11,14,25,35,t:7,27|	Z8873684829	**1**{*377*}	Nc1ncccc1OCC(F)F	**2**{*622*}	CCNC(=O)COc1ccc(C=O)cc1	**3**{*12*}	[C-]#[N+]CC1CCOC1	69.7	54
1285	**4**{*69*,*646*,*12*}	CNC(=O)C1=CC2=NC(=C(NCC3CCOC3)N2C=C1)C1=CNC(=N1)C1=CC=CC=C1 |c:20,26,31,33,t:4,6,8,23,29|	Z8854581158	**1**{*69*}	CNC(=O)c1ccnc(N)c1	**2**{*646*}	O=Cc1c[nH]c(n1)c2ccccc2	**3**{*12*}	[C-]#[N+]CC1CCOC1	61.1	54
1286	**4**{*353*,*123*,*53*}	CC1=NC=C(N=C1)C1=C(NCCOCC2=CC=CC=C2)N2C=C(NS(C)(=O)=O)C=CC2=N1 |c:3,5,8,17,19,30,33,t:1,15,23|	Z8878918609	**1**{*353*}	Cl.CS(=O)(=O)Nc1ccc(N)nc1	**2**{*123*}	Cc1cnc(C=O)cn1	**3**{*53*}	[C-]#[N+]CCOCc1ccccc1	66.3	54
1287	**4**{*193*,*647*,*10*}	CNC(=O)C1=CN2C(C=C1)=NC(=C2NC1CCOC1)C1=CC2=C(OC=C2)C=C1 |c:8,10,12,27,30,t:4,22,24|	Z8878918315	**1**{*193*}	CNC(=O)c1ccc(N)nc1	**2**{*647*}	O=Cc1ccc2occc2c1	**3**{*10*}	[C-]#[N+]C1CCOC1	55.2	54
1288	**4**{*400*,*648*,*22*}	CCC(CC1=C(NC2COC2)N2C=C(COC(C)C)C=CC2=N1)NC(=O)OC(C)(C)C |c:4,20,23,t:13|	Z8878918564	**1**{*400*}	CC(C)OCc1ccc(N)nc1	**2**{*648*}	CCC(CC=O)NC(=O)OC(C)(C)C	**3**{*22*}	[C-]#[N+]C1COC1	60.4	54
1289	**4**{*440*,*649*,*10*}	CN1N=C(C=C1C1=C(NC2CCOC2)N2C(C=CC=C2Cl)=N1)C(F)F |c:2,4,7,18,20,23|	Z8855739124	**1**{*440*}	Nc1cccc(Cl)n1	**2**{*649*}	Cn1nc(cc1C=O)C(F)F	**3**{*10*}	[C-]#[N+]C1CCOC1	53.8	54
1290	**4**{*129*,*110*,*29*}	CCOC(=O)CCNC1=C(N=C2C=CC=C(SC)N12)C1=CN=C(C=C1)P(C)(C)=O |c:12,23,25,t:8,10,14,21|	Z8873685433	**1**{*129*}	CSc1cccc(N)n1	**2**{*110*}	CP(=O)(C)c1ccc(C=O)cn1	**3**{*29*}	CCOC(=O)CC[N+]#[C-]	63.3	54

1291	**4**{*49*,*94*,*20*}	COC1=CC(C)=C(NC2=C(N=C3C=CC=C(CO)N23)C2=NC=CN=C2)C=C1 |c:12,23,25,28,t:2,5,8,10,14,21|	Z8878918384	**1**{*49*}	Nc1cccc(CO)n1	**2**{*94*}	O=Cc1cnccn1	**3**{*20*}	COc1ccc([N+]#[C-])c(C)c1	52.9	54
1292	**4**{*378*,*650*,*12*}	C(NC1=C(N=C2N1C=CC=C2OCC1=CN=CC=C1)C1=CC2=C(CCOC2)C=C1)C1CCOC1 |c:4,8,10,17,19,31,t:2,15,22,24|	Z8878918324	**1**{*378*}	Nc1ncccc1OCc2cccnc2	**2**{*650*}	O=Cc1ccc2CCOCc2c1	**3**{*12*}	[C-]#[N+]CC1CCOC1	66.8	54
1293	**4**{*41*,*651*,*14*}	COCCNC1=C(N=C2C=NC(C)=CN12)C1=C(C)OC(C)=C1 |c:9,12,17,22,t:5,7|	Z8846492117	**1**{*41*}	Cc1cnc(N)cn1	**2**{*651*}	Cc1cc(C=O)c(C)o1	**3**{*14*}	COCC[N+]#[C-]	43.9	54
1294	**4**{*79*,*116*,*13*}	COC1=CC=C(CNC2=C(N=C3C=NC=C(C)N23)C2=CN(CCC=C)N=N2)C=C1 |c:12,27,30,t:2,4,8,10,14,20|	Z8878918467	**1**{*79*}	Cc1cncc(N)n1	**2**{*116*}	C=CCCn1cc(C=O)nn1	**3**{*13*}	COc1ccc(C[N+]#[C-])cc1	56.8	54
1295	**4**{*441*,*534*,*7*}	CC(C)N1C=CC(=N1)C1=C(NC2CCOCC2)N2C=C(F)C(=CC2=N1)C(C)(F)F |c:4,6,9,23,26,t:20|	Z8878918385	**1**{*441*}	CC(F)(F)c1cc(N)ncc1F	**2**{*534*}	CC(C)n1ccc(C=O)n1	**3**{*7*}	[C-]#[N+]C1CCOCC1	59.4	54
1296	**4**{*153*,*575*,*15*}	CCNC1=C(CC(NC(=O)OC(C)(C)C)C(F)(F)F)N=C2C=CC=C(N12)C(C)(C)O |c:3,21,23,t:19|	Z8873684649	**1**{*153*}	CC(C)(O)c1cccc(N)n1	**2**{*575*}	CC(C)(C)OC(=O)NC(CC=O)C(F)(F)F	**3**{*15*}	CC[N+]#[C-]	62.7	54
1297	**4**{*50*,*11*,*7*}	CP(C)(=O)C1=CC=CC2=NC(=C(NC3CCOCC3)N12)C1=NN(CC(F)(F)F)C=C1 |c:6,31,t:4,8,10,23|	Z8878918428	**1**{*50*}	CP(=O)(C)c1cccc(N)n1	**2**{*11*}	FC(F)(F)Cn1ccc(C=O)n1	**3**{*7*}	[C-]#[N+]C1CCOCC1	64.3	54
1298	**4**{*28*,*11*,*7*}	FC(F)(F)CN1C=CC(=N1)C1=C(NC2CCOCC2)N2C=CC(=CC2=N1)C1=NOC=N1 |c:6,8,11,22,24,27,33,t:30|	Z8849597824	**1**{*28*}	Cl.Nc1cc(ccn1)c2ncon2	**2**{*11*}	FC(F)(F)Cn1ccc(C=O)n1	**3**{*7*}	[C-]#[N+]C1CCOCC1	63.0	54
1299	**4**{*35*,*420*,*13*}	COC1=CC=C(CNC2=C(CC(C)(C)C#N)N=C3C=CC(C)=CN23)C=C1 |c:8,18,21,26,t:2,4,16|	Z8837933159	**1**{*35*}	Cc1ccc(N)nc1	**2**{*420*}	CC(C)(CC=O)C#N	**3**{*13*}	COc1ccc(C[N+]#[C-])cc1	50.6	54
1300	**4**{*50*,*652*,*31*}	CSCCNC1=C(N=C2C=CC=C(N12)P(C)(C)=O)C1=CSC2=C1C(F)=CC=C2 |c:9,11,23,27,29,t:5,7,20|	Z8878918839	**1**{*50*}	CP(=O)(C)c1cccc(N)n1	**2**{*652*}	Fc1cccc2scc(C=O)c12	**3**{*31*}	CSCC[N+]#[C-]	62.8	54
1301	**4**{*362*,*497*,*10*}	CCN1N=CC(C)=C1C1=C(NC2CCOC2)N2C(C=CC=C2C(=O)NC)=N1 |c:3,6,9,20,22,28|	Z8855619571	**1**{*362*}	CNC(=O)c1cccc(N)n1	**2**{*497*}	CCn1ncc(C)c1C=O	**3**{*10*}	[C-]#[N+]C1CCOC1	53.1	53
1302	**4**{*104*,*144*,*46*}	CC(=C)CNC1=C(N=C2C=CC3=C(COCC3)N12)C1=NOC(=C1)C1CC1 |c:9,24,t:5,7,11,21|	Z8829498719	**1**{*104*}	Nc1ccc2CCOCc2n1	**2**{*144*}	O=Cc1cc(on1)C2CC2	**3**{*46*}	CC(=C)C[N+]#[C-]	50.4	53

<table>
<tr><td>1303</td><td>4{46,181,13}</td><td>COC1=CC=C(CNC2=C(N=C3C=CC=CN23)C2=CN=C3CCCN23)C=C1 |c:12,14,29,t:2,4,8,10,19,21|</td><td>Z8829498470</td><td>1{46}</td><td>Nc1ccccn1</td><td>2{181}</td><td>O=Cc1cnc2CCCn12</td><td>3{13}</td><td>COc1ccc(C[N+]#[C-])cc1</td><td>51.7</td><td>53</td></tr>
<tr><td>1304</td><td>4{77,653,23}</td><td>COC(=O)CCC(NC1=C(N=C2C=CC(=CN12)C1=CN=CC=C1)C1C2CCC(F)(F)C12)C(=O)OC |c:12,14,21,23,t:8,10,19|</td><td>Z8835022901</td><td>1{77}</td><td>Nc1ccc(cn1)c2cccnc2</td><td>2{653}</td><td>FC1(F)CCC2C(C=O)C21</td><td>3{23}</td><td>COC(=O)CCC([N+]#[C-])C(=O)OC</td><td>69.6</td><td>53</td></tr>
<tr><td>1305</td><td>4{367,600,34}</td><td>CC1=CN2C(NCC3CCCO3)=C(N=C2C(=C1)C#C)C1=CN(CCS(C)(=O)=O)N=C1 |c:12,14,17,31,t:1,22|</td><td>Z8873685409</td><td>1{367}</td><td>Cc1cnc(N)c(C#C)c1</td><td>2{600}</td><td>CS(=O)(=O)CCn1cc(C=O)cn1</td><td>3{34}</td><td>[C-]#[N+]CC1CCCO1</td><td>61.4</td><td>53</td></tr>
<tr><td>1306</td><td>4{164,596,24}</td><td>COC1=CC(CNC2=C(N=C3C=C(CN(C)C)C=CN23)C2=CC3=C(N=C2)N(C)N=C3C)=CC=C1 |c:17,24,26,31,34,36,t:2,7,9,11,22|</td><td>Z8873684817</td><td>1{164}</td><td>CN(C)Cc1ccnc(N)c1</td><td>2{596}</td><td>Cc1nn(C)c2ncc(C=O)cc12</td><td>3{24}</td><td>COc1cccc(C[N+]#[C-])c1</td><td>65.4</td><td>53</td></tr>
<tr><td>1307</td><td>4{50,374,26}</td><td>CP(C)(=O)C1=CC=CC2=NC(C3=CN(CC4CC4)N=N3)=C(NCC3=CC=C(F)C=C3)N12 |c:6,19,30,t:4,8,11,21,25,27|</td><td>Z8846492347</td><td>1{50}</td><td>CP(=O)(C)c1cccc(N)n1</td><td>2{374}</td><td>O=Cc1cn(CC2CC2)nn1</td><td>3{26}</td><td>Fc1ccc(C[N+]#[C-])cc1</td><td>62.6</td><td>53</td></tr>
<tr><td>1308</td><td>4{372,6,12}</td><td>CC1=C(C2=C(NCC3CCOC3)N3C=CC=C(OCC4=CC=NC=C4)C3=N2)C(C)=NC=C1 |c:1,3,14,22,24,28,32,34,t:16,20|</td><td>Z8878918442</td><td>1{372}</td><td>Nc1ncccc1OCc2ccncc2</td><td>2{6}</td><td>Cc1ccnc(C)c1C=O</td><td>3{12}</td><td>[C-]#[N+]CC1CCOC1</td><td>61.3</td><td>53</td></tr>
<tr><td>1309</td><td>4{107,334,24}</td><td>COC1=CC(CNC2=C(N=C3C=CC(OCCO)=CN23)C2=C(C)OC=N2)=CC=C1 |c:11,17,22,26,28,30,t:2,7,9|</td><td>Z8846491955</td><td>1{107}</td><td>Nc1ccc(OCCO)cn1</td><td>2{334}</td><td>Cc1ocnc1C=O</td><td>3{24}</td><td>COc1cccc(C[N+]#[C-])c1</td><td>56.3</td><td>53</td></tr>
<tr><td>1310</td><td>4{142,596,21}</td><td>CN1N=C(C)C2=C1N=CC(=C2)C1=C(NCC2CC2)N2C=CC=C(OCCS(C)(=O)=O)C2=N1 |c:5,8,10,13,22,34,t:2,24|</td><td>Z8878918663</td><td>1{142}</td><td>CS(=O)(=O)CCOc1cccnc1N</td><td>2{596}</td><td>Cc1nn(C)c2ncc(C=O)cc12</td><td>3{21}</td><td>[C-]#[N+]CC1CC1</td><td>64.9</td><td>53</td></tr>
<tr><td>1311</td><td>4{105,654,50}</td><td>NC(=O)C1=CC=CC2=NC(CCC3=CN=CO3)=C(NC3=CC4=C(OCCO4)C=C3)N12 |c:5,14,29,t:3,7,12,17,20,22|</td><td>Z8873684669</td><td>1{105}</td><td>NC(=O)c1cccc(N)n1</td><td>2{654}</td><td>O=CCCc1cnco1</td><td>3{50}</td><td>[C-]#[N+]c1ccc2OCCOc2c1</td><td>57.8</td><td>53</td></tr>
<tr><td>1312</td><td>4{193,655,12}</td><td>CNC(=O)C1=CN2C(NCC3CCOC3)=C(N=C2C=C1)C1=C(Cl)C=CC=C1F |c:15,17,20,23,26,28,t:4|</td><td>Z8855739170</td><td>1{193}</td><td>CNC(=O)c1ccc(N)nc1</td><td>2{655}</td><td>Fc1cccc(Cl)c1C=O</td><td>3{12}</td><td>[C-]#[N+]CC1CCOC1</td><td>57.4</td><td>53</td></tr>
<tr><td>1313</td><td>4{103,656,59}</td><td>CC1=NC(=C(C)S1)C1=C(NCCC2=CC(F)=CC=C2)N2C(C=NC=C2CO)=N1 |c:8,16,18,23,25,29,t:1,3,13|</td><td>Z8878918303</td><td>1{103}</td><td>Nc1cncc(CO)n1</td><td>2{656}</td><td>Cc1nc(C=O)c(C)s1</td><td>3{59}</td><td>Fc1cccc(CC[N+]#[C-])c1</td><td>56.6</td><td>53</td></tr>
<tr><td>1314</td><td>4{73,69,11}</td><td>CN1N=C(C=C1C)C1=C(NCC2=CC(Br)=CC=C2)N2N3C=CN=C3C=CC2=N1 |c:2,4,8,15,17,22,24,27,30,t:12|</td><td>Z8878918812</td><td>1{73}</td><td>Nc1ccc2nccn2n1</td><td>2{69}</td><td>Cc1cc(C=O)nn1C</td><td>3{11}</td><td>Brc1cccc(C[N+]#[C-])c1</td><td>62.1</td><td>53</td></tr>
</table>

1315	**4**{*442*,*294*,*5*}	COCCCNC1=C(N=C2N1C=C(Cl)C=C2C)C1=C(OC)C(=O)C=CO1 |c:8,15,19,25,t:6,12|	Z8878918660	**1**{*442*}	Cc1cc(Cl)cnc1N	**2**{*294*}	COc1c(C=O)occc1=O	**3**{*5*}	COCCC[N+]#[C-]	53.7	53
1316	**4**{*50*,*153*,*7*}	CP(C)(=O)C1=CC=CC2=NC(=C(NC3CCOCC3)N12)C1=CN(CC(F)(F)F)N=N1 |c:6,31,t:4,8,10,23|	Z8873684656	**1**{*50*}	CP(=O)(C)c1cccc(N)n1	**2**{*153*}	FC(F)(F)Cn1cc(C=O)nn1	**3**{*7*}	[C-]#[N+]C1CCOCC1	62.8	53
1317	**4**{*50*,*639*,*26*}	CC1=NN=C(O1)C1=CC(=CC=C1)C1=C(NCC2=CC=C(F)C=C2)N2C(C=CC=C2P(C)(C)=O)=N1 |c:3,9,11,14,23,28,30,36,t:1,7,18,20|	Z8873685338	**1**{*50*}	CP(=O)(C)c1cccc(N)n1	**2**{*639*}	Cc1nnc(o1)c2cccc(C=O)c2	**3**{*26*}	Fc1ccc(C[N+]#[C-])cc1	67.5	53
1318	**4**{*353*,*273*,*31*}	CSCCNC1=C(N=C2C=CC(NS(C)(=O)=O)=CN12)C1=C(Br)N(C)N=C1C |c:9,16,21,26,t:5,7|	Z8835022896	**1**{*353*}	Cl.CS(=O)(=O)Nc1ccc(N)nc1	**2**{*273*}	Cc1nn(C)c(Br)c1C=O	**3**{*31*}	CSCC[N+]#[C-]	67.2	53
1319	**4**{*92*,*441*,*7*}	CCC(C)C(OC)C1=C(NC2CCOCC2)N2C=CC(=CC2=N1)C(N)=O |c:7,18,20,23|	Z8873684751	**1**{*92*}	NC(=O)c1ccnc(N)c1	**2**{*441*}	CCC(C)C(OC)C=O	**3**{*7*}	[C-]#[N+]C1CCOCC1	51.1	52
1320	**4**{*73*,*607*,*11*}	CC(C)(C)N1C=C(C=N1)C1=C(NCC2=CC(Br)=CC=C2)N2N3C=CN=C3C=CC2=N1 |c:5,7,10,17,19,24,26,29,32,t:14|	Z8829498713	**1**{*73*}	Nc1ccc2nccn2n1	**2**{*607*}	CC(C)(C)n1cc(C=O)cn1	**3**{*11*}	Brc1cccc(C[N+]#[C-])c1	65.7	52
1321	**4**{*357*,*657*,*49*}	CC(C)N1C=C(C(C)=N1)C1=C(NC2CC(F)(F)C2)N2C=C(C=CC2=N1)N1CCC(CO)CC1 |c:4,7,10,21,23,26|	Z8873685401	**1**{*357*}	Nc1ccc(cn1)N2CCC(CO)CC2	**2**{*657*}	CC(C)n1cc(C=O)c(C)n1	**3**{*49*}	FC1(F)CC(C1)[N+]#[C-]	64.8	52
1322	**4**{*362*,*658*,*21*}	CNC(=O)C1=CC=CC2=NC(C3=CN=C(CNC(=O)OC(C)(C)C)S3)=C(NCC3CC3)N12 |c:6,t:4,8,11,13,25|	Z8878918429	**1**{*362*}	CNC(=O)c1cccc(N)n1	**2**{*658*}	CC(C)(C)OC(=O)NCc1ncc(C=O)s1	**3**{*21*}	[C-]#[N+]CC1CC1	64.5	52
1323	**4**{*50*,*659*,*6*}	CCOC(=O)CCCNC1=C(N=C2C=CC=C(N12)P(C)(C)=O)C1=CC=CC2=NN(C)C=C12 |c:13,15,26,t:9,11,24,28,32|	Z8835022890	**1**{*50*}	CP(=O)(C)c1cccc(N)n1	**2**{*659*}	Cn1cc2c(C=O)cccc2n1	**3**{*6*}	CCOC(=O)CCC[N+]#[C-]	64.0	52
1324	**4**{*69*,*660*,*10*}	CNC(=O)C1=CC2=NC(=C(NC3CCOC3)N2C=C1)C1=NSC=C1 |c:19,25,t:4,6,8,22|	Z8873684835	**1**{*69*}	CNC(=O)c1ccnc(N)c1	**2**{*660*}	O=Cc1ccsn1	**3**{*10*}	[C-]#[N+]C1CCOC1	48.4	52
1325	**4**{*32*,*308*,*31*}	CSCCNC1=C(CCC2CCCC2)N=C2C=C(C=CN12)P(C)(C)=O |c:5,17,19,t:15|	Z8873685265	**1**{*32*}	CP(=O)(C)c1ccnc(N)c1	**2**{*308*}	O=CCCC1CCCC1	**3**{*31*}	CSCC[N+]#[C-]	53.5	52
1326	**4**{*443*,*135*,*49*}	CN1N=C(C)C(=C1C)C1=C(NC2CC(F)(F)C2)N2C=C(Br)C(Cl)=CC2=N1 |c:5,9,24,27,t:2,20|	Z8878918557	**1**{*443*}	Nc1cc(Cl)c(Br)cn1	**2**{*135*}	Cc1nn(C)c(C)c1C=O	**3**{*49*}	FC1(F)CC(C1)[N+]#[C-]	62.6	52

1327	**4**{*444*,*326*,*10*}	CN1N=CC=C1C1=C(NC2CCOC2)N2C=C3CCCCC3=CC2=N1 |c:2,4,7,24,27,t:17|	Z8878918320	**1**{*444*}	Nc1cc2CCCCc2cn1	**2**{*326*}	Cn1nccc1C=O	**3**{*10*}	[C-]#[N+]C1CCOC1	47.5	52
1328	**4**{*121*,*314*,*10*}	BrC1=CC2=NC(C3=CNN=C3)=C(NC3CCOC3)N2C=C1 |c:9,22,t:1,3,6,11|	Z8855619525	**1**{*121*}	Nc1cc(Br)ccn1	**2**{*314*}	O=Cc1cn[nH]c1	**3**{*10*}	[C-]#[N+]C1CCOC1	49.0	52
1329	**4**{*153*,*61*,*35*}	CSCCCNC1=C(N=C2C=CC=C(N12)C(C)(C)O)C1=CC=C(C=C1)S(C)=O |c:10,12,23,25,t:6,8,21|	Z8873685333	**1**{*153*}	CC(C)(O)c1cccc(N)n1	**2**{*61*}	CS(=O)c1ccc(C=O)cc1	**3**{*35*}	CSCCC[N+]#[C-]	58.7	52
1330	**4**{*153*,*437*,*15*}	CCNC1=C(N=C2C=CC=C(N12)C(C)(C)O)C1=CN(CC)N=C1C |c:7,9,23,t:3,5,18|	Z8873684807	**1**{*153*}	CC(C)(O)c1cccc(N)n1	**2**{*437*}	CCn1cc(C=O)c(C)n1	**3**{*15*}	CC[N+]#[C-]	46.0	52
1331	**4**{*311*,*169*,*10*}	FC1=CC2=NC(=C(NC3CCOC3)N2C=C1)C1=NNC2=C1N=CC=C2 |c:16,22,25,27,t:1,3,5,19|	Z8873685463	**1**{*311*}	Nc1cc(F)ccn1	**2**{*169*}	O=Cc1n[nH]c2cccnc12	**3**{*10*}	[C-]#[N+]C1CCOC1	47.6	52
1332	**4**{*32*,*356*,*18*}	CC(C)CN1C=C(N=N1)C1=C(NC(C)(C)C)N2C=CC(=CC2=N1)P(C)(C)=O |c:5,7,10,18,20,23|	Z8878918506	**1**{*32*}	CP(=O)(C)c1ccnc(N)c1	**2**{*356*}	CC(C)Cn1cc(C=O)nn1	**3**{*18*}	CC(C)(C)[N+]#[C-]	54.6	52
1333	**4**{*362*,*291*,*10*}	CNC(=O)C1=CC=CC2=NC(=C(NC3CCOC3)N12)C1=C(C)C(OC)=CN=C1 |c:6,22,27,29,t:4,8,10|	Z8873684583	**1**{*362*}	CNC(=O)c1cccc(N)n1	**2**{*291*}	COc1cncc(C=O)c1C	**3**{*10*}	[C-]#[N+]C1CCOC1	53.4	52
1334	**4**{*186*,*630*,*69*}	COCCN1C=C(C=N1)C1=C(NC2=C(OC)C=CC=C2)N2C=CC(=CC2=N1)S(=O)(=O)N(C)C |c:5,7,10,13,17,19,23,25,28|	Z8878918547	**1**{*186*}	Cl.CN(C)S(=O)(=O)c1ccnc(N)c1	**2**{*630*}	COCCn1cc(C=O)cn1	**3**{*69*}	COc1ccccc1[N+]#[C-]	65.8	52
1335	**4**{*120*,*661*,*62*}	COC(=O)C1(CCCC1)NC1=C(N=C2C=C(C=CN12)C1=CN(C)N=C1)C1=CN(CC(F)(F)F)N=C1 |c:15,17,26,37,t:11,13,22,29|	Z8837933168	**1**{*120*}	Cn1cc(cn1)c2ccnc(N)c2	**2**{*661*}	FC(F)(F)Cn1cc(C=O)cn1	**3**{*62*}	COC(=O)C1(CCCC1)[N+]#[C-]	68.2	52
1336	**4**{*48*,*437*,*15*}	CCNC1=C(N=C2C=C(C=CN12)S(C)(=O)=O)C1=CN(CC)N=C1C |c:7,9,23,t:3,5,18|	Z8878918641	**1**{*48*}	CS(=O)(=O)c1ccnc(N)c1	**2**{*437*}	CCn1cc(C=O)c(C)n1	**3**{*15*}	CC[N+]#[C-]	48.6	52
1337	**4**{*387*,*446*,*39*}	CCOCCNC1=C(N=C2N1C=CC=C2OC(F)F)C1=CC(=CC=C1)C(=O)NC |c:8,12,14,23,25,t:6,21|	Z8878918827	**1**{*387*}	Nc1ncccc1OC(F)F	**2**{*446*}	CNC(=O)c1cccc(C=O)c1	**3**{*39*}	CCOCC[N+]#[C-]	59.4	52
1338	**4**{*142*,*662*,*10*}	CP(C)(=O)C1=CC(=CC=C1)C1=C(NC2CCOC2)N2C=CC=C(OCCS(C)(=O)=O)C2=N1 |c:6,8,11,21,33,t:4,23|	Z8878918611	**1**{*142*}	CS(=O)(=O)CCOc1cccnc1N	**2**{*662*}	CP(=O)(C)c1cccc(C=O)c1	**3**{*10*}	[C-]#[N+]C1CCOC1	66.8	52
1339	**4**{*141*,*60*,*35*}	CSCCCNC1=C(N=C2C=C(NC(C)=O)C=CN12)C(C)C1CCOC1 |c:16,t:6,8,10|	Z8878918520	**1**{*141*}	CC(=O)Nc1ccnc(N)c1	**2**{*60*}	CC(C=O)C1CCOC1	**3**{*35*}	CSCCC[N+]#[C-]	52.6	52

1340	**4**{*154*,*663*,*37*}	COC(=O)C1CC(C1)NC1=C(N=C2C=C(C=CN12)[N+]([O-])=O)C1=CC(OCC(N)=O)=C(OC)C=C1 |c:14,16,35,t:10,12,24,31|	Z8873684657	**1**{*154*}	Nc1cc(ccn1)[N+](=O)[O-]	**2**{*663*}	COc1ccc(C=O)cc1OCC(=O)N	**3**{*37*}	COC(=O)C1CC(C1)[N+]#[C-]	65.6	52
1341	**4**{*49*,*370*,*13*}	CNC(=O)C1=CC=C(C=C1)C1=C(NCC2=CC=C(OC)C=C2)N2C(C=CC=C2CO)=N1 |c:6,8,11,21,26,28,32,t:4,15,17|	Z8873684755	**1**{*49*}	Nc1cccc(CO)n1	**2**{*370*}	CNC(=O)c1ccc(C=O)cc1	**3**{*13*}	COc1ccc(C[N+]#[C-])cc1	58.1	52
1342	**4**{*351*,*67*,*44*}	CC(C)(C)OC(=O)NCCCCNC1=C(N=C2C=CC3=C(C(O)CC3)N12)C1CCCSC1 |c:17,t:13,15,19|	Z8878918545	**1**{*351*}	Nc1ccc2CCC(O)c2n1	**2**{*67*}	O=CC1CCCSC1	**3**{*44*}	CC(C)(C)OC(=O)NCCCC[N+]#[C-]	64.2	52
1343	**4**{*127*,*664*,*34*}	COC1=C(OC)C(=CC=C1)C1=C(NCC2CCCO2)N2C=CC=C(F)C2=N1 |c:2,6,8,11,22,28,t:24|	Z8878918847	**1**{*127*}	Nc1ncccc1F	**2**{*664*}	COc1cccc(C=O)c1OC	**3**{*34*}	[C-]#[N+]CC1CCCO1	51.7	52
1344	**4**{*412*,*291*,*10*}	COC1=C(C)C(=CN=C1)C1=C(NC2CCOC2)N2C=C(Br)C(CO)=CC2=N1 |c:2,5,7,10,25,28,t:20|	Z8878918318	**1**{*412*}	Nc1cc(CO)c(Br)cn1	**2**{*291*}	COc1cncc(C=O)c1C	**3**{*10*}	[C-]#[N+]C1CCOC1	60.2	51
1345	**4**{*35*,*307*,*9*}	CNC1=C(N=C2C=CC(C)=CN12)C1=CN=C(Cl)N1C |c:6,9,t:2,4,14,16|	Z8855619567	**1**{*35*}	Cc1ccc(N)nc1	**2**{*307*}	Cn1c(Cl)ncc1C=O	**3**{*9*}	C[N+]#[C-]	38.2	51
1346	**4**{*146*,*665*,*46*}	CN1N=NC(=C1C)C1=C(NCC(C)=C)N2C=C(C=CC2=N1)S(C)(=O)=O |c:2,4,8,16,18,21|	Z8873684789	**1**{*146*}	CS(=O)(=O)c1ccc(N)nc1	**2**{*665*}	Cc1c(C=O)nnn1C	**3**{*46*}	CC(=C)C[N+]#[C-]	49.9	51
1347	**4**{*166*,*666*,*22*}	COC1=C(OC)C=C(C2=C(NC3COC3)N3C=CC(CSCCO)=CC3=N2)C(F)=C1 |c:2,8,17,24,27,31,t:6|	Z8878918601	**1**{*166*}	Nc1cc(CSCCO)ccn1	**2**{*666*}	COc1cc(F)c(C=O)cc1OC	**3**{*22*}	[C-]#[N+]C1COC1	56.1	51
1348	**4**{*366*,*567*,*12*}	COC1=CC=CN2C(NCC3CCOC3)=C(N=C12)C1=C2CCCC2=NN1C |c:4,15,21,27,t:2,17|	Z8878918600	**1**{*366*}	COc1cccnc1N	**2**{*567*}	Cn1nc2CCCc2c1C=O	**3**{*12*}	[C-]#[N+]CC1COC1	50.8	51
1349	**4**{*62*,*49*,*11*}	CC1=NNC=C1C1=C(NCC2=CC(Br)=CC=C2)N2C=CC(CO)=CC2=N1 |c:4,7,14,16,20,24,27,t:1,11|	Z8878918555	**1**{*62*}	Nc1cc(CO)ccn1	**2**{*49*}	Cc1n[nH]cc1C=O	**3**{*11*}	Brc1cccc(C[N+]#[C-])c1	57.0	51
1350	**4**{*32*,*356*,*7*}	CC(C)CN1C=C(N=N1)C1=C(NC2CCOCC2)N2C=CC(=CC2=N1)P(C)(C)=O |c:5,7,10,21,23,26|	Z8878918404	**1**{*32*}	CP(=O)(C)c1ccnc(N)c1	**2**{*356*}	CC(C)Cn1cc(C=O)nn1	**3**{*7*}	[C-]#[N+]C1CCOCC1	57.5	51
1351	**4**{*142*,*567*,*10*}	CN1N=C2CCCC2=C1C1=C(NC2CCOC2)N2C=CC=C(OCCS(C)(=O)=O)C2=N1 |c:8,11,21,33,t:2,23|	Z8873684708	**1**{*142*}	CS(=O)(=O)CCOc1cccnc1N	**2**{*567*}	Cn1nc2CCCc2c1C=O	**3**{*10*}	[C-]#[N+]C1CCOC1	61.5	51

1352	**4**{*442* ,*42* ,*23* }	COC(=O)CCC(NC1=C(N=C2N1C=C(Cl)C=C2C)C1=CN(C)N=C1Cl)C(=O)OC |c:10,17,25,t:8,14,21|	Z8878918525	**1**{*442* }	Cc1cc(Cl)cnc1N	**2**{*42* }	Cn1cc(C=O)c(Cl)n1	**3**{*23* }	COC(=O)CCC([N+]#[C-])C(=O)OC	62.7	51
1353	**4**{*230* ,*667* ,*29* }	CCOC(=O)CCNC1=C(N=C2N1C=C(Br)C=C2C(C)O)C1=C2N=CC=CN2N=C1C |c:10,17,23,25,27,31,t:8,14|	Z8878918668	**1**{*230* }	CC(O)c1cc(Br)cnc1N	**2**{*667* }	Cc1nn2cccnc2c1C=O	**3**{*29* }	CCOC(=O)CC[N+]#[C-]	67.2	51
1354	**4**{*103* ,*668* ,*5* }	COCCCNC1=C(CC2CCCC(C)(C)O2)N=C2C=NC=C(CO)N12 |c:6,20,t:18,22|	Z8878918885	**1**{*103* }	Nc1cncc(CO)n1	**2**{*668* }	CC1(C)CCCC(CC=O)O1	**3**{*5* }	COCCC[N+]#[C-]	50.0	51
1355	**4**{*373* ,*352* ,*62* }	COCCCC1=C(NC2(CCCC2)C(=O)OC)N2C=C(CNC(=O)OC(C)(C)C)C=CC2=N1 |c:5,30,33,t:19|	Z8829498753	**1**{*373* }	CC(C)(C)OC(=O)NCc1ccc(N)nc1	**2**{*352* }	COCCCC=O	**3**{*62* }	COC(=O)C1(CCCC1)[N+]#[C-]	63.5	51
1356	**4**{*214* ,*536* ,*10* }	COC1=CC(Br)=C(C)N2C(NC3CCOC3)=C(N=C12)C1=CN=C(O1)C1CCOCC1 |c:16,24,t:2,5,18,22|	Z8873684853	**1**{*214* }	COc1cc(Br)c(C)nc1N	**2**{*536* }	O=Cc1cnc(o1)C2CCOCC2	**3**{*10* }	[C-]#[N+]C1CCOC1	65.8	51
1357	**4**{*445* ,*320* ,*29* }	CCOC(=O)CCNC1=C(N=C2C=NC(SC)=CN12)C1=NN(C)C=N1 |c:12,16,25,t:8,10,21|	Z8873684837	**1**{*445* }	CSc1cnc(N)cn1	**2**{*320* }	Cn1cnc(C=O)n1	**3**{*29* }	CCOC(=O)CC[N+]#[C-]	49.7	51
1358	**4**{*153* ,*277* ,*15* }	CCNC1=C(CC(NC(=O)OC(C)(C)C)C2CC2)N=C2C=CC=C(N12)C(C)(C)O |c:3,21,23,t:19|	Z8873684594	**1**{*153* }	CC(C)(O)c1cccc(N)n1	**2**{*277* }	CC(C)(C)OC(=O)NC(CC=O)C1CC1	**3**{*15* }	CC[N+]#[C-]	55.3	51
1359	**4**{*154* ,*669* ,*47* }	CC(C)NC1=C(N=C2C=C(C=CN12)[N+]([O-])=O)C1=C(Br)N(C)N=C1 |c:8,10,18,23,t:4,6|	Z8855619636	**1**{*154* }	Nc1cc(ccn1)[N+](=O)[O-]	**2**{*669* }	Cn1ncc(C=O)c1Br	**3**{*47* }	CC(C)[N+]#[C-]	52.0	51
1360	**4**{*359* ,*437* ,*22* }	CCN1C=C(C(C)=N1)C1=C(NC2COC2)N2C=C(OC(F)F)C=CC2=N1 |c:3,6,9,24,27,t:18|	Z8873684884	**1**{*359* }	Nc1ccc(OC(F)F)cn1	**2**{*437* }	CCn1cc(C=O)c(C)n1	**3**{*22* }	[C-]#[N+]C1COC1	49.7	51
1361	**4**{*105* ,*349* ,*66* }	CC(NC1=C(N=C2C=CC=C(N12)C(N)=O)C1C[C@H]2OC(C)(C)O[C@H]2C1)C1=CC=CC=C1 |c:7,9,31,33,t:3,5,29|	Z8873684852	**1**{*105* }	NC(=O)c1cccc(N)n1	**2**{*349* }	CC1(C)O[C@@H]2CC(C[C@@H]2O1)C=O	**3**{*66* }	CC([N+]#[C-])c1ccccc1	57.4	51
1362	**4**{*91* ,*670* ,*26* }	CN(C)C(=O)C1=NN(C=C1)C1=CC=C(C=C1)C1=C(NCC2=CC=C(F)C=C2)N2C=C(C=CC2=N1)C(N)=O |c:8,13,15,18,27,31,33,36,t:5,11,22,24|	Z8846491744	**1**{*91* }	NC(=O)c1ccc(N)nc1	**2**{*670* }	CN(C)C(=O)c1ccn(n1)c2ccc(C=O)cc2	**3**{*26* }	Fc1ccc(C[N+]#[C-])cc1	67.9	51

1363	**4**{*446*,*671*,*38*}	COCCC1=C(NC2=CC3=C(OCO3)C=C2)N2C=CC(=CC2=N1)C1=CN=CC=C1 |c:4,15,19,21,24,29,31,t:7,9,27|	Z8878918659	**1**{*446*}	Nc1cc(ccn1)c2cccnc2	**2**{*671*}	COCCC=O	**3**{*38*}	[C-]#[N+]c1ccc2OCOc2c1	53.0	51
1364	**4**{*44*,*672*,*9*}	CCC1=CN2C(C=N1)=NC(=C2NC)C1=C(C)SC=N1 |c:6,8,10,15,19,t:2|	Z8846491880	**1**{*44*}	CCc1cnc(N)cn1	**2**{*672*}	Cc1scnc1C=O	**3**{*9*}	C[N+]#[C-]	37.3	51
1365	**4**{*356*,*673*,*34*}	CN(C)C(=O)C1=C(Cl)C=CC2=NC(=C(NCC3CCCO3)N12)C1=NC=CC(Cl)=C1 |c:5,8,27,30,t:10,12,25|	Z8837933144	**1**{*356*}	CN(C)C(=O)c1nc(N)ccc1Cl	**2**{*673*}	Clc1ccnc(C=O)c1	**3**{*34*}	[C-]#[N+]CC1CCCO1	59.1	50
1366	**4**{*371*,*350*,*28*}	CCCNC1=C(N=C2N1C=CC=C2OCCCC#N)C1C2CCOCC12 |c:6,10,12,t:4|	Z8855739193	**1**{*371*}	Nc1ncccc1OCCCC#N	**2**{*350*}	O=CC1C2CCOCC12	**3**{*28*}	CCC[N+]#[C-]	48.1	50
1367	**4**{*105*,*674*,*66*}	CC(NC1=C(N=C2C=CC=C(N12)C(N)=O)C1=C(C)NN=C1Cl)C1=CC=CC=C1 |c:7,9,17,21,27,29,t:3,5,25|	Z8878918771	**1**{*105*}	NC(=O)c1cccc(N)n1	**2**{*674*}	Cc1[nH]nc(Cl)c1C=O	**3**{*66*}	CC([N+]#[C-])c1ccccc1	53.6	50
1368	**4**{*47*,*495*,*6*}	CCOC(=O)CCCNC1=C(N=C2C=C(C=CN12)S(N)(=O)=O)C1=C(Cl)C=CC=N1 |c:13,15,24,27,29,t:9,11|	Z8855739260	**1**{*47*}	Cl.Nc1cc(ccn1)S(=O)(=O)N	**2**{*495*}	Clc1cccnc1C=O	**3**{*6*}	CCOC(=O)CCC[N+]#[C-]	59.4	50
1369	**4**{*447*,*675*,*49*}	CN(C)C1=CN2C(C=C1)=NC(C1COCCN1C(=O)OC(C)(C)C)=C2NC1CC(F)(F)C1 |c:7,9,25,t:3|	Z8846492240	**1**{*447*}	CN(C)c1ccc(N)nc1	**2**{*675*}	CC(C)(C)OC(=O)N1CCOCC1C=O	**3**{*49*}	FC1(F)CC(C1)[N+]#[C-]	61.3	50
1370	**4**{*107*,*676*,*10*}	OCCOC1=CN2C(C=C1)=NC(=C2NC1CCOC1)C1=CC=CC2=NON=C12 |c:8,10,12,24,t:4,22,26,29|	Z8837933182	**1**{*107*}	Nc1ccc(OCCO)cn1	**2**{*676*}	O=Cc1cccc2nonc12	**3**{*10*}	[C-]#[N+]C1CCOC1	51.7	50
1371	**4**{*103*,*677*,*5*}	COCCCNC1=C(N=C2C=NC=C(CO)N12)C1=NN(C=C1)C1CN(C1)C(=O)OC(C)(C)C |c:10,22,t:6,8,12,19|	Z8873685498	**1**{*103*}	Nc1cncc(CO)n1	**2**{*677*}	CC(C)(C)OC(=O)N1CC(C1)n2ccc(C=O)n2	**3**{*5*}	COCCC[N+]#[C-]	62.1	50
1372	**4**{*431*,*603*,*10*}	COC(=O)C1=CC(=NS1)C1=C(NC2CCOC2)N2C=C(F)C=CC2=N1 |c:6,10,23,26,t:4,20|	Z8873685422	**1**{*431*}	Nc1ccc(F)cn1	**2**{*603*}	COC(=O)c1cc(C=O)ns1	**3**{*10*}	[C-]#[N+]C1CCOC1	49.1	50
1373	**4**{*448*,*645*,*31*}	CSCCNC1=C(N=C2C=C(F)C(C=C)=CN12)C1=CNN=N1 |c:14,22,t:5,7,9,19|	Z8878918630	**1**{*448*}	Nc1cc(F)c(C=C)cn1	**2**{*645*}	O=Cc1c[nH]nn1	**3**{*31*}	CSCC[N+]#[C-]	43.2	50
1374	**4**{*154*,*160*,*47*}	CC(C)NC1=C(N=C2C=C(C=CN12)[N+]([O-])=O)C1=CC(=NN1C)C1CC1 |c:8,10,20,t:4,6,18|	Z8873684929	**1**{*154*}	Nc1cc(ccn1)[N+](=O)[O-]	**2**{*160*}	Cn1nc(cc1C=O)C2CC2	**3**{*47*}	CC(C)[N+]#[C-]	46.1	50

1375	**4**{*83*,*393*,*18*}	CC(C)(C)NC1=C(N=C2C=CC(=CN12)P(C)(C)=O)C1=CC(Br)=C(F)C=C1 |c:9,11,26,t:5,7,20,23|	Z8878918877	**1**{*83*}	CP(=O)(C)c1ccc(N)nc1	**2**{*393*}	Fc1ccc(C=O)cc1Br	**3**{*18*}	CC(C)(C)[N+]#[C-]	59.3	50
1376	**4**{*153*,*318*,*53*}	CC(C)(O)C1=CC=CC2=NC(C3CC33CC3(F)F)=C(NCCOCC3=CC=CC=C3)N12 |c:6,28,30,t:4,8,19,26|	Z8873685482	**1**{*153*}	CC(C)(O)c1cccc(N)n1	**2**{*318*}	FC1(F)CC21CC2C=O	**3**{*53*}	[C-]#[N+]CCOCc1ccccc1	57.8	50
1377	**4**{*38*,*15*,*9*}	CNC1=C(N=C2C=C(C=CN12)C#N)C1=CC=C(OC)C=C1 |c:6,8,21,t:2,4,15,17|	Z8873684862	**1**{*38*}	Nc1cc(C#N)ccn1	**2**{*15*}	COc1ccc(C=O)cc1	**3**{*9*}	C[N+]#[C-]	37.6	50
1378	**4**{*366*,*164*,*12*}	COC1=CC=CN2C(NCC3CCOC3)=C(N=C12)C1=NN(C)C2=C1COCC2 |c:4,15,25,t:2,17,21|	Z8873684843	**1**{*366*}	COc1cccnc1N	**2**{*164*}	Cn1nc(C=O)c2COCCc21	**3**{*12*}	[C-]#[N+]CC1CCOC1	51.8	50
1379	**4**{*428*,*678*,*10*}	COC1=CC(OC)=C(Cl)C=C1C1=C(NC2CCOC2)N2C=C(Br)N=CC2=N1 |c:9,12,25,28,t:2,6,22|	Z8873685486	**1**{*428*}	Nc1cnc(Br)cn1	**2**{*678*}	COc1cc(OC)c(C=O)cc1Cl	**3**{*10*}	[C-]#[N+]C1CCOC1	61.3	50
1380	**4**{*349*,*312*,*23*}	COC(=O)CCC(NC1=C(N=C2C=CC(Br)=C(C)N12)C1CCOC1(C)C)C(=O)OC |c:12,t:8,10,15|	Z8878918386	**1**{*349*}	Cc1nc(N)ccc1Br	**2**{*312*}	CC1(C)OCCC1C=O	**3**{*23*}	COC(=O)CCC([N+]#[C-])C(=O)OC	65.1	50
1381	**4**{*32*,*679*,*7*}	CP(C)(=O)C1=CC2=NC(=C(NC3CCOCC3)N2C=C1)C1=CC(=CC=C1)C1CCC1 |c:20,25,27,t:4,6,8,23|	Z8846492376	**1**{*32*}	CP(=O)(C)c1ccnc(N)c1	**2**{*679*}	O=Cc1cccc(c1)C2CCC2	**3**{*7*}	[C-]#[N+]C1CCOCC1	57.0	50
1382	**4**{*69*,*33*,*46*}	CNC(=O)C1=CC2=NC(C3CCCN(C3)C(C)=O)=C(NCC(C)=C)N2C=C1 |c:27,t:4,6,18|	Z8873684791	**1**{*69*}	CNC(=O)c1ccnc(N)c1	**2**{*33*}	CC(=O)N1CCCC(C1)C=O	**3**{*46*}	CC(=C)C[N+]#[C-]	49.7	50
1383	**4**{*81*,*17*,*13*}	CCOC1=CN=C(N=C1)C1=C(NCC2=CC=C(OC)C=C2)N2C=CN=CC2=N1 |c:5,7,10,20,24,26,29,t:3,14,16|	Z8873684723	**1**{*81*}	Nc1cnccn1	**2**{*17*}	CCOc1cnc(C=O)nc1	**3**{*13*}	COc1ccc(C[N+]#[C-])cc1	50.6	50
1384	**4**{*175*,*33*,*46*}	CC(=C)CNC1=C(N=C2N1C=CC=C2OCCF)C1CCCN(C1)C(C)=O |c:7,11,13,t:5|	Z8878918376	**1**{*175*}	Nc1ncccc1OCCF	**2**{*33*}	CC(=O)N1CCCC(C1)C=O	**3**{*46*}	CC(=C)C[N+]#[C-]	50.4	50
1385	**4**{*103*,*668*,*59*}	CC1(C)CCCC(CC2=C(NCCC3=CC(F)=CC=C3)N3C(C=NC=C3CO)=N2)O1 |c:8,16,18,23,25,29,t:13|	Z8873685368	**1**{*103*}	Nc1cncc(CO)n1	**2**{*668*}	CC1(C)CCCC(CC=O)O1	**3**{*59*}	Fc1cccc(CC[N+]#[C-])c1	55.5	50
1386	**4**{*49*,*284*,*20*}	COC1=CC(C)=C(NC2=C(N=C3C=CC=C(CO)N23)C2=C(C)N=CC=N2)C=C1 |c:12,21,24,26,29,t:2,5,8,10,14|	Z8873684918	**1**{*49*}	Nc1cccc(CO)n1	**2**{*284*}	Cc1nccnc1C=O	**3**{*20*}	COc1ccc([N+]#[C-])c(C)c1	50.5	50
1387	**4**{*109*,*680*,*34*}	CC(C)(C)C1=NC(=CC=C1)C1=C(NCC2CCCO2)N2C(C=CC=C2S(N)(=O)=O)=N1 |c:6,8,11,23,25,31,t:4|	Z8873684828	**1**{*109*}	Cl.Nc1cccc(n1)S(=O)(=O)N	**2**{*680*}	CC(C)(C)c1cccc(C=O)n1	**3**{*34*}	[C-]#[N+]CC1CCCO1	57.7	50

1388	**4**{*91*,*681*,*7*}	NC(=O)C1=CN2C(C=C1)=NC(=C2NC1CCOCC1)C1=C(Br)C=CC(Cl)=N1 |c:7,9,11,22,25,28,t:3|	Z8855739117	**1**{*91*}	NC(=O)c1ccc(N)nc1	**2**{*681*}	Clc1ccc(Br)c(C=O)n1	**3**{*7*}	[C-]#[N+]C1CCOCC1	60.6	50
1389	**4**{*360*,*320*,*66*}	COC(=O)C1=CC2=NC(=C(NC(C)C3=CC=CC=C3)N2C=C1Br)C1=NN(C)C=N1 |c:15,17,22,30,t:4,6,8,13,26|	Z8873684804	**1**{*360*}	COC(=O)c1cc(N)ncc1Br	**2**{*320*}	Cn1cnc(C=O)n1	**3**{*66*}	CC([N+]#[C-])c1ccccc1	61.2	50
1390	**4**{*330*,*653*,*29*}	CCOC(=O)CCNC1=C(N=C2C=CC(=CN12)S(=O)(=O)N(C)C)C1C2CCC(F)(F)C12 |c:12,14,t:8,10|	Z8878918876	**1**{*330*}	CN(C)S(=O)(=O)c1ccc(N)nc1	**2**{*653*}	FC1(F)CCC2C(C=O)C21	**3**{*29*}	CCOC(=O)CC[N+]#[C-]	61.3	50
1391	**4**{*142*,*608*,*24*}	COC1=CC(CNC2=C(CC3(CCC3)C#N)N=C3N2C=CC=C3OCCS(C)(=O)=O)=CC=C1 |c:7,17,21,23,32,34,t:2|	Z8846491945	**1**{*142*}	CS(=O)(=O)CCOc1cccnc1N	**2**{*608*}	O=CCC1(CCC1)C#N	**3**{*24*}	COc1cccc(C[N+]#[C-])c1	62.9	50
1392	**4**{*292*,*634*,*31*}	CCOC1=CN=CC2=NC(C(C)C3CCOCC3)=C(NCCSC)N12 |c:5,t:3,7,18|	Z8878918654	**1**{*292*}	CCOc1cncc(N)n1	**2**{*634*}	CC(C=O)C1CCOCC1	**3**{*31*}	CSCC[N+]#[C-]	48.9	50
1393	**4**{*357*,*283*,*49*}	OCC1CCN(CC1)C1=CN2C(C=C1)=NC(=C2NC1CC(F)(F)C1)C1=C2N=CC=CC2=CC=C1 |c:13,15,17,28,30,32,35,37,t:9|	Z8878918884	**1**{*357*}	Nc1ccc(cn1)N2CCC(CO)CC2	**2**{*283*}	O=Cc1cccc2cccnc12	**3**{*49*}	FC1(F)CC(C1)[N+]#[C-]	62.1	50
1394	**4**{*128*,*229*,*29*}	CCOC(=O)CCNC1=C(N=C2C=CC(=CN12)N(C)CCOC)C1=CN=C(OCCO)C=C1 |c:12,14,33,t:8,10,25,27|	Z8873684803	**1**{*128*}	COCCN(C)c1ccc(N)nc1	**2**{*229*}	OCCOc1ccc(C=O)cn1	**3**{*29*}	CCOC(=O)CC[N+]#[C-]	61.2	50
1395	**4**{*32*,*338*,*18*}	CC1=C(OC=C1)C1=C(NC(C)(C)C)N2C=CC(=CC2=N1)P(C)(C)=O |c:4,7,15,17,20,t:1|	Z8878918367	**1**{*32*}	CP(=O)(C)c1ccnc(N)c1	**2**{*338*}	Cc1ccoc1C=O	**3**{*18*}	CC(C)(C)[N+]#[C-]	46.2	50
1396	**4**{*414*,*326*,*21*}	CN1N=CC=C1C1=C(NCC2CC2)N2C=C(Cl)N=CC2=N1 |c:2,4,7,19,22,t:16|	Z8873684909	**1**{*414*}	Nc1cnc(Cl)cn1	**2**{*326*}	Cn1nccc1C=O	**3**{*21*}	[C-]#[N+]CC1CC1	40.5	50
1397	**4**{*412*,*682*,*10*}	OCC1=CC2=NC(=C(NC3CCOC3)N2C=C1Br)C1=C(Cl)C(F)=NC=C1 |c:17,21,25,27,t:2,4,6|	Z8878918351	**1**{*412*}	Nc1cc(CO)c(Br)cn1	**2**{*682*}	Fc1nccc(C=O)c1Cl	**3**{*10*}	[C-]#[N+]C1CCOC1	59.1	50
1398	**4**{*435*,*312*,*23*}	COC(=O)CCC(NC1=C(N=C2N1C=C(Br)C=C2C)C1CCOC1(C)C)C(=O)OC |c:10,17,t:8,14|	Z8878918352	**1**{*435*}	Cc1cc(Br)cnc1N	**2**{*312*}	CC1(C)OCCC1C=O	**3**{*23*}	COC(=O)CCC([N+]#[C-])C(=O)OC	64.3	49
1399	**4**{*50*,*297*,*26*}	CCN1N=C(C)C(=N1)C1=C(NCC2=CC=C(F)C=C2)N2C(C=CC=C2P(C)(C)=O)=N1 |c:6,9,18,23,25,31,t:3,13,15|	Z8849597820	**1**{*50*}	CP(=O)(C)c1cccc(N)n1	**2**{*297*}	CCn1nc(C)c(C=O)n1	**3**{*26*}	Fc1ccc(C[N+]#[C-])cc1	56.8	49
1400	**4**{*40*,*545*,*22*}	COCCN1N=C(C)C(=C1C)C1=C(NC2COC2)N2C(C=C(C=C2C)C(F)(F)F)=N1 |c:8,12,22,24,31,t:5|	Z8873684600	**1**{*40*}	Cc1cc(cc(N)n1)C(F)(F)F	**2**{*545*}	COCCn1nc(C)c(C=O)c1C	**3**{*22*}	[C-]#[N+]C1COC1	56.4	49

1401	**4**{*449,683,14*}	COCCNC1=C(N=C2N1C=CC=C2I)C1=C(C)N(C)N=C1Br |c:7,11,13,17,22,t:5|	Z8873684661	**1**{*449*}	Nc1ncccc1I	**2**{*683*}	Cc1c(C=O)c(Br)nn1C	**3**{*14*}	COCC[N+]#[C-]	65.2	49
1402	**4**{*450,33,21*}	CCC1=CC(Br)=CN2C(NCC3CC3)=C(N=C12)C1CCCN(C1)C(C)=O |c:5,14,t:2,16|	Z8878918411	**1**{*450*}	CCc1cc(Br)cnc1N	**2**{*33*}	CC(=O)N1CCCC(C1)C=O	**3**{*21*}	[C-]#[N+]CC1CC1	55.8	49
1403	**4**{*32,684,18*}	CC(C)(C)NC1=C(N=C2C=C(C=CN12)P(C)(C)=O)C1=CN(N=N1)C1CCCCC1 |c:9,11,23,t:5,7,20|	Z8873685458	**1**{*32*}	CP(=O)(C)c1ccnc(N)c1	**2**{*684*}	O=Cc1cn(nn1)C2CCCCC2	**3**{*18*}	CC(C)(C)[N+]#[C-]	55.1	49
1404	**4**{*49,494,13*}	COC1=CC=C(CNC2=C(N=C3C=CC=C(CO)N23)C2=C(Cl)N=CS2)C=C1 |c:12,21,24,28,t:2,4,8,10,14|	Z8878918773	**1**{*49*}	Nc1cccc(CO)n1	**2**{*494*}	Clc1ncsc1C=O	**3**{*13*}	COc1ccc(C[N+]#[C-])cc1	53.3	49
1405	**4**{*142,531,21*}	CC1=NC=C(C=N1)C1=C(NCC2CC2)N2C=CC=C(OCCS(C)(=O)=O)C2=N1 |c:3,5,8,17,29,t:1,19|	Z8835022872	**1**{*142*}	CS(=O)(=O)CCOc1cccnc1N	**2**{*531*}	Cc1ncc(C=O)cn1	**3**{*21*}	[C-]#[N+]CC1CC1	53.3	49
1406	**4**{*389,584,23*}	COC(=O)CCC(NC1=C(N=C2N1C=C(C)C=C2C)C1=C(OC)C=C(C)N=C1)C(=O)OC |c:10,17,21,28,t:8,14,25|	Z8878918437	**1**{*389*}	Cc1cnc(N)c(C)c1	**2**{*584*}	COc1cc(C)ncc1C=O	**3**{*23*}	COC(=O)CCC([N+]#[C-])C(=O)OC	58.5	49
1407	**4**{*451,352,62*}	COCCCC1=C(NC2(CCCC2)C(=O)OC)N2C(C=CC3=C2C=CC=N3)=N1 |c:5,20,22,25,27,29|	Z8835022895	**1**{*451*}	Nc1ccc2ncccc2n1	**2**{*352*}	COCCCC=O	**3**{*62*}	COC(=O)C1(CCCC1)[N+]#[C-]	50.6	49
1408	**4**{*119,304,46*}	CC(=C)CNC1=C(N=C2C=CC(=CN12)C#N)C1=C(C)N(CC(F)F)N=C1 |c:9,11,18,26,t:5,7|	Z8829498647	**1**{*119*}	Nc1ccc(C#N)cn1	**2**{*304*}	Cc1c(C=O)cnn1CC(F)F	**3**{*46*}	CC(=C)C[N+]#[C-]	47.1	49
1409	**4**{*36,420,9*}	CNC1=C(CC(C)(C)C#N)N=C2C=CC(Br)=CN12 |c:2,12,15,t:10|	Z8873684797	**1**{*36*}	Nc1ccc(Br)cn1	**2**{*420*}	CC(C)(CC=O)C#N	**3**{*9*}	C[N+]#[C-]	40.6	49
1410	**4**{*136,78,14*}	COCCNC1=C(N=C2N1C=C(C)C=C2Br)C1COC(C)C1 |c:7,14,t:5,11|	Z8878918455	**1**{*136*}	Cc1cnc(N)c(Br)c1	**2**{*78*}	CC1CC(CO1)C=O	**3**{*14*}	COCC[N+]#[C-]	48.6	49
1411	**4**{*161,31,20*}	COC1=CC(C)=C(NC2=C(N=C3C=CC=NN23)C2=CN=CN2)C=C1 |c:12,14,21,25,t:2,5,8,10,19|	Z8878918346	**1**{*161*}	Nc1cccnn1	**2**{*31*}	O=Cc1cnc[nH]1	**3**{*20*}	COc1ccc([N+]#[C-])c(C)c1	42.2	49
1412	**4**{*452,350,74*}	CC(C)(C)OC(=O)N1CCCC(C1)NC1=C(N=C2C=CC3=C(C=NC=C3)N12)C1C2CCOCC12 |c:19,23,25,t:15,17,21|	Z8878918350	**1**{*452*}	Nc1ccc2ccncc2n1	**2**{*350*}	O=CC1C2CCOCC12	**3**{*74*}	CC(C)(C)OC(=O)N1CCC[C@H](C1)[N+]#[C-]	61.0	49
1413	**4**{*83,417,31*}	CSCCNC1=C(N=C2C=CC(=CN12)P(C)(C)=O)C1=C2N=NN(C)C2=CC=C1 |c:9,11,20,22,27,29,t:5,7|	Z8873685242	**1**{*83*}	CP(=O)(C)c1ccc(N)nc1	**2**{*417*}	Cn1nnc2c(C=O)cccc12	**3**{*31*}	CSCC[N+]#[C-]	54.5	49

1414	**4**{*209*,*62*,*14*}	COCCNC1=C(N=C2N1C=CC=C2N1CCCCC1)C1CC11CCOCC1 |c:7,11,13,t:5|	Z8878918420	**1**{*209*}	Nc1ncccc1N2CCCCC2	**2**{*62*}	O=CC1CC21CCOCC2	**3**{*14*}	COCC[N+]#[C-]	50.6	49
1415	**4**{*91*,*639*,*18*}	CC1=NN=C(O1)C1=CC(=CC=C1)C1=C(NC(C)(C)C)N2C=C(C=CC2=N1)C(N)=O |c:3,9,11,14,22,24,27,t:1,7|	Z8873685279	**1**{*91*}	NC(=O)c1ccc(N)nc1	**2**{*639*}	Cc1nnc(o1)c2cccc(C=O)c2	**3**{*18*}	CC(C)(C)[N+]#[C-]	51.1	49
1416	**4**{*155*,*685*,*7*}	ClC1=CC=CN2C(NC3CCOCC3)=C(N=C12)C1=CN(CC2CCOC2)N=N1 |c:3,14,30,t:1,16,20|	Z8849597800	**1**{*155*}	Nc1ncccc1Cl	**2**{*685*}	O=Cc1cn(CC2CCOC2)nn1	**3**{*7*}	[C-]#[N+]C1CCOCC1	52.6	48
1417	**4**{*93*,*171*,*43*}	COC1CC(C(OC)O1)C1=C(NC2CCN(CC2)C(=O)OC(C)(C)C)N2C=C(OC)N=CC2=N1 |c:10,32,35,t:28|	Z8854581175	**1**{*93*}	COc1cnc(N)cn1	**2**{*171*}	COC1CC(C=O)C(OC)O1	**3**{*43*}	CC(C)(C)OC(=O)N1CCC(CC1)[N+]#[C-]	62.4	48
1418	**4**{*36*,*274*,*13*}	COC1=CC=C(CNC2=C(CC3(CCOCC3)C#N)N=C3C=CC(Br)=CN23)C=C1 |c:8,22,25,30,t:2,4,20|	Z8873684625	**1**{*36*}	Nc1ccc(Br)cn1	**2**{*274*}	O=CCC1(CCOCC1)C#N	**3**{*13*}	COc1ccc(C[N+]#[C-])cc1	59.4	48
1419	**4**{*193*,*325*,*24*}	CNC(=O)C1=CN2C(C=C1)=NC(CCN1C(=O)C3=C(C=CC=C3)C1=O)=C2NCC1=CC(OC)=CC=C1 |c:8,10,20,22,27,36,38,t:4,18,32|	Z8878918431	**1**{*193*}	CNC(=O)c1ccc(N)nc1	**2**{*325*}	O=CCCN1C(=O)c2ccccc2C1=O	**3**{*24*}	COc1cccc(C[N+]#[C-])c1	63.0	48
1420	**4**{*93*,*425*,*60*}	COC(=O)C1=CC(NC2=C(N=C3C=NC(OC)=CN23)C2CCCN(C2)S(C)(=O)=O)=CC=C1 |c:12,16,31,33,t:4,8,10|	Z8873684614	**1**{*93*}	COc1cnc(N)cn1	**2**{*425*}	CS(=O)(=O)N1CCCC(C1)C=O	**3**{*60*}	COC(=O)c1cccc([N+]#[C-])c1	59.8	48
1421	**4**{*373*,*83*,*39*}	CCOCCNC1=C(N=C2C=CC(CNC(=O)OC(C)(C)C)=CN12)C1=CC(=CC=C1)S(N)(=O)=O |c:10,21,28,30,t:6,8,26|	Z8878918606	**1**{*373*}	CC(C)(C)OC(=O)NCc1ccc(N)nc1	**2**{*83*}	NS(=O)(=O)c1cccc(C=O)c1	**3**{*39*}	CCOCC[N+]#[C-]	69.0	48
1422	**4**{*99*,*686*,*34*}	CCNC(=O)C1=CC=C(C=C1)C1=C(NCC2CCCO2)N2C=C(C=CC2=N1)S(N)(=O)=O |c:7,9,12,23,25,28,t:5|	Z8878918473	**1**{*99*}	Nc1ccc(cn1)S(=O)(=O)N	**2**{*686*}	CCNC(=O)c1ccc(C=O)cc1	**3**{*34*}	[C-]#[N+]CC1CCCO1	57.7	48
1423	**4**{*363*,*687*,*62*}	COC(=O)C1=CC2=NC(=C(NC3(CCCC3)C(=O)OC)N2C=C1)C1=C2CSCCC2=NN1C |c:23,26,33,t:4,6,8|	Z8873684689	**1**{*363*}	COC(=O)c1ccnc(N)c1	**2**{*687*}	Cn1nc2CCSCc2c1C=O	**3**{*62*}	COC(=O)C1(CCCC1)[N+]#[C-]	61.1	48
1424	**4**{*107*,*688*,*10*}	COC1=C(C2=C(NC3CCOC3)N3C=C(OCCO)C=CC3=N2)C(C)=C(Br)C=N1 |c:2,4,20,23,30,t:14,27|	Z8873685266	**1**{*107*}	Nc1ccc(OCCO)cn1	**2**{*688*}	COc1ncc(Br)c(C)c1C=O	**3**{*10*}	[C-]#[N+]C1CCOC1	60.3	48

1425	**4**{*153* ,*689* ,*22* }	CC(=O)OCCN1C=C(C=N1)C1=C(NC2COC2)N2C(C=CC=C2C(C)(C)O)=N1 |c:7,9,12,22,24,30|	Z8873684893	**1**{*153* }	CC(C)(O)c1cccc(N)n1	**2**{*689* }	CC(=O)OCCn1cc(C=O)cn1	**3**{*22* }	[C-]#[N+]C1COC1	52.0	48
1426	**4**{*366* ,*296* ,*39* }	CCOCCNC1=C(N=C2N1C=CC=C2OC)C1=CN(CCF)N=C1 |c:8,12,14,25,t:6,19|	Z8849597807	**1**{*366* }	COc1cccnc1N	**2**{*296* }	FCCn1cc(C=O)cn1	**3**{*39* }	CCOCC[N+]#[C-]	45.2	48
1427	**4**{*174* ,*588* ,*61* }	CN1C=C(C(C)=N1)C1=C(NCCCNC(=O)OC(C)(C)C)N2C(C=CC=C2C#N)=N1 |c:2,5,8,24,26,30|	Z8878918523	**1**{*174* }	Nc1cccc(C#N)n1	**2**{*588* }	Cc1nn(C)cc1C=O	**3**{*61* }	CC(C)(C)OC(=O)NCCC[N+]#[C-]	53.3	48
1428	**4**{*193* ,*690* ,*10* }	CNC(=O)C1=CN2C(C=C1)=NC(CCC1(CCN(CC1)C(=O)OC(C)(C)C)C#N)=C2NC1CCOC1 |c:8,10,30,t:4|	Z8873684584	**1**{*193* }	CNC(=O)c1ccc(N)nc1	**2**{*690* }	CC(C)(C)OC(=O)N1CCC(CCC=O)(CC1)C#N	**3**{*10* }	[C-]#[N+]C1CCOC1	64.6	48
1429	**4**{*41* ,*25* ,*13* }	COC1=CC=C(CNC2=C(N=C3C=NC(C)=CN23)C2=CN=C(N=C2)C2CCOCC2)C=C1 |c:12,15,22,24,34,t:2,4,8,10,20|	Z8878918532	**1**{*41* }	Cc1cnc(N)cn1	**2**{*25* }	O=Cc1cnc(nc1)C2CCOCC2	**3**{*13* }	COc1ccc(C[N+]#[C-])cc1	56.0	48
1430	**4**{*167* ,*386* ,*23* }	COC(=O)CCC(NC1=C(N=C2N1C(C)=CN=C2C)[C@H]1CC1(C)C)C(=O)OC |c:10,15,17,t:8|	Z8878918593	**1**{*167* }	Cc1cnc(C)c(N)n1	**2**{*386* }	CC1(C)C[C@@H]1C=O	**3**{*23* }	COC(=O)CCC([N+]#[C-])C(=O)OC	50.3	48
1431	**4**{*70* ,*60* ,*31* }	CSCCNC1=C(N=C2N1C=CN=C2C1CC1)C(C)C1CCOC1 |c:7,11,13,t:5|	Z8873685406	**1**{*70* }	Nc1nccnc1C2CC2	**2**{*60* }	CC(C=O)C1CCOC1	**3**{*31* }	CSCC[N+]#[C-]	44.8	48
1432	**4**{*139* ,*369* ,*46* }	CC(=C)CNC1=C(N=C2C=C(C=CN12)C(F)F)C1=CON=C1C |c:9,11,22,t:5,7,19|	Z8873684771	**1**{*139* }	Nc1cc(ccn1)C(F)F	**2**{*369* }	Cc1nocc1C=O	**3**{*46* }	CC(=C)C[N+]#[C-]	41.2	48
1433	**4**{*453* ,*312* ,*23* }	CCC1=CN2C(C=C1)=NC(C1CCOC1(C)C)=C2NC(CCC(=O)OC)C(=O)OC |c:6,8,18,t:2|	Z8878918617	**1**{*453* }	CCc1ccc(N)nc1	**2**{*312* }	CC1(C)OCCC1C=O	**3**{*23* }	COC(=O)CCC([N+]#[C-])C(=O)OC	53.8	48
1434	**4**{*330* ,*674* ,*29* }	CCOC(=O)CCNC1=C(N=C2C=CC(=CN12)S(=O)(=O)N(C)C)C1=C(C)NN=C1Cl |c:12,14,25,29,t:8,10|	Z8878918825	**1**{*330* }	CN(C)S(=O)(=O)c1ccc(N)nc1	**2**{*674* }	Cc1[nH]nc(Cl)c1C=O	**3**{*29* }	CCOC(=O)CC[N+]#[C-]	58.6	48
1435	**4**{*454* ,*103* ,*37* }	COC(=O)C1CC(C1)NC1=C(N=C2C=C(OC(C)C(F)(F)F)C=CN12)C1CCC2(COC2)OC1 |c:23,t:10,12,14|	Z8873685311	**1**{*454* }	CC(Oc1ccnc(N)c1)C(F)(F)F	**2**{*103* }	O=CC1CCC2(COC2)OC1	**3**{*37* }	COC(=O)C1CC(C1)[N+]#[C-]	62.3	48
1436	**4**{*53* ,*691* ,*60* }	COC(=O)C1=CC(NC2=C(N=C3C=CC(CO)=CN23)C2=CC(=C(C)O2)S(C)(=O)=O)=CC=C1 |c:12,16,31,33,t:4,8,10,21,23|	Z8873684605	**1**{*53* }	Nc1ccc(CO)cn1	**2**{*691* }	Cc1oc(C=O)cc1S(=O)(=O)C	**3**{*60* }	COC(=O)c1cccc([N+]#[C-])c1	58.6	48
1437	**4**{*91* ,*439* ,*7* }	COC1=C(C=CC(=C1)C1=C(NC2CCOCC2)N2C=C(C=CC2=N1)C(N)=O)S(C)(=O)=O |c:4,6,9,20,22,25,t:2|	Z8878918831	**1**{*91* }	NC(=O)c1ccc(N)nc1	**2**{*439* }	COc1cc(C=O)ccc1S(=O)(=O)C	**3**{*7* }	[C-]#[N+]C1CCOCC1	57.1	48

1438	**4**{*47*,*158*,*26*}	NS(=O)(=O)C1=CC2=NC(C3=CN(C=C)N=C3)=C(NCC3=CC=C(F)C=C3)N2C=C1 |c:14,25,30,t:4,6,9,16,20,22|	Z8849597833	**1**{*47*}	Cl.Nc1cc(ccn1)S(=O)(=O)N	**2**{*158*}	C=Cn1cc(C=O)cn1	**3**{*26*}	Fc1ccc(C[N+]#[C-])cc1	53.0	48
1439	**4**{*327*,*47*,*41*}	COC(=O)C1=C(F)C2=NC(CCOCC=C)=C(NC3=CC=C(OC)C=C3)N2C=C1 |c:4,24,29,t:7,15,18,20|	Z8849597797	**1**{*327*}	COC(=O)c1ccnc(N)c1F	**2**{*47*}	C=CCOCCC=O	**3**{*41*}	COc1ccc([N+]#[C-])cc1	51.2	47
1440	**4**{*173*,*147*,*64*}	COCCCCC1=C(N[C@@H]2CC[C@@H](C2)C(=O)OC)N2C=C(C=C(Br)C2=N1)C(=O)N(C)C |&1:9,12,r,c:6,20,26,t:22|	Z8873684645	**1**{*173*}	CN(C)C(=O)c1cnc(N)c(Br)c1	**2**{*147*}	COCCCCC=O	**3**{*64*}	COC(=O)[C@@H]1CC[C@@H](C1)[N+]#[C-] |&1:4,7,r|	63.5	47
1441	**4**{*139*,*692*,*10*}	CN1N=C(C2=C1CCN(C2)C(=O)OC(C)(C)C)C1=C(NC2CCOC2)N2C=CC(=CC2=N1)C(F)F |c:2,4,19,29,31,34|	Z8854581147	**1**{*139*}	Nc1cc(ccn1)C(F)F	**2**{*692*}	Cn1nc(C=O)c2CN(CCc21)C(=O)OC(C)(C)C	**3**{*10*}	[C-]#[N+]C1CCOC1	62.3	47
1442	**4**{*107*,*139*,*72*}	CC1=NN(CCO)C(C)=C1C1=C(NC2=C(C)C=C(F)C=C2)N2C=C(OCCO)C=CC2=N1 |c:8,11,14,20,30,33,t:1,17,24|	Z8854581169	**1**{*107*}	Nc1ccc(OCCO)cn1	**2**{*139*}	Cc1nn(CCO)c(C)c1C=O	**3**{*72*}	Cc1cc(F)ccc1[N+]#[C-]	55.8	47
1443	**4**{*50*,*693*,*14*}	CCC1=C(SC=C1)C1=C(NCCOC)N2C(C=CC=C2P(C)(C)=O)=N1 |c:5,8,17,19,25,t:2|	Z8855739084	**1**{*50*}	CP(=O)(C)c1cccc(N)n1	**2**{*693*}	CCc1ccsc1C=O	**3**{*14*}	COCC[N+]#[C-]	47.9	47
1444	**4**{*455*,*403*,*23*}	COC(=O)CCC(NC1=C(N=C2C=C(Cl)C=C(Cl)N12)C1=CN=C(Cl)N1)C(=O)OC |t:8,10,12,15,21,23|	Z8873685301	**1**{*455*}	Nc1cc(Cl)cc(Cl)n1	**2**{*403*}	Clc1ncc(C=O)[nH]1	**3**{*23*}	COC(=O)CCC([N+]#[C-])C(=O)OC	58.5	47
1445	**4**{*369*,*441*,*7*}	CCC(C)C(OC)C1=C(NC2CCOCC2)N2C=C(C=CC2=N1)C(F)C(F)(F)F |c:7,18,20,23|	Z8873684615	**1**{*369*}	Cl.Nc1ccc(cn1)C(F)C(F)(F)F	**2**{*441*}	CCC(C)C(OC)C=O	**3**{*7*}	[C-]#[N+]C1CCOCC1	53.0	47
1446	**4**{*66*,*694*,*22*}	COCCC1=CC2=NC(C3CCCN(C3)C(=O)OC(C)(C)C)=C(NC3COC3)N2C=C1 |c:32,t:4,6,22|	Z8873684777	**1**{*66*}	COCCc1ccnc(N)c1	**2**{*694*}	CC(C)(C)OC(=O)N1CCCC(C1)C=O	**3**{*22*}	[C-]#[N+]C1COC1	54.7	47
1447	**4**{*42*,*695*,*7*}	COC(=O)COC1=C(OC)C=CC(=C1)C1=C(NC2CCOCC2)N2C=C(SC)C=CC2=N1 |c:6,10,12,15,30,33,t:26|	Z8873685254	**1**{*42*}	CSc1ccc(N)nc1	**2**{*695*}	COC(=O)COc1cc(C=O)ccc1OC	**3**{*7*}	[C-]#[N+]C1CCOCC1	58.1	47
1448	**4**{*69*,*111*,*21*}	CNC(=O)C1=CC2=NC(C3CCCC3)=C(NCC3CC3)N2C=C1 |c:24,t:4,6,14|	Z8873684662	**1**{*69*}	CNC(=O)c1ccnc(N)c1	**2**{*111*}	O=CC1CCCC1	**3**{*21*}	[C-]#[N+]CC1CC1	39.6	47

1449	**4**{*209*,*246*,*14*}	COCCNC1=C(N=C2N1C=CC=C2N1CCCCC1)C1CC11CCCOC1 |c:7,11,13,t:5|	Z8873684821	**1**{*209*}	Nc1ncccc1N2CCCCC2	**2**{*246*}	O=CC1CC21CCCOC2	**3**{*14*}	COCC[N+]#[C-]	48.8	47
1450	**4**{*456*,*597*,*7*}	CC1=C(C)N=C(O1)C1=C(NC2CCOCC2)N2C=CC(OCCNC(=O)OC(C)(C)C)=CC2=N1 |c:1,4,8,19,32,35|	Z8878918453	**1**{*456*}	CC(C)(C)OC(=O)NCCOc1ccnc(N)c1	**2**{*597*}	Cc1nc(C=O)oc1C	**3**{*7*}	[C-]#[N+]C1CCOCC1	59.7	47
1451	**4**{*351*,*156*,*44*}	CN1C=NC(Cl)=C1C1=C(NCCCCNC(=O)OC(C)(C)C)N2C(C=CC3=C2C(O)CC3)=N1 |c:2,5,8,25,27,34|	Z8878918530	**1**{*351*}	Nc1ccc2CCC(O)c2n1	**2**{*156*}	Cn1cnc(Cl)c1C=O	**3**{*44*}	CC(C)(C)OC(=O)NCCCC[N+]#[C-]	60.1	47
1452	**4**{*28*,*639*,*7*}	CC1=NN=C(O1)C1=CC(=CC=C1)C1=C(NC2CCOCC2)N2C=CC(=CC2=N1)C1=NOC=N1 |c:3,9,11,14,25,27,30,36,t:1,7,33|	Z8873684579	**1**{*28*}	Cl.Nc1cc(ccn1)c2ncon2	**2**{*639*}	Cc1nnc(o1)c2cccc(C=O)c2	**3**{*7*}	[C-]#[N+]C1CCOCC1	56.0	47
1453	**4**{*28*,*153*,*18*}	CC(C)(C)NC1=C(N=C2C=C(C=CN12)C1=NOC=N1)C1=CN(CC(F)(F)F)N=N1 |c:9,11,19,30,t:5,7,16,22|	Z8878918566	**1**{*28*}	Cl.Nc1cc(ccn1)c2ncon2	**2**{*153*}	FC(F)(F)Cn1cc(C=O)nn1	**3**{*18*}	CC(C)(C)[N+]#[C-]	51.3	47
1454	**4**{*193*,*392*,*10*}	CNC(=O)C1=CN2C(C=C1)=NC(C1CCOCC1)=C2NC1CCOC1 |c:8,10,19,t:4|	Z8837933148	**1**{*193*}	CNC(=O)c1ccc(N)nc1	**2**{*392*}	O=CC1CCOCC1	**3**{*10*}	[C-]#[N+]C1CCOC1	43.5	47
1455	**4**{*367*,*480*,*34*}	CC1=CN2C(NCC3CCCO3)=C(N=C2C(=C1)C#C)C1=CC2=C(CCOC2)N=C1 |c:12,14,17,31,t:1,22,24|	Z8878918521	**1**{*367*}	Cc1cnc(N)c(C#C)c1	**2**{*480*}	O=Cc1cnc2CCOCc2c1	**3**{*34*}	[C-]#[N+]CC1CCCO1	49.0	47
1456	**4**{*388*,*509*,*67*}	CCC(C)NC1=C(N=C2C=C3CCCOC3=CN12)C1=NN=CN1C |c:16,23,t:5,7,9,21|	Z8878918886	**1**{*388*}	Cl.Nc1cc2CCCOc2cn1	**2**{*509*}	Cn1cnnc1C=O	**3**{*67*}	CCC(C)[N+]#[C-]	41.1	47
1457	**4**{*78*,*629*,*75*}	CC1=CN2N3C(C=C(C)C2=N1)=NC(=C3NCC1=C(Br)C=CC=C1)C1=C(C)C=C(C)C=N1 |c:10,12,14,19,22,24,27,33,t:1,6,30|	Z8878918790	**1**{*78*}	Cc1cn2nc(N)cc(C)c2n1	**2**{*629*}	Cc1cnc(C=O)c(C)c1	**3**{*75*}	Brc1ccccc1C[N+]#[C-]	59.8	47
1458	**4**{*457*,*99*,*15*}	CCNC1=C(N=C2C=C(C(F)F)C(Br)=CN12)C1=COC=N1 |c:13,21,t:3,5,7,18|	Z8878918390	**1**{*457*}	Nc1cc(C(F)F)c(Br)cn1	**2**{*99*}	O=Cc1cocn1	**3**{*15*}	CC[N+]#[C-]	44.8	46
1459	**4**{*374*,*696*,*62*}	COCCC1=NC=C(S1)C1=C(NC2(CCCC2)C(=O)OC)N2C(C=CC=C2C(=O)OC)=N1 |c:6,10,25,27,33,t:4|	Z8878918657	**1**{*374*}	COC(=O)c1cccc(N)n1	**2**{*696*}	COCCc1ncc(C=O)s1	**3**{*62*}	COC(=O)C1(CCCC1)[N+]#[C-]	57.4	46
1460	**4**{*81*,*30*,*6*}	CCOC(=O)CCCNC1=C(N=C2C=NC=CN12)C1CCN(CC1)C(C)=O |c:13,15,t:9,11|	Z8846492161	**1**{*81*}	Nc1cnccn1	**2**{*30*}	CC(=O)N1CCC(CC1)C=O	**3**{*6*}	CCOC(=O)CCC[N+]#[C-]	46.4	46

<table>
<tr><th></th><th></th><th></th><th></th><th></th><th></th><th></th><th></th><th></th><th></th><th></th><th></th></tr>
<tr><td>1461</td><td>4{155,246,14}</td><td>COCCNC1=C(N=C2N1C=CC=C2Cl)C1CC11CCCOC1 |c:7,11,13,t:5|</td><td>Z8878918901</td><td>1{155}</td><td>Nc1ncccc1Cl</td><td>2{246}</td><td>O=CC1CC21CCCOC2</td><td>3{14}</td><td>COCC[N+]#[C-]</td><td>41.7</td><td>46</td></tr>
<tr><td>1462</td><td>4{79,697,13}</td><td>CCC1=NC=C(N1)C1=C(NCC2=CC=C(OC)C=C2)N2C(C=NC=C2C)=N1 |c:4,8,18,23,25,28,t:2,12,14|</td><td>Z8873685303</td><td>1{79}</td><td>Cc1cncc(N)n1</td><td>2{697}</td><td>CCc1ncc(C=O)[nH]1</td><td>3{13}</td><td>COc1ccc(C[N+]#[C-])cc1</td><td>44.9</td><td>46</td></tr>
<tr><td>1463</td><td>4{436,294,23}</td><td>CCC1=CC2=NC(=C(NC(CCC(=O)OC)C(=O)OC)N2C=C1)C1=C(OC)C(=O)C=CO1 |c:22,25,31,t:2,4,6|</td><td>Z8873684864</td><td>1{436}</td><td>CCc1ccnc(N)c1</td><td>2{294}</td><td>COc1c(C=O)occc1=O</td><td>3{23}</td><td>COC(=O)CCC([N+]#[C-])C(=O)OC</td><td>54.6</td><td>46</td></tr>
<tr><td>1464</td><td>4{166,698,22}</td><td>CC(C)(C)OC(=O)N1CCC(CC1)C1=C(NC2COC2)N2C=CC(CSCCO)=CC2=N1 |c:14,23,30,33|</td><td>Z8878918872</td><td>1{166}</td><td>Nc1cc(CSCCO)ccn1</td><td>2{698}</td><td>CC(C)(C)OC(=O)N1CCC(CC1)C=O</td><td>3{22}</td><td>[C-]#[N+]C1COC1</td><td>61.8</td><td>45</td></tr>
<tr><td>1465</td><td>4{348,699,39}</td><td>CCOCCNC1=C(N=C2C=NC(F)=CN12)C1=CC=C(C=C1)C1(F)COC1 |c:10,13,20,22,t:6,8,18|</td><td>Z8873684846</td><td>1{348}</td><td>Nc1cnc(F)cn1</td><td>2{699}</td><td>FC1(COC1)c2ccc(C=O)cc2</td><td>3{39}</td><td>CCOCC[N+]#[C-]</td><td>45.9</td><td>45</td></tr>
<tr><td>1466</td><td>4{359,700,22}</td><td>CC(C)(C)OC(=O)N1CC(C1)C1=C(NC2COC2)N2C=C(OC(F)F)C=CC2=N1 |c:12,27,30,t:21|</td><td>Z8878918370</td><td>1{359}</td><td>Nc1ccc(OC(F)F)cn1</td><td>2{700}</td><td>CC(C)(C)OC(=O)N1CC(C1)C=O</td><td>3{22}</td><td>[C-]#[N+]C1COC1</td><td>48.6</td><td>45</td></tr>
<tr><td>1467</td><td>4{458,662,46}</td><td>CC(=C)CNC1=C(N=C2C=CC=C(Br)N12)C1=CC(=CC=C1)P(C)(C)=O |c:9,19,21,t:5,7,11,17|</td><td>Z8849597795</td><td>1{458}</td><td>Nc1cccc(Br)n1</td><td>2{662}</td><td>CP(=O)(C)c1cccc(C=O)c1</td><td>3{46}</td><td>CC(=C)C[N+]#[C-]</td><td>51.0</td><td>45</td></tr>
<tr><td>1468</td><td>4{378,701,10}</td><td>C(OC1=CC=CN2C(NC3CCOC3)=C(N=C12)C1=CN=CC=C1)C1=CN=CC=C1 |c:4,14,22,24,29,31,t:2,16,20,27|</td><td>Z8873685385</td><td>1{378}</td><td>Nc1ncccc1OCc2cccnc2</td><td>2{701}</td><td>O=Cc1cccnc1</td><td>3{10}</td><td>[C-]#[N+]C1CCOC1</td><td>47.2</td><td>45</td></tr>
<tr><td>1469</td><td>4{66,700,22}</td><td>COCCC1=CC2=NC(C3CN(C3)C(=O)OC(C)(C)C)=C(NC3COC3)N2C=C1 |c:30,t:4,6,20|</td><td>Z8878918304</td><td>1{66}</td><td>COCCc1ccnc(N)c1</td><td>2{700}</td><td>CC(C)(C)OC(=O)N1CC(C1)C=O</td><td>3{22}</td><td>[C-]#[N+]C1COC1</td><td>45.9</td><td>45</td></tr>
<tr><td>1470</td><td>4{50,604,18}</td><td>CCN1N=C(C2=C1C=CC=C2)C1=C(NC(C)(C)C)N2C(C=CC=C2P(C)(C)=O)=N1 |c:3,5,8,10,13,22,24,30|</td><td>Z8873684874</td><td>1{50}</td><td>CP(=O)(C)c1cccc(N)n1</td><td>2{604}</td><td>CCn1nc(C=O)c2ccccc12</td><td>3{18}</td><td>CC(C)(C)[N+]#[C-]</td><td>49.8</td><td>45</td></tr>
<tr><td>1471</td><td>4{445,564,29}</td><td>CCOC(=O)CCNC1=C(N=C2C=NC(SC)=CN12)C1=CN=NN1C |c:12,16,23,t:8,10,21|</td><td>Z8873684880</td><td>1{445}</td><td>CSc1cnc(N)cn1</td><td>2{564}</td><td>Cn1nncc1C=O</td><td>3{29}</td><td>CCOC(=O)CC[N+]#[C-]</td><td>43.8</td><td>45</td></tr>
<tr><td>1472</td><td>4{378,702,12}</td><td>CC(C)CC1=C(NCC2CCOC2)N2C=CC=C(OCC3=CN=CC=C3)C2=N1 |c:4,15,23,25,29,t:17,21|</td><td>Z8873684951</td><td>1{378}</td><td>Nc1ncccc1OCc2cccnc2</td><td>2{702}</td><td>CC(C)CC=O</td><td>3{12}</td><td>[C-]#[N+]CC1CCOC1</td><td>45.9</td><td>45</td></tr>
</table>

1473	**4**{*425* ,*32* ,*46* }	CC(C)OC1=NC=CN2C(NCC(C)=C)=C(N=C12)[C@@H]1C[C@H]1C1=CC=NN1C |&1:18,20,r,c:6,14,26,t:4,16,24|	Z8878918354	**1**{*425* }	CC(C)Oc1nccnc1N	**2**{*32* }	Cn1nccc1[C@@H]2C[C@H]2C=O |&1:6,8,r|	**3**{*46* }	CC(=C)C[N+]#[C-]	44.1	45
1474	**4**{*141* ,*331* ,*35* }	CSCCCNC1=C(N=C2C=C(NC(C)=O)C=CN12)C1=CC2=C(OCCNC2=O)C=C1 |c:16,32,t:6,8,10,21,23|	Z8878918507	**1**{*141* }	CC(=O)Nc1ccnc(N)c1	**2**{*331* }	O=Cc1ccc2OCCNC(=O)c2c1	**3**{*35* }	CSCCC[N+]#[C-]	52.9	45
1475	**4**{*50* ,*703* ,*35* }	CSCCCNC1=C(N=C2C=CC=C(N12)P(C)(C)=O)C1=NC2=C(C=CC(Cl)=C2)C=C1 |c:10,12,25,28,31,t:6,8,21,23|	Z8829498499	**1**{*50* }	CP(=O)(C)c1cccc(N)n1	**2**{*703* }	Clc1ccc2ccc(C=O)nc2c1	**3**{*35* }	CSCCC[N+]#[C-]	55.1	44
1476	**4**{*348* ,*704* ,*39* }	CCOCCNC1=C(N=C2C=NC(F)=CN12)C1=NC(=NN1C)C1CC1 |c:10,13,20,t:6,8,18|	Z8878918625	**1**{*348* }	Nc1cnc(F)cn1	**2**{*704* }	Cn1nc(nc1C=O)C2CC2	**3**{*39* }	CCOCC[N+]#[C-]	47.4	44
1477	**4**{*88* ,*382* ,*34* }	COC(C)(C)CC1=C(NCC2CCCO2)N2C=CC(Br)=C(F)C2=N1 |c:6,17,24,t:20|	Z8878918372	**1**{*88* }	Nc1nccc(Br)c1F	**2**{*382* }	COC(C)(C)CC=O	**3**{*34* }	[C-]#[N+]CC1CCCO1	48.1	44
1478	**4**{*104* ,*705* ,*39* }	CCOCCNC1=C(CCCCCC#C)N=C2C=CC3=C(COCC3)N12 |c:6,17,t:15,19|	Z8855739223	**1**{*104* }	Nc1ccc2CCOCc2n1	**2**{*705* }	O=CCCCCCC#C	**3**{*39* }	CCOCC[N+]#[C-]	42.7	44
1479	**4**{*371* ,*395* ,*21* }	CC(C)(C)OC(=O)N[C@H]1C[C@@H](C1)C1=C(NCC2CC2)N2C=CC=C(OCCCC#N)C2=N1 |r,c:13,22,33,t:24|	Z8855619719	**1**{*371* }	Nc1ncccc1OCCCC#N	**2**{*395* }	CC(C)(C)OC(=O)N[C@H]1C[C@@H](C1)C=O	**3**{*21* }	[C-]#[N+]CC1CC1	52.7	44
1480	**4**{*330* ,*706* ,*29* }	CCOC(=O)CCNC1=C(N=C2C=CC(=CN12)S(=O)(=O)N(C)C)C1=CC(=NN1CC)C1CC1 |c:12,14,27,t:8,10,25|	Z8878918881	**1**{*330* }	CN(C)S(=O)(=O)c1ccc(N)nc1	**2**{*706* }	CCn1nc(cc1C=O)C2CC2	**3**{*29* }	CCOC(=O)CC[N+]#[C-]	56.8	44
1481	**4**{*70* ,*61* ,*35* }	CSCCCNC1=C(N=C2N1C=CN=C2C1CC1)C1=CC=C(C=C1)S(C)=O |c:8,12,14,23,25,t:6,21|	Z8829498725	**1**{*70* }	Nc1nccnc1C2CC2	**2**{*61* }	CS(=O)c1ccc(C=O)cc1	**3**{*35* }	CSCCC[N+]#[C-]	47.9	44
1482	**4**{*437* ,*217* ,*67* }	CCC(C)NC1=C(N=C2C=CC3=C(NC(=N3)C(F)(F)F)N12)C1=CC(=O)N(C)C=C1 |c:9,14,30,t:5,7,11,24|	Z8873684902	**1**{*437* }	Cl.Nc1ccc2nc([nH]c2n1)C(F)(F)F	**2**{*217* }	Cn1ccc(C=O)cc1=O	**3**{*67* }	CCC(C)[N+]#[C-]	48.3	44
1483	**4**{*113* ,*707* ,*6* }	CCOC(=O)CCCNC1=C(N=C2N1C=CC=C2N1CCOCC1)C1=NC(=NC=C1)N(C)C |c:11,15,17,29,31,t:9,27|	Z8873684707	**1**{*113* }	Nc1ncccc1N2CCOCC2	**2**{*707* }	CN(C)c1nccc(C=O)n1	**3**{*6* }	CCOC(=O)CCC[N+]#[C-]	54.2	44
1484	**4**{*73* ,*430* ,*20* }	COC1=CC(C)=C(NC2=C(N=C3C=CC4=NC=CN4N23)C2=C(C)N=C(OC)C=C2)C=C1 |c:12,16,23,30,33,t:2,5,8,10,14,26|	Z8873684873	**1**{*73* }	Nc1ccc2nccn2n1	**2**{*430* }	COc1ccc(C=O)c(C)n1	**3**{*20* }	COc1ccc([N+]#[C-])c(C)c1	49.5	44

1485	**4**{*333*,*535*,*34*}	CC(C)(O)C1=CC(=NO1)C1=C(NCC2CCCO2)N2C(C=CC=C2C2=CC=NC=C2)=N1 |c:6,10,22,24,29,31,33,t:4,27|	Z8837933154	**1**{*333*}	Nc1cccc(n1)c2ccncc2	**2**{*535*}	CC(C)(O)c1cc(C=O)no1	**3**{*34*}	[C-]#[N+]CC1CCCO1	50.1	44
1486	**4**{*99*,*349*,*50*}	CC1(C)O[C@@H]2CC(C[C@@H]2O1)C1=C(NC2=CC3=C(OCCO3)C=C2)N2C=C(C=CC2=N1)S(N)(=O)=O |c:12,24,28,30,33,t:15,17|	Z8873684900	**1**{*99*}	Nc1ccc(cn1)S(=O)(=O)N	**2**{*349*}	CC1(C)O[C@@H]2CC(C[C@@H]2O1)C=O	**3**{*50*}	[C-]#[N+]c1ccc2OCCOc2c1	58.0	44
1487	**4**{*155*,*344*,*14*}	COCCNC1=C(N=C2N1C=CC=C2Cl)C1=C(C)C=NN1COC |c:7,11,13,17,20,t:5|	Z8854581167	**1**{*155*}	Nc1ncccc1Cl	**2**{*344*}	COCn1ncc(C)c1C=O	**3**{*14*}	COCC[N+]#[C-]	41.5	44
1488	**4**{*123*,*299*,*22*}	COC1=C(OC)C(C2=C(NC3COC3)N3C=CC(OCCO)=CC3=N2)=C(F)C=C1 |c:2,7,16,22,25,30,t:27|	Z8878918466	**1**{*123*}	Cl.Nc1cc(OCCO)ccn1	**2**{*299*}	COc1ccc(F)c(C=O)c1OC	**3**{*22*}	[C-]#[N+]C1COC1	41.2	44
1489	**4**{*139*,*708*,*12*}	CC1=NC(C)=C(C=C1)C1=C(NCC2CCOC2)N2C=CC(=CC2=N1)C(F)F |c:4,6,9,20,22,25,t:1|	Z8829498785	**1**{*139*}	Nc1cc(ccn1)C(F)F	**2**{*708*}	Cc1ccc(C=O)c(C)n1	**3**{*12*}	[C-]#[N+]CC1COC1	43.9	44
1490	**4**{*433*,*340*,*18*}	CCN1C=NC(=N1)C1=C(NC(C)(C)C)N2C(C=CC(C#C)=C2C)=N1 |c:3,5,8,17,21,24|	Z8873684740	**1**{*433*}	Cc1nc(N)ccc1C#C	**2**{*340*}	CCn1cnc(C=O)n1	**3**{*18*}	CC(C)(C)[N+]#[C-]	37.8	43
1491	**4**{*95*,*709*,*31*}	CSCCNC1=C(N=C2C=CC(=CN12)C(F)F)C1=CC=C(OCCO)C=C1 |c:9,11,27,t:5,7,19,21|	Z8878918594	**1**{*95*}	Nc1ccc(cn1)C(F)F	**2**{*709*}	OCCOc1ccc(C=O)cc1	**3**{*31*}	CSCC[N+]#[C-]	46.0	43
1492	**4**{*69*,*710*,*46*}	CNC(=O)C1=CC2=NC(=C(NCC(C)=C)N2C=C1)C1=C(C)N=CC(F)=C1 |c:17,20,23,26,t:4,6,8|	Z8873684779	**1**{*69*}	CNC(=O)c1ccnc(N)c1	**2**{*710*}	Cc1ncc(F)cc1C=O	**3**{*46*}	CC(=C)C[N+]#[C-]	40.9	43
1493	**4**{*99*,*382*,*34*}	COC(C)(C)CC1=C(NCC2CCCO2)N2C=C(C=CC2=N1)S(N)(=O)=O |c:6,17,19,22|	Z8878918441	**1**{*99*}	Nc1ccc(cn1)S(=O)(=O)N	**2**{*382*}	COC(C)(C)CC=O	**3**{*34*}	[C-]#[N+]CC1CCCO1	44.2	43
1494	**4**{*431*,*711*,*10*}	CC1=C(N=C2C=CC=CC2=N1)C1=C(NC2CCOC2)N2C=C(F)C=CC2=N1 |c:5,7,10,13,26,29,t:1,3,23|	Z8855619764	**1**{*431*}	Nc1ccc(F)cn1	**2**{*711*}	Cc1nc2ccccc2nc1C=O	**3**{*10*}	[C-]#[N+]C1CCOC1	42.0	43
1495	**4**{*32*,*356*,*14*}	COCCNC1=C(N=C2C=C(C=CN12)P(C)(C)=O)C1=CN(CC(C)C)N=N1 |c:9,11,27,t:5,7,20|	Z8835022892	**1**{*32*}	CP(=O)(C)c1ccnc(N)c1	**2**{*356*}	CC(C)Cn1cc(C=O)nn1	**3**{*14*}	COCC[N+]#[C-]	45.1	43
1496	**4**{*459*,*424*,*5*}	COCCCNC1=C(N=C2C=CC(OC3=CN=CC=C3)=CN12)C1=NN(C)C(=N1)C(F)(F)F |c:10,16,18,20,29,t:6,8,14,25|	Z8855739211	**1**{*459*}	Nc1ccc(Oc2cccnc2)cn1	**2**{*424*}	Cn1nc(C=O)nc1C(F)(F)F	**3**{*5*}	COCCC[N+]#[C-]	51.5	43
1497	**4**{*113*,*538*,*6*}	CCOC(=O)CCCNC1=C(N=C2N1C=CC=C2N1CCOCC1)C1=CN=CN1C |c:11,15,17,29,t:9,27|	Z8873684619	**1**{*113*}	Nc1ncccc1N2CCOCC2	**2**{*538*}	Cn1cncc1C=O	**3**{*6*}	CCOC(=O)CCC[N+]#[C-]	47.5	43

1498	**4**{*62*,*469*,*6*}	CCOC(=O)CCCNC1=C(N=C2C=C(CO)C=CN12)C1CCSC1 |c:17,t:9,11,13|	Z8873684937	**1**{*62*}	Nc1cc(CO)ccn1	**2**{*469*}	O=CC1CCSC1	**3**{*6*}	CCOC(=O)CCC[N+]#[C-]	41.8	43
1499	**4**{*103*,*293*,*5*}	COCCCNC1=C(N=C2C=NC=C(CO)N12)C1=C(F)N(C)N=C1 |c:10,19,24,t:6,8,12|	Z8873684766	**1**{*103*}	Nc1cncc(CO)n1	**2**{*293*}	Cn1ncc(C=O)c1F	**3**{*5*}	COCCC[N+]#[C-]	38.1	42
1500	**4**{*125*,*712*,*31*}	CSCCNC1=C(N=C2C=CC(CCO)=CN12)C1COC2(CCOCC2)C1 |c:9,14,t:5,7|	Z8855739254	**1**{*125*}	Nc1ccc(CCO)cn1	**2**{*712*}	O=CC1COC2(CCOCC2)C1	**3**{*31*}	CSCC[N+]#[C-]	46.2	42
1501	**4**{*46*,*17*,*9*}	CCOC1=CN=C(N=C1)C1=C(NC)N2C=CC=CC2=N1 |c:5,7,10,15,17,20,t:3|	Z8873684778	**1**{*46*}	Nc1ccccn1	**2**{*17*}	CCOc1cnc(C=O)nc1	**3**{*9*}	C[N+]#[C-]	30.7	42
1502	**4**{*153*,*155*,*49*}	CC(C)(O)C1=CC=CC2=NC(=C(NC3CC(F)(F)C3)N12)C1=CN=CN=C1 |c:6,25,27,t:4,8,10,23|	Z8855739103	**1**{*153*}	CC(C)(O)c1cccc(N)n1	**2**{*155*}	O=Cc1cncnc1	**3**{*49*}	FC1(F)CC(C1)[N+]#[C-]	40.9	42
1503	**4**{*119*,*34*,*48*}	CC(C)(C)OC(=O)N1CCCC(C1)NC1=C(N=C2C=CC(=CN12)C#N)C1=CC=C(C=C1)P(C)(C)=O |c:19,21,30,32,t:15,17,28|	Z8878918824	**1**{*119*}	Nc1ccc(C#N)cn1	**2**{*34*}	CP(=O)(C)c1ccc(C=O)cc1	**3**{*48*}	CC(C)(C)OC(=O)N1CCCC(C1)[N+]#[C-]	56.1	42
1504	**4**{*103*,*713*,*73*}	CCN1N=C(Br)C(=N1)C1=C(NC2=C(OC)C=C(OC)C=C2)N2C(C=NC=C2CO)=N1 |c:6,9,12,20,25,27,31,t:3,16|	Z8873684774	**1**{*103*}	Nc1cncc(CO)n1	**2**{*713*}	CCn1nc(Br)c(C=O)n1	**3**{*73*}	COc1ccc([N+]#[C-])c(OC)c1	53.7	42
1505	**4**{*327*,*185*,*65*}	COC(=O)C1=C(F)C2=NC(C3=CN(C)C=N3)=C(NC3CCCC3)N2C=C1 |c:4,14,27,t:7,10,16|	Z8854581146	**1**{*327*}	COC(=O)c1ccnc(N)c1F	**2**{*185*}	Cn1cnc(C=O)c1	**3**{*65*}	[C-]#[N+]C1CCCC1	40.4	42
1506	**4**{*118*,*417*,*31*}	CSCCNC1=C(N=C2C=CC=C(C(C)O)N12)C1=C2N=NN(C)C2=CC=C1 |c:9,19,21,26,28,t:5,7,11|	Z8873684753	**1**{*118*}	Cl.CC(O)c1cccc(N)n1	**2**{*417*}	Cn1nnc2c(C=O)cccc12	**3**{*31*}	CSCC[N+]#[C-]	43.2	42
1507	**4**{*185*,*56*,*8*}	COC(=O)C(CC1=CC=CC=C1)NC1=C(N=C2C=CC(=C(C)N12)S(C)(=O)=O)C1=C(C)C=NC(F)=C1 |c:8,10,18,30,33,36,t:6,14,16,20|	Z8873684859	**1**{*185*}	Cc1nc(N)ccc1S(=O)(=O)C	**2**{*56*}	Cc1cnc(F)cc1C=O	**3**{*8*}	COC(=O)C(Cc1ccccc1)[N+]#[C-]	56.1	42
1508	**4**{*50*,*153*,*18*}	CC(C)(C)NC1=C(N=C2C=CC=C(N12)P(C)(C)=O)C1=CN(CC(F)(F)F)N=N1 |c:9,11,28,t:5,7,20|	Z8873684854	**1**{*50*}	CP(=O)(C)c1cccc(N)n1	**2**{*153*}	FC(F)(F)Cn1cc(C=O)nn1	**3**{*18*}	CC(C)(C)[N+]#[C-]	46.8	42
1509	**4**{*32*,*714*,*18*}	CC(C)OC1=C(F)C=C(C=C1)C1=C(NC(C)(C)C)N2C=CC(=CC2=N1)P(C)(C)=O |c:4,7,9,12,20,22,25|	Z8878918552	**1**{*32*}	CP(=O)(C)c1ccnc(N)c1	**2**{*714*}	CC(C)Oc1ccc(C=O)cc1F	**3**{*18*}	CC(C)(C)[N+]#[C-]	47.1	42

1510	**4**{*358*,*223*,*15*}	CCNC1=C(N=C2C=C(C=CN12)C(C)(C)O)C1=CN=C(C=C1)C(=O)OC |c:7,9,20,22,t:3,5,18|	Z8873684855	**1**{*358*}	CC(C)(O)c1ccnc(N)c1	**2**{*223*}	Cl.COC(=O)c1ccc(C=O)cn1	**3**{*15*}	CC[N+]#[C-]	40.0	42
1511	**4**{*386*,*549*,*5*}	COCCCNC1=C(N=C2C=C(C)C=CN12)C1=C(C)C=C(N=C1)C(=O)OC |c:13,18,21,23,t:6,8,10|	Z8873685491	**1**{*386*}	Cc1ccnc(N)c1	**2**{*549*}	COC(=O)c1cc(C)c(C=O)cn1	**3**{*5*}	COCCC[N+]#[C-]	41.4	42
1512	**4**{*180*,*212*,*32*}	COC(=O)C1=CC2=NC(=C(NC3CCC3)N2C=C1C)C1=NOC(=C1)C(F)F |c:18,25,t:4,6,8,22|	Z8878918640	**1**{*180*}	COC(=O)c1cc(N)ncc1C	**2**{*212*}	FC(F)c1cc(C=O)no1	**3**{*32*}	[C-]#[N+]C1CCC1	42.2	41
1513	**4**{*105*,*715*,*57*}	NC(=O)C1=CC=CC2=NC(CC3CCOCC3)=C(NCC3=CC=CC=C3)N12 |c:5,23,25,t:3,7,17,21|	Z8854581144	**1**{*105*}	NC(=O)c1cccc(N)n1	**2**{*715*}	O=CCC1CCOCC1	**3**{*57*}	[C-]#[N+]Cc1ccccc1	40.8	41
1514	**4**{*83*,*716*,*18*}	CC(C)N(C)C1=NC=C(C=C1)C1=C(NC(C)(C)C)N2C=C(C=CC2=N1)P(C)(C)=O |c:7,9,12,20,22,25,t:5|	Z8878918500	**1**{*83*}	CP(=O)(C)c1ccc(N)nc1	**2**{*716*}	CC(C)N(C)c1ccc(C=O)cn1	**3**{*18*}	CC(C)(C)[N+]#[C-]	46.3	41
1515	**4**{*28*,*153*,*7*}	FC(F)(F)CN1C=C(N=N1)C1=C(NC2CCOCC2)N2C=CC(=CC2=N1)C1=NOC=N1 |c:6,8,11,22,24,27,33,t:30|	Z8878918496	**1**{*28*}	Cl.Nc1cc(ccn1)c2ncon2	**2**{*153*}	FC(F)(F)Cn1cc(C=O)nn1	**3**{*7*}	[C-]#[N+]C1CCOCC1	48.6	41
1516	**4**{*32*,*717*,*18*}	COC1=CC(=CC=C1)C1=NC(=CS1)C1=C(NC(C)(C)C)N2C=CC(=CC2=N1)P(C)(C)=O |c:4,6,11,15,23,25,28,t:2,9|	Z8837933157	**1**{*32*}	CP(=O)(C)c1ccnc(N)c1	**2**{*717*}	COc1cccc(c1)c2nc(C=O)cs2	**3**{*18*}	CC(C)(C)[N+]#[C-]	50.7	41
1517	**4**{*183*,*148*,*51*}	COC1=C(C(C)=NN1C)C1=C(NCCNC(=O)OC(C)(C)C)N2C(=N1)C(OC)=CC=C2C |c:5,10,24,29,31,t:2|	Z8835022879	**1**{*183*}	COc1ccc(C)nc1N	**2**{*148*}	COc1c(C=O)c(C)nn1C	**3**{*51*}	CC(C)(C)OC(=O)NCC[N+]#[C-]	49.6	41
1518	**4**{*79*,*26*,*9*}	CNC1=C(CCC2=CC=CC=C2)N=C2C=NC=C(C)N12 |c:2,8,10,15,t:6,13,17|	Z8837933185	**1**{*79*}	Cc1cncc(N)n1	**2**{*26*}	O=CCCc1ccccc1	**3**{*9*}	C[N+]#[C-]	29.5	41
1519	**4**{*52*,*683*,*14*}	COCCNC1=C(N=C2C=CC(=CN12)C1(CC1)C(F)(F)F)C1=C(C)N(C)N=C1Br |c:9,11,24,29,t:5,7|	Z8854581180	**1**{*52*}	Nc1ccc(cn1)C2(CC2)C(F)(F)F	**2**{*683*}	Cc1c(C=O)c(Br)nn1C	**3**{*14*}	COCC[N+]#[C-]	52.3	41
1520	**4**{*149*,*317*,*7*}	CCN1N=CC(=N1)C1=C(NC2CCOCC2)N2C=C(Cl)C=C(Br)C2=N1 |c:3,5,8,26,t:19,22|	Z8878918369	**1**{*149*}	Nc1ncc(Cl)cc1Br	**2**{*317*}	CCn1ncc(C=O)n1	**3**{*7*}	[C-]#[N+]C1CCOCC1	47.1	41
1521	**4**{*101*,*707*,*14*}	COCCNC1=C(N=C2N1C=C(CO)C=C2Cl)C1=NC(=NC=C1)N(C)C |c:7,15,21,23,t:5,11,19|	Z8878918821	**1**{*101*}	Nc1ncc(CO)cc1Cl	**2**{*707*}	CN(C)c1nccc(C=O)n1	**3**{*14*}	COCC[N+]#[C-]	41.7	41
1522	**4**{*187*,*567*,*12*}	CN1N=C2CCCC2=C1C1=C(NCC2CCOC2)N2C=C(Cl)C(=CC2=N1)C#N |c:8,11,25,28,t:2,22|	Z8878918514	**1**{*187*}	Nc1cc(C#N)c(Cl)cn1	**2**{*567*}	Cn1nc2CCCc2c1C=O	**3**{*12*}	[C-]#[N+]CC1CCOC1	43.9	41

1523	**4**{*142*,*319*,*21*}	CC1=C(C=CC(OCC#N)=C1)C1=C(NCC2CC2)N2C=CC=C(OCCS(C)(=O)=O)C2=N1 |c:3,9,12,21,33,t:1,23|	Z8878918666	**1**{*142*}	CS(=O)(=O)CCOc1cccnc1N	**2**{*319*}	Cc1cc(OCC#N)ccc1C=O	**3**{*21*}	[C-]#[N+]CC1CC1	50.3	41
1524	**4**{*32*,*604*,*18*}	CCN1N=C(C2=C1C=CC=C2)C1=C(NC(C)(C)C)N2C=CC(=CC2=N1)P(C)(C)=O |c:3,5,8,10,13,21,23,26|	Z8837933166	**1**{*32*}	CP(=O)(C)c1ccnc(N)c1	**2**{*604*}	CCn1nc(C=O)c2ccccc12	**3**{*18*}	CC(C)(C)[N+]#[C-]	45.3	41
1525	**4**{*460*,*690*,*10*}	CC1=CC=CN2C(NC3CCOC3)=C(CCC3(CCN(CC3)C(=O)OC(C)(C)C)C#N)N=C12 |c:3,t:1,13,33|	Z8873685453	**1**{*460*}	Cc1cccnc1N	**2**{*690*}	CC(C)(C)OC(=O)N1CCC(CCC=O)(CC1)C#N	**3**{*10*}	[C-]#[N+]C1CCOC1	50.0	41
1526	**4**{*461*,*217*,*62*}	COC(=O)C1(CCCC1)NC1=C(N=C2C=C(C#N)C(Br)=CN12)C1=CC(=O)N(C)C=C1 |c:20,31,t:11,13,15,25|	Z8878918323	**1**{*461*}	Nc1cc(C#N)c(Br)cn1	**2**{*217*}	Cn1ccc(C=O)cc1=O	**3**{*62*}	COC(=O)C1(CCCC1)[N+]#[C-]	51.7	41
1527	**4**{*377*,*662*,*24*}	COC1=CC(CNC2=C(N=C3N2C=CC=C3OCC(F)F)C2=CC(=CC=C2)P(C)(C)=O)=CC=C1 |c:9,13,15,25,27,33,35,t:2,7,23|	Z8878918896	**1**{*377*}	Nc1ncccc1OCC(F)F	**2**{*662*}	CP(=O)(C)c1cccc(C=O)c1	**3**{*24*}	COc1cccc(C[N+]#[C-])c1	53.2	41
1528	**4**{*172*,*320*,*29*}	CCOC(=O)CCNC1=C(N=C2N1C=CC1=C2CCO1)C1=NN(C)C=N1 |c:10,14,16,27,t:8,23|	Z8873684906	**1**{*172*}	Nc1nccc2OCCc21	**2**{*320*}	Cn1cnc(C=O)n1	**3**{*29*}	CCOC(=O)CC[N+]#[C-]	39.0	41
1529	**4**{*396*,*324*,*70*}	CN1C=C(C=N1)C1=NN2C(C=C1)=NC(C1CC3(C1)CCCO3)=C2NC1CCN(C1)C(=O)OC(C)(C)C |c:2,4,11,13,25,t:7|	Z8873684660	**1**{*396*}	Cn1cc(cn1)c2ccc(N)nn2	**2**{*324*}	O=CC1CC2(C1)CCCO2	**3**{*70*}	CC(C)(C)OC(=O)N1CCC(C1)[N+]#[C-]	54.0	41
1530	**4**{*68*,*13*,*38*}	CC1=C(N=NN1)C1=C(NC2=CC3=C(OCO3)C=C2)N2C=CC(=CC2=N1)C#C |c:3,7,18,22,24,27,t:1,10,12|	Z8837933156	**1**{*68*}	Nc1cc(C#C)ccn1	**2**{*13*}	Cc1[nH]nnc1C=O	**3**{*38*}	[C-]#[N+]c1ccc2OCOc2c1	39.3	40
1531	**4**{*54*,*718*,*37*}	COC(=O)C1CC(C1)NC1=C(N=C2C=CC(=CN12)C(=O)N(C)C)C1=C(Br)C=CS1 |c:14,16,26,29,t:10,12|	Z8878918615	**1**{*54*}	Cl.CN(C)C(=O)c1ccc(N)nc1	**2**{*718*}	Brc1ccsc1C=O	**3**{*37*}	COC(=O)C1CC(C1)[N+]#[C-]	52.1	40
1532	**4**{*327*,*47*,*34*}	COC(=O)C1=C(F)C2=NC(CCOCC=C)=C(NC3CCCO3)N2C=C1 |c:4,27,t:7,15|	Z8855739171	**1**{*327*}	COC(=O)c1ccnc(N)c1F	**2**{*47*}	C=CCOCCC=O	**3**{*34*}	[C-]#[N+]CC1CCCO1	41.1	40
1533	**4**{*73*,*29*,*11*}	CCN1C=CC(=N1)C1=C(NCC2=CC(Br)=CC=C2)N2N3C=CN=C3C=CC2=N1 |c:3,5,8,15,17,22,24,27,30,t:12|	Z8873685497	**1**{*73*}	Nc1ccc2nccn2n1	**2**{*29*}	CCn1ccc(C=O)n1	**3**{*11*}	Brc1cccc(C[N+]#[C-])c1	47.5	40
1534	**4**{*434*,*189*,*26*}	CC(F)(F)C1=CN2C(NCC3=CC=C(F)C=C3)=C(N=C2C=C1)C1=CN(N=N1)C1CCOCC1 |c:15,17,19,22,28,t:4,10,12,25|	Z8878918893	**1**{*434*}	CC(F)(F)c1ccc(N)nc1	**2**{*189*}	O=Cc1cn(nn1)C2CCOCC2	**3**{*26*}	Fc1ccc(C[N+]#[C-])cc1	49.6	40

1535	**4**{*101*,*192*,*14*}	COCCNC1=C(N=C2N1C=C(CO)C=C2Cl)C1=NC(OC)=CC=C1 |c:7,15,23,25,t:5,11,19|	Z8878918840	**1**{*101*}	Nc1ncc(CO)cc1Cl	**2**{*192*}	COc1cccc(C=O)n1	**3**{*14*}	COCC[N+]#[C-]	39.3	40
1536	**4**{*38*,*420*,*9*}	CNC1=C(CC(C)(C)C#N)N=C2C=C(C=CN12)C#N |c:2,12,14,t:10|	Z8854581149	**1**{*38*}	Nc1cc(C#N)ccn1	**2**{*420*}	CC(C)(CC=O)C#N	**3**{*9*}	C[N+]#[C-]	27.3	40
1537	**4**{*327*,*638*,*34*}	COC(=O)C1=C(F)C2=NC(=C(NCC3CCCO3)N2C=C1)C1=CN(CC2CCOCC2)N=C1 |c:4,21,35,t:7,9,24|	Z8878918578	**1**{*327*}	COC(=O)c1ccnc(N)c1F	**2**{*638*}	O=Cc1cnn(CC2CCOCC2)c1	**3**{*34*}	[C-]#[N+]CC1CCCO1	49.3	40
1538	**4**{*456*,*189*,*18*}	CC(C)(C)NC1=C(N=C2C=C(OCCNC(=O)OC(C)(C)C)C=CN12)C1=CN(N=N1)C1CCOCC1 |c:22,30,t:5,7,9,27|	Z8878918782	**1**{*456*}	CC(C)(C)OC(=O)NCCOc1ccnc(N)c1	**2**{*189*}	O=Cc1cn(nn1)C2CCOCC2	**3**{*18*}	CC(C)(C)[N+]#[C-]	53.7	40
1539	**4**{*105*,*434*,*29*}	CCOC(=O)CCNC1=C(N=C2C=CC=C(N12)C(N)=O)C1=NC(C)=CC(OC)=C1 |c:12,14,25,29,t:8,10,22|	Z8878918662	**1**{*105*}	NC(=O)c1cccc(N)n1	**2**{*434*}	COc1cc(C)nc(C=O)c1	**3**{*29*}	CCOC(=O)CC[N+]#[C-]	42.7	40
1540	**4**{*83*,*50*,*18*}	CC(C)(C)NC1=C(N=C2C=CC(=CN12)P(C)(C)=O)[C@@H]1C[C@H]1C1OCCO1 |&1:18,20,r,c:9,11,t:5,7|	Z8878918397	**1**{*83*}	CP(=O)(C)c1ccc(N)nc1	**2**{*50*}	O=C[C@@H]1C[C@H]1C2OCCO2 |&1:2,4,r|	**3**{*18*}	CC(C)(C)[N+]#[C-]	40.5	40
1541	**4**{*73*,*142*,*13*}	COC1=CC=C(CNC2=C(N=C3C=CC4=NC=CN4N23)C2=C(CCC=C)C=CC=C2)C=C1 |c:12,16,23,29,31,34,t:2,4,8,10,14|	Z8855739261	**1**{*73*}	Nc1ccc2nccn2n1	**2**{*142*}	C=CCCc1ccccc1C=O	**3**{*13*}	COc1ccc(C[N+]#[C-])cc1	45.4	40
1542	**4**{*360*,*564*,*29*}	CCOC(=O)CCNC1=C(N=C2C=C(C(=O)OC)C(Br)=CN12)C1=CN=NN1C |c:19,26,t:8,10,12,24|	Z8878918408	**1**{*360*}	COC(=O)c1cc(N)ncc1Br	**2**{*564*}	Cn1nncc1C=O	**3**{*29*}	CCOC(=O)CC[N+]#[C-]	48.3	40
1543	**4**{*36*,*170*,*9*}	CCN1N=NC(=N1)C1=C(NC)N2C=C(Br)C=CC2=N1 |c:3,5,8,16,19,t:13|	Z8873685407	**1**{*36*}	Nc1ccc(Br)cn1	**2**{*170*}	CCn1nnc(C=O)n1	**3**{*9*}	C[N+]#[C-]	34.4	40
1544	**4**{*48*,*719*,*22*}	CS(=O)(=O)C1=CC2=NC(=C(NC3COC3)N2C=C1)C1=C(Cl)C=C(Br)C=C1 |c:18,21,27,t:4,6,8,24|	Z8873684833	**1**{*48*}	CS(=O)(=O)c1ccnc(N)c1	**2**{*719*}	Clc1cc(Br)ccc1C=O	**3**{*22*}	[C-]#[N+]C1COC1	48.6	39
1545	**4**{*83*,*720*,*26*}	CC[C@@H]1C[C@H]1C1=C(NCC2=CC=C(F)C=C2)N2C=C(C=CC2=N1)P(C)(C)=O |&1:2,4,r,c:6,15,19,21,24,t:10,12|	Z8837933151	**1**{*83*}	CP(=O)(C)c1ccc(N)nc1	**2**{*720*}	CC[C@@H]1C[C@H]1C=O |&1:2,4,r|	**3**{*26*}	Fc1ccc(C[N+]#[C-])cc1	40.9	39
1546	**4**{*175*,*405*,*12*}	CC(F)(F)C1=NC=C(S1)C1=C(NCC2CCOC2)N2C=CC=C(OCCF)C2=N1 |c:6,10,21,30,t:4,23|	Z8878918883	**1**{*175*}	Nc1ncccc1OCCF	**2**{*405*}	CC(F)(F)c1ncc(C=O)s1	**3**{*12*}	[C-]#[N+]CC1CCOC1	45.3	39

1547	**4**{*32 ,640 ,18* }	CC(C)N1C=C(N=N1)C1=C(NC(C)(C)C)N2C=CC(=CC2=N1)P(C)(C)=O |c:4,6,9,17,19,22|	Z8837933170	**1**{*32* }	CP(=O)(C)c1ccnc(N)c1	**2**{*640* }	CC(C)n1cc(C=O)nn1	**3**{*18* }	CC(C)(C)[N+]#[C-]	39.7	39
1548	**4**{*462 ,352 ,70* }	COCCCC1=C(NC2CCN(C2)C(=O)OC(C)(C)C)N2C=C(Br)C=C(CO)C2=N1 |c:5,30,t:22,25|	Z8873684582	**1**{*462* }	Nc1ncc(Br)cc1CO	**2**{*352* }	COCCCC=O	**3**{*70* }	CC(C)(C)OC(=O)N1CCC(C1)[N+]#[C-]	51.3	39
1549	**4**{*32 ,439 ,7* }	COC1=C(C=CC(=C1)C1=C(NC2CCOCC2)N2C=CC(=CC2=N1)P(C)(C)=O)S(C)(=O)=O |c:4,6,9,20,22,25,t:2|	Z8873684658	**1**{*32* }	CP(=O)(C)c1ccnc(N)c1	**2**{*439* }	COc1cc(C=O)ccc1S(=O)(=O)C	**3**{*7* }	[C-]#[N+]C1CCOCC1	50.7	39
1550	**4**{*372 ,721 ,12* }	COC(=O)C1=CC(=CN=C1)C1=C(NCC2CCOC2)N2C=CC=C(OCC3=CC=NC=C3)C2=N1 |c:6,8,11,22,30,32,36,t:4,24,28|	Z8878918479	**1**{*372* }	Nc1ncccc1OCc2ccncc2	**2**{*721* }	COC(=O)c1cncc(C=O)c1	**3**{*12* }	[C-]#[N+]CC1CCOC1	48.7	39
1551	**4**{*193 ,722 ,12* }	CNC(=O)C1=CN2C(C=C1)=NC(=C2NCC1CCOC1)C1=CC=C(C=C1)N1CCN(C)CC1 |c:8,10,12,25,27,t:4,23|	Z8849597829	**1**{*193* }	CNC(=O)c1ccc(N)nc1	**2**{*722* }	CN1CCN(CC1)c2ccc(C=O)cc2	**3**{*12* }	[C-]#[N+]CC1CCOC1	47.6	39
1552	**4**{*123 ,689 ,22* }	CC(=O)OCCN1C=C(C=N1)C1=C(NC2COC2)N2C=CC(OCCO)=CC2=N1 |c:7,9,12,21,27,30|	Z8878918424	**1**{*123* }	Cl.Nc1cc(OCCO)ccn1	**2**{*689* }	CC(=O)OCCn1cc(C=O)cn1	**3**{*22* }	[C-]#[N+]C1COC1	38.9	39
1553	**4**{*73 ,723 ,13* }	CCCC(C)C1=C(NCC2=CC=C(OC)C=C2)N2N3C=CN=C3C=CC2=N1 |c:5,15,20,22,25,28,t:9,11|	Z8854581156	**1**{*73* }	Nc1ccc2nccn2n1	**2**{*723* }	CCCC(C)C=O	**3**{*13* }	COc1ccc(C[N+]#[C-])cc1	38.4	39
1554	**4**{*83 ,340 ,18* }	CCN1C=NC(=N1)C1=C(NC(C)(C)C)N2C=C(C=CC2=N1)P(C)(C)=O |c:3,5,8,16,18,21|	Z8878918361	**1**{*83* }	CP(=O)(C)c1ccc(N)nc1	**2**{*340* }	CCn1cnc(C=O)n1	**3**{*18* }	CC(C)(C)[N+]#[C-]	38.1	39
1555	**4**{*144 ,724 ,15* }	CCNC1=C(N=C2C=CC(=CN12)S(=O)(=O)NC)C1=C(OC)C=CC=C1F |c:7,9,19,23,25,t:3,5|	Z8873684668	**1**{*144* }	CNS(=O)(=O)c1ccc(N)nc1	**2**{*724* }	COc1cccc(F)c1C=O	**3**{*15* }	CC[N+]#[C-]	40.0	39
1556	**4**{*133 ,61 ,22* }	CS(=O)C1=CC=C(C=C1)C1=C(NC2COC2)N2C=C(F)C=C(Br)C2=N1 |c:5,7,10,26,t:3,19,22|	Z8873684901	**1**{*133* }	Nc1ncc(F)cc1Br	**2**{*61* }	CS(=O)c1ccc(C=O)cc1	**3**{*22* }	[C-]#[N+]C1COC1	44.5	39
1557	**4**{*393 ,725 ,5* }	CCN1N=NC=C1C1=C(NCCCOC)N2C(C=CC(Cl)=C2Cl)=N1 |c:3,5,8,18,21,24|	Z8878918309	**1**{*393* }	Nc1ccc(Cl)c(Cl)n1	**2**{*725* }	CCn1nncc1C=O	**3**{*5* }	COCCC[N+]#[C-]	38.6	39
1558	**4**{*372 ,726 ,12* }	CS(=O)(=O)C1=CC(=CC=C1)C1=C(NCC2CCOC2)N2C=CC=C(OCC3=CC=NC=C3)C2=N1 |c:6,8,11,22,30,32,36,t:4,24,28|	Z8878918471	**1**{*372* }	Nc1ncccc1OCc2ccncc2	**2**{*726* }	CS(=O)(=O)c1cccc(C=O)c1	**3**{*12* }	[C-]#[N+]CC1CCOC1	49.9	39

1559	**4**{*347,536,46*}	CC(=C)CNC1=C(N=C2C=CC(Cl)=CN12)C1=CN=C(O1)C1CCOCC1 |c:9,12,19,t:5,7,17|	Z8849597802	**1**{*347*}	Nc1ccc(Cl)cn1	**2**{*536*}	O=Cc1cnc(o1)C2CCOCC2	**3**{*46*}	CC(=C)C[N+]#[C-]	38.6	38
1560	**4**{*366,721,10*}	COC(=O)C1=CC(=CN=C1)C1=C(NC2CCOC2)N2C=CC=C(OC)C2=N1 |c:6,8,11,21,28,t:4,23|	Z8849597815	**1**{*366*}	COc1cccnc1N	**2**{*721*}	COC(=O)c1cncc(C=O)c1	**3**{*10*}	[C-]#[N+]C1CCOC1	38.1	38
1561	**4**{*212,155,76*}	COCC1=CN2C(C=C1)=NC(=C2NC1CN(C1)C(=O)OC(C)(C)C)C1=CN=CN=C1 |c:7,9,11,29,31,t:3,27|	Z8873684912	**1**{*212*}	Cl.COCc1ccc(N)nc1	**2**{*155*}	O=Cc1cncnc1	**3**{*76*}	CC(C)(C)OC(=O)N1CC(C1)[N+]#[C-]	42.4	38
1562	**4**{*146,180,51*}	CC(C)(C)OC(=O)NCCNC1=C(CCCCCF)N=C2C=CC(=CN12)S(C)(=O)=O |c:11,21,23,t:19|	Z8878918796	**1**{*146*}	CS(=O)(=O)c1ccc(N)nc1	**2**{*180*}	FCCCCCC=O	**3**{*51*}	CC(C)(C)OC(=O)NCC[N+]#[C-]	45.7	38
1563	**4**{*79,25,20*}	COC1=CC(C)=C(NC2=C(N=C3C=NC=C(C)N23)C2=CN=C(N=C2)C2CCOCC2)C=C1 |c:12,22,24,34,t:2,5,8,10,14,20|	Z8878918845	**1**{*79*}	Cc1cncc(N)n1	**2**{*25*}	O=Cc1cnc(nc1)C2CCOCC2	**3**{*20*}	COc1ccc([N+]#[C-])c(C)c1	44.3	38
1564	**4**{*47,727,6*}	CCOC(=O)CCCNC1=C(N=C2C=C(C=CN12)S(N)(=O)=O)C1=CN=C(N=C1)C(C)C |c:13,15,26,28,t:9,11,24|	Z8849597798	**1**{*47*}	Cl.Nc1cc(ccn1)S(=O)(=O)N	**2**{*727*}	CC(C)c1ncc(C=O)cn1	**3**{*6*}	CCOC(=O)CCC[N+]#[C-]	45.9	38
1565	**4**{*79,728,9*}	CNC1=C(N=C2C=NC=C(C)N12)C1=CN=C(S1)C1CCC1 |c:6,16,t:2,4,8,14|	Z8878918882	**1**{*79*}	Cc1cncc(N)n1	**2**{*728*}	O=Cc1cnc(s1)C2CCC2	**3**{*9*}	C[N+]#[C-]	30.6	38
1566	**4**{*91,555,26*}	CC1=NN(C(C)=C1C1=C(NCC2=CC=C(F)C=C2)N2C=C(C=CC2=N1)C(N)=O)C1=NC=CC=C1 |c:5,8,17,21,23,26,34,36,t:1,12,14,32|	Z8873684863	**1**{*91*}	NC(=O)c1ccc(N)nc1	**2**{*555*}	Cc1nn(c(C)c1C=O)c2ccccn2	**3**{*26*}	Fc1ccc(C[N+]#[C-])cc1	46.6	38
1567	**4**{*59,729,49*}	COC1=C(C=CC=C1C#N)C1=C(NC2CC(F)(F)C2)N2C=C(CN3CCOCC3)C=CC2=N1 |c:4,6,11,32,35,t:2,22|	Z8873684701	**1**{*59*}	Nc1ccc(CN2CCOCC2)cn1	**2**{*729*}	COc1c(C=O)cccc1C#N	**3**{*49*}	FC1(F)CC(C1)[N+]#[C-]	46.2	38
1568	**4**{*239,119,37*}	CCOC(=O)C1=NN=C(N1)C1=C(NC2CC(C2)C(=O)OC)N2C=C(C=CC2=N1)N1CCCC1 |c:7,11,24,26,29,t:5|	Z8873684590	**1**{*239*}	Cl.Cl.Nc1ccc(cn1)N2CCCC2	**2**{*119*}	CCOC(=O)c1nnc(C=O)[nH]1	**3**{*37*}	COC(=O)C1CC(C1)[N+]#[C-]	46.1	38
1569	**4**{*351,730,28*}	CCCNC1=C(N=C2C=CC3=C(C(O)CC3)N12)C1=CN(N=C1C)C(C)(C)C |c:8,23,t:4,6,10,20|	Z8873684849	**1**{*351*}	Nc1ccc2CCC(O)c2n1	**2**{*730*}	Cc1nn(cc1C=O)C(C)(C)C	**3**{*28*}	CCC[N+]#[C-]	37.4	38
1570	**4**{*49,197,13*}	COC(=O)C1=CC(=NN1C)C1=C(NCC2=CC=C(OC)C=C2)N2C(C=CC=C2CO)=N1 |c:6,11,21,26,28,32,t:4,15,17|	Z8873684639	**1**{*49*}	Nc1cccc(CO)n1	**2**{*197*}	COC(=O)c1cc(C=O)nn1C	**3**{*13*}	COc1ccc(C[N+]#[C-])cc1	42.9	38

1571	**4**{*463,135,49*}	CN1N=C(C)C(=C1C)C1=C(NC2CC(F)(F)C2)N2C=C(C=CC2=N1)C(=O)OC(C)(C)C |c:5,9,20,22,25,t:2|	Z8849597818	**1**{*463*}	CC(C)(C)OC(=O)c1cccC(N)nc1	**2**{*135*}	Cc1nn(C)c(C)c1C=O	**3**{*49*}	FC1(F)CC(C1)[N+]#[C-]	43.9	38
1572	**4**{*83,731,31*}	COC1=C(C=CC(Br)=C1)C1=C(NCCSC)N2C=C(C=CC2=N1)P(C)(C)=O |c:4,7,10,18,20,23,t:2|	Z8873685488	**1**{*83*}	CP(=O)(C)c1ccc(N)nc1	**2**{*731*}	COc1cc(Br)ccc1C=O	**3**{*31*}	CSCC[N+]#[C-]	47.6	38
1573	**4**{*139,375,51*}	COCC1=C(N=CO1)C1=C(NCCNC(=O)OC(C)(C)C)N2C=CC(=CC2=N1)C(F)F |c:5,9,23,25,28,t:3|	Z8878918813	**1**{*139*}	Nc1cc(ccn1)C(F)F	**2**{*375*}	COCc1ocnc1C=O	**3**{*51*}	CC(C)(C)OC(=O)NCC[N+]#[C-]	44.4	38
1574	**4**{*358,732,15*}	CCNC1=C(N=C2C=C(C=CN12)C(C)(C)O)[C@@H]1C[C@H]1C1CC1 |&1:16,18,r,c:7,9,t:3,5|	Z8878918454	**1**{*358*}	CC(C)(O)c1ccnc(N)c1	**2**{*732*}	O=C[C@@H]1C[C@H]1C2CC2 |&1:2,4,r|	**3**{*15*}	CC[N+]#[C-]	30.3	37
1575	**4**{*41,733,13*}	COC1=CC=C(CNC2=C(N=C3C=NC(C)=CN23)C2=NN(N=N2)C(C)(C)C)C=C1 |c:12,15,23,30,t:2,4,8,10,20|	Z8855739216	**1**{*41*}	Cc1cnc(N)cn1	**2**{*733*}	CC(C)(C)n1nnc(C=O)n1	**3**{*13*}	COc1ccc(C[N+]#[C-])cc1	39.7	37
1576	**4**{*83,146,18*}	C[C@@H]1C[C@@H]1CCC1=C(NC(C)(C)C)N2C=C(C=CC2=N1)P(C)(C)=O |&1:1,3,r,c:7,15,17,20|	Z8878918375	**1**{*83*}	CP(=O)(C)c1ccc(N)nc1	**2**{*146*}	C[C@@H]1C[C@@H]1CCC=O |&1:1,3,r|	**3**{*18*}	CC(C)(C)[N+]#[C-]	35.1	37
1577	**4**{*41,95,9*}	CNC1=C(N=C2C=NC(C)=CN12)C1=CC(=NC=C1)N(C)C |c:6,9,16,18,t:2,4,14|	Z8855739107	**1**{*41*}	Cc1cnc(N)cn1	**2**{*95*}	CN(C)c1cc(C=O)ccn1	**3**{*9*}	C[N+]#[C-]	28.5	37
1578	**4**{*188,734,62*}	COC(=O)C1=CN=CC2=NC(=C(NC3(CCCC3)C(=O)OC)N12)C1=C(C)C(=NN1)C(F)(F)F |c:6,26,29,t:4,8,10|	Z8835022882	**1**{*188*}	COC(=O)c1cncc(N)n1	**2**{*734*}	Cc1c(C=O)[nH]nc1C(F)(F)F	**3**{*62*}	COC(=O)C1(CCCC1)[N+]#[C-]	47.0	37
1579	**4**{*62,94,11*}	OCC1=CC2=NC(=C(NCC3=CC(Br)=CC=C3)N2C=C1)C1=NC=CN=C1 |c:13,15,20,25,27,t:2,4,6,10,23|	Z8873684641	**1**{*62*}	Nc1cc(CO)ccn1	**2**{*94*}	O=Cc1cnccn1	**3**{*11*}	Brc1cccc(C[N+]#[C-])c1	41.0	37
1580	**4**{*69,369,24*}	CNC(=O)C1=CC2=NC(=C(NCC3=CC(OC)=CC=C3)N2C=C1)C1=CON=C1C |c:16,18,23,29,t:4,6,8,12,26|	Z8878918887	**1**{*69*}	CNC(=O)c1ccnc(N)c1	**2**{*369*}	Cc1nocc1C=O	**3**{*24*}	COc1cccc(C[N+]#[C-])c1	38.7	37
1581	**4**{*359,735,22*}	COC(=O)C1=CN=C(S1)C1=C(NC2COC2)N2C=C(OC(F)F)C=CC2=N1 |c:6,10,25,28,t:4,19|	Z8854581181	**1**{*359*}	Nc1ccc(OC(F)F)cn1	**2**{*735*}	COC(=O)c1cnc(C=O)s1	**3**{*22*}	[C-]#[N+]C1COC1	39.2	37
1582	**4**{*174,21,43*}	CC(C)(C)OC(=O)N1CCC(CC1)NC1=C(N=C2C=CC=C(C#N)N12)C1=CC2=NC=NN2C=C1 |c:19,32,36,t:15,17,21,28,30|	Z8873685273	**1**{*174*}	Nc1cccc(C#N)n1	**2**{*21*}	O=Cc1ccn2ncnc2c1	**3**{*43*}	CC(C)(C)OC(=O)N1CCC(CC1)[N+]#[C-]	45.0	36

1583	**4**{*166* ,*168* ,*35* }	CSCCCNC1=C(N=C2C=C(CSCCO)C=CN12)C1=NOC=C1 |c:17,25,t:6,8,10,22|	Z8854581179	**1**{*166* }	Nc1cc(CSCCO)ccn1	**2**{*168* }	O=Cc1ccon1	**3**{*35* }	CSCCC[N+]#[C-]	36.8	36
1584	**4**{*99* ,*736* ,*34* }	CC1(C)CC(C1)C1=C(NCC2CCCO2)N2C=C(C=CC2=N1)S(N)(=O)=O |c:7,18,20,23|	Z8835022874	**1**{*99* }	Nc1ccc(cn1)S(=O)(=O)N	**2**{*736* }	CC1(C)CC(C1)C=O	**3**{*34* }	[C-]#[N+]CC1CCCO1	36.8	36
1585	**4**{*28* ,*737* ,*7* }	FC1=C(C=CC(=C1)C1=C(NC2CCOCC2)N2C=CC(=CC2=N1)C1=NOC=N1)C#C |c:3,5,8,19,21,24,30,t:1,27|	Z8873684597	**1**{*28* }	Cl.Nc1cc(ccn1)c2ncon2	**2**{*737* }	Fc1cc(C=O)ccc1C#C	**3**{*7* }	[C-]#[N+]C1CCOCC1	39.0	36
1586	**4**{*180* ,*168* ,*32* }	COC(=O)C1=CC2=NC(=C(NC3CCC3)N2C=C1C)C1=NOC=C1 |c:18,25,t:4,6,8,22|	Z8878918534	**1**{*180* }	COC(=O)c1cc(N)ncc1C	**2**{*168* }	O=Cc1ccon1	**3**{*32* }	[C-]#[N+]C1CCC1	31.4	36
1587	**4**{*464* ,*390* ,*5* }	COCCCNC1=C(N=C2C=CC(=CN12)N1CCN(C)CC1)C1CC2C(C1)C2(F)F |c:10,12,t:6,8|	Z8855619556	**1**{*464* }	CN1CCN(CC1)c2ccc(N)nc2	**2**{*390* }	FC1(F)C2CC(CC21)C=O	**3**{*5* }	COCCC[N+]#[C-]	40.3	36
1588	**4**{*367* ,*364* ,*34* }	CC1=CN2C(NCC3CCCO3)=C(CCC3CCCO3)N=C2C(=C1)C#C |c:22,25,t:1,12|	Z8878918416	**1**{*367* }	Cc1cnc(N)c(C#C)c1	**2**{*364* }	O=CCCC1CCCO1	**3**{*34* }	[C-]#[N+]CC1CCCO1	33.8	35
1589	**4**{*73* ,*53* ,*20* }	COC1=CC(C)=C(NC2=C(N=C3C=CC4=NC=CN4N23)C2=C(F)C=CC(C)=N2)C=C1 |c:12,16,23,26,29,32,t:2,5,8,10,14|	Z8878918602	**1**{*73* }	Nc1ccc2nccn2n1	**2**{*53* }	Cc1ccc(F)c(C=O)n1	**3**{*20* }	COc1ccc([N+]#[C-])c(C)c1	38.5	35
1590	**4**{*48* ,*99* ,*15* }	CCNC1=C(N=C2C=C(C=CN12)S(C)(=O)=O)C1=COC=N1 |c:7,9,21,t:3,5,18|	Z8873685462	**1**{*48* }	CS(=O)(=O)c1ccnc(N)c1	**2**{*99* }	O=Cc1cocn1	**3**{*15* }	CC[N+]#[C-]	29.2	35
1591	**4**{*439* ,*148* ,*10* }	COC1=C(C(C)=NN1C)C1=C(NC2CCOC2)N2C=CC=C(OCC3=CC=CC=C3)C2=N1 |c:5,10,20,28,30,34,t:2,22,26|	Z8849597809	**1**{*439* }	Nc1ncccc1OCc2ccccc2	**2**{*148* }	COc1c(C=O)c(C)nn1C	**3**{*10* }	[C-]#[N+]C1CCOC1	41.3	35
1592	**4**{*73* ,*88* ,*11* }	BrC1=CC(CNC2=C(N=C3C=CC4=NC=CN4N23)C2=CC3=C(C=CC=N3)C=C2)=CC=C1 |c:10,14,25,27,30,32,34,t:1,6,8,12,21,23|	Z8873684895	**1**{*73* }	Nc1ccc2nccn2n1	**2**{*88* }	O=Cc1ccc2cccnc2c1	**3**{*11* }	Brc1cccc(C[N+]#[C-])c1	44.5	35
1593	**4**{*36* ,*672* ,*14* }	COCCNC1=C(N=C2C=CC(Br)=CN12)C1=C(C)SC=N1 |c:9,12,17,21,t:5,7|	Z8846492100	**1**{*36* }	Nc1ccc(Br)cn1	**2**{*672* }	Cc1scnc1C=O	**3**{*14* }	COCC[N+]#[C-]	34.5	35
1594	**4**{*405* ,*587* ,*38* }	COC1=NOC(C)=C1C1=C(NC2=CC3=C(OCO3)C=C2)N2C=C(C=CC2=N1)N1CCCC1=O |c:6,9,20,24,26,29,t:2,12,14|	Z8878918646	**1**{*405* }	Nc1ccc(cn1)N2CCCC2=O	**2**{*587* }	COc1noc(C)c1C=O	**3**{*38* }	[C-]#[N+]c1ccc2OCOc2c1	42.0	35
1595	**4**{*371* ,*486* ,*12* }	CN1C(Cl)=NC(Cl)=C1C1=C(NCC2CCOC2)N2C=CC=C(OCCCC#N)C2=N1 |c:3,6,9,20,31,t:22|	Z8873684601	**1**{*371* }	Nc1ncccc1OCCCC#N	**2**{*486* }	Cn1c(Cl)nc(Cl)c1C=O	**3**{*12* }	[C-]#[N+]CC1CCOC1	42.1	35

<table>
<tr><td>1596</td><td>4{185,738,29}</td><td>CCOC(=O)CCNC1=C(N=C2C=CC(=C(C)N12)S(C)(=O)=O)C1=CC(Cl)=CN=C1 |c:12,27,29,t:8,10,14,24|</td><td>Z8855739080</td><td>1{185}</td><td>Cc1nc(N)ccc1S(=O)(=O)C</td><td>2{738}</td><td>Clc1cncc(C=O)c1</td><td>3{29}</td><td>CCOC(=O)CC[N+]#[C-]</td><td>40.9</td><td>35</td></tr>
<tr><td>1597</td><td>4{47,116,26}</td><td>NS(=O)(=O)C1=CC2=NC(=C(NCC3=CC=C(F)C=C3)N2C=C1)C1=CN(CCC=C)N=N1 |c:17,22,32,t:4,6,8,12,14,25|</td><td>Z8849597831</td><td>1{47}</td><td>Cl.Nc1cc(ccn1)S(=O)(=O)N</td><td>2{116}</td><td>C=CCCn1cc(C=O)nn1</td><td>3{26}</td><td>Fc1ccc(C[N+]#[C-])cc1</td><td>41.3</td><td>35</td></tr>
<tr><td>1598</td><td>4{129,550,29}</td><td>CCOC(=O)CCNC1=C(N=C2C=CC=C(SC)N12)C1=NC(Br)=NN1C |c:12,24,t:8,10,14,21|</td><td>Z8873684879</td><td>1{129}</td><td>CSc1cccc(N)n1</td><td>2{550}</td><td>Cn1nc(Br)nc1C=O</td><td>3{29}</td><td>CCOC(=O)CC[N+]#[C-]</td><td>41.0</td><td>35</td></tr>
<tr><td>1599</td><td>4{206,344,14}</td><td>COCCNC1=C(N=C2N1C=CC(I)=C2F)C1=C(C)C=NN1COC |c:7,11,14,18,21,t:5|</td><td>Z8873684648</td><td>1{206}</td><td>Nc1nccc(I)c1F</td><td>2{344}</td><td>COCn1ncc(C)c1C=O</td><td>3{14}</td><td>COCC[N+]#[C-]</td><td>42.9</td><td>35</td></tr>
<tr><td>1600</td><td>4{41,161,13}</td><td>CCN1C=NC=C1C1=C(NCC2=CC=C(OC)C=C2)N2C=C(C)N=CC2=N1 |c:3,5,8,18,25,28,t:12,14,22|</td><td>Z8855739127</td><td>1{41}</td><td>Cc1cnc(N)cn1</td><td>2{161}</td><td>CCn1cncc1C=O</td><td>3{13}</td><td>COc1ccc(C[N+]#[C-])cc1</td><td>33.8</td><td>35</td></tr>
<tr><td>1601</td><td>4{359,689,15}</td><td>CCNC1=C(N=C2C=CC(OC(F)F)=CN12)C1=CN(CCOC(C)=O)N=C1 |c:7,13,27,t:3,5,18|</td><td>Z8873685423</td><td>1{359}</td><td>Nc1ccc(OC(F)F)cn1</td><td>2{689}</td><td>CC(=O)OCCn1cc(C=O)cn1</td><td>3{15}</td><td>CC[N+]#[C-]</td><td>35.3</td><td>34</td></tr>
<tr><td>1602</td><td>4{119,454,61}</td><td>COC(C)CC1=C(NCCCNC(=O)OC(C)(C)C)N2C=C(C=CC2=N1)C#N |c:5,20,22,25|</td><td>Z8878918653</td><td>1{119}</td><td>Nc1ccc(C#N)cn1</td><td>2{454}</td><td>COC(C)CC=O</td><td>3{61}</td><td>CC(C)(C)OC(=O)NCCC[N+]#[C-]</td><td>35.6</td><td>34</td></tr>
<tr><td>1603</td><td>4{109,357,41}</td><td>CCC1=C(C(C)=NO1)C1=C(NC2=CC=C(OC)C=C2)N2C(C=CC=C2S(N)(=O)=O)=N1 |c:5,9,18,23,25,31,t:2,12,14|</td><td>Z8854581176</td><td>1{109}</td><td>Cl.Nc1cccc(n1)S(=O)(=O)N</td><td>2{357}</td><td>CCc1onc(C)c1C=O</td><td>3{41}</td><td>COc1ccc([N+]#[C-])cc1</td><td>39.0</td><td>34</td></tr>
<tr><td>1604</td><td>4{73,739,20}</td><td>COC1=CC(C)=C(NC2=C(N=C3C=CC4=NC=CN4N23)C2=CC3=C(C=NN3C)C=C2)C=C1 |c:12,16,27,32,35,t:2,5,8,10,14,23,25|</td><td>Z8855619518</td><td>1{73}</td><td>Nc1ccc2nccn2n1</td><td>2{739}</td><td>Cn1ncc2ccc(C=O)cc12</td><td>3{20}</td><td>COc1ccc([N+]#[C-])c(C)c1</td><td>38.6</td><td>34</td></tr>
<tr><td>1605</td><td>4{286,740,23}</td><td>COC(=O)CCC(NC1=C(N=C2C=CC(=CN12)C1=NC=CC=C1)C1=CN(N=C1)C(F)F)C(=O)OC |c:12,14,21,23,29,t:8,10,19,26|</td><td>Z8829498781</td><td>1{286}</td><td>Cl.Cl.Nc1ccc(cn1)c2ccccn2</td><td>2{740}</td><td>FC(F)n1cc(C=O)cn1</td><td>3{23}</td><td>COC(=O)CCC([N+]#[C-])C(=O)OC</td><td>44.1</td><td>34</td></tr>
<tr><td>1606</td><td>4{287,603,10}</td><td>COC(=O)C1=CC(=NS1)C1=C(NC2CCOC2)N2C=CC(Cl)=CC2=N1 |c:6,10,20,23,26,t:4|</td><td>Z8873685424</td><td>1{287}</td><td>Nc1cc(Cl)ccn1</td><td>2{603}</td><td>COC(=O)c1cc(C=O)ns1</td><td>3{10}</td><td>[C-]#[N+]C1CCOC1</td><td>34.3</td><td>34</td></tr>
<tr><td>1607</td><td>4{36,257,9}</td><td>CNC1=C(N=C2C=CC(Br)=CN12)C1CC(C1)NC(=O)OC(C)(C)C |c:6,9,t:2,4|</td><td>Z8873685342</td><td>1{36}</td><td>Nc1ccc(Br)cn1</td><td>2{257}</td><td>CC(C)(C)OC(=O)NC1CC(C1)C=O</td><td>3{9}</td><td>C[N+]#[C-]</td><td>35.7</td><td>33</td></tr>
</table>

<table>
<tr><td>1608</td><td>4{465,741,49}</td><td>FC1(F)CC(C1)NC1=C(N=C2C=CC(=CN12)S(=O)(=O)NC1CC1)C1=CC=C(C=C1)N1C=NC=N1 |c:12,14,29,31,35,37,t:8,10,27|</td><td>Z8829498651</td><td>1{465}</td><td>Nc1ccc(cn1)S(=O)(=O)NC2CC2</td><td>2{741}</td><td>O=Cc1ccc(cc1)n2cncn2</td><td>3{49}</td><td>FC1(F)CC(C1)[N+]#[C-]</td><td>43.8</td><td>33</td></tr>
<tr><td>1609</td><td>4{146,328,24}</td><td>CCC1=NNC=C1C1=C(NCC2=CC(OC)=CC=C2)N2C=C(C=CC2=N1)S(C)(=O)=O |c:5,8,16,18,22,24,27,t:2,12|</td><td>Z8878918638</td><td>1{146}</td><td>CS(=O)(=O)c1ccc(N)nc1</td><td>2{328}</td><td>Cl.CCc1n[nH]cc1C=O</td><td>3{24}</td><td>COc1cccc(C[N+]#[C-])c1</td><td>38.3</td><td>33</td></tr>
<tr><td>1610</td><td>4{447,135,37}</td><td>COC(=O)C1CC(C1)NC1=C(N=C2C=CC(=CN12)N(C)C)C1=C(C)N(C)N=C1C |c:14,16,24,29,t:10,12|</td><td>Z8873684739</td><td>1{447}</td><td>CN(C)c1ccc(N)nc1</td><td>2{135}</td><td>Cc1nn(C)c(C)c1C=O</td><td>3{37}</td><td>COC(=O)C1CC(C1)[N+]#[C-]</td><td>35.6</td><td>33</td></tr>
<tr><td>1611</td><td>4{166,626,15}</td><td>CCNC1=C(N=C2C=C(CSCCO)C=CN12)C1=CC2=C(CCN(C2)C(C)=O)S1 |c:14,t:3,5,7,19,21|</td><td>Z8873684916</td><td>1{166}</td><td>Nc1cc(CSCCO)ccn1</td><td>2{626}</td><td>CC(=O)N1CCc2sc(C=O)cc2C1</td><td>3{15}</td><td>CC[N+]#[C-]</td><td>38.7</td><td>33</td></tr>
<tr><td>1612</td><td>4{460,369,21}</td><td>CC1=NOC=C1C1=C(NCC2CC2)N2C=CC=C(C)C2=N1 |c:4,7,16,22,t:1,18|</td><td>Z8855619657</td><td>1{460}</td><td>Cc1cccnc1N</td><td>2{369}</td><td>Cc1nocc1C=O</td><td>3{21}</td><td>[C-]#[N+]CC1CC1</td><td>25.4</td><td>33</td></tr>
<tr><td>1613</td><td>4{48,742,22}</td><td>CS(=O)(=O)C1=CC2=NC(=C(NC3COC3)N2C=C1)C1=C(F)C(Br)=CC=C1F |c:18,21,25,27,t:4,6,8|</td><td>Z8873684860</td><td>1{48}</td><td>CS(=O)(=O)c1ccnc(N)c1</td><td>2{742}</td><td>Fc1ccc(Br)c(F)c1C=O</td><td>3{22}</td><td>[C-]#[N+]C1COC1</td><td>41.1</td><td>33</td></tr>
<tr><td>1614</td><td>4{41,672,13}</td><td>COC1=CC=C(CNC2=C(N=C3C=NC(C)=CN23)C2=C(C)SC=N2)C=C1 |c:12,15,20,24,27,t:2,4,8,10|</td><td>Z8878918776</td><td>1{41}</td><td>Cc1cnc(N)cn1</td><td>2{672}</td><td>Cc1scnc1C=O</td><td>3{13}</td><td>COc1ccc(C[N+]#[C-])cc1</td><td>32.7</td><td>33</td></tr>
<tr><td>1615</td><td>4{49,29,13}</td><td>CCN1C=CC(=N1)C1=C(NCC2=CC=C(OC)C=C2)N2C(C=CC=C2CO)=N1 |c:3,5,8,18,23,25,29,t:12,14|</td><td>Z8878918879</td><td>1{49}</td><td>Nc1cccc(CO)n1</td><td>2{29}</td><td>CCn1ccc(C=O)n1</td><td>3{13}</td><td>COc1ccc(C[N+]#[C-])cc1</td><td>33.3</td><td>33</td></tr>
<tr><td>1616</td><td>4{48,115,35}</td><td>COC1=NN(C)C(=C1)C1=C(NCCCSC)N2C=CC(=CC2=N1)S(C)(=O)=O |c:6,9,18,20,23,t:2|</td><td>Z8873685307</td><td>1{48}</td><td>CS(=O)(=O)c1ccnc(N)c1</td><td>2{115}</td><td>COc1cc(C=O)n(C)n1</td><td>3{35}</td><td>CSCCC[N+]#[C-]</td><td>36.1</td><td>33</td></tr>
<tr><td>1617</td><td>4{62,49,6}</td><td>CCOC(=O)CCCNC1=C(N=C2C=C(CO)C=CN12)C1=CNN=C1C |c:17,25,t:9,11,13,22|</td><td>Z8873684869</td><td>1{62}</td><td>Nc1cc(CO)ccn1</td><td>2{49}</td><td>Cc1n[nH]cc1C=O</td><td>3{6}</td><td>CCOC(=O)CCC[N+]#[C-]</td><td>31.5</td><td>33</td></tr>
<tr><td>1618</td><td>4{358,87,15}</td><td>CCNC1=C(N=C2C=C(C=CN12)C(C)(C)O)[C@@H]1C[C@H]1C(=O)OC(C)(C)C |&1:16,18,r,c:7,9,t:3,5|</td><td>Z8878918449</td><td>1{358}</td><td>CC(C)(O)c1ccnc(N)c1</td><td>2{87}</td><td>CC(C)(C)OC(=O)[C@@H]1C[C@H]1C=O |&1:7,9,r|</td><td>3{15}</td><td>CC[N+]#[C-]</td><td>31.5</td><td>32</td></tr>
<tr><td>1619</td><td>4{166,60,32}</td><td>CC(C1CCOC1)C1=C(NC2CCC2)N2C=CC(CSCCO)=CC2=N1 |c:8,17,24,27|</td><td>Z8878918678</td><td>1{166}</td><td>Nc1cc(CSCCO)ccn1</td><td>2{60}</td><td>CC(C=O)C1CCOC1</td><td>3{32}</td><td>[C-]#[N+]C1CCC1</td><td>32.9</td><td>32</td></tr>
</table>

1620	**4**{*125*,*92*,*18*}	CC1=NOC(=C1)C1=C(NC(C)(C)C)N2C=C(CCO)C=CC2=N1 |c:4,7,20,23,t:1,15|	Z8873684676	**1**{*125*}	Nc1ccc(CCO)cn1	**2**{*92*}	Cc1cc(C=O)on1	**3**{*18*}	CC(C)(C)[N+]#[C-]	27.5	32
1621	**4**{*109*,*743*,*34*}	CCC1=NC=CC(=C1)C1=C(NCC2CCCO2)N2C(C=CC=C2S(N)(=O)=O)=N1 |c:4,6,9,21,23,29,t:2|	Z8873684764	**1**{*109*}	Cl.Nc1cccc(n1)S(=O)(=O)N	**2**{*743*}	CCc1cc(C=O)ccn1	**3**{*34*}	[C-]#[N+]CC1CCCO1	35.0	32
1622	**4**{*32*,*744*,*31*}	CSCCNC1=C(N=C2C=C(C=CN12)P(C)(C)=O)C1=C(F)C=CC(=C1)C#C |c:9,11,20,23,25,t:5,7|	Z8873684898	**1**{*32*}	CP(=O)(C)c1ccnc(N)c1	**2**{*744*}	Fc1ccc(C#C)cc1C=O	**3**{*31*}	CSCC[N+]#[C-]	35.0	32
1623	**4**{*153*,*745*,*15*}	CCNC1=C(N=C2C=CC=C(N12)C(C)(C)O)C1=C(C)C=NC=C1 |c:7,9,18,21,23,t:3,5|	Z8878918516	**1**{*153*}	CC(C)(O)c1cccc(N)n1	**2**{*745*}	Cc1cnccc1C=O	**3**{*15*}	CC[N+]#[C-]	27.0	32
1624	**4**{*466*,*294*,*5*}	COCCCNC1=C(N=C2C=CC(OCCC(C)C)=NN12)C1=C(OC)C(=O)C=CO1 |c:10,18,23,29,t:6,8|	Z8878918834	**1**{*466*}	CC(C)CCOc1ccc(N)nn1	**2**{*294*}	COc1c(C=O)occc1=O	**3**{*5*}	COCCC[N+]#[C-]	36.1	32
1625	**4**{*186*,*746*,*8*}	COC(=O)C(CC1=CC=CC=C1)NC1=C(N=C2C=C(C=CN12)S(=O)(=O)N(C)C)C1=C(C)C=CN=C1 |c:8,10,18,20,31,34,36,t:6,14,16|	Z8873684706	**1**{*186*}	Cl.CN(C)S(=O)(=O)c1ccnc(N)c1	**2**{*746*}	Cc1ccncc1C=O	**3**{*8*}	COC(=O)C(Cc1ccccc1)[N+]#[C-]	42.8	32
1626	**4**{*438*,*747*,*10*}	CCOC(=O)C1=COC(=N1)C1=C(NC2CCOC2)N2C(C=CC(Br)=C2CC)=N1 |c:8,11,22,25,29,t:5|	Z8855619574	**1**{*438*}	CCc1nc(N)ccc1Br	**2**{*747*}	CCOC(=O)c1coc(C=O)n1	**3**{*10*}	[C-]#[N+]C1CCOC1	39.0	32
1627	**4**{*46*,*19*,*9*}	CNC1=C(N=C2C=CC=CN12)C1=C(OC)C=NN1C |c:6,8,13,17,t:2,4|	Z8873684646	**1**{*46*}	Nc1ccccn1	**2**{*19*}	COc1cnn(C)c1C=O	**3**{*9*}	C[N+]#[C-]	22.2	32
1628	**4**{*105*,*480*,*50*}	NC(=O)C1=CC=CC2=NC(=C(NC3=CC4=C(OCCO4)C=C3)N12)C1=CC2=C(CCOC2)N=C1 |c:5,21,36,t:3,7,9,12,14,27,29|	Z8829498452	**1**{*105*}	NC(=O)c1cccc(N)n1	**2**{*480*}	O=Cc1cnc2CCOCc2c1	**3**{*50*}	[C-]#[N+]c1ccc2OCCOc2c1	38.1	32
1629	**4**{*171*,*318*,*15*}	CCNC1=C(N=C2C=C(C=CN12)N1C=CN=C1)C1CC11CC1(F)F |c:7,9,15,17,t:3,5|	Z8878918910	**1**{*171*}	Nc1cc(ccn1)n2ccnc2	**2**{*318*}	FC1(F)CC21CC2C=O	**3**{*15*}	CC[N+]#[C-]	28.3	32
1630	**4**{*353*,*748*,*32*}	CN1N=C(C=C1C1=C(NC2CCC2)N2C=C(NS(C)(=O)=O)C=CC2=N1)C(F)(F)F |c:2,4,7,23,26,t:16|	Z8873685348	**1**{*353*}	Cl.CS(=O)(=O)Nc1ccc(N)nc1	**2**{*748*}	Cn1nc(cc1C=O)C(F)(F)F	**3**{*32*}	[C-]#[N+]C1CCC1	36.5	32
1631	**4**{*92*,*749*,*14*}	COCCNC1=C(N=C2C=C(C=CN12)C(N)=O)[C@@H]1C[C@H]1C1=CC=CC=C1 |&1:17,19,r,c:9,11,25,27,t:5,7,23|	Z8854581153	**1**{*92*}	NC(=O)c1ccnc(N)c1	**2**{*749*}	O=C[C@@H]1C[C@H]1c2ccccc2 |&1:2,4,r|	**3**{*14*}	COCC[N+]#[C-]	29.8	32
1632	**4**{*83*,*203*,*26*}	CP(C)(=O)C1=CN2C(C=C1)=NC(C1=NN(CC(F)F)C=C1)=C2NCC1=CC=C(F)C=C1 |c:8,10,20,22,32,t:4,13,27,29|	Z8846491831	**1**{*83*}	CP(=O)(C)c1ccc(N)nc1	**2**{*203*}	FC(F)Cn1ccc(C=O)n1	**3**{*26*}	Fc1ccc(C[N+]#[C-])cc1	37.8	31

1633	**4**{*187,662,12*}	CP(C)(=O)C1=CC(=CC=C1)C1=C(NCC2CCOC2)N2C=C(Cl)C(=CC2=N1)C#N |c:6,8,11,25,28,t:4,22|	Z8873684757	**1**{*187*}	Nc1cc(C#N)c(Cl)cn1	**2**{*662*}	CP(=O)(C)c1cccc(C=O)c1	**3**{*12*}	[C-]#[N+]CC1COC1	36.1	31
1634	**4**{*377,665,46*}	CN1N=NC(=C1C)C1=C(NCC(C)=C)N2C=CC=C(OCC(F)F)C2=N1 |c:2,4,8,16,26,t:18|	Z8878918373	**1**{*377*}	Nc1ncccc1OCC(F)F	**2**{*665*}	Cc1c(C=O)nnn1C	**3**{*46*}	CC(=C)C[N+]#[C-]	30.5	31
1635	**4**{*84,645,31*}	CSCCNC1=C(N=C2N1C=CN=C2C(C)C)C1=CNN=N1 |c:7,11,13,22,t:5,19|	Z8873684861	**1**{*84*}	CC(C)c1nccnc1N	**2**{*645*}	O=Cc1c[nH]nn1	**3**{*31*}	CSCC[N+]#[C-]	26.7	31
1636	**4**{*41,262,9*}	CCC1=NN(C)C(=C1Cl)C1=C(NC)N2C=C(C)N=CC2=N1 |c:6,10,18,21,t:2,15|	Z8878918869	**1**{*41*}	Cc1cnc(N)cn1	**2**{*262*}	CCc1nn(C)c(C=O)c1Cl	**3**{*9*}	C[N+]#[C-]	25.5	31
1637	**4**{*92,101,7*}	NC(=O)C1=CC2=NC(=C(NC3CCOCC3)N2C=C1)C1=C(CO)C=CS1 |c:19,22,26,t:3,5,7|	Z8829498636	**1**{*92*}	NC(=O)c1ccnc(N)c1	**2**{*101*}	OCc1ccsc1C=O	**3**{*7*}	[C-]#[N+]C1CCOCC1	31.2	31
1638	**4**{*73,750,13*}	CCOC1=CC(=CC=C1)C1=C(NCC2=CC=C(OC)C=C2)N2N3C=CN=C3C=CC2=N1 |c:5,7,10,20,25,27,30,33,t:3,14,16|	Z8878918873	**1**{*73*}	Nc1ccc2nccn2n1	**2**{*750*}	CCOc1cccc(C=O)c1	**3**{*13*}	COc1ccc(C[N+]#[C-])cc1	34.6	31
1639	**4**{*467,751,47*}	COC1=CC2=C(C=C1)C=C(CO2)C1=C(NC(C)C)N2C=C(C=CC2=N1)C(=O)N1CCOCC1 |c:4,6,9,14,21,23,26,t:2|	Z8873685299	**1**{*467*}	Nc1ccc(cn1)C(=O)N2CCOCC2	**2**{*751*}	COc1ccc2C=C(COc2c1)C=O	**3**{*47*}	CC(C)[N+]#[C-]	37.4	31
1640	**4**{*352,752,49*}	CN1CCN(CC1=O)C1=CN2C(C=C1)=NC(=C2NC1CC(F)(F)C1)C1=CC(=CC=C1)C#N |c:13,15,17,30,32,t:9,28|	Z8873684814	**1**{*352*}	CN1CCN(CC1=O)c2ccc(N)nc2	**2**{*752*}	O=Cc1cccc(C#N)c1	**3**{*49*}	FC1(F)CC(C1)[N+]#[C-]	36.3	31
1641	**4**{*69,753,10*}	CNC(=O)C1=CC2=NC([C@@H]3CCC[C@H](C)N3C(=O)OC(C)(C)C)=C(NC3CCOC3)N2C=C1 |&1:9,13,r,c:34,t:4,6,23|	Z8878918622	**1**{*69*}	CNC(=O)c1ccnc(N)c1	**2**{*753*}	C[C@@H]1CCC[C@H](C=O)N1C(=O)OC(C)(C)C |&1:1,5,r|	**3**{*10*}	[C-]#[N+]C1CCOC1	38.0	31
1642	**4**{*28,115,7*}	COC1=NN(C)C(=C1)C1=C(NC2CCOCC2)N2C=CC(=CC2=N1)C1=NOC=N1 |c:6,9,20,22,25,31,t:2,28|	Z8855739208	**1**{*28*}	Cl.Nc1cc(ccn1)c2ncon2	**2**{*115*}	COc1cc(C=O)n(C)n1	**3**{*7*}	[C-]#[N+]C1CCOCC1	32.8	31
1643	**4**{*83,754,18*}	CC(C)(C)NC1=C(N=C2C=CC(=CN12)P(C)(C)=O)C1CC2CC2C1 |c:9,11,t:5,7|	Z8878918486	**1**{*83*}	CP(=O)(C)c1ccc(N)nc1	**2**{*754*}	O=CC1CC2CC2C1	**3**{*18*}	CC(C)(C)[N+]#[C-]	28.7	31
1644	**4**{*183,392,28*}	CCCNC1=C(N=C2N1C(C)=CC=C2OC)C1CCOCC1 |c:6,11,13,t:4|	Z8849597822	**1**{*183*}	COc1ccc(C)nc1N	**2**{*392*}	O=CC1CCOCC1	**3**{*28*}	CCC[N+]#[C-]	25.2	31

<table>
<tr><td>1645</td><td>4{350 ,354 ,5 }</td><td>COCCCNC1=C(N=C2C=NC(=CN12)C1=CC=CC=C1)C1=C(C=CC=C1)P(C)(C)=O |c:10,12,19,21,26,28,t:6,8,17,24|</td><td>Z8878918587</td><td>1{350 }</td><td>Nc1cnc(cn1)c2ccccc2</td><td>2{354 }</td><td>CP(=O)(C)c1ccccc1C=O</td><td>3{5 }</td><td>COCCC[N+]#[C-]</td><td>36.1</td><td>31</td></tr>
<tr><td>1646</td><td>4{154 ,468 ,47 }</td><td>CSC(C)CC1=C(NC(C)C)N2C=CC(=CC2=N1)[N+]([O-])=O |c:5,12,14,17|</td><td>Z8854581155</td><td>1{154 }</td><td>Nc1cc(ccn1)[N+](=O)[O-]</td><td>2{468 }</td><td>CSC(C)CC=O</td><td>3{47 }</td><td>CC(C)[N+]#[C-]</td><td>25.6</td><td>31</td></tr>
<tr><td>1647</td><td>4{85 ,755 ,15 }</td><td>CCNC1=C(N=C2C=C(C=C(C)N12)C#N)C1=CC2=C(C=C1)N(C)C(=O)O2 |c:7,19,21,t:3,5,9,17|</td><td>Z8849597804</td><td>1{85 }</td><td>Cc1cc(C#N)cc(N)n1</td><td>2{755 }</td><td>Cn1c(=O)oc2cc(C=O)ccc12</td><td>3{15 }</td><td>CC[N+]#[C-]</td><td>28.8</td><td>31</td></tr>
<tr><td>1648</td><td>4{79 ,137 ,13 }</td><td>COC1=CC=C(CNC2=C(N=C3C=NC=C(C)N23)C2=C(C)ON=C2C)C=C1 |c:12,20,24,28,t:2,4,8,10,14|</td><td>Z8854581163</td><td>1{79 }</td><td>Cc1cncc(N)n1</td><td>2{137 }</td><td>Cc1noc(C)c1C=O</td><td>3{13 }</td><td>COc1ccc(C[N+]#[C-])cc1</td><td>30.0</td><td>31</td></tr>
<tr><td>1649</td><td>4{72 ,536 ,46 }</td><td>COC1=NC(Br)=CN2C(NCC(C)=C)=C(N=C12)C1=CN=C(O1)C1CCOCC1 |c:5,13,21,t:2,15,19|</td><td>Z8835022870</td><td>1{72 }</td><td>COc1nc(Br)cnc1N</td><td>2{536 }</td><td>O=Cc1cnc(o1)C2CCOCC2</td><td>3{46 }</td><td>CC(=C)C[N+]#[C-]</td><td>36.9</td><td>30</td></tr>
<tr><td>1650</td><td>4{186 ,756 ,69 }</td><td>COC1=C(NC2=C(N=C3C=C(C=CN23)S(=O)(=O)N(C)C)C2=CC(=CO2)S(C)(=O)=O)C=CC=C1 |c:2,9,11,24,32,34,t:5,7,22|</td><td>Z8878918381</td><td>1{186 }</td><td>Cl.CN(C)S(=O)(=O)c1ccnc(N)c1</td><td>2{756 }</td><td>CS(=O)(=O)c1coc(C=O)c1</td><td>3{69 }</td><td>COc1ccccc1[N+]#[C-]</td><td>40.3</td><td>30</td></tr>
<tr><td>1651</td><td>4{41 ,757 ,13 }</td><td>CCCN1N=CN=C1C1=C(NCC2=CC=C(OC)C=C2)N2C=C(C)N=CC2=N1 |c:4,6,9,19,26,29,t:13,15,23|</td><td>Z8878918313</td><td>1{41 }</td><td>Cc1cnc(N)cn1</td><td>2{757 }</td><td>CCCn1ncnc1C=O</td><td>3{13 }</td><td>COc1ccc(C[N+]#[C-])cc1</td><td>31.0</td><td>30</td></tr>
<tr><td>1652</td><td>4{109 ,539 ,34 }</td><td>NS(=O)(=O)C1=CC=CC2=NC([C@@H]3CCCC[C@H]3C(F)(F)F)=C(NCC3CCCO3)N12 |&1:11,16,r,c:6,t:4,8,21|</td><td>Z8835022889</td><td>1{109 }</td><td>Cl.Nc1cccc(n1)S(=O)(=O)N</td><td>2{539 }</td><td>FC(F)(F)[C@@H]1CCCC[C@H]1C=O |&1:4,9,r|</td><td>3{34 }</td><td>[C-]#[N+]CC1CCCO1</td><td>36.6</td><td>30</td></tr>
<tr><td>1653</td><td>4{68 ,354 ,59 }</td><td>CP(C)(=O)C1=C(C=CC=C1)C1=C(NCCC2=CC(F)=CC=C2)N2C=CC(=CC2=N1)C#C |c:6,8,11,19,21,25,27,30,t:4,16|</td><td>Z8873684914</td><td>1{68 }</td><td>Nc1cc(C#C)ccn1</td><td>2{354 }</td><td>CP(=O)(C)c1ccccc1C=O</td><td>3{59 }</td><td>Fc1cccc(CC[N+]#[C-])c1</td><td>35.3</td><td>30</td></tr>
<tr><td>1654</td><td>4{46 ,396 ,9 }</td><td>CNC1=C(N=C2C=CC=CN12)C1=CN=C2COCCN12 |c:6,8,t:2,4,13,15|</td><td>Z8849597823</td><td>1{46 }</td><td>Nc1ccccn1</td><td>2{396 }</td><td>O=Cc1cnc2COCCn12</td><td>3{9 }</td><td>C[N+]#[C-]</td><td>22.1</td><td>30</td></tr>
<tr><td>1655</td><td>4{377 ,413 ,10 }</td><td>FC(F)COC1=CC=CN2C(NC3CCOC3)=C(N=C12)C1=CN=C(S1)C1=CC=NC=C1 |c:7,17,25,31,33,t:5,19,23,29|</td><td>Z8873684699</td><td>1{377 }</td><td>Nc1ncccc1OCC(F)F</td><td>2{413 }</td><td>O=Cc1cnc(s1)c2ccncc2</td><td>3{10 }</td><td>[C-]#[N+]C1CCOC1</td><td>36.2</td><td>30</td></tr>
<tr><td>1656</td><td>4{353 ,626 ,35 }</td><td>CSCCCNC1=C(N=C2C=CC(NS(C)(=O)=O)=CN12)C1=CC2=C(CCN(C2)C(C)=O)S1 |c:10,17,t:6,8,22,24|</td><td>Z8873685326</td><td>1{353 }</td><td>Cl.CS(=O)(=O)Nc1ccc(N)nc1</td><td>2{626 }</td><td>CC(=O)N1CCc2sc(C=O)cc2C1</td><td>3{35 }</td><td>CSCCC[N+]#[C-]</td><td>40.1</td><td>30</td></tr>
</table>

1657	**4**{*83*,*595*,*7*}	CN1C=C2C=C(C=CC2=N1)C1=C(NC2CCOC C2)N2C=C(C=CC2=N1)P(C)(C)=O |c:4,6,9,12,23,25,28,t:2|	Z8849597826	**1**{*83*}	CP(=O)(C)c 1ccc(N)nc1	**2**{*595*}	Cn1cc2cc(C= O)ccc2n1	**3**{*7*}	[C-]#[N+]C1CC OCC1	34.4	30
1658	**4**{*433*,*50*,*18*}	CC1=C(C=CC2=NC([C@@H]3C[C@H]3C3O CCO3)=C(NC(C)(C)C)N12)C#C |&1:8,10,r,c:3,t:1,5,17|	Z8873684729	**1**{*433*}	Cc1nc(N)cc c1C#C	**2**{*50*}	O=C[C@@H]1C[C@H]1 C2OCCO2 |&1:2,4,r|	**3**{*18*}	CC(C)(C)[N +]#[C-]	27.5	30
1659	**4**{*32*,*758*,*26*}	CP(C)(=O)C1=CC2=NC(=C(NCC3=CC=C(F) C=C3)N2C=C1)C1=C(F)C=C(C=C1)C(F)F |c:17,22,25,28,30,t:4,6,8,12,14|	Z8873685331	**1**{*32*}	CP(=O)(C)c 1ccnc(N)c1	**2**{*758*}	FC(F)c1ccc(C=O)c(F)c1	**3**{*26*}	Fc1ccc(C[N+]#[C-])cc1	37.4	30
1660	**4**{*68*,*673*,*23*}	COC(=O)CCC(NC1=C(N=C2C=C(C=CN12)C #C)C1=NC=CC(Cl)=C1)C(=O)OC |c:12,14,23,26,t:8,10,21|	Z8878918805	**1**{*68*}	Nc1cc(C#C) ccn1	**2**{*673*}	Clc1ccnc(C= O)c1	**3**{*23*}	COC(=O)CC C([N+]#[C-])C(=O)OC	34.4	30
1661	**4**{*171*,*408*,*15*}	CCNC1=C(CCN2CCOC2=O)N=C2C=C(C=C N12)N1C=CN=C1 |c:3,16,18,24,26,t:14|	Z8846491754	**1**{*171*}	Nc1cc(ccn1)n2ccnc2	**2**{*408*}	O=CCCN1C COC1=O	**3**{*15*}	CC[N+]#[C-]	27.4	30
1662	**4**{*428*,*665*,*24*}	COC1=CC(CNC2=C(N=C3C=NC(Br)=CN23) C2=C(C)N(C)N=N2)=CC=C1 |c:11,14,19,24,26,28,t:2,7,9|	Z8855739182	**1**{*428*}	Nc1cnc(Br) cn1	**2**{*665*}	Cc1c(C=O)n nn1C	**3**{*24*}	COc1cccc(C[N+]#[C-])c1	34.5	30
1663	**4**{*234*,*519*,*10*}	FC(F)C1=NC=C(S1)C1=C(NC2CCOC2)N2C= CN=C(OC3CCC3)C2=N1 |c:5,9,19,30,t:3,21|	Z8878918457	**1**{*234*}	Nc1nccnc1 OC2CCC2	**2**{*519*}	FC(F)c1ncc(C=O)s1	**3**{*10*}	[C-]#[N+]C1CC OC1	32.7	30
1664	**4**{*54*,*216*,*53*}	COC(=O)C1=NC=C(S1)C1=C(NCCOCC2=CC =CC=C2)N2C=C(C=CC2=N1)C(=O)N(C)C |c:6,10,19,21,25,27,30,t:4,17|	Z8878918850	**1**{*54*}	Cl.CN(C)C(=O)c1ccc(N)nc1	**2**{*216*}	COC(=O)c1n cc(C=O)s1	**3**{*53*}	[C-]#[N+]CCOC c1ccccc1	38.5	30
1665	**4**{*83*,*759*,*18*}	COCC1=CC=C(S1)C1=C(NC(C)(C)C)N2C=C(C=CC2=N1)P(C)(C)=O |c:5,9,17,19,22,t:3|	Z8846492332	**1**{*83*}	CP(=O)(C)c 1ccc(N)nc1	**2**{*759*}	COCc1ccc(C =O)s1	**3**{*18*}	CC(C)(C)[N +]#[C-]	31.2	30
1666	**4**{*197*,*407*,*67*}	CCC(C)NC1=C(N=C2C=CC(=CN12)S(=O)(= O)CC(C)C)C1=NN=C(CC)N1 |c:9,11,t:5,7,23,25|	Z8873685459	**1**{*197*}	CC(C)CS(= O)(=O)c1cc c(N)nc1	**2**{*407*}	CCc1nnc(C= O)[nH]1	**3**{*67*}	CCC(C)[N+] #[C-]	32.1	29
1667	**4**{*65*,*35*,*23*}	CCC1=CN=CC2=NC(=C(NC(CCC(=O)OC)C(=O)OC)N12)C1=NN(C)C(C)=C1Cl |c:4,30,t:2,6,8,25|	Z8878918830	**1**{*65*}	CCc1cncc(N)n1	**2**{*35*}	Cc1c(Cl)c(C =O)nn1C	**3**{*23*}	COC(=O)CC C([N+]#[C-])C(=O)OC	35.6	29
1668	**4**{*76*,*294*,*23*}	COC(=O)CCC(NC1=C(N=C2C=CC(C)=C(Cl) N12)C1=C(OC)C(=O)C=CO1)C(=O)OC |c:12,21,27,t:8,10,15|	Z8878918563	**1**{*76*}	Cc1ccc(N)n c1Cl	**2**{*294*}	COc1c(C=O) occc1=O	**3**{*23*}	COC(=O)CC C([N+]#[C-])C(=O)OC	36.7	29

1669	**4**{*83*,*760*,*31*}	CSCCNC1=C(N=C2C=CC(=CN12)P(C)(C)=O)C1=CC(F)=C(C=C1)C1CC1 |c:9,11,23,25,t:5,7,20|	Z8873685439	**1**{*83*}	CP(=O)(C)c1ccc(N)nc1	**2**{*760*}	Fc1cc(C=O)ccc1C2CC2	**3**{*31*}	CSCC[N+]#[C-]	32.9	29
1670	**4**{*186*,*386*,*23*}	COC(=O)CCC(NC1=C(N=C2C=C(C=CN12)S(=O)(=O)N(C)C)[C@H]1CC1(C)C)C(=O)OC |c:12,14,t:8,10|	Z8873685446	**1**{*186*}	Cl.CN(C)S(=O)(=O)c1ccnc(N)c1	**2**{*386*}	CC1(C)C[C@@H]1C=O	**3**{*23*}	COC(=O)CCC([N+]#[C-])C(=O)OC	36.5	29
1671	**4**{*32*,*761*,*7*}	CP(C)(=O)C1=CC2=NC(=C(NC3CCOCC3)N2C=C1)C1=C(Cl)C=C(C=C1)C#C |c:20,23,26,28,t:4,6,8|	Z8873684640	**1**{*32*}	CP(=O)(C)c1ccnc(N)c1	**2**{*761*}	Clc1cc(C#C)ccc1C=O	**3**{*7*}	[C-]#[N+]C1CCOCC1	33.3	29
1672	**4**{*83*,*760*,*18*}	CC(C)(C)NC1=C(N=C2C=CC(=CN12)P(C)(C)=O)C1=CC(F)=C(C=C1)C1CC1 |c:9,11,23,25,t:5,7,20|	Z8878918433	**1**{*83*}	CP(=O)(C)c1ccc(N)nc1	**2**{*760*}	Fc1cc(C=O)ccc1C2CC2	**3**{*18*}	CC(C)(C)[N+]#[C-]	31.1	29
1673	**4**{*431*,*762*,*10*}	FC1=CN2C(C=C1)=NC(C1CC1(F)F)=C2NC1CCOC1 |c:5,7,15,t:1|	Z8849597836	**1**{*431*}	Nc1ccc(F)cn1	**2**{*762*}	FC1(F)CC1C=O	**3**{*10*}	[C-]#[N+]C1CCOC1	23.1	29
1674	**4**{*28*,*373*,*18*}	C[C@@H]1[C@@H](C2=C(NC(C)(C)C)N3C=CC(=CC3=N2)C2=NOC=N2)C1(F)F |&1:1,2,r,c:3,11,13,16,22,t:19|	Z8846492415	**1**{*28*}	Cl.Nc1cc(ccn1)c2ncon2	**2**{*373*}	C[C@@H]1[C@@H](C=O)C1(F)F |&1:1,2,r|	**3**{*18*}	CC(C)(C)[N+]#[C-]	26.9	29
1675	**4**{*54*,*763*,*37*}	COC(=O)C1CC(C1)NC1=C(N=C2C=CC(=CN12)C(=O)N(C)C)C1=NNC2=C1C=C(C=C2)C(=O)OC |c:14,16,29,32,34,t:10,12,26|	Z8873684933	**1**{*54*}	Cl.CN(C)C(=O)c1ccc(N)nc1	**2**{*763*}	COC(=O)c1ccc2[nH]nc(C=O)c2c1	**3**{*37*}	COC(=O)C1CC(C1)[N+]#[C-]	38.0	29
1676	**4**{*73*,*764*,*20*}	COC1=CC(C)=C(NC2=C(N=C3C=CC4=NC=CN4N23)C2=C(OC)C(Cl)=CC=C2)C=C1 |c:12,16,23,28,30,33,t:2,5,8,10,14|	Z8878918364	**1**{*73*}	Nc1ccc2nccn2n1	**2**{*764*}	COc1c(Cl)cccc1C=O	**3**{*20*}	COc1ccc([N+]#[C-])c(C)c1	33.4	29
1677	**4**{*113*,*447*,*14*}	COCCNC1=C(N=C2N1C=CC=C2N1CCOCC1)C1=CC=C(C=C1)C#CC(C)(C)O |c:7,11,13,25,27,t:5,23|	Z8878918558	**1**{*113*}	Nc1ncccc1N2CCOCC2	**2**{*447*}	CC(C)(O)C#Cc1ccc(C=O)cc1	**3**{*14*}	COCC[N+]#[C-]	33.3	28
1678	**4**{*81*,*274*,*13*}	COC1=CC=C(CNC2=C(CC3(CCOCC3)C#N)N=C3C=NC=CN23)C=C1 |c:8,22,24,29,t:2,4,20|	Z8878918599	**1**{*81*}	Nc1cnccn1	**2**{*274*}	O=CCC1(CCOCC1)C#N	**3**{*13*}	COc1ccc(C[N+]#[C-])cc1	28.9	28
1679	**4**{*356*,*746*,*8*}	COC(=O)C(CC1=CC=CC=C1)NC1=C(N=C2C=CC(Cl)=C(N12)C(=O)N(C)C)C1=C(C)C=CN=C1 |c:8,10,18,21,31,34,36,t:6,14,16|	Z8878918444	**1**{*356*}	CN(C)C(=O)c1nc(N)ccc1Cl	**2**{*746*}	Cc1ccncc1C=O	**3**{*8*}	COC(=O)C(Cc1ccccc1)[N+]#[C-]	37.7	28

1680	**4**{*77*,*725*,*5*}	CCN1N=NC=C1C1=C(NCCCOC)N2C=C(C=CC2=N1)C1=CN=CC=C1 |c:3,5,8,17,19,22,27,29,t:25|	Z8878918529	**1**{*77*}	Nc1ccc(cn1)c2cccnc2	**2**{*725*}	CCn1nncc1C=O	**3**{*5*}	COCCC[N+]#[C-]	28.8	28
1681	**4**{*54*,*575*,*32*}	CN(C)C(=O)C1=CN2C(C=C1)=NC(CC(NC(=O)OC(C)(C)C)C(F)(F)F)=C2NC1CCC1 |c:9,11,27,t:5|	Z8873685300	**1**{*54*}	Cl.CN(C)C(=O)c1ccc(N)nc1	**2**{*575*}	CC(C)(C)OC(=O)NC(CC=O)C(F)(F)F	**3**{*32*}	[C-]#[N+]C1CCC1	35.8	28
1682	**4**{*199*,*563*,*34*}	CC(=O)NC1=NC=C(C=C1)C1=C(NCC2CCCO2)N2C=CC=C(C2=N1)C(C)(F)F |c:6,8,11,22,24,27,t:4|	Z8878918377	**1**{*199*}	CC(F)(F)c1cccnc1N	**2**{*563*}	CC(=O)Nc1ccc(C=O)cn1	**3**{*34*}	[C-]#[N+]CC1CCCO1	31.6	28
1683	**4**{*81*,*420*,*13*}	COC1=CC=C(CNC2=C(CC(C)(C)C#N)N=C3C=NC=CN23)C=C1 |c:8,18,20,25,t:2,4,16|	Z8878918554	**1**{*81*}	Nc1cnccn1	**2**{*420*}	CC(C)(CC=O)C#N	**3**{*13*}	COc1ccc(C[N+]#[C-])cc1	25.3	28
1684	**4**{*79*,*396*,*13*}	COC1=CC=C(CNC2=C(N=C3C=NC=C(C)N23)C2=CN=C3COCCN23)C=C1 |c:12,31,t:2,4,8,10,14,20,22|	Z8878918546	**1**{*79*}	Cc1cncc(N)n1	**2**{*396*}	O=Cc1cnc2COCCn12	**3**{*13*}	COc1ccc(C[N+]#[C-])cc1	29.4	28
1685	**4**{*105*,*654*,*66*}	CC(NC1=C(CCC2=CN=CO2)N=C2C=CC=C(N12)C(N)=O)C1=CC=CC=C1 |c:3,9,15,17,27,29,t:7,13,25|	Z8878918902	**1**{*105*}	NC(=O)c1cccc(N)n1	**2**{*654*}	O=CCCc1cnco1	**3**{*66*}	CC([N+]#[C-])c1ccccc1	28.1	28
1686	**4**{*154*,*552*,*49*}	[O-][N+](=O)C1=CC2=NC(=C(NC3CC(F)(F)C3)N2C=C1)C1=CN=C(C=C1)C#C |c:19,24,26,t:3,5,7,22|	Z8873685272	**1**{*154*}	Nc1cc(ccn1)[N+](=O)[O-]	**2**{*552*}	O=Cc1ccc(C#C)nc1	**3**{*49*}	FC1(F)CC(C1)[N+]#[C-]	27.7	28
1687	**4**{*50*,*744*,*7*}	CP(C)(=O)C1=CC=CC2=NC(=C(NC3CCOCC3)N12)C1=C(F)C=CC(=C1)C#C |c:6,23,26,28,t:4,8,10|	Z8846492306	**1**{*50*}	CP(=O)(C)c1cccc(N)n1	**2**{*744*}	Fc1ccc(C#C)cc1C=O	**3**{*7*}	[C-]#[N+]C1CCOCC1	30.8	28
1688	**4**{*468*,*756*,*69*}	COC1=C(NC2=C(N=C3C=CC=C(N23)C2=CN(C)N=C2)C2=CC(=CO2)S(C)(=O)=O)C=CC=C1 |c:2,9,11,20,25,33,35,t:5,7,16,23|	Z8878918317	**1**{*468*}	Cl.Cl.Cn1cc(cn1)c2cccc(N)n2	**2**{*756*}	CS(=O)(=O)c1coc(C=O)c1	**3**{*69*}	COc1ccccc1[N+]#[C-]	34.5	28
1689	**4**{*48*,*765*,*22*}	CS(=O)(=O)C1=CC2=NC(=C(NC3COC3)N2C=C1)C1=CN=C(OC2=CC=C(F)C=C2)C=C1 |c:18,31,34,t:4,6,8,21,23,26,28|	Z8873684798	**1**{*48*}	CS(=O)(=O)c1ccnc(N)c1	**2**{*765*}	Fc1ccc(Oc2ccc(C=O)cn2)cc1	**3**{*22*}	[C-]#[N+]C1COC1	33.7	27
1690	**4**{*95*,*61*,*37*}	COC(=O)C1CC(C1)NC1=C(N=C2C=CC(=CN12)C(F)F)C1=CC=C(C=C1)S(C)=O |c:14,16,26,28,t:10,12,24|	Z8849597796	**1**{*95*}	Nc1ccc(cn1)C(F)F	**2**{*61*}	CS(=O)c1ccc(C=O)cc1	**3**{*37*}	COC(=O)C1CC(C1)[N+]#[C-]	32.2	27

1691	**4**{*73 ,88 ,13* }	COC1=CC=C(CNC2=C(N=C3C=CC4=NC=CN4N23)C2=CC3=C(C=CC=N3)C=C2)C=C1 |c:12,16,27,29,32,35,t:2,4,8,10,14,23,25|	Z8829498653	**1**{*73* }	Nc1ccc2nccn2n1	**2**{*88* }	O=Cc1ccc2cccnc2c1	**3**{*13* }	COc1ccc(C[N+]#[C-])cc1	31.2	27
1692	**4**{*142 ,766 ,46* }	CC1CC1C1=C(NCC(C)=C)N2C=CC=C(OCCS(C)(=O)=O)C2=N1 |c:5,13,25,t:15|	Z8873684943	**1**{*142* }	CS(=O)(=O)CCOc1cccnc1N	**2**{*766* }	CC1CC1C=O	**3**{*46* }	CC(=C)C[N+]#[C-]	27.0	27
1693	**4**{*103 ,354 ,5* }	COCCCNC1=C(N=C2C=NC=C(CO)N12)C1=C(C=CC=C1)P(C)(C)=O |c:10,21,23,t:6,8,12,19|	Z8873685481	**1**{*103* }	Nc1cncc(CO)n1	**2**{*354* }	CP(=O)(C)c1ccccc1C=O	**3**{*5* }	COCCC[N+]#[C-]	28.8	27
1694	**4**{*464 ,438 ,5* }	COCCCNC1=C(N=C2C=CC(=CN12)N1CCN(C)CC1)C1=NC(C)=NC(=C1)C(F)F |c:10,12,28,30,t:6,8,25|	Z8878918570	**1**{*464* }	CN1CCN(CC1)c2ccc(N)nc2	**2**{*438* }	Cc1nc(C=O)cc(n1)C(F)F	**3**{*5* }	COCCC[N+]#[C-]	32.9	27
1695	**4**{*46 ,257 ,9* }	CNC1=C(N=C2C=CC=CN12)C1CC(C1)NC(=O)OC(C)(C)C |c:6,8,t:2,4|	Z8878918515	**1**{*46* }	Nc1ccccn1	**2**{*257* }	CC(C)(C)OC(=O)NC1CC(C1)C=O	**3**{*9* }	C[N+]#[C-]	23.3	27
1696	**4**{*123 ,149 ,15* }	CCNC1=C(N=C2C=C(OCCO)C=CN12)C1=C(OC)C(OC)=CC(Br)=C1 |c:13,18,24,27,t:3,5,7|	Z8878918634	**1**{*123* }	Cl.Nc1cc(OCCO)ccn1	**2**{*149* }	COc1cc(Br)cc(C=O)c1OC	**3**{*15* }	CC[N+]#[C-]	32.0	27
1697	**4**{*81 ,46 ,9* }	CNC1=C(CCCCCOC)N=C2C=NC=CN12 |c:2,13,15,t:11|	Z8873684589	**1**{*81* }	Nc1cnccn1	**2**{*46* }	COCCCCCC=O	**3**{*9* }	C[N+]#[C-]	18.2	27
1698	**4**{*434 ,92 ,31* }	CSCCNC1=C(N=C2C=CC(=CN12)C(C)(F)F)C1=CC(C)=NO1 |c:9,11,23,t:5,7,20|	Z8878918864	**1**{*434* }	CC(F)(F)c1ccc(N)nc1	**2**{*92* }	Cc1cc(C=O)on1	**3**{*31* }	CSCC[N+]#[C-]	25.8	27
1699	**4**{*81 ,25 ,13* }	COC1=CC=C(CNC2=C(N=C3C=NC=CN23)C2=CN=C(N=C2)C2CCOCC2)C=C1 |c:12,14,21,23,33,t:2,4,8,10,19|	Z8878918423	**1**{*81* }	Nc1cnccn1	**2**{*25* }	O=Cc1cnc(nc1)C2CCOCC2	**3**{*13* }	COc1ccc(C[N+]#[C-])cc1	30.4	27
1700	**4**{*386 ,725 ,5* }	CCN1N=NC=C1C1=C(NCCCOC)N2C=CC(C)=CC2=N1 |c:3,5,8,17,20,23|	Z8873684786	**1**{*386* }	Cc1ccnc(N)c1	**2**{*725* }	CCn1nncc1C=O	**3**{*5* }	COCCC[N+]#[C-]	22.8	27
1701	**4**{*99 ,767 ,73* }	CCCC(OC)C1=C(NC2=C(OC)C=C(OC)C=C2)N2C=C(C=CC2=N1)S(N)(=O)=O |c:6,9,17,21,23,26,t:13|	Z8855619704	**1**{*99* }	Nc1ccc(cn1)S(=O)(=O)N	**2**{*767* }	CCCC(OC)C=O	**3**{*73* }	COc1ccc([N+]#[C-])c(OC)c1	31.6	27
1702	**4**{*103 ,768 ,5* }	COCCCNC1=C(N=C2C=NC=C(CO)N12)C1=C(F)C(Br)=C(F)C=C1 |c:10,19,26,t:6,8,12,23|	Z8873685426	**1**{*103* }	Nc1cncc(CO)n1	**2**{*768* }	Fc1ccc(C=O)c(F)c1Br	**3**{*5* }	COCCC[N+]#[C-]	30.9	27
1703	**4**{*91 ,125 ,26* }	COC1=NC=NC(=C1)C1=C(NCC2=CC=C(F)C=C2)N2C=C(C=CC2=N1)C(N)=O |c:4,6,9,18,22,24,27,t:2,13,15|	Z8878918391	**1**{*91* }	NC(=O)c1ccc(N)nc1	**2**{*125* }	COc1cc(C=O)ncn1	**3**{*26* }	Fc1ccc(C[N+]#[C-])cc1	28.3	27

1704	**4**{*124*,*769*,*43*}	CN1N=CN=C1C1=C(NC2CCN(CC2)C(=O)OC(C)(C)C)N2C=CC=C(CO)C2=N1 |c:2,4,7,25,32,t:27|	Z8878918536	**1**{*124*}	Nc1ncccc1CO	**2**{*769*}	Cn1ncnc1C=O	**3**{*43*}	CC(C)(C)OC(=O)N1CCC(CC1)[N+]#[C-]	30.8	27
1705	**4**{*78*,*770*,*39*}	CCOCCNC1=C(N=C2C=C(C)C3=NC(C)=CN3N12)C1=C(OC)C=CC(Cl)=C1Cl |c:16,23,27,30,t:6,8,10,13|	Z8873684775	**1**{*78*}	Cc1cn2nc(N)cc(C)c2n1	**2**{*770*}	COc1ccc(Cl)c(Cl)c1C=O	**3**{*39*}	CCOCC[N+]#[C-]	32.3	27
1706	**4**{*155*,*450*,*14*}	COCCNC1=C(N=C2N1C=CC=C2Cl)C1=CC(=CC=C1)S(=O)(=O)N(C)C |c:7,11,13,19,21,t:5,17|	Z8873684599	**1**{*155*}	Nc1ncccc1Cl	**2**{*450*}	CN(C)S(=O)(=O)c1cccc(C=O)c1	**3**{*14*}	COCC[N+]#[C-]	29.4	27
1707	**4**{*48*,*216*,*15*}	CCNC1=C(N=C2C=C(C=CN12)S(C)(=O)=O)C1=CN=C(S1)C(=O)OC |c:7,9,20,t:3,5,18|	Z8878918866	**1**{*48*}	CS(=O)(=O)c1ccnc(N)c1	**2**{*216*}	COC(=O)c1ncc(C=O)s1	**3**{*15*}	CC[N+]#[C-]	27.2	27
1708	**4**{*175*,*516*,*46*}	CN1N=C(N=C1C1CC1)C1=C(NCC(C)=C)N2C=CC=C(OCCF)C2=N1 |c:2,4,11,19,28,t:21|	Z8873684651	**1**{*175*}	Nc1ncccc1OCCF	**2**{*516*}	Cn1nc(C=O)nc1C2CC2	**3**{*46*}	CC(=C)C[N+]#[C-]	26.5	26
1709	**4**{*32*,*771*,*15*}	CCNC1=C(N=C2C=C(C=CN12)P(C)(C)=O)C1=CC=C(C=C1)C(F)(F)Cl |c:7,9,20,22,t:3,5,18|	Z8846492144	**1**{*32*}	CP(=O)(C)c1ccnc(N)c1	**2**{*771*}	FC(F)(Cl)c1ccc(C=O)cc1	**3**{*15*}	CC[N+]#[C-]	28.1	26
1710	**4**{*103*,*772*,*5*}	COCCCNC1=C(N=C2C=NC=C(CO)N12)C1=NC=C(OC(F)(F)C(F)(F)F)C=C1 |c:10,31,t:6,8,12,19,21|	Z8873684802	**1**{*103*}	Nc1cncc(CO)n1	**2**{*772*}	FC(F)(F)C(F)(F)Oc1ccc(C=O)nc1	**3**{*5*}	COCCC[N+]#[C-]	31.6	26
1711	**4**{*123*,*119*,*32*}	CCOC(=O)C1=NN=C(N1)C1=C(NC2CCC2)N2C=CC(OCCO)=CC2=N1 |c:7,11,20,26,29,t:5|	Z8855739092	**1**{*123*}	Cl.Nc1cc(OCCO)ccn1	**2**{*119*}	CCOC(=O)c1nnc(C=O)[nH]1	**3**{*32*}	[C-]#[N+]C1CCC1	27.3	26
1712	**4**{*28*,*773*,*7*}	CC(C)CN1N=C(C)C(=C1Cl)C1=C(NC2CCOCC2)N2C=CC(=CC2=N1)C1=NOC=N1 |c:8,12,23,25,28,34,t:5,31|	Z8878918880	**1**{*28*}	Cl.Nc1cc(ccn1)c2ncon2	**2**{*773*}	CC(C)Cn1nc(C)c(C=O)c1Cl	**3**{*7*}	[C-]#[N+]C1CCOCC1	32.1	26
1713	**4**{*91*,*774*,*35*}	CSCCCNC1=C(N=C2C=CC(=CN12)C(N)=O)C1=NC(Br)=CS1 |c:10,12,23,t:6,8,20|	Z8835022912	**1**{*91*}	NC(=O)c1ccc(N)nc1	**2**{*774*}	Brc1csc(C=O)n1	**3**{*35*}	CSCCC[N+]#[C-]	30.0	26
1714	**4**{*46*,*48*,*9*}	CNC1=C(N=C2C=CC=CN12)C1=CNC(=N1)C1CCC1 |c:6,8,16,t:2,4,13|	Z8878918425	**1**{*46*}	Nc1ccccn1	**2**{*48*}	O=Cc1c[nH]c(n1)C2CCC2	**3**{*9*}	C[N+]#[C-]	18.8	26
1715	**4**{*186*,*775*,*66*}	COC(=O)C1=NC(C)=C(S1)C1=C(NC(C)C2=CC=CC=C2)N2C=CC(=CC2=N1)S(=O)(=O)N(C)C |c:7,11,18,20,24,26,29,t:4,16|	Z8855739085	**1**{*186*}	Cl.CN(C)S(=O)(=O)c1ccnc(N)c1	**2**{*775*}	COC(=O)c1nc(C)c(C=O)s1	**3**{*66*}	CC([N+]#[C-])c1ccccc1	35.0	26

1716	**4**{*28*,*225*,*7*}	ClC1=CC2=C(C=NN2)C(=C1)C1=C(NC2CCOCC2)N2C=CC(=CC2=N1)C1=NOC=N1 |c:5,9,12,23,25,28,34,t:1,3,31|	Z8835022876	**1**{*28*}	Cl.Nc1cc(ccn1)c2ncon2	**2**{*225*}	Clc1cc(C=O)c2cn[nH]c2c1	**3**{*7*}	[C-]#[N+]C1CCOCC1	30.4	26
1717	**4**{*144*,*776*,*22*}	CNS(=O)(=O)C1=CN2C(C=C1)=NC(=C2NC1COC1)C1=C(F)C=C(C=C1F)C#C |c:9,11,13,22,25,27,t:5|	Z8837933171	**1**{*144*}	CNS(=O)(=O)c1ccc(N)nc1	**2**{*776*}	Fc1cc(C#C)cc(F)c1C=O	**3**{*22*}	[C-]#[N+]C1COC1	29.1	26
1718	**4**{*187*,*777*,*12*}	COC(C1CCCC1)C1=C(NCC2CCOC2)N2C=C(Cl)C(=CC2=N1)C#N |c:9,23,26,t:20|	Z8878918559	**1**{*187*}	Nc1cc(C#N)c(Cl)cn1	**2**{*777*}	COC(C=O)C1CCCC1	**3**{*12*}	[C-]#[N+]CC1CCOC1	27.0	26
1719	**4**{*380*,*406*,*23*}	COC(=O)CCC(NC1=C(N=C2N1C=C(Cl)N=C2C)C1=CC=NS1)C(=O)OC |c:10,17,23,t:8,14,21|	Z8878918820	**1**{*380*}	Cc1nc(Cl)cnc1N	**2**{*406*}	O=Cc1ccns1	**3**{*23*}	COC(=O)CCC([N+]#[C-])C(=O)OC	29.3	26
1720	**4**{*47*,*469*,*6*}	CCOC(=O)CCCNC1=C(N=C2C=C(C=CN12)S(N)(=O)=O)C1CCSC1 |c:13,15,t:9,11|	Z8878918614	**1**{*47*}	Cl.Nc1cc(ccn1)S(=O)(=O)N	**2**{*469*}	O=CC1CCSC1	**3**{*6*}	CCOC(=O)CCC[N+]#[C-]	28.2	25
1721	**4**{*49*,*116*,*13*}	COC1=CC=C(CNC2=C(N=C3C=CC=C(CO)N23)C2=CN(CCC=C)N=N2)C=C1 |c:12,28,31,t:2,4,8,10,14,21|	Z8854581145	**1**{*49*}	Nc1cccc(CO)n1	**2**{*116*}	C=CCCn1cc(C=O)nn1	**3**{*13*}	COc1ccc(C[N+]#[C-])cc1	27.6	25
1722	**4**{*49*,*284*,*13*}	COC1=CC=C(CNC2=C(N=C3C=CC=C(CO)N23)C2=C(C)N=CC=N2)C=C1 |c:12,21,24,26,29,t:2,4,8,10,14|	Z8873684722	**1**{*49*}	Nc1cccc(CO)n1	**2**{*284*}	Cc1nccnc1C=O	**3**{*13*}	COc1ccc(C[N+]#[C-])cc1	25.4	25
1723	**4**{*50*,*760*,*18*}	CC(C)(C)NC1=C(N=C2C=CC=C(N12)P(C)(C)=O)C1=CC(F)=C(C=C1)C1CC1 |c:9,11,23,25,t:5,7,20|	Z8873684866	**1**{*50*}	CP(=O)(C)c1cccc(N)n1	**2**{*760*}	Fc1cc(C=O)ccc1C2CC2	**3**{*18*}	CC(C)(C)[N+]#[C-]	26.9	25
1724	**4**{*428*,*710*,*10*}	CC1=C(C=C(F)C=N1)C1=C(NC2CCOC2)N2C=C(Br)N=CC2=N1 |c:6,9,22,25,t:1,3,19|	Z8855739115	**1**{*428*}	Nc1cnc(Br)cn1	**2**{*710*}	Cc1ncc(F)cc1C=O	**3**{*10*}	[C-]#[N+]C1CCOC1	26.4	25
1725	**4**{*79*,*420*,*13*}	COC1=CC=C(CNC2=C(CC(C)(C)C#N)N=C3C=NC=C(C)N23)C=C1 |c:8,18,26,t:2,4,16,20|	Z8873684695	**1**{*79*}	Cc1cncc(N)n1	**2**{*420*}	CC(C)(CC=O)C#N	**3**{*13*}	COc1ccc(C[N+]#[C-])cc1	23.5	25
1726	**4**{*36*,*396*,*9*}	CNC1=C(N=C2C=CC(Br)=CN12)C1=CN=C2COCCN12 |c:6,9,t:2,4,14,16|	Z8855739232	**1**{*36*}	Nc1ccc(Br)cn1	**2**{*396*}	O=Cc1cnc2COCCn12	**3**{*9*}	C[N+]#[C-]	23.1	25
1727	**4**{*120*,*778*,*38*}	CCOC(=O)C1=NN(C)C(=N1)C1=C(NC2=CC3=C(OCO3)C=C2)N2C=CC(=CC2=N1)C1=CN(C)N=C1 |c:9,12,23,27,29,32,39,t:5,15,17,35|	Z8878918472	**1**{*120*}	Cn1cc(cn1)c2ccnc(N)c2	**2**{*778*}	CCOC(=O)c1nc(C=O)n(C)n1	**3**{*38*}	[C-]#[N+]c1ccc2OCOc2c1	32.2	24
1728	**4**{*139*,*104*,*46*}	CC(=C)CNC1=C(N=C2C=C(C=CN12)C(F)F)C1=NC(C)=CO1 |c:9,11,22,t:5,7,19|	Z8873684776	**1**{*139*}	Nc1cc(ccn1)C(F)F	**2**{*104*}	Cc1coc(C=O)n1	**3**{*46*}	CC(=C)C[N+]#[C-]	21.0	24

1729	**4**{*41*,*25*,*9*}	CNC1=C(N=C2C=NC(C)=CN12)C1=CN=C(N=C1)C1CCOCC1 |c:6,9,16,18,t:2,4,14|	Z8837933133	**1**{*41*}	Cc1cnc(N)cn1	**2**{*25*}	O=Cc1cnc(nc1)C2CCOCC2	**3**{*9*}	C[N+]#[C-]	21.4	24
1730	**4**{*146*,*608*,*72*}	CC1=C(NC2=C(CC3(CCC3)C#N)N=C3C=CC(=CN23)S(C)(=O)=O)C=CC(F)=C1 |c:1,4,16,18,27,30,t:14|	Z8835022903	**1**{*146*}	CS(=O)(=O)c1ccc(N)nc1	**2**{*608*}	O=CCC1(CCC1)C#N	**3**{*72*}	Cc1cc(F)ccc1[N+]#[C-]	27.2	24
1731	**4**{*397*,*312*,*23*}	COC(=O)CCC(NC1=C(N=C2N1C=CC(C)=C2C)C1CCOC1(C)C)C(=O)OC |c:10,14,17,t:8|	Z8873684611	**1**{*397*}	Cl.Cc1ccnc(N)c1C	**2**{*312*}	CC1(C)OCCC1C=O	**3**{*23*}	COC(=O)CCC([N+]#[C-])C(=O)OC	27.4	24
1732	**4**{*70*,*115*,*32*}	COC1=NN(C)C(=C1)C1=C(NC2CCC2)N2C=CN=C(C3CC3)C2=N1 |c:6,9,18,27,t:2,20|	Z8873685440	**1**{*70*}	Nc1nccnc1C2CC2	**2**{*115*}	COc1cc(C=O)n(C)n1	**3**{*32*}	[C-]#[N+]C1CCC1	22.1	24
1733	**4**{*138*,*236*,*61*}	CC(C)(C)OC(=O)NCCCNC1=C(CCC2OCCO2)N=C2C=CC=C(F)N12 |c:12,24,t:22,26|	Z8878918528	**1**{*138*}	Nc1cccc(F)n1	**2**{*236*}	O=CCCC1OCCO1	**3**{*61*}	CC(C)(C)OC(=O)NCCC[N+]#[C-]	26.7	24
1734	**4**{*428*,*334*,*10*}	CC1=C(N=CO1)C1=C(NC2CCOC2)N2C=C(Br)N=CC2=N1 |c:3,7,20,23,t:1,17|	Z8878918814	**1**{*428*}	Nc1cnc(Br)cn1	**2**{*334*}	Cc1ocnc1C=O	**3**{*10*}	[C-]#[N+]C1CCOC1	23.7	24
1735	**4**{*230*,*320*,*50*}	CC(O)C1=CC(Br)=CN2C(NC3=CC4=C(OCCO4)C=C3)=C(N=C12)C1=NN(C)C=N1 |c:6,20,22,32,t:3,11,13,24,28|	Z8873685280	**1**{*230*}	CC(O)c1cc(Br)cnc1N	**2**{*320*}	Cn1cnc(C=O)n1	**3**{*50*}	[C-]#[N+]c1ccc2OCCOc2c1	30.7	24
1736	**4**{*150*,*131*,*15*}	CCNC1=C(N=C2C=C(C=CN12)C1CCOC1)C1=NC(=NN1)C1CC1 |c:7,9,22,t:3,5,20|	Z8855619710	**1**{*150*}	Nc1cc(ccn1)C2CCOC2	**2**{*131*}	O=Cc1nc(n[nH]1)C2CC2	**3**{*15*}	CC[N+]#[C-]	22.0	24
1737	**4**{*38*,*697*,*9*}	CCC1=NC=C(N1)C1=C(NC)N2C=CC(=CC2=N1)C#N |c:4,8,13,15,18,t:2|	Z8837933174	**1**{*38*}	Nc1cc(C#N)ccn1	**2**{*697*}	CCc1ncc(C=O)[nH]1	**3**{*9*}	C[N+]#[C-]	17.3	24
1738	**4**{*361*,*164*,*28*}	CCCNC1=C(N=C2N1C=CC=C2OC(C)C)C1=NN(C)C2=C1COCC2 |c:6,10,12,23,t:4,19|	Z8855739088	**1**{*361*}	Cl.CC(C)Oc1cccnc1N	**2**{*164*}	Cn1nc(C=O)c2COCCc21	**3**{*28*}	CCC[N+]#[C-]	23.9	24
1739	**4**{*161*,*49*,*11*}	CC1=NNC=C1C1=C(NCC2=CC(Br)=CC=C2)N2N=CC=CC2=N1 |c:4,7,14,16,20,22,25,t:1,11|	Z8873685264	**1**{*161*}	Nc1cccnn1	**2**{*49*}	Cc1n[nH]cc1C=O	**3**{*11*}	Brc1cccc(C[N+]#[C-])c1	24.8	24
1740	**4**{*399*,*779*,*22*}	COC1=C2NC=NC2=CC(=C1)C1=C(NC2COC2)N2N=C(OC(C)C)C=CC2=N1 |c:2,5,8,10,13,28,31,t:22|	Z8854581138	**1**{*399*}	CC(C)Oc1ccc(N)nn1	**2**{*779*}	COc1cc(C=O)cc2nc[nH]c12	**3**{*22*}	[C-]#[N+]C1COC1	25.1	24

1741	**4**{*124* ,*460* ,*61* }	CC1=CN=C(O1)C1=C(NCCCNC(=O)OC(C)(C)C)N2C=CC=C(CO)C2=N1 |c:3,7,22,29,t:1,24|	Z8873684796	**1**{*124* }	Nc1ncccc1CO	**2**{*460* }	Cc1cnc(C=O)o1	**3**{*61* }	CC(C)(C)OC(=O)NCCC[N+]#[C-]	25.4	23
1742	**4**{*436* ,*235* ,*23* }	CCC1=CC2=NC(C3=CSC(=C3)C#N)=C(NC(CCC(=O)OC)C(=O)OC)N2C=C1 |c:10,30,t:2,4,7,14|	Z8873684840	**1**{*436* }	CCc1ccnc(N)c1	**2**{*235* }	O=Cc1csc(C#N)c1	**3**{*23* }	COC(=O)CCC([N+]#[C-])C(=O)OC	26.8	23
1743	**4**{*54* ,*780* ,*37* }	COC(=O)C1CC(C1)NC1=C(N=C2C=CC(=CN12)C(=O)N(C)C)[C@@H]1C[C@H]1C1=CSC=C1 |&1:23,25,r,c:14,16,33,t:10,12,30|	Z8873684637	**1**{*54* }	Cl.CN(C)C(=O)c1ccc(N)nc1	**2**{*780* }	O=C[C@@H]1C[C@H]1c2ccsc2 |&1:2,4,r|	**3**{*37* }	COC(=O)C1CC(C1)[N+]#[C-]	27.0	23
1744	**4**{*32* ,*744* ,*7* }	CP(C)(=O)C1=CC2=NC(=C(NC3CCOCC3)N2C=C1)C1=C(F)C=CC(=C1)C#C |c:20,23,26,28,t:4,6,8|	Z8873684685	**1**{*32* }	CP(=O)(C)c1ccnc(N)c1	**2**{*744* }	Fc1ccc(C#C)cc1C=O	**3**{*7* }	[C-]#[N+]C1CCOCC1	25.3	23
1745	**4**{*89* ,*569* ,*67* }	CCC(C)NC1=C(CC2CCOC2=O)N=C2C=CC3=C(NN=C3)N12 |c:5,17,22,t:15,19|	Z8873684913	**1**{*89* }	Nc1ccc2cn[nH]c2n1	**2**{*569* }	O=CCC1CCOC1=O	**3**{*67* }	CCC(C)[N+]#[C-]	20.1	23
1746	**4**{*78* ,*629* ,*44* }	CC1=CN2N3C(C=C(C)C2=N1)=NC(=C3NCCCCNC(=O)OC(C)(C)C)C1=C(C)C=C(C)C=N1 |c:10,12,14,30,36,t:1,6,33|	Z8878918890	**1**{*78* }	Cc1cn2nc(N)cc(C)c2n1	**2**{*629* }	Cc1cnc(C=O)c(C)c1	**3**{*44* }	CC(C)(C)OC(=O)NCCCC[N+]#[C-]	29.2	23
1747	**4**{*32* ,*439* ,*31* }	COC1=C(C=CC(=C1)C1=C(NCCSC)N2C=CC(=CC2=N1)P(C)(C)=O)S(C)(=O)=O |c:4,6,9,17,19,22,t:2|	Z8873684927	**1**{*32* }	CP(=O)(C)c1ccnc(N)c1	**2**{*439* }	COc1cc(C=O)ccc1S(=O)(=O)C	**3**{*31* }	CSCC[N+]#[C-]	28.2	22
1748	**4**{*50* ,*781* ,*18* }	CC(C)(C)NC1=C(N=C2C=CC=C(N12)P(C)(C)=O)C1=CC(Cl)=C(OCCBr)C=C1 |c:9,11,29,t:5,7,20,23|	Z8873685421	**1**{*50* }	CP(=O)(C)c1cccc(N)n1	**2**{*781* }	Clc1cc(C=O)ccc1OCCBr	**3**{*18* }	CC(C)(C)[N+]#[C-]	30.1	22
1749	**4**{*101* ,*538* ,*7* }	CN1C=NC=C1C1=C(NC2CCOCC2)N2C=C(CO)C=C(Cl)C2=N1 |c:2,4,7,26,t:18,22|	Z8854581140	**1**{*101* }	Nc1ncc(CO)cc1Cl	**2**{*538* }	Cn1cncc1C=O	**3**{*7* }	[C-]#[N+]C1CCOCC1	21.8	22
1750	**4**{*161* ,*84* ,*20* }	COC1=CC(C)=C(NC2=C(CCSC)N=C3C=CC=NN23)C=C1 |c:8,16,18,23,t:2,5,14|	Z8878918342	**1**{*161* }	Nc1cccnn1	**2**{*84* }	CSCCC=O	**3**{*20* }	COc1ccc([N+]#[C-])c(C)c1	19.8	22
1751	**4**{*118* ,*782* ,*35* }	CSCCCNC1=C(N=C2C=CC=C(C(C)O)N12)C1=C2N(C)C(=O)N(C)C2=CC=C1 |c:10,20,29,31,t:6,8,12|	Z8873684944	**1**{*118* }	Cl.CC(O)c1cccc(N)n1	**2**{*782* }	Cn1c(=O)n(C)c2c(C=O)cccc12	**3**{*35* }	CSCCC[N+]#[C-]	25.5	22
1752	**4**{*400* ,*155* ,*15* }	CCNC1=C(N=C2C=CC(COC(C)C)=CN12)C1=CN=CN=C1 |c:7,14,21,23,t:3,5,19|	Z8855739166	**1**{*400* }	CC(C)OCc1ccc(N)nc1	**2**{*155* }	O=Cc1cncnc1	**3**{*15* }	CC[N+]#[C-]	18.6	22

1753	**4**{*377,34,46*}	CC(=C)CNC1=C(N=C2N1C=CC=C2OCC(F)F)C1=CC=C(C=C1)P(C)(C)=O |c:7,11,13,23,25,t:5,21|	Z8873684633	**1**{*377*}	Nc1ncccc1OCC(F)F	**2**{*34*}	CP(=O)(C)c1ccc(C=O)cc1	**3**{*46*}	CC(=C)C[N+]#[C-]	25.0	22
1754	**4**{*414,236,60*}	COC(=O)C1=CC(NC2=C(CCC3OCCO3)N=C3C=NC(Cl)=CN23)=CC=C1 |c:8,20,23,27,29,t:4,18|	Z8878918661	**1**{*414*}	Nc1cnc(Cl)cn1	**2**{*236*}	O=CCCC1OCCO1	**3**{*60*}	COC(=O)c1cccc([N+]#[C-])c1	23.8	22
1755	**4**{*56,783,23*}	COC(=O)CCC(NC1=C(N=C2C=CC(=CN12)C1=NN(C)C=C1)C1=NOC(=C1)C(C)C)C(=O)OC |c:12,14,23,29,t:8,10,19,26|	Z8829498623	**1**{*56*}	Cn1ccc(n1)c2ccc(N)nc2	**2**{*783*}	CC(C)c1cc(C=O)no1	**3**{*23*}	COC(=O)CCC([N+]#[C-])C(=O)OC	28.3	22
1756	**4**{*150,488,35*}	CSCCCNC1=C(N=C2C=C(C=CN12)C1CCOC1)C1=CC2=C(NC(=O)CC2)N=C1 |c:10,12,33,t:6,8,23,25|	Z8855739215	**1**{*150*}	Nc1cc(ccn1)C2CCOC2	**2**{*488*}	O=Cc1cnc2NC(=O)CCc2c1	**3**{*35*}	CSCCC[N+]#[C-]	25.6	22
1757	**4**{*301,93,32*}	OCC1=NC(=CC=C1)C1=C(NC2CCC2)N2C=C(Cl)C=C(F)C2=N1 |c:4,6,9,25,t:2,18,21|	Z8878918582	**1**{*301*}	Nc1ncc(Cl)cc1F	**2**{*93*}	OCc1cccc(C=O)n1	**3**{*32*}	[C-]#[N+]C1CCC1	20.1	21
1758	**4**{*351,784,39*}	CCOCCNC1=C(N=C2C=CC3=C(C(O)CC3)N12)C1=C(Cl)N=C(OC)N1C |c:10,22,t:6,8,12,25|	Z8873685475	**1**{*351*}	Nc1ccc2CCC(O)c2n1	**2**{*784*}	COc1nc(Cl)c(C=O)n1C	**3**{*39*}	CCOCC[N+]#[C-]	23.4	21
1759	**4**{*442,177,23*}	COC(=O)CCC(NC1=C(CC2CC(C2)C(=O)OC)N=C2N1C=C(Cl)C=C2C)C(=O)OC |c:8,20,27,t:24|	Z8878918316	**1**{*442*}	Cc1cc(Cl)cnc1N	**2**{*177*}	COC(=O)C1CC(CC=O)C1	**3**{*23*}	COC(=O)CCC([N+]#[C-])C(=O)OC	26.8	21
1760	**4**{*469,745,22*}	CC1=C(C=CN=C1)C1=C(NC2COC2)N2C=C(OC(F)(F)F)C=CC2=N1 |c:3,5,8,24,27,t:1,17|	Z8878918481	**1**{*469*}	Nc1ccc(OC(F)(F)F)cn1	**2**{*745*}	Cc1cnccc1C=O	**3**{*22*}	[C-]#[N+]C1COC1	19.9	21
1761	**4**{*99,785,66*}	CCN1N=C(Br)N=C1C1=C(NC(C)C2=CC=CC=C2)N2C=C(C=CC2=N1)S(N)(=O)=O |c:6,9,16,18,22,24,27,t:3,14|	Z8873685434	**1**{*99*}	Nc1ccc(cn1)S(=O)(=O)N	**2**{*785*}	CCn1nc(Br)nc1C=O	**3**{*66*}	CC([N+]#[C-])c1ccccc1	28.1	21
1762	**4**{*103,786,5*}	COCCCNC1=C(N=C2C=NC=C(CO)N12)C1=C(F)C(F)=C(Cl)C=C1 |c:10,19,26,t:6,8,12,23|	Z8873684818	**1**{*103*}	Nc1cncc(CO)n1	**2**{*786*}	Fc1c(F)c(C=O)ccc1Cl	**3**{*5*}	COCCC[N+]#[C-]	21.9	21
1763	**4**{*188,787,62*}	COC(=O)C1=CN=CC2=NC(=C(NC3(CCCC3)C(=O)OC)N12)C1=CC2=C(C=C1)N(C)C(C)=N2 |c:6,28,30,36,t:4,8,10,26|	Z8873684885	**1**{*188*}	COC(=O)c1cncc(N)n1	**2**{*787*}	Cc1nc2cc(C=O)ccc2n1C	**3**{*62*}	COC(=O)C1(CCCC1)[N+]#[C-]	26.2	21
1764	**4**{*35,25,13*}	COC1=CC=C(CNC2=C(N=C3C=CC(C)=CN23)C2=CN=C(N=C2)C2CCOCC2)C=C1 |c:12,15,22,24,34,t:2,4,8,10,20|	Z8873685250	**1**{*35*}	Cc1ccc(N)nc1	**2**{*25*}	O=Cc1cnc(nc1)C2CCOCC2	**3**{*13*}	COc1ccc(C[N+]#[C-])cc1	24.3	21

1765	**4**{*138*,*729*,*49*}	COC1=C(C=CC=C1C#N)C1=C(NC2CC(F)(F)C2)N2C(C=CC=C2F)=N1 |c:4,6,11,23,25,28,t:2|	Z8873685330	**1**{*138*}	Nc1cccc(F)n1	**2**{*729*}	COc1c(C=O)cccc1C#N	**3**{*49*}	FC1(F)CC(C1)[N+]#[C-]	20.9	21
1766	**4**{*396*,*587*,*59*}	COC1=NOC(C)=C1C1=C(NCCC2=CC(F)=CC=C2)N2N=C(C=CC2=N1)C1=CN(C)N=C1 |c:6,9,17,19,23,25,28,35,t:2,14,31|	Z8873685432	**1**{*396*}	Cn1cc(cn1)c2ccc(N)nn2	**2**{*587*}	COc1noc(C)c1C=O	**3**{*59*}	Fc1cccc(CC[N+]#[C-])c1	24.9	21
1767	**4**{*128*,*653*,*34*}	COCCN(C)C1=CN2C(C=C1)=NC(C1C3CCC(F)(F)C13)=C2NCC1CCCO1 |c:10,12,24,t:6|	Z8829498615	**1**{*128*}	COCCN(C)c1ccc(N)nc1	**2**{*653*}	FC1(F)CCC2C(C=O)C21	**3**{*34*}	[C-]#[N+]CC1CCCO1	23.4	21
1768	**4**{*421*,*306*,*22*}	COC(=O)C1=C(Cl)N2C(C=C1)=NC(=C2NC1COC1)C1=CC=C(C=C1)N(C)CCO |c:4,9,11,13,24,26,t:22|	Z8837933165	**1**{*421*}	COC(=O)c1ccc(N)nc1Cl	**2**{*306*}	CN(CCO)c1ccc(C=O)cc1	**3**{*22*}	[C-]#[N+]C1COC1	23.9	21
1769	**4**{*351*,*788*,*39*}	CCOCCNC1=C(CC2(CCCC2)NC(=O)OC(C)(C)C)N=C2C=CC3=C(C(O)CC3)N12 |c:6,25,t:23,27|	Z8873685499	**1**{*351*}	Nc1ccc2CCC(O)c2n1	**2**{*788*}	CC(C)(C)OC(=O)NC1(CC=O)CCCC1	**3**{*39*}	CCOCC[N+]#[C-]	25.2	20
1770	**4**{*254*,*57*,*37*}	COC(=O)C1CC(C1)NC1=C(N=C2C=C(C)C(=CN12)C(=O)OC)C1CCOC1 |c:17,t:10,12,14|	Z8878918642	**1**{*254*}	COC(=O)c1cnc(N)cc1C	**2**{*57*}	O=CC1CCOC1	**3**{*37*}	COC(=O)C1CC(C1)[N+]#[C-]	21.2	20
1771	**4**{*50*,*789*,*18*}	CC(C)(C)NC1=C(N=C2C=CC=C(N12)P(C)(C)=O)C1=CC(F)=C(C=C1)C(F)(F)F |c:9,11,23,25,t:5,7,20|	Z8878918803	**1**{*50*}	CP(=O)(C)c1cccc(N)n1	**2**{*789*}	Fc1cc(C=O)ccc1C(F)(F)F	**3**{*18*}	CC(C)(C)[N+]#[C-]	23.3	20
1772	**4**{*356*,*110*,*69*}	COC1=C(NC2=C(N=C3C=CC(Cl)=C(N23)C(=O)N(C)C)C2=CN=C(C=C2)P(C)(C)=O)C=CC=C1 |c:2,9,12,24,26,33,35,t:5,7,22|	Z8849597827	**1**{*356*}	CN(C)C(=O)c1nc(N)ccc1Cl	**2**{*110*}	CP(=O)(C)c1ccc(C=O)cn1	**3**{*69*}	COc1ccccc1[N+]#[C-]	27.0	20
1773	**4**{*91*,*790*,*7*}	COC(=O)C1=C(F)C=C(C=C1F)C1=C(NC2CCOCC2)N2C=C(C=CC2=N1)C(N)=O |c:4,7,9,13,24,26,29|	Z8873684767	**1**{*91*}	NC(=O)c1ccc(N)nc1	**2**{*790*}	COC(=O)c1c(F)cc(C=O)cc1F	**3**{*7*}	[C-]#[N+]C1CCOCC1	23.3	20
1774	**4**{*112*,*627*,*24*}	CCC1=NC(C)=C(N1)C1=C(NCC2=CC(OC)=CC=C2)N2C(C=NC=C2OC)=N1 |c:5,9,17,19,24,26,30,t:2,13|	Z8873685496	**1**{*112*}	COc1cncc(N)n1	**2**{*627*}	CCc1nc(C)c(C=O)[nH]1	**3**{*24*}	COc1cccc(C[N+]#[C-])c1	20.9	20
1775	**4**{*199*,*654*,*34*}	CC(F)(F)C1=CC=CN2C(NCC3CCCO3)=C(CC3=CN=CO3)N=C12 |c:6,23,t:4,17,21,27|	Z8873684785	**1**{*199*}	CC(F)(F)c1cccnc1N	**2**{*654*}	O=CCCc1cnco1	**3**{*34*}	[C-]#[N+]CC1CCCO1	20.0	20
1776	**4**{*50*,*791*,*7*}	CC1=CN2C=CC=C(C2=N1)C1=C(NC2CCOCC2)N2C(C=CC=C2P(C)(C)=O)=N1 |c:4,6,9,12,24,26,32,t:1|	Z8854581150	**1**{*50*}	CP(=O)(C)c1cccc(N)n1	**2**{*791*}	Cc1cn2cccc(C=O)c2n1	**3**{*7*}	[C-]#[N+]C1CCOCC1	22.5	20

1777	**4**{*209*,*153*,*14*}	COCCNC1=C(N=C2N1C=CC=C2N1CCCCC1)C1=CN(CC(F)(F)F)N=N1 |c:7,11,13,31,t:5,23|	Z8855739191	**1**{*209*}	Nc1ncccc1N2CCCCC2	**2**{*153*}	FC(F)(F)Cn1cc(C=O)nn1	**3**{*14*}	COCC[N+]#[C-]	22.5	20
1778	**4**{*166*,*748*,*15*}	CCNC1=C(N=C2C=C(CSCCO)C=CN12)C1=CC(=NN1C)C(F)(F)F |c:14,21,t:3,5,7,19|	Z8873684936	**1**{*166*}	Nc1cc(CSCCO)ccn1	**2**{*748*}	Cn1nc(cc1C=O)C(F)(F)F	**3**{*15*}	CC[N+]#[C-]	21.1	20
1779	**4**{*73*,*77*,*20*}	COC1=CC(C)=C(NC2=C(CC3CCC3)N=C3C=CC4=NC=CN4N23)C=C1 |c:8,19,23,30,t:2,5,17,21|	Z8873684830	**1**{*73*}	Nc1ccc2nccn2n1	**2**{*77*}	O=CCC1CCC1	**3**{*20*}	COc1ccc([N+]#[C-])c(C)c1	19.8	19
1780	**4**{*470*,*704*,*39*}	CCOCCNC1=C(N=C2C=CC3=C(C=C(C=C3)C(=O)OC)N12)C1=NC(=NN1C)C1CC1 |c:10,14,16,28,t:6,8,12,26|	Z8878918435	**1**{*470*}	COC(=O)c1ccc2ccc(N)nc2c1	**2**{*704*}	Cn1nc(nc1C=O)C2CC2	**3**{*39*}	CCOCC[N+]#[C-]	22.1	19
1781	**4**{*87*,*81*,*23*}	COC(=O)CCC(NC1=C(N=C2C=C(C)C(Cl)=CN12)C1=NC(=CC=C1)C(F)F)C(=O)OC |c:16,23,25,t:8,10,12,21|	Z8873684939	**1**{*87*}	Cc1cc(N)ncc1Cl	**2**{*81*}	FC(F)c1cccc(C=O)n1	**3**{*23*}	COC(=O)CCC([N+]#[C-])C(=O)OC	23.8	19
1782	**4**{*83*,*412*,*18*}	CSC1=CC(=CC=C1)C1=C(NC(C)(C)C)N2C=C(C=CC2=N1)P(C)(C)=O |c:4,6,9,17,19,22,t:2|	Z8878918865	**1**{*83*}	CP(=O)(C)c1ccc(N)nc1	**2**{*412*}	CSc1cccc(C=O)c1	**3**{*18*}	CC(C)(C)[N+]#[C-]	19.7	19
1783	**4**{*99*,*767*,*50*}	CCCC(OC)C1=C(NC2=CC3=C(OCCO3)C=C2)N2C=C(C=CC2=N1)S(N)(=O)=O |c:6,18,22,24,27,t:9,11|	Z8873684922	**1**{*99*}	Nc1ccc(cn1)S(=O)(=O)N	**2**{*767*}	CCCC(OC)C=O	**3**{*50*}	[C-]#[N+]c1ccc2OCCOc2c1	21.5	18
1784	**4**{*184*,*695*,*14*}	COCCNC1=C(N=C2C=CC(=CN12)C(N)=S)C1=CC(OCC(=O)OC)=C(OC)C=C1 |c:9,11,31,t:5,7,19,27|	Z8878918894	**1**{*184*}	NC(=S)c1ccc(N)nc1	**2**{*695*}	COC(=O)COc1cc(C=O)ccc1OC	**3**{*14*}	COCC[N+]#[C-]	22.0	18
1785	**4**{*275*,*172*,*14*}	COCCNC1=C(N=C2N1C(Cl)=CC=C2F)C1=CNC(=N1)C1CC1 |c:7,12,14,21,t:5,18|	Z8835022886	**1**{*275*}	Nc1nc(Cl)ccc1F	**2**{*172*}	O=Cc1c[nH]c(n1)C2CC2	**3**{*14*}	COCC[N+]#[C-]	17.3	18
1786	**4**{*455*,*42*,*23*}	COC(=O)CCC(NC1=C(N=C2C=C(Cl)C=C(Cl)N12)C1=CN(C)N=C1Cl)C(=O)OC |c:25,t:8,10,12,15,21|	Z8873684883	**1**{*455*}	Nc1cc(Cl)cc(Cl)n1	**2**{*42*}	Cn1cc(C=O)c(Cl)n1	**3**{*23*}	COC(=O)CCC([N+]#[C-])C(=O)OC	23.2	18
1787	**4**{*379*,*792*,*14*}	COCCNC1=C(N=C2N1C=CC=C2Br)C1=NN(C)N=C1 |c:7,11,13,21,t:5,17|	Z8878918465	**1**{*379*}	Nc1ncccc1Br	**2**{*792*}	Cn1ncc(C=O)n1	**3**{*14*}	COCC[N+]#[C-]	17.1	18
1788	**4**{*103*,*455*,*5*}	COCCCNC1=C(N=C2C=NC=C(CO)N12)C1=C(C)ON=C1Br |c:10,19,23,t:6,8,12|	Z8873684822	**1**{*103*}	Nc1cncc(CO)n1	**2**{*455*}	Cc1onc(Br)c1C=O	**3**{*5*}	COCCC[N+]#[C-]	19.3	18
1789	**4**{*371*,*721*,*28*}	CCCNC1=C(N=C2N1C=CC=C2OCCCC#N)C1=CC(=CN=C1)C(=O)OC |c:6,10,12,23,25,t:4,21|	Z8878918541	**1**{*371*}	Nc1ncccc1OCCCC#N	**2**{*721*}	COC(=O)c1cncc(C=O)c1	**3**{*28*}	CCC[N+]#[C-]	19.1	18

1790	**4**{*186*,*746*,*69*}	COC1=C(NC2=C(N=C3C=C(C=CN23)S(=O)(=O)N(C)C)C2=C(C)C=CN=C2)C=CC=C1 |c:2,9,11,22,25,27,30,32,t:5,7|	Z8855619586	**1**{*186*}	Cl.CN(C)S(=O)(=O)c1ccnc(N)c1	**2**{*746*}	Cc1ccncc1C=O	**3**{*69*}	COc1ccccc1[N+]#[C-]	21.2	18
1791	**4**{*50*,*644*,*18*}	CC(C)(C)NC1=C(N=C2C=CC=C(N12)P(C)(C)=O)C1=CC(F)=C(OCC#C)C=C1 |c:9,11,29,t:5,7,20,23|	Z8878918524	**1**{*50*}	CP(=O)(C)c1cccc(N)n1	**2**{*644*}	Fc1cc(C=O)ccc1OCC#C	**3**{*18*}	CC(C)(C)[N+]#[C-]	20.0	18
1792	**4**{*32*,*793*,*31*}	CSCCNC1=C(N=C2C=C(C=CN12)P(C)(C)=O)C1=NC=C(S1)C(C)C |c:9,11,22,t:5,7,20|	Z8873684907	**1**{*32*}	CP(=O)(C)c1ccnc(N)c1	**2**{*793*}	CC(C)c1cnc(C=O)s1	**3**{*31*}	CSCC[N+]#[C-]	19.7	18
1793	**4**{*101*,*260*,*14*}	COCCNC1=C(N=C2N1C=C(CO)C=C2Cl)C1=NOC(C)=C1 |c:7,15,23,t:5,11,19|	Z8873684928	**1**{*101*}	Nc1ncc(CO)cc1Cl	**2**{*260*}	Cc1cc(C=O)no1	**3**{*14*}	COCC[N+]#[C-]	16.1	18
1794	**4**{*99*,*736*,*29*}	CCOC(=O)CCNC1=C(N=C2C=CC(=CN12)S(N)(=O)=O)C1CC(C)(C)C1 |c:12,14,t:8,10|	Z8878918655	**1**{*99*}	Nc1ccc(cn1)S(=O)(=O)N	**2**{*736*}	CC1(C)CC(C1)C=O	**3**{*29*}	CCOC(=O)CC[N+]#[C-]	18.8	18
1795	**4**{*137*,*69*,*13*}	COC1=CC=C(CNC2=C(N=C3C=CC(C)=NN23)C2=NN(C)C(C)=C2)C=C1 |c:12,15,25,28,t:2,4,8,10,20|	Z8873684693	**1**{*137*}	Cc1ccc(N)nn1	**2**{*69*}	Cc1cc(C=O)nn1C	**3**{*13*}	COc1ccc(C[N+]#[C-])cc1	17.0	17
1796	**4**{*36*,*27*,*9*}	CNC1=C(N=C2C=CC(Br)=CN12)C1=NN(C)C(C)=N1 |c:6,9,19,t:2,4,14|	Z8873685408	**1**{*36*}	Nc1ccc(Br)cn1	**2**{*27*}	Cc1nc(C=O)nn1C	**3**{*9*}	C[N+]#[C-]	15.1	17
1797	**4**{*101*,*727*,*14*}	COCCNC1=C(N=C2N1C=C(CO)C=C2Cl)C1=CN=C(N=C1)C(C)C |c:7,15,21,23,t:5,11,19|	Z8873684769	**1**{*101*}	Nc1ncc(CO)cc1Cl	**2**{*727*}	CC(C)c1ncc(C=O)cn1	**3**{*14*}	COCC[N+]#[C-]	17.4	17
1798	**4**{*386*,*419*,*23*}	COC(=O)CCC(NC1=C(N=C2C=C(C)C=CN12)C1=NC=CC(OC)=C1)C(=O)OC |c:15,22,26,t:8,10,12,20|	Z8854581171	**1**{*386*}	Cc1ccnc(N)c1	**2**{*419*}	COc1ccnc(C=O)c1	**3**{*23*}	COC(=O)CCC([N+]#[C-])C(=O)OC	18.7	17
1799	**4**{*171*,*763*,*22*}	COC(=O)C1=CC2=C(NN=C2C2=C(NC3COC3)N3C=CC(=CC3=N2)N2C=CN=C2)C=C1 |c:9,12,21,23,26,30,32,35,t:4,6|	Z8873685413	**1**{*171*}	Nc1cc(ccn1)n2ccnc2	**2**{*763*}	COC(=O)c1ccc2[nH]nc(C=O)c2c1	**3**{*22*}	[C-]#[N+]C1COC1	19.4	17
1800	**4**{*37*,*794*,*10*}	CC(C)(C)OC(=O)NCCC1=NC(=CS1)C1=C(NC2CCOC2)N2C(C=CC=C2C(F)F)=N1 |c:12,16,27,29,34,t:10|	Z8873684613	**1**{*37*}	Cl.Nc1cccc(n1)C(F)F	**2**{*794*}	CC(C)(C)OC(=O)NCCc1nc(C=O)cs1	**3**{*10*}	[C-]#[N+]C1CCOC1	21.4	17
1801	**4**{*103*,*795*,*5*}	COCCCNC1=C(N=C2C=NC=C(CO)N12)C1=C(Br)C=C(Cl)C=C1 |c:10,19,25,t:6,8,12,22|	Z8873685437	**1**{*103*}	Nc1cncc(CO)n1	**2**{*795*}	Clc1ccc(C=O)c(Br)c1	**3**{*5*}	COCCC[N+]#[C-]	18.9	16
1802	**4**{*184*,*189*,*26*}	NC(=S)C1=CN2C(NCC3=CC=C(F)C=C3)=C(N=C2C=C1)C1=CN(N=N1)C1CCOCC1 |c:14,16,18,21,27,t:3,9,11,24|	Z8878918818	**1**{*184*}	NC(=S)c1ccc(N)nc1	**2**{*189*}	O=Cc1cn(nn1)C2CCOCC2	**3**{*26*}	Fc1ccc(C[N+]#[C-])cc1	20.0	16

1803	**4**{*382*,*459*,*62*}	COC(=O)C1(CCCC1)NC1=C(N=C2C=NC(=CN12)C(F)(F)F)C1=NNC(C)=C1C |c:15,17,30,t:11,13,26|	Z8873684931	**1**{*382*}	Nc1cnc(cn1)C(F)(F)F	**2**{*459*}	Cc1[nH]nc(C=O)c1C	**3**{*62*}	COC(=O)C1(CCCC1)[N+]#[C-]	18.1	16
1804	**4**{*124*,*796*,*43*}	COC(=O)C1=C(F)C=C(C=N1)C1=C(NC2CCN(CC2)C(=O)OC(C)(C)C)N2C=CC=C(CO)C2=N1 |c:4,7,9,12,30,37,t:32|	Z8878918505	**1**{*124*}	Nc1ncccc1CO	**2**{*796*}	COC(=O)c1ncc(C=O)cc1F	**3**{*43*}	CC(C)(C)OC(=O)N1CCC(CC1)[N+]#[C-]	21.3	16
1805	**4**{*373*,*85*,*39*}	CCOCCNC1=C(N=C2C=CC(CNC(=O)OC(C)(C)C)=CN12)C1=CN=NS1 |c:10,21,28,t:6,8,26|	Z8878918358	**1**{*373*}	CC(C)(C)OC(=O)NCc1ccc(N)nc1	**2**{*85*}	O=Cc1cnns1	**3**{*39*}	CCOCC[N+]#[C-]	19.0	16
1806	**4**{*142*,*242*,*21*}	CS(=O)(=O)CCOC1=CC=CN2C(NCC3CC3)=C(N=C12)C1=CN2N=CC(C#N)=C2N=C1 |c:9,18,27,31,34,t:7,20,24|	Z8878918674	**1**{*142*}	CS(=O)(=O)CCOc1cccnc1N	**2**{*242*}	O=Cc1cnc2c(C#N)cnn2c1	**3**{*21*}	[C-]#[N+]CC1CC1	19.0	16
1807	**4**{*101*,*538*,*14*}	COCCNC1=C(N=C2N1C=C(CO)C=C2Cl)C1=CN=CN1C |c:7,15,21,t:5,11,19|	Z8878918498	**1**{*101*}	Nc1ncc(CO)cc1Cl	**2**{*538*}	Cn1cncc1C=O	**3**{*14*}	COCC[N+]#[C-]	14.1	16
1808	**4**{*375*,*375*,*51*}	COCC1=C(N=CO1)C1=C(NCCNC(=O)OC(C)(C)C)N2C(C=NC=C2OC(C)C)=N1 |c:5,9,24,26,32,t:3|	Z8855619770	**1**{*375*}	CC(C)Oc1cncc(N)n1	**2**{*375*}	COCc1ocnc1C=O	**3**{*51*}	CC(C)(C)OC(=O)NCC[N+]#[C-]	18.7	15
1809	**4**{*79*,*733*,*13*}	COC1=CC=C(CNC2=C(N=C3C=NC=C(C)N23)C2=NN(N=N2)C(C)(C)C)C=C1 |c:12,23,30,t:2,4,8,10,14,20|	Z8854581174	**1**{*79*}	Cc1cncc(N)n1	**2**{*733*}	CC(C)(C)n1nnc(C=O)n1	**3**{*13*}	COc1ccc(C[N+]#[C-])cc1	15.8	15
1810	**4**{*48*,*797*,*22*}	CS(=O)(=O)C1=CC2=NC(=C(NC3COC3)N2C=C1)C1=C(F)C=NC=C1 |c:18,21,24,26,t:4,6,8|	Z8878918421	**1**{*48*}	CS(=O)(=O)c1ccnc(N)c1	**2**{*797*}	Fc1cnccc1C=O	**3**{*22*}	[C-]#[N+]C1COC1	14.9	15
1811	**4**{*54*,*689*,*53*}	CN(C)C(=O)C1=CN2C(C=C1)=NC(C1=CN(CCOC(C)=O)N=C1)=C2NCCOCC1=CC=CC=C1 |c:9,11,23,25,35,37,t:5,14,33|	Z8878918851	**1**{*54*}	Cl.CN(C)C(=O)c1ccc(N)nc1	**2**{*689*}	CC(=O)OCCn1cc(C=O)cn1	**3**{*53*}	[C-]#[N+]CCOCc1ccccc1	19.5	15
1812	**4**{*464*,*271*,*70*}	CN1N=NC(=N1)C1=C(NC2CCN(C2)C(=O)OC(C)(C)C)N2C=C(C=CC2=N1)N1CCN(C)CC1 |c:2,4,7,24,26,29|	Z8837933135	**1**{*464*}	CN1CCN(CC1)c2ccc(N)nc2	**2**{*271*}	Cn1nnc(C=O)n1	**3**{*70*}	CC(C)(C)OC(=O)N1CCC(C1)[N+]#[C-]	18.9	15
1813	**4**{*433*,*340*,*31*}	CCN1C=NC(=N1)C1=C(NCCSC)N2C(C=CC(C#C)=C2C)=N1 |c:3,5,8,17,21,24|	Z8878918539	**1**{*433*}	Cc1nc(N)ccc1C#C	**2**{*340*}	CCn1cnc(C=O)n1	**3**{*31*}	CSCC[N+]#[C-]	13.3	14
1814	**4**{*78*,*798*,*51*}	CN1C=C(N=N1)C1=C(NCCNC(=O)OC(C)(C)C)N2N3C=C(C)N=C3C(C)=CC2=N1 |c:2,4,7,25,29,32,t:22|	Z8878918911	**1**{*78*}	Cc1cn2nc(N)cc(C)c2n1	**2**{*798*}	Cn1cc(C=O)nn1	**3**{*51*}	CC(C)(C)OC(=O)NCC[N+]#[C-]	16.6	14

1815	**4**{*340 ,725 ,5* }	CCOC1=CC=CN2C(NCCCOC)=C(N=C12)C1=CN=NN1CC |c:5,14,22,t:3,16,20|	Z8873685248	**1**{*340* }	CCOc1cccnc1N	**2**{*725* }	CCn1nncc1C=O	**3**{*5* }	COCCC[N+]#[C-]	13.3	14
1816	**4**{*185 ,799 ,8* }	COC(=O)C(CC1=CC=CC=C1)NC1=C(N=C2C=CC(=C(C)N12)S(C)(=O)=O)C1=NN=C(C)C=C1 |c:8,10,18,35,t:6,14,16,20,30,32|	Z8878918436	**1**{*185* }	Cc1nc(N)ccc1S(=O)(=O)C	**2**{*799* }	Cc1ccc(C=O)nn1	**3**{*8* }	COC(=O)C(Cc1ccccc1)[N+]#[C-]	18.4	14
1817	**4**{*154 ,800 ,47* }	CC(C)NC1=C(N=C2C=C(C=CN12)[N+]([O-])=O)C1=CC2=C(NC(=O)CO2)C=C1 |c:8,10,28,t:4,6,18,20|	Z8873684917	**1**{*154* }	Nc1cc(ccn1)[N+](=O)[O-]	**2**{*800* }	O=Cc1ccc2NC(=O)COc2c1	**3**{*47* }	CC(C)[N+]#[C-]	14.1	14
1818	**4**{*48 ,277 ,31* }	CSCCNC1=C(CC(NC(=O)OC(C)(C)C)C2CC2)N=C2C=C(C=CN12)S(C)(=O)=O |c:5,23,25,t:21|	Z8835022868	**1**{*48* }	CS(=O)(=O)c1ccnc(N)c1	**2**{*277* }	CC(C)(C)OC(=O)NC(CC=O)C1CC1	**3**{*31* }	CSCC[N+]#[C-]	17.9	14
1819	**4**{*462 ,801 ,39* }	CCOCCNC1=C(N=C2N1C=C(Br)C=C2CO)C1=CN=C(S1)C1=CN(C)N=C1 |c:8,15,22,30,t:6,12,20,26|	Z8855619696	**1**{*462* }	Nc1ncc(Br)cc1CO	**2**{*801* }	Cn1cc(cn1)c2ncc(C=O)s2	**3**{*39* }	CCOCC[N+]#[C-]	18.1	14
1820	**4**{*99 ,802 ,34* }	NS(=O)(=O)C1=CN2C(NCC3CCCO3)=C(N=C2C=C1)C1=CN2C=CN=C2S1 |c:15,17,20,26,28,t:4,23|	Z8878918406	**1**{*99* }	Nc1ccc(cn1)S(=O)(=O)N	**2**{*802* }	O=Cc1cn2ccnc2s1	**3**{*34* }	[C-]#[N+]CC1CCCO1	15.8	14
1821	**4**{*79 ,421 ,9* }	CNC1=C(N=C2C=NC=C(C)N12)C1=CN=C(S1)C1=CC=CO1 |c:6,16,22,t:2,4,8,14,20|	Z8873685441	**1**{*79* }	Cc1cncc(N)n1	**2**{*421* }	O=Cc1cnc(s1)c2ccco2	**3**{*9* }	C[N+]#[C-]	11.7	14
1822	**4**{*445 ,803 ,29* }	CCOC(=O)CCNC1=C(N=C2C=NC(SC)=CN12)C1=CC=C(C=C1)C1=NN=C(C)N=N1 |c:12,16,23,25,33,t:8,10,21,28,30|	Z8873684851	**1**{*445* }	CSc1cnc(N)cn1	**2**{*803* }	Cc1nnc(nn1)c2ccc(C=O)cc2	**3**{*29* }	CCOC(=O)CC[N+]#[C-]	16.7	14
1823	**4**{*414 ,804 ,46* }	CN1C=NC(Br)=C1C1=C(NCC(C)=C)N2C=C(Cl)N=CC2=N1 |c:2,5,8,19,22,t:16|	Z8873685477	**1**{*414* }	Nc1cnc(Cl)cn1	**2**{*804* }	Cn1cnc(Br)c1C=O	**3**{*46* }	CC(=C)C[N+]#[C-]	14.1	14
1824	**4**{*452 ,134 ,51* }	COC1=NC(=NC=C1)C1=C(NCCNC(=O)OC(C)(C)C)N2C(C=CC3=C2C=NC=C3)=N1 |c:4,6,9,24,26,29,31,33,t:2|	Z8878918681	**1**{*452* }	Nc1ccc2ccncc2n1	**2**{*134* }	COc1ccnc(C=O)n1	**3**{*51* }	CC(C)(C)OC(=O)NCC[N+]#[C-]	16.0	14
1825	**4**{*471 ,538 ,14* }	COCCNC1=C(N=C2N1C=C(C)C=C2I)C1=CN=CN1C |c:7,14,20,t:5,11,18|	Z8873684926	**1**{*471* }	Cc1cnc(N)c(I)c1	**2**{*538* }	Cn1cncc1C=O	**3**{*14* }	COCC[N+]#[C-]	15.1	14
1826	**4**{*154 ,805 ,47* }	CC(C)NC1=C(N=C2C=C(C=CN12)[N+]([O-])=O)C1=CC=C(C=C1)S(=O)(=O)N(C)C |c:8,10,20,22,t:4,6,18|	Z8878918643	**1**{*154* }	Nc1cc(ccn1)[N+](=O)[O-]	**2**{*805* }	CN(C)S(=O)(=O)c1ccc(C=O)cc1	**3**{*47* }	CC(C)[N+]#[C-]	14.7	14
1827	**4**{*176 ,538 ,14* }	COCCNC1=C(N=C2N1C=CN=C2N1CCCC1)C1=CN=CN1C |c:7,11,13,24,t:5,22|	Z8837933141	**1**{*176* }	Nc1nccnc1N2CCCC2	**2**{*538* }	Cn1cncc1C=O	**3**{*14* }	COCC[N+]#[C-]	12.4	13

1828	**4**{*91*,*806*,*7*}	NC(=O)C1=CN2C(C=C1)=NC(=C2NC1CCOCC1)C1=NC2=C(C=C1)C(F)=CC(F)=C2 |c:7,9,11,24,26,30,33,t:3,22|	Z8873684756	**1**{*91*}	NC(=O)c1ccc(N)nc1	**2**{*806*}	Fc1cc(F)c2ccc(C=O)nc2c1	**3**{*7*}	[C-]#[N+]C1CCOCC1	15.3	13
1829	**4**{*472*,*685*,*6*}	CCOC(=O)CCCNC1=C(N=C2N1C=C(C=C2C#N)C(C)C)C1=CN(CC2CCOC2)N=N1 |c:11,15,17,35,t:9,25|	Z8855619511	**1**{*472*}	CC(C)c1cnc(N)c(C#N)c1	**2**{*685*}	O=Cc1cn(CC2CCOC2)nn1	**3**{*6*}	CCOC(=O)CCC[N+]#[C-]	16.8	13
1830	**4**{*47*,*172*,*6*}	CCOC(=O)CCCNC1=C(N=C2C=C(C=CN12)S(N)(=O)=O)C1=CNC(=N1)C1CC1 |c:13,15,27,t:9,11,24|	Z8878918607	**1**{*47*}	Cl.Nc1cc(ccn1)S(=O)(=O)N	**2**{*172*}	O=Cc1c[nH]c(n1)C2CC2	**3**{*6*}	CCOC(=O)CCC[N+]#[C-]	15.5	13
1831	**4**{*137*,*94*,*20*}	COC1=CC(C)=C(NC2=C(N=C3C=CC(C)=NN23)C2=NC=CN=C2)C=C1 |c:12,15,22,24,27,t:2,5,8,10,20|	Z8873685256	**1**{*137*}	Cc1ccc(N)nn1	**2**{*94*}	O=Cc1cnccn1	**3**{*20*}	COc1ccc([N+]#[C-])c(C)c1	12.4	13
1832	**4**{*22*,*151*,*13*}	COC1=CC=C(CNC2=C(N=C3C=CC(Cl)=NN23)C2=CC(C)=NN2)C=C1 |c:12,15,23,27,t:2,4,8,10,20|	Z8878918393	**1**{*22*}	Nc1ccc(Cl)nn1	**2**{*151*}	Cc1cc(C=O)[nH]n1	**3**{*13*}	COc1ccc(C[N+]#[C-])cc1	12.9	13
1833	**4**{*390*,*189*,*22*}	CCC1=C(Cl)N2C(NC3COC3)=C(N=C2C=C1)C1=CN(N=N1)C1CCOCC1 |c:2,12,14,17,23,t:20|	Z8849597832	**1**{*390*}	CCc1ccc(N)nc1Cl	**2**{*189*}	O=Cc1cn(nn1)C2CCOCC2	**3**{*22*}	[C-]#[N+]C1COC1	14.1	13
1834	**4**{*354*,*704*,*62*}	COC(=O)C1=CN2C(C=C1)=NC(=C2NC1(CCCC1)C(=O)OC)C1=NC(=NN1C)C1CC1 |c:8,10,12,28,t:4,26|	Z8837933143	**1**{*354*}	COC(=O)c1ccc(N)nc1	**2**{*704*}	Cn1nc(nc1C=O)C2CC2	**3**{*62*}	COC(=O)C1(CCCC1)[N+]#[C-]	15.2	13
1835	**4**{*193*,*199*,*21*}	CNC(=O)C1=CN2C(C=C1)=NC(CN1C(=O)C3=C(C=CC=C3)C1=O)=C2NCC1CC1 |c:8,10,19,21,26,t:4,17|	Z8873684696	**1**{*193*}	CNC(=O)c1ccc(N)nc1	**2**{*199*}	O=CCN1C(=O)c2ccccc2C1=O	**3**{*21*}	[C-]#[N+]CC1CC1	13.7	13
1836	**4**{*118*,*347*,*35*}	CCCN1C=C(N=N1)C1=C(NCCCSC)N2C(C=CC=C2C(C)O)=N1 |c:4,6,9,19,21,26|	Z8873684940	**1**{*118*}	Cl.CC(O)c1cccc(N)n1	**2**{*347*}	CCCn1cc(C=O)nn1	**3**{*35*}	CSCCC[N+]#[C-]	12.6	12
1837	**4**{*391*,*425*,*47*}	CC(C)NC1=C(N=C2C=CC(=NN12)C1=C(F)C=CC=C1)C1CCCN(C1)S(C)(=O)=O |c:8,10,15,18,20,t:4,6|	Z8837933176	**1**{*391*}	Nc1ccc(nn1)c2ccccc2F	**2**{*425*}	CS(=O)(=O)N1CCCC(C1)C=O	**3**{*47*}	CC(C)[N+]#[C-]	14.5	12
1838	**4**{*119*,*647*,*10*}	N#CC1=CN2C(C=C1)=NC(=C2NC1CCOC1)C1=CC2=C(OC=C2)C=C1 |c:6,8,10,25,28,t:2,20,22|	Z8878918848	**1**{*119*}	Nc1ccc(C#N)cn1	**2**{*647*}	O=Cc1ccc2occc2c1	**3**{*10*}	[C-]#[N+]C1CCOC1	11.2	12
1839	**4**{*146*,*111*,*12*}	CS(=O)(=O)C1=CN2C(C=C1)=NC(C1CCCC1)=C2NCC1CCOC1 |c:8,10,18,t:4|	Z8878918852	**1**{*146*}	CS(=O)(=O)c1ccc(N)nc1	**2**{*111*}	O=CC1CCCC1	**3**{*12*}	[C-]#[N+]CC1CCOC1	11.4	12
1840	**4**{*32*,*659*,*18*}	CN1C=C2C(C=CC=C2C2=C(NC(C)(C)C)N3C=CC(=CC3=N2)P(C)(C)=O)=N1 |c:2,5,7,10,18,20,23,29|	Z8878918543	**1**{*32*}	CP(=O)(C)c1ccnc(N)c1	**2**{*659*}	Cn1cc2c(C=O)cccc2n1	**3**{*18*}	CC(C)(C)[N+]#[C-]	12.1	11

1841	**4**{*83*,*807*,*18*}	CC(C)(C)NC1=C(N=C2C=CC(=CN12)P(C)(C)=O)C1=C2N=CC=CC2=CC(F)=C1 |c:9,11,20,22,24,27,30,t:5,7|	Z8855739224	**1**{*83*}	CP(=O)(C)c1ccc(N)nc1	**2**{*807*}	Fc1cc(C=O)c2ncccc2c1	**3**{*18*}	CC(C)(C)[N+]#[C-]	12.5	11
1842	**4**{*46*,*170*,*9*}	CCN1N=NC(=N1)C1=C(NC)N2C=CC=CC2=N1 |c:3,5,8,13,15,18|	Z8873684867	**1**{*46*}	Nc1ccccn1	**2**{*170*}	CCn1nnc(C=O)n1	**3**{*9*}	C[N+]#[C-]	7.3	11
1843	**4**{*28*,*790*,*7*}	COC(=O)C1=C(F)C=C(C=C1F)C1=C(NC2CCOCC2)N2C=CC(=CC2=N1)C1=NOC=N1 |c:4,7,9,13,24,26,29,35,t:32|	Z8873684576	**1**{*28*}	Cl.Nc1cc(ccn1)c2ncon2	**2**{*790*}	COC(=O)c1c(F)cc(C=O)cc1F	**3**{*7*}	[C-]#[N+]C1CCOCC1	13.5	11
1844	**4**{*83*,*808*,*18*}	CC1=C(Cl)N=C(C2=C(NC(C)(C)C)N3C=C(C=CC3=N2)P(C)(C)=O)C(F)=C1 |c:1,6,14,16,19,27,t:4|	Z8855619564	**1**{*83*}	CP(=O)(C)c1ccc(N)nc1	**2**{*808*}	Cc1cc(F)c(C=O)nc1Cl	**3**{*18*}	CC(C)(C)[N+]#[C-]	11.8	11
1845	**4**{*473*,*809*,*15*}	CCNC1=C(N=C2C=CC=C(N12)C(=O)OCC)C1=CN=C(S1)C(=O)OCC |c:7,9,21,t:3,5,19|	Z8854581170	**1**{*473*}	Cl.CCOC(=O)c1cccc(N)n1	**2**{*809*}	CCOC(=O)c1ncc(C=O)s1	**3**{*15*}	CC[N+]#[C-]	11.1	11
1846	**4**{*22*,*44*,*13*}	CCN1C=C(C=N1)C1=C(NCC2=CC=C(OC)C=C2)N2N=C(Cl)C=CC2=N1 |c:3,5,8,18,25,28,t:12,14,22|	Z8878918382	**1**{*22*}	Nc1ccc(Cl)nn1	**2**{*44*}	CCn1cc(C=O)cn1	**3**{*13*}	COc1ccc(C[N+]#[C-])cc1	10.9	11
1847	**4**{*141*,*234*,*32*}	CC(=O)NC1=CC2=NC(=C(NC3CCC3)N2C=C1)C1=NN=C2CCCCCN12 |c:18,t:4,6,8,21,23|	Z8873684816	**1**{*141*}	CC(=O)Nc1ccnc(N)c1	**2**{*234*}	O=Cc1nnc2CCCCCn12	**3**{*32*}	[C-]#[N+]C1CCC1	10.7	10
1848	**4**{*394*,*328*,*12*}	CCC1=NNC=C1C1=C(NCC2CCOC2)N2C(C=CC(C=C)=C2F)=N1 |c:5,8,20,24,27,t:2|	Z8873685500	**1**{*394*}	Nc1ccc(C=C)c(F)n1	**2**{*328*}	Cl.CCc1n[nH]cc1C=O	**3**{*12*}	[C-]#[N+]CC1CCOC1	10.0	10
1849	**4**{*474*,*66*,*51*}	CCN1N=C(C)C(=C1C)C1=C(NCCNC(=O)OC(C)(C)C)N2N=C(Cl)C=C(OC)C2=N1 |c:6,10,32,t:3,24,27|	Z8829498507	**1**{*474*}	COc1cc(Cl)nnc1N	**2**{*66*}	CCn1nc(C)c(C=O)c1C	**3**{*51*}	CC(C)(C)OC(=O)NCC[N+]#[C-]	13.0	10
1850	**4**{*113*,*810*,*14*}	COCCNC1=C(N=C2N1C=CC=C2N1CCOCC1)C1=CN=C(NC(=O)OC(C)(C)C)S1 |c:7,11,13,t:5,23,25|	Z8878918584	**1**{*113*}	Nc1ncccc1N2CCOCC2	**2**{*810*}	CC(C)(C)OC(=O)Nc1ncc(C=O)s1	**3**{*14*}	COCC[N+]#[C-]	13.0	10
1851	**4**{*176*,*529*,*14*}	COCCNC1=C(N=C2N1C=CN=C2N1CCCC1)C1=CC(Cl)=C(C=C1)S(N)(=O)=O |c:7,11,13,25,27,t:5,22|	Z8878918347	**1**{*176*}	Nc1nccnc1N2CCCC2	**2**{*529*}	NS(=O)(=O)c1ccc(C=O)cc1Cl	**3**{*14*}	COCC[N+]#[C-]	12.3	10
1852	**4**{*78*,*811*,*10*}	CC1=CN2N3C(C=C(C)C2=N1)=NC(=C3NC1CCOC1)C1=CC=C(C=C1)C1=CC=CC=C1 |c:10,12,14,26,28,33,35,t:1,6,24,31|	Z8855619702	**1**{*78*}	Cc1cn2nc(N)cc(C)c2n1	**2**{*811*}	O=Cc1ccc(cc1)c2ccccc2	**3**{*10*}	[C-]#[N+]C1CCOC1	11.5	10
1853	**4**{*78*,*812*,*39*}	CCOCCNC1=C(N=C2C=C(C)C3=NC(C)=CN3N12)C1=CC2=C(O1)C=C(C=C2)C(=O)OC |c:16,25,29,31,t:6,8,10,13,23|	Z8855739169	**1**{*78*}	Cc1cn2nc(N)cc(C)c2n1	**2**{*812*}	COC(=O)c1ccc2cc(C=O)oc2c1	**3**{*39*}	CCOCC[N+]#[C-]	11.9	10

1854	**4**{*141*,*545*,*22*}	COCCN1N=C(C)C(=C1C)C1=C(NC2COC2)N2C=CC(NC(C)=O)=CC2=N1 |c:8,12,21,27,30,t:5|	Z8873684905	**1**{*141*}	CC(=O)Nc1ccnc(N)c1	**2**{*545*}	COCCn1nc(C)c(C=O)c1C	**3**{*22*}	[C-]#[N+]C1COC1	10.4	10
1855	**4**{*164*,*292*,*21*}	CN(C)CC1=CC2=NC(=C(NCC3CC3)N2C=C1)C1=C(C)N=CS1 |c:18,21,24,t:4,6,8|	Z8878918341	**1**{*164*}	CN(C)Cc1ccnc(N)c1	**2**{*292*}	Cc1ncsc1C=O	**3**{*21*}	[C-]#[N+]CC1CC1	8.9	10
1856	**4**{*48*,*813*,*22*}	CS(=O)(=O)C1=CC2=NC(=C(NC3COC3)N2C=C1)C1=C(Cl)C(F)=CC=C1F |c:18,21,25,27,t:4,6,8|	Z8878918903	**1**{*48*}	CS(=O)(=O)c1ccnc(N)c1	**2**{*813*}	Fc1ccc(F)c(C=O)c1Cl	**3**{*22*}	[C-]#[N+]C1COC1	11.2	10
1857	**4**{*188*,*687*,*62*}	COC(=O)C1=CN=CC2=NC(=C(NC3(CCCC3)C(=O)OC)N12)C1=C2CSCCC2=NN1C |c:6,26,33,t:4,8,10|	Z8873684948	**1**{*188*}	COC(=O)c1cncc(N)n1	**2**{*687*}	Cn1nc2CCSCc2c1C=O	**3**{*62*}	COC(=O)C1(CCCC1)[N+]#[C-]	11.9	9
1858	**4**{*43*,*814*,*39*}	CCOCCNC1=C(CCCC(=O)OC)N=C2C=CC3=C(CN(C3)C(=O)OC(C)(C)C)N12 |c:6,17,t:15,19|	Z8873684823	**1**{*43*}	CC(C)(C)OC(=O)N1Cc2ccc(N)nc2C1	**2**{*814*}	COC(=O)CCCC=O	**3**{*39*}	CCOCC[N+]#[C-]	11.2	9
1859	**4**{*112*,*815*,*12*}	COC1=NC(Cl)=C(S1)C1=C(NCC2CCOC2)N2C(C=NC=C2OC)=N1 |c:5,9,21,23,27,t:2|	Z8846492110	**1**{*112*}	COc1cncc(N)n1	**2**{*815*}	COc1nc(Cl)c(C=O)s1	**3**{*12*}	[C-]#[N+]CC1CCOC1	9.9	9
1860	**4**{*161*,*593*,*14*}	COCCNC1=C(N=C2C=CC=NN12)C1=CC=C(NC(C)=O)C=C1 |c:9,11,24,t:5,7,16,18|	Z8854581137	**1**{*161*}	Nc1cccnn1	**2**{*593*}	CC(=O)Nc1ccc(C=O)cc1	**3**{*14*}	COCC[N+]#[C-]	8.0	9
1861	**4**{*54*,*168*,*22*}	CN(C)C(=O)C1=CN2C(NC3COC3)=C(N=C2C=C1)C1=NOC=C1 |c:14,16,19,25,t:5,22|	Z8829498572	**1**{*54*}	Cl.CN(C)C(=O)c1ccc(N)nc1	**2**{*168*}	O=Cc1ccon1	**3**{*22*}	[C-]#[N+]C1COC1	8.0	9
1862	**4**{*146*,*816*,*46*}	COC1=C(OCCCC#N)C=C(C=C1)C1=C(NCC(C)=C)N2C=C(C=CC2=N1)S(C)(=O)=O |c:2,10,12,15,23,25,28|	Z8878918537	**1**{*146*}	CS(=O)(=O)c1ccc(N)nc1	**2**{*816*}	COc1ccc(C=O)cc1OCCCC#N	**3**{*46*}	CC(=C)C[N+]#[C-]	11.0	9
1863	**4**{*79*,*239*,*9*}	CNC1=C(N=C2C=NC=C(C)N12)C1=NOC2=C1CCCC2 |c:6,17,t:2,4,8,14|	Z8873685460	**1**{*79*}	Cc1cncc(N)n1	**2**{*239*}	O=Cc1noc2CCCCc12	**3**{*9*}	C[N+]#[C-]	6.8	9
1864	**4**{*119*,*70*,*10*}	CC(F)(F)C1=NC(=CS1)C1=C(NC2CCOC2)N2C=C(C=CC2=N1)C#N |c:6,10,20,22,25,t:4|	Z8855619533	**1**{*119*}	Nc1ccc(C#N)cn1	**2**{*70*}	CC(F)(F)c1nc(C=O)cs1	**3**{*10*}	[C-]#[N+]C1CCOC1	8.4	8
1865	**4**{*99*,*349*,*29*}	CCOC(=O)CCNC1=C(N=C2C=CC(=CN12)S(N)(=O)=O)C1C[C@H]2OC(C)(C)O[C@H]2C1 |c:12,14,t:8,10|	Z8878918664	**1**{*99*}	Nc1ccc(cn1)S(=O)(=O)N	**2**{*349*}	CC1(C)O[C@@H]2CC(C[C@@H]2O1)C=O	**3**{*29*}	CCOC(=O)CC[N+]#[C-]	10.7	8

1866	**4**{*81*,*58*,*13*}	COC1=CC=C(CNC2=C(N=C3C=NC=CN23)C2=NSN=C2)C=C1 |c:12,14,22,25,t:2,4,8,10,19|	Z8829498707	**1**{*81*}	Nc1cnccn1	**2**{*58*}	O=Cc1cnsn1	**3**{*13*}	COc1ccc(C[N+]#[C-])cc1	7.4	8
1867	**4**{*22*,*106*,*13*}	COC1=CC=C(CNC2=C(N=C3C=CC(Cl)=NN23)C2=C(OC)N=CC=N2)C=C1 |c:12,15,20,24,26,29,t:2,4,8,10|	Z8873684759	**1**{*22*}	Nc1ccc(Cl)nn1	**2**{*106*}	COc1nccnc1C=O	**3**{*13*}	COc1ccc(C[N+]#[C-])cc1	8.6	8
1868	**4**{*475*,*212*,*22*}	CC1=C(C=CC2=NC(=C(NC3COC3)N12)C1=NOC(=C1)C(F)F)C(F)(F)F |c:3,21,t:1,5,7,18|	Z8873685492	**1**{*475*}	Cc1nc(N)ccc1C(F)(F)F	**2**{*212*}	FC(F)c1cc(C=O)no1	**3**{*22*}	[C-]#[N+]C1COC1	8.4	8
1869	**4**{*378*,*427*,*28*}	CCCNC1=C(N=C2N1C=CC=C2OCC1=CN=CC=C1)C1=NOC2=C1COCC2 |c:6,10,12,19,21,27,t:4,17,24|	Z8855739093	**1**{*378*}	Nc1ncccc1OCc2cccnc2	**2**{*427*}	O=Cc1noc2CCOCc12	**3**{*28*}	CCC[N+]#[C-]	8.6	8
1870	**4**{*83*,*102*,*31*}	CSCCNC1=C(N=C2C=CC(=CN12)P(C)(C)=O)C1=C(Br)C(C)=CC(Cl)=N1 |c:9,11,20,24,27,t:5,7|	Z8878918762	**1**{*83*}	CP(=O)(C)c1ccc(N)nc1	**2**{*102*}	Cc1cc(Cl)nc(C=O)c1Br	**3**{*31*}	CSCC[N+]#[C-]	10.3	8
1871	**4**{*376*,*134*,*12*}	COC1=NC(=NC=C1)C1=C(NCC2CCOC2)N2C=C(Cl)N=C(OC)C2=N1 |c:4,6,9,28,t:2,20,23|	Z8873685405	**1**{*376*}	COc1nc(Cl)cnc1N	**2**{*134*}	COc1ccnc(C=O)n1	**3**{*12*}	[C-]#[N+]CC1CCOC1	8.2	8
1872	**4**{*50*,*817*,*18*}	CC(C)(C)NC1=C(N=C2C=CC=C(N12)P(C)(C)=O)C1=C(Br)C=CC(OC(F)F)=C1 |c:9,11,20,23,29,t:5,7|	Z8873684848	**1**{*50*}	CP(=O)(C)c1cccc(N)n1	**2**{*817*}	FC(F)Oc1ccc(Br)c(C=O)c1	**3**{*18*}	CC(C)(C)[N+]#[C-]	10.1	8
1873	**4**{*354*,*818*,*39*}	CCOCCNC1=C(N=C2C=CC(=CN12)C(=O)OC)C1=C(F)C(F)=NC=C1 |c:10,12,21,25,27,t:6,8|	Z8855619720	**1**{*354*}	COC(=O)c1ccc(N)nc1	**2**{*818*}	Fc1nccc(C=O)c1F	**3**{*39*}	CCOCC[N+]#[C-]	7.8	8
1874	**4**{*331*,*819*,*49*}	CN(C)CCN(C)CC1=CN2C(C=C1)=NC(=C2NC1CC(F)(F)C1)C1=NN(C)C=C1Br |c:12,14,16,31,t:8,27|	Z8878918459	**1**{*331*}	Cl.Cl.Cl.CN(C)CCN(C)Cc1ccc(N)nc1	**2**{*819*}	Cn1cc(Br)c(C=O)n1	**3**{*49*}	FC1(F)CC(C1)[N+]#[C-]	10.3	8
1875	**4**{*73*,*166*,*20*}	COC1=CC(C)=C(NC2=C(N=C3C=CC4=NC=CN4N23)C2=NC(=CS2)C2CC2)C=C1 |c:12,16,25,33,t:2,5,8,10,14,23|	Z8878918637	**1**{*73*}	Nc1ccc2nccn2n1	**2**{*166*}	O=Cc1nc(cs1)C2CC2	**3**{*20*}	COc1ccc([N+]#[C-])c(C)c1	8.4	7
1876	**4**{*109*,*686*,*50*}	CCNC(=O)C1=CC=C(C=C1)C1=C(NC2=CC3=C(OCCO3)C=C2)N2C(C=CC=C2S(N)(=O)=O)=N1 |c:7,9,12,24,29,31,37,t:5,15,17|	Z8873684650	**1**{*109*}	Cl.Nc1cccc(n1)S(=O)(=O)N	**2**{*686*}	CCNC(=O)c1ccc(C=O)cc1	**3**{*50*}	[C-]#[N+]c1ccc2OCCOc2c1	9.4	7
1877	**4**{*79*,*262*,*9*}	CCC1=NN(C)C(=C1Cl)C1=C(NC)N2C(C=NC=C2C)=N1 |c:6,10,16,18,21,t:2|	Z8837933139	**1**{*79*}	Cc1cncc(N)n1	**2**{*262*}	CCc1nn(C)c(C=O)c1Cl	**3**{*9*}	C[N+]#[C-]	5.8	7

1878	**4**{*400*,*820*,*22*}	CCN(CC)CCC1=NC(=CS1)C1=C(NC2COC2)N2C=C(COC(C)C)C=CC2=N1 |c:9,13,29,32,t:7,22|	Z8878918616	**1**{*400*}	CC(C)OCc1ccc(N)nc1	**2**{*820*}	CCN(CC)CCc1nc(C=O)cs1	**3**{*22*}	[C-]#[N+]C1COC1	7.9	7
1879	**4**{*87*,*821*,*23*}	COC(=O)CCC(NC1=C(N=C2C=C(C)C(Cl)=CN12)C1=CN=C(N=C1)C1CC1)C(=O)OC |c:16,23,25,t:8,10,12,21|	Z8873684681	**1**{*87*}	Cc1cc(N)ncc1Cl	**2**{*821*}	O=Cc1cnc(nc1)C2CC2	**3**{*23*}	COC(=O)CCC([N+]#[C-])C(=O)OC	8.5	7
1880	**4**{*161*,*137*,*20*}	COC1=CC(C)=C(NC2=C(N=C3C=CC=NN23)C2=C(C)ON=C2C)C=C1 |c:12,14,19,23,27,t:2,5,8,10|	Z8878918648	**1**{*161*}	Nc1cccnn1	**2**{*137*}	Cc1noc(C)c1C=O	**3**{*20*}	COc1ccc([N+]#[C-])c(C)c1	6.3	7
1881	**4**{*389*,*822*,*23*}	COC(=O)CCC(NC1=C(N=C2N1C=C(C)C=C2C)C1=NN(CC(F)(F)F)N=C1)C(=O)OC |c:10,17,29,t:8,14,21|	Z8878918365	**1**{*389*}	Cc1cnc(N)c(C)c1	**2**{*822*}	FC(F)(F)Cn1ncc(C=O)n1	**3**{*23*}	COC(=O)CCC([N+]#[C-])C(=O)OC	8.1	6
1882	**4**{*85*,*823*,*35*}	CSCCCNC1=C(N=C2C=C(C=C(C)N12)C#N)C1=NN(C)C2=C1C=CC=N2 |c:10,24,27,29,t:6,8,12,20|	Z8873684904	**1**{*85*}	Cc1cc(C#N)cc(N)n1	**2**{*823*}	Cn1nc(C=O)c2cccnc12	**3**{*35*}	CSCCC[N+]#[C-]	6.7	6
1883	**4**{*375*,*242*,*21*}	CC(C)OC1=CN=CC2=NC(=C(NCC3CC3)N12)C1=CN2N=CC(C#N)=C2N=C1 |c:6,24,28,31,t:4,8,10,21|	Z8873684892	**1**{*375*}	CC(C)Oc1cncc(N)n1	**2**{*242*}	O=Cc1cnc2c(C#N)cnn2c1	**3**{*21*}	[C-]#[N+]CC1CC1	6.6	6
1884	**4**{*161*,*824*,*6*}	CCOC(=O)CCCNC1=C(N=C2C=CC=NN12)C1=NC(Br)=CC=C1 |c:13,15,23,25,t:9,11,20|	Z8873684923	**1**{*161*}	Nc1cccnn1	**2**{*824*}	Brc1cccc(C=O)n1	**3**{*6*}	CCOC(=O)CCC[N+]#[C-]	6.8	6
1885	**4**{*146*,*104*,*46*}	CC(=C)CNC1=C(N=C2C=CC(=CN12)S(C)(=O)=O)C1=NC(C)=CO1 |c:9,11,23,t:5,7,20|	Z8873684784	**1**{*146*}	CS(=O)(=O)c1ccc(N)nc1	**2**{*104*}	Cc1coc(C=O)n1	**3**{*46*}	CC(=C)C[N+]#[C-]	5.8	6
1886	**4**{*50*,*825*,*18*}	CC(C)C1=NC=C(N1C)C1=C(NC(C)(C)C)N2C(C=CC=C2P(C)(C)=O)=N1 |c:5,10,19,21,27,t:3|	Z8878918793	**1**{*50*}	CP(=O)(C)c1cccc(N)n1	**2**{*825*}	CC(C)c1ncc(C=O)n1C	**3**{*18*}	CC(C)(C)[N+]#[C-]	6.4	6
1887	**4**{*46*,*274*,*9*}	CNC1=C(CC2(CCOCC2)C#N)N=C2C=CC=CN12 |c:2,16,18,t:14|	Z8878918469	**1**{*46*}	Nc1ccccn1	**2**{*274*}	O=CCC1(CCOCC1)C#N	**3**{*9*}	C[N+]#[C-]	4.5	6
1888	**4**{*395*,*101*,*7*}	CC1=CC(=NN2C(NC3CCOCC3)=C(N=C12)C1=C(CO)C=CS1)C(F)(F)F |c:3,14,20,24,t:1,16|	Z8878918535	**1**{*395*}	Cc1cc(nnc1N)C(F)(F)F	**2**{*101*}	OCc1ccsc1C=O	**3**{*7*}	[C-]#[N+]C1CCOCC1	6.6	6
1889	**4**{*79*,*826*,*9*}	CNC1=C(N=C2C=NC=C(C)N12)C1=NC(Cl)=CS1 |c:6,17,t:2,4,8,14|	Z8878918870	**1**{*79*}	Cc1cncc(N)n1	**2**{*826*}	Clc1csc(C=O)n1	**3**{*9*}	C[N+]#[C-]	4.5	6
1890	**4**{*54*,*827*,*37*}	CCN(CCO)C1=CC(C)=C(C=C1)C1=C(NC2CC(C2)C(=O)OC)N2C=C(C=CC2=N1)C(=O)N(C)C |c:9,11,14,27,29,32,t:6|	Z8854581161	**1**{*54*}	Cl.CN(C)C(=O)c1ccc(N)nc1	**2**{*827*}	CCN(CCO)c1ccc(C=O)c(C)c1	**3**{*37*}	COC(=O)C1CC(C1)[N+]#[C-]	7.8	6

1891	**4**{*91*,*828*,*26*}	CC(N1C(=O)C2=C(C=CC=C2)C1=O)C1=C(NCC2=CC=C(F)C=C2)N2C=C(C=CC2=N1)C(N)=O |c:7,9,15,24,28,30,33,t:5,19,21|	Z8878918519	**1**{*91*}	NC(=O)c1ccc(N)nc1	**2**{*828*}	CC(C=O)N1C(=O)c2ccccc2C1=O	**3**{*26*}	Fc1ccc(C[N+]#[C-])cc1	7.2	6
1892	**4**{*73*,*307*,*20*}	COC1=CC(C)=C(NC2=C(N=C3C=CC4=NC=CN4N23)C2=CN=C(Cl)N2C)C=C1 |c:12,16,31,t:2,5,8,10,14,23,25|	Z8855739267	**1**{*73*}	Nc1ccc2nccn2n1	**2**{*307*}	Cn1c(Cl)ncc1C=O	**3**{*20*}	COc1ccc([N+]#[C-])c(C)c1	6.0	5
1893	**4**{*73*,*829*,*20*}	COC1=CC(C)=C(NC2=C(N=C3C=CC4=NC=CN4N23)C2=CC(Cl)=C(OC)N=C2)C=C1 |c:12,16,30,33,t:2,5,8,10,14,23,26|	Z8878918518	**1**{*73*}	Nc1ccc2nccn2n1	**2**{*829*}	COc1ncc(C=O)cc1Cl	**3**{*20*}	COc1ccc([N+]#[C-])c(C)c1	6.4	5
1894	**4**{*331*,*741*,*49*}	CN(C)CCN(C)CC1=CN2C(C=C1)=NC(=C2NC1CC(F)(F)C1)C1=CC=C(C=C1)N1C=NC=N1 |c:12,14,16,29,31,35,37,t:8,27|	Z8873684908	**1**{*331*}	Cl.Cl.Cl.CN(C)CCN(C)Cc1ccc(N)nc1	**2**{*741*}	O=Cc1ccc(cc1)n2cncn2	**3**{*49*}	FC1(F)CC(C1)[N+]#[C-]	7.0	5
1895	**4**{*141*,*209*,*32*}	CC(=O)NC1=CC2=NC(=C(NC3CCC3)N2C=C1)C1=NN=C2CCCCN12 |c:18,t:4,6,8,21,23|	Z8873684831	**1**{*141*}	CC(=O)Nc1ccnc(N)c1	**2**{*209*}	O=Cc1nnc2CCCCn12	**3**{*32*}	[C-]#[N+]C1CCC1	5.3	5
1896	**4**{*99*,*241*,*34*}	CN1N=CC(Br)=C1C1=C(NCC2CCCO2)N2C=C(C=CC2=N1)S(N)(=O)=O |c:2,5,8,19,21,24|	Z8829498789	**1**{*99*}	Nc1ccc(cn1)S(=O)(=O)N	**2**{*241*}	Cn1ncc(Br)c1C=O	**3**{*34*}	[C-]#[N+]CC1CCCO1	6.2	5
1897	**4**{*137*,*94*,*13*}	COC1=CC=C(CNC2=C(N=C3C=CC(C)=NN23)C2=NC=CN=C2)C=C1 |c:12,15,22,24,27,t:2,4,8,10,20|	Z8873685365	**1**{*137*}	Cc1ccc(N)nn1	**2**{*94*}	O=Cc1cnccn1	**3**{*13*}	COc1ccc(C[N+]#[C-])cc1	4.5	5
1898	**4**{*73*,*307*,*13*}	COC1=CC=C(CNC2=C(N=C3C=CC4=NC=CN4N23)C2=CN=C(Cl)N2C)C=C1 |c:12,16,31,t:2,4,8,10,14,23,25|	Z8837933161	**1**{*73*}	Nc1ccc2nccn2n1	**2**{*307*}	Cn1c(Cl)ncc1C=O	**3**{*13*}	COc1ccc(C[N+]#[C-])cc1	4.7	4
1899	**4**{*22*,*170*,*9*}	CCN1N=NC(=N1)C1=C(NC)N2N=C(Cl)C=CC2=N1 |c:3,5,8,16,19,t:13|	Z8846491739	**1**{*22*}	Nc1ccc(Cl)nn1	**2**{*170*}	CCn1nnc(C=O)n1	**3**{*9*}	C[N+]#[C-]	3.1	4
1900	**4**{*81*,*170*,*9*}	CCN1N=NC(=N1)C1=C(NC)N2C=CN=CC2=N1 |c:3,5,8,13,15,18|	Z8855739229	**1**{*81*}	Nc1cnccn1	**2**{*170*}	CCn1nnc(C=O)n1	**3**{*9*}	C[N+]#[C-]	2.7	4
1901	**4**{*389*,*572*,*23*}	COC(=O)CCC(NC1=C(N=C2N1C=C(C)C=C2C)C1=NC(=CC(OC)=C1)C(=O)OC)C(=O)OC |c:10,17,23,27,t:8,14,21|	Z8878918405	**1**{*389*}	Cc1cnc(N)c(C)c1	**2**{*572*}	COC(=O)c1cc(OC)cc(C=O)n1	**3**{*23*}	COC(=O)CCC([N+]#[C-])C(=O)OC	5.3	4
1902	**4**{*476*,*279*,*47*}	CC(C)NC1=C(N=C2C=CC=C(CC3=NC=CC=C3)N12)[C@@H]1CCCO[C@H]1C1=NC=CN1C |&1:20,25,r,c:8,15,17,32,t:4,6,10,13,30|	Z8873685372	**1**{*476*}	Nc1cccc(Cc2ccccn2)n1	**2**{*279*}	Cn1ccnc1[C@@H]2OCCC[C@H]2C=O |&1:6,11,r|	**3**{*47*}	CC(C)[N+]#[C-]	4.7	4

1903	**4**{*187*,*427*,*46*}	CC(=C)CNC1=C(N=C2C=C(C#N)C(Cl)=CN12)C1=NOC2=C1COCC2 |c:14,22,t:5,7,9,19|	Z8873684945	**1**{*187*}	Nc1cc(C#N)c(Cl)cn1	**2**{*427*}	O=Cc1noc2CCOCc12	**3**{*46*}	CC(=C)C[N+]#[C-]	3.9	4
1904	**4**{*161*,*116*,*13*}	COC1=CC=C(CNC2=C(N=C3C=CC=NN23)C2=CN(CCC=C)N=N2)C=C1 |c:12,14,26,29,t:2,4,8,10,19|	Z8837933164	**1**{*161*}	Nc1cccnn1	**2**{*116*}	C=CCCn1cc(C=O)nn1	**3**{*13*}	COc1ccc(C[N+]#[C-])cc1	3.7	4
1905	**4**{*380*,*830*,*5*}	COCCCNC1=C(N=C2N1C=C(Cl)N=C2C)C1=NC(=CC=C1)C(=O)OC |c:8,15,21,23,t:6,12,19|	Z8878918321	**1**{*380*}	Cc1nc(Cl)cnc1N	**2**{*830*}	COC(=O)c1cccc(C=O)n1	**3**{*5*}	COCCC[N+]#[C-]	3.4	3
1906	**4**{*78*,*85*,*74*}	CC1=CN2N3C(NC4CCCN(C4)C(=O)OC(C)(C)C)=C(N=C3C=C(C)C2=N1)C1=CN=NS1 |c:20,22,29,34,t:1,25,32|	Z8873684642	**1**{*78*}	Cc1cn2nc(N)cc(C)c2n1	**2**{*85*}	O=Cc1cnns1	**3**{*74*}	CC(C)(C)OC(=O)N1CCC[C@H](C1)[N+]#[C-]	4.0	3
1907	**4**{*477*,*235*,*23*}	COC(=O)CCC(NC1=C(N=C2C=C(F)C(Br)=CN12)C1=CSC(=C1)C#N)C(=O)OC |c:16,24,t:8,10,12,21|	Z8878918562	**1**{*477*}	Nc1cc(F)c(Br)cn1	**2**{*235*}	O=Cc1csc(C#N)c1	**3**{*23*}	COC(=O)CCC([N+]#[C-])C(=O)OC	4.0	3
1908	**4**{*79*,*485*,*9*}	CNC1=C(N=C2C=NC=C(C)N12)C1=NOC2=C1CCC2 |c:6,17,t:2,4,8,14|	Z8873685257	**1**{*79*}	Cc1cncc(N)n1	**2**{*485*}	O=Cc1noc2CCCc12	**3**{*9*}	C[N+]#[C-]	2.0	3
1909	**4**{*73*,*137*,*20*}	COC1=CC(C)=C(NC2=C(N=C3C=CC4=NC=CN4N23)C2=C(C)ON=C2C)C=C1 |c:12,16,23,27,31,t:2,5,8,10,14|	Z8878918340	**1**{*73*}	Nc1ccc2nccn2n1	**2**{*137*}	Cc1noc(C)c1C=O	**3**{*20*}	COc1ccc([N+]#[C-])c(C)c1	2.8	3
1910	**4**{*87*,*235*,*23*}	COC(=O)CCC(NC1=C(N=C2C=C(C)C(Cl)=CN12)C1=CSC(=C1)C#N)C(=O)OC |c:16,24,t:8,10,12,21|	Z8873685450	**1**{*87*}	Cc1cc(N)ncc1Cl	**2**{*235*}	O=Cc1csc(C#N)c1	**3**{*23*}	COC(=O)CCC([N+]#[C-])C(=O)OC	3.2	3
1911	**4**{*164*,*831*,*10*}	CN(C)CC1=CC2=NC(C3=CN=C(S3)C3CN(C3)C(=O)OC(C)(C)C)=C(NC3CCOC3)N2C=C1 |c:11,37,t:4,6,9,26|	Z8878918533	**1**{*164*}	CN(C)Cc1ccnc(N)c1	**2**{*831*}	CC(C)(C)OC(=O)N1CC(C1)c2ncc(C=O)s2	**3**{*10*}	[C-]#[N+]C1CCOC1	3.4	3
1912	**4**{*209*,*260*,*14*}	COCCNC1=C(N=C2N1C=CC=C2N1CCCCC1)C1=NOC(C)=C1 |c:7,11,13,27,t:5,23|	Z8878918510	**1**{*209*}	Nc1ncccc1N2CCCCC2	**2**{*260*}	Cc1cc(C=O)no1	**3**{*14*}	COCC[N+]#[C-]	2.4	2
1913	**4**{*369*,*101*,*7*}	OCC1=C(SC=C1)C1=C(NC2CCOCC2)N2C=C(C=CC2=N1)C(F)C(F)(F)F |c:5,8,19,21,24,t:2|	Z8873684604	**1**{*369*}	Cl.Nc1ccc(cn1)C(F)C(F)(F)F	**2**{*101*}	OCc1ccsc1C=O	**3**{*7*}	[C-]#[N+]C1CCOCC1	2.8	2
1914	**4**{*141*,*832*,*22*}	CC1OC2=C(NC1=O)C=C(C=C2)C1=C(NC2COC2)N2C=CC(NC(C)=O)=CC2=N1 |c:3,9,11,14,23,29,32|	Z8878918783	**1**{*141*}	CC(=O)Nc1ccnc(N)c1	**2**{*832*}	CC1Oc2ccc(C=O)cc2NC1=O	**3**{*22*}	[C-]#[N+]C1COC1	2.8	2

1915	**4**{*61*,*756*,*23*}	CCC1=CC=CC2=NC(C3=CC(=CO3)S(C)(=O)=O)=C(NC(CCC(=O)OC)C(=O)OC)N12 |c:4,11,t:2,6,9,18|	Z8873685377	**1**{*61*}	CCc1cccc(N)n1	**2**{*756*}	CS(=O)(=O)c1coc(C=O)c1	**3**{*23*}	COC(=O)CCC([N+]#[C-])C(=O)OC	2.8	2
1916	**4**{*478*,*769*,*21*}	CN1N=CN=C1C1=C(NCC2CC2)N2C(C=C(Br)C=C2C)=N1 |c:2,4,7,20,23,t:17|	Z8873684620	**1**{*478*}	Cc1cc(Br)cc(N)n1	**2**{*769*}	Cn1ncnc1C=O	**3**{*21*}	[C-]#[N+]CC1CC1	2.0	2
1917	**4**{*479*,*408*,*47*}	CC(C)NC1=C(CCN2CCOC2=O)N=C2C=CC(Cl)=C(Br)N12 |c:4,17,t:15,20|	Z8878918400	**1**{*479*}	Nc1ccc(Cl)c(Br)n1	**2**{*408*}	O=CCCN1CCOC1=O	**3**{*47*}	CC(C)[N+]#[C-]	1.8	2
1918	**4**{*113*,*260*,*6*}	CCOC(=O)CCCNC1=C(N=C2N1C=CC=C2N1CCOCC1)C1=NOC(C)=C1 |c:11,15,17,31,t:9,27|	Z8873685448	**1**{*113*}	Nc1ncccc1N2CCOCC2	**2**{*260*}	Cc1cc(C=O)no1	**3**{*6*}	CCOC(=O)CCC[N+]#[C-]	1.8	2
1919	**4**{*395*,*439*,*7*}	COC1=C(C=CC(=C1)C1=C(NC2CCOCC2)N2N=C(C=C(C)C2=N1)C(F)(F)F)S(C)(=O)=O |c:4,6,9,20,26,t:2,22|	Z8878918392	**1**{*395*}	Cc1cc(nnc1N)C(F)(F)F	**2**{*439*}	COc1cc(C=O)ccc1S(=O)(=O)C	**3**{*7*}	[C-]#[N+]C1CCOCC1	2.0	2
1920	**4**{*187*,*413*,*12*}	ClC1=CN2C(NCC3CCOC3)=C(N=C2C=C1C#N)C1=CN=C(S1)C1=CC=NC=C1 |c:12,14,17,24,30,32,t:1,22,28|	Z8878918513	**1**{*187*}	Nc1cc(C#N)c(Cl)cn1	**2**{*413*}	O=Cc1cnc(s1)c2ccncc2	**3**{*12*}	[C-]#[N+]CC1COC1	1.6	1
1921	**4**{*327*,*833*,*34*}	COC(=O)C1=C(F)C2=NC(C3=CC=C(S3)C#N)=C(NCC3CCCO3)N2C=C1 |c:4,12,29,t:7,10,17|	Z8878918899	**1**{*327*}	COC(=O)c1ccnc(N)c1F	**2**{*833*}	O=Cc1ccc(C#N)s1	**3**{*34*}	[C-]#[N+]CC1CCCO1	0.0	0
1922	**4**{*480*,*168*,*22*}	CC1=C(C)C(Cl)=NN2C(NC3COC3)=C(N=C12)C1=NOC=C1 |c:1,5,14,23,t:16,20|	Z8878918862	**1**{*480*}	Cc1c(C)c(Cl)nnc1N	**2**{*168*}	O=Cc1ccon1	**3**{*22*}	[C-]#[N+]C1COC1	0.0	0
1923	**4**{*91*,*472*,*18*}	COC(=O)C1=CC2=C(C=NN2C=C1)C1=C(NC(C)(C)C)N2C=C(C=CC2=N1)C(N)=O |c:8,12,15,23,25,28,t:4,6|	Z8878918837	**1**{*91*}	NC(=O)c1ccc(N)nc1	**2**{*472*}	COC(=O)c1ccn2ncc(C=O)c2c1	**3**{*18*}	CC(C)(C)[N+]#[C-]	0.0	0
1924	**4**{*421*,*488*,*37*}	COC(=O)C1CC(C1)NC1=C(N=C2C=CC(C(=O)OC)=C(Cl)N12)C1=CC2=C(NC(=O)CC2)N=C1 |c:14,36,t:10,12,20,26,28|	Z8878918610	**1**{*421*}	COC(=O)c1ccc(N)nc1Cl	**2**{*488*}	O=Cc1cnc2NC(=O)CCc2c1	**3**{*37*}	COC(=O)C1CC(C1)[N+]#[C-]	0.0	0
1925	**4**{*218*,*649*,*10*}	CN1N=C(C=C1C1=C(NC2CCOC2)N2N=C(C=CC2=N1)C(C)(C)C)C(F)F |c:2,4,7,17,19,22|	Z8878918586	**1**{*218*}	CC(C)(C)c1ccc(N)nn1	**2**{*649*}	Cn1nc(cc1C=O)C(F)F	**3**{*10*}	[C-]#[N+]C1CCOC1	0.0	0
1926	**4**{*94*,*21*,*60*}	COC(=O)C1=CC(NC2=C(N=C3C=CC(F)=NN23)C2=CC3=NC=NN3C=C2)=CC=C1 |c:12,15,24,28,30,32,t:4,8,10,20,22|	Z8878918540	**1**{*94*}	Nc1ccc(F)nn1	**2**{*21*}	O=Cc1ccn2ncnc2c1	**3**{*60*}	COC(=O)c1cccc([N+]#[C-])c1	0.0	0
1927	**4**{*123*,*289*,*22*}	COC1=C(OCC#C)C=C(C=C1)C1=C(NC2COC2)N2C=CC(OCCO)=CC2=N1 |c:2,8,10,13,22,28,31|	Z8878918484	**1**{*123*}	Cl.Nc1cc(OCCO)ccn1	**2**{*289*}	COc1ccc(C=O)cc1OCC#C	**3**{*22*}	[C-]#[N+]C1COC1	0.0	0

<table>
<tr><td>1928</td><td>4{457,119,22}</td><td>CCOC(=O)C1=NN=C(N1)C1=C(NC2COC2)N2C=C(Br)C(=CC2=N1)C(F)F |c:7,11,23,26,t:5,20|</td><td>Z8878918474</td><td>1{457}</td><td>Nc1cc(C(F)F)c(Br)cn1</td><td>2{119}</td><td>CCOC(=O)c1nnc(C=O)[nH]1</td><td>3{22}</td><td>[C-]#[N+]C1COC1</td><td>0.0</td><td>0</td></tr>
<tr><td>1929</td><td>4{267,834,5}</td><td>COCCCNC1=C(CC2CCC(C)(C)O2)N=C2C=CC=C(C#C)N12 |c:6,19,t:17,21|</td><td>Z8878918458</td><td>1{267}</td><td>Nc1cccc(C#C)n1</td><td>2{834}</td><td>CC1(C)CCC(CC=O)O1</td><td>3{5}</td><td>COCCC[N+]#[C-]</td><td>0.0</td><td>0</td></tr>
<tr><td>1930</td><td>4{185,799,69}</td><td>COC1=C(NC2=C(N=C3C=CC(=C(C)N23)S(C)(=O)=O)C2=NN=C(C)C=C2)C=CC=C1 |c:2,9,26,29,31,t:5,7,11,21,23|</td><td>Z8878918417</td><td>1{185}</td><td>Cc1nc(N)ccc1S(=O)(=O)C</td><td>2{799}</td><td>Cc1ccc(C=O)nn1</td><td>3{69}</td><td>COc1ccccc1[N+]#[C-]</td><td>0.0</td><td>0</td></tr>
<tr><td>1931</td><td>4{267,383,5}</td><td>COCCCNC1=C(N=C2C=CC=C(C#C)N12)C1=NC=C(C)C=C1 |c:10,24,t:6,8,12,19,21|</td><td>Z8878918371</td><td>1{267}</td><td>Nc1cccc(C#C)n1</td><td>2{383}</td><td>Cc1ccc(C=O)nc1</td><td>3{5}</td><td>COCCC[N+]#[C-]</td><td>0.0</td><td>0</td></tr>
<tr><td>1932</td><td>4{47,179,6}</td><td>CCOC(=O)CCCNC1=C(N=C2C=C(C=CN12)S(N)(=O)=O)C1=CC=C(OC2CCOC2)C=C1 |c:13,15,35,t:9,11,24,26|</td><td>Z8873684752</td><td>1{47}</td><td>Cl.Nc1cc(ccn1)S(=O)(=O)N</td><td>2{179}</td><td>O=Cc1ccc(OC2CCOC2)cc1</td><td>3{6}</td><td>CCOC(=O)CCC[N+]#[C-]</td><td>0.0</td><td>0</td></tr>
<tr><td>1933</td><td>4{28,189,26}</td><td>FC1=CC=C(CNC2=C(N=C3C=C(C=CN23)C2=NOC=N2)C2=CN(N=N2)C2CCOCC2)C=C1 |c:11,13,21,27,37,t:1,3,7,9,18,24|</td><td>Z8873685403</td><td>1{28}</td><td>Cl.Nc1cc(ccn1)c2ncon2</td><td>2{189}</td><td>O=Cc1cn(nn1)C2CCOCC2</td><td>3{26}</td><td>Fc1ccc(C[N+]#[C-])cc1</td><td>0.0</td><td>0</td></tr>
<tr><td>1934</td><td>4{42,835,14}</td><td>COCCNC1=C(N=C2C=CC(SC)=CN12)C1=CSC(=N1)N(C(C)=O)C1=CC=CC=C1 |c:9,13,21,30,32,t:5,7,18,28|</td><td>Z8873685375</td><td>1{42}</td><td>CSc1ccc(N)nc1</td><td>2{835}</td><td>CC(=O)N(c1nc(C=O)cs1)c2ccccc2</td><td>3{14}</td><td>COCC[N+]#[C-]</td><td>0.0</td><td>0</td></tr>
<tr><td>1935</td><td>4{172,403,29}</td><td>CCOC(=O)CCNC1=C(N=C2N1C=CC1=C2CCO1)C1=CN=C(Cl)N1 |c:10,14,16,t:8,23,25|</td><td>Z8873684921</td><td>1{172}</td><td>Nc1nccc2OCCc21</td><td>2{403}</td><td>Clc1ncc(C=O)[nH]1</td><td>3{29}</td><td>CCOC(=O)CC[N+]#[C-]</td><td>0.0</td><td>0</td></tr>
<tr><td>1936</td><td>4{38,170,9}</td><td>CCN1N=NC(=N1)C1=C(NC)N2C=CC(=CC2=N1)C#N |c:3,5,8,13,15,18|</td><td>Z8873684882</td><td>1{38}</td><td>Nc1cc(C#N)ccn1</td><td>2{170}</td><td>CCn1nnc(C=O)n1</td><td>3{9}</td><td>C[N+]#[C-]</td><td>0.0</td><td>0</td></tr>
<tr><td>1937</td><td>4{184,28,26}</td><td>NC(=S)C1=CN2C(NCC3=CC=C(F)C=C3)=C(N=C2C=C1)C1=CN(N=C1)C1CCS(=O)(=O)C1 |c:14,16,18,21,27,t:3,9,11,24|</td><td>Z8873684871</td><td>1{184}</td><td>NC(=S)c1ccc(N)nc1</td><td>2{28}</td><td>O=Cc1cnn(c1)C2CCS(=O)(=O)C2</td><td>3{26}</td><td>Fc1ccc(C[N+]#[C-])cc1</td><td>0.0</td><td>0</td></tr>
<tr><td>1938</td><td>4{28,439,18}</td><td>COC1=C(C=CC(=C1)C1=C(NC(C)(C)C)N2C=CC(=CC2=N1)C1=NOC=N1)S(C)(=O)=O |c:4,6,9,17,19,22,28,t:2,25|</td><td>Z8873684857</td><td>1{28}</td><td>Cl.Nc1cc(ccn1)c2ncon2</td><td>2{439}</td><td>COc1cc(C=O)ccc1S(=O)(=O)C</td><td>3{18}</td><td>CC(C)(C)[N+]#[C-]</td><td>0.0</td><td>0</td></tr>
<tr><td>1939</td><td>4{73,98,20}</td><td>COC1=CC(C)=C(NC2=C(N=C3C=CC4=NC=CN4N23)C2=NC=C(OC)N=C2)C=C1 |c:12,16,29,32,t:2,5,8,10,14,23,25|</td><td>Z8873684847</td><td>1{73}</td><td>Nc1ccc2nccn2n1</td><td>2{98}</td><td>COc1cnc(C=O)cn1</td><td>3{20}</td><td>COc1ccc([N+]#[C-])c(C)c1</td><td>0.0</td><td>0</td></tr>
<tr><td>1940</td><td>4{209,377,14}</td><td>COCCNC1=C(N=C2N1C=CC=C2N1CCCCC1)C1=NNC=C1C |c:7,11,13,26,t:5,23|</td><td>Z8873684812</td><td>1{209}</td><td>Nc1ncccc1N2CCCCC2</td><td>2{377}</td><td>Cl.Cc1c[nH]nc1C=O</td><td>3{14}</td><td>COCC[N+]#[C-]</td><td>0.0</td><td>0</td></tr>
</table>

1941	**4**{*101*,*62*,*6*}	CCOC(=O)CCCNC1=C(N=C2N1C=C(CO)C=C2Cl)C1CC11CCOCC1 |c:11,19,t:9,15|	Z8829498700	**1**{*101*}	Nc1ncc(CO)cc1Cl	**2**{*62*}	O=CC1CC21CCOCC2	**3**{*6*}	CCOC(=O)CCC[N+]#[C-]	0.0	0
1942	**4**{*111*,*119*,*15*}	CCNC1=C(N=C2N1C=C(C=C2C)C(=O)OC)C1=NN=C(N1)C(=O)OCC |c:5,9,11,21,t:3,19|	Z8878918878	**1**{*111*}	COC(=O)c1cnc(N)c(C)c1	**2**{*119*}	CCOC(=O)c1nnc(C=O)[nH]1	**3**{*15*}	CC[N+]#[C-]	0.0	0
1943	**4**{*225*,*588*,*60*}	COC(=O)C1=CC(NC2=C(N=C3C=C(Cl)C=NN23)C2=CN(C)N=C2C)=CC=C1 |c:15,24,27,29,t:4,8,10,12,20|	Z8878918841	**1**{*225*}	Nc1cc(Cl)cnn1	**2**{*588*}	Cc1nn(C)cc1C=O	**3**{*60*}	COC(=O)c1cccc([N+]#[C-])c1	0.0	0
1944	**4**{*146*,*766*,*51*}	CC1CC1C1=C(NCCNC(=O)OC(C)(C)C)N2C=C(C=CC2=N1)S(C)(=O)=O |c:5,19,21,24|	Z8878918806	**1**{*146*}	CS(=O)(=O)c1ccc(N)nc1	**2**{*766*}	CC1CC1C=O	**3**{*51*}	CC(C)(C)OC(=O)NCC[N+]#[C-]	0.0	0
1945	**4**{*292*,*212*,*35*}	CCOC1=CN=CC2=NC(=C(NCCCSC)N12)C1=NOC(=C1)C(F)F |c:5,23,t:3,7,9,20|	Z8878918799	**1**{*292*}	CCOc1cncc(N)n1	**2**{*212*}	FC(F)c1cc(C=O)no1	**3**{*35*}	CSCCC[N+]#[C-]	0.0	0
1946	**4**{*301*,*735*,*22*}	COC(=O)C1=CN=C(S1)C1=C(NC2COC2)N2C=C(Cl)C=C(F)C2=N1 |c:6,10,26,t:4,19,22|	Z8878918778	**1**{*301*}	Nc1ncc(Cl)cc1F	**2**{*735*}	COC(=O)c1cnc(C=O)s1	**3**{*22*}	[C-]#[N+]C1COC1	0.0	0
1947	**4**{*73*,*558*,*20*}	COC1=CC(C)=C(NC2=C(N=C3C=CC4=NC=CN4N23)C2=CC(Cl)=NC(C)=C2)C=C1 |c:12,16,26,29,32,t:2,5,8,10,14,23|	Z8878918591	**1**{*73*}	Nc1ccc2nccn2n1	**2**{*558*}	Cc1cc(C=O)cc(Cl)n1	**3**{*20*}	COc1ccc([N+]#[C-])c(C)c1	0.0	0
1948	**4**{*394*,*395*,*10*}	CC(C)(C)OC(=O)N[C@H]1C[C@@H](C1)C1=C(NC2CCOC2)N2C(C=CC(C=C)=C2F)=N1 |r,c:13,24,28,31|	Z8878918567	**1**{*394*}	Nc1ccc(C=C)c(F)n1	**2**{*395*}	CC(C)(C)OC(=O)N[C@H]1C[C@@H](C1)C=O	**3**{*10*}	[C-]#[N+]C1CCOC1	0.0	0
1949	**4**{*399*,*216*,*15*}	CCNC1=C(N=C2C=CC(OC(C)C)=NN12)C1=CN=C(S1)C(=O)OC |c:7,13,20,t:3,5,18|	Z8878918443	**1**{*399*}	CC(C)Oc1ccc(N)nn1	**2**{*216*}	COC(=O)c1ncc(C=O)s1	**3**{*15*}	CC[N+]#[C-]	0.0	0
1950	**4**{*150*,*212*,*32*}	FC(F)C1=CC(=NO1)C1=C(NC2CCC2)N2C=CC(=CC2=N1)C1CCOC1 |c:5,9,18,20,23,t:3|	Z8878918415	**1**{*150*}	Nc1cc(ccn1)C2CCOC2	**2**{*212*}	FC(F)c1cc(C=O)no1	**3**{*32*}	[C-]#[N+]C1CCC1	0.0	0
1951	**4**{*78*,*134*,*77*}	COC1=NC(=NC=C1)C1=C(NC2CCCN(C2)C(=O)OC(C)(C)C)N2N3C=C(C)N=C3C(C)=CC2=N1 |c:4,6,9,31,35,38,t:2,28|	Z8878918312	**1**{*78*}	Cc1cn2nc(N)cc(C)c2n1	**2**{*134*}	COc1ccnc(C=O)n1	**3**{*77*}	CC(C)(C)OC(=O)N1CCC[C@@H](C1)[N+]#[C-]	0.0	0
1952	**4**{*78*,*836*,*28*}	CCCNC1=C(N=C2C=C(C)C3=NC(C)=CN3N12)C1=C(Cl)NN=C1CC |c:14,21,25,t:4,6,8,11|	Z8873684738	**1**{*78*}	Cc1cn2nc(N)cc(C)c2n1	**2**{*836*}	CCc1n[nH]c(Cl)c1C=O	**3**{*28*}	CCC[N+]#[C-]	0.0	0

1953	**4**{*59*,*837*,*49*}	CC(C)(C)OC(=O)N1C=NC(=C1)C1=C(NC2CC(F)(F)C2)N2C=C(CN3CCOCC3)C=CC2=N1 |c:8,10,13,34,37,t:24|	Z8873684673	**1**{*59*}	Nc1ccc(CN2CCOCC2)cn1	**2**{*837*}	CC(C)(C)OC(=O)n1cnc(C=O)c1	**3**{*49*}	FC1(F)CC(C1)[N+]#[C-]	0.0	0
1954	**4**{*73*,*838*,*13*}	COC1=CC=C(CNC2=C(N=C3C=CC4=NC=CN4N23)C2=CN=C(N=C2)C(C)(C)C)C=C1 |c:12,16,25,27,34,t:2,4,8,10,14,23|	Z8873685389	**1**{*73*}	Nc1ccc2nccn2n1	**2**{*838*}	CC(C)(C)c1ncc(C=O)cn1	**3**{*13*}	COc1ccc(C[N+]#[C-])cc1	0.0	0
1955	**4**{*171*,*123*,*22*}	CC1=NC=C(N=C1)C1=C(NC2COC2)N2C=CC(=CC2=N1)N1C=CN=C1 |c:3,5,8,17,19,22,26,28,t:1|	Z8873685373	**1**{*171*}	Nc1cc(ccn1)n2ccnc2	**2**{*123*}	Cc1cnc(C=O)cn1	**3**{*22*}	[C-]#[N+]C1COC1	0.0	0
1956	**4**{*81*,*40*,*9*}	CNC1=C(N=C2C=NC=CN12)C1=NC(CO)=CS1 |c:6,8,17,t:2,4,13|	Z8873685259	**1**{*81*}	Nc1cnccn1	**2**{*40*}	OCc1csc(C=O)n1	**3**{*9*}	C[N+]#[C-]	0.0	0
1957	**4**{*49*,*757*,*13*}	CCCN1N=CN=C1C1=C(NCC2=CC=C(OC)C=C2)N2C(C=CC=C2CO)=N1 |c:4,6,9,19,24,26,30,t:13,15|	Z8873684630	**1**{*49*}	Nc1cccc(CO)n1	**2**{*757*}	CCCn1ncnc1C=O	**3**{*13*}	COc1ccc(C[N+]#[C-])cc1	0.0	0
1958	**4**{*81*,*240*,*9*}	CCN1N=C(Br)C=C1C1=C(NC)N2C=CN=CC2=N1 |c:6,9,14,16,19,t:3|	Z8854581135	**1**{*81*}	Nc1cnccn1	**2**{*240*}	CCn1nc(Br)cc1C=O	**3**{*9*}	C[N+]#[C-]	0.0	0
1959	**4**{*481*,*654*,*69*}	COC(=O)C1=C(Br)N2C(NC3=C(OC)C=CC=C3)=C(CCC3=CN=CO3)N=C2C=C1 |c:4,10,14,16,24,28,31,t:18,22|	Z8855619774	**1**{*481*}	COC(=O)c1ccc(N)nc1Br	**2**{*654*}	O=CCCc1cnco1	**3**{*69*}	COc1ccccc1[N+]#[C-]	0.0	0
1960	**4**{*172*,*550*,*29*}	CCOC(=O)CCNC1=C(N=C2N1C=CC1=C2CCO1)C1=NC(Br)=NN1C |c:10,14,16,26,t:8,23|	Z8878918905	**1**{*172*}	Nc1nccc2OCCc21	**2**{*550*}	Cn1nc(Br)nc1C=O	**3**{*29*}	CCOC(=O)CC[N+]#[C-]	0.0	0
1961	**4**{*482*,*131*,*22*}	COC(=O)C1=C(C)C=CC2=NC(C3=NC(=NN3)C3CC3)=C(NC3COC3)N12 |c:4,7,14,t:9,12,21|	Z8878918858	**1**{*482*}	COC(=O)c1nc(N)ccc1C	**2**{*131*}	O=Cc1nc(n[nH]1)C2CC2	**3**{*22*}	[C-]#[N+]C1COC1	0.0	0
1962	**4**{*375*,*516*,*46*}	CC(C)OC1=CN=CC2=NC(C3=NN(C)C(=N3)C3CC3)=C(NCC(C)=C)N12 |c:6,15,t:4,8,11,21|	Z8878918857	**1**{*375*}	CC(C)Oc1cncc(N)n1	**2**{*516*}	Cn1nc(C=O)nc1C2CC2	**3**{*46*}	CC(=C)C[N+]#[C-]	0.0	0
1963	**4**{*288*,*350*,*28*}	CCCNC1=C(N=C2C=C(NC(=O)OC(C)(C)C)C=CN12)C1C2CCOCC12 |c:18,t:4,6,8|	Z8878918619	**1**{*288*}	CC(C)(C)OC(=O)Nc1ccnc(N)c1	**2**{*350*}	O=CC1C2CCOCC12	**3**{*28*}	CCC[N+]#[C-]	0.0	0
1964	**4**{*187*,*342*,*12*}	ClC1=CN2C(NCC3CCOC3)=C(N=C2C=C1C#N)C1=CN=C(S1)C1=NC=CC=C1 |c:12,14,17,24,30,32,t:1,22,28|	Z8878918585	**1**{*187*}	Nc1cc(C#N)c(Cl)cn1	**2**{*342*}	O=Cc1cnc(s1)c2ccccn2	**3**{*12*}	[C-]#[N+]CC1COC1	0.0	0
1965	**4**{*353*,*306*,*15*}	CCNC1=C(N=C2C=CC(NS(C)(=O)=O)=CN12)C1=CC=C(C=C1)N(C)CCO |c:7,14,21,23,t:3,5,19|	Z8878918483	**1**{*353*}	Cl.CS(=O)(=O)Nc1ccc(N)nc1	**2**{*306*}	CN(CCO)c1ccc(C=O)cc1	**3**{*15*}	CC[N+]#[C-]	0.0	0

1966	**4**{*261*,*148*,*28*}	CCCNC1=C(N=C2C=CC(=CN12)N1CCC(CC1)N(C)C)C1=C(OC)N(C)N=C1C |c:8,10,25,31,t:4,6|	Z8878918402	**1**{*261*}	CN(C)C1CCN(CC1)c2ccc(N)nc2	**2**{*148*}	COc1c(C=O)c(C)nn1C	**3**{*28*}	CCC[N+]#[C-]	0.0	0
1967	**4**{*79*,*197*,*13*}	COC(=O)C1=CC(=NN1C)C1=C(NCC2=CC=C(OC)C=C2)N2C(C=NC=C2C)=N1 |c:6,11,21,26,28,31,t:4,15,17|	Z8878918378	**1**{*79*}	Cc1cncc(N)n1	**2**{*197*}	COC(=O)c1cc(C=O)nn1C	**3**{*13*}	COc1ccc(C[N+]#[C-])cc1	0.0	0
1968	**4**{*78*,*839*,*28*}	CCCNC1=C(N=C2C=C(C)C3=NC(C)=CN3N12)C1=CN(C=N1)C1=CC=CC=C1 |c:14,24,29,31,t:4,6,8,11,21,27|	Z8873684735	**1**{*78*}	Cc1cn2nc(N)cc(C)c2n1	**2**{*839*}	O=Cc1cn(cn1)c2ccccc2	**3**{*28*}	CCC[N+]#[C-]	0.0	0
1969	**4**{*73*,*161*,*13*}	CCN1C=NC=C1C1=C(NCC2=CC=C(OC)C=C2)N2N3C=CN=C3C=CC2=N1 |c:3,5,8,18,23,25,28,31,t:12,14|	Z8873684734	**1**{*73*}	Nc1ccc2nccn2n1	**2**{*161*}	CCn1cncc1C=O	**3**{*13*}	COc1ccc(C[N+]#[C-])cc1	0.0	0
1970	**4**{*397*,*424*,*5*}	COCCCNC1=C(N=C2N1C=CC(C)=C2C)C1=NN(C)C(=N1)C(F)(F)F |c:8,12,15,23,t:6,19|	Z8873684721	**1**{*397*}	Cl.Cc1ccnc(N)c1C	**2**{*424*}	Cn1nc(C=O)nc1C(F)(F)F	**3**{*5*}	COCCC[N+]#[C-]	0.0	0
1971	**4**{*91*,*840*,*7*}	NC(=O)C1=CN2C(C=C1)=NC(C1=CN(N=C1)C1=CC=C(C=C1)[N+]([O-])=O)=C2NC1CCOCC1 |c:7,9,15,20,22,27,t:3,12,18|	Z8873685479	**1**{*91*}	NC(=O)c1ccc(N)nc1	**2**{*840*}	[O-][N+](=O)c1ccc(cc1)n2cc(C=O)cn2	**3**{*7*}	[C-]#[N+]C1CCOCC1	0.0	0
1972	**4**{*22*,*262*,*9*}	CCC1=NN(C)C(=C1Cl)C1=C(NC)N2N=C(Cl)C=CC2=N1 |c:6,10,18,21,t:2,15|	Z8873685395	**1**{*22*}	Nc1ccc(Cl)nn1	**2**{*262*}	CCc1nn(C)c(C=O)c1Cl	**3**{*9*}	C[N+]#[C-]	0.0	0
1973	**4**{*184*,*792*,*14*}	COCCNC1=C(N=C2C=CC(=CN12)C(N)=S)C1=NN(C)N=C1 |c:9,11,23,t:5,7,19|	Z8873685314	**1**{*184*}	NC(=S)c1ccc(N)nc1	**2**{*792*}	Cn1ncc(C=O)n1	**3**{*14*}	COCC[N+]#[C-]	0.0	0
1974	**4**{*62*,*184*,*6*}	CCOC(=O)CCCNC1=C(N=C2C=C(CO)C=CN12)C1CC1 |c:17,t:9,11,13|	Z8873685245	**1**{*62*}	Nc1cc(CO)ccn1	**2**{*184*}	O=CC1CC1	**3**{*6*}	CCOC(=O)CCC[N+]#[C-]	0.0	0
1975	**4**{*436*,*572*,*23*}	CCC1=CC2=NC(=C(NC(CCC(=O)OC)C(=O)OC)N2C=C1)C1=NC(=CC(OC)=C1)C(=O)OC |c:22,27,31,t:2,4,6,25|	Z8873684935	**1**{*436*}	CCc1ccnc(N)c1	**2**{*572*}	COC(=O)c1cc(OC)cc(C=O)n1	**3**{*23*}	COC(=O)CCC([N+]#[C-])C(=O)OC	0.0	0
1976	**4**{*103*,*841*,*5*}	COCCCNC1=C(N=C2C=NC=C(CO)N12)C1=C(Br)C(=NN1C)C(F)(F)F |c:10,19,22,t:6,8,12|	Z8873684832	**1**{*103*}	Nc1cncc(CO)n1	**2**{*841*}	Cn1nc(c(Br)c1C=O)C(F)(F)F	**3**{*5*}	COCCC[N+]#[C-]	0.0	0
1977	**4**{*375*,*531*,*10*}	CC(C)OC1=CN=CC2=NC(=C(NC3CCOC3)N12)C1=CN=C(C)N=C1 |c:6,27,t:4,8,10,22,24|	Z8873684827	**1**{*375*}	CC(C)Oc1cncc(N)n1	**2**{*531*}	Cc1ncc(C=O)cn1	**3**{*10*}	[C-]#[N+]C1CCOC1	0.0	0
1978	**4**{*187*,*682*,*12*}	FC1=C(Cl)C(=CC=N1)C1=C(NCC2CCOC2)N2C=C(Cl)C(=CC2=N1)C#N |c:1,4,6,9,23,26,t:20|	Z8849597819	**1**{*187*}	Nc1cc(C#N)c(Cl)cn1	**2**{*682*}	Fc1nccc(C=O)c1Cl	**3**{*12*}	[C-]#[N+]CC1CCOC1	0.0	0

1979	**4**{*46*,*842*,*9*}	CNC1=C(N=C2C=CC=CN12)C1=C(Br)C=C(O1)C(=O)OC |c:6,8,13,16,t:2,4|	Z8846491863	**1**{*46*}	Nc1ccccn1	**2**{*842*}	COC(=O)c1cc(Br)c(C=O)o1	**3**{*9*}	C[N+]#[C-]	0.0	0
1980	**4**{*50*,*843*,*18*}	CC(C)(C)NC1=C(N=C2C=CC=C(N12)P(C)(C)=O)C1=C(Br)C(F)=CC(F)=C1 |c:9,11,20,24,27,t:5,7|	Z8878918768	**1**{*50*}	CP(=O)(C)c1cccc(N)n1	**2**{*843*}	Fc1cc(F)c(Br)c(C=O)c1	**3**{*18*}	CC(C)(C)[N+]#[C-]	0.0	0
1981	**4**{*81*,*844*,*9*}	CNC1=C(N=C2C=NC=CN12)C1=NN2C=CC=CC2=C1 |c:6,8,16,18,21,t:2,4,13|	Z8878918767	**1**{*81*}	Nc1cnccn1	**2**{*844*}	O=Cc1cc2ccccn2n1	**3**{*9*}	C[N+]#[C-]	0.0	0
1982	**4**{*172*,*775*,*29*}	CCOC(=O)CCNC1=C(N=C2N1C=CC1=C2CCO1)C1=C(C)N=C(S1)C(=O)OC |c:10,14,16,23,26,t:8|	Z8878918761	**1**{*172*}	Nc1nccc2OCCc21	**2**{*775*}	COC(=O)c1nc(C)c(C=O)s1	**3**{*29*}	CCOC(=O)CC[N+]#[C-]	0.0	0
1983	**4**{*70*,*845*,*31*}	CSCCNC1=C(N=C2N1C=CN=C2C1CC1)C1=CC=CN2C=CN=C12 |c:7,11,13,22,25,t:5,20,27|	Z8878918676	**1**{*70*}	Nc1nccnc1C2CC2	**2**{*845*}	O=Cc1cccn2ccnc12	**3**{*31*}	CSCC[N+]#[C-]	0.0	0
1984	**4**{*94*,*846*,*60*}	COC(=O)C1=CC(NC2=C(N=C3C=CC(F)=NN23)C2=C(C)N=NS2)=CC=C1 |c:12,15,20,23,26,28,t:4,8,10|	Z8878918568	**1**{*94*}	Nc1ccc(F)nn1	**2**{*846*}	Cc1nnsc1C=O	**3**{*60*}	COC(=O)c1cccc([N+]#[C-])c1	0.0	0
1985	**4**{*161*,*137*,*11*}	CC1=C(C(C)=NO1)C1=C(NCC2=CC(Br)=CC=C2)N2N=CC=CC2=N1 |c:4,8,15,17,21,23,26,t:1,12|	Z8878918542	**1**{*161*}	Nc1cccnn1	**2**{*137*}	Cc1noc(C)c1C=O	**3**{*11*}	Brc1cccc(C[N+]#[C-])c1	0.0	0
1986	**4**{*150*,*735*,*32*}	COC(=O)C1=CN=C(S1)C1=C(NC2CCC2)N2C=CC(=CC2=N1)C1CCOC1 |c:6,10,19,21,24,t:4|	Z8878918432	**1**{*150*}	Nc1cc(ccn1)C2CCOC2	**2**{*735*}	COC(=O)c1cnc(C=O)s1	**3**{*32*}	[C-]#[N+]C1CCC1	0.0	0
1987	**4**{*73*,*195*,*20*}	COC1=CC(C)=C(NC2=C(N=C3C=CC4=NC=CN4N23)C2=CC(F)=C(OC)N=C2)C=C1 |c:12,16,30,33,t:2,5,8,10,14,23,26|	Z8873684780	**1**{*73*}	Nc1ccc2nccn2n1	**2**{*195*}	COc1ncc(C=O)cc1F	**3**{*20*}	COc1ccc([N+]#[C-])c(C)c1	0.0	0
1988	**4**{*28*,*847*,*7*}	CC1CC(CN1C(=O)OC(C)(C)C)C1=C(NC2CCOCC2)N2C=CC(=CC2=N1)C1=NOC=N1 |c:14,25,27,30,36,t:33|	Z8873684679	**1**{*28*}	Cl.Nc1cc(ccn1)c2ncon2	**2**{*847*}	CC1CC(CN1C(=O)OC(C)(C)C)C=O	**3**{*7*}	[C-]#[N+]C1CCOCC1	0.0	0
1989	**4**{*267*,*354*,*59*}	CP(C)(=O)C1=C(C=CC=C1)C1=C(NCCC2=CC(F)=CC=C2)N2C(C=CC=C2C#C)=N1 |c:6,8,11,19,21,26,28,32,t:4,16|	Z8873684664	**1**{*267*}	Nc1cccc(C#C)n1	**2**{*354*}	CP(=O)(C)c1ccccc1C=O	**3**{*59*}	Fc1cccc(CC[N+]#[C-])c1	0.0	0
1990	**4**{*99*,*767*,*66*}	CCCC(OC)C1=C(NC(C)C2=CC=CC=C2)N2C=C(C=CC2=N1)S(N)(=O)=O |c:6,13,15,19,21,24,t:11|	Z8873684891	**1**{*99*}	Nc1ccc(cn1)S(=O)(=O)N	**2**{*767*}	CCCC(OC)C=O	**3**{*66*}	CC([N+]#[C-])c1ccccc1	0.0	0

1991	**4**{*164*,*804*,*21*}	CN(C)CC1=CC2=NC(=C(NCC3CC3)N2C=C1)C1=C(Br)N=CN1C |c:18,21,24,t:4,6,8|	Z8873684889	**1**{*164*}	CN(C)Cc1ccnc(N)c1	**2**{*804*}	Cn1cnc(Br)c1C=O	**3**{*21*}	[C-]#[N+]CC1CC1	0.0	0
1992	**4**{*81*,*95*,*9*}	CNC1=C(N=C2C=NC=CN12)C1=CC(=NC=C1)N(C)C |c:6,8,15,17,t:2,4,13|	Z8873684629	**1**{*81*}	Nc1cnccn1	**2**{*95*}	CN(C)c1cc(C=O)ccn1	**3**{*9*}	C[N+]#[C-]	0.0	0
1993	**4**{*78*,*848*,*28*}	CCCNC1=C(N=C2C=C(C)C3=NC(C)=CN3N12)C1=CN(N=C1C)C1=CC=C(C)C=C1 |c:14,24,33,t:4,6,8,11,21,28,30|	Z8873684841	**1**{*78*}	Cc1cn2nc(N)cc(C)c2n1	**2**{*848*}	Cc1nn(cc1C=O)c2ccc(C)cc2	**3**{*28*}	CCC[N+]#[C-]	0.0	0
1994	**4**{*340*,*13*,*5*}	CCOC1=CC=CN2C(NCCCOC)=C(N=C12)C1=C(C)NN=N1 |c:5,14,20,24,t:3,16|	Z8873684808	**1**{*340*}	CCOc1cccnc1N	**2**{*13*}	Cc1[nH]nnc1C=O	**3**{*5*}	COCCC[N+]#[C-]	0.0	0
1995	**4**{*483*,*55*,*33*}	COC1=C(CNC2=C(N=C3C=CC(=CN23)N2C(C)=CC=C2C)C2=CC(=NC=C2)P(C)(C)=O)C=CC=C1 |c:2,10,12,19,21,27,29,36,38,t:6,8,25|	Z8854581164	**1**{*483*}	Cc1ccc(C)n1c2ccc(N)nc2	**2**{*55*}	CP(=O)(C)c1cc(C=O)ccn1	**3**{*33*}	COc1ccccc1C[N+]#[C-]	0.0	0
1996	**4**{*452*,*849*,*51*}	CC(C)(C)OC(=O)NCCNC1=C(CN2C(=O)COC3=C2C=CC=C3)N=C2C=CC3=C(C=NC=C3)N12 |c:11,19,22,24,29,33,35,t:27,31|	Z8835022887	**1**{*452*}	Nc1ccc2ccncc2n1	**2**{*849*}	O=CCN1C(=O)COc2ccccc12	**3**{*51*}	CC(C)(C)OC(=O)NCC[N+]#[C-]	0.0	0
1997	**4**{*123*,*779*,*31*}	COC1=C2NC=NC2=CC(=C1)C1=C(NCCSC)N2C=CC(OCCO)=CC2=N1 |c:2,5,8,10,13,21,27,30|	Z8835022867	**1**{*123*}	Cl.Nc1cc(OCCO)ccn1	**2**{*779*}	COc1cc(C=O)cc2nc[nH]c12	**3**{*31*}	CSCC[N+]#[C-]	0.0	0
1998	**4**{*73*,*284*,*20*}	COC1=CC(C)=C(NC2=C(N=C3C=CC4=NC=CN4N23)C2=C(C)N=CC=N2)C=C1 |c:12,16,23,26,28,31,t:2,5,8,10,14|	Z8849597812	**1**{*73*}	Nc1ccc2nccn2n1	**2**{*284*}	Cc1nccnc1C=O	**3**{*20*}	COc1ccc([N+]#[C-])c(C)c1	0.0	0
1999	**4**{*397*,*740*,*23*}	COC(=O)CCC(NC1=C(N=C2N1C=CC(C)=C2C)C1=CN(N=C1)C(F)F)C(=O)OC |c:10,14,17,24,t:8,21|	Z8855739214	**1**{*397*}	Cl.Cc1ccnc(N)c1C	**2**{*740*}	FC(F)n1cc(C=O)cn1	**3**{*23*}	COC(=O)CCC([N+]#[C-])C(=O)OC	0.0	0
2000	**4**{*65*,*850*,*23*}	CCC1=CN=CC2=NC(C3=NN(C=C3)C(F)F)=C(NC(CCC(=O)OC)C(=O)OC)N12 |c:4,12,t:2,6,9,17|	Z8846491975	**1**{*65*}	CCc1cncc(N)n1	**2**{*850*}	FC(F)n1ccc(C=O)n1	**3**{*23*}	COC(=O)CCC([N+]#[C-])C(=O)OC	0.0	0
2001	**4**{*433*,*191*,*31*}	CCN1C=NN=C1C1=C(NCCSC)N2C(C=CC(C#C)=C2C)=N1 |c:3,5,8,17,21,24|	Z8878918624	**1**{*433*}	Cc1nc(N)ccc1C#C	**2**{*191*}	CCn1cnnc1C=O	**3**{*31*}	CSCC[N+]#[C-]	0.0	0
2002	**4**{*46*,*46*,*9*}	CNC1=C(CCCCCOC)N=C2C=CC=CN12 |c:2,13,15,t:11|	Z8878918468	**1**{*46*}	Nc1ccccn1	**2**{*46*}	COCCCCCC=O	**3**{*9*}	C[N+]#[C-]	0.0	0
2003	**4**{*393*,*177*,*23*}	COC(=O)CCC(NC1=C(CC2CC(C2)C(=O)OC)N=C2C=CC(Cl)=C(Cl)N12)C(=O)OC |c:8,22,t:20,25|	Z8873684763	**1**{*393*}	Nc1ccc(Cl)c(Cl)n1	**2**{*177*}	COC(=O)C1CC(CC=O)C1	**3**{*23*}	COC(=O)CCC([N+]#[C-])C(=O)OC	0.0	0

2004	**4**{*80,258,67*}	CCC(C)NC1=C(N=C2N1C=CN=C2OC)C1=NN=C(S1)C1CC1 |c:7,11,13,20,t:5,18|	Z8873685494	**1**{*80*}	COc1nccnc1N	**2**{*258*}	O=Cc1nnc(s1)C2CC2	**3**{*67*}	CCC(C)[N+]#[C-]	0.0	0
2005	**4**{*22,197,13*}	COC(=O)C1=CC(=NN1C)C1=C(NCC2=CC=C(OC)C=C2)N2N=C(Cl)C=CC2=N1 |c:6,11,21,28,31,t:4,15,17,25|	Z8873685384	**1**{*22*}	Nc1ccc(Cl)nn1	**2**{*197*}	COC(=O)c1cc(C=O)nn1C	**3**{*13*}	COc1ccc(C[N+]#[C-])cc1	0.0	0
2006	**4**{*156,119,22*}	CCOC(=O)C1=NN=C(N1)C1=C(NC2COC2)N2C(=N1)C(Cl)=CC=C2Cl |c:7,11,20,24,26,t:5|	Z8873685327	**1**{*156*}	Nc1nc(Cl)ccc1Cl	**2**{*119*}	CCOC(=O)c1nnc(C=O)[nH]1	**3**{*22*}	[C-]#[N+]C1COC1	0.0	0
2007	**4**{*79,121,13*}	CCC1=CON=C1C1=C(NCC2=CC=C(OC)C=C2)N2C(C=NC=C2C)=N1 |c:5,8,18,23,25,28,t:2,12,14|	Z8873684603	**1**{*79*}	Cc1cncc(N)n1	**2**{*121*}	CCc1conc1C=O	**3**{*13*}	COc1ccc(C[N+]#[C-])cc1	0.0	0
2008	**4**{*28,851,7*}	COC1=C(SC)C=C(C=C1)C1=C(NC2CCOCC2)N2C=CC(=CC2=N1)C1=NOC=N1 |c:2,6,8,11,22,24,27,33,t:30|	Z8855739220	**1**{*28*}	Cl.Nc1cc(ccn1)c2ncon2	**2**{*851*}	COc1ccc(C=O)cc1SC	**3**{*7*}	[C-]#[N+]C1CCOCC1	0.0	0
2009	**4**{*94,762,21*}	FC1=NN2C(C=C1)=NC(C1CC1(F)F)=C2NCC1CC1 |c:5,7,15,t:1|	Z8855739147	**1**{*94*}	Nc1ccc(F)nn1	**2**{*762*}	FC1(F)CC1C=O	**3**{*21*}	[C-]#[N+]CC1CC1	0.0	0
2010	**4**{*484,569,78*}	CC(C)(C)OC(=O)NCC1=CC=C(NC2=C(CC3COC3=O)N=C3C=CC4=C(N=CC=C4)N23)C=C1 |c:14,26,30,32,38,t:9,11,24,28|	Z8878918838	**1**{*484*}	Br.Nc1ccc2cccnc2n1	**2**{*569*}	O=CCC1COC1=O	**3**{*78*}	CC(C)(C)OC(=O)NCc1ccc([N+]#[C-])cc1	0.0	0
2011	**4**{*398,689,15*}	CCNC1=C(N=C2C=C(C=CN12)C(C)(F)F)C1=CN(CCOC(C)=O)N=C1 |c:7,9,27,t:3,5,18|	Z8878918781	**1**{*398*}	Cl.CC(F)(F)c1ccnc(N)c1	**2**{*689*}	CC(=O)OCCn1cc(C=O)cn1	**3**{*15*}	CC[N+]#[C-]	0.0	0
2012	**4**{*103,455,40*}	CCOC(=O)C1=CC=C(NC2=C(N=C3C=NC=C(CO)N23)C2=C(C)ON=C2Br)C=C1 |c:14,23,27,31,t:5,7,10,12,16|	Z8878918650	**1**{*103*}	Nc1cncc(CO)n1	**2**{*455*}	Cc1onc(Br)c1C=O	**3**{*40*}	CCOC(=O)c1ccc([N+]#[C-])cc1	0.0	0
2013	**4**{*188,352,38*}	COCCCC1=C(NC2=CC3=C(OCO3)C=C2)N2C(C=NC=C2C(=O)OC)=N1 |c:5,16,21,23,29,t:8,10|	Z8878918549	**1**{*188*}	COC(=O)c1cncc(N)n1	**2**{*352*}	COCCCC=O	**3**{*38*}	[C-]#[N+]c1ccc2OCOc2c1	0.0	0
2014	**4**{*234,427,10*}	C1CC(C1)OC1=NC=CN2C(NC3CCOC3)=C(N=C12)C1=NOC2=C1COCC2 |c:8,18,27,t:6,20,24|	Z8873684715	**1**{*234*}	Nc1nccnc1OC2CCC2	**2**{*427*}	O=Cc1noc2CCOCc12	**3**{*10*}	[C-]#[N+]C1CCOC1	0.0	0
2015	**4**{*436,852,23*}	CCC1=CC2=NC(=C(NC(CCC(=O)OC)C(=O)OC)N2C=C1)C1=CC(=CN=C1)C(F)F |c:22,27,29,t:2,4,6,25|	Z8873684687	**1**{*436*}	CCc1ccnc(N)c1	**2**{*852*}	FC(F)c1cncc(C=O)c1	**3**{*23*}	COC(=O)CCC([N+]#[C-])C(=O)OC	0.0	0

2016	**4**{*332*,*454*,*21*}	CCC1=CC=CN2C(NCC3CC3)=C(CC(C)OC)N=C12 |c:4,t:2,13,20|	Z8873685478	**1**{*332*}	CCc1cccnc1N	**2**{*454*}	COC(C)CC=O	**3**{*21*}	[C-]#[N+]CC1CC1	0.0	0
2017	**4**{*448*,*50*,*31*}	CSCCNC1=C(N=C2C=C(F)C(C=C)=CN12)[C@@H]1C[C@H]1C1OCCO1 |&1:17,19,r,c:14,t:5,7,9|	Z8873684887	**1**{*448*}	Nc1cc(F)c(C=C)cn1	**2**{*50*}	O=C[C@@H]1C[C@H]1C2OCCO2 |&1:2,4,r|	**3**{*31*}	CSCC[N+]#[C-]	0.0	0
2018	**4**{*73*,*838*,*20*}	COC1=CC(C)=C(NC2=C(N=C3C=CC4=NC=CN4N23)C2=CN=C(N=C2)C(C)(C)C)C=C1 |c:12,16,25,27,34,t:2,5,8,10,14,23|	Z8873684626	**1**{*73*}	Nc1ccc2nccn2n1	**2**{*838*}	CC(C)(C)c1ncc(C=O)cn1	**3**{*20*}	COc1ccc([N+]#[C-])c(C)c1	0.0	0
2019	**4**{*48*,*131*,*15*}	CCNC1=C(N=C2C=C(C=CN12)S(C)(=O)=O)C1=NC(=NN1)C1CC1 |c:7,9,20,t:3,5,18|	Z8873684591	**1**{*48*}	CS(=O)(=O)c1ccnc(N)c1	**2**{*131*}	O=Cc1nc(n[nH]1)C2CC2	**3**{*15*}	CC[N+]#[C-]	0.0	0
2020	**4**{*78*,*853*,*46*}	CC(=C)CNC1=C(N=C2C=C(C)C3=NC(C)=CN3N12)C1=CN=C(N=C1)N1CCCC1 |c:15,24,26,t:5,7,9,12,22|	Z8835022902	**1**{*78*}	Cc1cn2nc(N)cc(C)c2n1	**2**{*853*}	O=Cc1cnc(nc1)N2CCCC2	**3**{*46*}	CC(=C)C[N+]#[C-]	0.0	0
2021	**4**{*161*,*727*,*6*}	CCOC(=O)CCCNC1=C(N=C2C=CC=NN12)C1=CN=C(N=C1)C(C)C |c:13,15,22,24,t:9,11,20|	Z8835022871	**1**{*161*}	Nc1cccnn1	**2**{*727*}	CC(C)c1ncc(C=O)cn1	**3**{*6*}	CCOC(=O)CCC[N+]#[C-]	0.0	0
2022	**4**{*73*,*162*,*13*}	COC1=CC=C(CNC2=C(N=C3C=CC4=NC=CN4N23)C2=CC(OC)=NC(C)=C2)C=C1 |c:12,16,27,30,33,t:2,4,8,10,14,23|	Z8855739268	**1**{*73*}	Nc1ccc2nccn2n1	**2**{*162*}	COc1cc(C=O)cc(C)n1	**3**{*13*}	COc1ccc(C[N+]#[C-])cc1	0.0	0
2023	**4**{*78*,*854*,*28*}	CCCNC1=C(N=C2C=C(C)C3=NC(C)=CN3N12)C1=CN(C)N=C1CN1C(=O)C2=C(C=CC=C2)C1=O |c:14,25,34,36,t:4,6,8,11,21,32|	Z8878918623	**1**{*78*}	Cc1cn2nc(N)cc(C)c2n1	**2**{*854*}	Cn1cc(C=O)c(CN2C(=O)c3ccccc3C2=O)n1	**3**{*28*}	CCC[N+]#[C-]	0.0	0
2024	**4**{*397*,*585*,*23*}	COC(=O)CCC(NC1=C(CC2CC2(Cl)Cl)N=C2N1C=CC(C)=C2C)C(=O)OC |c:8,17,21,24|	Z8878918590	**1**{*397*}	Cl.Cc1ccnc(N)c1C	**2**{*585*}	ClC1(Cl)CC1CC=O	**3**{*23*}	COC(=O)CCC([N+]#[C-])C(=O)OC	0.0	0
2025	**4**{*103*,*855*,*59*}	OCC1=CN=CC2=NC(C3=CC(=NO3)C3CCC3)=C(NCCC3=CC(F)=CC=C3)N12 |c:4,11,27,29,t:2,6,9,19,24|	Z8878918366	**1**{*103*}	Nc1cncc(CO)n1	**2**{*855*}	O=Cc1cc(no1)C2CCC2	**3**{*59*}	Fc1cccc(CC[N+]#[C-])c1	0.0	0
2026	**4**{*22*,*69*,*13*}	COC1=CC=C(CNC2=C(N=C3C=CC(Cl)=NN23)C2=NN(C)C(C)=C2)C=C1 |c:12,15,25,28,t:2,4,8,10,20|	Z8878918345	**1**{*22*}	Nc1ccc(Cl)nn1	**2**{*69*}	Cc1cc(C=O)nn1C	**3**{*13*}	COc1ccc(C[N+]#[C-])cc1	0.0	0
2027	**4**{*85*,*191*,*31*}	CCN1C=NN=C1C1=C(NCCSC)N2C(C=C(C=C2C)C#N)=N1 |c:3,5,8,17,19,24|	Z8873684761	**1**{*85*}	Cc1cc(C#N)cc(N)n1	**2**{*191*}	CCn1cnnc1C=O	**3**{*31*}	CSCC[N+]#[C-]	0.0	0

2028	**4**{*73* ,*91* ,*13* }	COC1=CC=C(CNC2=C(N=C3C=CC4=NC=CN4N23)C2=CC3=C(OCCC3)C=C2)C=C1 |c:12,16,32,35,t:2,4,8,10,14,23,25|	Z8873684728	**1**{*73* }	Nc1ccc2nccn2n1	**2**{*91* }	O=Cc1ccc2OCCCc2c1	**3**{*13* }	COc1ccc(C[N+]#[C-])cc1	0.0	0
2029	**4**{*261* ,*856* ,*12* }	CCC1=C(N(C)N=C1)C1=C(NCC2CCOC2)N2C=C(C=CC2=N1)N1CCC(CC1)N(C)C |c:6,9,20,22,25,t:2|	Z8873685402	**1**{*261* }	CN(C)C1CCN(CC1)c2ccc(N)nc2	**2**{*856* }	CCc1cnn(C)c1C=O	**3**{*12* }	[C-]#[N+]CC1CCOC1	0.0	0
2030	**4**{*119* ,*291* ,*21* }	COC1=C(C)C(=CN=C1)C1=C(NCC2CC2)N2C=C(C=CC2=N1)C#N |c:2,5,7,10,19,21,24|	Z8873684942	**1**{*119* }	Nc1ccc(C#N)cn1	**2**{*291* }	COc1cncc(C=O)c1C	**3**{*21* }	[C-]#[N+]CC1CC1	0.0	0
2031	**4**{*333* ,*857* ,*23* }	COC(=O)CCC(NC1=C(N=C2C=CC=C(N12)C1=CC=NC=C1)C1=C(F)C(OC)=NC=C1)C(=O)OC |c:12,14,21,23,26,31,33,t:8,10,19|	Z8873684932	**1**{*333* }	Nc1cccc(n1)c2ccncc2	**2**{*857* }	COc1nccc(C=O)c1F	**3**{*23* }	COC(=O)CCC([N+]#[C-])C(=O)OC	0.0	0
2032	**4**{*164* ,*242* ,*10* }	CN(C)CC1=CC2=NC(=C(NC3CCOC3)N2C=C1)C1=CN2N=CC(C#N)=C2N=C1 |c:19,25,29,32,t:4,6,8,22|	Z8873684815	**1**{*164* }	CN(C)Cc1ccnc(N)c1	**2**{*242* }	O=Cc1cnc2c(C#N)cnn2c1	**3**{*10* }	[C-]#[N+]C1CCOC1	0.0	0
2033	**4**{*100* ,*408* ,*37* }	COC(=O)C1CC(C1)NC1=C(CCN2CCOC2=O)N=C2C=NC3=C(C=CC=C3)N12 |c:10,23,27,29,t:21,25|	Z8846491960	**1**{*100* }	Nc1cnc2ccccc2n1	**2**{*408* }	O=CCCN1CCOC1=O	**3**{*37* }	COC(=O)C1CC(C1)[N+]#[C-]	0.0	0
2034	**4**{*160* ,*247* ,*14* }	CCOC1CC(C1)C1=C(NCCOC)N2C=C(Br)C=C(C#C)C2=N1 |c:8,24,t:16,19|	Z8878918815	**1**{*160* }	Nc1ncc(Br)cc1C#C	**2**{*247* }	CCOC1CC(C1)C=O	**3**{*14* }	COCC[N+]#[C-]	0.0	0
2035	**4**{*167* ,*294* ,*5* }	COCCCNC1=C(N=C2N1C(C)=CN=C2C)C1=C(OC)C(=O)C=CO1 |c:8,13,15,19,25,t:6|	Z8878918763	**1**{*167* }	Cc1cnc(C)c(N)n1	**2**{*294* }	COc1c(C=O)occc1=O	**3**{*5* }	COCCC[N+]#[C-]	0.0	0
2036	**4**{*452* ,*858* ,*44* }	COC1=CC(=O)OC(=C1)C1=C(NCCCCNC(=O)OC(C)(C)C)N2C(C=CC3=C2C=NC=C3)=N1 |c:7,10,27,29,32,34,36,t:2|	Z8878918580	**1**{*452* }	Nc1ccc2ccncc2n1	**2**{*858* }	COc1cc(C=O)oc(=O)c1	**3**{*44* }	CC(C)(C)OC(=O)NCCCC[N+]#[C-]	0.0	0
2037	**4**{*81* ,*826* ,*13* }	COC1=CC=C(CNC2=C(N=C3C=NC=CN23)C2=NC(Cl)=CS2)C=C1 |c:12,14,22,26,t:2,4,8,10,19|	Z8878918556	**1**{*81* }	Nc1cnccn1	**2**{*826* }	Clc1csc(C=O)n1	**3**{*13* }	COc1ccc(C[N+]#[C-])cc1	0.0	0
2038	**4**{*46* ,*859* ,*9* }	CNC1=C(N=C2C=CC=CN12)C1=CN2C=CC=C(F)C2=N1 |c:6,8,16,22,t:2,4,13,18|	Z8878918480	**1**{*46* }	Nc1ccccn1	**2**{*859* }	Fc1cccn2cc(C=O)nc12	**3**{*9* }	C[N+]#[C-]	0.0	0
2039	**4**{*267* ,*860* ,*5* }	CCOC(=O)C1=NC(=NN1C)C1=C(NCCCOC)N2C(C=CC=C2C#C)=N1 |c:7,12,22,24,28,t:5|	Z8878918426	**1**{*267* }	Nc1cccc(C#C)n1	**2**{*860* }	CCOC(=O)c1nc(C=O)nn1C	**3**{*5* }	COCCC[N+]#[C-]	0.0	0

2040	**4**{*103* ,*861* ,*5* }	COCCCNC1=C(N=C2C=NC=C(CO)N12)C1=C(OC)C(Cl)=CC=C1Cl |c:10,19,24,26,t:6,8,12|	Z8873684770	**1**{*103* }	Nc1cncc(CO)n1	**2**{*861* }	COc1c(Cl)ccc(Cl)c1C=O	**3**{*5* }	COCCC[N+]#[C-]	0.0	0
2041	**4**{*352* ,*862* ,*47* }	CC(C)NC1=C(N=C2C=CC(=CN12)N1CCN(C)C(=O)C1)C1=CSC(=N1)N(C)C(C)=O |c:8,10,27,t:4,6,24|	Z8873684746	**1**{*352* }	CN1CCN(CC1=O)c2ccc(N)nc2	**2**{*862* }	CN(C(=O)C)c1nc(C=O)cs1	**3**{*47* }	CC(C)[N+]#[C-]	0.0	0
2042	**4**{*78* ,*863* ,*28* }	CCCNC1=C(N=C2C=C(C)C3=NC(C)=CN3N12)C1=CC2=C(OC(C)(C)C2)C=C1 |c:14,31,t:4,6,8,11,21,23|	Z8873684710	**1**{*78* }	Cc1cn2nc(N)cc(C)c2n1	**2**{*863* }	CC1(C)Cc2cc(C=O)ccc2O1	**3**{*28* }	CCC[N+]#[C-]	0.0	0
2043	**4**{*125* ,*864* ,*18* }	COC(=O)C1=CN2C(C=C1)=NC=C2C1=C(NC(C)(C)C)N2C=C(CCO)C=CC2=N1 |c:8,10,12,15,28,31,t:4,23|	Z8873684647	**1**{*125* }	Nc1ccc(CCO)cn1	**2**{*864* }	COC(=O)c1ccc2ncc(C=O)n2c1	**3**{*18* }	CC(C)(C)[N+]#[C-]	0.0	0
2044	**4**{*327* ,*865* ,*34* }	COC(=O)C1=C(F)C2=NC(=C(NCC3CCCO3)N2C=C1)C1=NN2CCCCC2=C1 |c:4,21,32,t:7,9,24|	Z8873685355	**1**{*327* }	COC(=O)c1ccnc(N)c1F	**2**{*865* }	O=Cc1cc2CCCCn2n1	**3**{*34* }	[C-]#[N+]CC1CCCO1	0.0	0
2045	**4**{*485* ,*564* ,*29* }	CCOC(=O)CCNC1=C(N=C2N1C=CC1=C2CN(CC1)C(=O)OC(C)(C)C)C1=CN=NN1C |c:10,14,16,33,t:8,31|	Z8873684924	**1**{*485* }	CC(C)(C)OC(=O)N1CCc2ccnc(N)c2C1	**2**{*564* }	Cn1nncc1C=O	**3**{*29* }	CCOC(=O)CC[N+]#[C-]	0.0	0
2046	**4**{*161* ,*363* ,*11* }	CC1=NC(=CN1)C1=C(NCC2=CC(Br)=CC=C2)N2N=CC=CC2=N1 |c:3,7,14,16,20,22,25,t:1,11|	Z8873684806	**1**{*161* }	Nc1cccnn1	**2**{*363* }	Cc1nc(C=O)c[nH]1	**3**{*11* }	Brc1cccc(C[N+]#[C-])c1	0.0	0
2047	**4**{*364* ,*866* ,*38* }	FC(F)(F)COCCC1=C(NC2=CC3=C(OCO3)C=C2)N2C=C(C=CC2=N1)N1CCNC(=O)C1 |c:8,19,23,25,28,t:11,13|	Z8854581162	**1**{*364* }	Nc1ccc(cn1)N2CCNC(=O)C2	**2**{*866* }	FC(F)(F)COCCC=O	**3**{*38* }	[C-]#[N+]c1ccc2OCOc2c1	0.0	0
2048	**4**{*78* ,*867* ,*46* }	COC(=O)C1=NC=CC(=C1)C1=C(NCC(C)=C)N2N3C=C(C)N=C3C(C)=CC2=N1 |c:6,8,11,23,27,30,t:4,20|	Z8835022905	**1**{*78* }	Cc1cn2nc(N)cc(C)c2n1	**2**{*867* }	COC(=O)c1cc(C=O)ccn1	**3**{*46* }	CC(=C)C[N+]#[C-]	0.0	0
2049	**4**{*143* ,*862* ,*43* }	CCC1=NN2C(C=C1)=NC(=C2NC1CCN(CC1)C(=O)OC(C)(C)C)C1=CSC(=N1)N(C)C(C)=O |c:6,8,10,31,t:2,28|	Z8849597840	**1**{*143* }	CCc1ccc(N)nn1	**2**{*862* }	CN(C(=O)C)c1nc(C=O)cs1	**3**{*43* }	CC(C)(C)OC(=O)N1CCC(CC1)[N+]#[C-]	0.0	0
2050	**4**{*121* ,*272* ,*10* }	CC(C)C1=NN=C(S1)C1=C(NC2CCOC2)N2C=CC(Br)=CC2=N1 |c:5,9,19,22,25,t:3|	Z8849597839	**1**{*121* }	Nc1cc(Br)ccn1	**2**{*272* }	CC(C)c1nnc(C=O)s1	**3**{*10* }	[C-]#[N+]C1CCOC1	0.0	0
2051	**4**{*73* ,*161* ,*11* }	CCN1C=NC=C1C1=C(NCC2=CC(Br)=CC=C2)N2N3C=CN=C3C=CC2=N1 |c:3,5,8,15,17,22,24,27,30,t:12|	Z8849597806	**1**{*73* }	Nc1ccc2nccn2n1	**2**{*161* }	CCn1cncc1C=O	**3**{*11* }	Brc1cccc(C[N+]#[C-])c1	0.0	0

2052	**4**{*91*,*447*,*7*}	CC(C)(O)C#CC1=CC=C(C=C1)C1=C(NC2CCOCC2)N2C=C(C=CC2=N1)C(N)=O |c:8,10,13,24,26,29,t:6|	Z8837933136	**1**{*91*}	NC(=O)c1ccc(N)nc1	**2**{*447*}	CC(C)(O)C#Cc1ccc(C=O)cc1	**3**{*7*}	[C-]#[N+]C1CCOCC1	0.0	0
