# Supplementary material for: Generation of multimillion chemical space based on the parallel Groebke–Blackburn–Bienaymé reaction
Source: Beilstein J Org Chem. 2024 Jul 16;20:1604–13. doi: 10.3762/bjoc.20.143 (PMC11285076; doi:10.3762/bjoc.20.143)
Supplement: File 3 — Experimental part. [file Beilstein_J_Org_Chem-20-1604-s003.pdf]

BEILSTEIN JOURNAL OF ORGANIC CHEMISTRY

Supporting Information

for

Generation of multimillion chemical space based on the parallel Groebke–Blackburn–Bienaymé reaction

Evgen V. Govor, Vasyl Naumchyk, Ihor Nestorak, Dmytro S. Radchenko, Dmytro Dudenko, Yurii S. Moroz, Olexiy D. Kachkovsky and Oleksandr O. Grygorenko

Beilstein J. Org. Chem. **2024,** *20,* 1604–1613. doi:10.3762/bjoc.20.143

Experimental part

License and Terms: This is a supporting information file under the terms of the Creative Commons Attribution License (https://creativecommons.org/licenses/by/4.0). Please note that the reuse, redistribution and reproduction in particular requires that the author(s) and source are credited and that individual graphics may be subject to special legal provisions.
The license is subject to the *Beilstein Journal of Organic Chemistry* terms and conditions: (https://www.beilstein-journals.org/bjoc/terms)

Experimental

General

The solvents were purified according to the standard procedures [48]. All the starting materials were received from commercial sources. Melting points were measured on MPA100 OptiMelt automated melting point system. ^{1}H, $^{13}C\{^{1}H\}$, and $^{19}F\{^{1}H\}$ NMR spectra were recorded on an Agilent ProPulse 600 spectrometer (at 600 MHz for ^{1}H NMR, 151 MHz for $^{13}C\{^{1}H\}$ NMR), a Bruker 170 Avance 500 spectrometer (at 500 MHz for ^{1}H NMR, 126 MHz for $^{13}C\{^{1}H\}$ NMR, 470 MHz for $^{19}F\{^{1}H\}$ NMR), or a Varian Unity Plus 400 spectrometer (at 400 MHz for ^{1}H NMR, 101 MHz for $^{13}C\{^{1}H\}$ NMR, 376 MHz for $^{19}F\{^{1}H\}$ NMR) using DMSO-d_6, $CDCl_3$, CD_3OD, or D_2O as solvents. Chemical shifts are reported in ppm downfield from TMS as an internal standard. For most compounds obtained as mixtures of diastereomers or rotamers, closely located signals in $^{13}C\{^{1}H\}$ NMR spectra presumably corresponding to the same carbon atoms in the molecular graph are separated by the “and” conjunction. Mass spectra were recorded on an Agilent 1100 LCMSD SL instrument (chemical ionization (APCI)) and Agilent 5890 Series II 5972 GCMS instrument (electron impact ionization (EI)). High-resolution mass spectra were obtained on an Agilent 1260 Infinity UHPLC instrument coupled with an Agilent 6224 Accurate Mass TOF mass spectrometer. Mass spectra were recorded on an Agilent 1100 LCMSD SL instrument (chemical ionization (APCI), electrospray ionization (ESI)) and Agilent 5890 Series II 5972 GCMS instrument (electron impact ionization (EI)). HPLC purification was performed using an Agilent 1260 Infinity systems equipped with DAD and mass-detector, Waters Sunfire C18 OBD Prep Column, 100 A, 5 µm, 19 mm × 100 mm with SunFire C18 Prep Guard Cartridge, 100 A, 10 µm, 19 mm × 10 mm and gradient deionized water (phase A) –

HPLC-grade acetonitrile or methanol (phase B) as an eluent. In some cases, ammonia or TFA (1%) was used as an additive to improve the separation of the products. In these cases, free bases and trifluoroacetate salts of the products were formed, respectively.

General procedure for the synthesis of library members 4

Amine **1** (1.0 equiv, 0.270 mmol), aldehyde **2** (1.0 equiv, 0.270 mmol), isonitrile **3** (1.0 equiv, 0.270 mmol), scandium triflate (0.1 equiv, 0.027 mmol) or TsOH (0.1 equiv, 0.027 mmol), and iPr_2NEt (1.0 equiv, 0.270 mmol; only if amine **1** was used as the salt) were mixed in 0.5 mL of MeOH and stirred at room temperature for 16 h (Figure S4). Thereafter, the solvent was evaporated under reduced pressure in a centrifuge, and the residue was dissolved in DMSO (approx. 1 mL). The solution was filtered, analyzed by LCMS, and transferred for HPLC purification.

6-(6-Chloro-3-{[(3-methoxyphenyl)methyl]amino}imidazo[1,2-*a*]pyridin-2-yl)pyrazolo[1,5-*a*]pyrimidine-3-carbonitrile (4{347,242,24}). Yield 115 mg, 0.272 mmol, 99%. Yellow amorphous powder. ^{1}H NMR (500 MHz, DMSO-d_6) δ 9.47 (d, *J* = 2.1 Hz, 1H), 9.27 (d, *J* = 2.1 Hz, 1H), 8.79 (s, 1H), 8.37 (s, 1H), 7.55 (d, *J* = 9.5 Hz, 1H), 7.25 (dd, *J* = 9.5, 2.1 Hz, 1H), 6.95 (t, *J* = 7.8 Hz, 1H), 6.70 (s, 1H), 6.63 (d, *J* = 7.4 Hz, 1H), 6.52 (dd, *J* = 8.3, 2.6 Hz, 1H), 5.68 (t, *J* = 6.2 Hz, 1H), 4.03 (d, *J* = 6.2 Hz, 2H), 3.55 (s, 3H). ^{13}C{^{1}H} NMR (151 MHz, DMSO-d_6) δ 159.1, 152.6, 148.2, 148.1, 140.8, 139.7, 133.3, 129.9, 129.0, 128.0, 125.8, 121.5, 120.7, 119.3, 118.6, 117.9, 113.9, 113.3, 112.5, 80.9, 54.8, 51.2. HRMS (ESI/QTOF) m/z: $[M + Na]^+$ Calcd for $C_{22}H_{16}ClN_7ONa^+$: 452.0997; Found: 452.0985.

1,5-Dimethyl 2-{[5-chloro-2-(4-methoxy-6-methylpyridin-3-yl)-6-methylimidazo[1,2-*a*]pyridin-3-yl]amino}pentanedioate trifluoroacetate (4{76,584,23})

Yield 101 mg, 0.180 mmol, 81%. Yellowish viscous oil. ^{1}H NMR (500 MHz, DMSO-d_6) δ 8.78 (s, 1H), 7.73 (s, 1H), 7.57 (d, *J* = 9.1 Hz, 1H), 7.42 (d, *J* = 9.0 Hz, 1H), 5.00 (d, *J* = 6.8 Hz, 1H), 4.10 (s, 3H), 3.75 (d, *J* = 5.7 Hz, 1H), 3.53 (s, 3H), 3.41 (s, 3H), 2.74 (s, 3H), 2.39 (s, 3H), 2.20 – 2.05 (m, 2H), 1.89 – 1.76 (m, 1H), 1.73 – 1.61 (m, 1H). ^{13}C{^{1}H} NMR (151 MHz, DMSO-d_6) δ 172.5, 172.5, 168.3, 158.6 (q, *J*=36 Hz), 155.7, 142.7, 142.2, 130.3, 129.6, 128.3, 124.4, 122.4, 118.5, 116.8, 114.9, 109.2, 60.8, 58.0, 51.8, 51.4, 28.8, 26.5, 19.6, 17.8. HRMS (ESI/QTOF) m/z: [M + H]$^+$ Calcd for $C_{22}H_{26}ClN_4O_5^+$: 461.1586; Found: 461.1581.

Methyl 2-[1-(2-methoxyethyl)-3,5-dimethyl-1*H*-pyrazol-4-yl]-6-methyl-3-{[3-(methylsulfanyl)propyl]amino}imidazo[1,2-*a*]pyridine-7-carboxylate trifluoroacetate (4{180,545,35}). Yield 97 mg, 0.173 mmol, 81%. Yellow oil. ^{1}H NMR (500 MHz, DMSO-d_6) δ 8.56 (s, 1H), 8.13 (s, 1H), 5.75 (br s, 1H), 4.19 (t, *J* = 5.3 Hz, 2H), 3.90 (s, 3H), 3.65 (t, *J* = 5.2 Hz, 2H), 3.22 (s, 3H), 2.92 (t, *J* = 6.8 Hz, 2H), 2.55 (s, 3H), 2.34 (t, *J* = 7.2 Hz, 2H), 2.20 (s, 3H), 2.11 (s, 3H), 1.92 (s, 3H), 1.60 – 1.51 (m, 2H). ^{13}C{^{1}H} NMR (126 MHz, DMSO-d_6) δ 165.0, 158.2 (q, *J*= 3 Hz), 146.4, 140.4, 133.7, 130.6, 129.9, 123.9, 123.2, 115.6, 114.1, 105.1, 70.9, 58.2, 52.9, 48.4, 43.4, 30.3, 28.7, 17.8, 14.5, 12.2, 10.0. HRMS (ESI/QTOF) m/z: [M + H]$^+$ Calcd for $C_{22}H_{32}N_5O_3S^+$: 446.2220; Found: 446.2218.

***tert*-Butyl 3-({2-[3-(methoxymethyl)-1,2-oxazol-4-yl]-3-[(3-methoxypropyl)amino]imidazo[1,2-*a*]pyridin-6-yl}oxy)azetidine-1-carboxylate (4{29,8,5}).** Yield 95 mg, 0.194 mmol, 72%. Colourless viscous oil. ^{1}H NMR (500 MHz, DMSO-d_6) δ 9.25 (s, 1H), 7.60 (s, 1H), 7.47 (d, *J* = 9.7 Hz, 1H), 6.99 (dd, *J* = 9.7, 2.3 Hz, 1H), 5.08 – 5.00 (m, 1H), 4.91 (s, 2H), 4.79 (t, *J* = 6.2 Hz, 1H), 4.33 (br t, *J* = 8.1 Hz, 2H), 3.91 – 3.84 (m, 2H), 3.40 (t, *J* = 6.2 Hz, 2H), 3.30 (s, 3H), 3.21 (s, 3H), 2.92 (q, *J* = 6.8 Hz, 2H), 1.74 – 1.64 (m, 2H), 1.39 (s, 9H). ^{13}C{^{1}H} NMR (151 MHz, DMSO-

d$_6$) δ 158.2, 156.4, 155.5, 145.2, 137.8, 128.5, 125.7, 118.2, 117.8, 113.0, 105.7, 79.0, 69.4, 66.7, 64.7, 57.8 (d, *J* = 2 Hz), 55.9 (d, *J* = 15 Hz), 43.8, 29.9, 28.0. HRMS (ESI/QTOF) m/z: $[M + H]^+$ Calcd for $C_{24}H_{34}N_5O_6^+$: 488.2504; Found: 488.2498.

1-{2-[(2,2-Dichlorocyclopropyl)methyl]-3-[(3-methoxypropyl)amino]imidazo[1,2-*a*]pyridin-6-yl}pyrrolidin-2-one trifluoroacetate (4{405,585,5}). Yield 87 mg, 0.165 mmol, 78%. Yellowish viscous oil. ^{1}H NMR (500 MHz, DMSO-*d*$_6$) δ 14.64 (br s, 1H), 8.94 (s, 1H), 8.17 (dd, *J* = 9.7, 2.0 Hz, 1H), 7.94 (d, *J* = 9.6 Hz, 1H), 5.31 (br s, 1H), 3.95 (t, *J* = 7.0 Hz, 2H), 3.46 (t, *J* = 6.2 Hz, 2H), 3.37 (d, *J* = 5.7 Hz, 1H), 3.33 (d, *J* = 5.7 Hz, 1H), 3.04 (t, *J* = 6.9 Hz, 2H), 2.85 (d, *J* = 15.5 Hz, 1H), 2.83 (*J* = 15.5 Hz, 1H), 2.58 (t, *J* = 8.1 Hz, 2H), 2.24 – 2.10 (m, 3H), 1.83 – 1.72 (m, 3H), 1.59 (t, *J* = 7.6 Hz, 1H). ^{13}C{^{1}H} NMR (151 MHz, DMSO-*d*$_6$) δ 175.2, 158.7 (d, *J* = 33.3 Hz), 133.9, 130.8, 129.4, 126.1, 125.7, 117.4 (d, JC F= 296.3 Hz), 114.8, 112.6, 69.9, 61.6, 58.4, 48.5, 45.0, 32.3, 30.4, 28.8, 26.4, 24.9, 17.9. HRMS (ESI/QTOF) m/z: $[M + H]^+$ Calcd for $C_{19}H_{25}Cl_2N_4O_2^+$: 411.1349; Found: 411.1343.

2-(3-{[2-(Benzyloxy)ethyl]amino}-2-(3-methoxy-1,2-thiazol-4-yl)imidazo[1,2-*a*]pyridin-5-yl)propan-2-ol (4{153,336,53}). Yield 84 mg, 0.193 mmol, 72%. Colourless oil. ^{1}H NMR (500 MHz, DMSO-*d*$_6$) δ 9.07 (s, 1H), 7.59 (s, 1H), 7.39 (d, *J* = 8.8 Hz, 1H), 7.34 (s, 4H), 7.06 (t, *J* = 8.0 Hz, 1H), 6.85 (d, *J* = 7.1 Hz, 1H), 6.16 (t, *J* = 6.3 Hz, 1H), 4.44 (s, 2H), 3.95 (s, 3H), 3.47 (t, *J* = 5.3 Hz, 2H), 2.90 (q, *J* = 5.7 Hz, 2H), 1.65 (s, 6H). ^{13}C{^{1}H} NMR (151 MHz, DMSO-*d*$_6$) δ 166.9, 148.9, 144.8, 142.4, 138.2, 129.4, 128.2, 127.4, 127.4, 122.4, 120.2, 117.1, 109.6, 72.0, 70.4, 68.7, 56.0, 47.9, 30.3. HRMS (ESI/QTOF) m/z: $[M + H]^+$ Calcd for $C_{23}H_{27}N_4O_3S^+$: 439.1798; Found: 439.1798.

3-[(4-Methoxyphenyl)amino]-2-[1-(oxan-4-yl)-1*H*-pyrazol-4-yl]imidazo[1,2-*a*]pyridine-5-sulfonamide (4{109,335,41}). Yield 84 mg, 0.181 mmol, 67%. Colourless amorphous solid. ^{1}H NMR (500 MHz, DMSO-d_6) δ 7.99 (s, 1H), 7.81 (d, *J* = 8.9 Hz, 1H), 7.73 – 7.65 (m, 3H), 7.63 (d, *J* = 7.2 Hz, 1H), 7.46 (s, 1H), 7.41 (t, *J* = 8.0 Hz, 1H), 6.69 (d, *J* = 7.7 Hz, 2H), 6.41 (d, *J* = 8.3 Hz, 2H), 4.39 – 4.31 (m, 1H), 3.90 (d, *J* = 11.6 Hz, 1H), 3.61 (s, 3H), 3.43 (t, *J* = 11.6 Hz, 2H), 1.97 – 1.76 (m, 4H). ^{13}C{^{1}H} NMR (151 MHz, DMSO-d_6) δ 152.3, 143.0, 139.6, 137.9, 136.9, 135.4, 126.4, 123.0, 120.7, 120.3, 115.2, 114.7, 114.5, 114.0, 65.7, 57.0, 55.1, 32.8. HRMS (ESI/QTOF) m/z: [M + H]$^+$ Calcd for $C_{22}H_{25}N_6O_4S^+$: 469.1653; Found: 469.1650.

Methyl 3-(cyclobutylamino)-2-(3-methoxy-1,2-thiazol-4-yl)-6-methylimidazo[1,2-*a*]pyridine-7-carboxylate trifluoroacetate (4{180,336,32}). Yield 83 mg, 0.172 mmol, 83%. Yellowish amorphous powder. ^{1}H NMR (600 MHz, DMSO-d_6) δ 9.33 (s, 1H), 8.41 (s, 1H), 8.19 (s, 1H), 5.77 (s, 1H), 4.07 (s, 3H), 3.91 (s, 3H), 3.68 – 3.59 (m, 1H), 2.55 (s, 3H), 2.08 – 2.00 (m, 2H), 1.90 – 1.81 (m, 2H), 1.56 (q, *J* = 9.6 Hz, 1H), 1.50 – 1.39 (m, 1H). ^{13}C{^{1}H} NMR (151 MHz, DMSO-d_6) δ 166.1, 165.1, 158.2 (q, J = 33.7 Hz), 151.1, 135.3, 130.0, 128.3, 123.5, 123.0, 121.1, 115.9, 114.0, 56.4, 52.7, 52.0, 31.1, 17.9, 13.9. HRMS (ESI/QTOF) m/z: [M + H]$^+$ Calcd for $C_{18}H_{21}N_4O_3S^+$: 373.1329; Found: 373.1327.

2-[3-(3-Methoxypropoxy)phenyl]-*N*-methyl-3-{[(oxolan-3-yl)methyl]amino}imidazo[1,2-*a*]pyridine-7-carboxamide (4{69,287,12}). Yield 82 mg, 0.188 mmol, 70%. Colourless viscous oil. ^{1}H NMR (500 MHz, DMSO-d_6) δ 8.62 – 8.56 (m, 1H), 8.33 (d, *J* = 7.8 Hz, 1H), 8.00 (s, 1H), 7.73 (m, 2H), 7.72 – 7.69 (m, 2H), 7.39 – 7.29 (m, 2H), 6.92 – 6.86 (m, 1H), 5.09 (t, *J* = 6.0 Hz, 1H), 4.09 (t, *J* = 6.3 Hz, 2H), 3.72 – 3.63 (m, 2H), 3.57 (q, *J* = 7.6 Hz, 1H), 3.50 (t, *J* = 6.3 Hz, 1H), 3.43 (dd, *J* = 8.6, 5.8 Hz, 1H), 3.26 (s, 2H), 3.00 – 2.85 (m, 2H), 2.82 (d, *J* = 4.5 Hz, 3H), 2.40 –

2.28 (m, 1H), 2.03 – 1.93 (m, 2H), 1.95 – 1.86 (m, 1H), 1.58 – 1.48 (m, 1H). $^{13}C\{^1H\}$ NMR (151 MHz, DMSO-d_6) δ 165.2, 158.7, 139.4, 136.0, 135.5, 129.4, 129.2, 127.8, 122.7, 119.1, 115.7, 113.6, 112.5, 109.9, 70.9, 68.5, 66.8, 64.5, 58.0, 50.5, 29.9, 29.0, 26.3. HRMS (ESI/QTOF) m/z: $[M + H]^+$ Calcd for $C_{24}H_{31}N_4O_4^+$: 439.2340; Found: 439.2337.

7-(Dimethylphosphoryl)-*N*-(oxan-4-yl)-2-[1-(2,2,2-trifluoroethyl)-1*H*-pyrazol-3-yl]imidazo[1,2-*a*]pyridin-3-amine (4{32,11,7}). Yield 82 mg, 0.187 mmol, 69%. Colourless amorphous solid. ^1H NMR (500 MHz, DMSO-d_6) δ 8.36 (d, *J* = 6.5 Hz, 1H), 7.94 (s, 1H), 7.88 (d, *J* = 13.6 Hz, 1H), 7.17 (t, *J* = 7.9 Hz, 1H), 6.81 (s, 1H), 5.23 (q, *J* = 9.1 Hz, 2H), 5.04 (d, *J* = 8.6 Hz, 1H), 3.80 (d, *J* = 11.8 Hz, 1H), 3.46 – 3.37 (m, 4H), 3.22 (t, *J* = 11.5 Hz, 2H), 1.71 (d, *J* = 13.4 Hz, 6H), 1.51 – 1.40 (m, 2H). $^{13}C\{^1H\}$ NMR (151 MHz, DMSO-d_6) δ 148.3, 139.0 (d, *J* = 15.6 Hz), 133.1, 129.9 (d, *J* = 95.9 Hz), 129.1, 127.5, 123.7 (q, *J* = 283.6 Hz), 123.1 (d, *J* = 11.7 Hz), 119.7 (d, *J* = 9.3 Hz), 110.8 (d, *J* = 10.9 Hz), 104.9, 65.8, 52.3, 51.5 (q, *J* = 33.9 Hz), 33.6, 17.4 (d, *J* = 71.1 Hz). $^{19}F\{^1H\}$ NMR (376 MHz, DMSO-d_6) δ –70.6. HRMS (ESI/QTOF) m/z: $[M + H]^+$ Calcd for $C_{19}H_{24}F_3N_5O_2P^+$: 442.1614; Found: 442.1614.

Ethyl 3-({5-carbamoyl-2-[4-(morpholin-4-yl)phenyl]imidazo[1,2-*a*]pyridin-3-yl}amino)propanoate trifluoroacetate (4{105,591,29}). Yield 82 mg, 0.151 mmol, 69%. Beige amorphous solid. ^1H NMR (600 MHz, DMSO-d_6) δ 8.70 (s, 1H), 8.35 (s, 1H), 7.94 – 7.89 (m, 3H), 7.85 (t, *J* = 8.0 Hz, 1H), 7.53 (d, *J* = 7.0 Hz, 1H), 7.12 (d, *J* = 8.6 Hz, 2H), 4.55 (br s, 1H), 3.95 (q, *J* = 7.1 Hz, 2H), 3.76 (t, *J* = 4.8 Hz, 4H), 3.26 (t, *J* = 4.8 Hz, 1H), 3.06 (t, *J* = 6.9 Hz, 2H), 2.41 (t, *J* = 6.8 Hz, 2H), 1.10 (t, *J* = 7.1 Hz, 3H). $^{13}C\{^1H\}$ NMR (151 MHz, DMSO-d_6) δ 171.3, 163.9, 151.5, 137.2, 133.3, 130.9, 128.3, 128.1, 126.7, 119.6, 117.4, 116.9, 116.6 (q, JC F= 292.8 Hz), 115.5, 114.4,

113.7, 66.0, 60.0, 47.3, 43.2, 34.4, 14.0. HRMS (ESI/QTOF) m/z: $[M + H]^+$ Calcd for $C_{23}H_{28}N_5O_4^+$: 438.2136; Found: 438.2134.

Ethyl 4-{[5-(4-methylphenyl)-2-(thiophen-3-yl)imidazo[1,2-*a*]pyridin-3-yl]amino}butanoate trifluoroacetate (4{30,9,6}). Yield 80 mg, 0.150 mmol, 71%. Yellowish amorphous powder. ^{1}H NMR (500 MHz, DMSO-d_6) δ 8.21 (s, 1H), 7.91 – 7.80 (m, 2H), 7.78 (br s, 2H), 7.57 (d, *J* = 7.8 Hz, 2H), 7.35 (d, *J* = 7.8 Hz, 2H), 7.18 (d, *J* = 6.9 Hz, 1H), 3.97 (q, *J* = 7.1 Hz, 2H), 3.81 (br t, *J* = 5.8 Hz, 1H), 2.42 (s, 3H), 2.23 (q, *J* = 6.6 Hz, 2H), 1.91 (t, *J* = 7.6 Hz, 2H), 1.13 (t, *J* = 7.1 Hz, 3H), 1.10 – 1.02 (m, 2H). ^{13}C{^{1}H} NMR (151 MHz, DMSO-d_6) δ 172.8, 140.5, 139.8, 139.1, 131.7, 130.3, 129.8, 129.1, 128.7, 128.4, 128.1, 126.6, 125.9, 125.4, 119.5, 112.3, 60.1, 47.7, 31.2, 24.7, 21.4, 14.5. ^{19}F{^{1}H} NMR (376 MHz, DMSO-d_6) δ –74.3. HRMS (ESI/QTOF) m/z: $[M + H]^+$ Calcd for $C_{24}H_{26}N_3O_2S^+$: 420.1740; Found: 420.1739.

***tert*-Butyl *N*-({5-[5-(difluoromethyl)-3-[(oxolan-3-yl)amino]imidazo[1,2-*a*]pyridin-2-yl]-1-methyl-1*H*-pyrazol-4-yl}methyl)carbamate (4{37,16,10}).** Yield 79 mg, 0.171 mmol, 63%. Yellowish viscous oil. ^{1}H NMR (500 MHz, DMSO-d_6) δ 8.11 (t, *J* = 54.1 Hz, 1H), 7.72 (d, *J* = 8.2 Hz, 1H), 7.45 (s, 1H), 7.38 – 7.30 (m, 2H), 7.21 (t, *J* = 6.0 Hz, 1H), 4.69 (s, 1H), 3.92 (d, *J* = 5.9 Hz, 2H), 3.74 – 3.67 (m, 4H), 3.60 – 3.52 (m, 1H), 3.43 – 3.35 (m, 1H), 3.26 (d, *J* = 5.9 Hz, 1H), 1.76 – 1.65 (m, 1H), 1.60 – 1.50 (m, 1H), 1.31 (s, 9H). ^{13}C{^{1}H} NMR (151 MHz, DMSO-d_6) δ 156.1, 142.1, 138.0, 133.6, 131.7 (t, J= 27 Hz), 130.2, 128.9, 123.9, 120.6, 120.5, 111.9 (t, *J* = 8 Hz), 109.5 (t, *J* = 236 Hz), 78.2, 72.7, 66.7, 57.4, 37.5, 34.8, 32.6, 28.6. ^{19}F{^{1}H} NMR (376 MHz, DMSO-d_6) δ –113.9 (d, *J* = 290.3 Hz), -116.1 (d, *J* = 293.5 Hz). HRMS (ESI/QTOF) m/z: $[M + H]^+$ Calcd for $C_{22}H_{29}F_2N_6O_3^+$: 463.2264; Found: 463.2259.

2-[4-(Difluoromethyl)-2-methoxyphenyl]-8-(2-methanesulfonylethoxy)-*N*-(oxolan-3-yl)imidazo[1,2-*a*]pyridin-3-amine (4{142,605,10}). Yield 78 mg, 0.163 mmol, 60%. Yellow viscous oil. ^{1}H NMR (500 MHz, DMSO-d_6) δ 7.92 (d, J = 6.8 Hz, 1H), 7.76 (d, J = 7.8 Hz, 1H), 7.32 (s, 1H), 7.27 (d, J = 7.9 Hz, 1H), 7.02 (t, 1H), 6.85 (t, J = 7.1 Hz, 1H), 6.73 (d, J = 7.5 Hz, 1H), 4.54 (t, J = 5.5 Hz, 2H), 4.41 (d, J = 7.6 Hz, 1H), 3.91 (s, 3H), 3.73 (t, J = 5.6 Hz, 2H), 3.65 (q, J = 7.8 Hz, 1H), 3.62 – 3.52 (m, 2H), 3.45 (dd, J = 8.9, 4.9 Hz, 1H), 3.38 (dd, J = 8.9, 2.7 Hz, 1H), 3.28 (s, 3H), 1.82 – 1.72 (m, 1H), 1.58 – 1.49 (m, 1H). ^{13}C{^{1}H} NMR (151 MHz, DMSO-d_6) δ 156.0, 146.7, 135.0, 134.6 (t, J = 22.9 Hz), 131.7, 131.0, 128.00, 126.2, 118.0 (t, J = 5.8 Hz), 116.4, 114.8 (t, J = 236 Hz), 111.6, 108.9 (t, J = 6.3 Hz), 102.2, 72.4, 65.9, 63.1, 56.9, 55.9, 53.3, 42.9, 32.4. ^{19}F{^{1}H} NMR (376 MHz, DMSO-d_6) δ –109.7. HRMS (ESI/QTOF) m/z: [M + H]$^+$ Calcd for $C_{22}H_{26}F_2N_3O_5S^+$: 482.1556; Found: 482.1553.

1-[4-(6-Bromo-3-{[(4-methoxyphenyl)methyl]amino}imidazo[1,2-*a*]pyridin-2-yl)piperidin-1-yl]ethan-1-one (4{36,30,13}). Yield 78 mg, 0.170 mmol, 63%. Colourless oil. ^{1}H NMR (500 MHz, DMSO-d_6) δ 8.18 (s, 1H), 7.32 (d, J = 9.4 Hz, 1H), 7.19 – 7.13 (m, 3H), 6.83 (d, J = 8.4 Hz, 2H), 5.16 (t, J = 6.1 Hz, 1H), 4.39 (d, J = 12.9 Hz, 1H), 3.98 (d, J = 6.3 Hz, 2H), 3.82 (d, J = 13.6 Hz, 1H), 3.71 (s, 3H), 2.99 (t, J = 11.8 Hz, 1H), 2.83 – 2.72 (m, 1H), 2.49 – 2.42 (m, 1H), 2.00 (s, 3H), 1.64 (qd, J = 12.5, 4.1 Hz, 1H), 1.54 – 1.32 (m, 3H). ^{13}C{^{1}H} NMR (151 MHz, DMSO-d_6) δ 167.8, 158.5, 142.1, 138.5, 132.3, 129.7, 125.7, 125.2, 122.6, 117.5, 113.5, 105.0, 55.1, 51.1, 46.1, 41.2, 33.4, 31.9, 31.2, 21.3. HRMS (ESI/QTOF) m/z: [M + H]$^+$ Calcd for $C_{22}H_{26}BrN_4O_2^+$: 457.1234; Found: 457.1292.

4-{6-Iodo-7-methyl-3-[(oxetan-3-yl)amino]imidazo[1,2-*a*]pyridin-2-yl}benzamide (4{432,452,22}). Yield 77 mg, 0.172 mmol, 64%. Beige amorphous powder. ^{1}H NMR (500 MHz, DMSO-d_6) δ 8.63 (s, 1H), 8.15 (d, J = 8.0 Hz, 2H), 7.99 (br s, 1H), 7.93 (d,

J = 8.3 Hz, 2H), 7.51 (s, 1H), 7.35 (br s, 1H), 5.67 (d, *J* = 7.5 Hz, 1H), 4.59 (t, *J* = 6.7 Hz, 2H), 4.44 (t, *J* = 6.3 Hz, 2H), 4.36 – 4.26 (m, 1H), 2.40 (s, 3H). $^{13}C\{^{1}H\}$ NMR (151 MHz, DMSO-d_6) δ 167.6, 140.5, 136.9, 136.2, 133.8, 132.6, 128.1, 127.6, 126.1, 124.3, 115.4, 84.0, 78.2, 52.7, 26.5. HRMS (ESI/QTOF) m/z: [M + H]$^+$ Calcd for $C_{18}H_{18}IN_4O_2^+$: 449.0469; Found: 449.0467.

Methyl 2-(3-methoxy-5-methyl-1,2-oxazol-4-yl)-3-[(3-methoxypropyl)amino]imidazo[1,2-*a*]pyridine-5-carboxylate trifluoroacetate (4{374,587,5}). Yield 76 mg, 0.156 mmol, 75%. Colourless viscous oil. ^{1}H NMR (500 MHz, DMSO-d_6) δ 7.80 (d, *J* = 8.9 Hz, 1H), 7.50 (dd, *J* = 9.0, 7.0 Hz, 1H), 7.41 (d, *J* = 6.9 Hz, 1H), 3.95 (d, *J* = 1.5 Hz, 6H), 3.23 (t, *J* = 6.2 Hz, 2H), 3.13 (s, 3H), 2.72 (t, *J* = 6.9 Hz, 2H), 2.46 (s, 3H), 1.56 – 1.47 (m, 2H). $^{13}C\{^{1}H\}$ NMR (151 MHz, DMSO-d_6) δ 170.2, 170.1, 163.6, 140.0, 130.2, 128.8, 126.6, 122.7, 118.4, 116.3, 99.2, 70.2, 58.3, 57.7, 53.6, 45.4, 29.9, 12.9. HRMS (ESI/QTOF) m/z: [M + H]$^+$ Calcd for $C_{18}H_{23}N_4O_5^+$: 375.1663; Found: 375.1660.

2-(2-{4-[(3,5-Dimethyl-1,2-oxazol-4-yl)methoxy]-3-methoxyphenyl}-3-{[2-(methylsulfanyl)ethyl]amino}imidazo[1,2-*a*]pyridin-6-yl)ethan-1-ol (4{125,124,31}). Yield 76 mg, 0.157 mmol, 58%. Yellow amorphous powder. ^{1}H NMR (500 MHz, DMSO-d_6) δ 8.17 (s, 1H), 7.79 – 7.69 (m, 2H), 7.40 (d, *J* = 9.1 Hz, 1H), 7.14 – 7.07 (m, 2H), 4.93 (s, 2H), 4.92 – 4.88 (m, 1H), 4.73 (br t, *J* = 5.2 Hz, 1H), 3.84 (s, 3H), 3.67 (q, *J* = 6.3 Hz, 2H), 3.13 (q, *J* = 6.7 Hz, 2H), 2.75 (t, *J* = 6.7 Hz, 2H), 2.61 (t, *J* = 7.0 Hz, 2H), 2.38 (s, 3H), 2.23 (s, 3H), 2.03 (s, 3H). $^{13}C\{^{1}H\}$ NMR (151 MHz, DMSO-d_6) δ 167.4, 159.6, 149.6, 146.3, 139.6, 134.2, 128.7, 126.3, 125.3, 122.7, 121.1, 118.7, 116.0, 115.2, 110.6, 110.4, 61.5, 60.0, 55.5, 46.8, 35.8, 34.0, 14.8, 10.5, 9.6. HRMS (ESI/QTOF) m/z: [M + H]$^+$ Calcd for $C_{25}H_{31}N_4O_4S^+$: 483.2061; Found: 483.2057.

2-{3-[(3,3-Difluorocyclobutyl)amino]-2-(3,5-dimethoxyphenyl)imidazo[1,2-*a*]pyridin-6-yl}-1*λ*^{6},2-thiazolidine-1,1-dione (4{64,611,49}). Yield 75 mg, 0.157 mmol, 58%. Colourless amorphous powder. ^{1}H NMR (500 MHz, DMSO-*d*$_6$) δ 8.17 (s, 1H), 7.58 (d, *J* = 9.6 Hz, 1H), 7.37 (s, 2H), 7.31 (dd, *J* = 9.6, 2.2 Hz, 1H), 6.46 (s, 1H), 5.52 (d, *J* = 6.5 Hz, 1H), 3.80 and 3.77 (s+m, 8H), 3.69 – 3.59 (br m, 1H), 3.55 (t, *J* = 7.5 Hz, 2H), 2.84 – 2.72 (m, 2H), 2.60– 2.41 (s+m, 4H+DMSO-*d*$_6$). ^{13}C{^{1}H} NMR (151 MHz, DMSO-*d*$_6$) δ 160.5, 138.7, 136.0, 135.7, 125.3, 120.8, 119.3 (dd, *J* = 282.3, 269.0 Hz), 117.3, 115.1, 104.4, 99.7, 55.2, 47.5, 47.4, 42.7 (t, *J* = 22.6 Hz), 41.9 (dd, *J* = 17.2, 6.4 Hz), 18.6. ^{19}F{^{1}H} NMR (376 MHz, DMSO-*d*$_6$) δ –82.3 (d, *J* = 194.2 Hz), -96.6 (d, *J* = 194.1 Hz). HRMS (ESI/QTOF) m/z: [M + H]$^+$ Calcd for $C_{22}H_{25}F_2N_4O_4S^+$: 479.1559; Found: 479.1556.

***N*-Methyl-3-[(oxolan-3-yl)amino]-2-[3-(pyridin-2-yloxy)phenyl]imidazo[1,2-*a*]pyridine-6-carboxamide (4{193,290,10}).** Yield 75 mg, 0.174 mmol, 65%. Yellow amorphous powder. ^{1}H NMR (500 MHz, DMSO-*d*$_6$) δ 8.83 (s, 1H), 8.61 (q, *J* = 4.5 Hz, 1H), 8.20 – 8.13 (m, 1H), 8.07 (d, *J* = 7.9 Hz, 1H), 7.96 (s, 1H), 7.91 – 7.84 (m, 1H), 7.63 (dd, *J* = 9.4, 1.8 Hz, 1H), 7.53 (d, *J* = 9.3 Hz, 1H), 7.49 (t, *J* = 7.9 Hz, 1H), 7.18 – 7.12 (m, 1H), 7.11 – 7.05 (m, 2H), 5.14 (d, *J* = 4.5 Hz, 1H), 3.91 – 3.82 (m, 1H), 3.69 – 3.61 (m, 1H), 3.55 (dd, *J* = 9.0, 5.0 Hz, 1H), 3.49 (dd, *J* = 8.9, 3.0 Hz, 1H) 2.83 (d, *J* = 4.5 Hz, 3H), 1.90 – 1.79 (m, 1H), 1.75 – 1.65 (m, 1H). ^{13}C{^{1}H} NMR (151 MHz, DMSO-*d*$_6$) δ 164.5, 163.1, 154.2, 147.5, 140.8, 140.2, 135.9, 135.7, 129.7, 126.4, 124.4, 122.8, 122.7, 120.0, 119.4, 119.3, 119.0, 116.1, 111.6, 72.3, 66.4, 56.9, 32.5, 26.3. HRMS (ESI/QTOF) m/z: [M + H]$^+$ Calcd for $C_{24}H_{24}N_5O_3^+$: 430.1874; Found: 430.1871.

2-{[2-(5-Methoxy-4-methylpyridin-3-yl)-3-{[(3-methoxyphenyl)methyl]amino}imidazo[1,2-*a*]pyridin-6-yl]oxy}ethan-1-ol (4{107,291,24})

Yield 75 mg, 0.172 mmol, 64%. Colourless viscous oil. ^{1}H NMR (600 MHz, DMSO-d_6) δ 8.23 (s, 1H), 8.20 (s, 1H), 7.87 (s, 1H), 7.36 (d, *J* = 9.6 Hz, 1H), 7.03 (t, *J* = 7.8 Hz, 1H), 6.93 (dd, *J* = 9.7, 2.2 Hz, 1H), 6.67 (dd, *J* = 8.1, 2.5 Hz, 1H), 6.59 – 6.53 (m, 2H), 5.50 (t, *J* = 6.2 Hz, 1H), 4.97 (t, *J* = 5.5 Hz, 1H), 3.97 (t, *J* = 4.9 Hz, 2H), 3.93 (s, 3H), 3.90 (d, *J* = 6.2 Hz, 2H), 3.76 (q, *J* = 5.0 Hz, 2H), 3.60 (s, 3H), 2.03 (s, 3H). ^{13}C{^{1}H} NMR (151 MHz, DMSO-d_6) δ 159.0, 153.6, 147.3, 143.4, 141.5, 137.1, 133.6, 131.3, 131.1, 130.5, 128.9, 128.8, 119.7, 118.2, 117.2, 112.8, 112.5, 105.2, 70.7, 59.4, 56.2, 54.7, 50.4, 12.3. HRMS (ESI/QTOF) m/z: $[M + H]^+$ Calcd for $C_{24}H_{27}N_4O_4^+$: 435.2027; Found: 435.2018.

***tert*-Butyl 12-{[(oxolan-3-yl)methyl]amino}-11-{[1,2,4]triazolo[1,5-*a*]pyridin-7-yl}-1,4,10-triazatricyclo[7.3.0.0^{2,6}]dodeca-2(6),7,9,11-tetraene-4-carboxylate (4{43,21,12}).** Yield 76 mg, 0.161 mmol, 58%. Beige amorphous solid. ^{1}H NMR (500 MHz, DMSO-d_6) δ 8.98 (d, *J* = 7.2 Hz, 1H), 8.49 (s, 1H), 8.42 (s, 0.5×1H), 8.39 (s, 0.5×1H), 7.90 (t, *J* = 6.6 Hz, 2H), 7.49 (d, *J* = 9.2 Hz, 1H), 7.26 (dd, *J* = 9.2, 6.3 Hz, 1H), 5.19 (s, 1H), 5.16 (s, 1H), 5.00 (s, 0.5×1H), 4.94 (s, 0.5×1H), 4.56 (s, 1H), 4.53 (s, 1H), 3.81 (s, 0.5×1H), 3.71 (s, 0.5×1H), 3.68 – 3.54 (m, 1H), 3.48 (s, 0.5×1H), 3.39 (s, 0.5×1H), 2.91 (s, 2H), 2.40 (p, *J* = 7.1 Hz, 1H), 2.10 – 1.87 (m, 1H), 1.65 – 1.51 (m, 1H), 1.48 (s, 0.5×9H), 1.47 (s, 0.5×9H). ^{13}C{^{1}H} NMR (151 MHz, DMSO-d_6) δ 154.4, 153.4, 150.3, 141.3, 136.1, 133.6, 128.7 (d, *J* = 6.7 Hz), 128.5 (d, *J* = 10.4 Hz), 121.3, 116.4 (d, *J* = 14.3 Hz), 113.1 (d, *J* = 12.6 Hz), 111.8, 111.6, 79.3, 70.9, 66.8, 66.8, 52.7 (d, *J* = 42.5 Hz), 50.4 – 49.7 (m), 30.0, 30.0, 28.1. HRMS (ESI/QTOF) m/z: $[M + H]^+$ Calcd for $C_{25}H_{30}N_7O_3^+$: 476.2405; Found: 476.2401.

2-(3,6-Dibromopyridin-2-yl)-3-[(oxan-4-yl)amino]imidazo[1,2-*a*]pyridine-7-carboxamide (4{92,361,7}). Yield 76 mg, 0.153 mmol, 57%. Yellowish amorphous

powder. ^{1}H NMR (500 MHz, DMSO-d_6) δ 8.32 (d, *J* = 7.2 Hz, 1H), 8.16 – 8.10 (m, 3H), 7.58 (d, *J* = 8.3 Hz, 1H), 7.52 (br s, 1H), 7.36 (dd, *J* = 7.3, 1.7 Hz, 1H), 5.46 (d, *J* = 8.1 Hz, 1H), 3.81 – 3.73 (m, 2H), 3.23 – 3.14 (m, 2H), 3.30 – 3.25 (m, 1H), 1.71 (d, *J* = 12.2 Hz, 2H), 1.44 – 1.32 (m, 2H). $^{13}C\{^{1}H\}$ NMR (151 MHz, DMSO-d_6) δ 166.3, 152.6, 144.9, 138.3, 138.1, 131.3, 130.7, 128.9, 127.7, 123.0, 118.7, 116.9, 110.6, 65.7, 52.3, 33.6. HRMS (ESI/QTOF) m/z: [M + H]$^+$ Calcd for $C_{18}H_{18}Br_2N_5O_2^+$: 493.9822; Found: 495.9798.

1,5-Dimethyl 2-{[6-bromo-2-(5-cyano-1-methyl-1*H*-pyrrol-3-yl)-8-methylimidazo[1,2-*a*]pyridin-3-yl]amino}pentanedioate (4{435,219,23}). Yield 75 mg, 0.153 mmol, 57%. Yellow amorphous solid. ^{1}H NMR (500 MHz, DMSO-d_6) δ 8.37 (s, 1H), 7.63 (d, *J* = 1.8 Hz, 1H), 7.31 (d, *J* = 1.8 Hz, 1H), 7.12 (s, 1H), 5.49 (d, *J* = 8.5 Hz, 1H), 3.82 (s, 3H), 3.72 (q, *J* = 7.3 Hz, 1H), 3.62 (s, 3H), 3.42 (s, 3H), 2.64 – 2.44 (m, 1H), 2.44 (s, 3H), 2.15 – 1.99 (m, 2H). $^{13}C\{^{1}H\}$ NMR (151 MHz, DMSO-d_6) δ 173.6, 172.8, 139.2, 130.6, 127.2, 127.1, 124.8, 124.1, 121.3, 118.1, 117.4, 113.7, 104.9, 103.6, 58.2, 51.7, 51.4, 35.2, 29.4, 27.4, 15.8. HRMS (ESI/QTOF) m/z: [M + H]$^+$ Calcd for $C_{21}H_{23}BrN_5O_4^+$: 488.0928; Found: 488.0918.

5-(Difluoromethyl)-2-{1,3-dimethyl-1*H*-pyrazolo[3,4-*b*]pyridin-5-yl}-*N*-[(oxolan-3-yl)methyl]imidazo[1,2-*a*]pyridin-3-amine (4{37,596,12}). Yield 74 mg, 0.179 mmol, 66%. Colourless amorphous powder. ^{1}H NMR (500 MHz, DMSO-d_6) δ 9.18 (d, *J* = 2.0 Hz, 1H), 8.81 (d, *J* = 2.1 Hz, 1H), 8.13 (t, *J* = 55 Hz, 1H), 7.75 – 7.68 (m, 1H), 7.35 – 7.28 (m, 2H), 5.04 (t, *J* = 5.8 Hz, 1H), 4.02 (s, 3H), 3.69 – 3.58 (m, 2H), 3.54 (q, *J* = 7.5 Hz, 1H), 3.35 – 3.29 (m, 1H), 2.83 – 2.71 (m, 2H), 2.55 (s, 3H), 2.43 – 2.31 (m, 1H), 1.95 – 1.85 (m, 1H), 1.45 (m, 1H). $^{13}C\{^{1}H\}$ NMR (151 MHz, DMSO-d_6) δ 149.9, 148.2, 141.4, 140.3, 136.6, 131.2, 131.0 (t, *J* = 25.6 Hz), 130.9, 128.0, 127.7, 123.2, 122.3, 119.7, 114.3, 111.2 (t, *J* = 8.0 Hz), 109.4 (t, *J* = 236.4 Hz), 70.8, 66.8, 51.0, 39.1, 33.3,

29.9, 12.00. $^{19}F\{^1H\}$ NMR (376 MHz, DMSO-*d*6) δ –115.0 (d, *J* = 290.4 Hz), -116.0 (d, *J* = 288.1 Hz). HRMS (ESI/QTOF) m/z: $[M + H]^+$ Calcd for $C_{21}H_{23}F_2N_6O^+$: 413.1896; Found: 413.1893.

1-(2-{4-[(3,5-Dimethyl-1,2-oxazol-4-yl)methoxy]-3-methoxyphenyl}-3-{[2-(methylsulfanyl)ethyl]amino}imidazo[1,2-*a*]pyridin-5-yl)ethan-1-ol (4{118,124,31}). Yield 74 mg, 0.153 mmol, 57%. Yellowish viscous oil. ^1H NMR (500 MHz, DMSO-*d*6) δ 8.17 (s, 1H), 7.76 (d, *J* = 2.0 Hz, 1H), 7.72 (dd, *J* = 8.3, 2.0 Hz, 1H), 7.40 (d, *J* = 9.1 Hz, 1H), 7.18 – 7.01 (m, 2H), 4.98 – 4.87 (m, 3H), 4.72 (t, *J* = 5.2 Hz, 1H), 3.84 (s, 3H), 3.66 (t, *J* = 6.0 Hz, 2H), 3.13 (q, *J* = 6.7 Hz, 2H), 2.75 (t, *J* = 6.7 Hz, 2H), 2.61 (t, *J* = 7.0 Hz, 2H), 2.38 (s, 3H), 2.23 (s, 3H), 2.03 (s, 3H). $^{13}C\{^1H\}$ NMR (151 MHz, DMSO-*d*6) δ 167.4, 159.6, 149.6, 146.5, 144.3, 141.8, 136.1, 128.6, 127.3, 123.4, 119.3, 115.9, 115.1, 110.9, 110.6, 108.4, 62.6, 60.0, 55.6, 48.5, 33.4, 22.8, 14.8, 10.5, 9.6. HRMS (ESI/QTOF) m/z: $[M + H]^+$ Calcd for $C_{25}H_{31}N_4O_4S^+$: 483.2061; Found: 483.2050.

Methyl 4-{3-[(3-ethoxy-3-oxopropyl)amino]-5-(pyridin-4-yl)imidazo[1,2-*a*]pyridin-2-yl}pentanoate trifluoroacetate (4{333,152,29}). Yield 73 mg, 0.136 mmol, 64%. Colourless viscous oil. ^1H NMR (500 MHz, DMSO-*d*6) δ 8.81 – 8.76 (m, 2H), 7.99 (d, *J* = 9.0 Hz, 1H), 7.93 (dd, *J* = 8.9, 7.0 Hz, 1H), 7.80 – 7.75 (m, 2H), 7.41 – 7.34 (m, 1H), 4.54 (t, *J* = 6.3 Hz, 1H), 3.92 (q, *J* = 7.1 Hz, 1H), 3.54 (s, 3H), 3.19 – 3.07 (m, 1H), 2.44 – 2.31 (m, 3H), 2.33 – 2.21 (m, 1H), 2.00 – 1.90 (m, 2H), 1.90 – 1.73 (m, 2H), 1.32 (d, *J* = 7.1 Hz, 3H), 1.11 (t, *J* = 7.1 Hz, 3H). $^{13}C\{^1H\}$ NMR (126 MHz, DMSO-*d*6) δ 173.0, 171.1, 158.4 (q, *J* = 35.0 Hz), 147.2, 141.3, 137.8, 136.9, 132.9, 128.6, 125.1, 120.0, 116.00 (d, *J* = 292.9 Hz), 112.4, 59.9, 51.3, 43.6, 33.4, 31.3, 30.5, 28.4, 19.8, 14.0. HRMS (ESI/QTOF) m/z: $[M + H]^+$ Calcd for $C_{23}H_{29}N_4O_4^+$: 425.2183; Found: 425.2182.

3-{4-[3-(*tert*-Butylamino)-6-[1-(trifluoromethyl)cyclopropyl]imidazo[1,2-*a*]pyridin-2-yl]-1*H*-pyrazol-1-yl}-1*λ*^{6}-thiolane-1,1-dione (4{52,28,18}). Yield 74 mg, 0.153 mmol, 57%. Colourless amorphous solid. ^{1}H NMR (500 MHz, DMSO-*d*$_6$) δ 8.38 (s, 2H), 8.11 (s, 1H), 7.41 (d, *J* = 9.2 Hz, 1H), 7.20 (d, *J* = 9.3 Hz, 1H), 5.37 – 5.27 (m, 1H), 4.61 (s, 1H), 3.78 (dd, *J* = 13.7, 8.2 Hz, 1H), 3.50 (dd, *J* = 13.7, 7.4 Hz, 1H), 3.48 – 3.39 (m, 1H), 3.33 – 3.23 (m, 1H), 2.73 – 2.61 (m, 1H), 2.61 – 2.52 (m, 1H), 1.41 (s, 2H), 1.18 (s, 2H), 1.08 (s, 9H). ^{13}C{^{1}H} NMR (151 MHz, DMSO-*d*$_6$) δ 140.7, 138.3, 133.6, 127.8, 127.4, 126.2, 126.1, 125.6, 123.4, 122.9, 118.5, 116.9, 115.8, 56.2, 56.0, 54.9, 50.6, 50.6, 30.1, 29.3, 25.4 (q, *J* = 34.9 Hz), 9.6 (t, *J* = 58.7 Hz). HRMS (ESI/QTOF) m/z: [M + H]$^+$ Calcd for $C_{22}H_{27}F_3N_5O_2S^+$: 482.1832; Found: 482.1828.

2-[4-(Dimethylamino)phenyl]-*N*-methyl-3-{[(oxolan-3-yl)methyl]amino}imidazo[1,2-*a*]pyridine-5-carboxamide (4{362,594,12}). Yield 73 mg, 0.185 mmol, 69%. Colourless amorphous powder. ^{1}H NMR (500 MHz, DMSO-*d*$_6$) δ 9.10 (d, *J* = 14.0 Hz, 1H), 8.06 (d, *J* = 8.9 Hz, 2H), 7.59 (d, *J* = 8.9 Hz, 1H), 7.19 (s, 1H), 7.04 (d, *J* = 6.8 Hz, 1H), 6.78 (d, *J* = 9.3 Hz, 2H), 3.92 (s, 1H), 3.65 (s, 2H), 3.56 (s, 1H), 3.30 (s, 1H), 2.95 (s, 6H), 2.90 (d, *J* = 4.5 Hz, 3H), 2.69 (d, *J* = 21.2 Hz, 2H), 2.24 (s, 1H), 1.89 (d, *J* = 7.7 Hz, 1H), 1.42 (s, 1H). ^{13}C{^{1}H} NMR (151 MHz, DMSO-*d*$_6$) δ 164.3, 149.5, 141.1 136.4, 131.2, 127.7, 126.3, 122.1, 122.0, 118.4, 113.5, 111.9, 70.7, 66.8, 51.4, 40.0, 29.8, 26.3. HRMS (ESI/QTOF) m/z: [M + H]$^+$ Calcd for $C_{22}H_{28}N_5O_2^+$: 394.2238; Found: 394.2236.

1,5-Dimethyl 2-{[2-(5-cyano-1-methyl-1*H*-pyrrol-3-yl)-5-(pyridin-4-yl)imidazo[1,2-*a*]pyridin-3-yl]amino}pentanedioate (4{333,219,23}). Yield 73 mg, 0.154 mmol, 57%. Colourless amorphous solid. ^{1}H NMR (600 MHz, DMSO-*d*$_6$) δ 8.63 (d, *J* = 5.3 Hz, 2H), 7.72 (d, *J* = 1.7 Hz, 1H), 7.57 (d, *J* = 5.2 Hz, 2H), 7.54 (d, *J* = 8.9 Hz, 1H), 7.41 (d, *J* = 1.7 Hz, 1H), 7.27 (dd, *J* = 8.9, 6.9 Hz, 1H), 6.80 (d, *J* = 6.9 Hz, 1H), 4.49

(d, J = 5.9 Hz, 1H), 3.80 (s, 3H), 3.49 (s, 3H), 3.20 (s, 3H), 3.00 – 2.94 (m, 1H), 2.54 (s, 0H), 1.93 – 1.79 (m, 2H), 1.48 – 1.39 (m, 1H), 1.07 – 0.97 (m, 1H). $^{13}C\{^1H\}$ NMR (151 MHz, DMSO-d_6) δ 172.2, 171.6, 148.6, 142.1, 141.5, 135.1, 134.3, 127.6, 123.5, 123.4, 118.2, 118.2, 116.9, 115.9, 113.8, 103.3, 58.1, 51.3, 51.2, 35.2, 28.9, 26.0. HRMS (ESI/QTOF) m/z: $[M + H]^+$ Calcd for $C_{25}H_{25}N_6O_4^+$: 473.1932; Found: 473.1943.

Methyl (1r,3r)-3-(3-{3-[(3-methoxypropyl)amino]-6-phenylimidazo[1,2-*a*]pyrazin-2-yl}-1*H*-pyrazol-1-yl)cyclobutane-1-carboxylate (4{350,165,5}). Yield 72 mg, 0.157 mmol, 58%. Colourless amorphous powder. ^1H NMR (500 MHz, DMSO-d_6) δ 8.97 (s, 1H), 8.68 (s, 1H), 8.09 (d, J = 7.8 Hz, 2H), 7.97 – 7.89 (m, 1H), 7.51 (t, J = 7.7 Hz, 2H), 7.41 (t, J = 7.3 Hz, 1H), 6.76 – 6.71 (m, 1H), 5.44 (t, J = 6.8 Hz, 1H), 5.17 – 5.06 (m, 1H), 3.70 (s, 3H), 3.46 (t, J = 6.2 Hz, 2H), 3.40 – 3.33 (m, 2H), 3.31 – 3.21 (m, 1H), 3.19 (s, 3H), 2.93 – 2.83 (m, 2H), 2.75 – 2.65 (m, 2H), 1.84 – 1.75 (m, 2H). $^{13}C\{^1H\}$ NMR (151 MHz, DMSO-d_6) δ 175.3, 146.6, 141.7, 137.3, 136.6, 134.7, 130.4, 130.1, 129.2, 128.7, 128.3, 125.9, 111.7, 103.9, 69.5, 57.8, 53.2, 51.8, 43.2, 39.8, 32.6, 31.9, 30.0. HRMS (ESI/QTOF) m/z: $[M + H]^+$ Calcd for $C_{25}H_{29}N_6O_3^+$: 461.2296; Found: 461.2292.

2-{4-[8-(3-Cyanopropoxy)-3-{[(oxolan-3-yl)methyl]amino}imidazo[1,2-*a*]pyridin-2-yl]phenoxy}-*N*-ethylacetamide (4{371,622,12}). Yield 73 mg, 0.153 mmol, 57%. Yellowish viscous oil. ^1H NMR (500 MHz, DMSO-d_6) δ 8.13 (s, 1H), 8.04 (d, J = 9.0 Hz, 2H), 7.93 (d, J = 6.7 Hz, 1H), 7.05 (d, J = 8.8 Hz, 2H), 6.85 (s, 1H), 6.72 (d, J = 7.5 Hz, 1H), 4.95 (t, J = 6.0 Hz, 1H), 4.50 (s, 2H), 4.27 (t, J = 6.2 Hz, 2H), 3.65 (s, 2H), 3.55 (d, J = 7.7 Hz, 1H), 3.41 (s, 1H), 3.18 (s, 2H), 2.95 – 2.78 (m, 2H), 2.74 (s, 2H), 2.29 (s, 1H), 2.14 (s, 2H), 1.88 (s, 1H), 1.49 (d, J = 4.5 Hz, 1H), 1.05 (t, J = 7.1 Hz, 3H). $^{13}C\{^1H\}$ NMR (151 MHz, DMSO-d_6) δ 167.2, 157.0, 146.8, 134.2, 131.7, 128.00, 126.6, 120.2, 116.3, 115.2, 114.6, 111.8, 111.8, 70.9, 67.1, 66.8, 66.2, 50.5, 40.4,

33.2, 29.9, 24.7, 14.8, 13.4. HRMS (ESI/QTOF) m/z: $[M + H]^+$ Calcd for $C_{26}H_{32}N_5O_4^+$: 478.2449; Found: 478.2446.

Ethyl 4-{[5-(propylsulfanyl)-2-(thiophen-3-yl)imidazo[1,2-*a*]pyridin-3-yl]amino}butanoate trifluoroacetate (4{33,9,6}). Yield 72 mg, 0.139 mmol, 66%. Yellow amorphous solid. ^{1}H NMR (500 MHz, DMSO-d_6) δ 8.22 – 8.18 (m, 1H), 7.84 – 7.78 (m, 1H), 7.74 (d, *J* = 5.0 Hz, 1H), 7.67 (t, *J* = 8.2 Hz, 1H), 7.54 (d, *J* = 8.7 Hz, 1H), 7.17 (d, *J* = 7.7 Hz, 1H), 5.21 (t, *J* = 5.7 Hz, 1H), 4.02 (q, *J* = 7.1 Hz, 2H), 3.13 (t, *J* = 7.1 Hz, 2H), 2.96 (q, *J* = 6.5 Hz, 2H), 2.37 (t, *J* = 7.4 Hz, 2H), 1.85 – 1.71 (m, 4H), 1.14 (t, *J* = 7.1 Hz, 3H), 1.08 (t, *J* = 7.3 Hz, 3H). $^{13}C\{^1H\}$ NMR (101 MHz, DMSO-d_6) δ 173.1, 141.3, 139.4, 131.9, 128.8, 128.4, 126.9, 126.6, 125.6, 113.2, 108.3, 60.3, 50.1, 34.3, 31.6, 24.7, 21.2, 14.6, 13.8. HRMS (ESI/QTOF) m/z: $[M + H]^+$ Calcd for $C_{20}H_{26}N_3O_2S_2^+$: 404.1461; Found: 404.1458.

Ethyl 4-{[11-(4-methylpyridin-2-yl)-5-oxa-1,10-diazatricyclo[7.3.0.0^{2,6}]dodeca-2(6),7,9,11-tetraen-12-yl]amino}butanoate trifluoroacetate (4{31,10,6}). Yield 72 mg, 0.189 mmol, 70%. Beige amorphous solid. ^{1}H NMR (500 MHz, DMSO-d_6) δ 8.61 (d, *J* = 5.1 Hz, 1H), 8.12 (s, 1H), 7.59 (q, *J* = 9.5 Hz, 2H), 7.38 (d, *J* = 5.1 Hz, 1H), 5.35 (br s, 1H), 4.82 (t, *J* = 9.1 Hz, 2H), 4.03 (q, *J* = 7.1 Hz, 2H), 3.95 (t, *J* = 9.1 Hz, 2H), 3.02 (t, *J* = 7.2 Hz, 2H), 2.46 (s, 3H), 2.42 (t, *J* = 7.3 Hz, 2H), 1.87 – 1.78 (m, 2H), 1.14 (t, *J* = 7.1 Hz, 3H). $^{13}C\{^1H\}$ NMR (151 MHz, DMSO-d_6) δ 172.6, 150.2, 149.4, 148.7, 135.4, 129.6, 127.1, 125.4, 124.9, 123.6, 122.4, 118.9, 111.8, 71.7, 59.8, 49.5, 46.4, 30.9, 28.2, 24.8, 20.8, 14.1. $^{19}F\{^1H\}$ NMR (376 MHz, DMSO-d_6) δ –74.4. HRMS (ESI/QTOF) m/z: $[M + H]^+$ Calcd for $C_{21}H_{25}N_4O_3^+$: 381.1921; Found: 381.1921.

2-{2-[4-Methoxy-3-(prop-2-yn-1-yloxy)phenyl]-3-[(oxetan-3-yl)amino]imidazo[1,2-*a*]pyridin-5-yl}propan-2-ol (4{153,289,22}). Yield 72 mg,

0.176 mmol, 65%. Colourless viscous oil. ^{1}H NMR (500 MHz, DMSO-d_6) δ 7.45 – 7.36 (m, 3H), 7.35 (dd, *J* = 8.3, 2.0 Hz, 1H), 7.08 – 7.00 (m, 3H), 6.85 (d, *J* = 7.1 Hz, 1H), 4.84 (s, 2H), 4.37 (t, *J* = 6.6 Hz, 2H), 4.29 (t, *J* = 6.7 Hz, 2H), 4.16 – 4.05 (m, 1H), 3.82 (s, 3H), 1.65 (s, 6H). ^{13}C{^{1}H} NMR (151 MHz, DMSO-d_6) δ 148.7, 146.2, 144.3, 142.3, 134.3, 127.5, 127.2, 122.2, 121.6, 117.1, 114.1, 111.9, 109.7, 79.5, 78.4, 78.2, 70.6, 56.2, 55.6, 54.2, 48.6, 30.3. HRMS (ESI/QTOF) m/z: [M + H]$^+$ Calcd for $C_{23}H_{26}N_3O_4^+$: 408.1918; Found: 408.1913.

5-{3-[(3,3-Difluorocyclobutyl)amino]-6-(4-methyl-3-oxopiperazin-1-yl)imidazo[1,2-*a*]pyridin-2-yl}-2-fluoro-*N*-methylbenzamide (4{352,301,49}). Yield 74 mg, 0.151 mmol, 56%. Colourless amorphous powder. ^{1}H NMR (500 MHz, DMSO-d_6) δ 8.37 (d, *J* = 7.0 Hz, 1H), 8.29 – 8.21 (m, 2H), 7.67 (s, 1H), 7.41 (d, *J* = 9.7 Hz, 1H), 7.34 – 7.24 (m, 2H), 5.37 (d, *J* = 6.8 Hz, 1H), 3.70 (s, 2H), 3.62 – 3.54 (m, 1H), 3.44 (s, 4H), 2.90 (s, 3H), 2.81 – 2.72 (s+m, 5H), 2.57 – 2.50 (m, 2H+DMSO-d_6). ^{13}C{^{1}H} NMR (151 MHz, DMSO-d_6) δ 165.0, 164.1, 158.0 (d, *J* = 249.1 Hz), 137.8, 137.3, 133.3, 131.0, 129.6 (d, *J* = 8.3 Hz), 127.8, 125.1, 123.9 (d, *J* = 15.0 Hz), 121.2, 120.2, 119.4 (d, *J* = 14.8 Hz), 117.1, 116.1 (d, *J* = 22.8 Hz), 107.2, 52.9, 47.6, 46.4, 42.7 (t, *J* = 21.5 Hz), 42.3 (dd, *J* = 17.3, 5.5 Hz), 33.3, 26.3. ^{19}F{^{1}H} NMR (376 MHz, DMSO-d_6) δ –82.3 (d, *J* = 194.4 Hz), -97.3 (d, *J* = 194.3 Hz), -117.4. HRMS (ESI/QTOF) m/z: [M + H]$^+$ Calcd for $C_{24}H_{26}F_3N_6O_2^+$: 487.2064; Found: 487.2062.

2-(2-Methoxythiophen-3-yl)-*N*-(oxan-4-yl)-6-(1,2,2,2-tetrafluoroethyl)imidazo[1,2-*a*]pyridin-3-amine (4{369,54,7}). Yield 73 mg, 0.170 mmol, 61%. Yellowish viscous oil. ^{1}H NMR (500 MHz, DMSO-d_6) δ 8.60 (s, 1H), 7.59 (d, *J* = 9.3 Hz, 1H), 7.33 – 7.20 (m, 2H), 6.94 (d, *J* = 5.8 Hz, 1H), 6.73 – 6.46 (m, 1H), 4.44 (d, *J* = 6.7 Hz, 1H), 4.00 (s, 3H), 3.76 (ddt, *J* = 12.0, 7.9, 3.7 Hz, 2H), 3.23 – 3.11 (m, 2H), 3.05 – 2.94 (m, 1H), 1.70 – 1.53 (m, 2H), 1.42 – 1.21 (m, 2H). ^{13}C{^{1}H} NMR

(101 MHz, DMSO-d_6) δ 160.2, 140.3, 132.3, 127.0, 125.6, 124.4 (d, *J* = 9.7 Hz), 123.8 (d, *J* = 32.0 Hz) and 121.2 (d, *J* = 30.6 Hz), 120.7, 120.7, 117.3, 114.8, 114.3, 114.1, 112.2, 87.4 (d, *J* = 35.0 Hz) and 85.6 (d, *J* = 32.6 Hz) , 65.7 (d, *J* = 3.0 Hz), 62.2 (d, *J* = 2.0 Hz), 53.0, 33.4 (d, *J* = 2.0 Hz). $^{19}F\{^1H\}$ NMR (376 MHz, DMSO-d_6) δ –77.7 (d, *J* = 13.8 Hz), -192.7 (q, *J* = 13.8 Hz). Anal. calcd. for $C_{19}H_{19}F_4N_3O_2S$: C 53.14; H 4.46; N 9.79; S 7.47. Found: C 53.51; H 4.68; N 9.72; S 7.76.

5-(Dimethylphosphoryl)-2-(1-ethyl-1*H*-indazol-3-yl)-*N*-(oxan-4-yl)imidazo[1,2-*a*]pyridin-3-amine (4{50,604,7}). Yield 73 mg, 0.167 mmol, 60%. Colourless viscous oil. ^1H NMR (500 MHz, DMSO-d_6) δ 8.31 (d, *J* = 8.1 Hz, 1H), 7.74 – 7.65 (m, 2H), 7.41 (t, *J* = 7.0 Hz, 1H), 7.32 (dd, *J* = 10.7, 7.0 Hz, 1H), 7.17 (t, *J* = 7.5 Hz, 1H), 7.11 (t, *J* = 8.0 Hz, 1H), 6.64 (d, *J* = 4.1 Hz, 1H), 4.51 (q, *J* = 7.1 Hz, 2H), 4.05 – 3.95 (m, 1H), 3.67 (d, *J* = 10.9 Hz, 2H), 2.94 (t, *J* = 10.9 Hz, 2H), 2.03 (d, *J* = 13.9 Hz, 6H), 1.54 (d, *J* = 12.6 Hz, 2H), 1.48 (t, *J* = 7.1 Hz, 3H), 1.36 – 1.24 (m, 2H). $^{13}C\{^1H\}$ NMR (151 MHz, DMSO-d_6) δ 140.3, 140.3, 139.4, 138.7, 131.8 (d, *J* = 96.5 Hz), 129.2, 126.2, 122.8, 122.7, 121.2, 120.5, 120.3 (d, *J* = 10.9 Hz), 120.0 (d, *J* = 13.1 Hz), 109.4, 65.9, 49.7, 43.0, 32.8, 19.6 (d, *J* = 75.4 Hz), 15.0. HRMS (ESI/QTOF) m/z: $[M + H]^+$ Calcd for $C_{23}H_{29}N_5O_2P^+$: 438.2053; Found: 438.2049.

7-(Dimethylphosphoryl)-2-(6-methoxypyridin-3-yl)-*N*-(oxan-4-yl)imidazo[1,2-*a*]pyridin-3-amine (4{32,12,7}). Yield 709 mg, 0.177 mmol, 66%. Yellowish amorphous solid. ^1H NMR (500 MHz, DMSO-d_6) δ 8.97 (s, 1H), 8.50 – 8.43 (m, 2H), 7.90 (d, *J* = 13.6 Hz, 1H), 7.20 (t, *J* = 7.8 Hz, 1H), 6.93 (d, *J* = 8.6 Hz, 1H), 5.10 (d, *J* = 6.0 Hz, 1H), 3.90 (s, 3H), 3.81 – 3.74 (m, 2H), 3.18 (t, *J* = 11.6 Hz, 1H), 3.13 – 3.03 (m, 1H), 1.72 (d, *J* = 13.4 Hz, 6H), 1.63 (d, *J* = 11.2 Hz, 2H), 1.55 – 1.43 (m, 2H). $^{13}C\{^1H\}$ NMR (101 MHz, DMSO-d_6) δ 162.7, 145.0, 139.2 (d, *J* = 15.2 Hz), 137.3, 134.6 (*J* = 97.8 Hz), 130.7, 125.7, 123.8, 123.4 (*J* = 12.1 Hz), 119.3 (*J* = 10.1 Hz),

110.8 (J = 11.2 Hz), 110.3, 65.7, 53.6, 53.3, 33.6, 17.4 (d, J = 71.7 Hz). HRMS (ESI/QTOF) m/z: $[M + H]^+$ Calcd for $C_{20}H_{26}N_4O_3P^+$: 401.1737; Found: 401.1732.

***tert*-Butyl *N*-(2-{[2-(1-acetylpiperidin-3-yl)-7-(methylcarbamoyl)imidazo[1,2-*a*]pyridin-3-yl]amino}ethyl)carbamate (4{69,33,51}).** Yield 71 mg, 0.154 mmol, 57%. Yellow viscous oil. ^{1}H NMR (500 MHz, DMSO-d_6; compound exists as mixture of rotamers ca. 1:1) δ 8.56 – 8.49 (m, 1H), 8.19 (dd, J = 7.2, 3.4 Hz, 1H), 7.91 (d, J = 7.0 Hz, 1H), 7.26 (t, J = 6.8 Hz, 1H), 6.95 – 6.86 (m, 1H), 4.84 – 4.73 (m, 1H), 4.44 (t, J = 12.9 Hz, 1H), 4.10 (q, J = 5.2 Hz, 0.5×1H), 3.87 (d, J = 13.4 Hz, 0.5×1H), 3.79 (dd, J = 13.4, 4.0 Hz, 1H), 3.33 – 3.28 (m, 0.5×1H), 3.17 (d, J = 5.0 Hz, 1H), 3.10 – 3.03 (m, 3H), 3.03 – 2.91 (m, 2H), 2.90 – 2.83 (m, 0.5×1H), 2.80 (d, J = 4.6 Hz, 3H), 2.58 (t, J = 12.4 Hz, 1H), 2.02 (d, J = 13.5 Hz, 3H), 1.93 – 1.82 (m, 2H), 1.82 – 1.70 (m, 1H), 1.60 – 1.50 (m, 0.5×1H), 1.36 (s, 9H), 1.29 (s, 1H). ^{13}C{^{1}H} NMR (151 MHz, DMSO-d_6) δ 167.9, 165.3 and 165.3, 155.7, 140.6, 140.2, 139.0, 127.9 and 127.9, 127.2 and 127.1, 122.2, 115.4, 109.4 and 109.3, 77.7 and 77.6, 51.2, 48.6, 47.7 and 47.6, 46.2 and 46.1, 41.2 and 40.8, 34.7 and 34.1, 30.7 and 30.6, 28.2, 26.3, 25.7 and 24.8, 21.4 and 21.4. HRMS (ESI/QTOF) m/z: $[M + H]^+$ Calcd for $C_{23}H_{35}N_6O_4^+$: 459.2714; Found: 459.2712.

***tert*-Butyl 4-({2-[1-(2,2-difluoroethyl)-5-methyl-1*H*-pyrazol-4-yl]-8-(hydroxymethyl)imidazo[1,2-*a*]pyridin-3-yl}amino)piperidine-1-carboxylate (4{124,304,43}]** Yield 73 mg, 0.149 mmol, 55%. Colourless viscous oil. ^{1}H NMR (500 MHz, DMSO-d_6) δ 8.18 (d, J = 6.9 Hz, 1H), 8.03 (s, 1H), 7.17 (d, J = 7.1 Hz, 1H), 6.88 (t, J = 6.8 Hz, 1H), 6.53 – 6.25 (m, 1H), 5.29 (t, J = 5.7 Hz, 1H), 4.84 (d, J = 5.6 Hz, 2H), 4.72 (d, J = 5.6 Hz, 1H), 4.61 (s, 2H), 3.79 (s, 2H), 2.96 (s, 1H), 2.66 (s, 4H), 1.63 (d, J = 12.1 Hz, 2H), 1.37 (s, 9H), 1.29 (d, J = 11.5 Hz, 2H). ^{13}C{^{1}H} NMR (151 MHz, DMSO-d_6) δ 153.8, 138.8, 138.1, 137.7, 130.2, 124.4, 121.2, 118.8, 114.4 (t, J = 240.8

Hz), 113.3, 111.1, 78.5, 58.2, 54.1, 50.1 (t, *J* = 34.7 Hz), 42.2, 32.4, 28.1, 10.3. $^{19}F\{^1H\}$ NMR (376 MHz, DMSO-d_6) δ –122.5. HRMS (ESI/QTOF) m/z: $[M + H]^+$ Calcd for $C_{24}H_{33}F_2N_6O_3^+$: 491.2577; Found: 491.2574.

***N*-[(3-Bromophenyl)methyl]-2-(4-methoxy-1-methyl-1*H*-pyrazol-5-yl)-6-methylimidazo[1,2-*a*]pyrazin-3-amine (4{41,19,11}).** Yield 70 mg, 0.165 mmol, 61%. Yellow viscous oil. ^1H NMR (500 MHz, DMSO-d_6) δ 8.81 (s, 1H), 8.14 (s, 1H), 7.37 (s, 1H), 7.32 (dd, *J* = 7.9, 2.0 Hz, 1H), 7.16 (s, 1H), 7.11 (t, *J* = 7.8 Hz, 1H), 6.97 (d, *J* = 7.6 Hz, 1H), 5.81 (t, *J* = 6.7 Hz, 1H), 4.13 (d, *J* = 6.8 Hz, 2H), 3.73 (s, 3H), 3.52 (s, 3H), 2.44 (s, 3H). $^{13}C\{^1H\}$ NMR (126 MHz, DMSO-d_6) δ 144.3, 142.2, 141.7, 136.6, 134.8, 130.1, 130.00, 129.7, 129.5, 126.3, 123.7, 122.4, 121.5, 121.3, 112.2, 58.5, 48.0, 37.7, 20.5. HRMS (ESI/QTOF) m/z: $[M + H]^+$ Calcd for $C_{19}H_{20}BrN_6O^+$: 427.0876; Found: 427.0872.

2-[4-(5-Methyl-3-{[(2-methyl-2*H*-1,2,3,4-tetrazol-5-yl)methyl]amino}-7-(trifluoromethyl)imidazo[1,2-*a*]pyridin-2-yl)-1*H*-pyrazol-1-yl]ethan-1-ol (4{40,18,4}). Yield 70 mg, 0.165 mmol, 61%. Yellow amorphous powder. ^1H NMR (500 MHz, DMSO-d_6) δ 8.20 (s, 1H), 7.97 (s, 1H), 7.71 (s, 1H), 6.84 (s, 1H), 5.25 (t, *J* = 5.8 Hz, 1H), 4.94 (t, *J* = 5.3 Hz, 1H), 4.35 (d, *J* = 5.8 Hz, 2H), 4.28 (s, 3H), 4.19 (s, 1H), 3.78 (q, *J* = 5.6 Hz, 2H), 2.95 (s, 3H). $^{13}C\{^1H\}$ NMR (151 MHz, DMSO-d_6) δ 163.8, 140.4, 138.1, 137.0, 135.9, 128.7, 126.8, 124.7, 123.6 (q, *J* = 32.9 Hz), 122.9, 121.1, 115.0, 112.2, 107.5, 60.0, 54.1, 43.8, 18.8. $^{19}F\{^1H\}$ NMR (376 MHz, DMSO-d_6) δ –62.3. HRMS (ESI/QTOF) m/z: $[M + H]^+$ Calcd for $C_{17}H_{19}F_3N_9O^+$: 422.1659; Found: 422.1656.

1-(2-{4-Methoxy-3-[(pyridin-3-yl)methoxy]phenyl}-3-{[2-methylsulfanyl)ethyl]amino}imidazo[1,2-*a*]pyridin-5-yl)ethan-1-ol (4{118,625,31})

Yield 71 mg, 0.152 mmol, 56%. Yellowish amorphous solid. ^{1}H NMR (500 MHz, DMSO-d_6) δ 8.71 (s, 1H), 8.56 (d, *J* = 3.4 Hz, 1H), 7.91 (d, *J* = 7.9 Hz, 1H), 7.84 (s, 1H), 7.72 (d, *J* = 8.4 Hz, 1H), 7.45 (dd, *J* = 7.8, 4.8 Hz, 1H), 7.38 (d, *J* = 8.8 Hz, 1H), 7.19 – 7.12 (m, 1H), 7.07 (d, *J* = 8.5 Hz, 1H), 6.96 (d, *J* = 7.0 Hz, 1H), 5.84 – 5.73 (m, 2H), 5.21 (s, 2H), 5.05 (t, *J* = 6.0 Hz, 1H), 3.82 (s, 3H), 3.08 – 2.93 (m, 2H), 2.59 (t, *J* = 6.9 Hz, 2H), 2.00 (s, 3H), 1.54 (d, *J* = 6.1 Hz, 3H). ^{13}C{^{1}H} NMR (151 MHz, DMSO-d_6) δ 149.1, 149.1, 148.6, 147.4, 144.3, 141.7, 136.1, 135.7, 132.8, 127.3, 127.2, 123.6, 123.5, 120.3, 115.8, 112.7, 112.1, 108.4, 67.9, 62.6, 55.6, 48.4, 33.3, 22.8, 14.8. HRMS (ESI/QTOF) m/z: $[M + H]^+$ Calcd for $C_{25}H_{29}N_4O_3S^+$: 465.1955; Found: 465.1937.

***tert*-Butyl *N*-{3-[(2-{1,3-dimethyl-1*H*-pyrazolo[3,4-*b*]pyridin-5-yl}-7-(methylcarbamoyl)imidazo[1,2-*a*]pyridin-3-yl)amino]propyl}carbamate (4{69,596,61}).** Yield 73 mg, 0.148 mmol, 55%. Colourless amorphous solid. ^{1}H NMR (500 MHz, DMSO-d_6) δ 9.30 (d, *J* = 2.0 Hz, 1H), 8.78 (d, *J* = 2.0 Hz, 1H), 8.61 (q, *J* = 4.5 Hz, 1H), 8.39 (d, *J* = 7.2 Hz, 1H), 8.02 (s, 1H), 7.34 (dd, *J* = 7.2, 1.7 Hz, 1H), 6.81 (t, *J* = 5.7 Hz, 1H), 5.06 (t, *J* = 6.2 Hz, 1H), 4.01 (s, 3H), 3.04 – 2.91 (m, 4H), 2.83 (d, *J* = 4.5 Hz, 3H), 2.56 (s, 3H), 1.67 – 1.58 (m, 2H), 1.31 (s, 8H). ^{13}C{^{1}H} NMR (151 MHz, DMSO-d_6) δ 165.1, 155.6, 149.8, 147.8, 140.2, 139.7, 134.4, 129.2, 127.8, 126.8, 122.8, 122.7, 115.5, 114.3, 109.8, 77.4, 45.2, 37.7, 33.2, 30.7, 28.2, 26.3, 12.0. HRMS (ESI/QTOF) m/z: $[M + H]^+$ Calcd for $C_{25}H_{33}N_8O_3^+$: 493.2670; Found: 493.2672.

2-[4-(Difluoromethoxy)-3-ethoxyphenyl]-3-[(oxan-4-yl)amino]imidazo[1,2-*a*]pyridine-7-carboxamide (4{92,616,7}). Yield 69 mg, 0.155 mmol, 57%. Colourless amorphous powder. ^{1}H NMR (500 MHz, DMSO-d_6) δ 8.42 (d, *J* = 7.2 Hz, 1H), 8.11 (s, 1H), 8.08 (s, 1H), 7.99 (s, 1H), 7.85 (d, *J* = 8.4 Hz, 1H), 7.54 (s, 1H), 7.34 (d, *J* = 7.3 Hz, 1H), 7.24 (d, *J* = 8.4 Hz, 1H), 7.12 (t, *J* = 74.3 Hz, 1H), 5.11 (d, *J* = 6.1 Hz, 1H),

4.18 (q, *J* = 6.9 Hz, 2H), 3.79 (d, *J* = 12.0 Hz, 2H), 3.19 (t, *J* = 11.5 Hz, 2H), 3.13 (s, 1H), 1.63 (d, *J* = 9.5 Hz, 2H), 1.52 (d, *J* = 7.8 Hz, 2H), 1.40 (d, *J* = 6.9 Hz, 3H). $^{13}C\{^{1}H\}$ NMR (151 MHz, DMSO-d_6) δ 166.4, 149.6, 139.6, 138.9, 136.0, 132.7, 129.2, 126.3, 123.0, 121.1, 119.0, 116.8 (t, *J* = 257.4 Hz), 116.2, 112.0, 110.1, 65.7, 64.1, 53.6, 33.7, 14.6. $^{19}F\{^{1}H\}$ NMR (376 MHz, DMSO-d_6) δ –81.5. HRMS (ESI/QTOF) m/z: $[M + H]^+$ Calcd for $C_{22}H_{25}F_2N_4O_4^+$: 447.1838; Found: 447.1836.

***tert*-Butyl 4-{[6-(2-hydroxyethoxy)-2-(3-methylpyridin-2-yl)imidazo[1,2-*a*]pyridin-3-yl]amino}piperidine-1-carboxylate (4{107,621,43}).** Yield 718 mg, 0.154 mmol, 57%. Yellowish viscous oil. ^{1}H NMR (600 MHz, DMSO-d_6) δ 8.45 (d, *J* = 4.8 Hz, 1H), 7.70 – 7.64 (m, 2H), 7.46 (d, *J* = 9.7 Hz, 1H), 7.20 (dd, *J* = 7.6, 4.7 Hz, 1H), 6.98 (dd, *J* = 9.7, 2.3 Hz, 1H), 6.00 (d, *J* = 9.5 Hz, 1H), 4.96 (t, *J* = 5.4 Hz, 1H), 4.07 (t, *J* = 4.8 Hz, 2H), 3.77 (q, *J* = 5.0 Hz, 2H), 3.72 (d, *J* = 13.0 Hz, 2H), 3.21 – 3.12 (m, 1H), 2.73 (br s, 2H), 2.69 (s, 3H), 1.69 (d, *J* = 12.4 Hz, 2H), 1.35 (s, 9H), 1.20 – 1.11 (m, 2H). $^{13}C\{^{1}H\}$ NMR (151 MHz, DMSO-d_6) δ 153.7, 152.3, 147.7, 145.6, 139.2, 136.4, 133.1, 131.5, 130.7, 121.3, 118.5, 117.8, 105.1, 78.5, 70.9, 59.6, 52.5, 41.7, 32.3, 28.0, 20.6. HRMS (ESI/QTOF) m/z: $[M + H]^+$ Calcd for $C_{25}H_{34}N_5O_4^+$: 468.2605; Found: 468.2603.

***N*-(Cyclopropylmethyl)-6-iodo-2-[1-(pyridin-2-yl)-1*H*-pyrazol-4-yl]imidazo[1,2-*a*]pyrazin-3-amine (4{71,615,21})**. Yield 710 mg, 0.155 mmol, 58%. Yellow amorphous solid. ^{1}H NMR (600 MHz, DMSO-d_6) δ 9.12 (s, 1H), 8.73 (s, 1H), 8.69 (s, 1H), 8.52 (d, *J* = 4.8 Hz, 1H), 8.35 (s, 1H), 8.05 – 7.95 (m, 2H), 7.39 (dd, *J* = 7.2, 4.9 Hz, 1H), 5.35 (t, *J* = 6.2 Hz, 1H), 2.89 (t, *J* = 6.5 Hz, 2H), 0.97 – 0.89 (m, 1H), 0.32 (q, *J* = 7.5 Hz, 2H), 0.03 (q, *J* = 4.9 Hz, 2H). $^{13}C\{^{1}H\}$ NMR (151 MHz, DMSO-d_6) δ 150.6, 148.4, 141.9, 140.7, 139.6, 135.4, 131.8, 127.7, 124.3, 122.2, 121.4, 117.4,

112.0, 94.0, 51.9, 11.9, 3.3. HRMS (ESI/QTOF) m/z: $[M + H]^+$ Calcd for $C_{18}H_{17}IN_7^+$: 458.0585; Found: 458.0595.

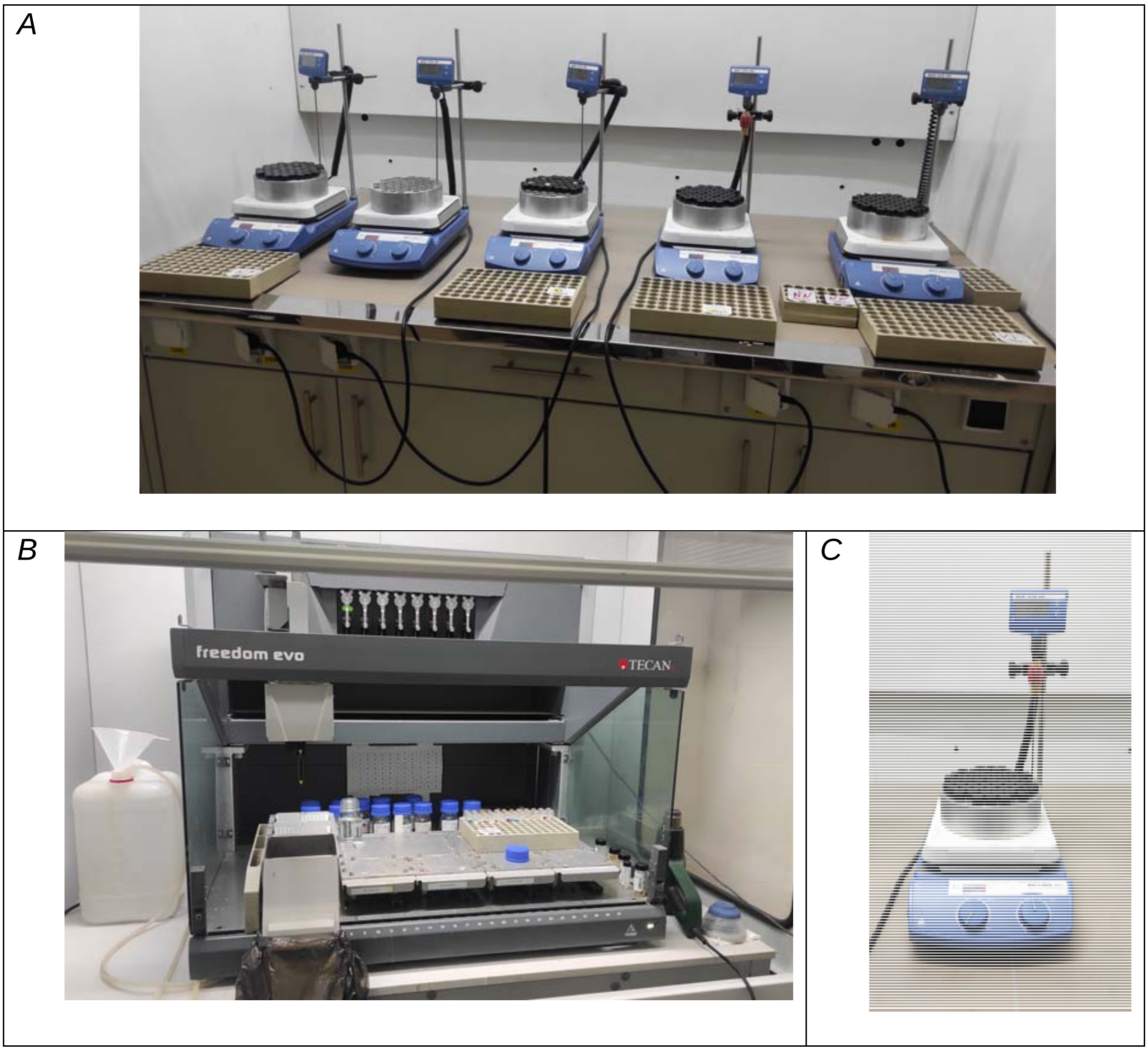

Figure S4. Parallel reaction setup: (*A*) before the reaction; (*B*) reagent loading; (*C*) reaction in progress
