# Supplementary material for: Generation of multimillion chemical space based on the parallel Groebke–Blackburn–Bienaymé reaction
Source: Beilstein J Org Chem. 2024 Jul 16;20:1604–13. doi: 10.3762/bjoc.20.143 (PMC11285076; doi:10.3762/bjoc.20.143)
Supplement: File 4 — Copies of NMR spectra. [file Beilstein_J_Org_Chem-20-1604-s004.pdf]

BEILSTEIN JOURNAL OF ORGANIC CHEMISTRY

Supporting Information

for

Generation of multimillion chemical space based on the parallel Groebke–Blackburn–Bienaymé reaction

Evgen V. Govor, Vasyl Naumchyk, Ihor Nestorak, Dmytro S. Radchenko, Dmytro Dudenko, Yurii S. Moroz, Olexiy D. Kachkovsky and Oleksandr O. Grygorenko

Beilstein J. Org. Chem. **2024,** *20,* 1604–1613. doi:10.3762/bjoc.20.143

Copies of NMR spectra

License and Terms: This is a supporting information file under the terms of the Creative Commons Attribution License (https://creativecommons.org/licenses/by/4.0). Please note that the reuse, redistribution and reproduction in particular requires that the author(s) and source are credited and that individual graphics may be subject to special legal provisions.
The license is subject to the *Beilstein Journal of Organic Chemistry* terms and conditions: (https://www.beilstein-journals.org/bjoc/terms)

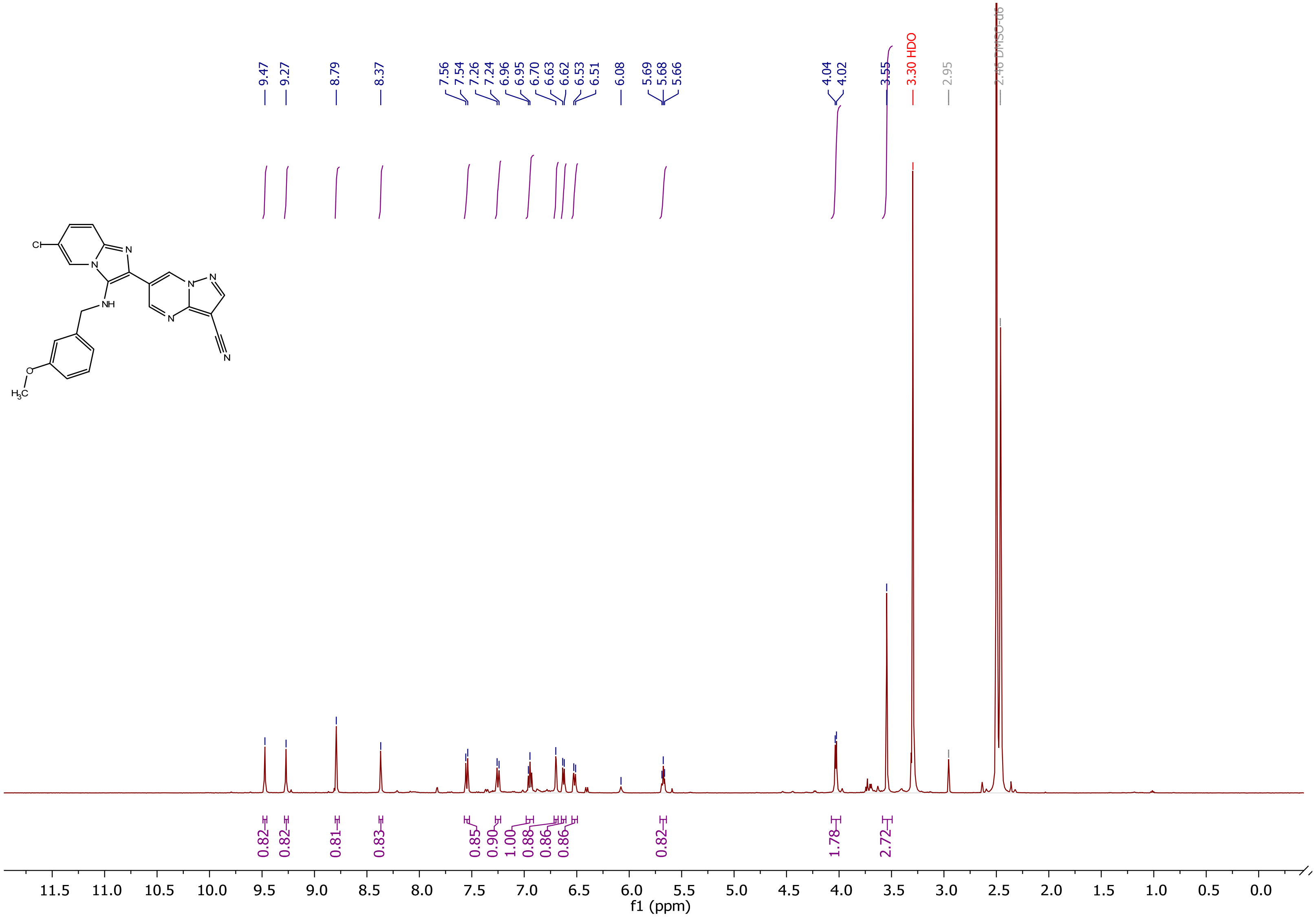

Spectrum 1. 6-(6-Chloro-3-{[(3-methoxyphenyl)methyl]amino}imidazo[1,2-*a*]pyridin-2-yl)pyrazolo[1,5-*a*]pyrimidine-3-carbonitrile **4**{347,242,24}, ^{1}H NMR (500 MHz, DMSO-d_6)

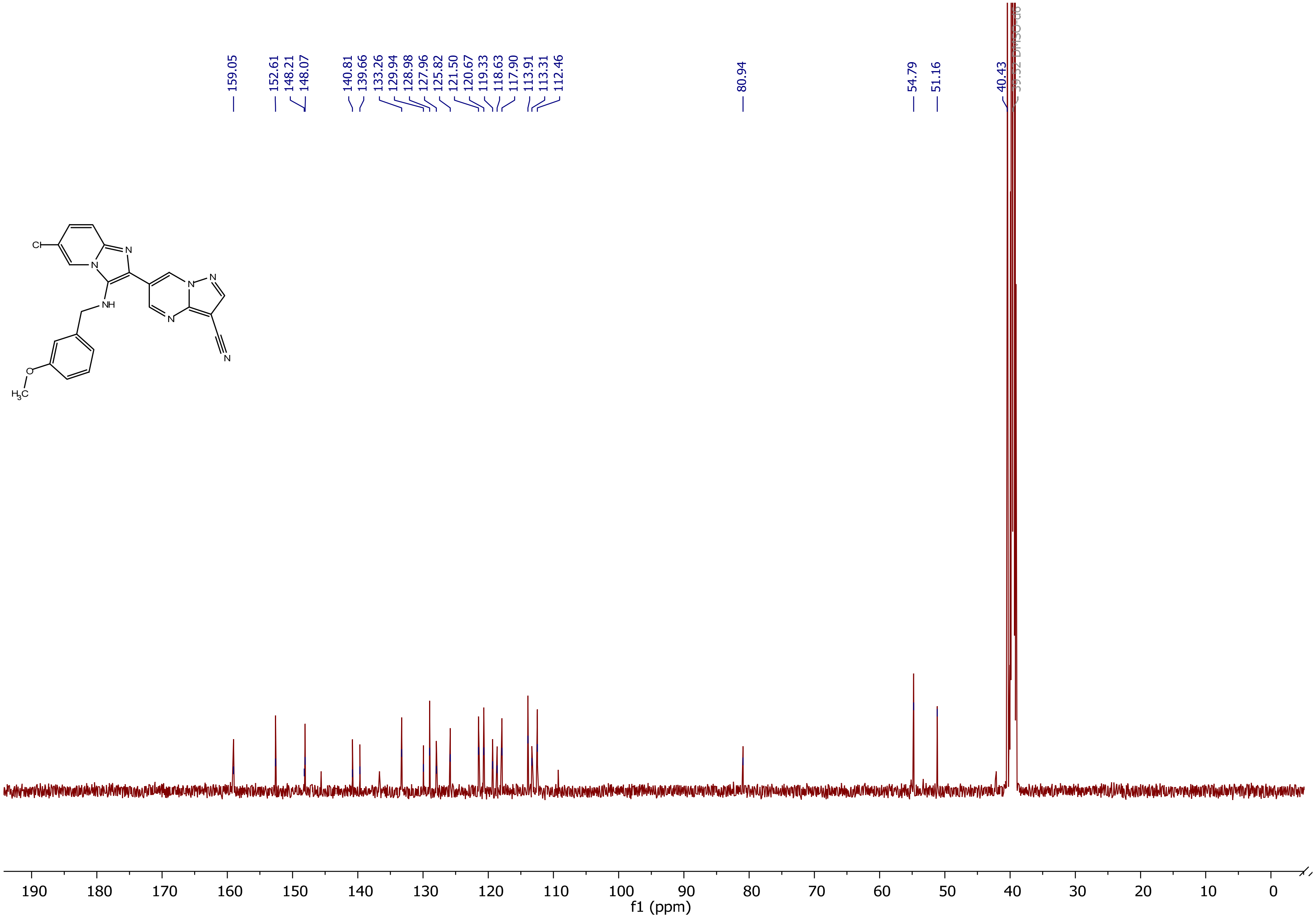

Spectrum 2. 6-(6-Chloro-3-{[(3-methoxyphenyl)methyl]amino}imidazo[1,2-*a*]pyridin-2-yl)pyrazolo[1,5-*a*]pyrimidine-3-carbonitrile **4**{347,242,24}, $^{13}C\{^1H\}$ NMR (151 MHz, DMSO-d_6)

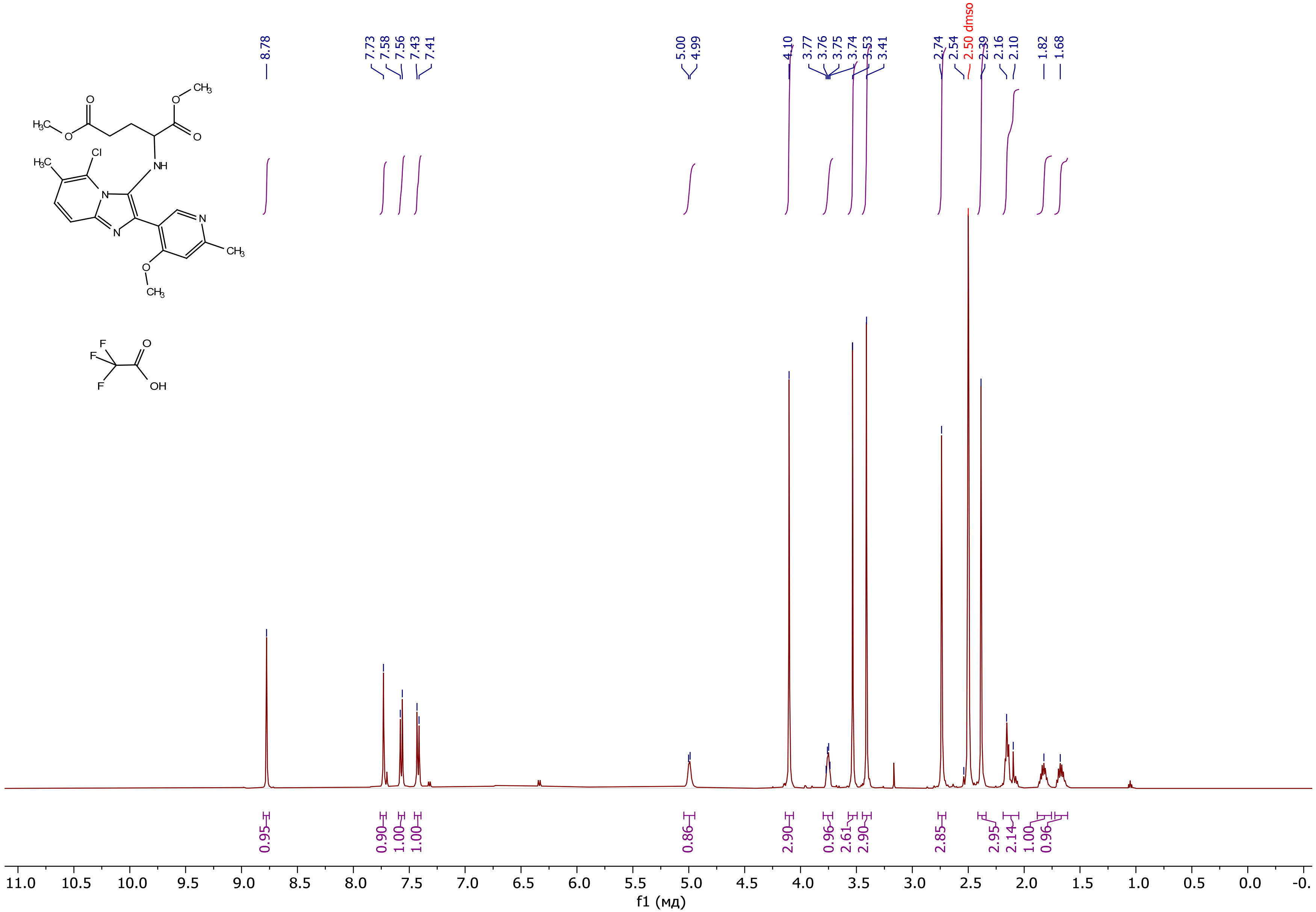

Spectrum 3. 1,5-Dimethyl 2-{[5-chloro-2-(4-methoxy-6-methylpyridin-3-yl)-6-methylimidazo[1,2-*a*]pyridin-3-yl]amino}pentanedioate trifluoroacetate **4**{76,584,23}, ^{1}H NMR (500 MHz, DMSO-d_6)

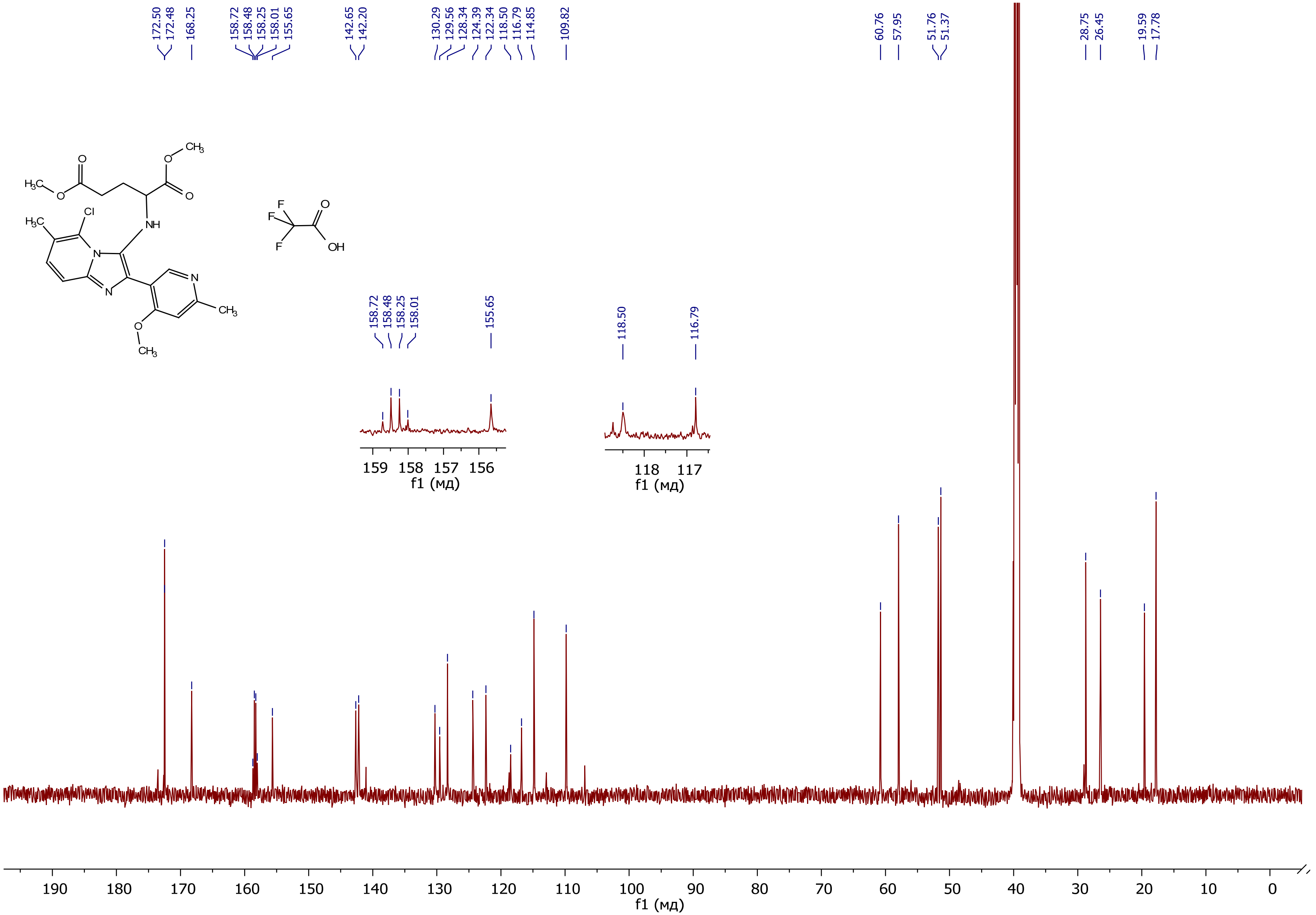

Spectrum 4. 1,5-Dimethyl 2-{[5-chloro-2-(4-methoxy-6-methylpyridin-3-yl)-6-methylimidazo[1,2-*a*]pyridin-3-yl]amino}pentanedioate trifluoroacetate **4**{76,584,23}, $^{13}C\{^{1}H\}$ NMR (151 MHz, DMSO-d_6)

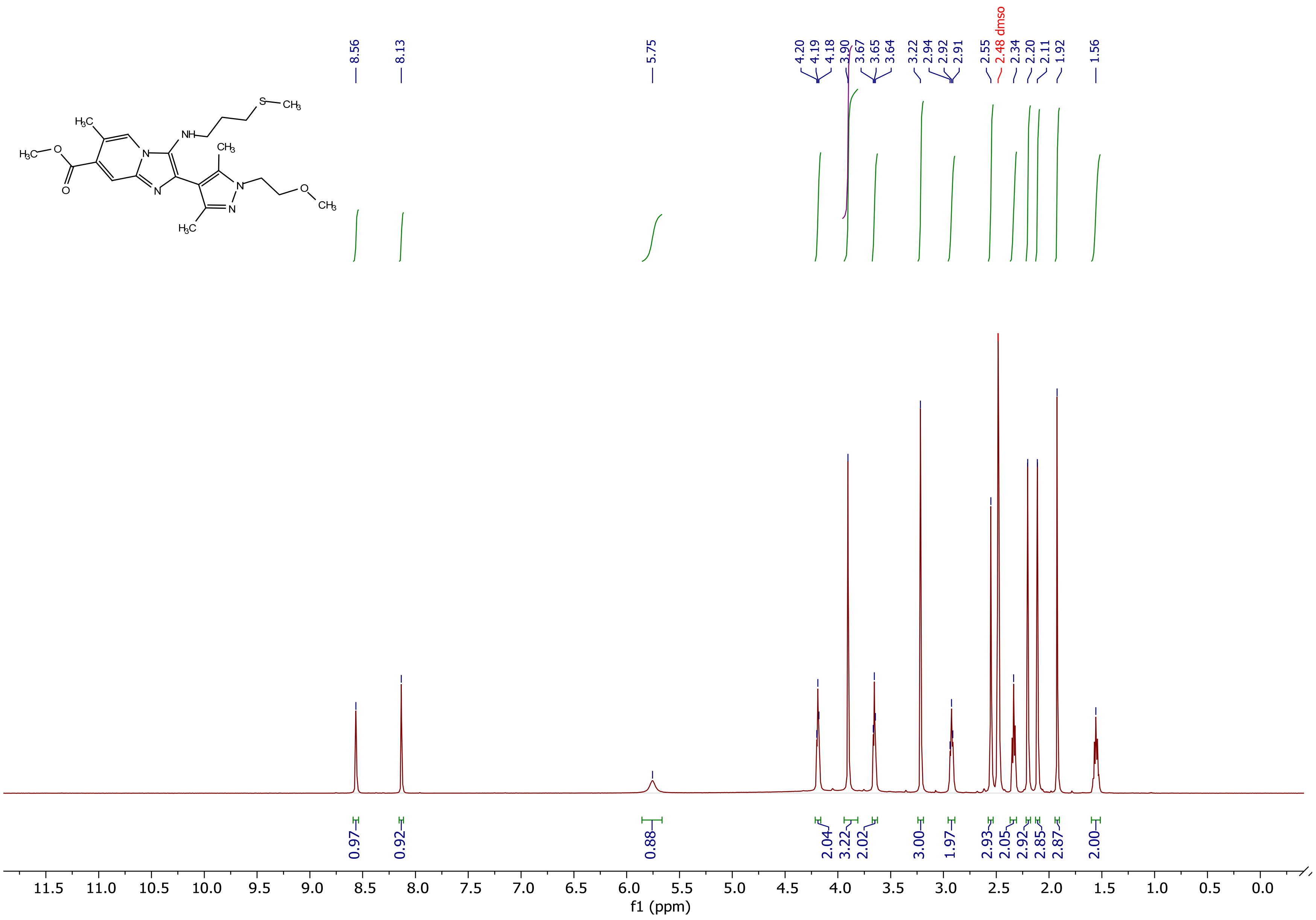

Spectrum 5. Methyl 2-[1-(2-methoxyethyl)-3,5-dimethyl-1*H*-pyrazol-4-yl]-6-methyl-3-{[3-(methylsulfanyl)propyl]amino}imidazo[1,2-*a*]pyridine-7-carboxylate trifluoroacetate **4**{180,545,35}, ^{1}H NMR (500 MHz, DMSO-d_6)

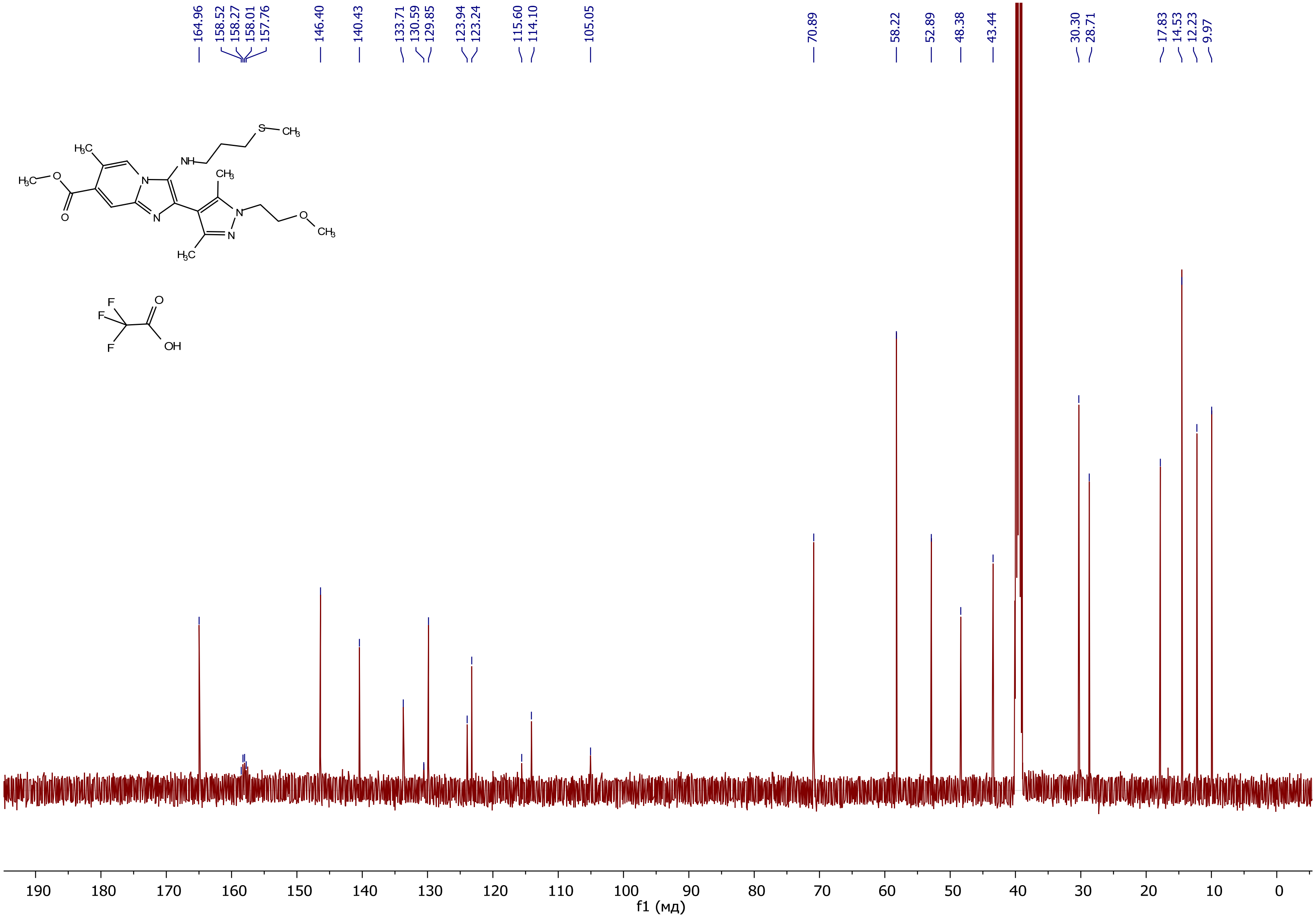

Spectrum 6. Methyl 2-[1-(2-methoxyethyl)-3,5-dimethyl-1*H*-pyrazol-4-yl]-6-methyl-3-{[3-(methylsulfanyl)propyl]amino}imidazo[1,2-*a*]pyridine-7-carboxylate trifluoroacetate **4**{180,545,35}, $^{13}C\{^{1}H\}$ NMR (126 MHz, DMSO-d_6)

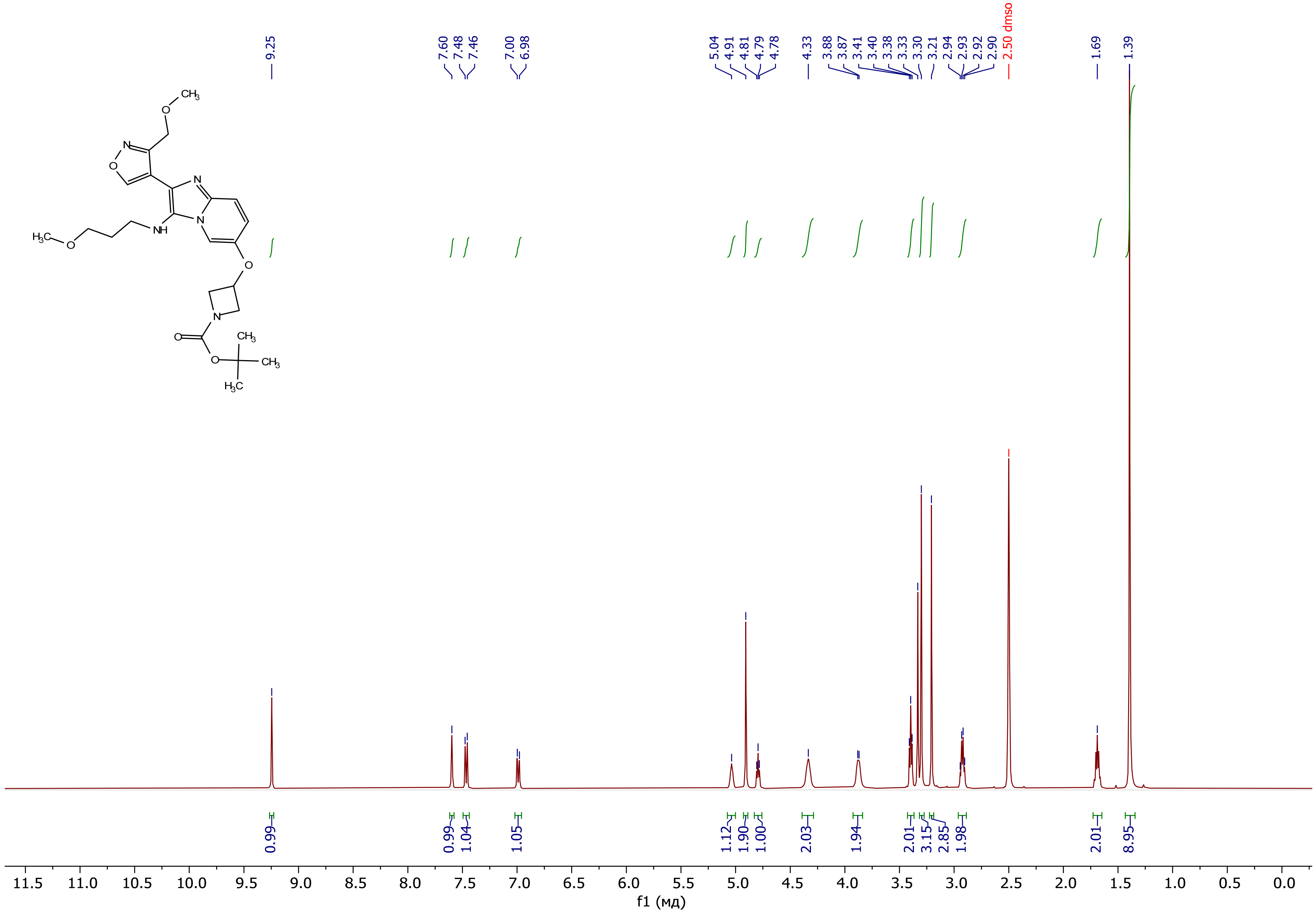

Spectrum 7. *tert*-Butyl 3-({2-[3-(methoxymethyl)-1,2-oxazol-4-yl]-3-[(3-methoxypropyl)amino]imidazo[1,2-*a*]pyridin-6-yl}oxy)azetidine-1-carboxylate **4**{29,8,5}, ^{1}H NMR (500 MHz, DMSO-d_6)

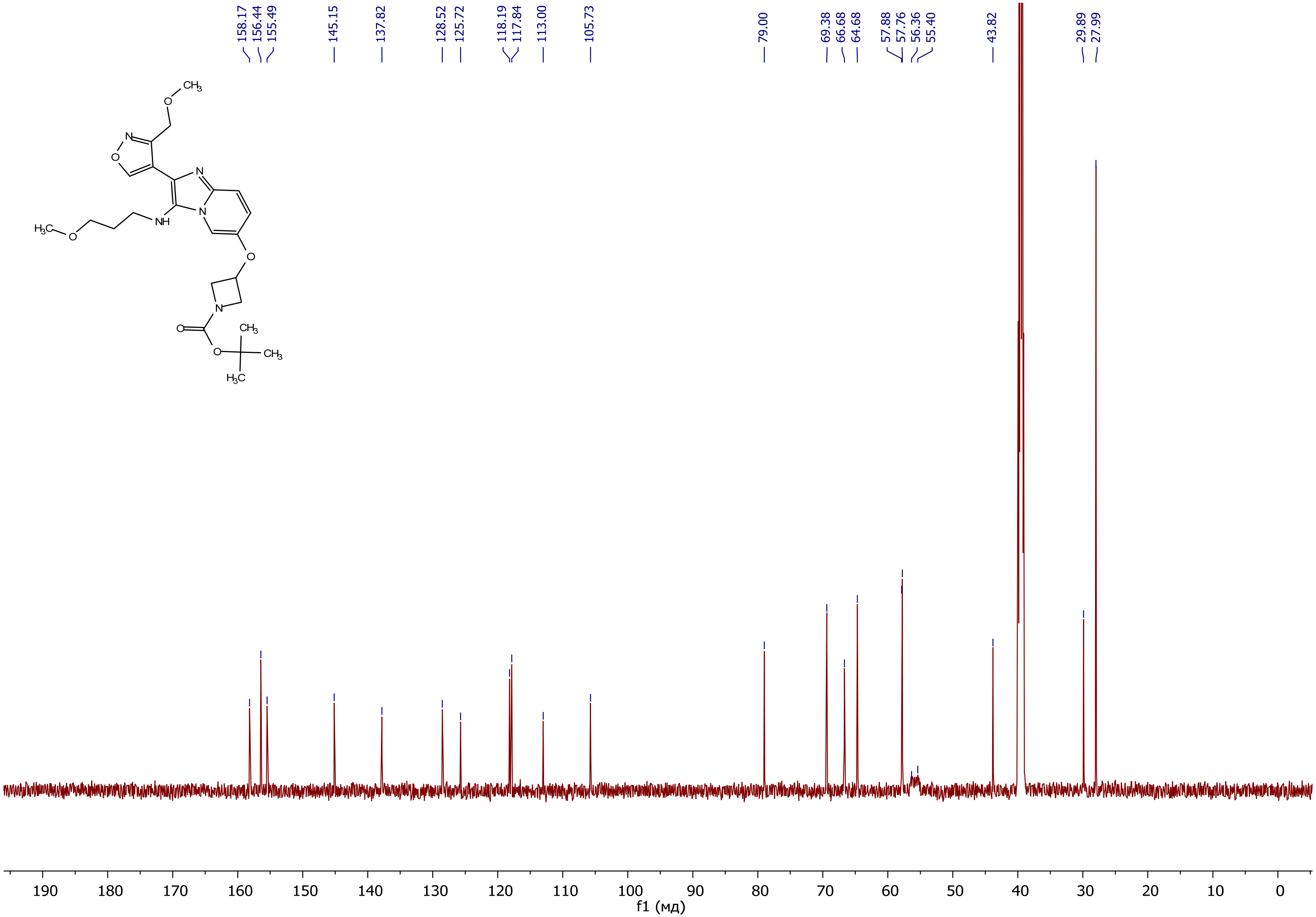

Spectrum 8. *tert*-Butyl 3-({2-[3-(methoxymethyl)-1,2-oxazol-4-yl]-3-[(3-methoxypropyl)amino]imidazo[1,2-*a*]pyridin-6-yl}oxy)azetidine-1-carboxylate **4**{29,8,5}, $^{13}C\{^{1}H\}$ NMR (151 MHz, DMSO-d_6)

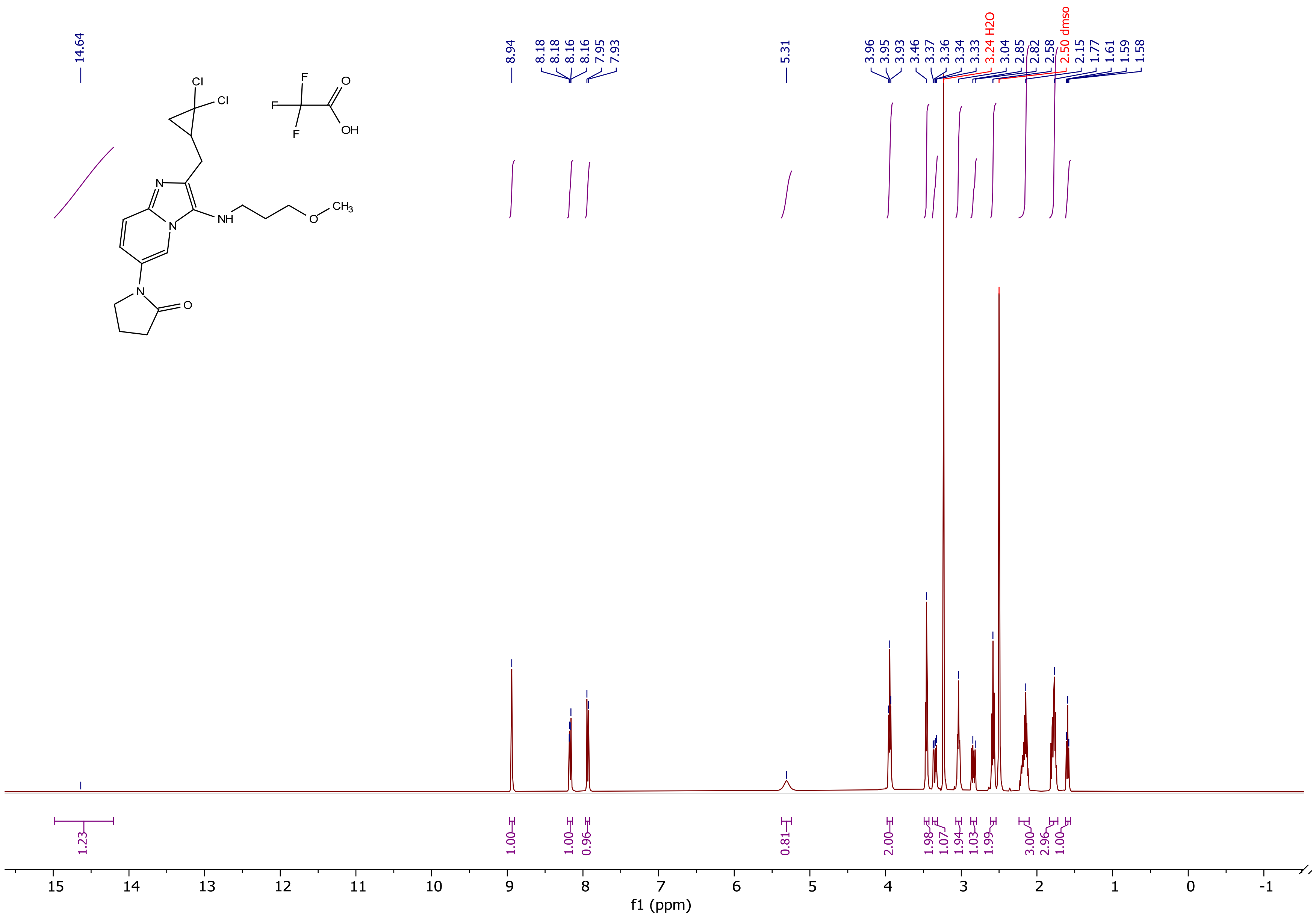

Spectrum 9. 1-{2-[(2,2-Dichlorocyclopropyl)methyl]-3-[(3-methoxypropyl)amino]imidazo[1,2-*a*]pyridin-6-yl}pyrrolidin-2-one trifluoroacetate **4**{405,585,5}, ^{1}H NMR (500 MHz, DMSO-d_6)

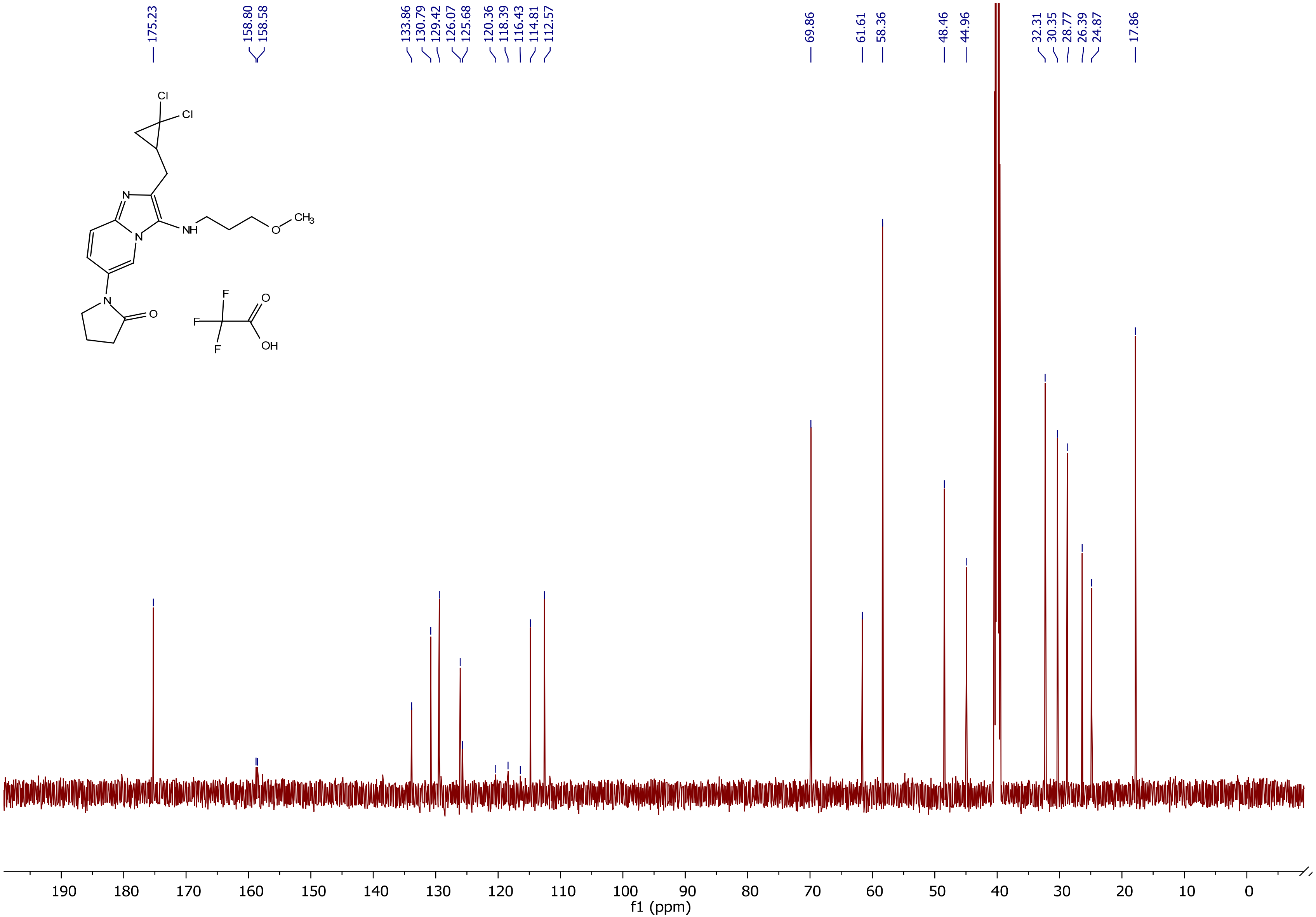

Spectrum 10. 1-{2-[(2,2-Dichlorocyclopropyl)methyl]-3-[(3-methoxypropyl)amino]imidazo[1,2-*a*]pyridin-6-yl}pyrrolidin-2-one trifluoroacetate **4**{405,585,5}, $^{13}C\{^{1}H\}$ NMR (151 MHz, DMSO-d_6)

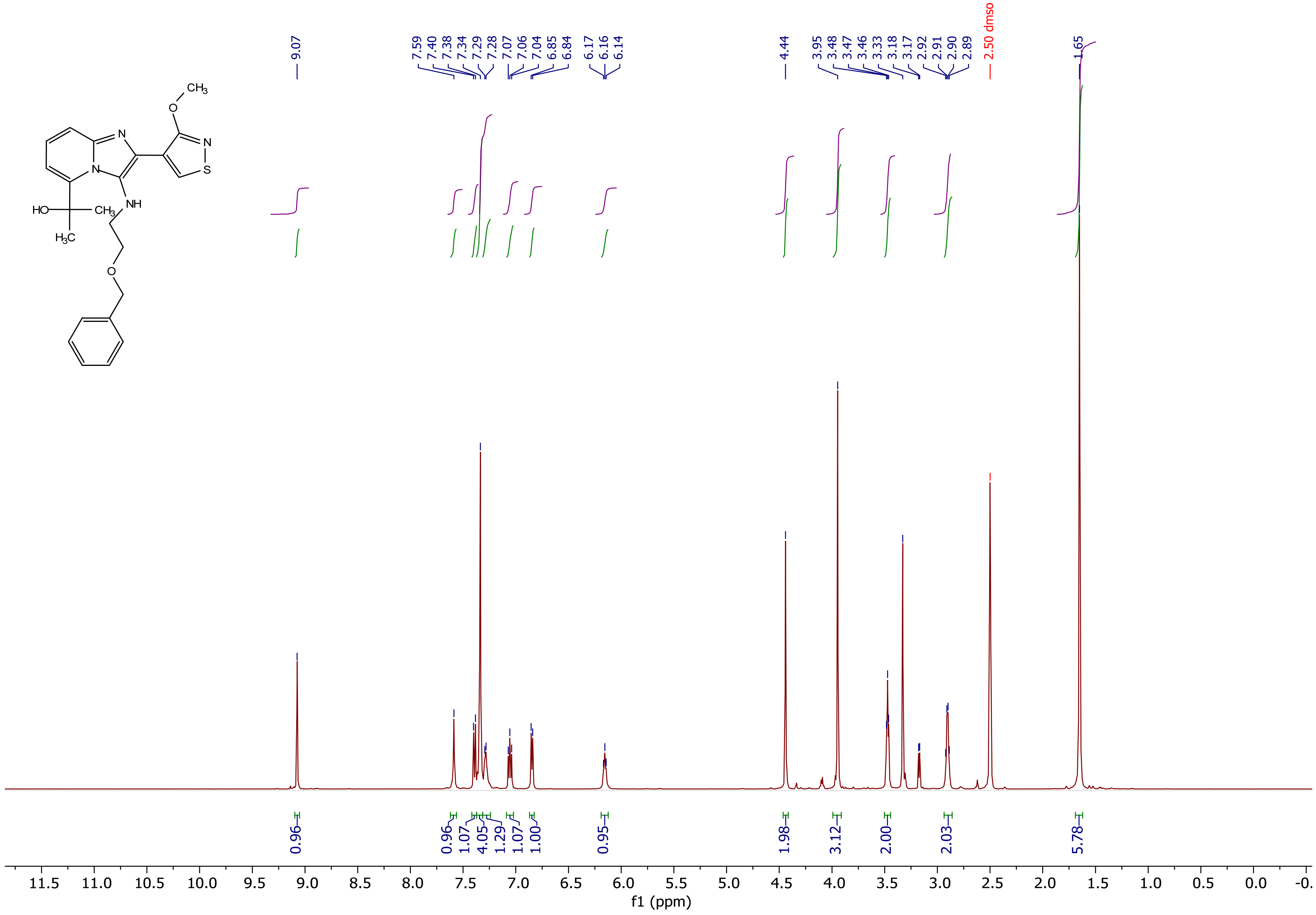

Spectrum 11. 2-(3-{[2-(Benzyloxy)ethyl]amino}-2-(3-methoxy-1,2-thiazol-4-yl)imidazo[1,2-*a*]pyridin-5-yl)propan-2-ol **4**{153,336,53}, ^{1}H NMR (500 MHz, DMSO-d_6)

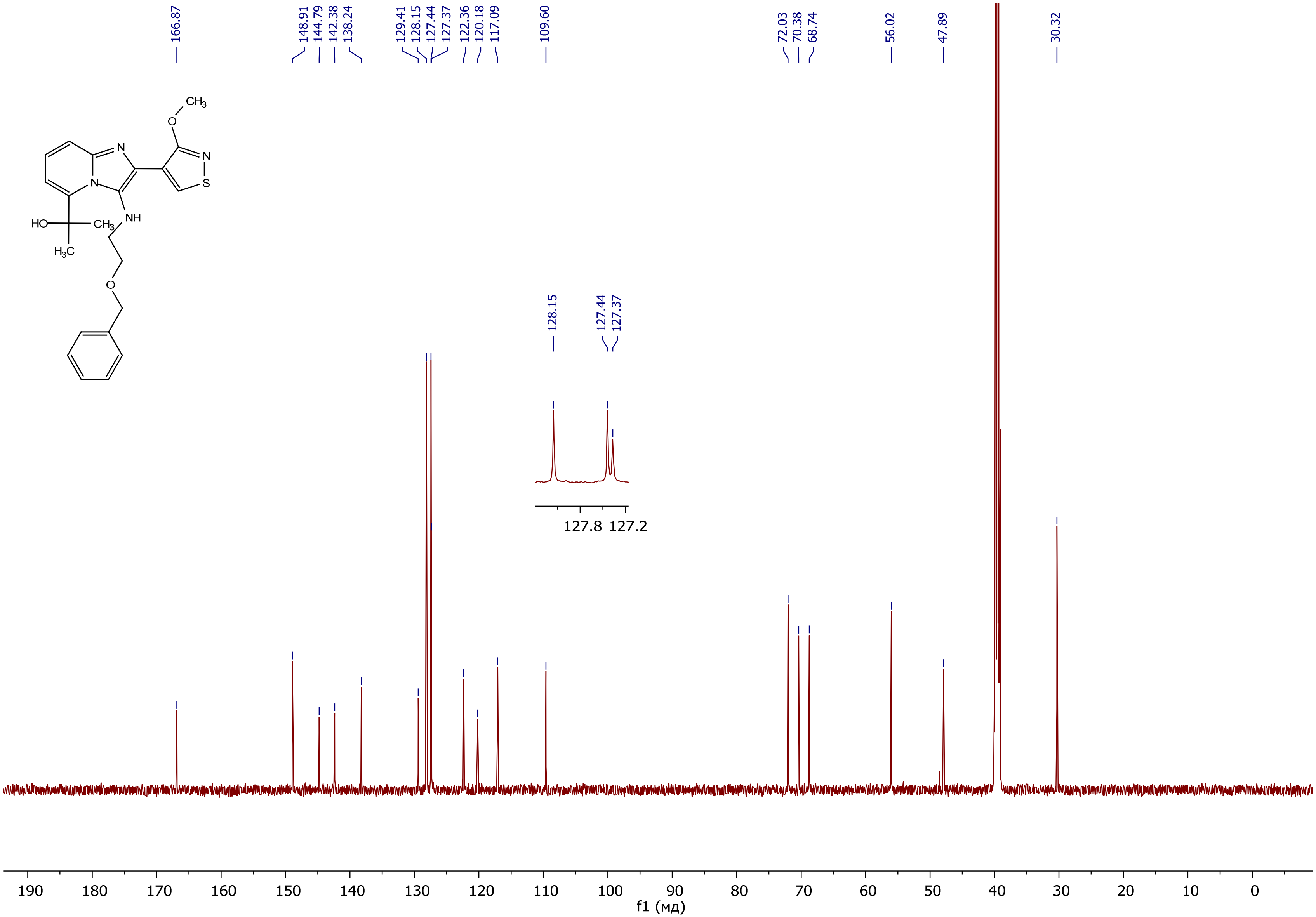

Spectrum 12. 2-(3-{[2-(Benzyloxy)ethyl]amino}-2-(3-methoxy-1,2-thiazol-4-yl)imidazo[1,2-*a*]pyridin-5-yl)propan-2-ol **4**{153,336,53}, $^{13}C\{^1H\}$ NMR (151 MHz, DMSO-d_6)

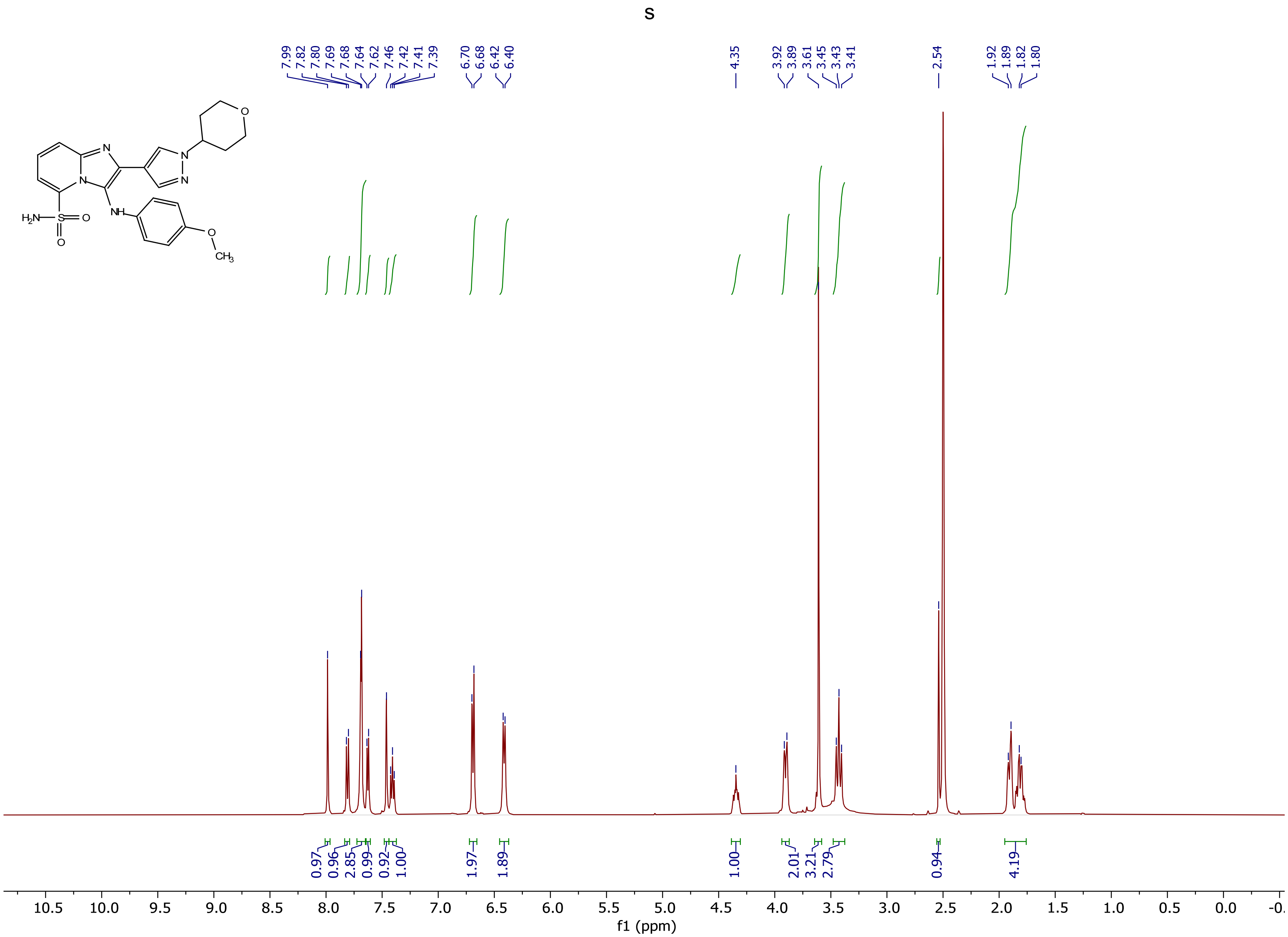

Spectrum 13. 3-((4-Methoxyphenyl)amino)-2-(1-(tetrahydro-2*H*-pyran-4-yl)-1*H*-pyrazol-4-yl)imidazo[1,2-*a*]pyridine-5-sulfonamide **4**{109,335,41}

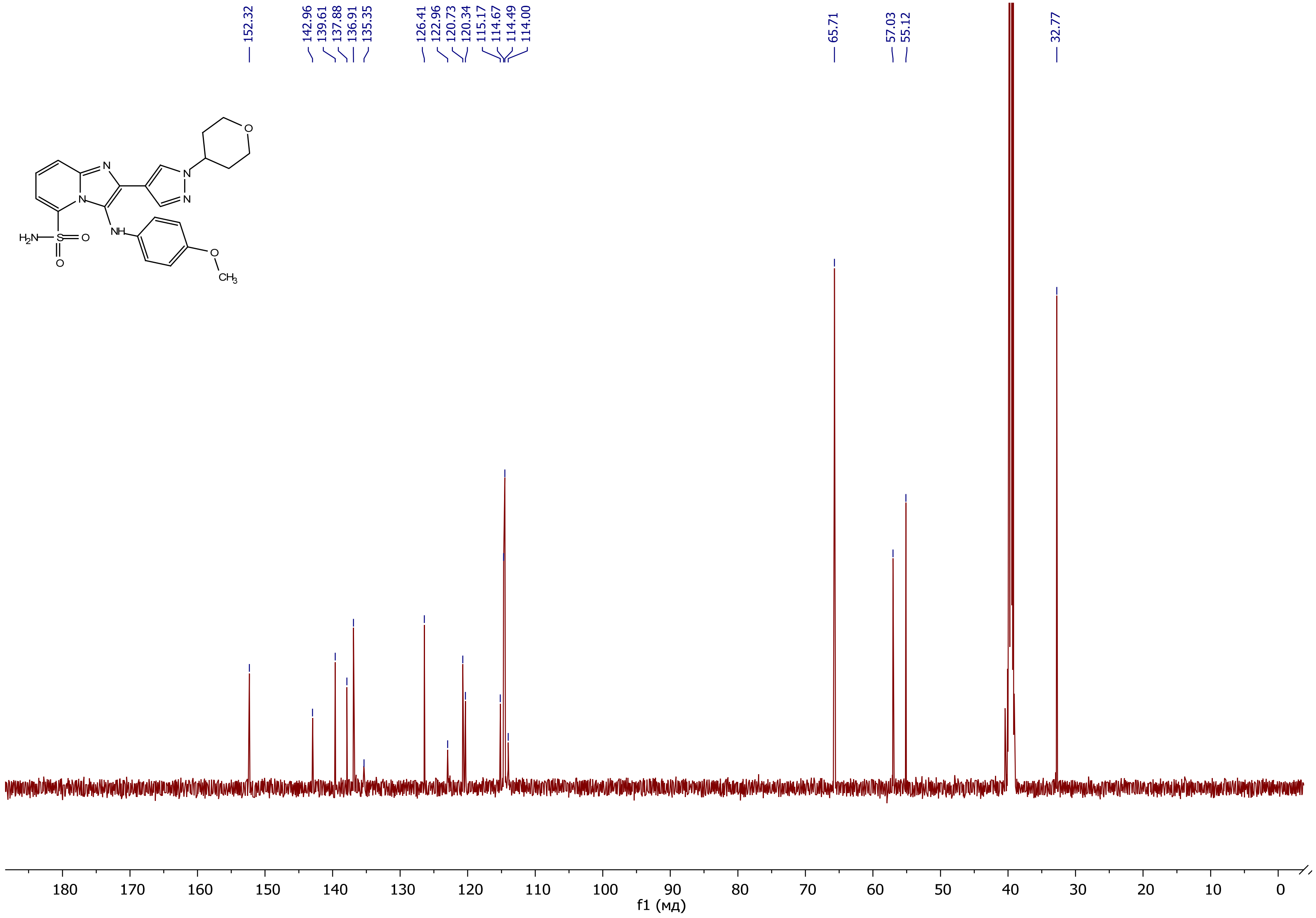

Spectrum 14. 3-[(4-Methoxyphenyl)amino]-2-[1-(oxan-4-yl)-1*H*-pyrazol-4-yl]imidazo[1,2-*a*]pyridine-5-sulfonamide **4**{109,335,41}, $^{13}C\{^{1}H\}$ NMR (151 MHz, DMSO-d_6)

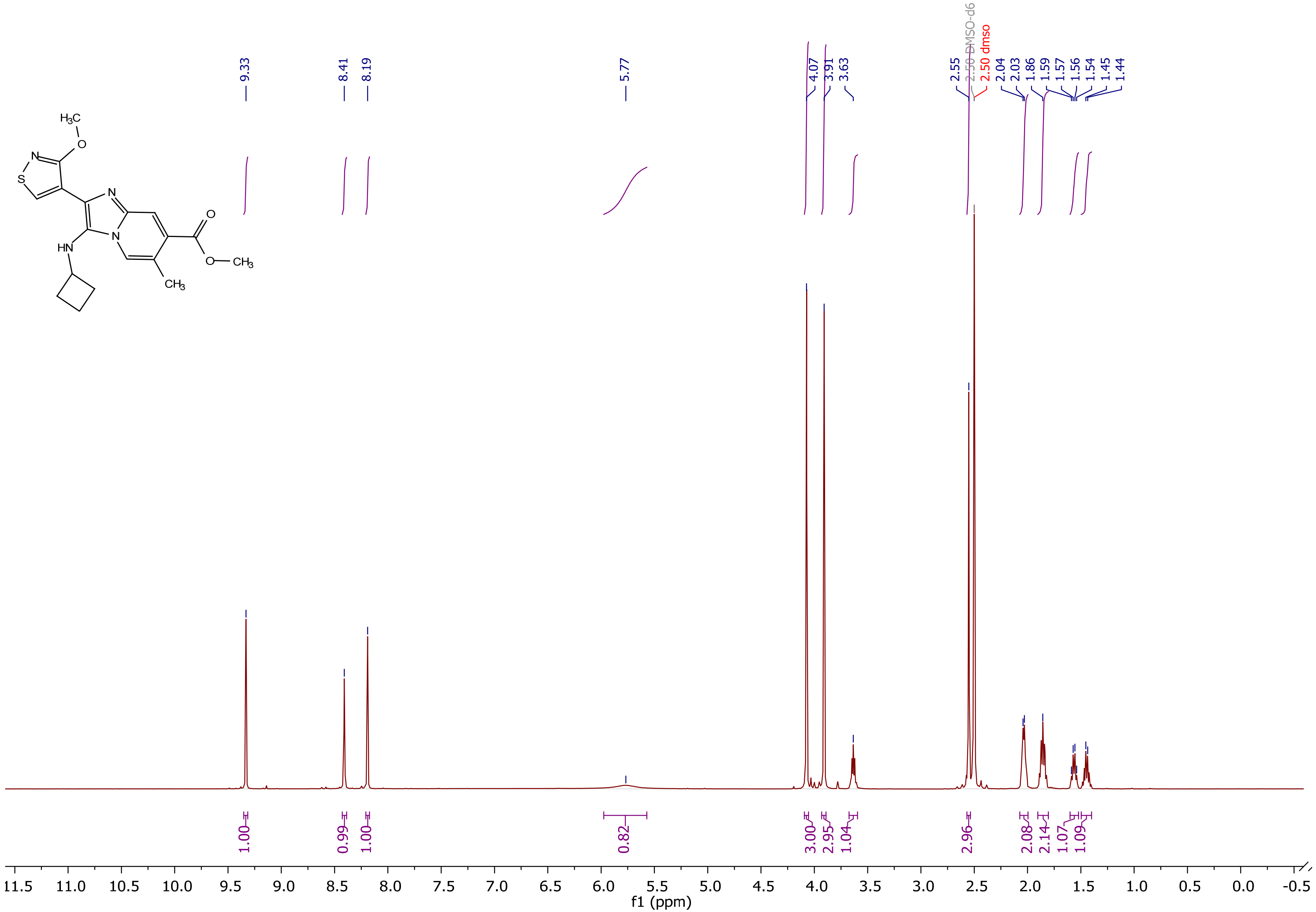

Spectrum 15. Methyl 3-(cyclobutylamino)-2-(3-methoxy-1,2-thiazol-4-yl)-6-methylimidazo[1,2-*a*]pyridine-7-carboxylate trifluoroacetate **4**{180,336,32}, ^{1}H NMR (600 MHz, DMSO-d_6)

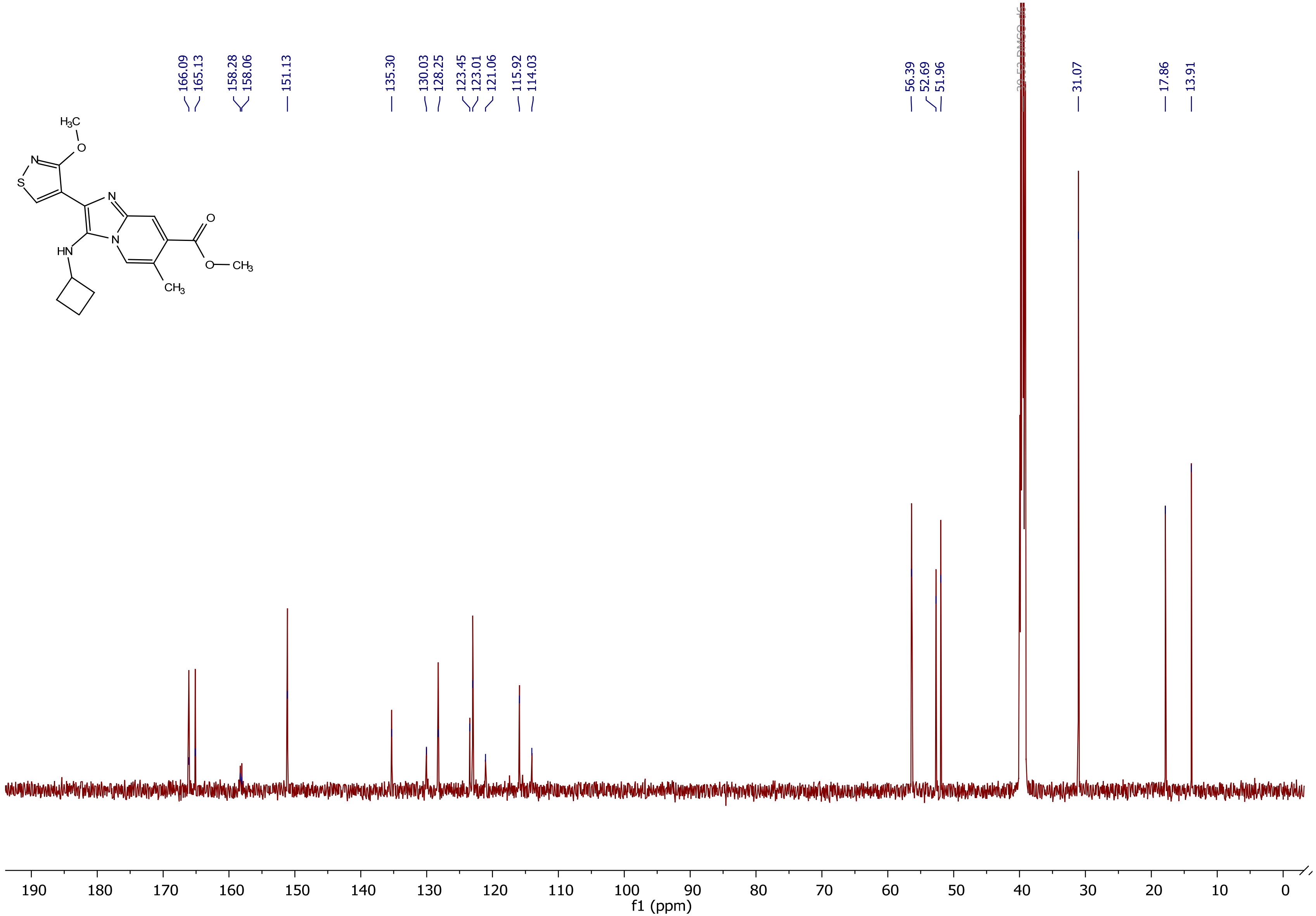

Spectrum 16. Methyl 3-(cyclobutylamino)-2-(3-methoxy-1,2-thiazol-4-yl)-6-methylimidazo[1,2-*a*]pyridine-7-carboxylate trifluoroacetate **4**{180,336,32}, $^{13}C\{^{1}H\}$ NMR (151 MHz, DMSO-d_6)

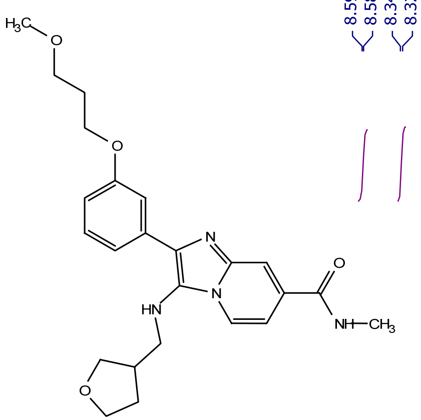

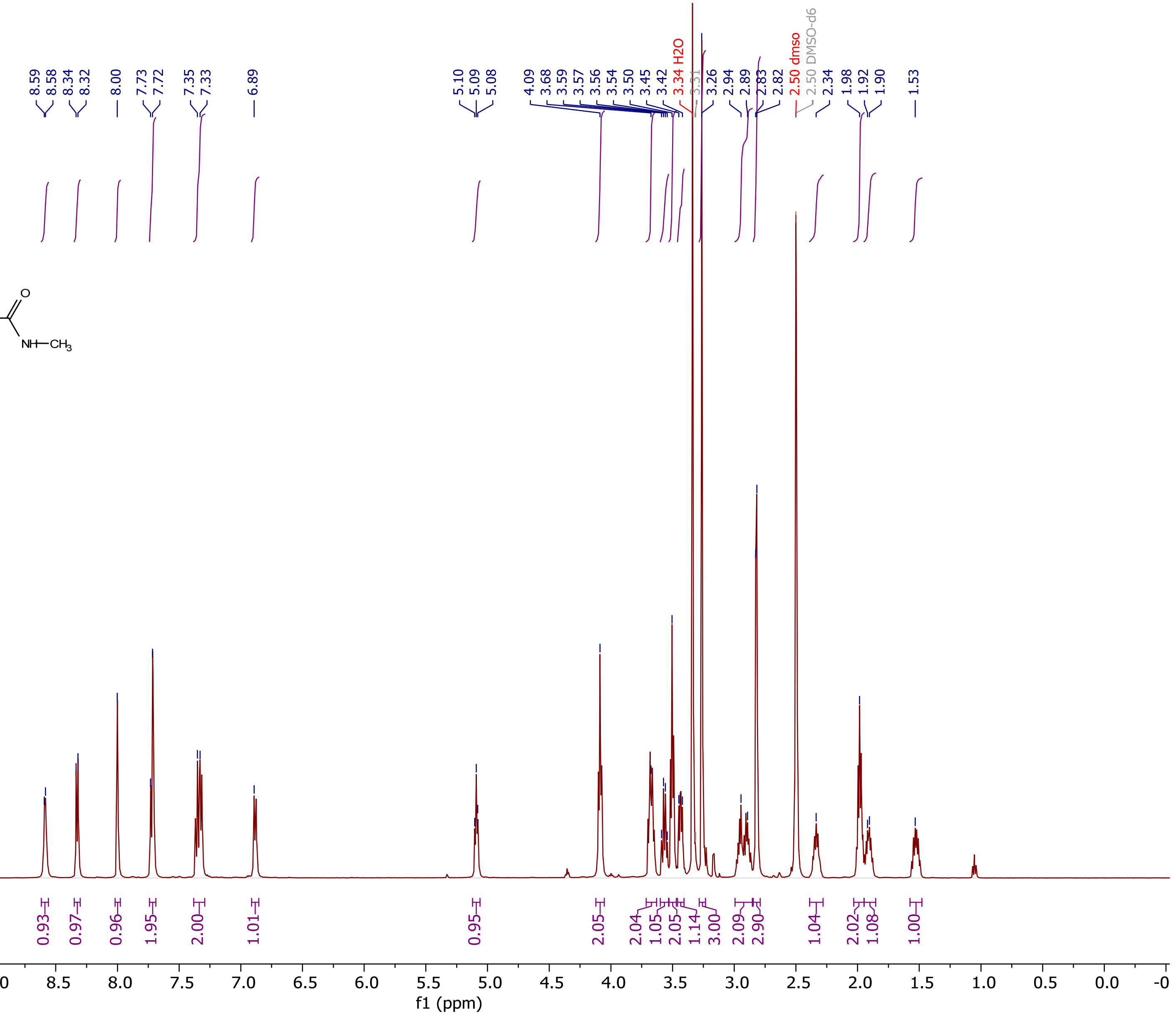

Spectrum 17. 2-[3-(3-Methoxypropoxy)phenyl]-*N*-methyl-3-{[(oxolan-3-yl)methyl]amino}imidazo[1,2-*a*]pyridine-7-carboxamide **4**{69,287,12}, ^{1}H NMR (500 MHz, DMSO-d_6)

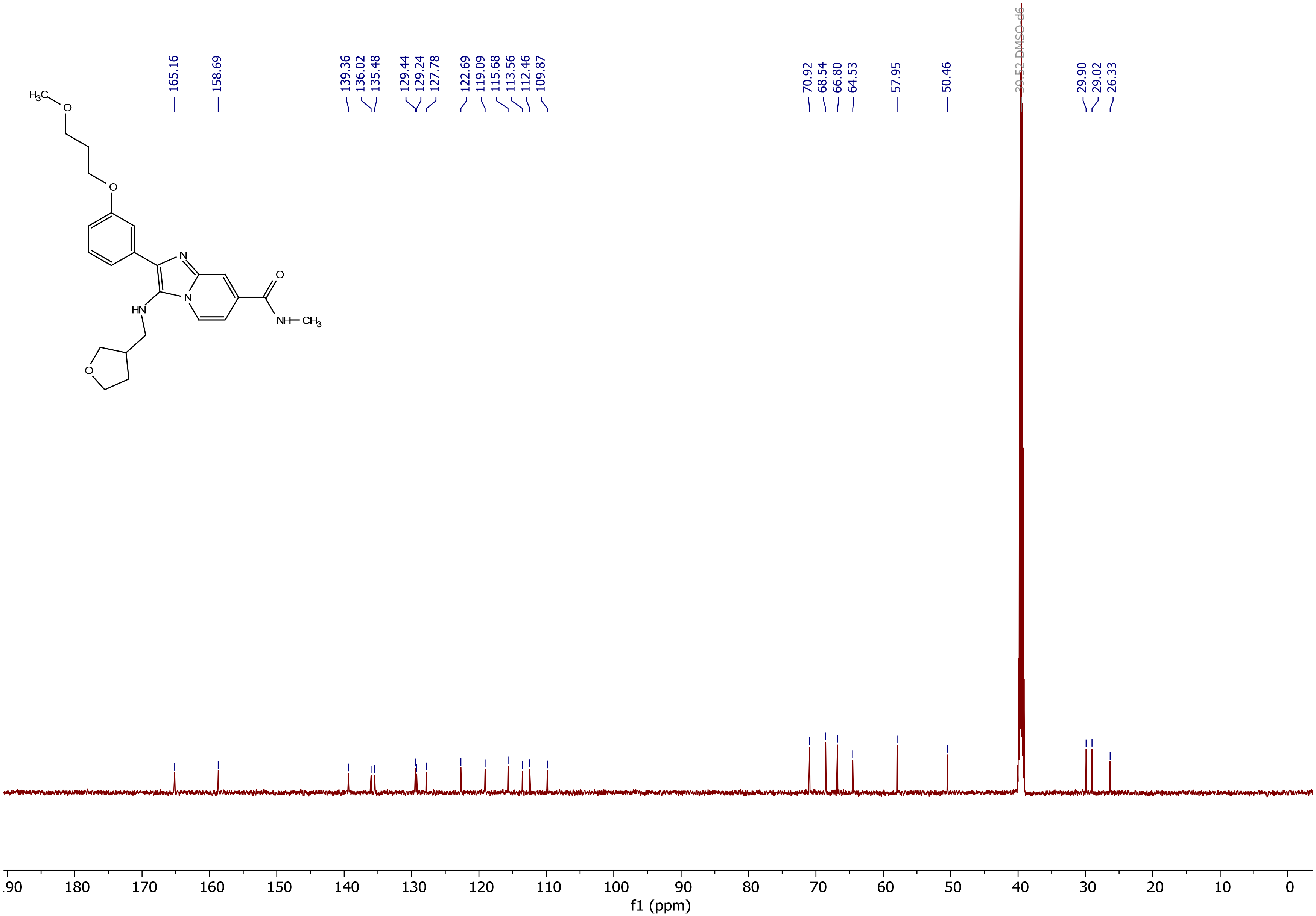

Spectrum 18. 2-[3-(3-Methoxypropoxy)phenyl]-*N*-methyl-3-{[(oxolan-3-yl)methyl]amino}imidazo[1,2-*a*]pyridine-7-carboxamide **4**{69,287,12}, $^{13}C\{^{1}H\}$ NMR (151 MHz, DMSO-d_6)

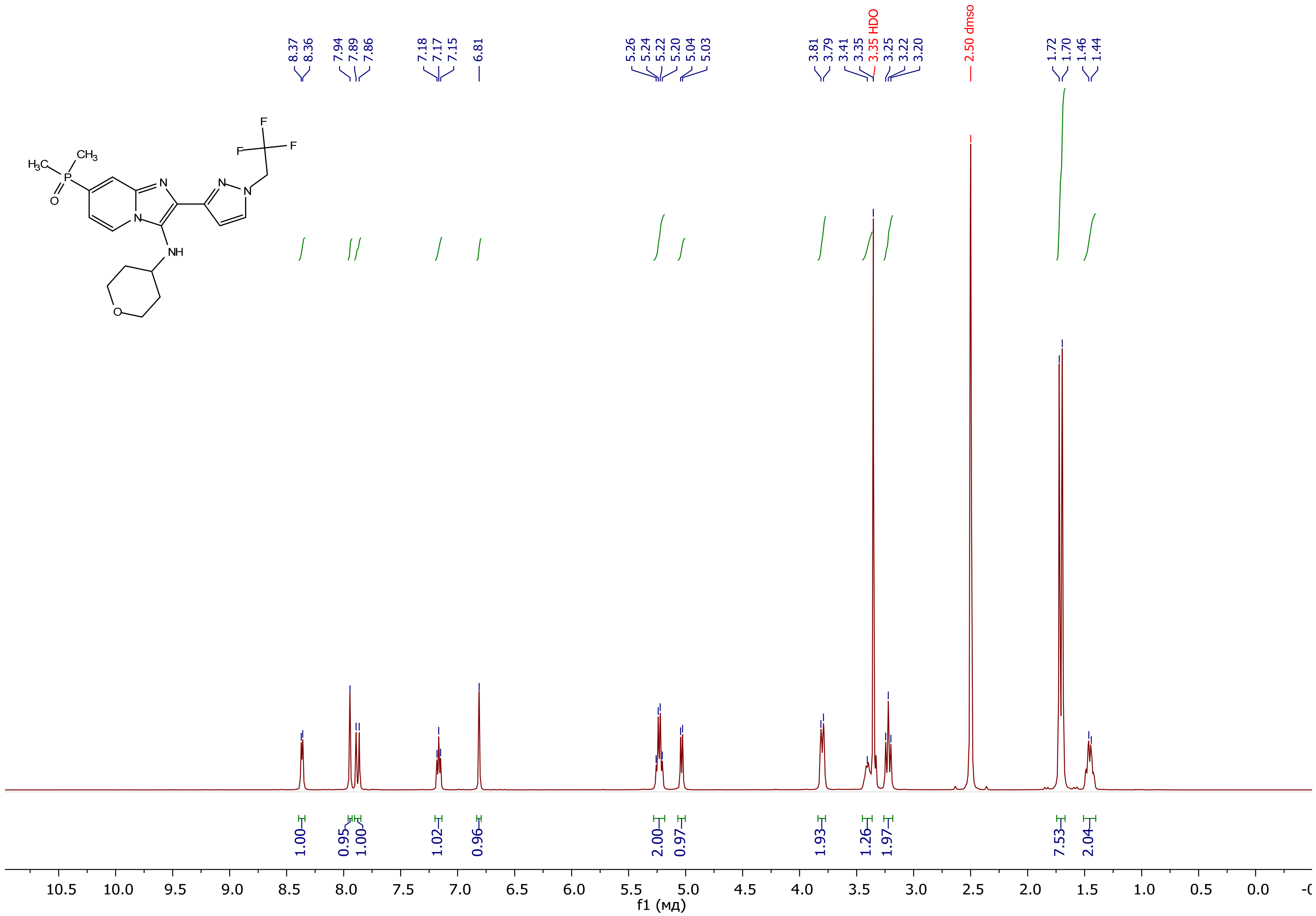

Spectrum 19. 7-(Dimethylphosphoryl)-*N*-(oxan-4-yl)-2-[1-(2,2,2-trifluoroethyl)-1*H*-pyrazol-3-yl]imidazo[1,2-*a*]pyridin-3-amine **4**{32,11,7}, ^{1}H NMR (500 MHz, DMSO-d_6)

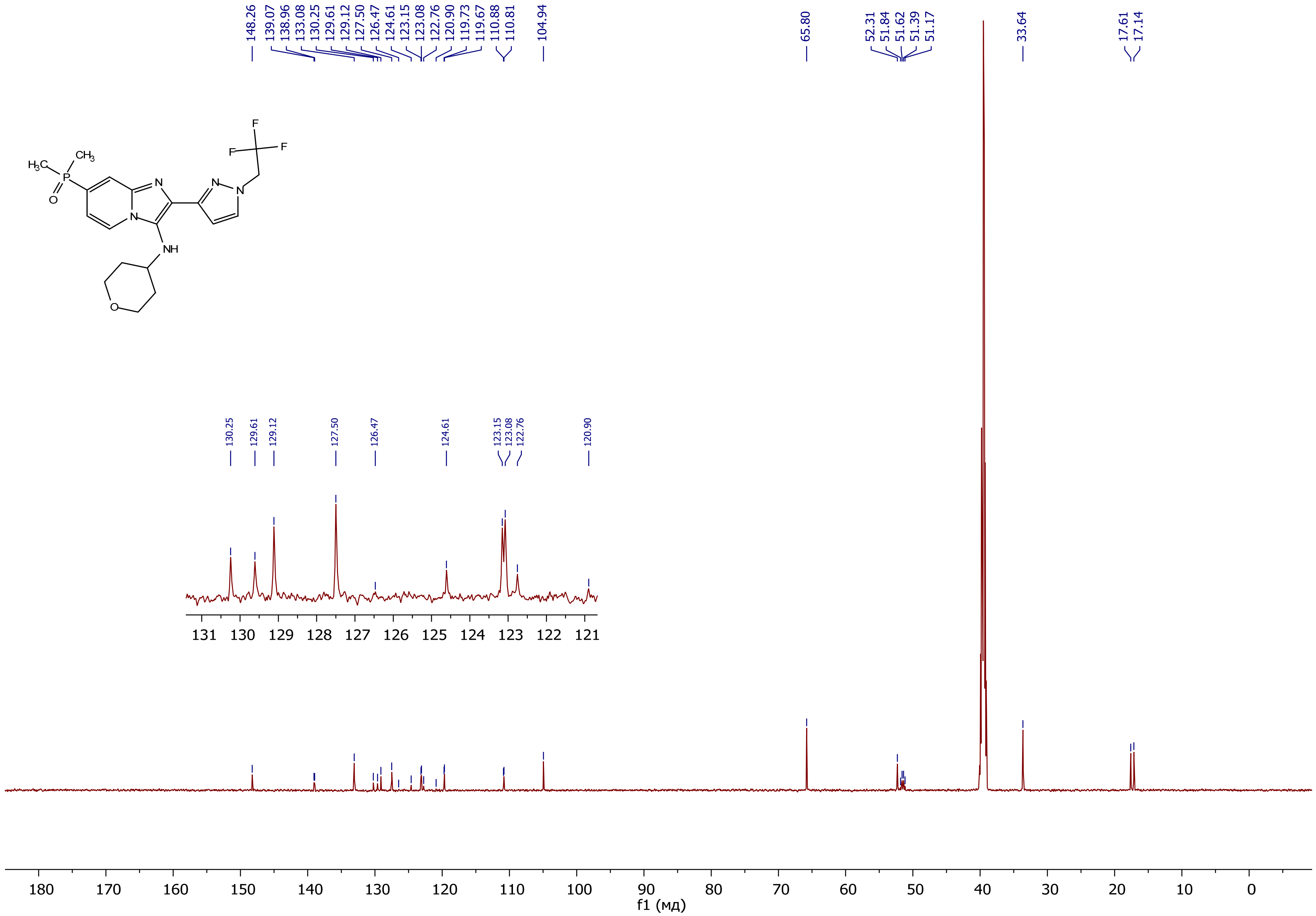

Spectrum 20. 7-(Dimethylphosphoryl)-*N*-(oxan-4-yl)-2-[1-(2,2,2-trifluoroethyl)-1*H*-pyrazol-3-yl]imidazo[1,2-*a*]pyridin-3-amine **4**{32,11,7}, $^{13}C\{^{1}H\}$ NMR (151 MHz, DMSO-d_6)

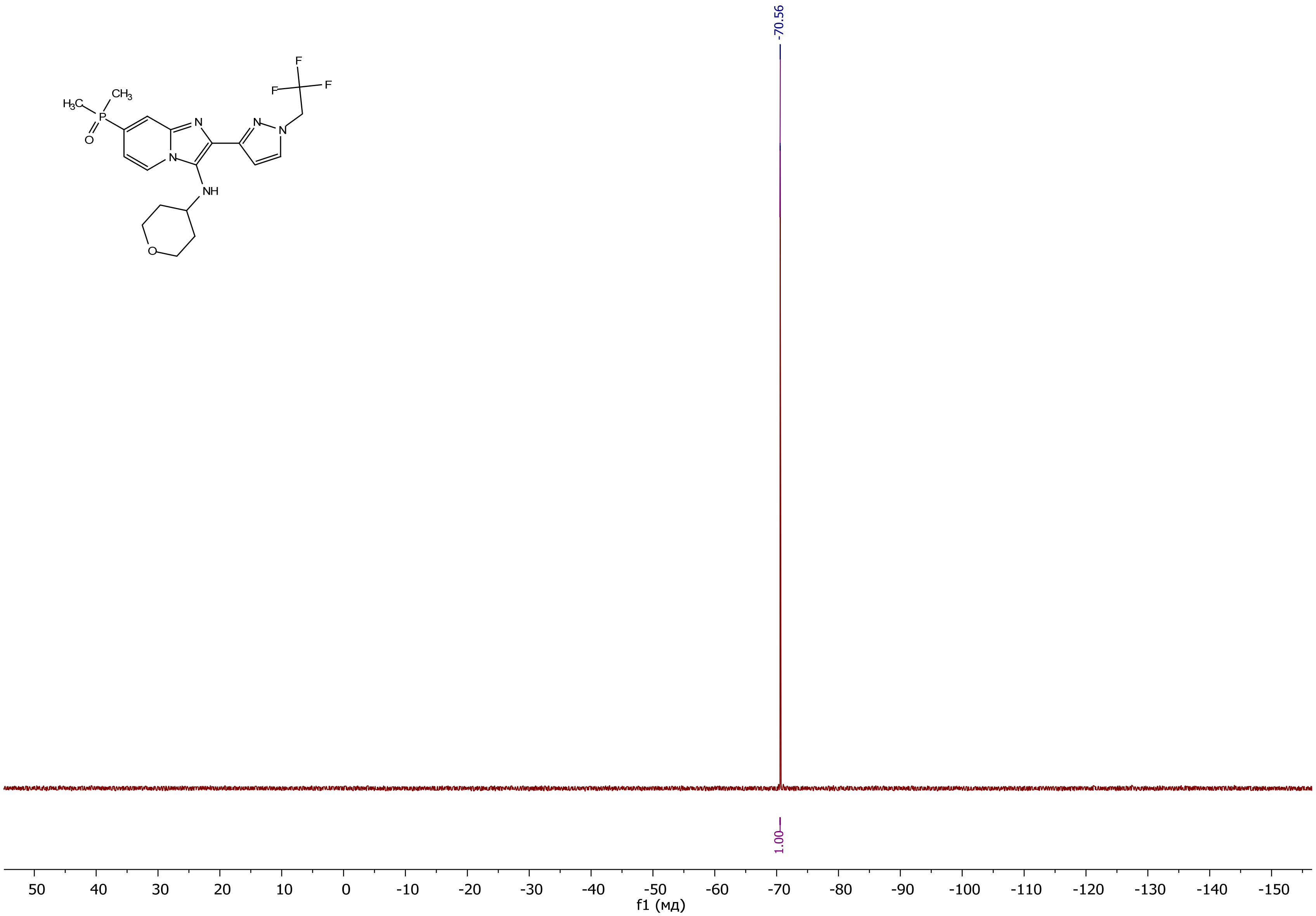

Spectrum 21. 7-(Dimethylphosphoryl)-*N*-(oxan-4-yl)-2-[1-(2,2,2-trifluoroethyl)-1*H*-pyrazol-3-yl]imidazo[1,2-*a*]pyridin-3-amine **4**{32,11,7}, $^{19}F\{^{1}H\}$ NMR (376 MHz, DMSO-d_6)

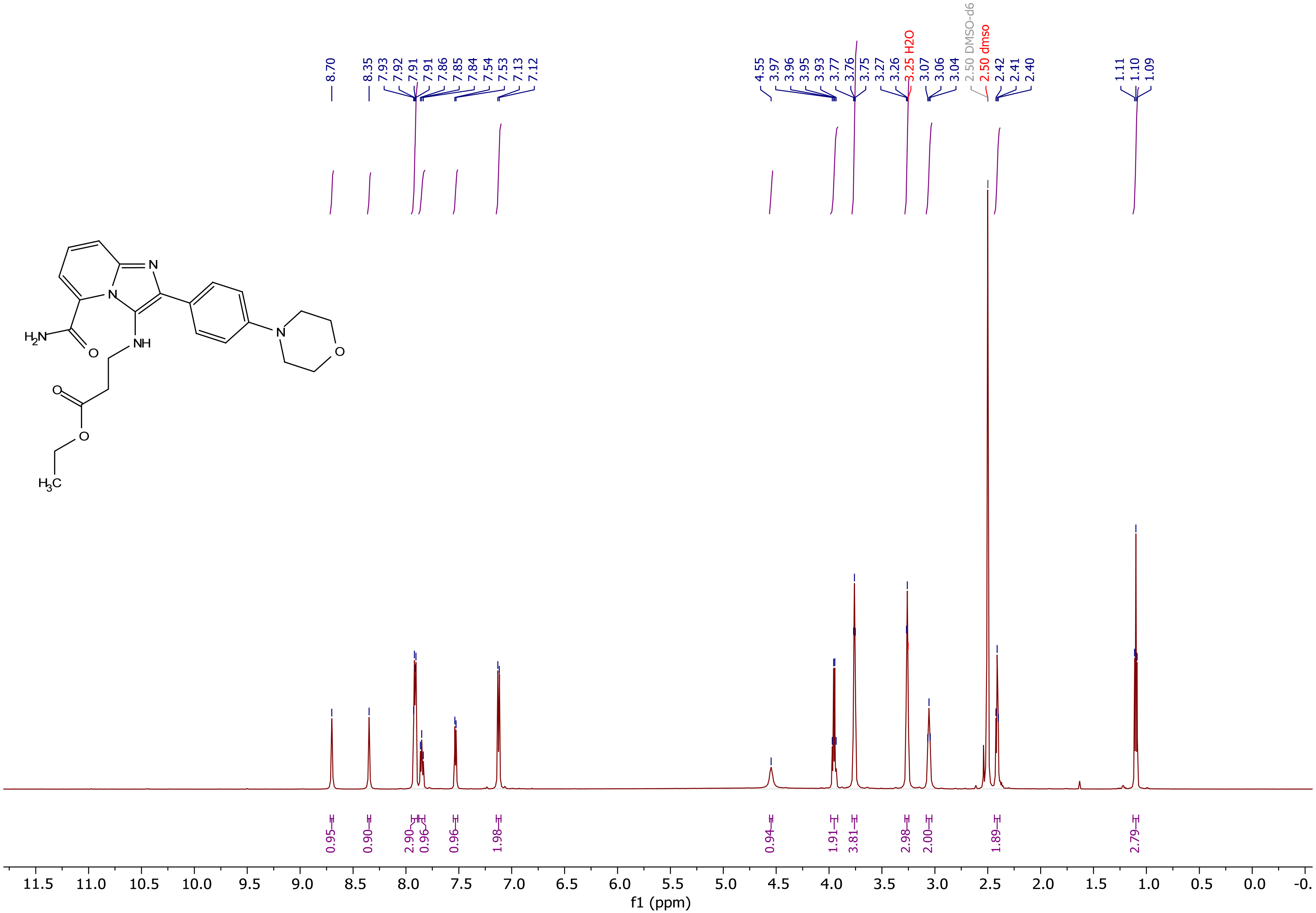

Spectrum 22. Ethyl 3-({5-carbamoyl-2-[4-(morpholin-4-yl)phenyl]imidazo[1,2-*a*]pyridin-3-yl}amino)propanoate **4**{105,591,29}, ^{1}H NMR (600 MHz, DMSO-d_6)

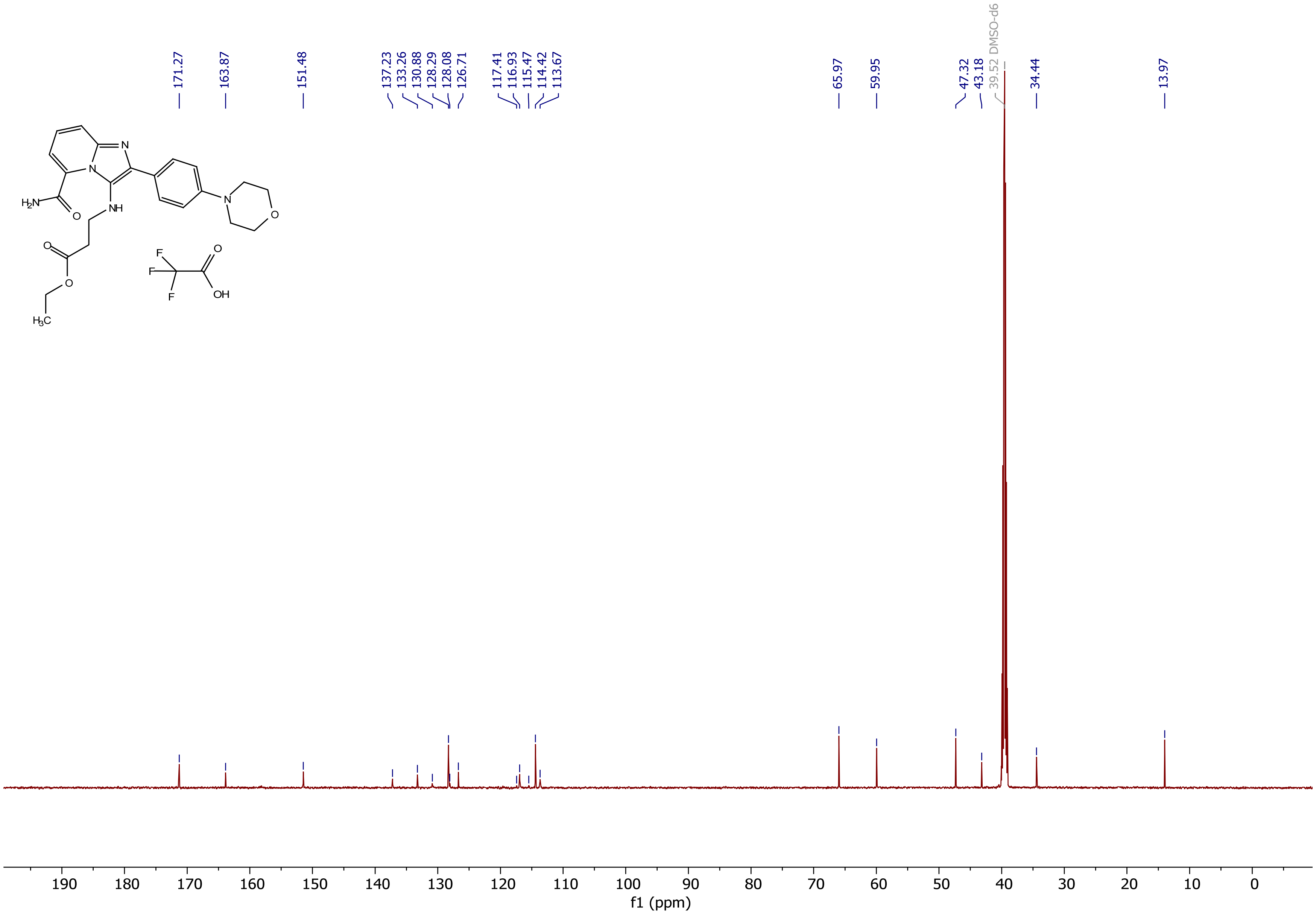

Spectrum 23. Ethyl 3-({5-carbamoyl-2-[4-(morpholin-4-yl)phenyl]imidazo[1,2-*a*]pyridin-3-yl}amino)propanoate **4**{105,591,29}, $^{13}C\{^{1}H\}$ NMR (151 MHz, DMSO-d_6)

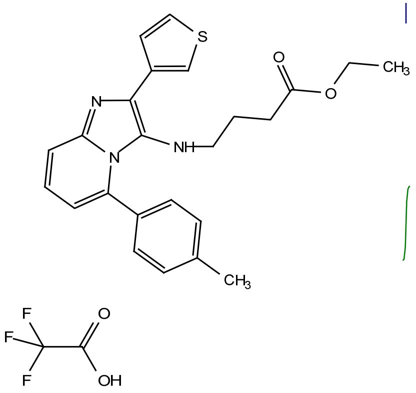

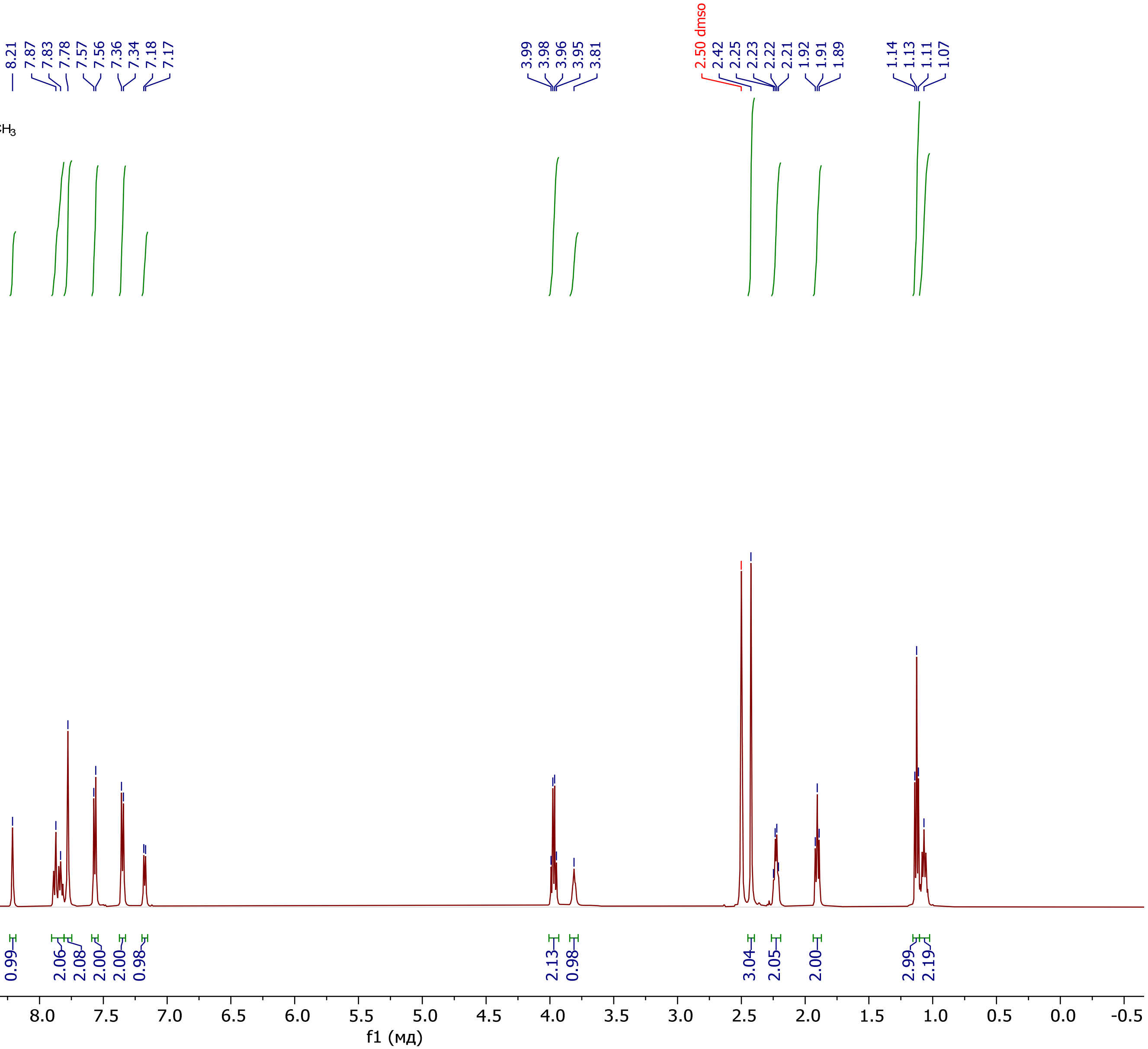

Spectrum 24. Ethyl 4-{[5-(4-methylphenyl)-2-(thiophen-3-yl)imidazo[1,2-*a*]pyridin-3-yl]amino}butanoate trifluoroacetate **4**{30,9,6}, ^{1}H NMR (500 MHz, DMSO-d_6)

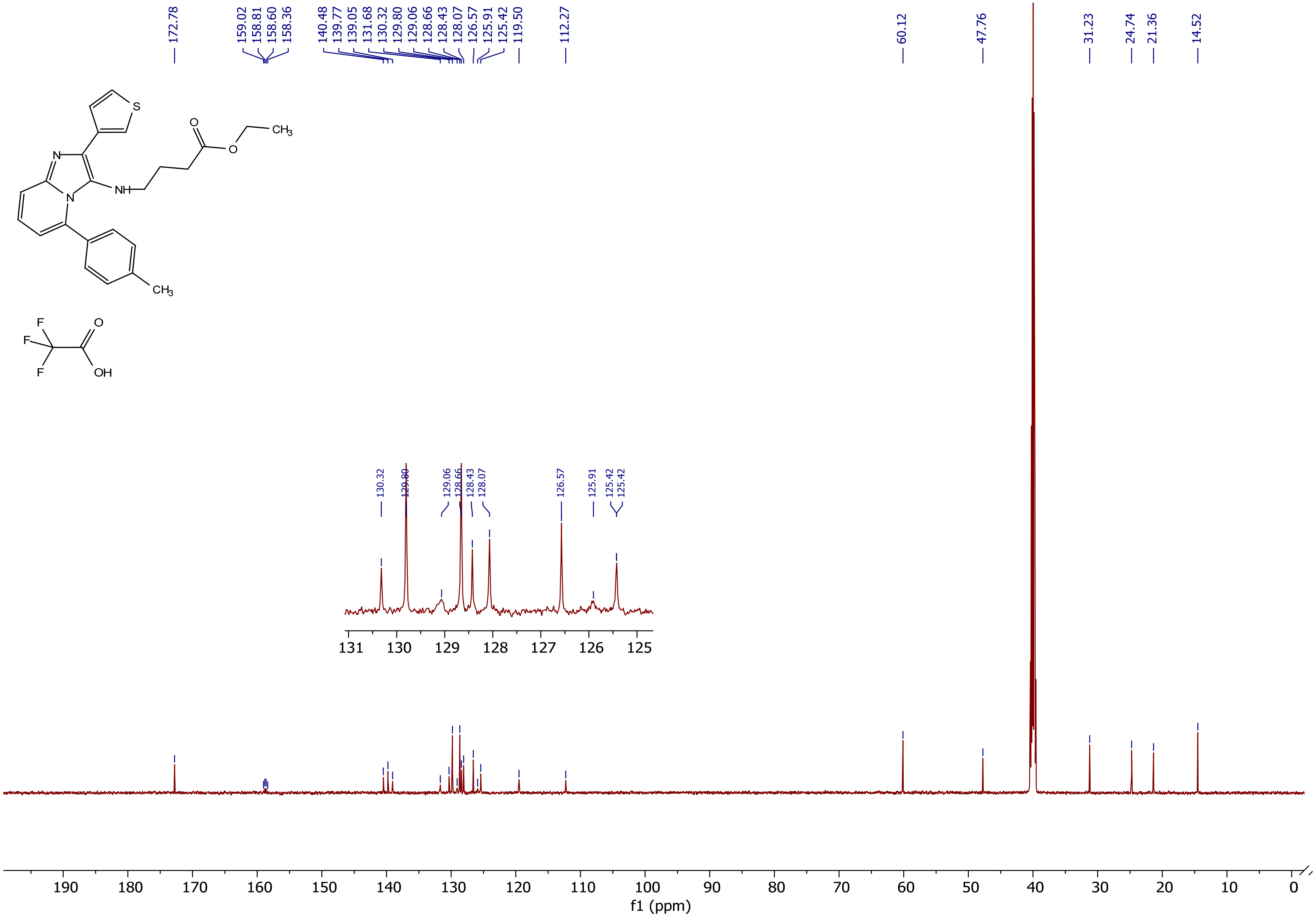

Spectrum 25. Ethyl 4-{[5-(4-methylphenyl)-2-(thiophen-3-yl)imidazo[1,2-*a*]pyridin-3-yl]amino}butanoate trifluoroacetate **4**{30,9,6}, $^{13}C\{^{1}H\}$ NMR (151 MHz, DMSO-d_6)

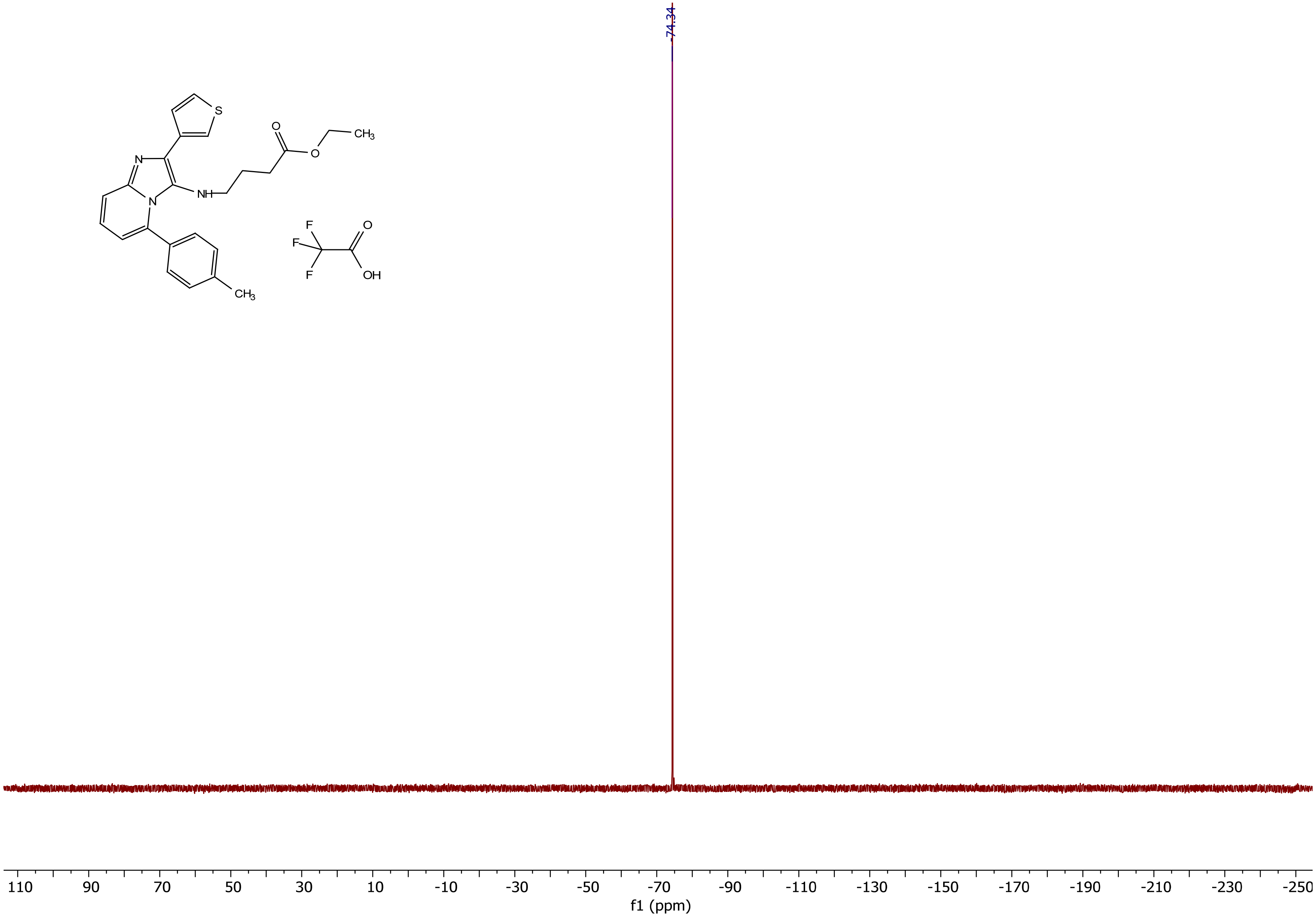

Spectrum 26. Ethyl 4-{[5-(4-methylphenyl)-2-(thiophen-3-yl)imidazo[1,2-*a*]pyridin-3-yl]amino}butanoate trifluoroacetate **4**{30,9,6}, $^{19}F\{^{1}H\}$ NMR (376 MHz, DMSO-d_6)

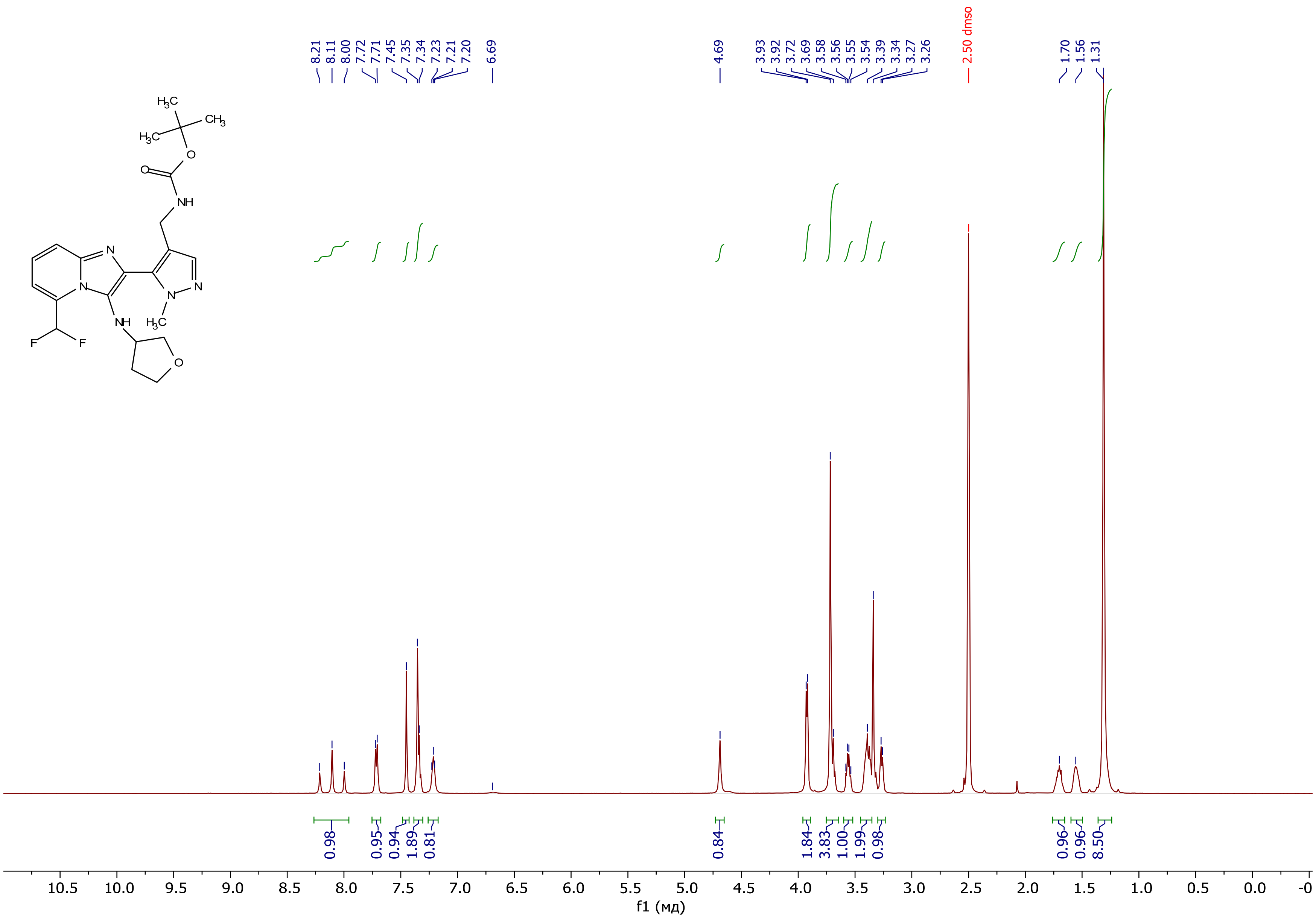

Spectrum 27. *tert*-Butyl N-({5-[5-(difluoromethyl)-3-[(oxolan-3-yl)amino]imidazo[1,2-*a*]pyridin-2-yl]-1-methyl-1*H*-pyrazol-4-yl}methyl)carbamate **4**{37,16,10}, ^{1}H NMR (500 MHz, DMSO-d_6)

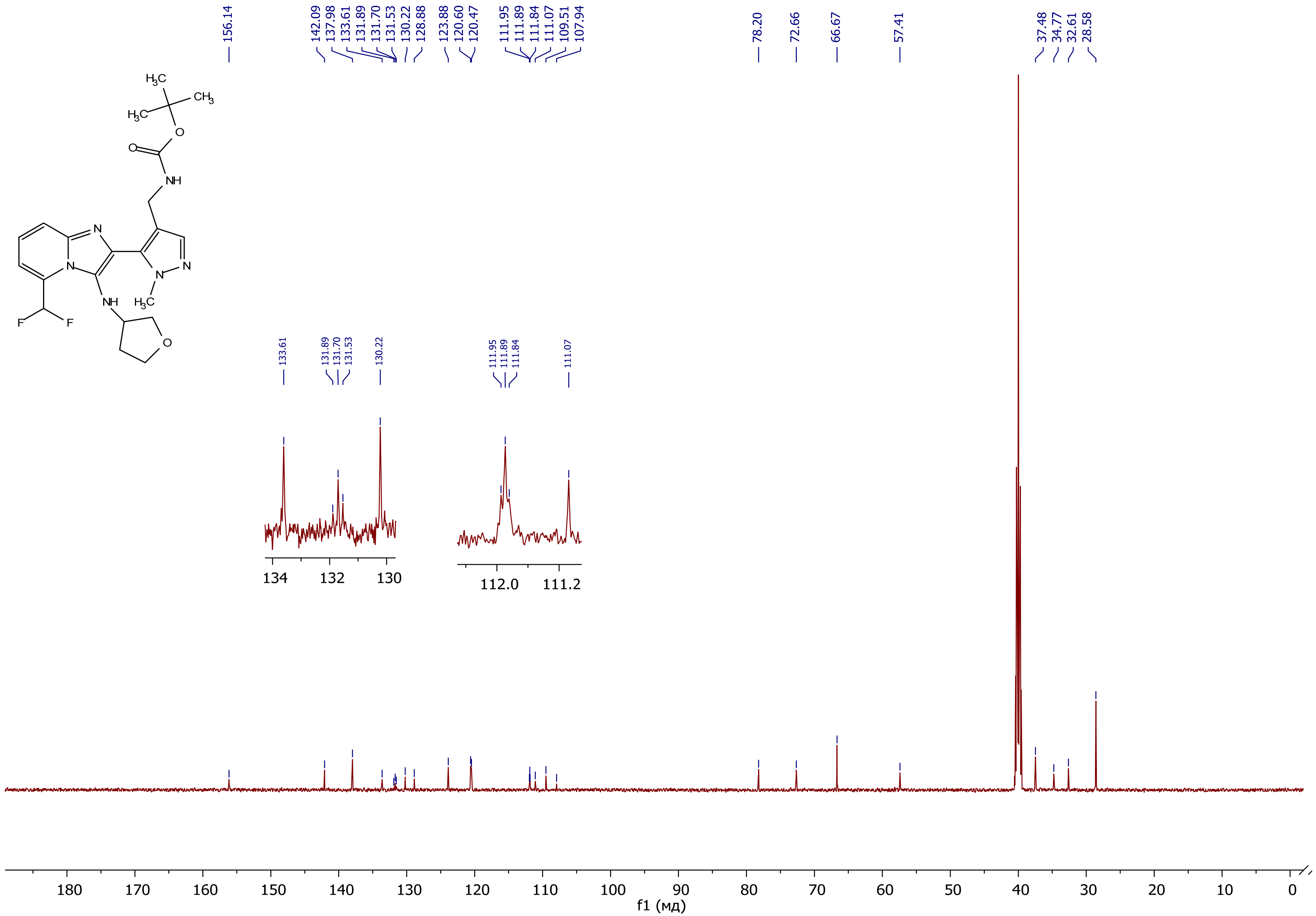

Spectrum 28. *tert*-Butyl N-({5-[5-(difluoromethyl)-3-[(oxolan-3-yl)amino]imidazo[1,2-*a*]pyridin-2-yl]-1-methyl-1*H*-pyrazol-4-yl}methyl)carbamate **4**{37,16,10}, $^{13}C\{^{1}H\}$ NMR (151 MHz, DMSO-d_6)

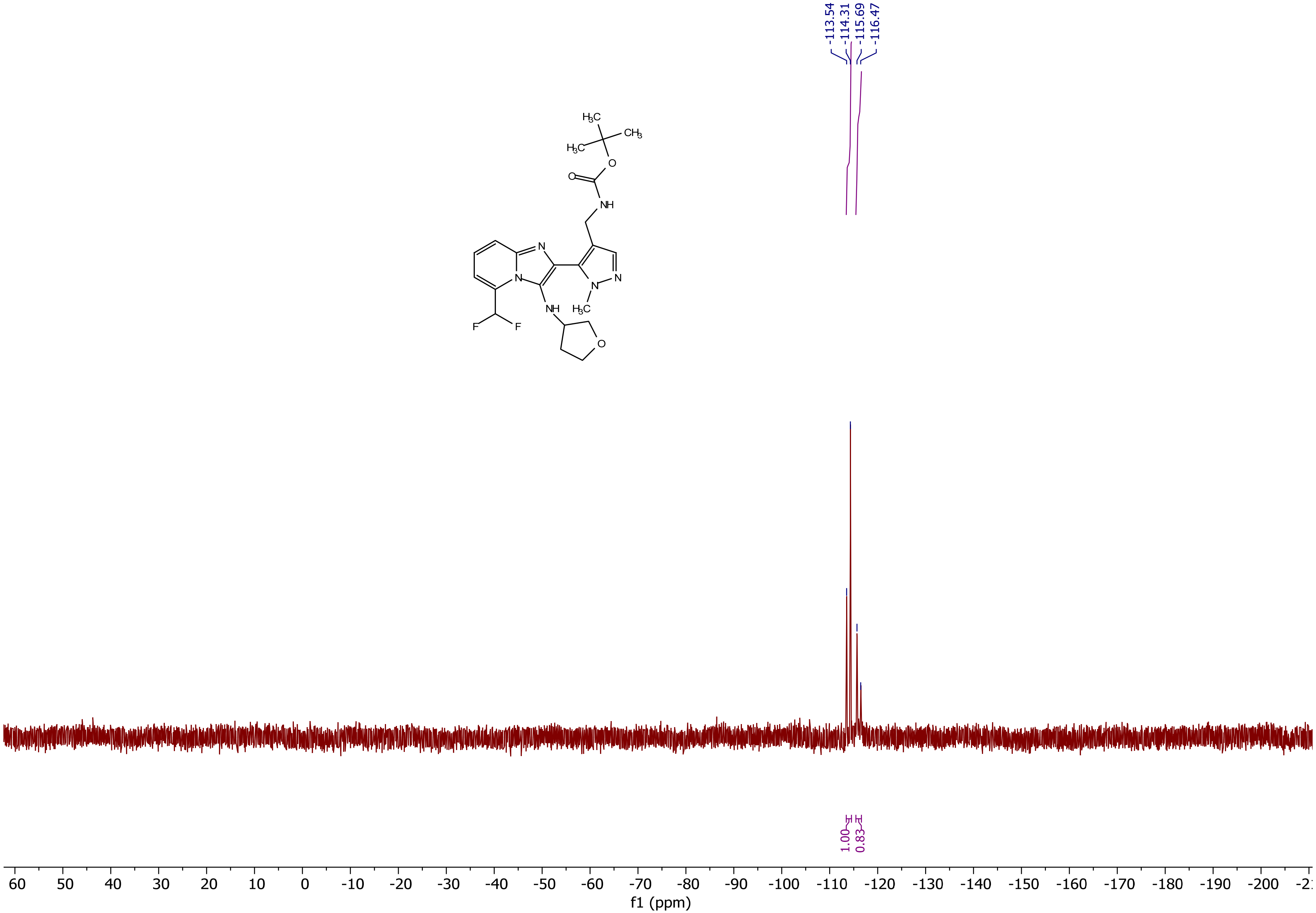

Spectrum 29. *tert*-Butyl N-({5-[5-(difluoromethyl)-3-[(oxolan-3-yl)amino]imidazo[1,2-*a*]pyridin-2-yl]-1-methyl-1*H*-pyrazol-4-yl}methyl)carbamate **4**{37,16,10}, $^{19}F\{^{1}H\}$ NMR (376 MHz, DMSO-d_6)

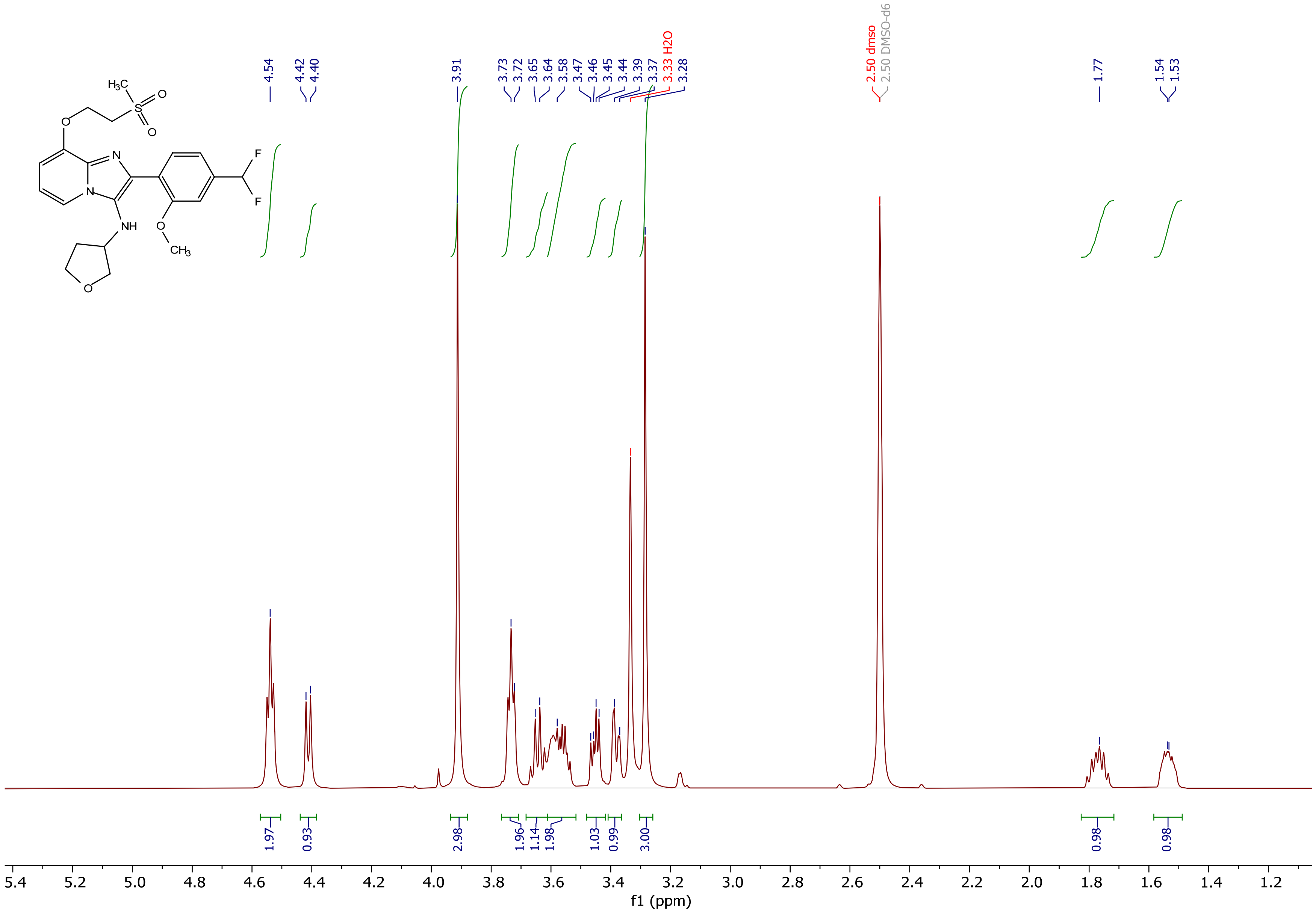

Spectrum 30. 2-[4-(Difluoromethyl)-2-methoxyphenyl]-8-(2-methanesulfonylethoxy)-*N*-(oxolan-3-yl)imidazo[1,2-*a*]pyridin-3-amine **4**{142,605,10}, ^{1}H NMR (500 MHz, DMSO-d_6)

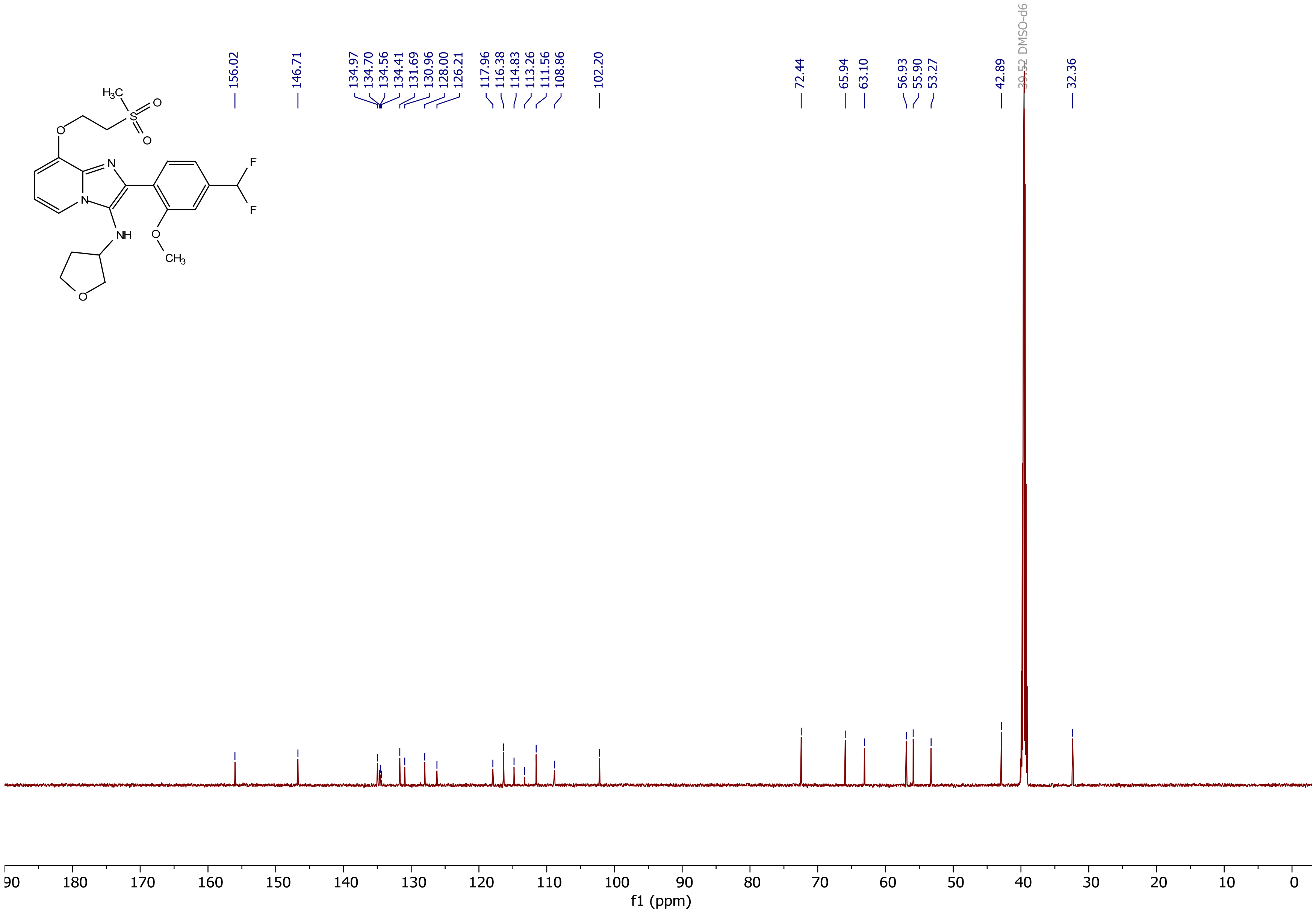

Spectrum 31. 2-[4-(Difluoromethyl)-2-methoxyphenyl]-8-(2-methanesulfonylethoxy)-*N*-(oxolan-3-yl)imidazo[1,2-*a*]pyridin-3-amine **4**{142,605,10}, $^{13}C\{^{1}H\}$ NMR (151 MHz, DMSO-d_6)

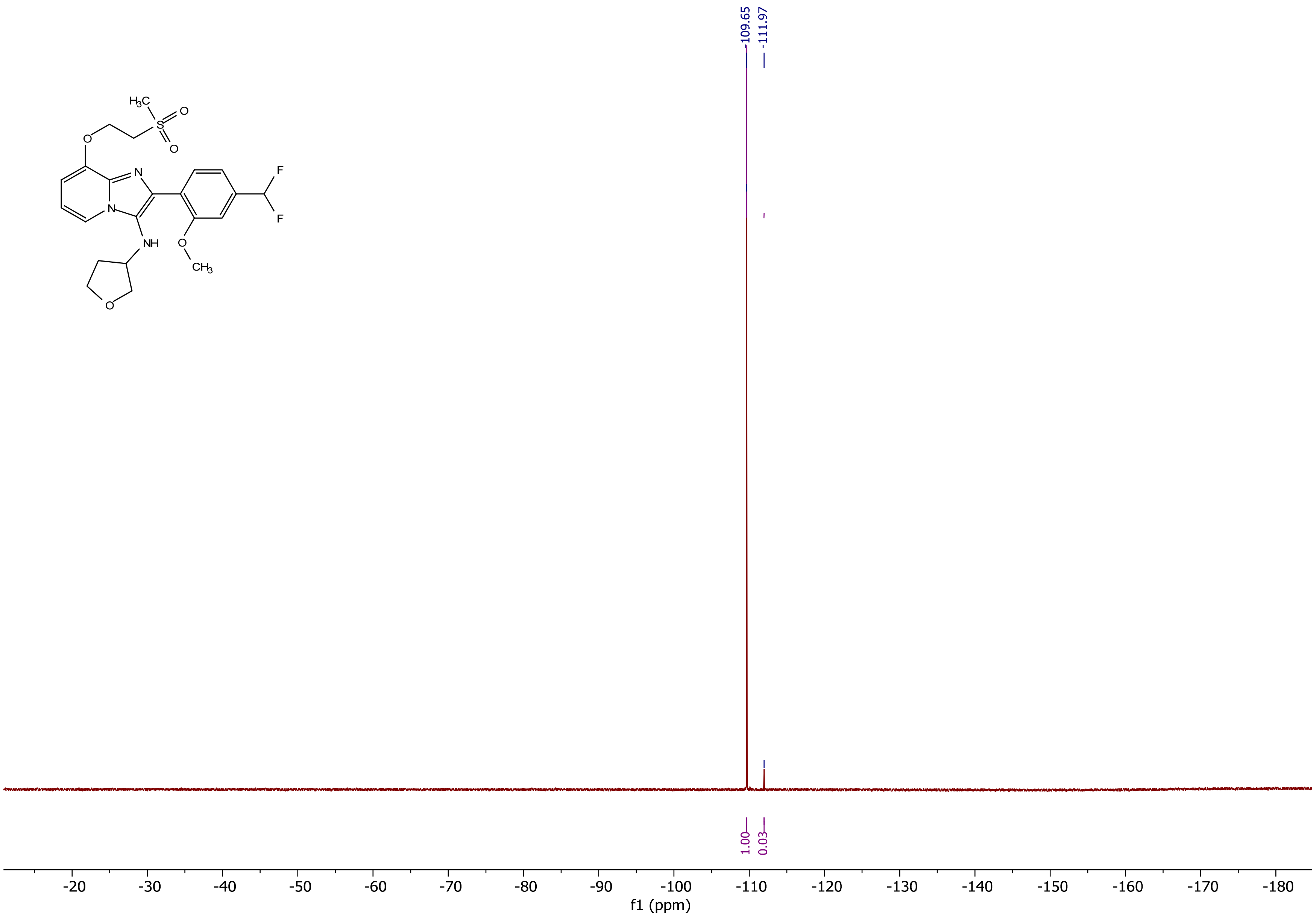

Spectrum 32. 2-[4-(Difluoromethyl)-2-methoxyphenyl]-8-(2-methanesulfonylethoxy)-*N*-(oxolan-3-yl)imidazo[1,2-*a*]pyridin-3-amine **4**{142,605,10}, $^{19}F\{^{1}H\}$ NMR (376 MHz, DMSO-d_6)

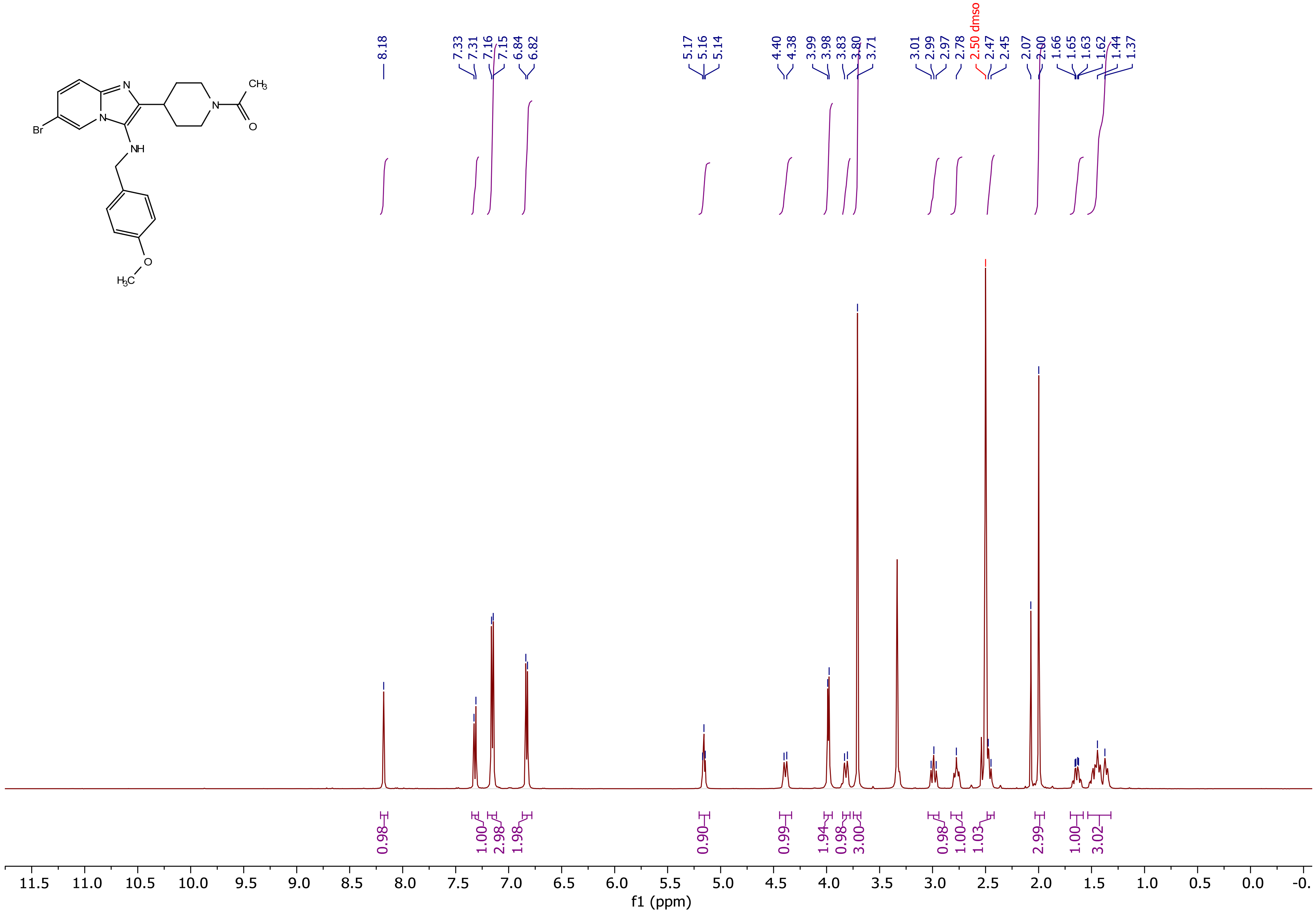

Spectrum 33. 1-[4-(6-Bromo-3-{[(4-methoxyphenyl)methyl]amino}imidazo[1,2-*a*]pyridin-2-yl)piperidin-1-yl]ethan-1-one **4**{36,30,13}, ^{1}H NMR (500 MHz, DMSO-d_6)

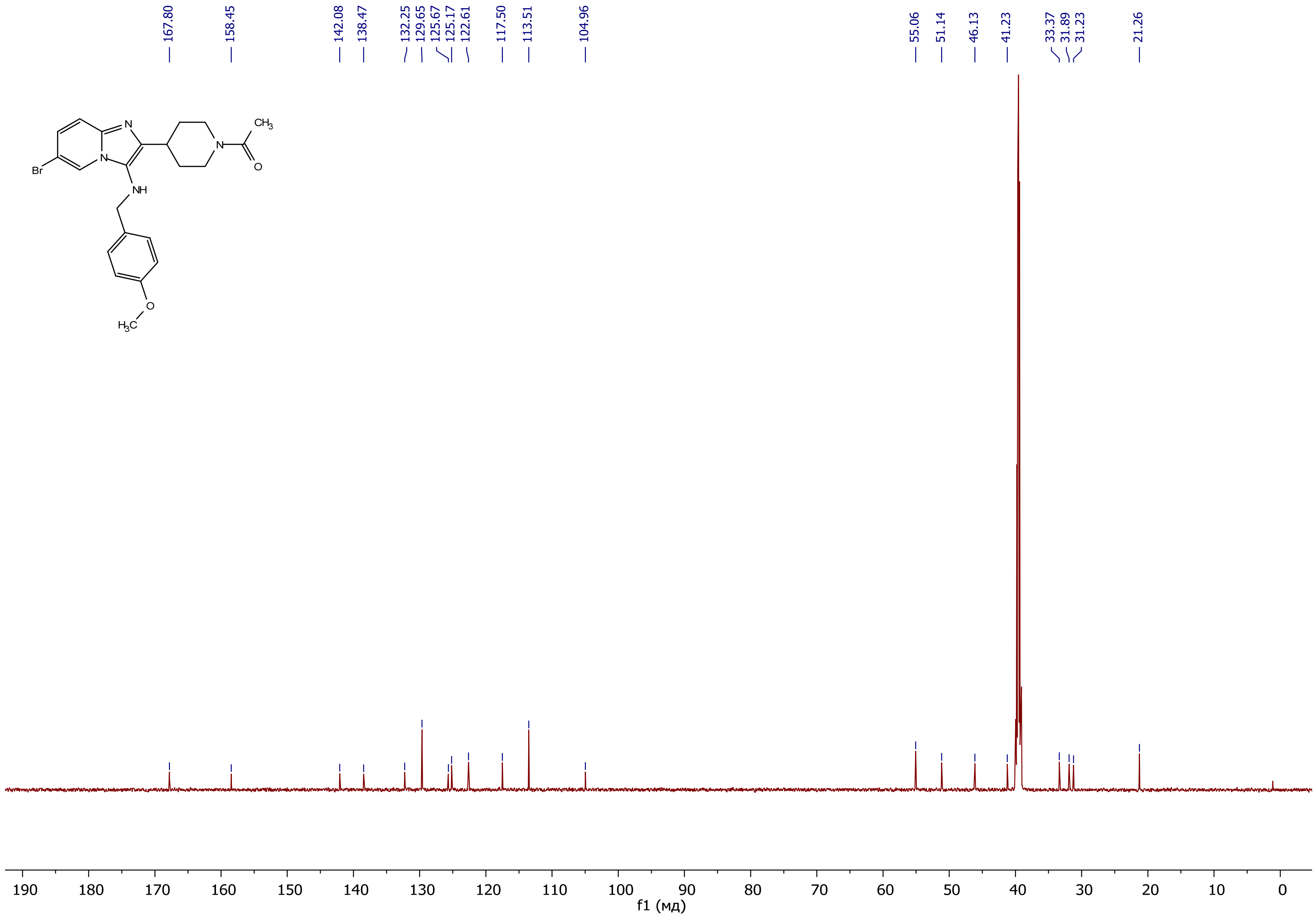

Spectrum 34. 1-[4-(6-Bromo-3-{[(4-methoxyphenyl)methyl]amino}imidazo[1,2-*a*]pyridin-2-yl)piperidin-1-yl]ethan-1-one **4**{36,30,13}, $^{13}C\{^{1}H\}$ NMR (151 MHz, DMSO-d_6)

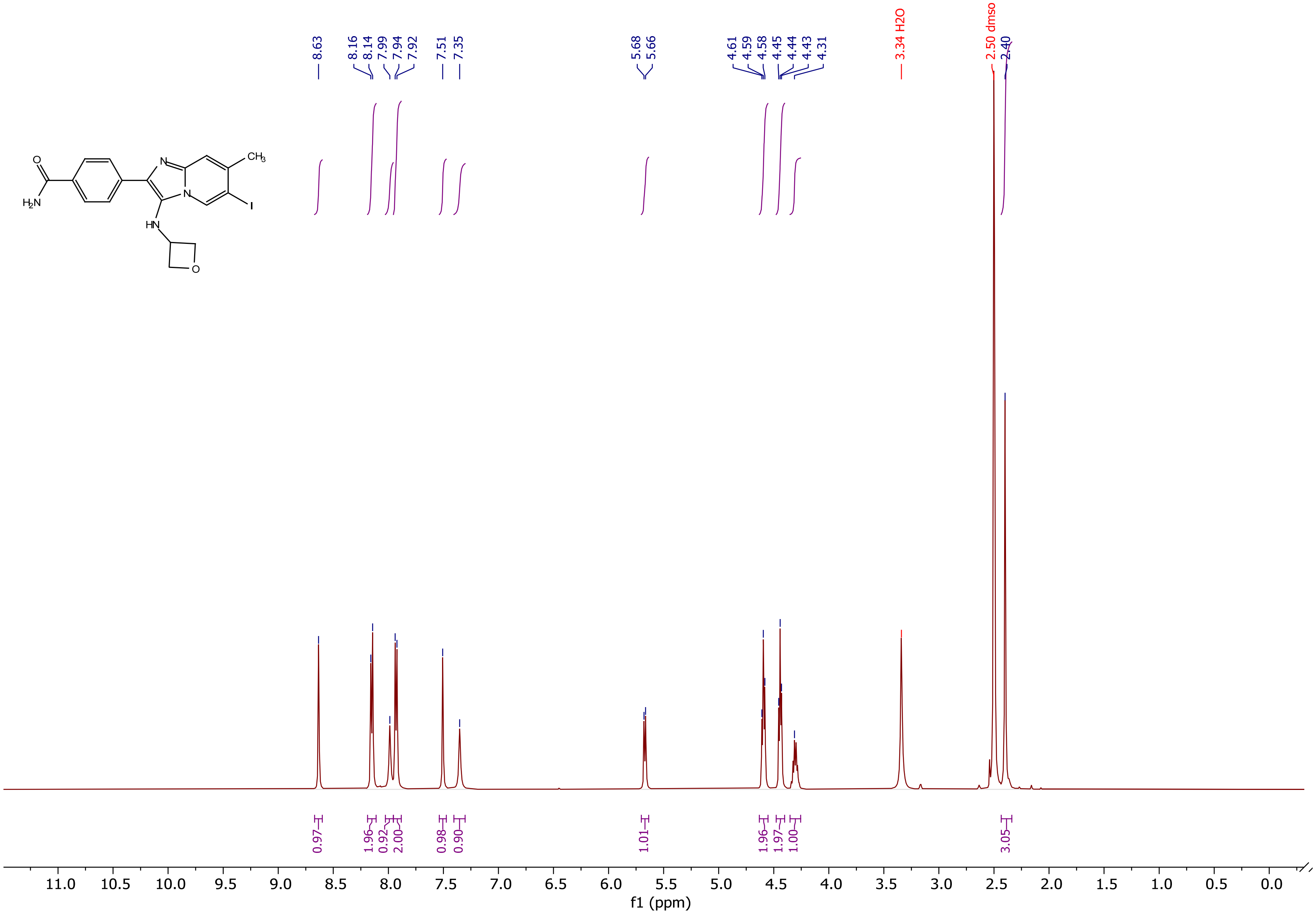

Spectrum 35. 4-{6-Iodo-7-methyl-3-[(oxetan-3-yl)amino]imidazo[1,2-*a*]pyridin-2-yl}benzamide **4**{432,452,22}, ^{1}H NMR (500 MHz, DMSO-d_6)

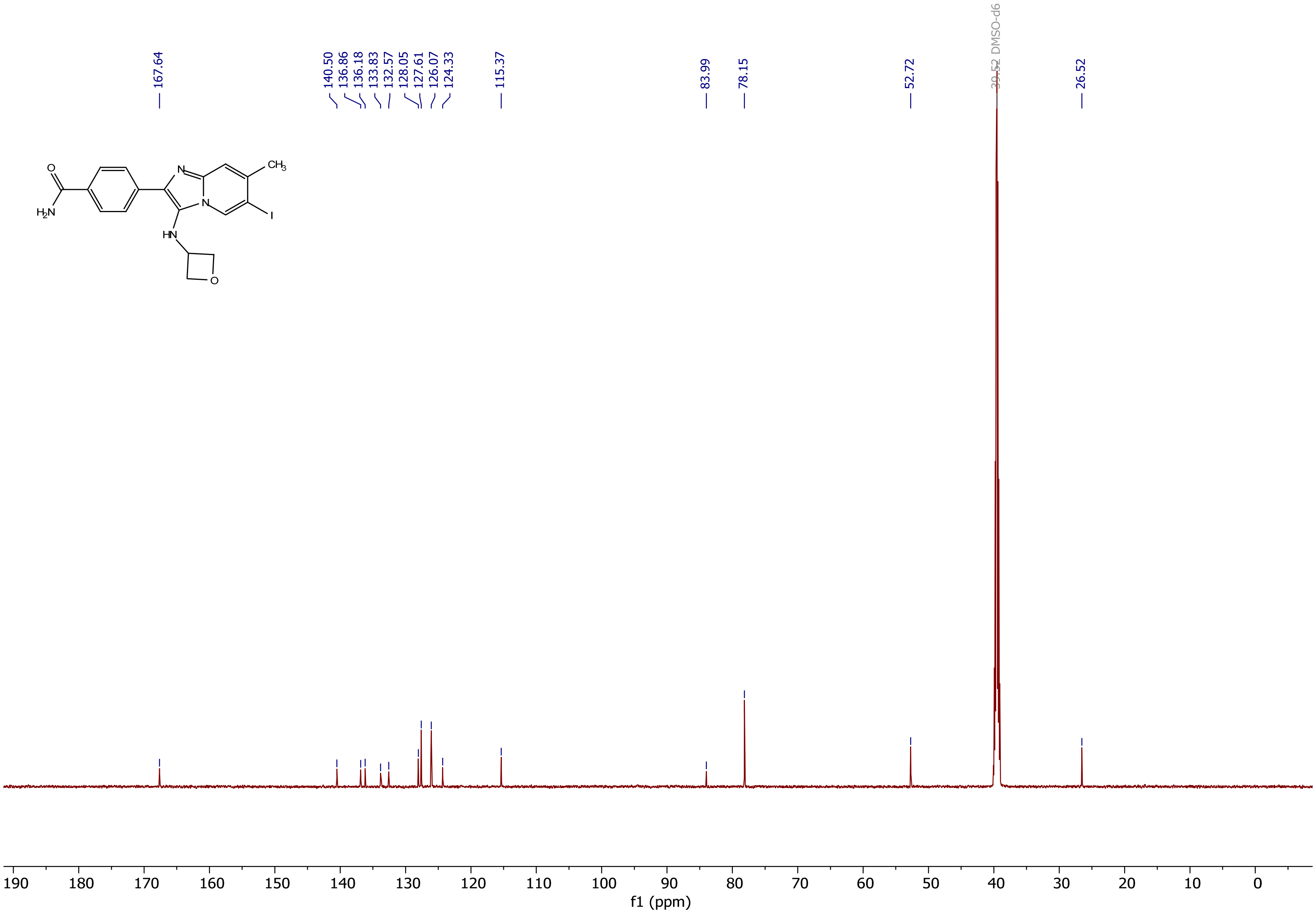

Spectrum 36. 4-{6-Iodo-7-methyl-3-[(oxetan-3-yl)amino]imidazo[1,2-*a*]pyridin-2-yl}benzamide **4**{432,452,22}, $^{13}C\{^{1}H\}$ NMR (151 MHz, DMSO-d_6)

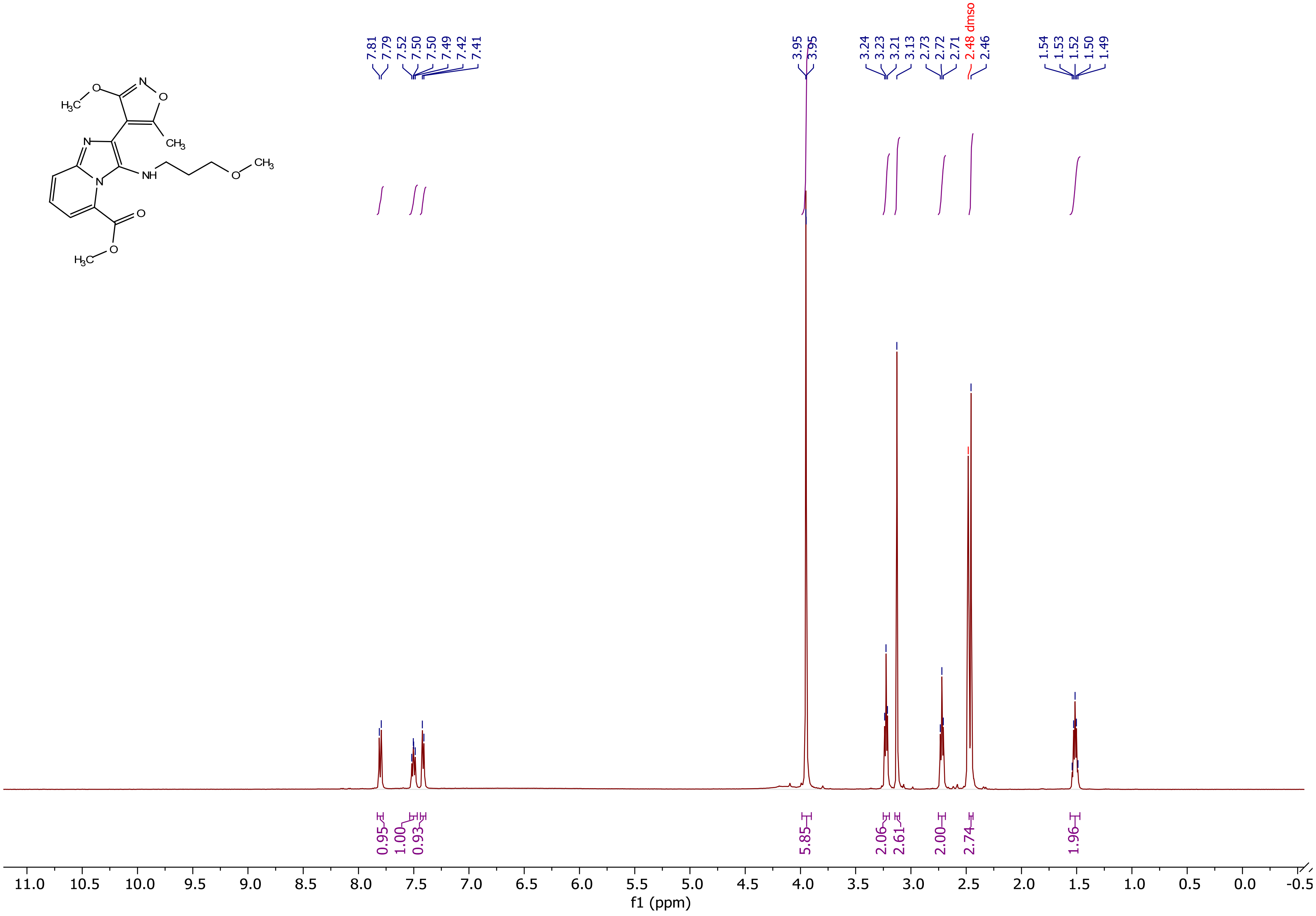

Spectrum 37. Methyl 2-(3-methoxy-5-methyl-1,2-oxazol-4-yl)-3-[(3-methoxypropyl)amino]imidazo[1,2-*a*]pyridine-5-carboxylate trifluoroacetate **4**{374,587,5}, ^{1}H NMR (500 MHz, DMSO-d_6)

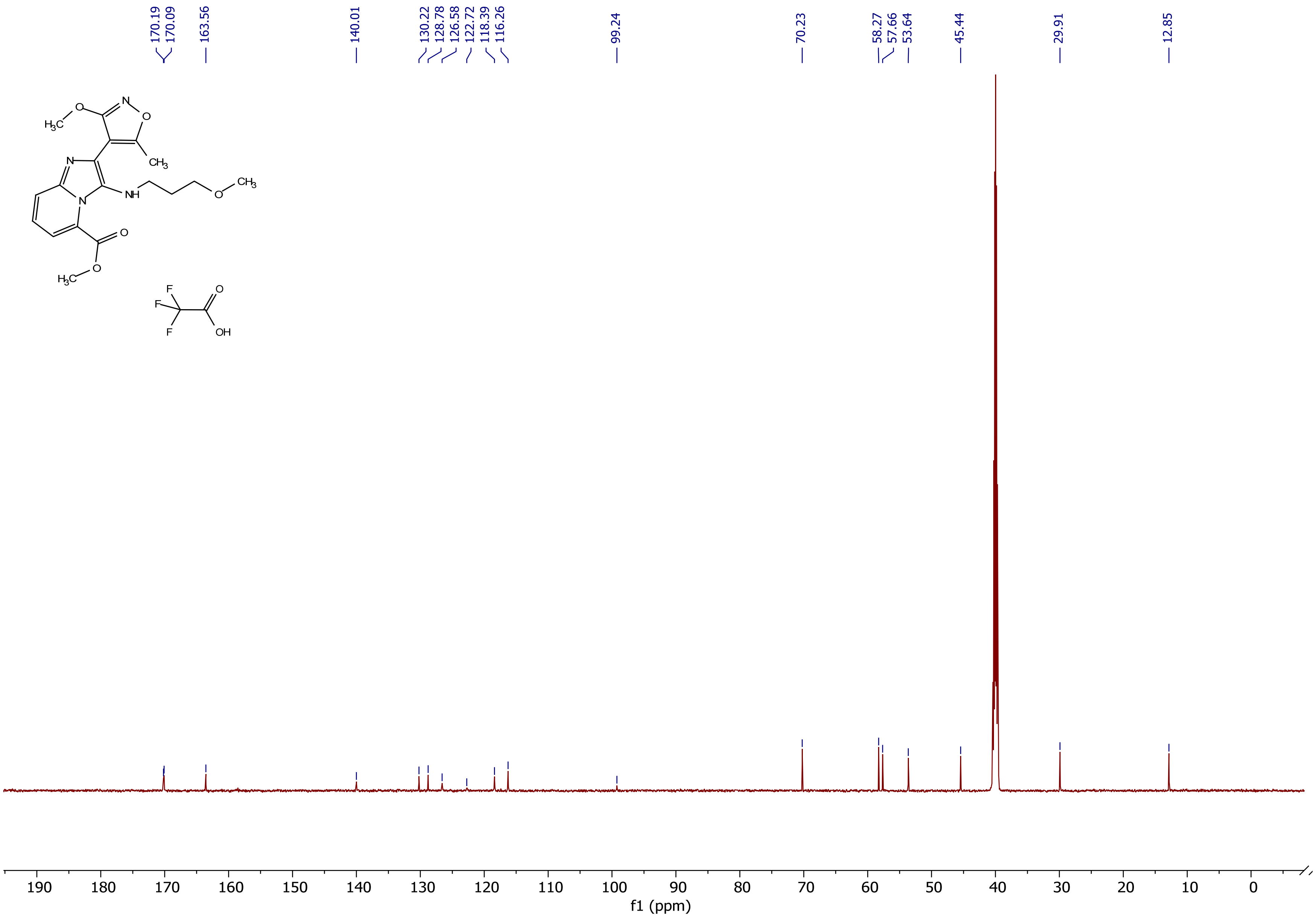

Spectrum 38. Methyl 2-(3-methoxy-5-methyl-1,2-oxazol-4-yl)-3-[(3-methoxypropyl)amino]imidazo[1,2-*a*]pyridine-5-carboxylate trifluoroacetate **4**{374,587,5}, $^{13}C\{^{1}H\}$ NMR (151 MHz, DMSO-d_6)

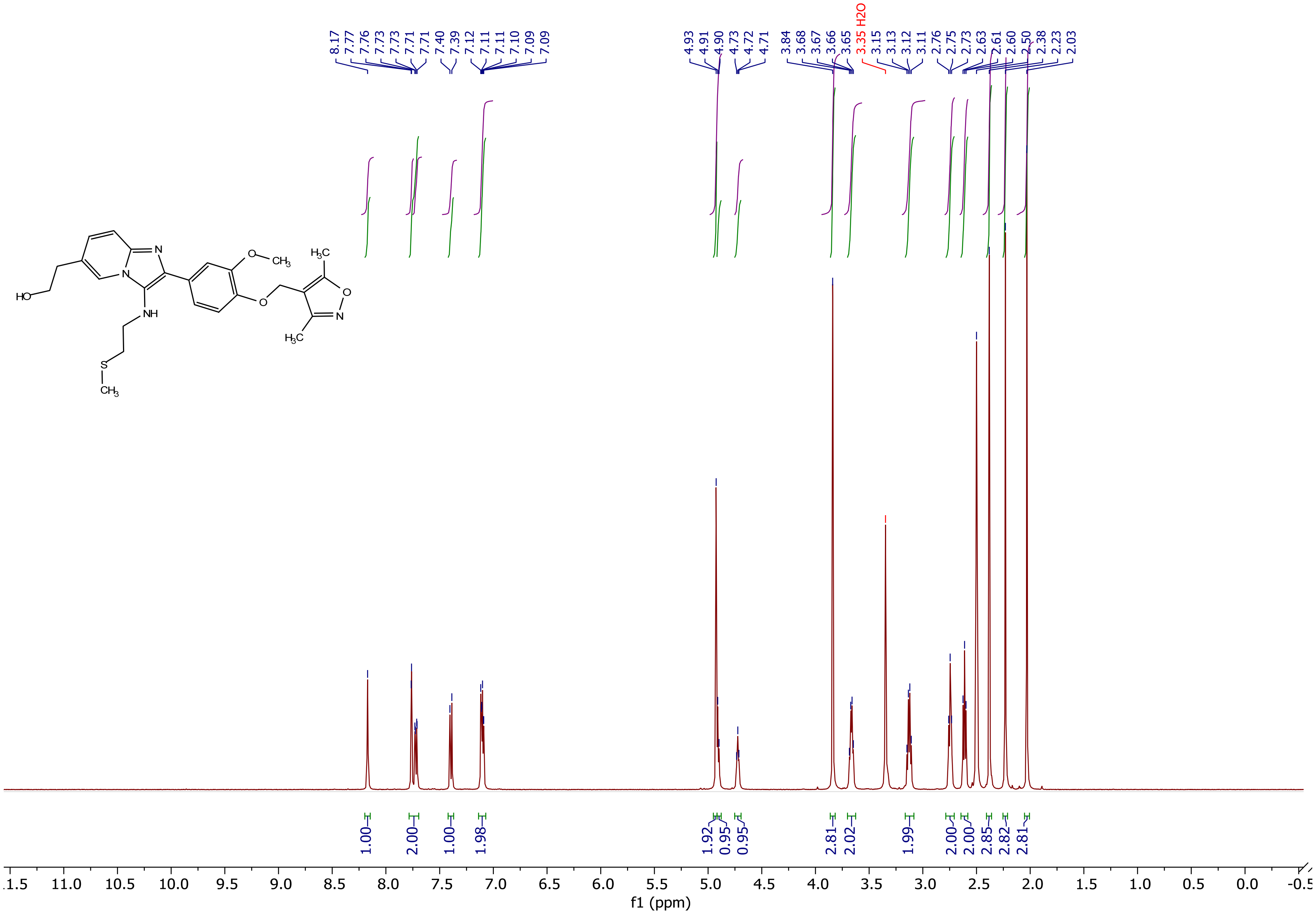

Spectrum 39. 2-(2-{4-[(3,5-Dimethyl-1,2-oxazol-4-yl)methoxy]-3-methoxyphenyl}-3-{[2-(methylsulfanyl)ethyl]amino}imidazo[1,2-*a*]pyridin-6-yl)ethan-1-ol **4**{125,124,31}, ^{1}H NMR (500 MHz, DMSO-d_6)

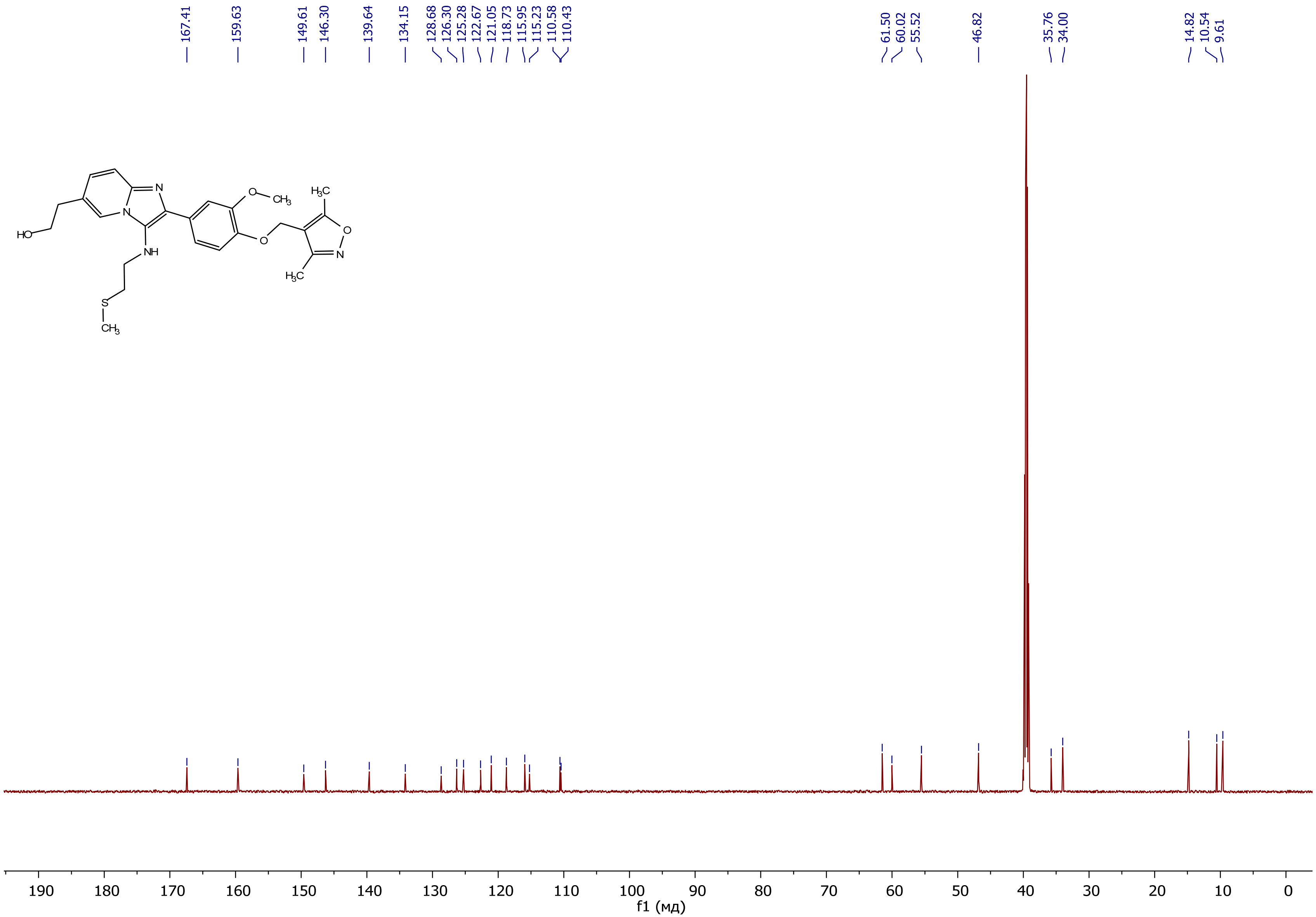

Spectrum 40. 2-(2-{4-[(3,5-Dimethyl-1,2-oxazol-4-yl)methoxy]-3-methoxyphenyl}-3-{[2-(methylsulfanyl)ethyl]amino}imidazo[1,2-*a*]pyridin-6-yl)ethan-1-ol **4**{125,124,31}, $^{13}C\{^{1}H\}$ NMR (151 MHz, DMSO-d_6)

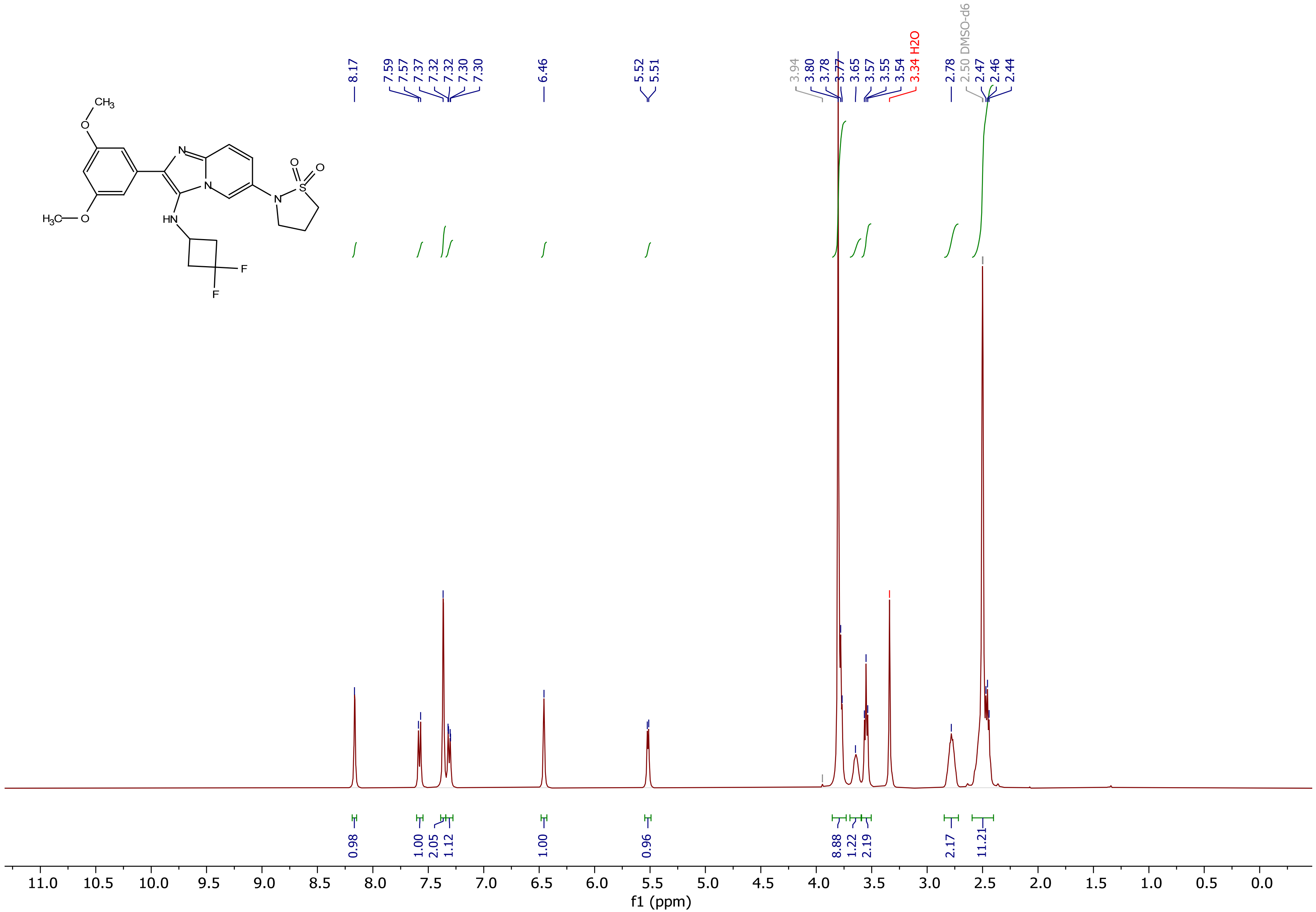

Spectrum 41. 2-{3-[(3,3-Difluorocyclobutyl)amino]-2-(3,5-dimethoxyphenyl)imidazo[1,2-*a*]pyridin-6-yl}-1λ^6,2-thiazolidine-1,1-dione **4**{64,611,49}, ^{1}H NMR (500 MHz, DMSO-d_6)

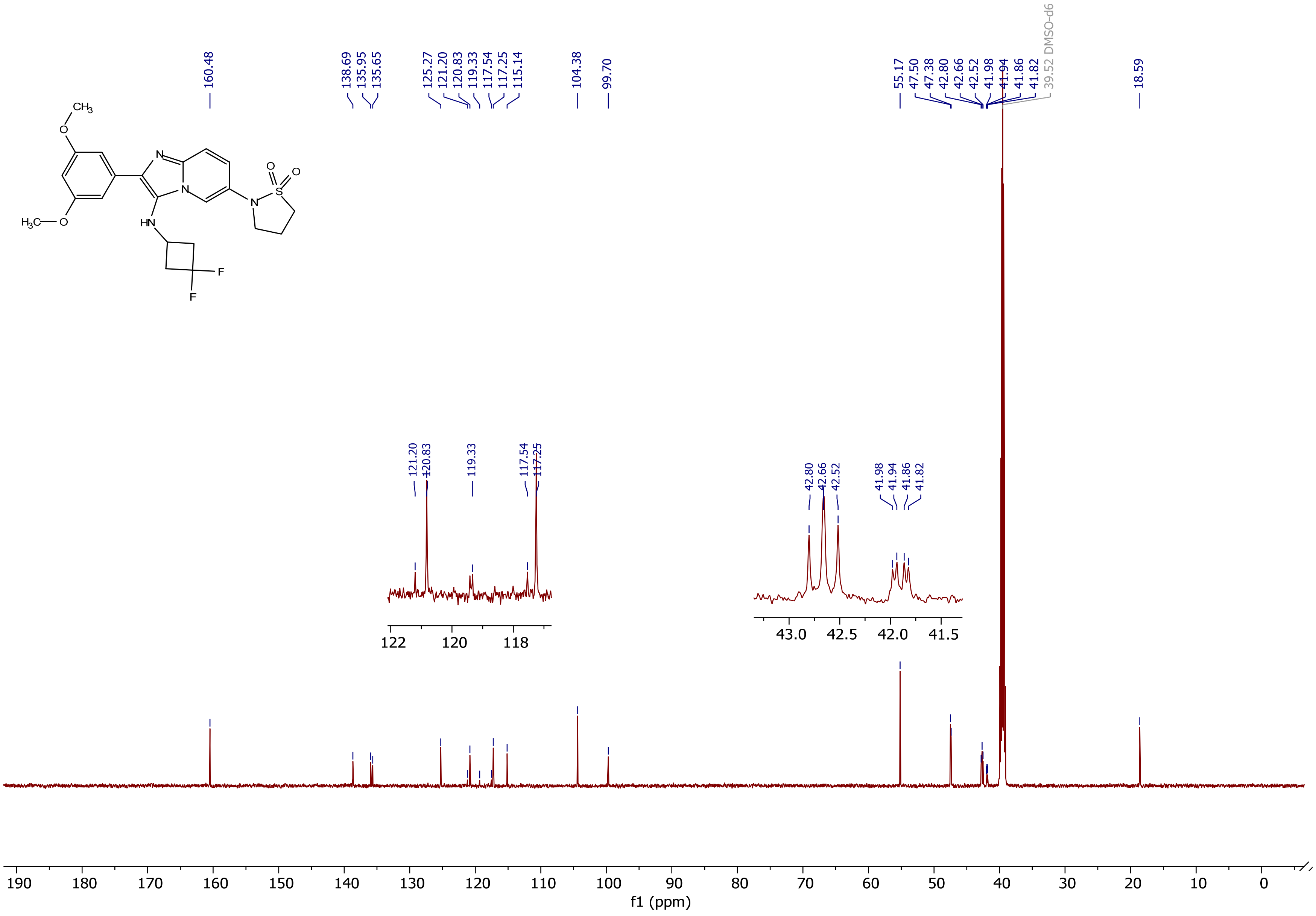

Spectrum 42. 2-{3-[(3,3-Difluorocyclobutyl)amino]-2-(3,5-dimethoxyphenyl)imidazo[1,2-*a*]pyridin-6-yl}-1λ⁶,2-thiazolidine-1,1-dione **4**{64,611,49}, $^{13}C\{^{1}H\}$ NMR (151 MHz, DMSO-d_6)

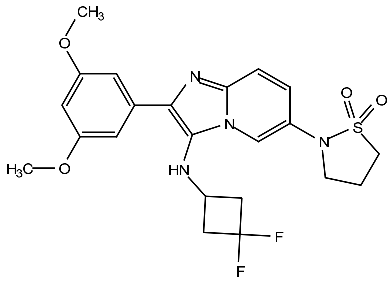

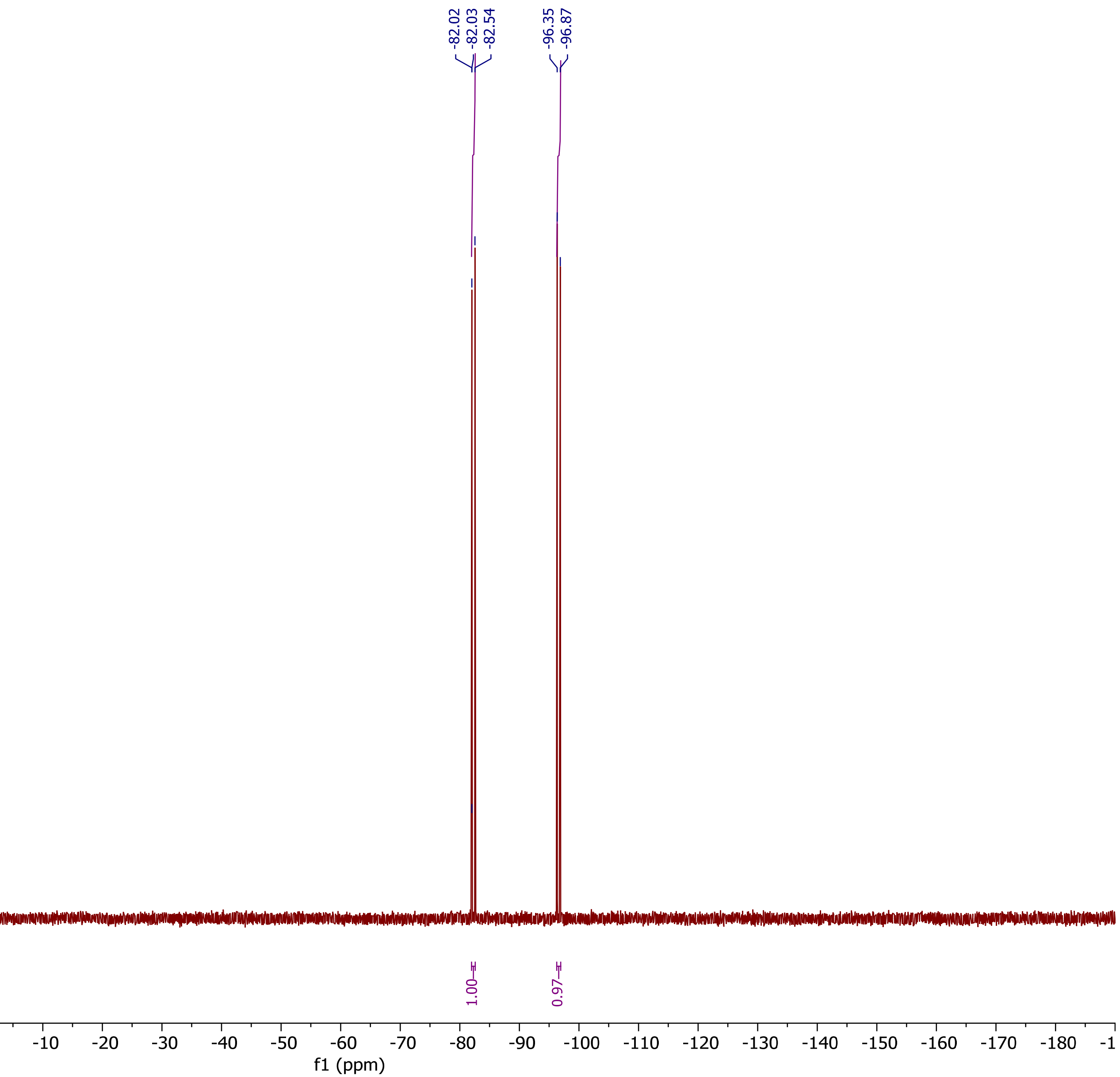

Spectrum 43. 2-{3-[(3,3-Difluorocyclobutyl)amino]-2-(3,5-dimethoxyphenyl)imidazo[1,2-*a*]pyridin-6-yl}-1λ^6,2-thiazolidine-1,1-dione **4**{64,611,49}, $^{19}F\{^1H\}$ NMR (376 MHz, DMSO-d_6)

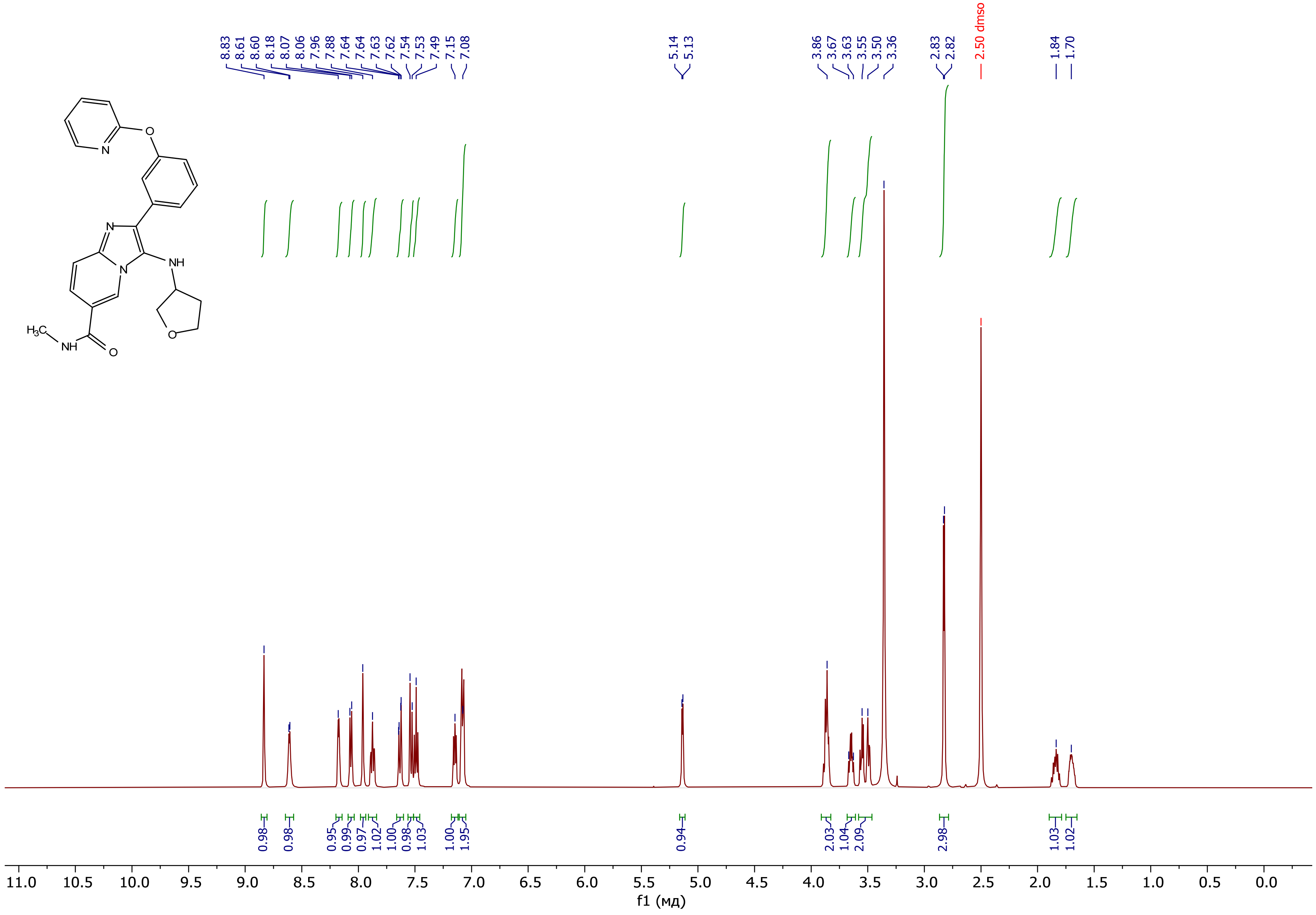

Spectrum 44. N-Methyl-3-[(oxolan-3-yl)amino]-2-[3-(pyridin-2-yloxy)phenyl]imidazo[1,2-*a*]pyridine-6-carboxamide **4**{193,290,10}, ^{1}H NMR (500 MHz, DMSO-d_6)

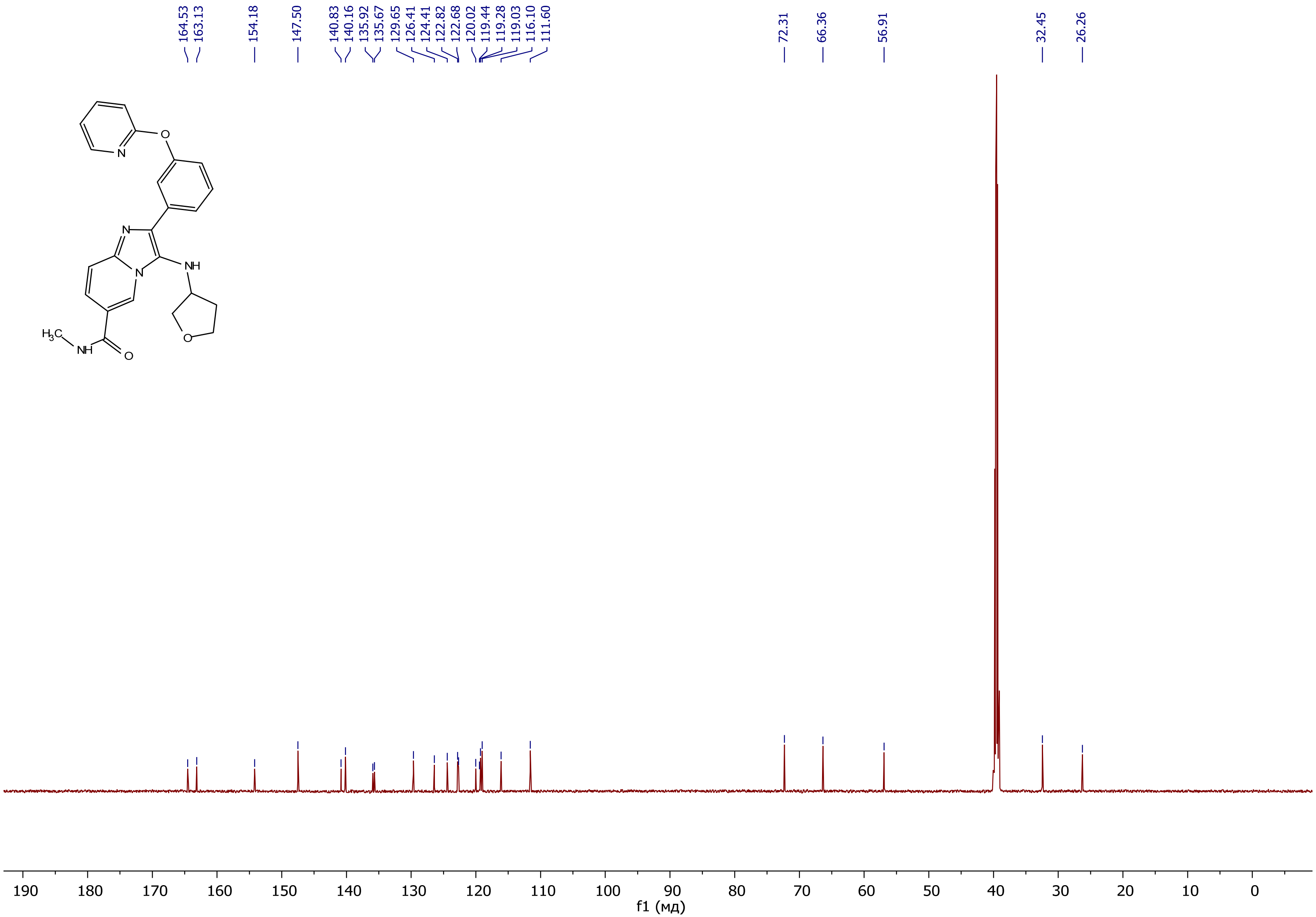

Spectrum 45. N-Methyl-3-[(oxolan-3-yl)amino]-2-[3-(pyridin-2-yloxy)phenyl]imidazo[1,2-*a*]pyridine-6-carboxamide **4**{193,290,10}, $^{13}C\{^{1}H\}$ NMR (151 MHz, DMSO-d_6)

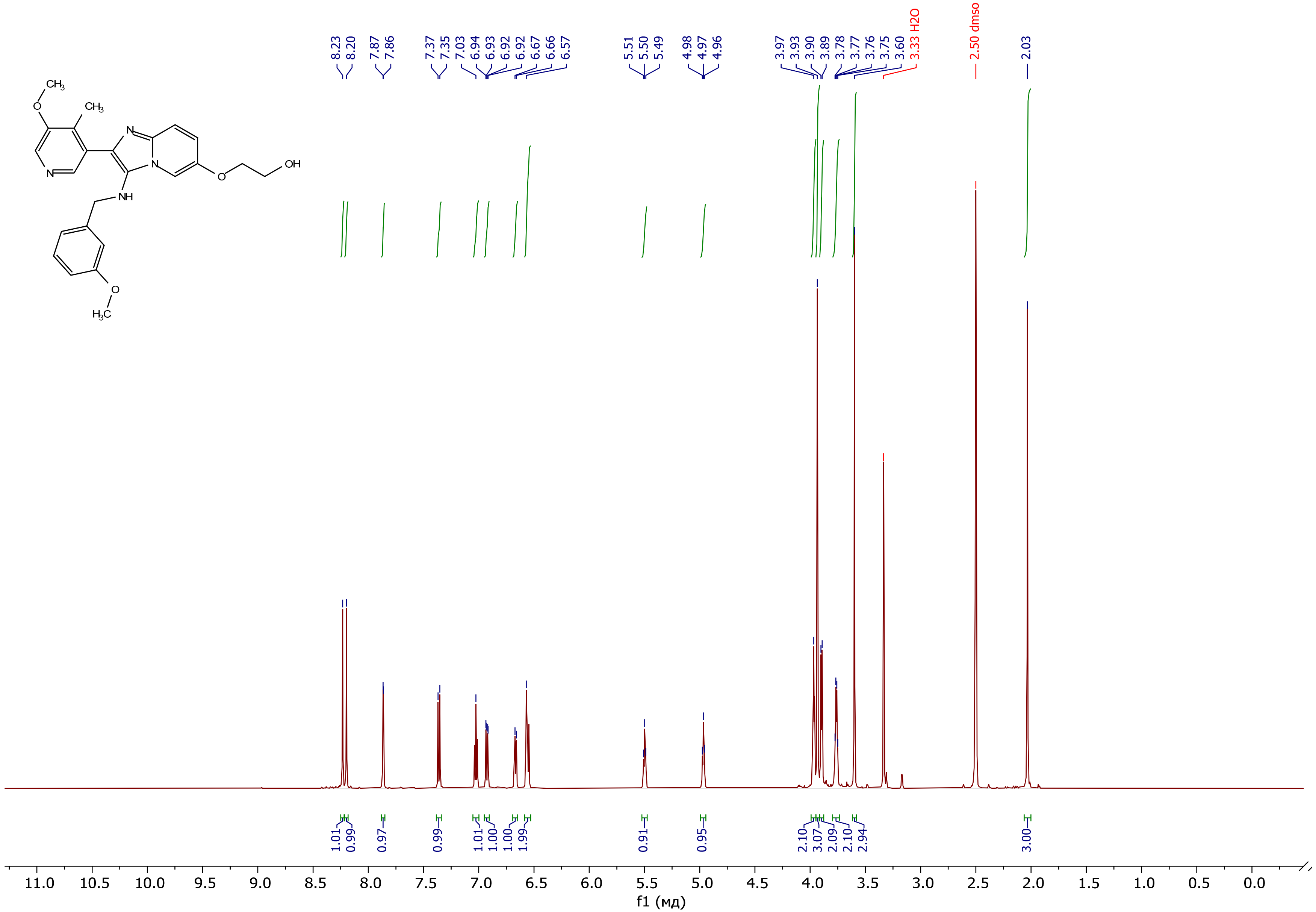

Spectrum 46. 2-{[2-(5-Methoxy-4-methylpyridin-3-yl)-3-{[(3-methoxyphenyl)methyl]amino}imidazo[1,2-*a*]pyridin-6-yl]oxy}ethan-1-ol **4**{107,291,24}, ^{1}H NMR (600 MHz, DMSO-d_6)

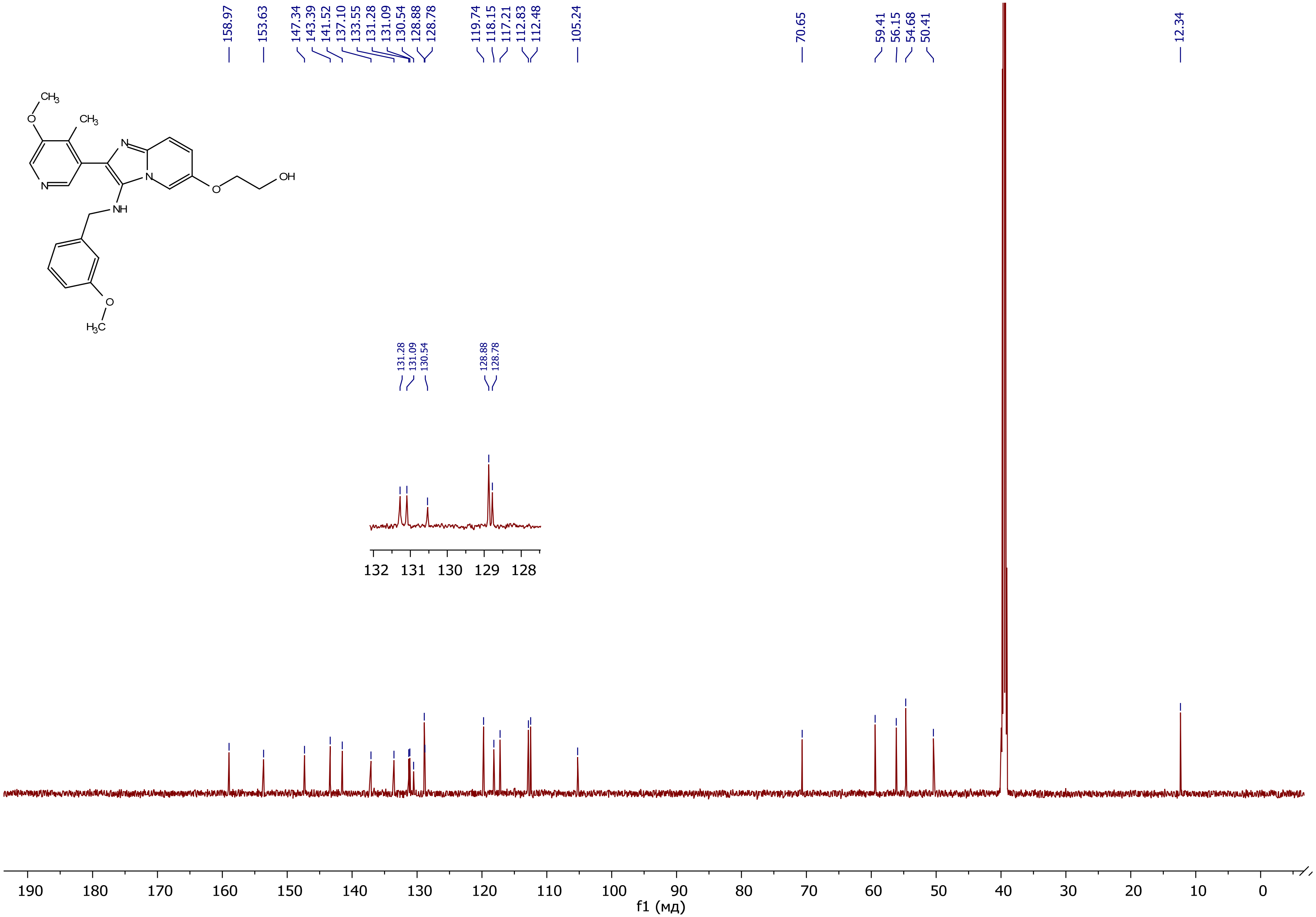

Spectrum 47. 2-{[2-(5-Methoxy-4-methylpyridin-3-yl)-3-{[(3-methoxyphenyl)methyl]amino}imidazo[1,2-*a*]pyridin-6-yl]oxy}ethan-1-ol **4**{107,291,24}, $^{13}C\{^{1}H\}$ NMR (151 MHz, DMSO-d_6)

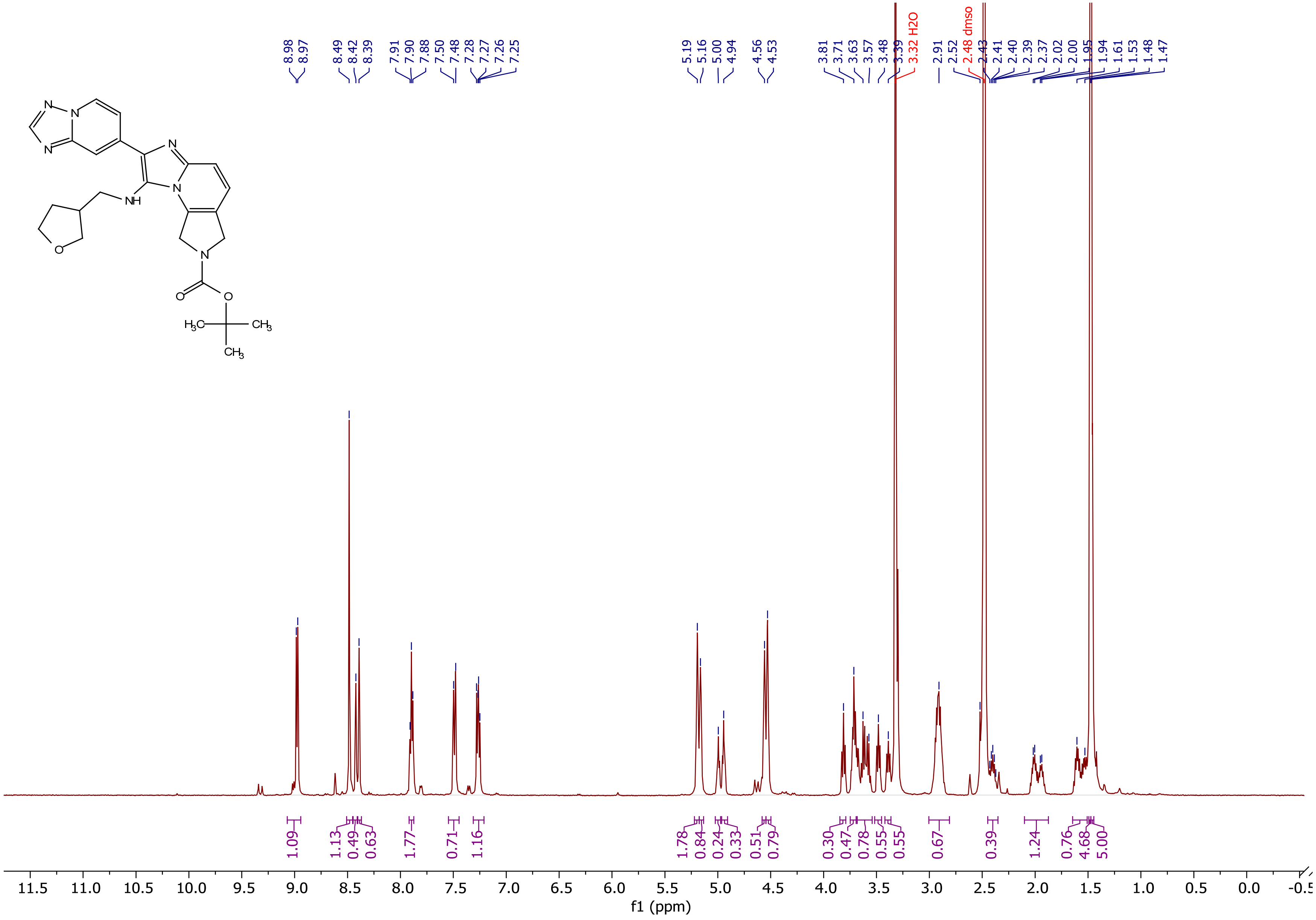

Spectrum 48. *tert*-Butyl 12-{[(oxolan-3-yl)methyl]amino}-11-{[1,2,4]triazolo[1,5-*a*]pyridin-7-yl}-1,4,10-triazatricyclo[7.3.0.0²,⁶]dodeca-2(6),7,9,11-tetraene-4-carboxylate **4**{43,21,12}, ¹H NMR (500 MHz, DMSO-d_6)

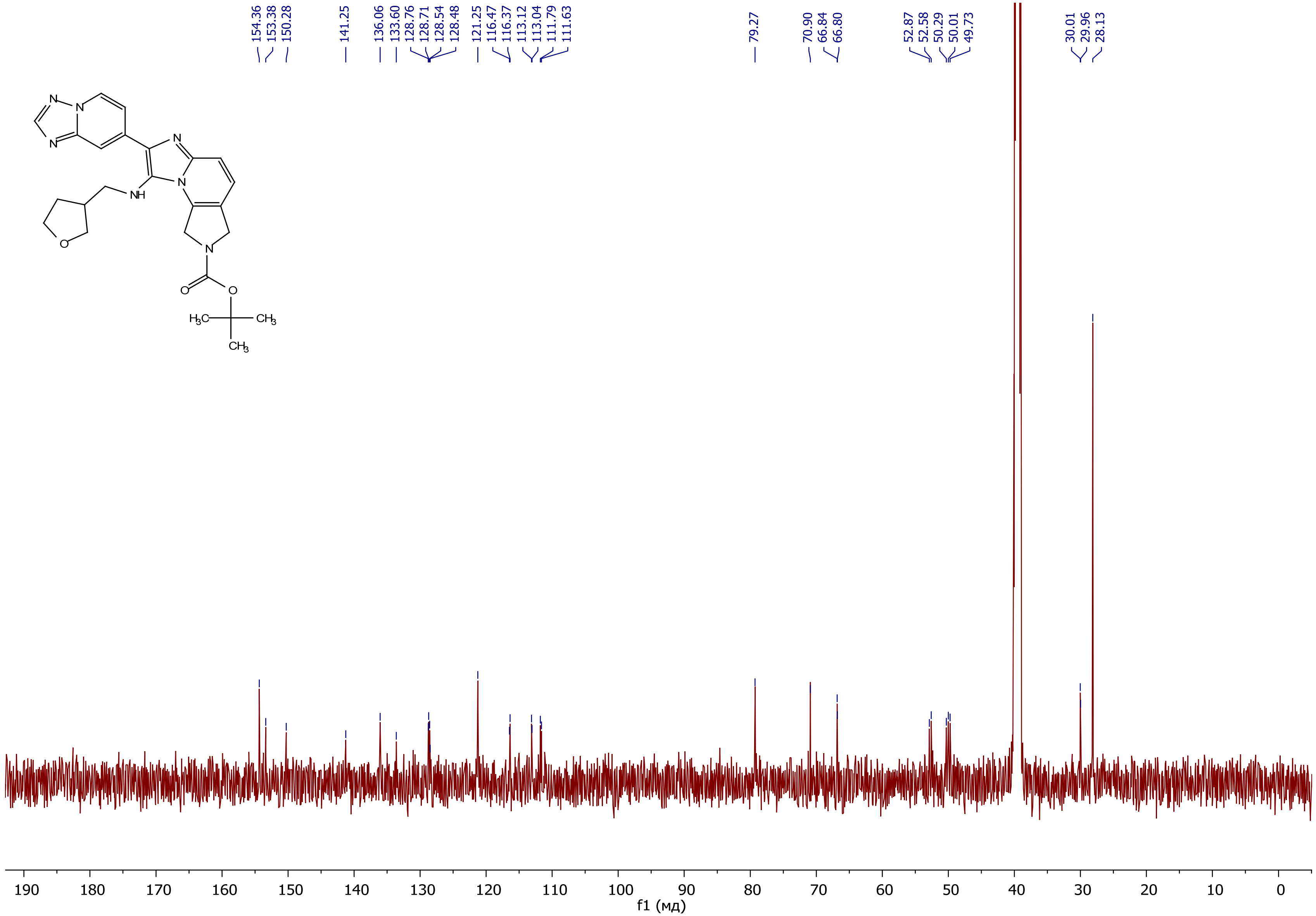

Spectrum 49. *tert*-Butyl 12-{[(oxolan-3-yl)methyl]amino}-11-{[1,2,4]triazolo[1,5-*a*]pyridin-7-yl}-1,4,10-triazatricyclo[7.3.0.0²,⁶]dodeca-2(6),7,9,11-tetraene-4-carboxylate **4**{43,21,12}, $^{13}C\{^1H\}$ NMR (151 MHz, DMSO-d_6)

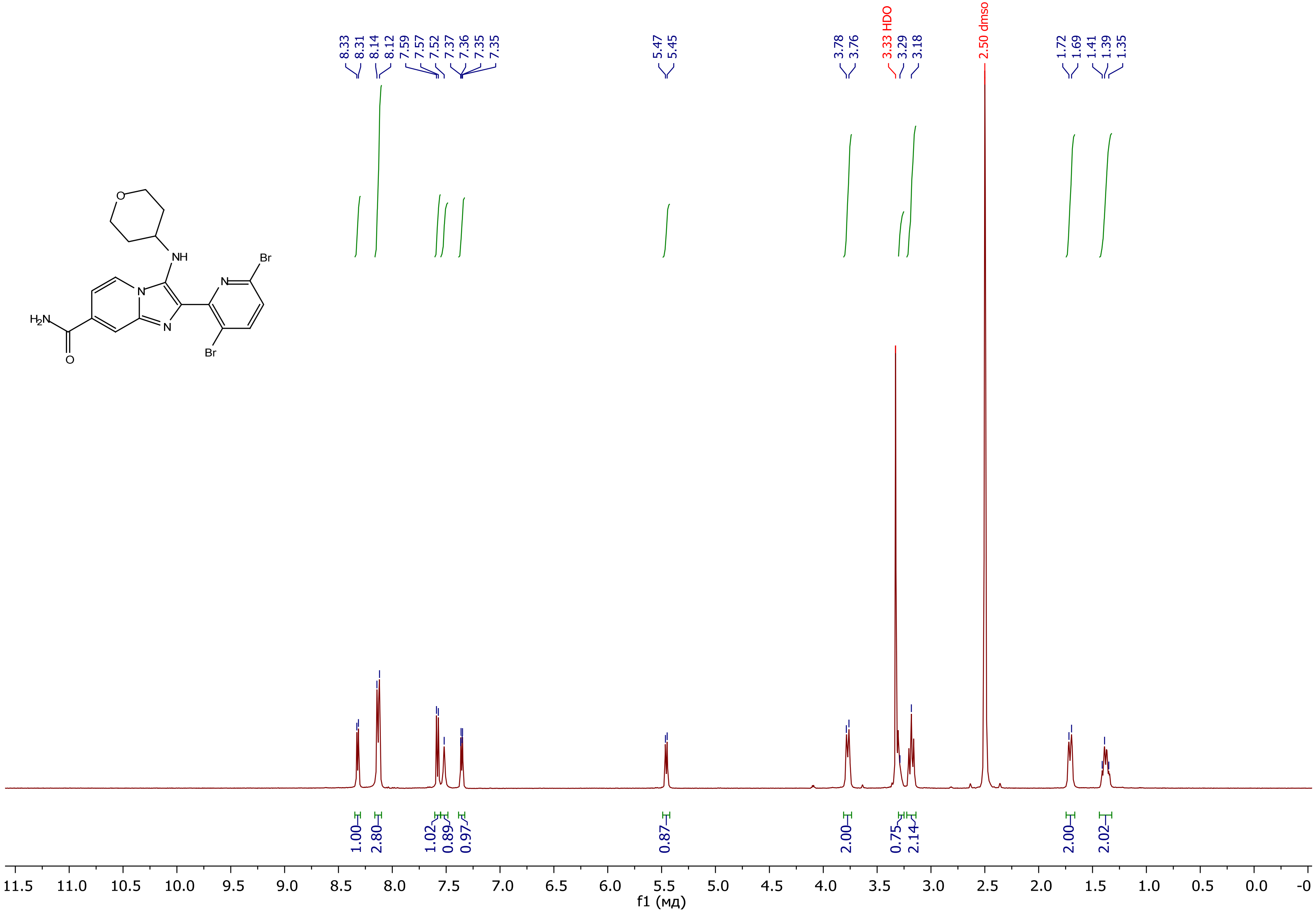

Spectrum 50. 2-(3,6-Dibromopyridin-2-yl)-3-[(oxan-4-yl)amino]imidazo[1,2-*a*]pyridine-7-carboxamide **4**{92,361,7}, ^{1}H NMR (500 MHz, DMSO-d_6)

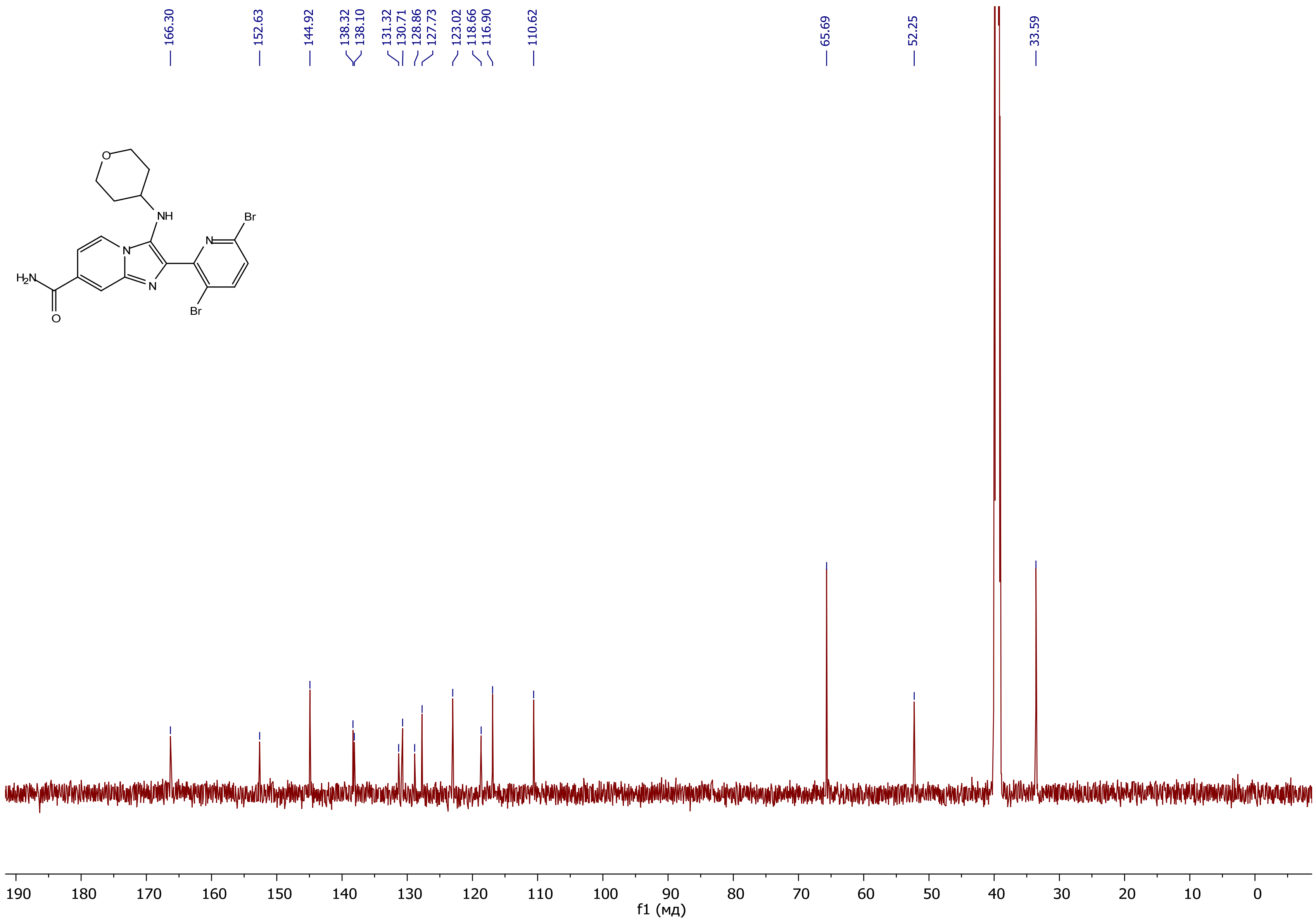

Spectrum 51. 2-(3,6-Dibromopyridin-2-yl)-3-[(oxan-4-yl)amino]imidazo[1,2-*a*]pyridine-7-carboxamide **4**{92,361,7}, $^{13}C\{^1H\}$ NMR (151 MHz, DMSO-d_6)

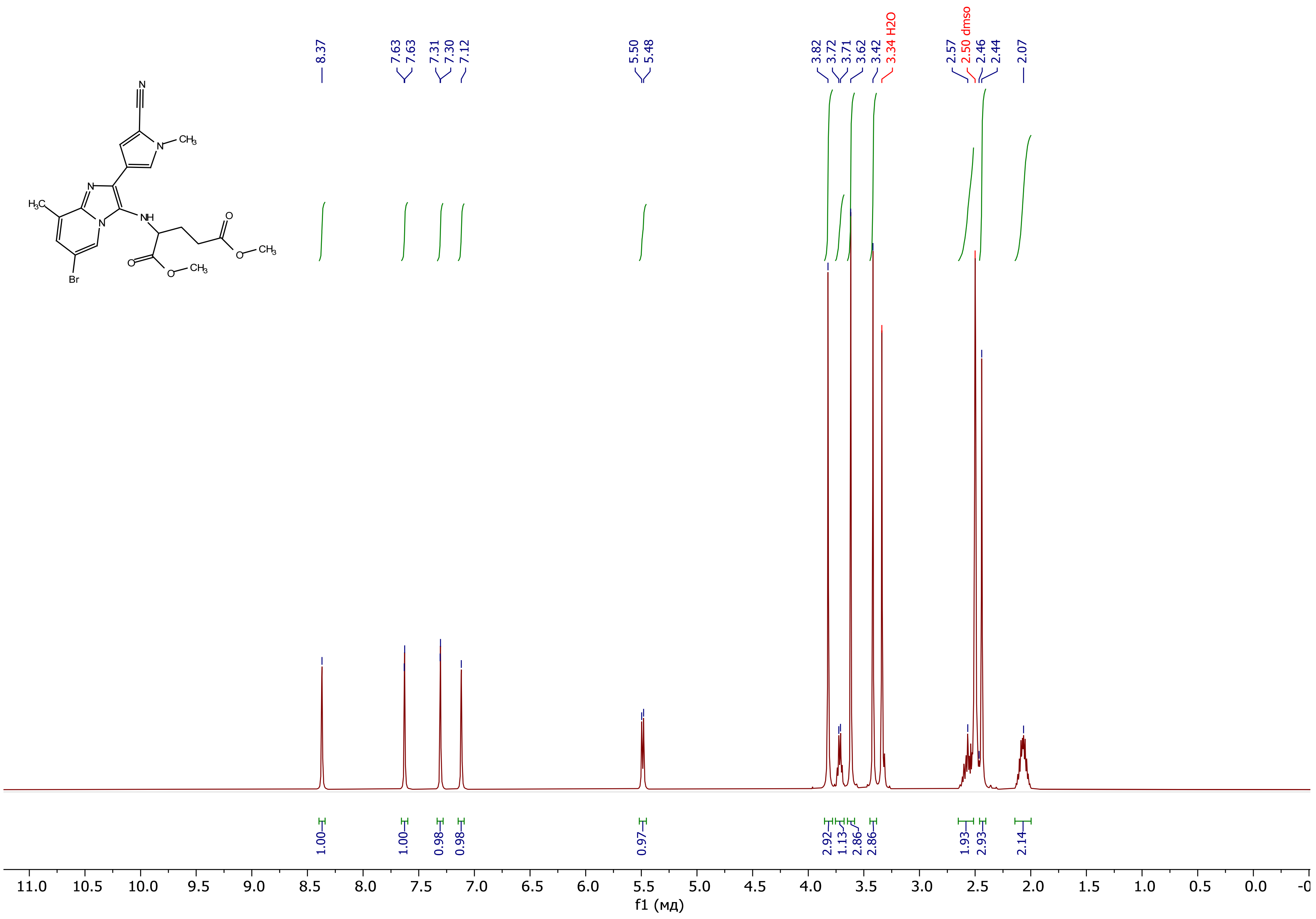

Spectrum 52. 1,5-Dimethyl 2-{[6-bromo-2-(5-cyano-1-methyl-1*H*-pyrrol-3-yl)-8-methylimidazo[1,2-*a*]pyridin-3-yl]amino}pentanedioate **4**{435,219,23}, ^{1}H NMR (500 MHz, DMSO-d_6)

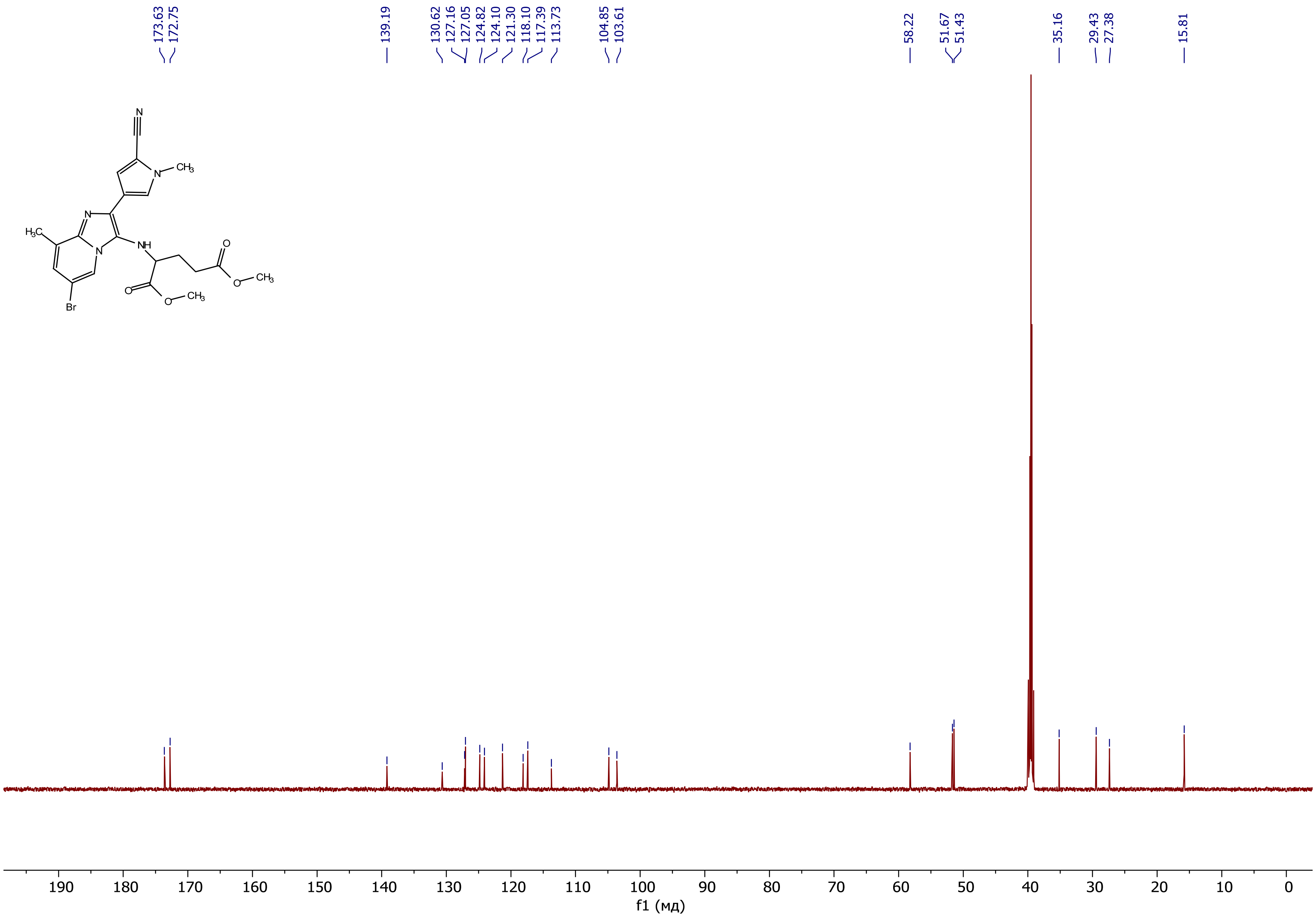

Spectrum 53. 1,5-Dimethyl 2-{[6-bromo-2-(5-cyano-1-methyl-1*H*-pyrrol-3-yl)-8-methylimidazo[1,2-*a*]pyridin-3-yl]amino}pentanedioate **4**{435,219,23}, $^{13}C\{^{1}H\}$ NMR (151 MHz, DMSO-d_6)

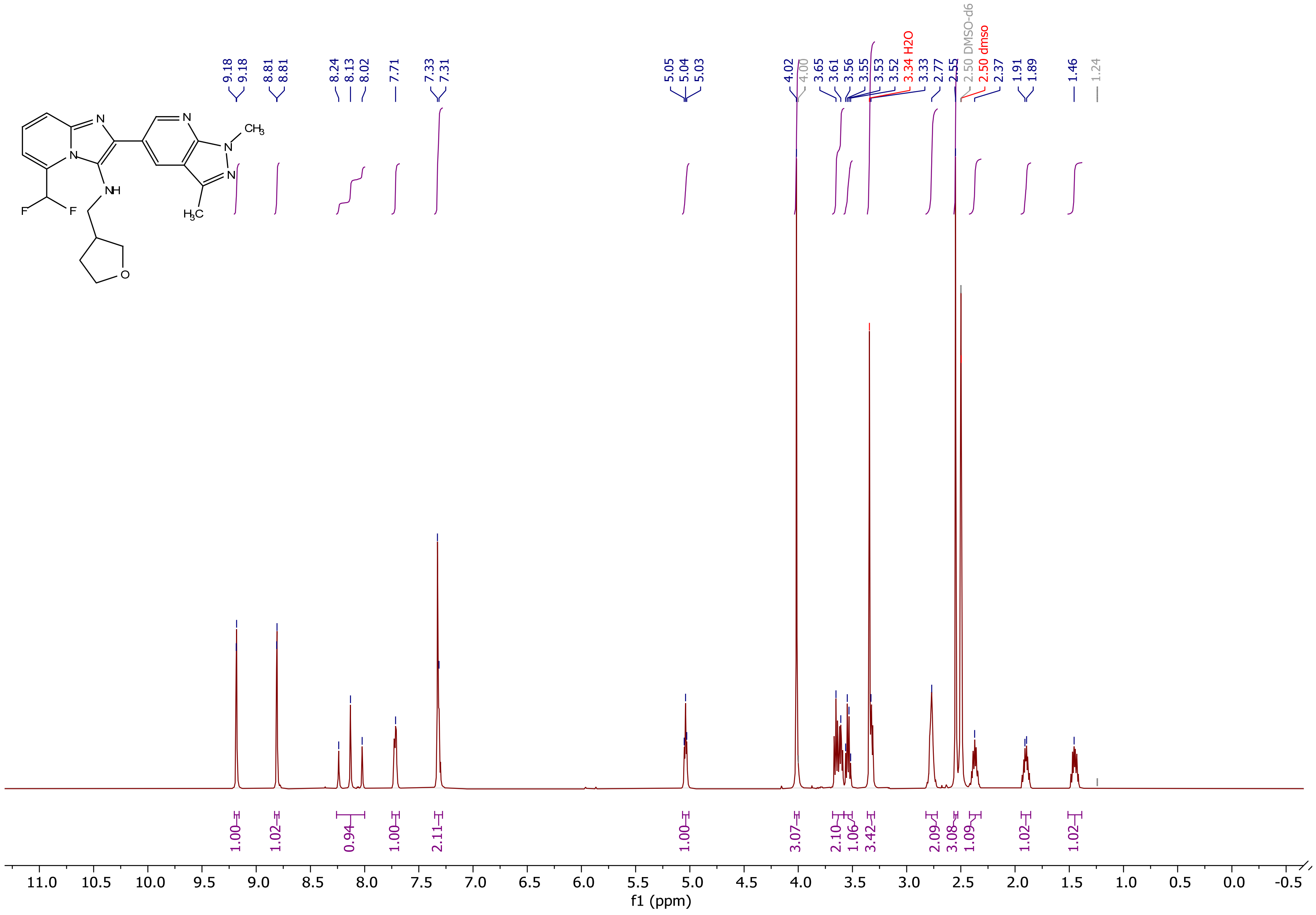

Spectrum 54. 5-(Difluoromethyl)-2-{1,3-dimethyl-1*H*-pyrazolo[3,4-*b*]pyridin-5-yl}-*N*-[(oxolan-3-yl)methyl]imidazo[1,2-*a*]pyridin-3-amine **4**{37,596,12}, ^{1}H NMR (500 MHz, DMSO-d_6)

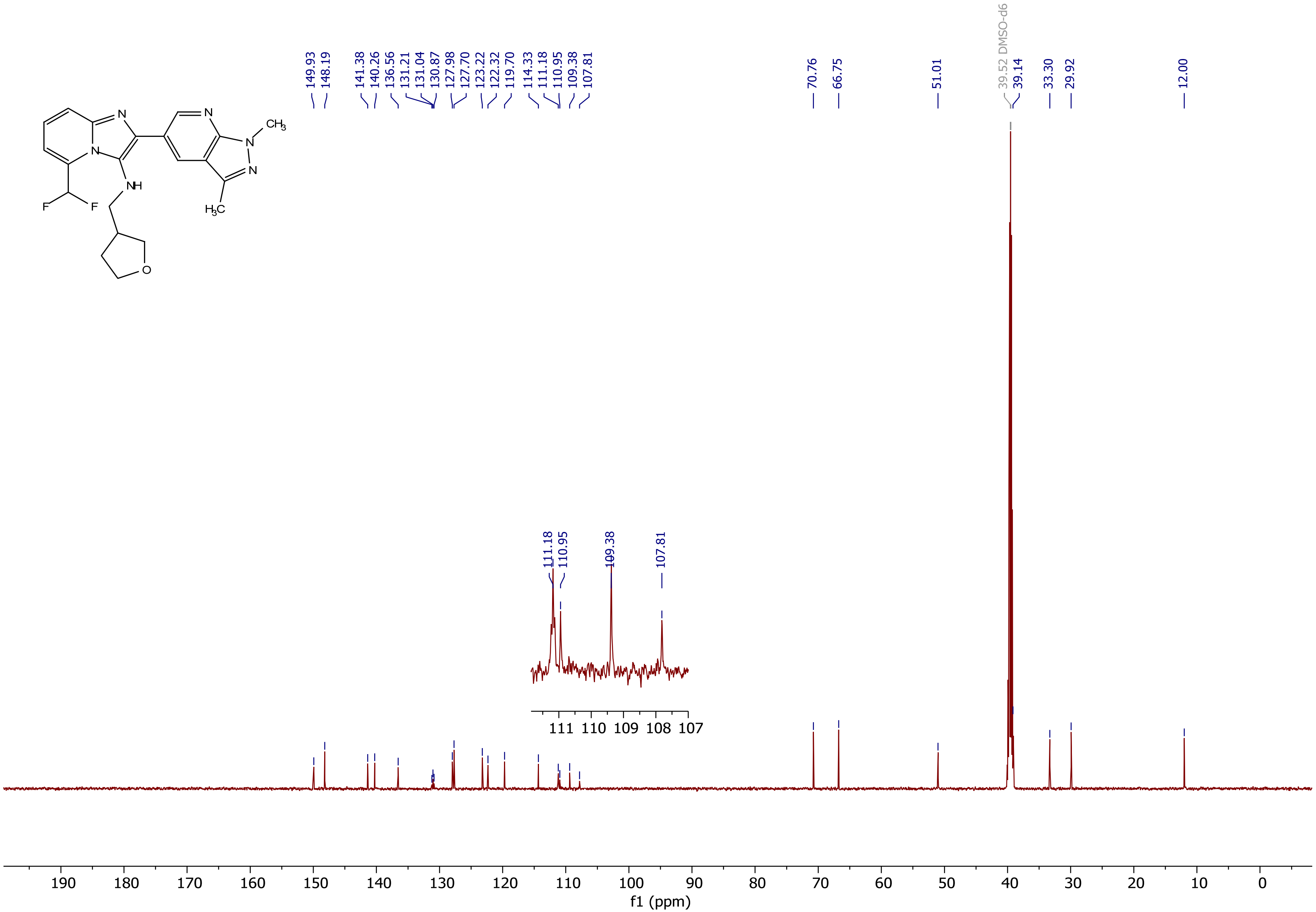

Spectrum 55. 5-(Difluoromethyl)-2-{1,3-dimethyl-1*H*-pyrazolo[3,4-*b*]pyridin-5-yl}-*N*-[(oxolan-3-yl)methyl]imidazo[1,2-*a*]pyridin-3-amine **4**{37,596,12}, $^{13}C\{^{1}H\}$ NMR (151 MHz, DMSO-d_6)

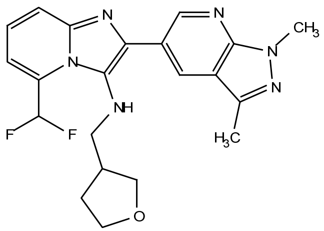

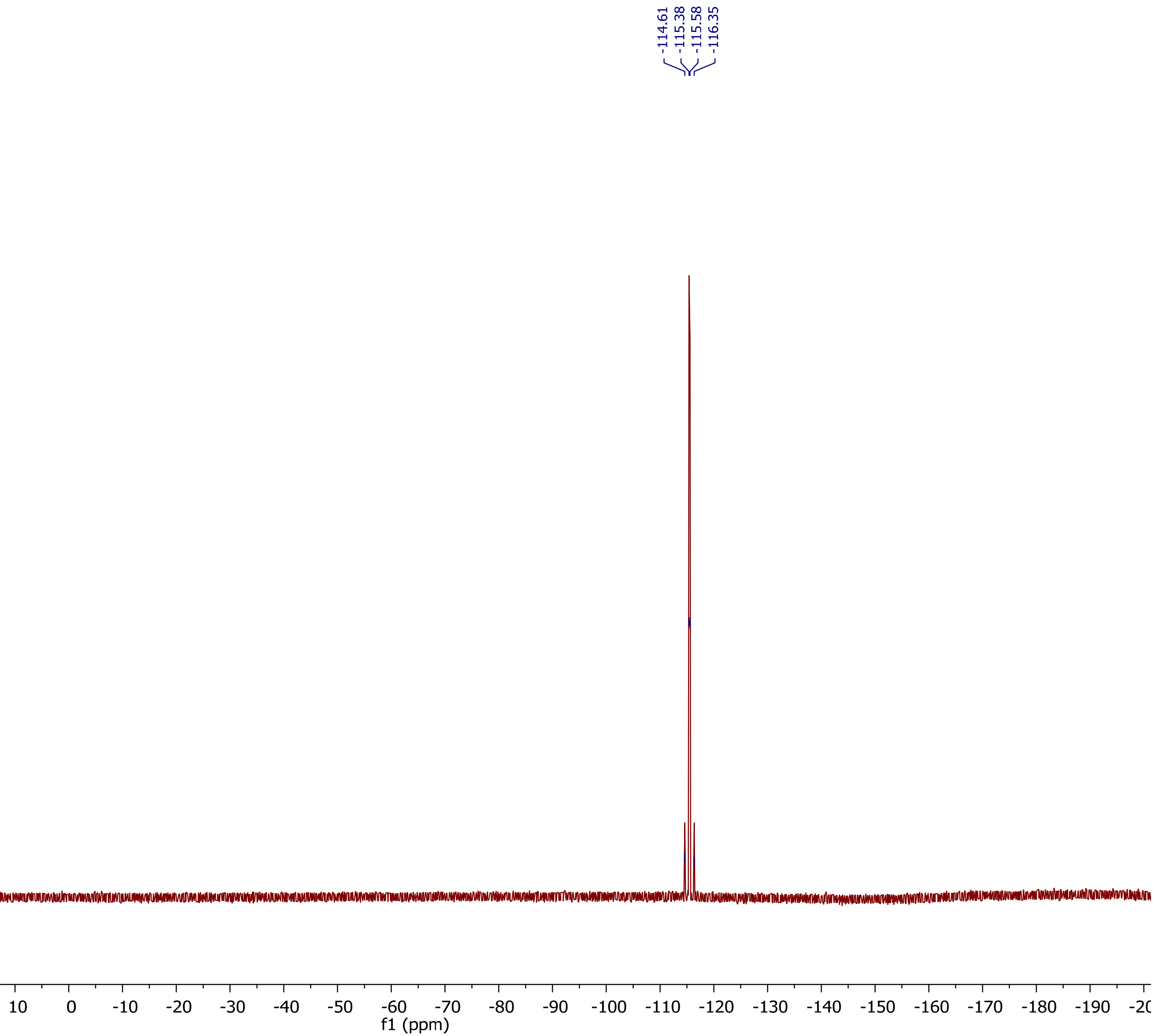

Spectrum 56. 5-(Difluoromethyl)-2-{1,3-dimethyl-1*H*-pyrazolo[3,4-*b*]pyridin-5-yl}-*N*-[(oxolan-3-yl)methyl]imidazo[1,2-*a*]pyridin-3-amine **4**{37,596,12}, $^{19}F\{^{1}H\}$ NMR (376 MHz, DMSO-d_6)

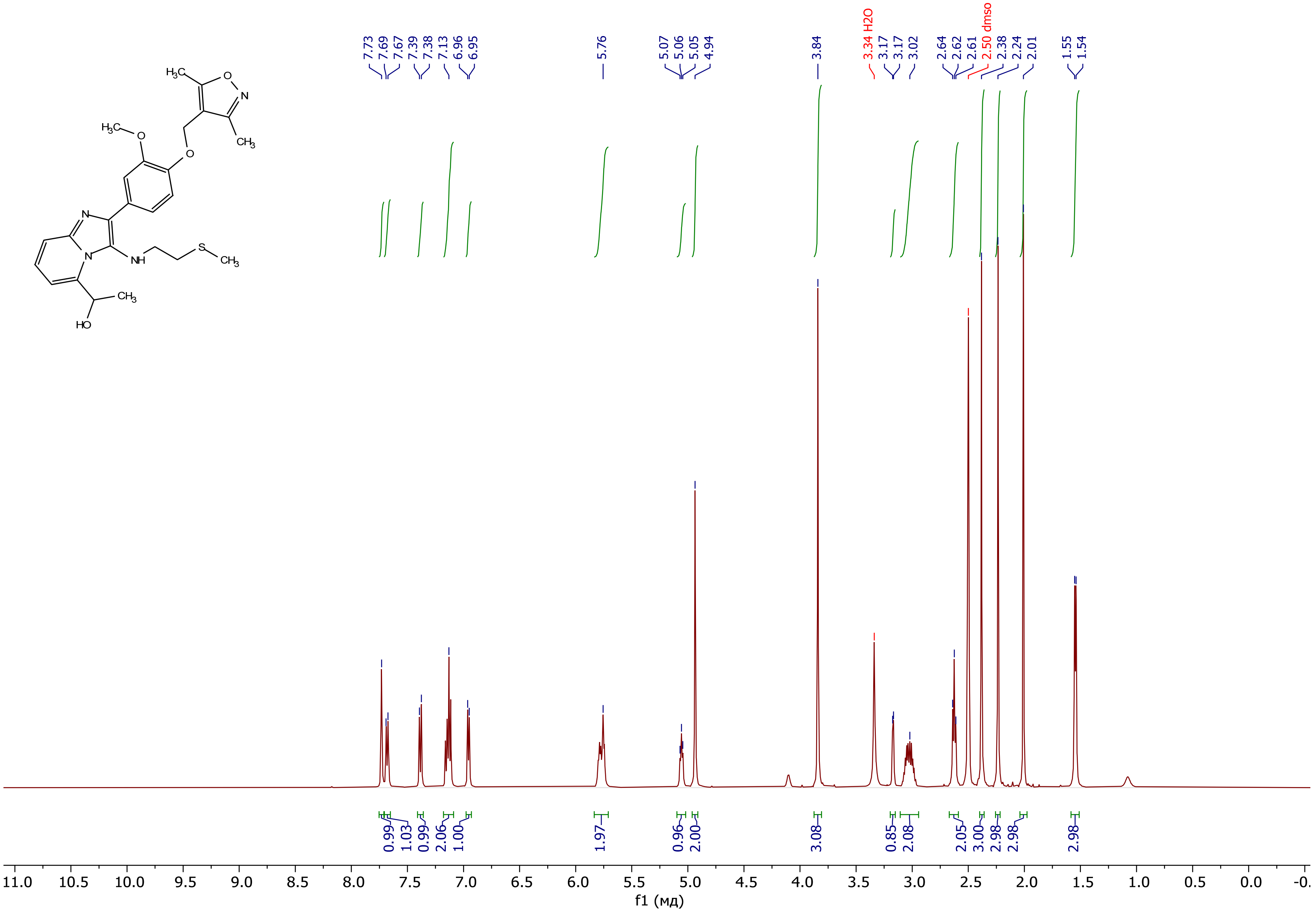

Spectrum 57. 1-(2-{4-[(3,5-Dimethyl-1,2-oxazol-4-yl)methoxy]-3-methoxyphenyl}-3-{[2-(methylsulfanyl)ethyl]amino}imidazo[1,2-*a*]pyridin-5-yl)ethan-1-ol **4**{118,124,31}, ^{1}H NMR (500 MHz, DMSO-d_6)

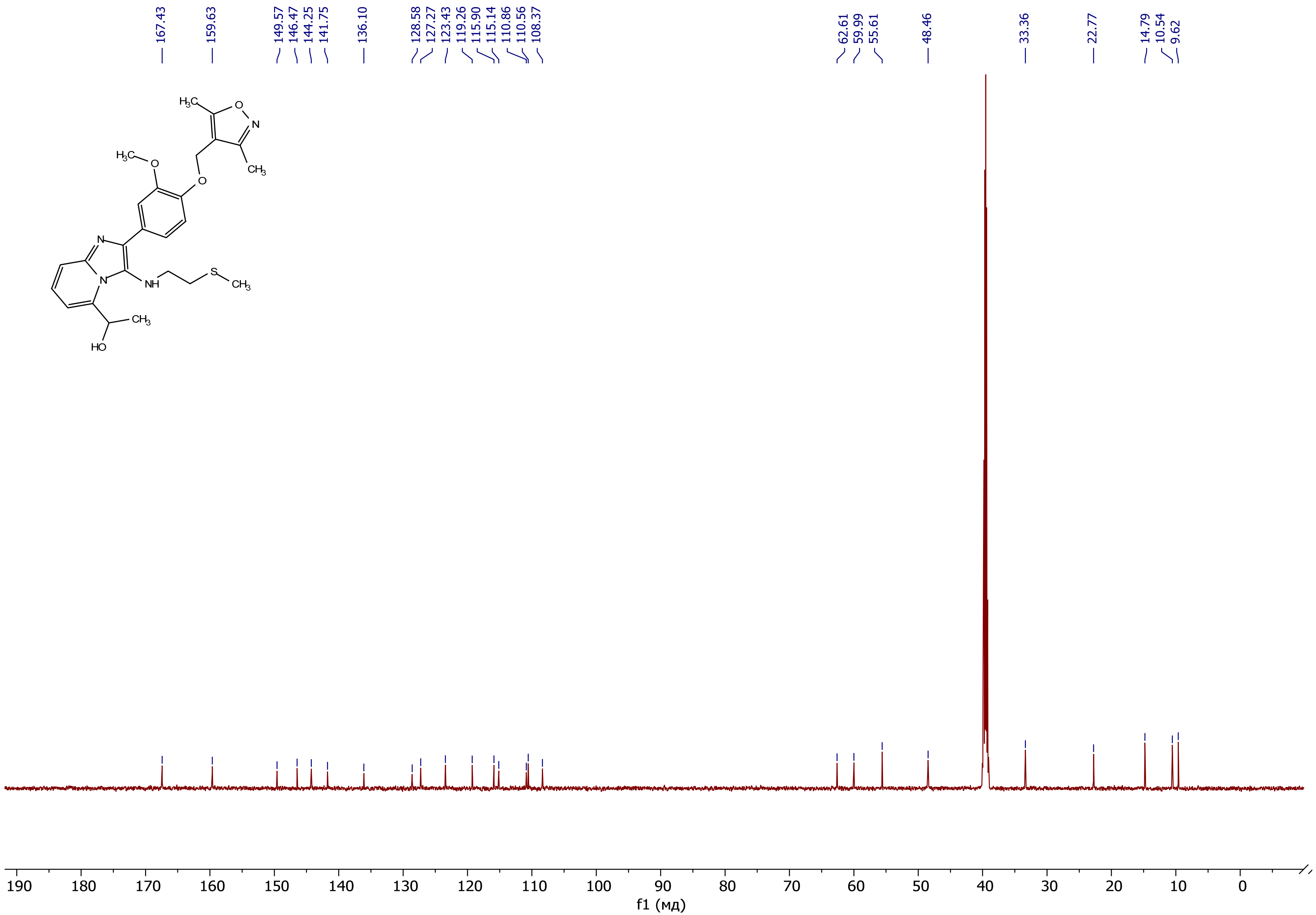

Spectrum 58. 1-(2-{4-[(3,5-Dimethyl-1,2-oxazol-4-yl)methoxy]-3-methoxyphenyl}-3-{[2-(methylsulfanyl)ethyl]amino}imidazo[1,2-*a*]pyridin-5-yl)ethan-1-ol **4**{118,124,31}, $^{13}C\{^{1}H\}$ NMR (151 MHz, DMSO-d_6)

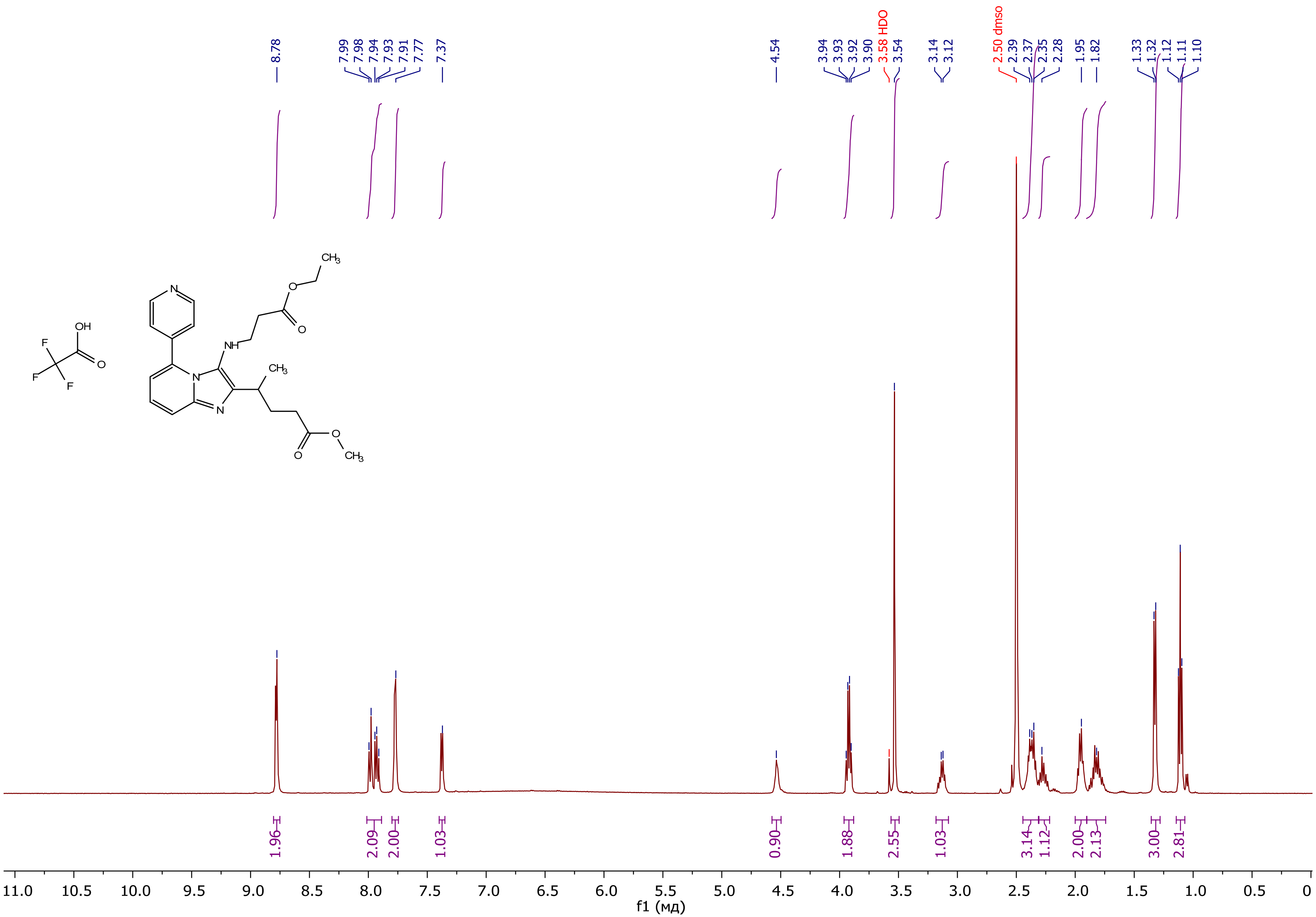

Spectrum 59. methyl 4-{3-[(3-Ethoxy-3-oxopropyl)amino]-5-(pyridin-4-yl)imidazo[1,2-*a*]pyridin-2-yl}pentanoate trifluoroacetate **4**{333,152,29}, ^{1}H NMR (500 MHz, DMSO-d_6)

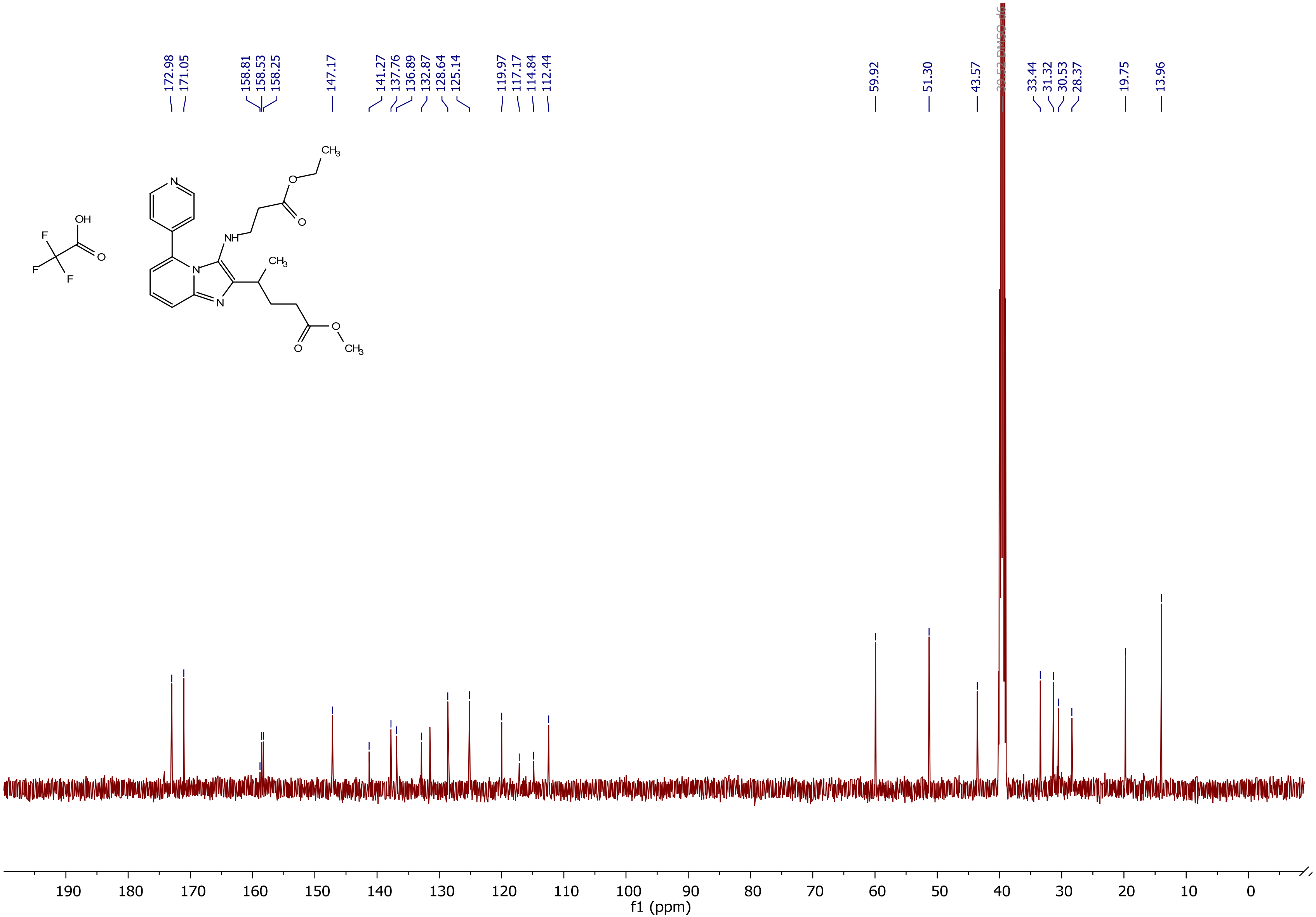

Spectrum 60. Methyl 4-{3-[(3-ethoxy-3-oxopropyl)amino]-5-(pyridin-4-yl)imidazo[1,2-*a*]pyridin-2-yl}pentanoate trifluoroacetate **4**{333,152,29}, $^{13}C\{^1H\}$ NMR (126 MHz, DMSO-d_6)

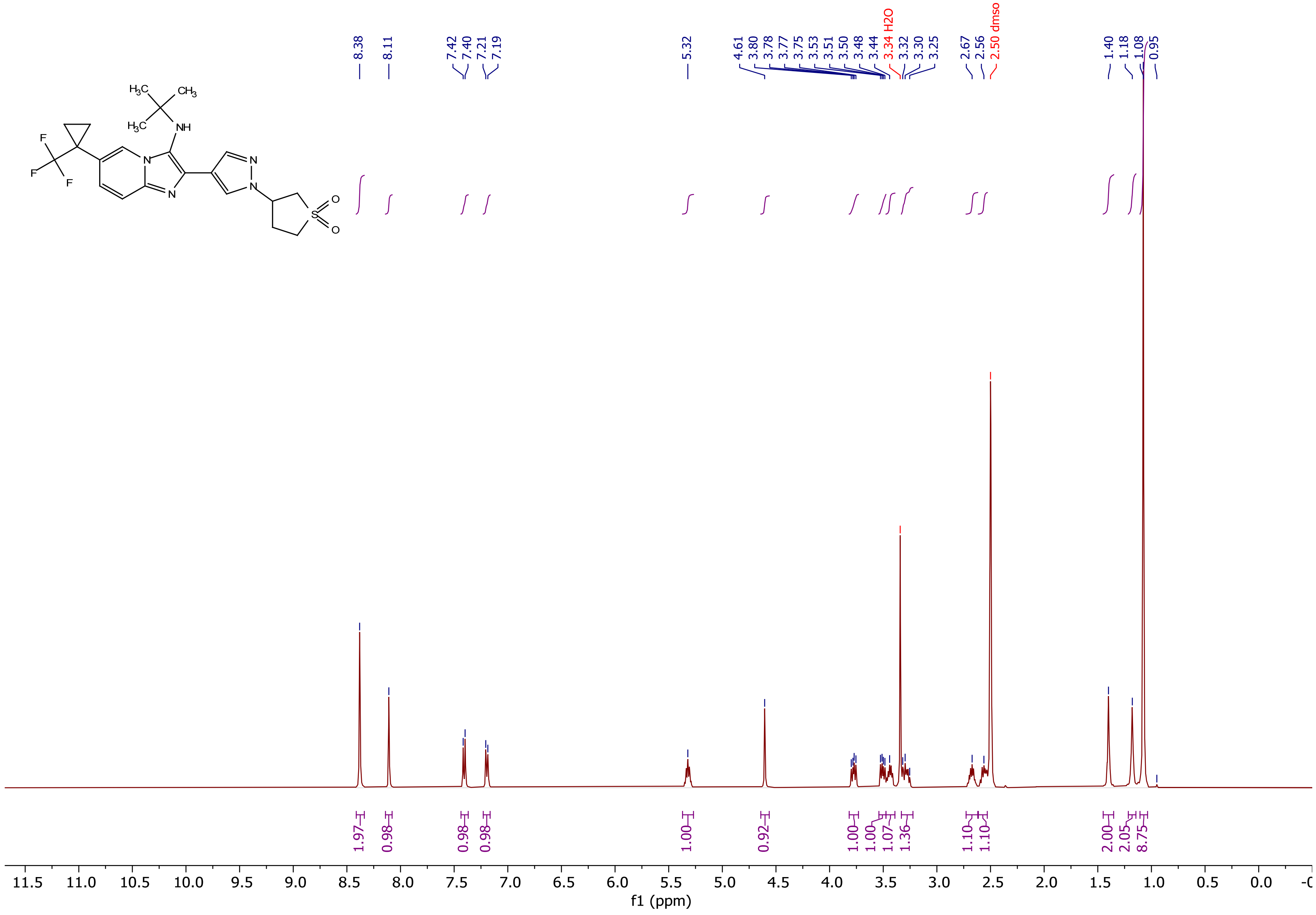

Spectrum 61. 3-{4-[3-(*tert*-Butylamino)-6-[1-(trifluoromethyl)cyclopropyl]imidazo[1,2-*a*]pyridin-2-yl]-1*H*-pyrazol-1-yl}-1λ^6-thiolane-1,1-dione **4**{52,28,18}, ^1H NMR (500 MHz, DMSO-d_6)

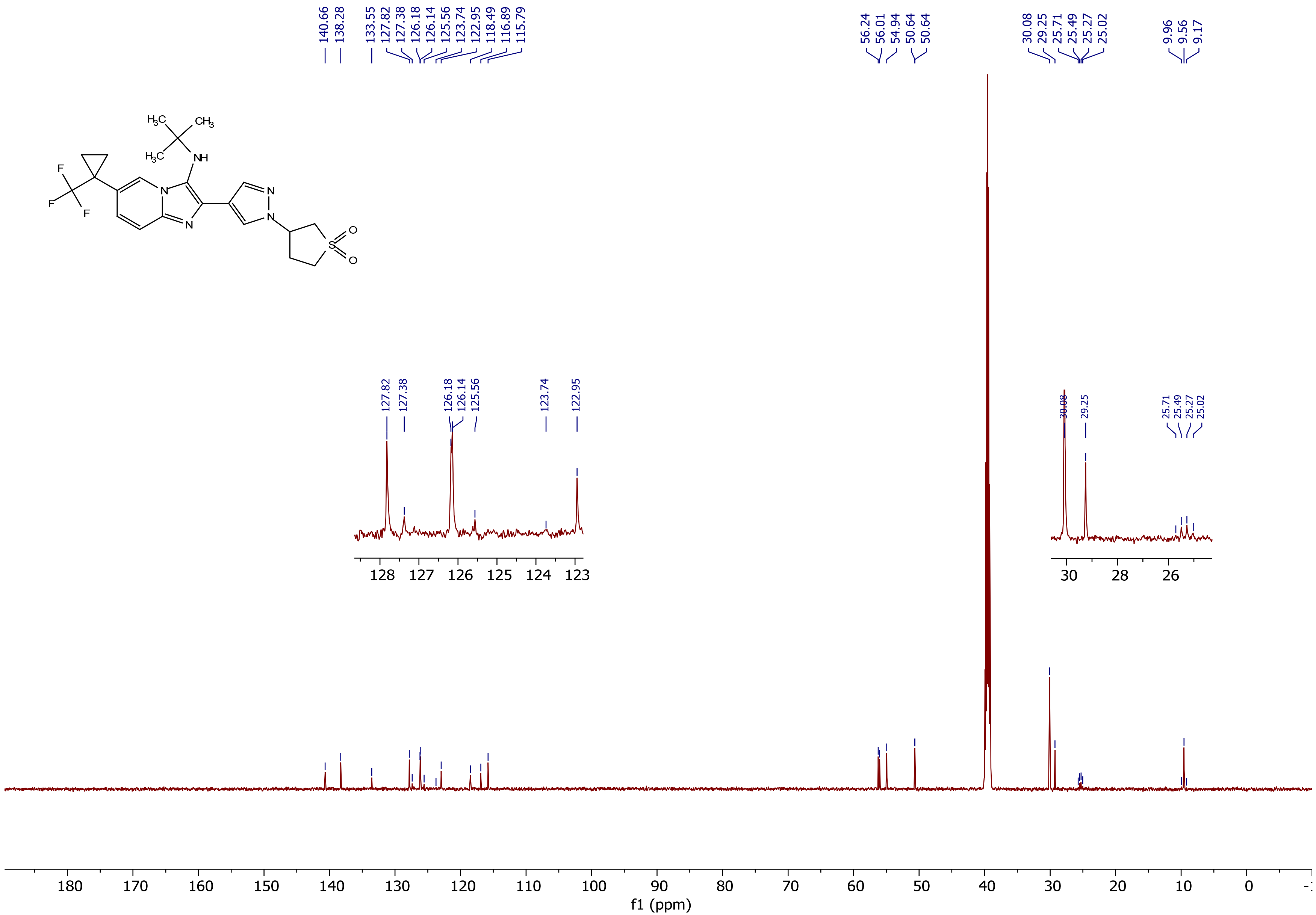

Spectrum 62. 3-{4-[3-(*tert*-Butylamino)-6-[1-(trifluoromethyl)cyclopropyl]imidazo[1,2-*a*]pyridin-2-yl]-1*H*-pyrazol-1-yl}-1λ⁶-thiolane-1,1-dione **4**{52,28,18}, $^{13}C\{^{1}H\}$ NMR (151 MHz, DMSO-d_6)

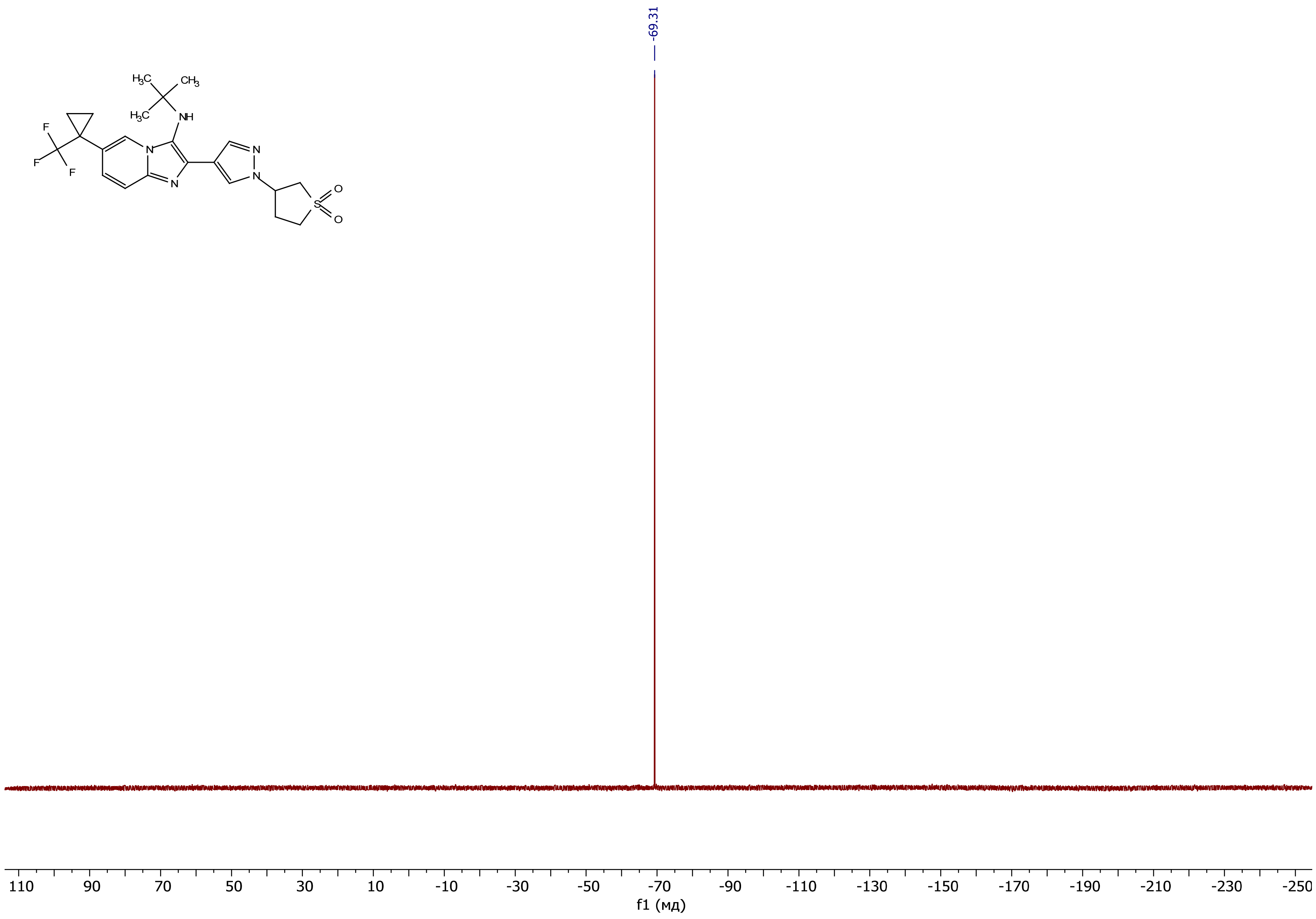

Spectrum 63. 3-{4-[3-(*tert*-Butylamino)-6-[1-(trifluoromethyl)cyclopropyl]imidazo[1,2-*a*]pyridin-2-yl]-1*H*-pyrazol-1-yl}-1λ^6-thiolane-1,1-dione **4**{52,28,18}, $^{19}F\{^1H\}$ NMR (376 MHz, DMSO-d_6)

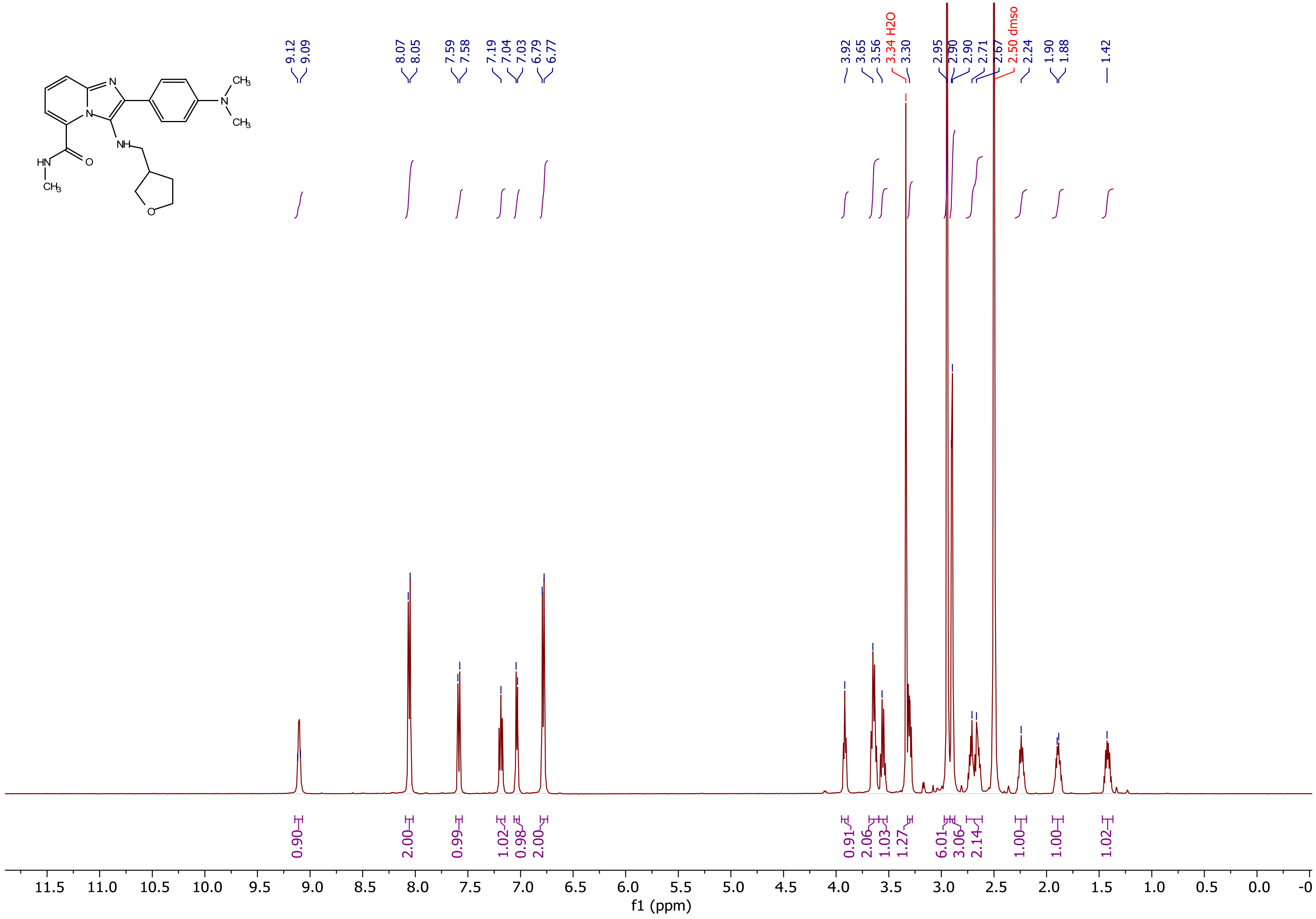

Spectrum 64. 2-[4-(Dimethylamino)phenyl]-*N*-methyl-3-{[(oxolan-3-yl)methyl]amino}imidazo[1,2-*a*]pyridine-5-carboxamide **4**{362,594,12}, ^{1}H NMR (500 MHz, DMSO-d_6)

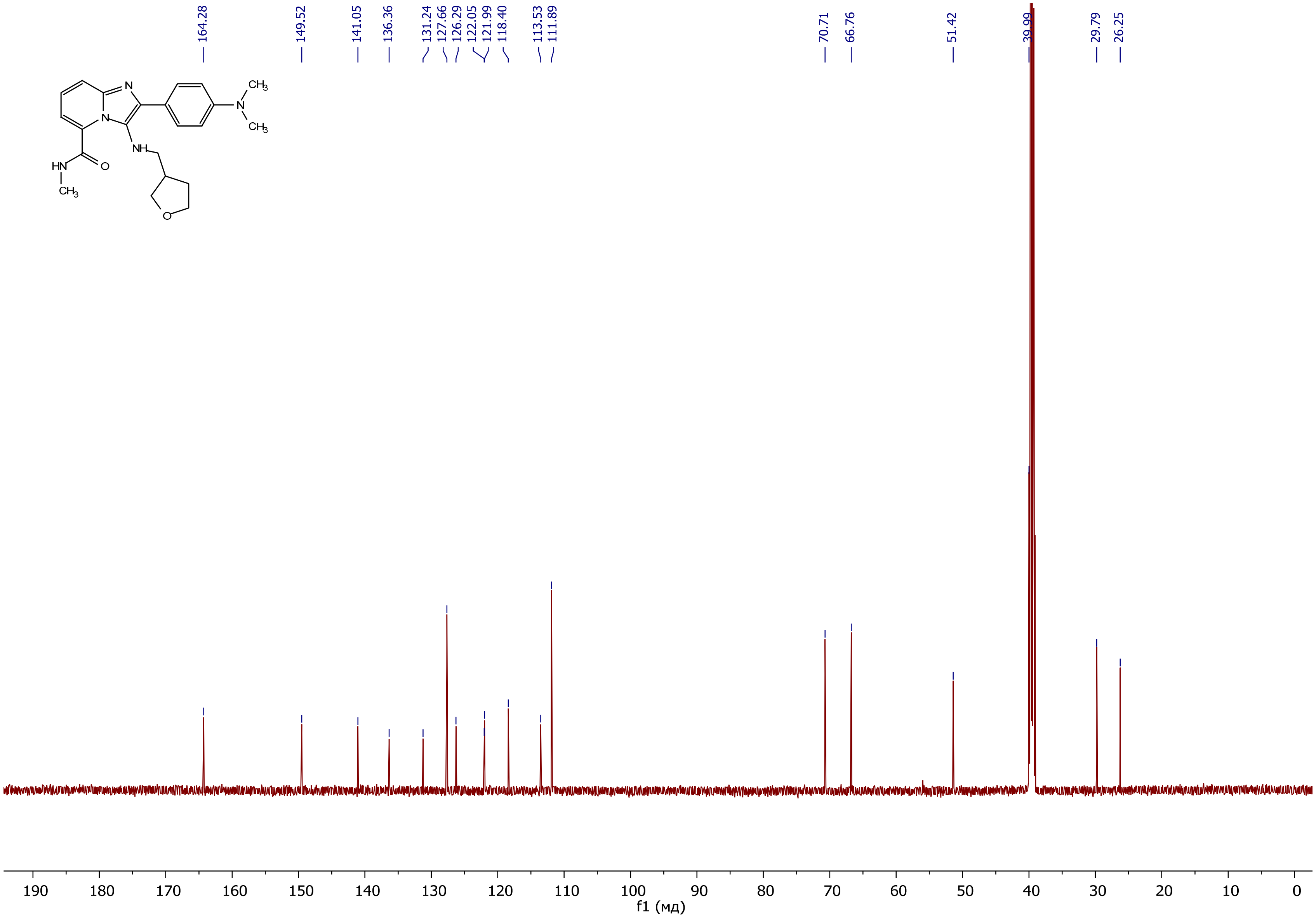

Spectrum 65. 2-[4-(Dimethylamino)phenyl]-*N*-methyl-3-{[(oxolan-3-yl)methyl]amino}imidazo[1,2-*a*]pyridine-5-carboxamide **4**{362,594,12}, $^{13}C\{^{1}H\}$ NMR (151 MHz, DMSO-d_6)

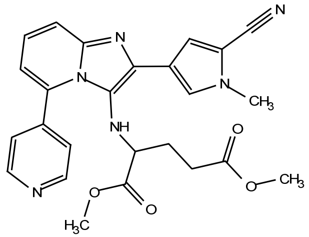

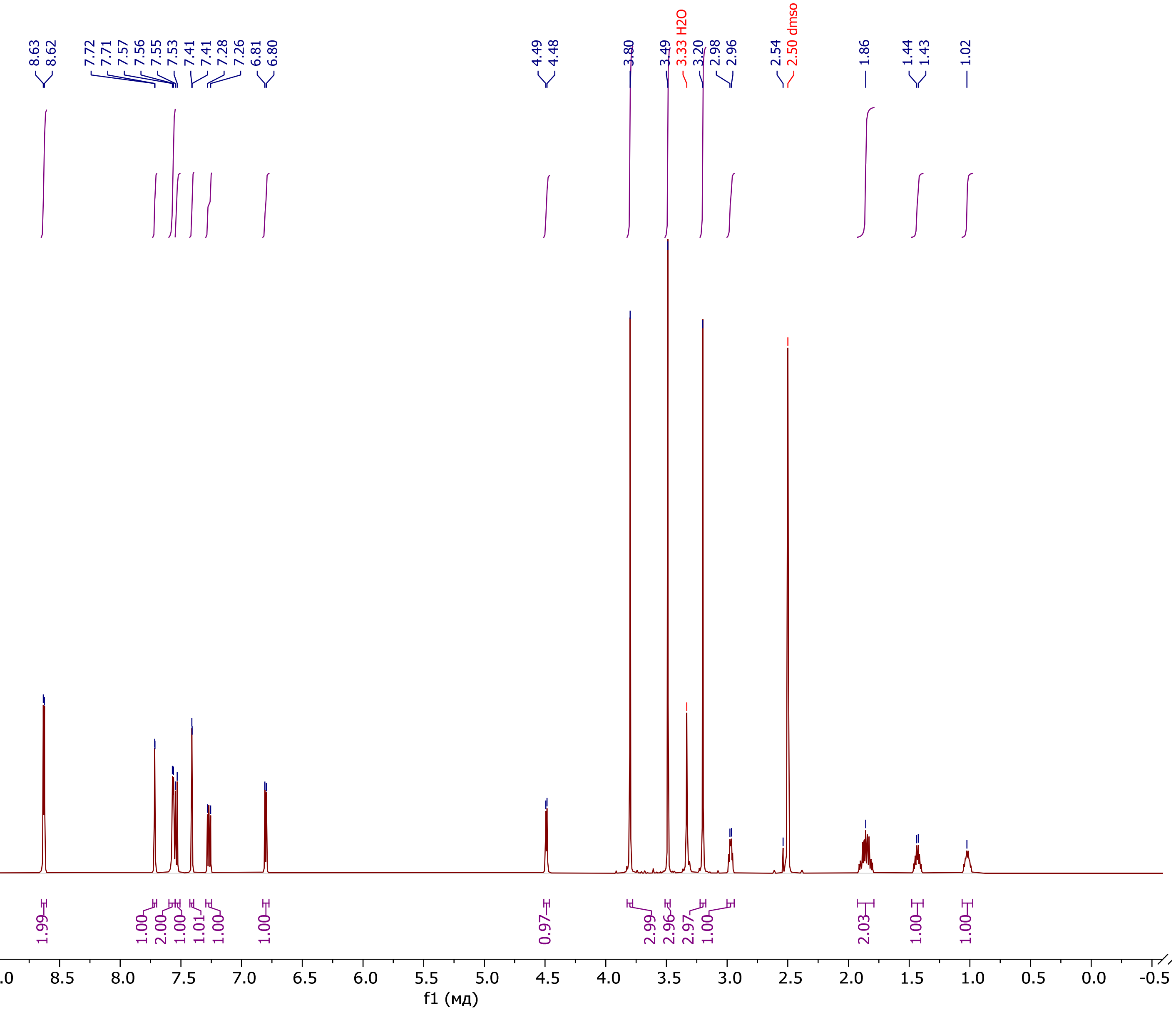

Spectrum 66. 1,5-Dimethyl 2-{[2-(5-cyano-1-methyl-1*H*-pyrrol-3-yl)-5-(pyridin-4-yl)imidazo[1,2-*a*]pyridin-3-yl]amino}pentanedioate **4**{333,219,23}, ^{1}H NMR (600 MHz, DMSO-d_6)

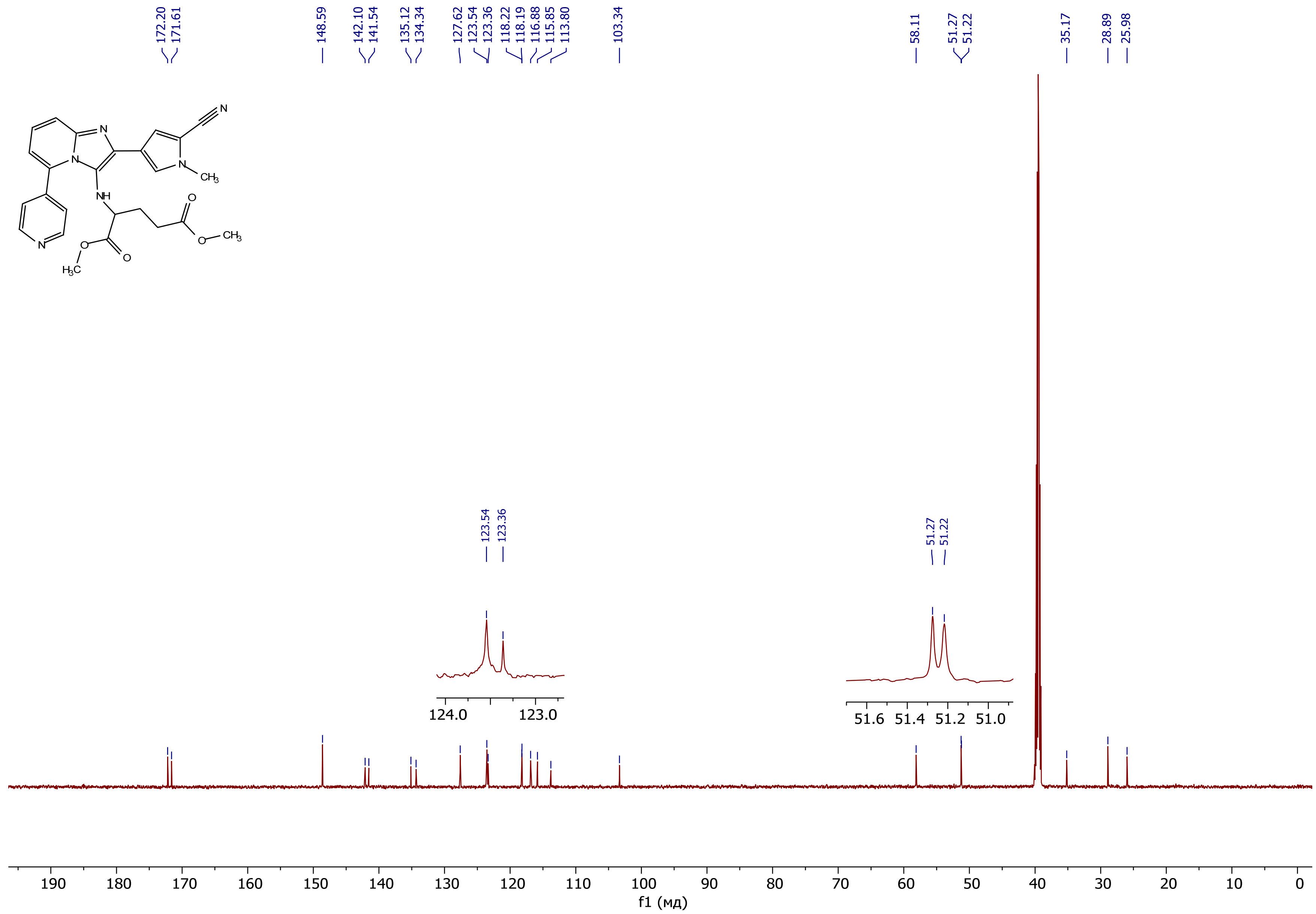

Spectrum 67. 1,5-Dimethyl 2-{[2-(5-cyano-1-methyl-1*H*-pyrrol-3-yl)-5-(pyridin-4-yl)imidazo[1,2-*a*]pyridin-3-yl]amino}pentanedioate **4**{333,219,23}, $^{13}C\{^{1}H\}$ NMR (151 MHz, DMSO-d_6)

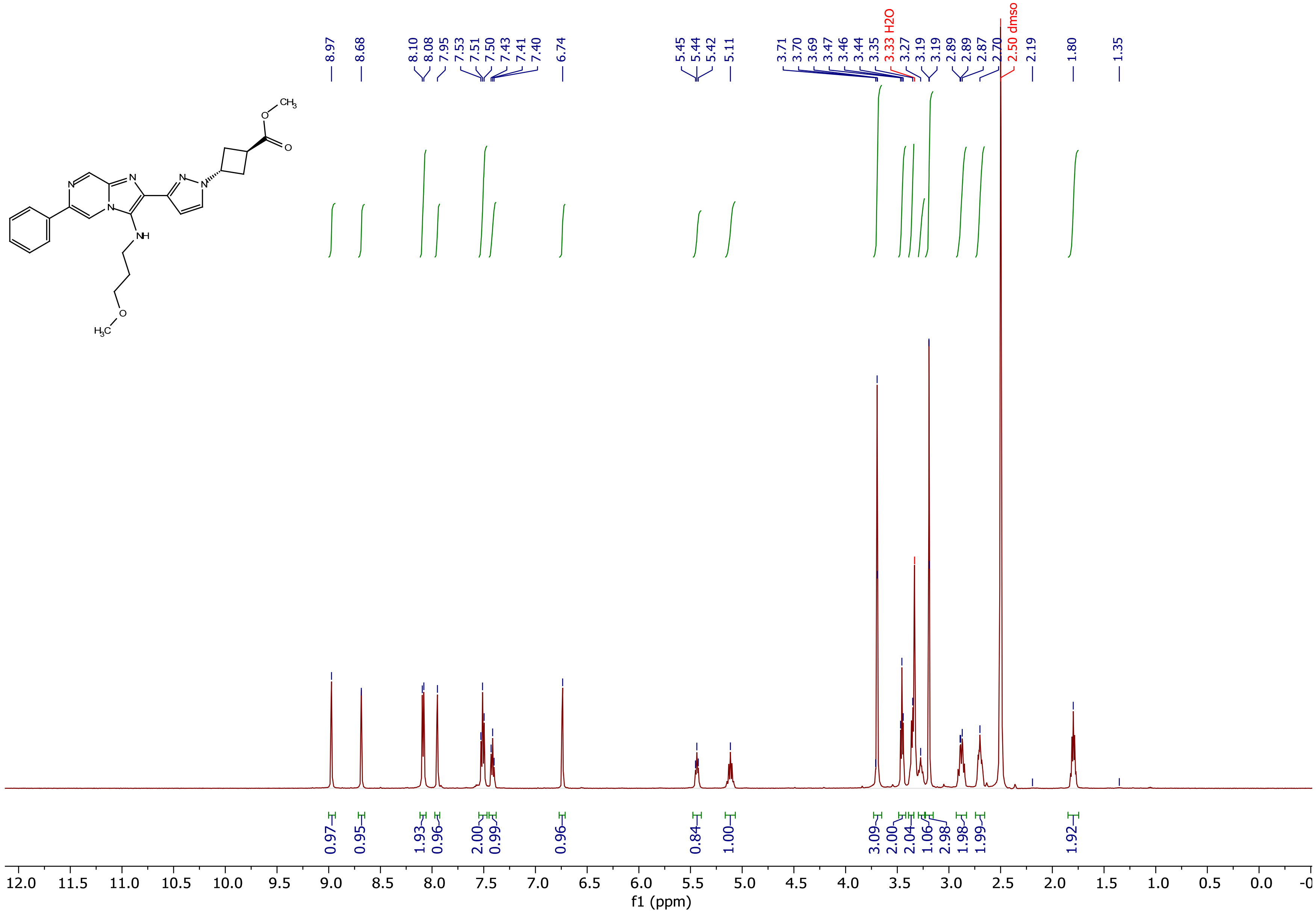

Spectrum 68. Methyl (1*r*,3*r*)-3-(3-{3-[(3-methoxypropyl)amino]-6-phenylimidazo[1,2-*a*]pyrazin-2-yl}-1*H*-pyrazol-1-yl)cyclobutane-1-carboxylate **4**{350,165,5}, ^{1}H NMR (500 MHz, DMSO-d_6)

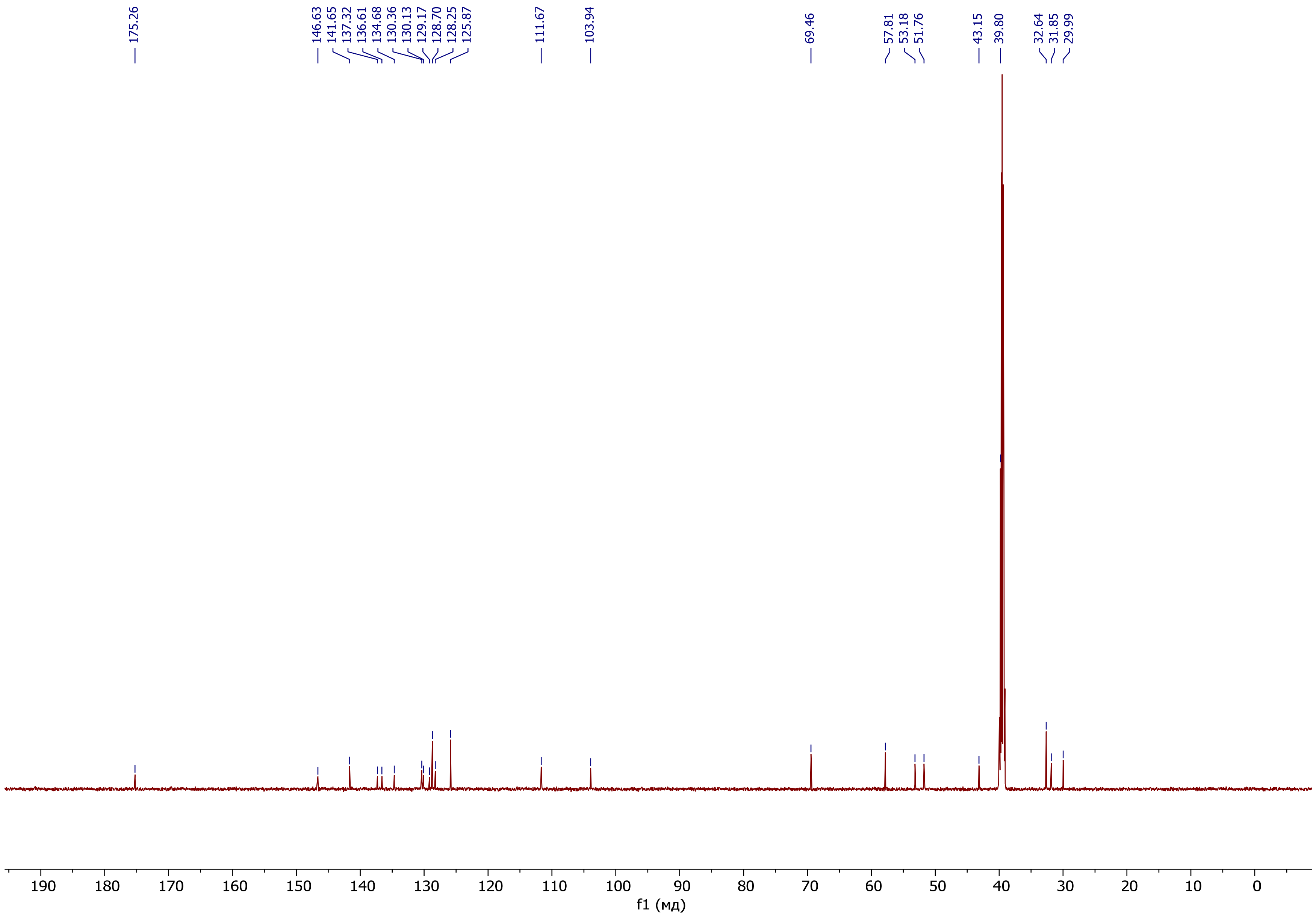

Spectrum 69. Methyl (1*r*,3*r*)-3-(3-{3-[(3-methoxypropyl)amino]-6-phenylimidazo[1,2-*a*]pyrazin-2-yl}-1*H*-pyrazol-1-yl)cyclobutane-1-carboxylate **4**{350,165,5}, $^{13}C\{^{1}H\}$ NMR (151 MHz, DMSO-d_6)

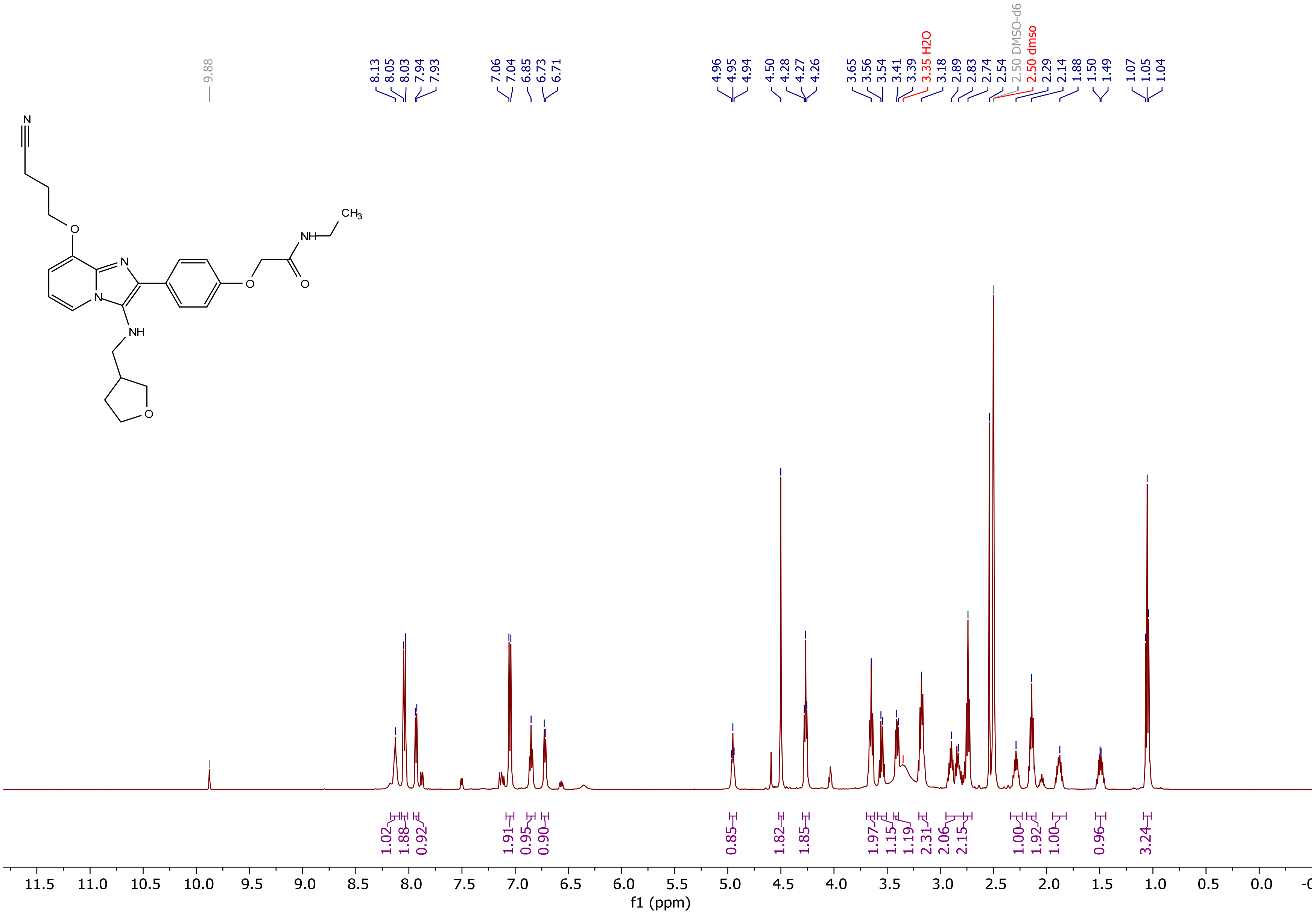

Spectrum 70. 2-{4-[8-(3-Cyanopropoxy)-3-{[(oxolan-3-yl)methyl]amino}imidazo[1,2-*a*]pyridin-2-yl]phenoxy}-*N*-ethylacetamide **4**{371,622,12}, ^{1}H NMR (500 MHz, DMSO-d_6)

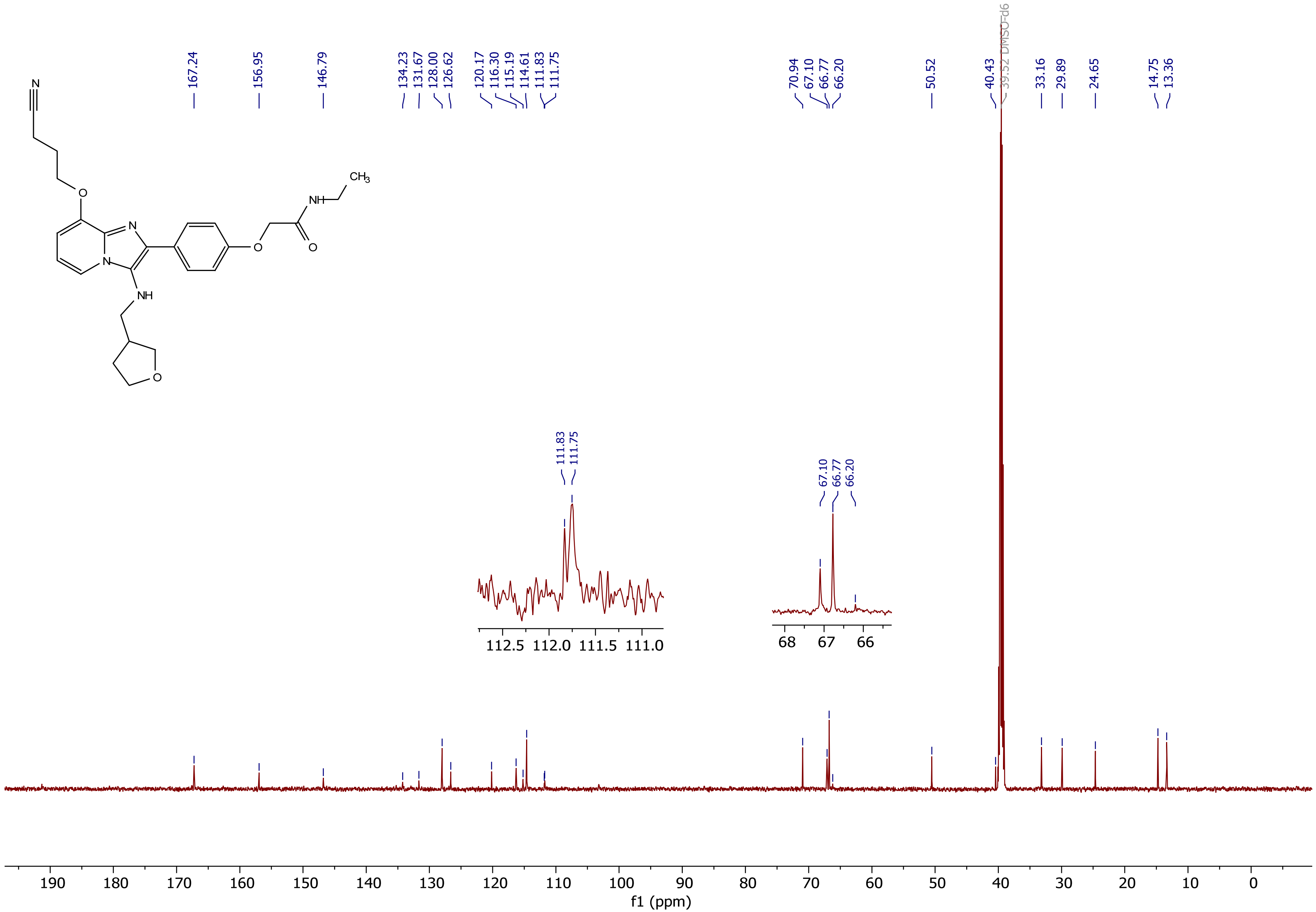

Spectrum 71. 2-{4-[8-(3-Cyanopropoxy)-3-{[(oxolan-3-yl)methyl]amino}imidazo[1,2-*a*]pyridin-2-yl]phenoxy}-*N*-ethylacetamide **4**{371,622,12}, $^{13}C\{^{1}H\}$ NMR (151 MHz, DMSO-d_6)

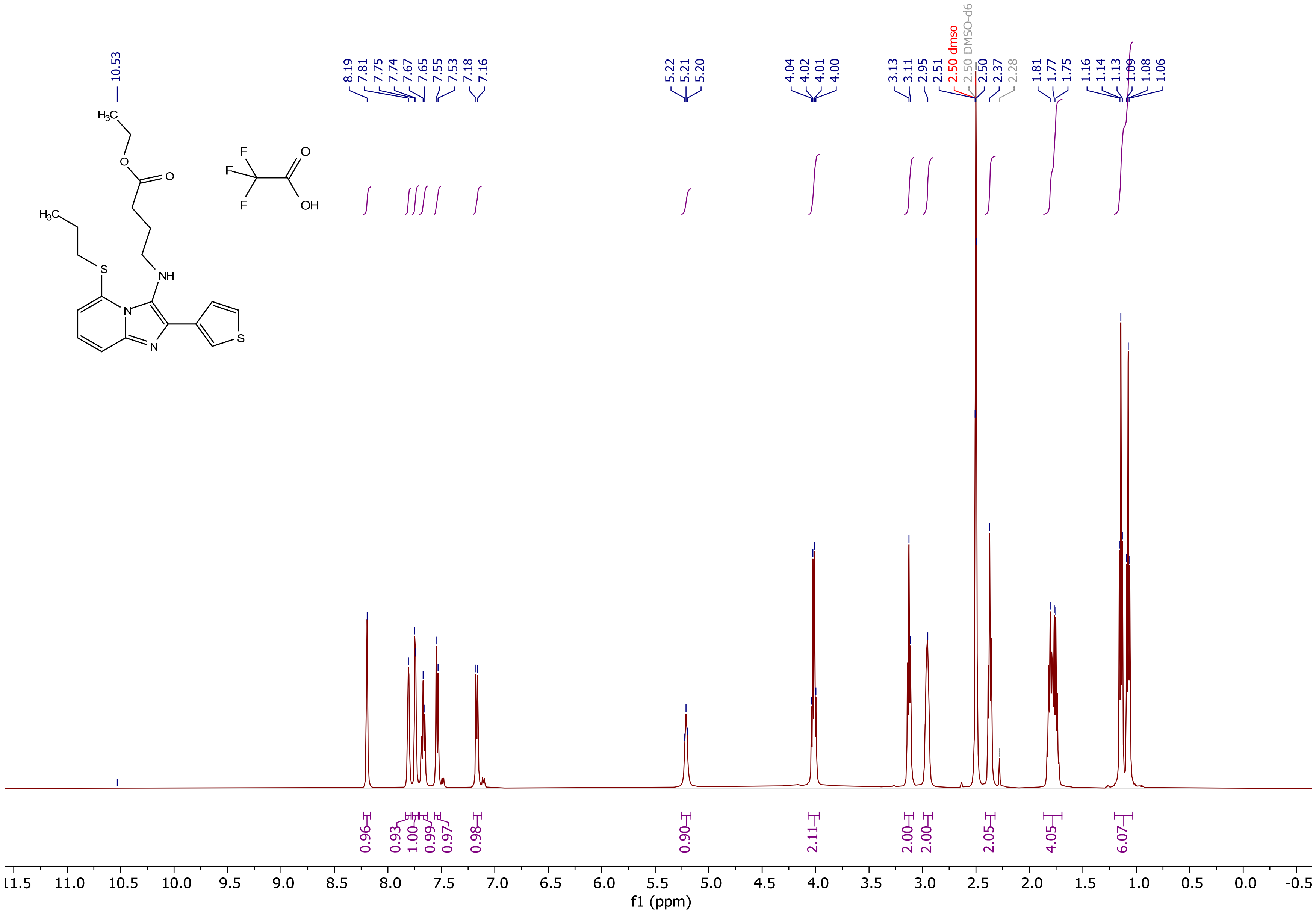

Spectrum 72. Ethyl 4-{[5-(propylsulfanyl)-2-(thiophen-3-yl)imidazo[1,2-*a*]pyridin-3-yl]amino}butanoate trifluoroacetate **4**{33,9,6}, ^{1}H NMR (500 MHz, DMSO-d_6)

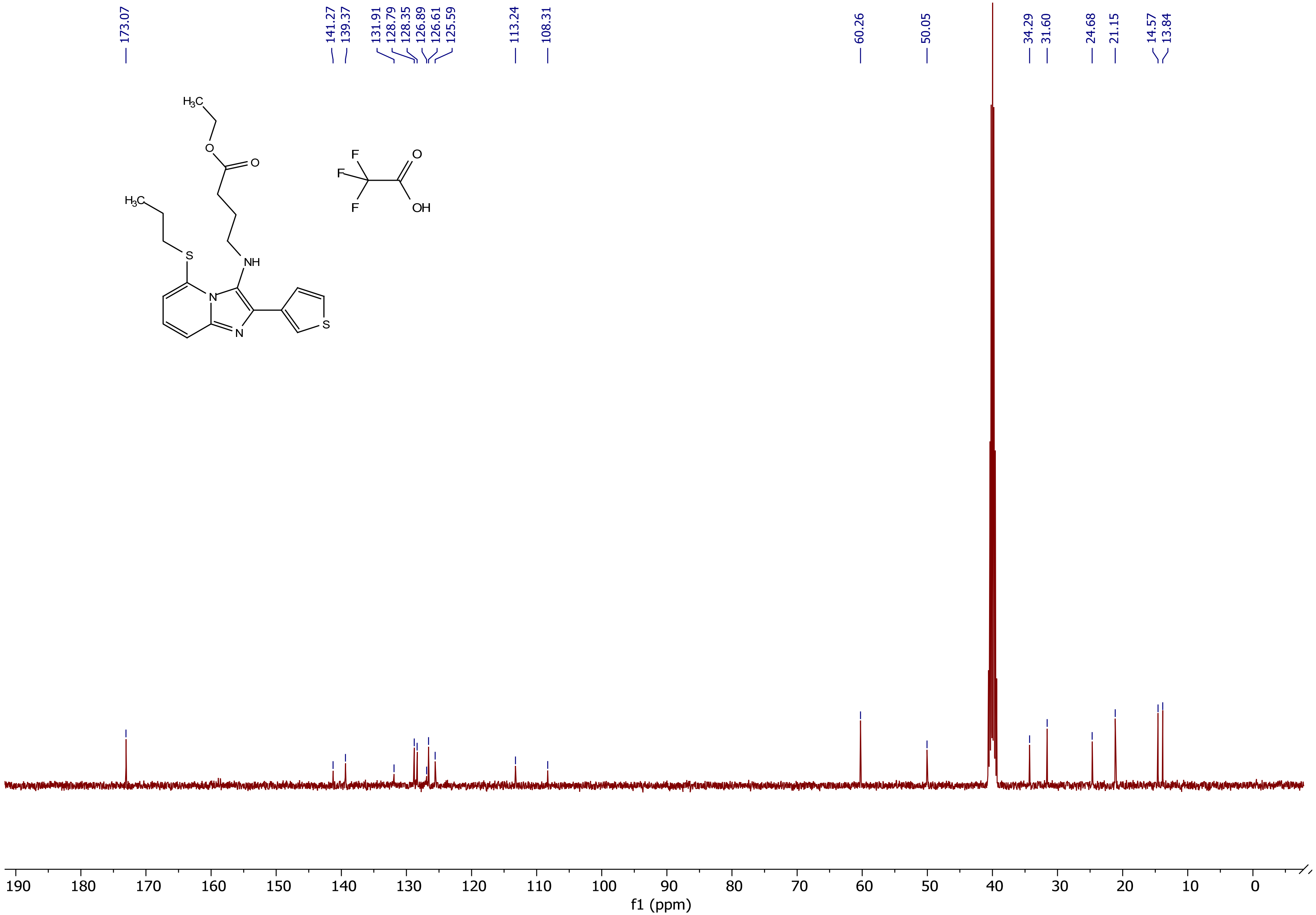

Spectrum 73. Ethyl 4-{[5-(propylsulfanyl)-2-(thiophen-3-yl)imidazo[1,2-*a*]pyridin-3-yl]amino}butanoate trifluoroacetate **4**{33,9,6}, $^{13}C\{^{1}H\}$ NMR (101 MHz, DMSO-d_6)

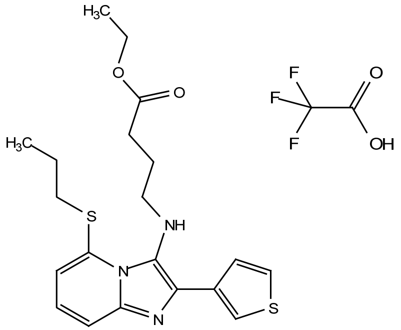

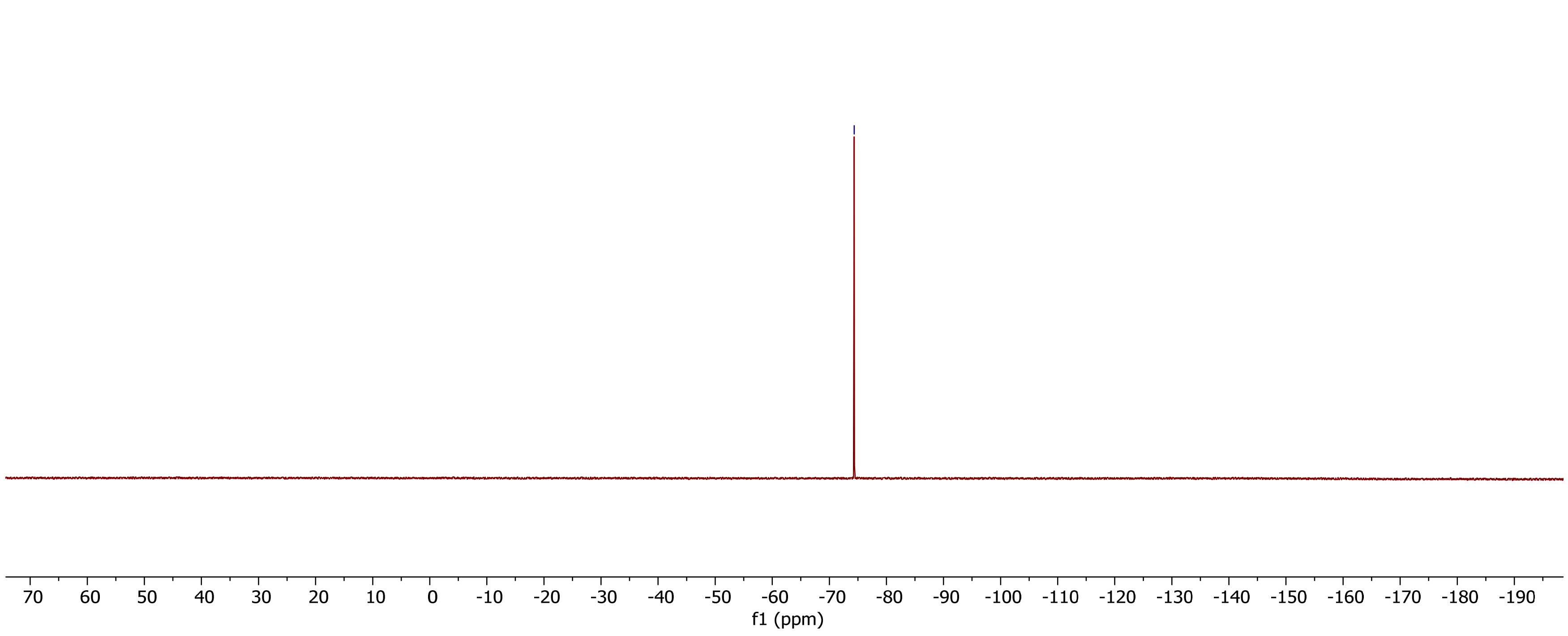

Spectrum 74. Ethyl 4-{[5-(propylsulfanyl)-2-(thiophen-3-yl)imidazo[1,2-*a*]pyridin-3-yl]amino}butanoate trifluoroacetate **4**{33,9,6}, $^{19}F\{^{1}H\}$ NMR (376 MHz, DMSO-d_6)

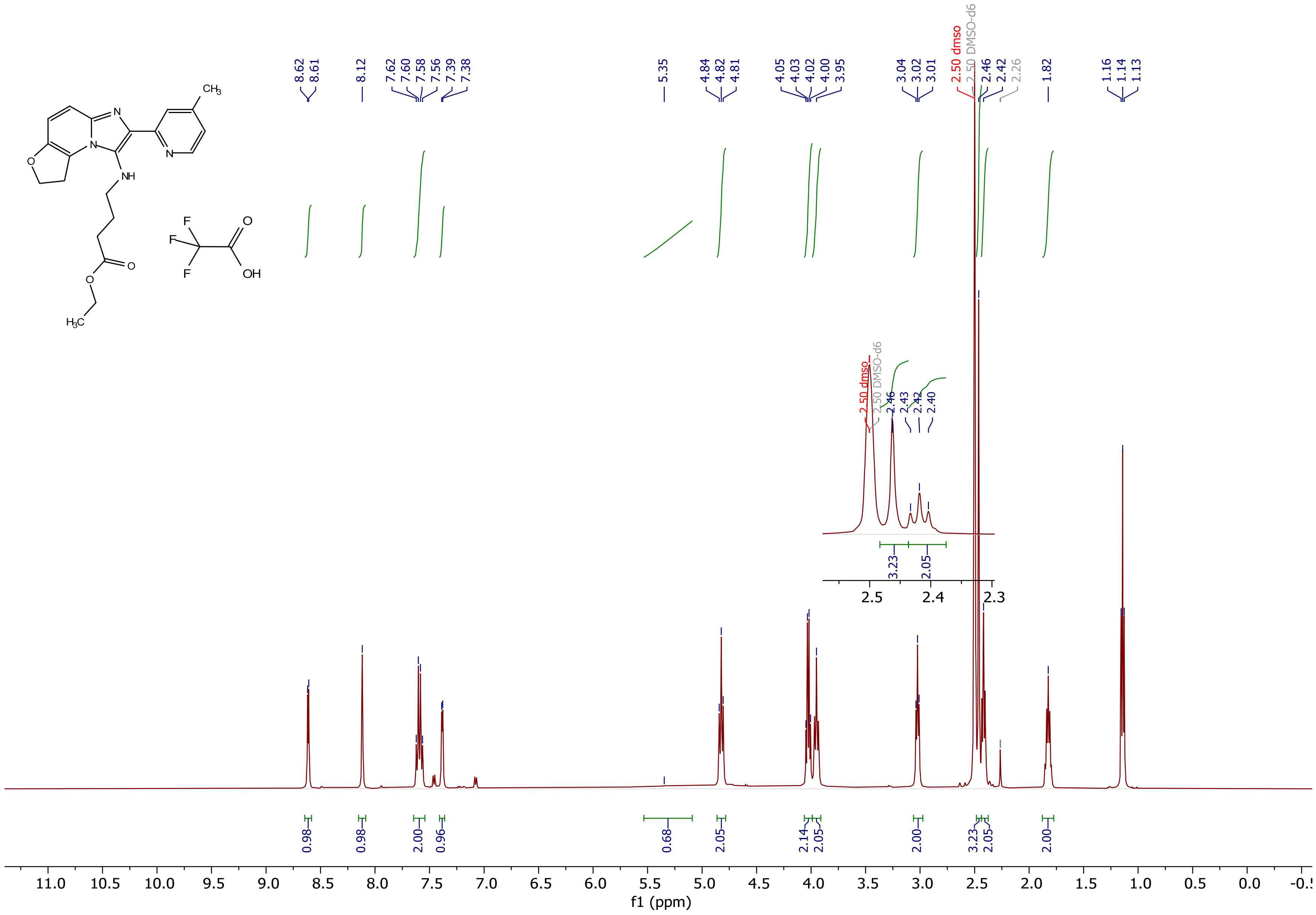

Spectrum 75. ethyl 4-{[11-(4-methylpyridin-2-yl)-5-oxa-1,10-diazatricyclo[7.3.0.0²,⁶]dodeca-2(6),7,9,11-tetraen-12-yl]amino}butanoate trifluoroacetate **4**{31,10,6}, ^{1}H NMR (500 MHz, DMSO-d_6)

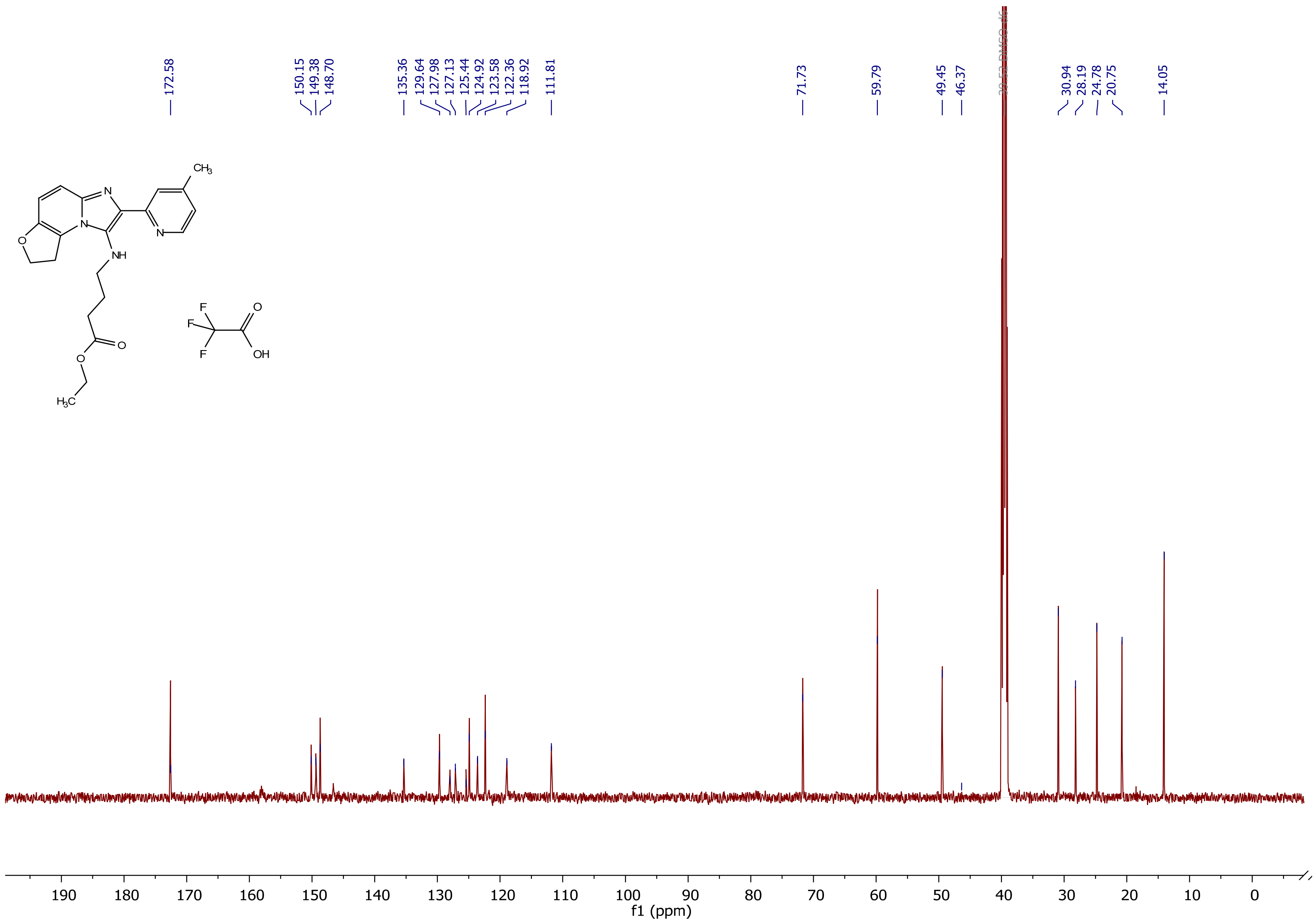

Spectrum 76. Ethyl 4-{[11-(4-methylpyridin-2-yl)-5-oxa-1,10-diazatricyclo[7.3.0.0²,⁶]dodeca-2(6),7,9,11-tetraen-12-yl]amino}butanoate trifluoroacetate **4**{31,10,6}, $^{13}C\{^{1}H\}$ NMR (151 MHz, DMSO-d_6)

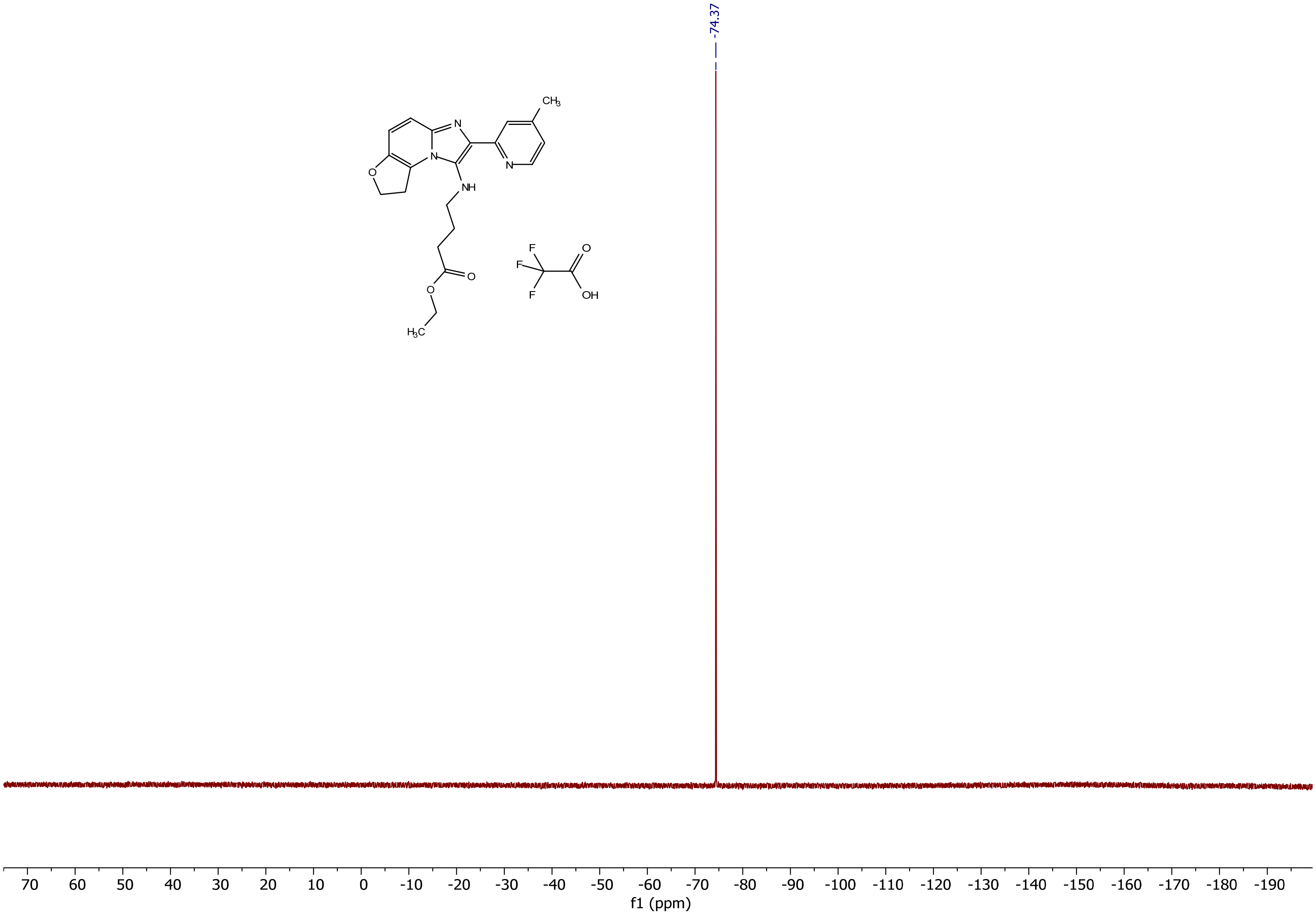

Spectrum 77. Ethyl 4-{[11-(4-methylpyridin-2-yl)-5-oxa-1,10-diazatricyclo[7.3.0.$0^{2,6}$]dodeca-2(6),7,9,11-tetraen-12-yl]amino}butanoate trifluoroacetate **4**{31,10,6}, $^{19}F\{^{1}H\}$ NMR (376 MHz, DMSO-d_6)

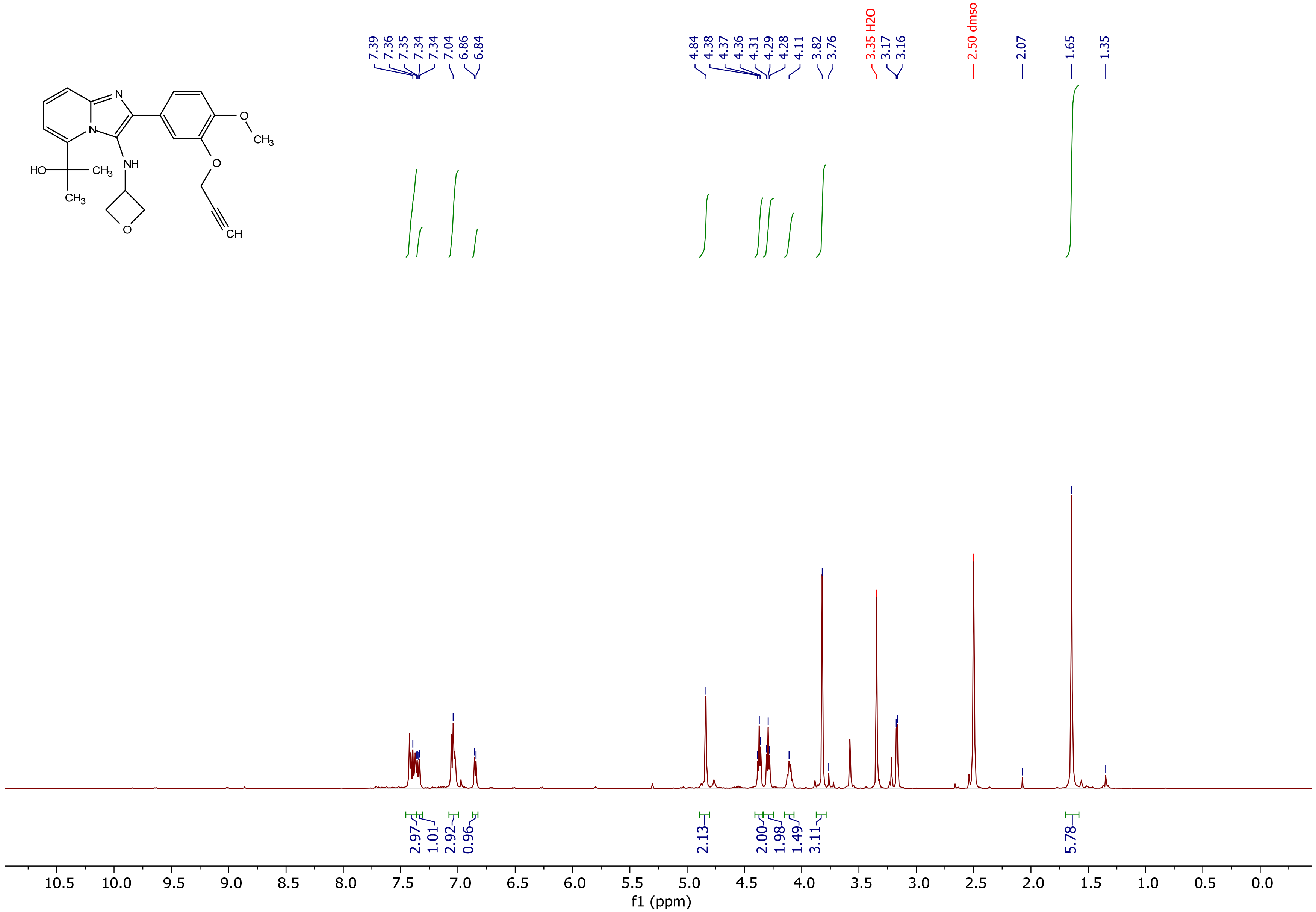

Spectrum 78. 2-{2-[4-Methoxy-3-(prop-2-yn-1-yloxy)phenyl]-3-[(oxetan-3-yl)amino]imidazo[1,2-*a*]pyridin-5-yl}propan-2-ol **4**{153,289,22}, ^{1}H NMR (500 MHz, DMSO-d_6)

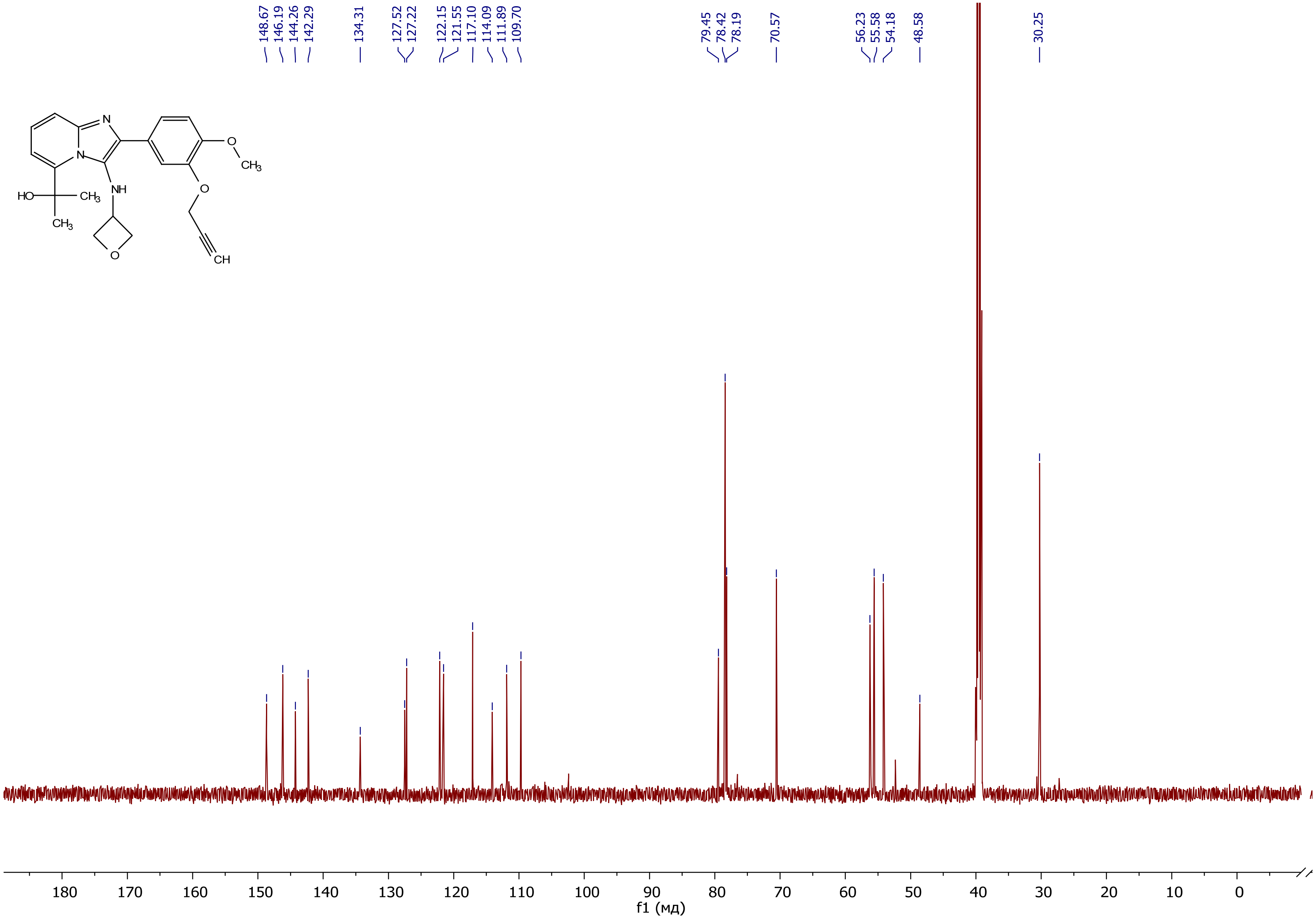

Spectrum 79. 2-{2-[4-methoxy-3-(prop-2-yn-1-yloxy)phenyl]-3-[(oxetan-3-yl)amino]imidazo[1,2-*a*]pyridin-5-yl}propan-2-ol **4**{153,289,22}, $^{13}C\{^{1}H\}$ NMR (151 Mhz, DMSO-d_6)

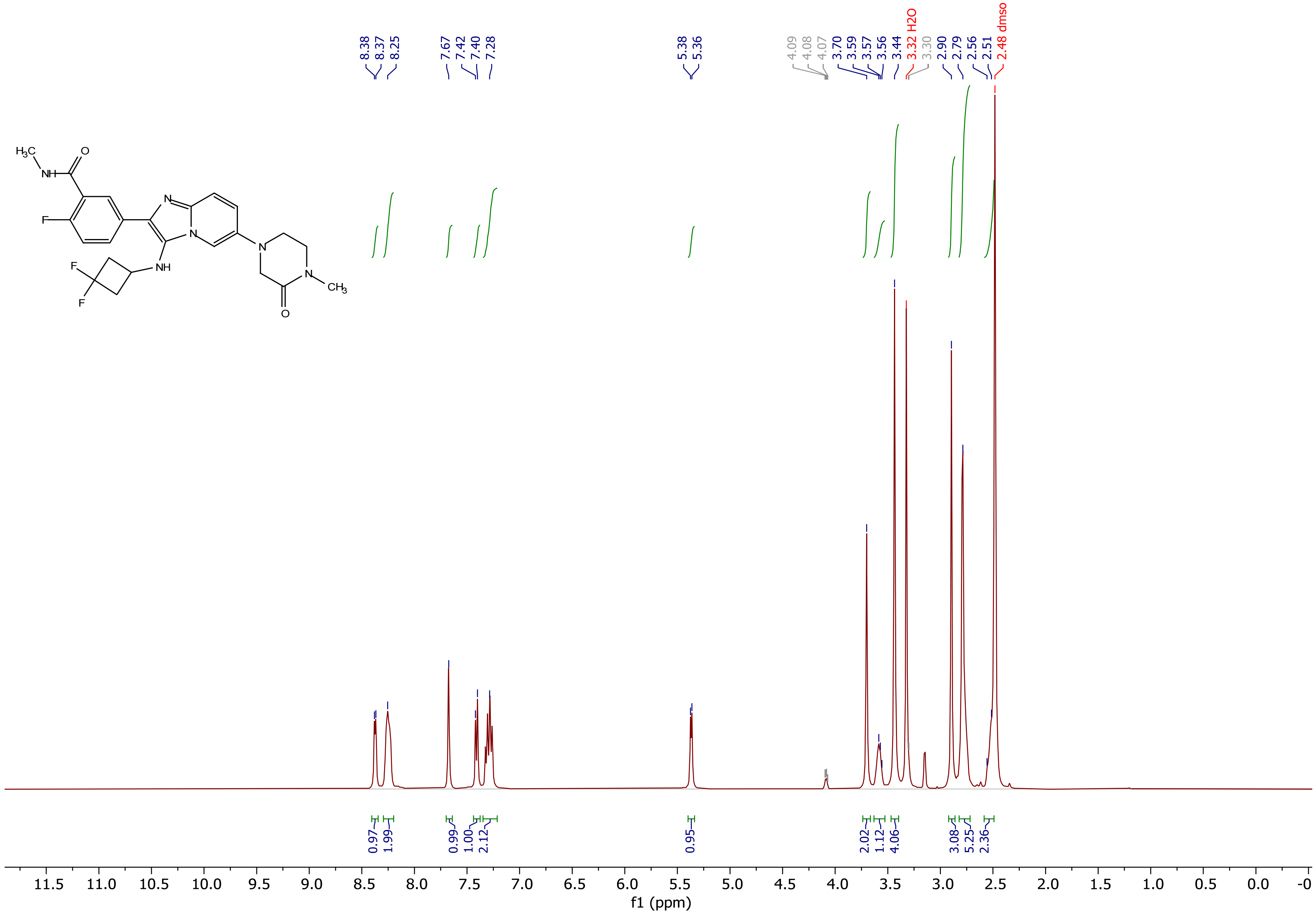

Spectrum 80. 5-{3-[(3,3-Difluorocyclobutyl)amino]-6-(4-methyl-3-oxopiperazin-1-yl)imidazo[1,2-*a*]pyridin-2-yl}-2-fluoro-*N*-methylbenzamide **4**{352,301,49}, ^{1}H NMR (500 MHz, DMSO-d_6)

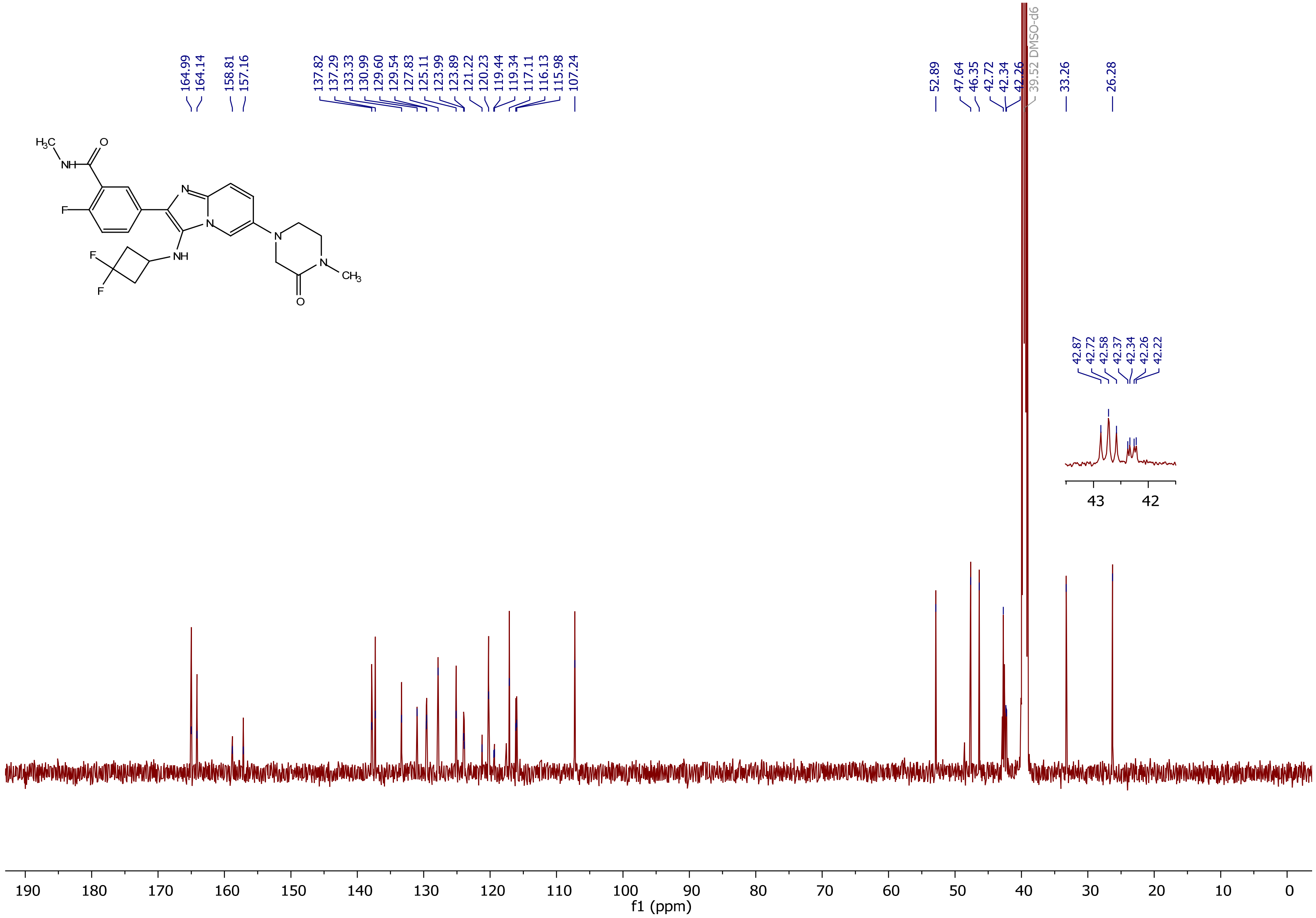

Spectrum 81. 5-{3-[(3,3-Difluorocyclobutyl)amino]-6-(4-methyl-3-oxopiperazin-1-yl)imidazo[1,2-*a*]pyridin-2-yl}-2-fluoro-*N*-methylbenzamide **4**{352,301,49}, $^{13}C\{^{1}H\}$ NMR (151 MHz, DMSO-d_6)

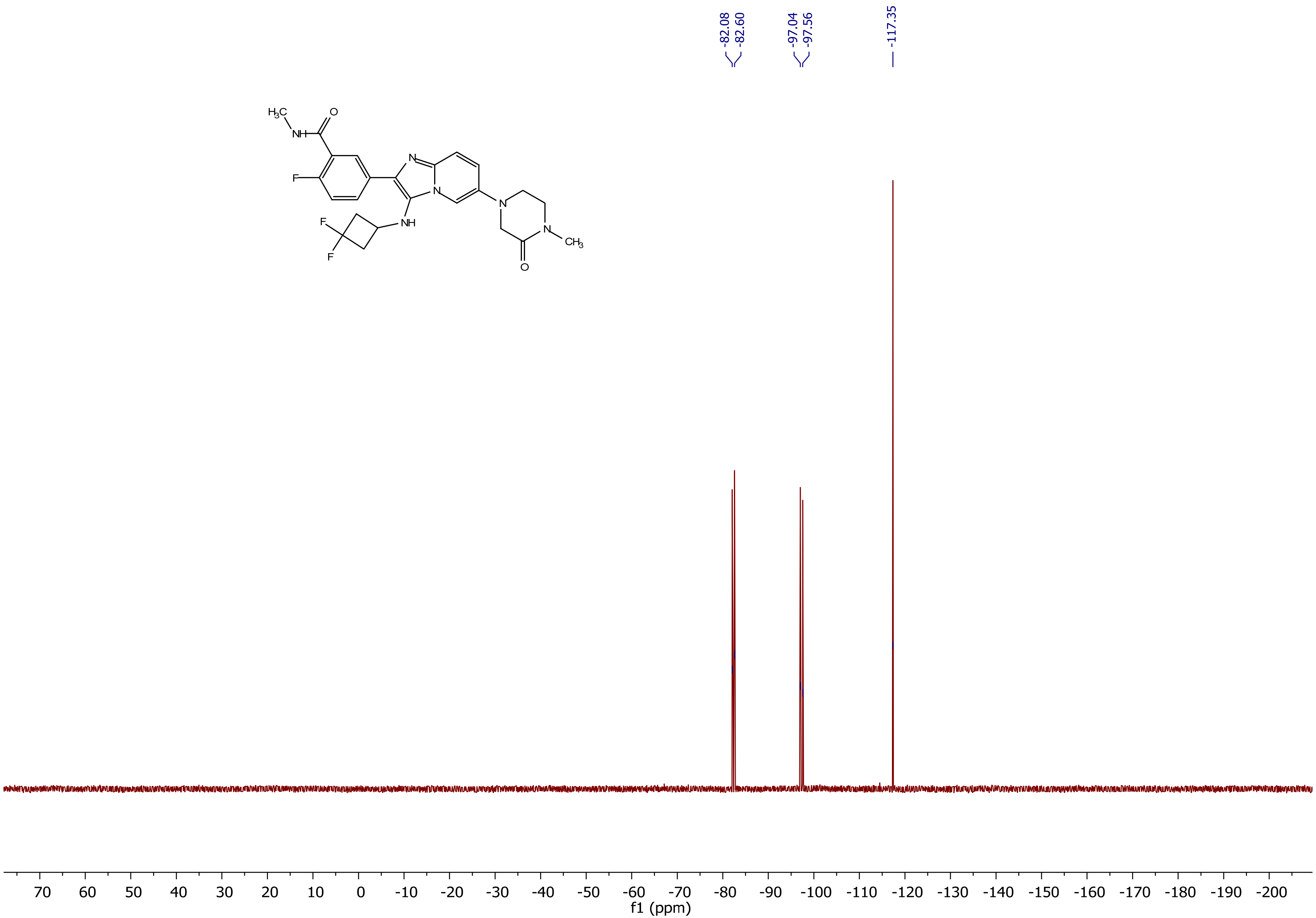

Spectrum 82. 5-{3-[(3,3-Difluorocyclobutyl)amino]-6-(4-methyl-3-oxopiperazin-1-yl)imidazo[1,2-*a*]pyridin-2-yl}-2-fluoro-*N*-methylbenzamide **4**{352,301,49}, $^{19}F\{^{1}H\}$ NMR (376 MHz, DMSO-d_6)

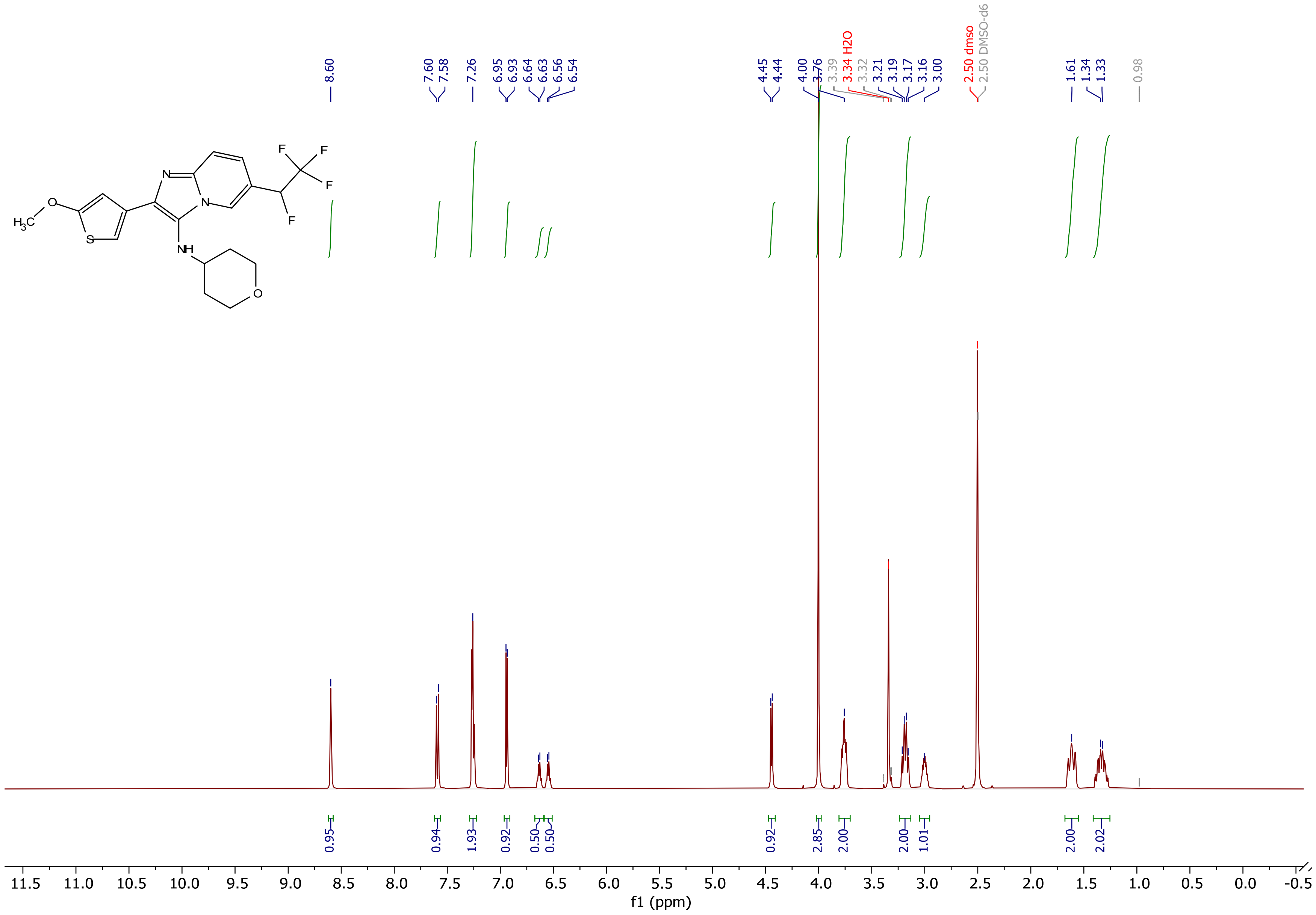

Spectrum 83. 2-(2-Methoxythiophen-3-yl)-*N*-(oxan-4-yl)-6-(1,2,2,2-tetrafluoroethyl)imidazo[1,2-*a*]pyridin-3-amine **4**{369,54,7}, ^{1}H NMR (500 MHz, DMSO-d_6

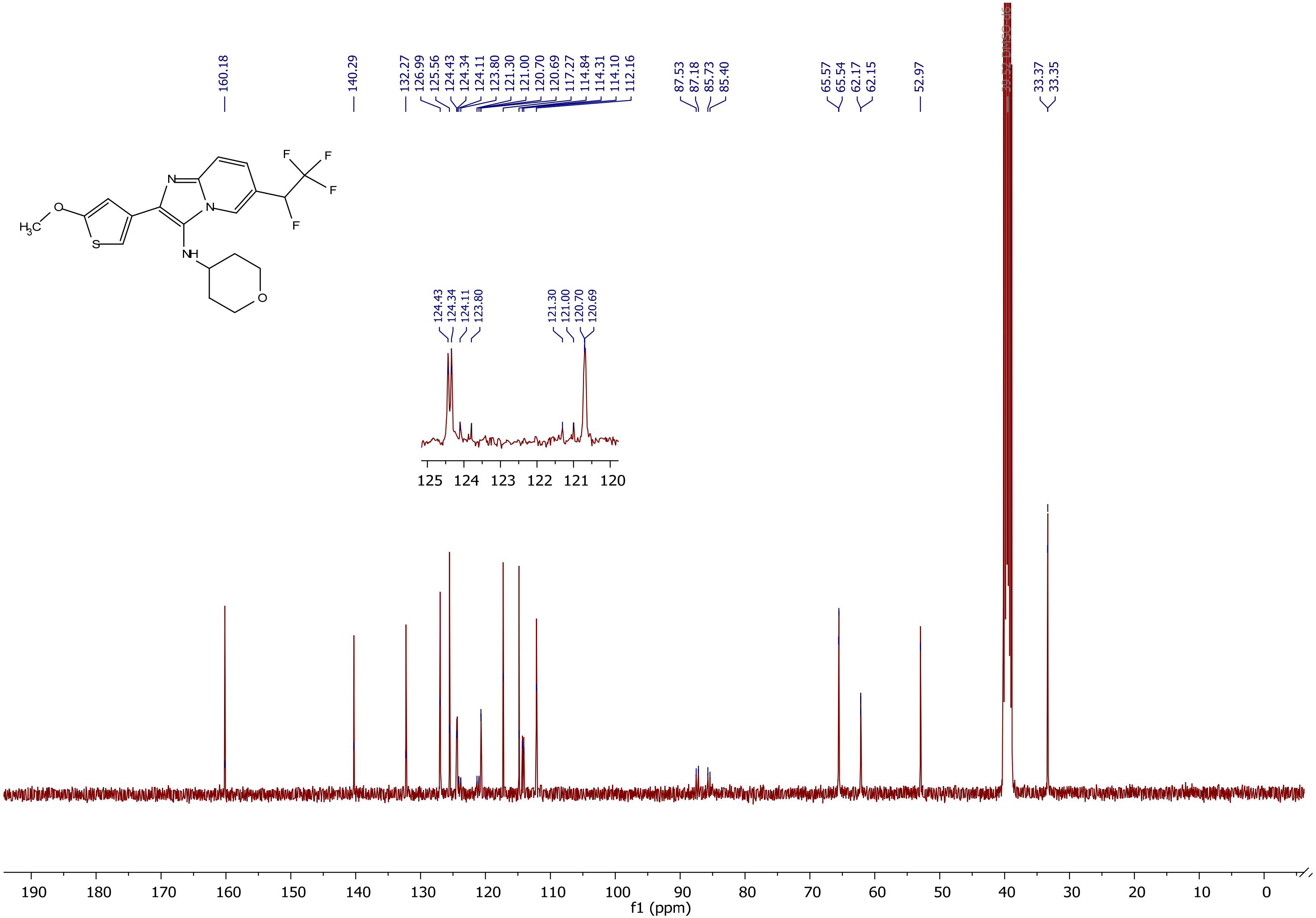

Spectrum 84. 2-(2-Methoxythiophen-3-yl)-*N*-(oxan-4-yl)-6-(1,2,2,2-tetrafluoroethyl)imidazo[1,2-*a*]pyridin-3-amine **4**{369,54,7}, $^{13}C\{^{1}H\}$ NMR (101 MHz, DMSO-d_6)

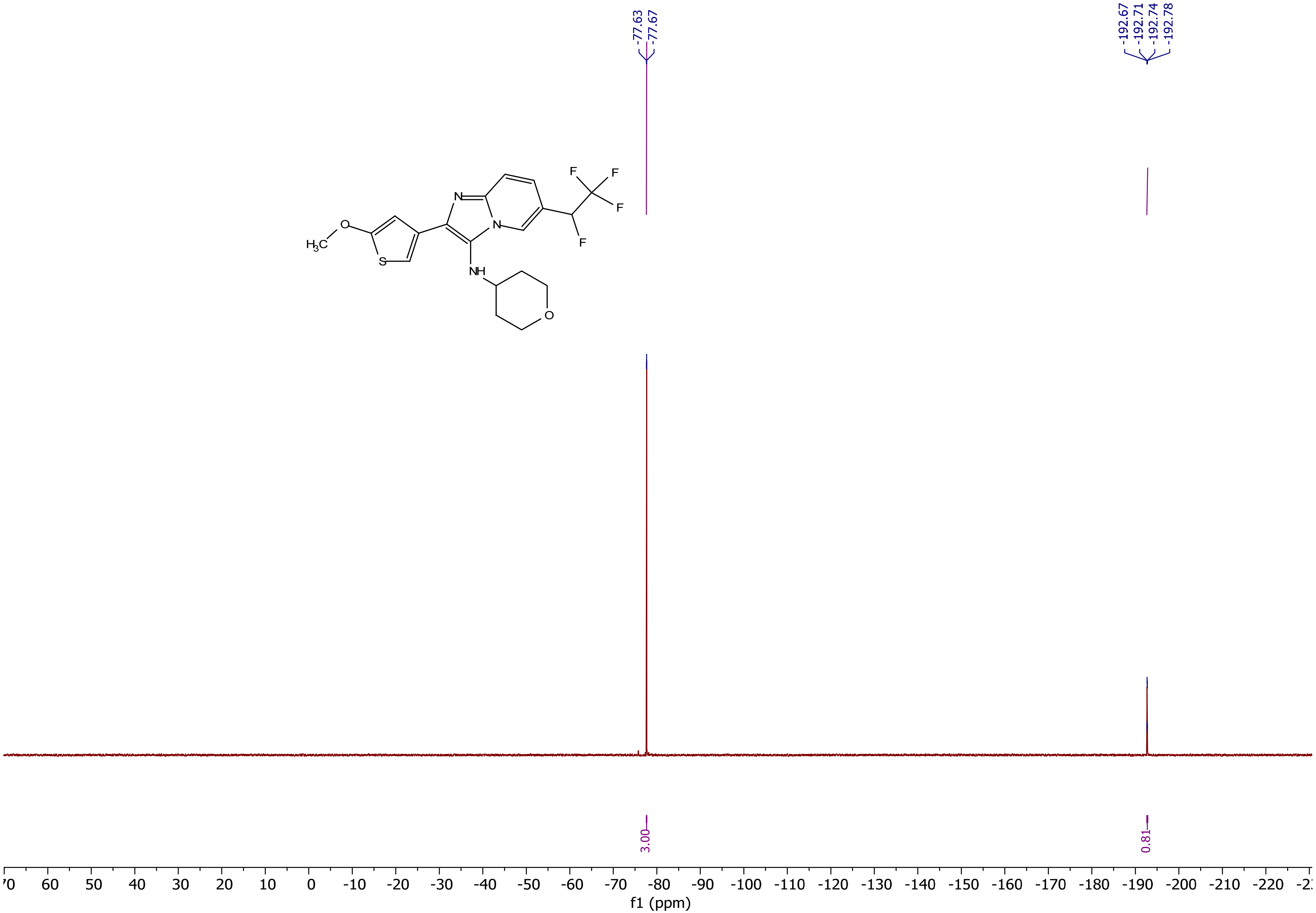

Spectrum 85. 2-(2-Methoxythiophen-3-yl)-*N*-(oxan-4-yl)-6-(1,2,2,2-tetrafluoroethyl)imidazo[1,2-*a*]pyridin-3-amine **4**{369,54,7}, $^{19}F\{^{1}H\}$ NMR (376 MHz, DMSO-d_6)

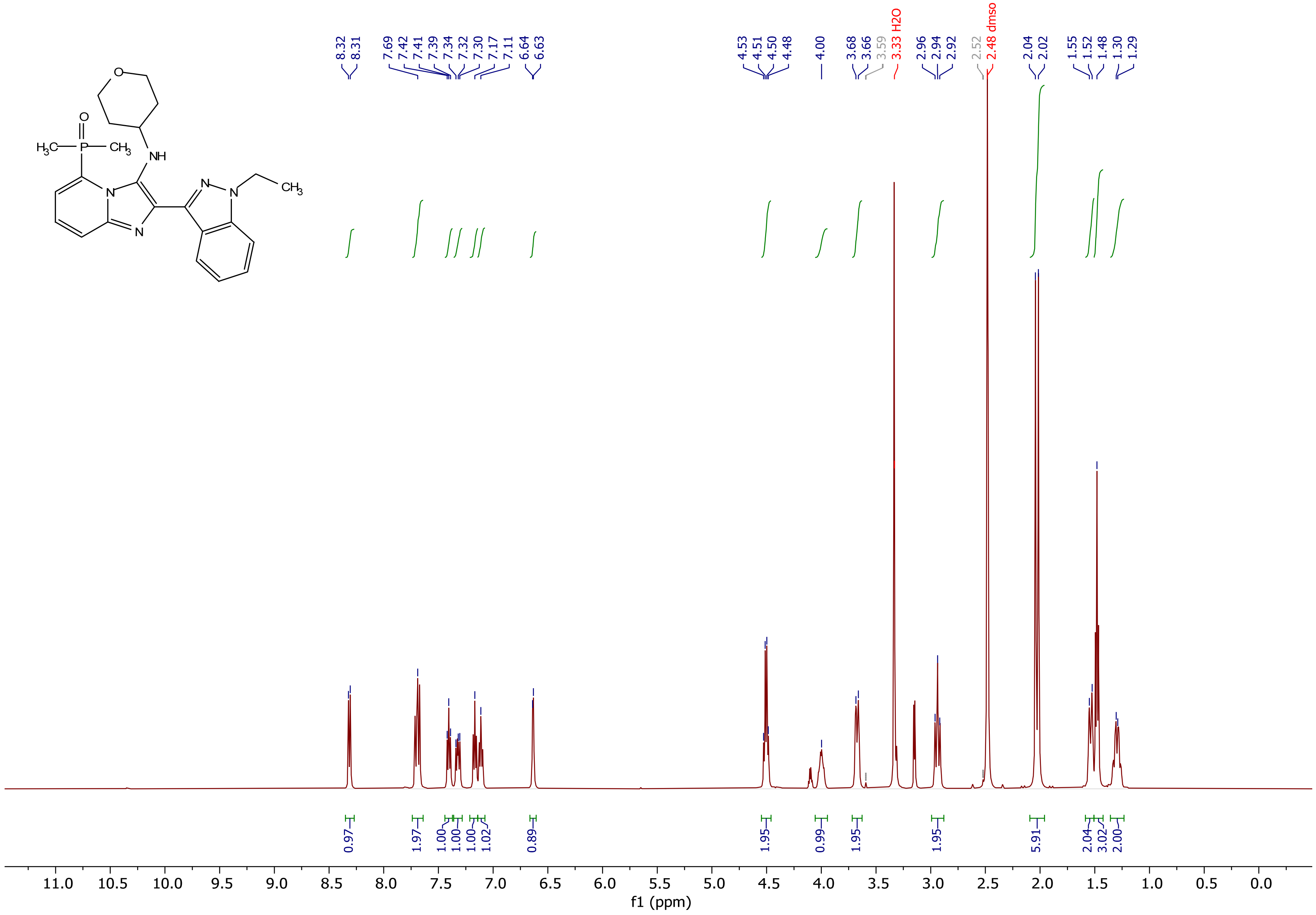

Spectrum 86. 5-(Dimethylphosphoryl)-2-(1-ethyl-1*H*-indazol-3-yl)-*N*-(oxan-4-yl)imidazo[1,2-*a*]pyridin-3-amine **4**{50,604,7}, ^{1}H NMR (500 MHz, DMSO-d_6)

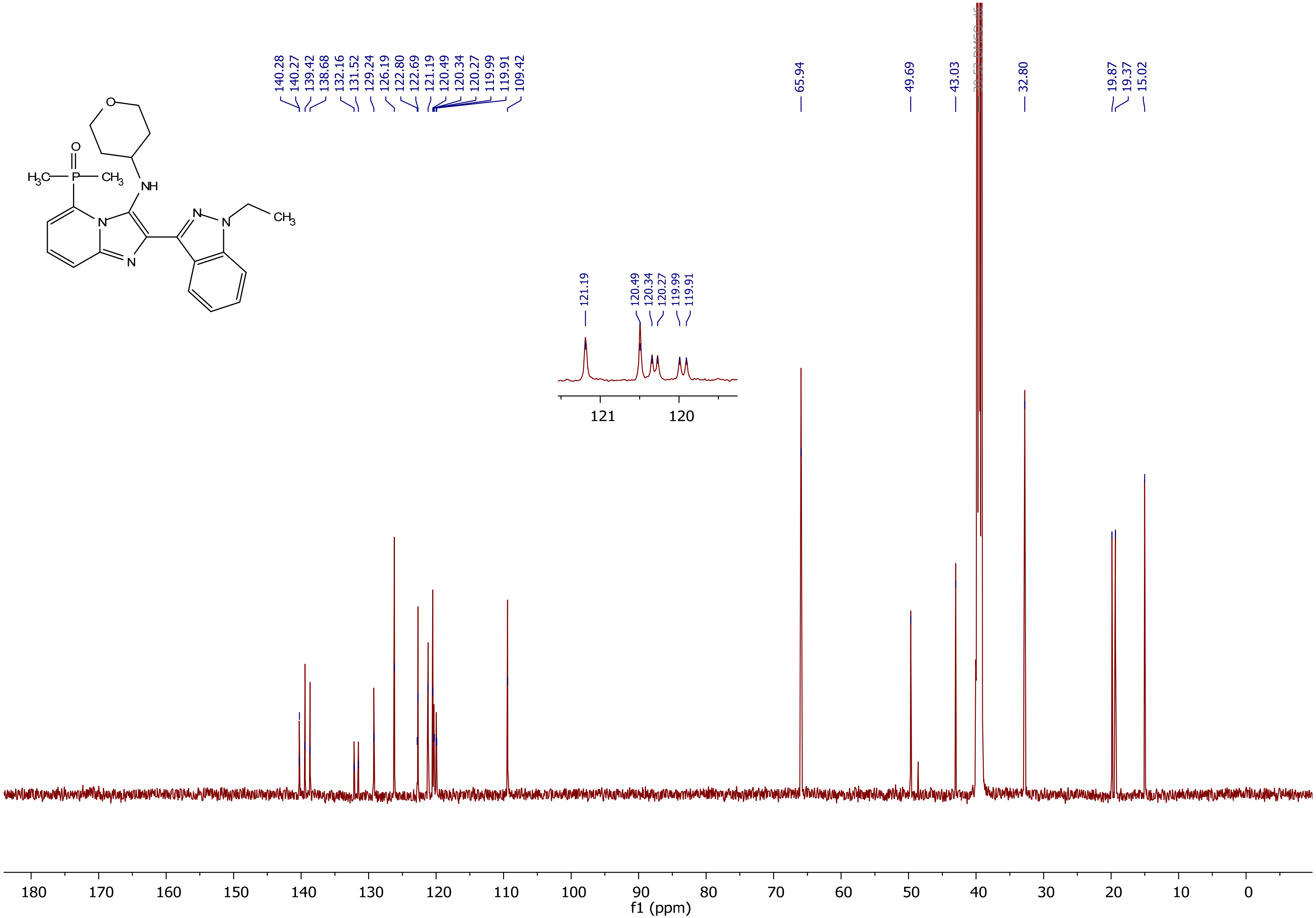

Spectrum 87. 5-(Dimethylphosphoryl)-2-(1-ethyl-1*H*-indazol-3-yl)-*N*-(oxan-4-yl)imidazo[1,2-*a*]pyridin-3-amine **4**{50,604,7}, $^{13}C\{^{1}H\}$ NMR (151 MHz, DMSO-d_6)

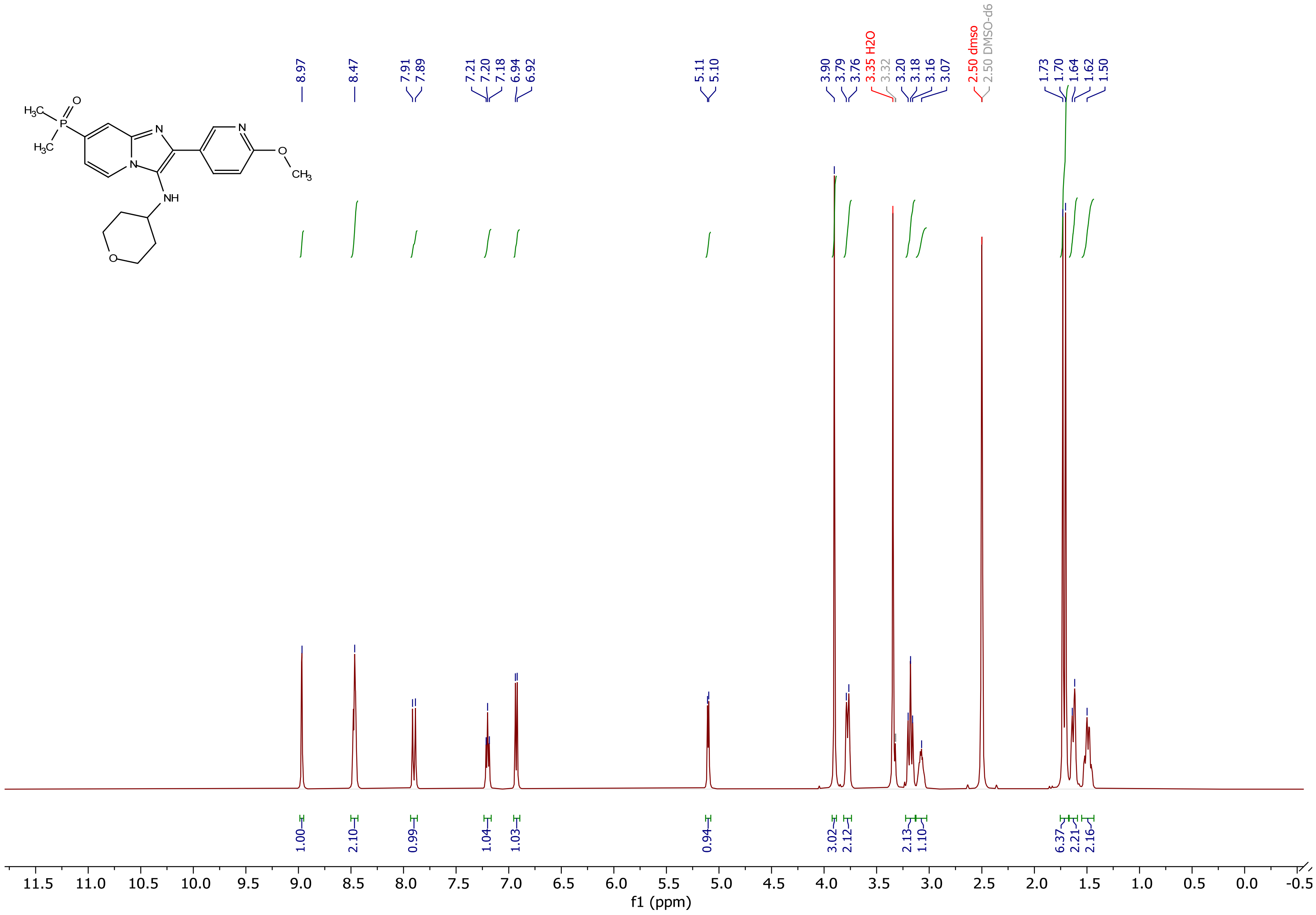

Spectrum 88. 7-(Dimethylphosphoryl)-2-(6-methoxypyridin-3-yl)-*N*-(oxan-4-yl)imidazo[1,2-*a*]pyridin-3-amine **4**{32,12,7}, ^{1}H NMR (500 MHz, DMSO-d_6)

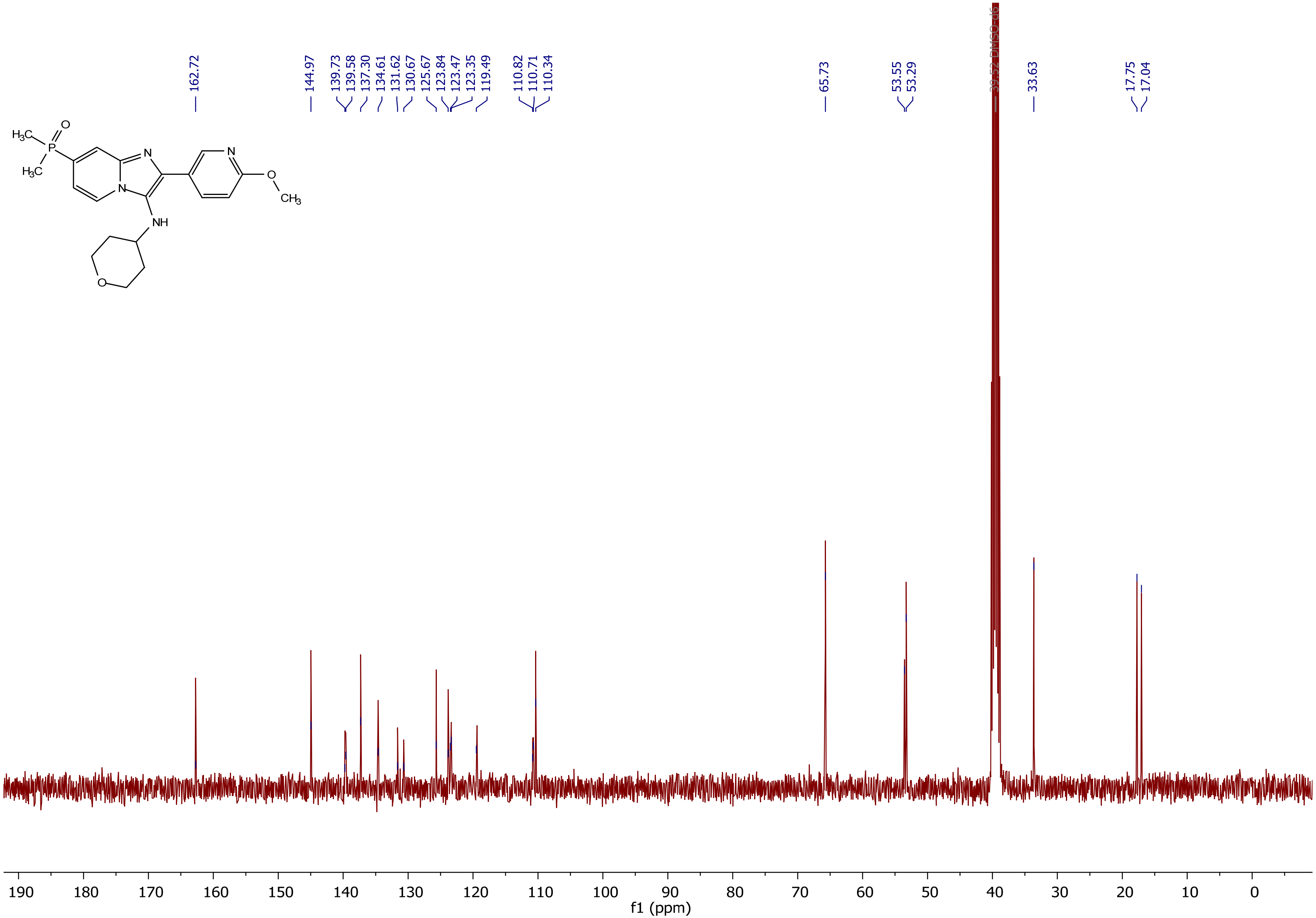

Spectrum 89. 7-(Dimethylphosphoryl)-2-(6-methoxypyridin-3-yl)-*N*-(oxan-4-yl)imidazo[1,2-*a*]pyridin-3-amine **4**{32,12,7}, $^{13}C\{^{1}H\}$ NMR (101 MHz, DMSO-d_6)

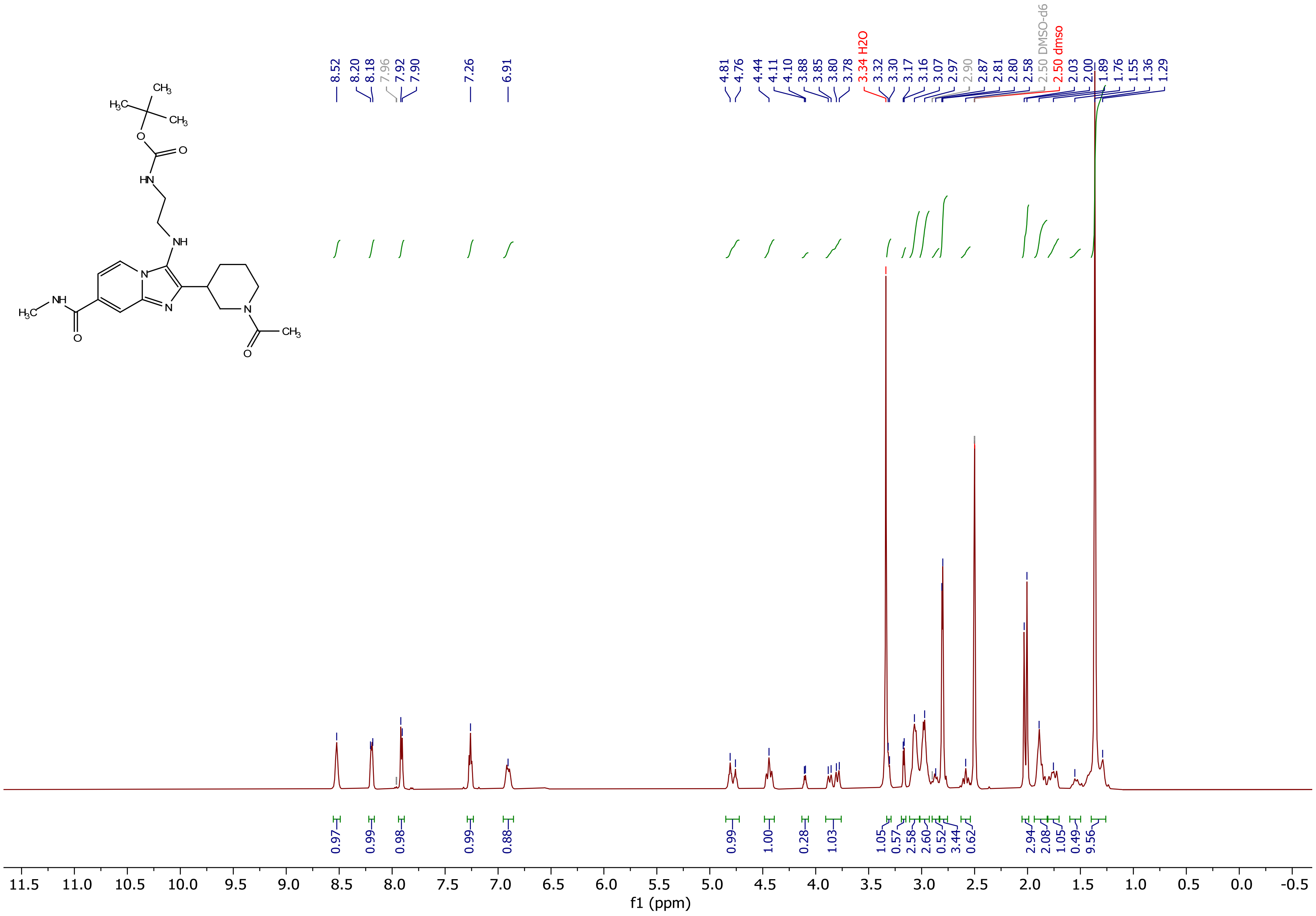

Spectrum 90. *tert*-Butyl N-(2-{[2-(1-acetylpiperidin-3-yl)-7-(methylcarbamoyl)imidazo[1,2-*a*]pyridin-3-yl]amino}ethyl)carbamate **4**{69,33,51}, ^{1}H NMR (500 MHz, DMSO-d_6; compound exists as a mixture of rotamers ca. 1:1)

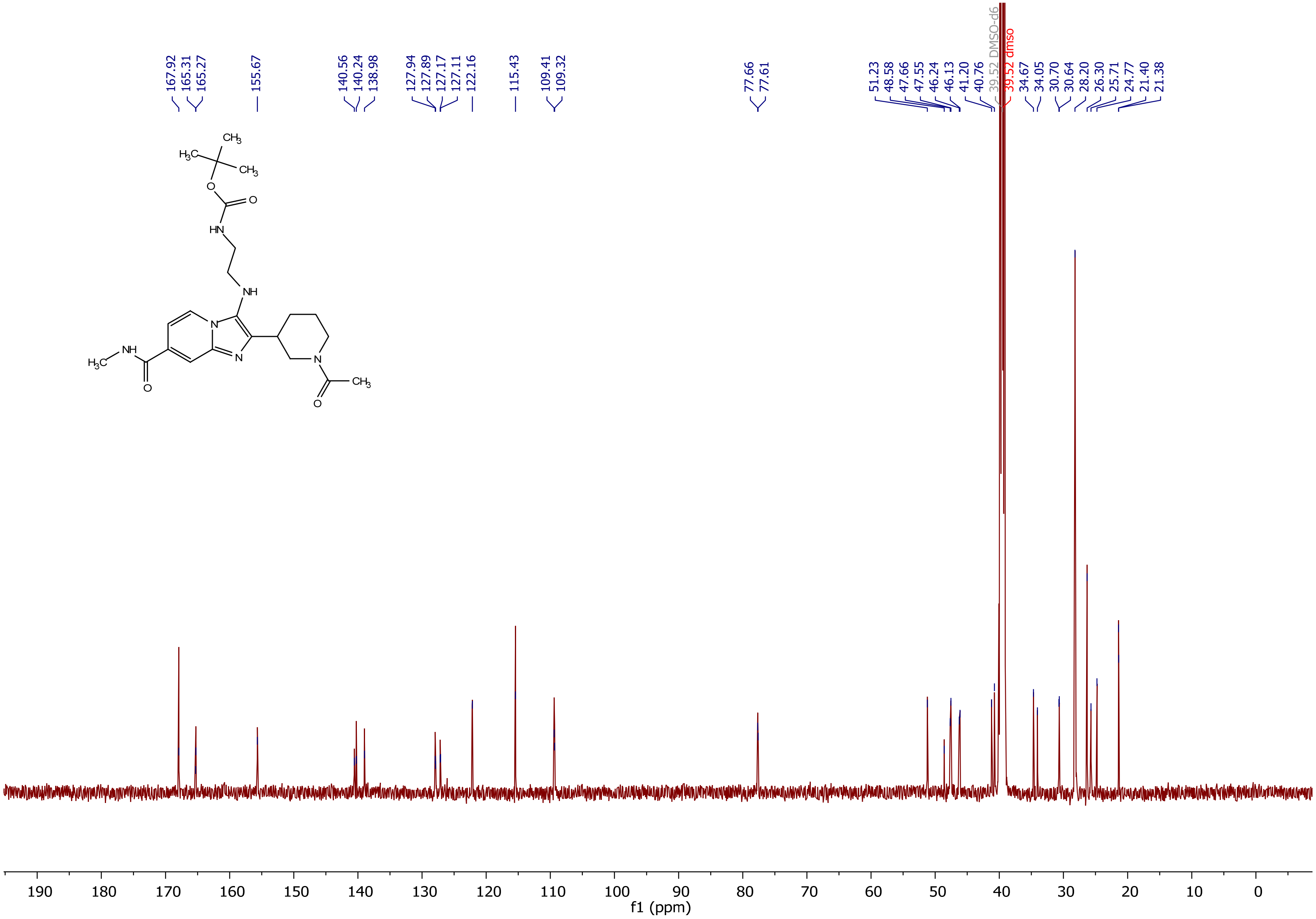

Spectrum 91. *tert*-Butyl N-(2-{[2-(1-acetylpiperidin-3-yl)-7-(methylcarbamoyl)imidazo[1,2-*a*]pyridin-3-yl]amino}ethyl)carbamate **4**{69,33,51}, $^{13}C\{^{1}H\}$ NMR (151 MHz, DMSO-d_6, compound exists as a mixture of rotamers ca. 1:1)

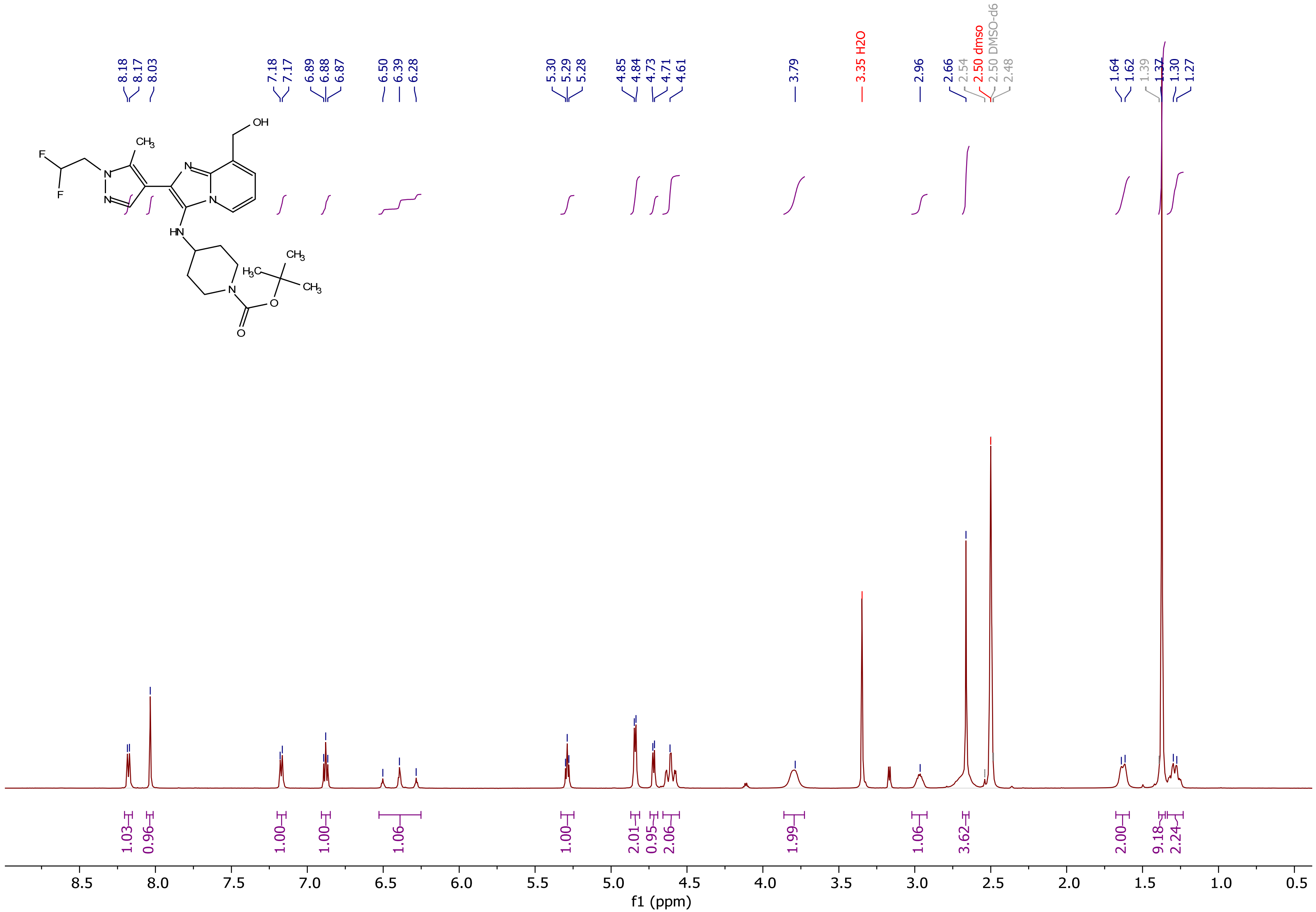

Spectrum 92. *tert*-Butyl 4-({2-[1-(2,2-difluoroethyl)-5-methyl-1*H*-pyrazol-4-yl]-8-(hydroxymethyl)imidazo[1,2-*a*]pyridin-3-yl}amino)piperidine-1-carboxylate **4**{124,304,43}, ^{1}H NMR (500 MHz, DMSO-d_6)

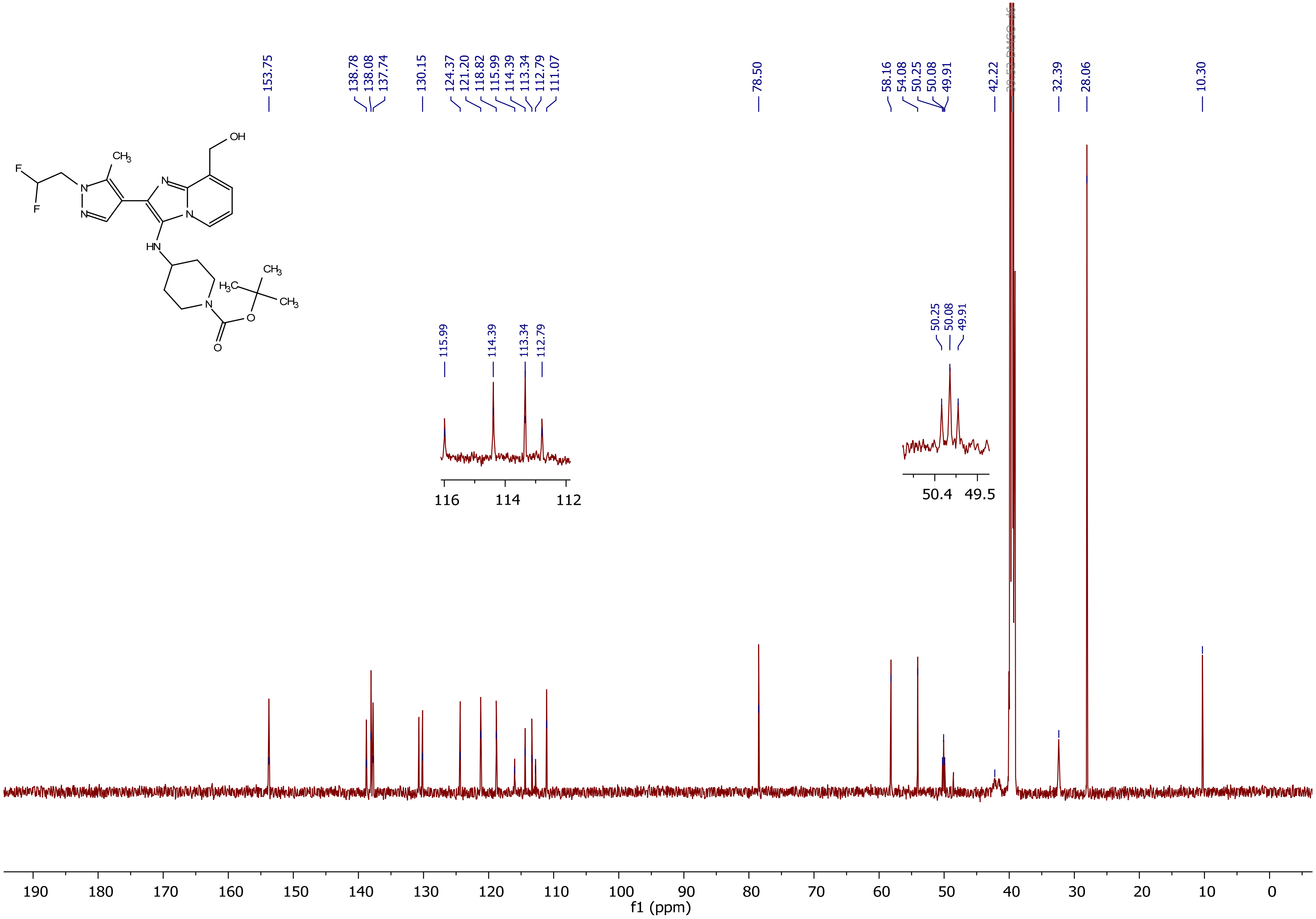

Spectrum 93. *tert*-Butyl 4-({2-[1-(2,2-difluoroethyl)-5-methyl-1*H*-pyrazol-4-yl]-8-(hydroxymethyl)imidazo[1,2-*a*]pyridin-3-yl}amino)piperidine-1-carboxylate **4**{124,304,43}, $^{13}C\{^{1}H\}$ NMR (151 MHz, DMSO-d_6)

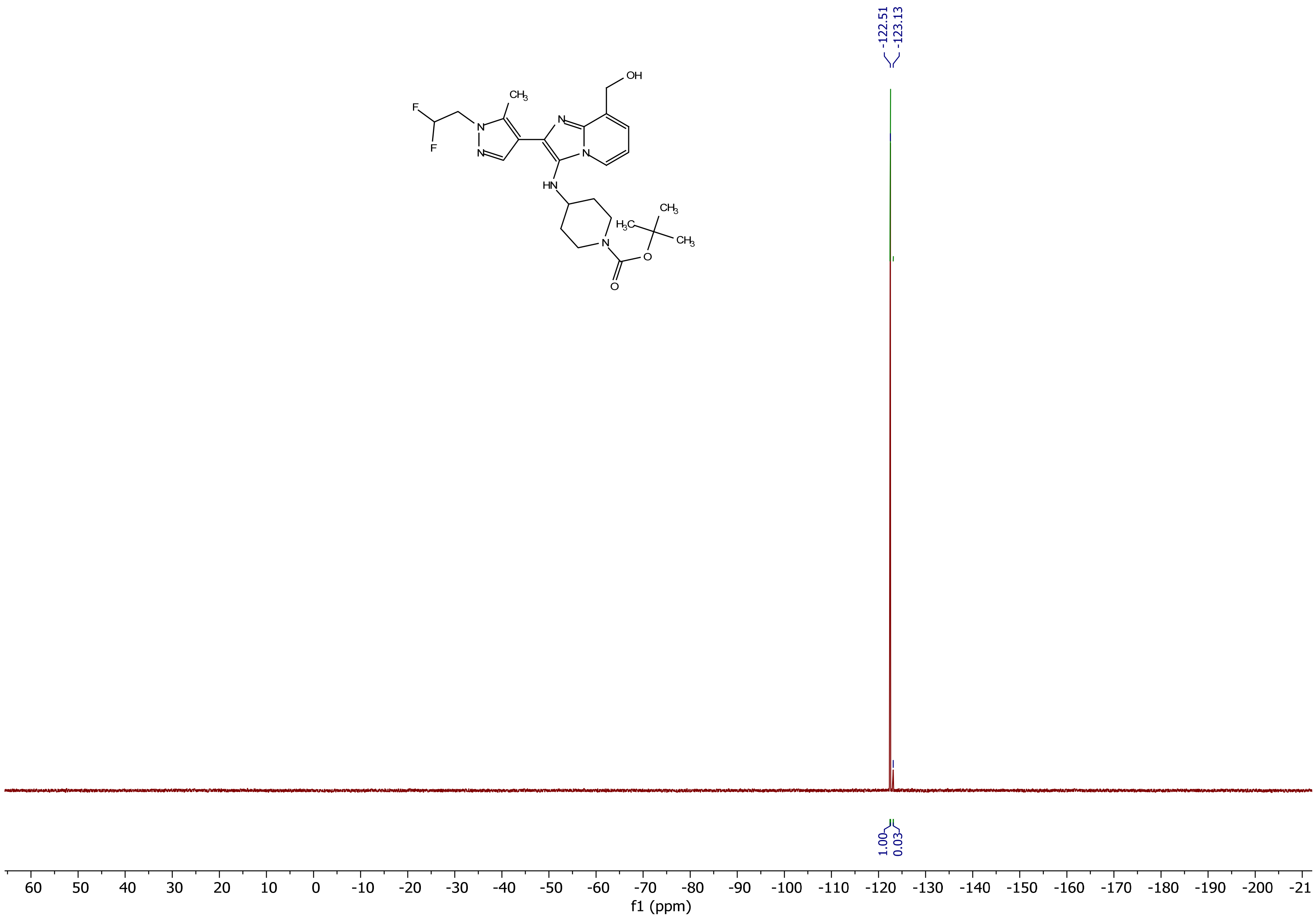

Spectrum 94. *tert*-Butyl 4-({2-[1-(2,2-difluoroethyl)-5-methyl-1*H*-pyrazol-4-yl]-8-(hydroxymethyl)imidazo[1,2-*a*]pyridin-3-yl}amino)piperidine-1-carboxylate **4**{124,304,43}, $^{19}F\{^{1}H\}$ NMR (376 MHz, DMSO-d_6)

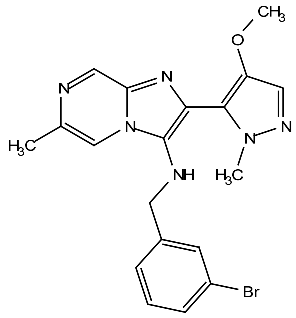

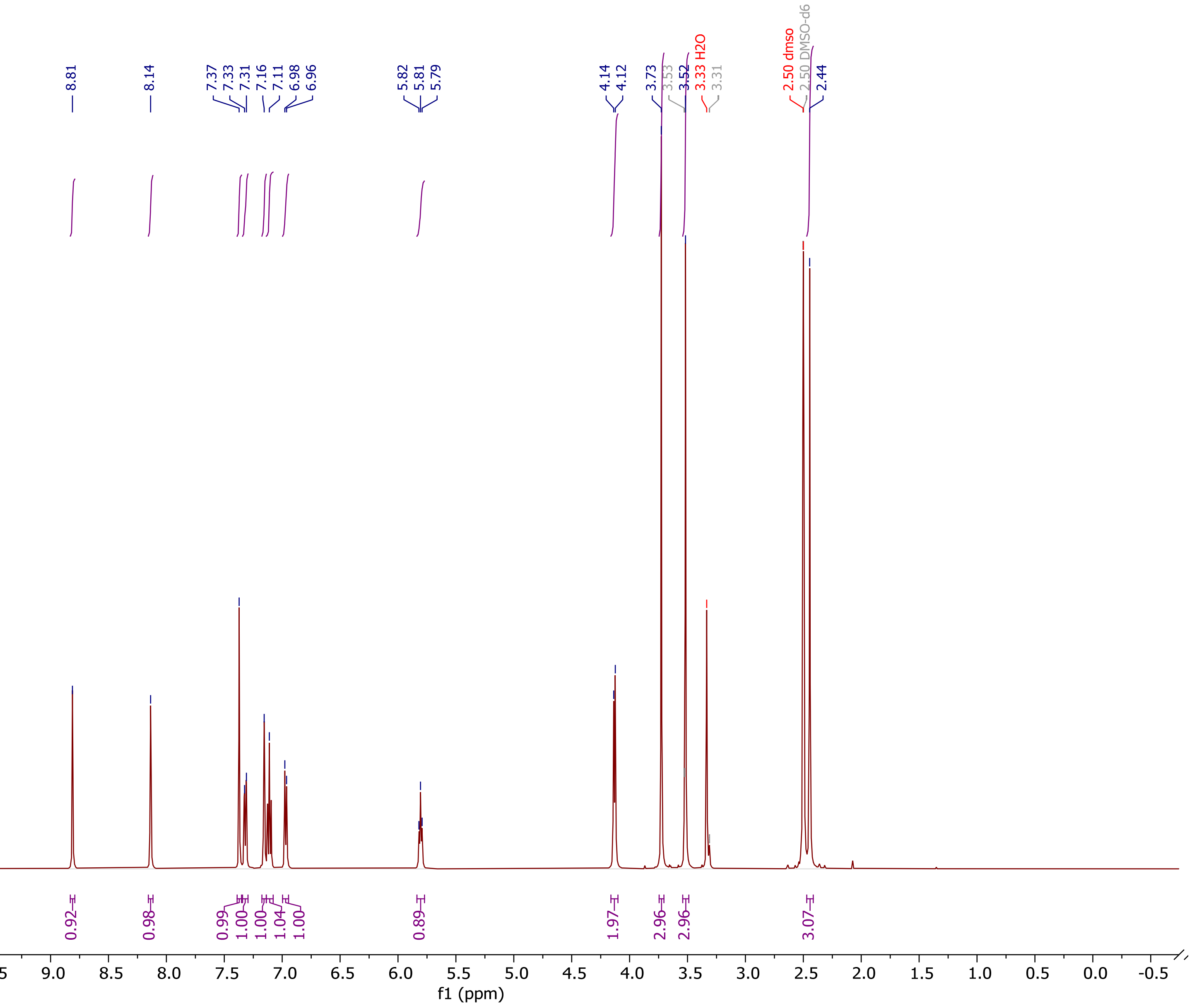

Spectrum 95. N-[(3-Bromophenyl)methyl]-2-(4-methoxy-1-methyl-1*H*-pyrazol-5-yl)-6-methylimidazo[1,2-*a*]pyrazin-3-amine **4**{41,19,11}, ^{1}H NMR (500 MHz, DMSO-d_6)

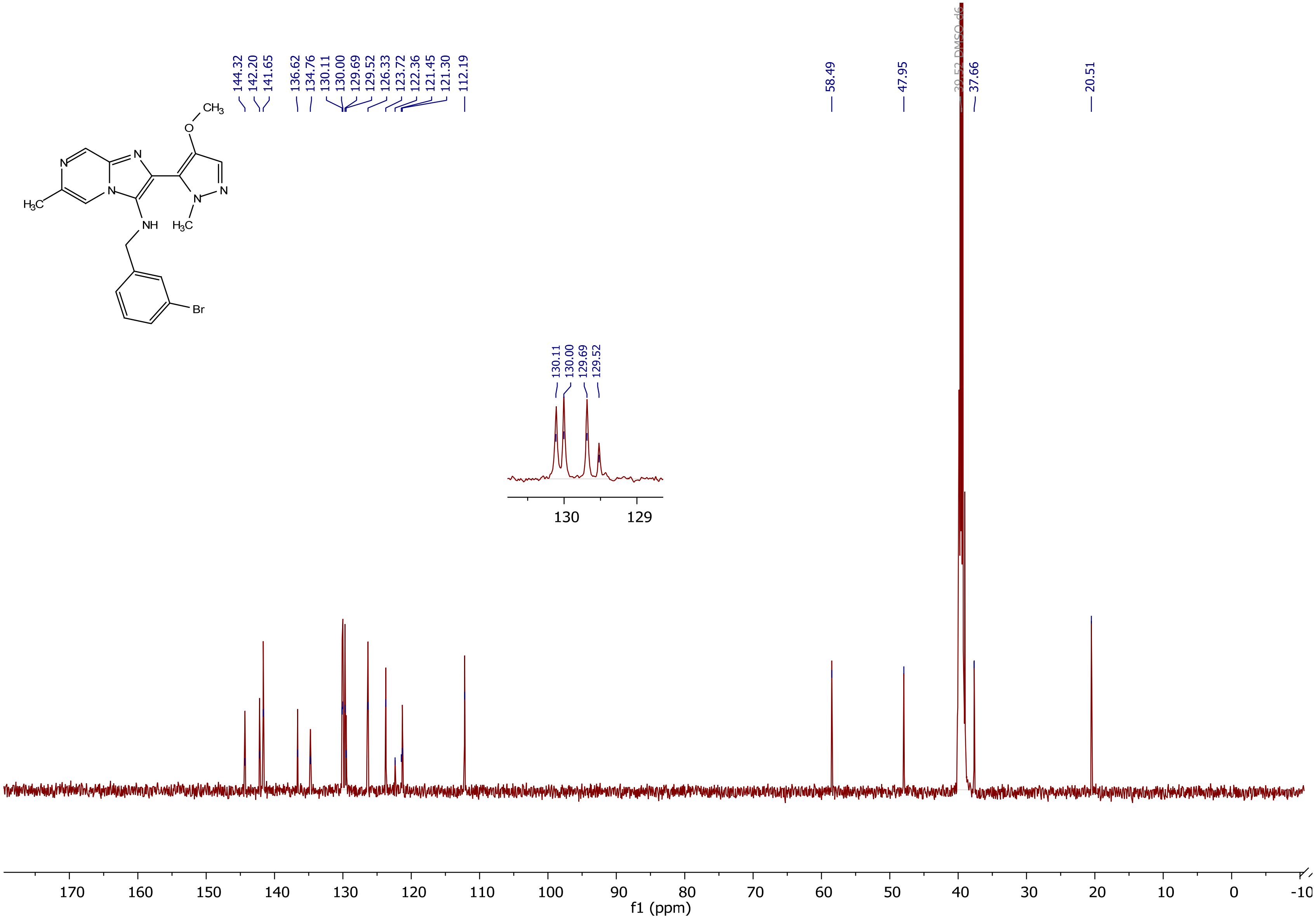

Spectrum 96. N-[(3-Bromophenyl)methyl]-2-(4-methoxy-1-methyl-1*H*-pyrazol-5-yl)-6-methylimidazo[1,2-*a*]pyrazin-3-amine **4**{41,19,11}, $^{13}C\{^{1}H\}$ NMR (126 Mhz, DMSO-d_6)

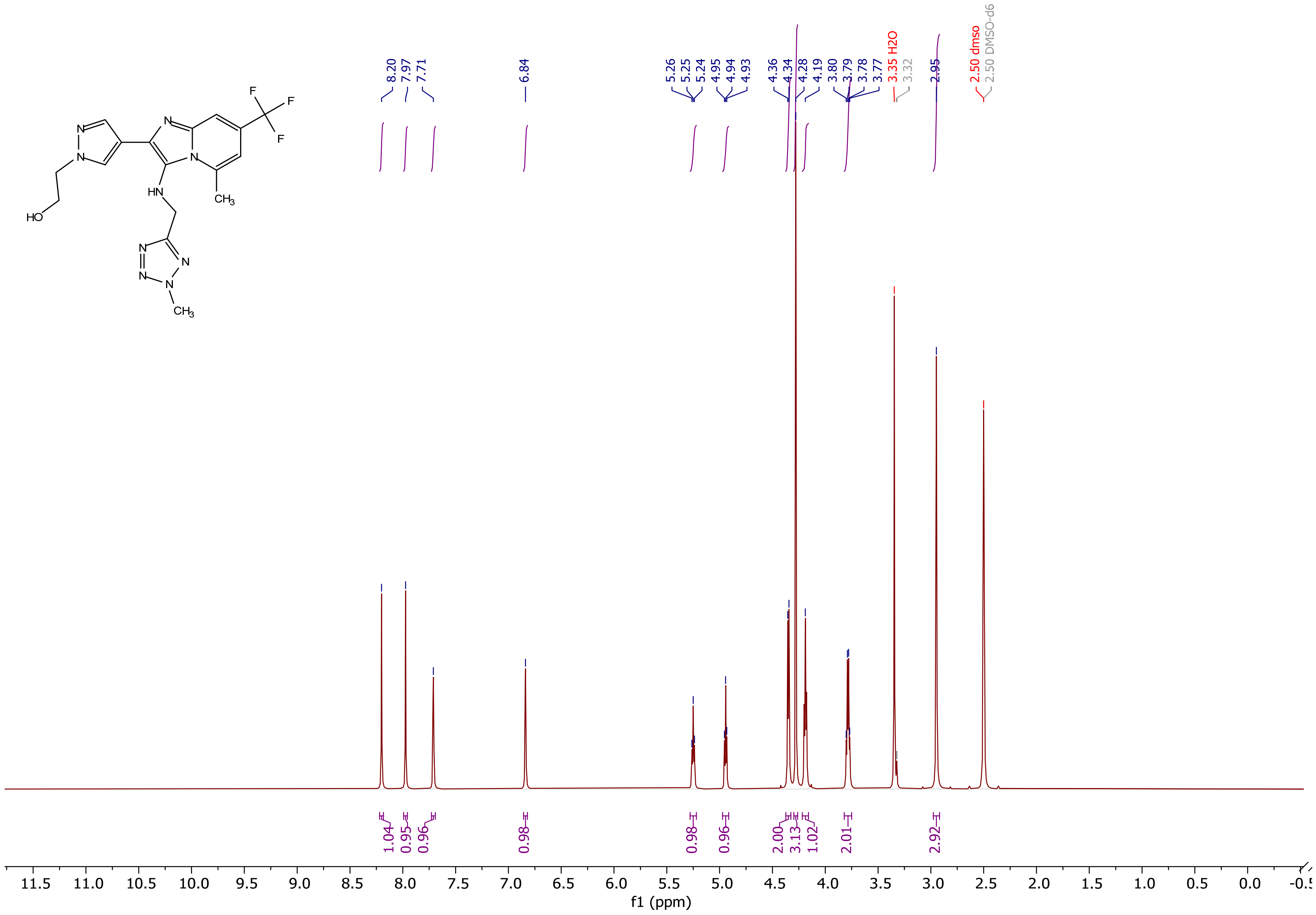

Spectrum 97. 2-[4-(5-Methyl-3-{[(2-methyl-2*H*-1,2,3,4-tetrazol-5-yl)methyl]amino}-7-(trifluoromethyl)imidazo[1,2-*a*]pyridin-2-yl)-1*H*-pyrazol-1-yl]ethan-1-ol **4**{40,18,4}, ^{1}H NMR (500 MHz, DMSO-d_6)

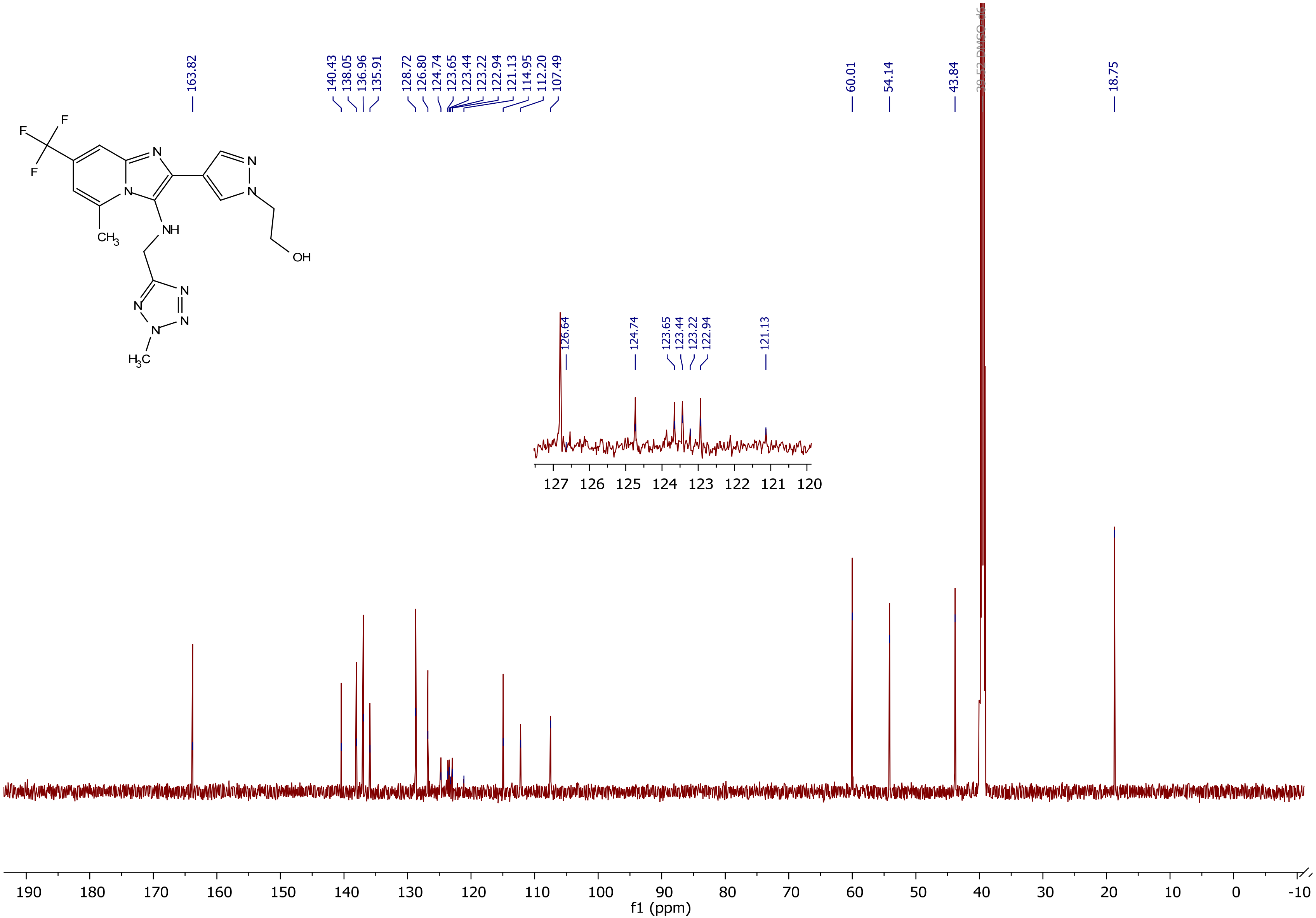

Spectrum 98. 2-[4-(5-Methyl-3-{[(2-methyl-2*H*-1,2,3,4-tetrazol-5-yl)methyl]amino}-7-(trifluoromethyl)imidazo[1,2-*a*]pyridin-2-yl)-1*H*-pyrazol-1-yl]ethan-1-ol **4**{40,18,4}, $^{13}C\{^{1}H\}$ NMR (151 MHz, DMSO-d_6)

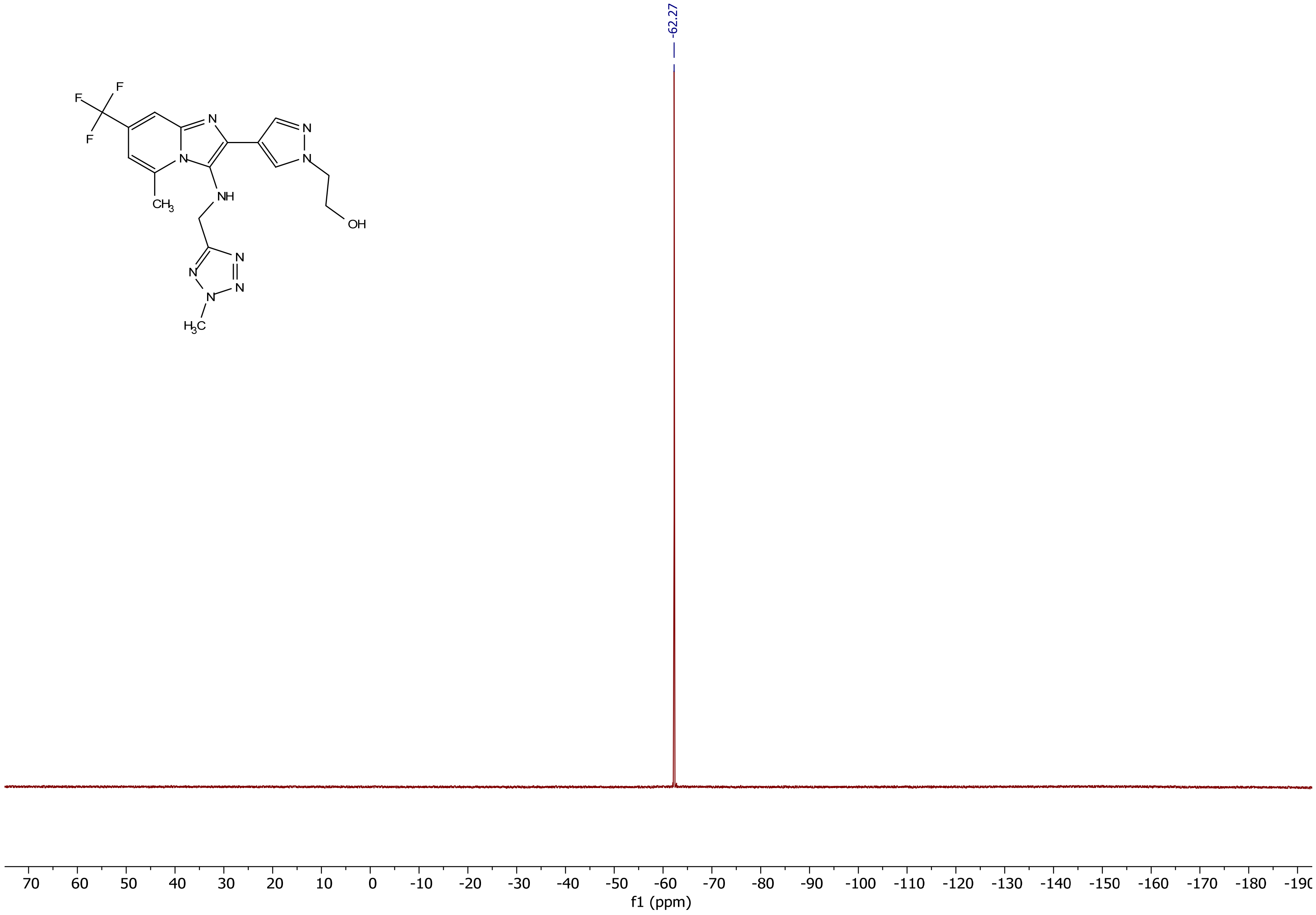

Spectrum 99. 2-[4-(5-Methyl-3-{[(2-methyl-2*H*-1,2,3,4-tetrazol-5-yl)methyl]amino}-7-(trifluoromethyl)imidazo[1,2-*a*]pyridin-2-yl)-1*H*-pyrazol-1-yl]ethan-1-ol **4**{40,18,4}, $^{19}F\{^1H\}$ NMR (376 MHz, DMSO-d_6)

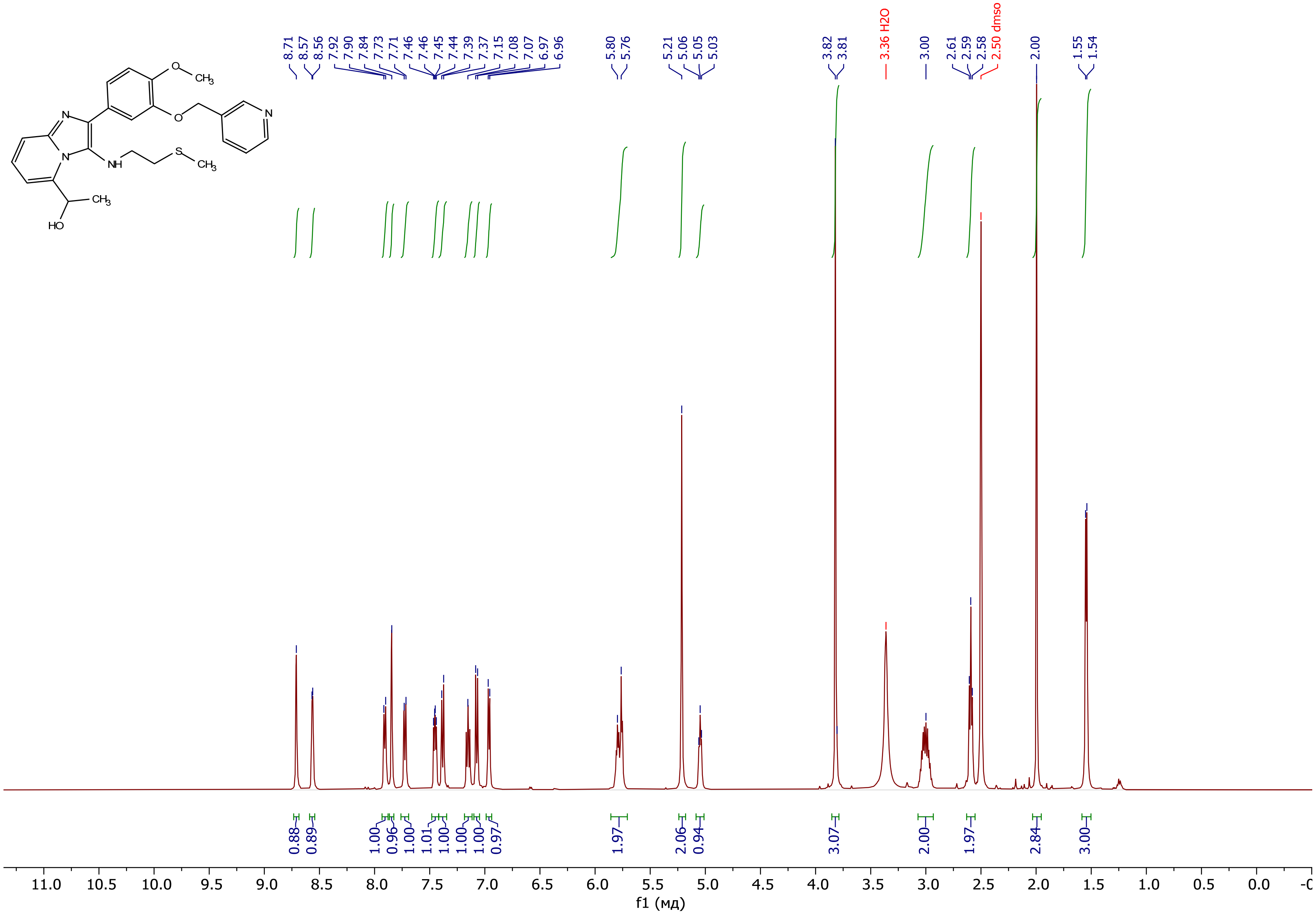

Spectrum 100. 1-(2-{4-Methoxy-3-[(pyridin-3-yl)methoxy]phenyl}-3-{[2-(methylsulfanyl)ethyl]amino}imidazo[1,2-*a*]pyridin-5-yl)ethan-1-ol **4**{118,625,31}, ^{1}H NMR (500 MHz, DMSO-d_6)

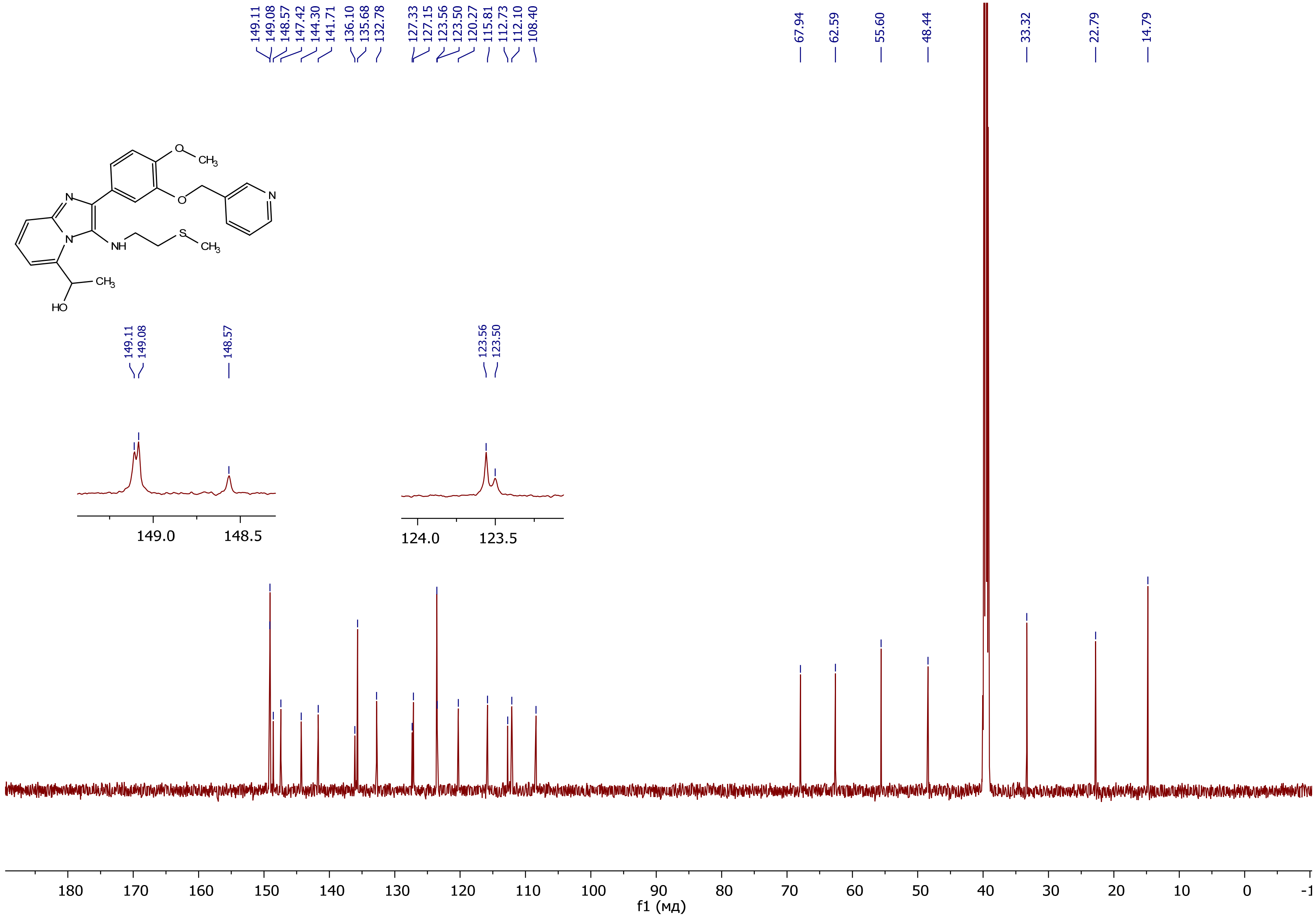

Spectrum 101. 1-(2-{4-Methoxy-3-[(pyridin-3-yl)methoxy]phenyl}-3-{[2-(methylsulfanyl)ethyl]amino}imidazo[1,2-*a*]pyridin-5-yl)ethan-1-ol **4**{118,625,31}, $^{13}C\{^{1}H\}$ NMR (151 MHz, DMSO-d_6)

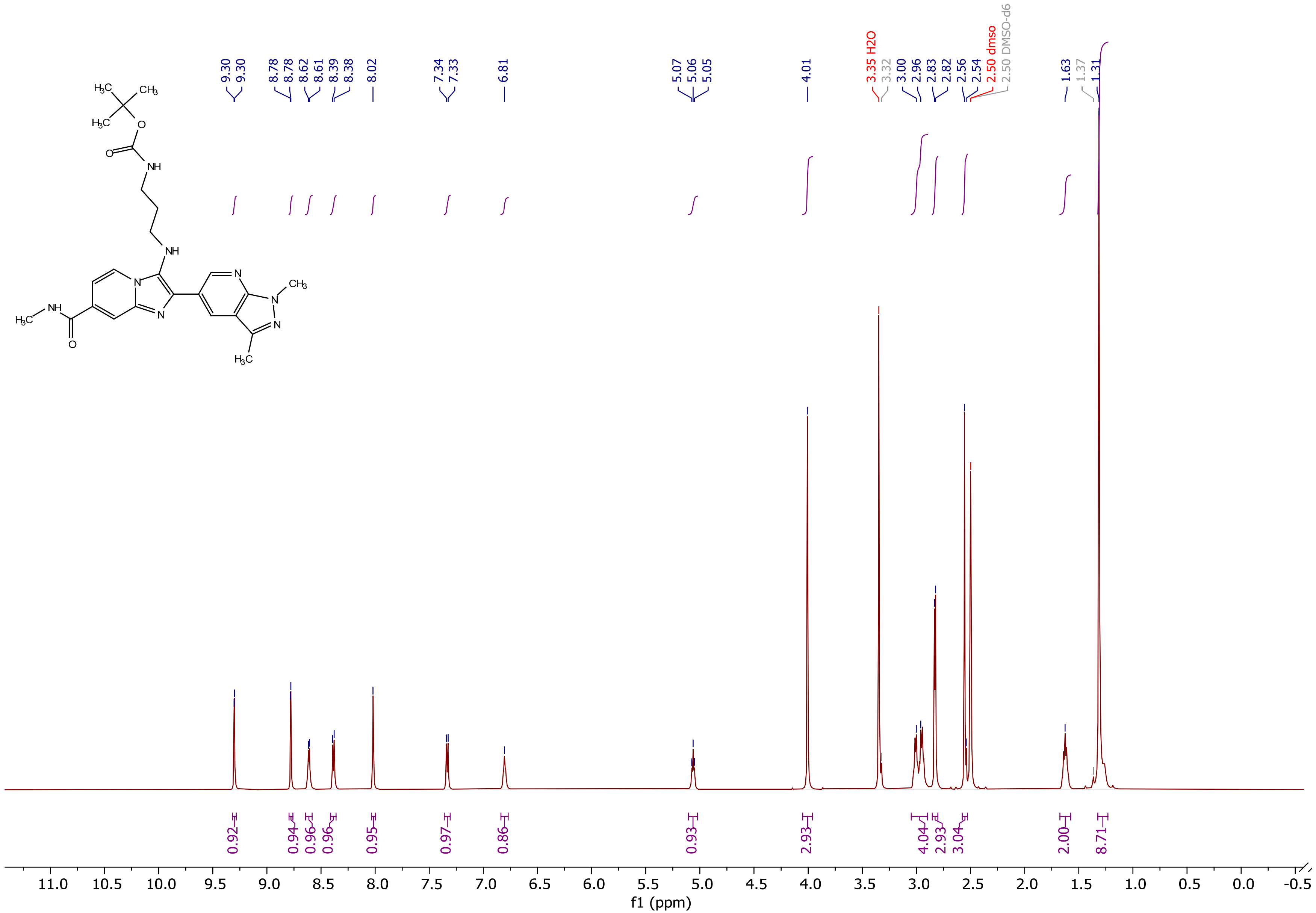

Spectrum 102. *tert*-Butyl N-{3-[(2-{1,3-dimethyl-1*H*-pyrazolo[3,4-*b*]pyridin-5-yl}-7-(methylcarbamoyl)imidazo[1,2-*a*]pyridin-3-yl)amino]propyl}carbamate **4**{69,596,61}, ^{1}H NMR (500 MHz, DMSO-d_6)

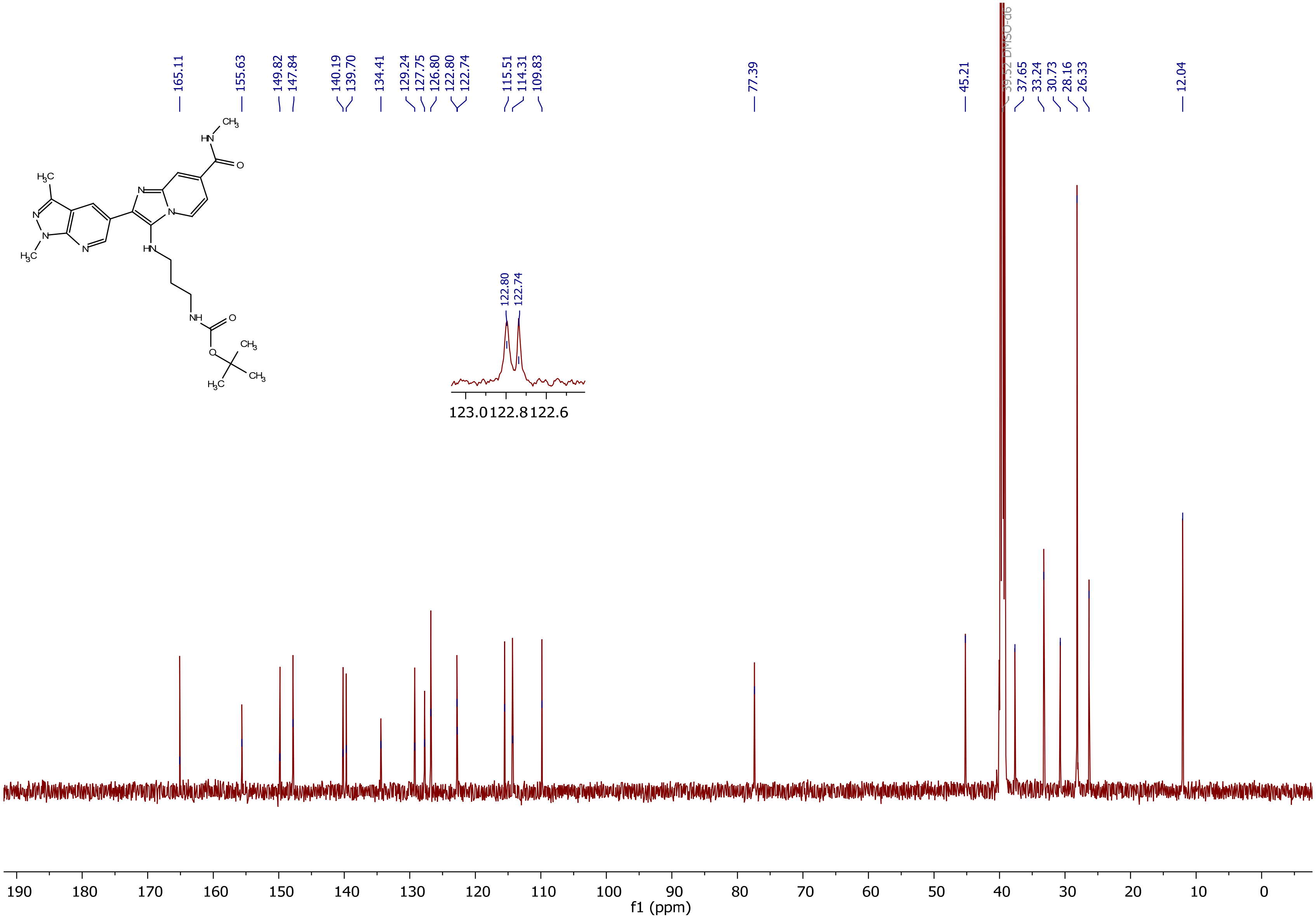

Spectrum 103. *tert*-Butyl N-{3-[(2-{1,3-dimethyl-1*H*-pyrazolo[3,4-*b*]pyridin-5-yl}-7-(methylcarbamoyl)imidazo[1,2-*a*]pyridin-3-yl)amino]propyl}carbamate **4**{69,596,61}, $^{13}C\{^{1}H\}$ NMR (151 MHz, DMSO-d_6)

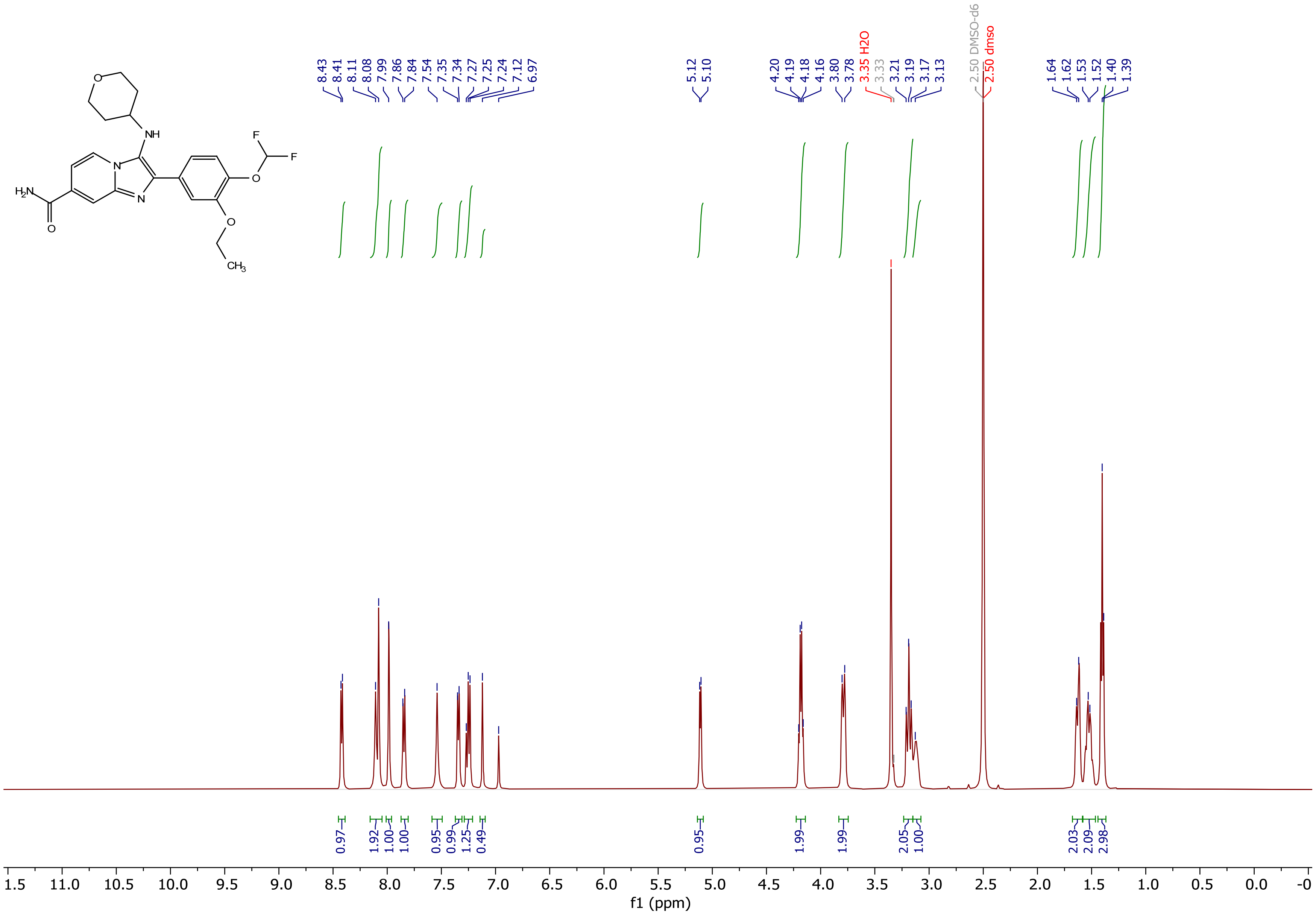

Spectrum 104. 2-[4-(Difluoromethoxy)-3-ethoxyphenyl]-3-[(oxan-4-yl)amino]imidazo[1,2-*a*]pyridine-7-carboxamide **4**{92,616,7}, ^{1}H NMR (500 MHz, DMSO-d_6)

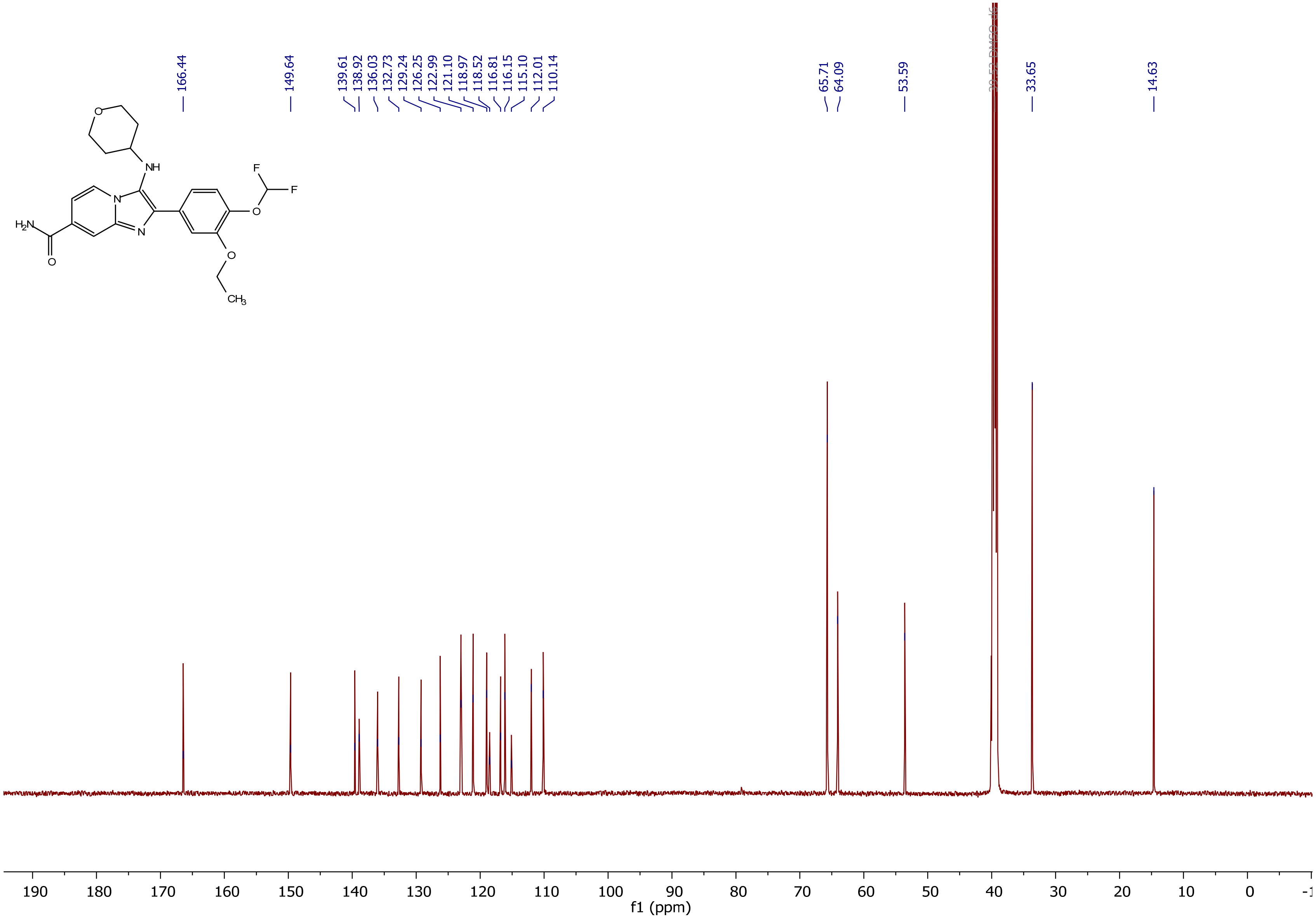

Spectrum 105. 2-[4-(Difluoromethoxy)-3-ethoxyphenyl]-3-[(oxan-4-yl)amino]imidazo[1,2-*a*]pyridine-7-carboxamide **4**{92,616,7}, $^{13}C\{^{1}H\}$ NMR (151 MHz, DMSO-d_6)

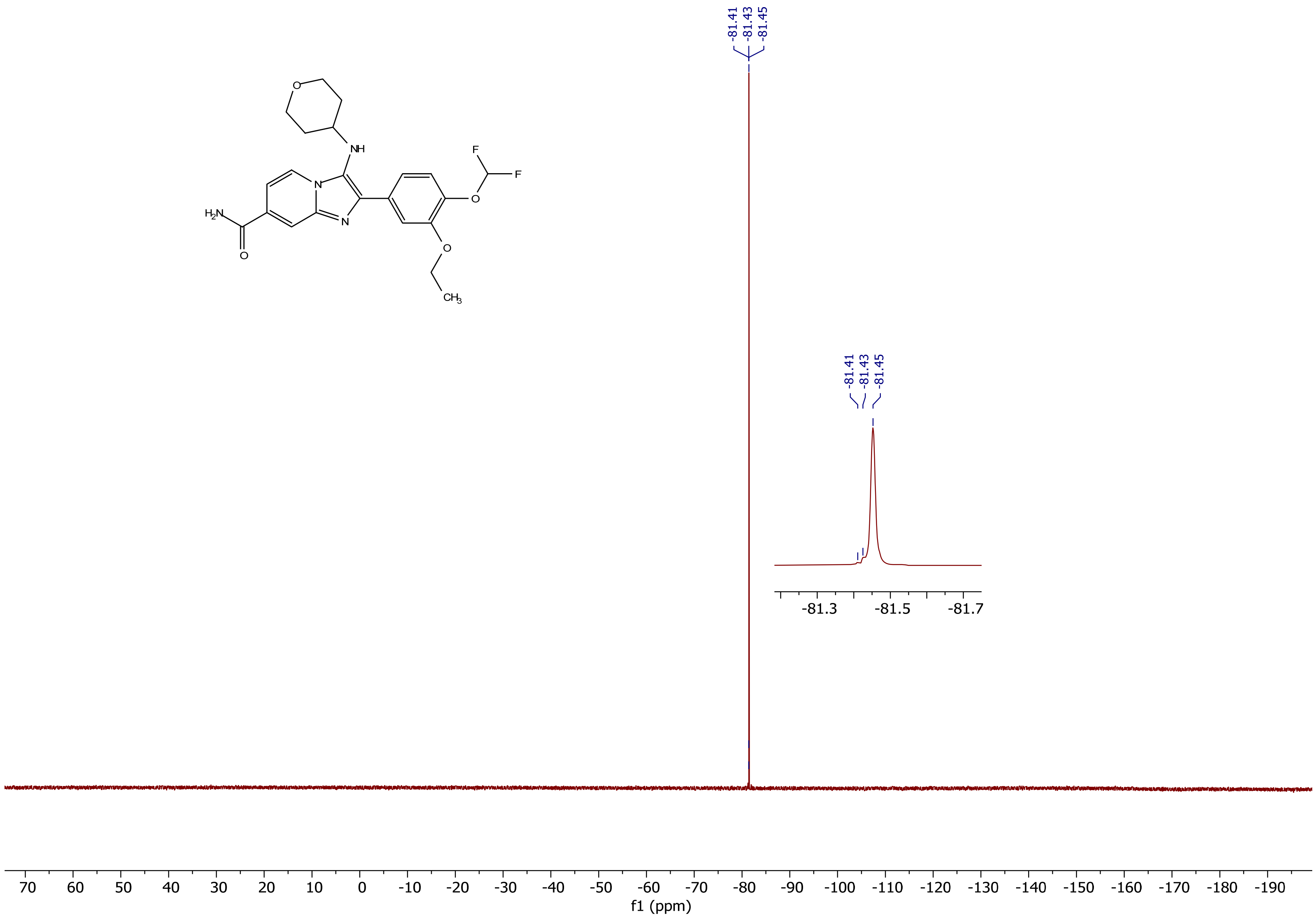

Spectrum 106. 2-[4-(Difluoromethoxy)-3-ethoxyphenyl]-3-[(oxan-4-yl)amino]imidazo[1,2-*a*]pyridine-7-carboxamide **4**{92,616,7}, $^{19}F\{^{1}H\}$ NMR (376 MHz, dmso-d_6)

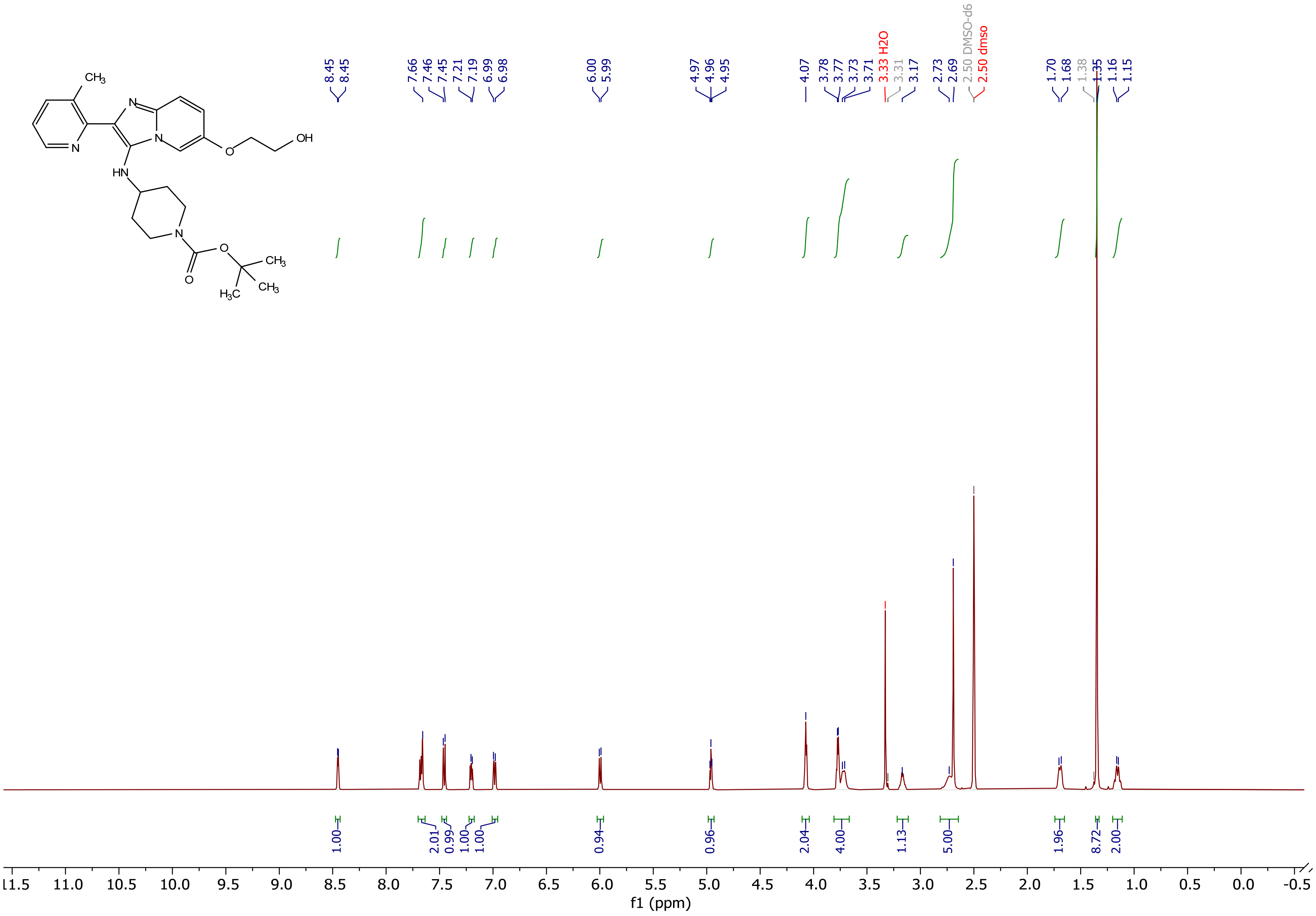

Spectrum 107. *tert*-Butyl 4-{[6-(2-hydroxyethoxy)-2-(3-methylpyridin-2-yl)imidazo[1,2-*a*]pyridin-3-yl]amino}piperidine-1-carboxylate **4**{107,621,43}, ^{1}H NMR (600 MHz, DMSO-d_6)

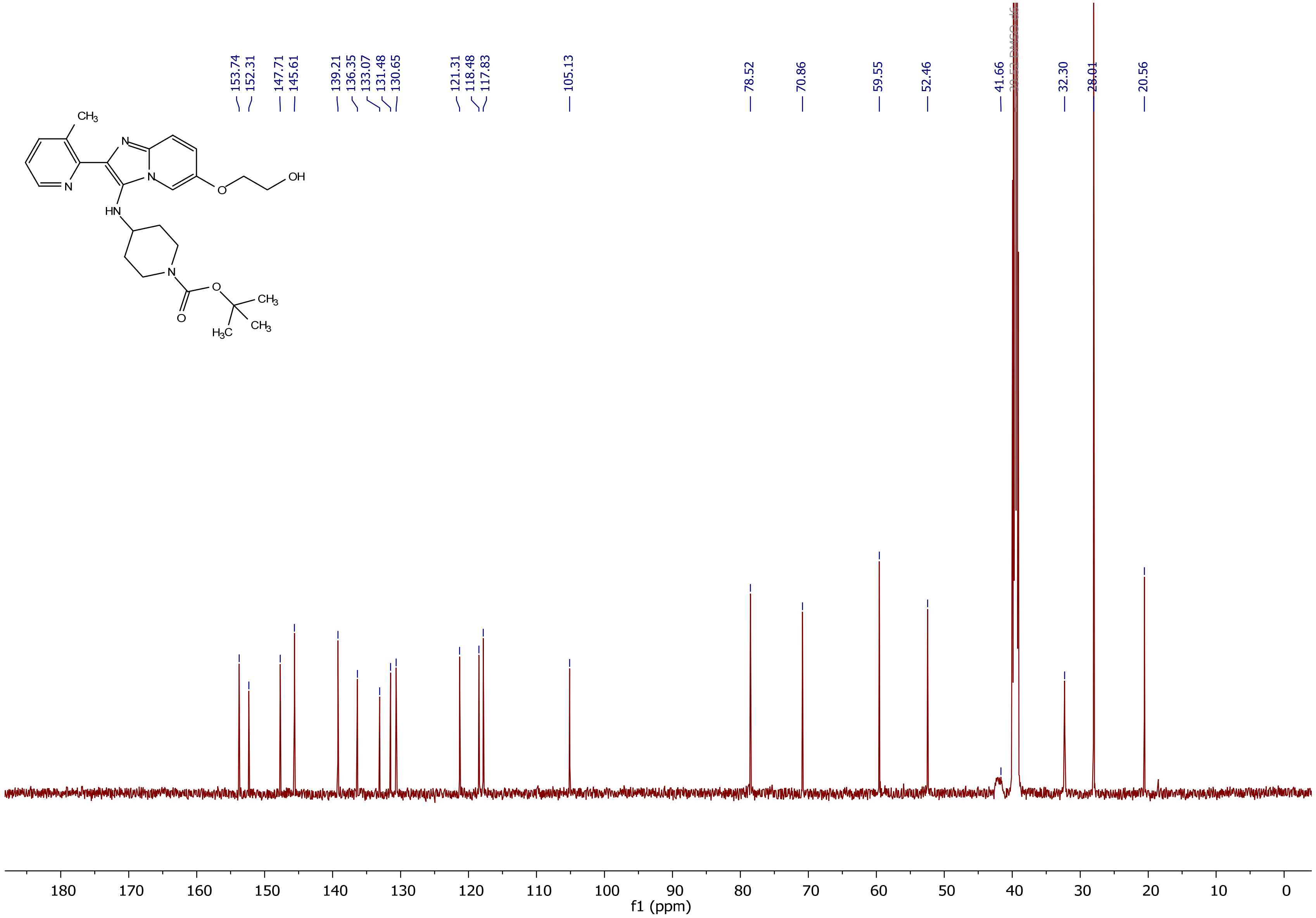

Spectrum 108. *tert*-Butyl 4-{[6-(2-hydroxyethoxy)-2-(3-methylpyridin-2-yl)imidazo[1,2-*a*]pyridin-3-yl]amino}piperidine-1-carboxylate **4**{107,621,43}, $^{13}C\{^{1}H\}$ NMR (151 MHz, DMSO-d_6)

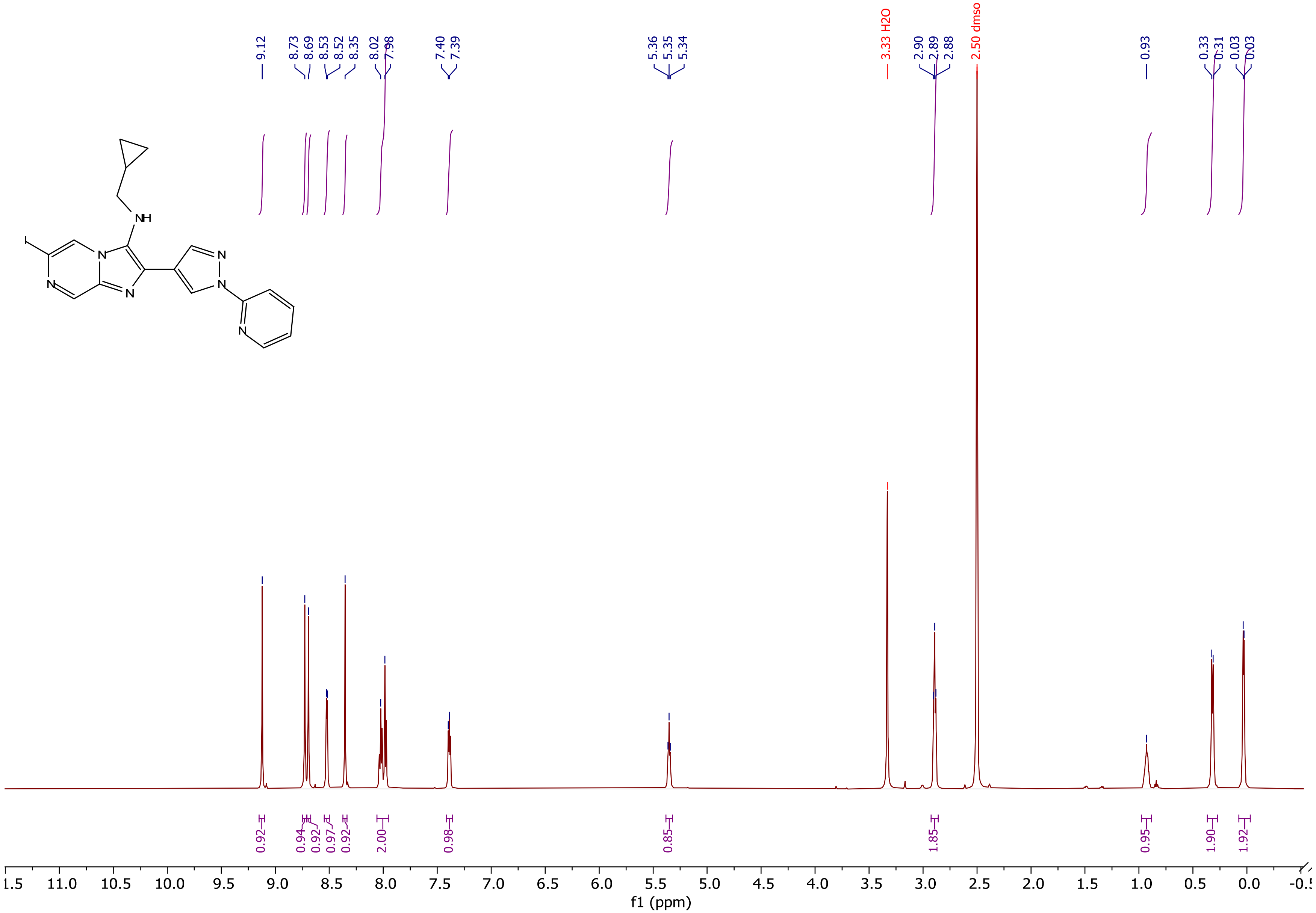

Spectrum 109. *N*-(Cyclopropylmethyl)-6-iodo-2-[1-(pyridin-2-yl)-1*H*-pyrazol-4-yl]imidazo[1,2-*a*]pyrazin-3-amine **4**{71,615,21}, ^{1}H NMR (600 MHz, DMSO-d_6)

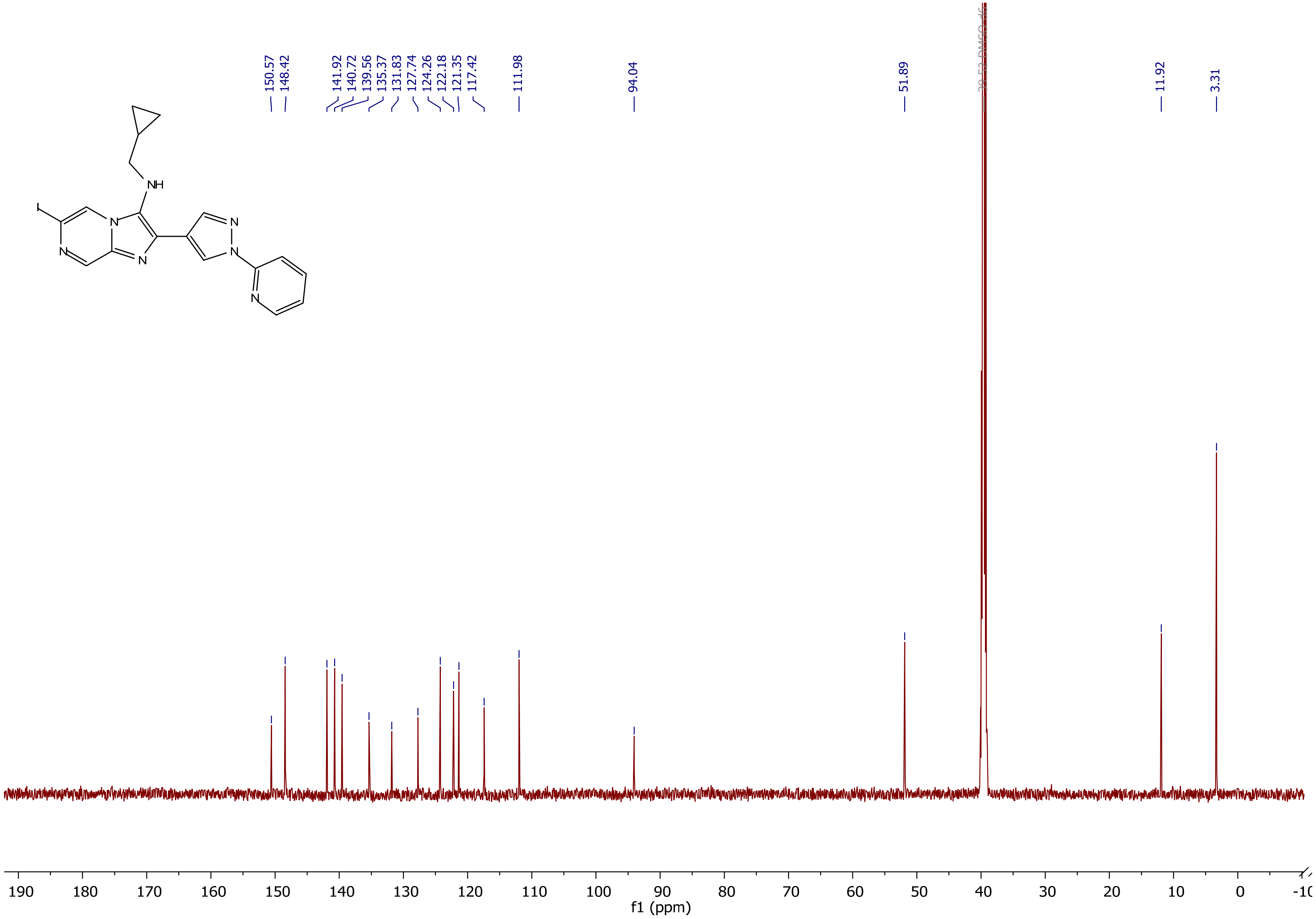

Spectrum 110. *N*-(Cyclopropylmethyl)-6-iodo-2-[1-(pyridin-2-yl)-1*H*-pyrazol-4-yl]imidazo[1,2-*a*]pyrazin-3-amine **4**{71,615,21}, $^{13}C\{^{1}H\}$ NMR (151 MHz, DMSO-d_6)
